# Discovery Proteomics Identifies a Molecular Link between the Coatomer Protein Complex I and Androgen Receptor-dependent Transcription*[Fn FN2]

**DOI:** 10.1074/jbc.M116.732313

**Published:** 2016-06-30

**Authors:** Jordy J. Hsiao, Melinda M. Smits, Brandon H. Ng, Jinhee Lee, Michael E. Wright

**Affiliations:** From the Department of Molecular Physiology and Biophysics, Carver College of Medicine, Iowa City, Iowa 52242

**Keywords:** androgen, androgen receptor, mass spectrometry (MS), protein trafficking (Golgi), transcription regulation, transcription

## Abstract

Aberrant androgen receptor (AR)-dependent transcription is a hallmark of human prostate cancers. At the molecular level, ligand-mediated AR activation is coordinated through spatial and temporal protein-protein interactions involving AR-interacting proteins, which we designate the “AR-interactome.” Despite many years of research, the ligand-sensitive protein complexes involved in ligand-mediated AR activation in prostate tumor cells have not been clearly defined. Here, we describe the development, characterization, and utilization of a novel human LNCaP prostate tumor cell line, N-AR, which stably expresses wild-type AR tagged at its N terminus with the streptavidin-binding peptide epitope (streptavidin-binding peptide-tagged wild-type androgen receptor; SBP-AR). A bioanalytical workflow involving streptavidin chromatography and label-free quantitative mass spectrometry was used to identify SBP-AR and associated ligand-sensitive cytosolic proteins/protein complexes linked to AR activation in prostate tumor cells. Functional studies verified that ligand-sensitive proteins identified in the proteomic screen encoded modulators of AR-mediated transcription, suggesting that these novel proteins were putative SBP-AR-interacting proteins in N-AR cells. This was supported by biochemical associations between recombinant SBP-AR and the ligand-sensitive coatomer protein complex I (COPI) retrograde trafficking complex *in vitro*. Extensive biochemical and molecular experiments showed that the COPI retrograde complex regulates ligand-mediated AR transcriptional activation, which correlated with the mobilization of the Golgi-localized ARA160 coactivator to the nuclear compartment of prostate tumor cells. Collectively, this study provides a bioanalytical strategy to validate the AR-interactome and define novel AR-interacting proteins involved in ligand-mediated AR activation in prostate tumor cells. Moreover, we describe a cellular system to study how compartment-specific AR-interacting proteins influence AR activation and contribute to aberrant AR-dependent transcription that underlies the majority of human prostate cancers.

## Introduction

Androgen receptor (AR^2^; NR3C4) is a steroid hormone receptor (SHR) that belongs to a subgroup of the nuclear receptor superfamily of ligand-induced transcription factors (1). Under normal physiological conditions, androgenic ligands activate AR to regulate gene expression programs involved in the development, differentiation, and maintenance of the male reproductive system (1). However, androgen-activated AR is also associated with pathophysiological processes such as oncogenesis in the human prostate (2). Recent studies have shown that ∼50% of patients with early stage organ-confined prostate cancer contain gene fusions (*i.e. TMPRSS2-ERG*) that place the ETS family of oncogenic transcription factors (*i.e.* ERG and ETV1) under the direct control of AR (3). These gene fusions facilitate the rewiring of AR-dependent transcription programs in prostate epithelial cells to increase their invasive potential at the cellular level (4–6). Aberrant AR-dependent transcriptional programs also underlie the development of late stage (*i.e.* metastatic) castration-resistant prostate cancers. To date, multiple mechanisms are known to elicit aberrant AR activity and thereby facilitate the proliferation and survival of castration-resistant prostate cancers in the context of castration levels of androgens. These include the expression of constitutively active AR splice variants, gain-of-function AR mutations, increased expression of androgen-biosynthesis genes, ligand-independent AR activation, aberrant AR coregulator expression, gain-of-function mutations in steroidogenesis enzymes, and activation of the glucocorticoid receptor bypass pathway (7, 8). The clinical significance of aberrant AR activity in the development and progression of human prostate cancers is underscored by the current therapeutic treatment modalities (*i.e.* the use of androgen deprivation therapies; second-generation anti-androgens, such as enzalutamide; and inhibitors of steroidogenesis, such as abiraterone), which target the AR signaling axis to disrupt aberrant AR activity in early and late stage human prostate cancers (9). Although multiple mechanisms underlying aberrant AR-dependent transcription have been clearly established at the molecular level, current therapeutic modalities lack the power to permanently disrupt aberrant AR activity in prostate tumor cells. This is especially significant in the treatment and management of castration-resistant prostate cancers because alternative therapies to cure patients afflicted by this lethal disease do not exist (9, 10).

Fundamental insights into the molecular steps involved in androgen-mediated AR activation have been gleaned from over 30 years of biochemical research (1). Current molecular models show that in the absence of ligand, AR is sequestered in the cytosolic compartment, where it is bound by molecular chaperones (11). Upon the binding of androgenic ligands, AR undergoes cytoplasmic-nuclear trafficking, supposedly through the actions of microtubule-associated motor proteins, with liganded AR traversing the nuclear pore through physical interactions with nuclear import receptors (12). Once in the nucleus, ligand-bound AR binds to chromatin-embedded androgen response elements and recruits transcriptional coactivator/corepressor complexes to target genes in the genome (1). Importantly, the process of ligand-mediated AR activation supposedly entails the direct physical interaction of over 350 proteins that bind to AR at the cellular level (13–16). These AR-interacting proteins, which we denote as the “AR-interactome,” were primarily discovered through binary protein-protein interaction assays (13–16). Many members of the AR-interactome function as coregulators of AR-mediated transcription, and broadly speaking, they encode proteins involved in general transcription (*e.g.* ARIP4 and BRG1), cellular proteins of diverse function that coactivate or corepress AR-mediated transcription (*e.g.* PTEN and HIP1), and specific transcription factors (*e.g.* ERα and FOXA1) (1). Importantly, the AR-interactome is incomplete because novel AR-interacting proteins continue to be reported in the scientific literature. This observation demonstrates that current molecular models of ligand-mediated AR activation are insufficient. This scenario makes it difficult to understand and predict the protein machinery, both spatially and temporally, that is required for androgen-mediated AR activation. This shortcoming has an even greater significance in the context of predicting how this molecular machinery might become perturbed and contribute to aberrant AR activation in prostate tumor cells. Therefore, a molecular model is needed to capture ligand-dependent interactions between AR and the AR-interactome across the different subcellular compartments during the process of androgen-mediated AR activation. Such a model would provide a molecular framework for testing and exploring how the AR-interactome contributes to aberrant AR-dependent transcriptional programs underlying early and late stage prostate cancers.

To this end, we developed a cellular system to identify ligand-sensitive AR-interacting protein complexes in prostate tumor cells using quantitative mass spectrometry. More specifically, streptavidin chromatography was used to affinity-purify streptavidin-binding peptide-tagged wild-type AR (SBP-AR) from the cytosolic compartment in the unliganded (*i.e.* androgen-depleted) and liganded (*i.e.* androgen-stimulated) states in LNCaP prostate tumor cells. Label-free directed mass spectrometry (dMS) facilitated the identification and quantitation of ligand-sensitive proteins. The proteomic data set enriched for the AR-interactome and functional studies verified that ligand-sensitive proteins encoded modulators of AR-mediated transcription in LNCaP cells. Further exploration of ligand-sensitive proteins showed that the coatomer protein complex I (COPI) retrograde complex encoded novel SBP-AR-interacting proteins that are functionally linked to AR-mediated transcription in LNCaP cells. Moreover, biochemical studies showed that AR was localized to the Golgi-enriched protein fraction (GEPF) in a ligand-sensitive manner in LNCaP cells. Interestingly, whereas AR-dependent transcription was attenuated by chemical or genetic disruptions of the COPI complex, androgen-mediated nuclear localization of AR was unperturbed in the context of these treatments. In contrast, the nuclear accumulation of the Golgi-localized coactivator ARA160 was disrupted under the same experimental conditions. These results demonstrated that androgen-mediated nuclear mobilization of ARA160 was required for AR-dependent transcription. This study provides a molecular framework for defining compartment-specific, ligand-sensitive AR-interacting proteins involved in androgen-mediated AR activation in prostate tumor cells.

## Results

### 

#### 

##### Molecular Properties of Wild-type AR in LNCaP Prostate Tumor Cells

**FIGURE 1.**
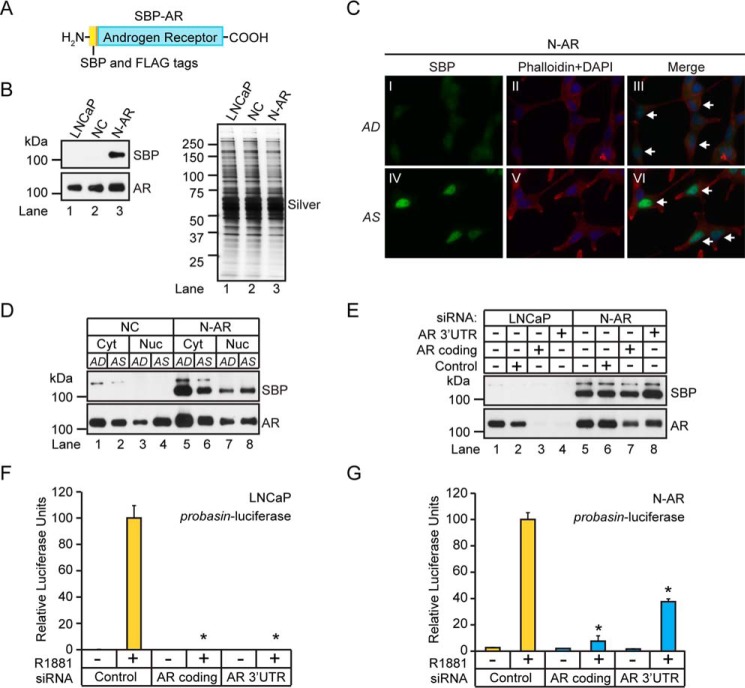
**Molecular characterization of SBP-AR expressed in LNCaP prostate tumor cells.** *A*, diagram of SBP-AR. The AR cDNA was cloned in-frame into the pcDNA3-SBP-FLAG mammalian expression vector and expressed as an N-terminal SBP fusion protein. *B*, Western blotting analysis of whole cell lysates extracted from LNCaP, NC, and N-AR cells using antibodies to SBP and AR (*left*). A silver-stained gel shows equivalent protein loading between the samples (*right*). Western blotting results are representative of two biological replicates. *C*, IF analysis of AD and AS N-AR cells, demonstrating androgen-dependent nuclear translocation of SBP-AR, as indicated by the *white arrows*. The cells were fixed, probed with SBP (*green*) antibodies, and co-stained with phalloidin (*red*) and DAPI (*blue*). IF results are representative of two biological replicates. *D*, Western blotting analysis of cytosolic (*Cyt*) and nuclear (*Nuc*) proteins isolated from NC and N-AR cells cultured under AD and AS conditions using antibodies to SBP and AR. Representative Western blotting results are derived from two biological replicates. *E*, Western blotting analysis of whole cell lysates extracted from LNCaP and N-AR cells transfected with siRNAs targeting the coding or 3′-UTR region of AR, using SBP and AR antibodies. Representative Western blotting results are derived from two biological replicates. *F* and *G*, SBP-AR possesses androgen-mediated transcriptional activity. Dual-Luciferase assays were performed following co-transfection of LNCaP and N-AR cells with the indicated siRNAs (100 nm) and the probasin-luciferase and pRLSV40-*Renilla* vectors. The measured luciferase activities were normalized to the activity of the vehicle control. Results are presented as the mean ± S.D. (*error bars*) of three biological replicates (*n* = 3). *Asterisks* indicate significant differences between cells transfected with target and control siRNAs (Student's *t* test; *, *p* < 0.05).

The AR-interactome represents a diverse population of proteins that regulates AR function at the molecular level (1). This prompted us to develop an experimental workflow to validate and identify novel members of the AR-interactome. Specifically, we built a heterologous AR expression system into LNCaP human prostate tumor cells to identify AR-interacting proteins using quantitative mass spectrometry. Although LNCaP cells express mutant AR (AR-T877A) (17), the goal was to identify AR-interacting proteins in LNCaP cells that expressed WT AR. Therefore, we developed the N-AR cell line, which expresses a WT AR harboring two tandem N-terminal epitope tags consisting of the streptavidin-binding peptide (SBP) and the minimal FLAG peptide sequences (SBP-AR) (Fig. 1*A*) (18, 19). The SBP epitope encodes a high-affinity streptavidin polypeptide sequence (*K_d_* = 2.5 nm) to facilitate the isolation of SBP-AR-interacting proteins from N-AR cells using streptavidin affinity chromatography (18). Western blotting analysis showed that SBP-AR is expressed in N-AR cells, which stably expressed the minimal SBP-FLAG polypeptide sequence, but not in LNCaP cells or the negative control (NC) cell line (Fig. 1*B*, *top*). The commercial monoclonal AR antibody AR441, which binds to a conserved epitope in AR-T887A and SBP-AR, confirmed that AR immunoreactivity was higher in N-AR cells than in LNCaP and NC cells (Fig. 1*B*, *bottom*). Overall, these results validated SBP-AR expression in N-AR cells.

Next, we wanted to test whether SBP-AR underwent ligand-dependent cytoplasmic-nuclear translocation similar to that in AR-T877A in androgen-sensitive LNCaP prostate tumor cells (20). Immunofluorescence (IF) microscopy was used to determine the subcellular localization of SBP-AR in androgen-depleted (AD) and androgen-stimulated (AS) N-AR cells (Fig. 1*C*). SBP-AR staining was restricted to the cytoplasmic space in vehicle-treated cells, phenocopying the subcellular localization of AR-T877A in LNCaP cells (Fig. 1*C*, *I*) (20). In contrast, robust nuclear SBP-AR staining was observed in 1-h androgen-treated cells (100 nm R1881) (Fig. 1*C*, *IV*), demonstrating that the ligand-dependent cytoplasmic-nuclear translocation of SBP-AR was preserved in N-AR cells. Concordant with the IF results, a cytoplasmic decrease and a corresponding nuclear increase in SBP-AR were observed in androgen-treated N-AR cells (Fig. 1*D*). These results showed that in N-AR cells, SBP-AR undergoes a ligand-dependent translocation from the cytoplasm to the nucleus that is identical to that of AR-T877A in LNCaP prostate tumor cells (20).

We then wanted to determine whether SBP-AR was functionally active and mediates transcription in N-AR cells. Therefore, N-AR cells were transfected with the androgen-responsive rat probasin luciferase reporter and treated with androgens to determine whether SBP-AR could mediate luciferase reporter expression similar to that of AR-T877A in LNCaP prostate tumor cells (21). To ablate background AR transcriptional activity encoded by AR-T877A in N-AR cells, we co-transfected the cells with siRNAs that target the 3′-UTR of *AR* to selectively knock down AR-T877A. SBP-AR-dependent expression of the luciferase reporter could then be measured in N-AR cells in the absence of AR-T877A. As predicted, siRNAs that targeted the *AR* coding regions greatly attenuated the expression of SBP-AR and AR-T877A in N-AR cells (Fig. 1*E*, compare *lanes 5* and *7*). In contrast, 3′-UTR AR siRNAs exclusively attenuated AR-T877A expression in N-AR cells (Fig. 1*E*, compare *lanes 5* and *8*). These results demonstrated that SBP-AR transcriptional activity could be measured in N-AR cells through selective knockdown of AR-T877A. To measure SBP-AR transcriptional activity in N-AR cells, LNCaP and N-AR cells were co-transfected with the probasin-luciferase reporter and control siRNAs or siRNAs targeting either the coding sequence of AR or its 3′-UTR (Fig. 1, *F* and *G*). As predicted, AR-dependent luciferase activity was strongly attenuated in LNCaP cells co-transfected with either the coding sequence- or 3′-UTR AR-directed siRNAs (Fig. 1*F*). Similarly, and as predicted, coding sequence-directed AR siRNAs strongly attenuated luciferase activity (*i.e.* ∼5%) in N-AR cells (Fig. 1*G*). However, attenuated luciferase activity was less pronounced (*i.e.* ∼40%) in N-AR cells transfected with the 3′-UTR AR siRNAs (Fig. 1*G*). This result suggested that ∼35% of AR transcriptional activity was dependent on SBP-AR after efficient knockdown of AR-T877A in N-AR cells. Overall, these results showed that SBP-AR undergoes ligand-dependent cytoplasmic-nuclear translocation, is transcriptionally activated by androgens in N-AR cells, and phenocopies AR-T877A functions in prostate tumor cells. Therefore, N-AR cells represent a valid cellular system in which to study SBP-AR-dependent functions in prostate tumor cells.

##### Quantitative Proteomics to Identify SBP-AR-interacting Proteins in N-AR Cells

**FIGURE 2.**
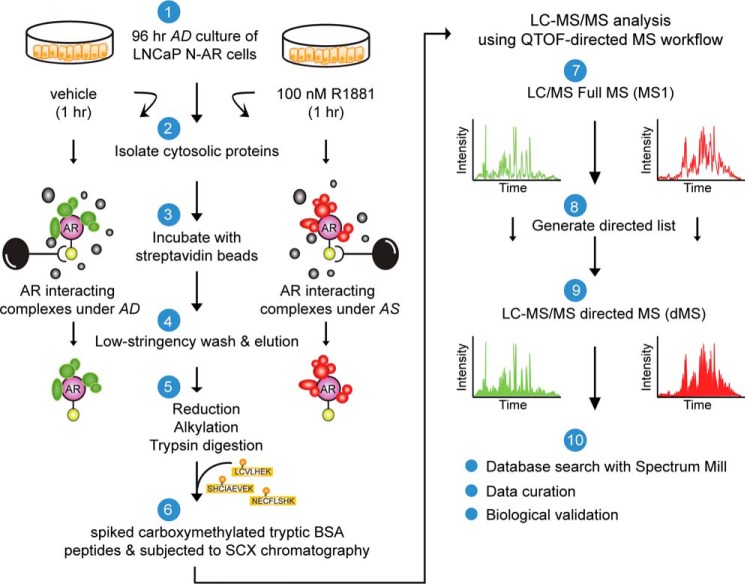
**Proteomics workflow for the identification of SBP-AR-interacting proteins.** Shown is the experimental platform for isolating cytosolic AR-interacting proteins from LNCaP cells stably expressing SBP-AR (N-AR). See “Experimental Procedures” for details of the purification workflow. Proteomic results are derived from one biological replicate.

**FIGURE 3.**
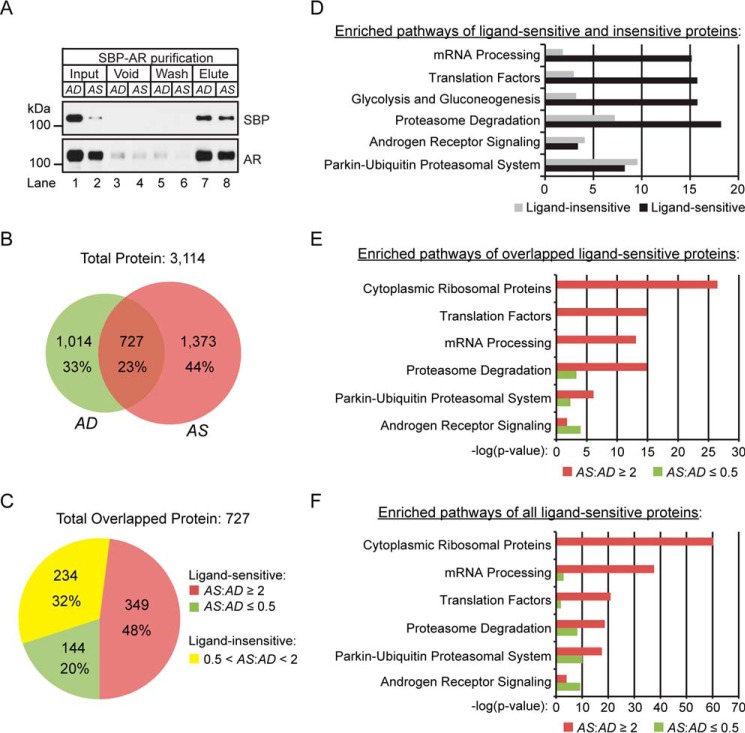
**Quantitative proteomics of ligand-sensitive proteins.** *A*, Western blotting analysis of copurified proteins to streptavidin beads under AD and AS conditions. Antibodies to SBP and AR were used to assess the efficiency of SBP-AR recovery in elution relative to input, void (unbound proteins), and wash fractions. 1% of each fraction was analyzed. *B*, Venn diagram summarizing the AD and AS proteins identified in the proteomic screen. *C*, a 2-fold difference in protein expression in the proteins showing overlap between the AD and AS samples was selected to distinguish between ligand-sensitive (*i.e.* AS/AD ratio ≤0.5 or AS/AD ratio ≥2) and ligand-insensitive (*i.e.* 0.5 < AS/AD ratio < 2) proteins. The distribution of the ligand-sensitive and -insensitive proteins is presented as a *pie chart. D–F*, WikiPathway analysis of ligand-sensitive and ligand-insensitive proteins (*D*), ligand-sensitive proteins in the AD (AS/AD ratio ≤0.5) and AS (AS/AD ratio ≥2) samples (*E*), and ligand-sensitive proteins with the inclusion of uniquely identified proteins in the AD and AS samples (*F*).

Next, we sought to identify SBP-AR-interacting proteins from N-AR cells because these proteins would both validate previously identified components of the AR-interactome and yield new ones. The bioanalytical approach involved isolating SBP-AR-interacting proteins using streptavidin affinity chromatography and the subsequent identification of copurified proteins using quantitative, label-free dMS (Fig. 2) (22–25). The experimental workflow consisted of the isolation of crude cytosolic protein extracts from androgen-starved (*i.e.* 96 h) N-AR cells that were challenged (*i.e.* 1 h) with vehicle (ethanol; AD) or androgen (100 nm R1881; AS). In essence, SBP-AR would be purified from the AD (*i.e.* inactive) and AS (*i.e.* active) cytosolic protein extracts to facilitate the purification of SBP-AR-interacting protein complexes under these treatment conditions. For the SBP-AR purification experiment, equal amounts of the AD and AS cytosolic protein extracts were subjected to streptavidin affinity chromatography involving low-stringency washes (to preserve the association of low-affinity SBP-AR-interacting proteins and protein complexes). The streptavidin affinity-purified samples were eluted, quantified, and subjected to Western blotting analysis for purification of SBP-AR. As shown in Fig. 3*A*, SBP-AR was efficiently purified from AD and AS cytosolic protein extracts. The remaining purified AD and AS samples were subjected to filter-assisted sample preparation to remove mass spectrometry-incompatible analytes (26). The samples were processed for tandem MS/MS using the dMS approach because this mass spectrometry-based workflow facilitates the in depth targeted sequencing of complex peptide mixtures through the utilization of preferred list peptide ions. Most importantly, the dMS approach, which closely follows a data-independent acquisition strategy, outperforms traditional data-dependent acquisition schemes for sequencing complex peptide mixtures using LC-MS/MS (22–25).

##### Network Analyses of Proteins Detected in Proteomic Screen

The dMS analyses resulted in 3,114 non-redundant protein identifications (false discovery rate <1%) across the AD and AS samples (Fig. 3*B*). This included 1,741 proteins in the AD sample and 2,100 proteins in the AS sample (Fig. 3*B*). A total of 727 proteins overlapped between the AD and AS samples, whereas 1,014 and 1,373 proteins were unique to the AD and AS samples, respectively (Fig. 3*B*). The results demonstrated a low degree of proteomic overlap between the AD and AS samples. Several factors may have contributed to this proteomic observation. First, cytosolic SBP-AR probably interacts with different types of proteins/protein complexes under the AD *versus* AS conditions because endogenous AR is localized primarily to the cytosolic compartment under androgen-depleted growth conditions, whereas AR undergoes dynamic cytoplasmic-nuclear recycling under androgen-stimulated growth conditions (27). Therefore, one would expect to identify many unique proteins across the AD and AS samples using mass spectrometry-based proteomic methods. Second, many sequenceable proteins in either sample may have been undersampled with the dMS approach. Although this was a plausible scenario, it was highly unlikely based upon the in depth tandem (MS/MS) sequencing power (*i.e.* targeted sequencing of preferred list peptide ions) of the dMS approach for comprehensive identification of proteins in complex peptide mixtures (22, 24, 25, 28). Initially, we applied a conservative bioinformatic evaluation of the proteomic findings by focusing on proteins that overlapped between the AD and AS samples. We sought to define “putative” ligand-sensitive proteins shared across the AD and AS populations in the proteomic screen. Due to the acute 1-h treatment with androgen, the proteomic screen would probably be enriched for cytosolic ligand-sensitive SBP-AR-interacting proteins. Therefore, a 2-fold protein expression ratio between the AD and AS samples was used to demarcate “ligand-sensitive” (*i.e.* AS/AD ratio ≤0.5 or AS/AD ratio ≥2) relative to “ligand-insensitive” (*i.e.* 0.5 < AS/AD ratio < 2) proteins. A total of 493 and 234 proteins were identified as ligand-sensitive and ligand-insensitive, respectively (Fig. 3*C*). The breakdown of ligand-sensitive proteins included 349 proteins enriched in the AS sample and 144 proteins enriched in the AD sample (Fig. 3*C*).

The purpose of our initial bioinformatic analysis was to determine whether there were any differences in functional protein networks represented by the ligand-sensitive and ligand-insensitive population of proteins. Therefore, both populations of proteins were uploaded into the WebGestalt bioinformatic program and subjected to WikiPathway analysis (Fig. 3*D*) (29). WikiPathway analysis of the six top-ranked pathways included mRNA processing, translation factors, glycolysis and gluconeogenesis, proteasome degradation, androgen receptor signaling, and the parkin-ubiquitin proteasomal system (Fig. 3*D*). With the exceptions of the androgen receptor signaling and parkin-ubiquitin proteasomal system protein networks, a greater level of enrichment in the four top-ranked protein networks was observed in the population of ligand-sensitive proteins (Fig. 3*D*). Overall, these findings suggested that the expression levels of putative SBP-AR-interacting proteins functionally representative of anabolic/catabolic processes were strongly influenced by acute exposure to androgens in N-AR cells.

Current models of ligand-mediated AR activation involve protein components, such as chaperones and motor proteins, that facilitate AR cytoplasmic-nuclear trafficking at the molecular level (11). This prompted us to determine whether ligand-sensitive proteins were enriched for protein networks involved in ligand-mediated AR activation. WikiPathway analysis identified cytoplasmic ribosomal proteins, translation factors, mRNA processing, proteasome degradation, the parkin-ubiquitin proteasomal system, and androgen receptor signaling as the six top-ranked pathways shared between the AD and AS samples (Fig. 3*E*). With the exception of the androgen receptor signaling pathway in the six top-ranked pathways, a greater level of enrichment of ligand-sensitive proteins was observed in the AS sample relative to the AD sample. Importantly, enriched networks in the AS sample were concordant with known biochemical processes that modulate AR function(s) at the molecular level in prostate tumor cells. For example, the proteasome system has an active role in androgen-dependent AR transcription, AR trafficking, and AR metabolism in prostate tumor cells (30). Similarly, the enrichment of cytoplasmic ribosomal proteins in the AS sample is corroborated by the finding that androgens acutely (*i.e.* within hours) stimulate ribosomal RNA synthesis and ribosome biogenesis through an AR-dependent mechanism in prostate tumor cells (31, 32).

To expand our understanding of the functional protein networks beyond the existence of overlap in the proteins identified in the proteomic screen, we performed a WikiPathway analysis on unique proteins observed in the AD and AS samples. This bioinformatic analysis tested whether the functional protein networks among the unique proteins were conserved with or distinct from those represented by the ligand-sensitive, overlapped proteins (Fig. 3*F*). Similar to ligand-sensitive overlapped proteins, the top-ranked six pathways included cytoplasmic ribosomal proteins, mRNA processing, translation factors, proteasome degradation, parkin-ubiquitin proteasomal system, and androgen receptor signaling (Fig. 3*F*). Although the rank order for protein networks encoded by translation factors and mRNA processing were reversed relative to ligand-sensitive overlapping proteins (Fig. 3*E*), the bioinformatic analysis demonstrated that the composition of enriched protein networks that copurified with SBP-AR was unaffected by the inclusion of unique proteins in either the AD or AS sample. Therefore, ligand-sensitive proteins, both overlapping and unique in the AD and AS samples, were included in subsequent protein network analyses.

##### Network Analyses of the AR-interactome

**FIGURE 4.**
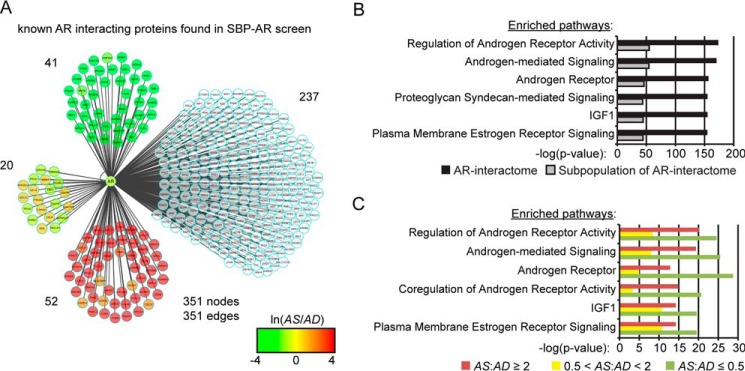
**Protein network analyses of the AR-interactome.** *A*, Cytoscape interaction network of 351 known AR-interacting proteins reported in the HPRD/BIOGRID/McGill/STRING databases. The protein interaction network was built in Cytoscape, and nodes were *color-coded* for proteins identified in the proteomic screen, to define the peptide intensity changes in the presence (*red*) or absence (*green*) of androgens. Proteins not found in the proteomic screen are not colored. *B*, WikiPathway analysis of known AR-interacting proteins for top differentially ranked pathways enriched in the AR-interactome and the subpopulation of the AR-interactome identified in the proteomic screen. *C*, WikiPathway analysis of the ligand-sensitive (*i.e.* AS/AD ratio ≤0.5 or AS/AD ratio ≥2) and ligand-insensitive (*i.e.* 0.5 < AS/AD ratio < 2) AR-interactomes identified in the proteomic data set.

Next, we wanted to determine whether members of the AR-interactome were enriched in the proteomic screen. A conservative analysis of the scientific literature and protein databases suggested that the AR-interactome is composed of ∼351 proteins (*i.e.* HPRD, BIOGRID, McGill, and STRING) (13–16). Our proteomic screen identified 113 components of the AR-interactome (Fig. 4*A* and Table 1), which demonstrated that ∼32% of the AR-interactome was observed across the AD and AS samples. Based upon the detection of ∼12,000 human proteins in cell lines/tissues using state-of-the-art mass spectrometry methodologies, this finding showed that a significant fraction of the AR-interactome was detected in the proteomic screen (*i.e.* Fisher's exact test, *p* = 7e−3; Table 1) (33). The majority of the AR-interactome components were androgen-sensitive (*i.e.* 93 of 113 proteins), with 52 enriched in the AS sample, 41 enriched in the AD sample, and 20 categorized as androgen-insensitive (Fig. 4*A* and Table 1). Importantly, sensitivity to androgen was higher in the AR-interactome than across all other proteins detected in the proteomic screen (*i.e.* Fisher's exact test, *p* = 5e−4; Table 1). These results demonstrated that a significant fraction of the AR-interactome was detected in the proteomic screen. Moreover, our findings showed that the AR-interactome was ligand-sensitive, suggesting that ligand-sensitive proteins in the data set could be enriched for novel SBP-AR-interacting proteins.

**TABLE 1:** **Streptavidin-copurifying protein statistics** Numbers in parentheses indicate proteins found in both vehicle- and androgen-treated samples.

Parameters	Values
SwissProt mass spectrometry-detectable human proteins	∼12,000
Known AR-interacting protein databases: HPRD/BIOGRID/McGill/STRING	351
Total non-redundant protein identifications	3,114
Ligand-sensitive proteins (AS/AD ≤0.5 or AS/AD ≥2.0)	2,880
Ligand-insensitive proteins (0.5 < AS/AD < 2.0)	234
Known AR-interacting proteins found in proteomic screen	113
AS/AD ≤0.5	41 (8)
AS/AD ≥2.0	52 (21)
0.5 < AS/AD < 2.0	20 (20)
Enrichment of known AR-interacting proteins found in proteomic screen	113/351 (Fisher's exact test; *p* = 7e − 3)
Known AR interactions found were more androgen-sensitive than -insensitive	Fisher's exact test; *p* = 5e − 4

Next, the top-ranked pathways between the AR-interactome and the subpopulation of the AR-interactome identified in the proteomic screen were compared to determine whether specific functional protein networks related to AR function were selectively enriched in the proteomic screen (Fig. 4*B*). The six top-ranked pathways identified by the WikiPathway analysis included regulation of androgen receptor activity, androgen-mediated signaling, androgen receptor, proteoglycan syndecan-mediated signaling, IGF1, and plasma membrane estrogen receptor signaling (Fig. 4*B*). The AR-interactome was enriched at higher levels for all of the top-ranked networks relative to the subpopulation of the AR-interactome identified in the proteomic screen. Thus, the subpopulation of the AR-interactome detected in the proteomic screen was not enriched for protein networks related to AR function relative to the entire AR-interactome.

Last, we examined whether specific protein networks related to AR function were selectively enriched under the androgen-depleted (*i.e.* AD sample) or androgen-stimulated (*i.e.* AS sample) conditions. WikiPathway analysis of the six top-ranked pathways identified regulation of androgen receptor activity, androgen-mediated signaling, androgen receptor, coregulation of androgen receptor activity, IGF1, and plasma membrane estrogen receptor signaling (Fig. 4*C*). There was greater enrichment for the top-ranked pathways in the AD sample, which suggests that these protein networks may be active under conditions of androgen depletion to modulate AR function in prostate tumor cells (Fig. 4*C*). This result was concordant with the enrichment of the androgen signaling pathway among ligand-sensitive proteins in the AD sample (Fig. 3, *E* and *F*). Overall, the bioinformatic analyses demonstrated that the proteomic screen enriched for the AR-interactome and molecular pathways related to AR function in prostate tumor cells.

##### Molecular Topology of the AR-interactome Detected in the Proteomic Screen

**FIGURE 5.**
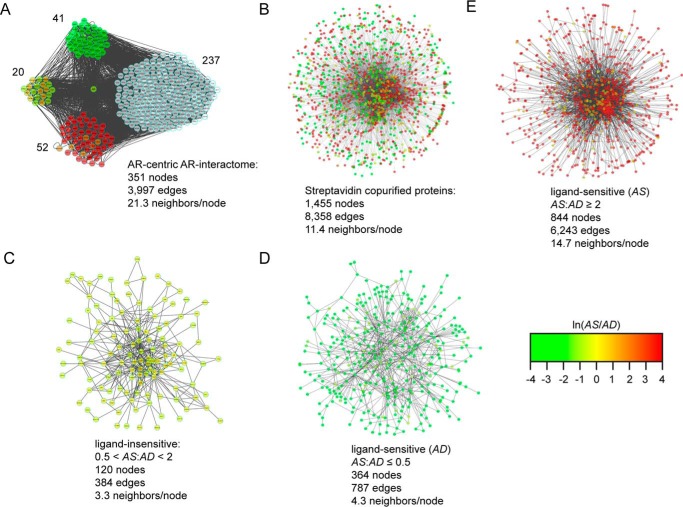
**Molecular topology of streptavidin-copurified proteins.** *A*, AR-centric protein interaction network of the AR-interactome identified in the proteomic data set. The network was analyzed by PINA and visualized in Cytoscape. *B–E*, forced directed graphs of streptavidin-copurified proteins (*B*), ligand-insensitive (*i.e.* 0.5 < AS/AD ratio < 2) proteins (*C*), ligand-sensitive AD proteins (*i.e.* AS/AD ratio ≤0.5) (*D*), and ligand-sensitive AS proteins (*i.e.* AS/AD ratio ≥2) (*E*). The numbers of nodes and edges within each network are indicated, and nodes are *color-coded* to define peptide intensity changes in the presence (*red*) or absence (*green*) of androgens.

Next, we sought to elucidate the molecular topology of protein-protein interaction (PPI) networks represented by the AR-interactome relative to proteins identified in the proteomic screen. This bioinformatic analysis was expected to achieve three goals. First, it would resolve the molecular composition and highlight the connectivity between the AR-interactome and proteins identified in the proteomic screen. Second, it would facilitate the comparative analysis of PPI networks between the AR-interactome and proteins identified in the proteomic screen. Third, it would enable us to explore differences in the molecular topology of PPI networks related to AR function between the androgen-depleted (*i.e.* AD sample) and androgen-stimulated (*i.e.* AS sample) conditions. PPI networks of the AR-interactome and proteins in the proteomic screen were constructed using the Protein Interaction Network Analysis (PINA) program and visualized with the Cytoscape software program (34–36). The AR-interactome consisted of 351 nodes with 3,997 edges (Fig. 5*A*), whereas the proteomic screen contained 1,455 nodes with 8,358 edges (Fig. 5, *A* and *B*). NetworkAnalyzer (35) showed that the AR-interactome contained ∼21 neighbors/node (Fig. 5*A*), whereas the proteomic screen contained ∼11 neighbors/node (Fig. 5*B*). This finding showed that there was a higher degree of connected neighbors in the PPI network of the AR-interactome relative to proteins detected in the proteomic screen. We constructed PPI networks of ligand-insensitive and ligand-sensitive interactomes to further characterize the molecular topology of PPIs among this subpopulation of proteins in the proteomic screen (Fig. 5, *C–E*). Furthermore, the ligand-sensitive interactome was separated into the AD and AS interactome to provide greater resolution of PPI networks between the androgen-depleted and androgen-stimulated conditions (Fig. 5, *D* and *E*). The ligand-insensitive interactome contained 120 nodes with 384 edges with ∼3 neighbors/node (Fig. 5*C*). Similarly, ∼4 neighbors/node were computed for the AD interactome, which consisted of 364 nodes and 787 edges (Fig. 5*D*). In contrast, ∼14 neighbors/node were detected in the AS interactome, which displayed 844 nodes and 6,243 edges (Fig. 5*E*). These results showed that the AS interactome contained a higher degree of connected edges and highlighted the complexity of PPIs between the AD and AS interactomes in the proteomic screen. Overall, the results suggested that androgens facilitated the recovery of more highly connected PPI networks among proteins detected in the proteomic screen.

##### Network Analyses of Protein Interaction Modules

**FIGURE 6.**
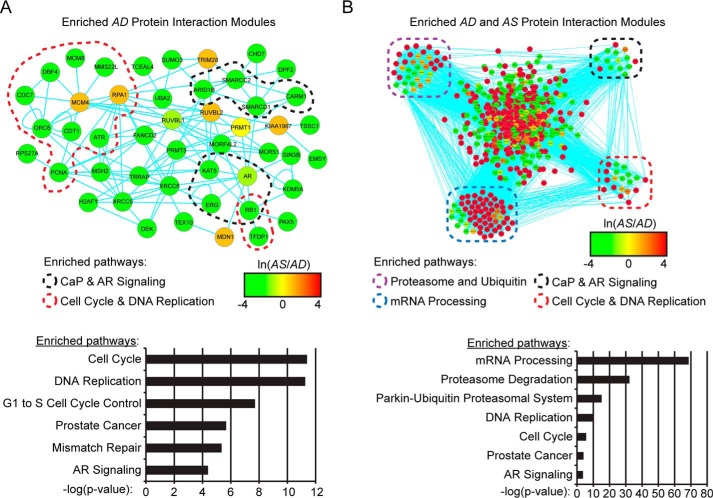
**Protein interaction network module analysis of streptavidin-copurified proteins.** *A* and *B*, protein interaction network module analysis of the AD and AS samples for enrichment of AR-containing protein interaction modules. Two AR-containing protein modules were enriched in the AD sample, with one also enriched in the AS sample. The proteins from the protein module analysis were analyzed by PINA and visualized in Cytoscape. WikiPathway analyses (*bottom panels*) were then carried out to identify the top pathways enriched in the protein interaction modules, and proteins belonging to defined pathways were manually drawn. Nodes were *color-coded* to define peptide intensity changes in the presence (*red*) or absence (*green*) of androgens.

Next, ligand-sensitive PPIs with SBP-AR were explored to determine whether protein interaction network (PIN) modules functionally linked to androgen-mediated AR activation were enriched in the proteomic screen. Therefore, the WebGestalt Protein Interaction Network Module Analysis program was used to identify statistically significant PIN modules enriched in either the AD or AS samples (37). PIN modules selected for further study included those that contained AR as a node because these PIN modules would be physically connected to AR at the molecular level. AR was detected in two PIN modules in the AD sample (*i.e.* protein module 160, *p* = 0.0178; protein module 39, *p* = 0.0257) (Fig. 6, *A* and *B*). Interestingly, the AS sample contained a single PIN module (*i.e.* protein module 39, *p* = 1.83e−10), and it was identical to the PIN module detected in the AD sample (Fig. 6*B*). To elucidate biological pathways associated with each PIN module, the proteins represented by PIN module 160, detected in the AD sample, and PIN module 39, detected in both the AD and AS samples, were subjected to WikiPathway analysis (Fig. 6, *A* and *B*). The pathways enriched in PIN module 160 of the AD sample included prostate cancer/AR signaling pathways and cell cycle/DNA replication pathways (Fig. 6*A*). These same pathways were enriched in PIN module 39, but this module was also enriched for the proteasome/ubiquitin and mRNA processing pathways (Fig. 6*B*). These results were concordant with the finding that under androgen-stimulated conditions (*i.e.* AS sample), protein modules related to the proteasome/ubiquitin, mRNA processing, and AR signaling pathways were enriched in the proteomic screen (Fig. 3, *E* and *F*).

**FIGURE 7.**
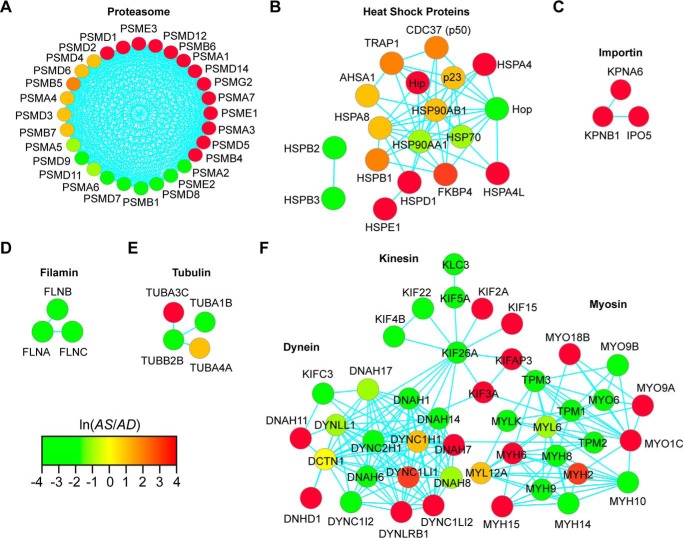
**Representative protein complexes involved in AR signaling and intracellular protein transport found in the proteomics data set.** The protein complexes included proteasome (*A*), heat shock proteins (*B*), importin (*C*), filamin (*D*), tubulin (*E*), and dynein, kinesin, and myosin (*F*). Nodes are *color-coded* to define the peptide intensity changes in the presence (*red*) or absence (*green*) of androgens.

Next, proteins detected in the proteomic screen were manually curated to highlight protein complexes involved in the early steps of ligand-mediated SHR activation. The protein classes selected for further annotation included the molecular chaperones (*i.e.* hsp90 and immunophilins) (11), cytoskeletal motor proteins (*i.e.* dynein, kinesin, and myosin) (38, 39), cytoskeletal proteins (*i.e.* tubulin and filamin) (40–42), and the proteasome and functionally related enzymes (30, 43–46). PPI networks representative of each protein class were generated with the PINA program and visualized as force-directed graphs with Cytoscape (Fig. 7). Previous studies showed that the proteasome is involved in the intracellular trafficking and transcriptional function of multiple SHRs, such as the estrogen receptor (ER), glucocorticoid receptor, and AR (30, 47, 48). Interestingly, several subunits of the catalytic (*i.e.* 12 of 32 total) and regulatory (*i.e.* 12 of 19 total) complexes of the 26S proteasome were enriched in the AD and AS samples (49). Notably, PSMA7, a subunit of the catalytic 20S core proteasome that potentiates AR-mediated transcription when overexpressed in prostate tumor cells (30), was enriched in the AS sample (Fig. 7*A*). The molecular chaperones interact with unliganded SHRs in the cytosolic compartment, as inactive protein complexes, to modulate SHR function at the molecular level (11). Many of the molecular chaperones were enriched in the AD and AS samples to verify their categorization as ligand-sensitive proteins (Fig. 7*B*). The next class of ligand-sensitive proteins included the karyopherins, which mediate the transport of molecules between the cytoplasm and the nucleus (50). Both karyopherin importin α (KPNA6) and karyopherin importin β (KPNB1) were enriched in the AS sample (Fig. 7*C*) and are known to mediate cytoplasmic nuclear AR trafficking (12, 51, 52). The cytoskeletal proteins filamin and tubulin are coregulators of AR-mediated transcription (39–42) and have been molecularly linked to ligand-mediated SHR activation (41). Interestingly, both cytoskeletal proteins were found to be ligand-sensitive in the proteomic screen (Fig. 7, *D* and *E*). The last group of proteins examined included the motor proteins, which mediate vesicle trafficking and organelle transport but have also been linked to ligand-mediated SHR activation (38, 39, 53). The motor protein families included the dyneins, myosins, and kinesins. Remarkably, many of the protein isoforms in each protein family were enriched in the AD and AS samples (Fig. 7*F*). Notably, the dynein isoforms, which are minus-end-directed motor proteins, were enriched in the AD and AS samples. The discordant enrichment of dynein protein isoforms was also observed for the plus-end-directed motor proteins kinesin and myosin (Fig. 7*F*). Of note, myosin VI (MYO6) is a minus-end-directed motor protein that binds AR, regulates AR stability, and modulates AR-dependent transcription in prostate tumor cells (38). Overall, these results show that known modulators of SHR activation were ligand-sensitive proteins in the proteomic screen. Moreover, the results probably suggest that ligand-mediated SHR activation is coordinated through the actions of isoform-specific modulators in prostate tumor cells.

##### Functional Screen to Identify Novel Modulators of AR-mediated Transcription

**FIGURE 8.**
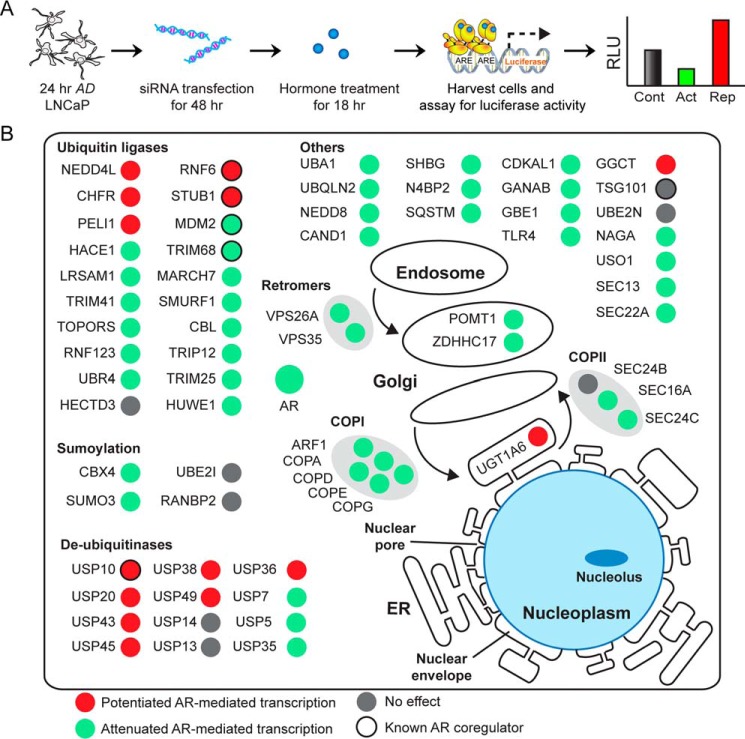
**siRNA luciferase screen to identify modulators of AR-mediated transcription.** *A*, diagram of siRNA luciferase screen. 24-h AD LNCaP cells were co-transfected with the probasin-luciferase and pRLSV40-*Renilla* vectors and either control or experimental siRNAs (100 nm) for 48 h. Vehicle (ethanol) or androgen (1 nm R1881) was then added to the cells, and luciferase readings were measured 18 h later. *B*, cellular visualization of proteins that were subjected to siRNA luciferase screen to monitor AR transcriptional activity. Nodes are *colored* in *red* and *green* to denote whether siRNA knockdown of the target proteins potentiated or attenuated, respectively, AR-mediated transcription. *Gray nodes* indicate that siRNA knockdown did not perturb AR-mediated transcription. Nodes *outlined* with *black* indicate that previous studies showed the protein to be an AR coregulator. Results are presented as the mean ± S.D. (*error bars*) of three biological replicates (*n* = 3). *Asterisks* indicate significant differences between cells transfected with target and control siRNAs (Student's *t* test; *, *p* < 0.05). RLU, relative luciferase units.

Next we wanted to test whether ligand-sensitive proteins could modulate AR-dependent transcription because this group of proteins is likely enriched for SBP-AR-interacting proteins. Thus, these molecules are predicted to modulate AR transcriptional activity in prostate tumor cells. Therefore, an siRNA-based transcriptional screen in LNCaP cells was utilized to test whether ligand-sensitive proteins had any effect (*i.e.* attenuation or potentiation) on AR-dependent transcription (21). Proteins involved in protein trafficking were targeted for siRNA-mediated knockdown because a functional relationship between this class of proteins and AR-dependent transcription has yet to be fully established at the molecular level in prostate tumor cells. Thus, 24-h androgen-depleted LNCaP cells were co-transfected with the probasin-luciferase reporter and experimentally validated siRNAs for 48 h. The co-transfected cells were challenged with vehicle (*i.e.* ethanol) or androgen (*i.e.* 1 nm R1881) for 18 h, and measured luciferase activity was compared between control and target siRNA knockdown cells (Fig. 8*A* and supplemental Table 3). As expected, luciferase activity was strongly attenuated in cells transfected with AR siRNAs, which demonstrated that probasin-luciferase expression/activity was AR-dependent in LNCaP cells (Fig. 8*A*). Similar to AR siRNA-transfected cells, the majority of experimental siRNAs tested (*i.e.* 38 of 56 total; Table 2) strongly attenuated luciferase activity in LNCaP cells (Fig. 8*B*). However, a number (*i.e.* 12 of 56 total; Table 2) of siRNAs also potentiated luciferase activity (Fig. 8*B*). For example, siRNAs directed against COPI, which controls retrograde protein trafficking (54), and coatomer II (COPII), which regulates anterograde protein trafficking (55), strongly attenuated luciferase activity. Similarly, luciferase activity was strongly attenuated by siRNAs directed against the retromer complex, which is involved in endosome/trans-Golgi trafficking (56). We also targeted components of the ubiquitination/SUMOylation pathways because these enzymes strongly affect AR metabolism and function in prostate tumor cells (57). For example, E3 protein ligases are known AR coregulators (58–61), and, as predicted, siRNAs targeted against the majority of the E3 ligases attenuated luciferase activity (Fig. 8*B*). In contrast, siRNAs targeting the deubiquitinases had the opposite effect and potentiated luciferase activity. Additionally, siRNAs that targeted enzymes involved in the maturation and post-translational processing of both plasma membrane and membrane-associated receptors strongly modulated luciferase activity (Fig. 8*B*). For example, cells transfected with siRNAs against mannosyltransferase POMT1, which mediates serine and threonine protein mannosylation (62), and the palmitoyltransferase ZDHHC17, which mediates the protein palmitoylation (63, 64), strongly attenuated luciferase activity (Fig. 8*B*). As expected, more in depth molecular studies will be required to establish a direct functional link between protein trafficking and AR-mediated transcription in prostate tumor cells. Nonetheless, the siRNA-based transcriptional screen revealed that the expression of ligand-sensitive proteins was required for optimal AR transcriptional activity in LNCaP prostate tumor cells.

**TABLE 2:** **Statistics of predicted modifiers**

Protein group	siRNA knockdown-modulated AR transcriptional activity (*p* < 0.05)	Coactivator-like activity	Corepressor-like activity	No activity
Ligand-sensitive				
AS/AD ≤0.5	17/18 (94%)	12/18 (66%)	5/18 (28%)	1/18 (6%)
AS/AD ≥2.0	24/28 (86%)	17/28 (61%)	7/28 (25%)	4/28 (14%)
Ligand-insensitive				
0.5 < AS/AD < 2.0	9/10 (90%)	9/10 (90%)	0/10 (0%)	1/10 (10%)
Total	50/56 (89%)	38/56 (68%)	12/56 (21%)	6/56 (11%)

##### Validation of Ligand-sensitive COPI Interaction with AR

**FIGURE 9.**
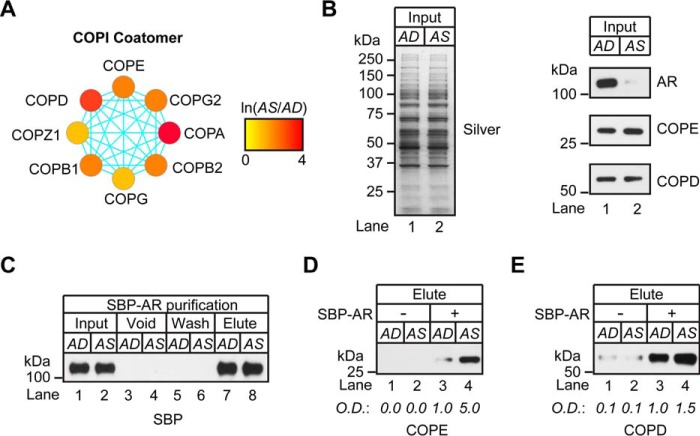
**The COPI complex copurified with SBP-AR.** *A*, protein interaction network of the COPI coatomer complex that copurified with the SBP-AR. Nodes are *color-coded* to define relative expression of the COPI complex that copurified with SBP-AR in the presence (*red*) or absence (*green*) of androgens. *B*, silver stain (*left*) and Western blotting (*right*) analyses of cytosolic proteins extracted from AD and AS LNCaP cells as input for streptavidin affinity purification. *C–E*, Western blotting analysis of the *in vitro* binding assay developed to validate ligand-sensitive interactions between SBP-AR and the COPI complex. Recombinant SBP-AR (rSBP-AR) was incubated with cytosolic extracts derived from AD and AS LNCaP cells and subjected to streptavidin chromatography with the addition of vehicle (ethanol) or androgen (100 nm R1881) for AD and AS samples, respectively. *C*, efficiency of the purification, as determined by Western blotting analysis of the input, void, wash, and eluted samples using antibodies to SBP. *D* and *E*, the eluted samples were subjected to Western blotting analysis using antibodies to COPE and COPD, and results were compared with those for samples without the addition of rSBP-AR. Western blotting results are representative of two biological replicates. Densitometry values are indicated *below* the blots.

Next, experiments were undertaken to validate interactions between SBP-AR and ligand-sensitive proteins observed in the proteomic screen. Components of the COPI complex were selected for further study because each subunit was enriched in the AS sample (Fig. 9*A*). Moreover, COPI siRNAs strongly attenuated AR-dependent transcription in LNCaP cells (Fig. 8*B*). Interactions between SBP-AR and the COPD and COPE subunits were selected for further study due to the availability of antibody reagents to detect their expression in LNCaP cells (Fig. 9*B*). An *in vitro* binding assay was developed to validate ligand-sensitive interactions between SBP-AR and the components of the COPI complex. For the assay, recombinant SBP-AR (rSBP-AR) was incubated with cytosolic extracts derived from AD and AS cells and subjected to streptavidin affinity chromatography, as performed in the original proteomic experiment (Fig. 2). As expected, rSBP-AR efficiently bound to the streptavidin beads in either the AD or AS sample because equivalent levels of rSBP-AR were detected across both samples after denaturation elution (Fig. 9*C*, compare *lanes 1* and *2* with *lanes 7* and *8*). Western blotting analysis showed that COPD and COPE levels were equivalent between the AD and AS samples before the addition of rSBP-AR (Fig. 9*B*, compare *lanes 1* and *2*). However, after the samples were subjected to streptavidin affinity chromatography, both COPD and COPE subunits were enriched in the AS sample relative to the AD sample (Fig. 9, *D* and *E*). These results showed that rSBP-AR interactions with the COPE and COPD subunits are enhanced in the presence of androgens (Fig. 9, *D* and *E*), and thus a ligand-sensitive interaction exists between rSBP-AR and components of the COPI complex. They further suggest that the proteomic screen identified ligand-sensitive protein interactions with SBP-AR.

##### Co-fractionation of AR with the Golgi-enriched Protein Fraction

**FIGURE 10.**
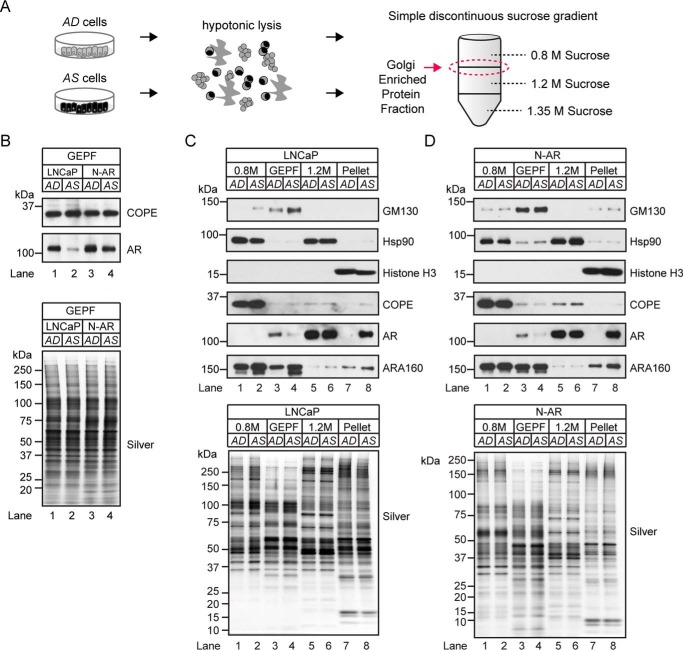
**Androgen-dependent mobilization of AR with the Golgi-enriched protein fraction.** *A*, workflow for isolating Golgi-enriched membranes from AD and AS prostate tumor cells using a simple discontinuous sucrose gradient. *B*, Western blotting analysis of GEPF from LNCaP and SBP-AR cells grown under AD or AS conditions. The samples were analyzed by immunoblotting with antibodies to AR and COPE. Silver staining (*bottom*) demonstrated equal sample loading across the lanes. *C* and *D*, Western blotting analysis of Golgi membrane proteins extracted from LNCaP (*C*) or N-AR cells (*D*) grown under AD or AS conditions. The samples were subjected to SDS-PAGE and immunoblotting analysis with antibodies to GM130, Hsp90, histone H3, COPE, AR, and ARA160. Silver-stained gel (*bottom panels*) shows equivalent sample loading between samples. Western blotting results are representative of two biological replicates. The percentage values of total AR based on densitometry values normalized to the silver-stained gels are indicated *below* the blots.

A biochemical association between SBP-AR and the COPI subunits might suggest that the AR cytoplasmic-nuclear translocation process was physically coupled to the COPI retrograde trafficking complex. Although AR co-localization to the Golgi apparatus has yet to be established, the protein ARA160 has been shown to colocalize to the Golgi and the nucleus (65), and it is the first N-terminal domain coactivator of AR-mediated transcription (66). These findings prompted us to examine AR for an association with the Golgi apparatus and to determine whether such an association is ligand-dependent in parental LNCaP cells and N-AR cells. Discontinuous centrifugation was performed on LNCaP cells and N-AR cells grown under AD and AS conditions to identify proteins associated with the Golgi apparatus (Fig. 10*A*). Western blotting analysis showed the COPE subunit associated with the Golgi-enriched protein fraction (GEPF; Fig. 10*B*), which validated the biochemical integrity of the Golgi-enriched protein in this sample. Notably, COPE levels were unchanged between the AD and AS sample in both LNCaP and N-AR cells (Fig. 10*B*), demonstrating that acute androgen exposure had no measurable effect on COPE levels in the GEPF. Surprisingly, AR was also detected in the GEPFs of LNCaP and N-AR cells (Fig. 10*B*), suggesting that it may be associated with the Golgi compartment in prostate tumor cells. Moreover, AR levels in the AS sample were noticeably reduced relative to those in the AD sample (Fig. 10*B*), which also showed that AR levels at the GEPF were sensitive to androgens.

Next, AR levels in the GEPF and other sucrose gradient-derived protein fractions were compared to determine whether the subpopulation associated with the Golgi in LNCaP and N-AR cells is relatively large or small. In addition to the GEPF, the 0.8 m sucrose protein fraction (*i.e.* microsomal), the 1.2 m sucrose protein fraction (*i.e.* soluble cytosolic and associated with the heavier membranes of the ER and nucleus), and pellet protein fraction (*i.e.* nucleoplasmic) were subjected to Western blotting analysis to verify subcellular marker protein expression in LNCaP and N-AR cells (Fig. 10, *C* and *D*). As expected, the Golgi marker protein GM130 was detected in the GEPF (Fig. 10, *C* and *D*, *lanes 3* and *4*), the molecular chaperone Hsp90 was detected in the 0.8 and 1.2 m sucrose protein fractions (Fig. 10, *C* and *D*, *lanes 1*, *2*, *5*, and *6*), and the nuclear marker histone H3 was restricted to the pellet sucrose protein fraction (Fig. 10, *C* and *D*, *lanes 7* and *8*). These results verified the subcellular composition of the discontinuous sucrose gradient fractions. Interestingly, robust COPE expression was detected in the 0.8 m sucrose protein fraction, with expression nearly undetectable in the GEPF (Fig. 10, *C* and *D*, compare *lanes 1* and *2* with *lanes 3* and *4*). This comparative Western blotting analysis demonstrated that only a small fraction (*i.e.* ∼8–13%) of COPE is present in the GEPF compared with the 0.8 m sucrose protein fraction (Fig. 10, compare Western blots in *B* to those in *C* and *D*). Western blotting analysis of ARA160 showed that it was distributed across the 0.8 m, GEPF, and pellet sucrose protein fractions (Fig. 10, *C* and *D*). Interestingly, higher levels of ARA160 were detected in the AS sample, which showed that ARA160 levels and/or subcellular compartmentalization were androgen-sensitive in LNCaP and N-AR cells (*i.e.* 1 h) (Fig. 10, *C* and *D*, compare *lanes 7* and *8*). Similar to COPE, AR was nearly undetectable in the GEPF (Fig. 10, *C* and *D*, *lanes 3* and *4*) but was robustly detected in the 1.2 m and pellet sucrose protein fractions (Fig. 10, *C* and *D*, *lanes 5–8*). These findings showed that AR was present in the GEPF, albeit in a smaller quantity (*i.e.* 5–29% of total AR) than the AR present in the 1.2 m and pellet sucrose protein fractions. Moreover, as was the case for ARA160, levels of AR were higher in the nuclear pellet of the AS sample. This finding was in agreement with androgen's role in increasing AR levels in the nuclear compartment through AR cytoplasmic-nuclear translocation. Overall, our biochemical findings are suggestive of a ligand-dependent association between AR and the Golgi compartment in prostate tumor cells.

##### AR Transcriptional Activity Requires the COPI Complex

**FIGURE 11.**
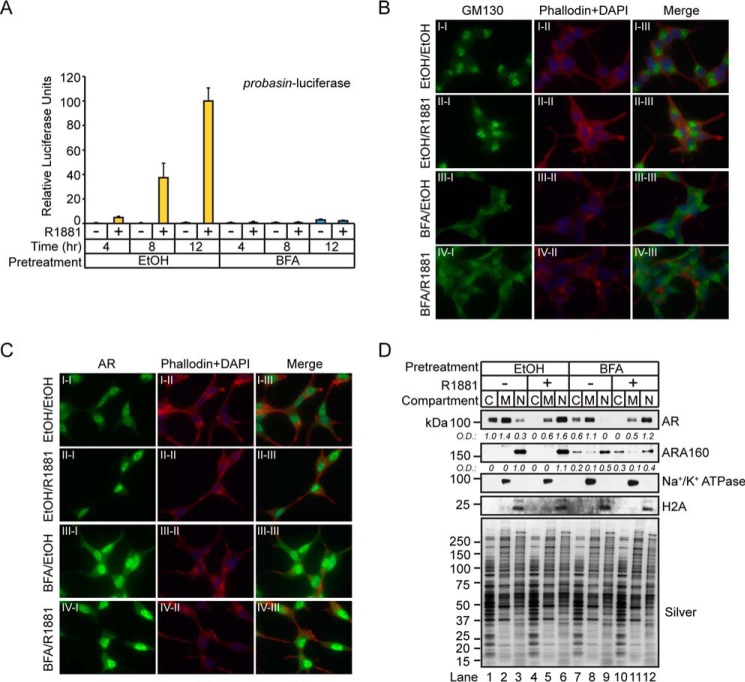
**Pharmacological inhibition of intracellular trafficking machinery disrupts AR transcriptional activity by blocking nuclear localization of ARA160.** *A*, LNCaP cells cultured in AD medium for 96 h were pretreated with vehicle (ethanol) or BFA (50 μm) for 30 min. After one wash with DPBS, the cells were treated with vehicle (ethanol) or androgen (100 nm R1881) for 1 h, followed by IF staining using AR antibodies (*green*) and co-staining with phalloidin (*red*) and DAPI (*blue*). *B*, cells prepared as in *A* and stained with GM130 antibodies. *C*, LNCaP cells cultured in AD medium for 24 h were transfected with the probasin-luciferase and pRLSV40-*Renilla* vectors for 48 h and then pretreated with vehicle (ethanol) or BFA (50 μm) for 30 min. After one DPBS wash, cells were treated with vehicle (ethanol, −) or androgen (1 nm R1881, +) for 4, 8, or 12 h. Total cell lysates were measured for Dual-Luciferase activity, and results are presented as mean ± S.D. (*error bars*) of three biological replicates (*n* = 3). *Asterisks* indicate statistical significance between ethanol- and BFA-treated samples (*i.e.* 4 h to 4 h, 8 h to 8 h, and 12 h to 12 h) in response to androgen (Student's *t* test; *, *p* < 0.05). *D*, cytosolic (*C*), membrane (*M*), and nuclear (*N*) proteins were isolated from LNCaP cells treated as described in *A*, and then analyzed by Western blotting with the indicated antibodies. Silver-stained gel shows equivalent sample loading between samples. IF and Western blotting results are representative of two biological replicates. Densitometry values were normalized to the silver-stained gel.

Next, we wanted to test whether disruption of the Golgi apparatus had any effect on AR-mediated transcription in prostate tumor cells. First, pharmacological disruption of protein trafficking at the Golgi apparatus would be tested to determine whether it had any impact on AR transcriptional activity in LNCaP cells. The drug brefeldin A (BFA) was selected for this experiment because it binds to and inhibits the activation of ARF1-guanine exchange factors to promote the disassembly of the COPI coat and the subsequent disruption of COPI retrograde trafficking to the Golgi (67). The experiment involved briefly treating androgen-depleted LNCaP cells, in which the majority of AR would be inactive in the cytosolic compartment, with BFA to disrupt the Golgi apparatus. After the BFA was removed, the cells were stimulated with androgens to determine whether AR transcriptional activity was preserved. This experiment was designed to directly test whether AR-mediated transcription is affected in LNCaP cells where COPI retrograde trafficking is acutely disrupted by BFA. More specifically, androgen-depleted LNCaP cells were transfected with the probasin-luciferase vector for 24 h, pretreated with BFA (*i.e.* 50 μm) for 30 min, washed to remove BFA, and then challenged with vehicle (*i.e.* ethanol) or androgen (*i.e.* 1 nm R1881) for 4, 8, or 12 h. As predicted, androgens increased luciferase activity in a time-dependent manner in vehicle-pretreated cells (Fig. 11*A*). However, in BFA-pretreated cells, luciferase activity was completely abolished in androgen-treated cells (Fig. 11*A*). These results showed that the time-dependent increase in androgen-mediated AR transcriptional activity was disrupted by BFA in LNCaP cells. These findings support a functional role of the COPI complex in the process of androgen-mediated AR-dependent transcription in LNCaP prostate tumor cells.

Next, we explored whether cytoplasmic-nuclear AR translocation was disrupted in BFA-treated cells because this molecular process could be functionally coupled to COPI-mediated retrograde protein trafficking. If these molecular pathways were coupled directly or indirectly, it could explain why BFA strongly attenuated AR transcriptional activity by disrupting AR nuclear localization in LNCaP cells. IF microscopy experiments were performed to determine whether androgen-mediated cytoplasmic-nuclear AR trafficking was disrupted in BFA-treated cells (Fig. 11, *B* and *C*). For the IF experiments, 72-h androgen-depleted LNCaP cells were incubated with BFA for 30 min, washed, and challenged with vehicle or androgen (*i.e.* 1 nm R1881) for 1 h. The cells were processed for IF analyses and stained for the Golgi marker protein GM130 and AR. As expected, GM130 staining was predominantly perinuclear in vehicle-treated cells, which suggested that it was localized to the Golgi in LNCaP cells (Fig. 11*B*, *I-I*). Similar to vehicle-treated cells, the GM130 perinuclear staining pattern was preserved in androgen-treated cells (Fig. 11*B*, *II-I*). ARF1 inactivation by BFA promotes dissolution of the Golgi apparatus at the cellular level (67), and, as predicted, the GM130 perinuclear staining pattern was lost in BFA-treated cells (Fig. 11*B*, *III-I* and *IV-I*). Instead, the staining pattern for GM130 was predominantly cytosolic (Fig. 11*B*, *III-I*). This staining pattern was preserved in androgen-treated cells (Fig. 11*B*, *IV-I*). These results suggested that COPI retrograde protein trafficking was compromised in BFA-treated cells. In contrast to the Golgi localization of GM130 in vehicle-treated cells, AR localization was predominantly cytosolic and nuclear under the same conditions (Fig. 11*C*, *I-I*). These IF results validate previous studies showing that unliganded AR is primarily cytosolic in prostate tumor cells (20). As anticipated, androgen treatment promoted strong nuclear AR staining (Fig. 11*C*, *II-I*), which showed that the cytosolic-nuclear AR translocation process was unperturbed in LNCaP cells. Surprisingly, in cells pretreated with BFA, cytosolic and nuclear AR staining was increased in vehicle or androgen-treated cells (Fig. 11*C*, *III-I* and *IV-I*). These results showed that the cytoplasmic-nuclear AR translocation process was unaffected in BFA-treated cells and that BFA influenced AR expression in LNCaP cells.

To determine whether BFA had any effects on AR levels in LNCaP cells, crude subcellular fractionated protein extracts were subjected to Western blotting analyses to determine AR levels across the cytosolic, membrane, and nuclear protein fractions of BFA-treated cells. Western blotting analysis of the membrane protein marker Na^+^/K^+^ ATPase and the nuclear protein marker histone H2A authenticated the purity of subcellular protein fractions because both markers were restricted to the membrane and nuclear protein fractions, respectively (Fig. 11*D*). As predicted, in vehicle-pretreated cells, unliganded AR was predominantly localized to the cytoplasmic space encompassing the cytosolic and membranous protein fractions (Fig. 11*D*, *lanes 1* and *2*). In contrast, liganded AR was primarily localized to the nuclear protein fraction in vehicle-pretreated cells (Fig. 11*D*, *lane 6*). Similar to vehicle-pretreated cells, unliganded AR was restricted to the cytosolic and membranous protein fractions in BFA-pretreated cells (Fig. 11*D*, *lanes 7* and *8*). Importantly, AR levels were reduced in BFA-pretreated cells relative to vehicle-pretreated cells treated with vehicle or androgen (Fig. 11*D*, compare *lanes 7* and *8* with *lanes 1* and *2* and *lanes 11* and *12* with *lanes 5* and *6*). However, similar to the IF results, AR levels in the nuclear compartment increased in BFA-pretreated cells treated with androgens (Fig. 11*D*, compare *lane 12* with *lane 9*). Again, this result demonstrated that the cytoplasmic-nuclear AR translocation process was unaffected by BFA. Furthermore, because AR levels were noticeably reduced across all protein fractions in BFA-pretreated cells, ARA160 expression was probed across these same protein fractions. In vehicle-pretreated cells, ARA160 was detected only in the nuclear protein fraction of cells challenged with vehicle or androgens (Fig. 11*D*, *lanes 3* and *6*). However, in BFA-pretreated cells, a noticeable reduction in ARA160 levels was detected in the nuclear protein fraction (Fig. 11*D*, compare *lanes 9* and *12* with *lanes 3* and *6*). Moreover, detectable levels of ARA160 were observed in the cytosolic and membrane protein fractions of BFA-treated cells (Fig. 11*D*, *lanes 7*, *8*, *10*, and *11*). These results demonstrated that BFA changed ARA160 intracellular localization in LNCaP cells.

**FIGURE 12.**
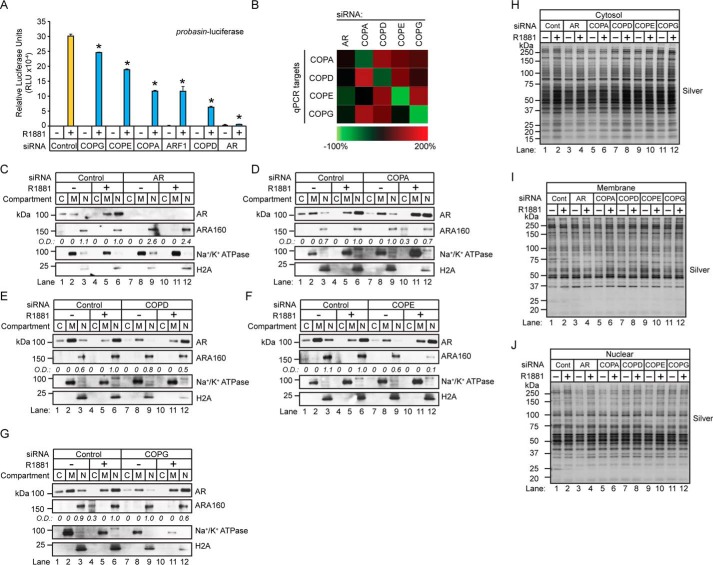
**COPI coatomer expression is required for optimal AR transcriptional activity.** *A*, LNCaP cells cultured in AD medium for 24 h were co-transfected with the indicated siRNAs targeting the COPI complex and the probasin-luciferase and pRLSV40-*Renilla* vectors for 48 h and then treated with vehicle (ethanol, −) or androgen (1 nm R1881, +) for 18 h. Total cell lysates were measured for Dual-Luciferase activity. Results are presented as means ± S.D. of three experiments. *Asterisks* indicate significant differences between cells transfected with target and control siRNAs (Student's *t* test; *, *p* < 0.05). *B*, quantitative PCR analysis of LNCaP cells transfected with siRNAs targeting COPA, COPD, COPE, and COPG to monitor knockdown efficacy. Quantitative PCR results were derived from three biological replicates. *C–G*, cytosolic (*C*), membrane (*M*), and nuclear (*N*) proteins were isolated from LNCaP cells transfected with the indicated siRNAs. The fractionated samples were analyzed by Western blotting with the indicated antibodies. Western blotting results are representative of two biological replicates. *H–J*, silver-stained gels show equivalent sample loading between samples. Densitometry values normalized to silver-stained gels are indicated *below* the blots.

The redistribution of nuclear ARA160 to the cytosolic and membrane protein fractions in BFA-treated cells suggested that the nuclear localization of ARA160 was regulated by the COPI complex. ARA160 is a critical coregulator of AR-mediated transcription (66), and, as a consequence, a defect in androgen-mediated ARA160 nuclear localization would probably attenuate AR-dependent transcription. First, we wanted to determine whether the AR-dependent transcription required COPI coatomer expression because androgen-mediated ARA160 nuclear localization would presumably require the COPI complex. Therefore, AR transcriptional activity was measured in LNCaP cells transfected with siRNAs directed against the COPI coatomer subunits (Fig. 12*A*). Briefly, 24-h androgen-depleted LNCaP cells were cotransfected with the probasin-luciferase reporter and siRNAs targeted against AR, COPA, COPE, COPG, COPD, and ARF1. The cells were challenged with vehicle (*i.e.* ethanol) or androgen (*i.e.* 1 nm R1881) for 18 h, and luciferase activity was determined between control and target siRNA knockdown cells (Fig. 12*A*). Quantitative RT-PCR confirmed greater than ∼70% knockdown of COPI mRNAs (*i.e.* COPA, COPD, COPE, and COPG) in siRNA-transfected cells (Fig. 12*B*). Notably, luciferase activity was strongly attenuated in COPA and COPD knockdown cells (Fig. 12*A*). Furthermore, siRNAs directed against ARF1, which coordinates the assembly of the COPI complex, strongly attenuated luciferase activity in LNCaP cells (Fig. 12*A*). Overall, these results showed that optimal AR transcriptional activity required COPI coatomer expression.

Next, we wanted to determine whether disrupted AR transcriptional activity in COPI knockdown cells was correlated with a defect in androgen-mediated ARA160 nuclear localization. Thus, ARA160 levels and subcellular localization were examined in COPI coatomer knockdown cells (Fig. 12, *C–G*). For this experiment, 24-h androgen-depleted LNCaP cells were transfected with siRNAs directed against AR, COPA, COPD, COPE, and COPG for 72 h. The cells were challenged for 1 h (*i.e.* acute) with vehicle (ethanol) or androgen (100 nm R1881), and the cells were subjected to subcellular fractionation to produce crude cytosolic, membrane, and nuclear protein fractions. Western blotting analyses demonstrated the effectiveness of the subcellular fractionation because the membrane marker Na^+^/K^+^ ATPase and the nuclear marker histone H2A were primarily restricted to the membrane and nuclear protein fractions in COPI coatomer knockdown cells (Fig. 12, *C–G*). As expected, the cytoplasmic-nuclear AR translocation process remained intact in control knockdown cells because androgens decreased cytosolic levels and increased the nuclear levels of AR (Fig. 12, *C–G*, *lanes 1–6*). Furthermore, AR levels were undetectable in the cytosolic, membrane, and nuclear protein fractions of AR knockdown cells (Fig. 12*C*). Notably, AR levels were relatively unchanged in the nuclear protein fraction of coatomer knockdown cells, demonstrating that cytoplasmic-nuclear translocation of AR was unperturbed in these cells (Fig. 12, *C–G*). Next, Western blotting analysis revealed that ARA160 levels were uniformly decreased in the nuclear protein fraction of androgen-treated coatomer knockdown cells (Fig. 12, *C–G*, compare *lane 12* with *lane 6*). These results suggested that COPI expression was required for optimal nuclear levels of ARA160 in androgen-treated LNCaP cells. Overall, these findings demonstrated that the process of translocating AR from the cytoplasm to the nucleus was unaffected by chemical or genetic disruptions in COPI-mediated retrograde protein trafficking and that the COPI complex regulated nuclear levels of ARA160 in LNCaP prostate tumor cells.

**FIGURE 13.**
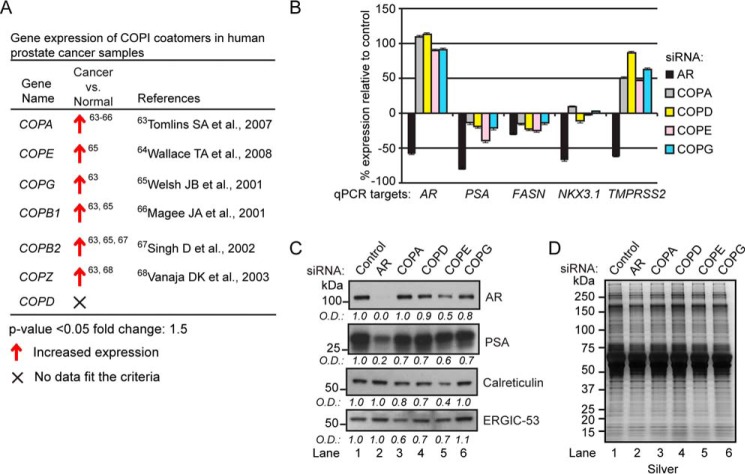
**COPI depletion does not induce a UPR.** *A*, Oncomine analysis of COPI coatomer expression in normal prostate tissue and localized cancers. *B*, quantitative PCR analysis of *AR*, *PSA*, *FASN*, *NKX3.1*, and *TMPRSS2* ARG expressions in LNCaP cells transfected with siRNAs targeting AR, COPA, COPD, COPE, and COPG. Results are presented as percentage difference relative to control ± S.D. (*error bars*) of three biological replicates. *C*, Western blotting analyses of AR, PSA, calreticulin, and ERGIC-53 levels in COPI coatomer knockdown cells. Western blotting results are representative of two biological replicates. *D*, silver-stained gels show equivalent sample loading between samples. Densitometry values normalized to silver-stained gels are indicated *below* the blots.

Aberrant AR signaling pathways promote prostate tumorigenesis (68), and because COPI coatomers modulated AR transcriptional activity in prostate tumor cells (Fig. 12*A*), we wanted to determine COPI coatomer expression across normal prostate tissue, localized prostate cancers, and metastatic prostate cancers. Gene expression changes in COPI coatomers (*i.e.* COPA, COPB1, COPB2, COPD, COPE, COPG, and COPZ) were queried with the Oncomine database (69). Comparative analysis of COPI coatomer expression between normal prostate tissue and localized cancers showed that all coatomers, with the exception of COPD, were up-regulated in localized cancers (Fig. 13*A*) (70–75). These results demonstrated that changes in COPI gene expression occur during the progression of human prostate cancers.

These results prompted us to establish a functional link between COPI coatomer expression and androgen-regulated gene (ARG) expression in prostate tumor cells. Therefore, we wanted to extend these findings by exploring whether COPI coatomer knockdown had any effect on the expression of a subset of ARGs. The ARGs measured included *AR*, prostate-specific antigen (*PSA*), fatty acid synthase (*FASN*), NK3 homeobox 1 (*NKX3.1*), and transmembrane protease serine 2 (*TMPRSS2*) (76, 77). As predicted, ARG mRNA expression was reduced in AR knockdown cells (Fig. 13*B*). Interestingly, COPI coatomer knockdown had a discordant effect on ARG expression. For example, whereas *AR* and *TMPRSS2* expression was potentiated in COPI coatomer knockdown cells, the opposite effect was observed for *PSA* and *FASN* (Fig. 13*B*). Surprisingly, COPI coatomer knockdown had minimal effect on *NKX3.1* expression (Fig. 13*B*). Interestingly, Western blotting analyses showed a reduction in AR protein in COPD, COPE, and COPG knockdown cells (Fig. 13*C*). This was an unanticipated finding because it represented a discordant relationship between expression of the AR mRNA and protein in COPI coatomer knockdown cells. As expected, the decrease in levels of the PSA protein was similar to that in of the PSA mRNA in COPI coatomer knockdown cells (Fig. 13*C*). This result demonstrated a congruent relationship between expression of the PSA mRNA and PSA protein in COPI coatomer knockdown cells. Collectively, these results show that COPI coatomers modulate the expression of AR and PSA in prostate tumor cells.

Many signaling pathways are connected through COPI retrograde protein trafficking (*e.g.* EGFR and glutamate receptor) (78, 79), and thus we wanted to determine whether ER stress response networks, such as the unfolded protein response (UPR) pathway (80), are activated in COPI coatomer knockdown cells. Western blotting analysis of the ER stress response markers calreticulin and ERGIC-53, whose levels increase during the UPR (81), was probed in AR and COPI coatomer knockdown cells (Fig. 13*D*). Densitometry analysis revealed minimal increases in calreticulin and ERGIC-53 levels in AR and COPI coatomer knockdown cells (Fig. 13*D*). However, measurable decreases in calreticulin and ERGIC-53 were observed in COPE < COPD < COPA < COPG knockdown cells (Fig. 13*D*). The functional significance of decreased calreticulin and ERGIC-53 in COPI coatomer knockdown cells has yet to be determined. Overall, these results show that the effects of COPI coatomer knockdown on AR-dependent transcription are independent of the UPR pathway in LNCaP prostate tumor cells.

## Discussion

We report the proteomic analysis of ligand-sensitive SBP-AR-interacting proteins in the cytosolic protein fraction of human prostate tumor cells using label-free quantitative mass spectrometry. A major goal of this study was to establish a bioanalytical workflow to determine how androgens coordinate physical interactions between SBP-AR and interacting proteins/protein complexes in the cytosolic compartment in the unliganded (*i.e.* androgen-depleted) and liganded (*i.e.* androgen-stimulated) states in prostate tumor cells. Despite years of experimental research, the biochemical composition of AR-interacting protein complexes in the unliganded and liganded states remains incomplete (13–16). This knowledge gap has made it difficult to establish which protein complexes and PPIs are critical for AR-dependent gene networks underlying the proliferation and survival of hormone-naive organ-confined and metastatic prostate tumors (1, 82). Molecular models of AR-dependent gene transcription include both dynamic and static PPIs between AR and the AR-interactome (1, 83–85). The large number of AR-interacting proteins (*i.e.* >350 proteins) that comprise the AR-interactome has made it difficult to understand how AR interacts with so many different types of proteins at the molecular level to mediate AR-dependent processes at the cellular level. AR has a finite number of protein interaction interfaces through which to mediate direct interactions between AR and the AR-interactome. Therefore, AR lacks the capacity to simultaneously bind to every member of the AR-interactome as a single supramolecular protein complex. Instead, it is reasonable to expect that both spatial and temporal constraints will determine what members of the AR-interactome bind to AR at the molecular level. This concept is underscored by the important experimental reality that the majority of the AR-interactome was discovered through the application of binary protein interaction assays (*i.e.* yeast two-hybrid, GST pull-down, and T7 phage display) (13–16). Although binary protein interaction assays are very powerful research tools for the discovery of novel PPIs, they are not without experimental limitations. For example, capturing physical interactions mediated by three or more proteins representative of a functional protein complex is a difficult task with these assays (86). Moreover, these assays typically lack the power to resolve interactions between functional protein complexes. Thus, a molecular model of ligand-mediated AR activation, which is based upon direct interactions between AR and the AR-interactome as well as indirect interactions between proteins that bind to the AR-interactome in a spatial and temporal context, has yet to be validated in prostate tumor cells. This was the main motivation for developing the N-AR cell line as a cellular system for elucidating ligand-dependent interactions between AR and the AR-interactome and also for facilitating the identification of novel AR-interacting proteins/protein complexes in the cytosolic, membranous, and nuclear protein fractions of prostate tumor cells. We have reported the identification of ligand-sensitive PPIs to SBP-AR in the cytosolic compartment of N-AR cells. The bioanalytical workflow presented in this study, which coupled streptavidin affinity chromatography and dMS to interrogate SBP-AR-interacting proteins, has methodological advantages over traditional immunoaffinity chromatography techniques and gel-based tandem mass spectrometry methods used in the proteomic identification of isolated protein complexes (87). First, the affinity of streptavidin-binding peptide sequence for streptavidin (*i.e. K_d_* value of ∼2 nm) is nearly equivalent to those of mouse monoclonal antibodies for their target antigens (18, 88). This biophysical characteristic of the SBP tag facilitates the isolation of SBP-tagged protein complexes using single-step streptavidin chromatography methods (18). Also, streptavidin chromatography avoids the destruction of antigen-binding sites that can occur when antibodies are covalently conjugated to beads for immunoaffinity purification experiments (89). Additionally, whereas binary interaction assays tend to identify high-affinity PPIs, streptavidin chromatography protocols can be tailored for the isolation of low-affinity PPIs with SBP-tagged proteins/protein complexes using low-stringency washes. These low-affinity PPIs could represent “piggy-back” interactions in which AR is bound indirectly, through another AR-interacting protein (*i.e.* AR-interactome). Last, the dMS approach utilized in this study facilitated an in depth proteomic analysis of the complex peptide samples representative of “streptavidin-copurified” proteins. The dMS approach is based upon principles of a targeted MS/MS acquisition scheme that selectively targets lower abundance ions for MS/MS. Previous studies have shown that targeted LC-MS/MS experiments are more sensitive and robust for the identification of proteins than traditional data-dependent LC-MS/MS experiments (22, 24, 25, 90). Thus, the dMS provided a superior proteomic method for interrogating affinity-captured proteins in this study. Despite these experimental advantages inherent to our bioanalytical workflow, it is not without limitations. For example, many of the proteins detected in the proteomic screen could represent background contaminants that bound tightly to the streptavidin beads during the isolation of the SBP-tagged target protein. Presumably, these proteins could represent endogenous biotin-labeled proteins, copurifying piggyback proteins that bind to the biotin-labeled proteins, biotin-deficient streptavidin-binding proteins, and piggyback proteins that bind biotin-deficient streptavidin-binding proteins. Endogenous streptavidin-binding proteins were not verified in our study; nor have they been defined in any other published study to date. Due to this limitation, the bioinformatic and downstream experimental validation studies were restricted to ligand-sensitive proteins in the proteomic screen because it would be reasonable to suspect that background proteins would be ligand-insensitive. Although this study focused on ligand-sensitive proteins, some members of the AR-interactome bind AR in a ligand-independent manner (15). Future proteomic experiments will address the molecular composition of endogenous streptavidin-binding proteins so that ligand-insensitive AR-interacting proteins can be adequately detected in androgen-responsive prostate tumor cells. Overall, our bioinformatic analyses showed that the proteomic screen was enriched for known coregulators of AR-mediated transcription (Fig. 4*A* and Table 1). Moreover, a greater level of ligand sensitivity was observed for AR coregulators detected in the proteomic screen (Table 1). Based upon these findings, we speculate that many of the ligand-sensitive proteins identified in the proteomic screen are physically linked, directly or indirectly, to molecules involved in ligand-mediated AR activation/function in prostate tumor cells.

A major finding of this proteomic study was that SBP-AR was associated with the Golgi environment and that it associated physically with the COPI retrograde protein complex in prostate tumor cells (Fig. 9, *D* and *E*). These findings validate previous reports of a biochemical interaction between AR and specific subunits of the COPI complex in prostate tumor cells (91, 92). More importantly, we showed that the COPI complex was required for ligand-dependent AR-mediated transcription and coordinated mobilization of the Golgi-localized AR coregulator ARA160 into the nuclear compartment in response to androgens (Figs. 11 and 12) (66). Our findings suggest that AR might also associate with the Golgi compartment in a ligand-dependent manner to coordinate other AR coregulators involved in AR-mediated transcription. Future experiments will test whether ARA160 expression is required for AR to associate with the Golgi compartment. Interestingly, whereas the COPI complex was not required for androgen-mediated trafficking of AR from the cytoplasm to the nucleus (Figs. 11 and 12), a functional COPI complex was required for such trafficking of ARA160 (Figs. 11 and 12). COPI retrograde trafficking might also regulate the subcellular trafficking of other well studied AR coregulators (*i.e.* NCOR and SMRT) in response to androgens. Additional experiments are warranted to test this hypothesis further. The COPI complex has been indirectly linked to the pathobiology of prostate tumor cells (93). For example, chronic exposure to BFA was shown to inhibit prostate tumor cell proliferation *in vitro* (93). Unfortunately, BFA is non-selective for tumor cells and is equally cytostatic to non-tumorigenic cells (94). Ideally, drugs that selectively disrupt the COPI complex and block AR coregulator trafficking would be developed as an effective molecular strategy for attenuating aberrant AR activity in human prostate cancers.

Another important finding of this study was the preliminary identification of AR modulators using the siRNA luciferase screen (Fig. 8). For example, a handful of E3 ligases previously implicated in AR-dependent transcription were shown to modulate AR transcriptional activity (43, 60, 61, 95). The siRNA luciferase screen revealed that STUB1 attenuated AR-mediated transcription (43), whereas E3 ligase TRIM68 potentiated AR transcriptional activity (95) (Fig. 8*B*). Interestingly, some of the results of the siRNA luciferase screen were discordant with the purported function of the E3 ligases and deubiquitinases on AR-mediated transcription (Fig. 8*B*). For example, MDM2 was shown to promote AR protein degradation (60), and thus MDM2 expression is predicted to attenuate AR-dependent transcription in prostate tumor cells. However, cells transfected with validated MDM2 siRNAs failed to potentiate AR transcriptional activity in LNCaP prostate tumor cells (Fig. 8*B*). Instead, AR transcriptional activity was attenuated in MDM2 siRNA-transfected cells (Fig. 8*B*). Assuming that MDM2 promotes AR protein degradation/turnover, our results suggest that this process is required for normal ligand-dependent AR transcriptional activity in prostate tumor cells. This outcome is supported by a previous study showing that AR turnover and transcriptional activity were blocked in LNCaP cells pretreated with the proteasomal inhibitor MG132 (30). Another discordant example detected in the siRNA luciferase screen is the E3 ligase RNF6 (Fig. 8*B*). This protein had initially been identified as an AR coactivator of the *PSA* gene in prostate tumor cells (61). However, the transfection of validated RNF6 siRNAs attenuated AR transcriptional activity in LNCaP cell (Fig. 8*B*). Interestingly, RNF6 is also an AR corepressor (61), which demonstrated that it is a promoter-dependent coregulator of AR transcription in prostate tumor cells. The last discordant finding of the siRNA luciferase screen involved the USP10 deubiquitinase (Fig. 8*B*). USP10 was shown to act as an AR coactivator in WT AR-expressing PC3 cells when AR transcriptional activity was measured using the mouse mammary tumor virus luciferase reporter (44). In contrast, the siRNA luciferase screen in LNCaP cells showed that transfection with validated siRNAs targeting USP10 potentiated AR transcriptional activity. We speculate that the discordant effects on AR-mediated transcription observed between the siRNA luciferase screen and reported AR coregulator functions of MDM2, RNF6, and USP10 in prostate tumor cells are due to differences in prostate tumor cell lines and reporter vectors. Regardless of these incongruent findings, the siRNA luciferase screen represents a powerful tool for identifying modulators of AR-mediated transcription in LNCaP prostate tumor cells.

This proteomic study provides a benchmark for the development of a ligand-dependent PPI map of AR-interacting proteins/protein complexes in the cytosolic compartment of human prostate tumor cells. Obviously, this PPI map will evolve and expand with the subsequent proteomic identification of AR-interacting proteins/protein complexes that exist in the membrane and nuclear compartments of prostate tumor cells. The integration of these compartment-specific PPIs into AR-interacting proteins/protein complexes will allow us to develop a quantitative model of ligand-mediated AR activation. This molecular model should resolve compartment-specific, ligand-dependent AR-interactome networks involved in the process of AR-mediated gene transcription. Time course experiments to capture dynamic and static PPIs with AR will provide further resolution of the molecular model. For example, the proteomic workflow presented can be interfaced with selected reaction monitoring methods to facilitate the validation of PPIs with the incorporation of heavy labeled peptides based upon stable isotope dilution MS (96). However, we recognize the physiological limitations of developing a ligand-dependent AR-interactome network based upon the proteomic findings of a single human prostate tumor cell line, such as LNCaP. This cell line was derived from the lymph node of a patient with metastatic disease and represents only one of many types of human prostate cancers. LNCaP cells are hypotetraploid and contain a series of genomic lesions. In particular, they harbor the AR-T877A mutation, which decreases the ligand specificity of the receptor (17, 97), and lack PTEN (98, 99). We envision that the recent development of CRISPR technology, which uses engineered nucleases for the purpose of editing the genome (100), will be used to develop new human prostate tumor cell lines that harbor lesions in genes (*e.g.* tumor suppressors and oncogenes) that are commonly mutated in human prostate cancers (101, 102). We predict that these mutation-specific human prostate tumor cells will inevitably influence the molecular composition of the AR-interactome. These novel cellular systems will provide new reagents to validate the ligand-dependent AR-interactome networks defined in LNCaP cells. However, they will also offer new opportunities to elucidate ligand-dependent AR-interactome networks in a tumor-specific background.

In summary, this study describes a cellular system and bioanalytical workflow for defining ligand-dependent AR-interactome networks in human prostate tumor cells. Our findings suggest that androgen-mediated AR activation is coupled to a number of PPIs between AR and various functional protein complexes in the cytosolic compartment of prostate tumor cells. We believe that these findings illustrate the power of discovery proteomics in the molecular dissection of signal transduction pathways and highlight the power of this approach in the development of new hypothesis-driven studies for future exploration.

## Experimental Procedures

### 

#### 

##### Cloning and Construction of SBP-tagged AR

The mammalian expression vector pSG5-AR was used as a template for PCR-based amplification of AR, which was carried out using Advantage GC-2 polymerase (Clontech). Amplified DNA was cloned in-frame into the 3′-end of the SBP and FLAG pcDNA3 plasmid, thus generating a pcDNA3-SBP-FLAG-AR plasmid. The DNA was cloned into the 5′ EcoRI and 3′ XhoI restriction sites of the pcDNA3 SBP vector. The SBP sequence used was 5′-ATGGACTACAAGGACGACGAC-3′. The oligonucleotide primers (Invitrogen) used for cloning pcDNA3-SBP-FLAG-AR were as follows: 5′ primer, 5′-GATCGATATCATATGGAAGTGCAGTTAGGGCTGGGAAGGGTCTAC-3′; 3′ primer, 5′-GATCCTCGAGTCACTGGGTGTGGAAATAGATGGGCTTGACTTTCCCA-3′. All constructs were confirmed by sequencing the coding region using both gene-specific and vector-specific primers.

##### Cell Culture and the Generation of Stable Cell Lines

LNCaP prostate cancer cells (American Type Culture Collection) were cultured in phenol red-deficient RPMI 1640 medium (Invitrogen) supplemented with 10% fetal bovine serum (Hyclone Laboratories, Logan, UT), 1× Glutamax, 100 units/ml penicillin, and 100 μg/ml streptomycin (Invitrogen). For the generation of cell lines, individual pcDNA3-SBP-FLAG (control, NC cell line) and pcDNA3-SBP-FLAG-AR cDNAs (N-AR cell line) were transfected into LNCaP cells using the Lipofectamine LTX reagent (Invitrogen) following the manufacturer's instructions. Two days after transfection, cells were selected in Geneticin (G418, 500 μg/ml). STR analysis was used to authenticate the genotype of all human prostate cancer cell lines (August, 2008) (103).

##### Western Blotting Analysis

Total protein extracts were quantified using the Pierce BCA protein assay kit (Thermo Fisher Scientific). 4 μg of total protein lysates were subjected to 4–12% SDS-PAGE (Invitrogen) and transferred to a PVDF membrane (Bio-Rad). Membranes were incubated with Blotto (4% (w/v) nonfat milk in TBST (Tris-buffered saline (TBS: 50 mm Tris, pH 7.5, 150 mm NaCl) + 0.1% (v/v) Tween 20)) for 1 h at room temperature and then incubated in TBST containing 5% bovine serum with one of the following antibodies: rabbit polyclonal AR (N-20) antibody (1:1,000 dilution; Santa Cruz Biotechnology); mouse monoclonal AR (catalog no. 441) antibody (1:250 dilution; Santa Cruz Biotechnology); mouse monoclonal Na^+^/K^+^ ATPase antibody (1:1,000 dilution; Santa Cruz Biotechnology); rabbit polyclonal PSA antibody (1:1,000 dilution; DAKO, Carpinteria, CA); H2A antibody (1:1,000 dilution; Cell Signaling Technology); or a mouse monoclonal ARA160 antibody (1:1,000 dilution; Proteintech, Chicago, IL). After three 5-min washes with TBST, the membranes were incubated in TBST containing 5% BSA with goat anti-mouse or goat anti-rabbit HRP-conjugated secondary antibodies (1:10,000; Bio-Rad) for 1 h at room temperature. After three 5-min washes with TBST, immunoreactive bands were developed and visualized using the ECL reagent kit (Thermo Fisher Scientific), and the membranes were exposed to Hyperfilm ECL film (GE Healthcare) for <5 min.

##### Immunofluorescence N-AR Characterization

N-AR cells were cultured in androgen-depleted medium (phenol red-deficient RPMI 1640 medium (Invitrogen) supplemented with 10% charcoal-stripped fetal bovine serum (Hyclone Laboratories, Logan, UT), 1× Glutamax, 100 units/ml penicillin, and 100 μg/ml streptomycin (Invitrogen)) for 96 h and then treated with vehicle (ethanol) or androgen (100 nm R1881; PerkinElmer Life Sciences) for 1 h. The medium was removed, and the cells were fixed in DPBS containing 4% formaldehyde for 20 min at room temperature. After three washes with DPBS, nonspecific protein binding sites were blocked with Blotto (4% (w/v) nonfat milk in TBS plus 0.1% (v/v) Triton X-100) for 1 h at room temperature and then incubated with mouse anti-SBP monoclonal antibody (1:50 dilution) for 1 h at room temperature. After washing three times with Blotto + Triton X-100, cells were incubated with Alexa 488 goat anti-mouse antibody, phalloidin, and DAPI nuclear dye (Invitrogen) for 1 h at room temperature, washed three times with DPBS, and mounted in ProLong Gold (Invitrogen). All cells in three randomly chosen fields in three independent samples were imaged using a digital camera at ×10 magnification on an Olympus IX70 inverted microscope. Post-imaging processing was performed using Adobe Photoshop software, taking care to maintain any linear differences in signal intensities present in the original samples.

##### BFA Treatment Experiments

LNCaP cells were cultured in androgen-depleted medium for 96 h and subsequently treated with vehicle (ethanol) or BFA (50 μm) for 30 min. After one wash with DPBS, cells were treated with vehicle (ethanol) or androgen (100 nm R1881) for 1 h and subjected to fixation and IF labeling with rabbit anti-AR polyclonal antibody (N-20; 1:100 dilution) and Alexa 488 goat anti-rabbit antibody as described above.

##### Subcellular Fractionation; N-AR Characterization

NC and N-AR cells were cultured in androgen-depleted medium for 96 h and subsequently treated with vehicle (ethanol) or androgen (100 nm R1881) for 1 h. Cytoplasmic and nuclear proteins were isolated from the cells using the Subcellular Protein Fractionation Kit for Cultured Cells according to the manufacturer's guidelines (Thermo Fisher Scientific).

##### BFA Treatment Experiments

LNCaP cells were cultured in androgen-depleted medium for 96 h and subsequently treated with vehicle (ethanol) or 50 μm BFA for 30 min. After one wash with DPBS, cells were treated with vehicle (ethanol) or androgen (100 nm R1881) for 1 h. Cells were then harvested and incubated in hypotonic solution (10 mm Hepes, 1.5 mm MgCl_2_, and 10 mm KCl, pH 7.9) for 10 min and passed through an 18-gauge syringe 15 times. Nuclei were pelleted by centrifugation at 600 × *g* for 20 min at 4 °C and resuspended in nuclear extraction buffer (20 mm Hepes, 600 mm KCl, 25% glycerol, 1.5 mm MgCl_2_, and 0.2 mm ZnCl_2_, pH 7.9). The supernatant was then subjected to ultracentrifugation at 100,000 × *g* for 3 h at 4 °C to separate the membranes (crude microsomes) from the cytosol.

##### siRNA Knockdown; N-AR Characterization

Validated siRNAs targeting the coding region or 3′-UTR of AR (Qiagen, Valencia, CA) were transfected into LNCaP and N-AR cells. Scrambled siRNA was used as a control. Transfection of siRNAs (100 nm) was performed using Oligofectamine (Invitrogen), and cells were harvested 72 h post-transfection with lysis buffer (1% SDS, 50 mm Tris-HCl, 150 mm NaCl, 5 mm EDTA, pH 7.4). The isolated protein extracts were subjected to Western blotting analysis with antibodies to SBP and AR.

##### Fractionation Experiments

LNCaP cells cultured in androgen-depleted medium for 24 h were transfected with validated siRNAs targeting AR, COPA, COPD, COPE, and COPG (Qiagen). Scrambled siRNA was used as a control. After transfection of siRNAs (100 nm) was performed using Oligofectamine (Life Technologies, Inc.) for 72 h, cells were treated with vehicle (ethanol) or androgen (100 nm R1881) for 1 h and subjected to subcellular fractionation and Western blotting analysis as described above.

##### Cell Stress Response Experiments

LNCaP cells were transfected with validated siRNAs targeting AR, COPA, COPD, COPE, and COPG (Qiagen) with Oligofectamine for 72 h. The isolated total protein extracts were subjected to Western blotting analysis with antibodies to AR, calreticulin, ERGIC-53, and PSA.

##### Quantitative PCR Experiments

LNCaP cells were transfected with validated siRNAs as described above. RNA extraction was carried out using the RNeasy Midi Kit using the manufacturer's instructions (Qiagen). Strand cDNA synthesis was performed using reverse transcription protocols detailed in the SuperScript® III first-strand synthesis kit (Invitrogen). Real-time quantitative PCR was carried out in a reaction containing cDNA, respective primer pairs, and SYBR Green PCR Master Mix (Applied Biosystems). GAPDH was used as an internal control for normalization. Relative expression values were calculated using the comparative *Ct* method (104).

##### Dual-Luciferase Reporter Assay; N-AR Characterization

LNCaP and N-AR cells were seeded into Falcon (BD Biosciences) 48-well tissue culture dishes at a density of 30,000 cells/cm^2^. The cells were cultured in androgen-depleted medium for 24 h and co-transfected with siRNAs targeting the coding region or 3′-UTR of AR (Qiagen, 100 nm) along with pGL4.10-Luc2-probasin (10 ng) and pRLSV40-*Renilla* (25 ng). After transfection was performed using Lipofectamine 2000 (Invitrogen) for 48 h, the cells were treated with vehicle (ethanol) or androgen (1 nm R1881) for 18 h and harvested for luciferase activity using the Dual-Luciferase reporter assay system (Promega, Madison, WI) according to the manufacturer's protocol. Analysis of variance was used to determine significant differences between experimental and control siRNA-transfected cells (*, *p* ≤ 0.05, *n* = 3).

##### BFA Treatment Experiments

LNCaP cells were seeded as described above. Transfections were carried out in triplicate with pGL4.10-Luc2-probasin (10 ng) and pRLSV40-*Renilla* (25 ng) for 48 h and then treated with vehicle (ethanol) or BFA (50 μm) for 30 min. The cells were then washed with DPBS once and treated with vehicle (ethanol) or androgen (1 nm R1881) for 4, 8, or 12 h. Luciferase activities were then quantified as described above.

##### siRNA Knockdown Experiments

LNCaP cells were seeded as described above, with the exception that the cells were co-transfected with validated siRNAs targeting COPG, COPE, COPA, ARF1, COPD, and AR (Qiagen, 100 nm) along with pGL4.10-Luc2-probasin (10 ng) and pRLSV40-*Renilla* (25 ng).

##### siRNA Luciferase Screen

LNCaP cells were seeded as described above, with the exception that the cells were co-transfected with validated siRNAs targeting proteins identified from the proteomic analysis. 62 total siRNAs were purchased as a Flexiplate from Qiagen (catalog no. 1027413), and a mix of four siRNAs targeting a single gene (100 nm) was used to transfect the cells.

##### Streptavidin Affinity Chromatography

N-AR cells were grown in androgen-depleted medium for 96 h and treated with vehicle (ethanol; AD) or androgen (100 nm R1881; AS) for 1 h. The cells were then harvested and incubated in hypotonic solution (10 mm Hepes, 1.5 mm MgCl_2_, and 10 mm KCl, pH 7.9) with 5 mm DTT and 1× protease inhibitor mixture (Thermo Fisher Scientific) for 10 min and subjected to nitrogen cavitation at 100 psi for 5 min. Lysed samples were centrifuged at 600 × *g* for 20 min at 4 °C to pellet the nuclei, and the resulting supernatant was centrifuged at 100,000 × *g* for 3 h at 4 °C to remove the microsomes (pellet) from the cytosolic proteins. 10 mg of cytosolic proteins were used for the affinity purification of AR protein complexes by incubating overnight with a 250-μl bed volume of UltraLink Plus streptavidin beads (Thermo Fisher Scientific) in AR purification buffer (50 mm Tris, 100 mm KCl, 20% glycerol, 1.5 mm MgCl_2_, 0.2 mm ZnCl_2_) with 5 mm DTT (Sigma), 1× protease inhibitor mixture (Thermo Fisher Scientific), 5 mm ATP (Sigma), and 0.025% Nonidet P-40 (Sigma) with the addition of vehicle (ethanol) or androgen (100 nm R1881) for AD and AS samples, respectively, at 4 °C. The next day, samples were centrifuged at 500 × *g* for 1 min to cluster the beads, which were then washed three times with 1 ml of wash buffer (AR purification buffer with 5 mm DTT, 1× protease inhibitor mixture, and 0.025% Nonidet P-40). Proteins were eluted with 8 m urea, 50 mm Tris, 1% SDS, 10 mm DTT, and 5 mm Biotin, pH 8.5, at room temperature for 1 h. The samples were dialyzed against 8 m urea, 50 mm Tris, and 100 mm β-mercaptoethanol, pH 8.5, with 10 kDa cut-off dialysis cassettes (Thermo Fisher Scientific) to remove detergent before mass spectrometry analysis. To measure purification efficiency, 1% of the input, void (unbound proteins), wash, and eluate were analyzed by Western blotting with antibodies to SBP and AR.

##### Sample Preparation for Mass Spectrometry

Samples were first reduced in 10 mm DTT (Thermo Fisher Scientific) for 1 h at 37 °C, alkylated in 55 mm iodoacetamide (Thermo Fisher Scientific) for 1 h at room temperature in the dark, and then digested with trypsin (1:50 trypsin/protein ratio; Promega) in 0.5 m urea. Next, each sample was added to a tryptic digest of BSA containing iodoacetic acid alkylated cysteine residues (Michrom Bioresources, Auburn, CA) at a 1:75 BSA/protein molar ratio. Samples were acidified before being desalted on Vydac C18 spin columns (Nest Group, Inc., Southborough, MA) and then subjected to strong cation exchange fractionation on polysulfoethyl A packed spin columns (Nest Group). Briefly, desalted samples were dissolved into strong cation exchange buffer A (5 mm KHPO_4_, 25% acetonitrile) and loaded onto strong cation exchange spin columns. Peptides were eluted from the strong cation exchange spin columns using a six-step (20, 30, 40, 50, 60, and 120 mm) KCl elution gradient developed from a mixture of buffer A and buffer B (5 mm KHPO_4_, 25% acetonitrile, 350 mm KCl). Salt-bumped, eluted fractions were desalted, dried, and redissolved in mass spectrometry loading buffer (1% acetic acid, 1% acetonitrile).

##### Mass Spectrometry Analysis

The samples were analyzed by nano-liquid chromatography-tandem mass spectrometry using an Agilent 6520 Accurate-Mass quadrupole time-of-flight mass spectrometer interfaced with an HPLC Chip Cube. The samples were loaded onto an Ultra High Capacity Chip (500-nl enrichment column, 75 μm × 150-mm analytical column). LC-MS/MS analysis was performed using a 180-min gradient ranging from 8 to 35% buffer B (100% acetonitrile, 0.8% acetic acid). Full MS (MS1) data were acquired with a mass range of 400–1250 *m*/*z* and acquisition rate of 1 spectrum/s. From these data, an ion preferred list was generated with Agilent MassHunter qualitative software with settings of 400–1,250 *m*/*z*, 2+ and 3+ charge states, and spectra with 2 or more ions. The dMS was performed with the following settings: a maximum of 10 ions/cycle, a narrow isolation width (∼1.3 atomic mass units), precursor masses dynamically excluded for 30 s after 8 MS/MS in a 30-s time window, and use of the preferred ion list. Mass spectrometry capillary voltage and capillary temperature settings were set to 1,800 V and 330 °C, respectively. The infused reference mass of 1,221.9906 was used to correct precursor *m*/*z* masses for each LC-MS/MS experiment.

The raw data files were searched against the UniProt human database using SpectrumMill software version B.04.00.127 and the following settings: precursor mass tolerance of 25 ppm, product mass tolerance of 200 ppm, and a maximum of two trypsin miscleavages. Search modifications included a static carbamidomethylation on cysteine residues (C = 57.02146 atomic mass units), and differential modifications for oxidized methionine (M = 15.9949 atomic mass units), phosphorylated serine, threonine, and tyrosine (STY = 79.9663 atomic mass units), and ubiquitinated lysine (K = 114.0429 atomic mass units) were used for post-translational modifications. A false discovery rate of <1% was accepted for this analysis. The identified proteins are presented in supplemental Table 1 and Table 4.

##### Streptavidin Affinity Purification of Recombinant AR

rSBP-AR was generated from the TNT® Quick Coupled Transcription/Translation Reticulocyte Lysate System (Promega). Equal amounts of SBP-AR, as determined by Western blotting, were incubated with 200 μg of cytosolic protein extracts isolated from AD and AS LNCaP cells and 25 μl of UltraLink Plus streptavidin beads with the addition of vehicle (ethanol) or androgen (100 nm R1881) for AD and AS samples, respectively. The samples were subjected to streptavidin affinity chromatography as performed in the original proteomic experiment and described under “Streptavidin Affinity Chromatography.”

##### AR-interactome Statistical Analyses

The annotation of known AR-interacting proteins and androgen-sensitive AR-interacting proteins in the proteomic data set for results presented in Table 1 was derived by comparing gene names of known AR-interacting proteins with gene names of copurified proteins identified in the streptavidin proteomic screen. The raw data files used for these comparative analyses are shown in supplemental Table 2. Fisher's exact test was carried out using the total number of estimated proteins detectable by mass spectrometry (∼12,000) (33).

##### Network Visualization and Accessed Public Protein Interaction Databases

Protein interaction networks were visualized with Cytoscape version 3.1.0 (34). Known interactions were obtained from the protein interaction network analysis platform PINA (36) for the putative AR-interacting proteins found in this study. Ontology and annotation information was downloaded through the Cytoscape interface and used to group putative AR-interacting proteins into biological process categories.

##### Isolation of Golgi Membranes

LNCaP and N-AR cells were grown in androgen-depleted medium for 96 h and treated with vehicle (ethanol; AD) or androgen (100 nm R1881; AS) for 1 h. The cells were then harvested and incubated in hypotonic solution (10 mm Hepes, 1.5 mm MgCl_2_, and 10 mm KCl, pH 7.9) with 5 mm DTT and 1× protease inhibitor mixture for 10 min, followed by nitrogen cavitation at 100 p.s.i. for 5 min. Ice-cold 2.3 m sucrose containing 10 mm Tris-HCl, pH 7.4, was added to 6 ml of crude homogenate to a final 1.4 m sucrose concentration. The mixture was loaded into a clear plastic SW27 centrifuge tube (Beckman Coulter, Pasadena, CA), and overlaid with 14 ml of 1.2 m sucrose (containing 10 mm Tris-HCl, pH 7.4) and then 9 ml of 0.8 m sucrose (containing 10 mm Tris-HCl, pH 7.4) for a final volume of 35 ml. The gradients were centrifuged at 90,000 × *g* for 2.5 h with a SW27 rotor. A turbid band containing the Golgi membrane formed at the 0.8 m/1.2 m sucrose interface and was collected for downstream analysis.

## Author Contributions

J. J. H. conducted most of the experiments, analyzed results, and co-wrote the paper. M. M. S., B. H. N., and J. L. performed experiments and bioinformatic analyses. M. E. W. conducted experiments, developed reagents, analyzed results, and co-wrote the paper.

## Supplementary Material

Supplemental Data

## Footnotes

2 The abbreviations used are:

AR: androgen receptor
AD: androgen-depleted
AS: androgen-stimulated
ARG: androgen-regulated gene
BFA: brefeldin A
dMS: directed mass spectrometry
ER: estrogen receptor
GEPF: Golgi-enriched protein fraction
MS/MS: tandem MS
PIN: protein interaction network
PPI: protein-protein interaction
SBP: streptavidin-binding peptide
SBP-AR: streptavidin-binding peptide tagged wild-type androgen receptor
rSBP-AR: recombinant SBP-AR
SHR: steroid hormone receptor
COPI: coatomer protein complex I
NC: negative control
IF: immunofluorescence
GEPF: Golgi-enriched protein fraction
UPR: unfolded protein response
DPBS: Dulbecco's PBS
PSA: prostate-specific antigen.

## Note Added in Proof

Tables 1 and 2 were inadvertently omitted in the version of this article that was published as a Paper in Press on June 30, 2016. The tables are now shown.
