# Supplementary material for: Discovery Proteomics Identifies a Molecular Link between the Coatomer Protein Complex I and Androgen Receptor-dependent Transcription
Source: J Biol Chem. 2016 Jun 30;291(36):18818–42. doi: 10.1074/jbc.M116.732313 (PMC5009256; doi:10.1074/jbc.M116.732313)
Supplement: Supplemental Data [file supp_291_36_18818__index.html]

Discovery Proteomics Identifies a Molecular Link between the Coatomer Protein Complex I and Androgen Receptor-dependent Transcription — Discovery Proteomics Identifies a Molecular Link between the Coatomer Protein Complex I and Androgen Receptor-dependent Transcription — COPI Interactions with Androgen Receptor — Supplemental Data 

# Discovery Proteomics Identifies a Molecular Link between the Coatomer Protein Complex I and Androgen Receptor-dependent Transcription

## Supplemental Data

- Supplemental Tables 1-4 (.pdf, 6.6 MB) - This files contains the supplemental tables of the mass spectrometry data.
