# Supplementary material for: Discovery Proteomics Identifies a Molecular Link between the Coatomer Protein Complex I and Androgen Receptor-dependent Transcription
Source: J Biol Chem. 2016 Jun 30;291(36):18818–42. doi: 10.1074/jbc.M116.732313 (PMC5009256; doi:10.1074/jbc.M116.732313)
Supplement: Supplemental Data [file 10.1074_M116.732313_jbc.M116.732313-1.pdf]

Discovery Proteomics Identifies a Molecular Link between the Coatomer Protein Complex I and Androgen Receptor-dependent Transcription

Jordy J. Hsiao[1], Melinda M. Smits[1], Brandon H. Ng[1], Jinhee Lee[1], Michael E. Wright[1]

Supplemental:

(S1) Supplemental Table 1: Protein identifications in the *AD* and *AS* samples……………..Pages 2- 91

(S2) Supplemental Table 2: Known AR-interacting proteins identified…………………….Pages 92- 110
in the proteomic screen.

(S3) Supplemental Table 3: Results of siRNA luciferase screen……………………………Pages 111-116

(S4) Supplemental Table 4: Exported MS/MS spectra of identified peptides……………….Pages 117-368

Supplemental Table 1. Proteins identified in the proteomic screen

Subgroup	Entry Name	Minus	Plus	Ratio	Fold Change	Adjusted Values (max and min)	Ln(AS/AD)	Accession Number	Gene Name
3184.1	NAD-dependent protein deacety	0.00E+00	1.25E+03	1.25E+03	1.25E+03	NULL	AS	Q8IXJ6	SIRT2
1989.1	Kelch domain-containing protei	0.00E+00	1.70E+03	1.70E+03	1.70E+03	NULL	AS	Q8IXV7	KLHDC8B
2276.1	Ig lambda chain V-VI region SUT	0.00E+00	1.79E+03	1.79E+03	1.79E+03	NULL	AS	P06317	LV603
3101.1	FYVE, RhoGEF and PH domain-co	0.00E+00	1.80E+03	1.80E+03	1.80E+03	NULL	AS	Q6ZV73	FGD6
2199.1	Endonuclease V	0.00E+00	2.26E+03	2.26E+03	2.26E+03	NULL	AS	E5RII5	ENDOV
824.1	Isoform 2 of Lysine-specific dem	0.00E+00	2.29E+03	2.29E+03	2.29E+03	NULL	AS	Q8NHM5-2	KDM2B
2160.1	T-cell activation inhibitor, mitoc	0.00E+00	2.33E+03	2.33E+03	2.33E+03	NULL	AS	Q8N3R3	TCAIM
2756.1	Olfactory receptor 5AR1	0.00E+00	2.39E+03	2.39E+03	2.39E+03	NULL	AS	Q8NGP9	OR5AR1
2767.1	Protein ALEX	0.00E+00	3.52E+03	3.52E+03	3.52E+03	NULL	AS	P84996	GNAS
2300.1	Chondroitin sulfate proteoglyca	0.00E+00	3.55E+03	3.55E+03	3.55E+03	NULL	AS	Q6UVK1	CSPG4
1092.1	Amidophosphoribosyltransferas	0.00E+00	3.95E+03	3.95E+03	3.95E+03	NULL	AS	Q06203	PPAT
1986.1	Cancer-associated gene 1 protei	0.00E+00	4.32E+03	4.32E+03	4.32E+03	NULL	AS	Q8TC20	CAGE1
2590.1	Ras-interacting protein 1	0.00E+00	4.82E+03	4.82E+03	4.82E+03	NULL	AS	Q5U651	RASIP1
1758.1	Ras-related protein Rab-11B	0.00E+00	4.96E+03	4.96E+03	4.96E+03	NULL	AS	Q15907	RAB11B
2912.1	Parafibromin	0.00E+00	4.98E+03	4.98E+03	4.98E+03	NULL	AS	Q6P1J9	CDC73
2772.1	Protein Shroom4	0.00E+00	5.08E+03	5.08E+03	5.08E+03	NULL	AS	Q9ULL8	SHROOM4
2153.1	mRNA-decapping enzyme 1A	0.00E+00	5.10E+03	5.10E+03	5.10E+03	NULL	AS	Q9NPI6	DCP1A
2123.1	Vacuolar protein sorting-associa	0.00E+00	5.17E+03	5.17E+03	5.17E+03	NULL	AS	O75436	VPS26A
1913.1	Peptidyl-alpha-hydroxyglycine a	0.00E+00	5.23E+03	5.23E+03	5.23E+03	NULL	AS	F8W8D9	PAM
3249.1	Zinc finger protein with KRAB ar	0.00E+00	5.38E+03	5.38E+03	5.38E+03	NULL	AS	Q63HK3	ZKSCAN2
1289.1	Hepatocyte cell adhesion molec	0.00E+00	5.67E+03	5.67E+03	5.67E+03	NULL	AS	Q14CZ8	HEPACAM
2583.1	Rab-like protein 5	0.00E+00	5.71E+03	5.71E+03	5.71E+03	NULL	AS	Q9H7X7	RABL5
1317.1	Rabenosyn-5	0.00E+00	6.09E+03	6.09E+03	6.09E+03	NULL	AS	Q9H1K0	ZFYVE20
3108.1	Myristoylated alanine-rich C-kin	0.00E+00	6.14E+03	6.14E+03	6.14E+03	NULL	AS	P29966	MARCKS
2853.1	Tumor protein p63-regulated ge	0.00E+00	6.20E+03	6.20E+03	6.20E+03	NULL	AS	Q5T0D9	TPRG1L
3054.1	BTB/POZ domain-containing prc	0.00E+00	6.23E+03	6.23E+03	6.23E+03	NULL	AS	Q6ZWB6	KCTD8
1757.1	Isoform 3 of PCNA-interacting p	0.00E+00	6.24E+03	6.24E+03	6.24E+03	NULL	AS	Q9NWS1-3	PARPBP
1716.1	Isoform 2 of Cysteine-rich with E	0.00E+00	6.28E+03	6.28E+03	6.28E+03	NULL	AS	Q96HD1-2	CRELD1
1675.1	TRAF family member-associated	0.00E+00	6.31E+03	6.31E+03	6.31E+03	NULL	AS	Q92844	TANK
2193.1	T-cell leukemia homeobox prote	0.00E+00	6.55E+03	6.55E+03	6.55E+03	NULL	AS	P31314	TLX1

3158.1	Complement receptor type 1	0.00E+00	6.64E+03	6.64E+03	6.64E+03	NULL	AS	E9PDY4	CR1
2252.1	Isoform 6 of Protein WWC2	0.00E+00	6.76E+03	6.76E+03	6.76E+03	NULL	AS	Q6AWC2-(	WWC2
2410.1	WASH complex subunit strumpe	0.00E+00	6.85E+03	6.85E+03	6.85E+03	NULL	AS	Q12768	KIAA0196
1878.1	Protocadherin beta-11	0.00E+00	7.24E+03	7.24E+03	7.24E+03	NULL	AS	Q9Y5F2	PCDHB11
2094.1	Anoctamin-4	0.00E+00	7.24E+03	7.24E+03	7.24E+03	NULL	AS	Q32M45	ANO4
2516.1	Isoform 2 of Nucleolysin TIAR	0.00E+00	7.24E+03	7.24E+03	7.24E+03	NULL	AS	Q01085-2	TIAL1
3257.1	Beta-galactosidase	0.00E+00	7.29E+03	7.29E+03	7.29E+03	NULL	AS	P16278	GLB1
906.1	Zinc finger protein 598	0.00E+00	7.46E+03	7.46E+03	7.46E+03	NULL	AS	Q86UK7	ZNF598
2942.1	Protein disulfide-isomerase A5	0.00E+00	7.79E+03	7.79E+03	7.79E+03	NULL	AS	Q14554	PDIA5
1697.1	C-C motif chemokine 19	0.00E+00	7.89E+03	7.89E+03	7.89E+03	NULL	AS	Q5VZ75	CCL19
2164.1	Protein C15orf38-AP3S2	0.00E+00	7.89E+03	7.89E+03	7.89E+03	NULL	AS	Q7Z6K5	C15orf38
2975.1	Sorting nexin-27	0.00E+00	8.00E+03	8.00E+03	8.00E+03	NULL	AS	Q96L92	SNX27
2689.1	Vacuolar protein sorting-associa	0.00E+00	8.23E+03	8.23E+03	8.23E+03	NULL	AS	Q96QK1	VPS35
2619.1	Zinc finger protein 248	0.00E+00	8.77E+03	8.77E+03	8.77E+03	NULL	AS	Q8NDW4	ZNF248
1705.1	Serine/arginine-rich splicing fact	0.00E+00	8.79E+03	8.79E+03	8.79E+03	NULL	AS	P84103	SRSF3
2296.1	Ig gamma-4 chain C region	0.00E+00	9.08E+03	9.08E+03	9.08E+03	NULL	AS	P01861	IGHG4
2638.1	Survival of motor neuron-relate	0.00E+00	9.14E+03	9.14E+03	9.14E+03	NULL	AS	O75940	SMNDC1
3142.1	Werner syndrome ATP-depende	0.00E+00	9.15E+03	9.15E+03	9.15E+03	NULL	AS	Q14191	WRN
1487.1	Zinc finger protein 469	0.00E+00	9.29E+03	9.29E+03	9.29E+03	NULL	AS	H3BS19	ZNF469
2361.1	Isoform 4 of Kinesin-like protein	0.00E+00	9.39E+03	9.39E+03	9.39E+03	NULL	AS	O60333-4	KIF1B
2552.1	Phospholipase DDHD1	0.00E+00	9.46E+03	9.46E+03	9.46E+03	NULL	AS	Q8NEL9	DDHD1
2871.1	Short transient receptor potenti	0.00E+00	9.56E+03	9.56E+03	9.56E+03	NULL	AS	Q8TEL6	TRPC4AP
2980.1	U4/U6 small nuclear ribonucleo	0.00E+00	9.56E+03	9.56E+03	9.56E+03	NULL	AS	Q8WWY3	PRPF31
1889.1	Leucine-rich repeats and immur	0.00E+00	9.64E+03	9.64E+03	9.64E+03	NULL	AS	Q6UXM1	LRIG3
4.2	Acetyl-CoA carboxylase 2	0.00E+00	9.82E+03	9.82E+03	9.82E+03	NULL	AS	O00763	ACACB
1278.1	Cleavage and polyadenylation s	0.00E+00	9.90E+03	9.90E+03	9.90E+03	NULL	AS	Q9UKF6	CPSF3
1061.1	Isoform 2 of Isochorismatase dc	0.00E+00	1.01E+04	1.01E+04	1.01E+04	NULL	AS	Q96AB3-2	ISOC2
2658.1	Isoform 2 of Protein KIBRA	0.00E+00	1.01E+04	1.01E+04	1.01E+04	NULL	AS	Q8IX03-2	WWC1
1679.1	Putative 60S ribosomal protein l	0.00E+00	1.02E+04	1.02E+04	1.02E+04	NULL	AS	A6NKH3	RPL37L
1306.1	E3 ubiquitin-protein ligase LRSA	0.00E+00	1.03E+04	1.03E+04	1.03E+04	NULL	AS	Q6UWE0	LRSAM1
2478.1	Methyl-CpG-binding domain prc	0.00E+00	1.03E+04	1.03E+04	1.03E+04	NULL	AS	Q96DN6	MBD6
781.2	Isoform 3 of Zinc finger and SCA	0.00E+00	1.06E+04	1.06E+04	1.06E+04	NULL	AS	Q8TBC5-3	ZSCAN18
3049.1	Histone-lysine N-methyltransfer	0.00E+00	1.06E+04	1.06E+04	1.06E+04	NULL	AS	O96028	WHSC1
2541.1	Olfactory receptor 52H1	0.00E+00	1.07E+04	1.07E+04	1.07E+04	NULL	AS	Q8NGJ2	OR52H1
1933.1	Ankyrin repeat domain-containi	0.00E+00	1.14E+04	1.14E+04	1.14E+04	NULL	AS	E9PIL2	ANKRD42

2202.1	Zinc finger protein 555	0.00E+00	1.15E+04	1.15E+04	1.15E+04	NULL	AS	Q8NEP9	ZNF555
1246.1	Abnormal spindle-like microcep	0.00E+00	1.16E+04	1.16E+04	1.16E+04	NULL	AS	Q8IZT6	ASPM
3181.1	Ankyrin repeat domain-containi	0.00E+00	1.16E+04	1.16E+04	1.16E+04	NULL	AS	Q8N283	ANKRD35
3085.1	Beta-galactoside alpha-2,6-sialy	0.00E+00	1.17E+04	1.17E+04	1.17E+04	NULL	AS	P15907	ST6GAL1
3018.1	Isoform 5 of Oxysterol-binding p	0.00E+00	1.18E+04	1.18E+04	1.18E+04	NULL	AS	Q9BZF3-5	OSBPL6
1925.1	TNF receptor-associated factor :	0.00E+00	1.22E+04	1.22E+04	1.22E+04	NULL	AS	Q13077	TRAF1
1005.1	FAST kinase domain-containing	0.00E+00	1.23E+04	1.23E+04	1.23E+04	NULL	AS	Q53R41	FASTKD1
2934.1	Mitochondrial ribonuclease P pr	0.00E+00	1.23E+04	1.23E+04	1.23E+04	NULL	AS	Q7L0Y3	TRMT10C
1958.1	Diamine acetyltransferase 2	0.00E+00	1.24E+04	1.24E+04	1.24E+04	NULL	AS	Q96F10	SAT2
3200.1	BTB/POZ domain-containing prc	0.00E+00	1.24E+04	1.24E+04	1.24E+04	NULL	AS	B4DIP9	KCTD20
1037.1	Fructosamine-3-kinase	0.00E+00	1.25E+04	1.25E+04	1.25E+04	NULL	AS	Q9H479	FN3K
3090.1	Isoform 4 of Probable helicase s	0.00E+00	1.25E+04	1.25E+04	1.25E+04	NULL	AS	Q7Z333-4	SETX
1335.1	Isoform 2 of MOB kinase activat	0.00E+00	1.26E+04	1.26E+04	1.26E+04	NULL	AS	Q7L9L4-2	MOB1B
2661.1	Rho guanine nucleotide exchang	0.00E+00	1.29E+04	1.29E+04	1.29E+04	NULL	AS	Q9NZN5	ARHGEF12
1874.1	Olfactory receptor 51A7	0.00E+00	1.31E+04	1.31E+04	1.31E+04	NULL	AS	Q8NH64	OR51A7
1429.1	Sec1 family domain-containing p	0.00E+00	1.33E+04	1.33E+04	1.33E+04	NULL	AS	Q8WVM8	SCFD1
2803.1	Chromosome 9 open reading fra	0.00E+00	1.33E+04	1.33E+04	1.33E+04	NULL	AS	Q5SQD6	C9orf47
1601.1	GTP:AMP phosphotransferase, r	0.00E+00	1.36E+04	1.36E+04	1.36E+04	NULL	AS	Q9UIJ7	AK3
2782.1	Son of sevenless homolog 1	0.00E+00	1.37E+04	1.37E+04	1.37E+04	NULL	AS	Q07889	SOS1
1303.1	Hyaluronan synthase 3	0.00E+00	1.39E+04	1.39E+04	1.39E+04	NULL	AS	O00219	HAS3
3043.1	Serine/arginine repetitive matri:	0.00E+00	1.44E+04	1.44E+04	1.44E+04	NULL	AS	E9PCT1	SRRM1
927.1	Diacylglycerol kinase eta	0.00E+00	1.45E+04	1.45E+04	1.45E+04	NULL	AS	Q86XP1	DGKH
2070.1	Glycine amidinotransferase, mit	0.00E+00	1.45E+04	1.45E+04	1.45E+04	NULL	AS	P50440	GATM
3102.1	Retinal dehydrogenase 2	0.00E+00	1.47E+04	1.47E+04	1.47E+04	NULL	AS	O94788	ALDH1A2
2246.1	Proteasome assembly chaperon	0.00E+00	1.52E+04	1.52E+04	1.52E+04	NULL	AS	Q969U7	PSMG2
1011.1	UBX domain-containing protein	0.00E+00	1.54E+04	1.54E+04	1.54E+04	NULL	AS	O94888	UBXN7
2129.1	E3 ubiquitin-protein ligase UHRI	0.00E+00	1.54E+04	1.54E+04	1.54E+04	NULL	AS	Q96PU4	UHRF2
2075.1	Isoform 7 of Protein MTO1 hom	0.00E+00	1.55E+04	1.55E+04	1.55E+04	NULL	AS	Q9Y2Z2-6	MTO1
2671.1	Homeobox protein BarH-like 1	0.00E+00	1.55E+04	1.55E+04	1.55E+04	NULL	AS	Q9HBU1	BARX1
1718.1	Isocitrate dehydrogenase [NAD]	0.00E+00	1.57E+04	1.57E+04	1.57E+04	NULL	AS	O43837	IDH3B
1031.1	Adenosine receptor A2b	0.00E+00	1.58E+04	1.58E+04	1.58E+04	NULL	AS	P29275	ADORA2B
1950.1	CUB and sushi domain-containir	0.00E+00	1.58E+04	1.58E+04	1.58E+04	NULL	AS	Q7Z407	CSMD3
2053.1	THO complex subunit 6 homolo	0.00E+00	1.58E+04	1.58E+04	1.58E+04	NULL	AS	Q86W42	THOC6
1497.1	Porphobilinogen deaminase	0.00E+00	1.62E+04	1.62E+04	1.62E+04	NULL	AS	P08397	HMBS
1802.1	Ankyrin repeat and LEM domair	0.00E+00	1.62E+04	1.62E+04	1.62E+04	NULL	AS	Q86XL3	ANKLE2

2939.1	Homeobox protein SIX6	0.00E+00	1.62E+04	1.62E+04	1.62E+04	NULL	AS	O95475	SIX6
2353.1	IQ domain-containing protein E	0.00E+00	1.65E+04	1.65E+04	1.65E+04	NULL	AS	Q6IPM2	IQCE
1754.1	Rho guanine nucleotide exchang	0.00E+00	1.66E+04	1.66E+04	1.66E+04	NULL	AS	Q5VV41	ARHGEF16
3027.1	Guanine nucleotide-binding pro	0.00E+00	1.67E+04	1.67E+04	1.67E+04	NULL	AS	Q14344	GNA13
3215.1	E3 ubiquitin-protein ligase RNF1	0.00E+00	1.67E+04	1.67E+04	1.67E+04	NULL	AS	B7Z3J5	RNF14
2588.1	Ras-specific guanine nucleotide-	0.00E+00	1.68E+04	1.68E+04	1.68E+04	NULL	AS	Q13972	RASGRF1
2392.1	Spermatogenesis-associated pro	0.00E+00	1.69E+04	1.69E+04	1.69E+04	NULL	AS	P0DKV0	SPATA31C1
1713.1	Ankyrin repeat and SAM domair	0.00E+00	1.75E+04	1.75E+04	1.75E+04	NULL	AS	Q92625	ANKS1A
2559.1	Lysine-specific histone demethy	0.00E+00	1.75E+04	1.75E+04	1.75E+04	NULL	AS	F6S0T5	KDM1A
2154.1	GRIP and coiled-coil domain-cor	0.00E+00	1.79E+04	1.79E+04	1.79E+04	NULL	AS	Q8IWJ2	GCC2
2543.1	60S ribosomal protein L39-like	0.00E+00	1.87E+04	1.87E+04	1.87E+04	NULL	AS	Q96EH5	RPL39L
2501.1	Centrosomal protein of 89 kDa	0.00E+00	1.91E+04	1.91E+04	1.91E+04	NULL	AS	K7EJF0	CEP89
1665.1	Plexin-A4	0.00E+00	1.92E+04	1.92E+04	1.92E+04	NULL	AS	Q9HCM2	PLXNA4
1805.1	Nitric oxide synthase-interacting	0.00E+00	1.93E+04	1.93E+04	1.93E+04	NULL	AS	Q9Y314	NOSIP
1655.1	Isoform 3 of Neurabin-1	0.00E+00	1.94E+04	1.94E+04	1.94E+04	NULL	AS	Q9ULJ8-3	PPP1R9A
2960.1	Protein canopy homolog 3	0.00E+00	1.94E+04	1.94E+04	1.94E+04	NULL	AS	Q9BT09	CNPY3
1991.1	Synaptojanin-1	0.00E+00	2.05E+04	2.05E+04	2.05E+04	NULL	AS	J3KQV8	SYNJ1
1131.1	GTP-binding protein 1	0.00E+00	2.08E+04	2.08E+04	2.08E+04	NULL	AS	O00178	GTPBP1
1774.1	Arf-GAP with GTPase, ANK repe	0.00E+00	2.09E+04	2.09E+04	2.09E+04	NULL	AS	E7ESL9	AGAP3
1937.1	SH2 domain-containing adapter	0.00E+00	2.09E+04	2.09E+04	2.09E+04	NULL	AS	Q15464	SHB
2973.1	Beta-2-syntrophin	0.00E+00	2.09E+04	2.09E+04	2.09E+04	NULL	AS	Q13425	SNTB2
2120.1	Transmembrane protein FLJ373	0.00E+00	2.11E+04	2.11E+04	2.11E+04	NULL	AS	Q8N1W0	YF005
2857.1	Rab11 family-interacting protein	0.00E+00	2.11E+04	2.11E+04	2.11E+04	NULL	AS	Q6WKZ4	RAB11FIP1
1948.1	Oligophrenin-1	0.00E+00	2.12E+04	2.12E+04	2.12E+04	NULL	AS	O60890	OPHN1
2904.1	Protein-L-isoaspartate O-methyl	0.00E+00	2.12E+04	2.12E+04	2.12E+04	NULL	AS	Q96MG8	PCMTD1
744.1	Zinc finger protein 287	0.00E+00	2.14E+04	2.14E+04	2.14E+04	NULL	AS	Q9HBT7	ZNF287
1615.1	Actin-related protein 2/3 compl	0.00E+00	2.16E+04	2.16E+04	2.16E+04	NULL	AS	Q9BPX5	ARPC5L
2747.1	Glutamine--fructose-6-phospha	0.00E+00	2.16E+04	2.16E+04	2.16E+04	NULL	AS	O94808	GFPT2
1659.1	Aldo-keto reductase family 1 me	0.00E+00	2.17E+04	2.17E+04	2.17E+04	NULL	AS	A6NHU4	AKR1C1
2429.1	Isoform SOLAT of Threonine syn	0.00E+00	2.19E+04	2.19E+04	2.19E+04	NULL	AS	Q86YJ6-5	THNSL2
308.2	Serine/arginine-rich splicing fact	0.00E+00	2.22E+04	2.22E+04	2.22E+04	NULL	AS	Q08170	SRSF4
1759.1	Isoform 1 of Ena/VASP-like prot	0.00E+00	2.22E+04	2.22E+04	2.22E+04	NULL	AS	Q9UI08-2	EVL
1980.1	Wolframin	0.00E+00	2.24E+04	2.24E+04	2.24E+04	NULL	AS	O76024	WFS1
2832.1	Thyrotropin-releasing hormone-	0.00E+00	2.26E+04	2.26E+04	2.26E+04	NULL	AS	Q9UKU6	TRHDE
3075.1	NACHT, LRR and PYD domains-c	0.00E+00	2.28E+04	2.28E+04	2.28E+04	NULL	AS	Q86W24	NLRP14

2668.1	Helicase SRCAP	0.00E+00	2.33E+04	2.33E+04	2.33E+04	NULL	AS	Q6ZRS2	SRCAP
1466.1	Nuclear fragile X mental retarda	0.00E+00	2.35E+04	2.35E+04	2.35E+04	NULL	AS	Q7Z417	NUFIP2
2872.1	Protein CMSS1	0.00E+00	2.35E+04	2.35E+04	2.35E+04	NULL	AS	Q9BQ75	CMSS1
1808.1	Coiled-coil domain-containing p	0.00E+00	2.36E+04	2.36E+04	2.36E+04	NULL	AS	Q567U6	CCDC93
1671.1	E3 ubiquitin-protein ligase NEDI	0.00E+00	2.38E+04	2.38E+04	2.38E+04	NULL	AS	P46934	NEDD4
2322.1	Neurexin-2-alpha	0.00E+00	2.38E+04	2.38E+04	2.38E+04	NULL	AS	Q9P2S2	NRXN2
966.1	MKL/myocardin-like 2, isoform (	0.00E+00	2.44E+04	2.44E+04	2.44E+04	NULL	AS	B4DGT8	MKL2
2824.1	Clathrin heavy chain linker doma	0.00E+00	2.44E+04	2.44E+04	2.44E+04	NULL	AS	Q8NHS4	CLHC1
2024.1	RUN and FYVE domain-containir	0.00E+00	2.47E+04	2.47E+04	2.47E+04	NULL	AS	Q8WXA3	RUFY2
886.1	DnaJ homolog subfamily C mem	0.00E+00	2.50E+04	2.50E+04	2.50E+04	NULL	AS	Q96KC8	DNAJC1
2885.1	Pancreas/duodenum homeobox	0.00E+00	2.50E+04	2.50E+04	2.50E+04	NULL	AS	P52945	PDX1
2290.1	Mitochondrial import inner mer	0.00E+00	2.57E+04	2.57E+04	2.57E+04	NULL	AS	O43615	TIMM44
3211.1	Isoform 2 of Suppressor of IKBK	0.00E+00	2.67E+04	2.67E+04	2.67E+04	NULL	AS	Q9BRV8-2	SIKE1
3214.1	DNA-directed RNA polymerase I	0.00E+00	2.69E+04	2.69E+04	2.69E+04	NULL	AS	Q9NVU0	POLR3E
2060.1	ATP-sensitive inward rectifier pc	0.00E+00	2.72E+04	2.72E+04	2.72E+04	NULL	AS	Q15842	KCNJ8
2355.1	Isoform 2 of Voltage-dependent	0.00E+00	2.73E+04	2.73E+04	2.73E+04	NULL	AS	Q13936-2	CACNA1C
2550.1	Ras-related protein Rab-5B	0.00E+00	2.73E+04	2.73E+04	2.73E+04	NULL	AS	P61020	RAB5B
1658.1	Structural maintenance of chror	0.00E+00	2.74E+04	2.74E+04	2.74E+04	NULL	AS	Q9UQE7	SMC3
1638.1	Bone morphogenetic protein 4	0.00E+00	2.78E+04	2.78E+04	2.78E+04	NULL	AS	P12644	BMP4
2371.1	Platelet-derived growth factor r	0.00E+00	2.78E+04	2.78E+04	2.78E+04	NULL	AS	P09619	PDGFRB
866.1	Asparagine synthetase [glutamir	0.00E+00	2.79E+04	2.79E+04	2.79E+04	NULL	AS	P08243	ASNS
1430.1	Coiled-coil domain-containing p	0.00E+00	2.80E+04	2.80E+04	2.80E+04	NULL	AS	Q0VFZ6	CCDC173
1213.1	Isoform 3 of Ataxin-2-like protei	0.00E+00	2.82E+04	2.82E+04	2.82E+04	NULL	AS	Q8WWM7	ATXN2L
2130.1	Nuclear pore complex-interactir	0.00E+00	2.83E+04	2.83E+04	2.83E+04	NULL	AS	C9JG80	NPIPL3
1920.1	Poliovirus receptor-related prot	0.00E+00	2.89E+04	2.89E+04	2.89E+04	NULL	AS	Q15223	PVRL1
1928.1	Deleted in bladder cancer prote	0.00E+00	2.93E+04	2.93E+04	2.93E+04	NULL	AS	O60477	DBC1
2581.1	Integrator complex subunit 5	0.00E+00	2.93E+04	2.93E+04	2.93E+04	NULL	AS	Q6P9B9	INTS5
1326.1	Lys-63-specific deubiquitinase B	0.00E+00	2.94E+04	2.94E+04	2.94E+04	NULL	AS	P46736	BRCC3
2377.1	Cysteine/serine-rich nuclear prc	0.00E+00	2.94E+04	2.94E+04	2.94E+04	NULL	AS	Q9H175	CSRNP2
2707.1	Ankyrin repeat domain-containi	0.00E+00	2.96E+04	2.96E+04	2.96E+04	NULL	AS	H7C425	ANKRD36C
1499.1	Rho guanine nucleotide exchang	0.00E+00	2.98E+04	2.98E+04	2.98E+04	NULL	AS	Q96PE2	ARHGEF17
2151.1	THO complex subunit 7 homolog	0.00E+00	2.99E+04	2.99E+04	2.99E+04	NULL	AS	Q6I9Y2	THOC7
2395.1	Apoptotic chromatin condensat	0.00E+00	3.00E+04	3.00E+04	3.00E+04	NULL	AS	Q9UKV3	ACIN1
3236.1	SLAIN motif-containing protein :	0.00E+00	3.03E+04	3.03E+04	3.03E+04	NULL	AS	Q9P270	SLAIN2
2561.1	RNA-binding protein 26	0.00E+00	3.04E+04	3.04E+04	3.04E+04	NULL	AS	Q5T8P6	RBM26

1291.1	FCH and double SH3 domains pr	0.00E+00	3.05E+04	3.05E+04	3.05E+04	NULL	AS	E9PG19	FCHSD2
2082.1	Protein FAM216A	0.00E+00	3.07E+04	3.07E+04	3.07E+04	NULL	AS	Q8WUB2	FAM216A
2627.1	Isoform 2 of WNT1-inducible-sig	0.00E+00	3.10E+04	3.10E+04	3.10E+04	NULL	AS	O95389-2	WISP3
1170.1	Dynein heavy chain 11, axonem	0.00E+00	3.12E+04	3.12E+04	3.12E+04	NULL	AS	Q96DT5	DNAH11
1826.1	G protein-activated inward recti	0.00E+00	3.12E+04	3.12E+04	3.12E+04	NULL	AS	P48051	KCNJ6
2709.1	Protein bassoon	0.00E+00	3.12E+04	3.12E+04	3.12E+04	NULL	AS	Q9UPA5	BSN
1764.1	Spectrin beta chain, non-erythro	0.00E+00	3.17E+04	3.17E+04	3.17E+04	NULL	AS	Q01082	SPTBN1
2066.1	Histone-lysine N-methyltransfer	0.00E+00	3.17E+04	3.17E+04	3.17E+04	NULL	AS	Q86TU7	SETD3
2336.1	RNA-binding motif protein, X ch	0.00E+00	3.17E+04	3.17E+04	3.17E+04	NULL	AS	P38159	RBMX
3183.1	Interferon-induced protein with	0.00E+00	3.20E+04	3.20E+04	3.20E+04	NULL	AS	Q13325	IFIT5
1760.1	3-ketoacyl-CoA thiolase, peroxis	0.00E+00	3.21E+04	3.21E+04	3.21E+04	NULL	AS	P09110	ACAA1
3093.1	Dual adapter for phosphotyrosi	0.00E+00	3.28E+04	3.28E+04	3.28E+04	NULL	AS	Q9UN19	DAPP1
1373.1	Leucine-rich repeat-containing p	0.00E+00	3.34E+04	3.34E+04	3.34E+04	NULL	AS	B4DJ72	LRRC27
3240.1	Acetoacetyl-CoA synthetase	0.00E+00	3.36E+04	3.36E+04	3.36E+04	NULL	AS	F5GYW2	AACS
1814.1	ADNP homeobox protein 2	0.00E+00	3.42E+04	3.42E+04	3.42E+04	NULL	AS	Q6IQ32	ADNP2
2497.1	Endonuclease 8-like 2	0.00E+00	3.43E+04	3.43E+04	3.43E+04	NULL	AS	Q969S2	NEIL2
2970.1	Prickle-like protein 2	0.00E+00	3.46E+04	3.46E+04	3.46E+04	NULL	AS	H3BNU0	PRICKLE2
1668.1	Protein DENND6B	0.00E+00	3.48E+04	3.48E+04	3.48E+04	NULL	AS	Q8NEG7	DENND6B
1331.1	Chloride channel CLIC-like prote	0.00E+00	3.50E+04	3.50E+04	3.50E+04	NULL	AS	Q96S66	CLCC1
3227.1	Noggin	0.00E+00	3.57E+04	3.57E+04	3.57E+04	NULL	AS	Q13253	NOG
1286.1	Translation initiation factor eIF-:	0.00E+00	3.62E+04	3.62E+04	3.62E+04	NULL	AS	P49770	EIF2B2
2085.1	Protein FAM149B1	0.00E+00	3.64E+04	3.64E+04	3.64E+04	NULL	AS	Q96BN6	FAM149B1
2622.1	Inhibitor of Bruton tyrosine kina	0.00E+00	3.64E+04	3.64E+04	3.64E+04	NULL	AS	Q9P2D0	IBTK
2277.1	Heat shock 70 kDa protein 4	0.00E+00	3.65E+04	3.65E+04	3.65E+04	NULL	AS	P34932	HSPA4
2312.1	Coiled-coil domain-containing p	0.00E+00	3.66E+04	3.66E+04	3.66E+04	NULL	AS	Q9NQR7	CCDC177
2352.1	Zinc finger protein 678	0.00E+00	3.71E+04	3.71E+04	3.71E+04	NULL	AS	F5GXA7	ZNF678
2203.1	Spermatogenesis-associated pro	0.00E+00	3.74E+04	3.74E+04	3.74E+04	NULL	AS	Q5VX52	SPATA1
2466.1	Peroxisome proliferator-activate	0.00E+00	3.76E+04	3.76E+04	3.76E+04	NULL	AS	Q86YN6	PPARGC1B
372.2	Nucleosome assembly protein 1	0.00E+00	3.78E+04	3.78E+04	3.78E+04	NULL	AS	F5HFY4	NAP1L4b
2472.1	Protein AKNAD1	0.00E+00	3.78E+04	3.78E+04	3.78E+04	NULL	AS	F2Z2Q7	AKNAD1
1873.1	Isoform 4 of Coiled-coil domain-	0.00E+00	3.79E+04	3.79E+04	3.79E+04	NULL	AS	Q6ZUS6-4	CCDC149
1782.1	Zinc finger protein 33A	0.00E+00	3.82E+04	3.82E+04	3.82E+04	NULL	AS	F6TH33	ZNF33A
2427.1	CD2 antigen cytoplasmic tail-bir	0.00E+00	3.85E+04	3.85E+04	3.85E+04	NULL	AS	O95400	CD2BP2
3116.1	Dimethylaniline monooxygenas	0.00E+00	3.85E+04	3.85E+04	3.85E+04	NULL	AS	P49326	FMO5
2673.1	Zinc finger protein GLI1	0.00E+00	3.87E+04	3.87E+04	3.87E+04	NULL	AS	P08151	GLI1

2930.1	Serine/threonine-protein kinase	0.00E+00	3.93E+04	3.93E+04	3.93E+04	NULL	AS	Q8IYT8	ULK2
2161.1	Polymerase delta-interacting pr	0.00E+00	4.00E+04	4.00E+04	4.00E+04	NULL	AS	Q9Y2S7	POLDIP2
2485.1	Isoform 3 of Epithelial-stromal i	0.00E+00	4.00E+04	4.00E+04	4.00E+04	NULL	AS	Q96J88-3	EPSTI1
2899.1	Zinc finger protein 564	0.00E+00	4.01E+04	4.01E+04	4.01E+04	NULL	AS	Q8TBZ8	ZNF564
3008.1	Podocan-like protein 1	0.00E+00	4.06E+04	4.06E+04	4.06E+04	NULL	AS	Q6PEZ8	PODNL1
2189.1	Membrane-associated phospha	0.00E+00	4.08E+04	4.08E+04	4.08E+04	NULL	AS	Q9BZ72	PITPNM2
1369.1	General transcription factor II-I	0.00E+00	4.13E+04	4.13E+04	4.13E+04	NULL	AS	P78347	GTF2I
810.1	Isoform 6 of Dynamin-1-like pro	0.00E+00	4.15E+04	4.15E+04	4.15E+04	NULL	AS	O00429-6	DNM1L
2217.1	Protein MCM10 homolog	0.00E+00	4.18E+04	4.18E+04	4.18E+04	NULL	AS	Q7L590	MCM10
1673.1	Isoform 3 of Zinc finger protein	0.00E+00	4.20E+04	4.20E+04	4.20E+04	NULL	AS	Q5VV52-3	ZNF691
638.1	YTH domain family protein 2	0.00E+00	4.27E+04	4.27E+04	4.27E+04	NULL	AS	Q9Y5A9	YTHDF2
1069.1	Pinin	0.00E+00	4.27E+04	4.27E+04	4.27E+04	NULL	AS	Q9H307	PNN
3167.1	Coiled-coil domain-containing p	0.00E+00	4.28E+04	4.28E+04	4.28E+04	NULL	AS	Q8IYE0	CCDC146
1837.1	Transcription factor Sp2	0.00E+00	4.31E+04	4.31E+04	4.31E+04	NULL	AS	Q02086	SP2
1898.1	WD repeat-containing protein 6	0.00E+00	4.32E+04	4.32E+04	4.32E+04	NULL	AS	Q8WVS4	WDR60
2103.1	Isoform 2 of TGF-beta receptor	0.00E+00	4.32E+04	4.32E+04	4.32E+04	NULL	AS	P37173-2	TGFBR2
3132.1	Fibronectin type-III domain-con	0.00E+00	4.33E+04	4.33E+04	4.33E+04	NULL	AS	F2Z333	C1orf233
1116.1	Testis-specific Y-encoded-like pr	0.00E+00	4.34E+04	4.34E+04	4.34E+04	NULL	AS	Q9H0U9	TSPYL1
1749.1	Beta-catenin-interacting protein	0.00E+00	4.41E+04	4.41E+04	4.41E+04	NULL	AS	Q9NSA3	CTNNBIP1
2345.1	Cyclin-dependent kinase 7	0.00E+00	4.41E+04	4.41E+04	4.41E+04	NULL	AS	P50613	CDK7
2526.1	Thimet oligopeptidase	0.00E+00	4.41E+04	4.41E+04	4.41E+04	NULL	AS	P52888	THOP1
2959.1	A disintegrin and metalloprotein	0.00E+00	4.41E+04	4.41E+04	4.41E+04	NULL	AS	Q8TE58	ADAMTS15
2845.1	Zinc finger protein ZFPM2	0.00E+00	4.51E+04	4.51E+04	4.51E+04	NULL	AS	Q8WW38	ZFPM2
1174.1	Ornithine decarboxylase antizym	0.00E+00	4.55E+04	4.55E+04	4.55E+04	NULL	AS	P54368	OAZ1
2651.1	Protein phosphatase 1 regulator	0.00E+00	4.55E+04	4.55E+04	4.55E+04	NULL	AS	Q7Z4L9	PPP1R42
2482.1	Transcriptional activator protein	0.00E+00	4.56E+04	4.56E+04	4.56E+04	NULL	AS	Q00577	PURA
2521.1	Collagen alpha-1(XXII) chain	0.00E+00	4.61E+04	4.61E+04	4.61E+04	NULL	AS	Q8NFW1	COL22A1
2412.1	Transcription elongation factor	0.00E+00	4.63E+04	4.63E+04	4.63E+04	NULL	AS	Q14241	TCEB3
2165.1	Exportin-T	0.00E+00	4.65E+04	4.65E+04	4.65E+04	NULL	AS	O43592	XPOT
2910.1	Zinc finger protein 253	0.00E+00	4.73E+04	4.73E+04	4.73E+04	NULL	AS	O75346	ZNF253
2741.1	Fumarate hydratase, mitochonc	0.00E+00	4.74E+04	4.74E+04	4.74E+04	NULL	AS	P07954	FH
2998.1	Coiled-coil domain-containing p	0.00E+00	4.76E+04	4.76E+04	4.76E+04	NULL	AS	J3KNW7	CCDC41
2071.1	Dihydropyrimidinase-related pro	0.00E+00	4.77E+04	4.77E+04	4.77E+04	NULL	AS	O14531	DPYSL4
3036.1	RUN domain-containing protein	0.00E+00	4.81E+04	4.81E+04	4.81E+04	NULL	AS	Q59EK9	RUNDC3A
2238.1	Lysine-specific demethylase 4D	0.00E+00	4.82E+04	4.82E+04	4.82E+04	NULL	AS	Q6B0I6	KDM4D

1635.1	Proteasomal ubiquitin receptor	0.00E+00	4.83E+04	4.83E+04	4.83E+04	NULL	AS	Q16186	ADRM1
2459.1	Isoform 2 of AF4/FMR2 family m	0.00E+00	4.83E+04	4.83E+04	4.83E+04	NULL	AS	P51826-2	AFF3
2049.1	Caspase recruitment domain-co	0.00E+00	4.87E+04	4.87E+04	4.87E+04	NULL	AS	Q9BWT7	CARD10
2879.1	Putative unconventional myosin	0.00E+00	4.90E+04	4.90E+04	4.90E+04	NULL	AS	Q96JP2	MYO15B
2372.1	Cornifin-B	0.00E+00	4.93E+04	4.93E+04	4.93E+04	NULL	AS	P22528	SPRR1B
3199.1	E3 ubiquitin-protein ligase TRIM	0.00E+00	4.96E+04	4.96E+04	4.96E+04	NULL	AS	G8JLN1	TRIM31
2091.1	Coiled-coil domain-containing p	0.00E+00	4.98E+04	4.98E+04	4.98E+04	NULL	AS	E9PKB1	CCDC15
2943.1	Signal transducer and activator	0.00E+00	4.98E+04	4.98E+04	4.98E+04	NULL	AS	Q14765	STAT4
1559.1	SAFB-like transcription modulat	0.00E+00	5.03E+04	5.03E+04	5.03E+04	NULL	AS	Q9NWH9	SLTM
1264.1	Echinoderm microtubule-associ	0.00E+00	5.05E+04	5.05E+04	5.05E+04	NULL	AS	Q6ZMW3	EML6
2462.1	Ubiquilin-3	0.00E+00	5.06E+04	5.06E+04	5.06E+04	NULL	AS	Q9H347	UBQLN3
2647.1	Receptor tyrosine-protein kinase	0.00E+00	5.06E+04	5.06E+04	5.06E+04	NULL	AS	Q15303	ERBB4
2524.1	Signal recognition particle 72 kD	0.00E+00	5.08E+04	5.08E+04	5.08E+04	NULL	AS	O76094	SRP72
1201.1	POC1 centriolar protein homolo	0.00E+00	5.10E+04	5.10E+04	5.10E+04	NULL	AS	F8VV91	POC1B
521.2	Transducin-like enhancer protei	0.00E+00	5.12E+04	5.12E+04	5.12E+04	NULL	AS	H0YL70	TLE3
1608.1	Cold shock domain containing E	0.00E+00	5.14E+04	5.14E+04	5.14E+04	NULL	AS	G5E9Q2	CSDE1
1685.1	Kinesin-like protein KIF15	0.00E+00	5.17E+04	5.17E+04	5.17E+04	NULL	AS	Q9NS87	KIF15
1746.1	Arf-GAP domain and FG repeat-	0.00E+00	5.17E+04	5.17E+04	5.17E+04	NULL	AS	O95081	AGFG2
2799.1	PRKC apoptosis WT1 regulator p	0.00E+00	5.17E+04	5.17E+04	5.17E+04	NULL	AS	Q96IZ0	PAWR
1589.1	60S ribosomal protein L17	0.00E+00	5.20E+04	5.20E+04	5.20E+04	NULL	AS	P18621	RPL17
1719.1	Mitogen-activated protein kinas	0.00E+00	5.25E+04	5.25E+04	5.25E+04	NULL	AS	Q02779	MAP3K10
2966.1	Arginyl-tRNA--protein transferas	0.00E+00	5.25E+04	5.25E+04	5.25E+04	NULL	AS	O95260	ATE1
2692.1	Dermokine	0.00E+00	5.28E+04	5.28E+04	5.28E+04	NULL	AS	Q6E0U4	DMKN
2640.1	PTB-containing, cubilin and LRP1	0.00E+00	5.31E+04	5.31E+04	5.31E+04	NULL	AS	Q7Z2X4	PID1
1890.1	Ubiquitin-like-conjugating enzyr	0.00E+00	5.38E+04	5.38E+04	5.38E+04	NULL	AS	Q9NT62	ATG3
1941.1	Golgi integral membrane protei	0.00E+00	5.38E+04	5.38E+04	5.38E+04	NULL	AS	O00461	GOLIM4
1257.1	Nuclear receptor-binding factor	0.00E+00	5.44E+04	5.44E+04	5.44E+04	NULL	AS	B4DWS0	NRBF2
2431.1	Ephrin type-B receptor 1	0.00E+00	5.46E+04	5.46E+04	5.46E+04	NULL	AS	P54762	EPHB1
1356.1	Arrestin domain-containing prot	0.00E+00	5.49E+04	5.49E+04	5.49E+04	NULL	AS	Q8TBH0	ARRDC2
1517.1	Armadillo repeat-containing prc	0.00E+00	5.53E+04	5.53E+04	5.53E+04	NULL	AS	Q5W041	ARMC3
2225.1	Isoform 2 of Secernin-1	0.00E+00	5.53E+04	5.53E+04	5.53E+04	NULL	AS	Q12765-2	SCRN1
2949.1	Biotinidase	0.00E+00	5.53E+04	5.53E+04	5.53E+04	NULL	AS	F8WCU5	BTD
1823.1	EMILIN-1	0.00E+00	5.57E+04	5.57E+04	5.57E+04	NULL	AS	Q9Y6C2	EMILIN1
2710.1	Adenosine deaminase-like prote	0.00E+00	5.68E+04	5.68E+04	5.68E+04	NULL	AS	Q6DHV7	ADAL
3222.1	REST corepressor 1	0.00E+00	5.73E+04	5.73E+04	5.73E+04	NULL	AS	J3KN32	RCOR1

1305.1	Transforming growth factor-bet	0.00E+00	5.75E+04	5.75E+04	5.75E+04	NULL	AS	Q8WUH2	TGFBRAP1
5.2	Keratin, type I cuticular Ha5	0.00E+00	5.77E+04	5.77E+04	5.77E+04	NULL	AS	C4AM86	KRT35
2347.1	Transcription factor E4F1	0.00E+00	5.81E+04	5.81E+04	5.81E+04	NULL	AS	Q66K89	E4F1
3056.1	PHD finger protein 6	0.00E+00	5.84E+04	5.84E+04	5.84E+04	NULL	AS	E9PC97	PHF6
2730.1	Type I inositol 1,4,5-trisphospha	0.00E+00	5.86E+04	5.86E+04	5.86E+04	NULL	AS	Q14642	INPP5A
2768.1	Isoform Beta of Apoptosis regul	0.00E+00	5.86E+04	5.86E+04	5.86E+04	NULL	AS	Q07812-2	BAX
2922.1	Kinesin-like protein KIF24	0.00E+00	5.87E+04	5.87E+04	5.87E+04	NULL	AS	Q5T7B8	KIF24
2764.1	Retinol dehydrogenase 11	0.00E+00	5.88E+04	5.88E+04	5.88E+04	NULL	AS	G3V3K0	RDH11
2138.1	Semaphorin-3F	0.00E+00	5.90E+04	5.90E+04	5.90E+04	NULL	AS	Q13275	SEMA3F
2646.1	Coiled-coil domain-containing p	0.00E+00	5.90E+04	5.90E+04	5.90E+04	NULL	AS	C9JGU1	CCDC66
2829.1	Isoform 2 of Peroxisomal acyl-co	0.00E+00	5.90E+04	5.90E+04	5.90E+04	NULL	AS	Q15067-2	ACOX1
2006.1	PHD finger protein 21A	0.00E+00	5.96E+04	5.96E+04	5.96E+04	NULL	AS	Q96BD5	PHF21A
2149.1	Isoform 2 of Tapasin	0.00E+00	6.01E+04	6.01E+04	6.01E+04	NULL	AS	O15533-2	TAPBP
2335.1	Calcium-binding protein 5	0.00E+00	6.01E+04	6.01E+04	6.01E+04	NULL	AS	Q9NP86	CABP5
2967.1	EH domain-binding protein 1	0.00E+00	6.02E+04	6.02E+04	6.02E+04	NULL	AS	Q8NDI1	EHBP1
1740.1	WD repeat-containing protein 7	0.00E+00	6.04E+04	6.04E+04	6.04E+04	NULL	AS	Q9NW82	WDR70
1600.1	Protein SMIM12	0.00E+00	6.06E+04	6.06E+04	6.06E+04	NULL	AS	E5RH51	SMIM12
1026.1	Progesterone-induced-blocking	0.00E+00	6.09E+04	6.09E+04	6.09E+04	NULL	AS	Q8WXW3	PIBF1
2394.1	Serine/threonine-protein kinase	0.00E+00	6.11E+04	6.11E+04	6.11E+04	NULL	AS	Q9BVS4	RIOK2
3092.1	Isoform 2 of Perilipin-4	0.00E+00	6.15E+04	6.15E+04	6.15E+04	NULL	AS	Q96Q06-2	PLIN4
1558.1	Zinc finger and SCAN domain-co	0.00E+00	6.16E+04	6.16E+04	6.16E+04	NULL	AS	Q96SZ4	ZSCAN10
2723.1	Isoform 2 of TGF-beta receptor	0.00E+00	6.23E+04	6.23E+04	6.23E+04	NULL	AS	P36897-2	TGFBR1
2781.1	Lethal(3)malignant brain tumor-	0.00E+00	6.23E+04	6.23E+04	6.23E+04	NULL	AS	Q8NA19	L3MBTL4
3035.1	Protein GREB1	0.00E+00	6.24E+04	6.24E+04	6.24E+04	NULL	AS	Q4ZG55	GREB1
2994.1	Isoform 3 of F-box only protein	0.00E+00	6.32E+04	6.32E+04	6.32E+04	NULL	AS	O75426-3	FBXO24
2126.1	G antigen family E member 1	0.00E+00	6.33E+04	6.33E+04	6.33E+04	NULL	AS	Q96GU1	PAGE5
1720.1	Melanoma-associated antigen B	0.00E+00	6.34E+04	6.34E+04	6.34E+04	NULL	AS	A8MXT2	MAGEB17
2582.1	Isoform 6 of A-kinase anchor pr	0.00E+00	6.34E+04	6.34E+04	6.34E+04	NULL	AS	Q99996-6	AKAP9
3231.1	Poly [ADP-ribose] polymerase 4	0.00E+00	6.34E+04	6.34E+04	6.34E+04	NULL	AS	Q9UKK3	PARP4
2147.1	Zinc finger protein with KRAB ar	0.00E+00	6.40E+04	6.40E+04	6.40E+04	NULL	AS	Q9BRR0	ZKSCAN3
1135.1	Eukaryotic translation initiation	0.00E+00	6.42E+04	6.42E+04	6.42E+04	NULL	AS	Q13347	EIF3I
1410.1	Methionine--tRNA ligase, mitocl	0.00E+00	6.42E+04	6.42E+04	6.42E+04	NULL	AS	Q96GW9	MARS2
1562.1	Unconventional myosin-Id	0.00E+00	6.42E+04	6.42E+04	6.42E+04	NULL	AS	O94832	MYO1D
1856.1	C-X-C motif chemokine 2	0.00E+00	6.42E+04	6.42E+04	6.42E+04	NULL	AS	P19875	CXCL2
2007.1	Histone-lysine N-methyltransfer	0.00E+00	6.44E+04	6.44E+04	6.44E+04	NULL	AS	Q9UMN6	KMT2B

1848.1	Centrosome-associated protein	0.00E+00	6.48E+04	6.48E+04	6.48E+04	NULL	AS	Q9BV73	CEP250
2255.1	Eukaryotic translation initiation	0.00E+00	6.51E+04	6.51E+04	6.51E+04	NULL	AS	P60228	EIF3E
1946.1	Quinone oxidoreductase	0.00E+00	6.55E+04	6.55E+04	6.55E+04	NULL	AS	Q08257	CRYZ
2220.1	Transcription cofactor HES-6	0.00E+00	6.58E+04	6.58E+04	6.58E+04	NULL	AS	Q96HZ4	HES6
2706.1	Sodium/potassium-transporting	0.00E+00	6.60E+04	6.60E+04	6.60E+04	NULL	AS	Q13733	ATP1A4
2473.1	Integrin alpha-V	0.00E+00	6.61E+04	6.61E+04	6.61E+04	NULL	AS	P06756	ITGAV
2286.1	Speriolin	0.00E+00	6.63E+04	6.63E+04	6.63E+04	NULL	AS	Q76KD6	SPATC1
985.1	Ig kappa chain V-III region HIC	0.00E+00	6.64E+04	6.64E+04	6.64E+04	NULL	AS	P18136	KV313
2682.1	B-cell CLL/lymphoma 7 protein f	0.00E+00	6.65E+04	6.65E+04	6.65E+04	NULL	AS	F8WDZ4	BCL7B
793.1	Protein quaking	0.00E+00	6.67E+04	6.67E+04	6.67E+04	NULL	AS	Q96PU8	QKI
1915.1	Guanine nucleotide-binding pro	0.00E+00	6.78E+04	6.78E+04	6.78E+04	NULL	AS	Q03113	GNA12
1793.1	Zinc finger protein 485	0.00E+00	6.79E+04	6.79E+04	6.79E+04	NULL	AS	Q8NCK3	ZNF485
921.1	ADP-ribosylation factor-like prot	0.00E+00	6.80E+04	6.80E+04	6.80E+04	NULL	AS	P36405	ARL3
1598.1	Splicing factor 45	0.00E+00	6.82E+04	6.82E+04	6.82E+04	NULL	AS	Q96I25	RBM17
2303.1	Proline-rich protein 7	0.00E+00	6.82E+04	6.82E+04	6.82E+04	NULL	AS	Q8TB68	PRR7
2330.1	cAMP-responsive element-bindi	0.00E+00	6.82E+04	6.82E+04	6.82E+04	NULL	AS	O60519	CREBL2
3129.1	Inositol 1,4,5-trisphosphate rece	0.00E+00	6.83E+04	6.83E+04	6.83E+04	NULL	AS	Q14643	ITPR1
2454.1	Myosin-15	0.00E+00	6.84E+04	6.84E+04	6.84E+04	NULL	AS	Q9Y2K3	MYH15
997.1	Methylosome protein 50	0.00E+00	6.88E+04	6.88E+04	6.88E+04	NULL	AS	Q9BQA1	WDR77
2500.1	Isoform 2 of Protein TANC2	0.00E+00	6.92E+04	6.92E+04	6.92E+04	NULL	AS	Q9HCD6-2	TANC2
3162.1	Cholecystokinin receptor type A	0.00E+00	6.95E+04	6.95E+04	6.95E+04	NULL	AS	P32238	CCKAR
509.1	Alpha/beta hydrolase domain-co	0.00E+00	6.97E+04	6.97E+04	6.97E+04	NULL	AS	Q8NFV4	ABHD11
1500.1	Whirlin	0.00E+00	7.08E+04	7.08E+04	7.08E+04	NULL	AS	Q9P202	DFNB31
1539.1	SH2 domain-containing protein	0.00E+00	7.09E+04	7.09E+04	7.09E+04	NULL	AS	A6NKC9	SH2D7
910.1	Microsomal triglyceride transfer	0.00E+00	7.14E+04	7.14E+04	7.14E+04	NULL	AS	E9PBP6	MTTP
935.1	RNA-binding protein 25	0.00E+00	7.18E+04	7.18E+04	7.18E+04	NULL	AS	P49756	RBM25
2515.1	Coiled-coil domain-containing p	0.00E+00	7.19E+04	7.19E+04	7.19E+04	NULL	AS	B8ZZV3	CCDC148
1570.1	Large neutral amino acids trans	0.00E+00	7.21E+04	7.21E+04	7.21E+04	NULL	AS	Q9UHI5	SLC7A8
2023.1	Isoform 2 of Probable gluconoki	0.00E+00	7.23E+04	7.23E+04	7.23E+04	NULL	AS	Q5T6J7-2	IDNK
2343.1	Phosphatidylinositol 4,5-bisphos	0.00E+00	7.27E+04	7.27E+04	7.27E+04	NULL	AS	Q15735	INPP5J
1419.1	Isoform 3 of Protein PRRC2A	0.00E+00	7.29E+04	7.29E+04	7.29E+04	NULL	AS	P48634-3	PRRC2A
667.1	Metallothionein-1G	0.00E+00	7.31E+04	7.31E+04	7.31E+04	NULL	AS	P13640	MT1G
1628.1	Transgelin-3	0.00E+00	7.35E+04	7.35E+04	7.35E+04	NULL	AS	Q9UI15	TAGLN3
1953.1	Rho guanine nucleotide exchang	0.00E+00	7.37E+04	7.37E+04	7.37E+04	NULL	AS	Q8N1W1	ARHGEF28
2218.1	Tankyrase-2	0.00E+00	7.44E+04	7.44E+04	7.44E+04	NULL	AS	Q9H2K2	TNKS2

2725.1	Aldose reductase	0.00E+00	7.47E+04	7.47E+04	7.47E+04	NULL	AS	P15121	AKR1B1
1094.1	Pre-mRNA-splicing factor RBM2	0.00E+00	7.48E+04	7.48E+04	7.48E+04	NULL	AS	Q9NW64	RBM22
2744.1	Actin filament-associated protei	0.00E+00	7.51E+04	7.51E+04	7.51E+04	NULL	AS	Q8TED9	AFAP1L1
1253.1	Maleylacetoacetate isomerase	0.00E+00	7.55E+04	7.55E+04	7.55E+04	NULL	AS	O43708	GSTZ1
2166.1	Isoform 3 of Deleted in lung anc	0.00E+00	7.61E+04	7.61E+04	7.61E+04	NULL	AS	Q9Y238-3	DLEC1
3107.1	Zinc finger protein 221	0.00E+00	7.63E+04	7.63E+04	7.63E+04	NULL	AS	Q9UK13	ZNF221
2624.1	Pre-mRNA-splicing factor SYF1	0.00E+00	7.66E+04	7.66E+04	7.66E+04	NULL	AS	Q9HCS7	XAB2
1524.1	Protein SFI1 homolog	0.00E+00	7.67E+04	7.67E+04	7.67E+04	NULL	AS	A8K8P3	SFI1
1553.1	ADP/ATP translocase 3	0.00E+00	7.67E+04	7.67E+04	7.67E+04	NULL	AS	P12236	SLC25A6
2808.1	Calcium-binding protein 39	0.00E+00	7.67E+04	7.67E+04	7.67E+04	NULL	AS	Q9Y376	CAB39
3237.1	rRNA-processing protein UTP23	0.00E+00	7.67E+04	7.67E+04	7.67E+04	NULL	AS	Q9BRU9	UTP23
2674.1	2-oxoisovalerate dehydrogenase	0.00E+00	7.72E+04	7.72E+04	7.72E+04	NULL	AS	P21953	BCKDHB
2869.1	Transmembrane protease serine	0.00E+00	7.73E+04	7.73E+04	7.73E+04	NULL	AS	Q7Z410	TMPRSS9
1346.1	GTP-binding protein SAR1b	0.00E+00	7.76E+04	7.76E+04	7.76E+04	NULL	AS	Q9Y6B6	SAR1B
2602.1	HAUS augmin-like complex subu	0.00E+00	7.77E+04	7.77E+04	7.77E+04	NULL	AS	Q9H6D7	HAUS4
1828.1	Cyclin-dependent kinase inhibit	0.00E+00	7.83E+04	7.83E+04	7.83E+04	NULL	AS	J3KQV0	CDKN1A
987.1	Perilipin-3	0.00E+00	7.86E+04	7.86E+04	7.86E+04	NULL	AS	O60664	PLIN3
1767.1	Eukaryotic translation initiation	0.00E+00	7.87E+04	7.87E+04	7.87E+04	NULL	AS	B0QY89	EIF3L
1845.1	Isoform 2 of Eukaryotic translati	0.00E+00	7.87E+04	7.87E+04	7.87E+04	NULL	AS	P55884-2	EIF3B
2259.1	Dimethyladenosine transferase	0.00E+00	7.93E+04	7.93E+04	7.93E+04	NULL	AS	Q9H5Q4	TFB2M
2381.1	Protein LOC100652824	0.00E+00	7.98E+04	7.98E+04	7.98E+04	NULL	AS	F5H626	LOC100652824
1709.1	Acidic leucine-rich nuclear phos	0.00E+00	7.99E+04	7.99E+04	7.99E+04	NULL	AS	O95626	ANP32D
2972.1	Putative ATP-dependent RNA he	0.00E+00	7.99E+04	7.99E+04	7.99E+04	NULL	AS	Q9H6R0	DHX33
2882.1	Beta-crystallin A3	0.00E+00	8.00E+04	8.00E+04	8.00E+04	NULL	AS	P05813	CRYBA1
2801.1	Laminin subunit alpha-4	0.00E+00	8.08E+04	8.08E+04	8.08E+04	NULL	AS	Q16363	LAMA4
1585.1	Zinc finger protein 616	0.00E+00	8.09E+04	8.09E+04	8.09E+04	NULL	AS	Q08AN1	ZNF616
1854.1	Lamina-associated polypeptide 2	0.00E+00	8.09E+04	8.09E+04	8.09E+04	NULL	AS	P42166	TMPO
2804.1	MICAL C-terminal-like protein	0.00E+00	8.11E+04	8.11E+04	8.11E+04	NULL	AS	Q6ZW33	MICALCL
1813.1	60S ribosomal protein L14	0.00E+00	8.15E+04	8.15E+04	8.15E+04	NULL	AS	P50914	RPL14
2374.1	TBC1 domain family member 26	0.00E+00	8.16E+04	8.16E+04	8.16E+04	NULL	AS	Q86UD7	TBC1D26
1104.1	HIV Tat-specific factor 1	0.00E+00	8.37E+04	8.37E+04	8.37E+04	NULL	AS	O43719	HTATSF1
2491.1	Unconventional myosin-XVIIIb	0.00E+00	8.39E+04	8.39E+04	8.39E+04	NULL	AS	F5GXR6	MYO18B
2270.1	Isoform 3 of Tyrosine-protein ki	0.00E+00	8.43E+04	8.43E+04	8.43E+04	NULL	AS	P06239-3	LCK
1886.1	Calcium-dependent secretion ac	0.00E+00	8.54E+04	8.54E+04	8.54E+04	NULL	AS	Q86UW7	CADPS2
1744.1	Bone morphogenetic protein 3	0.00E+00	8.55E+04	8.55E+04	8.55E+04	NULL	AS	P12645	BMP3

1960.1	Cleavage stimulation factor subı	0.00E+00	8.58E+04	8.58E+04	8.58E+04	NULL	AS	Q05048	CSTF1
2061.1	Isoform Mitochondrial of Lysine	0.00E+00	8.61E+04	8.61E+04	8.61E+04	NULL	AS	Q15046-2	KARS
2908.1	snRNA-activating protein compl	0.00E+00	8.61E+04	8.61E+04	8.61E+04	NULL	AS	O75971	SNAPC5
2486.1	Homeobox protein ARX	0.00E+00	8.62E+04	8.62E+04	8.62E+04	NULL	AS	Q96QS3	ARX
1695.1	AP2-associated protein kinase 1	0.00E+00	8.69E+04	8.69E+04	8.69E+04	NULL	AS	Q2M2I8	AAK1
1689.1	Dual specificity tyrosine-phosph	0.00E+00	8.78E+04	8.78E+04	8.78E+04	NULL	AS	Q13627	DYRK1A
2791.1	Protein FAM118A	0.00E+00	8.79E+04	8.79E+04	8.79E+04	NULL	AS	Q9NWS6	FAM118A
2728.1	Reelin	0.00E+00	8.85E+04	8.85E+04	8.85E+04	NULL	AS	J3KQ66	RELN
2022.1	72 kDa type IV collagenase	0.00E+00	8.87E+04	8.87E+04	8.87E+04	NULL	AS	P08253	MMP2
1609.1	Anamorsin	0.00E+00	8.91E+04	8.91E+04	8.91E+04	NULL	AS	Q6FI81	CIAPIN1
1471.1	WD repeat-containing protein 3	0.00E+00	8.96E+04	8.96E+04	8.96E+04	NULL	AS	Q8NI36	WDR36
2540.1	Nucleoside diphosphate-linked ı	0.00E+00	8.96E+04	8.96E+04	8.96E+04	NULL	AS	P0C025	NUDT17
2388.1	Ankyrin repeat domain-containi	0.00E+00	9.00E+04	9.00E+04	9.00E+04	NULL	AS	Q8IVF6	ANKRD18A
1798.1	Methionine aminopeptidase 2	0.00E+00	9.06E+04	9.06E+04	9.06E+04	NULL	AS	P50579	METAP2
3256.1	HCG1818442, isoform CRA_a	0.00E+00	9.06E+04	9.06E+04	9.06E+04	NULL	AS	I3L4Q0	FAM195B
2076.1	Protein fantom	0.00E+00	9.08E+04	9.08E+04	9.08E+04	NULL	AS	Q68CZ1	RPGRIP1L
2299.1	Coiled-coil domain-containing p	0.00E+00	9.29E+04	9.29E+04	9.29E+04	NULL	AS	Q2M329	CCDC96
2242.1	Protein Wnt	0.00E+00	9.32E+04	9.32E+04	9.32E+04	NULL	AS	D6RF47	WNT8A
2884.1	Alpha-1D adrenergic receptor	0.00E+00	9.32E+04	9.32E+04	9.32E+04	NULL	AS	P25100	ADRA1D
3139.1	Prostaglandin F2-alpha receptor	0.00E+00	9.32E+04	9.32E+04	9.32E+04	NULL	AS	P43088	PTGFR
1527.1	Wee1-like protein kinase 2	0.00E+00	9.36E+04	9.36E+04	9.36E+04	NULL	AS	P0C1S8	WEE2
1245.1	AP-2 complex subunit alpha-1	0.00E+00	9.40E+04	9.40E+04	9.40E+04	NULL	AS	O95782	AP2A1
1112.1	Plexin-A1	0.00E+00	9.41E+04	9.41E+04	9.41E+04	NULL	AS	Q9UIW2	PLXNA1
2471.1	Nucleolar and spindle-associate	0.00E+00	9.43E+04	9.43E+04	9.43E+04	NULL	AS	Q9BXS6	NUSAP1
2950.1	B-cell CLL/lymphoma 6 member	0.00E+00	9.48E+04	9.48E+04	9.48E+04	NULL	AS	Q8N143	BCL6B
1428.1	Isoform 4 of Nitrilase homolog 1	0.00E+00	9.52E+04	9.52E+04	9.52E+04	NULL	AS	Q86X76-3	NIT1
2315.1	Thiosulfate sulfurtransferase	0.00E+00	9.57E+04	9.57E+04	9.57E+04	NULL	AS	Q16762	TST
1555.1	Insulin receptor-related protein	0.00E+00	9.59E+04	9.59E+04	9.59E+04	NULL	AS	P14616	INSRR
2984.1	Ceramide synthase 3	0.00E+00	9.62E+04	9.62E+04	9.62E+04	NULL	AS	Q8IU89	CERS3
741.1	Prefoldin subunit 3	0.00E+00	9.64E+04	9.64E+04	9.64E+04	NULL	AS	P61758	VBP1
1204.1	Ubiquitin-conjugating enzyme E	0.00E+00	9.66E+04	9.66E+04	9.66E+04	NULL	AS	P49459	UBE2A
2033.1	Glycerol kinase	0.00E+00	9.69E+04	9.69E+04	9.69E+04	NULL	AS	F8WC39	GK
1436.1	Probable allantoicase	0.00E+00	9.75E+04	9.75E+04	9.75E+04	NULL	AS	Q8N6M5	ALLC
1084.1	Centrosomal protein POC5	0.00E+00	9.82E+04	9.82E+04	9.82E+04	NULL	AS	Q8NA72	POC5
1611.1	La-related protein 6	0.00E+00	9.83E+04	9.83E+04	9.83E+04	NULL	AS	Q9BRS8	LARP6

2206.1	Exonuclease 3'-5' domain-conta	0.00E+00	9.83E+04	9.83E+04	9.83E+04	NULL	AS	B7Z839	EXD1
3078.1	Macrophage erythroblast attach	0.00E+00	9.83E+04	9.83E+04	9.83E+04	NULL	AS	E7ESC7	MAEA
2408.1	Dimethylaniline monooxygenase	0.00E+00	9.93E+04	9.93E+04	9.93E+04	NULL	AS	B7Z3P4	FMO1
2198.1	Bifunctional methylenetetrahyd	0.00E+00	1.00E+05	1.00E+05	1.00E+05	NULL	AS	P13995	MTHFD2
2724.1	Growth arrest-specific protein 7	0.00E+00	1.01E+05	1.01E+05	1.01E+05	NULL	AS	O60861	GAS7
2839.1	Polycystic kidney disease protei	0.00E+00	1.01E+05	1.01E+05	1.01E+05	NULL	AS	Q8TDX9	PKD1L1
2915.1	Isoform 3 of Forkhead-associate	0.00E+00	1.01E+05	1.01E+05	1.01E+05	NULL	AS	B1AJZ9-3	FHAD1
3083.1	DENN domain-containing protei	0.00E+00	1.01E+05	1.01E+05	1.01E+05	NULL	AS	Q9ULE3	DENND2A
1123.1	Glutaryl-CoA dehydrogenase, m	0.00E+00	1.02E+05	1.02E+05	1.02E+05	NULL	AS	Q92947	GCDH
1819.1	SH3 and cysteine-rich domain-co	0.00E+00	1.02E+05	1.02E+05	1.02E+05	NULL	AS	Q96MF2	STAC3
2301.1	Kinesin-like protein KIF27	0.00E+00	1.02E+05	1.02E+05	1.02E+05	NULL	AS	Q86VH2	KIF27
3020.1	Isoform 5 of Afadin	0.00E+00	1.02E+05	1.02E+05	1.02E+05	NULL	AS	P55196-5	MLLT4
3069.1	Grancalcin	0.00E+00	1.03E+05	1.03E+05	1.03E+05	NULL	AS	P28676	GCA
1199.1	Isoform 2 of Splicing factor U2A	0.00E+00	1.04E+05	1.04E+05	1.04E+05	NULL	AS	Q01081-2	U2AF1
1568.1	V-type proton ATPase 116 kDa s	0.00E+00	1.05E+05	1.05E+05	1.05E+05	NULL	AS	Q9HBG4	ATP6V0A4
2810.1	Nucleolar protein of 40 kDa	0.00E+00	1.05E+05	1.05E+05	1.05E+05	NULL	AS	E7EPF0	ZCCHC17
2953.1	Arf-GAP with dual PH domain-co	0.00E+00	1.05E+05	1.05E+05	1.05E+05	NULL	AS	Q2V6Q1	CENTA2
996.1	Janus kinase and microtubule-in	0.00E+00	1.06E+05	1.06E+05	1.06E+05	NULL	AS	Q5VZ66	JAKMIP3
1421.1	C-C motif chemokine 5	0.00E+00	1.06E+05	1.06E+05	1.06E+05	NULL	AS	P13501	CCL5
2167.1	Dopamine beta-hydroxylase	0.00E+00	1.06E+05	1.06E+05	1.06E+05	NULL	AS	P09172	DBH
1188.1	Transmembrane protein 131	0.00E+00	1.07E+05	1.07E+05	1.07E+05	NULL	AS	Q92545	TMEM131
2488.1	Laminin subunit alpha-3	0.00E+00	1.07E+05	1.07E+05	1.07E+05	NULL	AS	Q16787	LAMA3
2596.1	Periostin	0.00E+00	1.07E+05	1.07E+05	1.07E+05	NULL	AS	Q15063	POSTN
717.1	Histone H1.5	0.00E+00	1.08E+05	1.08E+05	1.08E+05	NULL	AS	P16401	HIST1H1B
1189.1	T-cell surface protein tactile	0.00E+00	1.08E+05	1.08E+05	1.08E+05	NULL	AS	P40200	CD96
1614.1	Cancer/testis antigen 75	0.00E+00	1.08E+05	1.08E+05	1.08E+05	NULL	AS	Q6PK30	CT75
1622.1	Cytoplasmic polyadenylation ele	0.00E+00	1.08E+05	1.08E+05	1.08E+05	NULL	AS	Q17RY0	CPEB4
2038.1	Podoplanin	0.00E+00	1.08E+05	1.08E+05	1.08E+05	NULL	AS	D6RH07	PDPN
2093.1	Inositol polyphosphate-5-phosp	0.00E+00	1.08E+05	1.08E+05	1.08E+05	NULL	AS	B1ARF3	INPP5B
2775.1	Protein NipSnap homolog 2	0.00E+00	1.08E+05	1.08E+05	1.08E+05	NULL	AS	O75323	GBAS
2889.1	Zinc finger protein 608	0.00E+00	1.08E+05	1.08E+05	1.08E+05	NULL	AS	Q9ULD9	ZNF608
3153.1	Protein FAM83G	0.00E+00	1.08E+05	1.08E+05	1.08E+05	NULL	AS	A6ND36	FAM83G
1864.1	ATP-dependent DNA helicase Q!	0.00E+00	1.09E+05	1.09E+05	1.09E+05	NULL	AS	O94762	RECQL5
2333.1	Phakinin	0.00E+00	1.09E+05	1.09E+05	1.09E+05	NULL	AS	Q13515	BFSP2
3053.1	Thiosulfate sulfurtransferase/rh	0.00E+00	1.09E+05	1.09E+05	1.09E+05	NULL	AS	Q5T7W7	TSTD2

1224.1	HCF N-terminal chain 5	0.00E+00	1.10E+05	1.10E+05	1.10E+05	NULL	AS	A6NEM2	HCFC1
1629.1	F-box/LRR-repeat protein 14	0.00E+00	1.11E+05	1.11E+05	1.11E+05	NULL	AS	Q8N1E6	FBXL14
2446.1	Lipolysis-stimulated lipoprotein	0.00E+00	1.11E+05	1.11E+05	1.11E+05	NULL	AS	B4DKL4	LSR
2778.1	Probable global transcription ac	0.00E+00	1.11E+05	1.11E+05	1.11E+05	NULL	AS	P28370	SMARCA1
3201.1	Serpin B8	0.00E+00	1.11E+05	1.11E+05	1.11E+05	NULL	AS	P50452	SERPINB8
908.1	AP-2 complex subunit mu	0.00E+00	1.12E+05	1.12E+05	1.12E+05	NULL	AS	E9PFW3	AP2M1
1179.1	Isoform 3 of Serine/threonine-p	0.00E+00	1.12E+05	1.12E+05	1.12E+05	NULL	AS	Q96PY6-3	NEK1
1686.1	Exportin-6	0.00E+00	1.12E+05	1.12E+05	1.12E+05	NULL	AS	Q96QU8	XPO6
756.1	Coproporphyrinogen-III oxidase,	0.00E+00	1.13E+05	1.13E+05	1.13E+05	NULL	AS	P36551	CPOX
1895.1	Filamin A-interacting protein 1-l	0.00E+00	1.13E+05	1.13E+05	1.13E+05	NULL	AS	Q4L180	FILIP1L
1906.1	Sonic hedgehog protein	0.00E+00	1.13E+05	1.13E+05	1.13E+05	NULL	AS	Q15465	SHH
2401.1	Isoform 2 of Bromodomain-cont	0.00E+00	1.13E+05	1.13E+05	1.13E+05	NULL	AS	O95696-2	BRD1
1234.1	Prolow-density lipoprotein rece	0.00E+00	1.14E+05	1.14E+05	1.14E+05	NULL	AS	Q07954	LRP1
2649.1	Protein polybromo-1	0.00E+00	1.14E+05	1.14E+05	1.14E+05	NULL	AS	Q86U86	PBRM1
3.7	Keratin, type II cytoskeletal 1b	0.00E+00	1.16E+05	1.16E+05	1.16E+05	NULL	AS	Q7Z794	KRT77
787.1	Probable ATP-dependent RNA h	0.00E+00	1.16E+05	1.16E+05	1.16E+05	NULL	AS	Q9BUQ8	DDX23
3190.1	CAP-Gly domain-containing link	0.00E+00	1.16E+05	1.16E+05	1.16E+05	NULL	AS	P30622	CLIP1
1337.1	Ubiquitin carboxyl-terminal hyd	0.00E+00	1.17E+05	1.17E+05	1.17E+05	NULL	AS	Q70EL4	USP43
1448.1	Activated CDC42 kinase 1	0.00E+00	1.17E+05	1.17E+05	1.17E+05	NULL	AS	C9J1X3	TNK2
1498.1	Vesicle transport through intera	0.00E+00	1.17E+05	1.17E+05	1.17E+05	NULL	AS	Q9UEU0	VTI1B
2609.1	Protein kinase C theta type	0.00E+00	1.17E+05	1.17E+05	1.17E+05	NULL	AS	Q04759	PRKCQ
2806.1	Pleckstrin homology domain-co	0.00E+00	1.17E+05	1.17E+05	1.17E+05	NULL	AS	Q8IVE3	PLEKHH2
1518.1	Protein unc-13 homolog C	0.00E+00	1.18E+05	1.18E+05	1.18E+05	NULL	AS	F5H090	UNC13C
1780.1	Serine/threonine-protein kinase	0.00E+00	1.18E+05	1.18E+05	1.18E+05	NULL	AS	Q7L7X3	TAOK1
2390.1	VPS10 domain-containing recep	0.00E+00	1.18E+05	1.18E+05	1.18E+05	NULL	AS	Q96PQ0	SORCS2
2584.1	Isoform 2 of C-type lectin doma	0.00E+00	1.18E+05	1.18E+05	1.18E+05	NULL	AS	A5D8T8-2	CLEC18A
2251.1	Beta-ureidopropionase	0.00E+00	1.19E+05	1.19E+05	1.19E+05	NULL	AS	Q9UBR1	UPB1
1212.1	Isoform 2 of mRNA cap guanine-	0.00E+00	1.20E+05	1.20E+05	1.20E+05	NULL	AS	O43148-2	RNMT
2505.1	Histone acetyltransferase p300	0.00E+00	1.20E+05	1.20E+05	1.20E+05	NULL	AS	Q09472	EP300
2919.1	Pro-epidermal growth factor	0.00E+00	1.20E+05	1.20E+05	1.20E+05	NULL	AS	P01133	EGF
1194.1	Protein PML	0.00E+00	1.21E+05	1.21E+05	1.21E+05	NULL	AS	P29590	PML
2440.1	Isoform 2 of Plexin-A2	0.00E+00	1.21E+05	1.21E+05	1.21E+05	NULL	AS	O75051-2	PLXNA2
1425.1	Glycogen [starch] synthase, mu	0.00E+00	1.23E+05	1.23E+05	1.23E+05	NULL	AS	F5H1N8	GYS1
1717.1	Isoform 2 of Acid-sensing ion ch	0.00E+00	1.23E+05	1.23E+05	1.23E+05	NULL	AS	Q16515-2	ASIC2
3136.1	Tektin-5	0.00E+00	1.23E+05	1.23E+05	1.23E+05	NULL	AS	Q96M29	TEKT5

839.1	26S protease regulatory subunit	0.00E+00	1.24E+05	1.24E+05	1.24E+05	NULL	AS	P62191	PSMC1
944.1	Serine racemase	0.00E+00	1.24E+05	1.24E+05	1.24E+05	NULL	AS	Q9GZT4	SRR
1605.1	Ribosome-recycling factor, mito	0.00E+00	1.24E+05	1.24E+05	1.24E+05	NULL	AS	Q96E11	MRRF
1735.1	Limbic system-associated meml	0.00E+00	1.24E+05	1.24E+05	1.24E+05	NULL	AS	F5H5G1	LSAMP
2452.1	Isoform 3 of CD99 antigen-like p	0.00E+00	1.24E+05	1.24E+05	1.24E+05	NULL	AS	Q8TCZ2-3	CD99L2
3080.1	Protein transport protein Sec31	0.00E+00	1.24E+05	1.24E+05	1.24E+05	NULL	AS	D6RAB3	SEC31A
1372.1	Ubiquitin carboxyl-terminal hyd	0.00E+00	1.25E+05	1.25E+05	1.25E+05	NULL	AS	Q8NB14	USP38
1208.1	Glycerophosphocholine phosph	0.00E+00	1.26E+05	1.26E+05	1.26E+05	NULL	AS	Q9NPB8	GPCPD1
1902.1	Transcription factor 25	0.00E+00	1.27E+05	1.27E+05	1.27E+05	NULL	AS	Q9BQ70	TCF25
2310.1	Copine-9	0.00E+00	1.27E+05	1.27E+05	1.27E+05	NULL	AS	Q8IYJ1	CPNE9
2664.1	26S proteasome non-ATPase reg	0.00E+00	1.27E+05	1.27E+05	1.27E+05	NULL	AS	O00232	PSMD12
2887.1	Rho guanine nucleotide exchang	0.00E+00	1.27E+05	1.27E+05	1.27E+05	NULL	AS	Q12774	ARHGEF5
2932.1	Arginine and glutamate-rich pro	0.00E+00	1.27E+05	1.27E+05	1.27E+05	NULL	AS	Q9NWB6	ARGLU1
690.1	Ester hydrolase C11orf54	0.00E+00	1.28E+05	1.28E+05	1.28E+05	NULL	AS	Q9H0W9	C11orf54
1926.1	Isoform 2 of Zinc finger protein	0.00E+00	1.28E+05	1.28E+05	1.28E+05	NULL	AS	Q8NEA6-2	GLIS3
2574.1	Probable lipid phosphate phosp	0.00E+00	1.28E+05	1.28E+05	1.28E+05	NULL	AS	Q8NBV4	PPAPDC3
2079.1	RNA-binding E3 ubiquitin-protei	0.00E+00	1.29E+05	1.29E+05	1.29E+05	NULL	AS	Q5U5Q3	MEX3C
2418.1	Protein FAM184A	0.00E+00	1.29E+05	1.29E+05	1.29E+05	NULL	AS	Q8NB25	FAM184A
2433.1	Kelch-like protein 22	0.00E+00	1.29E+05	1.29E+05	1.29E+05	NULL	AS	Q53GT1	KLHL22
1464.1	Phenylalanine-4-hydroxylase	0.00E+00	1.30E+05	1.30E+05	1.30E+05	NULL	AS	P00439	PAH
1474.1	Small nuclear ribonucleoprotein	0.00E+00	1.30E+05	1.30E+05	1.30E+05	NULL	AS	P62318	SNRPD3
1538.1	PHD finger protein 10	0.00E+00	1.30E+05	1.30E+05	1.30E+05	NULL	AS	Q8WUB8	PHF10
2796.1	Microcephalin	0.00E+00	1.30E+05	1.30E+05	1.30E+05	NULL	AS	Q8NEM0	MCPH1
1726.1	Isoform 3 of FLYWCH-type zinc f	0.00E+00	1.31E+05	1.31E+05	1.31E+05	NULL	AS	Q4VC44-3	FLYWCH1
2256.1	Calcium-transporting ATPase typ	0.00E+00	1.31E+05	1.31E+05	1.31E+05	NULL	AS	E7ES94	ATP2C2
2545.1	Hydroxylysine kinase	0.00E+00	1.31E+05	1.31E+05	1.31E+05	NULL	AS	A2RU49	AGPHD1
2601.1	Dystrophin	0.00E+00	1.32E+05	1.32E+05	1.32E+05	NULL	AS	P11532	DMD
3137.1	Isoform 4 of DNA topoisomerase	0.00E+00	1.32E+05	1.32E+05	1.32E+05	NULL	AS	P11388-4	TOP2A
1118.1	Lysosomal alpha-glucosidase	0.00E+00	1.34E+05	1.34E+05	1.34E+05	NULL	AS	P10253	GAA
1972.1	Sodium channel protein type 9 s	0.00E+00	1.34E+05	1.34E+05	1.34E+05	NULL	AS	E9PBA5	SCN9A
736.1	RNA-binding protein PNO1	0.00E+00	1.35E+05	1.35E+05	1.35E+05	NULL	AS	Q9NRX1	PNO1
1664.1	Beta-taxilin	0.00E+00	1.35E+05	1.35E+05	1.35E+05	NULL	AS	Q8N3L3	TXLNB
2558.1	Malcavernin	0.00E+00	1.35E+05	1.35E+05	1.35E+05	NULL	AS	Q9BSQ5	CCM2
2567.1	Protein FAM133B	0.00E+00	1.35E+05	1.35E+05	1.35E+05	NULL	AS	Q5BKY9	FAM133B
2718.1	Alpha-(1,6)-fucosyltransferase	0.00E+00	1.35E+05	1.35E+05	1.35E+05	NULL	AS	Q9BYC5	FUT8

2634.1	Protein LOC642778	0.00E+00	1.36E+05	1.36E+05	1.36E+05	NULL	AS	E9PJ88	LOC642778
1153.1	Dedicator of cytokinesis protein	0.00E+00	1.37E+05	1.37E+05	1.37E+05	NULL	AS	A6NIW2	DOCK11
1739.1	DNA endonuclease RBBP8	0.00E+00	1.37E+05	1.37E+05	1.37E+05	NULL	AS	I6L8A6	RBBP8
1938.1	Catenin beta-1	0.00E+00	1.37E+05	1.37E+05	1.37E+05	NULL	AS	P35222	CTNNB1
2432.1	Zinc finger protein 398	0.00E+00	1.37E+05	1.37E+05	1.37E+05	NULL	AS	B4DXA9	ZNF398
2685.1	Alpha-mannosidase 2	0.00E+00	1.37E+05	1.37E+05	1.37E+05	NULL	AS	Q16706	MAN2A1
3219.1	Vasohibin-1	0.00E+00	1.37E+05	1.37E+05	1.37E+05	NULL	AS	G3V4N9	VASH1
2948.1	Protein FAM3D	0.00E+00	1.38E+05	1.38E+05	1.38E+05	NULL	AS	Q96BQ1	FAM3D
1352.1	Glycine cleavage system H prote	0.00E+00	1.39E+05	1.39E+05	1.39E+05	NULL	AS	P23434	GCSH
3066.1	WD repeat-containing protein K	0.00E+00	1.39E+05	1.39E+05	1.39E+05	NULL	AS	A6NE52	KIAA1875
1452.1	Cystic fibrosis transmembrane c	0.00E+00	1.40E+05	1.40E+05	1.40E+05	NULL	AS	P13569	CFTR
2219.1	ATP-dependent RNA helicase DI	0.00E+00	1.40E+05	1.40E+05	1.40E+05	NULL	AS	Q9NVP1	DDX18
2342.1	Olfactory receptor 1A2	0.00E+00	1.40E+05	1.40E+05	1.40E+05	NULL	AS	Q9Y585	OR1A2
1100.1	Ribonuclease P protein subunit	0.00E+00	1.40E+05	1.40E+05	1.40E+05	NULL	AS	P78346	RPP30
945.1	Zinc finger protein 709	0.00E+00	1.42E+05	1.42E+05	1.42E+05	NULL	AS	Q8N972	ZNF709
1492.1	Isoform 3 of Leucine-rich repeat	0.00E+00	1.42E+05	1.42E+05	1.42E+05	NULL	AS	Q8ND23-3	LRRC16B
1209.1	Isoform 2 of Ubiquitin carboxyl-	0.00E+00	1.44E+05	1.44E+05	1.44E+05	NULL	AS	Q14694-2	USP10
1344.1	Pyruvate dehydrogenase E1 con	0.00E+00	1.44E+05	1.44E+05	1.44E+05	NULL	AS	P11177	PDHB
2385.1	Arf-GAP with coiled-coil, ANK re	0.00E+00	1.44E+05	1.44E+05	1.44E+05	NULL	AS	Q15027	ACAP1
2807.1	Olfactory receptor 2C3	0.00E+00	1.44E+05	1.44E+05	1.44E+05	NULL	AS	Q8N628	OR2C3
1756.1	Isoform 9 of Multidrug resistanc	0.00E+00	1.45E+05	1.45E+05	1.45E+05	NULL	AS	P33527-9	ABCC1
2233.1	Alstrom syndrome protein 1	0.00E+00	1.45E+05	1.45E+05	1.45E+05	NULL	AS	Q8TCU4	ALMS1
2320.1	Isoform 3 of Granulocyte colony	0.00E+00	1.45E+05	1.45E+05	1.45E+05	NULL	AS	Q99062-3	CSF3R
2917.1	Serine/threonine-protein kinase	0.00E+00	1.45E+05	1.45E+05	1.45E+05	NULL	AS	D6RBG5	NEK1
2907.1	Geranylgeranyl transferase type	0.00E+00	1.46E+05	1.46E+05	1.46E+05	NULL	AS	Q92696	RABGGTA
1809.1	NEDD4-binding protein 2	0.00E+00	1.48E+05	1.48E+05	1.48E+05	NULL	AS	Q86UW6	N4BP2
1077.1	ATP-binding cassette sub-family	0.00E+00	1.49E+05	1.49E+05	1.49E+05	NULL	AS	C9JQE6	ABCA8
2662.1	Isoform 2 of Cactin	0.00E+00	1.49E+05	1.49E+05	1.49E+05	NULL	AS	Q8WUQ7-	CACTIN
1985.1	Acid trehalase-like protein 1	0.00E+00	1.50E+05	1.50E+05	1.50E+05	NULL	AS	E7EMA9	ATHL1
2194.1	Olfactory receptor 2M2	0.00E+00	1.51E+05	1.51E+05	1.51E+05	NULL	AS	Q96R28	OR2M2
2503.1	Ryanodine receptor 1	0.00E+00	1.52E+05	1.52E+05	1.52E+05	NULL	AS	P21817	RYR1
3218.1	Meiosis arrest female protein 1	0.00E+00	1.52E+05	1.52E+05	1.52E+05	NULL	AS	Q9Y4F3	KIAA0430
854.1	High mobility group nucleosome	0.00E+00	1.53E+05	1.53E+05	1.53E+05	NULL	AS	O00479	HMGN4
1386.1	Zinc finger protein 622	0.00E+00	1.53E+05	1.53E+05	1.53E+05	NULL	AS	Q969S3	ZNF622
1528.1	Serine incorporator 4	0.00E+00	1.53E+05	1.53E+05	1.53E+05	NULL	AS	A6NM42	SERINC4

1579.1	SEC14-like protein 4	0.00E+00	1.53E+05	1.53E+05	1.53E+05	NULL	AS	Q9UDX3	SEC14L4
2443.1	RNA-binding protein 20	0.00E+00	1.53E+05	1.53E+05	1.53E+05	NULL	AS	Q5T481	RBM20
1454.1	Potassium voltage-gated channe	0.00E+00	1.54E+05	1.54E+05	1.54E+05	NULL	AS	Q14721	KCNB1
1626.1	Ribosomal RNA processing prot	0.00E+00	1.54E+05	1.54E+05	1.54E+05	NULL	AS	Q14684	RRP1B
2069.1	Testosterone 17-beta-dehydrog	0.00E+00	1.54E+05	1.54E+05	1.54E+05	NULL	AS	P37058	HSD17B3
2570.1	snRNA-activating protein compl	0.00E+00	1.54E+05	1.54E+05	1.54E+05	NULL	AS	Q16533	SNAPC1
2593.1	Isoform 2 of Kynurenine/alpha-a	0.00E+00	1.54E+05	1.54E+05	1.54E+05	NULL	AS	Q8N5Z0-2	AADAT
3161.1	Retinoblastoma-like protein 2	0.00E+00	1.54E+05	1.54E+05	1.54E+05	NULL	AS	Q08999	RBL2
1196.1	Isoform 4 of Thiosulfate sulfurtr	0.00E+00	1.55E+05	1.55E+05	1.55E+05	NULL	AS	Q8NFU3-4	TSTD1
2297.1	Interferon-induced guanylate-bi	0.00E+00	1.55E+05	1.55E+05	1.55E+05	NULL	AS	P32455	GBP1
2672.1	Lipoyltransferase 1, mitochondr	0.00E+00	1.55E+05	1.55E+05	1.55E+05	NULL	AS	Q9Y234	LIPT1
2321.1	ATPase, Ca++ transporting, type	0.00E+00	1.55E+05	1.55E+05	1.55E+05	NULL	AS	P98194	ATP2C1
2175.1	E3 UFM1-protein ligase 1	0.00E+00	1.56E+05	1.56E+05	1.56E+05	NULL	AS	O94874	UFL1
2439.1	Glucagon-like peptide 2 recepto	0.00E+00	1.56E+05	1.56E+05	1.56E+05	NULL	AS	O95838	GLP2R
2666.1	Transcriptional repressor NF-X1	0.00E+00	1.56E+05	1.56E+05	1.56E+05	NULL	AS	Q12986	NFX1
632.1	CLIP-associating protein 2	0.00E+00	1.57E+05	1.57E+05	1.57E+05	NULL	AS	F5H3X0	CLASP2
1911.1	Cystatin-F	0.00E+00	1.57E+05	1.57E+05	1.57E+05	NULL	AS	O76096	CST7
1971.1	REST corepressor 2	0.00E+00	1.57E+05	1.57E+05	1.57E+05	NULL	AS	Q8IZ40	RCOR2
2293.1	Isoform 2 of Cytochrome P450 4	0.00E+00	1.57E+05	1.57E+05	1.57E+05	NULL	AS	P13584-2	CYP4B1
3115.1	Arf-GAP with GTPase, ANK repea	0.00E+00	1.57E+05	1.57E+05	1.57E+05	NULL	AS	E7EUN2	AGAP1
2633.1	Meckel syndrome type 1 protein	0.00E+00	1.58E+05	1.58E+05	1.58E+05	NULL	AS	Q9NXB0	MKS1
1113.1	Retinoblastoma-like protein 1	0.00E+00	1.59E+05	1.59E+05	1.59E+05	NULL	AS	P28749	RBL1
2926.1	Glutamate--cysteine ligase regu	0.00E+00	1.60E+05	1.60E+05	1.60E+05	NULL	AS	P48507	GCLM
3045.1	DNA polymerase beta	0.00E+00	1.60E+05	1.60E+05	1.60E+05	NULL	AS	P06746	POLB
1957.1	Low-density lipoprotein recepto	0.00E+00	1.61E+05	1.61E+05	1.61E+05	NULL	AS	H0YMD1	LDLR
2084.1	Serine/arginine-rich splicing fact	0.00E+00	1.61E+05	1.61E+05	1.61E+05	NULL	AS	O75494	SRSF10
2996.1	NADH dehydrogenase [ubiquinc	0.00E+00	1.61E+05	1.61E+05	1.61E+05	NULL	AS	O43678	NDUFA2
2997.1	N-terminal kinase-like protein	0.00E+00	1.61E+05	1.61E+05	1.61E+05	NULL	AS	Q96KG9	SCYL1
2055.1	Retinol dehydrogenase 14	0.00E+00	1.62E+05	1.62E+05	1.62E+05	NULL	AS	Q9HBH5	RDH14
1707.1	Isoform 4 of Putative ribosomal	0.00E+00	1.63E+05	1.63E+05	1.63E+05	NULL	AS	P46087-4	NOP2
1769.1	DNA-binding protein A	0.00E+00	1.63E+05	1.63E+05	1.63E+05	NULL	AS	P16989	YBX3
2699.1	Major vault protein	0.00E+00	1.63E+05	1.63E+05	1.63E+05	NULL	AS	Q14764	MVP
2262.1	Homeobox protein goosecoid	0.00E+00	1.64E+05	1.64E+05	1.64E+05	NULL	AS	P56915	GSC
3041.1	HCG2008157	0.00E+00	1.64E+05	1.64E+05	1.64E+05	NULL	AS	Q96NP5	hCG_2008157
1844.1	ZNF507 protein	0.00E+00	1.65E+05	1.65E+05	1.65E+05	NULL	AS	B9EGE7	ZNF507

2170.1	IQ calmodulin-binding motif-cor	0.00E+00	1.65E+05	1.65E+05	1.65E+05	NULL	AS	Q15051	IQCB1
2334.1	HCG1780554	0.00E+00	1.65E+05	1.65E+05	1.65E+05	NULL	AS	Q5T4B6	TMSB4XP4
1678.1	Isoform 3 of MAM domain-cont	0.00E+00	1.66E+05	1.66E+05	1.66E+05	NULL	AS	Q7Z553-3	MDGA2
1998.1	Oxysterol-binding protein	0.00E+00	1.66E+05	1.66E+05	1.66E+05	NULL	AS	J3KPA3	OSBPL9
2836.1	Inositol-trisphosphate 3-kinase I	0.00E+00	1.66E+05	1.66E+05	1.66E+05	NULL	AS	P27987	ITPKB
3203.1	Protein diaphanous homolog 3	0.00E+00	1.66E+05	1.66E+05	1.66E+05	NULL	AS	Q9NSV4	DIAPH3
1564.1	Carnitine O-palmitoyltransferas	0.00E+00	1.67E+05	1.67E+05	1.67E+05	NULL	AS	P23786	CPT2
1954.1	Phospholipase A-2-activating pr	0.00E+00	1.67E+05	1.67E+05	1.67E+05	NULL	AS	Q9Y263	PLAA
2423.1	Protein FAM71A	0.00E+00	1.68E+05	1.68E+05	1.68E+05	NULL	AS	Q8IYT1	FAM71A
1117.1	DnaJ homolog subfamily B mem	0.00E+00	1.69E+05	1.69E+05	1.69E+05	NULL	AS	P25685	DNAJB1
1508.1	DnaJ homolog subfamily C mem	0.00E+00	1.69E+05	1.69E+05	1.69E+05	NULL	AS	Q13217	DNAJC3
1704.1	Isoform 7 of Ras-specific guanin	0.00E+00	1.69E+05	1.69E+05	1.69E+05	NULL	AS	Q5JS13-7	RALGPS1
1328.1	Extracellular matrix protein FRA	0.00E+00	1.69E+05	1.69E+05	1.69E+05	NULL	AS	Q86XX4	FRAS1
2490.1	Cell division cycle-associated pr	0.00E+00	1.70E+05	1.70E+05	1.70E+05	NULL	AS	Q99618	CDCA3
2483.1	Isoform 6 of Tyrosine-protein pl	0.00E+00	1.71E+05	1.71E+05	1.71E+05	NULL	AS	Q4JDL3-6	PTPN20A
1243.1	Isoform 2 of Merlin	0.00E+00	1.72E+05	1.72E+05	1.72E+05	NULL	AS	P35240-2	NF2
1868.1	GRAM domain-containing prote	0.00E+00	1.72E+05	1.72E+05	1.72E+05	NULL	AS	Q8IYS0	GRAMD1C
1244.1	Phosphorylase b kinase regulatc	0.00E+00	1.73E+05	1.73E+05	1.73E+05	NULL	AS	P46019	PHKA2
1392.1	Carboxypeptidase E	0.00E+00	1.74E+05	1.74E+05	1.74E+05	NULL	AS	P16870	CPE
799.1	L-xylulose reductase	0.00E+00	1.75E+05	1.75E+05	1.75E+05	NULL	AS	Q7Z4W1	DCXR
1882.1	REST corepressor 3	0.00E+00	1.76E+05	1.76E+05	1.76E+05	NULL	AS	B4DV59	RCOR3
2204.1	28S ribosomal protein S18b, mit	0.00E+00	1.76E+05	1.76E+05	1.76E+05	NULL	AS	Q9Y676	MRPS18B
2683.1	WD repeat-containing protein 2	0.00E+00	1.76E+05	1.76E+05	1.76E+05	NULL	AS	Q64LD2	WDR25
657.1	Isoform 4 of PCI domain-contair	0.00E+00	1.77E+05	1.77E+05	1.77E+05	NULL	AS	Q5JVF3-4	PCID2
2536.1	Prefoldin subunit 4	0.00E+00	1.79E+05	1.79E+05	1.79E+05	NULL	AS	E9PQY2	PFDN4
1561.1	Pregnancy zone protein	0.00E+00	1.80E+05	1.80E+05	1.80E+05	NULL	AS	P20742	PZP
2720.1	Syntaxin-4	0.00E+00	1.80E+05	1.80E+05	1.80E+05	NULL	AS	Q12846	STX4
1109.1	Receptor-type tyrosine-protein	0.00E+00	1.81E+05	1.81E+05	1.81E+05	NULL	AS	Q15256	PTPRR
2936.1	ATP-binding cassette sub-family	0.00E+00	1.81E+05	1.81E+05	1.81E+05	NULL	AS	E7ETD3	ABCA5
2444.1	ADAMTS-like 2	0.00E+00	1.82E+05	1.82E+05	1.82E+05	NULL	AS	B1B0D4	ADAMTSL2
1682.1	Leucine-rich repeat-containing p	0.00E+00	1.84E+05	1.84E+05	1.84E+05	NULL	AS	Q8IWT6	LRRC8A
1846.1	Protein EFR3 homolog B	0.00E+00	1.84E+05	1.84E+05	1.84E+05	NULL	AS	Q9Y2G0	EFR3B
2068.1	Liprin-alpha-3	0.00E+00	1.84E+05	1.84E+05	1.84E+05	NULL	AS	O75145	PPFIA3
674.1	Protein NipSnap homolog 3A	0.00E+00	1.85E+05	1.85E+05	1.85E+05	NULL	AS	Q9UFN0	NIPSNAP3A
3172.1	Isoform 5 of Serine/threonine-p	0.00E+00	1.85E+05	1.85E+05	1.85E+05	NULL	AS	Q8IWQ3-5	BRSK2

1505.1	Cyclin-dependent kinase 5	0.00E+00	1.86E+05	1.86E+05	1.86E+05	NULL	AS	Q00535	CDK5
2604.1	GC-rich sequence DNA-binding f	0.00E+00	1.86E+05	1.86E+05	1.86E+05	NULL	AS	P16383	GCFC2
2031.1	Isocitrate dehydrogenase [NAD]	0.00E+00	1.87E+05	1.87E+05	1.87E+05	NULL	AS	P50213	IDH3A
2589.1	Zinc finger matrin-type protein :	0.00E+00	1.87E+05	1.87E+05	1.87E+05	NULL	AS	Q5H9K5	ZMAT1
2945.1	Isoform 2 of SWI/SNF-related m	0.00E+00	1.87E+05	1.87E+05	1.87E+05	NULL	AS	Q9H4L7-2	SMARCAD1
1322.1	Elongator complex protein 3	0.00E+00	1.88E+05	1.88E+05	1.88E+05	NULL	AS	Q9H9T3	ELP3
1406.1	A disintegrin and metalloprotei	0.00E+00	1.90E+05	1.90E+05	1.90E+05	NULL	AS	O75173	ADAMTS4
3052.1	Zinc finger protein 672	0.00E+00	1.90E+05	1.90E+05	1.90E+05	NULL	AS	Q499Z4	ZNF672
2143.1	Isoform 3 of Polypyrimidine trac	0.00E+00	1.91E+05	1.91E+05	1.91E+05	NULL	AS	P26599-3	PTBP1
2902.1	RCC1 domain-containing protei	0.00E+00	1.91E+05	1.91E+05	1.91E+05	NULL	AS	A6NED2	RCCD1
1396.1	Fanconi anemia group M protei	0.00E+00	1.92E+05	1.92E+05	1.92E+05	NULL	AS	Q8IYD8	FANCM
1566.1	E3 SUMO-protein ligase CBX4	0.00E+00	1.94E+05	1.94E+05	1.94E+05	NULL	AS	O00257	CBX4
2607.1	Isoform 3 of Trinucleotide repea	0.00E+00	1.96E+05	1.96E+05	1.96E+05	NULL	AS	Q8NDV7-3	TNRC6A
3151.1	Spindle and kinetochore-associa	0.00E+00	1.96E+05	1.96E+05	1.96E+05	NULL	AS	Q8IX90	SKA3
767.1	Ribosome-binding protein 1	0.00E+00	1.97E+05	1.97E+05	1.97E+05	NULL	AS	Q9P2E9	RRBP1
1951.1	Ig-like V-type domain-containing	0.00E+00	1.97E+05	1.97E+05	1.97E+05	NULL	AS	A6NFU0	FAM187A
2021.1	Calcyphosin-2	0.00E+00	1.97E+05	1.97E+05	1.97E+05	NULL	AS	B9A061	CAPS2
2569.1	Isoform 13 of Dysferlin	0.00E+00	1.97E+05	1.97E+05	1.97E+05	NULL	AS	O75923-13	DYSF
1205.1	COP9 signalosome complex sub	0.00E+00	1.99E+05	1.99E+05	1.99E+05	NULL	AS	Q7L5N1	COPS6
1800.1	Growth factor receptor-bound p	0.00E+00	1.99E+05	1.99E+05	1.99E+05	NULL	AS	Q13322	GRB10
1066.1	Cyclin-I	0.00E+00	2.00E+05	2.00E+05	2.00E+05	NULL	AS	Q14094	CCNI
1832.1	NADH dehydrogenase (ubiquinc	0.00E+00	2.00E+05	2.00E+05	2.00E+05	NULL	AS	B4DW83	NDUFAF6
3058.1	Isoform 7 of Histone deacetylas	0.00E+00	2.00E+05	2.00E+05	2.00E+05	NULL	AS	Q9UKV0-7	HDAC9
2063.1	Mitochondrial calcium uniporte	0.00E+00	2.01E+05	2.01E+05	2.01E+05	NULL	AS	Q96AQ8	MCUR1
3121.1	GTPase IMAP family member 6	0.00E+00	2.01E+05	2.01E+05	2.01E+05	NULL	AS	Q6P9H5	GIMAP6
2088.1	Sterile alpha motif domain-cont	0.00E+00	2.02E+05	2.02E+05	2.02E+05	NULL	AS	Q8IVG5	SAMD9L
2197.1	Putative short-chain dehydroge	0.00E+00	2.02E+05	2.02E+05	2.02E+05	NULL	AS	A6NKP2	SDR42E2
2715.1	POU domain, class 4, transcripti	0.00E+00	2.02E+05	2.02E+05	2.02E+05	NULL	AS	Q01851	POU4F1
3026.1	Tripartite motif-containing prot	0.00E+00	2.03E+05	2.03E+05	2.03E+05	NULL	AS	Q14142	TRIM14
847.1	Lupus La protein	0.00E+00	2.04E+05	2.04E+05	2.04E+05	NULL	AS	P05455	SSB
1017.1	39S ribosomal protein L37, mito	0.00E+00	2.04E+05	2.04E+05	2.04E+05	NULL	AS	Q9BZE1	MRPL37
2891.1	Lysosomal alpha-mannosidase	0.00E+00	2.04E+05	2.04E+05	2.04E+05	NULL	AS	O00754	MAN2B1
1183.1	Forkhead box protein C2	0.00E+00	2.05E+05	2.05E+05	2.05E+05	NULL	AS	Q99958	FOXC2
1597.1	Transcription elongation factor :	0.00E+00	2.05E+05	2.05E+05	2.05E+05	NULL	AS	Q7KZ85	SUPT6H
2927.1	Isoform 4 of RNA-binding protei	0.00E+00	2.06E+05	2.06E+05	2.06E+05	NULL	AS	P49756-4	RBM25

1956.1	Neural cell expressed, developm	0.00E+00	2.07E+05	2.07E+05	2.07E+05	NULL	AS	G3V3F1	NEDD1
2564.1	SH3 and multiple ankyrin repeat	0.00E+00	2.07E+05	2.07E+05	2.07E+05	NULL	AS	A6NHU9	SHANK2
1336.1	Golgin subfamily B member 1	0.00E+00	2.08E+05	2.08E+05	2.08E+05	NULL	AS	Q14789	GOLGB1
1167.1	TP53-regulating kinase	0.00E+00	2.09E+05	2.09E+05	2.09E+05	NULL	AS	Q96S44	TP53RK
3072.1	TATA-binding protein-associatec	0.00E+00	2.09E+05	2.09E+05	2.09E+05	NULL	AS	O14981	BTAF1
1374.1	Protein monoglycylase TTLL8	0.00E+00	2.10E+05	2.10E+05	2.10E+05	NULL	AS	A6PVC2	TTLL8
2686.1	Inactive N-acetylated-alpha-link	0.00E+00	2.10E+05	2.10E+05	2.10E+05	NULL	AS	Q58DX5	NAALADL2
2875.1	Alpha-N-acetylgalactosaminide .	0.00E+00	2.11E+05	2.11E+05	2.11E+05	NULL	AS	Q9NSC7	ST6GALNAC1
1052.1	HESB like domain containing 2, i	0.00E+00	2.12E+05	2.12E+05	2.12E+05	NULL	AS	B4DJI5	ISCA1
2492.1	Myosin light chain kinase 3	0.00E+00	2.12E+05	2.12E+05	2.12E+05	NULL	AS	Q32MK0	MYLK3
2470.1	Rho GTPase-activating protein 6	0.00E+00	2.14E+05	2.14E+05	2.14E+05	NULL	AS	O43182	ARHGAP6
3163.1	Putative FAM120A opposite stra	0.00E+00	2.14E+05	2.14E+05	2.14E+05	NULL	AS	Q5T036	FAM120AOS
1514.1	Butyrophilin subfamily 3 membe	0.00E+00	2.16E+05	2.16E+05	2.16E+05	NULL	AS	O00478	BTN3A3
813.1	Serine/arginine-rich splicing fact	0.00E+00	2.18E+05	2.18E+05	2.18E+05	NULL	AS	Q16629	SRSF7
2549.1	Cannabinoid receptor 2	0.00E+00	2.18E+05	2.18E+05	2.18E+05	NULL	AS	P34972	CNR2
1748.1	Nucleolar complex protein 4 hor	0.00E+00	2.19E+05	2.19E+05	2.19E+05	NULL	AS	Q9BVI4	NOC4L
1804.1	tRNA selenocysteine 1-associate	0.00E+00	2.19E+05	2.19E+05	2.19E+05	NULL	AS	Q9NX07	TRNAU1AP
684.1	Protein ABHD14A-ACY1	0.00E+00	2.21E+05	2.21E+05	2.21E+05	NULL	AS	C9JMV9	ABHD14A-ACY1
737.1	Kinesin-associated protein 3	0.00E+00	2.21E+05	2.21E+05	2.21E+05	NULL	AS	Q92845	KIFAP3
1101.1	Isoform 6 of Spermine oxidase	0.00E+00	2.21E+05	2.21E+05	2.21E+05	NULL	AS	Q9NWM0-	SMOX
2247.1	Centriolin	0.00E+00	2.21E+05	2.21E+05	2.21E+05	NULL	AS	Q7Z7A1	CNTRL
3099.1	Zinc finger protein 425	0.00E+00	2.21E+05	2.21E+05	2.21E+05	NULL	AS	Q6IV72	ZNF425
8.4	Heat shock 70 kDa protein 1A/1	0.00E+00	2.23E+05	2.23E+05	2.23E+05	NULL	AS	E7EP94	HSPA1A
648.1	N-alpha-acetyltransferase 10	0.00E+00	2.23E+05	2.23E+05	2.23E+05	NULL	AS	P41227	NAA10
814.1	Muscleblind-like protein 1	0.00E+00	2.23E+05	2.23E+05	2.23E+05	NULL	AS	Q9NR56	MBNL1
2448.1	Enhancer of rudimentary homol	0.00E+00	2.24E+05	2.24E+05	2.24E+05	NULL	AS	P84090	ERH
2393.1	SH3 and PX domain-containing p	0.00E+00	2.28E+05	2.28E+05	2.28E+05	NULL	AS	Q5TCZ1	SH3PXD2A
3171.1	Plexin-D1	0.00E+00	2.28E+05	2.28E+05	2.28E+05	NULL	AS	Q9Y4D7	PLXND1
3226.1	Matrix metalloproteinase-20	0.00E+00	2.28E+05	2.28E+05	2.28E+05	NULL	AS	O60882	MMP20
1006.1	Nucleolar protein 58	0.00E+00	2.29E+05	2.29E+05	2.29E+05	NULL	AS	Q9Y2X3	NOP58
1468.1	Mitogen-activated protein kinas	0.00E+00	2.29E+05	2.29E+05	2.29E+05	NULL	AS	Q8IVH8	MAP4K3
2294.1	Ribonuclease H2 subunit C	0.00E+00	2.29E+05	2.29E+05	2.29E+05	NULL	AS	E9PN81	RNASEH2C
1604.1	ATP-dependent RNA helicase DH	0.00E+00	2.30E+05	2.30E+05	2.30E+05	NULL	AS	Q14562	DHX8
1887.1	Dynein heavy chain domain-con	0.00E+00	2.30E+05	2.30E+05	2.30E+05	NULL	AS	Q96M86	DNHD1
2030.1	Surfeit 4, isoform CRA_c	0.00E+00	2.30E+05	2.30E+05	2.30E+05	NULL	AS	B7Z6A4	SURF4

2137.1	RNA-binding protein 5	0.00E+00	2.30E+05	2.30E+05	2.30E+05	NULL	AS	P52756	RBM5
563.1	Nuclear inhibitor of protein pho	0.00E+00	2.31E+05	2.31E+05	2.31E+05	NULL	AS	Q12972	PPP1R8
1627.1	Protein FAM188B2	0.00E+00	2.33E+05	2.33E+05	2.33E+05	NULL	AS	A8MYZ0	FAM188B2
1512.1	Headcase protein homolog	0.00E+00	2.34E+05	2.34E+05	2.34E+05	NULL	AS	Q9UBI9	HECA
2273.1	M-phase phosphoprotein 9	0.00E+00	2.34E+05	2.34E+05	2.34E+05	NULL	AS	Q99550	MPHOSPH9
2077.1	Centrosomal protein of 290 kDa	0.00E+00	2.35E+05	2.35E+05	2.35E+05	NULL	AS	J3KNF5	CEP290
2195.1	Kinesin-like protein KIF12	0.00E+00	2.35E+05	2.35E+05	2.35E+05	NULL	AS	Q96FN5	KIF12
2511.1	E3 ubiquitin-protein ligase pellir	0.00E+00	2.35E+05	2.35E+05	2.35E+05	NULL	AS	Q96FA3	PELI1
1018.1	Dpy-30-like protein, isoform CR/	0.00E+00	2.36E+05	2.36E+05	2.36E+05	NULL	AS	B4DIS3	LOC84661
1510.1	Ataxin-2	0.00E+00	2.36E+05	2.36E+05	2.36E+05	NULL	AS	Q99700	ATXN2
2999.1	Isoform 4 of Ankyrin repeat and	0.00E+00	2.36E+05	2.36E+05	2.36E+05	NULL	AS	Q9P2R3-4	ANKFY1
2403.1	Rho GTPase-activating protein 2	0.00E+00	2.37E+05	2.37E+05	2.37E+05	NULL	AS	Q5T5U3	ARHGAP21
595.1	Phosphatidylinositol-binding cla	0.00E+00	2.39E+05	2.39E+05	2.39E+05	NULL	AS	Q13492	PICALM
802.1	Lactotransferrin	0.00E+00	2.39E+05	2.39E+05	2.39E+05	NULL	AS	P02788	LTF
2965.1	Pecanex-like protein 1	0.00E+00	2.39E+05	2.39E+05	2.39E+05	NULL	AS	Q96RV3	PCNX
1371.1	WD repeat-containing and plan	0.00E+00	2.41E+05	2.41E+05	2.41E+05	NULL	AS	O95876	WDPCP
2258.1	Ribonuclease P protein subunit	0.00E+00	2.41E+05	2.41E+05	2.41E+05	NULL	AS	Q8N5L8	RPP25L
2694.1	Transient receptor potential cat	0.00E+00	2.42E+05	2.42E+05	2.42E+05	NULL	AS	E9PGK7	TRPM2
3267.1	GPI transamidase component PI	0.00E+00	2.42E+05	2.42E+05	2.42E+05	NULL	AS	Q969N2	PIGT
1042.1	Ribosome-releasing factor 2, mi	0.00E+00	2.43E+05	2.43E+05	2.43E+05	NULL	AS	Q969S9	GFM2
2769.1	Isoform 3 of Ran-binding protei	0.00E+00	2.43E+05	2.43E+05	2.43E+05	NULL	AS	Q86VV4-3	RANBP3L
1592.1	Isoform 3 of Acyl-coenzyme A sy	0.00E+00	2.44E+05	2.44E+05	2.44E+05	NULL	AS	Q6P461-3	ACSM6
1435.1	60S ribosomal protein L18a	0.00E+00	2.45E+05	2.45E+05	2.45E+05	NULL	AS	Q02543	RPL18A
2003.1	Disabled homolog 1	0.00E+00	2.45E+05	2.45E+05	2.45E+05	NULL	AS	O75553	DAB1
3103.1	Unconventional myosin-IXa	0.00E+00	2.45E+05	2.45E+05	2.45E+05	NULL	AS	H3BMM1	MYO9A
420.2	Long-chain-fatty-acid--CoA ligas	0.00E+00	2.46E+05	2.46E+05	2.46E+05	NULL	AS	D6RER0	ACSL1
2539.1	ADP-ribose pyrophosphatase, m	0.00E+00	2.46E+05	2.46E+05	2.46E+05	NULL	AS	Q9BW91	NUDT9
3051.1	Collagen alpha-1(VIII) chain	0.00E+00	2.47E+05	2.47E+05	2.47E+05	NULL	AS	P27658	COL8A1
3007.1	Signal transducer and activator	0.00E+00	2.48E+05	2.48E+05	2.48E+05	NULL	AS	P52630	STAT2
3229.1	Tetratricopeptide repeat protei	0.00E+00	2.50E+05	2.50E+05	2.50E+05	NULL	AS	Q8N584	TTC39C
2078.1	LIM/homeobox protein Lhx4	0.00E+00	2.51E+05	2.51E+05	2.51E+05	NULL	AS	Q969G2	LHX4
2453.1	Coenzyme Q-binding protein CC	0.00E+00	2.51E+05	2.51E+05	2.51E+05	NULL	AS	Q96MF6	COQ10A
781.1	Isoform 2 of Zinc finger and SCA	0.00E+00	2.52E+05	2.52E+05	2.52E+05	NULL	AS	Q8TBC5-2	ZSCAN18
2231.1	Protein GDF5OS, mitochondrial	0.00E+00	2.52E+05	2.52E+05	2.52E+05	NULL	AS	Q5U4N7	GDF5OS
2340.1	Isoform L' of Protein patched hc	0.00E+00	2.52E+05	2.52E+05	2.52E+05	NULL	AS	Q13635-2	PTCH1

940.1	Calcium homeostasis endoplasn	0.00E+00	2.53E+05	2.53E+05	2.53E+05	NULL	AS	J3QK89	CHERP
1115.1	1-phosphatidylinositol 4,5-bisph	0.00E+00	2.53E+05	2.53E+05	2.53E+05	NULL	AS	Q9P212	PLCE1
1240.1	FERM domain-containing protei	0.00E+00	2.53E+05	2.53E+05	2.53E+05	NULL	AS	A2A2Y4	FRMD3
1274.1	Isoform 4 of Sterol regulatory el	0.00E+00	2.53E+05	2.53E+05	2.53E+05	NULL	AS	P36956-4	SREBF1
1983.1	Melanoma-associated antigen E	0.00E+00	2.53E+05	2.53E+05	2.53E+05	NULL	AS	Q9HCI5	MAGEE1
2228.1	DNA-directed RNA polymerase I	0.00E+00	2.53E+05	2.53E+05	2.53E+05	NULL	AS	P05423	POLR3D
2230.1	Beta-nerve growth factor	0.00E+00	2.53E+05	2.53E+05	2.53E+05	NULL	AS	P01138	NGF
1310.1	Insulin-like growth factor 2 mRN	0.00E+00	2.54E+05	2.54E+05	2.54E+05	NULL	AS	F8W930	IGF2BP2
1779.1	Contactin-2	0.00E+00	2.54E+05	2.54E+05	2.54E+05	NULL	AS	Q02246	CNTN2
140.2	Rab GDP dissociation inhibitor a	0.00E+00	2.55E+05	2.55E+05	2.55E+05	NULL	AS	P31150	GDI1
1577.1	Neuroligin-2	0.00E+00	2.55E+05	2.55E+05	2.55E+05	NULL	AS	Q8NFZ4	NLGN2
2544.1	Interleukin-12 receptor subunit	0.00E+00	2.55E+05	2.55E+05	2.55E+05	NULL	AS	Q99665	IL12RB2
3010.1	Cyclin-dependent kinase-like 4	0.00E+00	2.55E+05	2.55E+05	2.55E+05	NULL	AS	Q2NME9	CDKL4
2774.1	Nucleolar GTP-binding protein 1	0.00E+00	2.56E+05	2.56E+05	2.56E+05	NULL	AS	Q9BZE4	GTPBP4
758.1	Myelin expression factor 2	0.00E+00	2.57E+05	2.57E+05	2.57E+05	NULL	AS	Q9P2K5	MYEF2
806.1	Nuclear pore complex protein N	0.00E+00	2.57E+05	2.57E+05	2.57E+05	NULL	AS	P57740	NUP107
1630.1	Beta-crystallin B2	0.00E+00	2.57E+05	2.57E+05	2.57E+05	NULL	AS	P43320	CRYBB2
1266.1	Unconventional myosin-Vc	0.00E+00	2.58E+05	2.58E+05	2.58E+05	NULL	AS	Q9NQX4	MYO5C
3082.1	Proprotein convertase subtilisin	0.00E+00	2.58E+05	2.58E+05	2.58E+05	NULL	AS	Q8NBP7	PCSK9
3032.1	Putative olfactory receptor 4A8	0.00E+00	2.60E+05	2.60E+05	2.60E+05	NULL	AS	P0C604	OR4A8P
3144.1	Multidrug resistance protein 3	0.00E+00	2.60E+05	2.60E+05	2.60E+05	NULL	AS	P21439	ABCB4
1866.1	Protein spire homolog 2	0.00E+00	2.62E+05	2.62E+05	2.62E+05	NULL	AS	Q8WWL2	SPIRE2
3029.1	BAH and coiled-coil domain-con	0.00E+00	2.62E+05	2.62E+05	2.62E+05	NULL	AS	F8WBW8	BAHCC1
901.1	ADP-ribosylation factor-like prot	0.00E+00	2.64E+05	2.64E+05	2.64E+05	NULL	AS	Q96BM9	ARL8A
1184.1	Dipeptidyl peptidase 2	0.00E+00	2.64E+05	2.64E+05	2.64E+05	NULL	AS	Q9UHL4	DPP7
1999.1	DmX-like protein 1	0.00E+00	2.65E+05	2.65E+05	2.65E+05	NULL	AS	F5H269	DMXL1
2621.1	Isoform 2 of Neuronal tyrosine-|	0.00E+00	2.66E+05	2.66E+05	2.66E+05	NULL	AS	Q6ZVC0-2	NYAP1
2913.1	ATP-binding cassette sub-family	0.00E+00	2.66E+05	2.66E+05	2.66E+05	NULL	AS	Q86UK0	ABCA12
2116.1	Isoform MOP3 of Aryl hydrocark	0.00E+00	2.67E+05	2.67E+05	2.67E+05	NULL	AS	O00327-7	ARNTL
3233.1	Clarin-2	0.00E+00	2.68E+05	2.68E+05	2.68E+05	NULL	AS	A0PK11	CLRN2
2632.1	Disheveled-associated activator	0.00E+00	2.71E+05	2.71E+05	2.71E+05	NULL	AS	Q9Y4D1	DAAM1
3059.1	Isoform 4 of Tensin-like C1 dom	0.00E+00	2.71E+05	2.71E+05	2.71E+05	NULL	AS	Q63HR2-4	TENC1
3213.1	Collagen alpha-1(XXV) chain	0.00E+00	2.71E+05	2.71E+05	2.71E+05	NULL	AS	Q9BXS0	COL25A1
2181.1	Leucine-rich repeat and fibrone	0.00E+00	2.72E+05	2.72E+05	2.72E+05	NULL	AS	Q9ULH4	LRFN2
3148.1	RNA-binding protein 28	0.00E+00	2.72E+05	2.72E+05	2.72E+05	NULL	AS	Q9NW13	RBM28

1150.1	Mannosyl-oligosaccharide 1,2-a	0.00E+00	2.73E+05	2.73E+05	2.73E+05	NULL	AS	P33908	MAN1A1
2087.1	Prolyl 4-hydroxylase subunit alp	0.00E+00	2.73E+05	2.73E+05	2.73E+05	NULL	AS	P13674	P4HA1
549.1	Isoform 2 of Catenin alpha-1	0.00E+00	2.74E+05	2.74E+05	2.74E+05	NULL	AS	P35221-2	CTNNA1
2323.1	Cell division cycle protein 123 h	0.00E+00	2.74E+05	2.74E+05	2.74E+05	NULL	AS	O75794	CDC123
1376.1	Chromosome transmission fidel	0.00E+00	2.75E+05	2.75E+05	2.75E+05	NULL	AS	E7EXA6	CHTF18
1221.1	Centromere/kinetochore protei	0.00E+00	2.77E+05	2.77E+05	2.77E+05	NULL	AS	O43264	ZW10
2186.1	Fibronectin type III domain-cont	0.00E+00	2.78E+05	2.78E+05	2.78E+05	NULL	AS	Q53EP0	FNDC3B
2657.1	Isoform 2 of Testis-expressed se	0.00E+00	2.79E+05	2.79E+05	2.79E+05	NULL	AS	Q8IWB9-2	TEX2
2222.1	Zinc finger protein 700	0.00E+00	2.80E+05	2.80E+05	2.80E+05	NULL	AS	Q9H0M5	ZNF700
2413.1	Zinc finger protein 664	0.00E+00	2.80E+05	2.80E+05	2.80E+05	NULL	AS	Q8N3J9	ZNF664
1258.1	Serine-rich coiled-coil domain-co	0.00E+00	2.81E+05	2.81E+05	2.81E+05	NULL	AS	Q9C0I3	CCSER1
1988.1	Small nuclear ribonucleoprotein	0.00E+00	2.81E+05	2.81E+05	2.81E+05	NULL	AS	P62304	SNRPE
2415.1	MICAL-like protein 1	0.00E+00	2.82E+05	2.82E+05	2.82E+05	NULL	AS	Q8N3F8	MICALL1
2981.1	Ral-GDS-related protein	0.00E+00	2.82E+05	2.82E+05	2.82E+05	NULL	AS	E9PH21	RGL4
2735.1	HLA class II histocompatibility ar	0.00E+00	2.82E+05	2.82E+05	2.82E+05	NULL	AS	P04440	HLA_DPB1
1383.1	Structural maintenance of chror	0.00E+00	2.83E+05	2.83E+05	2.83E+05	NULL	AS	Q14683	SMC1A
1698.1	HCG2002594, isoform CRA_c	0.00E+00	2.83E+05	2.83E+05	2.83E+05	NULL	AS	G3XAH0	SEPT5
2200.1	B-cell linker protein	0.00E+00	2.83E+05	2.83E+05	2.83E+05	NULL	AS	Q8WV28	BLNK
3264.1	Nance-Horan syndrome protein	0.00E+00	2.83E+05	2.83E+05	2.83E+05	NULL	AS	Q6T4R5	NHS
1515.1	Tektin-4	0.00E+00	2.84E+05	2.84E+05	2.84E+05	NULL	AS	Q8WW24	TEKT4
2183.1	Isoform 2 of Ubiquitin thioester	0.00E+00	2.84E+05	2.84E+05	2.84E+05	NULL	AS	Q96FW1-2	OTUB1
2278.1	Cyclin C	0.00E+00	2.84E+05	2.84E+05	2.84E+05	NULL	AS	Q5JV82	CCNC
2040.1	Thyroid receptor-interacting prc	0.00E+00	2.85E+05	2.85E+05	2.85E+05	NULL	AS	Q15643	TRIP11
3261.1	Isoform H7 of Myeloperoxidase	0.00E+00	2.85E+05	2.85E+05	2.85E+05	NULL	AS	P05164-3	MPO
2962.1	Kinesin-like protein KIF17	0.00E+00	2.86E+05	2.86E+05	2.86E+05	NULL	AS	Q9P2E2	KIF17
3251.1	Kelch repeat and BTB domain-co	0.00E+00	2.87E+05	2.87E+05	2.87E+05	NULL	AS	Q8NFY9	KBTBD8
1519.1	Ubiquinone biosynthesis monoc	0.00E+00	2.91E+05	2.91E+05	2.91E+05	NULL	AS	G3V4A6	COQ6
963.1	Isoform 4 of Kinesin-like protein	0.00E+00	2.92E+05	2.92E+05	2.92E+05	NULL	AS	O00139-4	KIF2A
1377.1	3-hydroxyisobutyrate dehydrog	0.00E+00	2.92E+05	2.92E+05	2.92E+05	NULL	AS	P31937	HIBADH
2698.1	Four and a half LIM domains prc	0.00E+00	2.93E+05	2.93E+05	2.93E+05	NULL	AS	Q13642	FHL1
3258.1	Ankyrin-2	0.00E+00	2.93E+05	2.93E+05	2.93E+05	NULL	AS	Q01484	ANK2
3255.1	ER degradation-enhancing alpha	0.00E+00	2.95E+05	2.95E+05	2.95E+05	NULL	AS	Q9BV94	EDEM2
281.2	High mobility group protein B2	0.00E+00	2.96E+05	2.96E+05	2.96E+05	NULL	AS	P26583	HMGB2
2026.1	Zinc finger protein 41	0.00E+00	2.96E+05	2.96E+05	2.96E+05	NULL	AS	P51814	ZNF41
2523.1	Acyl-coenzyme A thioesterase 8	0.00E+00	2.97E+05	2.97E+05	2.97E+05	NULL	AS	E9PIS4	ACOT8

1897.1	Mitotic checkpoint serine/threo	0.00E+00	2.98E+05	2.98E+05	2.98E+05	NULL	AS	O43683	BUB1
2802.1	Recombining binding protein su	0.00E+00	2.98E+05	2.98E+05	2.98E+05	NULL	AS	Q9UBG7	RBPJL
96.3	Far upstream element-binding p	0.00E+00	3.00E+05	3.00E+05	3.00E+05	NULL	AS	Q96I24	FUBP3
1529.1	Zinc finger protein 292	0.00E+00	3.03E+05	3.03E+05	3.03E+05	NULL	AS	O60281	ZNF292
1790.1	Double-strand-break repair prot	0.00E+00	3.03E+05	3.03E+05	3.03E+05	NULL	AS	O60216	RAD21
2894.1	Receptor-transporting protein 1	0.00E+00	3.04E+05	3.04E+05	3.04E+05	NULL	AS	P59025	RTP1
1230.1	Acyl-coenzyme A thioesterase 1	0.00E+00	3.07E+05	3.07E+05	3.07E+05	NULL	AS	Q9NPJ3	ACOT13
2704.1	Proline-rich protein PRCC	0.00E+00	3.07E+05	3.07E+05	3.07E+05	NULL	AS	Q92733	PRCC
960.1	SH3 domain-binding glutamic ac	0.00E+00	3.08E+05	3.08E+05	3.08E+05	NULL	AS	Q9H299	SH3BGRL3
1747.1	Isoform 2 of Lysine-specific dem	0.00E+00	3.09E+05	3.09E+05	3.09E+05	NULL	AS	Q9UGL1-2	KDM5B
2028.1	Paired immunoglobulin-like type	0.00E+00	3.09E+05	3.09E+05	3.09E+05	NULL	AS	Q9UKJ1	PILRA
2702.1	Isoform 2 of EVI5-like protein	0.00E+00	3.10E+05	3.10E+05	3.10E+05	NULL	AS	Q96CN4-2	EVI5L
3187.1	1-acyl-sn-glycerol-3-phosphate a	0.00E+00	3.10E+05	3.10E+05	3.10E+05	NULL	AS	Q9NUQ2	AGPAT5
663.1	mRNA export factor	0.00E+00	3.11E+05	3.11E+05	3.11E+05	NULL	AS	P78406	RAE1
1173.1	Isoform 3 of Cyclin-Y	0.00E+00	3.12E+05	3.12E+05	3.12E+05	NULL	AS	Q8ND76-3	CCNY
1290.1	SRSF protein kinase 3	0.00E+00	3.14E+05	3.14E+05	3.14E+05	NULL	AS	E7ETV6	SRPK3
897.1	E3 ubiquitin-protein ligase TRIM	0.00E+00	3.15E+05	3.15E+05	3.15E+05	NULL	AS	Q8WV44	TRIM41
1542.1	Cullin-3	0.00E+00	3.15E+05	3.15E+05	3.15E+05	NULL	AS	Q13618	CUL3
2663.1	THUMP domain-containing prot	0.00E+00	3.17E+05	3.17E+05	3.17E+05	NULL	AS	J3KQB0	THUMPD1
1307.1	Protein SERAC1	0.00E+00	3.20E+05	3.20E+05	3.20E+05	NULL	AS	Q96JX3	SERAC1
3067.1	Isoform 2 of Roundabout homol	0.00E+00	3.20E+05	3.20E+05	3.20E+05	NULL	AS	Q9Y6N7-2	ROBO1
729.1	Heterogeneous nuclear ribonuc	0.00E+00	3.21E+05	3.21E+05	3.21E+05	NULL	AS	Q9BUJ2	HNRNPUL1
2128.1	Protein naked cuticle homolog 1	0.00E+00	3.21E+05	3.21E+05	3.21E+05	NULL	AS	Q969G9	NKD1
3197.1	Mitogen-activated protein kinas	0.00E+00	3.21E+05	3.21E+05	3.21E+05	NULL	AS	Q99683	MAP3K5
2954.1	Bromodomain-containing prote	0.00E+00	3.22E+05	3.22E+05	3.22E+05	NULL	AS	O60885	BRD4
1829.1	YY1-associated protein 1	0.00E+00	3.23E+05	3.23E+05	3.23E+05	NULL	AS	Q9H869	YY1AP1
2235.1	Isoform 2 of Rho guanine nucleo	0.00E+00	3.25E+05	3.25E+05	3.25E+05	NULL	AS	Q9NR81-2	ARHGEF3
2848.1	Protein kinase C alpha type	0.00E+00	3.25E+05	3.25E+05	3.25E+05	NULL	AS	P17252	PRKCA
2740.1	Cerebellin-2	0.00E+00	3.26E+05	3.26E+05	3.26E+05	NULL	AS	Q8IUK8	CBLN2
2257.1	Myelin-associated oligodendroc	0.00E+00	3.27E+05	3.27E+05	3.27E+05	NULL	AS	G5E945	MOBP
1161.1	Coiled-coil-helix-coiled-coil-helix	0.00E+00	3.28E+05	3.28E+05	3.28E+05	NULL	AS	Q8N8C4	CHCHD5
3225.1	Transmembrane protease serine	0.00E+00	3.29E+05	3.29E+05	3.29E+05	NULL	AS	O60235	TMPRSS11D
2818.1	52 kDa repressor of the inhibito	0.00E+00	3.32E+05	3.32E+05	3.32E+05	NULL	AS	O43422	PRKRIR
2064.1	Isoform 2 of INO80 complex sub	0.00E+00	3.34E+05	3.34E+05	3.34E+05	NULL	AS	Q53TQ3-2	INO80D
2754.1	N-acetylated-alpha-linked acidic	0.00E+00	3.34E+05	3.34E+05	3.34E+05	NULL	AS	Q9Y3Q0	NAALAD2

1755.1	Leucine-rich PPR motif-containi	0.00E+00	3.36E+05	3.36E+05	3.36E+05	NULL	AS	P42704	LRPPRC
2528.1	Isoform 3 of Engulfment and cel	0.00E+00	3.37E+05	3.37E+05	3.37E+05	NULL	AS	Q92556-3	ELMO1
1807.1	Putative zinc finger protein 726I	0.00E+00	3.38E+05	3.38E+05	3.38E+05	NULL	AS	Q15940	ZNF726P1
1086.1	Probable G-protein coupled rec	0.00E+00	3.41E+05	3.41E+05	3.41E+05	NULL	AS	O15354	GPR37
1320.1	Isoform 2 of A disintegrin and m	0.00E+00	3.42E+05	3.42E+05	3.42E+05	NULL	AS	Q8TE60-2	ADAMTS18
2419.1	Histone deacetylase 7	0.00E+00	3.42E+05	3.42E+05	3.42E+05	NULL	AS	J3KPH8	HDAC7
1916.1	Advanced glycosylation end pro	0.00E+00	3.42E+05	3.42E+05	3.42E+05	NULL	AS	Q15109	AGER
1144.1	Alpha-N-acetylglucosaminidase	0.00E+00	3.44E+05	3.44E+05	3.44E+05	NULL	AS	P54802	NAGLU
1753.1	Lamin-B1	0.00E+00	3.44E+05	3.44E+05	3.44E+05	NULL	AS	P20700	LMNB1
1861.1	Insulin gene enhancer protein IS	0.00E+00	3.44E+05	3.44E+05	3.44E+05	NULL	AS	Q96A47	ISL2
1432.1	Sorbin and SH3 domain-contain	0.00E+00	3.44E+05	3.44E+05	3.44E+05	NULL	AS	O94875	SORBS2
3.5	Keratin, type II cuticular Hb4	0.00E+00	3.46E+05	3.46E+05	3.46E+05	NULL	AS	Q9NSB2	KRT84
1228.1	Synaptojanin-2	0.00E+00	3.47E+05	3.47E+05	3.47E+05	NULL	AS	O15056	SYNJ2
1226.1	Sentan	0.00E+00	3.48E+05	3.48E+05	3.48E+05	NULL	AS	C9JXY5	SNTN
3247.1	Ankyrin repeat domain-containi	0.00E+00	3.49E+05	3.49E+05	3.49E+05	NULL	AS	Q5CZ79	ANKRD20A8P
1588.1	Zinc finger protein 524	0.00E+00	3.50E+05	3.50E+05	3.50E+05	NULL	AS	Q96C55	ZNF524
2298.1	Hemoglobin subunit alpha	0.00E+00	3.50E+05	3.50E+05	3.50E+05	NULL	AS	P69905	HBA1
2903.1	Armadillo repeat protein delete	0.00E+00	3.50E+05	3.50E+05	3.50E+05	NULL	AS	O00192	ARVCF
3047.1	Versican core protein	0.00E+00	3.50E+05	3.50E+05	3.50E+05	NULL	AS	P13611	VCAN
1195.1	Probable E3 ubiquitin-protein lig	0.00E+00	3.51E+05	3.51E+05	3.51E+05	NULL	AS	Q15751	HERC1
1940.1	Sorcin	0.00E+00	3.52E+05	3.52E+05	3.52E+05	NULL	AS	P30626	SRI
2734.1	Protocadherin beta-1	0.00E+00	3.52E+05	3.52E+05	3.52E+05	NULL	AS	Q9Y5F3	PCDHB1
3135.1	Protein Z-dependent protease in	0.00E+00	3.52E+05	3.52E+05	3.52E+05	NULL	AS	G3V2W1	SERPINA10
3243.1	Eukaryotic translation initiation	0.00E+00	3.53E+05	3.53E+05	3.53E+05	NULL	AS	Q9P2K8	EIF2AK4
1351.1	HERV-K_5q33.3 provirus ancest	0.00E+00	3.54E+05	3.54E+05	3.54E+05	NULL	AS	P10266	POK10
1185.1	Cyclin-D1-binding protein 1	0.00E+00	3.55E+05	3.55E+05	3.55E+05	NULL	AS	O95273	CCNDBP1
1909.1	Methyltransferase-like protein 2	0.00E+00	3.55E+05	3.55E+05	3.55E+05	NULL	AS	Q9BUU2	METTL22
1051.1	Kelch domain-containing protei	0.00E+00	3.59E+05	3.59E+05	3.59E+05	NULL	AS	Q9Y2U9	KLHDC2
1360.2	Isoform 2 of Myosin phosphatas	0.00E+00	3.59E+05	3.59E+05	3.59E+05	NULL	AS	Q6WCQ1-2	MPRIP
3110.1	Isoform 3 of Protein shisa-6 hon	0.00E+00	3.60E+05	3.60E+05	3.60E+05	NULL	AS	Q6ZSJ9-3	SHISA6
849.1	Cathepsin Z	0.00E+00	3.61E+05	3.61E+05	3.61E+05	NULL	AS	Q9UBR2	CTSZ
2931.1	NXPE family member 3	0.00E+00	3.61E+05	3.61E+05	3.61E+05	NULL	AS	Q969Y0	NXPE3
981.1	Adenylate kinase isoenzyme 4, r	0.00E+00	3.63E+05	3.63E+05	3.63E+05	NULL	AS	P27144	AK4
1646.1	Enoyl-CoA delta isomerase 1, m	0.00E+00	3.63E+05	3.63E+05	3.63E+05	NULL	AS	P42126	ECI1
789.1	Small EDRK-rich factor 2	0.00E+00	3.64E+05	3.64E+05	3.64E+05	NULL	AS	P84101	SERF2

942.1	Protein SZT2	0.00E+00	3.64E+05	3.64E+05	3.64E+05	NULL	AS	Q5T011	SZT2
1218.1	Isoform 2 of MAX gene-associat	0.00E+00	3.66E+05	3.66E+05	3.66E+05	NULL	AS	Q8IWI9-2	MGA
2124.1	Telomeric repeat-binding factor	0.00E+00	3.66E+05	3.66E+05	3.66E+05	NULL	AS	Q15554	TERF2
1141.1	N-alpha-acetyltransferase 25, N	0.00E+00	3.68E+05	3.68E+05	3.68E+05	NULL	AS	Q14CX7	NAA25
1786.1	FYVE, RhoGEF and PH domain co	0.00E+00	3.68E+05	3.68E+05	3.68E+05	NULL	AS	G3XA97	FGD4
1456.1	Leucine-rich repeat and WD rep	0.00E+00	3.74E+05	3.74E+05	3.74E+05	NULL	AS	Q9UFC0	LRWD1
2852.1	Histone deacetylase 2	0.00E+00	3.74E+05	3.74E+05	3.74E+05	NULL	AS	J3KPW7	HDAC2
2971.1	Ubiquinone biosynthesis protein	0.00E+00	3.74E+05	3.74E+05	3.74E+05	NULL	AS	O75208	COQ9
1193.1	Potassium/sodium hyperpolariz	0.00E+00	3.76E+05	3.76E+05	3.76E+05	NULL	AS	Q9Y3Q4	HCN4
678.1	Protein flightless-1 homolog	0.00E+00	3.80E+05	3.80E+05	3.80E+05	NULL	AS	Q13045	FLII
2458.1	Dual specificity mitogen-activat	0.00E+00	3.80E+05	3.80E+05	3.80E+05	NULL	AS	P36507	MAP2K2
2888.1	Tetratricopeptide repeat protein	0.00E+00	3.81E+05	3.81E+05	3.81E+05	NULL	AS	Q6PGP7	TTC37
1992.1	Unconventional myosin-Ic	0.00E+00	3.82E+05	3.82E+05	3.82E+05	NULL	AS	O00159	MYO1C
3127.1	Testican-3	0.00E+00	3.83E+05	3.83E+05	3.83E+05	NULL	AS	Q9BQ16	SPOCK3
974.1	Isoform 3 of Importin-5	0.00E+00	3.84E+05	3.84E+05	3.84E+05	NULL	AS	O00410-3	IPO5
2987.1	DEP domain-containing protein	0.00E+00	3.84E+05	3.84E+05	3.84E+05	NULL	AS	Q5TB30	DEPDC1
841.1	U6 snRNA-associated Sm-like pr	0.00E+00	3.86E+05	3.86E+05	3.86E+05	NULL	AS	Q9Y4Z0	LSM4
2212.1	Isoform 2 of Janus kinase and m	0.00E+00	3.87E+05	3.87E+05	3.87E+05	NULL	AS	Q96N16-2	JAKMIP1
2350.1	Protein atonal homolog 1	0.00E+00	3.88E+05	3.88E+05	3.88E+05	NULL	AS	Q92858	ATOH1
2133.1	Transmembrane and TPR repeat	0.00E+00	3.91E+05	3.91E+05	3.91E+05	NULL	AS	Q6ZXV5	TMTC3
1151.1	Protein Dom3Z	0.00E+00	3.92E+05	3.92E+05	3.92E+05	NULL	AS	O77932	DOM3Z
1451.1	Isoform 3 of Twinfilin-1	0.00E+00	3.93E+05	3.93E+05	3.93E+05	NULL	AS	Q12792-3	TWF1
836.1	Eukaryotic translation initiation	0.00E+00	3.97E+05	3.97E+05	3.97E+05	NULL	AS	P55010	EIF5
2370.1	Semenogelin-1	0.00E+00	3.99E+05	3.99E+05	3.99E+05	NULL	AS	P04279	SEMG1
3154.1	Protein phosphatase 1F	0.00E+00	3.99E+05	3.99E+05	3.99E+05	NULL	AS	A8MX49	PPM1F
917.1	Neuron navigator 3	0.00E+00	4.00E+05	4.00E+05	4.00E+05	NULL	AS	Q8IVL0	NAV3
2866.1	Tudor and KH domain-containin	0.00E+00	4.04E+05	4.04E+05	4.04E+05	NULL	AS	Q9Y2W6	TDRKH
1586.1	DNA polymerase subunit gamm	0.00E+00	4.07E+05	4.07E+05	4.07E+05	NULL	AS	Q9UHN1	POLG2
3228.1	Ras-related GTP-binding protein	0.00E+00	4.13E+05	4.13E+05	4.13E+05	NULL	AS	Q5VZM2	RRAGB
795.1	Cleavage stimulation factor sub	0.00E+00	4.14E+05	4.14E+05	4.14E+05	NULL	AS	Q9H0L4	CSTF2T
1503.1	RNA polymerase-associated pro	0.00E+00	4.14E+05	4.14E+05	4.14E+05	NULL	AS	Q6PD62	CTR9
2375.1	Biorientation of chromosomes i	0.00E+00	4.15E+05	4.15E+05	4.15E+05	NULL	AS	Q8NFC6	BOD1L1
572.2	Adenine phosphoribosyltransfer	0.00E+00	4.18E+05	4.18E+05	4.18E+05	NULL	AS	H3BQZ9	APRT
2594.1	Serine/threonine-protein kinase	0.00E+00	4.22E+05	4.22E+05	4.22E+05	NULL	AS	Q9P0L2	MARK1
2145.1	Kinesin-like protein KIF3A	0.00E+00	4.26E+05	4.26E+05	4.26E+05	NULL	AS	E9PES4	KIF3A

2157.1	Transmembrane protein 199	0.00E+00	4.26E+05	4.26E+05	4.26E+05	NULL	AS	E9PBQ3	TMEM199
2398.1	Cyclin-dependent kinase 6	0.00E+00	4.26E+05	4.26E+05	4.26E+05	NULL	AS	Q00534	CDK6
745.1	Pre-rRNA-processing protein TSI	0.00E+00	4.31E+05	4.31E+05	4.31E+05	NULL	AS	Q2NL82	TSR1
1768.1	Isoform 3 of Cytosolic carboxype	0.00E+00	4.32E+05	4.32E+05	4.32E+05	NULL	AS	Q9UPW5-3	AGTPBP1
1418.1	ATP-binding cassette sub-family	0.00E+00	4.33E+05	4.33E+05	4.33E+05	NULL	AS	Q9NUQ8	ABCF3
2127.1	Isoform 7 of Aryl hydrocarbon re	0.00E+00	4.33E+05	4.33E+05	4.33E+05	NULL	AS	Q8WYA1-7	ARNTL2
613.1	60S ribosomal protein L12	0.00E+00	4.34E+05	4.34E+05	4.34E+05	NULL	AS	P30050	RPL12
1544.1	Leucine-rich repeat-containing p	0.00E+00	4.35E+05	4.35E+05	4.35E+05	NULL	AS	Q9BTT6	LRRC1
1944.1	5-azacytidine-induced protein 1	0.00E+00	4.35E+05	4.35E+05	4.35E+05	NULL	AS	Q9UPN4	AZI1
1745.1	Isoform B3 of Smoothelin	0.00E+00	4.38E+05	4.38E+05	4.38E+05	NULL	AS	P53814-6	SMTN
1251.1	Collagen alpha-3(VI) chain	0.00E+00	4.40E+05	4.40E+05	4.40E+05	NULL	AS	P12111	COL6A3
3198.1	Integrator complex subunit 7	0.00E+00	4.40E+05	4.40E+05	4.40E+05	NULL	AS	Q9NVH2	INTS7
676.1	Protein Red	0.00E+00	4.41E+05	4.41E+05	4.41E+05	NULL	AS	Q13123	IK
1486.1	Serine/threonine-protein kinase	0.00E+00	4.43E+05	4.43E+05	4.43E+05	NULL	AS	Q9C098	DCLK3
1034.1	Dynamin-like 120 kDa protein, m	0.00E+00	4.44E+05	4.44E+05	4.44E+05	NULL	AS	E5KLJ5	OPA1
1147.1	Isoform 2 of Calmodulin-regulat	0.00E+00	4.44E+05	4.44E+05	4.44E+05	NULL	AS	Q9P1Y5-2	CAMSAP3
1165.1	Eukaryotic translation initiation	0.00E+00	4.46E+05	4.46E+05	4.46E+05	NULL	AS	Q9UBQ5	EIF3K
826.1	Biliverdin reductase A	0.00E+00	4.47E+05	4.47E+05	4.47E+05	NULL	AS	P53004	BLVRA
1358.1	Synaptonemal complex protein	0.00E+00	4.47E+05	4.47E+05	4.47E+05	NULL	AS	Q8IZU3	SYCP3
1677.1	Transmembrane protein 168	0.00E+00	4.47E+05	4.47E+05	4.47E+05	NULL	AS	Q9H0V1	TMEM168
1776.1	Armadillo repeat-containing X-li	0.00E+00	4.47E+05	4.47E+05	4.47E+05	NULL	AS	Q7L311	ARMCX2
2302.1	Lysozyme g-like protein 1	0.00E+00	4.48E+05	4.48E+05	4.48E+05	NULL	AS	Q8N1E2	LYG1
1557.1	Protein FAM110D	0.00E+00	4.49E+05	4.49E+05	4.49E+05	NULL	AS	Q8TAY7	FAM110D
3096.1	ATP synthase subunit s-like prot	0.00E+00	4.53E+05	4.53E+05	4.53E+05	NULL	AS	Q9NW81	ATP5SL
1714.1	Interferon-induced helicase C do	0.00E+00	4.54E+05	4.54E+05	4.54E+05	NULL	AS	Q9BYX4	IFIH1
2957.1	Transcription factor HES-5	0.00E+00	4.54E+05	4.54E+05	4.54E+05	NULL	AS	Q5TA89	HES5
1216.1	Leucine-rich repeat serine/threo	0.00E+00	4.55E+05	4.55E+05	4.55E+05	NULL	AS	Q5S007	LRRK2
2958.1	F-box only protein 27	0.00E+00	4.55E+05	4.55E+05	4.55E+05	NULL	AS	Q8NI29	FBXO27
1772.1	Zinc finger protein 236	0.00E+00	4.57E+05	4.57E+05	4.57E+05	NULL	AS	J9JID5	ZNF236
650.1	Apoptosis-inducing factor 1, mit	0.00E+00	4.59E+05	4.59E+05	4.59E+05	NULL	AS	O95831	AIFM1
2636.1	Isoform 3 of Receptor-type tyro	0.00E+00	4.62E+05	4.62E+05	4.62E+05	NULL	AS	P23467-3	PTPRB
2812.1	Ankyrin repeat domain-containi	0.00E+00	4.62E+05	4.62E+05	4.62E+05	NULL	AS	C9JTQ0	ANKRD63
3157.1	Death-associated protein kinase	0.00E+00	4.63E+05	4.63E+05	4.63E+05	NULL	AS	Q5T8F1	DAPK1
1465.1	Sorting nexin-5	0.00E+00	4.64E+05	4.64E+05	4.64E+05	NULL	AS	Q9Y5X3	SNX5
1149.1	Alpha-mannosidase 2C1	0.00E+00	4.65E+05	4.65E+05	4.65E+05	NULL	AS	Q9NTJ4	MAN2C1

715.1	Coiled-coil domain-containing p	0.00E+00	4.67E+05	4.67E+05	4.67E+05	NULL	AS	Q16204	CCDC6
1163.1	Katanin p80 WD40 repeat-conta	0.00E+00	4.67E+05	4.67E+05	4.67E+05	NULL	AS	Q9BVA0	KATNB1
1381.1	Polyadenylate-binding protein-i	0.00E+00	4.67E+05	4.67E+05	4.67E+05	NULL	AS	Q9BPZ3	PAIP2
656.1	Peptidyl-prolyl cis-trans isomera	0.00E+00	4.68E+05	4.68E+05	4.68E+05	NULL	AS	P30405	PPIF
1427.1	Isoform 2 of HLA class I histocor	0.00E+00	4.69E+05	4.69E+05	4.69E+05	NULL	AS	P13746-2	HLA-A
1702.1	Tumor necrosis factor receptor :	0.00E+00	4.69E+05	4.69E+05	4.69E+05	NULL	AS	Q969Z4	RELT
1313.1	E3 ubiquitin-protein ligase HECT	0.00E+00	4.72E+05	4.72E+05	4.72E+05	NULL	AS	Q5T447	HECTD3
3004.1	SLIT and NTRK-like protein 4	0.00E+00	4.72E+05	4.72E+05	4.72E+05	NULL	AS	Q8IW52	SLITRK4
2842.1	KAT8 regulatory NSL complex su	0.00E+00	4.73E+05	4.73E+05	4.73E+05	NULL	AS	A0AUZ9	KANSL1L
1473.1	Nuclear pore complex protein N	0.00E+00	4.76E+05	4.76E+05	4.76E+05	NULL	AS	Q8N1F7	NUP93
636.1	Serine/arginine-rich splicing fact	0.00E+00	4.79E+05	4.79E+05	4.79E+05	NULL	AS	Q13242	SRSF9
2880.1	RING finger protein 113A	0.00E+00	4.80E+05	4.80E+05	4.80E+05	NULL	AS	O15541	RNF113A
2610.1	Phytanoyl-CoA hydroxylase-inte	0.00E+00	4.83E+05	4.83E+05	4.83E+05	NULL	AS	Q96FC7	PHYHIPL
1140.1	Ubiquitin-associated protein 2	0.00E+00	4.84E+05	4.84E+05	4.84E+05	NULL	AS	E7EWG4	UBAP2
1422.1	Signal peptide, CUB and EGF-like	0.00E+00	4.86E+05	4.86E+05	4.86E+05	NULL	AS	Q8IWY4	SCUBE1
752.1	Probable ATP-dependent RNA h	0.00E+00	4.89E+05	4.89E+05	4.89E+05	NULL	AS	Q7L014	DDX46
2264.1	Zinc/RING finger protein 4	0.00E+00	4.90E+05	4.90E+05	4.90E+05	NULL	AS	Q8WWF5	ZNRF4
1947.1	Kappa-type opioid receptor	0.00E+00	4.91E+05	4.91E+05	4.91E+05	NULL	AS	P41145	OPRK1
735.1	Peptidyl-prolyl cis-trans isomera	0.00E+00	4.93E+05	4.93E+05	4.93E+05	NULL	AS	Q5VVH2	FKBP1C
727.1	Hydroxysteroid dehydrogenase-	0.00E+00	4.94E+05	4.94E+05	4.94E+05	NULL	AS	Q6YN16	HSDL2
2860.1	Anion exchange protein 2	0.00E+00	4.94E+05	4.94E+05	4.94E+05	NULL	AS	P04920	SLC4A2
1434.1	C-type lectin domain family 4 m	0.00E+00	4.95E+05	4.95E+05	4.95E+05	NULL	AS	Q8N1N0	CLEC4F
3241.1	Galectin-3	0.00E+00	5.00E+05	5.00E+05	5.00E+05	NULL	AS	G3V3R6	LGALS3
1083.1	Sel1 repeat-containing protein 1	0.00E+00	5.02E+05	5.02E+05	5.02E+05	NULL	AS	Q96BR5	SELRC1
2221.1	Isoform 2 of Coiled-coil domain-	0.00E+00	5.02E+05	5.02E+05	5.02E+05	NULL	AS	Q8NEF3-2	CCDC112
2493.1	A disintegrin and metalloprotei	0.00E+00	5.02E+05	5.02E+05	5.02E+05	NULL	AS	Q9UNA0	ADAMTS5
878.1	Leucine--tRNA ligase, cytoplasm	0.00E+00	5.03E+05	5.03E+05	5.03E+05	NULL	AS	Q9P2J5	LARS
2542.1	Tsukushin	0.00E+00	5.03E+05	5.03E+05	5.03E+05	NULL	AS	Q8WUA8	TSKU
1458.1	Semaphorin-3A	0.00E+00	5.04E+05	5.04E+05	5.04E+05	NULL	AS	Q14563	SEMA3A
2313.1	Phytanoyl-CoA dioxygenase, pe	0.00E+00	5.08E+05	5.08E+05	5.08E+05	NULL	AS	O14832	PHYH
1003.1	General transcription factor IIF :	0.00E+00	5.09E+05	5.09E+05	5.09E+05	NULL	AS	P35269	GTF2F1
1142.1	Amine oxidase [flavin-containin	0.00E+00	5.10E+05	5.10E+05	5.10E+05	NULL	AS	P21397	MAOA
3079.1	Protein SOX-15	0.00E+00	5.13E+05	5.13E+05	5.13E+05	NULL	AS	O60248	SOX15
3262.1	Isoform 4 of Brain-derived neur	0.00E+00	5.13E+05	5.13E+05	5.13E+05	NULL	AS	P23560-4	BDNF
521.1	Transducin-like enhancer protei	0.00E+00	5.14E+05	5.14E+05	5.14E+05	NULL	AS	Q04724	TLE1

1399.1	Pleckstrin homology-like domai	0.00E+00	5.15E+05	5.15E+05	5.15E+05	NULL	AS	Q86SQ0	PHLDB2
1020.1	ATP-dependent DNA helicase Q	0.00E+00	5.20E+05	5.20E+05	5.20E+05	NULL	AS	P46063	RECQL
1871.1	Isoform B of Methyl-CpG-bindin	0.00E+00	5.21E+05	5.21E+05	5.21E+05	NULL	AS	P51608-2	MECP2
1029.1	Eukaryotic translation initiation	0.00E+00	5.22E+05	5.22E+05	5.22E+05	NULL	AS	B3KSH1	EIF3F
2976.1	Leucine-rich repeat-containing p	0.00E+00	5.23E+05	5.23E+05	5.23E+05	NULL	AS	A6NM11	LRRC37A2
1884.1	Homeobox protein Nkx-6.1	0.00E+00	5.24E+05	5.24E+05	5.24E+05	NULL	AS	P78426	NKX6-1
2327.1	Tyrosine-protein phosphatase n	0.00E+00	5.24E+05	5.24E+05	5.24E+05	NULL	AS	Q9Y2R2	PTPN22
2319.1	Aspartate beta-hydroxylase don	0.00E+00	5.27E+05	5.27E+05	5.27E+05	NULL	AS	Q6ICH7	ASPHD2
830.1	40S ribosomal protein S29	0.00E+00	5.29E+05	5.29E+05	5.29E+05	NULL	AS	P62273	RPS29
1276.1	Probable peptidyl-tRNA hydrola	0.00E+00	5.29E+05	5.29E+05	5.29E+05	NULL	AS	Q86Y79	PTRH1
1921.1	26S proteasome non-ATPase reg	0.00E+00	5.33E+05	5.33E+05	5.33E+05	NULL	AS	Q16401	PSMD5
2990.1	Periaxin	0.00E+00	5.38E+05	5.38E+05	5.38E+05	NULL	AS	Q9BXM0	PRX
617.2	Dynamin-2	0.00E+00	5.39E+05	5.39E+05	5.39E+05	NULL	AS	P50570	DNM2
700.1	Protein FAM114A2	0.00E+00	5.40E+05	5.40E+05	5.40E+05	NULL	AS	Q9NRY5	FAM114A2
1734.1	NLR family CARD domain-contai	0.00E+00	5.40E+05	5.40E+05	5.40E+05	NULL	AS	Q9NPP4	NLRC4
1160.1	Glutamate dehydrogenase 2, mi	0.00E+00	5.41E+05	5.41E+05	5.41E+05	NULL	AS	P49448	GLUD2
1961.1	Cytoskeleton-associated protein	0.00E+00	5.41E+05	5.41E+05	5.41E+05	NULL	AS	Q07065	CKAP4
198.2	Fragile X mental retardation syn	0.00E+00	5.43E+05	5.43E+05	5.43E+05	NULL	AS	P51116	FXR2
696.1	Succinyl-CoA ligase [ADP-formin	0.00E+00	5.44E+05	5.44E+05	5.44E+05	NULL	AS	Q9P2R7	SUCLA2
1000.1	Nuclear pore membrane glycopr	0.00E+00	5.46E+05	5.46E+05	5.46E+05	NULL	AS	Q8TEM1	NUP210
856.1	Eukaryotic translation initiation	0.00E+00	5.48E+05	5.48E+05	5.48E+05	NULL	AS	O60841	EIF5B
1775.1	AT-rich interactive domain-cont	0.00E+00	5.48E+05	5.48E+05	5.48E+05	NULL	AS	Q68CP9	ARID2
2712.1	Isoform 3 of TPA-induced transr	0.00E+00	5.48E+05	5.48E+05	5.48E+05	NULL	AS	Q5BVD1-3	TTMP
615.1	Serine/threonine-protein phosp	0.00E+00	5.49E+05	5.49E+05	5.49E+05	NULL	AS	Q96QC0	PPP1R10
2046.1	Taperin	0.00E+00	5.53E+05	5.53E+05	5.53E+05	NULL	AS	Q4KMQ1	TPRN
1838.1	Actin-related protein 2/3 compl	0.00E+00	5.54E+05	5.54E+05	5.54E+05	NULL	AS	O15145	ARPC3
2508.1	C-Maf-inducing protein	0.00E+00	5.62E+05	5.62E+05	5.62E+05	NULL	AS	Q8IY22	CMIP
1145.1	Protocadherin gamma-A9	0.00E+00	5.63E+05	5.63E+05	5.63E+05	NULL	AS	Q9Y5G4	PCDHGA9
2364.1	Mitochondrial dynamic protein	0.00E+00	5.63E+05	5.63E+05	5.63E+05	NULL	AS	B0QY95	SMCR7L
1172.1	Density-regulated protein	0.00E+00	5.66E+05	5.66E+05	5.66E+05	NULL	AS	O43583	DENR
3173.1	Vacuolar protein sorting-associa	0.00E+00	5.66E+05	5.66E+05	5.66E+05	NULL	AS	Q86XT2	VPS37D
2743.1	Protein CREG2	0.00E+00	5.68E+05	5.68E+05	5.68E+05	NULL	AS	Q8IUH2	CREG2
1120.1	Protein FAM26E	0.00E+00	5.70E+05	5.70E+05	5.70E+05	NULL	AS	Q8N5C1	FAM26E
1219.1	Sterile alpha motif domain-cont	0.00E+00	5.70E+05	5.70E+05	5.70E+05	NULL	AS	Q5K651	SAMD9
3011.1	Isoform 3 of Peripheral-type ber	0.00E+00	5.70E+05	5.70E+05	5.70E+05	NULL	AS	O95153-3	BZRAP1

1560.1	Isoform 2 of Dual specificity mit	0.00E+00	5.72E+05	5.72E+05	5.72E+05	NULL	AS	P45985-2	MAP2K4
2823.1	Inactive phospholipase D5	0.00E+00	5.75E+05	5.75E+05	5.75E+05	NULL	AS	Q8N7P1	PLD5
3223.1	ATP synthase mitochondrial F1 (	0.00E+00	5.75E+05	5.75E+05	5.75E+05	NULL	AS	J3KTB2	ATPAF2
2793.1	35 kDa inter-alpha-trypsin inhib	0.00E+00	5.78E+05	5.78E+05	5.78E+05	NULL	AS	B7ZKJ8	ITIH4
2703.1	Plasminogen receptor (KT)	0.00E+00	5.81E+05	5.81E+05	5.81E+05	NULL	AS	Q9HBL7	PLGRKT
3266.1	RNA exonuclease 1 homolog	0.00E+00	5.81E+05	5.81E+05	5.81E+05	NULL	AS	Q8N1G1	REXO1
962.1	AF4/FMR2 family member 4	0.00E+00	5.83E+05	5.83E+05	5.83E+05	NULL	AS	Q9UHB7	AFF4
1067.1	Enoyl-CoA hydratase, mitochon	0.00E+00	5.89E+05	5.89E+05	5.89E+05	NULL	AS	P30084	ECHS1
1903.1	Disco-interacting protein 2 hom	0.00E+00	5.90E+05	5.90E+05	5.90E+05	NULL	AS	Q9P265	DIP2B
2512.1	Sodium channel protein type 11	0.00E+00	5.90E+05	5.90E+05	5.90E+05	NULL	AS	Q9UI33	SCN11A
662.1	Lipoamide acyltransferase comp	0.00E+00	5.92E+05	5.92E+05	5.92E+05	NULL	AS	P11182	DBT
1904.1	von Willebrand factor A domain	0.00E+00	5.92E+05	5.92E+05	5.92E+05	NULL	AS	A3KMH1	VWA8
2272.1	Kinase D-interacting substrate o	0.00E+00	5.96E+05	5.96E+05	5.96E+05	NULL	AS	Q9ULH0	KIDINS220
1663.1	Isoform 2 of Rho GTPase-activat	0.00E+00	5.99E+05	5.99E+05	5.99E+05	NULL	AS	P42331-2	ARHGAP25
867.1	Isoform 5 of MAP7 domain-cont	0.00E+00	6.00E+05	6.00E+05	6.00E+05	NULL	AS	Q96T17-5	MAP7D2
502.1	Cordon-bleu protein-like 1	0.00E+00	6.01E+05	6.01E+05	6.01E+05	NULL	AS	G8JL86	COBLL1
873.1	Brain-specific angiogenesis inhib	0.00E+00	6.03E+05	6.03E+05	6.03E+05	NULL	AS	O14514	BAI1
526.1	Lon protease homolog, mitocho	0.00E+00	6.11E+05	6.11E+05	6.11E+05	NULL	AS	P36776	LONP1
628.1	ATP-binding cassette sub-family	0.00E+00	6.11E+05	6.11E+05	6.11E+05	NULL	AS	P61221	ABCE1
618.1	Carnitine O-acetyltransferase	0.00E+00	6.14E+05	6.14E+05	6.14E+05	NULL	AS	P43155	CRAT
1238.1	N-acetylaspartyl-glutamate synt	0.00E+00	6.14E+05	6.14E+05	6.14E+05	NULL	AS	Q8IXN7	RIMKLA
1770.1	Protein CEP170B	0.00E+00	6.20E+05	6.20E+05	6.20E+05	NULL	AS	J3KQR7	CEP170B
1943.1	Protein CASC5	0.00E+00	6.20E+05	6.20E+05	6.20E+05	NULL	AS	Q8NG31	CASC5
903.1	60S ribosomal protein L35a	0.00E+00	6.21E+05	6.21E+05	6.21E+05	NULL	AS	P18077	RPL35A
1583.1	Nucleolar GTP-binding protein 2	0.00E+00	6.21E+05	6.21E+05	6.21E+05	NULL	AS	Q13823	GNL2
1056.1	DKFZP586J0619 protein	0.00E+00	6.22E+05	6.22E+05	6.22E+05	NULL	AS	A4D212	DKFZP586J0619
425.1	26S proteasome non-ATPase re(	0.00E+00	6.23E+05	6.23E+05	6.23E+05	NULL	AS	Q99460	PSMD1
853.1	Nuclear pore complex protein N	0.00E+00	6.23E+05	6.23E+05	6.23E+05	NULL	AS	F6QR24	NUP153
919.1	Isoform 2 of E3 ubiquitin-protei	0.00E+00	6.28E+05	6.28E+05	6.28E+05	NULL	AS	Q63HN8-4	RNF213
1534.1	Tetratricopeptide repeat protei	0.00E+00	6.28E+05	6.28E+05	6.28E+05	NULL	AS	Q5T0N1	TTC18
2546.1	RNA polymerase II elongation fa	0.00E+00	6.28E+05	6.28E+05	6.28E+05	NULL	AS	O00472	ELL2
1763.1	5'-AMP-activated protein kinase	0.00E+00	6.29E+05	6.29E+05	6.29E+05	NULL	AS	P54619	PRKAG1
2019.1	HCG30379, isoform CRA_d	0.00E+00	6.30E+05	6.30E+05	6.30E+05	NULL	AS	K4DI98	NULL
1272.1	Leucine-rich repeat-containing (	0.00E+00	6.31E+05	6.31E+05	6.31E+05	NULL	AS	Q7Z2Q7	LRRC70
1297.1	Acyl-Coenzyme A dehydrogenas	0.00E+00	6.39E+05	6.39E+05	6.39E+05	NULL	AS	Q5T4U5	ACADM

3046.1	Nesprin-1	0.00E+00	6.40E+05	6.40E+05	6.40E+05	NULL	AS	Q8NF91	SYNE1
2344.1	Anoctamin-3	0.00E+00	6.41E+05	6.41E+05	6.41E+05	NULL	AS	Q9BYT9	ANO3
1533.1	Isoform 2 of Megakaryocyte-ass	0.00E+00	6.42E+05	6.42E+05	6.42E+05	NULL	AS	P42679-2	MATK
2422.1	Poly(ADP-ribose) glycohydrolase	0.00E+00	6.42E+05	6.42E+05	6.42E+05	NULL	AS	Q86W56	PARG
1875.1	Matrix metalloproteinase-27	0.00E+00	6.43E+05	6.43E+05	6.43E+05	NULL	AS	Q9H306	MMP27
1132.1	Pre-mRNA-processing factor 40	0.00E+00	6.46E+05	6.46E+05	6.46E+05	NULL	AS	O75400	PRPF40A
2477.1	Krueppel-like factor 4	0.00E+00	6.46E+05	6.46E+05	6.46E+05	NULL	AS	O43474	KLF4
2557.1	Cadherin-12	0.00E+00	6.50E+05	6.50E+05	6.50E+05	NULL	AS	P55289	CDH12
1299.1	Junction-mediating and -regulat	0.00E+00	6.54E+05	6.54E+05	6.54E+05	NULL	AS	Q8N9B5	JMY
2414.1	Junctional sarcoplasmic reticulu	0.00E+00	6.59E+05	6.59E+05	6.59E+05	NULL	AS	Q96MG2	JSRP1
2190.1	Metastasis-associated protein M	0.00E+00	6.69E+05	6.69E+05	6.69E+05	NULL	AS	E9PF88	MTA3
751.1	60S ribosomal protein L19	0.00E+00	6.70E+05	6.70E+05	6.70E+05	NULL	AS	P84098	RPL19
564.1	Purine nucleoside phosphorylas	0.00E+00	6.71E+05	6.71E+05	6.71E+05	NULL	AS	P00491	PNP
1411.1	Protein FAM186A	0.00E+00	6.72E+05	6.72E+05	6.72E+05	NULL	AS	A6NE01	FAM186A
2827.1	Glutathione S-transferase theta-	0.00E+00	6.73E+05	6.73E+05	6.73E+05	NULL	AS	A8MPT4	GSTT4
784.1	Charged multivesicular body prc	0.00E+00	6.75E+05	6.75E+05	6.75E+05	NULL	AS	Q8WUX9	CHMP7
1479.1	Isoform 2 of Coiled-coil domain-	0.00E+00	6.81E+05	6.81E+05	6.81E+05	NULL	AS	Q8IXS2-2	CCDC65
1509.1	Epsin-2	0.00E+00	6.84E+05	6.84E+05	6.84E+05	NULL	AS	O95208	EPN2
2009.1	Isoform 2 of Mitochondrial inne	0.00E+00	6.84E+05	6.84E+05	6.84E+05	NULL	AS	Q8N8Q8-2	COX18
1666.1	Dipeptidyl aminopeptidase-like	0.00E+00	6.93E+05	6.93E+05	6.93E+05	NULL	AS	P42658	DPP6
2510.1	Rho guanine nucleotide exchang	0.00E+00	6.97E+05	6.97E+05	6.97E+05	NULL	AS	Q8TER5	ARHGEF40
3168.1	Isoform 3 of Centromere protein	0.00E+00	6.99E+05	6.99E+05	6.99E+05	NULL	AS	Q96H22-3	CENPN
3260.1	Cytosolic 10-formyltetrahydrofc	0.00E+00	7.00E+05	7.00E+05	7.00E+05	NULL	AS	O75891	ALDH1L1
828.1	Down syndrome cell adhesion m	0.00E+00	7.01E+05	7.01E+05	7.01E+05	NULL	AS	Q8TD84	DSCAML1
693.1	Transcription termination factor	0.00E+00	7.04E+05	7.04E+05	7.04E+05	NULL	AS	Q9UNY4	TTF2
60.2	Protein RBM14-RBM4	0.00E+00	7.04E+05	7.04E+05	7.04E+05	NULL	AS	Q96PK6	RBM14
1402.1	Periplakin	0.00E+00	7.05E+05	7.05E+05	7.05E+05	NULL	AS	O60437	PPL
2150.1	F-box only protein 43	0.00E+00	7.06E+05	7.06E+05	7.06E+05	NULL	AS	Q4G163	FBXO43
2073.1	Pleckstrin homology domain-co	0.00E+00	7.07E+05	7.07E+05	7.07E+05	NULL	AS	Q6ZR37	PLEKHG7
994.1	ATP-binding cassette sub-family	0.00E+00	7.08E+05	7.08E+05	7.08E+05	NULL	AS	Q86UQ4	ABCA13
1378.1	DNA polymerase delta subunit 2	0.00E+00	7.10E+05	7.10E+05	7.10E+05	NULL	AS	P49005	POLD2
1593.1	Chromodomain-helicase-DNA-b	0.00E+00	7.11E+05	7.11E+05	7.11E+05	NULL	AS	Q9HCK8	CHD8
2102.1	UPF0505 protein C16orf62	0.00E+00	7.11E+05	7.11E+05	7.11E+05	NULL	AS	E7EWW0	C16orf62
3000.1	Usher syndrome type-1G protei	0.00E+00	7.11E+05	7.11E+05	7.11E+05	NULL	AS	J3KSN5	USH1G
2900.1	Retinol dehydrogenase 12	0.00E+00	7.16E+05	7.16E+05	7.16E+05	NULL	AS	Q96NR8	RDH12

24.3	Tubulin alpha-3C/D chain	0.00E+00	7.23E+05	7.23E+05	7.23E+05	NULL	AS	Q13748	TUBA3C
2396.1	Lysine-specific demethylase 7	0.00E+00	7.26E+05	7.26E+05	7.26E+05	NULL	AS	Q6ZMT4	JHDM1D
1978.1	Bcl2 antagonist of cell death	0.00E+00	7.27E+05	7.27E+05	7.27E+05	NULL	AS	F5GYS3	BAD
3123.1	Isoform 2 of Sphingosine kinase	0.00E+00	7.27E+05	7.27E+05	7.27E+05	NULL	AS	Q9NYA1-2	SPHK1
1370.1	NADH dehydrogenase [ubiquinc	0.00E+00	7.29E+05	7.29E+05	7.29E+05	NULL	AS	P51970	NDUFA8
991.1	CCR4-NOT transcription comple	0.00E+00	7.30E+05	7.30E+05	7.30E+05	NULL	AS	A5YKK6	CNOT1
1002.1	Glutamate receptor ionotropic,	0.00E+00	7.36E+05	7.36E+05	7.36E+05	NULL	AS	O60391	GRIN3B
2825.1	Immunoglobulin superfamily DC	0.00E+00	7.36E+05	7.36E+05	7.36E+05	NULL	AS	Q8TDY8	IGDCC4
1231.1	Ryanodine receptor 2	0.00E+00	7.39E+05	7.39E+05	7.39E+05	NULL	AS	H7BY35	RYR2
791.1	39S ribosomal protein L33, mito	0.00E+00	7.40E+05	7.40E+05	7.40E+05	NULL	AS	O75394	MRPL33
1513.1	Multiple epidermal growth facto	0.00E+00	7.47E+05	7.47E+05	7.47E+05	NULL	AS	Q7Z7M0	MEGF8
1416.1	Huntingtin-interacting protein 1	0.00E+00	7.51E+05	7.51E+05	7.51E+05	NULL	AS	O75146	HIP1R
600.1	Cathepsin B	0.00E+00	7.55E+05	7.55E+05	7.55E+05	NULL	AS	P07858	CTSB
1751.1	Isoform 3 of Neutrophil cytosol	0.00E+00	7.57E+05	7.57E+05	7.57E+05	NULL	AS	Q15080-3	NCF4
2430.1	Stabilin-2	0.00E+00	7.57E+05	7.57E+05	7.57E+05	NULL	AS	Q8WWQ8	STAB2
1043.1	Kelch domain-containing protei	0.00E+00	7.58E+05	7.58E+05	7.58E+05	NULL	AS	Q8IYD2	KLHDC8A
2792.1	TPR and ankyrin repeat-containi	0.00E+00	7.58E+05	7.58E+05	7.58E+05	NULL	AS	O15050	TRANK1
3022.1	Isoform 9 of DEP domain-contai	0.00E+00	7.78E+05	7.78E+05	7.78E+05	NULL	AS	O75140-9	DEPDC5
1016.1	Probable E3 ubiquitin-protein lig	0.00E+00	7.82E+05	7.82E+05	7.82E+05	NULL	AS	Q8IVU3	HERC6
1249.1	Type 2 lactosamine alpha-2,3-sia	0.00E+00	7.85E+05	7.85E+05	7.85E+05	NULL	AS	Q9Y274	ST3GAL6
1788.1	Galactose-3-O-sulfotransferase	0.00E+00	7.86E+05	7.86E+05	7.86E+05	NULL	AS	Q9H3Q3	GAL3ST2
712.1	Double-strand break repair prot	0.00E+00	7.87E+05	7.87E+05	7.87E+05	NULL	AS	B3KTC7	MRE11A
1648.1	PCNA-interacting partner	0.00E+00	7.87E+05	7.87E+05	7.87E+05	NULL	AS	B4DZ31	PARPBP
2000.1	E3 ubiquitin-protein ligase BRE1	0.00E+00	7.88E+05	7.88E+05	7.88E+05	NULL	AS	O75150	RNF40
2051.1	Arf-GAP with GTPase, ANK repea	0.00E+00	7.89E+05	7.89E+05	7.89E+05	NULL	AS	Q99490	AGAP2
536.1	Histone H1t	0.00E+00	7.91E+05	7.91E+05	7.91E+05	NULL	AS	P22492	HIST1H1T
1896.1	Protocadherin-15	0.00E+00	7.94E+05	7.94E+05	7.94E+05	NULL	AS	E7EMG8	PCDH15
869.1	ATP synthase subunit O, mitoch	0.00E+00	7.96E+05	7.96E+05	7.96E+05	NULL	AS	P48047	ATP5O
76.2	Heterogeneous nuclear ribonuc	0.00E+00	7.99E+05	7.99E+05	7.99E+05	NULL	AS	P55795	HNRNPH2
314.1	Isoform 2 of Hydroxyacyl-coenzy	0.00E+00	8.01E+05	8.01E+05	8.01E+05	NULL	AS	Q16836-2	HADH
397.1	Nuclear pore complex protein N	0.00E+00	8.06E+05	8.06E+05	8.06E+05	NULL	AS	Q99567	NUP88
2140.1	Galactose-3-O-sulfotransferase	0.00E+00	8.06E+05	8.06E+05	8.06E+05	NULL	AS	Q96A11	GAL3ST3
1407.1	Isoform 3 of E3 ubiquitin-protei	0.00E+00	8.09E+05	8.09E+05	8.09E+05	NULL	AS	Q14669-3	TRIP12
375.1	Plasminogen activator inhibitor	0.00E+00	8.10E+05	8.10E+05	8.10E+05	NULL	AS	Q8NC51	SERBP1
2010.1	Tigger transposable element-de	0.00E+00	8.21E+05	8.21E+05	8.21E+05	NULL	AS	Q96MW7	TIGD1

912.1	Growth/differentiation factor 1!	0.00E+00	8.23E+05	8.23E+05	8.23E+05	NULL	AS	Q99988	GDF15
2748.1	MAX gene-associated protein	0.00E+00	8.33E+05	8.33E+05	8.33E+05	NULL	AS	Q8IWI9	MGA
1799.1	Endonuclease III-like protein 1	0.00E+00	8.39E+05	8.39E+05	8.39E+05	NULL	AS	P78549	NTHL1
1952.1	Protein TBRG4	0.00E+00	8.39E+05	8.39E+05	8.39E+05	NULL	AS	Q969Z0	TBRG4
2326.1	Urocanate hydratase	0.00E+00	8.40E+05	8.40E+05	8.40E+05	NULL	AS	Q96N76	UROC1
1651.1	Peroxisomal biogenesis factor 1	0.00E+00	8.44E+05	8.44E+05	8.44E+05	NULL	AS	B7Z8B3	PEX19
2518.1	Transient receptor potential cat	0.00E+00	8.44E+05	8.44E+05	8.44E+05	NULL	AS	Q7Z2W7	TRPM8
308.1	Serine/arginine-rich splicing fact	0.00E+00	8.50E+05	8.50E+05	8.50E+05	NULL	AS	Q13247	SRSF6
437.1	Heterogeneous nuclear ribonuc	0.00E+00	8.50E+05	8.50E+05	8.50E+05	NULL	AS	Q13151	HNRNPA0
3126.1	Ral GTPase-activating protein su	0.00E+00	8.55E+05	8.55E+05	8.55E+05	NULL	AS	Q86X10	RALGAPB
429.1	40S ribosomal protein S8	0.00E+00	8.60E+05	8.60E+05	8.60E+05	NULL	AS	P62241	RPS8
3174.1	Isoform 2 of Ubiquitin carboxyl-	0.00E+00	8.60E+05	8.60E+05	8.60E+05	NULL	AS	Q9P275-2	USP36
1894.1	Chromosome 1 open reading fra	0.00E+00	8.61E+05	8.61E+05	8.61E+05	NULL	AS	J3KQM5	NULL
3002.1	Coiled-coil domain-containing p	0.00E+00	8.62E+05	8.62E+05	8.62E+05	NULL	AS	Q5T655	CCDC147
2877.1	Tetratricopeptide repeat protei	0.00E+00	8.66E+05	8.66E+05	8.66E+05	NULL	AS	Q7Z4L5	TTC21B
2139.1	Laminin subunit alpha-2	0.00E+00	8.67E+05	8.67E+05	8.67E+05	NULL	AS	P24043	LAMA2
2317.1	Protein NLRC5	0.00E+00	8.67E+05	8.67E+05	8.67E+05	NULL	AS	Q86WI3	NLRC5
1483.1	Enhancer of polycomb homolog	0.00E+00	8.70E+05	8.70E+05	8.70E+05	NULL	AS	Q9H2F5	EPC1
539.1	Chromosome transmission fidel	0.00E+00	8.73E+05	8.73E+05	8.73E+05	NULL	AS	P0CG12	CHTF8
69.2	ATP-dependent RNA helicase DI	0.00E+00	8.78E+05	8.78E+05	8.78E+05	NULL	AS	O00148	DDX39A
1382.1	Immunoglobulin iota chain	0.00E+00	8.82E+05	8.82E+05	8.82E+05	NULL	AS	P12018	VPREB1
335.1	Citrate synthase, mitochondrial	0.00E+00	8.87E+05	8.87E+05	8.87E+05	NULL	AS	O75390	CS
2178.1	Thioredoxin-related transmemb	0.00E+00	8.89E+05	8.89E+05	8.89E+05	NULL	AS	Q9H3N1	TMX1
2378.1	Protein FAM22F	0.00E+00	8.92E+05	8.92E+05	8.92E+05	NULL	AS	A1L443	NUTM2F
988.1	Serine/threonine-protein kinase	0.00E+00	8.96E+05	8.96E+05	8.96E+05	NULL	AS	O14757	CHEK1
3070.1	BUD13 homolog	0.00E+00	8.96E+05	8.96E+05	8.96E+05	NULL	AS	Q9BRD0	BUD13
825.1	Sulfotransferase 1A2	0.00E+00	9.05E+05	9.05E+05	9.05E+05	NULL	AS	P50226	SULT1A2
2274.1	Ras-related protein Rab-27B	0.00E+00	9.17E+05	9.17E+05	9.17E+05	NULL	AS	O00194	RAB27B
3105.1	PiggyBac transposable element	0.00E+00	9.18E+05	9.18E+05	9.18E+05	NULL	AS	J3KNK5	PGBD5
654.1	Dynein heavy chain 7, axonemal	0.00E+00	9.25E+05	9.25E+05	9.25E+05	NULL	AS	Q8WXX0	DNAH7
1395.1	THUMP domain-containing prot	0.00E+00	9.27E+05	9.27E+05	9.27E+05	NULL	AS	Q9BTF0	THUMPD2
351.1	U5 small nuclear ribonucleoprot	0.00E+00	9.39E+05	9.39E+05	9.39E+05	NULL	AS	O75643	SNRNP200
393.1	60S ribosomal protein L34	0.00E+00	9.46E+05	9.46E+05	9.46E+05	NULL	AS	P49207	RPL34
798.1	FRAS1-related extracellular mat	0.00E+00	9.47E+05	9.47E+05	9.47E+05	NULL	AS	Q5SZK8	FREM2
953.1	Isoform 2 of Septin-2	0.00E+00	9.66E+05	9.66E+05	9.66E+05	NULL	AS	Q15019-2	SEPT2

419.1	Isoform 2 of Aminoacyl tRNA sy	0.00E+00	9.67E+05	9.67E+05	9.67E+05	NULL	AS	Q12904-2	AIMP1
1777.1	Serine/threonine-protein phosp	0.00E+00	9.75E+05	9.75E+05	9.75E+05	NULL	AS	Q06190	PPP2R3A
1773.1	Integrin alpha-4	0.00E+00	9.78E+05	9.78E+05	9.78E+05	NULL	AS	P13612	ITGA4
1574.1	WD repeat-containing protein 4	0.00E+00	9.80E+05	9.80E+05	9.80E+05	NULL	AS	Q8TAF3	WDR48
3208.1	Integrator complex subunit 3	0.00E+00	9.86E+05	9.86E+05	9.86E+05	NULL	AS	Q68E01	INTS3
1701.1	Zinc finger protein 468	0.00E+00	9.94E+05	9.94E+05	9.94E+05	NULL	AS	Q5VIY5	ZNF468
732.1	Enhancer of yellow 2 transcripti	0.00E+00	1.00E+06	1.00E+06	1.00E+06	NULL	AS	Q9NPA8	ENY2
590.1	Heterogeneous nuclear ribonuc	0.00E+00	1.01E+06	1.01E+06	1.01E+06	NULL	AS	P51991	HNRNPA3
665.1	Isoform 2 of Acid ceramidase	0.00E+00	1.01E+06	1.01E+06	1.01E+06	NULL	AS	Q13510-2	ASAH1
2506.1	Growth hormone-regulated TBC	0.00E+00	1.01E+06	1.01E+06	1.01E+06	NULL	AS	Q5TC63	GRTP1
909.1	Protein FAM207A	0.00E+00	1.02E+06	1.02E+06	1.02E+06	NULL	AS	Q9NSI2	FAM207A
976.1	Glycylpeptide N-tetradecanoyltr	0.00E+00	1.03E+06	1.03E+06	1.03E+06	NULL	AS	P30419	NMT1
2755.1	Isoform 3 of Calmodulin-regulat	0.00E+00	1.03E+06	1.03E+06	1.03E+06	NULL	AS	Q5T5Y3-3	CAMSAP1
857.1	U6 snRNA-associated Sm-like pr	0.00E+00	1.04E+06	1.04E+06	1.04E+06	NULL	AS	P62310	LSM3
881.1	Discs, large homolog 3 (Neuroe	0.00E+00	1.05E+06	1.05E+06	1.05E+06	NULL	AS	Q5JUW8	DLG3
2693.1	Thioredoxin domain-containing	0.00E+00	1.05E+06	1.05E+06	1.05E+06	NULL	AS	Q8N427	NME8
796.1	ATP-dependent RNA helicase DH	0.00E+00	1.06E+06	1.06E+06	1.06E+06	NULL	AS	Q7Z478	DHX29
2356.1	Isoform 2 of Alcohol dehydroge	0.00E+00	1.06E+06	1.06E+06	1.06E+06	NULL	AS	P40394-2	ADH7
2644.1	1-phosphatidylinositol 4,5-bisph	0.00E+00	1.06E+06	1.06E+06	1.06E+06	NULL	AS	Q9NQ66	PLCB1
1280.1	Isoform 2 of Bifunctional coenzy	0.00E+00	1.07E+06	1.07E+06	1.07E+06	NULL	AS	Q13057-2	COASY
1478.1	Putative Polycomb group protei	0.00E+00	1.07E+06	1.07E+06	1.07E+06	NULL	AS	Q76L83	ASXL2
3016.1	Zinc finger protein 154	0.00E+00	1.07E+06	1.07E+06	1.07E+06	NULL	AS	Q13106	ZNF154
1645.1	Heat shock 70 kDa protein 4L	0.00E+00	1.08E+06	1.08E+06	1.08E+06	NULL	AS	E7ES43	HSPA4L
1973.1	Isoform 7 of CCR4-NOT transcrip	0.00E+00	1.08E+06	1.08E+06	1.08E+06	NULL	AS	O95628-7	CNOT4
2608.1	Tubby-related protein 4	0.00E+00	1.10E+06	1.10E+06	1.10E+06	NULL	AS	Q9NRJ4	TULP4
2695.1	Serine/threonine-protein kinase	0.00E+00	1.10E+06	1.10E+06	1.10E+06	NULL	AS	O00444	PLK4
459.1	Ras GTPase-activating-like prote	0.00E+00	1.11E+06	1.11E+06	1.11E+06	NULL	AS	P46940	IQGAP1
1612.1	Matrix metalloproteinase-26	0.00E+00	1.11E+06	1.11E+06	1.11E+06	NULL	AS	Q9NRE1	MMP26
597.1	Putative 40S ribosomal protein S	0.00E+00	1.12E+06	1.12E+06	1.12E+06	NULL	AS	Q5JNZ5	RPS26P11
834.1	Mitochondrial import inner mer	0.00E+00	1.12E+06	1.12E+06	1.12E+06	NULL	AS	O60220	TIMM8A
1130.1	Alpha-fetoprotein	0.00E+00	1.12E+06	1.12E+06	1.12E+06	NULL	AS	J3KMX3	AFP
3191.1	Laminin subunit gamma-1	0.00E+00	1.12E+06	1.12E+06	1.12E+06	NULL	AS	P11047	LAMC1
730.1	V-type proton ATPase subunit D	0.00E+00	1.13E+06	1.13E+06	1.13E+06	NULL	AS	Q9Y5K8	ATP6V1D
918.1	Transformer-2 protein homolog	0.00E+00	1.13E+06	1.13E+06	1.13E+06	NULL	AS	P62995	TRA2B
386.1	Mitotic checkpoint protein BUB	0.00E+00	1.14E+06	1.14E+06	1.14E+06	NULL	AS	O43684	BUB3

882.1	Ran GTPase-activating protein 1	0.00E+00	1.14E+06	1.14E+06	1.14E+06	NULL	AS	P46060	RANGAP1
507.1	Transportin-1	0.00E+00	1.15E+06	1.15E+06	1.15E+06	NULL	AS	Q92973	TNPO1
423.1	Isoform Beta of Nucleolar and co	0.00E+00	1.16E+06	1.16E+06	1.16E+06	NULL	AS	Q14978-2	NOLC1
1095.1	Ubiquitin-like protein 5	0.00E+00	1.16E+06	1.16E+06	1.16E+06	NULL	AS	Q9BZL1	UBL5
1207.1	GATA zinc finger domain contair	0.00E+00	1.16E+06	1.16E+06	1.16E+06	NULL	AS	B5MC40	GATAD2A
859.1	Synaptic vesicle membrane prot	0.00E+00	1.17E+06	1.17E+06	1.17E+06	NULL	AS	Q99536	VAT1
934.1	Cyclic AMP-dependent transcrip	0.00E+00	1.17E+06	1.17E+06	1.17E+06	NULL	AS	P18850	ATF6
1338.1	E3 ubiquitin-protein ligase TRIM	0.00E+00	1.17E+06	1.17E+06	1.17E+06	NULL	AS	Q6AZZ1	TRIM68
1613.1	T-cell receptor delta chain C reg	0.00E+00	1.17E+06	1.17E+06	1.17E+06	NULL	AS	B7Z8K6	TRDC
2332.1	E3 ubiquitin-protein ligase CBL	0.00E+00	1.17E+06	1.17E+06	1.17E+06	NULL	AS	P22681	CBL
452.1	40S ribosomal protein S27	0.00E+00	1.18E+06	1.18E+06	1.18E+06	NULL	AS	H0YMV8	RPS27L
547.1	Isoform 2 of Cytochrome c oxida	0.00E+00	1.18E+06	1.18E+06	1.18E+06	NULL	AS	Q5JTJ3-2	COA6
594.1	Coronin-1B	0.00E+00	1.18E+06	1.18E+06	1.18E+06	NULL	AS	Q9BR76	CORO1B
1640.1	Protein FAM221A	0.00E+00	1.18E+06	1.18E+06	1.18E+06	NULL	AS	A4D161	FAM221A
1738.1	Sterol 26-hydroxylase, mitochor	0.00E+00	1.18E+06	1.18E+06	1.18E+06	NULL	AS	Q02318	CYP27A1
2494.1	Olfactory receptor 51B4	0.00E+00	1.18E+06	1.18E+06	1.18E+06	NULL	AS	Q9Y5P0	OR51B4
2591.1	Treslin	0.00E+00	1.18E+06	1.18E+06	1.18E+06	NULL	AS	Q7Z2Z1	TICRR
544.1	Nucleolar protein 56	0.00E+00	1.19E+06	1.19E+06	1.19E+06	NULL	AS	O00567	NOP56
1482.1	Utrophin	0.00E+00	1.19E+06	1.19E+06	1.19E+06	NULL	AS	P46939	UTRN
1459.1	Protein phosphatase 1 regulator	0.00E+00	1.20E+06	1.20E+06	1.20E+06	NULL	AS	Q86XI6	PPP1R3B
1540.1	Neuralized-like protein 4	0.00E+00	1.21E+06	1.21E+06	1.21E+06	NULL	AS	Q96JN8	NEURL4
411.1	Short/branched chain specific ac	0.00E+00	1.23E+06	1.23E+06	1.23E+06	NULL	AS	P45954	ACADSB
1365.1	SUN domain-containing protein	0.00E+00	1.24E+06	1.24E+06	1.24E+06	NULL	AS	A4D2Q0	UNC84A
2155.1	Zinc finger MYM-type protein 6	0.00E+00	1.25E+06	1.25E+06	1.25E+06	NULL	AS	O95789	ZMYM6
1879.1	Tripartite motif-containing prot	0.00E+00	1.29E+06	1.29E+06	1.29E+06	NULL	AS	B5MCJ9	TRIM66
620.1	Eukaryotic translation initiation	0.00E+00	1.30E+06	1.30E+06	1.30E+06	NULL	AS	Q9BY44	EIF2A
2101.1	Hepatocyte nuclear factor 1-bet	0.00E+00	1.30E+06	1.30E+06	1.30E+06	NULL	AS	P35680	HNF1B
2840.1	Zinc finger protein 224	0.00E+00	1.32E+06	1.32E+06	1.32E+06	NULL	AS	Q9NZL3	ZNF224
1610.1	Catalase	0.00E+00	1.33E+06	1.33E+06	1.33E+06	NULL	AS	P04040	CAT
661.1	60S ribosomal protein L21	0.00E+00	1.34E+06	1.34E+06	1.34E+06	NULL	AS	P46778	RPL21
1541.1	Zinc finger CCCH domain-contai	0.00E+00	1.35E+06	1.35E+06	1.35E+06	NULL	AS	Q8IWR0	ZC3H7A
2786.1	Trinucleotide repeat-containing	0.00E+00	1.36E+06	1.36E+06	1.36E+06	NULL	AS	O15417	TNRC18
501.1	60S ribosomal protein L10	0.00E+00	1.41E+06	1.41E+06	1.41E+06	NULL	AS	P27635	RPL10
344.1	Eukaryotic translation initiation	0.00E+00	1.43E+06	1.43E+06	1.43E+06	NULL	AS	O15371	EIF3D
640.1	14 kDa phosphohistidine phospl	0.00E+00	1.46E+06	1.46E+06	1.46E+06	NULL	AS	Q9NRX4	PHPT1

977.1	Lysophospholipase-like protein	0.00E+00	1.48E+06	1.48E+06	1.48E+06	NULL	AS	Q5VWZ2	LYPLAL1
1443.1	Isoform 3 of Collagen type IV alp	0.00E+00	1.48E+06	1.48E+06	1.48E+06	NULL	AS	Q9Y5P4-3	COL4A3BP
1022.1	Putative SAGE1-like protein	0.00E+00	1.50E+06	1.50E+06	1.50E+06	NULL	AS	A6NJ88	SGE1L
2714.1	Zinc finger Ran-binding domain-	0.00E+00	1.51E+06	1.51E+06	1.51E+06	NULL	AS	O95218	ZRANB2
2224.1	Nck-associated protein 5	0.00E+00	1.53E+06	1.53E+06	1.53E+06	NULL	AS	O14513	NCKAP5
1877.1	Erlin-1	0.00E+00	1.56E+06	1.56E+06	1.56E+06	NULL	AS	O75477	ERLIN1
1294.1	Diphosphoinositol polyphospha	0.00E+00	1.57E+06	1.57E+06	1.57E+06	NULL	AS	O95989	NUDT3
440.1	Serine/arginine-rich-splicing fac	0.00E+00	1.59E+06	1.59E+06	1.59E+06	NULL	AS	J3KTL2	SRSF1
766.1	Hypermethylated in cancer 2 pr	0.00E+00	1.61E+06	1.61E+06	1.61E+06	NULL	AS	Q96JB3	HIC2
480.1	60S ribosomal protein L29	0.00E+00	1.62E+06	1.62E+06	1.62E+06	NULL	AS	P47914	RPL29
1712.1	Zinc finger and BTB domain-con	0.00E+00	1.63E+06	1.63E+06	1.63E+06	NULL	AS	Q86UZ6	ZBTB46
2236.1	Signal recognition particle recep	0.00E+00	1.68E+06	1.68E+06	1.68E+06	NULL	AS	Q9Y5M8	SRPRB
169.3	Keratin, type I cytoskeletal 27	0.00E+00	1.73E+06	1.73E+06	1.73E+06	NULL	AS	Q7Z3Y8	KRT27
1197.1	EF-hand calcium-binding domaiı	0.00E+00	1.74E+06	1.74E+06	1.74E+06	NULL	AS	Q5THR3	EFCAB6
2600.1	Coiled-coil domain-containing p	0.00E+00	1.74E+06	1.74E+06	1.74E+06	NULL	AS	Q96MC2	DRC1
342.1	Isoform 3 of Cleavage and polya	0.00E+00	1.75E+06	1.75E+06	1.75E+06	NULL	AS	Q8N684-3	CPSF7
407.1	RNA-binding protein 12B	0.00E+00	1.77E+06	1.77E+06	1.77E+06	NULL	AS	Q8IXT5	RBM12B
2940.1	Tryptophan 2,3-dioxygenase	0.00E+00	1.80E+06	1.80E+06	1.80E+06	NULL	AS	P48775	TDO2
746.1	40S ribosomal protein S23	0.00E+00	1.81E+06	1.81E+06	1.81E+06	NULL	AS	P62266	RPS23
341.1	Pyrroline-5-carboxylate reducta	0.00E+00	1.83E+06	1.83E+06	1.83E+06	NULL	AS	B4DMU0	PYCR1
422.1	60S ribosomal protein L27a	0.00E+00	1.83E+06	1.83E+06	1.83E+06	NULL	AS	P46776	RPL27A
373.1	Plakophilin-3	0.00E+00	1.84E+06	1.84E+06	1.84E+06	NULL	AS	Q9Y446	PKP3
506.1	Vigilin	0.00E+00	1.88E+06	1.88E+06	1.88E+06	NULL	AS	Q00341	HDLBP
3149.1	Liprin-alpha-2	0.00E+00	1.88E+06	1.88E+06	1.88E+06	NULL	AS	F8VWG6	PPFIA2
900.1	Protein syndesmos	0.00E+00	1.94E+06	1.94E+06	1.94E+06	NULL	AS	Q9BRJ7	NUDT16L1
1433.1	Isoform 4 of Vacuolar protein sc	0.00E+00	1.95E+06	1.95E+06	1.95E+06	NULL	AS	Q5VIR6-4	VPS53
475.1	Isoform 2 of Ferrochelatase, mit	0.00E+00	1.96E+06	1.96E+06	1.96E+06	NULL	AS	P22830-2	FECH
1936.1	Zinc finger and BTB domain-con	0.00E+00	1.96E+06	1.96E+06	1.96E+06	NULL	AS	O15062	ZBTB5
3113.1	Transmembrane protein 143	0.00E+00	1.96E+06	1.96E+06	1.96E+06	NULL	AS	B4DMT0	TMEM143
529.1	Isoform 2 of Chromodomain-hel	0.00E+00	1.97E+06	1.97E+06	1.97E+06	NULL	AS	Q14839-2	CHD4
891.1	WW domain-binding protein 11	0.00E+00	2.01E+06	2.01E+06	2.01E+06	NULL	AS	Q9Y2W2	WBP11
340.1	60S ribosomal protein L15	0.00E+00	2.03E+06	2.03E+06	2.03E+06	NULL	AS	P61313	RPL15
559.1	N-acetylglucosamine-6-sulfatase	0.00E+00	2.06E+06	2.06E+06	2.06E+06	NULL	AS	F6S8M0	GNS
447.1	60S ribosomal protein L23a	0.00E+00	2.07E+06	2.07E+06	2.07E+06	NULL	AS	A8MUS3	RPL23A
916.1	Isoform 2 of DNA (cytosine-5)-m	0.00E+00	2.09E+06	2.09E+06	2.09E+06	NULL	AS	P26358-2	DNMT1

817.1	Nuclear pore complex protein N	0.00E+00	2.10E+06	2.10E+06	2.10E+06	NULL	AS	P52948	NUP98
531.1	Septin-8	0.00E+00	2.12E+06	2.12E+06	2.12E+06	NULL	AS	Q92599	SEPT8
2008.1	Presenilin-1	0.00E+00	2.18E+06	2.18E+06	2.18E+06	NULL	AS	P49768	PSEN1
742.1	Activated RNA polymerase II tra	0.00E+00	2.21E+06	2.21E+06	2.21E+06	NULL	AS	P53999	SUB1
555.1	Glucosidase 2 subunit beta	0.00E+00	2.22E+06	2.22E+06	2.22E+06	NULL	AS	K7ELL7	PRKCSH
790.1	THO complex subunit 4	0.00E+00	2.22E+06	2.22E+06	2.22E+06	NULL	AS	E9PB61	ALYREF
333.1	Specifically androgen-regulated	0.00E+00	2.23E+06	2.23E+06	2.23E+06	NULL	AS	Q9BW04	SARG
2383.1	Serine/threonine-protein kinase	0.00E+00	2.24E+06	2.24E+06	2.24E+06	NULL	AS	P0C263	SBK2
850.1	CWF19-like protein 2	0.00E+00	2.28E+06	2.28E+06	2.28E+06	NULL	AS	Q2TBE0	CWF19L2
832.1	Importin subunit alpha	0.00E+00	2.31E+06	2.31E+06	2.31E+06	NULL	AS	F5GYL8	KPNA6
1621.1	Serine/threonine-protein kinase	0.00E+00	2.31E+06	2.31E+06	2.31E+06	NULL	AS	O00506	STK25
432.1	Eukaryotic translation initiation	0.00E+00	2.34E+06	2.34E+06	2.34E+06	NULL	AS	P20042	EIF2S2
278.1	60S ribosomal protein L3	0.00E+00	2.37E+06	2.37E+06	2.37E+06	NULL	AS	P39023	RPL3
410.1	DNA replication licensing factor	0.00E+00	2.37E+06	2.37E+06	2.37E+06	NULL	AS	P33992	MCM5
190.1	Kinectin	0.00E+00	2.39E+06	2.39E+06	2.39E+06	NULL	AS	Q86UP2	KTN1
707.1	Aldehyde dehydrogenase X, mit	0.00E+00	2.40E+06	2.40E+06	2.40E+06	NULL	AS	P30837	ALDH1B1
392.1	Selenoprotein H	0.00E+00	2.42E+06	2.42E+06	2.42E+06	NULL	AS	Q8IZQ5	SELH
599.1	Isoform 2 of Apoptosis inhibitor	0.00E+00	2.42E+06	2.42E+06	2.42E+06	NULL	AS	Q9BZZ5-2	API5
961.1	Huntingtin	0.00E+00	2.42E+06	2.42E+06	2.42E+06	NULL	AS	P42858	HTT
44.3	Peroxiredoxin-4	0.00E+00	2.45E+06	2.45E+06	2.45E+06	NULL	AS	Q13162	PRDX4
713.1	Isoform 3 of Protein scribble ho	0.00E+00	2.46E+06	2.46E+06	2.46E+06	NULL	AS	Q14160-3	SCRIB
434.1	Heterogeneous nuclear ribonuc	0.00E+00	2.48E+06	2.48E+06	2.48E+06	NULL	AS	P31942	HNRNPH3
359.1	E3 ubiquitin/ISG15 ligase TRIM2	0.00E+00	2.52E+06	2.52E+06	2.52E+06	NULL	AS	Q14258	TRIM25
1493.1	UPF0568 protein C14orf166	0.00E+00	2.53E+06	2.53E+06	2.53E+06	NULL	AS	Q9Y224	C14orf166
360.1	ATP-dependent RNA helicase A	0.00E+00	2.55E+06	2.55E+06	2.55E+06	NULL	AS	Q08211	DHX9
703.1	Protocadherin Fat 1	0.00E+00	2.56E+06	2.56E+06	2.56E+06	NULL	AS	Q14517	FAT1
842.1	Isoform Beta-4B of Integrin beta	0.00E+00	2.56E+06	2.56E+06	2.56E+06	NULL	AS	P16144-3	ITGB4
275.1	Regulator of microtubule dynan	0.00E+00	2.59E+06	2.59E+06	2.59E+06	NULL	AS	Q96DB5	RMDN1
2613.1	Cubilin	0.00E+00	2.68E+06	2.68E+06	2.68E+06	NULL	AS	O60494	CUBN
377.1	Ras GTPase-activating protein-b	0.00E+00	2.79E+06	2.79E+06	2.79E+06	NULL	AS	Q9UN86	G3BP2
837.1	Isoform SM-B1 of Small nuclear	0.00E+00	2.79E+06	2.79E+06	2.79E+06	NULL	AS	P14678-3	SNRPB
358.1	60S ribosomal protein L11	0.00E+00	2.85E+06	2.85E+06	2.85E+06	NULL	AS	P62913	RPL11
405.1	Interleukin enhancer binding fa	0.00E+00	2.90E+06	2.90E+06	2.90E+06	NULL	AS	Q12906	ILF3
472.1	Thioredoxin	0.00E+00	3.03E+06	3.03E+06	3.03E+06	NULL	AS	P10599	TXN
412.1	Isoform 2 of Succinyl-CoA ligase	0.00E+00	3.04E+06	3.04E+06	3.04E+06	NULL	AS	Q96I99-2	SUCLG2

385.1	U1 small nuclear ribonucleoprot	0.00E+00	3.08E+06	3.08E+06	3.08E+06	NULL	AS	P08621	SNRNP70
238.1	SAFB protein	0.00E+00	3.13E+06	3.13E+06	3.13E+06	NULL	AS	Q15424	SAFB
355.1	Serine/threonine-protein kinase	0.00E+00	3.33E+06	3.33E+06	3.33E+06	NULL	AS	O96013	PAK4
1470.1	Thioredoxin domain-containing	0.00E+00	3.37E+06	3.37E+06	3.37E+06	NULL	AS	Q6PKC3	TXNDC11
291.1	Isoform 3 of Cytoskeleton-assoc	0.00E+00	3.41E+06	3.41E+06	3.41E+06	NULL	AS	Q14008-3	CKAP5
259.1	60S ribosomal protein L7	0.00E+00	3.48E+06	3.48E+06	3.48E+06	NULL	AS	P18124	RPL7
327.1	Transcription elongation regulat	0.00E+00	3.53E+06	3.53E+06	3.53E+06	NULL	AS	O14776	TCERG1
415.1	Arginine--tRNA ligase, cytoplasn	0.00E+00	3.56E+06	3.56E+06	3.56E+06	NULL	AS	P54136	RARS
399.1	Alpha-L-iduronidase	0.00E+00	3.68E+06	3.68E+06	3.68E+06	NULL	AS	P35475	IDUA
292.1	ATP-binding cassette sub-family	0.00E+00	3.82E+06	3.82E+06	3.82E+06	NULL	AS	Q75MJ1	ABCF2
348.1	Heterogeneous nuclear ribonuc	0.00E+00	3.82E+06	3.82E+06	3.82E+06	NULL	AS	O14979	HNRPDL
279.1	Caprin-1	0.00E+00	3.92E+06	3.92E+06	3.92E+06	NULL	AS	Q14444	CAPRIN1
326.1	3-hydroxyacyl-CoA dehydrogena	0.00E+00	4.06E+06	4.06E+06	4.06E+06	NULL	AS	Q99714	HSD17B10
454.1	Elongation factor 1-beta	0.00E+00	4.08E+06	4.08E+06	4.08E+06	NULL	AS	P24534	EEF1B2
759.1	40S ribosomal protein S15	0.00E+00	4.09E+06	4.09E+06	4.09E+06	NULL	AS	K7ELC2	RPS15
490.1	Isoform 2 of Peptidyl-prolyl cis-t	0.00E+00	4.12E+06	4.12E+06	4.12E+06	NULL	AS	Q9Y237-2	PIN4
357.1	Septin-9	0.00E+00	4.16E+06	4.16E+06	4.16E+06	NULL	AS	Q9UHD8	SEPT9
404.1	40S ribosomal protein S4, X isof	0.00E+00	4.18E+06	4.18E+06	4.18E+06	NULL	AS	P62701	RPS4X
302.1	Mitochondrial import inner mer	0.00E+00	4.22E+06	4.22E+06	4.22E+06	NULL	AS	G3XAN8	TIMM8B
205.1	Heterogeneous nuclear ribonuc	0.00E+00	4.32E+06	4.32E+06	4.32E+06	NULL	AS	P14866	HNRNPL
309.1	60S ribosomal protein L7a	0.00E+00	4.43E+06	4.43E+06	4.43E+06	NULL	AS	P62424	RPL7A
538.1	Very long-chain-specific acyl-Co.	0.00E+00	4.70E+06	4.70E+06	4.70E+06	NULL	AS	P49748	ACADVL
591.1	Dihydrolipoyl dehydrogenase, m	0.00E+00	4.77E+06	4.77E+06	4.77E+06	NULL	AS	P09622	DLD
281.1	High mobility group protein B1	0.00E+00	4.95E+06	4.95E+06	4.95E+06	NULL	AS	P09429	HMGB1
76.1	Heterogeneous nuclear ribonuc	0.00E+00	5.00E+06	5.00E+06	5.00E+06	NULL	AS	G8JLB6	HNRNPH1
406.1	Small nuclear ribonucleoprotein	0.00E+00	5.07E+06	5.07E+06	5.07E+06	NULL	AS	P62306	SNRPF
408.1	Small nuclear ribonucleoprotein	0.00E+00	5.11E+06	5.11E+06	5.11E+06	NULL	AS	P62316	SNRPD2
134.1	Poly [ADP-ribose] polymerase 1	0.00E+00	5.12E+06	5.12E+06	5.12E+06	NULL	AS	P09874	PARP1
578.1	Dedicator of cytokinesis protein	0.00E+00	5.37E+06	5.37E+06	5.37E+06	NULL	AS	Q14185	DOCK1
122.1	Serine hydroxymethyltransferas	0.00E+00	5.38E+06	5.38E+06	5.38E+06	NULL	AS	P34897	SHMT2
151.1	Electron transfer flavoprotein su	0.00E+00	5.45E+06	5.45E+06	5.45E+06	NULL	AS	P13804	ETFA
316.1	Protein RCC2	0.00E+00	5.46E+06	5.46E+06	5.46E+06	NULL	AS	Q9P258	RCC2
364.1	60S ribosomal protein L13a	0.00E+00	5.47E+06	5.47E+06	5.47E+06	NULL	AS	P40429	RPL13A
288.1	Isoform 4 of Double-stranded RI	0.00E+00	5.48E+06	5.48E+06	5.48E+06	NULL	AS	P55265-4	ADAR
154.1	Phenylalanine--tRNA ligase beta	0.00E+00	5.53E+06	5.53E+06	5.53E+06	NULL	AS	Q9NSD9	FARSB

249.1	Coiled-coil domain-containing p	0.00E+00	5.60E+06	5.60E+06	5.60E+06	NULL	AS	Q96CT7	CCDC124
226.1	Isoform 2 of Cleavage and polya	0.00E+00	5.65E+06	5.65E+06	5.65E+06	NULL	AS	Q16630-2	CPSF6
588.1	Isoform 5 of RNA-binding protei	0.00E+00	5.75E+06	5.75E+06	5.75E+06	NULL	AS	Q01844-5	EWSR1
162.1	60S ribosomal protein L4	0.00E+00	6.22E+06	6.22E+06	6.22E+06	NULL	AS	P36578	RPL4
171.1	Bcl-2-associated transcription fa	0.00E+00	6.38E+06	6.38E+06	6.38E+06	NULL	AS	Q9NYF8	BCLAF1
159.1	Protein transport protein Sec24.	0.00E+00	6.51E+06	6.51E+06	6.51E+06	NULL	AS	O95486	SEC24A
334.1	Glutathione reductase, mitocho	0.00E+00	6.55E+06	6.55E+06	6.55E+06	NULL	AS	P00390	GSR
320.1	40S ribosomal protein S16	0.00E+00	6.56E+06	6.56E+06	6.56E+06	NULL	AS	P62249	RPS16
213.1	Protein RPSAP58	0.00E+00	6.66E+06	6.66E+06	6.66E+06	NULL	AS	A6NE09	RPSAP58
936.1	Interleukin enhancer-binding fa	0.00E+00	6.70E+06	6.70E+06	6.70E+06	NULL	AS	Q12905	ILF2
285.1	Endothelial differentiation-relat	0.00E+00	7.09E+06	7.09E+06	7.09E+06	NULL	AS	O60869	EDF1
203.1	Isoform 7 of Fragile X mental ret	0.00E+00	7.16E+06	7.16E+06	7.16E+06	NULL	AS	Q06787-7	FMR1
144.1	60S ribosomal protein L6	0.00E+00	7.28E+06	7.28E+06	7.28E+06	NULL	AS	Q02878	RPL6
282.1	40S ribosomal protein S21	0.00E+00	7.38E+06	7.38E+06	7.38E+06	NULL	AS	P63220	RPS21
499.1	Protein NipSnap homolog 1	0.00E+00	7.39E+06	7.39E+06	7.39E+06	NULL	AS	Q9BPW8	NIPSNAP1
240.1	60S ribosomal protein L27	0.00E+00	7.95E+06	7.95E+06	7.95E+06	NULL	AS	P61353	RPL27
86.1	ATP-dependent RNA helicase DI	0.00E+00	8.39E+06	8.39E+06	8.39E+06	NULL	AS	Q92499	DDX1
256.1	Isoform 5 of Ubiquitin-associate	0.00E+00	8.67E+06	8.67E+06	8.67E+06	NULL	AS	Q14157-5	UBAP2L
301.1	Phenylalanine--tRNA ligase alph	0.00E+00	8.72E+06	8.72E+06	8.72E+06	NULL	AS	K7ER00	FARSA
207.1	60S ribosomal protein L8	0.00E+00	9.83E+06	9.83E+06	9.83E+06	NULL	AS	P62917	RPL8
180.1	60S ribosomal protein L24	0.00E+00	9.89E+06	9.89E+06	9.89E+06	NULL	AS	P83731	RPL24
156.1	Cold-inducible RNA-binding prot	0.00E+00	1.01E+07	1.01E+07	1.01E+07	NULL	AS	Q14011	CIRBP
387.1	60S ribosomal protein L32	0.00E+00	1.04E+07	1.04E+07	1.04E+07	NULL	AS	F8W727	RPL32
48.1	Serine/arginine repetitive matri:	0.00E+00	1.07E+07	1.07E+07	1.07E+07	NULL	AS	Q9UQ35	SRRM2
619.1	Isoform 2 of Protein FAM98B	0.00E+00	1.08E+07	1.08E+07	1.08E+07	NULL	AS	Q52LJ0-2	FAM98B
165.1	60S ribosomal protein L5	0.00E+00	1.09E+07	1.09E+07	1.09E+07	NULL	AS	P46777	RPL5
102.1	Heterogeneous nuclear ribonuc	0.00E+00	1.10E+07	1.10E+07	1.10E+07	NULL	AS	P07910	HNRNPC
209.1	40S ribosomal protein S5	0.00E+00	1.15E+07	1.15E+07	1.15E+07	NULL	AS	P46782	RPS5
575.1	Protein FAM98A	0.00E+00	1.20E+07	1.20E+07	1.20E+07	NULL	AS	Q8NCA5	FAM98A
150.1	40S ribosomal protein S14	0.00E+00	1.30E+07	1.30E+07	1.30E+07	NULL	AS	P62263	RPS14
153.1	Thyroid hormone receptor-asso	0.00E+00	1.42E+07	1.42E+07	1.42E+07	NULL	AS	Q9Y2W1	THRAP3
246.1	Cleavage and polyadenylation s	0.00E+00	1.54E+07	1.54E+07	1.54E+07	NULL	AS	O43809	NUDT21
60.1	RNA-binding protein 14	0.00E+00	1.57E+07	1.57E+07	1.57E+07	NULL	AS	Q96PK6	RBM14
73.1	Paraspeckle component 1	0.00E+00	1.72E+07	1.72E+07	1.72E+07	NULL	AS	Q8WXF1	PSPC1
148.1	Ras GTPase-activating protein-b	0.00E+00	1.83E+07	1.83E+07	1.83E+07	NULL	AS	Q13283	G3BP1

198.1	Fragile X mental retardation syn	0.00E+00	2.12E+07	2.12E+07	2.12E+07	NULL	AS	P51114	FXR1
82.1	Protein transport protein Sec24	0.00E+00	2.53E+07	2.53E+07	2.53E+07	NULL	AS	B7ZKM8	SEC24B
268.1	KH domain-containing, RNA-bin	0.00E+00	2.59E+07	2.59E+07	2.59E+07	NULL	AS	Q07666	KHDRBS1
193.1	40S ribosomal protein S25	0.00E+00	2.70E+07	2.70E+07	2.70E+07	NULL	AS	P62851	RPS25
139.1	60S ribosomal protein L9	0.00E+00	3.13E+07	3.13E+07	3.13E+07	NULL	AS	P32969	RPL9
56.1	Prelamin-A/C	0.00E+00	3.28E+07	3.28E+07	3.28E+07	NULL	AS	P02545	LMNA
45.1	Isoform 4 of YLP motif-containir	0.00E+00	4.07E+07	4.07E+07	4.07E+07	NULL	AS	P49750-4	YLPM1
34.1	Nuclear receptor coactivator 5	0.00E+00	4.37E+07	4.37E+07	4.37E+07	NULL	AS	Q9HCD5	NCOA5
104.1	40S ribosomal protein S10	0.00E+00	6.52E+07	6.52E+07	6.52E+07	NULL	AS	P46783	RPS10
189.1	Isoform 2 of 40S ribosomal prot	0.00E+00	8.27E+07	8.27E+07	8.27E+07	NULL	AS	P60866-2	RPS20
57.1	40S ribosomal protein S19	0.00E+00	1.27E+08	1.27E+08	1.27E+08	NULL	AS	P39019	RPS19
66.1	TATA-binding protein-associate	0.00E+00	1.64E+08	1.64E+08	1.64E+08	NULL	AS	Q92804	TAF15
6.1	Splicing factor, proline- and glut	0.00E+00	2.51E+08	2.51E+08	2.51E+08	NULL	AS	P23246	SFPQ
2985.1	WD repeat domain phosphoino:	4.27E+02	0.00E+00	-4.27E+02	-4.27E+02	NULL	AD	Q5MNZ6	WDR45B
1965.1	NACHT, LRR and PYD domains-c	9.93E+02	0.00E+00	-9.93E+02	-9.93E+02	NULL	AD	Q9NX02	NLRP2
2696.1	Isoform 2 of Small G protein sigı	1.72E+03	0.00E+00	-1.72E+03	-1.72E+03	NULL	AD	O43147-2	SGSM2
1729.1	Probable helicase with zinc finge	1.74E+03	0.00E+00	-1.74E+03	-1.74E+03	NULL	AD	J3QS41	HELZ
1849.1	Methyl-CpG-binding domain prc	1.97E+03	0.00E+00	-1.97E+03	-1.97E+03	NULL	AD	O95983	MBD3
2993.1	Isoform 2 of Tumor suppressor ı	2.10E+03	0.00E+00	-2.10E+03	-2.10E+03	NULL	AD	Q12888-2	TP53BP1
2870.1	Aurora kinase A	2.14E+03	0.00E+00	-2.14E+03	-2.14E+03	NULL	AD	O14965	AURKA
2625.1	Immunoglobulin-like and fibron	2.16E+03	0.00E+00	-2.16E+03	-2.16E+03	NULL	AD	Q86VF2	IGFN1
2243.1	Protein very KIND	2.54E+03	0.00E+00	-2.54E+03	-2.54E+03	NULL	AD	E7ETM1	KNDC1
1447.1	Isoform 2 of Centromere protei	2.57E+03	0.00E+00	-2.57E+03	-2.57E+03	NULL	AD	Q8N0S6-2	CENPL
2467.1	Isoform 4 of Forkhead box prote	2.58E+03	0.00E+00	-2.58E+03	-2.58E+03	NULL	AD	Q08050-3	FOXM1
2184.1	X-linked retinitis pigmentosa GT	2.60E+03	0.00E+00	-2.60E+03	-2.60E+03	NULL	AD	Q96KN7	RPGRIP1
2863.1	Isoform 2A of N-glycosylase/DN	2.66E+03	0.00E+00	-2.66E+03	-2.66E+03	NULL	AD	O15527-4	OGG1
1881.1	RAP1, GTP-GDP dissociation stin	2.70E+03	0.00E+00	-2.70E+03	-2.70E+03	NULL	AD	P52306	RAP1GDS1
2306.1	SLIT-ROBO Rho GTPase-activatir	3.01E+03	0.00E+00	-3.01E+03	-3.01E+03	NULL	AD	O75044	SRGAP2
2896.1	Polymerase I and transcript rele	3.30E+03	0.00E+00	-3.30E+03	-3.30E+03	NULL	AD	Q6NZI2	PTRF
1918.1	3-oxo-5-alpha-steroid 4-dehydr	3.84E+03	0.00E+00	-3.84E+03	-3.84E+03	NULL	AD	F5GXK9	SRD5A1
1859.1	1-phosphatidylinositol 4,5-bisph	4.22E+03	0.00E+00	-4.22E+03	-4.22E+03	NULL	AD	Q01970	PLCB3
2529.1	Protein SSUH2 homolog	4.73E+03	0.00E+00	-4.73E+03	-4.73E+03	NULL	AD	Q9Y2M2	SSUH2
959.1	Small subunit processome comp	4.78E+03	0.00E+00	-4.78E+03	-4.78E+03	NULL	AD	O75691	UTP20
1071.1	Leucine-rich repeat-containing ı	4.84E+03	0.00E+00	-4.84E+03	-4.84E+03	NULL	AD	Q9Y546	LRRC42
1836.1	Protein-arginine deiminase type	5.36E+03	0.00E+00	-5.36E+03	-5.36E+03	NULL	AD	Q9UM07	PADI4

2142.1	Cysteine-rich protein 2-binding p	5.39E+03	0.00E+00	-5.39E+03	-5.39E+03	NULL	AD	Q9H8E8	CSRP2BP
1403.1	Leucine-rich repeat neuronal pr	5.42E+03	0.00E+00	-5.42E+03	-5.42E+03	NULL	AD	Q8WUT4	LRRN4
2800.1	One cut domain family member	5.42E+03	0.00E+00	-5.42E+03	-5.42E+03	NULL	AD	O60422	ONECUT3
1367.1	Bromodomain-containing prote	5.43E+03	0.00E+00	-5.43E+03	-5.43E+03	NULL	AD	Q9H0E9	BRD8
1636.1	Unconventional myosin-VIIa	5.50E+03	0.00E+00	-5.50E+03	-5.50E+03	NULL	AD	Q13402	MYO7A
2726.1	Mitogen-activated protein kinas	5.52E+03	0.00E+00	-5.52E+03	-5.52E+03	NULL	AD	Q9Y6R4	MAP3K4
2428.1	Replication factor C subunit 1	5.54E+03	0.00E+00	-5.54E+03	-5.54E+03	NULL	AD	P35251	RFC1
2914.1	Atrial natriuretic peptide recept	5.54E+03	0.00E+00	-5.54E+03	-5.54E+03	NULL	AD	B0ZBF1	NPR1
1830.1	Tenascin-X	5.78E+03	0.00E+00	-5.78E+03	-5.78E+03	NULL	AD	P22105	TNXB
2612.1	Death-associated protein kinase	5.87E+03	0.00E+00	-5.87E+03	-5.87E+03	NULL	AD	O43293	DAPK3
3098.1	Ubiquitin carboxyl-terminal hyd	5.89E+03	0.00E+00	-5.89E+03	-5.89E+03	NULL	AD	B5MEG5	USP19
2112.1	Coiled-coil domain-containing p	5.95E+03	0.00E+00	-5.95E+03	-5.95E+03	NULL	AD	Q6ZRK6	CCDC73
1825.1	Extracellular calcium-sensing re	5.97E+03	0.00E+00	-5.97E+03	-5.97E+03	NULL	AD	E7ENE0	CASR
2538.1	Isoform 2 of Protein FAM35A	6.16E+03	0.00E+00	-6.16E+03	-6.16E+03	NULL	AD	Q86V20-2	FAM35A
3169.1	Adenylate kinase 8	6.21E+03	0.00E+00	-6.21E+03	-6.21E+03	NULL	AD	Q96MA6	AK8
2573.1	ATPase family AAA domain-cont	6.23E+03	0.00E+00	-6.23E+03	-6.23E+03	NULL	AD	Q6PL18	ATAD2
1080.1	Isoform 3 of Zinc-activated ligan	6.31E+03	0.00E+00	-6.31E+03	-6.31E+03	NULL	AD	Q401N2-3	ZACN
1412.1	Isoform 4 of Protein CBFA2T2	6.40E+03	0.00E+00	-6.40E+03	-6.40E+03	NULL	AD	O43439-4	CBFA2T2
2098.1	Retinoic acid receptor alpha	6.40E+03	0.00E+00	-6.40E+03	-6.40E+03	NULL	AD	A8MUP8	RARA
2267.1	Nucleoporin GLE1	6.50E+03	0.00E+00	-6.50E+03	-6.50E+03	NULL	AD	Q53GS7	GLE1
2856.1	Thioredoxin domain-containing	6.58E+03	0.00E+00	-6.58E+03	-6.58E+03	NULL	AD	Q86VQ3	TXNDC2
1311.1	Calmodulin-binding transcriptio	6.58E+03	0.00E+00	-6.58E+03	-6.58E+03	NULL	AD	O94983	CAMTA2
2441.1	Heat shock protein beta-2	6.60E+03	0.00E+00	-6.60E+03	-6.60E+03	NULL	AD	Q16082	HSPB2
1624.1	Isoform 2 of Semaphorin-4B	6.68E+03	0.00E+00	-6.68E+03	-6.68E+03	NULL	AD	Q9NPR2-2	SEMA4B
2207.1	Isoamyl acetate-hydrolyzing est	6.89E+03	0.00E+00	-6.89E+03	-6.89E+03	NULL	AD	Q2TAA2	IAH1
1035.1	Isoform 2 of Dual specificity mit	6.92E+03	0.00E+00	-6.92E+03	-6.92E+03	NULL	AD	P46734-3	MAP2K3
1997.1	Protein TSSC4	6.92E+03	0.00E+00	-6.92E+03	-6.92E+03	NULL	AD	Q9Y5U2	TSSC4
1063.1	Solute carrier family 12 member	6.97E+03	0.00E+00	-6.97E+03	-6.97E+03	NULL	AD	Q9Y666	SLC12A7
1057.1	Isoform 2 of Gamma-aminobuty	6.98E+03	0.00E+00	-6.98E+03	-6.98E+03	NULL	AD	P28472-2	GABRB3
2402.1	von Willebrand factor D and EG	7.12E+03	0.00E+00	-7.12E+03	-7.12E+03	NULL	AD	Q8N2E2	VWDE
2141.1	RNA polymerase II-associated p	7.35E+03	0.00E+00	-7.35E+03	-7.35E+03	NULL	AD	Q9H6T3	RPAP3
1271.1	NDRG family member 3	7.42E+03	0.00E+00	-7.42E+03	-7.42E+03	NULL	AD	Q5TH30	NDRG3
1865.1	V-set and immunoglobulin doma	7.52E+03	0.00E+00	-7.52E+03	-7.52E+03	NULL	AD	Q8N0Z9	VSIG10
2056.1	Ribosomal protein S6 kinase del	7.65E+03	0.00E+00	-7.65E+03	-7.65E+03	NULL	AD	Q96S38	RPS6KC1
3128.1	Isoform 3 of GRAM domain-con	7.73E+03	0.00E+00	-7.73E+03	-7.73E+03	NULL	AD	Q96HH9-3	GRAMD3

1333.1	Protein sidekick-1	8.15E+03	0.00E+00	-8.15E+03	-8.15E+03	NULL	AD	Q7Z5N4	SDK1
3246.1	Receptor-interacting serine/thre	8.22E+03	0.00E+00	-8.22E+03	-8.22E+03	NULL	AD	Q13546	RIPK1
1260.1	UDP-N-acetylglucosamine trans	8.40E+03	0.00E+00	-8.40E+03	-8.40E+03	NULL	AD	Q96F25	ALG14
2114.1	ELAV (Embryonic lethal, abnorm	8.48E+03	0.00E+00	-8.48E+03	-8.48E+03	NULL	AD	B1AM49	ELAVL2
1743.1	Isoform 3 of Trafficking protein	8.49E+03	0.00E+00	-8.49E+03	-8.49E+03	NULL	AD	P0DI81-3	TRAPPC2
2213.1	DNA-directed RNA polymerase I	8.57E+03	0.00E+00	-8.57E+03	-8.57E+03	NULL	AD	Q9NW08	POLR3B
1727.1	Brefeldin A-inhibited guanine nu	8.65E+03	0.00E+00	-8.65E+03	-8.65E+03	NULL	AD	Q5TH69	ARFGEF3
2677.1	Probable ATP-dependent DNA h	8.65E+03	0.00E+00	-8.65E+03	-8.65E+03	NULL	AD	A2PYH4	HFM1
2614.1	Non-receptor tyrosine-protein k	8.83E+03	0.00E+00	-8.83E+03	-8.83E+03	NULL	AD	Q13470	TNK1
1771.1	Protein TSSC1	8.89E+03	0.00E+00	-8.89E+03	-8.89E+03	NULL	AD	F8WB12	TSSC1
3232.1	DnaJ homolog subfamily C mem	9.32E+03	0.00E+00	-9.32E+03	-9.32E+03	NULL	AD	Q9Y5T4	DNAJC15
169.2	Keratin, type I cytoskeletal 13	9.41E+03	0.00E+00	-9.41E+03	-9.41E+03	NULL	AD	P13646	KRT13
1976.1	Zinc finger protein 331	9.49E+03	0.00E+00	-9.49E+03	-9.49E+03	NULL	AD	Q9NQX6	ZNF331
2480.1	Isoform 5 of Golgin subfamily A	9.51E+03	0.00E+00	-9.51E+03	-9.51E+03	NULL	AD	Q13439-5	GOLGA4
3095.1	Dual specificity protein kinase C	9.59E+03	0.00E+00	-9.59E+03	-9.59E+03	NULL	AD	P49760	CLK2
2745.1	HEAT repeat-containing protein	9.69E+03	0.00E+00	-9.69E+03	-9.69E+03	NULL	AD	A6NES4	MROH2A
2387.1	WD repeat-containing protein 8	9.84E+03	0.00E+00	-9.84E+03	-9.84E+03	NULL	AD	Q6ZMY6	WDR88
2716.1	Isoform 3 of Zinc transporter 10	1.01E+04	0.00E+00	-1.01E+04	-1.01E+04	NULL	AD	Q6XR72-3	SLC30A10
2659.1	Protein PRR5-ARHGAP8	1.02E+04	0.00E+00	-1.02E+04	-1.02E+04	NULL	AD	B1AHC4	ARHGAP8
2487.1	Group 3 secretory phospholipas	1.03E+04	0.00E+00	-1.03E+04	-1.03E+04	NULL	AD	Q9NZ20	PLA2G3
2507.1	Pikachurin	1.06E+04	0.00E+00	-1.06E+04	-1.06E+04	NULL	AD	Q63HQ2	EGFLAM
1423.1	Liprin alpha4	1.14E+04	0.00E+00	-1.14E+04	-1.14E+04	NULL	AD	B1N949	PPFIA4
2048.1	MRG/MORF4L-binding protein	1.15E+04	0.00E+00	-1.15E+04	-1.15E+04	NULL	AD	Q9NV56	MRGBP
1796.1	Leucine-rich repeat-containing p	1.17E+04	0.00E+00	-1.17E+04	-1.17E+04	NULL	AD	Q96NW7	LRRC7
2016.1	Isoform 3 of Probable G-protein	1.21E+04	0.00E+00	-1.21E+04	-1.21E+04	NULL	AD	Q8IZF2-3	GPR116
3106.1	Transcription elongation factor S	1.26E+04	0.00E+00	-1.26E+04	-1.26E+04	NULL	AD	P63272	SUPT4H1
943.2	Isoform 3 of Obscurin	1.27E+04	0.00E+00	-1.27E+04	-1.27E+04	NULL	AD	Q5VST9-3	OBSCN
1984.1	Zinc finger CCCH domain-contai	1.27E+04	0.00E+00	-1.27E+04	-1.27E+04	NULL	AD	Q9UPT8	ZC3H4
2261.1	Protein eyes shut homolog	1.29E+04	0.00E+00	-1.29E+04	-1.29E+04	NULL	AD	Q5T1H1	EYS
1794.1	Nucleosome-remodeling factor	1.30E+04	0.00E+00	-1.30E+04	-1.30E+04	NULL	AD	Q12830	BPTF
1876.1	E3 ubiquitin-protein ligase Praja	1.30E+04	0.00E+00	-1.30E+04	-1.30E+04	NULL	AD	Q8NG27	PJA1
2292.1	Protein FAM193A	1.30E+04	0.00E+00	-1.30E+04	-1.30E+04	NULL	AD	P78312	FAM193A
2956.1	Argininosuccinate synthase	1.30E+04	0.00E+00	-1.30E+04	-1.30E+04	NULL	AD	P00966	ASS1
1121.1	AH receptor-interacting protein	1.31E+04	0.00E+00	-1.31E+04	-1.31E+04	NULL	AD	O00170	AIP
2535.1	Neuronal migration protein dou	1.31E+04	0.00E+00	-1.31E+04	-1.31E+04	NULL	AD	O43602	DCX

1441.1	Free fatty acid receptor 4	1.35E+04	0.00E+00	-1.35E+04	-1.35E+04	NULL	AD	Q5NUL3	FFAR4
1795.1	Centrosomal protein C10orf90	1.35E+04	0.00E+00	-1.35E+04	-1.35E+04	NULL	AD	F5GZL2	C10orf90
2474.1	Zinc finger protein 408	1.35E+04	0.00E+00	-1.35E+04	-1.35E+04	NULL	AD	Q9H9D4	ZNF408
2732.1	Prokineticin-2	1.36E+04	0.00E+00	-1.36E+04	-1.36E+04	NULL	AD	Q9HC23	PROK2
1781.1	Frataxin, mitochondrial	1.36E+04	0.00E+00	-1.36E+04	-1.36E+04	NULL	AD	Q16595	FXN
2379.1	DNA mismatch repair protein M	1.37E+04	0.00E+00	-1.37E+04	-1.37E+04	NULL	AD	P52701	MSH6
2188.1	RRP15-like protein	1.40E+04	0.00E+00	-1.40E+04	-1.40E+04	NULL	AD	Q9Y3B9	RRP15
2468.1	Hypothetical gene supported by	1.42E+04	0.00E+00	-1.42E+04	-1.42E+04	NULL	AD	Q8N5S0	LOC389199
3064.1	NACHT, LRR and PYD domains-c	1.42E+04	0.00E+00	-1.42E+04	-1.42E+04	NULL	AD	Q7RTR0	NLRP9
3120.1	Isoform 2 of Single-strand select	1.44E+04	0.00E+00	-1.44E+04	-1.44E+04	NULL	AD	Q53HV7-2	SMUG1
1858.1	Ribosomal protein S6 kinase alp	1.46E+04	0.00E+00	-1.46E+04	-1.46E+04	NULL	AD	E9PKU3	RPS6KA4
1357.1	Probable tRNA threonylcarbamo	1.48E+04	0.00E+00	-1.48E+04	-1.48E+04	NULL	AD	Q9H4B0	OSGEPL1
1439.1	Solute carrier organic anion trar	1.49E+04	0.00E+00	-1.49E+04	-1.49E+04	NULL	AD	F8W9W8	SLCO2A1
2132.1	PR domain zinc finger protein 2	1.51E+04	0.00E+00	-1.51E+04	-1.51E+04	NULL	AD	Q13029	PRDM2
2571.1	FERM and PDZ domain-containi	1.51E+04	0.00E+00	-1.51E+04	-1.51E+04	NULL	AD	Q5JV73	FRMPD3
3100.1	Serine/threonine-protein kinase	1.51E+04	0.00E+00	-1.51E+04	-1.51E+04	NULL	AD	F5H2J7	ULK1
1694.1	Isoform 2 of Chondroitin sulfate	1.54E+04	0.00E+00	-1.54E+04	-1.54E+04	NULL	AD	Q8N6G5-2	CSGALNACT2
3179.1	Diacylglycerol kinase kappa	1.54E+04	0.00E+00	-1.54E+04	-1.54E+04	NULL	AD	Q5KSL6	DGKK
1030.1	DNA-binding protein RFX7	1.57E+04	0.00E+00	-1.57E+04	-1.57E+04	NULL	AD	Q2KHR2	RFX7
1891.1	Poly [ADP-ribose] polymerase 2	1.58E+04	0.00E+00	-1.58E+04	-1.58E+04	NULL	AD	Q9UGN5	PARP2
1495.1	High affinity cAMP-specific and	1.60E+04	0.00E+00	-1.60E+04	-1.60E+04	NULL	AD	O60658	PDE8A
3224.1	60S ribosome subunit biogenesi	1.65E+04	0.00E+00	-1.65E+04	-1.65E+04	NULL	AD	Q9Y221	NIP7
1783.1	Tektin-2	1.66E+04	0.00E+00	-1.66E+04	-1.66E+04	NULL	AD	Q9UIF3	TEKT2
1438.1	Plastin-1	1.67E+04	0.00E+00	-1.67E+04	-1.67E+04	NULL	AD	Q14651	PLS1
2514.1	CUGBP Elav-like family member	1.68E+04	0.00E+00	-1.68E+04	-1.68E+04	NULL	AD	Q96J87	CELF6
2586.1	Hypoxia-inducible factor 1-alpha	1.68E+04	0.00E+00	-1.68E+04	-1.68E+04	NULL	AD	Q9NWT6	HIF1AN
1068.1	Isoform 4 of Piezo-type mechan	1.69E+04	0.00E+00	-1.69E+04	-1.69E+04	NULL	AD	Q9H5I5-4	PIEZO2
2814.1	Isoform 2 of Adenosine recepto	1.70E+04	0.00E+00	-1.70E+04	-1.70E+04	NULL	AD	P33765-2	ADORA3
13.2	Pyruvate kinase isozymes R/L	1.71E+04	0.00E+00	-1.71E+04	-1.71E+04	NULL	AD	P30613	PKLR
1792.1	Putative olfactory receptor 52A	1.74E+04	0.00E+00	-1.74E+04	-1.74E+04	NULL	AD	A6NMU1	OR52A4
2765.1	Isoform 2 of Tyrosine-protein ph	1.74E+04	0.00E+00	-1.74E+04	-1.74E+04	NULL	AD	P78324-2	SIRPA
3124.1	Zinc finger protein 560	1.74E+04	0.00E+00	-1.74E+04	-1.74E+04	NULL	AD	Q96MR9	ZNF560
2304.1	Phosphatase and actin regulato	1.77E+04	0.00E+00	-1.77E+04	-1.77E+04	NULL	AD	Q96KR7	PHACTR3
1543.1	DDB1- and CUL4-associated fact	1.78E+04	0.00E+00	-1.78E+04	-1.78E+04	NULL	AD	P61962	DCAF7
1982.1	Receptor-type tyrosine-protein	1.79E+04	0.00E+00	-1.79E+04	-1.79E+04	NULL	AD	Q16827	PTPRO

3130.1	Coiled-coil domain-containing p	1.79E+04	0.00E+00	-1.79E+04	-1.79E+04	NULL	AD	C9JV39	CCDC102B
1547.1	E3 ubiquitin-protein ligase HACE	1.82E+04	0.00E+00	-1.82E+04	-1.82E+04	NULL	AD	Q8IYU2	HACE1
1571.1	Isoform 7 of LETM1 domain-con	1.82E+04	0.00E+00	-1.82E+04	-1.82E+04	NULL	AD	Q6P1Q0-7	LETMD1
1649.1	Methylcytosine dioxygenase TE	1.82E+04	0.00E+00	-1.82E+04	-1.82E+04	NULL	AD	E7EQS8	TET2
2187.1	Neuropeptide W	1.82E+04	0.00E+00	-1.82E+04	-1.82E+04	NULL	AD	Q8N729	NPW
1007.1	Serine/threonine-protein kinase	1.83E+04	0.00E+00	-1.83E+04	-1.83E+04	NULL	AD	Q8TDX7	NEK7
3040.1	LIM domain-containing protein .	1.84E+04	0.00E+00	-1.84E+04	-1.84E+04	NULL	AD	Q96IF1	AJUBA
2855.1	Zinc fingers and homeoboxes pr	1.85E+04	0.00E+00	-1.85E+04	-1.85E+04	NULL	AD	E5RGB8	ZHX1
2365.1	UDP-N-acetylglucosamine--pept	1.86E+04	0.00E+00	-1.86E+04	-1.86E+04	NULL	AD	O15294	OGT
2598.1	Kinesin-like protein KIF22	1.88E+04	0.00E+00	-1.88E+04	-1.88E+04	NULL	AD	Q14807	KIF22
769.1	BRO1 domain-containing protei	1.90E+04	0.00E+00	-1.90E+04	-1.90E+04	NULL	AD	Q5VW32	BROX
1390.1	Serine/threonine-protein kinase	1.92E+04	0.00E+00	-1.92E+04	-1.92E+04	NULL	AD	Q8TDR2	STK35
2014.1	Mediator of RNA polymerase II t	1.94E+04	0.00E+00	-1.94E+04	-1.94E+04	NULL	AD	Q96HR3	MED30
2331.1	Isoform 2 of Peroxisome biogen	2.00E+04	0.00E+00	-2.00E+04	-2.00E+04	NULL	AD	O60683-2	PEX10
2005.1	Pumilio homolog 1	2.04E+04	0.00E+00	-2.04E+04	-2.04E+04	NULL	AD	Q5T1Z8	PUM1
1314.1	5'-3' exoribonuclease 2	2.05E+04	0.00E+00	-2.05E+04	-2.05E+04	NULL	AD	Q9H0D6	XRN2
2359.1	MAP3K12-binding inhibitory prc	2.06E+04	0.00E+00	-2.06E+04	-2.06E+04	NULL	AD	Q9NS73	MBIP
1923.1	Zinc finger protein 174	2.08E+04	0.00E+00	-2.08E+04	-2.08E+04	NULL	AD	Q15697	ZNF174
2045.1	Myelin-associated glycoprotein	2.09E+04	0.00E+00	-2.09E+04	-2.09E+04	NULL	AD	P20916	MAG
2065.1	cAMP-dependent protein kinase	2.11E+04	0.00E+00	-2.11E+04	-2.11E+04	NULL	AD	P17612	PRKACA
2463.1	Protein MMS22-like	2.11E+04	0.00E+00	-2.11E+04	-2.11E+04	NULL	AD	Q6ZRQ5	MMS22L
2249.1	Protein mago nashi homolog 2	2.13E+04	0.00E+00	-2.13E+04	-2.13E+04	NULL	AD	Q96A72	MAGOHB
1039.1	Isoform 2 of Transcription elong	2.18E+04	0.00E+00	-2.18E+04	-2.18E+04	NULL	AD	Q96EI5-2	TCEAL4
771.1	Target of rapamycin complex 2 s	2.19E+04	0.00E+00	-2.19E+04	-2.19E+04	NULL	AD	Q9BPZ7	MAPKAP1
1590.1	Mediator of RNA polymerase II t	2.19E+04	0.00E+00	-2.19E+04	-2.19E+04	NULL	AD	Q15648	MED1
1156.1	Caprin-2	2.20E+04	0.00E+00	-2.20E+04	-2.20E+04	NULL	AD	Q6IMN6	CAPRIN2
772.1	7SK snRNA methylphosphate ca	2.21E+04	0.00E+00	-2.21E+04	-2.21E+04	NULL	AD	Q7L2J0	MEPCE
2404.1	Nuclear receptor coactivator 2	2.24E+04	0.00E+00	-2.24E+04	-2.24E+04	NULL	AD	Q15596	NCOA2
1523.1	Isoform 4 of Sperm flagellar pro	2.27E+04	0.00E+00	-2.27E+04	-2.27E+04	NULL	AD	Q9C093-4	SPEF2
3117.1	Partitioning defective 3 homolo	2.27E+04	0.00E+00	-2.27E+04	-2.27E+04	NULL	AD	Q8TEW8	PARD3B
1791.1	Rho guanine nucleotide exchang	2.32E+04	0.00E+00	-2.32E+04	-2.32E+04	NULL	AD	A1IGU5	ARHGEF37
1625.1	Spermatogenesis-associated prc	2.33E+04	0.00E+00	-2.33E+04	-2.33E+04	NULL	AD	Q7Z572	SPATA21
1516.1	Isoform 2 of Nuclear protein loc	2.34E+04	0.00E+00	-2.34E+04	-2.34E+04	NULL	AD	Q8TAT6-2	NPLOC4
3152.1	Probable G-protein coupled rec	2.36E+04	0.00E+00	-2.36E+04	-2.36E+04	NULL	AD	Q8IZ08	GPR135
876.1	Protein strawberry notch homol	2.37E+04	0.00E+00	-2.37E+04	-2.37E+04	NULL	AD	A3KN83	SBNO1

2025.1	Interferon regulatory factor 8	2.38E+04	0.00E+00	-2.38E+04	-2.38E+04	NULL	AD	Q02556	IRF8
2886.1	Isoform 5 of cAMP-specific 3',5'-	2.40E+04	0.00E+00	-2.40E+04	-2.40E+04	NULL	AD	Q08499-6	PDE4D
1736.1	N-acyl-phosphatidylethanolamin	2.41E+04	0.00E+00	-2.41E+04	-2.41E+04	NULL	AD	J3KR15	NAPEPLD
1785.1	Isoform 3 of Sodium channel pro	2.41E+04	0.00E+00	-2.41E+04	-2.41E+04	NULL	AD	Q9NY46-3	SCN3A
1981.1	Beta-adducin	2.41E+04	0.00E+00	-2.41E+04	-2.41E+04	NULL	AD	P35612	ADD2
1742.1	Beta-1,4-galactosyltransferase 3	2.43E+04	0.00E+00	-2.43E+04	-2.43E+04	NULL	AD	O60512	B4GALT3
168.2	Isoform 2 of Nucleoside diphosp	2.44E+04	0.00E+00	-2.44E+04	-2.44E+04	NULL	AD	P15531-2	NME1
1550.1	Paired box protein Pax-1	2.44E+04	0.00E+00	-2.44E+04	-2.44E+04	NULL	AD	P15863	PAX1
2305.1	Vacuolar protein sorting-associa	2.47E+04	0.00E+00	-2.47E+04	-2.47E+04	NULL	AD	Q96RL7	VPS13A
2122.1	Tubulin polyglutamylase comple	2.51E+04	0.00E+00	-2.51E+04	-2.51E+04	NULL	AD	Q68CL5	TPGS2
1385.1	Cytoplasmic tRNA 2-thiolation p	2.54E+04	0.00E+00	-2.54E+04	-2.54E+04	NULL	AD	Q7Z7A3	CTU1
2925.1	Selenide, water dikinase 2	2.54E+04	0.00E+00	-2.54E+04	-2.54E+04	NULL	AD	J3KR58	SEPHS2
2665.1	Indoleamine 2,3-dioxygenase 1	2.57E+04	0.00E+00	-2.57E+04	-2.57E+04	NULL	AD	P14902	IDO1
1050.1	Protein unc-45 homolog A	2.59E+04	0.00E+00	-2.59E+04	-2.59E+04	NULL	AD	Q9H3U1	UNC45A
1496.1	Polymerase delta-interacting pro	2.62E+04	0.00E+00	-2.62E+04	-2.62E+04	NULL	AD	Q9BY77	POLDIP3
1703.1	Isoform 7 of Calcium/calmodulin	2.62E+04	0.00E+00	-2.62E+04	-2.62E+04	NULL	AD	Q13554-7	CAMK2B
1975.1	MOSC domain-containing prote	2.62E+04	0.00E+00	-2.62E+04	-2.62E+04	NULL	AD	Q969Z3	MARC2
2148.1	Glycerol kinase	2.62E+04	0.00E+00	-2.62E+04	-2.62E+04	NULL	AD	P32189	GK
2358.1	Neutral cholesterol ester hydrol	2.62E+04	0.00E+00	-2.62E+04	-2.62E+04	NULL	AD	F5H7K4	NCEH1
2648.1	Fukutin	2.62E+04	0.00E+00	-2.62E+04	-2.62E+04	NULL	AD	O75072	FKTN
2811.1	Next to BRCA1 gene 1 protein	2.62E+04	0.00E+00	-2.62E+04	-2.62E+04	NULL	AD	J3KPL5	NBR1
2058.1	EGF-like module-containing muc	2.64E+04	0.00E+00	-2.64E+04	-2.64E+04	NULL	AD	B4DWB8	EMR2
1384.1	Lysosomal protective protein	2.65E+04	0.00E+00	-2.65E+04	-2.65E+04	NULL	AD	P10619	CTSA
1044.1	Ras-related protein Rab-34, isof	2.75E+04	0.00E+00	-2.75E+04	-2.75E+04	NULL	AD	P0DI83	RAB34
1057.2	Isoform 3 of Gamma-aminobuty	2.77E+04	0.00E+00	-2.77E+04	-2.77E+04	NULL	AD	P47870-3	GABRB2
2163.1	TBC1 domain family member 19	2.77E+04	0.00E+00	-2.77E+04	-2.77E+04	NULL	AD	Q8N5T2	TBC1D19
923.1	Thioredoxin-like protein 1	2.82E+04	0.00E+00	-2.82E+04	-2.82E+04	NULL	AD	O43396	TXNL1
2042.1	Dynein heavy chain 6, axonemal	2.82E+04	0.00E+00	-2.82E+04	-2.82E+04	NULL	AD	Q9C0G6	DNAH6
1617.1	Protein arginine N-methyltransf	2.85E+04	0.00E+00	-2.85E+04	-2.85E+04	NULL	AD	O60678	PRMT3
1642.1	Isoform 4 of Fc receptor-like B	2.86E+04	0.00E+00	-2.86E+04	-2.86E+04	NULL	AD	Q6BAA4-4	FCRLB
1681.1	Poly(A) polymerase gamma	2.88E+04	0.00E+00	-2.88E+04	-2.88E+04	NULL	AD	Q9BWT3	PAPOLG
3109.1	Isoform 2 of Zinc finger protein	2.88E+04	0.00E+00	-2.88E+04	-2.88E+04	NULL	AD	Q6ZN55-2	ZNF574
2815.1	Zinc finger protein 821	2.95E+04	0.00E+00	-2.95E+04	-2.95E+04	NULL	AD	O75541	ZNF821
1672.1	Transmembrane emp24 domain	3.00E+04	0.00E+00	-3.00E+04	-3.00E+04	NULL	AD	Q13445	TMED1
2434.1	Ras-related protein R-Ras2	3.00E+04	0.00E+00	-3.00E+04	-3.00E+04	NULL	AD	B7Z5Z2	RRAS2

1128.1	CTTNBP2 N-terminal-like protei	3.02E+04	0.00E+00	-3.02E+04	-3.02E+04	NULL	AD	Q9P2B4	CTTNBP2NL
2034.1	Poly [ADP-ribose] polymerase 1	3.04E+04	0.00E+00	-3.04E+04	-3.04E+04	NULL	AD	Q460N5	PARP14
2580.1	Ataxin-7-like protein 2	3.08E+04	0.00E+00	-3.08E+04	-3.08E+04	NULL	AD	Q5T6C5	ATXN7L2
2831.1	Isoform 3 of Solute carrier famil	3.10E+04	0.00E+00	-3.10E+04	-3.10E+04	NULL	AD	O76082-3	SLC22A5
2616.1	Ras-related protein Rab-39B	3.25E+04	0.00E+00	-3.25E+04	-3.25E+04	NULL	AD	Q96DA2	RAB39B
2652.1	Zinc fingers and homeoboxes pr	3.25E+04	0.00E+00	-3.25E+04	-3.25E+04	NULL	AD	Q9UKY1	ZHX1
2986.1	Perilipin-1	3.29E+04	0.00E+00	-3.29E+04	-3.29E+04	NULL	AD	O60240	PLIN1
2417.1	Isoform 2 of Phosphatidylserine	3.31E+04	0.00E+00	-3.31E+04	-3.31E+04	NULL	AD	Q9UG56-2	PISD
956.1	Small glutamine-rich tetratricop	3.33E+04	0.00E+00	-3.33E+04	-3.33E+04	NULL	AD	O43765	SGTA
955.1	Cytoplasmic dynein 1 intermedi	3.46E+04	0.00E+00	-3.46E+04	-3.46E+04	NULL	AD	Q13409	DYNC1I2
1463.1	Insulin-like growth factor-bindin	3.54E+04	0.00E+00	-3.54E+04	-3.54E+04	NULL	AD	P18065	IGFBP2
2223.1	Krev interaction trapped protei	3.57E+04	0.00E+00	-3.57E+04	-3.57E+04	NULL	AD	O00522	KRIT1
2208.1	Probable ubiquitin carboxyl-terr	3.60E+04	0.00E+00	-3.60E+04	-3.60E+04	NULL	AD	O00507	USP9Y
2684.1	Isoform BMP1-7 of Bone morph	3.63E+04	0.00E+00	-3.63E+04	-3.63E+04	NULL	AD	P13497-6	BMP1
2237.1	WD repeat-containing protein 9	3.82E+04	0.00E+00	-3.82E+04	-3.82E+04	NULL	AD	F8VUX9	WDR90
1684.1	RB1-inducible coiled-coil protei	3.89E+04	0.00E+00	-3.89E+04	-3.89E+04	NULL	AD	Q8TDY2	RB1CC1
3194.1	NF-X1-type zinc finger protein N	3.96E+04	0.00E+00	-3.96E+04	-3.96E+04	NULL	AD	Q6ZNB6	NFXL1
3234.1	Isoform 4 of Transmembrane pr	3.96E+04	0.00E+00	-3.96E+04	-3.96E+04	NULL	AD	A2VDJ0-5	KIAA0922
2697.1	Steroidogenic factor 1	3.98E+04	0.00E+00	-3.98E+04	-3.98E+04	NULL	AD	Q13285	NR5A1
2895.1	Myosin light chain kinase, smoo	3.99E+04	0.00E+00	-3.99E+04	-3.99E+04	NULL	AD	Q15746	MYLK
3112.1	F-box only protein 40	3.99E+04	0.00E+00	-3.99E+04	-3.99E+04	NULL	AD	Q9UH90	FBXO40
2635.1	Vasoactive intestinal polypeptid	4.02E+04	0.00E+00	-4.02E+04	-4.02E+04	NULL	AD	P32241	VIPR1
2118.1	Isoform A of Pituitary-specific p	4.04E+04	0.00E+00	-4.04E+04	-4.04E+04	NULL	AD	P28069-2	POU1F1
2121.1	DnaJ homolog subfamily C mem	4.05E+04	0.00E+00	-4.05E+04	-4.05E+04	NULL	AD	Q99543	DNAJC2
1964.1	SLC5A2 protein	4.06E+04	0.00E+00	-4.06E+04	-4.06E+04	NULL	AD	A2RRD2	SLC5A2
2062.1	Spectrin alpha chain, erythrocyt	4.13E+04	0.00E+00	-4.13E+04	-4.13E+04	NULL	AD	P02549	SPTA1
2012.1	Complement C1r subcomponen	4.16E+04	0.00E+00	-4.16E+04	-4.16E+04	NULL	AD	F5H2Z5	C1RL
2050.1	Zinc finger protein 638	4.19E+04	0.00E+00	-4.19E+04	-4.19E+04	NULL	AD	Q14966	ZNF638
2227.1	Interferon alpha/beta receptor	4.23E+04	0.00E+00	-4.23E+04	-4.23E+04	NULL	AD	P17181	IFNAR1
2407.1	Synaptonemal complex protein	4.24E+04	0.00E+00	-4.24E+04	-4.24E+04	NULL	AD	Q9BX26	SYCP2
2527.1	Nitrogen permease regulator 2-	4.25E+04	0.00E+00	-4.25E+04	-4.25E+04	NULL	AD	Q8WTW4	NPRL2
2421.1	UV-stimulated scaffold protein	4.28E+04	0.00E+00	-4.28E+04	-4.28E+04	NULL	AD	Q2YD98	UVSSA
529.2	Chromodomain-helicase-DNA-b	4.34E+04	0.00E+00	-4.34E+04	-4.34E+04	NULL	AD	Q8TDI0	CHD5
2989.1	Ras-related protein Rab-3C	4.34E+04	0.00E+00	-4.34E+04	-4.34E+04	NULL	AD	Q96E17	RAB3C
2522.1	Isoform 2 of Scavenger recepto	4.39E+04	0.00E+00	-4.39E+04	-4.39E+04	NULL	AD	Q86VB7-2	CD163

1108.1	Pyridoxal kinase	4.45E+04	0.00E+00	-4.45E+04	-4.45E+04	NULL	AD	O00764	PDXK
1722.1	Dual oxidase maturation factor :	4.45E+04	0.00E+00	-4.45E+04	-4.45E+04	NULL	AD	Q1HG44	DUOXA2
3185.1	EPS8L2 protein	4.45E+04	0.00E+00	-4.45E+04	-4.45E+04	NULL	AD	B7ZKL3	EPS8L2
2606.1	Nucleolar protein 8	4.46E+04	0.00E+00	-4.46E+04	-4.46E+04	NULL	AD	Q76FK4	NOL8
1733.1	Thioredoxin domain-containing	4.47E+04	0.00E+00	-4.47E+04	-4.47E+04	NULL	AD	O95881	TXNDC12
2789.1	Transient receptor potential cat	4.49E+04	0.00E+00	-4.49E+04	-4.49E+04	NULL	AD	Q9H1D0	TRPV6
1133.1	Nuclear factor of-activated T-ce	4.49E+04	0.00E+00	-4.49E+04	-4.49E+04	NULL	AD	O94916	NFAT5
2687.1	Isoform 3 of Solute carrier famil	4.50E+04	0.00E+00	-4.50E+04	-4.50E+04	NULL	AD	Q5BKX6-3	SLC45A4
3089.1	Isoform 2 of Protein FAM198B	4.52E+04	0.00E+00	-4.52E+04	-4.52E+04	NULL	AD	Q6UWH4-:	FAM198B
2562.1	Isoform 2 of Zinc transporter ZIF	4.53E+04	0.00E+00	-4.53E+04	-4.53E+04	NULL	AD	Q9BRY0-2	SLC39A3
1353.1	26S proteasome non-ATPase reg	4.58E+04	0.00E+00	-4.58E+04	-4.58E+04	NULL	AD	P48556	PSMD8
2911.1	Conserved oligomeric Golgi com	4.64E+04	0.00E+00	-4.64E+04	-4.64E+04	NULL	AD	P83436	COG7
2397.1	Putative upstream-binding facto	4.73E+04	0.00E+00	-4.73E+04	-4.73E+04	NULL	AD	P0CB48	UBTFL6
2517.1	Leucine-rich repeat serine/threo	4.90E+04	0.00E+00	-4.90E+04	-4.90E+04	NULL	AD	Q38SD2	LRRK1
2788.1	Isoform 3 of Centrosomal protei	4.97E+04	0.00E+00	-4.97E+04	-4.97E+04	NULL	AD	Q9C0D2-3	KIAA1731
1152.1	Isoleucine--tRNA ligase, mitocho	4.99E+04	0.00E+00	-4.99E+04	-4.99E+04	NULL	AD	Q9NSE4	IARS2
2597.1	Oxysterol-binding protein-relate	5.00E+04	0.00E+00	-5.00E+04	-5.00E+04	NULL	AD	Q9H0X9	OSBPL5
2670.1	Zinc finger protein 385D	5.07E+04	0.00E+00	-5.07E+04	-5.07E+04	NULL	AD	Q9H6B1	ZNF385D
3176.1	Origin recognition complex subu	5.16E+04	0.00E+00	-5.16E+04	-5.16E+04	NULL	AD	Q9Y5N6	ORC6
870.1	BAG family molecular chaperone	5.17E+04	0.00E+00	-5.17E+04	-5.17E+04	NULL	AD	O95817	BAG3
1778.1	Coiled-coil domain-containing p	5.18E+04	0.00E+00	-5.18E+04	-5.18E+04	NULL	AD	Q9H6F5	CCDC86
2192.1	E3 ubiquitin-protein ligase HECT	5.20E+04	0.00E+00	-5.20E+04	-5.20E+04	NULL	AD	Q9ULT8	HECTD1
2739.1	Ectonucleoside triphosphate dip	5.20E+04	0.00E+00	-5.20E+04	-5.20E+04	NULL	AD	O75356	ENTPD5
2771.1	E3 ubiquitin-protein ligase TRIM	5.21E+04	0.00E+00	-5.21E+04	-5.21E+04	NULL	AD	Q9BRZ2	TRIM56
2676.1	Sodium-dependent multivitamin	5.31E+04	0.00E+00	-5.31E+04	-5.31E+04	NULL	AD	Q9Y289	SLC5A6
1893.1	Isoform 2 of SKI family transcrip	5.37E+04	0.00E+00	-5.37E+04	-5.37E+04	NULL	AD	P84550-2	SKOR1
1962.1	Zinc finger protein ZFPM1	5.37E+04	0.00E+00	-5.37E+04	-5.37E+04	NULL	AD	Q8IX07	ZFPM1
1268.1	Armadillo repeat-containing pro	5.45E+04	0.00E+00	-5.45E+04	-5.45E+04	NULL	AD	Q6NXE6	ARMC6
3021.1	Bestrophin-1	5.47E+04	0.00E+00	-5.47E+04	-5.47E+04	NULL	AD	B7Z1N8	BEST1
3159.1	NACHT, LRR and PYD domains-c	5.48E+04	0.00E+00	-5.48E+04	-5.48E+04	NULL	AD	Q9C000	NLRP1
755.1	DCC-interacting protein 13-alph	5.50E+04	0.00E+00	-5.50E+04	-5.50E+04	NULL	AD	Q9UKG1	APPL1
3216.1	POM121-like protein 2	5.55E+04	0.00E+00	-5.55E+04	-5.55E+04	NULL	AD	C9J1I7	POM121L2
2946.1	Isoform 2 of Dolichol-phosphate	5.56E+04	0.00E+00	-5.56E+04	-5.56E+04	NULL	AD	Q9P2X0-2	DPM3
782.1	Interferon regulatory factor 6	5.60E+04	0.00E+00	-5.60E+04	-5.60E+04	NULL	AD	O14896	IRF6
1654.1	Vesicle-trafficking protein SEC22	5.61E+04	0.00E+00	-5.61E+04	-5.61E+04	NULL	AD	Q96IW7	SEC22A

1023.1	Isoform 7 of T-cell surface glyco	5.62E+04	0.00E+00	-5.62E+04	-5.62E+04	NULL	AD	P10966-8	CD8B
1643.1	Ras-related protein Rab-6B	5.68E+04	0.00E+00	-5.68E+04	-5.68E+04	NULL	AD	Q9NRW1	RAB6B
3164.1	Mitogen-activated protein kinas	5.68E+04	0.00E+00	-5.68E+04	-5.68E+04	NULL	AD	Q5TCX8	MLK4
1521.1	Serine--tRNA ligase, cytoplasmic	5.75E+04	0.00E+00	-5.75E+04	-5.75E+04	NULL	AD	Q5T5C7	SARS
1158.1	WD repeat-containing protein m	5.77E+04	0.00E+00	-5.77E+04	-5.77E+04	NULL	AD	Q9NXC5	MIOS
2451.1	Bicaudal D-related protein 1	5.77E+04	0.00E+00	-5.77E+04	-5.77E+04	NULL	AD	Q6ZP65	CCDC64
2532.1	Isoform 4 of Phosphatidylinosito	5.77E+04	0.00E+00	-5.77E+04	-5.77E+04	NULL	AD	P27986-4	PIK3R1
2436.1	CUGBP Elav-like family member	5.81E+04	0.00E+00	-5.81E+04	-5.81E+04	NULL	AD	E9PC62	CELF2
1397.1	Neurofilament heavy polypeptic	5.85E+04	0.00E+00	-5.85E+04	-5.85E+04	NULL	AD	P12036	NEFH
1202.1	Ubinuclein-1	5.86E+04	0.00E+00	-5.86E+04	-5.86E+04	NULL	AD	Q9NPG3	UBN1
2502.1	Interstitial collagenase	5.88E+04	0.00E+00	-5.88E+04	-5.88E+04	NULL	AD	P03956	MMP1
2475.1	TRAF3-interacting protein 1	5.90E+04	0.00E+00	-5.90E+04	-5.90E+04	NULL	AD	A8MTK4	TRAF3IP1
3180.1	Isoform 2 of Telomerase-binding	5.94E+04	0.00E+00	-5.94E+04	-5.94E+04	NULL	AD	Q86US8-2	SMG6
1817.1	Protein FAM53C	6.01E+04	0.00E+00	-6.01E+04	-6.01E+04	NULL	AD	D6RE00	FAM53C
3119.1	Nuclear pore complex protein N	6.02E+04	0.00E+00	-6.02E+04	-6.02E+04	NULL	AD	F5H5C2	NUP133
3239.1	Hexokinase-2	6.03E+04	0.00E+00	-6.03E+04	-6.03E+04	NULL	AD	P52789	HK2
1969.1	Unconventional myosin-Ig	6.04E+04	0.00E+00	-6.04E+04	-6.04E+04	NULL	AD	B0I1T2	MYO1G
1900.1	Isoform 2 of Choriogonadotropi	6.06E+04	0.00E+00	-6.06E+04	-6.06E+04	NULL	AD	Q6NT52-2	CGB2
2460.1	Isoform 6 of DENN domain-cont	6.14E+04	0.00E+00	-6.14E+04	-6.14E+04	NULL	AD	Q8TEH3-6	DENND1A
1839.1	Fer-1-like protein 5	6.17E+04	0.00E+00	-6.17E+04	-6.17E+04	NULL	AD	A0AVI2	FER1L5
1090.1	Actin-binding protein anillin	6.21E+04	0.00E+00	-6.21E+04	-6.21E+04	NULL	AD	Q9NQW6	ANLN
1661.1	Leucine-rich repeat-containing p	6.21E+04	0.00E+00	-6.21E+04	-6.21E+04	NULL	AD	Q6F5E8	RLTPR
2234.1	Prolyl 4-hydroxylase subunit alp	6.24E+04	0.00E+00	-6.24E+04	-6.24E+04	NULL	AD	O15460	P4HA2
1752.1	Cytochrome P450 4F11	6.25E+04	0.00E+00	-6.25E+04	-6.25E+04	NULL	AD	Q9HBI6	CYP4F11
2229.1	Tyrosine-protein phosphatase n	6.25E+04	0.00E+00	-6.25E+04	-6.25E+04	NULL	AD	Q06124	PTPN11
3182.1	Tubulin polyglutamylase TTLL13	6.29E+04	0.00E+00	-6.29E+04	-6.29E+04	NULL	AD	A6NNM8	TTLL13
1833.1	Macoilin	6.32E+04	0.00E+00	-6.32E+04	-6.32E+04	NULL	AD	Q8N5G2	TMEM57
1531.1	Prefoldin subunit 2	6.39E+04	0.00E+00	-6.39E+04	-6.39E+04	NULL	AD	Q9UHV9	PFDN2
1931.1	Isoform 2 of Synaptotagmin-like	6.43E+04	0.00E+00	-6.43E+04	-6.43E+04	NULL	AD	Q8TDW5-2	SYTL5
3195.1	Isoform 4 of Inter-alpha-trypsin	6.47E+04	0.00E+00	-6.47E+04	-6.47E+04	NULL	AD	Q86UX2-4	ITIH5
1741.1	5-hydroxytryptamine (Serotonin	6.50E+04	0.00E+00	-6.50E+04	-6.50E+04	NULL	AD	Q5VX04	HTR7
1567.1	von Willebrand factor A domain	6.55E+04	0.00E+00	-6.55E+04	-6.55E+04	NULL	AD	O00534	VWA5A
1691.1	Nuclear receptor subfamily 4 gr	6.67E+04	0.00E+00	-6.67E+04	-6.67E+04	NULL	AD	F5GXF0	NR4A1
2991.1	Translation initiation factor IF-3,	6.71E+04	0.00E+00	-6.71E+04	-6.71E+04	NULL	AD	Q9H2K0	MTIF3
774.1	B-cell CLL/lymphoma 9-like prot	6.75E+04	0.00E+00	-6.75E+04	-6.75E+04	NULL	AD	Q86UU0	BCL9L

1993.1	Potassium voltage-gated channe	6.78E+04	0.00E+00	-6.78E+04	-6.78E+04	NULL	AD	Q96PR1	KCNC2
2435.1	Hexokinase-1	6.78E+04	0.00E+00	-6.78E+04	-6.78E+04	NULL	AD	E7ENR4	HK1
2929.1	Putative homeodomain transcri	6.79E+04	0.00E+00	-6.79E+04	-6.79E+04	NULL	AD	Q9UMS5	PHTF1
2119.1	Phosphatidylinositol 3,4,5-trispl	6.80E+04	0.00E+00	-6.80E+04	-6.80E+04	NULL	AD	P60484	PTEN
2620.1	G-protein-coupled receptor fam	6.81E+04	0.00E+00	-6.81E+04	-6.81E+04	NULL	AD	B7Z831	GPRC5B
2209.1	Zinc finger protein 85	6.83E+04	0.00E+00	-6.83E+04	-6.83E+04	NULL	AD	Q03923	ZNF85
1480.1	PiggyBac transposable element-	6.94E+04	0.00E+00	-6.94E+04	-6.94E+04	NULL	AD	Q96DM1	PGBD4
3244.1	Isoform 2 of Choline transporte	6.94E+04	0.00E+00	-6.94E+04	-6.94E+04	NULL	AD	Q8WWI5-2	SLC44A1
2260.1	E3 ubiquitin-protein ligase RNF6	6.96E+04	0.00E+00	-6.96E+04	-6.96E+04	NULL	AD	Q9Y252	RNF6
1211.1	RING finger protein 150	7.02E+04	0.00E+00	-7.02E+04	-7.02E+04	NULL	AD	Q9ULK6	RNF150
2461.1	Isoform 2 of Signal transducing a	7.02E+04	0.00E+00	-7.02E+04	-7.02E+04	NULL	AD	Q92783-2	STAM
2838.1	Xylosyltransferase 1	7.04E+04	0.00E+00	-7.04E+04	-7.04E+04	NULL	AD	Q86Y38	XYLT1
2978.1	Rab proteins geranylgeranyltrar	7.08E+04	0.00E+00	-7.08E+04	-7.08E+04	NULL	AD	P26374	CHML
1288.1	Piwi-like protein 2	7.10E+04	0.00E+00	-7.10E+04	-7.10E+04	NULL	AD	Q8TC59	PIWIL2
1366.1	Zinc finger protein with KRAB ar	7.10E+04	0.00E+00	-7.10E+04	-7.10E+04	NULL	AD	P17029	ZKSCAN1
2067.1	Dual oxidase 2	7.12E+04	0.00E+00	-7.12E+04	-7.12E+04	NULL	AD	Q9NRD8	DUOX2
1761.1	Prenylcysteine oxidase-like	7.13E+04	0.00E+00	-7.13E+04	-7.13E+04	NULL	AD	Q8NBM8	PCYOX1L
2628.1	Phosphatidylcholine-sterol acylt	7.21E+04	0.00E+00	-7.21E+04	-7.21E+04	NULL	AD	P04180	LCAT
808.1	Tumor protein D53	7.22E+04	0.00E+00	-7.22E+04	-7.22E+04	NULL	AD	J3KNE7	TPD52L1
1968.1	Putative TBC1 domain family me	7.24E+04	0.00E+00	-7.24E+04	-7.24E+04	NULL	AD	Q9UFV1	TBC1D29
2918.1	Acyl-coenzyme A synthetase AC	7.24E+04	0.00E+00	-7.24E+04	-7.24E+04	NULL	AD	Q53FZ2	ACSM3
2176.1	Isoform 12 of Sorbin and SH3 dc	7.26E+04	0.00E+00	-7.26E+04	-7.26E+04	NULL	AD	Q9BX66-12	SORBS1
2603.1	Zinc finger protein 189	7.42E+04	0.00E+00	-7.42E+04	-7.42E+04	NULL	AD	O75820	ZNF189
2821.1	UDP-glucuronosyltransferase 2A	7.46E+04	0.00E+00	-7.46E+04	-7.46E+04	NULL	AD	Q6UWM9	UGT2A3
2017.1	Synaptobrevin homolog YKT6	7.47E+04	0.00E+00	-7.47E+04	-7.47E+04	NULL	AD	O15498	YKT6
1599.1	Syntaxin-11	7.49E+04	0.00E+00	-7.49E+04	-7.49E+04	NULL	AD	O75558	STX11
2626.1	Isoform 2 of Alpha-1,6-mannosy	7.51E+04	0.00E+00	-7.51E+04	-7.51E+04	NULL	AD	Q3V5L5-2	MGAT5B
3209.1	YrdC domain-containing protein	7.51E+04	0.00E+00	-7.51E+04	-7.51E+04	NULL	AD	Q86U90	YRDC
2952.1	NmrA-like family domain-contai	7.52E+04	0.00E+00	-7.52E+04	-7.52E+04	NULL	AD	Q9HBL8	NMRAL1
1186.1	von Willebrand factor A domain	7.66E+04	0.00E+00	-7.66E+04	-7.66E+04	NULL	AD	Q9Y334	VWA7
1810.1	Serine/threonine-protein kinase	7.70E+04	0.00E+00	-7.70E+04	-7.70E+04	NULL	AD	Q9Y3S1	WNK2
3263.1	von Willebrand factor A domain	7.71E+04	0.00E+00	-7.71E+04	-7.71E+04	NULL	AD	Q6PCB0	VWA1
1227.1	Integrin-linked kinase-associate	7.78E+04	0.00E+00	-7.78E+04	-7.78E+04	NULL	AD	Q9H0C8	ILKAP
2865.1	PDZ domain-containing protein	7.84E+04	0.00E+00	-7.84E+04	-7.84E+04	NULL	AD	O15018	PDZD2
2760.1	Methyltransferase-like protein 8	7.88E+04	0.00E+00	-7.88E+04	-7.88E+04	NULL	AD	E7ETE0	METTL8

2449.1	AP-4 complex subunit epsilon-1	7.97E+04	0.00E+00	-7.97E+04	-7.97E+04	NULL	AD	Q9UPM8	AP4E1
3097.1	Roundabout homolog 4	8.05E+04	0.00E+00	-8.05E+04	-8.05E+04	NULL	AD	Q8WZ75	ROBO4
2554.1	Coiled-coil domain-containing p	8.11E+04	0.00E+00	-8.11E+04	-8.11E+04	NULL	AD	A2RUR9	CCDC144A
1417.1	Isoform 2 of Protein FAM76B	8.17E+04	0.00E+00	-8.17E+04	-8.17E+04	NULL	AD	Q5HYJ3-2	FAM76B
1637.1	Ran-binding protein 17	8.19E+04	0.00E+00	-8.19E+04	-8.19E+04	NULL	AD	Q9H2T7	RANBP17
2074.1	GTPase IMAP family member 7	8.23E+04	0.00E+00	-8.23E+04	-8.23E+04	NULL	AD	Q8NHV1	GIMAP7
2575.1	Cingulin	8.24E+04	0.00E+00	-8.24E+04	-8.24E+04	NULL	AD	Q9P2M7	CGN
2794.1	Potassium voltage-gated channe	8.24E+04	0.00E+00	-8.24E+04	-8.24E+04	NULL	AD	Q12809	KCNH2
1699.1	Phostensin	8.29E+04	0.00E+00	-8.29E+04	-8.29E+04	NULL	AD	Q6NYC8	PPP1R18
1955.1	UPF0764 protein C16orf89	8.30E+04	0.00E+00	-8.30E+04	-8.30E+04	NULL	AD	Q6UX73	C16orf89
673.1	Protein FAM168A	8.32E+04	0.00E+00	-8.32E+04	-8.32E+04	NULL	AD	Q92567	FAM168A
1300.1	Interferon-stimulated 20 kDa ex	8.36E+04	0.00E+00	-8.36E+04	-8.36E+04	NULL	AD	Q9H9L3	ISG20L2
2210.1	Gem-associated protein 7	8.40E+04	0.00E+00	-8.40E+04	-8.40E+04	NULL	AD	Q9H840	GEMIN7
3265.1	G protein-regulated inducer of r	8.41E+04	0.00E+00	-8.41E+04	-8.41E+04	NULL	AD	Q7Z2K8	GPRIN1
1424.1	Isoform 3 of LIM domain kinase	8.42E+04	0.00E+00	-8.42E+04	-8.42E+04	NULL	AD	P53671-3	LIMK2
2253.1	Immunoglobulin superfamily me	8.48E+04	0.00E+00	-8.48E+04	-8.48E+04	NULL	AD	Q6WRI0	IGSF10
2284.1	DNA excision repair protein ERC	8.57E+04	0.00E+00	-8.57E+04	-8.57E+04	NULL	AD	Q03468	ERCC6
3031.1	YTH domain-containing protein	8.62E+04	0.00E+00	-8.62E+04	-8.62E+04	NULL	AD	J3QR07	YTHDC1
1690.1	HCG2004619	8.63E+04	0.00E+00	-8.63E+04	-8.63E+04	NULL	AD	H0YNJ1	hCG_2004619
1766.1	TCF3 fusion partner	8.63E+04	0.00E+00	-8.63E+04	-8.63E+04	NULL	AD	A8MTQ3	TFPT
3087.1	Protein FAM219A	8.67E+04	0.00E+00	-8.67E+04	-8.67E+04	NULL	AD	Q8IW50	FAM219A
1633.1	Ubiquitin carboxyl-terminal hyd	8.68E+04	0.00E+00	-8.68E+04	-8.68E+04	NULL	AD	Q9P2H5	USP35
2951.1	HCG1987119, isoform CRA_a	8.68E+04	0.00E+00	-8.68E+04	-8.68E+04	NULL	AD	I3L077	MYO15B
1379.1	Ran-binding protein 3	8.72E+04	0.00E+00	-8.72E+04	-8.72E+04	NULL	AD	K7EJ17	RANBP3
1939.1	Heparan-sulfate 6-O-sulfotransf	8.79E+04	0.00E+00	-8.79E+04	-8.79E+04	NULL	AD	Q8IZP7	HS6ST3
2738.1	Mitochondrial-processing peptic	9.00E+04	0.00E+00	-9.00E+04	-9.00E+04	NULL	AD	O75439	PMPCB
2004.1	Chromodomain-helicase-DNA-b	9.06E+04	0.00E+00	-9.06E+04	-9.06E+04	NULL	AD	Q9P2D1	CHD7
1036.1	Nuclear speckle splicing regulat	9.07E+04	0.00E+00	-9.07E+04	-9.07E+04	NULL	AD	Q9H0G5	NSRP1
1551.1	Relaxin-3 receptor 1	9.13E+04	0.00E+00	-9.13E+04	-9.13E+04	NULL	AD	Q9NSD7	RXFP3
1935.1	Inactive phospholipase C-like pr	9.16E+04	0.00E+00	-9.16E+04	-9.16E+04	NULL	AD	Q15111	PLCL1
1476.1	GMP reductase	9.17E+04	0.00E+00	-9.17E+04	-9.17E+04	NULL	AD	H0YNJ6	GMPR2
3050.1	Plasma membrane calcium-tran	9.19E+04	0.00E+00	-9.19E+04	-9.19E+04	NULL	AD	P20020	ATP2B1
1967.1	RelA-associated inhibitor	9.20E+04	0.00E+00	-9.20E+04	-9.20E+04	NULL	AD	Q8WUF5	PPP1R13L
1275.1	Isoform 8 of C-type lectin domai	9.23E+04	0.00E+00	-9.23E+04	-9.23E+04	NULL	AD	Q9BXN2-8	CLEC7A
2819.1	Serine/threonine-protein kinase	9.24E+04	0.00E+00	-9.24E+04	-9.24E+04	NULL	AD	Q13535	ATR

3073.1	Isoform 3 of Phosphatidylinosit	9.25E+04	0.00E+00	-9.25E+04	-9.25E+04	NULL	AD	O60331-3	PIP5K1C
1293.1	RNA polymerase-associated pro	9.27E+04	0.00E+00	-9.27E+04	-9.27E+04	NULL	AD	Q92541	RTF1
2496.1	Kinesin-like protein KIFC3	9.28E+04	0.00E+00	-9.28E+04	-9.28E+04	NULL	AD	B7Z484	KIFC3
2645.1	Isoform 5 of Glutamate recepto	9.28E+04	0.00E+00	-9.28E+04	-9.28E+04	NULL	AD	Q05586-5	GRIN1
2579.1	Endothelin-2	9.33E+04	0.00E+00	-9.33E+04	-9.33E+04	NULL	AD	P20800	EDN2
2758.1	GAS2-like protein 1	9.35E+04	0.00E+00	-9.35E+04	-9.35E+04	NULL	AD	Q99501	GAS2L1
2615.1	Probable cation-transporting AT	9.37E+04	0.00E+00	-9.37E+04	-9.37E+04	NULL	AD	Q4VNC0	ATP13A5
1692.1	Isoform 5 of Regulator of G-prot	9.39E+04	0.00E+00	-9.39E+04	-9.39E+04	NULL	AD	P49802-5	RGS7
1164.1	Trifunctional enzyme subunit al	9.42E+04	0.00E+00	-9.42E+04	-9.42E+04	NULL	AD	P40939	HADHA
2708.1	Laminin subunit gamma-3	9.42E+04	0.00E+00	-9.42E+04	-9.42E+04	NULL	AD	Q5JTC4	LAMC3
809.1	Omega-amidase NIT2	9.55E+04	0.00E+00	-9.55E+04	-9.55E+04	NULL	AD	Q9NQR4	NIT2
1308.1	Putative GTP-binding protein 6	9.60E+04	0.00E+00	-9.60E+04	-9.60E+04	NULL	AD	O43824	GTPBP6
1046.1	Interferon-related IFRD2 (PC4-B	9.62E+04	0.00E+00	-9.62E+04	-9.62E+04	NULL	AD	Q9UJ88	IFRD2
1797.1	Isoform 3 of Forkhead box prote	9.76E+04	0.00E+00	-9.76E+04	-9.76E+04	NULL	AD	Q9BZS1-3	FOXP3
2380.1	Peroxisomal NADH pyrophosph	9.80E+04	0.00E+00	-9.80E+04	-9.80E+04	NULL	AD	Q9BQG2	NUDT12
1847.1	Glutathione S-transferase omeg	9.83E+04	0.00E+00	-9.83E+04	-9.83E+04	NULL	AD	P78417	GSTO1
1892.1	Mitochondrial uncoupling prote	9.87E+04	0.00E+00	-9.87E+04	-9.87E+04	NULL	AD	P55851	UCP2
2992.1	Protein dopey-2	9.91E+04	0.00E+00	-9.91E+04	-9.91E+04	NULL	AD	Q9Y3R5	DOPEY2
2790.1	Putative Polycomb group protei	9.92E+04	0.00E+00	-9.92E+04	-9.92E+04	NULL	AD	Q8IXJ9	ASXL1
2820.1	Protein RRP5 homolog	9.92E+04	0.00E+00	-9.92E+04	-9.92E+04	NULL	AD	Q14690	PDCD11
2382.1	Gamma-butyrobetaine dioxyger	9.95E+04	0.00E+00	-9.95E+04	-9.95E+04	NULL	AD	O75936	BBOX1
2587.1	Thrombospondin type-1 domair	9.96E+04	0.00E+00	-9.96E+04	-9.96E+04	NULL	AD	Q9NS62	THSD1
1907.1	Pre-mRNA-splicing factor 38A	9.99E+04	0.00E+00	-9.99E+04	-9.99E+04	NULL	AD	Q8NAV1	PRPF38A
569.1	Inositol monophosphatase 1	9.99E+04	0.00E+00	-9.99E+04	-9.99E+04	NULL	AD	P29218	IMPA1
2081.1	HCG2044781	1.01E+05	0.00E+00	-1.01E+05	-1.01E+05	NULL	AD	G3V2F7	TMEM189
1210.1	Isoform 1 of Transmembrane pr	1.02E+05	0.00E+00	-1.02E+05	-1.02E+05	NULL	AD	Q96A57-2	TMEM230
1888.1	Isoform 2 of Endophilin-B2	1.02E+05	0.00E+00	-1.02E+05	-1.02E+05	NULL	AD	Q9NR46-2	SH3GLB2
2770.1	EF-hand domain-containing fam	1.02E+05	0.00E+00	-1.02E+05	-1.02E+05	NULL	AD	Q8N7U6	EFHB
3254.1	PHD finger protein 21B	1.02E+05	0.00E+00	-1.02E+05	-1.02E+05	NULL	AD	B1AHC5	PHF21B
1816.1	Heat shock 27kDa protein family	1.03E+05	0.00E+00	-1.03E+05	-1.03E+05	NULL	AD	Q8N241	HSPB7
2036.1	IgGFc-binding protein	1.03E+05	0.00E+00	-1.03E+05	-1.03E+05	NULL	AD	Q9Y6R7	FCGBP
2083.1	SWI/SNF complex subunit SMAF	1.03E+05	0.00E+00	-1.03E+05	-1.03E+05	NULL	AD	F8VXC8	SMARCC2
2090.1	Ankyrin repeat domain-containi	1.03E+05	0.00E+00	-1.03E+05	-1.03E+05	NULL	AD	Q9BXX2	ANKRD30B
3038.1	Isoform 7 of Serpin A9	1.03E+05	0.00E+00	-1.03E+05	-1.03E+05	NULL	AD	Q86WD7-7	SERPINA9
1028.1	Septin 10, isoform CRA_c	1.04E+05	0.00E+00	-1.04E+05	-1.04E+05	NULL	AD	B5ME97	SEPT10

2639.1	Ribosome production factor 2 h	1.04E+05	0.00E+00	-1.04E+05	-1.04E+05	NULL	AD	Q9H7B2	RPF2
2947.1	Isoform 6 of GTPase-activating p	1.04E+05	0.00E+00	-1.04E+05	-1.04E+05	NULL	AD	Q14C86-6	GAPVD1
3033.1	Ephrin type-A receptor 8	1.04E+05	0.00E+00	-1.04E+05	-1.04E+05	NULL	AD	P29322	EPHA8
1334.1	CTD small phosphatase-like prot	1.05E+05	0.00E+00	-1.05E+05	-1.05E+05	NULL	AD	F8WED2	CTDSPL
1380.1	E3 ubiquitin-protein ligase MAR	1.06E+05	0.00E+00	-1.06E+05	-1.06E+05	NULL	AD	F5H6W4	MARCH7
1683.1	Plasma membrane calcium-tran	1.06E+05	0.00E+00	-1.06E+05	-1.06E+05	NULL	AD	P23634	ATP2B4
3177.1	Probable G-protein coupled rec	1.06E+05	0.00E+00	-1.06E+05	-1.06E+05	NULL	AD	Q15760	GPR19
1934.1	Immunoglobulin-like domain-co	1.07E+05	0.00E+00	-1.07E+05	-1.07E+05	NULL	AD	Q71H61	ILDR2
2215.1	Ig lambda chain V-II region NIG-	1.07E+05	0.00E+00	-1.07E+05	-1.07E+05	NULL	AD	P04209	LV211
2553.1	Isoform 2 of L-amino-acid oxida	1.07E+05	0.00E+00	-1.07E+05	-1.07E+05	NULL	AD	Q96RQ9-2	IL4I1
2780.1	Ubiquitin-conjugating enzyme E	1.07E+05	0.00E+00	-1.07E+05	-1.07E+05	NULL	AD	F8W8F0	UBE2E2
2826.1	Potassium voltage-gated channe	1.07E+05	0.00E+00	-1.07E+05	-1.07E+05	NULL	AD	Q9NS40	KCNH7
970.1	Dipeptidyl peptidase 3	1.08E+05	0.00E+00	-1.08E+05	-1.08E+05	NULL	AD	G3V180	DPP3
1472.1	Supervillin	1.08E+05	0.00E+00	-1.08E+05	-1.08E+05	NULL	AD	O95425	SVIL
2890.1	Vitamin K-dependent protein C	1.08E+05	0.00E+00	-1.08E+05	-1.08E+05	NULL	AD	B4DPQ7	PROC
2104.1	Isoform 2 of Protein SSX2	1.10E+05	0.00E+00	-1.10E+05	-1.10E+05	NULL	AD	Q16385-2	SSX2
2455.1	Probable ribonuclease ZC3H12B	1.10E+05	0.00E+00	-1.10E+05	-1.10E+05	NULL	AD	E9PAJ6	ZC3H12B
2921.1	Isoform 2 of Microspherule prot	1.10E+05	0.00E+00	-1.10E+05	-1.10E+05	NULL	AD	Q96EZ8-2	MCRS1
1269.1	Interleukin-34	1.11E+05	0.00E+00	-1.11E+05	-1.11E+05	NULL	AD	Q6ZMJ4	IL34
1820.1	PAS domain-containing protein	1.11E+05	0.00E+00	-1.11E+05	-1.11E+05	NULL	AD	Q8IV76	PASD1
2250.1	Target of Nesh-SH3	1.11E+05	0.00E+00	-1.11E+05	-1.11E+05	NULL	AD	D3YTG3	ABI3BP
2537.1	NGFI-A-binding protein 1	1.11E+05	0.00E+00	-1.11E+05	-1.11E+05	NULL	AD	F8W8J7	NAB1
2933.1	Kinocilin	1.11E+05	0.00E+00	-1.11E+05	-1.11E+05	NULL	AD	A6PVL3	KNCN
3006.1	Solute carrier family 12 member	1.11E+05	0.00E+00	-1.11E+05	-1.11E+05	NULL	AD	Q9UHW9	SLC12A6
3165.1	Metallo-beta-lactamase domain	1.11E+05	0.00E+00	-1.11E+05	-1.11E+05	NULL	AD	Q68D91	MBLAC2
1054.1	Protein-L-isoaspartate O-methyl	1.12E+05	0.00E+00	-1.12E+05	-1.12E+05	NULL	AD	H7BY58	PCMT1
1603.1	Leucine zipper transcription fact	1.12E+05	0.00E+00	-1.12E+05	-1.12E+05	NULL	AD	F2Z2E6	LZTFL1
1910.1	Putative adenosylhomocysteina	1.12E+05	0.00E+00	-1.12E+05	-1.12E+05	NULL	AD	O43865	AHCYL1
864.1	Zinc finger protein 10	1.13E+05	0.00E+00	-1.13E+05	-1.13E+05	NULL	AD	P21506	ZNF10
1343.1	SCO-spondin	1.13E+05	0.00E+00	-1.13E+05	-1.13E+05	NULL	AD	A2VEC9	SSPO
1822.1	Keratin, type II cuticular Hb2	1.13E+05	0.00E+00	-1.13E+05	-1.13E+05	NULL	AD	Q9NSB4	KRT82
2097.1	Probable cation-transporting AT	1.13E+05	0.00E+00	-1.13E+05	-1.13E+05	NULL	AD	Q9HD20	ATP13A1
2736.1	Isoform 3 of Receptor expressio	1.13E+05	0.00E+00	-1.13E+05	-1.13E+05	NULL	AD	Q9H902-3	REEP1
2982.1	Monocarboxylate transporter 1(	1.13E+05	0.00E+00	-1.13E+05	-1.13E+05	NULL	AD	Q8TF71	SLC16A10
2680.1	Zinc finger MIZ domain-containi	1.14E+05	0.00E+00	-1.14E+05	-1.14E+05	NULL	AD	Q9ULJ6	ZMIZ1

3188.1	Tubulin polyglutamylase TTLL7	1.14E+05	0.00E+00	-1.14E+05	-1.14E+05	NULL	AD	Q6ZT98	TTLL7
1700.1	Tripartite motif-containing prot	1.15E+05	0.00E+00	-1.15E+05	-1.15E+05	NULL	AD	O00635	TRIM38
3042.1	Orexin receptor type 1	1.15E+05	0.00E+00	-1.15E+05	-1.15E+05	NULL	AD	O43613	HCRTR1
1490.1	Arf-GAP domain and FG repeat-	1.15E+05	0.00E+00	-1.15E+05	-1.15E+05	NULL	AD	P52594	AGFG1
922.1	Proteasome activator complex s	1.16E+05	0.00E+00	-1.16E+05	-1.16E+05	NULL	AD	Q9UL46	PSME2
2185.1	Histamine H1 receptor	1.16E+05	0.00E+00	-1.16E+05	-1.16E+05	NULL	AD	P35367	HRH1
1728.1	Isoform 2 of Zinc finger protein	1.17E+05	0.00E+00	-1.17E+05	-1.17E+05	NULL	AD	Q8N988-2	ZNF557
1912.1	ETS-related transcription factor	1.17E+05	0.00E+00	-1.17E+05	-1.17E+05	NULL	AD	Q99607	ELF4
1945.1	DNA nucleotidylexotransferase	1.17E+05	0.00E+00	-1.17E+05	-1.17E+05	NULL	AD	P04053	DNTT
2254.1	Protein OSCP1	1.17E+05	0.00E+00	-1.17E+05	-1.17E+05	NULL	AD	Q8WVF1	OSCP1
1192.1	Isoform 3 of Rap guanine nucleo	1.18E+05	0.00E+00	-1.18E+05	-1.18E+05	NULL	AD	Q13905-3	RAPGEF1
2961.1	F-box-like/WD repeat-containin	1.18E+05	0.00E+00	-1.18E+05	-1.18E+05	NULL	AD	Q9BZK7	TBL1XR1
3250.1	E3 ubiquitin-protein ligase TRIM	1.18E+05	0.00E+00	-1.18E+05	-1.18E+05	NULL	AD	Q86WT6	TRIM69
1001.1	Neurabin-2	1.19E+05	0.00E+00	-1.19E+05	-1.19E+05	NULL	AD	Q96SB3	PPP1R9B
1632.1	Isoform 2 of Presequence prote	1.19E+05	0.00E+00	-1.19E+05	-1.19E+05	NULL	AD	Q5JRX3-2	PITRM1
2409.1	Lysine-specific demethylase 2A	1.19E+05	0.00E+00	-1.19E+05	-1.19E+05	NULL	AD	Q9Y2K7	KDM2A
2776.1	Receptor-type tyrosine-protein	1.20E+05	0.00E+00	-1.20E+05	-1.20E+05	NULL	AD	Q9HD43	PTPRH
3133.1	Polyhomeotic-like protein 1	1.20E+05	0.00E+00	-1.20E+05	-1.20E+05	NULL	AD	P78364	PHC1
668.1	Isoform 2 of S-methyl-5'-thioade	1.21E+05	0.00E+00	-1.21E+05	-1.21E+05	NULL	AD	Q13126-2	MTAP
2617.1	Band 4.1-like protein 4A	1.21E+05	0.00E+00	-1.21E+05	-1.21E+05	NULL	AD	Q9HCS5	EPB41L4A
3055.1	Dual specificity protein phospha	1.21E+05	0.00E+00	-1.21E+05	-1.21E+05	NULL	AD	Q9H1R2	DUSP15
2787.1	Isoform 2 of F-box/WD repeat-c	1.22E+05	0.00E+00	-1.22E+05	-1.22E+05	NULL	AD	Q5XX13-2	FBXW10
1323.1	Endophilin-A2	1.23E+05	0.00E+00	-1.23E+05	-1.23E+05	NULL	AD	Q99961	SH3GL1
1312.1	PC4 and SFRS1-interacting prote	1.24E+05	0.00E+00	-1.24E+05	-1.24E+05	NULL	AD	O75475	PSIP1
1319.1	Sulfhydryl oxidase 1	1.24E+05	0.00E+00	-1.24E+05	-1.24E+05	NULL	AD	O00391	QSOX1
2484.1	Protein-associating with the car	1.24E+05	0.00E+00	-1.24E+05	-1.24E+05	NULL	AD	Q8IZE3	SCYL3
1929.1	Early endosome antigen 1	1.25E+05	0.00E+00	-1.25E+05	-1.25E+05	NULL	AD	Q15075	EEA1
1996.1	Anthrax toxin receptor 1	1.25E+05	0.00E+00	-1.25E+05	-1.25E+05	NULL	AD	Q9H6X2	ANTXR1
1575.1	C5a anaphylatoxin chemotactic	1.26E+05	0.00E+00	-1.26E+05	-1.26E+05	NULL	AD	P21730	C5AR1
2883.1	Homeobox protein Hox-B5	1.26E+05	0.00E+00	-1.26E+05	-1.26E+05	NULL	AD	P09067	HOXB5
3134.1	AF4/FMR2 family member 2	1.26E+05	0.00E+00	-1.26E+05	-1.26E+05	NULL	AD	P51816	AFF2
3202.1	Golgin subfamily A member 2	1.26E+05	0.00E+00	-1.26E+05	-1.26E+05	NULL	AD	Q08379	GOLGA2
812.1	Core histone macro-H2A.1	1.27E+05	0.00E+00	-1.27E+05	-1.27E+05	NULL	AD	O75367	H2AFY
905.1	Zinc finger protein 607	1.27E+05	0.00E+00	-1.27E+05	-1.27E+05	NULL	AD	Q96SK3	ZNF607
1987.1	Isoform 3 of Histone acetyltrans	1.27E+05	0.00E+00	-1.27E+05	-1.27E+05	NULL	AD	Q92993-3	KAT5

2179.1	Kallikrein-14	1.27E+05	0.00E+00	-1.27E+05	-1.27E+05	NULL	AD	Q9P0G3	KLK14
2248.1	Solute carrier family 25 member	1.27E+05	0.00E+00	-1.27E+05	-1.27E+05	NULL	AD	Q6PIV7	SLC25A34
1501.1	AP-3 complex subunit beta-1	1.29E+05	0.00E+00	-1.29E+05	-1.29E+05	NULL	AD	O00203	AP3B1
2717.1	Probable UDP-sugar transporter	1.29E+05	0.00E+00	-1.29E+05	-1.29E+05	NULL	AD	Q9BS91	SLC35A5
2979.1	Isoform 2 of Bromodomain testi	1.29E+05	0.00E+00	-1.29E+05	-1.29E+05	NULL	AD	Q58F21-2	BRDT
3170.1	Protocadherin gamma-A11	1.29E+05	0.00E+00	-1.29E+05	-1.29E+05	NULL	AD	Q9Y5H2	PCDHGA11
2348.1	Cadherin-18	1.30E+05	0.00E+00	-1.30E+05	-1.30E+05	NULL	AD	Q13634	CDH18
1827.1	Cyclin-G-associated kinase	1.31E+05	0.00E+00	-1.31E+05	-1.31E+05	NULL	AD	O14976	GAK
872.1	Serine dehydratase-like	1.32E+05	0.00E+00	-1.32E+05	-1.32E+05	NULL	AD	Q96GA7	SDSL
1285.1	Isoform LCRMP-1 of Dihydropyr	1.32E+05	0.00E+00	-1.32E+05	-1.32E+05	NULL	AD	Q14194-2	CRMP1
1932.1	Olfactory receptor 2S2	1.32E+05	0.00E+00	-1.32E+05	-1.32E+05	NULL	AD	Q9NQN1	OR2S2
3155.1	NRF1 protein	1.32E+05	0.00E+00	-1.32E+05	-1.32E+05	NULL	AD	Q96AN2	NRF1
2592.1	Filamin-A-interacting protein 1	1.33E+05	0.00E+00	-1.33E+05	-1.33E+05	NULL	AD	Q7Z7B0	FILIP1
3253.1	Brain-specific angiogenesis inhib	1.33E+05	0.00E+00	-1.33E+05	-1.33E+05	NULL	AD	Q9UHR4	BAIAP2L1
1281.1	Isoform 3 of Kinesin light chain 3	1.34E+05	0.00E+00	-1.34E+05	-1.34E+05	NULL	AD	Q6P597-3	KLC3
1990.1	Nucleus accumbens-associated	1.35E+05	0.00E+00	-1.35E+05	-1.35E+05	NULL	AD	Q96RE7	NACC1
2405.1	ATP-dependent RNA helicase DI	1.35E+05	0.00E+00	-1.35E+05	-1.35E+05	NULL	AD	Q9GZR7	DDX24
1602.1	Isoform 2 of Deleted in azoospe	1.36E+05	0.00E+00	-1.36E+05	-1.36E+05	NULL	AD	Q92904-2	DAZL
2152.1	Non-specific cytotoxic cell recep	1.36E+05	0.00E+00	-1.36E+05	-1.36E+05	NULL	AD	Q6ZVX7	NCCRP1
2279.1	Nipped-B-like protein	1.36E+05	0.00E+00	-1.36E+05	-1.36E+05	NULL	AD	Q6KC79	NIPBL
2844.1	General transcription factor IIH	1.36E+05	0.00E+00	-1.36E+05	-1.36E+05	NULL	AD	Q6P1K8	GTF2H2C
2924.1	Small membrane A-kinase ancho	1.37E+05	0.00E+00	-1.37E+05	-1.37E+05	NULL	AD	Q9BSF0	C2orf88
807.1	Proteasome subunit beta type-1	1.39E+05	0.00E+00	-1.39E+05	-1.39E+05	NULL	AD	P20618	PSMB1
1200.1	Ankyrin repeat domain-containi	1.39E+05	0.00E+00	-1.39E+05	-1.39E+05	NULL	AD	O75179	ANKRD17
2341.1	Isoform 2 of Inversin	1.39E+05	0.00E+00	-1.39E+05	-1.39E+05	NULL	AD	Q9Y283-2	INVS
2729.1	Serine/threonine-protein kinase	1.39E+05	0.00E+00	-1.39E+05	-1.39E+05	NULL	AD	O94768	STK17B
1787.1	Isoform D of Nuclear RNA expor	1.41E+05	0.00E+00	-1.41E+05	-1.41E+05	NULL	AD	Q9H1B4-4	NXF5
2309.1	Contactin-associated protein 1	1.42E+05	0.00E+00	-1.42E+05	-1.42E+05	NULL	AD	K7EMM9	CNTNAP1
2847.1	Isoform 6 of Myocardial zonula	1.42E+05	0.00E+00	-1.42E+05	-1.42E+05	NULL	AD	P0CAP1-6	MYZAP
3060.1	Zinc finger protein 143	1.43E+05	0.00E+00	-1.43E+05	-1.43E+05	NULL	AD	P52747	ZNF143
1162.1	Netrin receptor DCC	1.44E+05	0.00E+00	-1.44E+05	-1.44E+05	NULL	AD	P43146	DCC
1252.1	Proline and serine-rich protein 2	1.44E+05	0.00E+00	-1.44E+05	-1.44E+05	NULL	AD	Q86WR7	PROSER2
2389.1	Isoform 3 of Phosphatidylinosito	1.44E+05	0.00E+00	-1.44E+05	-1.44E+05	NULL	AD	Q8TBX8-3	PIP4K2C
764.1	CD2-associated protein	1.45E+05	0.00E+00	-1.45E+05	-1.45E+05	NULL	AD	Q9Y5K6	CD2AP
1481.1	Fatty acid desaturase 6	1.45E+05	0.00E+00	-1.45E+05	-1.45E+05	NULL	AD	Q8N9I5	FADS6

1784.1	Ankyrin repeat domain 30A	1.45E+05	0.00E+00	-1.45E+05	-1.45E+05	NULL	AD	Q5W026	ANKRD30A
1867.1	Occludin/ELL domain-containing	1.46E+05	0.00E+00	-1.46E+05	-1.46E+05	NULL	AD	Q9H607	OCEL1
2308.1	Isoform 2 of Splicing factor, sup	1.46E+05	0.00E+00	-1.46E+05	-1.46E+05	NULL	AD	Q12872-2	SFSWAP
2351.1	Gastricsin	1.46E+05	0.00E+00	-1.46E+05	-1.46E+05	NULL	AD	P20142	PGC
1870.1	T-box transcription factor TBX3	1.47E+05	0.00E+00	-1.47E+05	-1.47E+05	NULL	AD	O15119	TBX3
2354.1	Transferrin receptor protein 2	1.47E+05	0.00E+00	-1.47E+05	-1.47E+05	NULL	AD	Q9UP52	TFR2
1232.1	5'-3' exoribonuclease 1	1.48E+05	0.00E+00	-1.48E+05	-1.48E+05	NULL	AD	Q8IZH2	XRN1
574.2	Isoform 2 of Zinc finger CCCH dc	1.49E+05	0.00E+00	-1.49E+05	-1.49E+05	NULL	AD	Q86VM9-2	ZC3H18
1855.1	Ras-GEF domain-containing fam	1.49E+05	0.00E+00	-1.49E+05	-1.49E+05	NULL	AD	Q0VAM2	RASGEF1B
1548.1	Ankyrin repeat domain-containi	1.50E+05	0.00E+00	-1.50E+05	-1.50E+05	NULL	AD	A2A2Z9	ANKRD18B
1572.1	Cullin-1	1.50E+05	0.00E+00	-1.50E+05	-1.50E+05	NULL	AD	Q13616	CUL1
2367.1	Laminin subunit beta-1	1.50E+05	0.00E+00	-1.50E+05	-1.50E+05	NULL	AD	G3XAI2	LAMB1
1327.1	Ankyrin repeat domain-containi	1.51E+05	0.00E+00	-1.51E+05	-1.51E+05	NULL	AD	Q9ULJ7	ANKRD50
2057.1	Protein FAM54A	1.51E+05	0.00E+00	-1.51E+05	-1.51E+05	NULL	AD	Q6P444	MTFR2
2174.1	Formin-1	1.51E+05	0.00E+00	-1.51E+05	-1.51E+05	NULL	AD	Q68DA7	FMN1
1607.1	Serine beta-lactamase-like prote	1.52E+05	0.00E+00	-1.52E+05	-1.52E+05	NULL	AD	P83111	LACTB
1942.1	SNW domain-containing proteir	1.52E+05	0.00E+00	-1.52E+05	-1.52E+05	NULL	AD	G3V4X8	SNW1
2700.1	ARHGEF1 protein	1.52E+05	0.00E+00	-1.52E+05	-1.52E+05	NULL	AD	Q6NX52	ARHGEF1
3013.1	PH domain leucine-rich repeat-c	1.52E+05	0.00E+00	-1.52E+05	-1.52E+05	NULL	AD	H3BMS5	PHLPP2
1355.1	Tripartite motif-containing 2, isc	1.52E+05	0.00E+00	-1.52E+05	-1.52E+05	NULL	AD	Q9C040	TRIM2
1535.1	SH3 domain-containing kinase-b	1.54E+05	0.00E+00	-1.54E+05	-1.54E+05	NULL	AD	Q96B97	SH3KBP1
2240.1	39S ribosomal protein L28, mito	1.54E+05	0.00E+00	-1.54E+05	-1.54E+05	NULL	AD	Q13084	MRPL28
2391.1	Ras-related protein Rab-5A	1.54E+05	0.00E+00	-1.54E+05	-1.54E+05	NULL	AD	P20339	RAB5A
3143.1	Probable E3 ubiquitin-protein lig	1.54E+05	0.00E+00	-1.54E+05	-1.54E+05	NULL	AD	Q5TC82	RC3H1
1442.1	Protein S100-A13	1.55E+05	0.00E+00	-1.55E+05	-1.55E+05	NULL	AD	Q99584	S100A13
2275.1	Lebercilin-like protein	1.56E+05	0.00E+00	-1.56E+05	-1.56E+05	NULL	AD	O95447	LCA5L
2977.1	Isoform 2 of Kin of IRRE-like pro	1.56E+05	0.00E+00	-1.56E+05	-1.56E+05	NULL	AD	Q96J84-2	KIRREL
3217.1	Exophilin-5	1.56E+05	0.00E+00	-1.56E+05	-1.56E+05	NULL	AD	Q8NEV8	EXPH5
1863.1	Ras-related protein Rab-44	1.57E+05	0.00E+00	-1.57E+05	-1.57E+05	NULL	AD	Q7Z6P3	RAB44
2384.1	Phosphatidylinositol 4,5-bisphos	1.57E+05	0.00E+00	-1.57E+05	-1.57E+05	NULL	AD	P42338	PIK3CB
893.1	Serine/arginine repetitive matri	1.58E+05	0.00E+00	-1.58E+05	-1.58E+05	NULL	AD	A7MD48	SRRM4
3003.1	Butyrophilin-like protein 3	1.58E+05	0.00E+00	-1.58E+05	-1.58E+05	NULL	AD	Q6UXE8	BTNL3
2861.1	Spermatogenesis-associated prc	1.60E+05	0.00E+00	-1.60E+05	-1.60E+05	NULL	AD	Q9P0W8	SPATA7
1620.1	5'-AMP-activated protein kinase	1.61E+05	0.00E+00	-1.61E+05	-1.61E+05	NULL	AD	Q9UGJ0	PRKAG2
1634.1	CMP-sialic acid transporter	1.61E+05	0.00E+00	-1.61E+05	-1.61E+05	NULL	AD	P78382	SLC35A1

1730.1	VWFA and cache domain-contai	1.61E+05	0.00E+00	-1.61E+05	-1.61E+05	NULL	AD	Q5VU97	CACHD1
1469.1	Fanconi anemia group D2 protei	1.62E+05	0.00E+00	-1.62E+05	-1.62E+05	NULL	AD	Q9BXW9	FANCD2
2106.1	Brefeldin A-inhibited guanine nu	1.62E+05	0.00E+00	-1.62E+05	-1.62E+05	NULL	AD	Q9Y6D5	ARFGEF2
2761.1	Sushi, nidogen and EGF-like dom	1.62E+05	0.00E+00	-1.62E+05	-1.62E+05	NULL	AD	Q8TER0	SNED1
2110.1	Down syndrome cell adhesion m	1.63E+05	0.00E+00	-1.63E+05	-1.63E+05	NULL	AD	O60469	DSCAM
2457.1	Syntaxin-binding protein 3	1.63E+05	0.00E+00	-1.63E+05	-1.63E+05	NULL	AD	O00186	STXBP3
3088.1	tRNA wybutosine-synthesizing p	1.63E+05	0.00E+00	-1.63E+05	-1.63E+05	NULL	AD	Q6NUM6	TYW1B
3220.1	ER membrane protein complex s	1.63E+05	0.00E+00	-1.63E+05	-1.63E+05	NULL	AD	Q8N766	EMC1
1569.1	Isoform 4 of Protein SMG7	1.64E+05	0.00E+00	-1.64E+05	-1.64E+05	NULL	AD	Q92540-4	SMG7
2032.1	Sodium channel protein type 5 s	1.64E+05	0.00E+00	-1.64E+05	-1.64E+05	NULL	AD	H9KVD2	SCN5A
2339.1	Isoform 3 of MBT domain-conta	1.64E+05	0.00E+00	-1.64E+05	-1.64E+05	NULL	AD	Q05BQ5-3	MBTD1
3074.1	Isoform 3 of Disks large-associat	1.64E+05	0.00E+00	-1.64E+05	-1.64E+05	NULL	AD	Q15398-3	DLGAP5
3.8	Desmin	1.66E+05	0.00E+00	-1.66E+05	-1.66E+05	NULL	AD	P17661	DES
2830.1	Ecotropic viral integration site 5	1.66E+05	0.00E+00	-1.66E+05	-1.66E+05	NULL	AD	F5H4R0	EVI5
1644.1	Phosphatidylinositol 3,4,5-trisph	1.67E+05	0.00E+00	-1.67E+05	-1.67E+05	NULL	AD	Q92835	INPP5D
1494.1	Charged multivesicular body pro	1.68E+05	0.00E+00	-1.68E+05	-1.68E+05	NULL	AD	Q14D22	CHMP4A
2015.1	Cyclin-dependent kinase 15	1.69E+05	0.00E+00	-1.69E+05	-1.69E+05	NULL	AD	Q96Q40	CDK15
2809.1	RCC1 and BTB domain-containir	1.69E+05	0.00E+00	-1.69E+05	-1.69E+05	NULL	AD	B4DWG0	RCBTB2
1723.1	Isoform 2 of E3 ubiquitin-protei	1.70E+05	0.00E+00	-1.70E+05	-1.70E+05	NULL	AD	Q5T4S7-2	UBR4
2265.1	Hamartin	1.70E+05	0.00E+00	-1.70E+05	-1.70E+05	NULL	AD	Q92574	TSC1
2269.1	Melanoma-associated antigen 8	1.70E+05	0.00E+00	-1.70E+05	-1.70E+05	NULL	AD	P43361	MAGEA8
2750.1	Tetratricopeptide repeat protein	1.70E+05	0.00E+00	-1.70E+05	-1.70E+05	NULL	AD	G3V3A5	TTC6
2988.1	KH domain-containing, RNA-bin	1.70E+05	0.00E+00	-1.70E+05	-1.70E+05	NULL	AD	Q5VWX1	KHDRBS2
2241.1	Alpha-2,8-sialyltransferase 8B	1.71E+05	0.00E+00	-1.71E+05	-1.71E+05	NULL	AD	Q92186	ST8SIA2
299.2	ATP-citrate synthase	1.72E+05	0.00E+00	-1.72E+05	-1.72E+05	NULL	AD	B4E3P0	ACLY
1048.1	LDLR chaperone MESD	1.72E+05	0.00E+00	-1.72E+05	-1.72E+05	NULL	AD	Q14696	MESDC2
2599.1	Mis18-binding protein 1	1.72E+05	0.00E+00	-1.72E+05	-1.72E+05	NULL	AD	Q6P0N0	MIS18BP1
2172.1	Copper-transporting ATPase 2	1.73E+05	0.00E+00	-1.73E+05	-1.73E+05	NULL	AD	P35670	ATP7B
2324.1	Membrane-associated guanylate	1.73E+05	0.00E+00	-1.73E+05	-1.73E+05	NULL	AD	Q96QZ7	MAGI1
7.2	Filamin-C	1.74E+05	0.00E+00	-1.74E+05	-1.74E+05	NULL	AD	Q14315	FLNC
2835.1	Protein cramped-like	1.74E+05	0.00E+00	-1.74E+05	-1.74E+05	NULL	AD	J3KR07	CRAMP1L
697.1	Protein arginine N-methyltransf	1.76E+05	0.00E+00	-1.76E+05	-1.76E+05	NULL	AD	O14744	PRMT5
2445.1	Period circadian protein homolo	1.77E+05	0.00E+00	-1.77E+05	-1.77E+05	NULL	AD	O15534	PER1
2733.1	Isoform 4 of DNA helicase MCM	1.77E+05	0.00E+00	-1.77E+05	-1.77E+05	NULL	AD	Q9UJA3-4	MCM8
2841.1	Transmembrane emp24 domain	1.77E+05	0.00E+00	-1.77E+05	-1.77E+05	NULL	AD	Q9Y3A6	TMED5

1803.1	Synaptotagmin-like protein 4	1.78E+05	0.00E+00	-1.78E+05	-1.78E+05	NULL	AD	F8W9B9	SYTL4
1329.1	Ubiquitin carboxyl-terminal hyd	1.79E+05	0.00E+00	-1.79E+05	-1.79E+05	NULL	AD	Q70EL2	USP45
3131.1	SURP and G-patch domain-conta	1.80E+05	0.00E+00	-1.80E+05	-1.80E+05	NULL	AD	Q8IX01	SUGP2
3086.1	Deoxynucleotidyltransferase ter	1.82E+05	0.00E+00	-1.82E+05	-1.82E+05	NULL	AD	Q5QJE6	DNTTIP2
1073.1	Pantothenate kinase 4	1.84E+05	0.00E+00	-1.84E+05	-1.84E+05	NULL	AD	Q9NVE7	PANK4
951.1	Testis-expressed sequence 10 p	1.85E+05	0.00E+00	-1.85E+05	-1.85E+05	NULL	AD	Q9NXF1	TEX10
2281.1	EMILIN-2	1.85E+05	0.00E+00	-1.85E+05	-1.85E+05	NULL	AD	Q9BXX0	EMILIN2
2705.1	Treacle protein	1.86E+05	0.00E+00	-1.86E+05	-1.86E+05	NULL	AD	E9PHK9	TCOF1
3125.1	Isoform 3 of Semaphorin-6C	1.86E+05	0.00E+00	-1.86E+05	-1.86E+05	NULL	AD	Q9H3T2-3	SEMA6C
1676.1	Zinc finger FYVE domain-contair	1.88E+05	0.00E+00	-1.88E+05	-1.88E+05	NULL	AD	Q68DK2	ZFYVE26
2349.1	DC-STAMP domain-containing p	1.90E+05	0.00E+00	-1.90E+05	-1.90E+05	NULL	AD	Q5T1A1	DCST2
2641.1	Probable ATP-dependent RNA h	1.90E+05	0.00E+00	-1.90E+05	-1.90E+05	NULL	AD	Q8IY37	DHX37
3192.1	Forkhead box protein O4	1.90E+05	0.00E+00	-1.90E+05	-1.90E+05	NULL	AD	P98177	FOXO4
2654.1	Alpha-2-macroglobulin	1.91E+05	0.00E+00	-1.91E+05	-1.91E+05	NULL	AD	P01023	A2M
2905.1	Probable tRNA threonylcarbamo	1.91E+05	0.00E+00	-1.91E+05	-1.91E+05	NULL	AD	Q9NPF4	OSGEP
860.1	Annexin A6	1.92E+05	0.00E+00	-1.92E+05	-1.92E+05	NULL	AD	P08133	ANXA6
1872.1	Ferritin light chain	1.92E+05	0.00E+00	-1.92E+05	-1.92E+05	NULL	AD	P02792	FTL
2843.1	Vacuolar protein sorting-associa	1.92E+05	0.00E+00	-1.92E+05	-1.92E+05	NULL	AD	Q9H267	VPS33B
3037.1	Transcription factor HIVEP3	1.92E+05	0.00E+00	-1.92E+05	-1.92E+05	NULL	AD	Q5T1R4	HIVEP3
3077.1	Protein SOGA2	1.93E+05	0.00E+00	-1.93E+05	-1.93E+05	NULL	AD	Q9Y4B5	SOGA2
2002.1	Isoform 2 of M-phase phosphop	1.94E+05	0.00E+00	-1.94E+05	-1.94E+05	NULL	AD	Q99549-2	MPHOSPH8
1394.1	Phosphatidylinositol 3,4,5-trisph	1.96E+05	0.00E+00	-1.96E+05	-1.96E+05	NULL	AD	O15357	INPPL1
2159.1	Testis-expressed sequence 13A	1.96E+05	0.00E+00	-1.96E+05	-1.96E+05	NULL	AD	Q9BXU3	TEX13A
2531.1	Tumor necrosis factor receptor s	1.96E+05	0.00E+00	-1.96E+05	-1.96E+05	NULL	AD	P19438	TNFRSF1A
827.1	G protein-regulated inducer of r	1.97E+05	0.00E+00	-1.97E+05	-1.97E+05	NULL	AD	Q6ZVF9	GPRIN3
2773.1	Putative zinc finger protein 705I	1.97E+05	0.00E+00	-1.97E+05	-1.97E+05	NULL	AD	A8MWA4	ZNF705E
2968.1	COMM domain-containing prote	1.97E+05	0.00E+00	-1.97E+05	-1.97E+05	NULL	AD	Q9Y6G5	COMMD10
2560.1	Taste receptor type 1 member 1	1.98E+05	0.00E+00	-1.98E+05	-1.98E+05	NULL	AD	Q7RTX1	TAS1R1
2749.1	Frizzled-10	1.98E+05	0.00E+00	-1.98E+05	-1.98E+05	NULL	AD	F5H450	FZD10
2851.1	Glutamate receptor-interacting	1.98E+05	0.00E+00	-1.98E+05	-1.98E+05	NULL	AD	Q9C0E4	GRIP2
2211.1	Dedicator of cytokinesis protein	1.99E+05	0.00E+00	-1.99E+05	-1.99E+05	NULL	AD	Q8NF50	DOCK8
2283.1	RUN domain-containing protein	1.99E+05	0.00E+00	-1.99E+05	-1.99E+05	NULL	AD	Q96NL0	RUNDC3B
2239.1	NudC domain-containing protei	2.01E+05	0.00E+00	-2.01E+05	-2.01E+05	NULL	AD	Q96RS6	NUDCD1
1919.1	Ephrin type-B receptor 6	2.02E+05	0.00E+00	-2.02E+05	-2.02E+05	NULL	AD	O15197	EPHB6
1446.1	TMF-regulated nuclear protein 1	2.03E+05	0.00E+00	-2.03E+05	-2.03E+05	NULL	AD	Q6NT89	TRNP1

2530.1	SH2 domain-containing protein	2.03E+05	0.00E+00	-2.03E+05	-2.03E+05	NULL	AD	Q8N5H7	SH2D3C
1667.1	Otolin-1	2.05E+05	0.00E+00	-2.05E+05	-2.05E+05	NULL	AD	A6NHN0	OTOL1
2437.1	Protein SOGA3	2.05E+05	0.00E+00	-2.05E+05	-2.05E+05	NULL	AD	Q5TF21	SOGA3
2416.1	Oncoprotein-induced transcript	2.06E+05	0.00E+00	-2.06E+05	-2.06E+05	NULL	AD	Q8WWZ8	OIT3
2876.1	Chloride channel protein 1	2.06E+05	0.00E+00	-2.06E+05	-2.06E+05	NULL	AD	P35523	CLCN1
638.2	YTH domain family protein 1	2.07E+05	0.00E+00	-2.07E+05	-2.07E+05	NULL	AD	Q9BYJ9	YTHDF1
1315.1	Myopalladin	2.07E+05	0.00E+00	-2.07E+05	-2.07E+05	NULL	AD	F5GWA6	MYPN
2399.1	Inositol 1,4,5-trisphosphate rece	2.07E+05	0.00E+00	-2.07E+05	-2.07E+05	NULL	AD	Q14571	ITPR2
2504.1	Lactoperoxidase	2.07E+05	0.00E+00	-2.07E+05	-2.07E+05	NULL	AD	P22079	LPO
2867.1	Growth hormone-inducible tran	2.07E+05	0.00E+00	-2.07E+05	-2.07E+05	NULL	AD	Q9H3K2	GHITM
1354.1	Wilms tumor protein 1-interacti	2.07E+05	0.00E+00	-2.07E+05	-2.07E+05	NULL	AD	A6NIX2	WTIP
1725.1	Steryl-sulfatase	2.08E+05	0.00E+00	-2.08E+05	-2.08E+05	NULL	AD	P08842	STS
2162.1	Fibulin-1	2.08E+05	0.00E+00	-2.08E+05	-2.08E+05	NULL	AD	F8W7M9	FBLN1
2386.1	Shugoshin-like 2	2.08E+05	0.00E+00	-2.08E+05	-2.08E+05	NULL	AD	Q562F6	SGOL2
1656.1	Pancreatic lipase-related protei	2.09E+05	0.00E+00	-2.09E+05	-2.09E+05	NULL	AD	Q17RR3	PNLIPRP3
3189.1	Transformation/transcription do	2.09E+05	0.00E+00	-2.09E+05	-2.09E+05	NULL	AD	Q9Y4A5	TRRAP
1674.1	Isoform 11 of Peroxisomal N(1)-	2.10E+05	0.00E+00	-2.10E+05	-2.10E+05	NULL	AD	Q6QHF9-9	PAOX
3084.1	PWWP domain-containing prote	2.10E+05	0.00E+00	-2.10E+05	-2.10E+05	NULL	AD	Q96N64	PWWP2A
3065.1	Histone-lysine N-methyltransfer	2.11E+05	0.00E+00	-2.11E+05	-2.11E+05	NULL	AD	Q96L73	NSD1
894.1	Plastin-3	2.12E+05	0.00E+00	-2.12E+05	-2.12E+05	NULL	AD	P13797	PLS3
1181.1	Zinc finger CCCH-type antiviral p	2.12E+05	0.00E+00	-2.12E+05	-2.12E+05	NULL	AD	C9J6P4	ZC3HAV1
1214.1	Meckelin	2.12E+05	0.00E+00	-2.12E+05	-2.12E+05	NULL	AD	Q5HYA8	TMEM67
1235.1	Nucleosome assembly protein 1	2.12E+05	0.00E+00	-2.12E+05	-2.12E+05	NULL	AD	Q9ULW6	NAP1L2
1576.1	SUMO-interacting motif-contair	2.12E+05	0.00E+00	-2.12E+05	-2.12E+05	NULL	AD	Q8NDZ2	SIMC1
2650.1	Maternal embryonic leucine zip	2.13E+05	0.00E+00	-2.13E+05	-2.13E+05	NULL	AD	Q14680	MELK
2563.1	Euchromatic histone-lysine N-m	2.14E+05	0.00E+00	-2.14E+05	-2.14E+05	NULL	AD	B0UZY1	EHMT2
2944.1	DIS3-like exonuclease 1	2.14E+05	0.00E+00	-2.14E+05	-2.14E+05	NULL	AD	Q8TF46	DIS3L
3009.1	Isoform 7 of Protein PRRC2C	2.14E+05	0.00E+00	-2.14E+05	-2.14E+05	NULL	AD	Q9Y520-7	PRRC2C
911.1	Ribonuclease H2 subunit A	2.16E+05	0.00E+00	-2.16E+05	-2.16E+05	NULL	AD	O75792	RNASEH2A
2196.1	Sodium channel and clathrin linl	2.17E+05	0.00E+00	-2.17E+05	-2.17E+05	NULL	AD	Q96NL6	SCLT1
2282.1	Dolichyl-diphosphooligosacchar	2.18E+05	0.00E+00	-2.18E+05	-2.18E+05	NULL	AD	P04843	RPN1
573.1	Isoform 2 of COP9 signalosome	2.19E+05	0.00E+00	-2.19E+05	-2.19E+05	NULL	AD	Q13098-7	GPS1
617.1	Dynamin-1	2.19E+05	0.00E+00	-2.19E+05	-2.19E+05	NULL	AD	Q05193	DNM1
1282.1	Protein Daple	2.19E+05	0.00E+00	-2.19E+05	-2.19E+05	NULL	AD	Q9P219	CCDC88C
1850.1	Death-inducer obliterator 1	2.19E+05	0.00E+00	-2.19E+05	-2.19E+05	NULL	AD	Q9BTC0	DIDO1

1096.1	Zinc finger protein 461	2.20E+05	0.00E+00	-2.20E+05	-2.20E+05	NULL	AD	Q8TAF7	ZNF461
3122.1	Protein PAT1 homolog 1	2.20E+05	0.00E+00	-2.20E+05	-2.20E+05	NULL	AD	Q86TB9	PATL1
1917.1	Erythrocyte band 7 integral mer	2.21E+05	0.00E+00	-2.21E+05	-2.21E+05	NULL	AD	P27105	STOM
2029.1	Zinc finger protein 500	2.21E+05	0.00E+00	-2.21E+05	-2.21E+05	NULL	AD	O60304	ZNF500
2935.1	Paired box protein Pax-5	2.21E+05	0.00E+00	-2.21E+05	-2.21E+05	NULL	AD	E7ERK2	PAX5
1532.1	Choline-phosphate cytidylyltran	2.22E+05	0.00E+00	-2.22E+05	-2.22E+05	NULL	AD	C9JEJ2	PCYT1A
1959.1	Myosin-8	2.22E+05	0.00E+00	-2.22E+05	-2.22E+05	NULL	AD	P13535	MYH8
2244.1	28S ribosomal protein S22, mito	2.22E+05	0.00E+00	-2.22E+05	-2.22E+05	NULL	AD	P82650	MRPS22
2464.1	Acyl-coenzyme A thioesterase 8	2.22E+05	0.00E+00	-2.22E+05	-2.22E+05	NULL	AD	O14734	ACOT8
2941.1	Coiled-coil domain-containing p	2.23E+05	0.00E+00	-2.23E+05	-2.23E+05	NULL	AD	Q9UL16	CCDC19
1477.1	Cyclin-dependent kinase 3	2.25E+05	0.00E+00	-2.25E+05	-2.25E+05	NULL	AD	Q00526	CDK3
2566.1	Protocadherin Fat 3	2.27E+05	0.00E+00	-2.27E+05	-2.27E+05	NULL	AD	Q8TDW7	FAT3
2667.1	Secreted phosphoprotein 24	2.27E+05	0.00E+00	-2.27E+05	-2.27E+05	NULL	AD	Q13103	SPP2
2795.1	WD repeat-containing protein 2	2.27E+05	0.00E+00	-2.27E+05	-2.27E+05	NULL	AD	Q8TBZ3	WDR20
1203.1	Isoform 2 of BRCA2 and CDKN1/	2.28E+05	0.00E+00	-2.28E+05	-2.28E+05	NULL	AD	Q9P287-2	BCCIP
2901.1	CUB domain-containing protein	2.28E+05	0.00E+00	-2.28E+05	-2.28E+05	NULL	AD	Q9H5V8	CDCP1
2035.1	Insulinoma-associated protein 2	2.29E+05	0.00E+00	-2.29E+05	-2.29E+05	NULL	AD	Q96T92	INSM2
1706.1	Zinc finger protein 667	2.31E+05	0.00E+00	-2.31E+05	-2.31E+05	NULL	AD	E7EPS0	ZNF667
2369.1	Truncated apolipoprotein C-I	2.31E+05	0.00E+00	-2.31E+05	-2.31E+05	NULL	AD	K7EPF9	APOC1
1171.1	Isoform 2 of Nuclear factor relat	2.32E+05	0.00E+00	-2.32E+05	-2.32E+05	NULL	AD	Q6P4R8-2	NFRKB
2168.1	Integrin alpha-1	2.32E+05	0.00E+00	-2.32E+05	-2.32E+05	NULL	AD	P56199	ITGA1
2631.1	Isoform 3 of Fanconi anemia-as:	2.32E+05	0.00E+00	-2.32E+05	-2.32E+05	NULL	AD	Q6NZ36-3	FAAP20
2169.1	Zinc finger protein 280B	2.33E+05	0.00E+00	-2.33E+05	-2.33E+05	NULL	AD	Q86YH2	ZNF280B
1852.1	Sodium-dependent neutral amir	2.34E+05	0.00E+00	-2.34E+05	-2.34E+05	NULL	AD	Q9H1V8	SLC6A17
2285.1	Paired mesoderm homeobox pr	2.35E+05	0.00E+00	-2.35E+05	-2.35E+05	NULL	AD	Q99453	PHOX2B
3245.1	Lethal(2) giant larvae protein hc	2.35E+05	0.00E+00	-2.35E+05	-2.35E+05	NULL	AD	Q6P1M3	LLGL2
2813.1	H/ACA ribonucleoprotein compl	2.35E+05	0.00E+00	-2.35E+05	-2.35E+05	NULL	AD	Q96HR8	NAF1
2746.1	Isoform 2 of Ubiquitin conjugati	2.36E+05	0.00E+00	-2.36E+05	-2.36E+05	NULL	AD	Q14139-2	UBE4A
3.6	Keratin, type II cytoskeletal 2 or	2.37E+05	0.00E+00	-2.37E+05	-2.37E+05	NULL	AD	Q01546	KRT76
1914.1	Zinc finger protein 567	2.37E+05	0.00E+00	-2.37E+05	-2.37E+05	NULL	AD	Q8N184	ZNF567
21.3	Tropomyosin 2 (Beta)	2.38E+05	0.00E+00	-2.38E+05	-2.38E+05	NULL	AD	Q5TCU8	TPM2
1404.1	Zinc finger protein 64 homolog,	2.38E+05	0.00E+00	-2.38E+05	-2.38E+05	NULL	AD	Q9NTW7	ZFP64
993.1	Baculoviral IAP repeat-containin	2.39E+05	0.00E+00	-2.39E+05	-2.39E+05	NULL	AD	Q13490	BIRC2
1013.1	Ankyrin repeat and SOCS box pr	2.40E+05	0.00E+00	-2.40E+05	-2.40E+05	NULL	AD	J3KQJ5	ASB10
1460.1	Fibrous sheath-interacting prote	2.40E+05	0.00E+00	-2.40E+05	-2.40E+05	NULL	AD	J3QTJ6	FSIP2

1880.1	Transmembrane protein 176A	2.43E+05	0.00E+00	-2.43E+05	-2.43E+05	NULL	AD	Q96HP8	TMEM176A
2547.1	Tyrosine-protein kinase Fes/Fps	2.44E+05	0.00E+00	-2.44E+05	-2.44E+05	NULL	AD	P07332	FES
2721.1	Zinc finger protein 99	2.44E+05	0.00E+00	-2.44E+05	-2.44E+05	NULL	AD	A8MXY4	ZNF99
1641.1	Guanine nucleotide-binding pro	2.46E+05	0.00E+00	-2.46E+05	-2.46E+05	NULL	AD	P19086	GNAZ
1883.1	Protein FAM65C	2.47E+05	0.00E+00	-2.47E+05	-2.47E+05	NULL	AD	F5H0X2	FAM65C
2329.1	SUMO-activating enzyme subun	2.48E+05	0.00E+00	-2.48E+05	-2.48E+05	NULL	AD	Q9UBT2	UBA2
1905.1	Protein phosphatase 1 regulato	2.49E+05	0.00E+00	-2.49E+05	-2.49E+05	NULL	AD	Q13522	PPP1R1A
3062.1	Isoform 3 of SH3-containing GRI	2.49E+05	0.00E+00	-2.49E+05	-2.49E+05	NULL	AD	Q9BQI5-3	SGIP1
941.1	Nucleolar pre-ribosomal-associa	2.50E+05	0.00E+00	-2.50E+05	-2.50E+05	NULL	AD	O60287	URB1
2719.1	Phosphorylated adapter RNA ex	2.50E+05	0.00E+00	-2.50E+05	-2.50E+05	NULL	AD	Q9H814	PHAX
629.1	tRNA-splicing endonuclease sub	2.51E+05	0.00E+00	-2.51E+05	-2.51E+05	NULL	AD	E7EQB3	TSEN34
2043.1	Zinc finger protein ubi-d4	2.53E+05	0.00E+00	-2.53E+05	-2.53E+05	NULL	AD	J3KMZ8	DPF2
3238.1	Lysine-specific demethylase 5A	2.53E+05	0.00E+00	-2.53E+05	-2.53E+05	NULL	AD	P29375	KDM5A
1137.1	T-lymphoma invasion and meta:	2.59E+05	0.00E+00	-2.59E+05	-2.59E+05	NULL	AD	Q13009	TIAM1
1248.1	Isoform 2 of LIM and SH3 domai	2.59E+05	0.00E+00	-2.59E+05	-2.59E+05	NULL	AD	Q14847-2	LASP1
1342.1	Ankyrin repeat domain-containi	2.59E+05	0.00E+00	-2.59E+05	-2.59E+05	NULL	AD	Q6UB99	ANKRD11
1669.1	Isoform 2 of Diphosphoinositol	2.59E+05	0.00E+00	-2.59E+05	-2.59E+05	NULL	AD	Q9NZJ9-2	NUDT4
2316.1	Coiled-coil and C2 domain-conta	2.59E+05	0.00E+00	-2.59E+05	-2.59E+05	NULL	AD	Q6P1N0	CC2D1A
3156.1	Transcription factor Dp-1	2.60E+05	0.00E+00	-2.60E+05	-2.60E+05	NULL	AD	F5H452	TFDP1
2089.1	E3 ubiquitin-protein ligase RNF1	2.62E+05	0.00E+00	-2.62E+05	-2.62E+05	NULL	AD	Q96K19	RNF170
523.1	Cyclin-dependent kinase 1	2.63E+05	0.00E+00	-2.63E+05	-2.63E+05	NULL	AD	P06493	CDK1
1259.1	Rho-associated protein kinase 2	2.63E+05	0.00E+00	-2.63E+05	-2.63E+05	NULL	AD	O75116	ROCK2
1545.1	BTB/POZ domain-containing pro	2.64E+05	0.00E+00	-2.64E+05	-2.64E+05	NULL	AD	Q9BQ13	KCTD14
2642.1	Isoform 2 of MORN repeat-cont	2.64E+05	0.00E+00	-2.64E+05	-2.64E+05	NULL	AD	Q8NDC4-2	MORN4
1721.1	Vasoactive intestinal polypeptid	2.65E+05	0.00E+00	-2.65E+05	-2.65E+05	NULL	AD	E9PCR5	VIPR2
1462.1	A-kinase anchor protein 8	2.66E+05	0.00E+00	-2.66E+05	-2.66E+05	NULL	AD	O43823	AKAP8
1437.1	Phosphatidylinositol 4-phospha	2.68E+05	0.00E+00	-2.68E+05	-2.68E+05	NULL	AD	O00750	PIK3C2B
3081.1	Sodium/hydrogen exchanger 3	2.69E+05	0.00E+00	-2.69E+05	-2.69E+05	NULL	AD	P48764	SLC9A3
3206.1	Neurexin-1-beta	2.69E+05	0.00E+00	-2.69E+05	-2.69E+05	NULL	AD	P58400	NRXN1
9.3	Tubulin beta-2B chain	2.71E+05	0.00E+00	-2.71E+05	-2.71E+05	NULL	AD	Q9BVA1	TUBB2B
1594.1	EGF-like module-containing muc	2.71E+05	0.00E+00	-2.71E+05	-2.71E+05	NULL	AD	Q14246	EMR1
2656.1	Isoform 3 of Coiled-coil domain-	2.71E+05	0.00E+00	-2.71E+05	-2.71E+05	NULL	AD	Q6TFL3-4	CCDC171
937.1	Centrosomal protein of 128 kDa	2.72E+05	0.00E+00	-2.72E+05	-2.72E+05	NULL	AD	Q6ZU80	CEP128
1552.1	Rho GTPase-activating protein 3	2.72E+05	0.00E+00	-2.72E+05	-2.72E+05	NULL	AD	Q2M1Z3	ARHGAP31
2572.1	Zinc finger protein 549	2.72E+05	0.00E+00	-2.72E+05	-2.72E+05	NULL	AD	Q6P9A3	ZNF549

913.1	Ferritin heavy polypeptide-like 1	2.75E+05	0.00E+00	-2.75E+05	-2.75E+05	NULL	AD	Q9BXU8	FTHL17
1225.1	Lipoma-preferred partner	2.76E+05	0.00E+00	-2.76E+05	-2.76E+05	NULL	AD	Q93052	LPP
1596.1	Charged multivesicular body pro	2.76E+05	0.00E+00	-2.76E+05	-2.76E+05	NULL	AD	Q9H444	CHMP4B
1619.1	UPF0489 protein C5orf22	2.77E+05	0.00E+00	-2.77E+05	-2.77E+05	NULL	AD	B4DR92	C5orf22
2556.1	Nik-related protein kinase	2.77E+05	0.00E+00	-2.77E+05	-2.77E+05	NULL	AD	F5H049	NRK
3104.1	UHRF1-binding protein 1	2.77E+05	0.00E+00	-2.77E+05	-2.77E+05	NULL	AD	Q6BDS2	UHRF1BP1
1507.1	Disintegrin and metalloproteina	2.77E+05	0.00E+00	-2.77E+05	-2.77E+05	NULL	AD	P78325	ADAM8
3111.1	Polyadenylate-binding protein 5	2.78E+05	0.00E+00	-2.78E+05	-2.78E+05	NULL	AD	Q96DU9	PABPC5
2362.1	Isoform 2 of TNF receptor-assoc	2.80E+05	0.00E+00	-2.80E+05	-2.80E+05	NULL	AD	Q12933-2	TRAF2
1835.1	AP20 region protein 1	2.81E+05	0.00E+00	-2.81E+05	-2.81E+05	NULL	AD	Q8IVJ8	APRG1
2551.1	Coiled-coil domain-containing p	2.81E+05	0.00E+00	-2.81E+05	-2.81E+05	NULL	AD	Q8ND07	CCDC176
2373.1	ETS translocation variant 3	2.82E+05	0.00E+00	-2.82E+05	-2.82E+05	NULL	AD	P41162	ETV3
740.1	Chromosome 1 open reading fra	2.83E+05	0.00E+00	-2.83E+05	-2.83E+05	NULL	AD	Q5VSH5	C1orf122
2797.1	Uroporphyrinogen decarboxylas	2.83E+05	0.00E+00	-2.83E+05	-2.83E+05	NULL	AD	P06132	UROD
2846.1	Forkhead box protein D1	2.83E+05	0.00E+00	-2.83E+05	-2.83E+05	NULL	AD	J3KT45	FOXD1
2859.1	1,4-alpha-glucan-branching enzy	2.83E+05	0.00E+00	-2.83E+05	-2.83E+05	NULL	AD	Q04446	GBE1
2784.1	Putative U6 snRNA phosphodies	2.84E+05	0.00E+00	-2.84E+05	-2.84E+05	NULL	AD	Q9BQ65	USB1
1582.1	Polypeptide N-acetylgalactosam	2.86E+05	0.00E+00	-2.86E+05	-2.86E+05	NULL	AD	Q8IXK2	GALNT12
2311.1	Killer cell immunoglobulin-like r	2.88E+05	0.00E+00	-2.88E+05	-2.88E+05	NULL	AD	Q8NHK3	KIR2DL5B
2013.1	Envoplakin	2.90E+05	0.00E+00	-2.90E+05	-2.90E+05	NULL	AD	K7EKI0	EVPL
1536.1	Adapter molecule crk	2.91E+05	0.00E+00	-2.91E+05	-2.91E+05	NULL	AD	P46108	CRK
1175.1	Protein FAM217B	2.93E+05	0.00E+00	-2.93E+05	-2.93E+05	NULL	AD	Q9NTX9	FAM217B
2108.1	RRP12-like protein	2.93E+05	0.00E+00	-2.93E+05	-2.93E+05	NULL	AD	Q5JTH9	RRP12
2499.1	Quinone oxidoreductase PIG3	2.93E+05	0.00E+00	-2.93E+05	-2.93E+05	NULL	AD	Q53FA7	TP53I3
3178.1	Isoform 2 of Transcription facto	2.94E+05	0.00E+00	-2.94E+05	-2.94E+05	NULL	AD	Q33E94-2	RFX4
2995.1	O-acetyl-ADP-ribose deacetylase	2.96E+05	0.00E+00	-2.96E+05	-2.96E+05	NULL	AD	Q9Y530	OARD1
3141.1	Breast cancer anti-estrogen resi	2.98E+05	0.00E+00	-2.98E+05	-2.98E+05	NULL	AD	Q05GC8	BCAR2
1345.1	Cytokine inducible SH2-containi	3.00E+05	0.00E+00	-3.00E+05	-3.00E+05	NULL	AD	Q9NSE2	CISH
1834.1	DNA-binding protein RFX8	3.03E+05	0.00E+00	-3.03E+05	-3.03E+05	NULL	AD	Q6ZV50	RFX8
943.1	Obscurin	3.05E+05	0.00E+00	-3.05E+05	-3.05E+05	NULL	AD	H3BPX2	OBSCN
2578.1	Dynein heavy chain 1, axonemal	3.05E+05	0.00E+00	-3.05E+05	-3.05E+05	NULL	AD	Q9P2D7	DNAH1
2363.1	Multimerin-1	3.08E+05	0.00E+00	-3.08E+05	-3.08E+05	NULL	AD	Q13201	MMRN1
1693.1	Ankyrin repeat domain-containi	3.11E+05	0.00E+00	-3.11E+05	-3.11E+05	NULL	AD	Q69YU3	ANKRD34A
855.1	Golgin subfamily A member 3	3.12E+05	0.00E+00	-3.12E+05	-3.12E+05	NULL	AD	Q08378	GOLGA3
1008.1	UDP-GlcNAc:betaGal beta-1,3-N	3.12E+05	0.00E+00	-3.12E+05	-3.12E+05	NULL	AD	Q8NFL0	B3GNT7

1653.1	Vacuolar protein sorting-associa	3.12E+05	0.00E+00	-3.12E+05	-3.12E+05	NULL	AD	Q9P1Q0	VPS54
2691.1	DNA ligase 1	3.14E+05	0.00E+00	-3.14E+05	-3.14E+05	NULL	AD	P18858	LIG1
1862.1	Protein YIPF4	3.15E+05	0.00E+00	-3.15E+05	-3.15E+05	NULL	AD	Q9BSR8	YIPF4
10.2	Clathrin heavy chain 2	3.16E+05	0.00E+00	-3.16E+05	-3.16E+05	NULL	AD	P53675	CLTCL1
708.1	Na(+)/H(+) exchange regulatory	3.16E+05	0.00E+00	-3.16E+05	-3.16E+05	NULL	AD	O14745	SLC9A3R1
1504.1	UBX domain-containing protein	3.16E+05	0.00E+00	-3.16E+05	-3.16E+05	NULL	AD	Q14CS0	UBXN2B
2669.1	FtsJ methyltransferase domain-c	3.18E+05	0.00E+00	-3.18E+05	-3.18E+05	NULL	AD	Q8IYT2	FTSJD1
3140.1	Homeobox protein MOX-2	3.18E+05	0.00E+00	-3.18E+05	-3.18E+05	NULL	AD	P50222	MEOX2
1974.1	Heat shock protein beta-3	3.20E+05	0.00E+00	-3.20E+05	-3.20E+05	NULL	AD	Q12988	HSPB3
943.3	Obscurin	3.21E+05	0.00E+00	-3.21E+05	-3.21E+05	NULL	AD	H7BY31	OBSCN
1250.1	HERV-K_3q12.3 provirus ancestr	3.21E+05	0.00E+00	-3.21E+05	-3.21E+05	NULL	AD	Q9HDB9	ERVK-5
1930.1	Anoctamin-5	3.23E+05	0.00E+00	-3.23E+05	-3.23E+05	NULL	AD	Q75V66	ANO5
1506.1	Isoform 6 of Calcium/calmodulir	3.24E+05	0.00E+00	-3.24E+05	-3.24E+05	NULL	AD	Q13555-6	CAMK2G
1750.1	Sentrin-specific protease 1	3.25E+05	0.00E+00	-3.25E+05	-3.25E+05	NULL	AD	Q9P0U3	SENP1
2011.1	PHD and RING finger domain-co	3.25E+05	0.00E+00	-3.25E+05	-3.25E+05	NULL	AD	Q9P1Y6	PHRF1
2376.1	Isoform 4 of Tether containing U	3.27E+05	0.00E+00	-3.27E+05	-3.27E+05	NULL	AD	Q9BZE9-4	ASPSCR1
1731.1	Dendritic cell nuclear protein 1	3.28E+05	0.00E+00	-3.28E+05	-3.28E+05	NULL	AD	Q8TF63	DCNP1
2679.1	Isoform 5 of Bromodomain-cont	3.29E+05	0.00E+00	-3.29E+05	-3.29E+05	NULL	AD	Q9H8M2-1	BRD9
709.1	Isoform 3 of Clathrin interactor	3.30E+05	0.00E+00	-3.30E+05	-3.30E+05	NULL	AD	Q14677-3	CLINT1
1657.1	Frizzled-3	3.30E+05	0.00E+00	-3.30E+05	-3.30E+05	NULL	AD	Q9NPG1	FZD3
1239.1	Chromosome-associated kinesin	3.31E+05	0.00E+00	-3.31E+05	-3.31E+05	NULL	AD	Q2VIQ3	KIF4B
2136.1	CAAX prenyl protease 1 homolo	3.31E+05	0.00E+00	-3.31E+05	-3.31E+05	NULL	AD	O75844	ZMPSTE24
2618.1	ATP-binding cassette sub-family	3.31E+05	0.00E+00	-3.31E+05	-3.31E+05	NULL	AD	Q9UBJ2	ABCD2
1413.1	Olfactory receptor 52B6	3.32E+05	0.00E+00	-3.32E+05	-3.32E+05	NULL	AD	Q8NGF0	OR52B6
2565.1	Protein DBF4 homolog A	3.32E+05	0.00E+00	-3.32E+05	-3.32E+05	NULL	AD	Q9UBU7	DBF4
1138.1	Inner centromere protein	3.33E+05	0.00E+00	-3.33E+05	-3.33E+05	NULL	AD	Q9NQS7	INCENP
2325.1	Olfactory receptor 14J1	3.36E+05	0.00E+00	-3.36E+05	-3.36E+05	NULL	AD	Q9UGF5	OR14J1
2144.1	V-set and immunoglobulin doma	3.37E+05	0.00E+00	-3.37E+05	-3.37E+05	NULL	AD	Q9Y279	VSIG4
2266.1	Protein FAM184B	3.37E+05	0.00E+00	-3.37E+05	-3.37E+05	NULL	AD	Q9ULE4	FAM184B
1711.1	Macrophage metalloelastase	3.39E+05	0.00E+00	-3.39E+05	-3.39E+05	NULL	AD	P39900	MMP12
1765.1	Probable G-protein coupled rece	3.39E+05	0.00E+00	-3.39E+05	-3.39E+05	NULL	AD	Q6PRD1	GPR179
890.1	Isoform 2 of Allograft inflammat	3.40E+05	0.00E+00	-3.40E+05	-3.40E+05	NULL	AD	Q9BQI0-2	AIF1L
2027.1	F-box/LRR-repeat protein 5	3.40E+05	0.00E+00	-3.40E+05	-3.40E+05	NULL	AD	Q9UKA1	FBXL5
2605.1	Kinesin-like protein KIF19	3.40E+05	0.00E+00	-3.40E+05	-3.40E+05	NULL	AD	Q2TAC6	KIF19
1475.1	Collagen alpha-6(VI) chain	3.41E+05	0.00E+00	-3.41E+05	-3.41E+05	NULL	AD	F8W6Y7	COL6A6

2822.1	Radial spoke head protein 3 hor	3.41E+05	0.00E+00	-3.41E+05	-3.41E+05	NULL	AD	Q86UC2	RSPH3
2295.1	Probable tubulin polyglutamyla:	3.43E+05	0.00E+00	-3.43E+05	-3.43E+05	NULL	AD	O95922	TTLL1
2338.1	Cytoplasmic tyrosine-protein kir	3.43E+05	0.00E+00	-3.43E+05	-3.43E+05	NULL	AD	P51813	BMX
2742.1	PI-PLC X domain-containing prot	3.43E+05	0.00E+00	-3.43E+05	-3.43E+05	NULL	AD	Q0VAA5	PLCXD2
2864.1	Cyclin-dependent kinase 11B	3.44E+05	0.00E+00	-3.44E+05	-3.44E+05	NULL	AD	P21127	CDK11B
1453.1	Actin-related protein 10	3.47E+05	0.00E+00	-3.47E+05	-3.47E+05	NULL	AD	Q9NZ32	ACTR10
1398.1	Serine/threonine-protein kinase	3.51E+05	0.00E+00	-3.51E+05	-3.51E+05	NULL	AD	P51955	NEK2
2059.1	Sentrin-specific protease 6	3.51E+05	0.00E+00	-3.51E+05	-3.51E+05	NULL	AD	Q9GZR1	SENP6
1024.1	Protein misato homolog 1	3.52E+05	0.00E+00	-3.52E+05	-3.52E+05	NULL	AD	Q9BUK6	MSTO1
840.1	Ubiquitin carboxyl-terminal hyd	3.54E+05	0.00E+00	-3.54E+05	-3.54E+05	NULL	AD	P45974	USP5
1341.1	Glomulin	3.56E+05	0.00E+00	-3.56E+05	-3.56E+05	NULL	AD	Q92990	GLMN
2115.1	Zinc finger protein basonuclin-1	3.56E+05	0.00E+00	-3.56E+05	-3.56E+05	NULL	AD	Q01954	BNC1
1811.1	Flotillin-2	3.57E+05	0.00E+00	-3.57E+05	-3.57E+05	NULL	AD	E7EMK3	FLOT2
885.1	Kinesin-like protein KIF26A	3.59E+05	0.00E+00	-3.59E+05	-3.59E+05	NULL	AD	Q9ULI4	KIF26A
2509.1	C3 and PZP-like alpha-2-macrog	3.59E+05	0.00E+00	-3.59E+05	-3.59E+05	NULL	AD	Q8IZJ3	CPAMD8
2837.1	Desmoglein-3	3.59E+05	0.00E+00	-3.59E+05	-3.59E+05	NULL	AD	P32926	DSG3
3039.1	Ubiquitin-associated and SH3 dc	3.59E+05	0.00E+00	-3.59E+05	-3.59E+05	NULL	AD	Q8TF42	UBASH3B
1233.1	Histone-arginine methyltransfer	3.62E+05	0.00E+00	-3.62E+05	-3.62E+05	NULL	AD	Q86X55	CARM1
1206.1	Latent-transforming growth fact	3.63E+05	0.00E+00	-3.63E+05	-3.63E+05	NULL	AD	Q14767	LTBP2
2287.1	1-aminocyclopropane-1-carboxy	3.63E+05	0.00E+00	-3.63E+05	-3.63E+05	NULL	AD	Q96QU6	ACCS
2577.1	Prickle-like protein 1	3.63E+05	0.00E+00	-3.63E+05	-3.63E+05	NULL	AD	Q96MT3	PRICKLE1
2777.1	Nucleolar transcription factor 1	3.65E+05	0.00E+00	-3.65E+05	-3.65E+05	NULL	AD	E9PKP7	UBTF
3252.1	Ubiquitin carboxyl-terminal hyd	3.65E+05	0.00E+00	-3.65E+05	-3.65E+05	NULL	AD	Q92995	USP13
9.4	Tubulin beta-3 chain	3.69E+05	0.00E+00	-3.69E+05	-3.69E+05	NULL	AD	Q13509	TUBB3
958.1	Spindlin-2A	3.71E+05	0.00E+00	-3.71E+05	-3.71E+05	NULL	AD	Q99865	SPIN2A
2037.1	Procollagen-lysine,2-oxoglutara	3.74E+05	0.00E+00	-3.74E+05	-3.74E+05	NULL	AD	O60568	PLOD3
1129.1	Rap guanine nucleotide exchang	3.75E+05	0.00E+00	-3.75E+05	-3.75E+05	NULL	AD	O95398	RAPGEF3
2424.1	A-kinase anchor protein 11	3.76E+05	0.00E+00	-3.76E+05	-3.76E+05	NULL	AD	Q9UKA4	AKAP11
1339.1	Isoform SERCA3F of Sarcoplasm	3.81E+05	0.00E+00	-3.81E+05	-3.81E+05	NULL	AD	Q93084-7	ATP2A3
2779.1	Rho GTPase-activating protein 2	3.81E+05	0.00E+00	-3.81E+05	-3.81E+05	NULL	AD	Q52LW3	ARHGAP29
2955.1	Paired amphipathic helix protei	3.81E+05	0.00E+00	-3.81E+05	-3.81E+05	NULL	AD	O75182	SIN3B
2232.1	Isoform 4 of Melanoma-associat	3.84E+05	0.00E+00	-3.84E+05	-3.84E+05	NULL	AD	Q96JG8-4	MAGED4
2318.1	TBC1 domain family member 8E	3.84E+05	0.00E+00	-3.84E+05	-3.84E+05	NULL	AD	Q0IIM8	TBC1D8B
1449.1	Isoform 7 of Tetratricopeptide r	3.87E+05	0.00E+00	-3.87E+05	-3.87E+05	NULL	AD	Q8NDW8-7	TTC21A
2731.1	Anterior gradient protein 3 hom	3.88E+05	0.00E+00	-3.88E+05	-3.88E+05	NULL	AD	Q8TD06	AGR3

1060.1	MON1 homolog A (Yeast), isofor	3.90E+05	0.00E+00	-3.90E+05	-3.90E+05	NULL	AD	Q86VX9	MON1A
1584.1	Solute carrier family 15 member	3.93E+05	0.00E+00	-3.93E+05	-3.93E+05	NULL	AD	P46059	SLC15A1
2737.1	Centrosomal protein of 192 kDa	3.94E+05	0.00E+00	-3.94E+05	-3.94E+05	NULL	AD	E9PF99	CEP192
2759.1	A disintegrin and metalloprotein	3.94E+05	0.00E+00	-3.94E+05	-3.94E+05	NULL	AD	Q9UP79	ADAMTS8
2881.1	Apolipoprotein B-100	3.98E+05	0.00E+00	-3.98E+05	-3.98E+05	NULL	AD	P04114	APOB
1520.1	Microtubule-associated tumor s	4.03E+05	0.00E+00	-4.03E+05	-4.03E+05	NULL	AD	J3KQA9	MTUS2
1148.1	Probable ribonuclease ZC3H12D	4.04E+05	0.00E+00	-4.04E+05	-4.04E+05	NULL	AD	J3QT81	ZC3H12D
645.1	Isoform 5 of Protein piccolo	4.05E+05	0.00E+00	-4.05E+05	-4.05E+05	NULL	AD	Q9Y6V0-5	PCLO
3068.1	Kinesin heavy chain isoform 5A	4.08E+05	0.00E+00	-4.08E+05	-4.08E+05	NULL	AD	Q12840	KIF5A
318.2	Vacuolar protein sorting-associa	4.09E+05	0.00E+00	-4.09E+05	-4.09E+05	NULL	AD	Q9UN37	VPS4A
2180.1	Intraflagellar transport protein 1	4.11E+05	0.00E+00	-4.11E+05	-4.11E+05	NULL	AD	Q9UG01	IFT172
3138.1	Ciliary neurotrophic factor recep	4.13E+05	0.00E+00	-4.13E+05	-4.13E+05	NULL	AD	P26992	CNTFR
2052.1	Translation initiation factor IF-2,	4.16E+05	0.00E+00	-4.16E+05	-4.16E+05	NULL	AD	P46199	MTIF2
3235.1	Zinc finger CW-type PWWP dom	4.17E+05	0.00E+00	-4.17E+05	-4.17E+05	NULL	AD	Q504Y3	ZCWPW2
1680.1	COBW domain-containing prote	4.20E+05	0.00E+00	-4.20E+05	-4.20E+05	NULL	AD	Q5RIA9	CBWD5
3242.1	DNA polymerase delta subunit 3	4.24E+05	0.00E+00	-4.24E+05	-4.24E+05	NULL	AD	Q15054	POLD3
1652.1	Transcriptional regulator ERG	4.26E+05	0.00E+00	-4.26E+05	-4.26E+05	NULL	AD	B4E3C5	ERG
1136.1	Isoform 3 of Charged multivesic	4.27E+05	0.00E+00	-4.27E+05	-4.27E+05	NULL	AD	Q9Y3E7-3	CHMP3
3210.1	Exosome complex component R	4.29E+05	0.00E+00	-4.29E+05	-4.29E+05	NULL	AD	Q96B26	EXOSC8
2135.1	Zinc finger protein 180	4.30E+05	0.00E+00	-4.30E+05	-4.30E+05	NULL	AD	Q9UJW8	ZNF180
1304.1	Nuclear receptor-interacting pro	4.35E+05	0.00E+00	-4.35E+05	-4.35E+05	NULL	AD	P48552	NRIP1
938.1	DNA polymerase theta	4.39E+05	0.00E+00	-4.39E+05	-4.39E+05	NULL	AD	O75417	POLQ
1409.1	Phospholipase D3	4.39E+05	0.00E+00	-4.39E+05	-4.39E+05	NULL	AD	Q8IV08	PLD3
2177.1	COMM domain-containing prote	4.39E+05	0.00E+00	-4.39E+05	-4.39E+05	NULL	AD	Q86X83	COMMD2
1580.1	Transcription elongation factor I	4.40E+05	0.00E+00	-4.40E+05	-4.40E+05	NULL	AD	Q15370	TCEB2
1082.1	Protein QN1 homolog	4.41E+05	0.00E+00	-4.41E+05	-4.41E+05	NULL	AD	Q5TB80	KIAA1009
824.2	Isoform 4 of Lysine-specific dem	4.44E+05	0.00E+00	-4.44E+05	-4.44E+05	NULL	AD	Q8NHM5-4	KDM2B
1273.1	PWWP domain-containing prote	4.46E+05	0.00E+00	-4.46E+05	-4.46E+05	NULL	AD	Q5H9M0	MUM1L1
2520.1	Kinesin-like protein KIF14	4.53E+05	0.00E+00	-4.53E+05	-4.53E+05	NULL	AD	Q15058	KIF14
2291.1	Msx2-interacting protein	4.57E+05	0.00E+00	-4.57E+05	-4.57E+05	NULL	AD	Q96T58	SPEN
2018.1	ATP-binding cassette sub-family	4.58E+05	0.00E+00	-4.58E+05	-4.58E+05	NULL	AD	Q9H222	ABCG5
363.1	Tubulin--tyrosine ligase-like pro	4.60E+05	0.00E+00	-4.60E+05	-4.60E+05	NULL	AD	Q14166	TTLL12
2533.1	Cyclic nucleotide-gated olfactor	4.60E+05	0.00E+00	-4.60E+05	-4.60E+05	NULL	AD	Q16280	CNGA2
1217.1	Alpha-centractin	4.69E+05	0.00E+00	-4.69E+05	-4.69E+05	NULL	AD	P61163	ACTR1A
1126.1	C11orf30 protein	4.73E+05	0.00E+00	-4.73E+05	-4.73E+05	NULL	AD	B7ZKU0	C11orf30

1159.1	Toll-interacting protein	4.74E+05	0.00E+00	-4.74E+05	-4.74E+05	NULL	AD	Q9H0E2	TOLLIP
3248.1	Putative lipocalin 1-like protein	4.75E+05	0.00E+00	-4.75E+05	-4.75E+05	NULL	AD	Q5VSP4	LCN1P1
2849.1	Fibroleukin	4.78E+05	0.00E+00	-4.78E+05	-4.78E+05	NULL	AD	Q14314	FGL2
803.1	Tight junction protein 1 (Zona o	4.79E+05	0.00E+00	-4.79E+05	-4.79E+05	NULL	AD	G3V1L9	TJP1
2226.1	NXPE family member 2	4.83E+05	0.00E+00	-4.83E+05	-4.83E+05	NULL	AD	Q96DL1	NXPE2
829.1	Isoform 3 of Dehydrogenase/re	4.84E+05	0.00E+00	-4.84E+05	-4.84E+05	NULL	AD	A0PJE2-3	DHRS12
1040.1	YEATS domain-containing prote	4.84E+05	0.00E+00	-4.84E+05	-4.84E+05	NULL	AD	Q9ULM3	YEATS2
1076.1	Centrin-2	4.88E+05	0.00E+00	-4.88E+05	-4.88E+05	NULL	AD	P41208	CETN2
2288.1	DNA replication factor Cdt1	4.89E+05	0.00E+00	-4.89E+05	-4.89E+05	NULL	AD	Q9H211	CDT1
3230.1	Isoform 4 of Transcription facto	4.90E+05	0.00E+00	-4.90E+05	-4.90E+05	NULL	AD	Q92994-4	BRF1
1696.1	Zinc finger protein 438	4.92E+05	0.00E+00	-4.92E+05	-4.92E+05	NULL	AD	Q7Z4V0	ZNF438
2785.1	Fractalkine	4.96E+05	0.00E+00	-4.96E+05	-4.96E+05	NULL	AD	H3BV86	CX3CL1
688.1	Cytoplasmic dynein 2 heavy cha	5.01E+05	0.00E+00	-5.01E+05	-5.01E+05	NULL	AD	Q8NCM8	DYNC2H1
1431.1	PH and SEC7 domain-containing	5.01E+05	0.00E+00	-5.01E+05	-5.01E+05	NULL	AD	Q9BQI7	PSD2
2039.1	Isoform 2 of Serine/threonine-p	5.08E+05	0.00E+00	-5.08E+05	-5.08E+05	NULL	AD	Q5VT25-2	CDC42BPA
2205.1	Helicase SKI2W	5.13E+05	0.00E+00	-5.13E+05	-5.13E+05	NULL	AD	Q15477	SKIV2L
1489.1	Collagen triple helix repeat-cont	5.14E+05	0.00E+00	-5.14E+05	-5.14E+05	NULL	AD	E7EVQ5	CTHRC1
1885.1	NF-kappa-B-repressing factor	5.14E+05	0.00E+00	-5.14E+05	-5.14E+05	NULL	AD	O15226	NKRF
920.1	Synaptotagmin-6	5.15E+05	0.00E+00	-5.15E+05	-5.15E+05	NULL	AD	Q5T7P8	SYT6
589.1	Isoform 3 of Ankyrin repeat don	5.24E+05	0.00E+00	-5.24E+05	-5.24E+05	NULL	AD	Q6ZTN6-3	ANKRD13D
2099.1	Protein FAM134C	5.24E+05	0.00E+00	-5.24E+05	-5.24E+05	NULL	AD	Q86VR2	FAM134C
2366.1	Coiled-coil domain-containing p	5.25E+05	0.00E+00	-5.25E+05	-5.25E+05	NULL	AD	Q502W7	CCDC38
2216.1	Isoform 2 of PDZ domain-contai	5.28E+05	0.00E+00	-5.28E+05	-5.28E+05	NULL	AD	Q5EBL8-2	PDZD11
572.1	Adenine phosphoribosyltransfer	5.30E+05	0.00E+00	-5.30E+05	-5.30E+05	NULL	AD	P07741	APRT
3028.1	Glycosyltransferase 8 domain-co	5.31E+05	0.00E+00	-5.31E+05	-5.31E+05	NULL	AD	Q9H1C3	GLT8D2
2519.1	E3 ubiquitin-protein ligase RBBP	5.32E+05	0.00E+00	-5.32E+05	-5.32E+05	NULL	AD	Q7Z6E9	RBBP6
2280.1	Leucine-rich repeat-containing p	5.38E+05	0.00E+00	-5.38E+05	-5.38E+05	NULL	AD	Q8N456	LRRC18
1857.1	RNA-binding protein 47	5.41E+05	0.00E+00	-5.41E+05	-5.41E+05	NULL	AD	A0AV96	RBM47
3207.1	A disintegrin and metalloprotein	5.41E+05	0.00E+00	-5.41E+05	-5.41E+05	NULL	AD	Q8TE57	ADAMTS16
1606.1	Inositol hexakisphosphate and d	5.45E+05	0.00E+00	-5.45E+05	-5.45E+05	NULL	AD	Q6PFW1	PPIP5K1
1114.1	RNA-binding protein 43	5.52E+05	0.00E+00	-5.52E+05	-5.52E+05	NULL	AD	Q6ZSC3	RBM43
3015.1	Mesoderm posterior protein 2	5.53E+05	0.00E+00	-5.53E+05	-5.53E+05	NULL	AD	Q0VG99	MESP2
2100.1	Thyroid adenoma-associated pr	5.56E+05	0.00E+00	-5.56E+05	-5.56E+05	NULL	AD	Q6YHU6	THADA
1347.1	Lipase maturation factor 2	5.70E+05	0.00E+00	-5.70E+05	-5.70E+05	NULL	AD	Q9BU23	LMF2
3005.1	Dual specificity protein phospha	5.72E+05	0.00E+00	-5.72E+05	-5.72E+05	NULL	AD	Q8WTR2	DUSP19

675.1	Protocadherin gamma-C5	5.76E+05	0.00E+00	-5.76E+05	-5.76E+05	NULL	AD	Q9Y5F6	PCDHGC5
2916.1	Isoform 4 of Unconventional my	5.76E+05	0.00E+00	-5.76E+05	-5.76E+05	NULL	AD	B2RTY4-4	MYO9A
932.1	V-type proton ATPase 16 kDa pr	5.77E+05	0.00E+00	-5.77E+05	-5.77E+05	NULL	AD	P27449	ATP6V0C
3025.1	Atherin	5.79E+05	0.00E+00	-5.79E+05	-5.79E+05	NULL	AD	Q6SPF0	SAMD1
1901.1	Olfactory receptor 51Q1	5.89E+05	0.00E+00	-5.89E+05	-5.89E+05	NULL	AD	Q8NH59	OR51Q1
1241.1	Isoform 2 of Zinc finger protein	5.91E+05	0.00E+00	-5.91E+05	-5.91E+05	NULL	AD	Q6NUN9-2	ZNF746
624.1	Tubulin-folding cofactor B	5.95E+05	0.00E+00	-5.95E+05	-5.95E+05	NULL	AD	Q99426	TBCB
3091.1	UPF0461 protein C5orf24	5.96E+05	0.00E+00	-5.96E+05	-5.96E+05	NULL	AD	Q7Z6I8	C5orf24
1359.1	Isoform 2 of Protein ELYS	6.00E+05	0.00E+00	-6.00E+05	-6.00E+05	NULL	AD	Q8WYP5-2	AHCTF1
687.1	Niban-like protein 1	6.10E+05	0.00E+00	-6.10E+05	-6.10E+05	NULL	AD	Q96TA1	FAM129B
875.1	Cation-independent mannose-6	6.10E+05	0.00E+00	-6.10E+05	-6.10E+05	NULL	AD	P11717	IGF2R
3204.1	Charged multivesicular body pro	6.19E+05	0.00E+00	-6.19E+05	-6.19E+05	NULL	AD	Q9HD42	CHMP1A
858.1	Protein KTI12 homolog	6.44E+05	0.00E+00	-6.44E+05	-6.44E+05	NULL	AD	Q96EK9	WDR52
1332.1	Isoform 2 of WD repeat-contain	6.49E+05	0.00E+00	-6.49E+05	-6.49E+05	NULL	AD	Q96MT7-2	WDR52
2630.1	Hephaestin-like protein 1	6.50E+05	0.00E+00	-6.50E+05	-6.50E+05	NULL	AD	Q6MZM0	HEPHL1
1361.1	Negative elongation factor C/D	6.54E+05	0.00E+00	-6.54E+05	-6.54E+05	NULL	AD	Q8IXH7	NELFCD
904.1	E3 ubiquitin-protein ligase CHIP	6.56E+05	0.00E+00	-6.56E+05	-6.56E+05	NULL	AD	Q9UNE7	STUB1
546.1	Thioredoxin reductase 1, cytopla	6.59E+05	0.00E+00	-6.59E+05	-6.59E+05	NULL	AD	Q16881	TXNRD1
1325.1	Inactive pancreatic lipase-relate	6.60E+05	0.00E+00	-6.60E+05	-6.60E+05	NULL	AD	P54315	PNLIPRP1
3259.1	Cartilage intermediate layer pro	6.69E+05	0.00E+00	-6.69E+05	-6.69E+05	NULL	AD	K7EPJ4	CILP2
2465.1	SAM domain-containing protein	6.70E+05	0.00E+00	-6.70E+05	-6.70E+05	NULL	AD	F8WAA1	SAMSN1
1070.1	Coiled-coil domain-containing p	6.75E+05	0.00E+00	-6.75E+05	-6.75E+05	NULL	AD	Q8NCX0	CCDC150
1168.1	Deoxycytidine kinase	6.82E+05	0.00E+00	-6.82E+05	-6.82E+05	NULL	AD	D6RFG8	DCK
2681.1	Polycystic kidney disease 2-like	6.96E+05	0.00E+00	-6.96E+05	-6.96E+05	NULL	AD	Q9NZM6	PKD2L2
1662.1	E3 ubiquitin-protein ligase NEDI	6.99E+05	0.00E+00	-6.99E+05	-6.99E+05	NULL	AD	Q96PU5	NEDD4L
1004.1	Glutathione S-transferase Mu 3	7.01E+05	0.00E+00	-7.01E+05	-7.01E+05	NULL	AD	P21266	GSTM3
474.1	UDP-glucose 6-dehydrogenase	7.14E+05	0.00E+00	-7.14E+05	-7.14E+05	NULL	AD	O60701	UGDH
438.1	Serine/threonine-protein kinase	7.19E+05	0.00E+00	-7.19E+05	-7.19E+05	NULL	AD	Q9UHD2	TBK1
1511.1	Poly(ADP-ribose) glycohydrolase	7.21E+05	0.00E+00	-7.21E+05	-7.21E+05	NULL	AD	Q9NX46	ADPRHL2
537.1	Ataxin-10	7.24E+05	0.00E+00	-7.24E+05	-7.24E+05	NULL	AD	Q9UBB4	ATXN10
3186.1	DNA-directed RNA polymerase I	7.40E+05	0.00E+00	-7.40E+05	-7.40E+05	NULL	AD	O95602	POLR1A
883.1	Isoform 2 of Extended synaptota	7.42E+05	0.00E+00	-7.42E+05	-7.42E+05	NULL	AD	Q9BSJ8-2	ESYT1
2479.1	Major facilitator superfamily do	7.65E+05	0.00E+00	-7.65E+05	-7.65E+05	NULL	AD	Q8NBP5	MFSD9
1842.1	Kinesin-like protein KLP6	7.74E+05	0.00E+00	-7.74E+05	-7.74E+05	NULL	AD	B7ZC32	KLP6
2629.1	Zinc finger protein 660	7.92E+05	0.00E+00	-7.92E+05	-7.92E+05	NULL	AD	Q6AZW8	ZNF660

3044.1	Neogenin	8.02E+05	0.00E+00	-8.02E+05	-8.02E+05	NULL	AD	Q92859	NEO1
2456.1	Prefoldin subunit 5	8.04E+05	0.00E+00	-8.04E+05	-8.04E+05	NULL	AD	Q99471	PFDN5
1169.1	Acetoacetyl-CoA synthetase	8.07E+05	0.00E+00	-8.07E+05	-8.07E+05	NULL	AD	Q86V21	AACS
3019.1	Olfactory receptor 10A4	8.13E+05	0.00E+00	-8.13E+05	-8.13E+05	NULL	AD	Q9H209	OR10A4
2489.1	Pleckstrin homology domain-co	8.15E+05	0.00E+00	-8.15E+05	-8.15E+05	NULL	AD	E9PKC0	PLEKHA7
1660.1	Lysosomal acid phosphatase	8.17E+05	0.00E+00	-8.17E+05	-8.17E+05	NULL	AD	P11117	ACP2
514.1	Unconventional myosin-XVIIIa	8.32E+05	0.00E+00	-8.32E+05	-8.32E+05	NULL	AD	J3KNX9	MYO18A
2937.1	Isoform Flip of Glutamate recep	8.90E+05	0.00E+00	-8.90E+05	-8.90E+05	NULL	AD	P42263-2	GRIA3
1922.1	Olfactory receptor 51B6	9.09E+05	0.00E+00	-9.09E+05	-9.09E+05	NULL	AD	Q9H340	OR51B6
20.4	Putative heat shock protein HSP	9.10E+05	0.00E+00	-9.10E+05	-9.10E+05	NULL	AD	Q58FG1	HSP90AA4P
1522.1	Isoform 4 of Dynein heavy chain	9.17E+05	0.00E+00	-9.17E+05	-9.17E+05	NULL	AD	Q0VDD8-4	DNAH14
2690.1	Frizzled-8	9.33E+05	0.00E+00	-9.33E+05	-9.33E+05	NULL	AD	Q9H461	FZD8
427.1	EF-hand domain-containing pro	9.64E+05	0.00E+00	-9.64E+05	-9.64E+05	NULL	AD	Q96C19	EFHD2
1420.1	Probable tRNA(His) guanylyltrar	9.64E+05	0.00E+00	-9.64E+05	-9.64E+05	NULL	AD	Q9NWX6	THG1L
706.1	Glutathione synthetase	9.70E+05	0.00E+00	-9.70E+05	-9.70E+05	NULL	AD	P48637	GSS
1549.1	Chromodomain-helicase-DNA-b	9.76E+05	0.00E+00	-9.76E+05	-9.76E+05	NULL	AD	O14647	CHD2
3057.1	Caspase recruitment domain-co	9.81E+05	0.00E+00	-9.81E+05	-9.81E+05	NULL	AD	Q9BXL6	CARD14
318.3	Fidgetin-like protein 1	1.00E+06	0.00E+00	-1.00E+06	-1.00E+06	NULL	AD	Q6PIW4	FIGNL1
1994.1	Coiled-coil domain-containing p	1.01E+06	0.00E+00	-1.01E+06	-1.01E+06	NULL	AD	Q5T0U0	CCDC122
2766.1	Nexilin	1.01E+06	0.00E+00	-1.01E+06	-1.01E+06	NULL	AD	Q0ZGT2	NEXN
710.1	Tropomodulin-3	1.02E+06	0.00E+00	-1.02E+06	-1.02E+06	NULL	AD	Q9NYL9	TMOD3
1831.1	Piwi-like protein 1	1.03E+06	0.00E+00	-1.03E+06	-1.03E+06	NULL	AD	Q96J94	PIWIL1
3150.1	1-phosphatidylinositol 4,5-bisph	1.03E+06	0.00E+00	-1.03E+06	-1.03E+06	NULL	AD	Q4KWH8	PLCH1
488.1	Titin	1.03E+06	0.00E+00	-1.03E+06	-1.03E+06	NULL	AD	Q8WZ42	TTN
2534.1	Mitogen-activated protein kinas	1.05E+06	0.00E+00	-1.05E+06	-1.05E+06	NULL	AD	P53778	MAPK12
2701.1	Histone acetyltransferase KAT6I	1.06E+06	0.00E+00	-1.06E+06	-1.06E+06	NULL	AD	Q8WYB5	KAT6B
630.1	Annexin A11	1.07E+06	0.00E+00	-1.07E+06	-1.07E+06	NULL	AD	P50995	ANXA11
1072.1	Kit ligand	1.07E+06	0.00E+00	-1.07E+06	-1.07E+06	NULL	AD	P21583	KITLG
1055.1	NEU3 protein	1.09E+06	0.00E+00	-1.09E+06	-1.09E+06	NULL	AD	A8K327	NEU3
1062.1	Sister chromatid cohesion prote	1.09E+06	0.00E+00	-1.09E+06	-1.09E+06	NULL	AD	Q29RF7	PDS5A
1309.1	A disintegrin and metalloprotei	1.09E+06	0.00E+00	-1.09E+06	-1.09E+06	NULL	AD	Q8TE56	ADAMTS17
753.1	Isoform 6 of Calpastatin	1.12E+06	0.00E+00	-1.12E+06	-1.12E+06	NULL	AD	P20810-6	CAST
1125.1	ADP-ribosylation factor 3	1.13E+06	0.00E+00	-1.13E+06	-1.13E+06	NULL	AD	F5H423	ARF3
2862.1	Thiamine-triphosphatase	1.14E+06	0.00E+00	-1.14E+06	-1.14E+06	NULL	AD	Q9BU02	THTPA
1098.1	6-pyruvoyl tetrahydrobiopterin	1.15E+06	0.00E+00	-1.15E+06	-1.15E+06	NULL	AD	Q03393	PTS

1012.1	Isoform 3 of BEN domain-contai	1.27E+06	0.00E+00	-1.27E+06	-1.27E+06	NULL	AD	Q6ZU67-3	BEND4
585.1	IST1 homolog	1.31E+06	0.00E+00	-1.31E+06	-1.31E+06	NULL	AD	P53990	IST1
950.1	Nuclear transcription factor Y su	1.33E+06	0.00E+00	-1.33E+06	-1.33E+06	NULL	AD	Q13952	NFYC
1537.1	Receptor expression-enhancing	1.33E+06	0.00E+00	-1.33E+06	-1.33E+06	NULL	AD	Q6NUK4	REEP3
517.1	m7GpppX diphosphatase	1.34E+06	0.00E+00	-1.34E+06	-1.34E+06	NULL	AD	Q96C86	DCPS
580.1	UV excision repair protein RAD2	1.35E+06	0.00E+00	-1.35E+06	-1.35E+06	NULL	AD	P54727	RAD23B
2920.1	Coiled-coil domain-containing p	1.37E+06	0.00E+00	-1.37E+06	-1.37E+06	NULL	AD	Q8IWA6	CCDC60
14.3	Myosin-14	1.39E+06	0.00E+00	-1.39E+06	-1.39E+06	NULL	AD	G8JLL9	MYH14
565.1	Protein FAM83H	1.39E+06	0.00E+00	-1.39E+06	-1.39E+06	NULL	AD	Q6ZRV2	FAM83H
1841.1	SURP and G-patch domain-conta	1.44E+06	0.00E+00	-1.44E+06	-1.44E+06	NULL	AD	Q8IWZ8	SUGP1
975.1	DNA mismatch repair protein M	1.46E+06	0.00E+00	-1.46E+06	-1.46E+06	NULL	AD	P43246	MSH2
770.1	Reticulocalbin-1	1.52E+06	0.00E+00	-1.52E+06	-1.52E+06	NULL	AD	Q15293	RCN1
1081.1	Coronin	1.52E+06	0.00E+00	-1.52E+06	-1.52E+06	NULL	AD	P57737	CORO7
530.1	Annexin A7	1.53E+06	0.00E+00	-1.53E+06	-1.53E+06	NULL	AD	P20073	ANXA7
1970.1	Aldo-keto reductase family 1 me	1.55E+06	0.00E+00	-1.55E+06	-1.55E+06	NULL	AD	P17516	AKR1C4
731.1	Reticulocalbin-2	1.62E+06	0.00E+00	-1.62E+06	-1.62E+06	NULL	AD	F8WCY5	RCN2
558.1	Charged multivesicular body pro	1.70E+06	0.00E+00	-1.70E+06	-1.70E+06	NULL	AD	Q9UQN3	CHMP2B
931.1	V-set domain containing T cell a	1.75E+06	0.00E+00	-1.75E+06	-1.75E+06	NULL	AD	Q5T2L0	VTCN1
818.1	Diablo homolog, mitochondrial	1.76E+06	0.00E+00	-1.76E+06	-1.76E+06	NULL	AD	Q9NR28	DIABLO
1908.1	Zinc finger protein 350	1.89E+06	0.00E+00	-1.89E+06	-1.89E+06	NULL	AD	Q9GZX5	ZNF350
1064.1	Spondin-2	1.97E+06	0.00E+00	-1.97E+06	-1.97E+06	NULL	AD	Q9BUD6	SPON2
12.2	Isoform 3 of Alpha-actinin-1	2.23E+06	0.00E+00	-2.23E+06	-2.23E+06	NULL	AD	P12814-3	ACTN1
1801.1	Phospholipid scramblase 2	2.49E+06	0.00E+00	-2.49E+06	-2.49E+06	NULL	AD	Q9NRY7	PLSCR2
272.1	ATPase WRNIP1	2.68E+06	0.00E+00	-2.68E+06	-2.68E+06	NULL	AD	Q96S55	WRNIP1
1340.1	Protein DEK	2.69E+06	0.00E+00	-2.69E+06	-2.69E+06	NULL	AD	P35659	DEK
2020.1	Isoform 2 of Beta-parvin	2.76E+06	0.00E+00	-2.76E+06	-2.76E+06	NULL	AD	Q9HBI1-2	PARVB
680.1	Myoglobin	2.87E+06	0.00E+00	-2.87E+06	-2.87E+06	NULL	AD	P02144	MB
1087.1	Probable ATP-dependent RNA h	2.87E+06	0.00E+00	-2.87E+06	-2.87E+06	NULL	AD	F5GZS0	DHX36
336.1	Serine/threonine-protein kinase	2.90E+06	0.00E+00	-2.90E+06	-2.90E+06	NULL	AD	F8WBA3	PRKD1
1.3	Epiplakin	3.00E+06	0.00E+00	-3.00E+06	-3.00E+06	NULL	AD	E9PPU0	EPPK1
318.1	Vacuolar protein sorting-associa	3.03E+06	0.00E+00	-3.03E+06	-3.03E+06	NULL	AD	O75351	VPS4B
642.1	Sequestosome-1	3.07E+06	0.00E+00	-3.07E+06	-3.07E+06	NULL	AD	Q13501	SQSTM1
2420.1	Cell division cycle 7-related prot	3.43E+06	0.00E+00	-3.43E+06	-3.43E+06	NULL	AD	O00311	CDC7
2072.1	Tubulin epsilon chain	3.44E+06	0.00E+00	-3.44E+06	-3.44E+06	NULL	AD	Q9UJT0	TUBE1
416.1	Calpain small subunit 1	3.86E+06	0.00E+00	-3.86E+06	-3.86E+06	NULL	AD	K7ELJ7	CAPNS1

284.1	Vacuolar protein sorting-associa	4.42E+06	0.00E+00	-4.42E+06	-4.42E+06	NULL	AD	Q9NP79	VTA1
466.1	Programmed cell death protein	4.51E+06	0.00E+00	-4.51E+06	-4.51E+06	NULL	AD	O75340	PDCD6
321.1	Inorganic pyrophosphatase	4.65E+06	0.00E+00	-4.65E+06	-4.65E+06	NULL	AD	Q15181	PPA1
382.1	Tubulin-specific chaperone A	5.27E+06	0.00E+00	-5.27E+06	-5.27E+06	NULL	AD	E5RJD8	TBCA
126.1	Cytospin-A	6.73E+06	0.00E+00	-6.73E+06	-6.73E+06	NULL	AD	Q69YQ0	SPECC1L
163.1	Copine-3	7.68E+06	0.00E+00	-7.68E+06	-7.68E+06	NULL	AD	O75131	CPNE3
98.1	Drebrin	1.33E+07	0.00E+00	-1.33E+07	-1.33E+07	NULL	AD	A8MV58	DBN1
63.1	X-ray repair cross-complementi	1.37E+07	0.00E+00	-1.37E+07	-1.37E+07	NULL	AD	P13010	XRCC5
14.2	Myosin-10	1.37E+07	0.00E+00	-1.37E+07	-1.37E+07	NULL	AD	P35580	MYH10
61.1	X-ray repair cross-complementi	1.48E+07	0.00E+00	-1.48E+07	-1.48E+07	NULL	AD	P12956	XRCC6
7.1	Isoform 8 of Filamin-B	2.67E+07	0.00E+00	-2.67E+07	-2.67E+07	NULL	AD	O75369-8	FLNB
21.2	Tropomyosin alpha-1 chain	3.14E+07	0.00E+00	-3.14E+07	-3.14E+07	NULL	AD	B7Z596	TPM1
17.1	Probable ATP-dependent RNA h	1.23E+04	6.68E+07	5.42E+03	5.42E+03	10.00	8.60	H3BLZ8	DDX17
17.2	ATP-dependent RNA helicase DI	4.21E+04	5.59E+07	1.33E+03	1.33E+03	10.00	7.19	O00571	DDX3X
15.1	Non-POU domain-containing oc	4.68E+05	2.68E+08	5.73E+02	5.73E+02	10.00	6.35	Q15233	NONO
237.1	Catenin delta-1	1.56E+04	7.69E+06	4.94E+02	4.94E+02	10.00	6.20	O60716	CTNND1
1047.1	Protein furry homolog	2.96E+03	1.38E+06	4.66E+02	4.66E+02	10.00	6.14	Q5TBA9	FRY
113.1	40S ribosomal protein S3	3.27E+04	1.47E+07	4.50E+02	4.50E+02	10.00	6.11	P23396	RPS3
131.1	60S ribosomal protein L30	2.80E+04	1.17E+07	4.18E+02	4.18E+02	10.00	6.03	P62888	RPL30
184.1	Pyruvate carboxylase, mitochon	3.07E+04	7.65E+06	2.49E+02	2.49E+02	10.00	5.52	P11498	PC
451.1	Protein BUD31 homolog	1.32E+04	2.68E+06	2.02E+02	2.02E+02	10.00	5.31	P41223	BUD31
106.1	Delta(3,5)-Delta(2,4)-dienoyl-Co	3.89E+04	7.69E+06	1.98E+02	1.98E+02	10.00	5.29	Q13011	ECH1
946.1	Protein AHNAK2	4.77E+03	9.24E+05	1.94E+02	1.94E+02	10.00	5.27	Q8IVF2	AHNAK2
11.1	Heterogeneous nuclear ribonuc	7.09E+05	1.37E+08	1.93E+02	1.93E+02	10.00	5.26	P52272	HNRNPM
188.1	60S ribosomal protein L10a	3.86E+04	7.14E+06	1.85E+02	1.85E+02	10.00	5.22	P62906	RPL10A
778.1	Isoform 2 of Prostatic acid phos	1.85E+03	3.15E+05	1.70E+02	1.70E+02	10.00	5.14	P15309-2	ACPP
17.3	Probable ATP-dependent RNA h	1.41E+05	2.33E+07	1.66E+02	1.66E+02	10.00	5.11	P17844	DDX5
649.1	Polypeptide N-acetylgalactosam	5.52E+03	6.85E+05	1.24E+02	1.24E+02	10.00	4.82	Q7Z7M9	GALNT5
245.1	Mitochondrial import inner mer	1.98E+05	2.33E+07	1.18E+02	1.18E+02	10.00	4.77	Q9Y5L4	TIMM13
252.1	Methylcrotonoyl-CoA carboxyla	1.12E+04	1.22E+06	1.09E+02	1.09E+02	10.00	4.69	Q96RQ3	MCCC1
513.1	Small nuclear ribonucleoprotein	2.65E+04	2.80E+06	1.06E+02	1.06E+02	10.00	4.66	P62314	SNRPD1
260.1	Splicing factor 3A subunit 1	3.72E+04	3.88E+06	1.04E+02	1.04E+02	10.00	4.65	Q15459	SF3A1
90.1	Keratin, type I cytoskeletal 9	1.22E+05	1.19E+07	9.73E+01	9.73E+01	10.00	4.58	P35527	KRT9
450.1	Isoform 5 of Nuclear pore comp	5.90E+03	5.70E+05	9.65E+01	9.65E+01	10.00	4.57	P35658-5	NUP214
191.1	Putative RNA-binding protein 3	1.83E+05	1.73E+07	9.45E+01	9.45E+01	10.00	4.55	P98179	RBM3

1391.1	Remodeling and spacing factor :	6.47E+03	5.74E+05	8.87E+01	8.87E+01	10.00	4.49	Q96T23	RSF1
129.1	Elongation factor Tu, mitochonc	1.02E+05	8.90E+06	8.72E+01	8.72E+01	10.00	4.47	P49411	TUFM
1261.1	Protein disulfide-isomerase-like	1.02E+04	8.64E+05	8.46E+01	8.46E+01	10.00	4.44	Q8N807	PDILT
83.1	Heterogeneous nuclear ribonuc	3.04E+05	2.44E+07	8.02E+01	8.02E+01	10.00	4.38	P22626	HNRNPA2B1
409.1	Eukaryotic translation initiation	2.51E+04	1.80E+06	7.18E+01	7.18E+01	10.00	4.27	B5ME19	EIF3CL
66.2	RNA-binding protein FUS	3.99E+05	2.50E+07	6.26E+01	6.26E+01	10.00	4.14	H3BPE7	FUS
371.1	Peptidyl-prolyl cis-trans isomera	4.99E+04	2.85E+06	5.71E+01	5.71E+01	10.00	4.04	Q00688	FKBP3
175.1	60S ribosomal protein L38	3.42E+05	1.93E+07	5.65E+01	5.65E+01	10.00	4.03	P63173	RPL38
77.1	Malate dehydrogenase, mitocho	3.92E+05	2.14E+07	5.46E+01	5.46E+01	10.00	4.00	P40926	MDH2
174.1	Cathepsin D	1.92E+05	1.04E+07	5.41E+01	5.41E+01	10.00	3.99	P07339	CTSD
1563.1	40S ribosomal protein S24	3.77E+04	1.94E+06	5.14E+01	5.14E+01	10.00	3.94	P62847	RPS24
570.1	Palmitoyl-protein thioesterase 1	4.24E+04	2.07E+06	4.89E+01	4.89E+01	10.00	3.89	P50897	PPT1
779.1	Proteasome subunit beta type-4	1.19E+04	5.70E+05	4.78E+01	4.78E+01	10.00	3.87	P28070	PSMB4
1457.1	Myosin-6	4.65E+04	2.18E+06	4.69E+01	4.69E+01	10.00	3.85	P13533	MYH6
1085.1	E3 ubiquitin-protein ligase Topo	6.50E+03	3.02E+05	4.65E+01	4.65E+01	10.00	3.84	Q9NS56	TOPORS
838.1	Pre-mRNA-processing factor 19	9.21E+03	4.21E+05	4.57E+01	4.57E+01	10.00	3.82	Q9UMS4	PRPF19
576.1	Translation machinery-associate	6.22E+04	2.58E+06	4.15E+01	4.15E+01	10.00	3.73	Q9Y2S6	TMA7
96.2	Far upstream element-binding p	1.10E+05	4.56E+06	4.14E+01	4.14E+01	10.00	3.72	B4DT31	FUBP1
267.1	40S ribosomal protein S2	2.48E+05	1.02E+07	4.12E+01	4.12E+01	10.00	3.72	P15880	RPS2
442.1	60S acidic ribosomal protein P2	1.07E+05	4.41E+06	4.12E+01	4.12E+01	10.00	3.72	P05387	RPLP2
939.1	Probable E3 ubiquitin-protein lig	5.13E+03	2.00E+05	3.90E+01	3.90E+01	10.00	3.66	F8VWT9	HECTD4
112.1	ATP synthase subunit alpha, mit	3.35E+05	1.30E+07	3.88E+01	3.88E+01	10.00	3.66	P25705	ATP5A1
164.1	60S ribosomal protein L28	5.39E+05	2.03E+07	3.77E+01	3.77E+01	10.00	3.63	P46779	RPL28
582.1	26S proteasome non-ATPase reg	2.98E+04	1.09E+06	3.65E+01	3.65E+01	10.00	3.60	O00487	PSMD14
2.1	Desmoplakin	1.66E+06	5.85E+07	3.53E+01	3.53E+01	10.00	3.56	P15924	DSP
274.1	V-type proton ATPase catalytic s	3.63E+05	1.27E+07	3.50E+01	3.50E+01	10.00	3.56	P38606	ATP6V1A
47.1	Protein transport protein Sec23	6.01E+05	2.08E+07	3.46E+01	3.46E+01	10.00	3.54	Q15437	SEC23B
1027.1	Fer-1-like protein 4	5.87E+03	2.03E+05	3.46E+01	3.46E+01	10.00	3.54	A9Z1Z3	FER1L4
223.1	40S ribosomal protein S28	3.64E+05	1.20E+07	3.30E+01	3.30E+01	10.00	3.50	P62857	RPS28
1166.1	Nuclear valosin-containing prote	1.13E+05	3.72E+06	3.29E+01	3.29E+01	10.00	3.49	O15381	NVL
97.1	Nucleophosmin	9.64E+05	3.16E+07	3.28E+01	3.28E+01	10.00	3.49	P06748	NPM1
210.1	Stress-70 protein, mitochondria	1.97E+05	6.44E+06	3.27E+01	3.27E+01	10.00	3.49	P38646	HSPA9
611.1	Cytochrome c oxidase subunit 6	1.98E+04	6.47E+05	3.27E+01	3.27E+01	10.00	3.49	P14854	COX6B1
264.1	Chromobox protein homolog 3	2.55E+05	8.29E+06	3.25E+01	3.25E+01	10.00	3.48	Q13185	CBX3
417.1	Proteasome subunit alpha type-	8.47E+03	2.73E+05	3.22E+01	3.22E+01	10.00	3.47	P25788	PSMA3

586.1	Eukaryotic translation initiation	1.94E+04	6.23E+05	3.21E+01	3.21E+01	10.00	3.47	Q14152	EIF3A
211.1	40S ribosomal protein S12	1.10E+05	3.53E+06	3.20E+01	3.20E+01	10.00	3.47	P25398	RPS12
117.1	60S ribosomal protein L13	1.00E+06	3.16E+07	3.14E+01	3.14E+01	10.00	3.45	P26373	RPL13
225.1	40S ribosomal protein S6	8.37E+05	2.57E+07	3.07E+01	3.07E+01	10.00	3.42	P62753	RPS6
228.1	Beta-hexosaminidase subunit be	1.44E+05	4.38E+06	3.05E+01	3.05E+01	10.00	3.42	P07686	HEXB
108.1	Microtubule-associated protein	2.15E+05	6.42E+06	2.98E+01	2.98E+01	10.00	3.39	P46821	MAP1B
317.1	Pre-mRNA-processing factor 6	7.69E+04	2.25E+06	2.92E+01	2.92E+01	10.00	3.38	O94906	PRPF6
166.1	Heterogeneous nuclear ribonuc	3.17E+05	9.20E+06	2.90E+01	2.90E+01	10.00	3.37	Q14103	HNRNPD
100.1	tRNA-splicing ligase RtcB homol	4.29E+05	1.23E+07	2.87E+01	2.87E+01	10.00	3.36	Q9Y3I0	C22orf28
115.1	Isoform 2 of Regulator of chrom	8.59E+05	2.38E+07	2.77E+01	2.77E+01	10.00	3.32	P18754-2	RCC1
235.1	Puromycin-sensitive aminopept	9.18E+04	2.52E+06	2.75E+01	2.75E+01	10.00	3.31	P55786	NPEPPS
722.1	V-type proton ATPase subunit G	6.98E+04	1.85E+06	2.65E+01	2.65E+01	10.00	3.28	O75348	ATP6V1G1
283.1	Probable ATP-dependent RNA h	1.19E+05	3.14E+06	2.63E+01	2.63E+01	10.00	3.27	P26196	DDX6
47.2	Protein transport protein Sec23.	7.20E+05	1.82E+07	2.53E+01	2.53E+01	10.00	3.23	Q15436	SEC23A
428.1	DNA replication licensing factor	5.20E+04	1.30E+06	2.50E+01	2.50E+01	10.00	3.22	P25205	MCM3
370.1	Splicing factor 3A subunit 3	1.26E+05	3.16E+06	2.50E+01	2.50E+01	10.00	3.22	Q12874	SF3A3
556.1	Eukaryotic translation initiation	6.22E+04	1.55E+06	2.49E+01	2.49E+01	10.00	3.22	P47813	EIF1AX
797.1	NEDD8-activating enzyme E1 re	1.08E+04	2.67E+05	2.47E+01	2.47E+01	10.00	3.21	A6NCK0	NAE1
2095.1	11-cis retinol dehydrogenase	1.01E+04	2.48E+05	2.47E+01	2.47E+01	10.00	3.20	Q92781	RDH5
347.1	Cysteine and glycine-rich protei	1.78E+04	4.38E+05	2.46E+01	2.46E+01	10.00	3.20	P21291	CSRP1
495.1	Protein FLJ22184	2.93E+04	7.21E+05	2.46E+01	2.46E+01	10.00	3.20	F5H1R7	FLJ22184
28.1	Isocitrate dehydrogenase [NADI	1.81E+06	4.42E+07	2.44E+01	2.44E+01	10.00	3.20	P48735	IDH2
261.1	Isoform Long of Proteasome sul	3.86E+05	8.82E+06	2.28E+01	2.28E+01	10.00	3.13	P25786-2	PSMA1
783.1	Eukaryotic translation initiation	2.04E+04	4.46E+05	2.18E+01	2.18E+01	10.00	3.08	P78344	EIF4G2
343.1	Mesencephalic astrocyte-derive	2.28E+05	4.94E+06	2.16E+01	2.16E+01	10.00	3.07	P55145	MANF
669.1	Regulation of nuclear pre-mRNA	7.31E+03	1.52E+05	2.08E+01	2.08E+01	10.00	3.03	Q96P16	RPRD1A
187.1	Isoform 2 of Protein disulfide-is	4.49E+05	9.06E+06	2.02E+01	2.02E+01	10.00	3.00	Q15084-2	PDIA6
71.1	Guanine nucleotide-binding pro	8.68E+05	1.75E+07	2.02E+01	2.02E+01	10.00	3.00	P63244	GNB2L1
263.1	28 kDa heat- and acid-stable ph	1.52E+05	3.05E+06	2.01E+01	2.01E+01	10.00	3.00	Q13442	PDAP1
232.1	Valine--tRNA ligase	2.64E+05	5.22E+06	1.98E+01	1.98E+01	10.00	2.98	P26640	VARS
394.1	Aspartate aminotransferase, cyt	2.48E+04	4.61E+05	1.86E+01	1.86E+01	10.00	2.92	P17174	GOT1
229.1	Staphylococcal nuclease domair	2.06E+05	3.81E+06	1.85E+01	1.85E+01	10.00	2.92	Q7KZF4	SND1
694.1	Cullin-4B	7.30E+04	1.34E+06	1.84E+01	1.84E+01	10.00	2.91	Q13620	CUL4B
843.1	Transcription initiation factor TI	1.28E+05	2.31E+06	1.80E+01	1.80E+01	10.00	2.89	Q5VWG9	TAF3
179.1	Creatine kinase U-type, mitochc	7.41E+05	1.32E+07	1.78E+01	1.78E+01	10.00	2.88	P12532	CKMT1A

381.1	Microtubule-associated protein	1.82E+05	3.19E+06	1.75E+01	1.75E+01	10.00	2.86	Q15691	MAPRE1
201.1	ATP synthase subunit beta, mitc	5.64E+05	9.86E+06	1.75E+01	1.75E+01	10.00	2.86	P06576	ATP5B
216.1	40S ribosomal protein S18	4.70E+05	8.15E+06	1.73E+01	1.73E+01	10.00	2.85	P62269	RPS18
485.1	Serrate RNA effector molecule h	8.19E+04	1.40E+06	1.71E+01	1.71E+01	10.00	2.84	Q9BXP5	SRRT
1362.1	CDK5 regulatory subunit-associa	2.34E+05	3.85E+06	1.65E+01	1.65E+01	10.00	2.80	F8WBJ0	CDK5RAP2
637.1	UPF0556 protein C19orf10	1.22E+05	1.96E+06	1.60E+01	1.60E+01	10.00	2.77	Q969H8	C19orf10
1134.1	Protein pelota homolog	4.48E+04	7.00E+05	1.56E+01	1.56E+01	10.00	2.75	Q9BRX2	PELO
705.1	Protein phosphatase 1M	7.42E+06	1.15E+08	1.55E+01	1.55E+01	10.00	2.74	B7XGB9	ppm1m
670.1	Selenocysteine-specific elongati	1.61E+04	2.47E+05	1.54E+01	1.54E+01	10.00	2.73	P57772	EEFSEC
31.1	Microtubule-associated protein	1.62E+06	2.47E+07	1.53E+01	1.53E+01	10.00	2.73	E7EVA0	MAP4
172.1	Bifunctional glutamate/proline-	1.57E+05	2.37E+06	1.51E+01	1.51E+01	10.00	2.72	P07814	EPRS
560.1	AP-1 complex subunit gamma-1	1.26E+05	1.89E+06	1.50E+01	1.50E+01	10.00	2.71	B3KXW5	AP1G1
1485.1	Leukotriene A-4 hydrolase	7.95E+03	1.18E+05	1.48E+01	1.48E+01	10.00	2.70	P09960	LTA4H
158.1	Adenylate kinase 2, mitochondr	4.12E+05	6.12E+06	1.48E+01	1.48E+01	10.00	2.70	P54819	AK2
160.1	Leucine-rich repeat-containing p	4.86E+05	7.02E+06	1.44E+01	1.44E+01	10.00	2.67	Q8N1G4	LRRC47
176.1	40S ribosomal protein S9	6.58E+05	9.38E+06	1.43E+01	1.43E+01	10.00	2.66	P46781	RPS9
212.1	60S ribosomal protein L35	4.30E+06	6.08E+07	1.42E+01	1.42E+01	10.00	2.65	P42766	RPL35
518.1	Thioredoxin-dependent peroxid	6.00E+05	8.47E+06	1.41E+01	1.41E+01	10.00	2.65	P30048	PRDX3
542.1	Acylamino-acid-releasing enzym	1.83E+05	2.53E+06	1.38E+01	1.38E+01	10.00	2.63	C9JIF9	APEH
149.1	40S ribosomal protein S3a	7.76E+05	1.06E+07	1.37E+01	1.37E+01	10.00	2.61	P61247	RPS3A
388.1	Isoform 2 of Proteasome activat	2.53E+05	3.45E+06	1.37E+01	1.37E+01	10.00	2.61	P61289-2	PSME3
702.1	Non-histone chromosomal prot	1.66E+05	2.25E+06	1.36E+01	1.36E+01	10.00	2.61	P05114	HMGN1
418.1	Flap endonuclease 1	6.37E+05	8.58E+06	1.35E+01	1.35E+01	10.00	2.60	P39748	FEN1
266.1	60S ribosomal protein L23	5.83E+05	7.80E+06	1.34E+01	1.34E+01	10.00	2.59	P62829	RPL23
142.1	Isoform 3 of Elongation factor 1	8.05E+05	1.07E+07	1.33E+01	1.33E+01	10.00	2.59	P29692-3	EEF1D
243.1	Serine/threonine-protein phosp	4.23E+05	5.58E+06	1.32E+01	1.32E+01	10.00	2.58	P62140	PPP1CB
186.1	Heterogeneous nuclear ribonuc	1.13E+06	1.48E+07	1.31E+01	1.31E+01	10.00	2.57	P09651	HNRNPA1
598.1	ATP-dependent RNA helicase DI	8.38E+04	1.09E+06	1.30E+01	1.30E+01	10.00	2.57	Q86XP3	DDX42
516.1	U2 snRNP-associated SURP mot	1.08E+05	1.40E+06	1.29E+01	1.29E+01	10.00	2.56	E7ET15	U2SURP
464.1	Proteasome subunit beta type-6	5.40E+04	6.90E+05	1.28E+01	1.28E+01	10.00	2.55	P28072	PSMB6
639.1	Keratin, type I cuticular Ha1	1.43E+06	1.81E+07	1.27E+01	1.27E+01	10.00	2.54	Q15323	KRT31
65.1	DNA-dependent protein kinase	8.35E+05	1.05E+07	1.26E+01	1.26E+01	10.00	2.53	P78527	PRKDC
644.1	Vacuolar protein sorting-associa	1.23E+04	1.51E+05	1.22E+01	1.22E+01	10.00	2.50	Q709C8	VPS13C
439.1	40S ribosomal protein S30	3.71E+05	4.48E+06	1.21E+01	1.21E+01	10.00	2.49	E9PR30	FAU
497.1	Hematological and neurological	2.60E+04	3.05E+05	1.17E+01	1.17E+01	10.00	2.46	B4DLH4	HN1L

114.1	Proliferation-associated protein	7.66E+05	8.97E+06	1.17E+01	1.17E+01	10.00	2.46	Q9UQ80	PA2G4
178.1	NHP2 non-histone chromosome	1.41E+06	1.62E+07	1.15E+01	1.15E+01	10.00	2.44	B1AHD1	NHP2L1
18.1	Propionyl-CoA carboxylase alpha	3.96E+06	4.34E+07	1.10E+01	1.10E+01	10.00	2.39	P05165	PCCA
3.4	Keratin, type II cytoskeletal 2 ep	2.45E+05	2.64E+06	1.08E+01	1.08E+01	10.00	2.38	P35908	KRT2
289.1	Tripeptidyl-peptidase 1	4.50E+05	4.81E+06	1.07E+01	1.07E+01	10.00	2.37	O14773	TPP1
22.1	Propionyl-CoA carboxylase beta	3.26E+06	3.40E+07	1.04E+01	1.04E+01	10.00	2.35	E7EX59	PCCB
622.1	Proteasome subunit alpha type-	5.87E+04	6.00E+05	1.02E+01	1.02E+01	10.00	2.32	O14818	PSMA7
552.1	Eukaryotic translation initiation	2.02E+06	2.04E+07	1.01E+01	1.01E+01	10.00	2.31	K7EM18	EIF1
659.1	Developmentally-regulated GTP	9.18E+04	9.14E+05	9.96E+00	9.96E+00	9.96	2.30	Q9Y295	DRG1
651.1	AP-1 complex subunit beta-1	2.10E+05	2.08E+06	9.89E+00	9.89E+00	9.89	2.29	Q10567	AP1B1
51.3	Ezrin	1.01E+05	9.88E+05	9.77E+00	9.77E+00	9.77	2.28	P15311	EZR
664.1	Selenide, water dikinase 1	2.72E+05	2.53E+06	9.30E+00	9.30E+00	9.30	2.23	P49903	SEPHS1
72.1	10 kDa heat shock protein, mito	3.16E+06	2.94E+07	9.29E+00	9.29E+00	9.29	2.23	P61604	HSPE1
568.1	60S ribosomal protein L3-like	4.87E+04	4.52E+05	9.28E+00	9.28E+00	9.28	2.23	Q92901	RPL3L
647.1	Ubiquilin-4	8.04E+04	7.39E+05	9.19E+00	9.19E+00	9.19	2.22	Q9NRR5	UBQLN4
254.1	6-phosphofructokinase	1.25E+06	1.15E+07	9.17E+00	9.17E+00	9.17	2.22	P08237	PFKM
436.1	Ribonuclease inhibitor	2.54E+05	2.30E+06	9.06E+00	9.06E+00	9.06	2.20	P13489	RNH1
848.1	WD repeat-containing protein W	6.80E+04	6.14E+05	9.02E+00	9.02E+00	9.02	2.20	Q9P2S5	WRAP73
204.1	Exportin-2	6.15E+05	5.37E+06	8.74E+00	8.74E+00	8.74	2.17	P55060	CSE1L
330.1	Aspartate aminotransferase, mi	1.36E+05	1.19E+06	8.72E+00	8.72E+00	8.72	2.17	P00505	GOT2
804.1	Histone-lysine N-methyltransfer	4.95E+04	4.17E+05	8.42E+00	8.42E+00	8.42	2.13	Q9NR48	ASH1L
593.1	Serine-threonine kinase recepto	4.52E+04	3.77E+05	8.34E+00	8.34E+00	8.34	2.12	B4DNJ6	STRAP
79.1	Keratin, type II cytoskeletal 1	2.02E+06	1.67E+07	8.26E+00	8.26E+00	8.26	2.11	P04264	KRT1
350.1	Pyrroline-5-carboxylate reducta	5.14E+05	4.24E+06	8.26E+00	8.26E+00	8.26	2.11	Q53H96	PYCRL
1154.1	Isoform 2 of Nesprin-2	8.40E+04	6.89E+05	8.20E+00	8.20E+00	8.20	2.10	Q8WXH0-2	SYNE2
280.1	Eukaryotic translation initiation	2.46E+05	2.01E+06	8.18E+00	8.18E+00	8.18	2.10	P05198	EIF2S1
297.1	Prosaposin	1.01E+06	8.16E+06	8.07E+00	8.07E+00	8.07	2.09	B1AVU8	PSAP
239.1	Talin-1	6.50E+05	5.22E+06	8.03E+00	8.03E+00	8.03	2.08	Q9Y490	TLN1
445.1	Cancer-related nucleoside-triph	7.70E+04	6.01E+05	7.80E+00	7.80E+00	7.80	2.05	Q9BSD7	NTPCR
222.1	Inosine-5'-monophosphate dehy	8.66E+05	6.52E+06	7.53E+00	7.53E+00	7.53	2.02	P12268	IMPDH2
515.1	Programmed cell death protein	1.86E+05	1.39E+06	7.47E+00	7.47E+00	7.47	2.01	Q53EL6	PDCD4
353.1	Isoform 2 of C-terminal-binding	9.31E+04	6.95E+05	7.46E+00	7.46E+00	7.46	2.01	P56545-2	CTBP2
413.1	Eukaryotic peptide chain release	4.09E+05	3.05E+06	7.45E+00	7.45E+00	7.45	2.01	P62495	ETF1
43.2	116 kDa U5 small nuclear ribonu	1.21E+05	9.02E+05	7.43E+00	7.43E+00	7.43	2.01	Q15029	EFTUD2
231.1	Isoform 2 of Threonine--tRNA lig	3.31E+05	2.45E+06	7.41E+00	7.41E+00	7.41	2.00	P26639-2	TARS

478.1	Keratin, type I cuticular Ha7	2.42E+06	1.78E+07	7.37E+00	7.37E+00	7.37	2.00	O76014	KRT37
786.1	Mitogen-activated protein kinas	1.66E+05	1.22E+06	7.36E+00	7.36E+00	7.36	2.00	P27361	MAPK3
483.1	Nuclear mitotic apparatus prote	7.18E+04	5.26E+05	7.33E+00	7.33E+00	7.33	1.99	Q14980	NUMA1
602.1	SUMO-conjugating enzyme UBC	6.51E+05	4.72E+06	7.25E+00	7.25E+00	7.25	1.98	B0QYN7	UBE2I
479.1	HLA-B associated transcript 3, is	8.86E+04	6.40E+05	7.23E+00	7.23E+00	7.23	1.98	G3V1Z5	NULL
623.1	Epithelial splicing regulatory prc	7.85E+04	5.66E+05	7.21E+00	7.21E+00	7.21	1.98	Q6NXG1	ESRP1
965.1	Zinc finger protein 207	1.16E+05	8.28E+05	7.12E+00	7.12E+00	7.12	1.96	O43670	ZNF207
895.1	S-adenosylmethionine synthase	2.87E+05	2.03E+06	7.07E+00	7.07E+00	7.07	1.96	Q00266	MAT1A
367.1	E3 SUMO-protein ligase RanBP2	5.39E+05	3.76E+06	6.98E+00	6.98E+00	6.98	1.94	P49792	RANBP2
306.1	Isoform 5 of Splicing factor 1	1.50E+06	1.03E+07	6.88E+00	6.88E+00	6.88	1.93	Q15637-5	SF1
1348.1	Isoform 1 of Metabotropic gluta	1.33E+05	9.18E+05	6.88E+00	6.88E+00	6.88	1.93	P41594-2	GRM5
395.1	DNA damage-binding protein 1	3.81E+04	2.58E+05	6.77E+00	6.77E+00	6.77	1.91	Q16531	DDB1
484.1	Proteasome activator complex s	1.72E+05	1.14E+06	6.63E+00	6.63E+00	6.63	1.89	Q06323	PSME1
482.1	Isoform 2 of TAR DNA-binding p	2.39E+05	1.56E+06	6.54E+00	6.54E+00	6.54	1.88	Q13148-2	TARDBP
362.1	Importin subunit beta-1	2.24E+05	1.45E+06	6.46E+00	6.46E+00	6.46	1.87	Q14974	KPNB1
1316.1	Kallistatin	2.38E+04	1.53E+05	6.44E+00	6.44E+00	6.44	1.86	P29622	SERPINA4
400.1	Isoform 2 of Partner of Y14 and	3.81E+04	2.45E+05	6.43E+00	6.43E+00	6.43	1.86	Q9BRP8-2	WIBG
535.1	Isoform 2 of Serine/threonine-p	4.07E+05	2.60E+06	6.38E+00	6.38E+00	6.38	1.85	P63151-2	PPP2R2A
236.1	Hsc70-interacting protein	6.19E+05	3.89E+06	6.29E+00	6.29E+00	6.29	1.84	P50502	ST13
1139.1	Superoxide dismutase [Cu-Zn]	2.52E+05	1.58E+06	6.28E+00	6.28E+00	6.28	1.84	P00441	SOD1
299.1	ATP-citrate synthase	1.51E+05	9.44E+05	6.27E+00	6.27E+00	6.27	1.84	P53396	ACLY
902.1	Laminin subunit beta-3	7.52E+04	4.68E+05	6.22E+00	6.22E+00	6.22	1.83	Q13751	LAMB3
36.1	Transitional endoplasmic reticul	3.61E+06	2.20E+07	6.10E+00	6.10E+00	6.10	1.81	P55072	VCP
227.1	Aspartate--tRNA ligase, cytoplas	5.16E+05	3.14E+06	6.09E+00	6.09E+00	6.09	1.81	P14868	DARS
844.1	Dynein light chain roadblock-typ	9.89E+04	5.98E+05	6.05E+00	6.05E+00	6.05	1.80	B1AKR6	DYNLRB1
592.1	60 kDa SS-A/Ro ribonucleoprote	1.51E+05	8.95E+05	5.94E+00	5.94E+00	5.94	1.78	P10155	TROVE2
137.1	40S ribosomal protein S11	2.93E+06	1.74E+07	5.93E+00	5.93E+00	5.93	1.78	P62280	RPS11
54.1	Peptidyl-prolyl cis-trans isomera	5.66E+06	3.34E+07	5.90E+00	5.90E+00	5.90	1.77	P23284	PPIB
16.1	60 kDa heat shock protein, mito	1.70E+07	1.00E+08	5.89E+00	5.89E+00	5.89	1.77	P10809	HSPD1
369.1	Profilin-2	1.05E+05	6.13E+05	5.83E+00	5.83E+00	5.83	1.76	P35080	PFN2
356.1	Isoform 2 of Regulator of nonse	3.64E+05	2.11E+06	5.80E+00	5.80E+00	5.80	1.76	Q92900-2	UPF1
305.1	26S protease regulatory subunit	5.62E+05	3.23E+06	5.75E+00	5.75E+00	5.75	1.75	P35998	PSMC2
548.1	Isoform 2 of Histone-binding pr	2.09E+05	1.20E+06	5.73E+00	5.73E+00	5.73	1.75	Q16576-2	RBBP7
132.1	Poly(rC)-binding protein 1	6.25E+05	3.49E+06	5.59E+00	5.59E+00	5.59	1.72	Q15365	PCBP1
1176.1	Mucin-16	1.08E+05	6.00E+05	5.55E+00	5.55E+00	5.55	1.71	Q8WXI7	MUC16

295.1	Alcohol dehydrogenase [NADP(-	8.46E+05	4.65E+06	5.50E+00	5.50E+00	5.50	1.70	P14550	AKR1A1
426.1	26S protease regulatory subunit	5.90E+05	3.23E+06	5.47E+00	5.47E+00	5.47	1.70	P43686	PSMC4
194.1	Isoform 2 of General vesicular tı	1.18E+06	6.13E+06	5.18E+00	5.18E+00	5.18	1.65	O60763-2	USO1
603.1	Microtubule-actin cross-linking	3.44E+05	1.75E+06	5.09E+00	5.09E+00	5.09	1.63	H3BQK9	MACF1
699.1	Clathrin light chain B	9.43E+04	4.78E+05	5.07E+00	5.07E+00	5.07	1.62	P09497	CLTB
89.1	Cytochrome c	6.38E+06	3.23E+07	5.06E+00	5.06E+00	5.06	1.62	P99999	CYCS
130.1	Thymocyte nuclear protein 1	2.72E+06	1.37E+07	5.04E+00	5.04E+00	5.04	1.62	Q9P016	THYN1
214.1	Alpha-2-HS-glycoprotein	9.77E+05	4.85E+06	4.97E+00	4.97E+00	4.97	1.60	P02765	AHSG
871.1	ATPase inhibitor, mitochondrial	6.56E+05	3.19E+06	4.86E+00	4.86E+00	4.86	1.58	Q9UII2	ATPIF1
1279.1	GDP-fucose protein O-fucosyltra	5.90E+04	2.80E+05	4.74E+00	4.74E+00	4.74	1.56	Q9H488	POFUT1
718.1	Galactokinase	1.32E+05	6.27E+05	4.73E+00	4.73E+00	4.73	1.55	B4E1G6	GALK1
110.1	Isoform 2 of Coatomer subunit a	2.69E+06	1.26E+07	4.69E+00	4.69E+00	4.69	1.54	P53621-2	COPA
254.3	Isoform 2 of 6-phosphofructokir	5.05E+05	2.36E+06	4.67E+00	4.67E+00	4.67	1.54	P17858-2	PFKL
1021.1	Sodium/potassium/calcium excl	4.80E+04	2.22E+05	4.62E+00	4.62E+00	4.62	1.53	Q71RS6	SLC24A5
1010.1	Cystatin-C	6.43E+04	2.96E+05	4.60E+00	4.60E+00	4.60	1.53	P01034	CST3
522.1	Cytoplasmic dynein 1 light inter	7.47E+04	3.42E+05	4.58E+00	4.58E+00	4.58	1.52	O43237	DYNC1LI2
551.1	Isoform 2 of Cytoplasmic FMR1-	3.21E+05	1.47E+06	4.57E+00	4.57E+00	4.57	1.52	Q7L576-2	CYFIP1
96.1	Far upstream element-binding p	7.40E+05	3.34E+06	4.51E+00	4.51E+00	4.51	1.51	Q92945	KHSRP
1401.1	Serine/threonine-protein phosp	5.25E+04	2.36E+05	4.50E+00	4.50E+00	4.50	1.50	P67775	PPP2CA
349.1	Methionine--tRNA ligase, cytopl	1.10E+06	4.95E+06	4.49E+00	4.49E+00	4.49	1.50	P56192	MARS
476.1	Eukaryotic translation initiation	2.07E+05	9.14E+05	4.41E+00	4.41E+00	4.41	1.48	E7EX17	EIF4B
500.1	V(D)J recombination-activating p	1.76E+05	7.62E+05	4.33E+00	4.33E+00	4.33	1.47	P15918	RAG1
121.1	Elongation factor 1-gamma	3.57E+06	1.54E+07	4.32E+00	4.32E+00	4.32	1.46	B4DTG2	EEF1G
967.1	Isoform 1 of Tudor domain-cont	2.22E+05	9.59E+05	4.31E+00	4.31E+00	4.31	1.46	Q8NAT2-1	TDRD5
1393.1	Folliculin-interacting protein 1	3.35E+03	1.44E+04	4.30E+00	4.30E+00	4.30	1.46	E9PCH4	FNIP1
124.1	Nucleolin	2.68E+06	1.14E+07	4.26E+00	4.26E+00	4.26	1.45	P19338	NCL
136.1	26S proteasome non-ATPase reg	7.15E+05	3.03E+06	4.24E+00	4.24E+00	4.24	1.44	Q13200	PSMD2
169.1	Keratin, type I cytoskeletal 10	9.94E+05	4.13E+06	4.16E+00	4.16E+00	4.16	1.42	P13645	KRT10
352.1	26S protease regulatory subunit	1.28E+06	5.31E+06	4.14E+00	4.14E+00	4.14	1.42	P17980	PSMC3
487.1	Protein phosphatase 1G	1.09E+05	4.51E+05	4.13E+00	4.13E+00	4.13	1.42	O15355	PPM1G
132.2	Isoform 2 of Poly(rC)-binding pr	1.41E+06	5.66E+06	4.03E+00	4.03E+00	4.03	1.39	Q15366-2	PCBP2
1127.1	Isoform Alpha-2 of Glucocortico	1.55E+05	5.94E+05	3.84E+00	3.84E+00	3.84	1.35	P04150-3	NR3C1
1.1	Plectin	5.07E+07	1.94E+08	3.83E+00	3.83E+00	3.83	1.34	Q15149	PLEC
242.1	Calcyclin-binding protein	1.14E+06	4.37E+06	3.83E+00	3.83E+00	3.83	1.34	Q9HB71	CACYBP
999.1	Malate dehydrogenase	3.60E+05	1.37E+06	3.81E+00	3.81E+00	3.81	1.34	P40925	MDH1

614.1	ADP-ribosylation factor-like prot	7.47E+05	2.84E+06	3.80E+00	3.80E+00	3.80	1.34	F8VYN9	ARL1
265.1	Polyadenylate-binding protein 1	1.07E+06	4.07E+06	3.80E+00	3.80E+00	3.80	1.33	P11940	PABPC1
926.1	ATP-binding cassette sub-family	1.19E+04	4.51E+04	3.78E+00	3.78E+00	3.78	1.33	Q8NE71	ABCF1
69.1	Spliceosome RNA helicase DDX3	2.78E+06	1.05E+07	3.78E+00	3.78E+00	3.78	1.33	F8VQ10	DDX39B
1375.1	CD180 antigen	9.31E+04	3.49E+05	3.75E+00	3.75E+00	3.75	1.32	Q99467	CD180
105.1	Histone H4	7.46E+06	2.78E+07	3.73E+00	3.73E+00	3.73	1.32	P62805	HIST1H4A
510.1	Structural maintenance of chror	2.32E+05	8.61E+05	3.72E+00	3.72E+00	3.72	1.31	A6NHR9	SMCHD1
446.1	Dihydropyrimidinase-related pro	4.40E+05	1.63E+06	3.71E+00	3.71E+00	3.71	1.31	Q16555	DPYSL2
954.1	Cytoplasmic dynein 1 light interi	1.92E+04	7.07E+04	3.68E+00	3.68E+00	3.68	1.30	Q9Y6G9	DYNC1LI1
877.1	FYN-binding protein	1.75E+05	6.27E+05	3.58E+00	3.58E+00	3.58	1.28	O15117	FYB
1059.1	60S ribosomal protein L31	1.86E+05	6.63E+05	3.56E+00	3.56E+00	3.56	1.27	B7Z4C8	RPL31
1223.1	Probable phospholipid-transpor	1.20E+05	4.20E+05	3.49E+00	3.49E+00	3.49	1.25	Q9P241	ATP10D
10.1	Clathrin heavy chain 1	1.28E+07	4.45E+07	3.47E+00	3.47E+00	3.47	1.24	Q00610	CLTC
1014.1	DNA fragmentation factor subui	1.98E+05	6.84E+05	3.45E+00	3.45E+00	3.45	1.24	O00273	DFFA
863.1	Isoform 6 of Ankyrin repeat and	1.85E+05	6.32E+05	3.42E+00	3.42E+00	3.42	1.23	Q8IWZ3-6	ANKHD1
761.1	Transcription factor BTF3	9.29E+05	3.16E+06	3.40E+00	3.40E+00	3.40	1.22	P20290	BTF3
329.1	Isoleucine--tRNA ligase, cytoplas	6.04E+05	2.04E+06	3.38E+00	3.38E+00	3.38	1.22	P41252	IARS
107.1	Archain 1, isoform CRA_a	4.48E+06	1.51E+07	3.37E+00	3.37E+00	3.37	1.22	B0YIW6	ARCN1
1467.1	SET and MYND domain-containi	8.94E+04	2.97E+05	3.32E+00	3.32E+00	3.32	1.20	Q9H7B4	SMYD3
93.1	Peptidyl-prolyl cis-trans isomera	7.29E+06	2.37E+07	3.25E+00	3.25E+00	3.25	1.18	Q02790	FKBP4
874.1	Centromere-associated protein	1.09E+05	3.54E+05	3.24E+00	3.24E+00	3.24	1.18	Q02224	CENPE
254.2	6-phosphofructokinase	1.52E+06	4.89E+06	3.22E+00	3.22E+00	3.22	1.17	Q01813	PFKP
1041.1	Myosin-2	8.46E+04	2.71E+05	3.20E+00	3.20E+00	3.20	1.16	Q9UKX2	MYH2
473.1	Splicing factor 3B subunit 3	3.10E+05	9.88E+05	3.18E+00	3.18E+00	3.18	1.16	Q15393	SF3B3
315.1	Chloride intracellular channel pr	9.26E+05	2.94E+06	3.17E+00	3.17E+00	3.17	1.16	O00299	CLIC1
39.1	Alpha-enolase	1.69E+07	5.27E+07	3.12E+00	3.12E+00	3.12	1.14	P06733	ENO1
87.1	40S ribosomal protein S13	1.06E+07	3.28E+07	3.09E+00	3.09E+00	3.09	1.13	P62277	RPS13
173.1	Phosphoenolpyruvate carboxyki	1.64E+06	5.05E+06	3.08E+00	3.08E+00	3.08	1.13	Q16822	PCK2
29.1	D-3-phosphoglycerate dehydrog	1.42E+07	4.36E+07	3.08E+00	3.08E+00	3.08	1.13	O43175	PHGDH
294.1	Fructose-bisphosphate aldolase	2.15E+06	6.62E+06	3.07E+00	3.07E+00	3.07	1.12	P09972	ALDOC
311.1	Nucleoprotein TPR	9.67E+05	2.94E+06	3.04E+00	3.04E+00	3.04	1.11	P12270	TPR
660.1	Phosphoserine phosphatase	9.50E+04	2.88E+05	3.03E+00	3.03E+00	3.03	1.11	P78330	PSPH
1122.1	Isoform 5 of Nitric oxide synthas	7.65E+04	2.30E+05	3.01E+00	3.01E+00	3.01	1.10	P29475-5	NOS1
819.1	Multidrug resistance-associated	6.96E+04	2.09E+05	3.00E+00	3.00E+00	3.00	1.10	O15440	ABCC5
384.1	Oxysterol-binding protein 1	1.27E+05	3.75E+05	2.94E+00	2.94E+00	2.94	1.08	P22059	OSBP

94.1	Glyceraldehyde-3-phosphate de	2.85E+07	8.38E+07	2.94E+00	2.94E+00	2.94	1.08	P04406	GAPDH
821.1	Hsp90 co-chaperone Cdc37	2.67E+05	7.79E+05	2.92E+00	2.92E+00	2.92	1.07	Q16543	CDC37
51.2	Moesin	1.83E+06	5.33E+06	2.91E+00	2.91E+00	2.91	1.07	P26038	MSN
646.1	Hydroxyacylglutathione hydrola	2.65E+05	7.69E+05	2.90E+00	2.90E+00	2.90	1.07	Q16775	HAGH
1491.1	DNA polymerase	3.04E+05	8.77E+05	2.88E+00	2.88E+00	2.88	1.06	E7ETS8	POLE
44.2	Peroxiredoxin-2	2.25E+07	6.39E+07	2.83E+00	2.83E+00	2.83	1.04	P32119	PRDX2
307.1	Serine/threonine-protein kinase	1.55E+06	4.34E+06	2.81E+00	2.81E+00	2.81	1.03	Q13177	PAK2
800.1	Unconventional myosin-Va	9.09E+04	2.54E+05	2.79E+00	2.79E+00	2.79	1.03	F8W6H6	MYO5A
494.1	Putative RNA-binding protein Lu	4.68E+05	1.29E+06	2.76E+00	2.76E+00	2.76	1.01	Q9Y383	LUC7L2
140.1	Rab GDP dissociation inhibitor b	1.82E+06	5.01E+06	2.75E+00	2.75E+00	2.75	1.01	E7EU23	GDI2
20.5	Heat shock protein 75 kDa, mitc	3.36E+06	9.21E+06	2.74E+00	2.74E+00	2.74	1.01	Q12931	TRAP1
366.1	Eukaryotic translation initiation	4.00E+05	1.09E+06	2.72E+00	2.72E+00	2.72	1.00	Q15056	EIF4H
457.1	182 kDa tankyrase-1-binding pro	1.96E+05	5.32E+05	2.71E+00	2.71E+00	2.71	1.00	Q9C0C2	TNKS1BP1
947.1	V-type proton ATPase subunit E	9.49E+05	2.57E+06	2.71E+00	2.71E+00	2.71	1.00	P36543	ATP6V1E1
528.1	Enhancer of mRNA-decapping p	1.43E+06	3.84E+06	2.69E+00	2.69E+00	2.69	0.99	Q6P2E9	EDC4
550.1	Acetyl-CoA acetyltransferase, cy	3.48E+05	9.30E+05	2.67E+00	2.67E+00	2.67	0.98	B7Z233	ACAT2
241.1	V-type proton ATPase subunit B	3.94E+06	1.05E+07	2.66E+00	2.66E+00	2.66	0.98	P21281	ATP6V1B2
561.1	COP9 signalosome complex sub	2.19E+05	5.83E+05	2.66E+00	2.66E+00	2.66	0.98	D6RFN0	COPS4
300.1	Ribose-phosphate pyrophospho	1.84E+06	4.87E+06	2.65E+00	2.65E+00	2.65	0.97	P21108	PRPS1L1
1236.1	Ubiquitin carboxyl-terminal hyd	7.56E+04	2.00E+05	2.64E+00	2.64E+00	2.64	0.97	Q70CQ1	USP49
155.1	40S ribosomal protein S7	9.33E+06	2.45E+07	2.63E+00	2.63E+00	2.63	0.97	P62081	RPS7
725.1	Peptidyl-prolyl cis-trans isomera	3.40E+05	8.90E+05	2.62E+00	2.62E+00	2.62	0.96	K7EMU7	PIN1
219.1	Isoform 2 of Electron transfer fl	3.14E+06	8.23E+06	2.62E+00	2.62E+00	2.62	0.96	P38117-2	ETFB
1220.1	Zinc finger and BTB domain-con	1.99E+05	5.20E+05	2.61E+00	2.61E+00	2.61	0.96	P10074	ZBTB48
390.1	Eukaryotic translation initiation	9.74E+05	2.51E+06	2.58E+00	2.58E+00	2.58	0.95	Q04637	EIF4G1
739.1	Protein TFG	1.96E+05	4.92E+05	2.51E+00	2.51E+00	2.51	0.92	Q92734	TFG
652.1	CAD protein	1.60E+04	3.99E+04	2.50E+00	2.50E+00	2.50	0.92	P27708	CAD
125.1	Transcription elongation factor	6.40E+06	1.59E+07	2.48E+00	2.48E+00	2.48	0.91	P23193	TCEA1
103.1	Heat shock protein beta-1	1.51E+07	3.72E+07	2.47E+00	2.47E+00	2.47	0.90	P04792	HSPB1
1440.1	Actin-related protein 2/3 compl	1.37E+04	3.37E+04	2.45E+00	2.45E+00	2.45	0.90	O15143	ARPC1B
323.1	Peroxiredoxin-5, mitochondrial	1.93E+06	4.72E+06	2.44E+00	2.44E+00	2.44	0.89	P30044	PRDX5
2001.1	Ran-specific GTPase-activating p	2.08E+05	5.09E+05	2.44E+00	2.44E+00	2.44	0.89	F8WCY3	RANBP1
424.1	Leucine-rich repeat flightless-int	5.61E+04	1.37E+05	2.44E+00	2.44E+00	2.44	0.89	Q32MZ4	LRRFIP1
220.1	DNA replication licensing factor	2.01E+06	4.90E+06	2.44E+00	2.44E+00	2.44	0.89	P33993	MCM7
68.1	Fructose-bisphosphate aldolase	8.85E+06	2.15E+07	2.43E+00	2.43E+00	2.43	0.89	J3KPS3	ALDOA

324.1	26S protease regulatory subunit	7.14E+05	1.73E+06	2.42E+00	2.42E+00	2.42	0.89	P62195	PSMC5
42.1	Selenium-binding protein 1	5.18E+06	1.25E+07	2.41E+00	2.41E+00	2.41	0.88	Q13228	SELENBP1
511.1	Nicotinamide phosphoribosyltra	5.27E+05	1.27E+06	2.41E+00	2.41E+00	2.41	0.88	P43490	NAMPT
1324.1	Histone-lysine N-methyltransfer	9.53E+04	2.29E+05	2.40E+00	2.40E+00	2.40	0.88	Q8TEK3	DOT1L
2411.1	Isoform 3 of Oral-facial-digital sy	1.79E+05	4.29E+05	2.40E+00	2.40E+00	2.40	0.87	O75665-3	OFD1
862.1	Ubiquitin-like modifier-activatin	1.80E+05	4.30E+05	2.39E+00	2.39E+00	2.39	0.87	Q9GZZ9	UBA5
750.1	Isoform 3 of Calcium-binding mi	1.25E+05	2.97E+05	2.37E+00	2.37E+00	2.37	0.86	Q6KCM7-3	SLC25A25
641.1	Isoform 2 of Hematological and	1.25E+06	2.97E+06	2.37E+00	2.37E+00	2.37	0.86	Q9UK76-2	HN1
74.1	Adenosylhomocysteinase	6.02E+06	1.40E+07	2.33E+00	2.33E+00	2.33	0.84	P23526	AHCY
27.1	T-complex protein 1 subunit del	1.81E+07	4.17E+07	2.30E+00	2.30E+00	2.30	0.83	P50991	CCT4
58.2	L-lactate dehydrogenase B chair	8.67E+06	1.98E+07	2.28E+00	2.28E+00	2.28	0.83	P07195	LDHB
13.1	Pyruvate kinase isozymes M1/M	4.47E+07	1.02E+08	2.28E+00	2.28E+00	2.28	0.83	P14618	PKM
43.1	Elongation factor 2	7.10E+06	1.61E+07	2.27E+00	2.27E+00	2.27	0.82	P13639	EEF2
493.1	Macrophage migration inhibitor	2.40E+07	5.42E+07	2.26E+00	2.26E+00	2.26	0.82	P14174	MIF
532.1	dCTP pyrophosphatase 1	5.99E+05	1.35E+06	2.26E+00	2.26E+00	2.26	0.81	Q9H773	DCTPP1
1254.1	Tubby-related protein 1	1.85E+05	4.17E+05	2.25E+00	2.25E+00	2.25	0.81	O00294	TULP1
119.1	Ubiquitin-like modifier-activatin	5.20E+06	1.17E+07	2.25E+00	2.25E+00	2.25	0.81	P22314	UBA1
391.1	Thioredoxin domain-containing	9.35E+05	2.10E+06	2.25E+00	2.25E+00	2.25	0.81	Q9BRA2	TXNDC17
545.1	GMP synthase [glutamine-hydrc	4.38E+05	9.82E+05	2.24E+00	2.24E+00	2.24	0.81	P49915	GMPS
610.1	Phosphoglucomutase-1	4.04E+05	9.07E+05	2.24E+00	2.24E+00	2.24	0.81	P36871	PGM1
58.1	Isoform 3 of L-lactate dehydroge	6.02E+06	1.33E+07	2.21E+00	2.21E+00	2.21	0.79	P00338-3	LDHA
924.1	Protein unc-119 homolog B	4.68E+04	1.03E+05	2.20E+00	2.20E+00	2.20	0.79	A6NIH7	UNC119B
147.1	Eukaryotic translation initiation	3.80E+06	8.33E+06	2.19E+00	2.19E+00	2.19	0.78	P41091	EIF2S3
1321.1	RNA-binding protein Raly	2.80E+05	6.05E+05	2.16E+00	2.16E+00	2.16	0.77	Q9UKM9	RALY
206.1	Coatomer subunit beta	2.22E+06	4.74E+06	2.13E+00	2.13E+00	2.13	0.76	P53618	COPB1
754.1	Splicing factor U2AF 65 kDa sub	7.63E+05	1.62E+06	2.12E+00	2.12E+00	2.12	0.75	P26368	U2AF2
1587.1	Protein KIAA0368	6.69E+04	1.42E+05	2.12E+00	2.12E+00	2.12	0.75	J3KN16	KIAA0368
933.1	TBC1 domain family member 8	7.40E+04	1.56E+05	2.11E+00	2.11E+00	2.11	0.75	J3KQ40	TBC1D8
1631.1	Melanoma-associated antigen D	1.34E+06	2.83E+06	2.10E+00	2.10E+00	2.10	0.74	Q9UNF1	MAGED2
345.1	Proteasome subunit beta type-5	4.27E+05	8.97E+05	2.10E+00	2.10E+00	2.10	0.74	P28074	PSMB5
978.1	ADP-ribosylation factor 5	1.39E+05	2.90E+05	2.09E+00	2.09E+00	2.09	0.74	P84085	ARF5
562.1	High mobility group nucleosome	7.95E+05	1.66E+06	2.09E+00	2.09E+00	2.09	0.74	P82970	HMGN5
671.1	Casein kinase II subunit alpha	2.58E+06	5.28E+06	2.05E+00	2.05E+00	2.05	0.72	E7EU96	CSNK2A1
2146.1	E3 ubiquitin-protein ligase CHFR	5.72E+05	1.17E+06	2.04E+00	2.04E+00	2.04	0.72	Q96EP1	CHFR
609.1	Isoform 2 of COP9 signalosome	2.00E+05	4.08E+05	2.04E+00	2.04E+00	2.04	0.71	P61201-2	COPS2

127.1	Translational activator GCN1	1.70E+06	3.46E+06	2.04E+00	2.04E+00	2.04	0.71 Q92616	GCN1L1
46.1	Heterogeneous nuclear ribonuc	1.91E+07	3.88E+07	2.03E+00	2.03E+00	2.03	0.71 P61978	HNRNPK
273.1	Mitochondrial import receptor s	3.38E+06	6.81E+06	2.02E+00	2.02E+00	2.02	0.70 Q15785	TOMM34
820.1	Pro-cathepsin H	1.59E+05	3.19E+05	2.01E+00	2.01E+00	2.01	0.70 P09668	CTSH
331.1	Glutamate carboxypeptidase 2	1.37E+06	2.74E+06	1.99E+00	1.99E+00	1.99	0.69 Q04609	FOLH1
33.1	Protein disulfide-isomerase A3	1.52E+07	3.00E+07	1.98E+00	1.98E+00	1.98	0.68 P30101	PDIA3
884.1	Coatomer subunit epsilon	1.60E+06	3.15E+06	1.97E+00	1.97E+00	1.97	0.68 O14579	COPE
95.1	DNA-(apurinic or apyrimidinic si	1.68E+07	3.30E+07	1.97E+00	1.97E+00	1.97	0.68 P27695	APEX1
383.1	Histidine triad nucleotide-bindir	4.17E+06	8.15E+06	1.96E+00	1.96E+00	1.96	0.67 P49773	HINT1
971.1	Guanosine-3',5'-bis(diphosphate	4.79E+04	9.33E+04	1.95E+00	1.95E+00	1.95	0.67 Q8N4P3	HDDC3
868.1	Protein Shroom3	1.54E+05	2.99E+05	1.95E+00	1.95E+00	1.95	0.67 Q8TF72	SHROOM3
202.1	Coatomer subunit beta'	1.17E+06	2.26E+06	1.93E+00	1.93E+00	1.93	0.66 P35606	COPB2
5.1	Keratin, type I cytoskeletal 18	2.35E+08	4.52E+08	1.93E+00	1.93E+00	1.93	0.66 P05783	KRT18
972.1	Pescadillo homolog	1.07E+05	2.06E+05	1.92E+00	1.92E+00	1.92	0.65 B3KXD6	PES1
567.1	Protein NEDD8-MDP1	2.91E+05	5.59E+05	1.92E+00	1.92E+00	1.92	0.65 E9PS38	NEDD8-MDP1
26.1	Cytoplasmic dynein 1 heavy cha	7.19E+06	1.38E+07	1.92E+00	1.92E+00	1.92	0.65 Q14204	DYNC1H1
749.1	Midasin	1.37E+05	2.63E+05	1.91E+00	1.91E+00	1.91	0.65 Q9NU22	MDN1
51.1	Radixin	8.82E+05	1.68E+06	1.91E+00	1.91E+00	1.91	0.64 P35241	RDX
75.1	Cytosolic non-specific dipeptida	2.58E+06	4.91E+06	1.90E+00	1.90E+00	1.90	0.64 Q96KP4	CNDP2
217.1	Bifunctional purine biosynthesis	3.89E+06	7.38E+06	1.90E+00	1.90E+00	1.90	0.64 P31939	ATIC
325.1	Phosphatidylethanolamine-bind	2.27E+06	4.27E+06	1.88E+00	1.88E+00	1.88	0.63 P30086	PEBP1
9.2	Tubulin beta chain	1.10E+07	2.06E+07	1.87E+00	1.87E+00	1.87	0.63 P07437	TUBB
1461.1	Isoform 2 of Kinesin-like protein	1.09E+05	2.04E+05	1.87E+00	1.87E+00	1.87	0.63 Q12756-2	KIF1A
925.1	Isoform 2 of Melanoma-associat	1.00E+06	1.87E+06	1.86E+00	1.86E+00	1.86	0.62 Q8TD91-2	MAGEC3
177.1	Signal recognition particle 14 kD	1.23E+07	2.29E+07	1.86E+00	1.86E+00	1.86	0.62 P37108	SRP14
711.1	Adenosine kinase	1.66E+06	3.05E+06	1.84E+00	1.84E+00	1.84	0.61 P55263	ADK
248.1	Serine/threonine-protein phosp	8.70E+05	1.59E+06	1.83E+00	1.83E+00	1.83	0.60 P30153	PPP2R1A
402.1	26S proteasome non-ATPase re	1.03E+06	1.87E+06	1.81E+00	1.81E+00	1.81	0.60 O43242	PSMD3
990.1	Programmed cell death protein	2.89E+04	5.19E+04	1.79E+00	1.79E+00	1.79	0.58 Q9BRP1	PDCD2L
979.1	Talin-2	1.33E+04	2.38E+04	1.78E+00	1.78E+00	1.78	0.58 Q9Y4G6	TLN2
449.1	N-alpha-acetyltransferase 15, N	1.65E+05	2.90E+05	1.76E+00	1.76E+00	1.76	0.57 Q9BXJ9	NAA15
1038.1	Ras-like protein family member	3.44E+06	6.04E+06	1.76E+00	1.76E+00	1.76	0.56 Q6T310	RASL11A
748.1	Alpha-endosulfine	2.58E+05	4.51E+05	1.75E+00	1.75E+00	1.75	0.56 Q5T5H1	ENSA
566.1	Diacylglycerol kinase zeta	1.10E+06	1.92E+06	1.74E+00	1.74E+00	1.74	0.56 F5H033	DGKZ
914.1	Hepatoma-derived growth factc	9.64E+04	1.67E+05	1.73E+00	1.73E+00	1.73	0.55 Q7Z4V5	HDGFRP2

631.1	Prostaglandin E synthase 3	1.73E+05	2.99E+05	1.73E+00	1.73E+00	1.73	0.55	Q15185	PTGES3
604.1	Isoform 3 of Chitinase domain-c	2.94E+06	5.07E+06	1.72E+00	1.72E+00	1.72	0.54	Q9BWS9-3	CHID1
698.1	Isoform 2 of Glycogen synthase	8.70E+05	1.50E+06	1.72E+00	1.72E+00	1.72	0.54	P49841-2	GSK3B
805.1	Transcription factor BTF3 homo	3.56E+05	6.13E+05	1.72E+00	1.72E+00	1.72	0.54	Q96K17	BTF3L4
776.1	Vinculin	3.46E+05	5.93E+05	1.72E+00	1.72E+00	1.72	0.54	P18206	VCL
747.1	Proteasome subunit beta type-7	4.50E+05	7.69E+05	1.71E+00	1.71E+00	1.71	0.54	Q99436	PSMB7
1143.1	Alpha-galactosidase A	1.13E+05	1.93E+05	1.70E+00	1.70E+00	1.70	0.53	P06280	GLA
1107.1	Integrator complex subunit 12	2.00E+05	3.41E+05	1.70E+00	1.70E+00	1.70	0.53	Q96CB8	INTS12
218.1	Serum albumin	2.09E+07	3.56E+07	1.70E+00	1.70E+00	1.70	0.53	P02768	ALB
471.1	Translationally-controlled tumo	8.36E+05	1.42E+06	1.70E+00	1.70E+00	1.70	0.53	Q5W0H4	TPT1
681.1	Trafficking protein particle comp	1.81E+05	3.00E+05	1.66E+00	1.66E+00	1.66	0.51	O43617	TRAPPC3
28.2	Isocitrate dehydrogenase [NADP	2.90E+06	4.75E+06	1.64E+00	1.64E+00	1.64	0.49	O75874	IDH1
1242.1	Protein cTAGE-2	4.75E+05	7.73E+05	1.63E+00	1.63E+00	1.63	0.49	Q96RT6	CTAGE1
92.1	Transaldolase	5.42E+06	8.80E+06	1.62E+00	1.62E+00	1.62	0.48	P37837	TALDO1
1284.1	Rho GTPase-activating protein 1	4.30E+05	6.78E+05	1.58E+00	1.58E+00	1.58	0.46	Q6P4F7	ARHGAP11A
253.1	Protein transport protein Sec24	3.89E+06	6.11E+06	1.57E+00	1.57E+00	1.57	0.45	P53992	SEC24C
88.4	14-3-3 protein theta	3.32E+05	5.20E+05	1.57E+00	1.57E+00	1.57	0.45	P27348	YWHAQ
716.1	S-formylglutathione hydrolase	2.96E+06	4.64E+06	1.57E+00	1.57E+00	1.57	0.45	P10768	ESD
533.1	Vesicle-fusing ATPase	1.51E+06	2.35E+06	1.56E+00	1.56E+00	1.56	0.44	P46459	NSF
888.1	Peptidyl-prolyl cis-trans isomera	3.92E+04	6.11E+04	1.56E+00	1.56E+00	1.56	0.44	Q9Y3C6	PPIL1
38.1	Putative elongation factor 1-alp	3.46E+06	5.38E+06	1.56E+00	1.56E+00	1.56	0.44	Q5VTE0	EEF1A1P5
40.1	T-complex protein 1 subunit gar	1.08E+07	1.68E+07	1.55E+00	1.55E+00	1.55	0.44	P49368	CCT3
608.1	Replication protein A 70 kDa DN	2.70E+05	4.16E+05	1.54E+00	1.54E+00	1.54	0.43	P27694	RPA1
286.1	Eukaryotic translation initiation	6.64E+06	1.02E+07	1.54E+00	1.54E+00	1.54	0.43	I3L504	EIF5A
303.1	WD repeat-containing protein 1	2.08E+06	3.16E+06	1.52E+00	1.52E+00	1.52	0.42	O75083	WDR1
20.2	Heat shock protein HSP 90-beta	1.64E+07	2.47E+07	1.51E+00	1.51E+00	1.51	0.41	P08238	HSP90AB1
477.1	Eukaryotic peptide chain release	8.02E+05	1.20E+06	1.50E+00	1.50E+00	1.50	0.40	P15170	GSPT1
1554.1	Isoform 3 of PR domain zinc fing	9.27E+04	1.38E+05	1.49E+00	1.49E+00	1.49	0.40	Q9HAZ2-3	PRDM16
433.1	Coatomer subunit gamma-2	1.23E+06	1.83E+06	1.48E+00	1.48E+00	1.48	0.39	Q9UBF2	COPG2
1530.1	Zinc finger and BTB domain-con	1.56E+05	2.28E+05	1.46E+00	1.46E+00	1.46	0.38	O95625	ZBTB11
1155.1	Microtubule-associated protein	1.22E+05	1.79E+05	1.46E+00	1.46E+00	1.46	0.38	Q66K74	MAP1S
969.1	Protein transport protein Sec31	3.16E+05	4.63E+05	1.46E+00	1.46E+00	1.46	0.38	Q9NQW1	SEC31B
221.1	Calmodulin	8.89E+06	1.30E+07	1.46E+00	1.46E+00	1.46	0.38	E7EMB3	CALM2
157.1	Ankyrin-3	4.77E+06	6.95E+06	1.46E+00	1.46E+00	1.46	0.38	Q12955	ANK3
123.1	T-complex protein 1 subunit eta	6.95E+06	1.01E+07	1.45E+00	1.45E+00	1.45	0.37	Q99832	CCT7

520.1	Farnesyl pyrophosphate synthas	1.51E+05	2.18E+05	1.45E+00	1.45E+00	1.45	0.37	P14324	FDPS
8.1	Heat shock cognate 71 kDa prot	1.09E+08	1.58E+08	1.45E+00	1.45E+00	1.45	0.37	P11142	HSPA8
374.1	Coatomer subunit zeta-1	1.28E+06	1.85E+06	1.44E+00	1.44E+00	1.44	0.37	F8VVA7	COPZ1
1287.1	Zinc finger ZZ-type and EF-hand	2.10E+05	3.02E+05	1.44E+00	1.44E+00	1.44	0.36	O43149	ZZEF1
679.1	UMP-CMP kinase	1.25E+06	1.80E+06	1.44E+00	1.44E+00	1.44	0.36	P30085	CMPK1
3.1	Keratin, type II cytoskeletal 8	5.01E+08	7.18E+08	1.43E+00	1.43E+00	1.43	0.36	P05787	KRT8
677.1	Keratin, type II cuticular Hb1	3.31E+05	4.72E+05	1.43E+00	1.43E+00	1.43	0.36	Q14533	KRT81
88.2	14-3-3 protein beta/alpha	1.16E+06	1.65E+06	1.42E+00	1.42E+00	1.42	0.35	P31946	YWHAB
368.1	Lipopolysaccharide-responsive a	1.04E+06	1.46E+06	1.40E+00	1.40E+00	1.40	0.34	F5H1X8	LRBA
116.1	Matrin-3	5.49E+06	7.57E+06	1.38E+00	1.38E+00	1.38	0.32	A8MXP9	MATR3
2722.1	Inhibitor of nuclear factor kappa	4.91E+05	6.77E+05	1.38E+00	1.38E+00	1.38	0.32	O14920	IKBKB
276.1	Protein SET	1.61E+07	2.20E+07	1.37E+00	1.37E+00	1.37	0.31	Q01105	SET
20.3	Endoplasmin	1.32E+07	1.81E+07	1.37E+00	1.37E+00	1.37	0.31	P14625	HSP90B1
25.1	Fatty acid synthase	1.14E+07	1.56E+07	1.37E+00	1.37E+00	1.37	0.31	P49327	FASN
30.1	Triosephosphate isomerase	3.32E+07	4.52E+07	1.36E+00	1.36E+00	1.36	0.31	P60174	TPI1
1445.1	FERM domain-containing protei	1.96E+05	2.67E+05	1.36E+00	1.36E+00	1.36	0.31	Q9Y2L6	FRMD4B
197.1	RuvB-like 2	1.58E+06	2.14E+06	1.36E+00	1.36E+00	1.36	0.31	Q9Y230	RUVBL2
24.2	Tubulin alpha-4A chain	2.07E+06	2.80E+06	1.35E+00	1.35E+00	1.35	0.30	P68366	TUBA4A
949.1	KIAA0423, isoform CRA_a	1.43E+06	1.92E+06	1.35E+00	1.35E+00	1.35	0.30	G3XAE9	FAM179B
505.1	26S protease regulatory subunit	6.24E+05	8.38E+05	1.34E+00	1.34E+00	1.34	0.30	P62333	PSMC6
53.1	T-complex protein 1 subunit zet	1.40E+07	1.84E+07	1.32E+00	1.32E+00	1.32	0.28	P40227	CCT6A
38.2	Elongation factor 1-alpha 2	2.86E+06	3.76E+06	1.31E+00	1.31E+00	1.31	0.27	Q05639	EEF1A2
701.1	Xin actin-binding repeat-contair	1.35E+05	1.78E+05	1.31E+00	1.31E+00	1.31	0.27	J3KNB1	XIRP2
62.1	Peptidyl-prolyl cis-trans isomera	9.59E+07	1.26E+08	1.31E+00	1.31E+00	1.31	0.27	P62937	PPIA
183.1	Phosphoserine aminotransferas	2.40E+06	3.14E+06	1.31E+00	1.31E+00	1.31	0.27	Q9Y617	PSAT1
80.1	Transcription intermediary facto	1.16E+07	1.52E+07	1.31E+00	1.31E+00	1.31	0.27	Q13263	TRIM28
255.1	Coatomer subunit gamma-1	2.29E+06	3.00E+06	1.31E+00	1.31E+00	1.31	0.27	Q9Y678	COPG1
571.1	Symplekin	3.80E+05	4.96E+05	1.30E+00	1.30E+00	1.30	0.27	Q92797	SYMPK
508.1	DnaJ homolog subfamily C mem	2.08E+06	2.68E+06	1.29E+00	1.29E+00	1.29	0.25	O75937	DNAJC8
823.1	Isoform Long of Ubiquitin fusior	4.00E+05	5.13E+05	1.28E+00	1.28E+00	1.28	0.25	Q92890-1	UFD1L
760.1	Protein FAM50A	1.39E+05	1.72E+05	1.24E+00	1.24E+00	1.24	0.22	Q14320	FAM50A
70.1	Alanine--tRNA ligase, cytoplasm	4.25E+06	5.25E+06	1.24E+00	1.24E+00	1.24	0.21	P49588	AARS
1045.1	Isoform 3 of LIM and calponin h	4.55E+04	5.61E+04	1.23E+00	1.23E+00	1.23	0.21	Q9UPQ0-3	LIMCH1
81.1	Isoform 2 of Glucose-6-phospha	1.45E+07	1.77E+07	1.22E+00	1.22E+00	1.22	0.20	P06744-2	GPI
181.1	Isoform 2 of Multifunctional prc	5.42E+06	6.56E+06	1.21E+00	1.21E+00	1.21	0.19	P22234-2	PAICS

612.1	Pterin-4-alpha-carbinolamine de	7.38E+05	8.88E+05	1.20E+00	1.20E+00	1.20	0.18	P61457	PCBD1
120.1	Profilin-1	1.77E+07	2.12E+07	1.20E+00	1.20E+00	1.20	0.18	P07737	PFN1
498.1	Activator of 90 kDa heat shock p	8.22E+05	9.76E+05	1.19E+00	1.19E+00	1.19	0.17	O95433	AHSA1
930.1	Glycine dehydrogenase [decarb	1.59E+05	1.88E+05	1.18E+00	1.18E+00	1.18	0.17	P23378	GLDC
635.1	Myosin regulatory light chain 12	1.60E+06	1.89E+06	1.18E+00	1.18E+00	1.18	0.17	J3QRS3	MYL12A
49.1	T-complex protein 1 subunit eps	2.00E+07	2.36E+07	1.18E+00	1.18E+00	1.18	0.16	P48643	CCT5
815.1	E3 ubiquitin-protein ligase SMU	1.72E+05	2.02E+05	1.18E+00	1.18E+00	1.18	0.16	Q9HCE7	SMURF1
67.1	Protein disulfide-isomerase	9.76E+06	1.14E+07	1.17E+00	1.17E+00	1.17	0.16	P07237	P4HB
1301.1	Glycogen [starch] synthase, mus	7.04E+04	8.09E+04	1.15E+00	1.15E+00	1.15	0.14	P13807	GYS1
195.1	Hepatoma-derived growth facto	7.98E+06	9.16E+06	1.15E+00	1.15E+00	1.15	0.14	P51858	HDGF
85.1	14-3-3 protein epsilon	3.29E+07	3.77E+07	1.15E+00	1.15E+00	1.15	0.14	P62258	YWHAE
726.1	Signal transducer and activator	8.35E+05	9.58E+05	1.15E+00	1.15E+00	1.15	0.14	P40763	STAT3
489.1	Proteasome subunit alpha type-	1.42E+06	1.62E+06	1.14E+00	1.14E+00	1.14	0.14	P25789	PSMA4
199.1	Isoform 2 of DBIRD complex sub	3.53E+06	4.01E+06	1.14E+00	1.14E+00	1.14	0.13	Q8N163-2	KIAA1967
91.1	Asparagine--tRNA ligase, cytopla	9.24E+06	1.05E+07	1.14E+00	1.14E+00	1.14	0.13	O43776	NARS
230.1	Splicing factor 3B subunit 2	3.60E+06	4.07E+06	1.13E+00	1.13E+00	1.13	0.12	Q13435	SF3B2
626.1	Capping protein (Actin filament)	3.70E+05	4.15E+05	1.12E+00	1.12E+00	1.12	0.11	B1AK88	CAPZB
339.1	DNA replication licensing factor	1.05E+06	1.17E+06	1.11E+00	1.11E+00	1.11	0.11	P33991	MCM4
59.1	Isoform 2 of Neutral alpha-gluco	1.46E+07	1.60E+07	1.10E+00	1.10E+00	1.10	0.09	Q14697-2	GANAB
296.1	Programmed cell death protein	3.11E+06	3.42E+06	1.10E+00	1.10E+00	1.10	0.09	O14737	PDCD5
738.1	Transcription elongation factor	1.92E+05	2.10E+05	1.09E+00	1.09E+00	1.09	0.09	Q5H9L2	TCEAL5
270.1	Isoform 2 of AP-1 complex subu	7.26E+05	7.85E+05	1.08E+00	1.08E+00	1.08	0.08	Q9Y6Q5-2	AP1M2
1075.1	Serine/threonine-protein kinase	8.00E+04	8.63E+04	1.08E+00	1.08E+00	1.08	0.08	I3L0C1	SMG1
258.1	14-3-3 protein eta	9.22E+06	9.91E+06	1.07E+00	1.07E+00	1.07	0.07	Q04917	YWHAH
822.1	Kinesin-like protein KIF7	2.75E+05	2.93E+05	1.07E+00	1.07E+00	1.07	0.06	Q2M1P5	KIF7
519.1	26S proteasome non-ATPase reg	1.86E+06	1.98E+06	1.06E+00	1.06E+00	1.06	0.06	C9IZE4	PSMD6
312.1	14-3-3 protein sigma	9.00E+06	9.57E+06	1.06E+00	1.06E+00	1.06	0.06	P31947	SFN
78.1	Creatine kinase B-type	3.33E+07	3.52E+07	1.06E+00	1.06E+00	1.06	0.06	P12277	CKB
443.1	E3 ubiquitin-protein ligase HUW	8.39E+05	8.82E+05	1.05E+00	1.05E+00	1.05	0.05	Q7Z6Z7	HUWE1
1198.1	Isoform 7 of Granulocyte-macro	2.36E+05	2.47E+05	1.05E+00	1.05E+00	1.05	0.05	P15509-7	CSF2RA
322.1	Calreticulin	2.00E+06	2.09E+06	1.04E+00	1.04E+00	1.04	0.04	P27797	CALR
655.1	Selenocysteine lyase	3.72E+05	3.88E+05	1.04E+00	1.04E+00	1.04	0.04	Q96I15	SCLY
167.1	26S proteasome non-ATPase reg	5.42E+06	5.57E+06	1.03E+00	1.03E+00	1.03	0.03	Q5VWC4	PSMD4
4.1	Isoform 4 of Acetyl-CoA carboxy	4.50E+07	4.60E+07	1.02E+00	1.02E+00	1.02	0.02	Q13085-4	ACACA
616.1	Sister chromatid cohesion prote	4.29E+05	4.38E+05	1.02E+00	1.02E+00	1.02	0.02	Q9NTI5	PDS5B

37.1	Cullin-associated NEDD8-dissoci	8.80E+06	8.96E+06	1.02E+00	1.02E+00	1.02	0.02	Q86VP6	CAND1
109.1	Hypoxia up-regulated protein 1	8.33E+06	8.43E+06	1.01E+00	1.01E+00	1.01	0.01	Q9Y4L1	HYOU1
1812.1	Serine protease 23	1.24E+05	1.25E+05	1.01E+00	1.01E+00	1.01	0.01	O95084	PRSS23
852.1	SUMO-activating enzyme subun	2.38E+05	2.38E+05	1.00E+00	1.00E+00	1.00	0.00	Q9UBE0	SAE1
527.1	Isoform 2 of 26S proteasome nc	1.06E+06	1.06E+06	9.99E-01	-1.00E+00	-1.00	0.00	O00231-2	PSMD11
338.1	Protein S100-A11	3.21E+06	3.21E+06	9.98E-01	-1.00E+00	-1.00	0.00	P31949	S100A11
577.1	NAD(P)H-hydrate epimerase	3.58E+05	3.56E+05	9.95E-01	-1.01E+00	-1.01	-0.01	Q8NCW5	APOA1BP
431.1	NEDD8-conjugating enzyme Ubc	6.74E+05	6.64E+05	9.85E-01	-1.02E+00	-1.02	-0.02	P61081	UBE2M
118.1	Neuroblast differentiation-assoc	3.72E+06	3.66E+06	9.84E-01	-1.02E+00	-1.02	-0.02	Q09666	AHNAK
372.1	Nucleosome assembly protein 1	2.61E+06	2.52E+06	9.66E-01	-1.04E+00	-1.04	-0.03	P55209	NAP1L1
141.1	Cofilin-1	1.48E+07	1.41E+07	9.55E-01	-1.05E+00	-1.05	-0.05	E9PK25	CFL1
50.2	Isoform 2 of Eukaryotic initiatior	1.16E+06	1.11E+06	9.55E-01	-1.05E+00	-1.05	-0.05	Q14240-2	EIF4A2
247.1	4-trimethylaminobutyraldehyde	5.01E+06	4.78E+06	9.53E-01	-1.05E+00	-1.05	-0.05	P49189	ALDH9A1
728.1	Platelet-activating factor acetylh	3.87E+05	3.68E+05	9.50E-01	-1.05E+00	-1.05	-0.05	Q15102	PAFAH1B3
557.1	Proteasome subunit alpha type-	9.22E+05	8.74E+05	9.48E-01	-1.05E+00	-1.05	-0.05	P28066	PSMA5
182.1	Carbonyl reductase [NADPH] 1	5.38E+06	5.09E+06	9.46E-01	-1.06E+00	-1.06	-0.06	P16152	CBR1
435.1	Androgen receptor	1.11E+06	1.05E+06	9.44E-01	-1.06E+00	-1.06	-0.06	P10275	AR
1079.1	Leucine-rich repeat-containing p	1.45E+05	1.35E+05	9.34E-01	-1.07E+00	-1.07	-0.07	Q9H9A6	LRRC40
269.1	Protein disulfide-isomerase A4	1.02E+06	9.51E+05	9.31E-01	-1.07E+00	-1.07	-0.07	P13667	PDIA4
584.1	Isoform 3 of NADPH:adrenodoxi	1.14E+05	1.06E+05	9.28E-01	-1.08E+00	-1.08	-0.07	P22570-3	FDXR
8.3	Heat shock 70 kDa protein 1A/1	6.59E+05	6.10E+05	9.25E-01	-1.08E+00	-1.08	-0.08	P08107	HSPA1A
607.1	Dynein light chain 1, cytoplasmi	1.58E+06	1.45E+06	9.19E-01	-1.09E+00	-1.09	-0.08	P63167	DYNLL1
1237.1	WASH complex subunit 7	3.93E+03	3.60E+03	9.15E-01	-1.09E+00	-1.09	-0.09	Q2M389	KIAA1033
1088.1	Drebrin-like protein	2.08E+04	1.90E+04	9.12E-01	-1.10E+00	-1.10	-0.09	Q9UJU6	DBNL
332.1	cAMP-dependent protein kinase	5.77E+05	5.26E+05	9.11E-01	-1.10E+00	-1.10	-0.09	P13861	PRKAR2A
76.3	Heterogeneous nuclear ribonuc	7.07E+06	6.42E+06	9.08E-01	-1.10E+00	-1.10	-0.10	P52597	HNRNPF
32.1	T-complex protein 1 subunit bet	3.08E+07	2.76E+07	8.95E-01	-1.12E+00	-1.12	-0.11	P78371	CCT2
8.2	78 kDa glucose-regulated protei	2.19E+07	1.96E+07	8.93E-01	-1.12E+00	-1.12	-0.11	P11021	HSPA5
1178.1	ATP synthase subunit b, mitocho	6.39E+05	5.70E+05	8.92E-01	-1.12E+00	-1.12	-0.11	P24539	ATP5F1
460.1	Tumor susceptibility gene 101 p	1.94E+06	1.72E+06	8.86E-01	-1.13E+00	-1.13	-0.12	Q99816	TSG101
365.1	Thioredoxin domain-containing	1.03E+06	9.13E+05	8.85E-01	-1.13E+00	-1.13	-0.12	Q8NBS9	TXNDC5
414.1	Phosphoribosyl pyrophosphate	9.55E+05	8.43E+05	8.82E-01	-1.13E+00	-1.13	-0.13	O60256	PRPSAP2
224.1	Protein DJ-1	7.34E+06	6.44E+06	8.77E-01	-1.14E+00	-1.14	-0.13	Q99497	PARK7
496.1	NSFL1 cofactor p47	3.70E+05	3.24E+05	8.76E-01	-1.14E+00	-1.14	-0.13	J3QK90	NSFL1C
262.1	Sialic acid synthase	8.98E+06	7.86E+06	8.76E-01	-1.14E+00	-1.14	-0.13	Q9NR45	NANS

35.1	Phosphoglycerate kinase 1	4.73E+07	4.14E+07	8.75E-01	-1.14E+00	-1.14	-0.13	P00558	PGK1
271.1	Parathymosin	1.61E+07	1.38E+07	8.58E-01	-1.16E+00	-1.16	-0.15	P20962	PTMS
1450.1	TBC1 domain family member 9	1.28E+05	1.09E+05	8.49E-01	-1.18E+00	-1.18	-0.16	Q6ZT07	TBC1D9
583.1	UPF0468 protein C16orf80	2.80E+04	2.35E+04	8.39E-01	-1.19E+00	-1.19	-0.18	Q9Y6A4	C16orf80
492.1	Dynactin subunit 1	1.49E+06	1.22E+06	8.21E-01	-1.22E+00	-1.22	-0.20	Q14203	DCTN1
907.1	DNA topoisomerase I, mitochon	6.39E+05	5.22E+05	8.17E-01	-1.22E+00	-1.22	-0.20	Q969P6	TOP1MT
2111.1	Flavin reductase (NADPH)	4.07E+04	3.29E+04	8.08E-01	-1.24E+00	-1.24	-0.21	P30043	BLVRB
20.1	Isoform 2 of Heat shock protein	3.47E+07	2.76E+07	7.96E-01	-1.26E+00	-1.26	-0.23	P07900-2	HSP90AA1
540.1	RNA-binding protein 12	3.39E+05	2.68E+05	7.91E-01	-1.26E+00	-1.26	-0.23	Q9NTZ6	RBM12
633.1	Isoform 2 of Histone H2A.J	1.07E+06	8.46E+05	7.89E-01	-1.27E+00	-1.27	-0.24	Q9BTM1-2	H2AFJ
401.1	Isoform 2 of Casein kinase I isof	1.25E+06	9.87E+05	7.87E-01	-1.27E+00	-1.27	-0.24	P48729-2	CSNK1A1
146.1	Isoform 2 of Programmed cell d	4.07E+06	3.20E+06	7.85E-01	-1.27E+00	-1.27	-0.24	Q8WUM4-	PDCD6IP
723.1	60S ribosomal protein L39	6.43E+06	5.05E+06	7.85E-01	-1.27E+00	-1.27	-0.24	P62891	RPL39
1015.1	Engulfment and cell motility pro	9.07E+05	7.10E+05	7.83E-01	-1.28E+00	-1.28	-0.24	Q92556	ELMO1
692.1	HEAT repeat-containing protein	2.45E+05	1.91E+05	7.81E-01	-1.28E+00	-1.28	-0.25	Q9P2D3	HEATR5B
811.1	Ran-binding protein 3	4.51E+05	3.50E+05	7.76E-01	-1.29E+00	-1.29	-0.25	Q9H6Z4	RANBP3
686.1	Charged multivesicular body pro	1.62E+06	1.24E+06	7.67E-01	-1.30E+00	-1.30	-0.27	Q7LBR1	CHMP1B
44.1	Peroxiredoxin-1	2.35E+07	1.79E+07	7.63E-01	-1.31E+00	-1.31	-0.27	Q06830	PRDX1
743.1	HBS1-like protein	4.85E+05	3.68E+05	7.58E-01	-1.32E+00	-1.32	-0.28	Q9Y450	HBS1L
133.1	Peroxiredoxin-6	2.83E+07	2.14E+07	7.56E-01	-1.32E+00	-1.32	-0.28	P30041	PRDX6
672.1	Protein NOXP20	3.06E+05	2.31E+05	7.54E-01	-1.33E+00	-1.33	-0.28	Q8IWE2	FAM114A1
298.1	Adenylate kinase 1	1.68E+06	1.26E+06	7.51E-01	-1.33E+00	-1.33	-0.29	Q5T9B7	AK1
50.1	Eukaryotic initiation factor 4A-I	1.30E+07	9.69E+06	7.43E-01	-1.35E+00	-1.35	-0.30	P60842	EIF4A1
52.1	T-complex protein 1 subunit alp	2.36E+07	1.75E+07	7.43E-01	-1.35E+00	-1.35	-0.30	P17987	TCP1
128.1	Transketolase	8.60E+06	6.31E+06	7.33E-01	-1.36E+00	-1.36	-0.31	P29401	TKT
973.1	Dynein heavy chain 17, axonem	9.42E+04	6.87E+04	7.29E-01	-1.37E+00	-1.37	-0.32	Q9UFH2	DNAH17
948.1	Nuclear export mediator factor	9.29E+04	6.77E+04	7.29E-01	-1.37E+00	-1.37	-0.32	O60524	NEMF
264.2	Chromobox protein homolog 1	1.13E+05	8.10E+04	7.15E-01	-1.40E+00	-1.40	-0.33	P83916	CBX1
208.1	Protein transport protein Sec31	3.72E+06	2.63E+06	7.07E-01	-1.41E+00	-1.41	-0.35	D6REX3	SEC31A
257.1	RuvB-like 1	1.02E+06	7.11E+05	6.96E-01	-1.44E+00	-1.44	-0.36	Q9Y265	RUVBL1
1190.1	NADP-dependent oxidoreducta	6.73E+05	4.67E+05	6.94E-01	-1.44E+00	-1.44	-0.37	Q6NXP6	NOXRED1
468.1	Endonuclease domain-containin	2.15E+05	1.49E+05	6.92E-01	-1.45E+00	-1.45	-0.37	O94919	ENDOD1
658.1	Isoform 2 of F-box only protein	3.90E+05	2.68E+05	6.87E-01	-1.46E+00	-1.46	-0.38	Q8NFZ0-2	FBXO18
9.1	Tubulin beta-4B chain	4.64E+07	3.15E+07	6.79E-01	-1.47E+00	-1.47	-0.39	P68371	TUBB4B
964.1	G-protein coupled receptor 98	2.45E+05	1.66E+05	6.78E-01	-1.47E+00	-1.47	-0.39	Q8WXG9	GPR98

1215.1	Nuclear RNA export factor 1	9.43E+04	6.39E+04	6.77E-01	-1.48E+00	-1.48	-0.39	Q9UBU9	NXF1
1270.1	Centriolar coiled-coil protein of	7.87E+05	5.30E+05	6.74E-01	-1.48E+00	-1.48	-0.39	O43303	CCP110
524.1	LanC-like protein 1	3.53E+05	2.37E+05	6.72E-01	-1.49E+00	-1.49	-0.40	O43813	LANCL1
250.1	Tumor protein D52	9.86E+05	6.62E+05	6.72E-01	-1.49E+00	-1.49	-0.40	F5H0B0	TPD52
581.1	Zinc finger homeobox protein 3	4.67E+05	3.10E+05	6.64E-01	-1.51E+00	-1.51	-0.41	Q15911	ZFHX3
574.1	Zinc finger CCCH domain-contai	1.04E+05	6.91E+04	6.64E-01	-1.51E+00	-1.51	-0.41	E7ERS3	ZC3H18
1097.1	Dynein assembly factor 1, axone	1.26E+05	8.32E+04	6.58E-01	-1.52E+00	-1.52	-0.42	Q8NEP3	DNAAF1
1292.1	Isoform 3 of AT-rich interactive	1.05E+05	6.81E+04	6.48E-01	-1.54E+00	-1.54	-0.43	Q8NFD5-3	ARID1B
1.2	Epiplakin	6.89E+07	4.45E+07	6.45E-01	-1.55E+00	-1.55	-0.44	P58107	EPPK1
596.1	Tyrosine--tRNA ligase, cytoplasn	7.27E+05	4.67E+05	6.42E-01	-1.56E+00	-1.56	-0.44	P54577	YARS
1146.1	Dynein heavy chain 8, axonemal	1.17E+05	7.46E+04	6.36E-01	-1.57E+00	-1.57	-0.45	H0Y7V4	DNAH8
851.1	Ubiquitin carboxyl-terminal hyd	5.07E+05	3.21E+05	6.34E-01	-1.58E+00	-1.58	-0.46	Q70CQ2	USP34
1065.1	Nuclear pore membrane glycopı	5.90E+05	3.73E+05	6.32E-01	-1.58E+00	-1.58	-0.46	Q5VU65	NUP210L
469.1	Putative pre-mRNA-splicing fact	1.07E+06	6.69E+05	6.24E-01	-1.60E+00	-1.60	-0.47	O43143	DHX15
1078.1	Isoform KvB1.2 of Voltage-gatec	3.59E+05	2.24E+05	6.24E-01	-1.60E+00	-1.60	-0.47	Q14722-3	KCNAB1
606.1	Haloacid dehalogenase-like hydı	3.55E+06	2.20E+06	6.20E-01	-1.61E+00	-1.61	-0.48	Q9H0R4	HDHD2
1025.1	Putative rhophilin-2-like protein	9.67E+04	5.88E+04	6.08E-01	-1.64E+00	-1.64	-0.50	A8MT19	RHPN2P1
101.1	Myosin light polypeptide 6	1.58E+07	9.59E+06	6.08E-01	-1.64E+00	-1.64	-0.50	B7Z6Z4	MYL6
1191.1	Synaptic vesicle glycoprotein 2B	3.78E+05	2.25E+05	5.95E-01	-1.68E+00	-1.68	-0.52	Q7L1I2	SV2B
462.1	N-alpha-acetyltransferase 50	1.04E+06	6.17E+05	5.93E-01	-1.69E+00	-1.69	-0.52	Q9GZZ1	NAA50
420.1	Long-chain-fatty-acid--CoA ligas	1.03E+05	6.06E+04	5.88E-01	-1.70E+00	-1.70	-0.53	P33121	ACSL1
1124.1	Adenomatous polyposis coli pro	4.91E+05	2.83E+05	5.76E-01	-1.74E+00	-1.74	-0.55	O95996	APC2
541.1	Protein HID1	8.54E+05	4.90E+05	5.74E-01	-1.74E+00	-1.74	-0.56	Q8IV36	HID1
88.3	14-3-3 protein gamma	6.54E+06	3.73E+06	5.70E-01	-1.75E+00	-1.75	-0.56	P61981	YWHAG
135.1	Sorbitol dehydrogenase	5.80E+06	3.28E+06	5.65E-01	-1.77E+00	-1.77	-0.57	Q00796	SORD
734.1	Vacuolar protein sorting-associa	1.01E+06	5.64E+05	5.58E-01	-1.79E+00	-1.79	-0.58	C9JJN9	VPS8
898.1	Interferon-induced very large G	1.27E+05	7.06E+04	5.54E-01	-1.80E+00	-1.80	-0.59	Q7Z2Y8	GVINP1
986.1	UPF0693 protein C10orf32	1.12E+04	6.14E+03	5.47E-01	-1.83E+00	-1.83	-0.60	Q96B45	C10orf32
794.1	Hyaluronan synthase 1	5.66E+05	3.08E+05	5.44E-01	-1.84E+00	-1.84	-0.61	Q92839	HAS1
328.1	DnaJ homolog subfamily A mem	1.13E+06	6.02E+05	5.32E-01	-1.88E+00	-1.88	-0.63	P31689	DNAJA1
685.1	Tektin-1	3.08E+04	1.63E+04	5.29E-01	-1.89E+00	-1.89	-0.64	Q969V4	TEKT1
304.1	Phosphoglycerate mutase 1	4.62E+06	2.43E+06	5.26E-01	-1.90E+00	-1.90	-0.64	P18669	PGAM1
234.1	C-1-tetrahydrofolate synthase, c	3.70E+06	1.93E+06	5.22E-01	-1.92E+00	-1.92	-0.65	F5H2F4	MTHFD1
1455.1	Zinc finger CCCH domain-contai	2.59E+05	1.33E+05	5.14E-01	-1.95E+00	-1.95	-0.67	Q5T200	ZC3H13
380.1	Isoform 2 of Spectrin alpha chai	1.27E+06	6.45E+05	5.06E-01	-1.97E+00	-1.97	-0.68	Q13813-2	SPTAN1

145.1	Protein transport protein Sec16,	6.59E+06	3.32E+06	5.04E-01	-1.99E+00	-1.99	-0.69	J3KNL6	SEC16A
653.1	Acidic leucine-rich nuclear phos	1.15E+06	5.80E+05	5.03E-01	-1.99E+00	-1.99	-0.69	P39687	ANP32A
441.1	N(G),N(G)-dimethylarginine dim	1.86E+06	9.36E+05	5.03E-01	-1.99E+00	-1.99	-0.69	O94760	DDAH1
757.1	Protein LOC100653515	4.04E+05	1.98E+05	4.90E-01	-2.04E+00	-2.04	-0.71	Q96MC4	DDC8
1578.1	Nucleolar complex protein 3 hoı	5.51E+04	2.69E+04	4.88E-01	-2.05E+00	-2.05	-0.72	A6NJZ9	NOC3L
310.1	PEST proteolytic signal-containi	1.58E+06	7.67E+05	4.86E-01	-2.06E+00	-2.06	-0.72	Q8WW12	PCNP
704.1	Obg-like ATPase 1	2.44E+05	1.18E+05	4.84E-01	-2.06E+00	-2.06	-0.73	J3KQ32	OLA1
185.1	Paternally-expressed gene 3 prc	3.20E+06	1.55E+06	4.84E-01	-2.07E+00	-2.07	-0.73	Q9GZU2	PEG3
1368.1	Integrin alpha-2	1.78E+05	8.55E+04	4.81E-01	-2.08E+00	-2.08	-0.73	P17301	ITGA2
41.1	Unconventional myosin-VI	1.42E+07	6.71E+06	4.74E-01	-2.11E+00	-2.11	-0.75	Q9UM54	MYO6
444.1	Putative ubiquitin-conjugating e	3.54E+05	1.67E+05	4.72E-01	-2.12E+00	-2.12	-0.75	Q5JXB2	UBE2NL
899.1	Sorting nexin-6	1.56E+05	7.30E+04	4.69E-01	-2.13E+00	-2.13	-0.76	Q9UNH7	SNX6
929.1	C-Myc-binding protein	1.03E+05	4.68E+04	4.54E-01	-2.20E+00	-2.20	-0.79	Q99417	MYCBP
1414.1	Isoform 2 of Nck-associated pro	1.90E+05	8.59E+04	4.52E-01	-2.21E+00	-2.21	-0.79	Q9Y2A7-2	NCKAP1
24.1	Tubulin alpha-1B chain	3.59E+07	1.61E+07	4.49E-01	-2.23E+00	-2.23	-0.80	P68363	TUBA1B
491.1	Isoform 3 of Shootin-1	6.91E+05	3.09E+05	4.47E-01	-2.23E+00	-2.23	-0.80	A0MZ66-3	KIAA1598
780.1	Isoform 3 of DCC-interacting prc	6.14E+05	2.70E+05	4.40E-01	-2.27E+00	-2.27	-0.82	Q8NEU8-3	APPL2
983.1	26S proteasome non-ATPase reg	1.48E+05	6.49E+04	4.40E-01	-2.27E+00	-2.27	-0.82	O00233	PSMD9
170.1	Glycogen phosphorylase, brain f	6.16E+06	2.69E+06	4.37E-01	-2.29E+00	-2.29	-0.83	P11216	PYGB
398.1	Ran-specific GTPase-activating p	5.97E+05	2.61E+05	4.37E-01	-2.29E+00	-2.29	-0.83	P43487	RANBP1
1298.1	Interferon-induced guanylate-bi	9.12E+05	3.95E+05	4.33E-01	-2.31E+00	-2.31	-0.84	P32456	GBP2
534.1	Transcriptional activator proteir	2.40E+05	1.03E+05	4.30E-01	-2.33E+00	-2.33	-0.84	Q96QR8	PURB
1789.1	Sorting nexin-2	1.62E+04	6.94E+03	4.29E-01	-2.33E+00	-2.33	-0.85	O60749	SNX2
293.1	Charged multivesicular body prc	3.95E+06	1.69E+06	4.28E-01	-2.34E+00	-2.34	-0.85	O43633	CHMP2A
465.1	6-phosphogluconate dehydroge	9.78E+05	4.03E+05	4.12E-01	-2.43E+00	-2.43	-0.89	P52209	PGD
503.1	Trifunctional purine biosyntheti	1.92E+06	7.85E+05	4.09E-01	-2.45E+00	-2.45	-0.89	P22102	GART
621.1	Alpha/beta hydrolase domain-c	3.51E+06	1.42E+06	4.05E-01	-2.47E+00	-2.47	-0.90	Q96IU4	ABHD14B
1387.1	Protocadherin beta-16	4.55E+05	1.82E+05	4.00E-01	-2.50E+00	-2.50	-0.92	Q9NRJ7	PCDHB16
762.1	Calcium-regulated heat stable p	6.21E+05	2.43E+05	3.91E-01	-2.55E+00	-2.55	-0.94	Q9Y2V2	CARHSP1
816.1	Isoform 4 of Myotubularin-relat	6.83E+05	2.60E+05	3.80E-01	-2.63E+00	-2.63	-0.97	O95248-4	SBF1
1363.1	Centrosomal protein of 152 kDa	1.24E+05	4.70E+04	3.78E-01	-2.65E+00	-2.65	-0.97	O94986	CEP152
765.1	Isoform 2 of Eukaryotic translati	2.99E+05	1.13E+05	3.78E-01	-2.65E+00	-2.65	-0.97	P06730-2	EIF4E
695.1	Proteasome subunit alpha type-	1.05E+06	3.91E+05	3.72E-01	-2.69E+00	-2.69	-0.99	P25787	PSMA2
403.1	FLYWCH family member 2	1.40E+06	5.09E+05	3.65E-01	-2.74E+00	-2.74	-1.01	I3L1Y9	FLYWCH2
792.1	Amyotrophic lateral sclerosis 2 (	3.44E+04	1.23E+04	3.58E-01	-2.79E+00	-2.79	-1.03	Q53TS8	ALS2CR11

458.1	Cellular nucleic acid-binding pro	4.39E+06	1.57E+06	3.58E-01	-2.79E+00	-2.79	-1.03	P62633	CNBP
525.1	Tumor protein D52-like 2	1.95E+06	6.91E+05	3.54E-01	-2.82E+00	-2.82	-1.04	O43399	TPD52L2
168.1	Nucleoside diphosphate kinase	3.28E+05	1.16E+05	3.54E-01	-2.82E+00	-2.82	-1.04	Q32Q12	NME1-NME2
719.1	Acidic leucine-rich nuclear phos	1.10E+06	3.88E+05	3.52E-01	-2.84E+00	-2.84	-1.04	Q92688	ANP32B
1182.1	Neuroendocrine convertase 1	2.99E+05	1.04E+05	3.48E-01	-2.88E+00	-2.88	-1.06	P29120	PCSK1
1265.1	Serine/threonine-protein kinase	6.57E+04	2.28E+04	3.47E-01	-2.88E+00	-2.88	-1.06	Q96GX5	MASTL
1177.1	28S ribosomal protein S7, mitoc	1.12E+05	3.83E+04	3.41E-01	-2.93E+00	-2.93	-1.08	J3QLS3	MRPS7
998.1	Neurogenic locus notch homolo	3.44E+06	1.17E+06	3.40E-01	-2.94E+00	-2.94	-1.08	Q04721	NOTCH2
1295.1	Egl nine homolog 1	1.64E+05	5.51E+04	3.36E-01	-2.97E+00	-2.97	-1.09	Q9GZT9	EGLN1
801.1	Nuclear transport factor 2	1.27E+06	4.07E+05	3.20E-01	-3.13E+00	-3.13	-1.14	P61970	NUTF2
64.1	Calpain-1 catalytic subunit	1.48E+07	4.71E+06	3.19E-01	-3.13E+00	-3.13	-1.14	P07384	CAPN1
504.1	Small ubiquitin-related modifier	7.44E+06	2.34E+06	3.14E-01	-3.18E+00	-3.18	-1.16	B4DUW4	SUMO3
682.1	Son of sevenless homolog 2	1.03E+06	3.24E+05	3.14E-01	-3.18E+00	-3.18	-1.16	Q07890	SOS2
215.1	Lactoylglutathione lyase	8.38E+06	2.62E+06	3.13E-01	-3.20E+00	-3.20	-1.16	Q04760	GLO1
461.1	ADP-ribosylation factor GTPase-	3.43E+05	1.05E+05	3.06E-01	-3.26E+00	-3.26	-1.18	Q9NP61	ARFGAP3
55.1	T-complex protein 1 subunit the	1.27E+07	3.87E+06	3.04E-01	-3.29E+00	-3.29	-1.19	P50990	CCT8
1484.1	Adenylate cyclase type 8	5.04E+05	1.53E+05	3.03E-01	-3.30E+00	-3.30	-1.19	P40145	ADCY8
512.1	Tryptophan--tRNA ligase, cytopl	1.07E+07	3.24E+06	3.02E-01	-3.31E+00	-3.31	-1.20	P23381	WARS
773.1	Neuroblastoma-amplified seque	1.03E+06	3.09E+05	3.00E-01	-3.34E+00	-3.34	-1.21	A2RRP1	NBAS
835.1	G antigen 1	2.46E+06	7.29E+05	2.97E-01	-3.37E+00	-3.37	-1.21	Q13065	GAGE1
554.1	Endoplasmic reticulum resident	4.80E+05	1.39E+05	2.89E-01	-3.45E+00	-3.45	-1.24	P30040	ERP29
448.1	PDZ and LIM domain protein 5	5.38E+05	1.55E+05	2.88E-01	-3.47E+00	-3.47	-1.24	Q96HC4	PDLIM5
543.1	Ras-related protein Rab-10	4.85E+05	1.39E+05	2.86E-01	-3.49E+00	-3.49	-1.25	P61026	RAB10
896.1	Acidic leucine-rich nuclear phos	7.27E+05	2.02E+05	2.78E-01	-3.60E+00	-3.60	-1.28	Q9BTT0	ANP32E
161.1	Ubiquitin-40S ribosomal protein	5.14E+07	1.42E+07	2.76E-01	-3.62E+00	-3.62	-1.29	P62979	RPS27A
396.1	Proteasome subunit alpha type	3.04E+05	8.40E+04	2.76E-01	-3.62E+00	-3.62	-1.29	G3V5Z7	PSMA6
3.2	Vimentin	2.12E+07	5.80E+06	2.73E-01	-3.66E+00	-3.66	-1.30	P08670	VIM
952.1	Lysine-specific demethylase 3B	1.17E+05	3.20E+04	2.73E-01	-3.66E+00	-3.66	-1.30	Q7LBC6	KDM3B
337.1	Phosphoglucomutase-2	2.20E+06	5.94E+05	2.70E-01	-3.71E+00	-3.71	-1.31	Q96G03	PGM2
601.1	Isoform 3 of Serine/threonine-p	1.14E+06	3.04E+05	2.66E-01	-3.76E+00	-3.76	-1.32	O00743-3	PPP6C
763.1	Intercellular adhesion molecule	4.75E+04	1.24E+04	2.61E-01	-3.83E+00	-3.83	-1.34	Q9UMF0	ICAM5
1102.1	Na(+)/H(+) exchange regulatory	2.51E+05	6.48E+04	2.58E-01	-3.87E+00	-3.87	-1.35	Q15599	SLC9A3R2
1502.1	WAP, Kazal, immunoglobulin, Ku	3.81E+05	9.85E+04	2.58E-01	-3.87E+00	-3.87	-1.35	Q8TEU8	WFIKKN2
379.1	Protein SEC13 homolog	6.21E+06	1.60E+06	2.58E-01	-3.88E+00	-3.88	-1.36	B4DXJ1	SEC13
421.1	F-actin-capping protein subunit	1.49E+06	3.77E+05	2.54E-01	-3.94E+00	-3.94	-1.37	P47755	CAPZA2

430.1	Bifunctional ATP-dependent dih	1.62E+06	4.10E+05	2.53E-01	-3.95E+00	-3.95	-1.37	Q3LXA3	DAK
1444.1	EF-hand calcium-binding domai	2.72E+05	6.89E+04	2.53E-01	-3.95E+00	-3.95	-1.37	A4FU69	EFCAB5
1106.1	Cytochrome b-c1 complex subu	3.08E+05	7.77E+04	2.52E-01	-3.97E+00	-3.97	-1.38	P31930	UQCRC1
880.1	1-phosphatidylinositol 4,5-bisph	1.46E+06	3.64E+05	2.50E-01	-4.00E+00	-4.00	-1.39	E2QRH8	PLCB4
14.1	Myosin-9	2.88E+07	6.88E+06	2.39E-01	-4.19E+00	-4.19	-1.43	P35579	MYH9
346.1	Gamma-glutamylcyclotransfera:	1.27E+06	2.97E+05	2.33E-01	-4.29E+00	-4.29	-1.46	O75223	GGCT
376.1	Nuclear autoantigenic sperm pr	6.41E+04	1.48E+04	2.31E-01	-4.33E+00	-4.33	-1.47	P49321	NASP
290.1	Glycine--tRNA ligase	1.73E+06	3.90E+05	2.26E-01	-4.43E+00	-4.43	-1.49	P41250	GARS
138.1	Proliferating cell nuclear antiger	9.11E+06	2.02E+06	2.22E-01	-4.51E+00	-4.51	-1.51	P12004	PCNA
233.1	tRNA (cytosine(34)-C(5))-methyl	1.94E+06	4.22E+05	2.17E-01	-4.60E+00	-4.60	-1.53	Q08J23	NSUN2
683.1	Sorting nexin-12	8.88E+05	1.92E+05	2.16E-01	-4.62E+00	-4.62	-1.53	Q9UMY4	SNX12
455.1	Exportin-1	3.90E+06	8.06E+05	2.07E-01	-4.84E+00	-4.84	-1.58	O14980	XPO1
587.1	Nebulin	1.02E+06	2.09E+05	2.05E-01	-4.89E+00	-4.89	-1.59	F8WCL5	NEB
724.1	Coiled-coil domain-containing p	5.30E+06	1.08E+06	2.04E-01	-4.91E+00	-4.91	-1.59	Q8NDH2	CCDC168
2158.1	SWI/SNF-related matrix-associa	3.23E+05	6.41E+04	1.99E-01	-5.03E+00	-5.03	-1.62	Q96GM5	SMARCD1
643.1	Multiple PDZ domain protein	8.79E+05	1.74E+05	1.98E-01	-5.05E+00	-5.05	-1.62	F5H1U9	MPDZ
353.2	C-terminal-binding protein 1	1.07E+06	2.08E+05	1.94E-01	-5.15E+00	-5.15	-1.64	Q13363	CTBP1
141.2	Destrin	3.55E+06	6.86E+05	1.93E-01	-5.17E+00	-5.17	-1.64	P60981	DSTN
486.1	Transgelin-2	8.40E+05	1.61E+05	1.92E-01	-5.22E+00	-5.22	-1.65	P37802	TAGLN2
1330.1	Signal transducer and activator	1.73E+05	3.24E+04	1.87E-01	-5.34E+00	-5.34	-1.67	P42224	STAT1
456.1	Ladinin-1	5.85E+05	1.08E+05	1.85E-01	-5.42E+00	-5.42	-1.69	E9PDI4	LAD1
1019.1	Clustered mitochondria protein	1.28E+05	2.34E+04	1.82E-01	-5.49E+00	-5.49	-1.70	O75153	CLUH
1033.1	Homeobox protein TGIF1	2.59E+05	4.62E+04	1.79E-01	-5.60E+00	-5.60	-1.72	Q15583	TGIF1
915.1	Spectrin beta chain, non-erythro	9.02E+05	1.57E+05	1.74E-01	-5.74E+00	-5.74	-1.75	Q9NRC6	SPTBN5
1187.1	1-phosphatidylinositol 4,5-bisph	7.87E+06	1.36E+06	1.73E-01	-5.78E+00	-5.78	-1.76	Q86YW0	PLCZ1
1053.1	Cell division cycle and apoptosis	1.73E+05	2.95E+04	1.71E-01	-5.86E+00	-5.86	-1.77	Q8IX12	CCAR1
378.1	Nascent polypeptide-associated	6.83E+06	1.16E+06	1.70E-01	-5.89E+00	-5.89	-1.77	E9PAV3	NACA
777.1	Mortality factor 4-like protein 2	1.63E+05	2.44E+04	1.50E-01	-6.67E+00	-6.67	-1.90	Q15014	MORF4L2
3.3	Keratin, type II cytoskeletal 6B	2.37E+05	3.51E+04	1.48E-01	-6.74E+00	-6.74	-1.91	P04259	KRT6B
1488.1	Protein-tyrosine sulfotransferas	2.02E+05	2.88E+04	1.42E-01	-7.02E+00	-7.02	-1.95	O60507	TPST1
111.1	Spectrin beta chain, non-erythro	5.18E+06	7.31E+05	1.41E-01	-7.08E+00	-7.08	-1.96	O15020	SPTBN2
251.1	Protein arginine N-methyltransf	5.16E+06	7.17E+05	1.39E-01	-7.19E+00	-7.19	-1.97	H7C2I1	PRMT1
200.1	Stress-induced-phosphoprotein	2.36E+06	3.17E+05	1.35E-01	-7.43E+00	-7.43	-2.01	P31948	STIP1
152.1	Src substrate cortactin	7.55E+06	1.01E+06	1.34E-01	-7.48E+00	-7.48	-2.01	Q14247	CTTN
19.1	Actin, cytoplasmic 2	2.20E+08	2.86E+07	1.30E-01	-7.71E+00	-7.71	-2.04	P63261	ACTG1

1255.1	Protein EFR3 homolog A	3.77E+05	4.80E+04	1.27E-01	-7.86E+00	-7.86	-2.06	H9KV44	EFR3A
980.1	Serine/threonine-protein phosp	9.06E+04	1.10E+04	1.21E-01	-8.23E+00	-8.23	-2.11	Q13362	PPP2R5C
845.1	Annexin A5	1.61E+05	1.94E+04	1.21E-01	-8.29E+00	-8.29	-2.11	P08758	ANXA5
277.1	Signal recognition particle 9 kDa	8.61E+06	1.02E+06	1.18E-01	-8.44E+00	-8.44	-2.13	P49458	SRP9
1009.1	Matrix metalloproteinase-28	1.01E+06	1.19E+05	1.18E-01	-8.45E+00	-8.45	-2.13	K7ENP0	MMP28
354.1	Transcription initiation factor IIE	1.29E+06	1.48E+05	1.14E-01	-8.74E+00	-8.74	-2.17	Q00403	GTF2B
625.1	UDP-glucose:glycoprotein gluco	2.45E+06	2.72E+05	1.11E-01	-8.99E+00	-8.99	-2.20	Q9NYU2	UGGT1
689.1	PHD finger protein 3	8.54E+05	9.44E+04	1.11E-01	-9.05E+00	-9.05	-2.20	Q92576	PHF3
361.1	Catechol O-methyltransferase	2.70E+06	2.55E+05	9.45E-02	-1.06E+01	-10.00	-2.36	P21964	COMT
244.1	LIM domain and actin-binding p	4.91E+06	4.56E+05	9.28E-02	-1.08E+01	-10.00	-2.38	Q9UHB6	LIMA1
714.1	Nuclear receptor corepressor 1	9.60E+04	8.70E+03	9.06E-02	-1.10E+01	-10.00	-2.40	O75376	NCOR1
1049.1	Protein NDRG1	1.17E+05	1.01E+04	8.61E-02	-1.16E+01	-10.00	-2.45	Q92597	NDRG1
928.1	Unconventional myosin-IXb	1.09E+06	8.69E+04	7.96E-02	-1.26E+01	-10.00	-2.53	Q13459	MYO9B
627.1	E3 ubiquitin-protein ligase RNF1	4.13E+06	3.03E+05	7.33E-02	-1.36E+01	-10.00	-2.61	C9J266	RNF123
775.1	Hydroxyacid-oxoacid transhydro	4.66E+05	3.33E+04	7.14E-02	-1.40E+01	-10.00	-2.64	Q8IWW8	ADHFE1
632.2	CLASP1 protein	1.59E+05	1.12E+04	7.06E-02	-1.42E+01	-10.00	-2.65	B7ZLX3	CLASP1
861.1	Splicing factor 3B subunit 1	1.37E+05	9.64E+03	7.01E-02	-1.43E+01	-10.00	-2.66	O75533	SF3B1
733.1	Beta-hexosaminidase subunit al	1.44E+05	9.31E+03	6.49E-02	-1.54E+01	-10.00	-2.74	H3BP20	HEXA
23.1	Isoform 3 of Caldesmon	2.89E+07	1.74E+06	6.02E-02	-1.66E+01	-10.00	-2.81	Q05682-3	CALD1
768.1	Isoform 2 of L-fucose kinase	2.82E+05	1.63E+04	5.78E-02	-1.73E+01	-10.00	-2.85	Q8N0W3-2	FUK
720.1	26S proteasome non-ATPase reg	3.08E+06	1.41E+05	4.57E-02	-2.19E+01	-10.00	-3.09	P51665	PSMD7
1318.1	Antigen KI-67	3.87E+05	1.54E+04	3.98E-02	-2.51E+01	-10.00	-3.22	P46013	MKI67
1032.1	Microtubule-associated protein	4.79E+05	1.40E+04	2.92E-02	-3.42E+01	-10.00	-3.53	P10636	MAPT
481.1	Serpin B6	5.73E+05	1.39E+04	2.42E-02	-4.12E+01	-10.00	-3.72	P35237	SERPINB6
470.1	Heterogeneous nuclear ribonuc	3.03E+06	7.32E+04	2.41E-02	-4.14E+01	-10.00	-3.72	Q00839	HNRNPU
831.1	Sacsin	1.22E+07	2.91E+05	2.38E-02	-4.20E+01	-10.00	-3.74	Q9NZJ4	SACS
1099.1	Receptor-type tyrosine-protein	5.29E+05	1.17E+04	2.21E-02	-4.52E+01	-10.00	-3.81	P10586	PTPRF
84.1	Filamin-A	6.94E+06	1.51E+05	2.18E-02	-4.59E+01	-10.00	-3.83	P21333	FLNA
1283.1	Isoform 2 of Nebulin-related-an	9.17E+05	1.83E+04	2.00E-02	-5.01E+01	-10.00	-3.91	Q86VF7-2	NRAP
1222.1	Retinoblastoma-associated prot	2.29E+05	4.49E+03	1.96E-02	-5.11E+01	-10.00	-3.93	P06400	RB1
21.1	Isoform 2 of Tropomyosin alpha	5.43E+07	6.98E+05	1.29E-02	-7.78E+01	-10.00	-4.35	P06753-2	TPM3
1180.1	Segment polarity protein dishev	5.70E+05	7.13E+03	1.25E-02	-8.00E+01	-10.00	-4.38	O14640	DVL1
12.1	Alpha-actinin-4	9.17E+07	1.03E+06	1.12E-02	-8.90E+01	-10.00	-4.49	O43707	ACTN4
992.1	Tetratricopeptide repeat protei	4.23E+06	3.88E+04	9.18E-03	-1.09E+02	-10.00	-4.69	Q8IYW2	TTC40
19.2	Actin, aortic smooth muscle	5.62E+07	5.00E+05	8.90E-03	-1.12E+02	-10.00	-4.72	P62736	ACTA2

389.1	Translin	3.71E+06	3.00E+04	8.09E-03	-1.24E+02	-10.00	-4.82	E9PGT1	TSN
196.1	Coiled-coil domain-containing p	4.60E+06	1.94E+04	4.22E-03	-2.37E+02	-10.00	-5.47	Q8IVM0	CCDC50
788.1	Nuclear migration protein nudC	6.35E+05	1.88E+03	2.96E-03	-3.38E+02	-10.00	-5.82	Q9Y266	NUDC
192.1	Protein phosphatase 1 regulatoı	4.25E+06	8.99E+03	2.12E-03	-4.72E+02	-10.00	-6.16	O14974	PPP1R12A

Supplemental Table 2. Known AR-interacting proteins identified in the proteomic screen

Data was collated through PUBMED, HPPRD, BIOGRID, BIND and McGill AR database and current publications

Note: Cells highlighted in green indicate proteins enriched in the *AD* sample, highlighted in red indicate proteins enriched in the *AS* sample, highlighted in yellow indicate 1:1 *AS*

Cells not highlighted were AR-interacting proteins not found in the proteomic screen.

Subgroup	Official Gene symbol	Other names	UniProt ID	Binding	Description	Author	Pubmed ID	Journal	MS Data (Fold Change)
16	ACTN4		O43707	Direct Binding	ACTN4_HUMAN Alpha-actinin-4	Jasavala R et al	17052974	Mol Cell Proteomics	-10.00
81	FLNA		P21333	direct binding	FLNA_HUMAN Filamin-A				-10.00
25	NCOR1		O75376	direct binding	NCOR1_HUMAN Nuclear receptor corepressor 1				-10.00
211	TGIF1		Q15583	direct, coR	TGIF1_HUMAN Homeobox protein TGIF1				-5.60
264	SMARCD1		Q96GM5	direct, coA	SMRD1_HUMAN SWI/SNF-related matrix-associated actin-dependent regulator of chromatin subfamily D member 1		19762545	van de Wijngaart DJ et al Mol Endocrinol 2009	-5.03
147	SUMO3		P55854	coA, coR	SUMO3_HUMAN Small ubiquitin-related modifier 3				-3.18
316	MYO6		Q9UM54	direct, coA	MYO6_HUMAN Myosin-VI		19787211		-2.11
112	ANP32A		P39687	direct binding	AN32A_HUMAN Acidic leucine-rich nuclear phosphoprotein 32 family member A				-1.99
313	APPL1		Q9UKG1	indirect, coR	DP13A_HUMAN DCC-interacting protein 13-alpha				Unique-AD
47	CDK1		P06493	shown to phosphoryla	CDK1_HUMAN Cyclin-dependent kinase 1				Unique-AD
219	HSPB2		Q16082	direct, coA	HSPB2_HUMAN Heat shock protein beta-2				Unique-AD
212	NCOA2		Q15596	direct, coA	NCOA2_HUMAN Nuclear receptor coactivator 2				Unique-AD
122	NRIP1		P48552	direct binding	NRIP1_HUMAN Nuclear receptor-interacting protein 1				Unique-AD
267	NSD1	STO	Q96L73	direct, coA	NSD1_HUMAN Histone-lysine N-methyltransferase, H3 lysine-36 and H4 lysine-20 specific				Unique-AD

118	PIK3CB		P42338	p110b required for AR	PK3CB_HUMAN Phosphatidylinositol-4,5-bisphosphate 3-kinase catalytic subunit beta isoform			Unique-AD
92	PIK3R1		P27986	p85 required for AR-DI	P85A_HUMAN Phosphatidylinositol 3-kinase regulatory subunit alpha			Unique-AD
62	XRCC5		P13010	direct binding	XRCC5_HUMAN X-ray repair cross-complementing protein 5			Unique-AD
61	XRCC6		P12956	direct binding	XRCC6_HUMAN X-ray repair cross-complementing protein 6			Unique-AD
315	ZMIZ1		Q9ULJ6	direct, coA	ZMIZ1_HUMAN Zinc finger MIZ domain-containing protein 1			Unique-AD
237	TH1L		Q8IXH7	direct, affinity capture	NELFD_HUMAN Negative elongation factor C/D			Unique-AD
33	SVIL		O95425	direct binding	SVIL_HUMAN Supervillin			Unique-AD
213	MED1		Q15648	direct, coA	MED1_HUMAN Mediator of RNA polymerase II transcription subunit 1			Unique-AD
235	CARM1		Q86X55	indirect, coA	CARM1_HUMAN Histone-arginine methyltransferase CARM1			Unique-AD
208	PRKD1		Q15139	direct, coR	KPCD1_HUMAN Serine/threonine-protein kinase D1			Unique-AD
283	BRD8		Q9H0E9	direct, coA	BRD8_HUMAN Bromodomain-containing protein 8	18560202	Hosoya T et al Endocr J 55(4) 657-65 2008	Unique-AD
349	STUB1	CHIP	Q9UNE7	direct, target for degra	E3 ubiquitin-protein ligase CHIP	16725394	Rees I et al Bichim Biophys Acta. 2006 Jun; 1764(6): 1073-9. Epub 2006 Apr 4	Unique-AD
80	CDK11B		P21127	direct binding	CD11B_HUMAN Cell division protein kinase 11B			Unique-AD
12	DAPK3		O43293	Direct Binding	DAPK3_HUMAN Death-associated protein kinase 3			Unique-AD

266	EHMT2		Q96KQ7	indirect, coA	EHMT2_HUMAN Histone-lysine N-methyltransferase, H3 lysine-9 specific 3			Unique-AD
260	KAT5		Q92993	direct, coA	KAT5_HUMAN Histone acetyltransferase KAT5			Unique-AD
11	PER1		O15534	Direct Binding, coA	PER1_HUMAN Period circadian protein homolog 1			Unique-AD
149	PTEN		P60484	direct binidng	PTEN_HUMAN Phosphatidylinositol-3,4,5-trisphosphate 3-phosphatase and dual-specificity protein phosphatase PTEN			Unique-AD
321	RNF6		Q9Y252	direct, coA	RNF6_HUMAN RING finger protein 6			Unique-AD
17	SGTA		O43765	Direct Binding	SGTA_HUMAN Small glutamine-rich tetratricopeptide repeat-containing protein alpha			Unique-AD
342	CHD8		Q9HCK8	direct binding/coA	chromatin remodeling factor CHD8	20308527	Menon T et al Mol Endocrinol 24(6) 1165-74 2010	2.04
152	COPS2		P61201	direct bindinig	CSN2_HUMAN COP9 signalosome complex subunit 2			2.04
300	SENP1		Q9P0U3	indirect, coA	SENP1_HUMAN Sentrin-specific protease 1			2.41
257	KIFAP3		Q92845	direct, y-2-hybrid	KIFA3_HUMAN Kinesin-associated protein 3			2.44
103	MCM7		P33993	direct binding	MCM7_HUMAN DNA replication licensing factor MCM7			2.44
343	CBX1	HP1beta	P83916	direct binding/coA	Human heterochrmatin protein 1 isoform HP1beta	20308360	Shiota M et al Endocr Relat Cancer 17(2) 2010	2.92
221	CDC37		Q16543	direct, coA	CDC37_HUMAN Hsp90 co-chaperone Cdc37			2.92
7	GAK		O14976	Direct Binding	GAK_HUMAN Cyclin-G-associated kinase			2.94

41	GAPDH	P04406	direct binding	G3P_HUMAN Glyceraldehyde-3-phosphate dehydrogenase			2.94
169	FKBP4	Q02790	indirect	FKBP4_HUMAN Peptidyl-prolyl cis-trans isomerase FKBP4			3.25
58	ERG	P11308	direct binding	ERG_HUMAN Transcriptional regulator ERG			3.25
48	NPM1	P06748	direct	NPM_HUMAN Nucleophosmin			3.49
39	NR3C1	P04150	direct binding	GCR_HUMAN Glucocorticoid receptor			3.84
190	NR5A1	Q13285	direct	STF1_HUMAN Steroidogenic factor 1			3.84
249	PSPC1	Q8WXF1	direct, coA	PSPC1_HUMAN Paraspeckle component 1			4.14
46	RB1	P06400	direct	RB_HUMAN Retinoblastoma-associated protein			6.05
159	UBE2I	P63279	direct binding	UBC9_HUMAN SUMO-conjugating enzyme UBC9			7.25
209	NONO	Q15233	direct, coA/coR	NONO_HUMAN Non-POU domain-containing octamer-binding protein			10.00
319	PA2G4	Q9UQ80	direct, coR	PA2G4_HUMAN Proliferation-associated protein 2G4			10.00
162	PRKDC	P78527	indirect binding	PRKDC_HUMAN DNA-dependent protein kinase catalytic subunit			10.00
31	PRPF6	O94906	direct binding	PRP6_HUMAN Pre-mRNA-processing factor 6			10.00
71	DDX5	P17844	direct binding	DDX5_HUMAN Probable ATP-dependent RNA helicase DDX5	18829551	Clark EL et al Cancer Res 68(19) 7938-46 2008	10.00
158	GNB2L1	P63244	direct binding	GBLP_HUMAN Guanine nucleotide-binding protein subunit beta-2-like 1			10.00
351	CDK5	Q00535	direct binding/phosph	Cyclin-dependent kinase 5	21799006	Hsu FN et al J Biol Chem 2011 Sep 23;286(38):33141-9.	10.00

256	DDX17		Q92841	direct	DDX17_HUMAN Probable ATP-dependent RNA helicase DDX17			10.00
54	HNRNPA1		P09651	indirect	ROA1_HUMAN Heterogeneous nuclear ribonucleoprotein A1			10.00
231	ATAD2		Q6PL18	direct, coA	ATAD2_HUMAN ATPase family AAA domain-containing protein 2	20855524		Unique-AS
195	CUL4B		Q13620	indirect binding throug	CUL4B_HUMAN Cullin-4B			Unique-AS
226	EFCAB6		Q5THR3	direct, coA	EFCB6_HUMAN EF-hand calcium-binding domain-containing protein 6			Unique-AS
185	EP300		Q09472	direct	EP300_HUMAN Histone acetyltransferase p300			Unique-AS
51	HSPA1A		P08107	direct	HSP71_HUMAN Heat shock 70 kDa protein 1A/1B			Unique-AS
269	RBM14		Q96PK6	direct	RBM14_HUMAN RNA-binding protein 14			Unique-AS
233	SRCAP		Q6ZRS2	direct, affinity capture	SRCAP_HUMAN Helicase SRCAP			Unique-AS
334	ARD1	NAA10	P41227	direct binding	NAA10 N(alpha)-acetyltransferase 10, NatA catalytic subunit	PMID: 22315407		Unique-AS
8	HNRPDL		O14979	coA	HNRDL_HUMAN Heterogeneous nuclear ribonucleoprotein D-like			Unique-AS
265	PAWR		Q96IZ0	direct, coA	PAWR_HUMAN PRKC apoptosis WT1 regulator protein			Unique-AS
82	SFPQ		P23246	direct bindng	SFPQ_HUMAN Splicing factor, proline- and glutamine-rich			Unique-AS
181	KHDRBS1		Q07666	direct binding	KHDR1_HUMAN KH domain-containing, RNA-binding, signal transduction-associated protein 1			Unique-AS
165	CDK6		Q00534	direct binding	CDK6_HUMAN Cell division protein kinase 6			Unique-AS
129	CDK7		P50613	direct binding	CDK7_HUMAN Cell division protein kinase 7	21157430	Chymkowitch P et al EMBO J 2011	Unique-AS
104	CTNNB1		P35222	direct binding	CTNB1_HUMAN Catenin beta-1			Unique-AS
88	DNAJB1		P25685	direct binding	DNJB1_HUMAN DnaJ homolog subfamily B member 1			Unique-AS

196	DYRK1A	Q13627	indirect	DYR1A_HUMAN Dual specificity tyrosine-phosphorylation-regulated kinase 1A	Unique-AS
290	ENY2	Q9NPA8	coA	ENY2_HUMAN Enhancer of yellow 2 transcription factor homolog	Unique-AS
106	GTF2F1	P35269	direct binding	T2FA_HUMAN General transcription factor IIF subunit 1	Unique-AS
247	HDAC7	Q8WUI4	direct, coR	HDAC7_HUMAN Histone deacetylase 7	Unique-AS
53	HMGB1	P09429	direct binding	HMGB1_HUMAN High mobility group protein B1	Unique-AS
89	HMGB2	P26583	direct binding	HMGB2_HUMAN High mobility group protein B2	Unique-AS
20	KDM1A	O60341	direct binding	KDM1A_HUMAN Lysine-specific histone demethylase 1A (LSD1)	Unique-AS
229	KDM4D	Q6B0I6	direct, coA	KDM4D_HUMAN Lysine-specific demethylase 4D	Unique-AS
309	KDM5B	Q9UGL1	direct, coA	KDM5B_HUMAN Lysine-specific demethylase 5B	Unique-AS
307	RNF14	Q9UBS8	direct, coA	RNF14_HUMAN E3 ubiquitin-protein ligase RNF14	Unique-AS
110	SREBF1	P36956	direct binding	SRBP1_HUMAN Sterol regulatory element-binding protein 1	Unique-AS
182	TNK2	Q07912	direct	ACK1_HUMAN Activated CDC42 kinase 1	Unique-AS
228	TRIM68	Q6AZZ1	direct, coA	TRI68_HUMAN E3 ubiquitin-protein ligase TRIM68	Unique-AS
202	USP10	Q14694	direct	UBP10_HUMAN Ubiquitin carboxyl-terminal hydrolase 10	Unique-AS
278	WDR77	Q9BQA1	direct, coA	MEP50_HUMAN Methylosome protein 50	Unique-AS
34	WHSC1	O96028	direct binding	NSD2_HUMAN Probable histone-lysine N-methyltransferase NSD2	Unique-AS
275	PRMT1	Q99873	indirect, coA	ANM1_HUMAN Protein arginine N-methyltransferase 1	-1.91
99	DNAJA1	P31689	indirect binding	DNJA1_HUMAN DnaJ homolog subfamily A member 1	-1.88
243	ARID1B	Q8NFD5	coA	ARI1B_HUMAN AT-rich interactive domain-containing protein 1B	-1.54

145	TPD52	P55327	may upregulate AR an	TPD52_HUMAN Tumor protein D52				-1.49
179	RUVBL1	Q06455	direct biniding (wester	MTG8_HUMAN Protein CBFA2T1				-1.44
180	PRDX1	Q06830	direct binding	PRDX1_HUMAN Peroxiredoxin-1				-1.31
49	HSP90AA1	P07900	direct	HS90A_HUMAN Heat shock protein HSP 90-alpha				-1.26
272	PARK7	Q99497	direct, coA	PARK7_HUMAN Protein DJ-1				-1.14
274	TSG101	Q99816	indirect, coA/coR	TS101_HUMAN Tumor susceptibility gene 101 protein				-1.13
56	AR	P10275	direct binding	ANDR_HUMAN Androgen receptor				-1.06
189	SELENBP1	Q13228	direct (affinity caputur	SBP1_HUMAN Selenium-binding protein 1				0.88
91	CALR	P27797	direct	CALR_HUMAN Calreticulin				1.04
101	SFN	P31947	coA,	1433S_HUMAN 14-3-3 protein sigma				1.06
174	YWHAH	Q04917	direct	1433F_HUMAN 14-3-3 protein eta				1.07
239	KIAA1967	Q8N163	direct, coA	K1967_HUMAN Protein KIAA1967				1.14
114	STAT3	P40763	direct binding	STAT3_HUMAN Signal transducer and activator of transcription 3				1.15
72	PSMC3	P17980	direct binding	PRS6A_HUMAN 26S protease regulatory subunit 6A		19325002	Satoh T et al Endocrinology 150(7) 3283-90 2009	1.42
90	YWHAQ	P27348	direct binding	1433T_HUMAN 14-3-3 protein theta				1.57
127	GSK3B	P49841	direct binding	GSK3B_HUMAN Glycogen synthase kinase-3 beta				1.72
95	PPP2R1A	P30153	direct binding	2AAA_HUMAN Serine/threonine-protein phosphatase 2A 65 kDa regulatory subunit A alpha isoform				1.83
186	KIF1A	Q12756	direct	KIF1A_HUMAN Kinesin-like protein KIF1A				1.87
296	MDN1	Q9NU22	direct, y-2-hybrid	MDN1_HUMAN Midasin				1.91
298	AATF	Q9NY61	direct, coA	AATF_HUMAN Protein AATF	Leister P et al, Burgdorf S et al	14761944	Signal Transduction and JBC	NA
150	ACTB	P60709	enhance supervillin-m	ACTB_HUMAN Actin, cytoplasmic 1	Ting HJ et al	15178419	BBRC	NA
108	ACTN2	P35609	indirect binding	ACTN2_HUMAN Alpha-actinin-2	Huang SM et al	15072553	J Mol Endocrinol	NA

6	ADAM10	O14672	Direct Binding	ADA10_HUMAN Disintegrin and metalloproteinase domain-containing protein 10	Arima T et al	17727679	Cancer Sci	NA
183	AES	Q08117	direct	AES_HUMAN Amino-terminal enhancer of split				NA
109	AHR	P35869	direct binding	AHR_HUMAN Aryl hydrocarbon receptor				NA
100	AKT1	P31749	direct binding	AKT1_HUMAN RAC-alpha serine/threonine-protein kinase				NA
250	APPBP2	Q92624	direct, coR	APBP2_HUMAN Amyloid protein-binding protein 2				NA
140	ARHGDIA	P52565	enhance AR transcripti	GDIR1_HUMAN Rho GDP-dissociation inhibitor 1				NA
3	ARID1A	O14497	coA	ARI1A_HUMAN AT-rich interactive domain-containing protein 1A				NA
171	ARID5A	Q03989	direct	ARI5A_HUMAN AT-rich interactive domain-containing protein 5A				NA
204	ARRB2	Q14865	direct	ARI5B_HUMAN AT-rich interactive domain-containing protein 5B				NA
68	ATF2	P15336	direct binding	ATF2_HUMAN Cyclic AMP-dependent transcription factor ATF-2				NA
346	ATF3	P18847	direct binding/corepres	Activating transcription factor (ATF3)		22665497	Wang H., et al Mol Cell Biol 2012 Jun 4 Epub ahead of print	NA
206	ATXN7L3	Q14CW9	direct, coA	AT7L3_HUMAN Ataxin-7-like protein 3				NA
276	BAG1	Q99933	direct, coA	BAG1_HUMAN BAG family molecular chaperone regulator 1				NA
111	BRCA1	P38398	direct binding	BRCA1_HUMAN Breast cancer type 1 susceptibility protein				NA
135	BRCA2	P51587	direct binding	BRCA2_HUMAN Breast cancer type 2 susceptibility protein				NA
291	BRD7	Q9NPI1	coR	BRD7_HUMAN Bromodomain-containing protein 7				NA
301	CALCOCO1	Q9P1Z2	indirect, coA	CACO1_HUMAN Calcium-binding and coiled-coil domain-containing protein 1				NA
155	CALM1	P62158	direct binding	CALM_HUMAN Calmodulin				NA
94	CASP1	P29466	direct binding	CASP1_HUMAN Caspase-1				NA
119	CASP3	P42574	direct binding	CASP3_HUMAN Caspase-3				NA
142	CASP7	P55210	direct binding	CASP7_HUMAN Caspase-7				NA

203	CASP8	Q14790	direct	CASP8_HUMAN Caspase-8		NA
170	CAV1	Q03135	direct	CAV1_HUMAN Caveolin-1		NA
85	CCND1	P24385	direct binding	CCND1_HUMAN G1/S-specific cyclin-D1		NA
96	CCND3	P30281	direct binding	CCND3_HUMAN G1/S-specific cyclin-D3		NA
86	CCNE1	P24864	direct binding	CCNE1_HUMAN G1/S-specific cyclin-E1		NA
139	CCNH	P51946	direct binding	CCNH_HUMAN Cyclin-H		NA
97	CDC25A	P30304	direct binding/corepres	MPIP1_HUMAN M-phase inducer phosphatase 1	19013180	NA
98	CDC25B	P30305	direct binding	MPIP2_HUMAN M-phase inducer phosphatase 2		NA
320	CDK11A	Q9UQ88	coA	CD11A_HUMAN Cell division protein kinase 11A		NA
4	CDK2AP1	O14519	coA	CDKA1_HUMAN Cyclin-dependent kinase 2-associated protein 1		NA
131	CDK9	P50750	direct binding	CDK9_HUMAN Cell division protein kinase 9		NA
125	CEBPA	P49715	direct binding	CEBPA_HUMAN CCAAT/enhancer-binding protein alpha		NA
70	CEBPB	P17676	direct binding	CEBPB_HUMAN CCAAT/enhancer-binding protein beta		NA
268	CMTM3	Q96MX0	coR	CKLF3_HUMAN CKLF-like MARVEL transmembrane domain-containing protein 3		NA
248	COBRA1	Q8WX92	direct, coR	NELFB_HUMAN Negative elongation factor B		NA
258	COPS5	Q92905	coA	CSN5_HUMAN COP9 signalosome complex subunit 5		NA
57	COX5B	P10606	direct binding	COX5B_HUMAN Cytochrome c oxidase subunit 5B, mitochondrial		NA
252	CREBBP	Q92793	direct, coA	CBP_HUMAN CREB-binding protein		NA
5	CTDSP2	O14595	Direct Binding	CTDS2_HUMAN Carboxy-terminal domain RNA polymerase II polypeptide A small phosphatase 2		NA
312	DACH1	Q9UI36	coR	DACH1_HUMAN Dachshund homolog 1		NA
132	DAP3	P51398	direct binding	RT29_HUMAN 28S ribosomal protein S29, mitochondrial		NA
308	DAXX	Q9UER7	direct, coR	DAXX_HUMAN Death domain-associated protein 6	15572661	NA
224	DCAF6	Q58WW2	direct, coA	DCAF6_HUMAN DDB1- and CUL4-associated factor 6		NA

348	DDB2	Q92466	direct, target for degrac	DNA damage-binding protein 2	22846800	Chang SW et al Int J Biochem Cell Biolo 2012 July 28 Epub ahead of print	NA
78	DDC	P20711	direct binding	DDC_HUMAN Aromatic-L-amino-acid decarboxylase			NA
230	DENND5A	Q6IQ26	direct, y-2-hybrid	DEN5A_HUMAN DENN domain-containing protein 5A			NA
332	E2F3	O00716	direct/indirect	Cell cycle regulator, transcription factor E2F3	Mallik I et al. Biochim Biophys Acta 1783:1737-1744, 2008		NA
35	EGFR	P00533	direct binding	EGFR_HUMAN Epidermal growth factor receptor			NA
73	EGR1	P18146	direct binding	EGR1_HUMAN Early growth response protein 1			NA
42	ERBB2	P04626	indirect binding	ERBB2_HUMAN Receptor tyrosine-protein kinase erbB-2			NA
37	ESR1	P03372	direct binding	ESR1_HUMAN Estrogen receptor			NA
67	ETS1	P14921	direct binding	ETS1_HUMAN Protein C-ets-1			NA
128	ETV1	P50549	direct binding	ETV1_HUMAN ETS translocation variant 1			NA
115	ETV5	P41161	direct binding	ETV5_HUMAN ETS translocation variant 5			NA
200	FHL2	Q14192	direct	FHL2_HUMAN Four and a half LIM domains protein 2	17975004	Heemers HV, et al Cancer Res2007	NA
191	FKBP5	Q13451	indirect	FKBP5_HUMAN Peptidyl-prolyl cis-trans isomerase FKBP5			NA
36	FOS	P01100	direct binding	FOS_HUMAN Proto-oncogene c-Fos			NA
144	FOXA1	P55317	direct binding	FOXA1_HUMAN Hepatocyte nuclear factor 3-alpha			NA
322	FOXA2	Q9Y261	direct, coA/coR	FOXA2_HUMAN Hepatocyte nuclear factor 3-beta			NA
143	FOXG1	P55316	direct binding	FOXG1_HUMAN Forkhead box protein G1			NA
28	FOXH1	O75593	direct binding	FOXH1_HUMAN Forkhead box protein H1			NA
187	FOXO1	Q12778	direct	FOXO1_HUMAN Forkhead box protein O1			NA

14	FOXO3	O43524	Direct Binding	FOXO3_HUMAN Forkhead box protein O3			NA
284	FOXP1	Q9H334	direct, coR	FOXP1_HUMAN Forkhead box protein P1			NA
244	GADD45GIP1	Q8TAE8	direct, coR	G45IP_HUMAN Growth arrest and DNA damage-inducible proteins-interacting protein 1			NA
83	GATA2	P23769	direct binding	GATA2_HUMAN Endothelial transcription factor GATA-2	25489091	He B et al Proc Natl Acad Sci U S A. 2014	NA
84	GATA3	P23771	coA	GATA3_HUMAN Trans-acting T-cell-specific transcription factor GATA-3			NA
324	GRIP1	Q9Y3R0	direct, coA	GRIP1_HUMAN Glutamate receptor-interacting protein 1			NA
126	GSK3A	P49840	direct binding	GSK3A_HUMAN Glycogen synthase kinase-3 alpha			NA
45	GSN	P06396	direct	GELS_HUMAN Gelsolin			NA
64	GTF2F2	P13984	direct binding	T2FB_HUMAN General transcription factor IIF subunit 2			NA
102	GTF2H1	P32780	direct binding	TF2H1_HUMAN General transcription factor IIH subunit 1			NA
141	HAP1	P54257	direct binding	HAP1_HUMAN Huntingtin-associated protein 1			NA
193	HDAC1	Q13547	direct	HDAC1_HUMAN Histone deacetylase 1			NA
10	HDAC3	O15379	Direct Binding	HDAC3_HUMAN Histone deacetylase 3			NA
148	HDAC4	P56524	direct binding	HDAC4_HUMAN Histone deacetylase 4			NA
305	HDAC6	Q9UBN7	indirect, coA	HDAC6_HUMAN Histone deacetylase 6			NA
328	HEY1	Q9Y5J3	direct, coR	HEY1_HUMAN Hairy/enhancer-of-split related with YRPW motif protein 1			NA
306	HEY2	Q9UBP5	coR	HEY2_HUMAN Hairy/enhancer-of-split related with YRPW motif protein 2			NA
222	HIF1A	Q16665	direct, coA	HIF1A_HUMAN Hypoxia-inducible factor 1-alpha			NA
2	HIP1	O00291	Direct Binding	HIP1_HUMAN Huntingtin-interacting protein 1			NA
286	HIPK3	Q9H422	direct, coA	HIPK3_HUMAN Homeodomain-interacting protein kinase 3			NA

116	HNF4A		P41235	colocalization	HNF4A_HUMAN Hepatocyte nuclear factor 4-alpha			NA
253	HOXB13		Q92826	direct, coR	HXB13_HUMAN Homeobox protein Hox-B13			NA
280	HTATIP2		Q9BUP3	coA	HTAI2_HUMAN Oxidoreductase HTATIP2			NA
65	IDE		P14735	direct binding	IDE_HUMAN Insulin-degrading enzyme			NA
223	IFI16		Q16666	direct, coR	IF16_HUMAN Gamma-interferon-inducible protein 16			NA
113	IL6ST		P40189	direct binding	IL6RB_HUMAN Interleukin-6 receptor subunit beta			NA
107	IRS1		P35568	direct binding	IRS1_HUMAN Insulin receptor substrate 1			NA
215	JMJD1C		Q15652	direct, coA	JHD2C_HUMAN Probable JmjC domain-containing histone demethylation protein 2C			NA
44	JUN		P05412	direct	JUN_HUMAN Transcription factor AP-1			NA
341	JunD		P17535	directbinding/coA	Transcription factor jun-D	20460526	Mehraein-Ghomi F et al Cancer Res 70(11) 4560-8	NA
254	KAT2A		Q92830	coA	KAT2A_HUMAN Histone acetyltransferase KAT2A			NA
255	KAT2B		Q92831	direct, coA	KAT2B_HUMAN Histone acetyltransferase KAT2B			NA
333	KAT7	MYST2	O95251	direct binding	KAT7_HUMAN Histone acetyltransferase			NA
326	KDM3A		Q9Y4C1	direct, coA	KDM3A_HUMAN Lysine-specific demethylase 3A			NA
24	KDM4A		O75164	direct binding	KDM4A_HUMAN Lysine-specific demethylase 4A (JMJD2A)			NA
285	KDM4C		Q9H3R0	direct, coA	KDM4C_HUMAN Lysine-specific demethylase 4C			NA
350	L1RE1	LINE-1 OR	Q9UN81	direct binding/coA	The pro-oncogene human Long Interspersed Nucleotide acid Element-1 (LINE-1) encodes LINE-1 ORF-1p	23153584	Lu Y et al Cell Signal. 2012 Nov; 25(2):479-489	NA
295	LATS2		Q9NRM7	direct, coR	LATS2_HUMAN Serine/threonine-protein kinase LATS2			NA
336	LCOR		Q96JN0	direct binding	Ligand-dependent corepressor	21856747		NA
22	LPXN		O60711	direct binding	LPXN_HUMAN Leupaxin			NA

277	MACROD1	Q9BQ69	direct, coA	MACD1_HUMAN MACRO domain-containing protein 1			NA
120	MAGEA11	P43364	direct binding	MAGAB_HUMAN Melanoma-associated antigen 11	18212060	Bai S, and Wilson EM Mol Cell Biol 2008	NA
79	MAK	P20794	direct binding	MAK_HUMAN Serine/threonine-protein kinase MAK			NA
93	MAPK1	P28482	direct binding	MK01_HUMAN Mitogen-activated protein kinase 1			NA
246	MAPK15	Q8TD08	indirect, coR	MK15_HUMAN Mitogen-activated protein kinase 15			NA
166	MDM2	Q00987	direct binding	MDM2_HUMAN E3 ubiquitin-protein ligase Mdm2			NA
236	MED12L	Q86YW9	direct, y-2-hybrid	MD12L_HUMAN Mediator of RNA polymerase II transcription subunit 12-like protein			NA
19	MED14	O60244	Direct Binding	MED14_HUMAN Mediator of RNA polymerase II transcription subunit 14			NA
297	MED17	Q9NVC6	coA	MED17_HUMAN Mediator of RNA polymerase II transcription subunit 17			NA
26	MED24	O75448	direct binding	MED24_HUMAN Mediator of RNA polymerase II transcription subunit 24			NA
311	MKRN1	Q9UHC7	coR	MKRN1_HUMAN E3 ubiquitin-protein ligase makorin-1			NA
310	MLH3	Q9UHC1	direct, y-2-hybrid	MLH3_HUMAN DNA mismatch repair protein Mlh3			NA
279	MYBBP1A	Q9BQG0	direct	MBB1A_HUMAN Myb-binding protein 1A			NA
216	NCOA1	Q15788	direct, coA	NCOA1_HUMAN Nuclear receptor coactivator 1			NA
330	NCOA3	Q9Y6Q9	direct, coA	NCOA3_HUMAN Nuclear receptor coactivator 3			NA
197	NCOA4	Q13772	direct	NCOA4_HUMAN Nuclear receptor coactivator 4			NA
201	NCOA6	Q14686	indirect	NCOA6_HUMAN Nuclear receptor coactivator 6			NA
329	NCOR2	Q9Y618	direct, coR	NCOR2_HUMAN Nuclear receptor corepressor 2			NA
205	NFIX	Q14938	direct, coA	NFIX_HUMAN Nuclear factor 1 X-type			NA
75	NFKB1	P19838	interact with p65, p52 r	NFKB1_HUMAN Nuclear factor NF-kappa-B p105 subunit			NA
303	NLK	Q9UBE8	direct, coR	NLK_HUMAN Serine/threonine-protein kinase NLK			NA

234	NLRP10	Q86W26	direct, y-2-hybrid	NAL10_HUMAN NACHT, LRR and PYD domains-containing protein 10			NA
138	NR0B1	P51843	direct binding	NR0B1_HUMAN Nuclear receptor subfamily 0 group B member 1			NA
210	NR0B2	Q15466	direct, coR	NR0B2_HUMAN Nuclear receptor subfamily 0 group B member 2			NA
63	NR2C1	P13056	direct binding	NR2C1_HUMAN Nuclear receptor subfamily 2 group C member 1			NA
124	NR2C2	P49116	direct binding	NR2C2_HUMAN Nuclear receptor subfamily 2 group C member 2			NA
294	PAK6	Q9NQU5	direct, coR	PAK6_HUMAN Serine/threonine-protein kinase PAK 6			NA
288	PATZ1	Q9HBE1	indirect, coR	PATZ1_HUMAN POZ-, AT hook-, and zinc finger-containing protein 1			NA
338	PAX6	P26367	directbinding/coR	paired box protein Pax6	21935435	Elvenes J et al PLoS One 6(9) 2011	NA
238	PELP1	Q8IZL8	direct, coA	PELP1_HUMAN Proline-, glutamic acid- and leucine-rich protein 1			NA
105	PHB	P35232	indirect binding	PHB_HUMAN Prohibitin			NA
29	PIAS1	O75925	direct binding	PIAS1_HUMAN E3 SUMO-protein ligase PIAS1			NA
30	PIAS2	O75928	direct binding	PIAS2_HUMAN E3 SUMO-protein ligase PIAS2			NA
331	PIAS3	Q9Y6X2	direct, coA/coR	PIAS3_HUMAN E3 SUMO-protein ligase PIAS3			NA
240	PIAS4	Q8N2W9	direct, coR	PIAS4_HUMAN E3 SUMO-protein ligase PIAS4			NA
335	PIM1	P11309	direct binding	Serine/threonine-protein kinase pim-1	PMID: 22584579		NA
220	PKN1	Q16512	direct, coA	PKN1_HUMAN Serine/threonine-protein kinase N1			NA
317	PLAGL1	Q9UM63	direct, coA	PLAL1_HUMAN Zinc finger protein PLAGL1			NA
262	PMEPA1	Q969W9	direct	PMEPA_HUMAN Transmembrane prostate androgen-induced protein			NA
188	PNRC1	Q12796	direct	PNRC1_HUMAN Proline-rich nuclear receptor coactivator 1			NA
292	PNRC2	Q9NPJ4	direct	PNRC2_HUMAN Proline-rich nuclear receptor coactivator 2			NA
66	POU2F1	P14859	direct binding	PO2F1_HUMAN POU domain, class 2, transcription factor 1 (Oct1)			NA
52	POU2F2	P09086	direct binding	PO2F2_HUMAN POU domain, class 2, transcription factor 2			NA

77	POU3F3		P20264	direct biniding	PO3F3_HUMAN POU domain, class 3, transcription factor 3			NA
184	PPID		Q08752	direct	PPID_HUMAN Peptidyl-prolyl cis-trans isomerase D			NA
154	PPP1CA		P62136	direct bindng	PP1A_HUMAN Serine/threonine-protein phosphatase PP1-alpha catalytic subunit			NA
23	PQBP1		O60828	Direct Binding	PQBP1_HUMAN Polyglutamine-binding protein 1			NA
345	PRGC1	PPARGC1	Q9UBK2	direct binding/coA	Peroxisome proliferator-activated receptor gamma coactivator 1-alpha	19884383	Shiota M., et al Mol Endocrinol 24(1):114-27	NA
282	PRIC285		Q9BYK8	coA	PR285_HUMAN Peroxisomal proliferator-activated receptor A-interacting complex 285 kDa protein			NA
146	PRMT2		P55345	direct binding	ANM2_HUMAN Protein arginine N-methyltransferase 2			NA
302	PSMC3IP		Q9P2W1	direct, coA	HOP2_HUMAN Homologous-pairing protein 2 homolog	19325002	Satoh T et al Endocrinology 150(7) 3283-90 2009	NA
123	PXN		P49023	direct binding	PAXI_HUMAN Paxillin			NA
151	RAC3		P60763	direct binding	RAC3_HUMAN Ras-related C3 botulinum toxin substrate 3			NA
325	RAD54L2		Q9Y4B4	direct, coA	ARIP4_HUMAN Helicase ARIP4	17003240	Domanskyi A et al Am J Physiol Metab 2007	NA
273	RAD9A		Q99638	direct, coR	RAD9A_HUMAN Cell cycle checkpoint control protein RAD9A			NA
38	RAF1		P04049	direct binding	RAF1_HUMAN RAF proto-oncogene serine/threonine-protein kinase			NA
156	RAN		P62826	direct binding	RAN_HUMAN GTP-binding nuclear protein Ran	18565325	Harada N et al 373(3) 373-7 2008	NA
232	RANBP10		Q6VN20	direct, coA	RBP10_HUMAN Ran-binding protein 10			NA
271	RANBP9		Q96S59	direct, coA	RANB9_HUMAN Ran-binding protein 9			NA
299	RBAK		Q9NYW8	direct, coA	RBAK_HUMAN RB-associated KRAB zinc finger protein			NA

270	RCHY1		Q96PM5	direct, coA	ZN363_HUMAN RING finger and CHY zinc finger domain-containing protein 1 (PIRH2)	16914734	Logan IR et al., Mol Cell Biol 2006	NA
173	REL		Q04864	direct	REL_HUMAN Proto-oncogene c-Rel			NA
172	RELA		Q04206	direct	TF65_HUMAN Transcription factor p65			NA
178	RNASEL		Q05823	direct	RN5A_HUMAN 2-5A-dependent ribonuclease			NA
161	RNF4		P78317	direct binding	RNF4_HUMAN RING finger protein 4			NA
137	RPS6KA3		P51812	modulate PSA express	KS6A3_HUMAN Ribosomal protein S6 kinase alpha-3			NA
251	RREB1		Q92766	direct, coR	RREB1_HUMAN Ras-responsive element-binding protein 1	17550981	Mukhopadhyay NK et al Mol Endocrinol 2007	NA
167	RUNX1		Q01196	direct binding	RUNX1_HUMAN Runt-related transcription factor 1			NA
198	RUNX2		Q13950	direct	RUNX2_HUMAN Runt-related transcription factor 2			NA
287	RWDD1		Q9H446	coA	RWDD1_HUMAN RWD domain-containing protein 1	22406838	Grotsch H et al Mol Cell Endocrinol 2012	NA
74	RXRA		P19793	direct binding	RXRA_HUMAN Retinoic acid receptor RXR-alpha			NA
207	SART3		Q15020	direct, coR	SART3_HUMAN Squamous cell carcinoma antigen recognized by T-cells 3			NA
340	SET9	SETD7	Q8WTS6	directbinding/coA	Histone-lysine N-methyltransferase SETD7 (SET9)	21273441	Ko S et al Mol Endorinol 25(3):433-44 2011	NA
241	SFRP1		Q8N474	coR	SFRP1_HUMAN Secreted frizzled-related protein 1			NA
1	SGK1		O00141	coR, no direct binding	SGK1_HUMAN Serine/threonine-protein kinase Sgk1			NA
263	SIRT1		Q96EB6	direct, coR	SIRT1_HUMAN NAD-dependent deacetylase sirtuin-1			NA
59	SLC25A4		P12235	direct binding (y-2-h)	ADT1_HUMAN ADP/ATP translocase 1			NA

347	SLUG	SNAI2	O43623	coactivator	Zinc finger protein SNAI2	22745193	Wu K., et al Mol Endocriol 2012 Jun 28 Epub ahead of print	NA
217	SMAD1		Q15797	direct, coR	SMAD1_HUMAN Mothers against decapentaplegic homolog 1			NA
164	SMAD3		P84022	direct binding	SMAD3_HUMAN Mothers against decapentaplegic homolog 3			NA
192	SMAD4		Q13485	direct	SMAD4_HUMAN Mothers against decapentaplegic homolog 4			NA
133	SMARCA2		P51531	imapct AR-mediated tra	SMCA2_HUMAN Probable global transcription activator SNF2L2			NA
134	SMARCA4		P51532	imapct AR-mediated tra	SMCA4_HUMAN Transcription activator BRG1			NA
259	SMARCC1		Q92922	direct, coA	SMRC1_HUMAN SWI/SNF complex subunit SMARCC1			NA
261	SMARCE1		Q969G3	direct, coA	SMCE1_HUMAN SWI/SNF-related matrix-associated actin-dependent regulator of chromatin subfamily E member 1			NA
40	SOD2		P04179	coR, no direct binding	SODM_HUMAN Superoxide dismutase [Mn], mitochondrial			NA
21	SORBS3		O60504	direct binding	VINEX_HUMAN Vinexin			NA
121	SOX9		P48436	direct binding	SOX9_HUMAN Transcription factor SOX-9			NA
50	SP1		P08047	direct	SP1_HUMAN Transcription factor Sp1			NA
339	SPBP	TCF20	Q9UGU0	directbinding/coA	transcription factor 20	21935435	Elvenes J et al PLoS One 6(9) 2011	NA
32	SPDEF		O95238	direct binding	SPDEF_HUMAN SAM pointed domain-containing Ets transcription factor			NA
18	SPOP		O43791	Direct Binding (y-2-h)	SPOP_HUMAN Speckle-type POZ protein			NA
289	SRA1		Q9HD15	direct, coA/coR	SRA1_HUMAN Steroid receptor RNA activator 1			NA
60	SRC		P12931	direct binding	SRC_HUMAN Proto-oncogene tyrosine-protein kinase Src			NA
175	SRY		Q05066	direct	SRY_HUMAN Sex-determining region Y protein			NA
117	STAT5A		P42229	direct binding	STA5A_HUMAN Signal transducer and activator of transcription 5A			NA

136	STAT5B	P51692	direct binding	STA5B_HUMAN Signal transducer and activator of transcription 5B			NA
157	SUMO1	P63165	coR	SUMO1_HUMAN Small ubiquitin-related modifier 1			NA
153	SUMO2	P61956	coA	SUMO2_HUMAN Small ubiquitin-related modifier 2			NA
27	TADA3	O75528	direct binding	TADA3_HUMAN Transcriptional adapter 3			NA
344	TAF1	P21675	direct binding/coA	TATA binding protein-associated factor 1	20181722	Tavassoli P et al Mol Endocrinol 24(4) 696-708	NA
168	TAGLN	Q01995	indirect	TAGL_HUMAN Transgelin			NA
76	TBP	P20226	direct binding	TBP_HUMAN TATA-box-binding protein			NA
15	TCF21	O43680	Direct Binding	TCF21_HUMAN Transcription factor 21			NA
69	TCF4	P15884	direct binding	ITF2_HUMAN Transcription factor 4			NA
293	TCF7L2	Q9NQB0	direct, coR	TF7L2_HUMAN Transcription factor 7-like 2			NA
194	TDG	Q13569	direct	TDG_HUMAN G/T mismatch-specific thymine DNA glycosylase			NA
13	TGFB1I1	O43294	Direct Binding	TGFI1_HUMAN Transforming growth factor beta-1-induced transcript 1 protein			NA
163	TMF1	P82094	direct binding	TMF1_HUMAN TATA element modulatory factor			NA
130	TOB1	P50616	direct binding	TOB1_HUMAN Protein Tob1			NA
199	TOB2	Q14106	indirect, coR, suppress	TOB2_HUMAN Protein Tob2			NA
43	TP53	P04637	indirect binding	P53_HUMAN Cellular tumor antigen p53			NA
9	TRIM24	O15164	Direct Binding	TIF1A_HUMAN Transcription intermediary factor 1-alpha			NA
214	TRIP4	Q15650	direct, coA	TRIP4_HUMAN Activating signal cointegrator 1			NA
245	UBA3	Q8TBC4	indirect, coR	UBA3_HUMAN NEDD8-activating enzyme E1 catalytic subunit			NA
55	UBC	P0CG48	ubiquitin	UBC_HUMAN Polyubiquitin-C			NA
160	UBE2L3	P68036	coA	UB2L3_HUMAN Ubiquitin-conjugating enzyme E2 L3			NA
176	UBE3A	Q05086	direct	UBE3A_HUMAN Ubiquitin-protein ligase E3A			NA

218	USF2		Q15853	direct/indirect, coA	USF2_HUMAN Upstream stimulatory factor 2			NA
318	USP22		Q9UPT9	direct, coA	UBP22_HUMAN Ubiquitin carboxyl-terminal hydrolase 22			NA
281	USP26		Q9BXU7	direct, affinity capture-v	UBP26_HUMAN Ubiquitin carboxyl-terminal hydrolase 26			NA
304	UXT		Q9UBK9	direct, coA	UXT_HUMAN Protein UXT			NA
314	VAV3		Q9UKW4	indirect, coA	VAV3_HUMAN Guanine nucleotide exchange factor VAV3	21765461	Rao S et al Oncogene 2012	NA
225	WIPI1		Q5MNZ9	direct	WIPI1_HUMAN WD repeat domain phosphoinositide-interacting protein 1			NA
87	YY1		P25490	direct binding	TYY1_HUMAN Transcriptional repressor protein YY1			NA
323	ZBTB1		Q9Y2K1	direct, y-2-hybrid	ZBTB1_HUMAN Zinc finger and BTB domain-containing protein 1			NA
177	ZBTB16		Q05516	direct (y-2-hybrid)	ZBT16_HUMAN Zinc finger and BTB domain-containing protein 16			NA
242	ZMIZ2		Q8NF64	direct, coA	ZMIZ2_HUMAN Zinc finger MIZ domain-containing protein 2			NA
227	ZNF318		Q5VUA4	direct, coR	ZN318_HUMAN Zinc finger protein 318			NA
337	ZNF366	DC-SCRIP	Q8N895	co-IP	Zinc finger protein 366	22473304	Ansems M et al Prostate	NA
327	ZNF451		Q9Y4E5	direct/indirect, coA	ZN451_HUMAN Zinc finger protein 451	18656483	Karvonen et al J. Mol Biol 2008	NA

Supplemental Table 3. Summary of siRNA luciferase screen for modulators of AR-mediated transcription.					
**E3 ubiquitin protein ligases**	**Description**	**Effect**	**% increase (+) or decrease (-)**		
NEDD4L	E3 ubiquitin-protein ligase which accepts ubiquitin from an E2 ubiquitin-conjugating enzyme in the form of a thioester and then directly transfers the ubiquitin to targeted substrates. Inhibits TGF-beta signaling by triggering SMAD2 and TGFBR1 ubiquitination and proteasome-dependent degradation. Promotes ubiquitination and internalization of various plasma membrane channels such as ENaC, Nav1.2, Nav1.3, Nav1.5, Nav1.7, Nav1.8, Kv1.3, EAAT1 or CLC5. Promotes ubiquitination and degradation of SGK1 and TNK2.	Potentiate	48%		
CHFR	E3 ubiquitin-protein ligase that functions in the antephase checkpoint by actively delaying passage into mitosis in response to microtubule poisons. Acts in early prophase before chromosome condensation, when the centrosome move apart from each other along the periphery of the nucleus. Probably involved in signaling the presence of mitotic stress caused by microtubule poisons by mediating the 'Lys-48'-linked ubiquitination of target proteins, leading to their degradation by the proteasome. Promotes the ubiquitination and subsequent degradation of AURKA and PLK1. Probably acts as a tumor suppressor, possibly by mediating the polyubiquitination of HDAC1, leading to its degradation. May also promote the formation of 'Lys-63'-linked polyubiquitin chains and functions with the specific ubiquitin-conjugating UBC13-MMS2 (UBE2N-UBE2V2) heterodimer. Substrates that are polyubiquitinated at 'Lys-63' are usually not targeted for degradation, but are rather involved in signaling cellular stress.	Potentiate	42%		
PELI1	E3 ubiquitin ligase catalyzing the covalent attachment of ubiquitin moieties onto substrate proteins. Involved in the TLR and IL-1 signaling pathways via interaction with the complex containing IRAK kinases and TRAF6. Mediates 'Lys-63'-linked polyubiquitination of IRAK1 allowing subsequent NF-kappa-B activation.	Potentiate	48%		
RNF6	E3 ubiquitin-protein ligase mediating 'Lys-48'-linked polyubiquitination of LIMK1 and its subsequent targeting to the proteasome for degradation. Negatively regulates axonal outgrowth through regulation of the LIMK1 turnover. Mediates 'Lys-6' and 'Lys-27'-linked polyubiquitination of AR/androgen receptor thereby modulating its transcriptional activity. May also bind DNA and function as a transcriptional regulator.	Potentiate	39%		
STUB1	E3 ubiquitin-protein ligase which targets misfolded chaperone substrates towards proteasomal degradation. Collaborates with ATXN3 in the degradation of misfolded chaperone substrates: ATXN3 restricting the length of ubiquitin chain attached to STUB1/CHIP substrates and preventing further chain extension. Ubiquitinates NOS1 in concert with Hsp70 and Hsp40. Modulates the activity of several chaperone complexes, including Hsp70, Hsc70 and Hsp90. Mediates transfer of non-canonical short ubiquitin chains to HSPA8 that have no effect on HSPA8 degradation. Mediates polyubiquitination of DNA polymerase beta (POLB) at 'Lys-41', 'Lys-61' and 'Lys-81', thereby playing a role in base-excision repair: catalyzes polyubiquitination by amplifying the HUWE1/ARF-BP1-dependent monoubiquitination and leading to POLB-degradation by the proteasome. Mediates polyubiquitination of CYP3A4. Ubiquitinates EPHA2 and may regulate the receptor stability and activity through proteasomal degradation.	Potentiate	34%		
MDM2	E3 ubiquitin-protein ligase that mediates ubiquitination of p53/TP53, leading to its degradation by the proteasome. Inhibits p53/TP53- and p73/TP73-mediated cell cycle arrest and apoptosis by binding its transcriptional activation domain. Also acts as a ubiquitin ligase E3 toward itself and ARRB1. Permits the nuclear export of p53/TP53. Promotes proteasome-dependent ubiquitin-independent degradation of retinoblastoma RB1 protein. Inhibits DAXX-mediated apoptosis by inducing its ubiquitination and degradation. Component of the TRIM28/KAP1-MDM2-p53/TP53 complex involved in stabilizing p53/TP53. Also component of the TRIM28/KAP1-ERBB4-MDM2 complex which links growth factor and DNA damage response pathways. Mediates ubiquitination and subsequent proteasome degradation of DYRK2 in nucleus. Ubiquitinates IGF1R and SNAI1 and promotes them to proteasomal degradation.	attenuate	-64%		
TRIM68	Functions as a ubiquitin E3 ligase. Acts as a coactivator of androgen receptor (AR) depending on its ubiquitin ligase activity.	attenuate	-74%		
HACE1	E3 ubiquitin-protein ligase involved in Golgi membrane fusion and regulation of small GTPases. Acts as a regulator of Golgi membrane dynamics during the cell cycle: recruited to Golgi membrane by Rab proteins and regulates postmitotic Golgi membrane fusion. Acts by mediating ubiquitination during mitotic Golgi disassembly, ubiquitination serving as a signal for Golgi reassembly later, after cell division. Specifically interacts with GTP-bound RAC1, mediating ubiquitination and subsequent degradation of active RAC1, thereby playing a role in host defense against pathogens. May also act as a transcription regulator via its interaction with RARB.	attenuate	-22%		
LRSAM1	E3 ubiquitin-protein ligase that mediates monoubiquitination of TSG101 at multiple sites, leading to inactivate the ability of TSG101 to sort endocytic (EGF receptors) and exocytic (HIV-1 viral proteins) cargos.	attenuate	-34%		
TRIM41	Functions as an E3 ligase that catalyzes the ubiquitin-mediated degradation of protein kinase C.	attenuate	-52%		

TOPORS	Functions as an E3 ubiquitin-protein ligase and as an E3 SUMO1-protein ligase. Probable tumor suppressor involved in cell growth, cell proliferation and apoptosis that regulates p53/TP53 stability through ubiquitin-dependent degradation. May regulate chromatin modification through sumoylation of several chromatin modification-associated proteins. May be involved in DNA damage-induced cell death through IKBKE sumoylation.	attenuate	-62%		
RNF123	Catalytic subunit of the KPC complex that acts as E3 ubiquitin-protein ligase. Required for poly-ubiquitination and proteasome-mediated degradation of CDKN1B during G1 phase of the cell cycle.	attenuate	-62%		
UBR4	E3 ubiquitin-protein ligase which is a component of the N-end rule pathway. Recognizes and binds to proteins bearing specific N-terminal residues that are destabilizing according to the N-end rule, leading to their ubiquitination and subsequent degradation. Together with clathrin, forms meshwork structures involved in membrane morphogenesis and cytoskeletal organization. Regulates integrin-mediated signaling. May play a role in activation of FAK in response to cell-matrix interactions. Mediates ubiquitination of ACLY, leading to its subsequent degradation.	attenuate	-16%		
HECTD3	E3 ubiquitin ligases accepts ubiquitin from an E2 ubiquitin-conjugating enzyme in the form of a thioester and then directly transfers the ubiquitin to targeted substrates. Mediates ubiquitination of TRIOBP and its subsequent proteasomal degradation, thus faciliting cell cycle progression by regulating the turn-over of TRIOBP. Mediates also ubiquitination of STX8	No effect	14%		
MARCH7	E3 ubiquitin-protein ligase which may specifically enhance the E2 activity of HIP2. E3 ubiquitin ligases accept ubiquitin from an E2 ubiquitin-conjugating enzyme in the form of a thioester and then directly transfer the ubiquitin to targeted substrates.	attenuate	-21%		
SMURF1	E3 ubiquitin-protein ligase that acts as a negative regulator of BMP signaling pathway. Mediates ubiquitination and degradation of SMAD1 and SMAD5, 2 receptor-regulated SMADs specific for the BMP pathway. Promotes ubiquitination and subsequent proteasomal degradation of TRAF family members and RHOA.	attenuate	-14%		
CBL	Adapter protein that functions as a negative regulator of many signaling pathways that are triggered by activation of cell surface receptors. Acts as an E3 ubiquitin-protein ligase, which accepts ubiquitin from specific E2 ubiquitin-conjugating enzymes, and then transfers it to substrates promoting their degradation by the proteasome. Recognizes activated receptor tyrosine kinases, including KIT, FLT1, FGFR1, FGFR2, PDGFRA, PDGFRB, EGFR, CSF1R, EPHA8 and KDR and terminates signaling. Recognizes membrane-bound HCK, SRC and other kinases of the SRC family and mediates their ubiquitination and degradation. Participates in signal transduction in hematopoietic cells. Plays an important role in the regulation of osteoblast differentiation and apoptosis. Essential for osteoclastic bone resorption. The 'Tyr-731' phosphorylated form induces the activation and recruitment of phosphatidylinositol 3-kinase to the cell membrane in a signaling pathway that is critical for osteoclast function. May be functionally coupled with the E2 ubiquitin-protein ligase UB2D3.	attenuate	-44%		
TRIP12	E3 ubiquitin-protein ligase involved in ubiquitin fusion degradation (UFD) pathway and regulation of DNA repair. Part of the ubiquitin fusion degradation (UFD) pathway, a process that mediates ubiquitination of protein at their N-terminus, regardeless of the presence of lysine residues in target proteins. In normal cells, mediates ubiquitination and degradation of isoform p19ARF/ARFof CDKN2A, a lysine-less tumor suppressor required for p53/TP53 activation under oncogenic stress. In cancer cells, however, isoform p19ARF/ARFand TRIP12 are located in different cell compartments, preventing isoform p19ARF/ARFubiquitination and degradation. Does not mediate ubiquitination of isoform p16-INK4aof CDKN2A. Also catalyzes ubiquitination of NAE1 and SMARCE1, leading to their degradation. Ubiquitination and degradation of target proteins is regulated by interaction with proteins such as MYC, TRADD or SMARCC1, which disrupt the interaction between TRIP12 and target proteins. Acts as a key regulator of DNA damage response by acting as a suppressor of RNF168, an E3 ubiquitin-protein ligase that promotes accumulation of 'Lys-63'-linked histone H2A and H2AX at DNA damage sites, thereby acting as a guard against excessive spreading of ubiquitinated chromatin at damaged chromosomes.	attenuate	-74%		
TRIM25	Functions as a ubiquitin E3 ligase and as an ISG15 E3 ligase. Involved in innate immune defense against viruses by mediating ubiquitination of DDX58. Mediates 'Lys-63'-linked polyubiquitination of the DDX58 N-terminal CARD-like region which is crucial for triggering the cytosolic signal transduction that leads to the production of interferons in response to viral infection. Promotes ISGylation of 14-3-3 sigma (SFN), an adapter protein implicated in the regulation of a large spectrum signaling pathway. Mediates estrogen action in various target organs.	attenuate	-80%		
HUWE1	E3 ubiquitin-protein ligase which mediates ubiquitination and subsequent proteasomal degradation of target proteins. Regulates apoptosis by catalyzing the polyubiquitination and degradation of MCL1. Mediates monoubiquitination of DNA polymerase beta (POLB) at 'Lys-41', 'Lys-61' and 'Lys-81', thereby playing a role in base-excision repair. Also ubiquitinates the p53/TP53 tumor suppressor and core histones including H1, H2A, H2B, H3 and H4. Binds to an upstream initiator-like sequence in the preprodynorphin gene. Regulates neural differentiation and proliferation by catalyzing the polyubiquitination and degradation of MYCN. May regulate abundance of CDC6 after DNA damage by polyubiquitinating and targeting CDC6 to degradation.	attenuate	-25%		

Sumoylation	Description	Effect	% increase (+) or decrease (-)		
CBX4	E3 SUMO-protein ligase CBX4	attenuate	-61%		
SUMO3	Small ubiquitin-related modifier 3	attenuate	-73%		
UBE2I	SUMO-conjugating enzyme UBC9	No effect	-14%		
RANBP2	E3 SUMO-protein ligase RanBP2	No effect	-9%		
**De-ubiquitinases**	**Description**	**Effect**	**% increase (+) or decrease (-)**		
USP10	Hydrolase that can remove conjugated ubiquitin from target proteins such as p53/TP53, BECN1, SNX3 and CFTR. Acts as an essential regulator of p53/TP53 stability: in unstressed cells, specifically deubiquitinates p53/TP53 in the cytoplasm, leading to counteract MDM2 action and stabilize p53/TP53. Following DNA damage, translocates to the nucleus and deubiquitinates p53/TP53, leading to regulate the p53/TP53-dependent DNA damage response. Component of a regulatory loop that controls autophagy and p53/TP53 levels: mediates deubiquitination of BECN1, a key regulator of autophagy, leading to stabilize the PIK3C3/VPS34-containing complexes. In turn, PIK3C3/VPS34-containing complexes regulate USP10 stability, suggesting the existence of a regulatory system by which PIK3C3/VPS34-containing complexes regulate p53/TP53 protein levels via USP10 and USP13. Does not deubiquitinate MDM2. Deubiquitinates CFTR in early endosomes, enhancing its endocytic recycling.	Potentiate	186%		
USP20	Deubiquitinating enzyme involved in beta-2 adrenergic receptor (ADRB2) recycling. Acts as a regulator of G-protein coupled receptor (GPCR) signaling by mediating the deubiquitination beta-2 adrenergic receptor (ADRB2). Plays a central role in ADRB2 recycling and resensitization after prolonged agonist stimulation by constitutively binding ADRB2, mediating deubiquitination of ADRB2 and inhibiting lysosomal trafficking of ADRB2. Upon dissociation, it is probably transferred to the translocated beta-arrestins, possibly leading to beta-arrestins deubiquitination and disengagement from ADRB2. This suggests the existence of a dynamic exchange between the ADRB2 and beta-arrestins. Deubiquitinates DIO2, thereby regulating thyroid hormone regulation. Deubiquitinates HIF1A, leading to stabilize HIF1A and enhance HIF1A-mediated activity. Mediates deubiquitination of both 'Lys-48'- and 'Lys-63'-linked polyubiquitin chains.	Potentiate	161%		
USP43	May recognize and hydrolyze the peptide bond at the C-terminal Gly of ubiquitin. Involved in the processing of poly-ubiquitin precursors as well as that of ubiquitinated proteins	Potentiate	218%		
USP45	Thiol-dependent hydrolysis of ester, thioester, amide, peptide and isopeptide bonds formed by the C-terminal Gly of ubiquitin (a 76-residue protein attached to proteins as an intracellular targeting signal).	Potentiate	80%		
USP38	Deubiquitinating enzyme exhibiting a preference towards 'Lys-63'-linked Ubiquitin chains.	Potentiate	73%		
USP49	Specifically deubiquitinates histone H2B at 'Lys-120' (H2BK120Ub). H2BK120Ub is a specific tag for epigenetic transcriptional activation and acts as a regulator of mRNA splicing. Deubiquitination is required for efficient cotranscriptional splicing of a large set of exons.	Potentiate	47%		
USP14	Proteasome-associated deubiquitinase which releases ubiquitin from the proteasome targeted ubiquitinated proteins. Ensures the regeneration of ubiquitin at the proteasome. Is a reversibly associated subunit of the proteasome and a large fraction of proteasome-free protein exists within the cell. Required for the degradation of the chemokine receptor CXCR4 which is critical for CXCL12-induced cell chemotaxis. Serves also as a physiological inhibitor of endoplasmic reticulum-associated degradation (ERAD) under the non-stressed condition by inhibiting the degradation of unfolded endoplasmic reticulum proteins via interaction with ERN1. Indispensable for synaptic development and function at neuromuscular junctions (NMJs).	no effect	5%		
USP13	Deubiquitinase that mediates deubiquitination of target proteins such as BECN1, MITF, SKP2 and USP10 and is involved in various processes such as autophagy and endoplasmic reticulum-associated degradation (ERAD). Component of a regulatory loop that controls autophagy and p53/TP53 levels: mediates deubiquitination of BECN1, a key regulator of autophagy, leading to stabilize the PIK3C3/VPS34-containing complexes. Also deubiquitinates USP10, an essential regulator of p53/TP53 stability. In turn, PIK3C3/VPS34-containing complexes regulate USP13 stability, suggesting the existence of a regulatory system by which PIK3C3/VPS34-containing complexes regulate p53/TP53 protein levels via USP10 and USP13. Recruited by nuclear UFD1 and mediates deubiquitination of SKP2, thereby regulating endoplasmic reticulum-associated degradation (ERAD). Mediates stabilization of SIAH2 independently of deubiquitinase activity: binds ubiquitinated SIAH2 and acts by impairing SIAH2 autoubiquitination. Has a weak deubiquitinase activity in vitro and preferentially cleaves 'Lys-63'-linked polyubiquitin chains. In contrast to USP5, it is not able to mediate unanchored polyubiquitin disassembly. Able to cleave ISG15 in vitro; however, additional experiments are required to confirm such data	No effect	-6%		

USP36	May be required for maintaining multiple types of adult stem cells. May function as a transcriptional repressor by continually deubiquiting histone H2B at the promoters of genes critical for cellular differentiation, thereby preventing histone H3 'Lys-4' trimethylation (H3K4)	potentiate	41%		
USP7	Hydrolase that deubiquitinates target proteins such as FOXO4, p53/TP53, MDM2, ERCC6, DNMT1, UHRF1, PTEN and DAXX. Together with DAXX, prevents MDM2 self-ubiquitination and enhances the E3 ligase activity of MDM2 towards p53/TP53, thereby promoting p53/TP53 ubiquitination and proteasomal degradation. Deubiquitinates p53/TP53 and MDM2 and strongly stabilizes p53/TP53 even in the presence of excess MDM2, and also induces p53/TP53-dependent cell growth repression and apoptosis. Deubiquitination of FOXO4 in presence of hydrogen peroxide is not dependent on p53/TP53 and inhibits FOXO4-induced transcriptional activity. In association with DAXX, is involved in the deubiquitination and translocation of PTEN from the nucleus to the cytoplasm, both processes that are counteracted by PML. Involved in cell proliferation during early embryonic development. Involved in transcription-coupled nucleotide excision repair (TC-NER) in response to UV damage: recruited to DNA damage sites following interaction with KIAA1530/UVSSA and promotes deubiquitination of ERCC6, preventing UV-induced degradation of ERCC6. Contributes to the overall stabilization and trans-activation capability of the herpesvirus 1 trans-acting transcriptional protein ICP0/VMW110 during HSV-1 infection. Involved in maintenance of DNA methylation via its interaction with UHRF1 and DNMT1: acts by mediating deubiquitination of UHRF1 and DNMT1, preventing their degradation and promoting DNA methylation by DNMT1. Exhibits a preference towards 'Lys-48'-linked Ubiquitin chains.	attenuate	-56%		
USP5	Cleaves linear and branched multiubiquitin polymers with a marked preference for branched polymers. Involved in unanchored 'Lys-48'-linked polyubiquitin disassembly. Binds linear and 'Lys-63'-linked polyubiquitin with a lower affinity. Knock-down of USP5 causes the accumulation o p53/TP53 and an increase in p53/TP53 transcriptional activity because the unanchored polyubiquitin that accumulates is able to compete with ubiquitinated p53/TP53 but not with MDM2 for proteasomal recognition.	attenuate	-41%		
USP35	Thiol-dependent hydrolysis of ester, thioester, amide, peptide and isopeptide bonds formed by the C-terminal Gly of ubiquitin (a 76-residue protein attached to proteins as an intracellular targeting signal).	attenuate	-51%		
**COPI retrograde complex**	**Description**	**Effect**	**% increase (+) or decrease (-)**		
ARF1	GTP-binding protein that functions as an allosteric activator of the cholera toxin catalytic subunit, an ADP-ribosyltransferase. Involved in protein trafficking among different compartments. Modulates vesicle budding and uncoating within the Golgi complex. Deactivation induces the redistribution of the entire Golgi complex to the endoplasmic reticulum, suggesting a crucial role in protein trafficking. In its GTP-bound form, its triggers the association with coat proteins with the Golgi membrane. The hydrolysis of ARF1-bound GTP, which is mediated by ARFGAPs proteins, is required for dissociation of coat proteins from Golgi membranes and vesicles. The GTP-bound form interacts with PICK1 to limit PICK1-mediated inhibition of Arp2/3 complex activity; the function is linked to AMPA receptor (AMPAR) trafficking, regulation of synaptic plasicity of excitatory synapses and spine shrinkage during long-term depression (LTD).	attenuate	-62%		
COPA	The coatomer is a cytosolic protein complex that binds to dilysine motifs and reversibly associates with Golgi non-clathrin-coated vesicles, which further mediate biosynthetic protein transport from the ER, via the Golgi up to the trans Golgi network. Coatomer complex is required for budding from Golgi membranes, and is essential for the retrograde Golgi-to-ER transport of dilysine-tagged proteins. In mammals, the coatomer can only be recruited by membranes associated to ADP-ribosylation factors (ARFs), which are small GTP-binding proteins; the complex also influences the Golgi structural integrity, as well as the processing, activity, and endocytic recycling of LDL receptors	attenuate	-61%		
COPD	The coatomer is a cytosolic protein complex that binds to dilysine motifs and reversibly associates with Golgi non-clathrin-coated vesicles, which further mediate biosynthetic protein transport from the ER, via the Golgi up to the trans Golgi network. Coatomer complex is required for budding from Golgi membranes, and is essential for the retrograde Golgi-to-ER transport of dilysine-tagged proteins. In mammals, the coatomer can only be recruited by membranes associated to ADP-ribosylation factors (ARFs), which are small GTP-binding proteins; the complex also influences the Golgi structural integrity, as well as the processing, activity, and endocytic recycling of LDL receptors	attenuate	-76%		
COPE	The coatomer is a cytosolic protein complex that binds to dilysine motifs and reversibly associates with Golgi non-clathrin-coated vesicles, which further mediate biosynthetic protein transport from the ER, via the Golgi up to the trans Golgi network. Coatomer complex is required for budding from Golgi membranes, and is essential for the retrograde Golgi-to-ER transport of dilysine-tagged proteins. In mammals, the coatomer can only be recruited by membranes associated to ADP-ribosylation factors (ARFs), which are small GTP-binding proteins; the complex also influences the Golgi structural integrity, as well as the processing, activity, and endocytic recycling of LDL receptors	attenuate	-43%		

COPG	The coatomer is a cytosolic protein complex that binds to dilysine motifs and reversibly associates with Golgi non-clathrin-coated vesicles, which further mediate biosynthetic protein transport from the ER, via the Golgi up to the trans Golgi network. Coatomer complex is required for budding from Golgi membranes, and is essential for the retrograde Golgi-to-ER transport of dilysine-tagged proteins. In mammals, the coatomer can only be recruited by membranes associated to ADP-ribosylation factors (ARFs), which are small GTP-binding proteins; the complex also influences the Golgi structural integrity, as well as the processing, activity, and endocytic recycling of LDL receptors	attenuate	-20%		
**ER to Golgi**					
**Retromers and COPII complex**	**Description**	**Effect**	**% increase (+) or decrease (-)**		
VPS26A	Essential component of the retromer complex, a complex required to retrieve lysosomal enzyme receptors (IGF2R and M6PR) from endosomes to the trans-Golgi network. Also required to regulate transcytosis of the polymeric immunoglobulin receptor (pIgR-pIgA). Component of the retromer complex composed of VPS26 (VPS26A or VPS26B), VPS29, VPS35, SNX1 and SNX2. Interacts directly with VPS35. Found in a complex with XPO7, EIF4A1, ARHGAP1, VPS26A, VPS29, VPS35 and SFN. Interacts with ECM29.	Attenuate	-36%		
VPS35	Essential component of the retromer complex, a complex required to retrieve lysosomal enzyme receptors (IGF2R and M6PR) from endosomes to the trans-Golgi network. Also required to regulate transcytosis of the polymeric immunoglobulin receptor (pIgR-pIgA).	Attenuate	-43%		
SEC24B	Component of the COPII coat, that covers ER-derived vesicles involved in transport from the endoplasmic reticulum to the Golgi apparatus. COPII acts in the cytoplasm to promote the transport of secretory, plasma membrane, and vacuolar proteins from the endoplasmic reticulum to the Golgi complex. COPII is composed of at least five proteins: the Sec23/24 complex, the Sec13/31 complex and SAR1. SEC24B is capable of forming heterodimers with SEC24A. Interacts with RNF139. Interacts with TMED2 and TMED10.	No effect	-45%		
SEC16A	Defines endoplasmic reticulum exit sites (ERES) and is required for secretory cargo traffic from the endoplasmic reticulum to the Golgi apparatus. SAR1A-GTP-dependent assembly of SEC16A on the ER membrane forms an organized scaffold defining an ERES. Required for normal transitional endoplasmic reticulum (tER) organization.	attenuate	-16%		
SEC24C	Component of the COPII coat, that covers ER-derived vesicles involved in transport from the endoplasmic reticulum to the Golgi apparatus. COPII acts in the cytoplasm to promote the transport of secretory, plasma membrane, and vacuolar proteins from the endoplasmic reticulum to the Golgi complex.	Attenuate	-65%		
**Golgi and ER proteins**	**Description**	**Effect**	**% increase (+) or decrease (-)**		
POMT1	Transfers mannosyl residues to the hydroxyl group of serine or threonine residues. Coexpression of both POMT1 and POMT2 is necessary for enzyme activity, expression of either POMT1 or POMT2 alone is insufficient.	attenuate	-87%		
ZDHHC17	Palmitoyltransferase specific for a subset of neuronal proteins, including SNAP25, DLG4/PSD95, GAD2, SYT1 and HD. Palmitoylates MPP1 in erythrocytes. May be involved in the sorting or targeting of critical proteins involved in the initiating events of endocytosis at the plasma membrane. Has transforming activity. Mediates Mg2+ transport.	attenuate	-57%		
UGT1A6	UDPGT is of major importance in the conjugation and subsequent elimination of potentially toxic xenobiotics and endogenous compounds. This isoform hasspecificity for phenols. Isoform 3 lacks transferase activity but acts as a negative regulator of isoform 1	potentiate	61%		
**Others**	**Description**	**Effect**	**% increase (+) or decrease (-)**		
UBA1	Activates ubiquitin by first adenylating its C-terminal glycine residue with ATP, and thereafter linking this residue to the side chain of a cysteine residue in E1, yielding a ubiquitin-E1 thioester and free AMP.	attenuate	-78%		
UBQLN2	Increases the half-life of proteins destined to be degraded by the proteasome; may modulate proteasome-mediated protein degradation.	attenuate	-84%		
NEDD8	Ubiquitin-like protein which plays an important role in cell cycle control and embryogenesis. Covalent attachment to its substrates requires prior activation by the E1 complex UBE1C-APPBP1 and linkage to the E2 enzyme UBE2M. Attachment of NEDD8 to cullins activates their associated E3 ubiquitin ligase activity, and thus promotes polyubiquitination and proteasomal degradation of cyclins and other regulatory proteins.	attenuate	-77%		
CAND1	Key assembly factor of SCF (SKP1-CUL1-F-box protein) E3 ubiquitin ligase complexes that promotes the exchange of the substrate-recognition F-box subunit in SCF complexes, thereby playing a key role in the cellular repertoire of SCF complexes.	attenuate	-78%		

SHBG	Functions as an androgen transport protein, but may also be involved in receptor mediated processes. Each dimer binds one molecule of steroid. Specific for 5-alpha-dihydrotestosterone, testosterone, and 17-beta-estradiol. Regulates the plasma metabolic clearance rate of steroid hormones by controlling their plasma concentration.	attenuate	-81%		
N4BP2	Has 5'-polynucleotide kinase and nicking endonuclease activity. May play a role in DNA repair or recombination.	attenuate	-77%		
SQSTM	Required both for the formation and autophagic degradation of polyubiquitin-containing bodies, called ALIS (aggresome-like induced structures). Links ALIS to the autophagic machinery via direct interaction with MAP1 LC3 family members. May regulate the activation of NFKB1 by TNF-alpha, nerve growth factor (NGF) and interleukin-1. May play a role in titin/TTN downstream signaling in muscle cells. May regulate signaling cascades through ubiquitination. Adapter that mediates the interaction between TRAF6 and CYLD By similarity. May be involved in cell differentiation, apoptosis, immune response and regulation of K+ channels	attenuate	-74%		
CDKAL1	Catalyzes the methylthiolation of N6-threonylcarbamoyladenosine (t6A), leading to the formation of 2-methylthio-N6-threonylcarbamoyladenosine (ms2t6A) at position 37 in tRNAs that read codons beginning with adenine	attenuate	-84%		
GANAB	Cleaves sequentially the 2 innermost alpha-1,3-linked glucose residues from the Glc2Man9GlcNAc2 oligosaccharide precursor of immature glycoproteins.	attenuate	-75%		
GBE1	Required for sufficient glycogen accumulation. The alpha 1-6 branches of glycogen play an important role in increasing the solubility of the molecule and, consequently, in reducing the osmotic pressure within cells.	attenuate	-60%		
TLR4	Cooperates with LY96 and CD14 to mediate the innate immune response to bacterial lipopolysaccharide (LPS). Acts via MYD88, TIRAP and TRAF6, leading to NF-kappa-B activation, cytokine secretion and the inflammatory response. Also involved in LPS-independent inflammatory responses triggered by Ni2+. These responses require non-conserved histidines and are, therefore, species-specific.	attenuate	-53%		
GGCT	Catalyzes the formation of 5-oxoproline from gamma-glutamyl dipeptides and may play a significant role in glutathione homeostasis. Induces release of cytochrome c from mitochondria with resultant induction of apoptosis	potentiate	800%		
TSG101	Component of the ESCRT-I complex, a regulator of vesicular trafficking process. Binds to ubiquitinated cargo proteins and is required for the sorting of endocytic ubiquitinated cargos into multivesicular bodies (MVBs). Mediates the association between the ESCRT-0 and ESCRT-I complex. Required for completion of cytokinesis; the function requires CEP55. May be involved in cell growth and differentiation. Acts as a negative growth regulator. Involved in the budding of many viruses through an interaction with viral proteins that contain a late-budding motif P-[ST]-A-P. This interaction is essential for viral particle budding of numerous retroviruses.	no effect	7%		
UBE2N	The UBE2V1-UBE2N and UBE2V2-UBE2N heterodimers catalyze the synthesis of non-canonical 'Lys-63'-linked polyubiquitin chains. This type of polyubiquitination does not lead to protein degradation by the proteasome. Mediates transcriptional activation of target genes. Plays a role in the control of progress through the cell cycle and differentiation. Plays a role in the error-free DNA repair pathway and contributes to the survival of cells after DNA damage. Acts together with the E3 ligases, HLTF and SHPRH, in the 'Lys-63'-linked poly-ubiquitination of PCNA upon genotoxic stress, which is required for DNA repair. Appears to act together with E3 ligase RNF5 in the 'Lys-63'-linked polyubiquitination of JKAMP thereby regulating JKAMP function by decreasing its association with components of the proteasome and ERAD. Promotes TRIM5 capsid-specific restriction activity and the UBE2V1-UBE2N heterodimer acts in concert with TRIM5 to generate 'Lys-63'-linked polyubiquitin chains which activate the MAP3K7/TAK1 complex which in turn results in the induction and expression of NF-kappa-B and MAPK-responsive inflammatory genes	no effect	12%		
NAGA	Removes terminal alpha-N-acetylgalactosamine residues from glycolipids and glycopeptides. Required for the breakdown of glycolipids.	attenuate	-47%		
USO1	Required for protein transport from the ER to the Golgi complex. Homodimer. Dimerizes by parallel association of the tails, resulting in an elongated structure with two globular head domains side by side, and a long rod-like tail structure.	attenuate	-46%		
SEC13	Functions as a component of the nuclear pore complex (NPC) and the COPII coat. At the endoplasmic reticulum, SEC13 is involved in the biogenesis of COPII-coated vesicles. As a component of the GATOR2 complex, inhibits GATOR1 complex, an inhibitor of the amino acid-sensing branch of the TORC1 pathway.	attenuate	-62%		
SEC22A	May be involved in vesicle transport between the ER and the Golgi complex	attenuate	-62%		

Supplemental Table 4. Detailed description of the peptides identified in the proteomic screen

<table>
<tr><th>Number</th><th>File Name</th><th>Charge State (z)</th><th>Score</th><th># of Miss Cleavages</th><th>Total Intensity</th><th>Variable Modification Sites</th><th>Sequence</th><th>Sequence Map</th><th>Modifications</th><th>Retention Time (min)</th><th>m/z</th><th>Parent Mass</th><th>Delta Parent Mass</th><th>Delta Parent Mass (ppm)</th><th>Peptide PI</th><th>Protein Molecular Weight</th><th>Protein PI</th><th>Species</th><th>Accession Number</th><th>Entry Name</th></tr>
<tr><td>1</td><td>dirMS_041712_NAR_50mM_minus2.11374.11874.3</td><td>3</td><td>27.22</td><td>1</td><td>3.47E+06</td><td></td><td>(K)DVDEAY</td><td>(K)D V|D|E/A/Y/M/N/K/V/E/L/E/S R(L)</td><td></td><td>134.43</td><td>599.9481</td><td>1797.832</td><td>-0.0026</td><td>-1.5</td><td>4.18</td><td>13588.1</td><td>4.66</td><td>HUMAN</td><td>F8VP67</td><td>Keratin, type II cytoskeletal 8 (Fragment)</td></tr>
<tr><td>2</td><td>dirMS_041712_NAR_20mM_minus.12370.12509.2</td><td>2</td><td>26.5</td><td>0</td><td>1.76E+06</td><td>M256m</td><td>(R)SLDmDS</td><td>(R)S/L|D|m|D|S/I/I|A/E/V/K(A)</td><td>m:Oxidized methionine</td><td>171.83</td><td>668.8364</td><td>1320.671</td><td>15.994</td><td>-0.7</td><td>4.03</td><td>37296.3</td><td>5.3</td><td>HUMAN</td><td>F8VU64</td><td>Keratin, type II cytoskeletal 8</td></tr>
<tr><td>3</td><td>dirMS_041712_NAR_30mM_plus2.11767.11828.2</td><td>2</td><td>26.5</td><td>0</td><td>5.40E+05</td><td></td><td>(K)VASGCLI</td><td>(K)V/A|S|G|C|L|D/I/N/S S V/K(G)</td><td>C:Carbamidomethylation</td><td>114.82</td><td>675.3393</td><td>1349.673</td><td>-0.0016</td><td>-1.2</td><td>5.81</td><td>56837</td><td>7.19</td><td>HUMAN</td><td>E7ETT1</td><td>Propionyl-CoA carboxylase beta chain, mitochondrial</td></tr>
<tr><td>4</td><td>dirMS_041712_NAR_120mM_minus.9646.9913.3</td><td>3</td><td>26.42</td><td>0</td><td>1.34E+07</td><td></td><td>(K)QEYDES</td><td>(K)Q E/Y/D E/S/G/P/S/I/V/H/R(K)</td><td></td><td>108.05</td><td>506.2424</td><td>1516.703</td><td>0.01</td><td>6.6</td><td>4.65</td><td>123097.1</td><td>5.83</td><td>HUMAN</td><td>A5A3E0</td><td>POTE ankyrin domain family member F</td></tr>
<tr><td>5</td><td>dirMS_041712_NAR_60mM_minus.9926.9945.3</td><td>3</td><td>26.39</td><td>1</td><td>5.20E+05</td><td></td><td>(K)GILAADE</td><td>(K)G I|L|A/A/D/E/S/T/G S I/A K/R(L)</td><td></td><td>115.15</td><td>496.9379</td><td>1488.802</td><td>-0.0025</td><td>-1.7</td><td>6.07</td><td>30711.4</td><td>7.15</td><td>HUMAN</td><td>H3BPS8</td><td>Fructose-bisphosphate aldolase A (Fragment)</td></tr>
<tr><td>6</td><td>dirMS_041712_NAR_50mM_plus2.19239.19494.3</td><td>3</td><td>26.13</td><td>1</td><td>7.33E+05</td><td></td><td>(K)AIDLFTD</td><td>(K)A I|D|L/F/T/D/A/I/K/L/N/P R(L)</td><td></td><td>170.02</td><td>529.6346</td><td>1586.89</td><td>-0.0008</td><td>-0.5</td><td>6</td><td>18141.9</td><td>4.7</td><td>HUMAN</td><td>F6VDH7</td><td>Hsc70-interacting protein (Fragment)</td></tr>
<tr><td>7</td><td>dirMS_041712_NAR_50mM_minus2.17263.17557.3</td><td>3</td><td>25.96</td><td>1</td><td>9.35E+05</td><td></td><td>(K)IILDLISE</td><td>(K)I/I/L/D|L/I/S/E/S/P I/K/G R(A)</td><td></td><td>167.7</td><td>518.6444</td><td>1553.926</td><td>-0.0075</td><td>-4.8</td><td>6.07</td><td>51260.6</td><td>5.39</td><td>HUMAN</td><td>P61978</td><td>Heterogeneous nuclear ribonucleoprotein K</td></tr>
<tr><td>8</td><td>dirMS_041712_NAR_50mM_plus2.20242.20602.3</td><td>3</td><td>25.85</td><td>1</td><td>1.67E+06</td><td></td><td>(R)TLAEIAK</td><td>(R)T/L|A|E/I/A/K/A/E/L/D/G T/I L/K(S)</td><td></td><td>175.92</td><td>562.6601</td><td>1685.968</td><td>-0.0026</td><td>-1.6</td><td>4.68</td><td>58856.7</td><td>6.26</td><td>HUMAN</td><td>Q8WXF1</td><td>Paraspeckle component 1</td></tr>
<tr><td>9</td><td>dirMS_041712_NAR_20mM_minus.12030.12362.2</td><td>2</td><td>25.51</td><td>0</td><td>2.70E+06</td><td>M256m</td><td>(R)SLDmDS</td><td>(R)S L|D|m/D|S/I|I|A|E|V/K(A)</td><td>m:Oxidized methionine</td><td>171.22</td><td>668.8364</td><td>1320.671</td><td>15.994</td><td>-0.7</td><td>4.03</td><td>37296.3</td><td>5.3</td><td>HUMAN</td><td>F8VU64</td><td>Keratin, type II cytoskeletal 8</td></tr>
<tr><td>10</td><td>dirMS_041712_NAR_30mM_plus2.6010.6010.2</td><td>2</td><td>25.48</td><td>0</td><td>1.72E+05</td><td></td><td>(R)GGSGGS</td><td>(R)G G/S G G S\Y|G/G G/G|S/G G G/Y/G/G/G S/G S/R(G)</td><td></td><td>85.83</td><td>896.3659</td><td>1791.728</td><td>-0.0032</td><td>-1.8</td><td>8.59</td><td>39641.8</td><td>4.93</td><td>HUMAN</td><td>K7EQQ3</td><td>Keratin, type I cytoskeletal 9</td></tr>
<tr><td>11</td><td>dirMS_041712_NAR_60mM_minus.13205.13246.3</td><td>3</td><td>25.37</td><td>1</td><td>9.10E+05</td><td></td><td>(K)DVDEAY</td><td>(K)D V|D|E/A/Y/M/N K/V/E/L/E/S R(L)</td><td></td><td>133.75</td><td>599.9463</td><td>1797.832</td><td>-0.008</td><td>-4.5</td><td>4.18</td><td>13588.1</td><td>4.66</td><td>HUMAN</td><td>F8VP67</td><td>Keratin, type II cytoskeletal 8 (Fragment)</td></tr>
<tr><td>12</td><td>dirMS_041712_NAR_20mM_plus.18632.18838.2</td><td>2</td><td>25.32</td><td>0</td><td>2.48E+05</td><td></td><td>(K)IYVDDGI</td><td>(K)I/Y|V|D|D|G|L/I|S/L/Q/V/K(Q)</td><td></td><td>200.73</td><td>731.9068</td><td>1462.815</td><td>-0.0088</td><td>-6</td><td>4.21</td><td>30947.9</td><td>6.47</td><td>HUMAN</td><td>H3BQ34</td><td>Pyruvate kinase</td></tr>
<tr><td>13</td><td>dirMS_041712_NAR_60mM_plus.15198.15373.3</td><td>3</td><td>25.31</td><td>0</td><td>1.86E+06</td><td></td><td>(K)LGDVYV</td><td>(K)L/G|D|V|Y/V/N|D/A/F G T/A H/R(A)</td><td></td><td>136.52</td><td>545.6025</td><td>1634.792</td><td>0.0008</td><td>0.5</td><td>5.21</td><td>41827.5</td><td>7</td><td>HUMAN</td><td>B7Z7A9</td><td>Phosphoglycerate kinase</td></tr>
<tr><td>14</td><td>dirMS_041712_NAR_50mM_plus2.15826.15939.3</td><td>3</td><td>25.17</td><td>1</td><td>8.82E+06</td><td></td><td>(R)ALAEIAK</td><td>(R)A L|A|E|I/A/K/A/E|L/D/D/T|P M R(G)</td><td></td><td>151.43</td><td>581.9697</td><td>1743.895</td><td>0</td><td>0</td><td>4.32</td><td>76262.2</td><td>9.53</td><td>HUMAN</td><td>P23246</td><td>Splicing factor, proline- and glutamine-rich</td></tr>
<tr><td>15</td><td>dirMS_041712_NAR_60mM_plus.7116.7180.3</td><td>3</td><td>25.12</td><td>0</td><td>1.83E+06</td><td></td><td>(K)TIEAEAA</td><td>(K)T|I|E/A/E/A/A/H/G T V T/R(H)</td><td></td><td>95.58</td><td>452.5687</td><td>1355.691</td><td>0.0002</td><td>0.1</td><td>5.37</td><td>45578.2</td><td>7.83</td><td>HUMAN</td><td>B4DFL2</td><td>Isocitrate dehydrogenase [NADP]</td></tr>
<tr><td>16</td><td>dirMS_041712_NAR_20mM_minus.12023.12355.2</td><td>2</td><td>25.07</td><td>0</td><td>2.70E+06</td><td>M256m</td><td>(R)SLDmDS</td><td>(R)S L|D|m/D|S/I|I|A/E|V/K(A)</td><td>m:Oxidized methionine</td><td>171.22</td><td>668.8364</td><td>1320.671</td><td>15.994</td><td>-0.7</td><td>4.03</td><td>37296.3</td><td>5.3</td><td>HUMAN</td><td>F8VU64</td><td>Keratin, type II cytoskeletal 8</td></tr>
<tr><td>17</td><td>dirMS_041712_NAR_60mM_plus.9174.9327.3</td><td>3</td><td>24.99</td><td>1</td><td>1.31E+06</td><td></td><td>(R)FNEVAA</td><td>(R)F/N|E|V/A/A/Q/Y/S E D K/A R(Q)</td><td></td><td>106.1</td><td>543.2616</td><td>1627.771</td><td>-0.0008</td><td>-0.5</td><td>4.68</td><td>14236.2</td><td>10.25</td><td>HUMAN</td><td>F5H1P5</td><td>Peptidyl-prolyl cis-trans isomerase NIMA-interacting 4</td></tr>
<tr><td>18</td><td>dirMS_041712_NAR_30mM_plus2.17905.18433.2</td><td>2</td><td>24.97</td><td>0</td><td>2.52E+07</td><td></td><td>(K)LLCGLLA</td><td>(K)L/L|C|G|L|L|A/E/R(L)</td><td>C:Carbamidomethylation</td><td>149.37</td><td>522.7969</td><td>1044.587</td><td>-0.0005</td><td>-0.5</td><td>6</td><td>12647.2</td><td>8.07</td><td>HUMAN</td><td>P14174</td><td>Macrophage migration inhibitory factor</td></tr>
<tr><td>19</td><td>dirMS_041712_NAR_60mM_plus.7190.7346.2</td><td>2</td><td>24.9</td><td>1</td><td>1.58E+06</td><td></td><td>(R)AAPGAE</td><td>(R)A/A/P/G|A/E/F|A/P N K/R(R)</td><td></td><td>96.2</td><td>614.8248</td><td>1228.643</td><td>-0.001</td><td>-0.8</td><td>8.79</td><td>43979.5</td><td>8.92</td><td>HUMAN</td><td>F5GYZ3</td><td>Non-POU domain-containing octamer-binding protein</td></tr>
<tr><td>20</td><td>dirMS_041712_NAR_30mM_plus2.19610.19610.2</td><td>2</td><td>24.73</td><td>0</td><td>1.56E+06</td><td></td><td>(R)ISEQFTA</td><td>(R)I/S|E/Q/F|T/A/M/F R(R)</td><td></td><td>157.08</td><td>615.3024</td><td>1229.598</td><td>-0.0008</td><td>-0.6</td><td>6</td><td>42831.5</td><td>4.86</td><td>HUMAN</td><td>A8K854</td><td>HCG1983504, isoform CRA_f</td></tr>
<tr><td>21</td><td>dirMS_041712_NAR_40mM_minus2.12093.12464.3</td><td>3</td><td>24.67</td><td>0</td><td>4.82E+05</td><td></td><td>(K)IAILTCPF</td><td>(K)I/A|I|L/T/C/P F/E/P/P K/P K(T)</td><td>C:Carbamidomethylation</td><td>155.75</td><td>537.6382</td><td>1610.898</td><td>0.0026</td><td>1.6</td><td>8.59</td><td>49982</td><td>5.86</td><td>HUMAN</td><td>B4DYD8</td><td>T-complex protein 1 subunit epsilon</td></tr>
<tr><td>22</td><td>dirMS_041712_NAR_20mM_minus.12015.12351.2</td><td>2</td><td>24.66</td><td>0</td><td>2.70E+06</td><td>M256m</td><td>(R)SLDmDS</td><td>(R)S/L|D|m|D|S/I|I|A/E|V/K(A)</td><td>m:Oxidized methionine</td><td>171.22</td><td>668.8364</td><td>1320.671</td><td>15.994</td><td>-0.7</td><td>4.03</td><td>37296.3</td><td>5.3</td><td>HUMAN</td><td>F8VU64</td><td>Keratin, type II cytoskeletal 8</td></tr>
<tr><td>23</td><td>dirMS_041712_NAR_120mM_minus.10520.10874.3</td><td>3</td><td>24.61</td><td>1</td><td>1.14E+06</td><td></td><td>(R)YLAEFAT</td><td>(R)Y/L|A|E/F/A/T/G N D/R K(E)</td><td></td><td>113.15</td><td>462.2338</td><td>1384.686</td><td>0.0013</td><td>0.9</td><td>6.07</td><td>12926.1</td><td>4.4</td><td>HUMAN</td><td>K7EM20</td><td>14-3-3 protein epsilon (Fragment)</td></tr>
<tr><td>24</td><td>dirMS_041712_NAR_50mM_plus2.18704.19214.3</td><td>3</td><td>24.56</td><td>1</td><td>6.37E+06</td><td></td><td>(R)GLFIIDD</td><td>(R)G L|F|I/I/D/D/K/G I L/R(Q)</td><td></td><td>167.33</td><td>453.9379</td><td>1359.8</td><td>-0.0003</td><td>-0.2</td><td>5.96</td><td>18498.6</td><td>5.82</td><td>HUMAN</td><td>H7C3T4</td><td>Peroxiredoxin-4 (Fragment)</td></tr>
<tr><td>25</td><td>dirMS_041712_NAR_50mM_plus2.14519.14660.3</td><td>3</td><td>24.54</td><td>0</td><td>6.08E+05</td><td></td><td>(R)DLSYCLS</td><td>(R)D L|S|Y/C/L/S/G/M/Y D/H/R(Y)</td><td>C:Carbamidomethylation</td><td>144.83</td><td>539.5651</td><td>1616.683</td><td>-0.0024</td><td>-1.5</td><td>5.21</td><td>46013.4</td><td>5.38</td><td>HUMAN</td><td>P52597</td><td>Heterogeneous nuclear ribonucleoprotein F</td></tr>
<tr><td>26</td><td>dirMS_041712_NAR_40mM_plus2.15542.15873.3</td><td>3</td><td>24.51</td><td>1</td><td>1.20E+06</td><td></td><td>(K)VLDSGA</td><td>(K)V/L|D|S/G/A|P I K I|P/V/G/P E/T L G R(I)</td><td></td><td>159.17</td><td>640.3668</td><td>1919.096</td><td>-0.0102</td><td>-5.3</td><td>6.04</td><td>55307.5</td><td>5.25</td><td>HUMAN</td><td>F8VPV9</td><td>ATP synthase subunit beta</td></tr>
<tr><td>27</td><td>dirMS_041712_NAR_60mM_minus.8692.8956.3</td><td>3</td><td>24.48</td><td>0</td><td>3.43E+07</td><td></td><td>(K)QEYDES</td><td>(K)Q E/Y/D E/S/G/P/S/I/V/H/R(K)</td><td></td><td>110.32</td><td>506.2391</td><td>1516.703</td><td>0.0001</td><td>0.1</td><td>4.65</td><td>123097.1</td><td>5.83</td><td>HUMAN</td><td>A5A3E0</td><td>POTE ankyrin domain family member F</td></tr>
<tr><td>28</td><td>dirMS_041712_NAR_50mM_plus2.18191.18666.3</td><td>3</td><td>24.39</td><td>1</td><td>6.46E+06</td><td></td><td>(K)IILDLISE</td><td>(K)I/I|L/D|L/I/S/E/S/P I K G R(A)</td><td></td><td>165.22</td><td>518.6453</td><td>1553.926</td><td>-0.0048</td><td>-3.1</td><td>6.07</td><td>51260.6</td><td>5.39</td><td>HUMAN</td><td>P61978</td><td>Heterogeneous nuclear ribonucleoprotein K</td></tr>
<tr><td>29</td><td>dirMS_041712_NAR_20mM_plus.14147.14498.2</td><td>2</td><td>24.35</td><td>0</td><td>2.99E+06</td><td>M256m</td><td>(R)SLDmDS</td><td>(R)S/L|D|m|D|S/I/I|A/E/V/K(A)</td><td>m:Oxidized methionine</td><td>170.42</td><td>668.8371</td><td>1320.671</td><td>15.9954</td><td>0.3</td><td>4.03</td><td>37296.3</td><td>5.3</td><td>HUMAN</td><td>F8VU64</td><td>Keratin, type II cytoskeletal 8</td></tr>
<tr><td>30</td><td>dirMS_041712_NAR_50mM_plus2.18028.18329.3</td><td>3</td><td>24.35</td><td>1</td><td>1.46E+06</td><td></td><td>(K)VVLDDK</td><td>(K)V/V|L/D/D/K/D/Y/F/L/F/R(D)</td><td></td><td>164.12</td><td>510.6044</td><td>1529.8</td><td>-0.0012</td><td>-0.8</td><td>4.43</td><td>5189.9</td><td>4.56</td><td>HUMAN</td><td>B8ZZ54</td><td>10 kDa heat shock protein, mitochondrial</td></tr>
<tr><td>31</td><td>dirMS_041712_NAR_60mM_minus.8312.8435.3</td><td>3</td><td>24.3</td><td>0</td><td>8.83E+05</td><td></td><td>(K)VSYGIGD</td><td>(K)V/S|Y/G|I/G/D E E/H/D/Q/E/G R(V)</td><td></td><td>106.73</td><td>564.2471</td><td>1690.73</td><td>-0.0036</td><td>-2.1</td><td>4.17</td><td>19395.5</td><td>5.38</td><td>HUMAN</td><td>G3V359</td><td>DNA-(apurinic or apyrimidinic site) lyase (Fragment)</td></tr>
<tr><td>32</td><td>dirMS_041712_NAR_60mM_plus.17341.17554.3</td><td>3</td><td>24.3</td><td>1</td><td>1.26E+06</td><td></td><td>(R)FLQDYFI</td><td>(R)F/L|Q/D/Y|F/D/G N L K/R(Y)</td><td></td><td>148.32</td><td>505.9247</td><td>1515.759</td><td>0.0005</td><td>0.3</td><td>5.96</td><td>55361.8</td><td>6.42</td><td>HUMAN</td><td>G5EA52</td><td>Protein disulfide isomerase family A, member 3, isoform CRA_b</td></tr>
<tr><td>33</td><td>dirMS_041712_NAR_60mM_minus.13943.14052.3</td><td>3</td><td>24.26</td><td>0</td><td>3.54E+05</td><td></td><td>(K)DCGATV</td><td>(K)D\C|G|A/T/W/V/V/L/G H S E R(R)</td><td>C:Carbamidomethylation</td><td>138.02</td><td>529.5838</td><td>1586.738</td><td>-0.0012</td><td>-0.7</td><td>5.32</td><td>31075.7</td><td>5.65</td><td>HUMAN</td><td>P60174</td><td>Triosephosphate isomerase</td></tr>
<tr><td>34</td><td>dirMS_041712_NAR_60mM_plus.16586.16586.2</td><td>2</td><td>24.25</td><td>0</td><td>7.89E+05</td><td></td><td>(R)EHALLAY</td><td>(R)E/H|A|L|L|A/Y/T L G V K(Q)</td><td></td><td>143.77</td><td>657.8735</td><td>1314.742</td><td>-0.0019</td><td>-1.4</td><td>6.85</td><td>50482.3</td><td>9.31</td><td>HUMAN</td><td>P68104</td><td>Elongation factor 1-alpha 1</td></tr>
<tr><td>35</td><td>dirMS_041712_NAR_30mM_minus2.7759.8008.2</td><td>2</td><td>24.16</td><td>0</td><td>1.38E+08</td><td></td><td>(K)AGFAGD</td><td>(K)A/G|F|A|G/D/D/A/P/R(A)</td><td></td><td>98.17</td><td>488.7304</td><td>976.448</td><td>0.0052</td><td>5.4</td><td>4.21</td><td>123097.1</td><td>5.83</td><td>HUMAN</td><td>A5A3E0</td><td>POTE ankyrin domain family member F</td></tr>
<tr><td>36</td><td>dirMS_041712_NAR_30mM_minus2.10293.10293.2</td><td>2</td><td>24.11</td><td>0</td><td>2.81E+05</td><td></td><td>(R)ETAEAD</td><td>(R)E T|A|E/A/D/V|A/S/L/N R(R)</td><td></td><td>110.08</td><td>638.3134</td><td>1275.618</td><td>0.002</td><td>1.6</td><td>4.14</td><td>31923.6</td><td>4.85</td><td>HUMAN</td><td>B7Z596</td><td>Tropomyosin alpha-1 chain</td></tr>
<tr><td>37</td><td>dirMS_041712_NAR_50mM_plus2.18566.18715.3</td><td>3</td><td>24.08</td><td>1</td><td>5.28E+05</td><td></td><td>(K)TGISDVF</td><td>(K)T/G/I|S/D/V/F/A K N D|L/A/V|V/D/V R(I)</td><td></td><td>166.27</td><td>640.3449</td><td>1919.023</td><td>-0.0031</td><td>-1.6</td><td>4.43</td><td>76670.1</td><td>4.6</td><td>HUMAN</td><td>P19338</td><td>Nucleolin</td></tr>
<tr><td>38</td><td>dirMS_041712_NAR_50mM_plus2.20429.20706.3</td><td>3</td><td>24.05</td><td>1</td><td>1.51E+06</td><td></td><td>(K)SYLNMD</td><td>(K)S/Y|L/N/M/D/A/I/M/E/A I/K K(T)</td><td></td><td>177.6</td><td>542.9473</td><td>1626.823</td><td>0.0044</td><td>2.7</td><td>5.79</td><td>80685.1</td><td>7.31</td><td>HUMAN</td><td>P05165</td><td>Propionyl-CoA carboxylase alpha chain, mitochondrial</td></tr>
<tr><td>39</td><td>dirMS_041712_NAR_30mM_plus2.22541.22620.2</td><td>2</td><td>24.03</td><td>0</td><td>2.50E+06</td><td></td><td>(R)LEGLTDE</td><td>(R)L/E|G|L|T|D|E|I/N/F/L/R(Q)</td><td></td><td>175.42</td><td>710.3766</td><td>1419.748</td><td>-0.0019</td><td>-1.3</td><td>4.14</td><td>13588.1</td><td>4.66</td><td>HUMAN</td><td>F8VP67</td><td>Keratin, type II cytoskeletal 8 (Fragment)</td></tr>
<tr><td>40</td><td>dirMS_041712_NAR_30mM_minus2.16515.16515.2</td><td>2</td><td>23.96</td><td>0</td><td>4.38E+05</td><td>M185m</td><td>(K)LAVNm</td><td>(K)L/A/V/N/m|V/P F/P R(L)</td><td>m:Oxidized methionine</td><td>142.67</td><td>580.3178</td><td>1143.634</td><td>15.994</td><td>-0.8</td><td>9.75</td><td>42230.8</td><td>4.77</td><td>HUMAN</td><td>A6NKZ8</td><td>Putative tubulin beta chain-like protein ENSP00000290377</td></tr>
<tr><td>41</td><td>dirMS_041712_NAR_50mM_plus2.18057.18199.3</td><td>3</td><td>23.88</td><td>0</td><td>1.47E+06</td><td></td><td>(K)ALSAIAD</td><td>(K)A L|S|A/I/A/D/L/L/T/N/E H E R(V)</td><td></td><td>163.75</td><td>551.6254</td><td>1652.86</td><td>0.0014</td><td>0.9</td><td>4.65</td><td>105417.2</td><td>6.41</td><td>HUMAN</td><td>C9JZR2</td><td>Catenin delta-1</td></tr>
<tr><td>42</td><td>dirMS_041712_NAR_60mM_minus.15869.16146.3</td><td>3</td><td>23.86</td><td>1</td><td>5.23E+05</td><td></td><td>(R)IFDSEEIL</td><td>(R)I/F|D/S/E E/I/L/A/G/Y K R(E)</td><td></td><td>148.88</td><td>514.2713</td><td>1540.801</td><td>-0.0012</td><td>-0.8</td><td>4.68</td><td>35548.6</td><td>4.91</td><td>HUMAN</td><td>B4DN60</td><td>Asparagine--tRNA ligase, cytoplasmic</td></tr>
<tr><td>43</td><td>dirMS_041712_NAR_20mM_plus.17653.17653.2</td><td>2</td><td>23.84</td><td>0</td><td>9.58E+04</td><td>M148m</td><td>(K)TFAPEEI</td><td>(K)T F/A/P E E I/S/A m|V L T K(M)</td><td>m:Oxidized methionine</td><td>193.53</td><td>776.8996</td><td>1536.798</td><td>15.9941</td><td>-0.5</td><td>4.53</td><td>72445.7</td><td>5.07</td><td>HUMAN</td><td>P11021</td><td>78 kDa glucose-regulated protein</td></tr>
<tr><td>44</td><td>dirMS_041712_NAR_30mM_minus2.10792.11262.2</td><td>2</td><td>23.79</td><td>0</td><td>6.68E+06</td><td></td><td>(R)ISVYYNE</td><td>(R)I/S|V|Y|Y/N/E|A/T/G G K(Y)</td><td></td><td>113.98</td><td>651.322</td><td>1301.637</td><td>-0.0005</td><td>-0.4</td><td>6</td><td>27549.9</td><td>4.39</td><td>HUMAN</td><td>E9PBJ4</td><td>Tubulin beta chain</td></tr>
<tr><td>45</td><td>dirMS_041712_NAR_30mM_plus2.20410.20809.2</td><td>2</td><td>23.78</td><td>0</td><td>4.74E+05</td><td></td><td>(R)SLVPAAI</td><td>(R)S L|V|P/A/A/E/L|L/E/S/R(V)</td><td></td><td>162.17</td><td>642.8602</td><td>1284.716</td><td>-0.0027</td><td>-2.1</td><td>4.53</td><td>533778</td><td>5.74</td><td>HUMAN</td><td>Q15149</td><td>Plectin</td></tr>
<tr><td>46</td><td>dirMS_041712_NAR_40mM_minus2.11731.11772.3</td><td>3</td><td>23.77</td><td>1</td><td>2.86E+06</td><td></td><td>(R)IINEPTA</td><td>(R)I/I|N|E|P T/A/A/A/I/A/Y G L D/K K(V)</td><td></td><td>153.08</td><td>596.6671</td><td>1787.99</td><td>-0.0034</td><td>-1.9</td><td>6.07</td><td>17973</td><td>5.16</td><td>HUMAN</td><td>E9PI65</td><td>Heat shock cognate 71 kDa protein (Fragment)</td></tr>
<tr><td>47</td><td>dirMS_041712_NAR_50mM_minus2.13783.13937.3</td><td>3</td><td>23.77</td><td>1</td><td>3.91E+05</td><td></td><td>(K)ITDIIGKE</td><td>(K)I/T|D|I|I|G K/E E/G\I|G/P E N L\R(G)</td><td></td><td>146.9</td><td>618.665</td><td>1853.997</td><td>-0.0163</td><td>-8.8</td><td>4.41</td><td>267260.7</td><td>5.95</td><td>HUMAN</td><td>Q13085</td><td>Acetyl-CoA carboxylase 1</td></tr>
<tr><td>48</td><td>dirMS_041712_NAR_120mM_plus.6082.6341.3</td><td>3</td><td>23.69</td><td>1</td><td>1.97E+06</td><td></td><td>(K)DSYVGD</td><td>(K)D S|Y|V/G/D/E/A/Q/S K R(G)</td><td></td><td>86.32</td><td>452.2133</td><td>1354.623</td><td>0.002</td><td>1.5</td><td>4.56</td><td>32616.5</td><td>5.18</td><td>HUMAN</td><td>A6NL76</td><td>Actin, alpha skeletal muscle</td></tr>
<tr><td>49</td><td>dirMS_041712_NAR_30mM_plus2.6085.6086.2</td><td>2</td><td>23.67</td><td>0</td><td>8.14E+05</td><td></td><td>(R)NQGGY</td><td>(R)N/Q|G/G|Y/G G/S/S/S/S/S/S/Y/G/S G R(R)</td><td></td><td>86.18</td><td>847.8524</td><td>1694.7</td><td>-0.0026</td><td>-1.5</td><td>8.59</td><td>33268.7</td><td>9.31</td><td>HUMAN</td><td>F8VRQ1</td><td>Heterogeneous nuclear ribonucleoprotein A1</td></tr>
<tr><td>50</td><td>dirMS_041712_NAR_50mM_minus2.12370.12391.3</td><td>3</td><td>23.66</td><td>0</td><td>1.08E+06</td><td></td><td>(K)LGDVYV</td><td>(K)L/G|D|V|Y|V/N/D/A/F G T A H R(A)</td><td></td><td>138.38</td><td>545.6023</td><td>1634.792</td><td>0.0002</td><td>0.1</td><td>5.21</td><td>41827.5</td><td>7</td><td>HUMAN</td><td>B7Z7A9</td><td>Phosphoglycerate kinase</td></tr>
<tr><td>51</td><td>dirMS_041712_NAR_30mM_plus2.15014.15522.2</td><td>2</td><td>23.66</td><td>0</td><td>1.03E+07</td><td></td><td>(K)LSELEAA</td><td>(K)L/S|E|L|E\A/A/L/Q/R(A)</td><td></td><td>132.32</td><td>565.3227</td><td>1129.621</td><td>0.017</td><td>15</td><td>4.53</td><td>56607.4</td><td>5.37</td><td>HUMAN</td><td>F8VXB4</td><td>Keratin, type II cytoskeletal 8</td></tr>
<tr><td>52</td><td>dirMS_041712_NAR_20mM_plus.17576.17576.2</td><td>2</td><td>23.65</td><td>0</td><td>1.61E+05</td><td>M74m</td><td>(R)mIAGQV</td><td>(R)m I|A/G Q/V|L/D/I/N/L A\A E/P K(V)</td><td>m:Oxidized methionine</td><td>192.85</td><td>849.9568</td><td>1682.915</td><td>15.9918</td><td>-1.9</td><td>4.37</td><td>15791.7</td><td>9.22</td><td>HUMAN</td><td>B4DSU6</td><td>Heterogeneous nuclear ribonucleoproteins C1/C2</td></tr>
<tr><td>53</td><td>dirMS_041712_NAR_30mM_minus2.9185.9289.2</td><td>2</td><td>23.59</td><td>0</td><td>2.85E+06</td><td></td><td>(R)VIGSGCI</td><td>(R)V/I|G|S/G|C/N/L/D/S A/R(F)</td><td>C:Carbamidomethylation</td><td>104.52</td><td>624.8035</td><td>1248.6</td><td>-0.0004</td><td>-0.3</td><td>5.81</td><td>25445.8</td><td>5.52</td><td>HUMAN</td><td>A8MW50</td><td>L-lactate dehydrogenase (Fragment)</td></tr>
<tr><td>54</td><td>dirMS_041712_NAR_50mM_plus2.15833.15884.3</td><td>3</td><td>23.59</td><td>0</td><td>1.21E+06</td><td></td><td>(K)IAILTCPF</td><td>(K)I/A|I|L/T/C/P F/E/P P K/P K(T)</td><td>C:Carbamidomethylation</td><td>151.62</td><td>537.6393</td><td>1610.898</td><td>0.0059</td><td>3.7</td><td>8.59</td><td>49982</td><td>5.86</td><td>HUMAN</td><td>B4DYD8</td><td>T-complex protein 1 subunit epsilon</td></tr>
<tr><td>55</td><td>dirMS_041712_NAR_60mM_plus.6358.6380.2</td><td>2</td><td>23.59</td><td>1</td><td>1.56E+06</td><td></td><td>(K)DSYVGD</td><td>(K)D/S|Y|V|G/D/E/A/Q/S K/R(G)</td><td></td><td>91.98</td><td>677.8149</td><td>1354.623</td><td>-0.0008</td><td>-0.6</td><td>4.56</td><td>32616.5</td><td>5.18</td><td>HUMAN</td><td>A6NL76</td><td>Actin, alpha skeletal muscle</td></tr>
<tr><td>56</td><td>dirMS_041712_NAR_120mM_plus.17875.18084.3</td><td>3</td><td>23.58</td><td>0</td><td>7.30E+05</td><td></td><td>(R)HLLQAP</td><td>(R)H/L|L/Q|A|P V D D|A\Q/E|I/L/H S R(F)</td><td></td><td>146.82</td><td>648.0088</td><td>1942.014</td><td>-0.0023</td><td>-1.2</td><td>5.21</td><td>63613.4</td><td>7.9</td><td>HUMAN</td><td>B3KXI2</td><td>Protein transport protein Sec23A</td></tr>
<tr><td>57</td><td>dirMS_041712_NAR_120mM_plus.22179.22245.4</td><td>4</td><td>23.56</td><td>1</td><td>5.80E+05</td><td></td><td>(R)SLHDAL</td><td>(R)S L H\D A\L|C|V|L|A/Q/T/V/K/D/S R(T)</td><td>C:Carbamidomethylation</td><td>170.13</td><td>479.0094</td><td>1912.991</td><td>0.0248</td><td>13</td><td>6.47</td><td>57204.9</td><td>6.01</td><td>HUMAN</td><td>F5GWF6</td><td>T-complex protein 1 subunit beta</td></tr>
<tr><td>58</td><td>dirMS_041712_NAR_60mM_plus.11231.11231.2</td><td>2</td><td>23.51</td><td>0</td><td>2.12E+06</td><td></td><td>(K)GIVEFAS</td><td>(K)G I|V|E|F|A/S K/P A A/R(K)</td><td></td><td>116.18</td><td>623.3504</td><td>1245.695</td><td>-0.0015</td><td>-1.2</td><td>8.75</td><td>76262.2</td><td>9.53</td><td>HUMAN</td><td>P23246</td><td>Splicing factor, proline- and glutamine-rich</td></tr>
<tr><td>59</td><td>dirMS_041712_NAR_50mM_plus2.18646.18685.3</td><td>3</td><td>23.46</td><td>1</td><td>5.28E+05</td><td></td><td>(K)TGISDVF</td><td>(K)T/G|I|S/D/V|F/A K/N D|L/A|V/V D/V R(I)</td><td></td><td>166.27</td><td>640.3449</td><td>1919.023</td><td>-0.0031</td><td>-1.6</td><td>4.43</td><td>76670.1</td><td>4.6</td><td>HUMAN</td><td>P19338</td><td>Nucleolin</td></tr>
<tr><td>60</td><td>dirMS_041712_NAR_30mM_plus2.22038.22454.2</td><td>2</td><td>23.43</td><td>0</td><td>5.20E+06</td><td></td><td>(R)SLDMDS</td><td>(R)S L|D|M/D|S|I|I/A/E/V/K(A)</td><td></td><td>172.65</td><td>660.8437</td><td>1320.671</td><td>0.0086</td><td>6.5</td><td>4.03</td><td>37296.3</td><td>5.3</td><td>HUMAN</td><td>F8VU64</td><td>Keratin, type II cytoskeletal 8</td></tr>
<tr><td>61</td><td>dirMS_041712_NAR_60mM_plus.8483.8715.3</td><td>3</td><td>23.41</td><td>0</td><td>1.39E+06</td><td></td><td>(K)VDNDEN</td><td>(K)V/D|N/D/E/N/E/H/Q/L/S L/R(T)</td><td></td><td>102.82</td><td>523.5814</td><td>1568.73</td><td>-0.0003</td><td>-0.2</td><td>4.31</td><td>6862.5</td><td>4.32</td><td>HUMAN</td><td>E5RGW4</td><td>Nucleophosmin (Fragment)</td></tr>
<tr><td>62</td><td>dirMS_041712_NAR_50mM_minus2.9685.10118.3</td><td>3</td><td>23.34</td><td>0</td><td>4.86E+05</td><td></td><td>(R)EALLSSA</td><td>(R)E A|L|L/S/S/A/V|D|H G S D E/V/K(F)</td><td></td><td>124.27</td><td>552.9403</td><td>1656.807</td><td>-0.0012</td><td>-0.7</td><td>4.31</td><td>57204.9</td><td>6.01</td><td>HUMAN</td><td>F5GWF6</td><td>T-complex protein 1 subunit beta</td></tr>
<tr><td>63</td><td>dirMS_041712_NAR_50mM_minus2.14827.15085.3</td><td>3</td><td>23.27</td><td>0</td><td>2.89E+05</td><td></td><td>(K)IAILTCPF</td><td>(K)I/A|I/L/T/C|P F/E/P P K/P K(T)</td><td>C:Carbamidomethylation</td><td>153.68</td><td>537.6375</td><td>1610.898</td><td>0.0005</td><td>0.3</td><td>8.59</td><td>49982</td><td>5.86</td><td>HUMAN</td><td>B4DYD8</td><td>T-complex protein 1 subunit epsilon</td></tr>
<tr><td>64</td><td>dirMS_041712_NAR_50mM_plus2.14468.14468.3</td><td>3</td><td>23.27</td><td>0</td><td>1.06E+06</td><td></td><td>(R)ETHSAL</td><td>(R)E T H S A/L|G|P/A|L/Q|A/A F/K(L)</td><td></td><td>144.3</td><td>514.2745</td><td>1540.812</td><td>-0.0029</td><td>-1.9</td><td>6.85</td><td>141616.6</td><td>6.1</td><td>HUMAN</td><td>B7ZKM8</td><td>Protein transport protein Sec24B</td></tr>
<tr><td>65</td><td>dirMS_041712_NAR_60mM_minus.5545.5545.3</td><td>3</td><td>23.26</td><td>1</td><td>3.66E+07</td><td></td><td>(K)DSYVGD</td><td>(K)D S|Y|V/G D/E/A/Q/S K/R(G)</td><td></td><td>87.63</td><td>452.2129</td><td>1354.623</td><td>0.0008</td><td>0.6</td><td>4.56</td><td>32616.5</td><td>5.18</td><td>HUMAN</td><td>A6NL76</td><td>Actin, alpha skeletal muscle</td></tr>
<tr><td>66</td><td>dirMS_041712_NAR_30mM_plus2.18721.18879.2</td><td>2</td><td>23.23</td><td>0</td><td>6.67E+05</td><td></td><td>(R)SYNDEL</td><td>(R)S/Y|N|D|E/L|Q/F/L/E/K(I)</td><td></td><td>152.13</td><td>693.335</td><td>1385.658</td><td>0.0044</td><td>3.2</td><td>4.14</td><td>19338.9</td><td>5.33</td><td>HUMAN</td><td>E7EQS9</td><td>tRNA-splicing ligase RtcB homolog</td></tr>
<tr><td>67</td><td>dirMS_041712_NAR_30mM_minus2.17505.18006.2</td><td>2</td><td>23.19</td><td>0</td><td>1.10E+07</td><td></td><td>(K)LLCGLLA</td><td>(K)L/L|C|G|L|L/A E/R(L)</td><td>C:Carbamidomethylation</td><td>151.02</td><td>522.7963</td><td>1044.587</td><td>-0.0017</td><td>-1.6</td><td>6</td><td>12647.2</td><td>8.07</td><td>HUMAN</td><td>P14174</td><td>Macrophage migration inhibitory factor</td></tr>
<tr><td>68</td><td>dirMS_041712_NAR_50mM_plus2.17516.17716.3</td><td>3</td><td>23.16</td><td>1</td><td>3.01E+06</td><td></td><td>(K)TVTAMD</td><td>(K)T V|T|A/M/D/V/V/Y A L K/R(Q)</td><td></td><td>161</td><td>489.6059</td><td>1466.804</td><td>-0.0004</td><td>-0.3</td><td>8.25</td><td>11367.1</td><td>11.36</td><td>HUMAN</td><td>P62805</td><td>Histone H4</td></tr>
<tr><td>69</td><td>dirMS_041712_NAR_30mM_minus2.14938.15247.2</td><td>2</td><td>23.15</td><td>0</td><td>1.44E+07</td><td></td><td>(K)LSELEAA</td><td>(K)L/S|E|L|E\A/A|L/Q/R(A)</td><td></td><td>135.17</td><td>565.3142</td><td>1129.621</td><td>0</td><td>0</td><td>4.53</td><td>56607.4</td><td>5.37</td><td>HUMAN</td><td>F8VXB4</td><td>Keratin, type II cytoskeletal 8</td></tr>
<tr><td>70</td><td>dirMS_041712_NAR_60mM_minus.16949.17072.3</td><td>3</td><td>23.15</td><td>0</td><td>1.02E+06</td><td></td><td>(K)ICDQWD</td><td>(K)I/C|D/Q/W D/A|L/G/S/L/T/H S R(R)</td><td>C:Carbamidomethylation</td><td>154.87</td><td>586.9446</td><td>1758.823</td><td>-0.0035</td><td>-2</td><td>5.21</td><td>80254.2</td><td>5.05</td><td>HUMAN</td><td>D6PXK4</td><td>Alpha actinin 4 short isoform</td></tr>
<tr><td>71</td><td>dirMS_041712_NAR_50mM_plus2.12167.12387.3</td><td>3</td><td>23.11</td><td>1</td><td>1.69E+06</td><td></td><td>(K)ILIANTG</td><td>(K)I/L|I/A/N/T/G M/D/T D/K I K(I)</td><td></td><td>132.52</td><td>511.6172</td><td>1532.835</td><td>0.0018</td><td>1.2</td><td>5.96</td><td>57204.9</td><td>6.01</td><td>HUMAN</td><td>F5GWF6</td><td>T-complex protein 1 subunit beta</td></tr>
<tr><td>72</td><td>dirMS_041712_NAR_40mM_plus2.14497.14828.3</td><td>3</td><td>23.09</td><td>0</td><td>5.43E+05</td><td></td><td>(K)IAILTCPF</td><td>(K)I/A|I/L/T/C/P F/E/P P K/P K(T)</td><td>C:Carbamidomethylation</td><td>153.55</td><td>537.6381</td><td>1610.898</td><td>0.0023</td><td>1.4</td><td>8.59</td><td>49982</td><td>5.86</td><td>HUMAN</td><td>B4DYD8</td><td>T-complex protein 1 subunit epsilon</td></tr>
<tr><td>73</td><td>dirMS_041712_NAR_60mM_minus.16799.17030.3</td><td>3</td><td>23.07</td><td>0</td><td>1.02E+06</td><td></td><td>(K)ICDQWD</td><td>(K)I/C|D/Q/W/D A|L/G/S L/T/H S R(R)</td><td>C:Carbamidomethylation</td><td>154.87</td><td>586.9446</td><td>1758.823</td><td>-0.0035</td><td>-2</td><td>5.21</td><td>80254.2</td><td>5.05</td><td>HUMAN</td><td>D6PXK4</td><td>Alpha actinin 4 short isoform</td></tr>
<tr><td>74</td><td>dirMS_041712_NAR_30mM_plus2.21279.21279.2</td><td>2</td><td>23.07</td><td>0</td><td>2.95E+05</td><td></td><td>(R)GVVQEL</td><td>(R)G V|V|Q/E|L|Q Q/A I/S/K(L)</td><td></td><td>165.93</td><td>650.3632</td><td>1299.727</td><td>-0.0076</td><td>-5.8</td><td>6</td><td>22489.6</td><td>5.38</td><td>HUMAN</td><td>E9PI39</td><td>Elongation factor 1-delta (Fragment)</td></tr>
<tr><td>75</td><td>dirMS_041712_NAR_30mM_plus2.20024.20419.2</td><td>2</td><td>23.06</td><td>0</td><td>7.28E+05</td><td></td><td>(K)SYELPDG</td><td>(K)S Y|E|L|P/D/G Q/V|I|T/I|G N E R(F)</td><td></td><td>159.77</td><td>895.9487</td><td>1790.892</td><td>-0.0018</td><td>-1</td><td>4.14</td><td>123097.1</td><td>5.83</td><td>HUMAN</td><td>A5A3E0</td><td>POTE ankyrin domain family member F</td></tr>
<tr><td>76</td><td>dirMS_041712_NAR_40mM_plus2.18063.18189.3</td><td>3</td><td>23.06</td><td>1</td><td>1.58E+06</td><td></td><td>(K)TFVNITP</td><td>(K)T F|V|N|I T/P A E V/G V/L|V/G K D/R(S)</td><td></td><td>173.02</td><td>639.0251</td><td>1915.065</td><td>-0.004</td><td>-2.1</td><td>5.74</td><td>17630.6</td><td>11.62</td><td>HUMAN</td><td>I3L3D5</td><td>Profilin-1 (Fragment)</td></tr>
<tr><td>77</td><td>dirMS_041712_NAR_120mM_plus.9769.9814.3</td><td>3</td><td>23.05</td><td>0</td><td>7.61E+05</td><td></td><td>(K)TVAIHSD</td><td>(K)T/V|A/I/H S D|V|D/A/S S V/H V K(M)</td><td></td><td>104.53</td><td>555.6244</td><td>1664.86</td><td>-0.0016</td><td>-0.9</td><td>5.91</td><td>80685.1</td><td>7.31</td><td>HUMAN</td><td>P05165</td><td>Propionyl-CoA carboxylase alpha chain, mitochondrial</td></tr>
<tr><td>78</td><td>dirMS_041712_NAR_30mM_minus2.16152.16559.2</td><td>2</td><td>23.03</td><td>0</td><td>4.42E+05</td><td>M185m</td><td>(K)LAVNm</td><td>(K)L/A|V|N/m|V|P/F/P/R(L)</td><td>m:Oxidized methionine</td><td>142.58</td><td>580.3178</td><td>1143.634</td><td>15.994</td><td>-0.8</td><td>9.75</td><td>42230.8</td><td>4.77</td><td>HUMAN</td><td>A6NKZ8</td><td>Putative tubulin beta chain-like protein ENSP00000290377</td></tr>
<tr><td>79</td><td>dirMS_041712_NAR_60mM_plus.14548.14888.3</td><td>3</td><td>23.03</td><td>0</td><td>1.55E+06</td><td></td><td>(K)YYVTIID</td><td>(K)Y/Y|V/T/I/I/D/A/P/G H/R(D)</td><td></td><td>133.28</td><td>468.9136</td><td>1404.727</td><td>-0.0008</td><td>-0.6</td><td>6.74</td><td>12137.8</td><td>9.3</td><td>HUMAN</td><td>A6PW80</td><td>Elongation factor 1-alpha 1 (Fragment)</td></tr>
<tr><td>80</td><td>dirMS_041712_NAR_30mM_minus2.11859.12019.2</td><td>2</td><td>23.02</td><td>0</td><td>2.09E+06</td><td></td><td>(K)GISQEQI</td><td>(K)G I|S|Q|E|Q|M/Q/E/F/R(A)</td><td></td><td>118.58</td><td>676.8161</td><td>1352.626</td><td>-0.0014</td><td>-1</td><td>4.53</td><td>80254.2</td><td>5.05</td><td>HUMAN</td><td>D6PXK4</td><td>Alpha actinin 4 short isoform</td></tr>
<tr><td>81</td><td>dirMS_041712_NAR_50mM_plus2.10324.10326.3</td><td>3</td><td>23.02</td><td>0</td><td>3.78E+05</td><td></td><td>(R)MVQVH</td><td>(R)M/V|Q|V/H/E/L/S/C E/G/I S K(S)</td><td>C:Carbamidomethylation</td><td>121.6</td><td>539.5992</td><td>1616.777</td><td>0.006</td><td>3.7</td><td>5.38</td><td>87447.6</td><td>6.44</td><td>HUMAN</td><td>Q15437</td><td>Protein transport protein Sec23B</td></tr>
<tr><td>82</td><td>dirMS_041712_NAR_60mM_plus.20191.20362.3</td><td>3</td><td>23.01</td><td>1</td><td>3.02E+05</td><td></td><td>(K)IILDLISE</td><td>(K)I/I|L/D/L/I/S/E/S/P I K/G R(A)</td><td></td><td>166.2</td><td>518.6452</td><td>1553.926</td><td>-0.0051</td><td>-3.3</td><td>6.07</td><td>51260.6</td><td>5.39</td><td>HUMAN</td><td>P61978</td><td>Heterogeneous nuclear ribonucleoprotein K</td></tr>
<tr><td>83</td><td>dirMS_041712_NAR_30mM_plus2.18896.19212.2</td><td>2</td><td>23</td><td>0</td><td>3.29E+06</td><td></td><td>(R)QAQEYE</td><td>(R)Q A|Q|E|Y/E/A/L/L/N/I/K(V)</td><td></td><td>153.28</td><td>710.3766</td><td>1419.748</td><td>-0.0019</td><td>-1.3</td><td>4.53</td><td>43773.3</td><td>5.26</td><td>HUMAN</td><td>F8VZY9</td><td>Keratin, type I cytoskeletal 18</td></tr>
<tr><td>84</td><td>dirMS_041712_NAR_30mM_plus2.8906.8906.2</td><td>2</td><td>22.99</td><td>0</td><td>7.37E+05</td><td></td><td>(R)GMGPG</td><td>(R)G M/G/P G/T/P/A G Y/G R(G)</td><td></td><td>100.28</td><td>560.7657</td><td>1120.52</td><td>0.0037</td><td>3.3</td><td>8.75</td><td>76262.2</td><td>9.53</td><td>HUMAN</td><td>P23246</td><td>Splicing factor, proline- and glutamine-rich</td></tr>
<tr><td>85</td><td>dirMS_041712_NAR_30mM_minus2.18844.18914.2</td><td>2</td><td>22.95</td><td>0</td><td>4.54E+05</td><td></td><td>(R)ISEQFTA</td><td>(R)I/S|E/Q|F/T/A/M/F/R(R)</td><td></td><td>157.42</td><td>615.3038</td><td>1229.598</td><td>0.002</td><td>1.6</td><td>6</td><td>42831.5</td><td>4.86</td><td>HUMAN</td><td>A8K854</td><td>HCG1983504, isoform CRA_f</td></tr>
<tr><td>86</td><td>dirMS_041712_NAR_60mM_plus.14889.14938.3</td><td>3</td><td>22.95</td><td>0</td><td>6.52E+05</td><td></td><td>(R)GIYAYGI</td><td>(R)G I|Y|A|Y|G/F/E/K/P S A I Q Q R(A)</td><td></td><td>134.65</td><td>609.9803</td><td>1827.939</td><td>-0.0125</td><td>-6.8</td><td>8.5</td><td>16071.1</td><td>4.8</td><td>HUMAN</td><td>B4E102</td><td>Eukaryotic initiation factor 4A-I</td></tr>
<tr><td>87</td><td>dirMS_041712_NAR_40mM_plus2.15894.16181.3</td><td>3</td><td>22.94</td><td>0</td><td>3.61E+05</td><td></td><td>(K)VAIVKPC</td><td>(K)V/A|I|V/K/P G V|P|M/E I V L N/K(E)</td><td></td><td>161.22</td><td>569.6778</td><td>1707.024</td><td>-0.0049</td><td>-2.9</td><td>8.56</td><td>73146.9</td><td>5.45</td><td>HUMAN</td><td>E9PJ21</td><td>Hypoxia up-regulated protein 1 (Fragment)</td></tr>
<tr><td>88</td><td>dirMS_041712_NAR_60mM_plus.20165.20165.3</td><td>3</td><td>22.91</td><td>0</td><td>2.36E+05</td><td></td><td>(K)ALSAIAD</td><td>(K)A L/S/A/I A/D L/L/T/N E H E/R(V)</td><td></td><td>165.23</td><td>551.6246</td><td>1652.86</td><td>-0.001</td><td>-0.6</td><td>4.65</td><td>105417.2</td><td>6.41</td><td>HUMAN</td><td>C9JZR2</td><td>Catenin delta-1</td></tr>
<tr><td>89</td><td>dirMS_041712_NAR_120mM_minus.6663.6858.2</td><td>2</td><td>22.9</td><td>1</td><td>8.82E+06</td><td></td><td>(K)DSYVGD</td><td>(K)D/S|Y|V|G/D|E/A/Q/S K/R(G)</td><td></td><td>91.17</td><td>677.8145</td><td>1354.623</td><td>-0.0016</td><td>-1.2</td><td>4.56</td><td>32616.5</td><td>5.18</td><td>HUMAN</td><td>A6NL76</td><td>Actin, alpha skeletal muscle</td></tr>
<tr><td>90</td><td>dirMS_041712_NAR_120mM_plus.7561.7561.3</td><td>3</td><td>22.89</td><td>1</td><td>6.54E+05</td><td></td><td>(R)SGGYGG</td><td>(R)S/G|G/Y/G/G S R D Y Y/S S R(S)</td><td></td><td>93.37</td><td>504.5549</td><td>1511.651</td><td>-0.0008</td><td>-0.5</td><td>8.22</td><td>26585.7</td><td>11.25</td><td>HUMAN</td><td>B3KT17</td><td>Cold-inducible RNA-binding protein</td></tr>
<tr><td>91</td><td>dirMS_041712_NAR_30mM_plus2.18454.18633.2</td><td>2</td><td>22.89</td><td>0</td><td>2.28E+07</td><td></td><td>(K)LLCGLLA</td><td>(K)L/L|C|G|L|L|A E/R(L)</td><td>C:Carbamidomethylation</td><td>149.47</td><td>522.7969</td><td>1044.587</td><td>-0.0005</td><td>-0.5</td><td>6</td><td>12647.2</td><td>8.07</td><td>HUMAN</td><td>P14174</td><td>Macrophage migration inhibitory factor</td></tr>
<tr><td>92</td><td>dirMS_041712_NAR_30mM_plus2.11558.11826.2</td><td>2</td><td>22.87</td><td>0</td><td>1.28E+06</td><td></td><td>(R)GGGGNI</td><td>(R)G G/G/G\N|F|G|P/G/P/G S/N F/R(G)</td><td></td><td>114.4</td><td>689.3175</td><td>1377.629</td><td>-0.0017</td><td>-1.2</td><td>9.75</td><td>37486.1</td><td>9.03</td><td>HUMAN</td><td>P22626</td><td>Heterogeneous nuclear ribonucleoproteins A2/B1</td></tr>
<tr><td>93</td><td>dirMS_041712_NAR_30mM_plus2.6220.6338.2</td><td>2</td><td>22.87</td><td>0</td><td>5.37E+05</td><td></td><td>(K)DSYVGD</td><td>(K)D S|Y|V|G|D/E/A/Q/S/K(R)</td><td></td><td>87.18</td><td>599.7645</td><td>1198.522</td><td>-0.0005</td><td>-0.4</td><td>4.03</td><td>32616.5</td><td>5.18</td><td>HUMAN</td><td>A6NL76</td><td>Actin, alpha skeletal muscle</td></tr>
<tr><td>94</td><td>dirMS_041712_NAR_40mM_plus2.14663.14663.3</td><td>3</td><td>22.84</td><td>0</td><td>5.43E+05</td><td></td><td>(K)IAILTCPF</td><td>(K)I/A|I/L/T/C/P/F/E/P P K P K(T)</td><td>C:Carbamidomethylation</td><td>153.55</td><td>537.6381</td><td>1610.898</td><td>0.0023</td><td>1.4</td><td>8.59</td><td>49982</td><td>5.86</td><td>HUMAN</td><td>B4DYD8</td><td>T-complex protein 1 subunit epsilon</td></tr>
<tr><td>95</td><td>dirMS_041712_NAR_30mM_plus2.16475.16761.2</td><td>2</td><td>22.83</td><td>0</td><td>7.62E+04</td><td></td><td>(K)LGEWV</td><td>(K)L/G|E|W|V|G/L/C|K(I)</td><td>C:Carbamidomethylation</td><td>139.5</td><td>531.2766</td><td>1061.545</td><td>0.0011</td><td>1</td><td>6</td><td>14914.1</td><td>6.84</td><td>HUMAN</td><td>P25398</td><td>40S ribosomal protein S12</td></tr>
<tr><td>96</td><td>dirMS_041712_NAR_50mM_plus2.15824.15965.3</td><td>3</td><td>22.8</td><td>1</td><td>1.33E+06</td><td></td><td>(R)VFIGNLI</td><td>(R)V/F|I/G N L/N/T/L/V/V/K/K(S)</td><td></td><td>151.57</td><td>482.3007</td><td>1444.889</td><td>-0.0011</td><td>-0.7</td><td>10</td><td>15791.7</td><td>9.22</td><td>HUMAN</td><td>B4DSU6</td><td>Heterogeneous nuclear ribonucleoproteins C1/C2</td></tr>
<tr><td>97</td><td>dirMS_041712_NAR_60mM_plus.16048.16417.3</td><td>3</td><td>22.77</td><td>0</td><td>1.53E+06</td><td></td><td>(K)SLTNDW</td><td>(K)S L|T/N/D/W/E/D H/L/A V/K(H)</td><td></td><td>141.98</td><td>509.919</td><td>1527.744</td><td>-0.0014</td><td>-0.9</td><td>4.53</td><td>85057.6</td><td>4.94</td><td>HUMAN</td><td>P07900</td><td>Heat shock protein HSP 90-alpha</td></tr>
<tr><td>98</td><td>dirMS_041712_NAR_30mM_minus2.18014.18220.2</td><td>2</td><td>22.76</td><td>0</td><td>4.12E+05</td><td></td><td>(R)LLLNNDI</td><td>(R)L/L|L|N/N|D/N/L/L/R(E)</td><td></td><td>153.13</td><td>599.3487</td><td>1197.695</td><td>-0.0049</td><td>-4.1</td><td>5.84</td><td>19945.8</td><td>4.97</td><td>HUMAN</td><td>B4DG50</td><td>F-actin-capping protein subunit alpha-2</td></tr>
<tr><td>99</td><td>dirMS_041712_NAR_60mM_minus.7960.8074.3</td><td>3</td><td>22.75</td><td>1</td><td>4.84E+05</td><td></td><td>(R)YLAEVAT</td><td>(R)Y/L|A/E/V/A/T/G E K/R(A)</td><td></td><td>104.27</td><td>412.8902</td><td>1236.658</td><td>-0.0022</td><td>-1.8</td><td>6.14</td><td>28473.3</td><td>4.8</td><td>HUMAN</td><td>P61981</td><td>14-3-3 protein gamma</td></tr>
<tr><td>100</td><td>dirMS_041712_NAR_20mM_plus.16621.16621.2</td><td>2</td><td>22.74</td><td>0</td><td>5.45E+05</td><td></td><td>(K)LGGSAV</td><td>(K)L G G S A/V|I/S L|E/G\K|P L(-)</td><td></td><td>185.73</td><td>670.8917</td><td>1340.778</td><td>-0.0023</td><td>-1.7</td><td>6</td><td>22956</td><td>8.8</td><td>HUMAN</td><td>E9PK25</td><td>Cofilin-1</td></tr>
<tr><td>101</td><td>dirMS_041712_NAR_50mM_plus2.17513.17513.3</td><td>3</td><td>22.73</td><td>0</td><td>5.30E+05</td><td></td><td>(R)GGYVLH</td><td>(R)G G|Y/V|L/H/I/G T I/Y G D/L/K(V)</td><td></td><td>160.33</td><td>535.9588</td><td>1605.863</td><td>-0.0017</td><td>-1</td><td>6.74</td><td>30427.2</td><td>7.21</td><td>HUMAN</td><td>H3BPK7</td><td>Alanine--tRNA ligase, cytoplasmic (Fragment)</td></tr>
<tr><td>102</td><td>dirMS_041712_NAR_30mM_minus2.16981.17142.2</td><td>2</td><td>22.72</td><td>0</td><td>1.97E+05</td><td></td><td>(R)SIQEELC</td><td>(R)S/I|Q|E|E|L/Q/Q/L/R(Q)</td><td></td><td>146.5</td><td>622.3359</td><td>1243.664</td><td>0.0004</td><td>0.3</td><td>4.53</td><td>533778</td><td>5.74</td><td>HUMAN</td><td>Q15149</td><td>Plectin</td></tr>
<tr><td>103</td><td>dirMS_041712_NAR_60mM_plus.9518.9518.2</td><td>2</td><td>22.72</td><td>0</td><td>1.98E+06</td><td></td><td>(R)FATHAA</td><td>(R)F/A|T/H/A|A|A\L S V R(N)</td><td></td><td>107.55</td><td>572.3143</td><td>1143.627</td><td>-0.0056</td><td>-4.9</td><td>9.75</td><td>76262.2</td><td>9.53</td><td>HUMAN</td><td>P23246</td><td>Splicing factor, proline- and glutamine-rich</td></tr>
<tr><td>104</td><td>dirMS_041712_NAR_40mM_plus2.17759.17893.3</td><td>3</td><td>22.69</td><td>0</td><td>2.92E+05</td><td></td><td>(K)LLYDLAD</td><td>(K)L/L|Y|D|L/A/D/Q L/H/A/A/V|G A S R(A)</td><td></td><td>171.35</td><td>604.9967</td><td>1812.96</td><td>0.0153</td><td>8.4</td><td>5.21</td><td>24273.5</td><td>8.98</td><td>HUMAN</td><td>H0YK49</td><td>Electron transfer flavoprotein subunit alpha, mitochondrial</td></tr>
</table>

<table>
<tr><td>105</td><td>dirMS_041712_NAR_120mM_minus.7109.7216.3</td><td>3</td><td>22.66</td><td>1</td><td>6.92E+05</td><td></td><td>(K)LEEAEKA</td><td>(K)L/E|E/A/E/K/A/A/D/E/S E/R(G)</td><td></td><td>93.33</td><td>492.8986</td><td>1476.681</td><td>0</td><td>0</td><td>4.14</td><td>31923.6</td><td>4.85</td><td>HUMAN</td><td>B7Z596</td><td>Tropomyosin alpha-1 chain</td></tr>
<tr><td>106</td><td>dirMS_041712_NAR_30mM_minus2.13352.13475.2</td><td>2</td><td>22.65</td><td>0</td><td>5.19E+05</td><td></td><td>(R)YALYDAT</td><td>(R)Y/A|L|Y|D/A/T/Y/E/T/K(E)</td><td></td><td>126.48</td><td>669.3172</td><td>1337.626</td><td>0.0011</td><td>0.9</td><td>4.37</td><td>22956</td><td>8.8</td><td>HUMAN</td><td>E9PK25</td><td>Cofilin-1</td></tr>
<tr><td>107</td><td>dirMS_041712_NAR_30mM_plus2.14084.14281.2</td><td>2</td><td>22.62</td><td>0</td><td>9.98E+05</td><td></td><td>(R)MGQMA</td><td>(R)M/G/Q|M|A|M|G/G/A|M|G I/N/N/R(G)</td><td></td><td>127.32</td><td>769.8376</td><td>1538.67</td><td>-0.0015</td><td>-1</td><td>9.5</td><td>43979.5</td><td>8.92</td><td>HUMAN</td><td>F5GYZ3</td><td>Non-POU domain-containing octamer-binding protein</td></tr>
<tr><td>108</td><td>dirMS_041712_NAR_30mM_plus2.10864.10952.2</td><td>2</td><td>22.61</td><td>0</td><td>5.99E+05</td><td></td><td>(R)IIYGGSV</td><td>(R)I/I|Y|G|G|S/V/T/G A/T/C K(E)</td><td>C:Carbamidomethylation</td><td>110.2</td><td>663.839</td><td>1326.672</td><td>-0.0015</td><td>-1.1</td><td>8.59</td><td>31075.7</td><td>5.65</td><td>HUMAN</td><td>P60174</td><td>Triosephosphate isomerase</td></tr>
<tr><td>109</td><td>dirMS_041712_NAR_40mM_plus2.16629.16682.3</td><td>3</td><td>22.61</td><td>1</td><td>5.08E+05</td><td></td><td>(K)FGEVVD</td><td>(K)F/G|E|V|V/D C T L/K/L/D|P I\T G R(S)</td><td>C:Carbamidomethylation</td><td>164.38</td><td>640.6667</td><td>1919.989</td><td>-0.004</td><td>-2.1</td><td>4.56</td><td>33782.7</td><td>8.78</td><td>HUMAN</td><td>B4DTC3</td><td>Heterogeneous nuclear ribonucleoprotein D0</td></tr>
<tr><td>110</td><td>dirMS_041712_NAR_40mM_plus2.12260.12533.3</td><td>3</td><td>22.6</td><td>0</td><td>9.90E+05</td><td></td><td>(K)AGVVGF</td><td>(K)A/G|V|V|G|P E L/H/E/Q L L/S A E K(A)</td><td></td><td>139.35</td><td>592.987</td><td>1776.949</td><td>-0.0026</td><td>-1.5</td><td>4.75</td><td>533778</td><td>5.74</td><td>HUMAN</td><td>Q15149</td><td>Plectin</td></tr>
<tr><td>111</td><td>dirMS_041712_NAR_50mM_minus2.14685.14751.3</td><td>3</td><td>22.59</td><td>0</td><td>6.40E+04</td><td></td><td>(K)VYTDVQ</td><td>(K)V/Y|T/D/V/Q/Q/V|A/S S L T/H/P R(F)</td><td></td><td>151.52</td><td>600.976</td><td>1800.924</td><td>-0.0104</td><td>-5.8</td><td>6.71</td><td>74230.8</td><td>5.34</td><td>HUMAN</td><td>B1AH89</td><td>Tubulin tyrosine ligase-like family, member 12</td></tr>
<tr><td>112</td><td>dirMS_041712_NAR_30mM_plus2.14728.14768.2</td><td>2</td><td>22.59</td><td>0</td><td>7.82E+05</td><td></td><td>(R)LDIDSPP</td><td>(R)L/D|I|D|S|P/P I/T A R(N)</td><td></td><td>130.72</td><td>599.3284</td><td>1197.647</td><td>0.0021</td><td>1.8</td><td>4.21</td><td>5755.5</td><td>5.75</td><td>HUMAN</td><td>H3BN34</td><td>Pyruvate kinase isozymes M1/M2 (Fragment)</td></tr>
<tr><td>113</td><td>dirMS_041712_NAR_60mM_minus.8984.9232.3</td><td>3</td><td>22.57</td><td>0</td><td>2.40E+06</td><td></td><td>(K)STAGDT</td><td>(K)S/T|A/G/D/T/H/L/G/G E/D F D/N R(M)</td><td></td><td>110.85</td><td>564.5795</td><td>1691.726</td><td>-0.0016</td><td>-1</td><td>4.22</td><td>69032.8</td><td>5.37</td><td>HUMAN</td><td>E9PKE3</td><td>Heat shock cognate 71 kDa protein</td></tr>
<tr><td>114</td><td>dirMS_041712_NAR_40mM_plus2.13215.13547.3</td><td>3</td><td>22.55</td><td>1</td><td>2.56E+06</td><td></td><td>(K)VGEVIVT</td><td>(K)V/G|E|V/I|V/T/K/D D A M L L/K(G)</td><td></td><td>145.32</td><td>544.307</td><td>1630.908</td><td>-0.002</td><td>-1.2</td><td>4.56</td><td>61224.7</td><td>5.7</td><td>HUMAN</td><td>P10809</td><td>60 kDa heat shock protein, mitochondrial</td></tr>
<tr><td>115</td><td>dirMS_041712_NAR_50mM_plus2.22248.22300.3</td><td>3</td><td>22.55</td><td>0</td><td>1.53E+06</td><td></td><td>(R)DALSDLA</td><td>(R)D A|L/S|D/L/A/L/H/F L/N/K(M)</td><td></td><td>191.78</td><td>486.2644</td><td>1456.78</td><td>-0.0008</td><td>-0.6</td><td>5.21</td><td>42755</td><td>7.81</td><td>HUMAN</td><td>B7Z2F4</td><td>T-complex protein 1 subunit delta</td></tr>
<tr><td>116</td><td>dirMS_041712_NAR_20mM_plus.13986.14433.2</td><td>2</td><td>22.54</td><td>0</td><td>2.99E+06</td><td>M256m</td><td>(R)SLDmDS</td><td>(R)S L|D|m|D|S/I\I|A/E/V/K(A)</td><td>m:Oxidized methionine</td><td>170.42</td><td>668.8371</td><td>1320.671</td><td>15.9954</td><td>0.3</td><td>4.03</td><td>37296.3</td><td>5.3</td><td>HUMAN</td><td>F8VU64</td><td>Keratin, type II cytoskeletal 8</td></tr>
<tr><td>117</td><td>dirMS_041712_NAR_60mM_plus.4777.4943.3</td><td>3</td><td>22.51</td><td>1</td><td>1.78E+06</td><td></td><td>(R)AAEEED</td><td>(R)A/A|E/E/E/D/E/A/D/P K/R(Q)</td><td></td><td>80</td><td>453.872</td><td>1359.602</td><td>-0.0008</td><td>-0.6</td><td>4.08</td><td>11529.7</td><td>4.14</td><td>HUMAN</td><td>P20962</td><td>Parathymosin</td></tr>
<tr><td>118</td><td>dirMS_041712_NAR_40mM_plus2.18649.18908.3</td><td>3</td><td>22.46</td><td>1</td><td>4.43E+05</td><td></td><td>(R)TLAEIAK</td><td>(R)T/L|A/E/I/A/K/V/E L/D/N M/P L/R(G)</td><td></td><td>176.68</td><td>604.9967</td><td>1812.989</td><td>-0.0132</td><td>-7.3</td><td>4.68</td><td>23729.5</td><td>9.89</td><td>HUMAN</td><td>C9IZL7</td><td>Non-POU domain-containing octamer-binding protein (Fragment)</td></tr>
<tr><td>119</td><td>dirMS_041712_NAR_60mM_minus.13983.14005.3</td><td>3</td><td>22.44</td><td>0</td><td>3.54E+05</td><td></td><td>(K)DCGATV</td><td>(K)D C|G A/T/W/V/V/L/G H/S E R(R)</td><td>C:Carbamidomethylation</td><td>138.02</td><td>529.5838</td><td>1586.738</td><td>-0.0012</td><td>-0.7</td><td>5.32</td><td>31075.7</td><td>5.65</td><td>HUMAN</td><td>P60174</td><td>Triosephosphate isomerase</td></tr>
<tr><td>120</td><td>dirMS_041712_NAR_30mM_minus2.17319.17710.2</td><td>2</td><td>22.41</td><td>0</td><td>1.10E+07</td><td></td><td>(K)LLCGLLA</td><td>(K)L/L|C|G|L|L/A/E/R(L)</td><td>C:Carbamidomethylation</td><td>151.02</td><td>522.7963</td><td>1044.587</td><td>-0.0017</td><td>-1.6</td><td>6</td><td>12647.2</td><td>8.07</td><td>HUMAN</td><td>P14174</td><td>Macrophage migration inhibitory factor</td></tr>
<tr><td>121</td><td>dirMS_041712_NAR_50mM_minus2.18797.18842.3</td><td>3</td><td>22.41</td><td>1</td><td>2.24E+06</td><td></td><td>(R)LIALLEVI</td><td>(R)L/I|A|L|L/E/V/L/S Q K/R(M)</td><td></td><td>183.72</td><td>461.6289</td><td>1382.873</td><td>-0.0008</td><td>-0.6</td><td>8.75</td><td>280326.9</td><td>5.48</td><td>HUMAN</td><td>O75369</td><td>Filamin-B</td></tr>
<tr><td>122</td><td>dirMS_041712_NAR_40mM_plus2.14414.14743.3</td><td>3</td><td>22.41</td><td>1</td><td>7.60E+05</td><td></td><td>(K)IIVDELKC</td><td>(K)I/I|V/D/E|L/K/Q|E/V/I/S T/S S K(A)</td><td></td><td>152.95</td><td>597.0023</td><td>1788.995</td><td>-0.003</td><td>-1.7</td><td>4.68</td><td>30149.5</td><td>5.96</td><td>HUMAN</td><td>D6RAC2</td><td>Guanine nucleotide-binding protein subunit beta-2-like 1</td></tr>
<tr><td>123</td><td>dirMS_041712_NAR_20mM_plus.16784.17048.2</td><td>2</td><td>22.4</td><td>0</td><td>3.93E+05</td><td></td><td>(K)VQIAVA</td><td>(K)V Q|I|A|V/A|N/A/Q|E L/L/Q R(M)</td><td></td><td>187.38</td><td>776.9419</td><td>1552.881</td><td>-0.004</td><td>-2.6</td><td>5.97</td><td>10728.1</td><td>9.15</td><td>HUMAN</td><td>Q9Y5L4</td><td>Mitochondrial import inner membrane translocase subunit Tim13</td></tr>
<tr><td>124</td><td>dirMS_041712_NAR_50mM_plus2.14478.14695.3</td><td>3</td><td>22.38</td><td>0</td><td>2.11E+06</td><td></td><td>(R)QVSASE</td><td>(R)Q/V|S/A/S E/L/H/T/S G I/L/G/P E T L R(D)</td><td></td><td>144.9</td><td>665.6855</td><td>1995.051</td><td>-0.0086</td><td>-4.3</td><td>5.4</td><td>558007.1</td><td>5.44</td><td>HUMAN</td><td>P58107</td><td>Epiplakin</td></tr>
<tr><td>125</td><td>dirMS_041712_NAR_30mM_plus2.13891.14114.2</td><td>2</td><td>22.36</td><td>0</td><td>2.02E+06</td><td></td><td>(K)YGEPGE</td><td>(K)Y/G|E|P/G E V|F/I/N/K(G)</td><td></td><td>126.27</td><td>626.8136</td><td>1252.621</td><td>-0.0009</td><td>-0.7</td><td>4.53</td><td>76262.2</td><td>9.53</td><td>HUMAN</td><td>P23246</td><td>Splicing factor, proline- and glutamine-rich</td></tr>
<tr><td>126</td><td>dirMS_041712_NAR_30mM_minus2.14968.15028.2</td><td>2</td><td>22.35</td><td>0</td><td>5.54E+05</td><td>M316m</td><td>(R)ISEQFTA</td><td>(R)I/S|E/Q/F/T/A/m/F/R(R)</td><td>m:Oxidized methionine</td><td>135.18</td><td>623.3014</td><td>1229.598</td><td>15.9972</td><td>1.8</td><td>6</td><td>42831.5</td><td>4.86</td><td>HUMAN</td><td>A8K854</td><td>HCG1983504, isoform CRA_f</td></tr>
<tr><td>127</td><td>dirMS_041712_NAR_40mM_minus2.12508.12525.3</td><td>3</td><td>22.35</td><td>1</td><td>5.98E+05</td><td></td><td>(R)VCENIPI</td><td>(R)V/C/E/N/I|P I V/L/C G N K V D|I/K(D)</td><td>C:Carbamidomethylation</td><td>157.7</td><td>657.6842</td><td>1971.04</td><td>-0.0021</td><td>-1.1</td><td>6.04</td><td>14844.8</td><td>5.89</td><td>HUMAN</td><td>B4DV51</td><td>GTP-binding nuclear protein Ran</td></tr>
<tr><td>128</td><td>dirMS_041712_NAR_50mM_plus2.19178.19505.3</td><td>3</td><td>22.35</td><td>1</td><td>4.80E+06</td><td></td><td>(K)TFVNITP</td><td>(K)T F|V|N|I/T/P A E V/G/V L/V/G K D R(S)</td><td></td><td>170.17</td><td>639.0247</td><td>1915.065</td><td>-0.0052</td><td>-2.7</td><td>5.74</td><td>17630.6</td><td>11.62</td><td>HUMAN</td><td>I3L3D5</td><td>Profilin-1 (Fragment)</td></tr>
<tr><td>129</td><td>dirMS_041712_NAR_60mM_plus.16636.16697.3</td><td>3</td><td>22.35</td><td>0</td><td>3.03E+05</td><td></td><td>(R)DLSYCLS</td><td>(R)D L|S/Y/C/L/S/G/M/Y/D/H/R(Y)</td><td>C:Carbamidomethylation</td><td>143.93</td><td>539.5662</td><td>1616.683</td><td>0.0009</td><td>0.5</td><td>5.21</td><td>46013.4</td><td>5.38</td><td>HUMAN</td><td>P52597</td><td>Heterogeneous nuclear ribonucleoprotein F</td></tr>
<tr><td>130</td><td>dirMS_041712_NAR_50mM_minus2.13513.13971.3</td><td>3</td><td>22.34</td><td>0</td><td>1.98E+06</td><td></td><td>(K)MLDAED</td><td>(K)M/L|D|A|E/D/I/V/N T A R|P D/E K(A)</td><td></td><td>147.25</td><td>606.2965</td><td>1816.875</td><td>0.0004</td><td>0.2</td><td>4.1</td><td>105308.6</td><td>5.27</td><td>HUMAN</td><td>O43707</td><td>Alpha-actinin-4</td></tr>
<tr><td>131</td><td>dirMS_041712_NAR_120mM_minus.13490.13534.3</td><td>3</td><td>22.33</td><td>2</td><td>9.83E+05</td><td></td><td>(R)AQKDEE</td><td>(R)A Q|K/D E/E/K/M E|I|Q/E|I/Q\L/K(E)</td><td></td><td>128.13</td><td>654.0058</td><td>1960.006</td><td>-0.0027</td><td>-1.4</td><td>4.58</td><td>31923.6</td><td>4.85</td><td>HUMAN</td><td>B7Z596</td><td>Tropomyosin alpha-1 chain</td></tr>
<tr><td>132</td><td>dirMS_041712_NAR_50mM_plus2.9971.10230.3</td><td>3</td><td>22.32</td><td>1</td><td>1.70E+06</td><td></td><td>(K)TLQEVTE</td><td>(K)T L|Q/E/V/T/E/M/D/S V K/R(Y)</td><td></td><td>120.07</td><td>512.5958</td><td>1535.773</td><td>-0.0005</td><td>-0.3</td><td>4.68</td><td>558007.1</td><td>5.44</td><td>HUMAN</td><td>P58107</td><td>Epiplakin</td></tr>
<tr><td>133</td><td>dirMS_041712_NAR_20mM_plus.9586.9891.2</td><td>2</td><td>22.31</td><td>0</td><td>6.61E+05</td><td></td><td>(R)ISVYYNE</td><td>(R)I/S|V|Y|Y/N/E\A/T/G G K(Y)</td><td></td><td>138.4</td><td>651.3211</td><td>1301.637</td><td>-0.0023</td><td>-1.8</td><td>6</td><td>27549.9</td><td>4.39</td><td>HUMAN</td><td>E9PBJ4</td><td>Tubulin beta chain</td></tr>
<tr><td>134</td><td>dirMS_041712_NAR_30mM_plus2.11097.11141.2</td><td>2</td><td>22.31</td><td>0</td><td>2.79E+05</td><td></td><td>(R)TLIQNCC</td><td>(R)T L|I|Q|N|C/G A/S/T/I/R(L)</td><td>C:Carbamidomethylation</td><td>111.48</td><td>667.3485</td><td>1333.689</td><td>0.0005</td><td>0.3</td><td>9.41</td><td>56186.7</td><td>5.47</td><td>HUMAN</td><td>B4DUR8</td><td>T-complex protein 1 subunit gamma</td></tr>
<tr><td>135</td><td>dirMS_041712_NAR_60mM_minus.5567.5879.2</td><td>2</td><td>22.3</td><td>1</td><td>4.42E+06</td><td></td><td>(K)DSYVGD</td><td>(K)D/S|Y|V|G/D/E/A Q/S/K/R(G)</td><td></td><td>87.58</td><td>677.8144</td><td>1354.623</td><td>-0.0018</td><td>-1.3</td><td>4.56</td><td>32616.5</td><td>5.18</td><td>HUMAN</td><td>A6NL76</td><td>Actin, alpha skeletal muscle</td></tr>
<tr><td>136</td><td>dirMS_041712_NAR_60mM_plus.11688.11819.3</td><td>3</td><td>22.3</td><td>1</td><td>2.17E+06</td><td></td><td>(K)TLQEVTE</td><td>(K)T/L|Q/E/V/T/E/M/D/S V K/R(Y)</td><td></td><td>118.97</td><td>512.5957</td><td>1535.773</td><td>-0.0008</td><td>-0.5</td><td>4.68</td><td>558007.1</td><td>5.44</td><td>HUMAN</td><td>P58107</td><td>Epiplakin</td></tr>
<tr><td>137</td><td>dirMS_041712_NAR_50mM_minus2.14184.14477.3</td><td>3</td><td>22.29</td><td>1</td><td>6.62E+05</td><td></td><td>(R)VEKIPGC</td><td>(R)V E|K I|P G G\I|I|E|D/S/C/V|L/R(G)</td><td>C:Carbamidomethylation</td><td>149.1</td><td>595.6534</td><td>1784.958</td><td>-0.0118</td><td>-6.6</td><td>4.68</td><td>56186.7</td><td>5.47</td><td>HUMAN</td><td>B4DUR8</td><td>T-complex protein 1 subunit gamma</td></tr>
<tr><td>138</td><td>dirMS_041712_NAR_50mM_plus2.17791.17974.3</td><td>3</td><td>22.27</td><td>1</td><td>1.07E+05</td><td></td><td>(K)TVDNFV</td><td>(K)T/V|D/N|F/V/A/L|A T/G E K G F G Y K(N)</td><td></td><td>161.83</td><td>639.6639</td><td>1916.975</td><td>0.0019</td><td>1</td><td>5.73</td><td>23799.1</td><td>9.48</td><td>HUMAN</td><td>P23284</td><td>Peptidyl-prolyl cis-trans isomerase B</td></tr>
<tr><td>139</td><td>dirMS_041712_NAR_60mM_plus.7014.7056.3</td><td>3</td><td>22.26</td><td>1</td><td>2.99E+06</td><td></td><td>(K)IVSGKDY</td><td>(K)I/V|S/G/K/D/Y/N|V/T/A N/S K(L)</td><td></td><td>95.17</td><td>499.2612</td><td>1495.775</td><td>-0.0061</td><td>-4</td><td>8.5</td><td>12539.4</td><td>5.78</td><td>HUMAN</td><td>F5GXH2</td><td>L-lactate dehydrogenase A chain (Fragment)</td></tr>
<tr><td>140</td><td>dirMS_041712_NAR_60mM_plus.11619.11800.3</td><td>3</td><td>22.25</td><td>0</td><td>2.82E+06</td><td></td><td>(K)INEKPQ\</td><td>(K)I/N|E/K/P\Q\V|I|A|D|Y/E/S G R(A)</td><td></td><td>118.52</td><td>573.6312</td><td>1718.871</td><td>0.0083</td><td>4.8</td><td>4.68</td><td>16368.4</td><td>9.95</td><td>HUMAN</td><td>O60869</td><td>Endothelial differentiation-related factor 1</td></tr>
<tr><td>141</td><td>dirMS_041712_NAR_30mM_plus2.18410.18507.2</td><td>2</td><td>22.24</td><td>0</td><td>2.03E+05</td><td></td><td>(R)AVLNPLC</td><td>(R)A V|L|N|P/L/C Q/V/D/Y R(A)</td><td>C:Carbamidomethylation</td><td>150.18</td><td>724.3703</td><td>1447.736</td><td>-0.0029</td><td>-2</td><td>5.88</td><td>83880.6</td><td>7.33</td><td>HUMAN</td><td>F5H365</td><td>Protein transport protein Sec23A</td></tr>
<tr><td>142</td><td>dirMS_041712_NAR_50mM_plus2.10999.11247.3</td><td>3</td><td>22.23</td><td>0</td><td>1.32E+06</td><td></td><td>(K)VGSLDN</td><td>(K)V/G|S|L/D/N V/G/H/L|P/A G G A V/K(I)</td><td></td><td>126.15</td><td>530.9567</td><td>1590.86</td><td>-0.0043</td><td>-2.7</td><td>6.71</td><td>26233.5</td><td>10.95</td><td>HUMAN</td><td>B4DSQ1</td><td>Microtubule-associated protein</td></tr>
<tr><td>143</td><td>dirMS_041712_NAR_30mM_minus2.10796.11283.2</td><td>2</td><td>22.22</td><td>0</td><td>5.46E+06</td><td></td><td>(R)INVYYNE</td><td>(R)I/N|V|Y|Y|N/E/A/T/G G K(Y)</td><td></td><td>113.98</td><td>664.8273</td><td>1328.648</td><td>-0.0008</td><td>-0.6</td><td>6</td><td>50286.6</td><td>4.79</td><td>HUMAN</td><td>P68371</td><td>Tubulin beta-4B chain</td></tr>
<tr><td>144</td><td>dirMS_041712_NAR_30mM_plus2.6165.6171.2</td><td>2</td><td>22.22</td><td>0</td><td>1.84E+05</td><td></td><td>(K)CDSSPD</td><td>(K)C/D|S|S|P/D/S/A/E|D|V/R(K)</td><td>C:Carbamidomethylation</td><td>86.6</td><td>669.2671</td><td>1337.527</td><td>-0.0005</td><td>-0.4</td><td>3.84</td><td>40122.7</td><td>5.43</td><td>HUMAN</td><td>P02765</td><td>Alpha-2-HS-glycoprotein</td></tr>
<tr><td>145</td><td>dirMS_041712_NAR_60mM_plus.14265.14266.3</td><td>3</td><td>22.22</td><td>0</td><td>1.28E+06</td><td></td><td>(R)VRPCVV</td><td>(R)V R\P C\V\V|Y|G G\A/D|I|G\Q/Q I/R(D)</td><td>C:Carbamidomethylation</td><td>131.43</td><td>629.9993</td><td>1887.986</td><td>-0.0024</td><td>-1.3</td><td>8.71</td><td>73386.6</td><td>8.73</td><td>HUMAN</td><td>B4DXX7</td><td>ATP-dependent RNA helicase DDX3Y</td></tr>
<tr><td>146</td><td>dirMS_041712_NAR_50mM_plus2.22481.22496.3</td><td>3</td><td>22.21</td><td>1</td><td>1.26E+06</td><td></td><td>(R)GVMLA\</td><td>(R)G V|M/L/A/V/D/A V I/A E L K/K(Q)</td><td></td><td>195.7</td><td>519.6393</td><td>1556.908</td><td>-0.0047</td><td>-3</td><td>6.07</td><td>25225.7</td><td>9.52</td><td>HUMAN</td><td>E7ESH4</td><td>60 kDa heat shock protein, mitochondrial (Fragment)</td></tr>
<tr><td>147</td><td>dirMS_041712_NAR_60mM_plus.6333.6424.3</td><td>3</td><td>22.21</td><td>1</td><td>7.26E+06</td><td></td><td>(K)DSYVGD</td><td>(K)D S|Y|V/G/D/E/A/Q/S K R(G)</td><td></td><td>91.98</td><td>452.213</td><td>1354.623</td><td>0.0011</td><td>0.8</td><td>4.56</td><td>32616.5</td><td>5.18</td><td>HUMAN</td><td>A6NL76</td><td>Actin, alpha skeletal muscle</td></tr>
<tr><td>148</td><td>dirMS_041712_NAR_50mM_plus2.15840.16066.3</td><td>3</td><td>22.19</td><td>0</td><td>8.00E+06</td><td></td><td>(R)LLIHQSL</td><td>(R)L/L|I/H Q|S\L|A/G/G I/I/G V/K(G)</td><td></td><td>151.85</td><td>506.9836</td><td>1518.937</td><td>-0.0004</td><td>-0.2</td><td>8.76</td><td>51260.6</td><td>5.39</td><td>HUMAN</td><td>P61978</td><td>Heterogeneous nuclear ribonucleoprotein K</td></tr>
<tr><td>149</td><td>dirMS_041712_NAR_120mM_plus.8745.9153.3</td><td>3</td><td>22.18</td><td>0</td><td>8.60E+06</td><td></td><td>(R)FACHSA</td><td>(R)F/A|C/H/S/A/S|L/T\V/R(N)</td><td>C:Carbamidomethylation</td><td>101.02</td><td>416.8772</td><td>1248.615</td><td>0.0017</td><td>1.3</td><td>9.75</td><td>23729.5</td><td>9.89</td><td>HUMAN</td><td>C9IZL7</td><td>Non-POU domain-containing octamer-binding protein (Fragment)</td></tr>
<tr><td>150</td><td>dirMS_041712_NAR_30mM_minus2.15723.16244.2</td><td>2</td><td>22.17</td><td>0</td><td>4.79E+06</td><td></td><td>(K)YMACCL</td><td>(K)Y/M|A|C|C/L/L|Y/R(G)</td><td>C:Carbamidomethylation</td><td>140.28</td><td>625.2802</td><td>1249.553</td><td>0.0005</td><td>0.4</td><td>8.59</td><td>50706.6</td><td>5.68</td><td>HUMAN</td><td>A6NHL2</td><td>Tubulin alpha chain-like 3</td></tr>
<tr><td>151</td><td>dirMS_041712_NAR_120mM_minus.6639.7209.3</td><td>3</td><td>22.15</td><td>1</td><td>3.27E+07</td><td></td><td>(K)DSYVGD</td><td>(K)D S|Y|V/G/D/E/A/Q/S K R(G)</td><td></td><td>91.23</td><td>452.2134</td><td>1354.623</td><td>0.0023</td><td>1.7</td><td>4.56</td><td>32616.5</td><td>5.18</td><td>HUMAN</td><td>A6NL76</td><td>Actin, alpha skeletal muscle</td></tr>
<tr><td>152</td><td>dirMS_041712_NAR_40mM_plus2.13574.13991.3</td><td>3</td><td>22.15</td><td>1</td><td>1.93E+06</td><td></td><td>(K)TVGVEP.</td><td>(K)T/V|G|V/E/P A A D/G K G V V V/V/I/K(R)</td><td></td><td>147.37</td><td>580.0077</td><td>1738.011</td><td>-0.0024</td><td>-1.4</td><td>5.73</td><td>7945.1</td><td>11.03</td><td>HUMAN</td><td>C9JB50</td><td>60S ribosomal protein L28</td></tr>
<tr><td>153</td><td>dirMS_041712_NAR_40mM_minus2.14676.14676.3</td><td>3</td><td>22.13</td><td>1</td><td>1.37E+06</td><td></td><td>(K)TFVNITP</td><td>(K)T F|V/N|I|T/P A E V/G V L V G K D R(S)</td><td></td><td>171.75</td><td>639.0252</td><td>1915.065</td><td>-0.0037</td><td>-1.9</td><td>5.74</td><td>17630.6</td><td>11.62</td><td>HUMAN</td><td>I3L3D5</td><td>Profilin-1 (Fragment)</td></tr>
<tr><td>154</td><td>dirMS_041712_NAR_50mM_minus2.18150.18359.3</td><td>3</td><td>22.12</td><td>0</td><td>4.52E+06</td><td></td><td>(K)ALESPEF</td><td>(K)A/L|E|S|P E/R P/F L|A/I/L|G G A K(V)</td><td></td><td>174.72</td><td>590.3356</td><td>1768.996</td><td>-0.0033</td><td>-1.9</td><td>6.19</td><td>41827.5</td><td>7</td><td>HUMAN</td><td>B7Z7A9</td><td>Phosphoglycerate kinase</td></tr>
<tr><td>155</td><td>dirMS_041712_NAR_30mM_plus2.19575.19903.2</td><td>2</td><td>22.1</td><td>0</td><td>1.62E+06</td><td></td><td>(R)ISEQFTA</td><td>(R)I/S|E/Q|F/T/A/M/F/R(R)</td><td></td><td>157.12</td><td>615.3024</td><td>1229.598</td><td>-0.0008</td><td>-0.6</td><td>6</td><td>42831.5</td><td>4.86</td><td>HUMAN</td><td>A8K854</td><td>HCG1983504, isoform CRA_f</td></tr>
<tr><td>156</td><td>dirMS_041712_NAR_30mM_plus2.6115.6115.2</td><td>2</td><td>22.09</td><td>0</td><td>8.14E+05</td><td></td><td>(R)NQGGY(</td><td>(R)N/Q|G/G|Y/G G S S/S S/S S Y|G S G R(R)</td><td></td><td>86.18</td><td>847.8524</td><td>1694.7</td><td>-0.0026</td><td>-1.5</td><td>8.59</td><td>33268.7</td><td>9.31</td><td>HUMAN</td><td>F8VRQ1</td><td>Heterogeneous nuclear ribonucleoprotein A1</td></tr>
<tr><td>157</td><td>dirMS_041712_NAR_50mM_plus2.10344.10364.3</td><td>3</td><td>22.08</td><td>1</td><td>8.18E+05</td><td></td><td>(K)LLEDKN(</td><td>(K)L/L|E|D/K/N|G\E|V/Q N L A/V/K(C)</td><td></td><td>121.98</td><td>557.3066</td><td>1669.912</td><td>-0.0067</td><td>-4</td><td>4.68</td><td>6116.1</td><td>5.2</td><td>HUMAN</td><td>F5H6I6</td><td>Cullin-associated NEDD8-dissociated protein 1 (Fragment)</td></tr>
<tr><td>158</td><td>dirMS_041712_NAR_40mM_plus2.13885.14237.2</td><td>2</td><td>22.07</td><td>0</td><td>5.22E+05</td><td></td><td>(K)LLCGLLA</td><td>(K)L/L|C|G|L|L/A E/R(L)</td><td>C:Carbamidomethylation</td><td>148.95</td><td>522.7962</td><td>1044.587</td><td>-0.0019</td><td>-1.8</td><td>6</td><td>12647.2</td><td>8.07</td><td>HUMAN</td><td>P14174</td><td>Macrophage migration inhibitory factor</td></tr>
<tr><td>159</td><td>dirMS_041712_NAR_60mM_minus.18877.19124.3</td><td>3</td><td>22.06</td><td>1</td><td>3.75E+06</td><td></td><td>(R)GLFIIDD</td><td>(R)G L|F|I/I/D/D/K/G I/L/R(Q)</td><td></td><td>168.1</td><td>453.9368</td><td>1359.8</td><td>-0.0036</td><td>-2.7</td><td>5.96</td><td>18498.6</td><td>5.82</td><td>HUMAN</td><td>H7C3T4</td><td>Peroxiredoxin-4 (Fragment)</td></tr>
<tr><td>160</td><td>dirMS_041712_NAR_30mM_plus2.15962.16172.2</td><td>2</td><td>22.06</td><td>0</td><td>6.55E+05</td><td></td><td>(R)ICCDLD\</td><td>(R)I/C|C|D|L/D/V/L|A/S/K(K)</td><td>C:Carbamidomethylation</td><td>137.37</td><td>647.3131</td><td>1293.618</td><td>0.0012</td><td>0.9</td><td>4.21</td><td>61363</td><td>8.73</td><td>HUMAN</td><td>C9JQS9</td><td>Propionyl-CoA carboxylase beta chain, mitochondrial</td></tr>
<tr><td>161</td><td>dirMS_041712_NAR_20mM_minus.8898.9091.2</td><td>2</td><td>22.05</td><td>0</td><td>1.86E+06</td><td></td><td>(K)LVSESSD</td><td>(K)L/V|S|E/S/S D|V/L|P K(-)</td><td></td><td>139.67</td><td>587.321</td><td>1173.636</td><td>-0.0014</td><td>-1.2</td><td>4.37</td><td>56607.4</td><td>5.37</td><td>HUMAN</td><td>F8VXB4</td><td>Keratin, type II cytoskeletal 8</td></tr>
<tr><td>162</td><td>dirMS_041712_NAR_30mM_minus2.10066.10660.2</td><td>2</td><td>22.05</td><td>0</td><td>7.01E+07</td><td></td><td>(R)GYSFTTT</td><td>(R)G/Y|S|F/T/T/T/A/E/R(E)</td><td></td><td>109.67</td><td>566.7699</td><td>1132.527</td><td>0.0056</td><td>4.9</td><td>6</td><td>23901.9</td><td>5.28</td><td>HUMAN</td><td>I3L1U9</td><td>Actin, cytoplasmic 2, N-terminally processed (Fragment)</td></tr>
<tr><td>163</td><td>dirMS_041712_NAR_60mM_plus.14323.14446.3</td><td>3</td><td>22.05</td><td>1</td><td>1.92E+06</td><td></td><td>(K)DVDEAY</td><td>(K)D V|D|E|A/Y/M/N K/V/E/L E/S R(L)</td><td></td><td>131.82</td><td>599.9485</td><td>1797.832</td><td>-0.0014</td><td>-0.8</td><td>4.18</td><td>13588.1</td><td>4.66</td><td>HUMAN</td><td>F8VP67</td><td>Keratin, type II cytoskeletal 8 (Fragment)</td></tr>
<tr><td>164</td><td>dirMS_041712_NAR_40mM_minus2.12206.12206.3</td><td>3</td><td>22.03</td><td>0</td><td>4.57E+05</td><td></td><td>(K)IAILTCPF</td><td>(K)I/A/I/L/T C|P F E/P P K/P K(T)</td><td>C:Carbamidomethylation</td><td>155.68</td><td>537.6382</td><td>1610.898</td><td>0.0026</td><td>1.6</td><td>8.59</td><td>49982</td><td>5.86</td><td>HUMAN</td><td>B4DYD8</td><td>T-complex protein 1 subunit epsilon</td></tr>
<tr><td>165</td><td>dirMS_041712_NAR_20mM_plus.17508.17640.2</td><td>2</td><td>22.01</td><td>0</td><td>3.08E+05</td><td></td><td>(K)SYELPDC</td><td>(K)S Y|E|L|P D/G Q/V|I|T|I|G N E R(F)</td><td></td><td>193.17</td><td>895.9492</td><td>1790.892</td><td>-0.0008</td><td>-0.4</td><td>4.14</td><td>123097.1</td><td>5.83</td><td>HUMAN</td><td>A5A3E0</td><td>POTE ankyrin domain family member F</td></tr>
<tr><td>166</td><td>dirMS_041712_NAR_120mM_plus.5522.5607.3</td><td>3</td><td>21.96</td><td>1</td><td>2.74E+06</td><td></td><td>(R)SGGGYG</td><td>(R)S/G|G|G|Y/G/G D/R|G G G/Y/G G D/R(G)</td><td></td><td>82.1</td><td>515.5539</td><td>1544.647</td><td>-0.0001</td><td>-0.1</td><td>5.68</td><td>21563.9</td><td>10.01</td><td>HUMAN</td><td>K7EKB2</td><td>TATA-binding protein-associated factor 2N (Fragment)</td></tr>
<tr><td>167</td><td>dirMS_041712_NAR_120mM_minus.7110.7112.3</td><td>3</td><td>21.93</td><td>1</td><td>6.92E+05</td><td></td><td>(K)LEEAEKA</td><td>(K)L/E|E\A/E/K/A/A/D/E S E/R(G)</td><td></td><td>93.33</td><td>492.8986</td><td>1476.681</td><td>0</td><td>0</td><td>4.14</td><td>31923.6</td><td>4.85</td><td>HUMAN</td><td>B7Z596</td><td>Tropomyosin alpha-1 chain</td></tr>
<tr><td>168</td><td>dirMS_041712_NAR_60mM_minus.8691.9230.3</td><td>3</td><td>21.93</td><td>0</td><td>3.43E+07</td><td></td><td>(K)QEYDES</td><td>(K)Q E/Y/D E/S/G/P/S/I/V/H/R(K)</td><td></td><td>110.32</td><td>506.2391</td><td>1516.703</td><td>0.0001</td><td>0.1</td><td>4.65</td><td>123097.1</td><td>5.83</td><td>HUMAN</td><td>A5A3E0</td><td>POTE ankyrin domain family member F</td></tr>
<tr><td>169</td><td>dirMS_041712_NAR_20mM_plus.9658.9658.2</td><td>2</td><td>21.88</td><td>0</td><td>3.06E+05</td><td></td><td>(K)LVLVGD</td><td>(K)L V|L|V/G D/G G T G/K(T)</td><td></td><td>137.43</td><td>508.292</td><td>1015.578</td><td>-0.0015</td><td>-1.5</td><td>5.84</td><td>26394.9</td><td>6.5</td><td>HUMAN</td><td>B5MDF5</td><td>GTP-binding nuclear protein Ran</td></tr>
<tr><td>170</td><td>dirMS_041712_NAR_20mM_minus.6164.6206.2</td><td>2</td><td>21.85</td><td>0</td><td>3.80E+06</td><td></td><td>(K)DSYVGD</td><td>(K)D S|Y|V|G/D/E/A/Q/S K(R)</td><td></td><td>103.87</td><td>599.7642</td><td>1198.522</td><td>-0.0011</td><td>-0.9</td><td>4.03</td><td>32616.5</td><td>5.18</td><td>HUMAN</td><td>A6NL76</td><td>Actin, alpha skeletal muscle</td></tr>
<tr><td>171</td><td>dirMS_041712_NAR_30mM_minus2.10375.10539.2</td><td>2</td><td>21.83</td><td>0</td><td>3.64E+05</td><td></td><td>(R)IIYGGSV</td><td>(R)I/I|Y|G/G/S/V|T/G/A T C K(E)</td><td>C:Carbamidomethylation</td><td>110.8</td><td>663.8388</td><td>1326.672</td><td>-0.0019</td><td>-1.4</td><td>8.59</td><td>31075.7</td><td>5.65</td><td>HUMAN</td><td>P60174</td><td>Triosephosphate isomerase</td></tr>
<tr><td>172</td><td>dirMS_041712_NAR_30mM_minus2.13401.13511.2</td><td>2</td><td>21.82</td><td>0</td><td>6.43E+05</td><td></td><td>(K)YLGQDY</td><td>(K)Y/L|G|Q|D|Y/E/Q L/R(V)</td><td></td><td>126.6</td><td>642.814</td><td>1284.622</td><td>-0.0012</td><td>-0.9</td><td>4.37</td><td>7312.2</td><td>6.55</td><td>HUMAN</td><td>E9PIA9</td><td>Calpain-1 catalytic subunit (Fragment)</td></tr>
<tr><td>173</td><td>dirMS_041712_NAR_40mM_plus2.11905.12188.3</td><td>3</td><td>21.82</td><td>1</td><td>1.25E+06</td><td>M356m</td><td>(K)VGEVIVT</td><td>(K)V/G|E|V|I|V/T/K D D A m L L/K(G)</td><td>m:Oxidized methionine</td><td>137</td><td>549.6385</td><td>1630.908</td><td>15.9925</td><td>-1.5</td><td>4.56</td><td>61224.7</td><td>5.7</td><td>HUMAN</td><td>P10809</td><td>60 kDa heat shock protein, mitochondrial</td></tr>
<tr><td>174</td><td>dirMS_041712_NAR_30mM_plus2.8204.8287.2</td><td>2</td><td>21.8</td><td>0</td><td>2.10E+05</td><td></td><td>(R)DSYDSY</td><td>(R)D S|Y/D|S|Y/A/T/H/N/E(-)</td><td></td><td>97.03</td><td>651.2492</td><td>1301.492</td><td>-0.0005</td><td>-0.4</td><td>4.31</td><td>18647.7</td><td>9.51</td><td>HUMAN</td><td>Q14011</td><td>Cold-inducible RNA-binding protein</td></tr>
<tr><td>175</td><td>dirMS_041712_NAR_60mM_plus.18103.18432.3</td><td>3</td><td>21.78</td><td>1</td><td>1.39E+06</td><td></td><td>(K)SIYGEKF</td><td>(K)S I|Y/G/E/K/F/E/D/E N F/I/L/K(H)</td><td></td><td>152.97</td><td>611.3091</td><td>1831.911</td><td>0.0015</td><td>0.8</td><td>4.41</td><td>13378.8</td><td>6.27</td><td>HUMAN</td><td>C9J5S7</td><td>Peptidyl-prolyl cis-trans isomerase</td></tr>
<tr><td>176</td><td>dirMS_041712_NAR_30mM_plus2.16301.16715.2</td><td>2</td><td>21.77</td><td>0</td><td>1.64E+06</td><td></td><td>(R)FGLNVS</td><td>(R)F/G|L|N|V/S/S/I/S/R(K)</td><td></td><td>139.38</td><td>540.3016</td><td>1079.584</td><td>0.0115</td><td>10.7</td><td>9.75</td><td>24161.9</td><td>5.27</td><td>HUMAN</td><td>F8VZQ9</td><td>SAP domain-containing ribonucleoprotein</td></tr>
<tr><td>177</td><td>dirMS_041712_NAR_40mM_plus2.18667.18667.3</td><td>3</td><td>21.75</td><td>1</td><td>4.00E+05</td><td></td><td>(R)TLAEIAK</td><td>(R)T L|A/E/I/A/K V E/L D/N M/P L/R(G)</td><td></td><td>176.57</td><td>604.9967</td><td>1812.989</td><td>-0.0132</td><td>-7.3</td><td>4.68</td><td>23729.5</td><td>9.89</td><td>HUMAN</td><td>C9IZL7</td><td>Non-POU domain-containing octamer-binding protein (Fragment)</td></tr>
<tr><td>178</td><td>dirMS_041712_NAR_50mM_plus2.12284.12550.3</td><td>3</td><td>21.75</td><td>1</td><td>6.98E+06</td><td></td><td>(K)DVDEAY</td><td>(K)D V|D|E/A|Y/M/N/K V/E L E/S R(L)</td><td></td><td>133.37</td><td>599.9499</td><td>1797.832</td><td>0.0028</td><td>1.6</td><td>4.18</td><td>13588.1</td><td>4.66</td><td>HUMAN</td><td>F8VP67</td><td>Keratin, type II cytoskeletal 8 (Fragment)</td></tr>
<tr><td>179</td><td>dirMS_041712_NAR_30mM_minus2.15500.15543.2</td><td>2</td><td>21.73</td><td>0</td><td>3.51E+05</td><td></td><td>(K)LLYNNV</td><td>(K)L/L|Y|N|N|V|S/N/F/G R(L)</td><td></td><td>137.97</td><td>648.8355</td><td>1296.67</td><td>-0.0058</td><td>-4.5</td><td>8.75</td><td>71165.3</td><td>5.57</td><td>HUMAN</td><td>J3KS13</td><td>Clathrin heavy chain 1</td></tr>
<tr><td>180</td><td>dirMS_041712_NAR_60mM_minus.9133.9274.3</td><td>3</td><td>21.73</td><td>0</td><td>2.84E+05</td><td></td><td>(K)ATAGDT</td><td>(K)A/T|A/G/D/T/H/L/G G E D F D/N R(L)</td><td></td><td>111.4</td><td>559.2488</td><td>1675.731</td><td>0.0012</td><td>0.7</td><td>4.22</td><td>58502.6</td><td>6.09</td><td>HUMAN</td><td>B4DXY3</td><td>Heat shock 70 kDa protein 1-like</td></tr>
<tr><td>181</td><td>dirMS_041712_NAR_20mM_plus.18769.18769.2</td><td>2</td><td>21.73</td><td>0</td><td>2.87E+05</td><td></td><td>(K)LAVEALS</td><td>(K)L A|V/E\A/L|S/S L/D/G D L A/G R(Y)</td><td></td><td>200.48</td><td>793.9214</td><td>1586.838</td><td>-0.0029</td><td>-1.8</td><td>4.03</td><td>24286.7</td><td>4.89</td><td>HUMAN</td><td>G3V4N7</td><td>Creatine kinase B-type (Fragment)</td></tr>
<tr><td>182</td><td>dirMS_041712_NAR_60mM_plus.15550.15591.3</td><td>3</td><td>21.73</td><td>0</td><td>3.56E+05</td><td></td><td>(K)GVGISVI</td><td>(K)G V|G/I/S/V/L/E/M/S H/R(S)</td><td></td><td>137.95</td><td>428.8957</td><td>1284.673</td><td>-0.0003</td><td>-0.3</td><td>6.75</td><td>40821.3</td><td>7.79</td><td>HUMAN</td><td>Q9Y617</td><td>Phosphoserine aminotransferase</td></tr>
<tr><td>183</td><td>dirMS_041712_NAR_20mM_minus.10715.10715.2</td><td>2</td><td>21.72</td><td>0</td><td>1.92E+05</td><td></td><td>(R)TTPSYVA</td><td>(R)T T|P/S/Y\V/A/F T D/T E/R(L)</td><td></td><td>154.67</td><td>744.3547</td><td>1487.701</td><td>0.0009</td><td>0.6</td><td>4.37</td><td>58502.6</td><td>6.09</td><td>HUMAN</td><td>B4DXY3</td><td>Heat shock 70 kDa protein 1-like</td></tr>
<tr><td>184</td><td>dirMS_041712_NAR_50mM_plus2.20823.21133.3</td><td>3</td><td>21.72</td><td>0</td><td>4.90E+06</td><td></td><td>(R)AGKPVIC</td><td>(R)A G K\P V\I\C\A|T|Q|M/L/E/S M I/K(K)</td><td>C:Carbamidomethylation</td><td>179.65</td><td>626.3278</td><td>1876.969</td><td>-0.0005</td><td>-0.3</td><td>8.64</td><td>18466.1</td><td>5.78</td><td>HUMAN</td><td>H3BSU3</td><td>Pyruvate kinase (Fragment)</td></tr>
<tr><td>185</td><td>dirMS_041712_NAR_60mM_plus.11793.11822.3</td><td>3</td><td>21.72</td><td>1</td><td>3.83E+05</td><td></td><td>(R)LAEVEA</td><td>(R)L/A|E/V/E/A/A/L/E K Q/R(Q)</td><td></td><td>118.88</td><td>452.9164</td><td>1356.748</td><td>-0.0135</td><td>-10</td><td>4.79</td><td>533778</td><td>5.74</td><td>HUMAN</td><td>Q15149</td><td>Plectin</td></tr>
<tr><td>186</td><td>dirMS_041712_NAR_50mM_plus2.16401.16470.3</td><td>3</td><td>21.71</td><td>0</td><td>5.57E+05</td><td></td><td>(R)QVVQGI</td><td>(R)Q V|V/Q|G|L\L/S/E/T/Y/L|E A H R(I)</td><td></td><td>154.53</td><td>614.9984</td><td>1842.971</td><td>0.0098</td><td>5.3</td><td>5.4</td><td>81934.1</td><td>6.08</td><td>HUMAN</td><td>P33993</td><td>DNA replication licensing factor MCM7</td></tr>
<tr><td>187</td><td>dirMS_041712_NAR_30mM_plus2.17013.17033.2</td><td>2</td><td>21.7</td><td>0</td><td>5.59E+05</td><td>M256m</td><td>(R)SLDmDS</td><td>(R)S L|D/m|D/S/I/I|A/E/V/K(A)</td><td>m:Oxidized methionine</td><td>142.3</td><td>668.8384</td><td>1320.671</td><td>15.998</td><td>2.3</td><td>4.03</td><td>37296.3</td><td>5.3</td><td>HUMAN</td><td>F8VU64</td><td>Keratin, type II cytoskeletal 8</td></tr>
<tr><td>188</td><td>dirMS_041712_NAR_50mM_plus2.15772.15850.3</td><td>3</td><td>21.69</td><td>0</td><td>1.47E+06</td><td></td><td>(R)SLESLHS</td><td>(R)S L|E/S/L/H/S/F V/A A/A/T/K(E)</td><td></td><td>151.33</td><td>487.5964</td><td>1460.774</td><td>0.0003</td><td>0.2</td><td>6.47</td><td>81007.1</td><td>6.31</td><td>HUMAN</td><td>E9PMV1</td><td>Plectin (Fragment)</td></tr>
<tr><td>189</td><td>dirMS_041712_NAR_30mM_minus2.10630.10712.2</td><td>2</td><td>21.67</td><td>0</td><td>4.29E+05</td><td></td><td>(R)TLIQNCC</td><td>(R)T L|I|Q|N/C|G A/S/T I/R(L)</td><td>C:Carbamidomethylation</td><td>111.82</td><td>667.348</td><td>1333.689</td><td>-0.0005</td><td>-0.4</td><td>9.41</td><td>56186.7</td><td>5.47</td><td>HUMAN</td><td>B4DUR8</td><td>T-complex protein 1 subunit gamma</td></tr>
<tr><td>190</td><td>dirMS_041712_NAR_40mM_minus2.14585.14585.3</td><td>3</td><td>21.67</td><td>1</td><td>1.39E+06</td><td>M116m</td><td>(R)ELAILLG</td><td>(R)E L|A\I/L/L/G m/L D/P A E K D E K(G)</td><td>m:Oxidized methionine</td><td>171.13</td><td>634.338</td><td>1884.999</td><td>16.0008</td><td>3.1</td><td>4.18</td><td>25148.6</td><td>6</td><td>HUMAN</td><td>P30041</td><td>Peroxiredoxin-6</td></tr>
<tr><td>191</td><td>dirMS_041712_NAR_60mM_minus.12175.12219.3</td><td>3</td><td>21.67</td><td>0</td><td>7.53E+05</td><td></td><td>(K)QADLYIS</td><td>(K)Q A/D\L/Y/I/S/E/G L/H/P R(I)</td><td></td><td>128.07</td><td>500.2603</td><td>1498.765</td><td>0.0015</td><td>1</td><td>5.32</td><td>55323</td><td>6.02</td><td>HUMAN</td><td>B4DPJ8</td><td>T-complex protein 1 subunit zeta</td></tr>
<tr><td>192</td><td>dirMS_041712_NAR_20mM_minus.6161.6256.2</td><td>2</td><td>21.66</td><td>0</td><td>3.80E+06</td><td></td><td>(K)DSYVGD</td><td>(K)D S|Y|V|G/D/E/A/Q|S K(R)</td><td></td><td>103.87</td><td>599.7642</td><td>1198.522</td><td>-0.0011</td><td>-0.9</td><td>4.03</td><td>32616.5</td><td>5.18</td><td>HUMAN</td><td>A6NL76</td><td>Actin, alpha skeletal muscle</td></tr>
<tr><td>193</td><td>dirMS_041712_NAR_30mM_plus2.20464.20854.2</td><td>2</td><td>21.65</td><td>0</td><td>8.74E+05</td><td></td><td>(R)TVQSLEI</td><td>(R)T/V|Q|S|L|E|I\D/L/D/S M R(N)</td><td></td><td>162.18</td><td>753.8768</td><td>1506.747</td><td>-0.0005</td><td>-0.3</td><td>4.03</td><td>43773.3</td><td>5.26</td><td>HUMAN</td><td>F8VZY9</td><td>Keratin, type I cytoskeletal 18</td></tr>
<tr><td>194</td><td>dirMS_041712_NAR_50mM_plus2.11559.11841.3</td><td>3</td><td>21.65</td><td>1</td><td>1.68E+06</td><td></td><td>(R)ISVYYNE</td><td>(R)I/S|V/Y/Y/N/E\A/T/G G\K/Y V/P R(A)</td><td></td><td>128.42</td><td>606.3123</td><td>1816.923</td><td>-0.0005</td><td>-0.3</td><td>8.43</td><td>27549.9</td><td>4.39</td><td>HUMAN</td><td>E9PBJ4</td><td>Tubulin beta chain</td></tr>
<tr><td>195</td><td>dirMS_041712_NAR_60mM_minus.5537.5919.3</td><td>3</td><td>21.64</td><td>1</td><td>4.91E+07</td><td></td><td>(K)DSYVGD</td><td>(K)D S|Y|V/G/D/E/A/Q/S K R(G)</td><td></td><td>87.95</td><td>452.2129</td><td>1354.623</td><td>0.0008</td><td>0.6</td><td>4.56</td><td>32616.5</td><td>5.18</td><td>HUMAN</td><td>A6NL76</td><td>Actin, alpha skeletal muscle</td></tr>
<tr><td>196</td><td>dirMS_041712_NAR_40mM_plus2.12274.12516.3</td><td>3</td><td>21.64</td><td>0</td><td>3.98E+05</td><td></td><td>(K)AASDIAN</td><td>(K)A A|S D/I/A/M/T E/L/P/P T H/P I R(L)</td><td></td><td>139.83</td><td>607.3167</td><td>1819.937</td><td>-0.0015</td><td>-0.9</td><td>5.32</td><td>12926.1</td><td>4.4</td><td>HUMAN</td><td>K7EM20</td><td>14-3-3 protein epsilon (Fragment)</td></tr>
<tr><td>197</td><td>dirMS_041712_NAR_30mM_minus2.14981.14981.2</td><td>2</td><td>21.61</td><td>0</td><td>5.54E+05</td><td>M316m</td><td>(R)ISEQFTA</td><td>(R)I/S/E/Q/F/T/A m/F R(R)</td><td>m:Oxidized methionine</td><td>135.18</td><td>623.3014</td><td>1229.598</td><td>15.9972</td><td>1.8</td><td>6</td><td>42831.5</td><td>4.86</td><td>HUMAN</td><td>A8K854</td><td>HCG1983504, isoform CRA_f</td></tr>
<tr><td>198</td><td>dirMS_041712_NAR_50mM_minus2.13028.13255.3</td><td>3</td><td>21.61</td><td>1</td><td>7.72E+05</td><td></td><td>(K)VLAGDK</td><td>(K)V/L|A/G D K/N/F|I|T/A E/E/L R(R)</td><td></td><td>142.88</td><td>559.3044</td><td>1675.901</td><td>-0.0027</td><td>-1.6</td><td>4.68</td><td>80254.2</td><td>5.05</td><td>HUMAN</td><td>D6PXK4</td><td>Alpha actinin 4 short isoform</td></tr>
<tr><td>199</td><td>dirMS_041712_NAR_20mM_minus.8912.8965.2</td><td>2</td><td>21.6</td><td>0</td><td>1.86E+06</td><td></td><td>(K)LVSESSD</td><td>(K)L/V|S|E/S/S D V/L/P/K(-)</td><td></td><td>139.67</td><td>587.321</td><td>1173.636</td><td>-0.0014</td><td>-1.2</td><td>4.37</td><td>56607.4</td><td>5.37</td><td>HUMAN</td><td>F8VXB4</td><td>Keratin, type II cytoskeletal 8</td></tr>
<tr><td>200</td><td>dirMS_041712_NAR_30mM_plus2.16201.16467.2</td><td>2</td><td>21.59</td><td>0</td><td>8.03E+05</td><td></td><td>(R)GLCAIAC</td><td>(R)G L/C|A|I|A|Q|A/E|S L|R(Y)</td><td>C:Carbamidomethylation</td><td>138.63</td><td>644.8372</td><td>1288.668</td><td>-0.0007</td><td>-0.5</td><td>6</td><td>18529.2</td><td>9.98</td><td>HUMAN</td><td>E9PL45</td><td>40S ribosomal protein S3</td></tr>
<tr><td>201</td><td>dirMS_041712_NAR_30mM_minus2.18473.18796.2</td><td>2</td><td>21.54</td><td>0</td><td>8.57E+05</td><td></td><td>(K)LLQDFFN</td><td>(K)L/L|Q|D/F|F/N/G/K(E)</td><td></td><td>156.12</td><td>541.2823</td><td>1081.568</td><td>-0.0103</td><td>-9.6</td><td>5.84</td><td>45508.6</td><td>5.5</td><td>HUMAN</td><td>A8K7Q2</td><td>Heat shock cognate 71 kDa protein</td></tr>
<tr><td>202</td><td>dirMS_041712_NAR_30mM_plus2.11417.11811.2</td><td>2</td><td>21.54</td><td>0</td><td>3.02E+06</td><td></td><td>(R)INVYYNE</td><td>(R)I/N|V|Y|Y|N/E/A/T/G G K(Y)</td><td></td><td>114.07</td><td>664.8272</td><td>1328.648</td><td>-0.001</td><td>-0.7</td><td>6</td><td>50286.6</td><td>4.79</td><td>HUMAN</td><td>P68371</td><td>Tubulin beta-4B chain</td></tr>
<tr><td>203</td><td>dirMS_041712_NAR_50mM_plus2.13286.13286.3</td><td>3</td><td>21.52</td><td>0</td><td>2.96E+06</td><td></td><td>(K)LGDVYV</td><td>(K)L/G|D\V/Y V/N/D A/F G/T A H/R(A)</td><td></td><td>138.15</td><td>545.6029</td><td>1634.792</td><td>0.002</td><td>1.2</td><td>5.21</td><td>41827.5</td><td>7</td><td>HUMAN</td><td>B7Z7A9</td><td>Phosphoglycerate kinase</td></tr>
<tr><td>204</td><td>dirMS_041712_NAR_60mM_plus.9934.10245.3</td><td>3</td><td>21.51</td><td>0</td><td>1.00E+07</td><td></td><td>(R)LESGMC</td><td>(R)L/E|S G/M|Q/N/M/S/I/H/T K(T)</td><td></td><td>110.2</td><td>492.5712</td><td>1475.698</td><td>0.0009</td><td>0.6</td><td>6.75</td><td>56607.4</td><td>5.37</td><td>HUMAN</td><td>F8VXB4</td><td>Keratin, type II cytoskeletal 8</td></tr>
<tr><td>205</td><td>dirMS_041712_NAR_60mM_minus.15664.15891.3</td><td>3</td><td>21.49</td><td>1</td><td>4.07E+05</td><td></td><td>(R)AEGAIAC</td><td>(R)A/E|G|A|I/A/G F\R D/P F/S/G/K(Q)</td><td></td><td>147.98</td><td>508.2601</td><td>1522.765</td><td>0.0009</td><td>0.6</td><td>6.12</td><td>266161</td><td>5.68</td><td>HUMAN</td><td>E9PPU0</td><td>Epiplakin</td></tr>
<tr><td>206</td><td>dirMS_041712_NAR_60mM_minus.6037.6305.3</td><td>3</td><td>21.46</td><td>1</td><td>2.51E+06</td><td></td><td>(K)LEEAEKA</td><td>(K)L/E|E|A/E/K/A/A/D/E/S E R(G)</td><td></td><td>93.27</td><td>492.8981</td><td>1476.681</td><td>-0.0015</td><td>-1</td><td>4.14</td><td>31923.6</td><td>4.85</td><td>HUMAN</td><td>B7Z596</td><td>Tropomyosin alpha-1 chain</td></tr>
<tr><td>207</td><td>dirMS_041712_NAR_50mM_plus2.10120.10258.3</td><td>3</td><td>21.45</td><td>0</td><td>6.15E+05</td><td></td><td>(K)VGQEIE\</td><td>(K)V/G|Q|E/I E/V/R P G/I V/S K(D)</td><td></td><td>121.05</td><td>504.2907</td><td>1510.859</td><td>-0.0012</td><td>-0.8</td><td>6.11</td><td>51679.1</td><td>9.11</td><td>HUMAN</td><td>P41091</td><td>Eukaryotic translation initiation factor 2 subunit 3</td></tr>
<tr><td>208</td><td>dirMS_041712_NAR_50mM_minus2.14690.14916.3</td><td>3</td><td>21.44</td><td>1</td><td>9.62E+05</td><td></td><td>(R)IINEPTA.</td><td>(R)I/I|N|E/P T A/A/A I/A/Y/G L D/K/R(E)</td><td></td><td>152.6</td><td>606.0022</td><td>1815.996</td><td>-0.0043</td><td>-2.4</td><td>6.07</td><td>72445.7</td><td>5.07</td><td>HUMAN</td><td>P11021</td><td>78 kDa glucose-regulated protein</td></tr>
<tr><td>209</td><td>dirMS_041712_NAR_60mM_plus.11253.11253.3</td><td>3</td><td>21.44</td><td>0</td><td>1.93E+06</td><td></td><td>(R)EIVHIQA</td><td>(R)E/I/V H I Q A G/Q/C/G N Q I/G A/K(F)</td><td>C:Carbamidomethylation</td><td>116.42</td><td>608.311</td><td>1822.923</td><td>-0.0044</td><td>-2.4</td><td>6.85</td><td>40069.4</td><td>4.83</td><td>HUMAN</td><td>E7EWR1</td><td>Tubulin beta chain</td></tr>
<tr><td>210</td><td>dirMS_041712_NAR_30mM_plus2.16217.16712.2</td><td>2</td><td>21.43</td><td>0</td><td>5.57E+06</td><td></td><td>(K)YMACCL</td><td>(K)Y/M|A|C/C/L|L|Y/R(G)</td><td>C:Carbamidomethylation</td><td>139.3</td><td>625.2798</td><td>1249.553</td><td>-0.0003</td><td>-0.2</td><td>8.59</td><td>50706.6</td><td>5.68</td><td>HUMAN</td><td>A6NHL2</td><td>Tubulin alpha chain-like 3</td></tr>
<tr><td>211</td><td>dirMS_041712_NAR_30mM_plus2.6742.6801.2</td><td>2</td><td>21.4</td><td>0</td><td>2.92E+05</td><td></td><td>(R)EAGEQG</td><td>(R)E A|G\E/Q|G/D/I/E/P R(R)</td><td></td><td>89.77</td><td>600.7772</td><td>1200.549</td><td>-0.002</td><td>-1.7</td><td>4</td><td>31450.7</td><td>5.73</td><td>HUMAN</td><td>F5H4D6</td><td>Ras GTPase-activating protein-binding protein 1</td></tr>
<tr><td>212</td><td>dirMS_041712_NAR_30mM_plus2.6145.6152.2</td><td>2</td><td>21.39</td><td>0</td><td>1.41E+05</td><td></td><td>(R)CSQAVY</td><td>(R)C/S|Q|A|V/Y/A/A E/K(V)</td><td>C:Carbamidomethylation</td><td>86.47</td><td>563.7624</td><td>1126.52</td><td>-0.0022</td><td>-2</td><td>6</td><td>16685.6</td><td>9.7</td><td>HUMAN</td><td>E9PND2</td><td>Cysteine and glycine-rich protein 1 (Fragment)</td></tr>
<tr><td>213</td><td>dirMS_041712_NAR_50mM_plus2.16479.16479.3</td><td>3</td><td>21.37</td><td>1</td><td>2.02E+06</td><td></td><td>(K)QGGLGF</td><td>(K)Q G/G/L/G P M N I/P/L/V S/D/P K R(T)</td><td></td><td>154.68</td><td>593.6536</td><td>1778.958</td><td>-0.0119</td><td>-6.7</td><td>8.75</td><td>22338.2</td><td>8.65</td><td>HUMAN</td><td>Q06830</td><td>Peroxiredoxin-1</td></tr>
</table>

214	dirMS_041712_NAR_50mM_plus2.19552.19846.3	3	21.36	1	5.91E+05		(R)VLEALLP	(R)V/L|E/A/L L/P L/K/G L E/E/R(V)		172.03	527.318	1579.942	-0.0023	-1.5	4.79	276046.1	6.01	HUMAN	P49327	Fatty acid synthase
215	dirMS_041712_NAR_60mM_plus.6783.6783.3	3	21.36	0	1.82E+06		(K)CIGKPG(	(K)C I|G/K/P G G\S L/D N/S E Q K(C)	C:Carbamidomethylation	94.07	530.5904	1589.759	-0.0021	-1.4	6.07	9122.2	9.22	HUMAN	K7EIT2	Mitochondrial import inner membrane translocase subunit Tim13
216	dirMS_041712_NAR_30mM_minus2.16618.16618.2	2	21.33	0	2.83E+05		(R)ASLEAAI	(R)A S|L/E|A/A|I/A/D A E Q R(G)		143.92	672.8404	1344.675	-0.0019	-1.4	4.14	56607.4	5.37	HUMAN	F8VXB4	Keratin, type II cytoskeletal 8
217	dirMS_041712_NAR_50mM_minus2.5658.5671.3	3	21.33	1	3.66E+05		(K)DSYVGD	(K)D S|Y|V/G/D/E/A/Q/S K R(G)		92.95	452.2119	1354.623	-0.0022	-1.6	4.56	32616.5	5.18	HUMAN	A6NL76	Actin, alpha skeletal muscle
218	dirMS_041712_NAR_40mM_plus2.13719.14138.3	3	21.33	1	3.53E+06		(R)IINEPTA	(R)I/I|N|E/P T A/A/A/I/A/Y G L D/K K(V)		148.57	596.6671	1787.99	-0.0034	-1.9	6.07	17973	5.16	HUMAN	E9PI65	Heat shock cognate 71 kDa protein (Fragment)
219	dirMS_041712_NAR_30mM_plus2.6644.6644.2	2	21.32	0	2.92E+05		(R)EAGEQG	(R)E A G E/Q/G/D I/E/P/R(R)		89.77	600.7772	1200.549	-0.002	-1.7	4	31450.7	5.73	HUMAN	F5H4D6	Ras GTPase-activating protein-binding protein 1
220	dirMS_041712_NAR_20mM_minus.13467.13467.2	2	21.31	0	6.67E+05		(K)DAGTIA(	(K)D/A\G|T/I|A/G/L N/V/L R(I)		184.97	600.3403	1199.674	-0.0009	-0.8	5.84	17973	5.16	HUMAN	E9PI65	Heat shock cognate 71 kDa protein (Fragment)
221	dirMS_041712_NAR_30mM_minus2.7087.7087.2	2	21.31	0	2.76E+05		(R)CGCLDE	(R)C/G\C|L|D/E/D T Q R(Q)	C:Carbamidomethylation	93.07	627.2485	1253.488	0.0012	1	4.03	266161	5.68	HUMAN	E9PPU0	Epiplakin
222	dirMS_041712_NAR_40mM_minus2.13969.14030.3	3	21.31	0	3.46E+05		(R)LLEVLSG	(R)L/L\E|V/L/S/G E|I L/P K/P T K(G)		166.62	546.333	1636.988	-0.0039	-2.4	6.14	13611	6.13	HUMAN	E9PJZ2	Spectrin beta chain, non-erythrocytic 2 (Fragment)
223	dirMS_041712_NAR_30mM_plus2.6064.6064.2	2	21.31	0	8.14E+05		(R)NQGGY(	(R)N|Q|G G Y|G/G/S/S/S S S/S Y/G S G R(R)		86.18	847.8524	1694.7	-0.0026	-1.5	8.59	33268.7	9.31	HUMAN	F8VRQ1	Heterogeneous nuclear ribonucleoprotein A1
224	dirMS_041712_NAR_50mM_plus2.16023.16294.3	3	21.31	1	1.20E+06		(K)CEFQDA	(K)C/E|F/Q D\A|Y|V|L/L/S E/K K(I)	C:Carbamidomethylation	152.32	577.2866	1729.847	-0.0013	-0.8	4.68	61224.7	5.7	HUMAN	P10809	60 kDa heat shock protein, mitochondrial
225	dirMS_041712_NAR_30mM_plus2.6045.6045.2	2	21.3	0	1.50E+05		(R)VGSTSEI	(R)V/G/S|T/S/E/N I/T Q/K(V)		85.97	582.2983	1163.59	-0.0009	-0.8	5.97	73386.6	8.73	HUMAN	B4DXX7	ATP-dependent RNA helicase DDX3Y
226	dirMS_041712_NAR_30mM_minus2.8734.8997.2	2	21.29	0	1.42E+06		(K)SNVSDA	(K)S/N|V|S|D/A|V|A|Q/S T R(I)		102.9	617.8045	1234.602	-0.0005	-0.4	5.55	31075.7	5.65	HUMAN	P60174	Triosephosphate isomerase
227	dirMS_041712_NAR_60mM_minus.10546.10745.3	3	21.29	1	1.64E+06		(K)TLQEVTI	(K)T L|Q/E/V/T/E/M/D/S V K/R(Y)		119.37	512.5957	1535.773	-0.0008	-0.5	4.68	558007.1	5.44	HUMAN	P58107	Epiplakin
228	dirMS_041712_NAR_30mM_plus2.18229.18506.2	2	21.29	0	5.45E+05		(R)GYISPYF	(R)G Y|I|S|P/Y/F/I/N T S K(G)		149.72	695.3554	1389.705	-0.0014	-1	8.5	25225.7	9.52	HUMAN	E7ESH4	60 kDa heat shock protein, mitochondrial (Fragment)
229	dirMS_041712_NAR_50mM_plus2.9823.9863.3	3	21.29	1	2.67E+03		(R)TAGVKV	(R)T|A|G/V/K/V/E T/T/E/D L V/A K(L)		120.5	520.9473	1560.848	-0.0206	-13.2	4.68	28071.3	8.6	HUMAN	P38117	Electron transfer flavoprotein subunit beta
230	dirMS_041712_NAR_50mM_plus2.17668.17805.3	3	21.27	1	9.79E+05		(R)SELELTL(	(R)S/E|L/E/L/T/L/G/K L/E Q V/R(S)		161.43	538.9727	1614.906	-0.0026	-1.6	4.78	533778	5.74	HUMAN	Q15149	Plectin
231	dirMS_041712_NAR_50mM_minus2.15638.15905.3	3	21.25	1	6.66E+05		(K)SIYGEKF	(K)S I|Y/G E/K/F/E/D/E/N F/I/L/K(H)		158	611.3086	1831.911	0	0	4.41	13378.8	6.27	HUMAN	C9J5S7	Peptidyl-prolyl cis-trans isomerase
232	dirMS_041712_NAR_30mM_plus2.17547.17801.2	2	21.25	0	5.16E+05		(K)LLGELLG	(K)L/L|G|E|L|L|Q/D/N A K(L)		145.75	607.3423	1213.679	-0.0014	-1.1	4.37	35556.4	9.3	HUMAN	F2Z393	Transaldolase
233	dirMS_041712_NAR_40mM_plus2.12376.12376.3	3	21.25	0	3.04E+05		(K)AASDIAI	(K)A A S D|I/A/M/T/E L/P/P T H P I R(L)		139.47	607.3167	1819.937	-0.0015	-0.9	5.32	12926.1	4.4	HUMAN	K7EM20	14-3-3 protein epsilon (Fragment)
234	dirMS_041712_NAR_50mM_plus2.6416.6650.3	3	21.25	1	6.47E+05		(R)IVDGKV	(R)I/V|D/G K/V/V/S/E/T N D/T/K(V)		98.57	502.2651	1504.785	-0.0046	-3	4.56	48057	5.34	HUMAN	P05783	Keratin, type I cytoskeletal 18
235	dirMS_041712_NAR_40mM_plus2.17359.17812.3	3	21.24	1	3.01E+06		(R)LFVGNLI	(R)L/F|V/G/N/L/P A D/I/T E/D E F K R(L)		170.08	655.3421	1964.012	-0.0006	-0.3	4.32	76262.2	9.53	HUMAN	P23246	Splicing factor, proline- and glutamine-rich
236	dirMS_041712_NAR_60mM_plus.8502.8943.3	3	21.24	1	4.46E+06		(R)VVASDK	(R)V/V|A/S/D/K/E/T/Y/E/L/R(Y)		103.67	470.5798	1409.727	-0.0022	-1.6	4.68	14478.1	9.37	HUMAN	C9JYQ9	60S ribosomal protein L22-like 1
237	dirMS_041712_NAR_40mM_plus2.19252.19362.3	3	21.22	0	1.61E+06		(R)AGKPVI(	(R)A G K\P V\I\C\A|T|Q|M/L/E/S M I/K(K)	C:Carbamidomethylation	180.63	626.3273	1876.969	-0.002	-1.1	8.64	18466.1	5.78	HUMAN	H3BSU3	Pyruvate kinase (Fragment)
238	dirMS_041712_NAR_20mM_plus.12569.12768.2	2	21.21	0	9.33E+05	M92m	(R)ImNTFS	(R)I/m|N|T|F/S/V/V|P/S/P K(V)	m:Oxidized methionine	160.22	668.3516	1319.703	15.9931	-1.3	8.75	42831.5	4.86	HUMAN	A8K854	HCG1983504, isoform CRA_f
239	dirMS_041712_NAR_20mM_plus.18580.18580.2	2	21.18	0	1.75E+05	M332m	(R)IVSQLLT	(R)I V|S Q\L/L/T L/m D G L K(Q)	m:Oxidized methionine	199.38	723.9145	1430.829	15.993	-1.3	5.84	90004.9	5.14	HUMAN	P55072	Transitional endoplasmic reticulum ATPase
240	dirMS_041712_NAR_50mM_minus2.14497.14497.3	3	21.16	1	7.89E+05		(K)EAFSLFD	(K)E A/F/S/L/F/D K/D G D/G T I T T K(E)		150.35	615.6336	1844.891	-0.005	-2.7	4.23	21860.1	4.45	HUMAN	E7EMB3	Calmodulin
241	dirMS_041712_NAR_30mM_plus2.18668.18824.2	2	21.16	0	3.77E+05		(R)LLLNNDI	(R)L/L|L|N|N/D/N L/L/R(E)		151.37	599.3506	1197.695	-0.0011	-0.9	5.84	19945.8	4.97	HUMAN	B4DG50	F-actin-capping protein subunit alpha-2
242	dirMS_041712_NAR_60mM_plus.10321.10826.3	3	21.16	1	3.53E+07		(R)LQAEIEG	(R)L/Q|A/E/I/E/G L/K/G Q/R(A)		112.38	447.9186	1341.748	-0.0072	-5.4	6.14	56607.4	5.37	HUMAN	F8VXB4	Keratin, type II cytoskeletal 8
243	dirMS_041712_NAR_50mM_minus2.15087.15397.3	3	21.15	0	9.01E+05		(R)LLIHQSL	(R)L L|I/H/Q\S\L|A|G G I/I/G V/K(G)		154.83	506.9833	1518.937	-0.0013	-0.8	8.76	51260.6	5.39	HUMAN	P61978	Heterogeneous nuclear ribonucleoprotein K
244	dirMS_041712_NAR_60mM_plus.11699.11883.3	3	21.15	0	4.34E+06		(K)TGNLGN	(K)T/G|N|L/G N V/V/H/I/E/R(F)		119.1	436.9042	1308.702	-0.0038	-2.9	6.41	14478.1	9.37	HUMAN	C9JYQ9	60S ribosomal protein L22-like 1
245	dirMS_041712_NAR_30mM_plus2.15362.15362.2	2	21.11	0	3.86E+05		(R)MAGAIA	(R)M/A/G\A/I/A/Q G L/I/R(A)		133.18	550.816	1100.624	0.0002	0.2	9.5	4449.1	11.42	HUMAN	F8WEI0	Pyrroline-5-carboxylate reductase 3
246	dirMS_041712_NAR_60mM_plus.5551.5673.3	3	21.1	0	1.11E+06		(K)TATPQQ	(K)T A|T/P Q/Q/A/Q/E/V/H E K(L)		87.6	489.5727	1466.723	-0.0198	-13.5	5.37	31075.7	5.65	HUMAN	P60174	Triosephosphate isomerase
247	dirMS_041712_NAR_120mM_minus.11767.11847.3	3	21.08	0	6.27E+05		(K)FYEEVHI	(K)F/Y/E/E/V/H/D/L E R(K)		118.58	446.2102	1336.617	-0.0008	-0.6	4.4	18209.2	5.03	HUMAN	A8MXH2	Nucleosome assembly protein 1-like 4 (Fragment)
248	dirMS_041712_NAR_120mM_minus.7402.7935.3	3	21.08	1	8.15E+05		(K)IQDKEGI	(K)I Q|D K E G|I|P/P/D/Q Q/R(L)		94.9	508.5981	1523.781	-0.0015	-1	4.56	17271.6	6.75	HUMAN	B4DV12	Ubiquitin
249	dirMS_041712_NAR_50mM_minus2.7228.7330.3	3	21.08	0	1.02E+06		(K)QEYDES	(K)Q E/Y/D E/S/G/P/S/I/V/H/R(K)		108.38	506.2404	1516.703	0.004	2.6	4.65	123097.1	5.83	HUMAN	A5A3E0	POTE ankyrin domain family member F
250	dirMS_041712_NAR_50mM_minus2.7702.7745.3	3	21.08	0	2.46E+05		(R)AALAHS	(R)A/A\L A H S/E\E\V|T|A/S/Q/V/A|A\T K(T)		110.93	595.3104	1783.919	-0.0018	-1	5.4	23903	4.94	HUMAN	H0YDN1	Plectin (Fragment)
251	dirMS_041712_NAR_50mM_minus2.10265.10432.3	3	21.07	1	5.22E+05		(K)IAQLEEC	(K)I/A|Q|L/E/E/Q/L/D/N E/T K E R(Q)		127.12	605.9737	1815.908	-0.0018	-1	4.25	227783.5	5.5	HUMAN	P35579	Myosin-9
252	dirMS_041712_NAR_60mM_plus.20478.20766.3	3	21.07	1	3.50E+06		(R)GLFIIDD	(R)G L|F|I/I/D/D/K/G I L/R(Q)		168.3	453.9368	1359.8	-0.0036	-2.7	5.96	18498.6	5.82	HUMAN	H7C3T4	Peroxiredoxin-4 (Fragment)
253	dirMS_041712_NAR_40mM_minus2.8174.8349.3	3	21.06	1	5.29E+05		(R)DVVICPI	(R)D V|V|I/C/P D/A S L E D/A K K(E)	C:Carbamidomethylation	129.32	553.9467	1659.826	-0.0003	-0.2	4.23	18080.7	7.92	HUMAN	K7ELW0	Protein DJ-1
254	dirMS_041712_NAR_30mM_plus2.12277.12277.2	2	21.06	0	2.21E+05		(K)VLQATV	(K)V L|Q/A|T/V|V/A/V/G S G S K(G)		117.57	658.3806	1315.758	-0.0041	-3.1	8.72	10803.5	9.82	HUMAN	B8ZZL8	10 kDa heat shock protein, mitochondrial
255	dirMS_041712_NAR_40mM_plus2.11300.11372.3	3	21.06	0	1.67E+05		(R)SVIDPVF	(R)S V|I|D|P/V/P A/P V G D S H V D G A A K(S)		132.95	644.3327	1930.987	-0.0033	-1.7	4.41	58326.8	5.7	HUMAN	Q13177	Serine/threonine-protein kinase PAK 2
256	dirMS_041712_NAR_20mM_plus.12303.12303.2	2	21.05	0	1.17E+05		(K)VNQIGS	(K)V N/Q|I|G/S/V/T E/S I/Q A C K(L)	C:Carbamidomethylation	157.67	817.4114	1633.821	-0.0059	-3.6	5.97	47328.4	7.76	HUMAN	P13929	Beta-enolase
257	dirMS_041712_NAR_30mM_plus2.18461.18461.2	2	21.05	0	2.03E+05		(R)AVLNPL(	(R)A V|L|N/P\L/C/Q V D/Y R(A)	C:Carbamidomethylation	150.18	724.3703	1447.736	-0.0029	-2	5.88	83880.6	7.33	HUMAN	F5H365	Protein transport protein Sec23A
258	dirMS_041712_NAR_50mM_plus2.18585.19063.3	3	21.03	1	6.37E+06		(R)GLFIIDD	(R)G L|F|I/I/D/D/K/G I L/R(Q)		167.33	453.9379	1359.8	-0.0003	-0.2	5.96	18498.6	5.82	HUMAN	H7C3T4	Peroxiredoxin-4 (Fragment)
259	dirMS_041712_NAR_120mM_plus.13426.13492.3	3	21.02	0	1.03E+06		(K)THINIVV	(K)T H|I|N\I\V|V|I/G/H V/D|S G K(S)		123.78	530.2975	1588.881	-0.0026	-1.6	6.61	12137.8	9.3	HUMAN	A6PW80	Elongation factor 1-alpha 1 (Fragment)
260	dirMS_041712_NAR_20mM_plus.16540.16540.2	2	21.02	0	1.75E+05		(R)LLEAQIA	(R)L L|E|A\Q/I|A/T/G G I I/D|P K(E)		185.47	769.9362	1538.879	-0.0137	-8.9	4.37	334221.3	6.44	HUMAN	P15924	Desmoplakin
261	dirMS_041712_NAR_60mM_minus.14195.14255.3	3	21.01	1	8.82E+05		(R)SILKIDD	(R)S I|L/K/I/D/D|V|V/N T R(-)		139.22	458.2637	1372.78	-0.0029	-2.1	5.68	42755	7.81	HUMAN	B7Z2F4	T-complex protein 1 subunit delta
262	dirMS_041712_NAR_40mM_plus2.13909.14086.3	3	21.01	0	3.53E+05		(K)LVEALC	(K)L/V|E/A/L/C/A/E/H Q I N L/I/K(V)	C:Carbamidomethylation	148.62	584.3205	1750.952	-0.0051	-2.9	5.4	14914.1	6.84	HUMAN	P25398	40S ribosomal protein S12
263	dirMS_041712_NAR_120mM_plus.19984.20517.4	4	21	1	3.55E+07		(K)AAIDWF	(K)A A|I/D/W/F/D/G K E/F H/G N I I/K(V)		159.08	491.0027	1960.992	-0.0026	-1.3	5.38	49181	4.93	HUMAN	K7EPT6	TATA-binding protein-associated factor 2N
264	dirMS_041712_NAR_20mM_plus.10018.10215.2	2	21	0	1.43E+06		(K)LVSESSD	(K)L/V|S|E|S/S/D\V/L/P/K(-)		143.17	587.3192	1173.636	-0.005	-4.3	4.37	56607.4	5.37	HUMAN	F8VXB4	Keratin, type II cytoskeletal 8
265	dirMS_041712_NAR_20mM_plus.15583.15583.2	2	20.99	0	2.37E+05		(K)TITLEVEI	(K)T I/T|L E|V/E|P S D T I E N V K(A)		179.03	894.4658	1787.927	-0.003	-1.7	4	17271.6	6.75	HUMAN	B4DV12	Ubiquitin
266	dirMS_041712_NAR_30mM_plus2.18710.18886.2	2	20.99	0	5.47E+05		(R)NFWVS(	(R)N F|W|V|S|G/L/S/S/T T R(A)		151.8	677.8411	1354.675	-0.0001	0	9.75	103309.5	5.34	HUMAN	A0AV56	SAFB protein
267	dirMS_041712_NAR_50mM_plus2.9415.9796.3	3	20.99	1	2.21E+06		(K)ALIEMEI	(K)A L|I|E/M/E/K/Q/Q/Q/D/Q V D/R(N)		117.33	610.9732	1830.901	0.0036	2	4.32	29678.2	9.57	HUMAN	C9JYS8	Non-POU domain-containing octamer-binding protein (Fragment)
268	dirMS_041712_NAR_50mM_plus2.14479.14757.3	3	20.96	1	3.88E+06		(K)VGEVIVT	(K)V/G|E|V/I/V|T K/D D A M L/L/K(G)		145.3	544.3052	1630.908	-0.0074	-4.5	4.56	61224.7	5.7	HUMAN	P10809	60 kDa heat shock protein, mitochondrial
269	dirMS_041712_NAR_50mM_minus2.15141.15549.3	3	20.95	1	8.73E+05		(K)ILLAELE(	(K)I/L|L/A/E/L/E Q/L/K/G Q/G K(S)		155.78	513.9739	1539.911	-0.0033	-2.2	6.14	49709.6	5.19	HUMAN	B0YJC4	Vimentin
270	dirMS_041712_NAR_60mM_minus.8538.8538.3	3	20.95	0	9.13E+04		(R)ISVYYNE	(R)I/S V/Y/Y N E A/S S/H K(Y)		107.72	466.5604	1397.67	-0.0029	-2.1	6.75	18402.2	5.06	HUMAN	G3V2N6	HCG1983504, isoform CRA_d
271	dirMS_041712_NAR_20mM_minus.12096.12461.2	2	20.94	0	9.01E+05	M185m	(K)LAVNm\	(K)L/A|V|N/m|V|P/F/P R(L)	m:Oxidized methionine	171.88	580.3173	1143.634	15.993	-1.6	9.75	42230.8	4.77	HUMAN	A6NKZ8	Putative tubulin beta chain-like protein ENSP00000290377
272	dirMS_041712_NAR_30mM_minus2.16134.16406.2	2	20.94	0	4.42E+05	M185m	(K)LAVNm\	(K)L/A|V/N|m|V|P F/P R(L)	m:Oxidized methionine	142.58	580.3178	1143.634	15.994	-0.8	9.75	42230.8	4.77	HUMAN	A6NKZ8	Putative tubulin beta chain-like protein ENSP00000290377
273	dirMS_041712_NAR_20mM_plus.9211.9211.2	2	20.94	0	4.54E+04		(R)IIYGGSV	(R)I/I|Y/G/G S\V T G A T/C K(E)	C:Carbamidomethylation	135.4	663.8412	1326.672	0.0029	2.2	8.59	31075.7	5.65	HUMAN	P60174	Triosephosphate isomerase
274	dirMS_041712_NAR_50mM_plus2.17433.17690.3	3	20.94	0	9.60E+05		(R)VLDPFTI	(R)V/L|D|P/F/T/I/K/P L D/R(K)		160.52	471.9408	1413.81	-0.0022	-1.5	5.93	63448.4	7.29	HUMAN	B4E022	Transketolase
275	dirMS_041712_NAR_50mM_plus2.17808.18100.3	3	20.94	1	1.43E+06		(K)AILILDNI	(K)A I|L|I|L/D/N D G D/R L F/A/K(Y)		162.65	558.6425	1673.922	-0.0091	-5.5	4.43	18430.9	4.79	HUMAN	F8VS17	Coatomer subunit zeta-1
276	dirMS_041712_NAR_20mM_minus.9246.9283.2	2	20.93	0	2.79E+05		(R)AQIFAN	(R)A Q|I|F|A|N/T/V/D/N A/R(I)		142.32	660.3388	1319.67	0.0001	0.1	5.88	43773.3	5.26	HUMAN	F8VZY9	Keratin, type I cytoskeletal 18
277	dirMS_041712_NAR_50mM_minus2.14104.14336.3	3	20.93	1	1.24E+06		(R)LLDAQL	(R)L/L|D|A/Q|L|A/T/G G L V C P A R/R(L)	C:Carbamidomethylation	149.03	604.3359	1810.996	-0.0025	-1.4	9.6	266161	5.68	HUMAN	E9PPU0	Epiplakin
278	dirMS_041712_NAR_30mM_minus2.13279.13279.2	2	20.92	0	3.00E+05		(K)STYGWT	(K)S T|Y/G|W/T/A N/M E R(I)		125.7	658.2918	1315.574	0.0028	2.1	5.72	47825.1	5.07	HUMAN	Q58FG1	Putative heat shock protein HSP 90-alpha A4
279	dirMS_041712_NAR_60mM_minus.6938.7029.3	3	20.92	1	2.74E+05		(R)IVDGKV	(R)I/V|D/G K/V/V/S/E/T N D/T/K(V)		97.95	502.2642	1504.785	-0.0073	-4.8	4.56	48057	5.34	HUMAN	P05783	Keratin, type I cytoskeletal 18
280	dirMS_041712_NAR_60mM_minus.5948.6095.3	3	20.91	1	3.30E+06		(K)DSYVGD	(K)D S|Y|V/G/D/E/A/Q/S K R(G)		90.38	452.2129	1354.623	0.0008	0.6	4.56	32616.5	5.18	HUMAN	A6NL76	Actin, alpha skeletal muscle
281	dirMS_041712_NAR_20mM_plus.12043.12043.2	2	20.9	0	1.20E+05		(R)IYVVDV(	(R)I Y|V|V|D|V/G S E P R(A)		156.17	617.3237	1233.647	-0.0073	-5.9	4.37	39066	6.61	HUMAN	A6PVW9	Selenium binding protein 1 (Fragment)
282	dirMS_041712_NAR_50mM_plus2.14862.15203.3	3	20.9	1	1.13E+06		(R)VEKIPG(	(R)V E|K I|P G G\I|I|E|D/S/C V|L/R(G)	C:Carbamidomethylation	146.32	595.6548	1784.958	-0.0076	-4.3	4.68	56186.7	5.47	HUMAN	B4DUR8	T-complex protein 1 subunit gamma
283	dirMS_041712_NAR_20mM_minus.10850.10850.2	2	20.89	0	1.35E+06	M92m	(R)ImNTFS	(R)I/m|N/T|F S/V/V|P S/P K(V)	m:Oxidized methionine	157.03	668.3513	1319.703	15.9925	-1.8	8.75	42831.5	4.86	HUMAN	A8K854	HCG1983504, isoform CRA_f
284	dirMS_041712_NAR_30mM_minus2.6914.7024.2	2	20.89	0	5.37E+05		(K)ISSPTETI	(K)I/S|S/P/T/E/T E/R(C)		92.23	510.2598	1019.5	0.0119	11.7	4.53	11854.4	6.57	HUMAN	P31949	Protein S100-A11
285	dirMS_041712_NAR_50mM_minus2.12203.12272.3	3	20.89	0	4.54E+05		(R)GIYAYGI	(R)G I|Y|A|Y|G/F E K/P S A/I Q Q R(A)		137.8	609.9832	1827.939	-0.0038	-2.1	8.5	16071.1	4.8	HUMAN	B4E102	Eukaryotic initiation factor 4A-I
286	dirMS_041712_NAR_30mM_plus2.7058.7058.2	2	20.89	0	1.83E+04		(R)SGGGG(	(R)S G/G G G/G/G|G/L/G S/G G S I|R(S)		92.03	616.8023	1232.598	-0.0005	-0.4	9.47	62291.5	5.13	HUMAN	P35527	Keratin, type I cytoskeletal 9
287	dirMS_041712_NAR_40mM_plus2.18211.18211.3	3	20.89	1	3.46E+04		(K)TFVNITP	(K)T F|V/N/I/T/P A E V/G V L V G K/D/R(S)		173.53	639.0251	1915.065	-0.004	-2.1	5.74	17630.6	11.62	HUMAN	I3L3D5	Profilin-1 (Fragment)
288	dirMS_041712_NAR_60mM_plus.13252.13291.3	3	20.87	1	8.50E+05		(R)INVYYNI	(R)I/N|V|Y/Y|N E/A/T/G G K/Y/V P R(A)		126.17	615.3146	1843.934	-0.0045	-2.4	8.43	50286.6	4.79	HUMAN	P68371	Tubulin beta-4B chain
289	dirMS_041712_NAR_60mM_plus.6821.6823.3	3	20.87	0	4.10E+06		(R)SGSMDI	(R)S/G|S|M/D/P/S G A/H P/S V/R(Q)		94.32	462.2129	1384.627	-0.0032	-2.3	6.46	48340.6	8.84	HUMAN	Q07666	KH domain-containing, RNA-binding, signal transduction-associated protein 1
290	dirMS_041712_NAR_30mM_plus2.11892.11924.2	2	20.86	0	1.89E+06		(K)LVSESSD	(K)L/V|S|E/S/S D|V/L|P/K(-)		115.87	587.3208	1173.636	-0.0018	-1.6	4.37	56607.4	5.37	HUMAN	F8VXB4	Keratin, type II cytoskeletal 8
291	dirMS_041712_NAR_30mM_plus2.17255.17784.2	2	20.86	0	2.26E+06		(K)FLDGIYV	(K)F/L|D|G|I|Y|V/S E/K(G)		145.7	585.8057	1170.604	0	0	4.37	20931.1	10.28	HUMAN	D6RAN4	60S ribosomal protein L9 (Fragment)
292	dirMS_041712_NAR_30mM_plus2.22101.22497.2	2	20.86	0	1.20E+06		(K)IGFPWSI	(K)I/G|F|P/W/S/E/I/R(N)		173.2	552.8032	1104.584	0.0155	14	6	71218.8	6.3	HUMAN	A7YIJ8	Radixin
293	dirMS_041712_NAR_20mM_plus.17686.18043.2	2	20.85	0	4.39E+05		(R)ILVATNL	(R)I/L|V|A|T|N/L/F G R(G)		194.57	552.3309	1103.657	-0.0026	-2.4	9.75	40918.8	9.33	HUMAN	B4DP52	HCG2005638, isoform CRA_c
294	dirMS_041712_NAR_30mM_plus2.12054.12298.2	2	20.85	0	3.34E+06		(R)AQIFAN	(R)A Q|I|F|A|N|T/V/D/N A R(I)		116.82	660.338	1319.67	-0.0015	-1.1	5.88	43773.3	5.26	HUMAN	F8VZY9	Keratin, type I cytoskeletal 18
295	dirMS_041712_NAR_60mM_minus.7573.7819.3	3	20.84	1	6.66E+05		(K)IVADKD	(K)I/V|A D/K/D/Y/S/V/T/A N/S/K(I)		102.58	504.2624	1510.775	-0.0021	-1.4	5.96	25445.8	5.52	HUMAN	A8MW50	L-lactate dehydrogenase (Fragment)
296	dirMS_041712_NAR_40mM_plus2.13318.13318.3	3	20.84	0	6.01E+05		(R)QVSASE	(R)Q V/S A/S E/L/H T S G I/L/G/P/E T L R(D)		145.08	665.6807	1995.051	-0.023	-11.5	5.4	558007.1	5.44	HUMAN	P58107	Epiplakin
297	dirMS_041712_NAR_40mM_plus2.13962.14006.3	3	20.84	0	2.92E+05		(R)LASTLVI	(R)L/A|S T L/V/H L|G E\Y\Q A|A/V/D/G A R(K)		148.43	657.6841	1971.029	0.0083	4.2	5.32	71165.3	5.57	HUMAN	J3KS13	Clathrin heavy chain 1
298	dirMS_041712_NAR_60mM_minus.18717.18768.3	3	20.83	0	2.88E+05		(K)TFLVWV	(K)T F|L|V/W/V/N/E E D/H L R(V)		165.82	553.2818	1657.833	-0.0024	-1.5	4.65	20203.9	6.24	HUMAN	H0YJG0	Creatine kinase B-type (Fragment)
299	dirMS_041712_NAR_50mM_plus2.5960.5978.3	3	20.83	0	2.45E+05		(R)VEAKPE	(R)V/E|A/K/P E|V|Q/S/Q/P P R(V)		95.53	488.9312	1464.781	-0.0015	-1	6.11	54177.2	5.41	HUMAN	Q9UN86	Ras GTPase-activating protein-binding protein 2
300	dirMS_041712_NAR_120mM_plus.9981.10506.3	3	20.82	1	4.08E+06		(R)LQAEIEG	(R)L/Q|A/E/I/E/G L/K/G Q R(A)		106.57	447.9207	1341.748	-0.0009	-0.7	6.14	56607.4	5.37	HUMAN	F8VXB4	Keratin, type II cytoskeletal 8
301	dirMS_041712_NAR_30mM_plus2.12402.12924.2	2	20.82	0	1.13E+06		(R)YSGSYNI	(R)Y/S|G/S/Y/N/D/Y/L/R(A)		119.33	619.2776	1237.548	-0.0005	-0.4	5.83	69661.6	9.75	HUMAN	Q96PK6	RNA-binding protein 14
302	dirMS_041712_NAR_30mM_plus2.14248.14706.2	2	20.82	0	4.31E+06		(K)LLQLVEI	(K)L/L|Q|L|V/E/D/R(G)		129.2	493.2867	985.568	-0.0015	-1.6	4.37	61018.6	9.44	HUMAN	B4DLW8	Probable ATP-dependent RNA helicase DDX5
303	dirMS_041712_NAR_50mM_plus2.11379.11434.3	3	20.82	1	9.91E+05		(K)VIQEIVD	(K)V/I|Q/E/I/V/D/K/S/G V V/R(V)		127.82	481.2834	1441.837	-0.0017	-1.1	6.04	68668.3	5.77	HUMAN	B4DXZ6	Fragile X mental retardation syndrome-related protein 1
304	dirMS_041712_NAR_50mM_minus2.11070.11090.3	3	20.81	1	4.01E+05		(R)INVYYNI	(R)I/N|V|Y/Y|N/E/A/T/G G K/Y/V/P R(A)		131.28	615.3184	1843.934	0.0069	3.8	8.43	50286.6	4.79	HUMAN	P68371	Tubulin beta-4B chain
305	dirMS_041712_NAR_20mM_plus.10886.10886.2	2	20.81	0	2.19E+05	M33m	(K)VAGmD	(K)V/A|G/m\D V|E L/T/V|E E/R(N)	m:Oxidized methionine	147.28	732.3555	1447.71	15.994	-0.6	4	10949.3	5.28	HUMAN	B4DJF2	14-3-3 protein epsilon
306	dirMS_041712_NAR_20mM_plus.15139.15520.2	2	20.81	0	8.10E+05		(K)LLCGLLA	(K)L/L|C|G|L|L/A E/R(L)	C:Carbamidomethylation	177.65	522.7971	1044.587	-0.0001	-0.1	6	12647.2	8.07	HUMAN	P14174	Macrophage migration inhibitory factor
307	dirMS_041712_NAR_60mM_plus.11855.11884.3	3	20.81	0	8.64E+05		(R)NSNLVG	(R)N/S|N|L|V/G A/A/H/E/E/L Q Q S R(I)		119.28	584.9576	1752.862	-0.0041	-2.3	5.4	63136.9	5.83	HUMAN	D6RAQ3	Prelamin-A/C
308	dirMS_041712_NAR_120mM_minus.10076.10591.3	3	20.8	0	1.94E+06		(R)GFCLPPI	(R)G F|C|L/P/P/H/C S R(G)	C:Carbamidomethylation	110.38	410.858	1230.551	0.0088	7.1	9.75	14391.8	4.99	HUMAN	G3V461	Creatine kinase B-type (Fragment)
309	dirMS_041712_NAR_20mM_plus.13156.13562.2	2	20.8	0	8.91E+05		(R)VLQALE(	(R)V/L|Q|A/L/E/G L/K(M)		164.68	485.8004	970.593	0.0004	0.4	5.97	16060.2	10.31	HUMAN	P39019	40S ribosomal protein S19
310	dirMS_041712_NAR_50mM_minus2.8998.9175.3	3	20.79	1	9.01E+05		(K)TLQEVTI	(K)T L|Q|E/V/T/E/M/D/S V K/R(Y)		119.9	512.5962	1535.773	0.0007	0.4	4.68	558007.1	5.44	HUMAN	P58107	Epiplakin
311	dirMS_041712_NAR_40mM_plus2.13447.13447.3	3	20.79	0	2.62E+05		(R)LSAMLV	(R)L/S/A M\L|V/P V T/P E V K/P K(R)		145.78	536.9848	1608.939	0.0005	0.3	8.59	31524.1	4.5	HUMAN	P82970	High mobility group nucleosome-binding domain-containing protein 5
312	dirMS_041712_NAR_40mM_plus2.6587.6782.2	2	20.79	0	4.08E+05		(K)AGFAGD	(K)A/G/F|A|G/D D/A/P R(A)		99.45	488.728	976.448	0.0004	0.4	4.21	123097.1	5.83	HUMAN	A5A3E0	POTE ankyrin domain family member F
313	dirMS_041712_NAR_40mM_minus2.9134.9256.3	3	20.78	1	6.96E+05	M356m	(K)VGEVIVT	(K)V G|E|V/I/V/T/K/D/D A m L L/K(G)	m:Oxidized methionine	136.93	549.6354	1630.908	15.9832	-7.1	4.56	61224.7	5.7	HUMAN	P10809	60 kDa heat shock protein, mitochondrial
314	dirMS_041712_NAR_60mM_minus.9314.9316.3	3	20.77	0	1.02E+06		(K)QEYDES	(K)Q E/Y/D E/S/G/P/S/I/V/H/R(K)		111.27	506.2391	1516.703	0.0001	0.1	4.65	123097.1	5.83	HUMAN	A5A3E0	POTE ankyrin domain family member F
315	dirMS_041712_NAR_30mM_minus2.16659.16659.2	2	20.76	0	2.71E+05	M2243m	(K)mSIYQA	(K)m\S|I|Y/Q/A/M/W/K(G)	m:Oxidized methionine	144.3	587.2756	1157.548	15.9957	0.7	8.34	266161	5.68	HUMAN	E9PPU0	Epiplakin
316	dirMS_041712_NAR_60mM_minus.7978.7978.3	3	20.73	1	4.69E+05		(R)YLAEVA	(R)Y/L|A E/V/A T/G E/K/R(A)		104.32	412.8902	1236.658	-0.0022	-1.8	6.14	28473.3	4.8	HUMAN	P61981	14-3-3 protein gamma
317	dirMS_041712_NAR_60mM_minus.17116.17158.3	3	20.72	1	6.23E+05		(K)EVNKFQ	(K)E V/N K F Q|M/A|Y/S/N/L L/R(A)		155.47	571.6279	1712.879	-0.0097	-5.7	8.69	14683.8	10.19	HUMAN	P37108	Signal recognition particle 14 kDa protein
318	dirMS_041712_NAR_20mM_plus.13932.14162.2	2	20.7	0	2.45E+05		(R)AALQELI	(R)A/A|L|Q/E/L/L/S K(G)		168.75	486.789	972.572	-0.0017	-1.7	6.05	13798.9	10.17	HUMAN	P62851	40S ribosomal protein S25
319	dirMS_041712_NAR_20mM_plus.9939.10187.2	2	20.7	0	1.43E+06		(K)LVSESSD	(K)L/V|S/E/S|S D|V/L/P K(-)		143.17	587.3192	1173.636	-0.005	-4.3	4.37	56607.4	5.37	HUMAN	F8VXB4	Keratin, type II cytoskeletal 8
320	dirMS_041712_NAR_50mM_plus2.15701.15770.3	3	20.7	1	2.33E+05		(R)IFDSEEIL	(R)I/F|D/S E/E/I/L/A/G Y K/R(E)		151.1	514.2745	1540.801	0.0084	5.4	4.68	35548.6	4.91	HUMAN	B4DN60	Asparagine--tRNA ligase, cytoplasmic
321	dirMS_041712_NAR_50mM_plus2.15480.15703.3	3	20.69	0	1.14E+06		(R)LVLEVA(	(R)L/V|L/E/V A Q/H/L/G E/S T V/R(T)		150.15	550.9763	1650.917	-0.003	-1.8	5.4	55307.5	5.25	HUMAN	F8VPV9	ATP synthase subunit beta
322	dirMS_041712_NAR_20mM_plus.14686.14686.2	2	20.68	0	1.30E+05		(K)LVILANN	(K)L V|I|L|A|N/N C|P A L/R(K)	C:Carbamidomethylation	172.67	677.3851	1353.767	-0.0042	-3.1	9.75	12826.8	9.82	HUMAN	E5RI99	60S ribosomal protein L30 (Fragment)

<table>
<tr><td>323</td><td>dirMS_041712_NAR_30mM_minus2.14870.14870.2</td><td>2</td><td>20.67</td><td>0</td><td>1.09E+07</td><td></td><td>(R)FPGQLN</td><td>(R)F/P/G/Q|L/N A D|L R(K)</td><td></td><td>134.72</td><td>565.7971</td><td>1130.595</td><td>-0.0084</td><td>-7.4</td><td>5.84</td><td>42230.8</td><td>4.77</td><td>HUMAN</td><td>A6NKZ8</td><td>Putative tubulin beta chain-like protein ENSP00000290377</td></tr>
<tr><td>324</td><td>dirMS_041712_NAR_50mM_plus2.16028.16028.3</td><td>3</td><td>20.67</td><td>0</td><td>1.05E+06</td><td></td><td>(R)LSFQHD</td><td>(R)L/S/F Q H D\P\E/T/S/V/L/V/L R(K)</td><td></td><td>152.42</td><td>580.9801</td><td>1740.928</td><td>-0.0022</td><td>-1.2</td><td>5.32</td><td>96827.3</td><td>5.58</td><td>HUMAN</td><td>E9PKU7</td><td>Neutral alpha-glucosidase AB</td></tr>
<tr><td>325</td><td>dirMS_041712_NAR_50mM_plus2.19209.19271.3</td><td>3</td><td>20.67</td><td>0</td><td>5.40E+05</td><td></td><td>(K)HLLQAP</td><td>(K)H L L Q\A\P\L|D\D\A|Q/E|I/L/Q/A/R(F)</td><td></td><td>169.5</td><td>644.3484</td><td>1931.035</td><td>-0.0039</td><td>-2</td><td>4.54</td><td>87447.6</td><td>6.44</td><td>HUMAN</td><td>Q15437</td><td>Protein transport protein Sec23B</td></tr>
<tr><td>326</td><td>dirMS_041712_NAR_60mM_minus.13375.13606.3</td><td>3</td><td>20.66</td><td>1</td><td>1.16E+06</td><td></td><td>(R)LATALQI</td><td>(R)L/A|T/A/L Q/K/L/E E/A E/K(A)</td><td></td><td>135.37</td><td>481.9376</td><td>1443.805</td><td>-0.0071</td><td>-4.9</td><td>4.79</td><td>31923.6</td><td>4.85</td><td>HUMAN</td><td>B7Z596</td><td>Tropomyosin alpha-1 chain</td></tr>
<tr><td>327</td><td>dirMS_041712_NAR_60mM_minus.4659.4719.3</td><td>3</td><td>20.66</td><td>1</td><td>1.35E+06</td><td></td><td>(R)AAEEED</td><td>(R)A/A|E/E/E/D/E/A D/P K/R(Q)</td><td></td><td>78.07</td><td>453.872</td><td>1359.602</td><td>-0.0008</td><td>-0.6</td><td>4.08</td><td>11529.7</td><td>4.14</td><td>HUMAN</td><td>P20962</td><td>Parathymosin</td></tr>
<tr><td>328</td><td>dirMS_041712_NAR_60mM_plus.9285.9322.3</td><td>3</td><td>20.66</td><td>0</td><td>4.31E+06</td><td></td><td>(K)QEYDES</td><td>(K)Q E/Y/D E/S/G/P/S/I/V/H/R(K)</td><td></td><td>107.12</td><td>506.2392</td><td>1516.703</td><td>0.0004</td><td>0.3</td><td>4.65</td><td>123097.1</td><td>5.83</td><td>HUMAN</td><td>A5A3E0</td><td>POTE ankyrin domain family member F</td></tr>
<tr><td>329</td><td>dirMS_041712_NAR_20mM_minus.10124.10124.2</td><td>2</td><td>20.65</td><td>0</td><td>1.24E+05</td><td></td><td>(R)YALYDA</td><td>(R)Y A|L|Y/D/A T Y/E T K(E)</td><td></td><td>150.82</td><td>669.3156</td><td>1337.626</td><td>-0.0021</td><td>-1.5</td><td>4.37</td><td>22956</td><td>8.8</td><td>HUMAN</td><td>E9PK25</td><td>Cofilin-1</td></tr>
<tr><td>330</td><td>dirMS_041712_NAR_30mM_plus2.21438.21438.2</td><td>2</td><td>20.65</td><td>0</td><td>1.58E+05</td><td></td><td>(R)VFIGNLI</td><td>(R)V F|I/G/N L|N T L V/V K(K)</td><td></td><td>167.38</td><td>658.8983</td><td>1316.794</td><td>-0.0043</td><td>-3.3</td><td>8.72</td><td>15791.7</td><td>9.22</td><td>HUMAN</td><td>B4DSU6</td><td>Heterogeneous nuclear ribonucleoproteins C1/C2</td></tr>
<tr><td>331</td><td>dirMS_041712_NAR_20mM_plus.17561.17641.3</td><td>3</td><td>20.64</td><td>0</td><td>1.96E+05</td><td></td><td>(K)SYELPD</td><td>(K)S/Y\E\L/P D/G Q|V|I/T/I/G/N E R(F)</td><td></td><td>193.17</td><td>597.6357</td><td>1790.892</td><td>0.0006</td><td>0.4</td><td>4.14</td><td>123097.1</td><td>5.83</td><td>HUMAN</td><td>A5A3E0</td><td>POTE ankyrin domain family member F</td></tr>
<tr><td>332</td><td>dirMS_041712_NAR_50mM_minus2.12498.12622.3</td><td>3</td><td>20.63</td><td>0</td><td>1.09E+06</td><td>M114m M:</td><td>(R)AGKPVI</td><td>(R)A G K|P V/I|C A\T|Q|m|L E/S m I/K(K)</td><td>C:Carbamidomethylation m:Oxid</td><td>139.87</td><td>636.9917</td><td>1876.969</td><td>31.9912</td><td>0.7</td><td>8.64</td><td>18466.1</td><td>5.78</td><td>HUMAN</td><td>H3BSU3</td><td>Pyruvate kinase (Fragment)</td></tr>
<tr><td>333</td><td>dirMS_041712_NAR_30mM_plus2.21784.21784.2</td><td>2</td><td>20.63</td><td>0</td><td>3.77E+05</td><td></td><td>(R)FSWNV</td><td>(R)F S/W/N|V W/P\S S R(L)</td><td></td><td>169.4</td><td>633.3085</td><td>1265.606</td><td>0.0035</td><td>2.8</td><td>9.75</td><td>83880.6</td><td>7.33</td><td>HUMAN</td><td>F5H365</td><td>Protein transport protein Sec23A</td></tr>
<tr><td>334</td><td>dirMS_041712_NAR_30mM_minus2.8675.9044.2</td><td>2</td><td>20.62</td><td>0</td><td>2.22E+07</td><td></td><td>(K)AQYEDI</td><td>(K)A/Q|Y|E|D/I|A/N/R(S)</td><td></td><td>103</td><td>540.2601</td><td>1079.512</td><td>0.0013</td><td>1.2</td><td>4.37</td><td>37296.3</td><td>5.3</td><td>HUMAN</td><td>F8VU64</td><td>Keratin, type II cytoskeletal 8</td></tr>
<tr><td>335</td><td>dirMS_041712_NAR_20mM_plus.17067.17110.3</td><td>3</td><td>20.62</td><td>0</td><td>2.36E+05</td><td></td><td>(R)AIVAIEN</td><td>(R)A I\V|A\I\E/N|P A/D|V/S/V/I/S S R(N)</td><td></td><td>188.88</td><td>580.9864</td><td>1740.949</td><td>-0.0044</td><td>-2.5</td><td>4.37</td><td>33022.6</td><td>4.78</td><td>HUMAN</td><td>A6NE09</td><td>Protein RPSAP58</td></tr>
<tr><td>336</td><td>dirMS_041712_NAR_30mM_plus2.15151.15729.2</td><td>2</td><td>20.62</td><td>0</td><td>7.20E+06</td><td></td><td>(R)VLQALE</td><td>(R)V/L|Q|A|L/E/G L/K(M)</td><td></td><td>133.6</td><td>485.7997</td><td>970.593</td><td>-0.001</td><td>-1.1</td><td>5.97</td><td>16060.2</td><td>10.31</td><td>HUMAN</td><td>P39019</td><td>40S ribosomal protein S19</td></tr>
<tr><td>337</td><td>dirMS_041712_NAR_30mM_plus2.7678.7679.2</td><td>2</td><td>20.62</td><td>0</td><td>6.45E+05</td><td></td><td>(K)TGQAPG</td><td>(K)T G\Q|A|P|G Y/S/Y T A/A/N/K(N)</td><td></td><td>94.28</td><td>714.841</td><td>1428.675</td><td>-0.0007</td><td>-0.5</td><td>8.16</td><td>11447.2</td><td>9.81</td><td>HUMAN</td><td>C9JFR7</td><td>Cytochrome c (Fragment)</td></tr>
<tr><td>338</td><td>dirMS_041712_NAR_50mM_plus2.15871.15895.3</td><td>3</td><td>20.6</td><td>1</td><td>2.05E+06</td><td></td><td>(K)VVEEAP</td><td>(K)V/V|E|E|A/P/S I/F L D/A E T R/R(A)</td><td></td><td>151.53</td><td>611.3256</td><td>1831.955</td><td>0.0074</td><td>4</td><td>4.41</td><td>80685.1</td><td>7.31</td><td>HUMAN</td><td>P05165</td><td>Propionyl-CoA carboxylase alpha chain, mitochondrial</td></tr>
<tr><td>339</td><td>dirMS_041712_NAR_60mM_plus.9965.9995.2</td><td>2</td><td>20.6</td><td>0</td><td>9.64E+05</td><td></td><td>(R)LESGMC</td><td>(R)L/E|S\G/M/Q/N M/S I/H/T K(T)</td><td></td><td>110.1</td><td>738.3522</td><td>1475.698</td><td>-0.001</td><td>-0.7</td><td>6.75</td><td>56607.4</td><td>5.37</td><td>HUMAN</td><td>F8VXB4</td><td>Keratin, type II cytoskeletal 8</td></tr>
<tr><td>340</td><td>dirMS_041712_NAR_50mM_minus2.18443.18502.3</td><td>3</td><td>20.58</td><td>1</td><td>1.47E+06</td><td></td><td>(R)VISGVLC</td><td>(R)V/I|S/G V/L/Q/L/G N I V/F/K/K(E)</td><td></td><td>177</td><td>539.0018</td><td>1614.994</td><td>-0.0033</td><td>-2</td><td>10</td><td>227783.5</td><td>5.5</td><td>HUMAN</td><td>P35579</td><td>Myosin-9</td></tr>
<tr><td>341</td><td>dirMS_041712_NAR_30mM_plus2.7765.8299.2</td><td>2</td><td>20.58</td><td>0</td><td>1.86E+07</td><td></td><td>(K)AQYEDI</td><td>(K)A/Q|Y|E/D/I|A/N/R(S)</td><td></td><td>95.88</td><td>540.2594</td><td>1079.512</td><td>-0.0001</td><td>-0.1</td><td>4.37</td><td>37296.3</td><td>5.3</td><td>HUMAN</td><td>F8VU64</td><td>Keratin, type II cytoskeletal 8</td></tr>
<tr><td>342</td><td>dirMS_041712_NAR_50mM_minus2.14140.14161.3</td><td>3</td><td>20.57</td><td>1</td><td>4.43E+05</td><td></td><td>(R)LYSILGT</td><td>(R)L/Y|S/I/L/G T T L/K D/E/G K(L)</td><td></td><td>148.45</td><td>513.2863</td><td>1537.847</td><td>-0.0029</td><td>-1.9</td><td>6.07</td><td>66876.9</td><td>6.17</td><td>HUMAN</td><td>O75083</td><td>WD repeat-containing protein 1</td></tr>
<tr><td>343</td><td>dirMS_041712_NAR_20mM_plus.18311.18393.3</td><td>3</td><td>20.57</td><td>0</td><td>3.15E+05</td><td></td><td>(R)AAVEEG</td><td>(R)A A V E E G I\V|L/G/G/G/C A L/L/R(C)</td><td>C:Carbamidomethylation</td><td>198.02</td><td>562.3058</td><td>1684.905</td><td>-0.0022</td><td>-1.3</td><td>4.53</td><td>61224.7</td><td>5.7</td><td>HUMAN</td><td>P10809</td><td>60 kDa heat shock protein, mitochondrial</td></tr>
<tr><td>344</td><td>dirMS_041712_NAR_60mM_plus.15562.15563.3</td><td>3</td><td>20.57</td><td>0</td><td>4.04E+05</td><td></td><td>(K)YGYTHL</td><td>(K)Y/G|Y/T/H/L/S/T G/D/L/L/R(S)</td><td></td><td>138.05</td><td>499.2612</td><td>1495.754</td><td>0.0151</td><td>10.1</td><td>6.74</td><td>21748.6</td><td>8.92</td><td>HUMAN</td><td>P00568</td><td>Adenylate kinase isoenzyme 1</td></tr>
<tr><td>345</td><td>dirMS_041712_NAR_30mM_minus2.7389.7390.2</td><td>2</td><td>20.55</td><td>0</td><td>3.68E+05</td><td></td><td>(K)VAVTEG</td><td>(K)V/A|V|T|E/G/C/Q|P S R(V)</td><td>C:Carbamidomethylation</td><td>94.73</td><td>602.2935</td><td>1203.579</td><td>0.0011</td><td>0.9</td><td>5.97</td><td>258215.9</td><td>5.48</td><td>HUMAN</td><td>E7EN95</td><td>Filamin-B</td></tr>
<tr><td>346</td><td>dirMS_041712_NAR_50mM_plus2.15574.15721.3</td><td>3</td><td>20.55</td><td>0</td><td>5.05E+06</td><td></td><td>(R)MGPLGI</td><td>(R)M/G|P/L/G/L D/H/M/A/S S I E/R(M)</td><td></td><td>150.08</td><td>538.6007</td><td>1613.777</td><td>0.0101</td><td>6.3</td><td>5.3</td><td>77799.7</td><td>9.09</td><td>HUMAN</td><td>P52272</td><td>Heterogeneous nuclear ribonucleoprotein M</td></tr>
<tr><td>347</td><td>dirMS_041712_NAR_50mM_minus2.17627.17920.3</td><td>3</td><td>20.54</td><td>1</td><td>2.12E+06</td><td></td><td>(R)GLFIIDD</td><td>(R)G L|F|I/I/D/D/K/G I L R(Q)</td><td></td><td>170.03</td><td>453.9381</td><td>1359.8</td><td>0.0003</td><td>0.2</td><td>5.96</td><td>18498.6</td><td>5.82</td><td>HUMAN</td><td>H7C3T4</td><td>Peroxiredoxin-4 (Fragment)</td></tr>
<tr><td>348</td><td>dirMS_041712_NAR_60mM_minus.9592.9718.3</td><td>3</td><td>20.54</td><td>0</td><td>3.48E+05</td><td></td><td>(R)QLSQAG</td><td>(R)Q L|S|Q/A/G/S/F/S/D/G T H/G G L R(Y)</td><td></td><td>113.6</td><td>573.2781</td><td>1717.825</td><td>-0.0055</td><td>-3.2</td><td>6.74</td><td>266161</td><td>5.68</td><td>HUMAN</td><td>E9PPU0</td><td>Epiplakin</td></tr>
<tr><td>349</td><td>dirMS_041712_NAR_50mM_plus2.12928.13230.3</td><td>3</td><td>20.54</td><td>0</td><td>1.86E+06</td><td></td><td>(R)GIYAYGI</td><td>(R)G I|Y|A|Y/G/F E/K/P/S A I Q Q R(A)</td><td></td><td>136.68</td><td>609.9821</td><td>1827.939</td><td>-0.0071</td><td>-3.9</td><td>8.5</td><td>16071.1</td><td>4.8</td><td>HUMAN</td><td>B4E102</td><td>Eukaryotic initiation factor 4A-I</td></tr>
<tr><td>350</td><td>dirMS_041712_NAR_20mM_minus.13250.13250.2</td><td>2</td><td>20.53</td><td>0</td><td>2.34E+05</td><td></td><td>(R)GVVDSE</td><td>(R)G V|V|D/S/E/D I|P/L N/L S R(E)</td><td></td><td>183.02</td><td>757.3944</td><td>1513.786</td><td>-0.0041</td><td>-2.7</td><td>4.03</td><td>74494.3</td><td>7.79</td><td>HUMAN</td><td>F5H897</td><td>Heat shock protein 75 kDa, mitochondrial</td></tr>
<tr><td>351</td><td>dirMS_041712_NAR_60mM_minus.6371.6435.3</td><td>3</td><td>20.53</td><td>0</td><td>3.61E+05</td><td></td><td>(K)AVTEQG</td><td>(K)A V|T/E/Q/G/H/E/L/S N E/E R(N)</td><td></td><td>94.27</td><td>533.585</td><td>1598.741</td><td>-0.0001</td><td>0</td><td>4.48</td><td>28196</td><td>4.76</td><td>HUMAN</td><td>P31946</td><td>14-3-3 protein beta/alpha</td></tr>
<tr><td>352</td><td>dirMS_041712_NAR_30mM_plus2.7761.8213.2</td><td>2</td><td>20.53</td><td>0</td><td>1.59E+06</td><td></td><td>(K)SNVSDA</td><td>(K)S/N|V|S|D/A|V|A/Q/S T R(I)</td><td></td><td>95.68</td><td>617.8044</td><td>1234.602</td><td>-0.0007</td><td>-0.6</td><td>5.55</td><td>31075.7</td><td>5.65</td><td>HUMAN</td><td>P60174</td><td>Triosephosphate isomerase</td></tr>
<tr><td>353</td><td>dirMS_041712_NAR_50mM_plus2.15440.15708.3</td><td>3</td><td>20.53</td><td>1</td><td>1.57E+06</td><td></td><td>(R)ISGLIYEE</td><td>(R)I/S|G|L|I/Y/E E T R G V/L/K(V)</td><td></td><td>149.97</td><td>526.6345</td><td>1577.89</td><td>-0.0008</td><td>-0.5</td><td>6.14</td><td>11367.1</td><td>11.36</td><td>HUMAN</td><td>P62805</td><td>Histone H4</td></tr>
<tr><td>354</td><td>dirMS_041712_NAR_50mM_plus2.22130.22130.3</td><td>3</td><td>20.53</td><td>1</td><td>2.51E+05</td><td></td><td>(R)KEGGLG</td><td>(R)K E G G L G P L N\I|P/L/L|A/D V T R(R)</td><td></td><td>189.68</td><td>621.6925</td><td>1863.07</td><td>-0.0069</td><td>-3.7</td><td>6.07</td><td>15252.2</td><td>5.82</td><td>HUMAN</td><td>A6NIW5</td><td>Peroxiredoxin 2, isoform CRA_a</td></tr>
<tr><td>355</td><td>dirMS_041712_NAR_60mM_plus.6542.6660.3</td><td>3</td><td>20.53</td><td>0</td><td>1.19E+06</td><td></td><td>(K)AVTEQG</td><td>(K)A/V|T/E/Q/G/H/E/L/S/N E/E/R(N)</td><td></td><td>93.05</td><td>533.5844</td><td>1598.741</td><td>-0.0019</td><td>-1.2</td><td>4.48</td><td>28196</td><td>4.76</td><td>HUMAN</td><td>P31946</td><td>14-3-3 protein beta/alpha</td></tr>
<tr><td>356</td><td>dirMS_041712_NAR_50mM_minus2.13486.13853.3</td><td>3</td><td>20.52</td><td>0</td><td>5.60E+05</td><td></td><td>(K)LQMEAI</td><td>(K)L/Q|M/E/A/P/H/I/I/V/G T P G R(V)</td><td></td><td>145.63</td><td>540.2947</td><td>1618.873</td><td>-0.0038</td><td>-2.4</td><td>6.75</td><td>20859.5</td><td>4.85</td><td>HUMAN</td><td>J3KS25</td><td>Eukaryotic initiation factor 4A-I (Fragment)</td></tr>
<tr><td>357</td><td>dirMS_041712_NAR_30mM_plus2.12922.12922.2</td><td>2</td><td>20.51</td><td>0</td><td>3.14E+05</td><td></td><td>(K)QQIQSI</td><td>(K)Q Q/I|Q/S/I/Q Q/S/I E R(L)</td><td></td><td>120.75</td><td>729.3893</td><td>1457.771</td><td>0.0006</td><td>0.4</td><td>6</td><td>14855.1</td><td>9.26</td><td>HUMAN</td><td>B1AHD1</td><td>NHP2 non-histone chromosome protein 2-like 1 (S. cerevisiae)</td></tr>
<tr><td>358</td><td>dirMS_041712_NAR_40mM_plus2.14899.14953.3</td><td>3</td><td>20.51</td><td>1</td><td>3.92E+05</td><td></td><td>(K)VTADVI</td><td>(K)V/T|A/D|V|I/N A/A E K/L/Q V V G R(A)</td><td></td><td>154.68</td><td>595.0062</td><td>1783.007</td><td>-0.0032</td><td>-1.8</td><td>6.04</td><td>57391.2</td><td>6.29</td><td>HUMAN</td><td>O43175</td><td>D-3-phosphoglycerate dehydrogenase</td></tr>
<tr><td>359</td><td>dirMS_041712_NAR_40mM_plus2.7966.8020.3</td><td>3</td><td>20.51</td><td>1</td><td>1.36E+05</td><td></td><td>(K)GAVAED</td><td>(K)G A|V|A|E/D/G D/E/L R T E/P E/A/K(K)</td><td></td><td>107.38</td><td>596.2867</td><td>1786.845</td><td>0.0002</td><td>0.1</td><td>4.08</td><td>19284.3</td><td>5.23</td><td>HUMAN</td><td>G3V3C7</td><td>DNA-(apurinic or apyrimidinic site) lyase (Fragment)</td></tr>
<tr><td>360</td><td>dirMS_041712_NAR_50mM_plus2.17934.18302.3</td><td>3</td><td>20.49</td><td>1</td><td>1.06E+06</td><td></td><td>(R)LFVGNLI</td><td>(R)L/F|V/G/N/L/P T D/I/T E E D F K R(L)</td><td></td><td>163.23</td><td>665.3443</td><td>1994.023</td><td>-0.0046</td><td>-2.3</td><td>4.32</td><td>58856.7</td><td>6.26</td><td>HUMAN</td><td>Q8WXF1</td><td>Paraspeckle component 1</td></tr>
<tr><td>361</td><td>dirMS_041712_NAR_60mM_minus.13593.13714.3</td><td>3</td><td>20.47</td><td>1</td><td>1.05E+06</td><td></td><td>(R)VFDKDG</td><td>(R)V/F|D K D G N/G\Y|I/S/A/A/E/L R(H)</td><td></td><td>136.05</td><td>585.6277</td><td>1754.871</td><td>-0.0022</td><td>-1.3</td><td>4.56</td><td>21860.1</td><td>4.45</td><td>HUMAN</td><td>E7EMB3</td><td>Calmodulin</td></tr>
<tr><td>362</td><td>dirMS_041712_NAR_60mM_plus.7009.7035.3</td><td>3</td><td>20.47</td><td>1</td><td>1.65E+06</td><td></td><td>(R)IVDGKV</td><td>(R)I/V|D/G K/V/V/S/E/T N D/T K(V)</td><td></td><td>95.08</td><td>502.2639</td><td>1504.785</td><td>-0.0082</td><td>-5.4</td><td>4.56</td><td>48057</td><td>5.34</td><td>HUMAN</td><td>P05783</td><td>Keratin, type I cytoskeletal 18</td></tr>
<tr><td>363</td><td>dirMS_041712_NAR_50mM_minus2.12652.12861.3</td><td>3</td><td>20.45</td><td>1</td><td>6.87E+05</td><td></td><td>(R)TFNTST</td><td>(R)T F|N/T/S/T/G G L|L/L/P S D/T K R(S)</td><td></td><td>140.78</td><td>603.3247</td><td>1807.955</td><td>0.0047</td><td>2.6</td><td>8.41</td><td>64302.5</td><td>6.4</td><td>HUMAN</td><td>B1AHC7</td><td>X-ray repair complementing defective repair in Chinese hamster cells 6 (Ku autoantigen, 70kDa)</td></tr>
<tr><td>364</td><td>dirMS_041712_NAR_30mM_plus2.8236.8273.2</td><td>2</td><td>20.45</td><td>0</td><td>8.54E+05</td><td></td><td>(R)ALANSL</td><td>(R)A L|A|N/S/L\A/C/Q/G K(Y)</td><td>C:Carbamidomethylation</td><td>97.53</td><td>566.7922</td><td>1132.578</td><td>-0.0008</td><td>-0.7</td><td>8.8</td><td>13951.6</td><td>9.48</td><td>HUMAN</td><td>H3BR68</td><td>Fructose-bisphosphate aldolase A (Fragment)</td></tr>
<tr><td>365</td><td>dirMS_041712_NAR_30mM_minus2.15403.15696.2</td><td>2</td><td>20.44</td><td>0</td><td>4.05E+05</td><td></td><td>(K)EIIDPVLI</td><td>(K)E I|I/D|P/V/L/D/R(I)</td><td></td><td>138.15</td><td>535.299</td><td>1069.589</td><td>0.0019</td><td>1.8</td><td>4.03</td><td>49012.3</td><td>4.9</td><td>HUMAN</td><td>A8MUB1</td><td>Tubulin alpha-4A chain</td></tr>
<tr><td>366</td><td>dirMS_041712_NAR_20mM_plus.16903.16903.2</td><td>2</td><td>20.44</td><td>0</td><td>3.11E+05</td><td></td><td>(K)VQIAVA</td><td>(K)V Q|I\A/V/A/N/A/Q E/L/L Q R(M)</td><td></td><td>187.77</td><td>776.9419</td><td>1552.881</td><td>-0.004</td><td>-2.6</td><td>5.97</td><td>10728.1</td><td>9.15</td><td>HUMAN</td><td>Q9Y5L4</td><td>Mitochondrial import inner membrane translocase subunit Tim13</td></tr>
<tr><td>367</td><td>dirMS_041712_NAR_40mM_plus2.18538.18799.3</td><td>3</td><td>20.44</td><td>0</td><td>4.34E+05</td><td></td><td>(K)ALIEVLQ</td><td>(K)A L|I|E\V|L/Q/P L/I/A\E H Q A R(R)</td><td></td><td>176.12</td><td>601.0146</td><td>1801.033</td><td>-0.0038</td><td>-2.1</td><td>5.4</td><td>53506.8</td><td>5.83</td><td>HUMAN</td><td>P23381</td><td>Tryptophan--tRNA ligase, cytoplasmic</td></tr>
<tr><td>368</td><td>dirMS_041712_NAR_60mM_minus.18153.18419.3</td><td>3</td><td>20.43</td><td>1</td><td>1.56E+06</td><td></td><td>(K)SLLGKD</td><td>(K)S/L|L/G/K/D\V|L F/L/K(D)</td><td></td><td>163.42</td><td>411.5912</td><td>1232.761</td><td>-0.0022</td><td>-1.8</td><td>8.31</td><td>41827.5</td><td>7</td><td>HUMAN</td><td>B7Z7A9</td><td>Phosphoglycerate kinase</td></tr>
<tr><td>369</td><td>dirMS_041712_NAR_30mM_plus2.13411.13423.2</td><td>2</td><td>20.42</td><td>0</td><td>3.82E+05</td><td></td><td>(R)GATQQI</td><td>(R)G A/T|Q|Q|I|L/D|E|A E/R(S)</td><td></td><td>123.63</td><td>665.834</td><td>1330.66</td><td>0.001</td><td>0.7</td><td>4.14</td><td>57204.9</td><td>6.01</td><td>HUMAN</td><td>F5GWF6</td><td>T-complex protein 1 subunit beta</td></tr>
<tr><td>370</td><td>dirMS_041712_NAR_20mM_plus.18418.18418.2</td><td>2</td><td>20.41</td><td>0</td><td>1.95E+05</td><td></td><td>(R)AAVEEG</td><td>(R)A A/V/E E|G I|V|L/G/G/G/C A L L R(C)</td><td>C:Carbamidomethylation</td><td>197.9</td><td>842.9549</td><td>1684.905</td><td>-0.0025</td><td>-1.5</td><td>4.53</td><td>61224.7</td><td>5.7</td><td>HUMAN</td><td>P10809</td><td>60 kDa heat shock protein, mitochondrial</td></tr>
<tr><td>371</td><td>dirMS_041712_NAR_30mM_plus2.13855.13977.2</td><td>2</td><td>20.41</td><td>0</td><td>1.26E+06</td><td></td><td>(R)NTGIICT</td><td>(R)N/T|G|I|I|C/T/I|G|P A S R(S)</td><td>C:Carbamidomethylation</td><td>125.83</td><td>680.3557</td><td>1359.705</td><td>-0.0008</td><td>-0.6</td><td>9.75</td><td>30947.9</td><td>6.47</td><td>HUMAN</td><td>H3BQ34</td><td>Pyruvate kinase</td></tr>
<tr><td>372</td><td>dirMS_041712_NAR_50mM_plus2.18844.19195.3</td><td>3</td><td>20.41</td><td>0</td><td>9.37E+05</td><td></td><td>(K)LLYDLAD</td><td>(K)L/L|Y|D|L/A/D/Q L/H/A A/V|G A S R(A)</td><td></td><td>168.2</td><td>604.9965</td><td>1812.96</td><td>0.0147</td><td>8.1</td><td>5.21</td><td>24273.5</td><td>8.98</td><td>HUMAN</td><td>H0YK49</td><td>Electron transfer flavoprotein subunit alpha, mitochondrial</td></tr>
<tr><td>373</td><td>dirMS_041712_NAR_50mM_plus2.22290.22290.3</td><td>3</td><td>20.41</td><td>0</td><td>1.53E+06</td><td></td><td>(R)DALSDL</td><td>(R)D/A|L|S D L/A L/H/F L/N/K(M)</td><td></td><td>191.78</td><td>486.2644</td><td>1456.78</td><td>-0.0008</td><td>-0.6</td><td>5.21</td><td>42755</td><td>7.81</td><td>HUMAN</td><td>B7Z2F4</td><td>T-complex protein 1 subunit delta</td></tr>
<tr><td>374</td><td>dirMS_041712_NAR_20mM_minus.13198.13258.2</td><td>2</td><td>20.4</td><td>0</td><td>4.63E+05</td><td></td><td>(K)TITLEVEI</td><td>(K)T I|T|L/E|V|E|P S D T I/E/N V K(A)</td><td></td><td>183.05</td><td>894.4654</td><td>1787.927</td><td>-0.0038</td><td>-2.1</td><td>4</td><td>17271.6</td><td>6.75</td><td>HUMAN</td><td>B4DV12</td><td>Ubiquitin</td></tr>
<tr><td>375</td><td>dirMS_041712_NAR_120mM_minus.19359.19501.3</td><td>3</td><td>20.39</td><td>1</td><td>3.72E+05</td><td></td><td>(K)NLRDIDI</td><td>(K)N L/R D I\D|E|V|S|S/L/L/R(T)</td><td></td><td>161.42</td><td>510.614</td><td>1529.828</td><td>-0.0007</td><td>-0.5</td><td>4.56</td><td>58214.7</td><td>5.25</td><td>HUMAN</td><td>B4DEM7</td><td>T-complex protein 1 subunit theta</td></tr>
<tr><td>376</td><td>dirMS_041712_NAR_40mM_minus2.10853.11006.3</td><td>3</td><td>20.39</td><td>1</td><td>1.95E+06</td><td></td><td>(K)TITLEVEI</td><td>(K)T I|T|L/E|V/E/P S D T I E N V K A K(I)</td><td></td><td>146.88</td><td>663.0226</td><td>1987.059</td><td>-0.0061</td><td>-3.1</td><td>4.41</td><td>17271.6</td><td>6.75</td><td>HUMAN</td><td>B4DV12</td><td>Ubiquitin</td></tr>
<tr><td>377</td><td>dirMS_041712_NAR_50mM_plus2.15861.16082.3</td><td>3</td><td>20.39</td><td>1</td><td>6.63E+06</td><td></td><td>(R)VVVDAL</td><td>(R)V/V|V/D/A/L/S/G/L/K/G D L A G R(Y)</td><td></td><td>151.98</td><td>523.9715</td><td>1569.896</td><td>0.0041</td><td>2.6</td><td>5.93</td><td>26949.2</td><td>7.99</td><td>HUMAN</td><td>C9JSQ1</td><td>Creatine kinase U-type, mitochondrial (Fragment)</td></tr>
<tr><td>378</td><td>dirMS_041712_NAR_60mM_plus.20100.20163.3</td><td>3</td><td>20.39</td><td>1</td><td>2.57E+05</td><td></td><td>(K)VVLDDK</td><td>(K)V/V|L/D/D/K/D/Y/F/L/F/R(D)</td><td></td><td>165.47</td><td>510.6056</td><td>1529.8</td><td>0.0024</td><td>1.6</td><td>4.43</td><td>5189.9</td><td>4.56</td><td>HUMAN</td><td>B8ZZ54</td><td>10 kDa heat shock protein, mitochondrial</td></tr>
<tr><td>379</td><td>dirMS_041712_NAR_60mM_plus.9284.9451.3</td><td>3</td><td>20.39</td><td>0</td><td>4.31E+06</td><td></td><td>(K)QEYDES</td><td>(K)Q E/Y/D E/S/G/P/S/I/V/H/R(K)</td><td></td><td>107.12</td><td>506.2392</td><td>1516.703</td><td>0.0004</td><td>0.3</td><td>4.65</td><td>123097.1</td><td>5.83</td><td>HUMAN</td><td>A5A3E0</td><td>POTE ankyrin domain family member F</td></tr>
<tr><td>380</td><td>dirMS_041712_NAR_120mM_plus.6094.6118.3</td><td>3</td><td>20.38</td><td>1</td><td>1.75E+06</td><td></td><td>(K)DSYVGD</td><td>(K)D S|Y|V/G D/E/A/Q/S K R(G)</td><td></td><td>86.23</td><td>452.2133</td><td>1354.623</td><td>0.002</td><td>1.5</td><td>4.56</td><td>32616.5</td><td>5.18</td><td>HUMAN</td><td>A6NL76</td><td>Actin, alpha skeletal muscle</td></tr>
<tr><td>381</td><td>dirMS_041712_NAR_40mM_plus2.18043.18251.3</td><td>3</td><td>20.38</td><td>0</td><td>2.92E+06</td><td></td><td>(K)ISSIQSIV</td><td>(K)I/S|S I/Q/S/I/V/P A L E I/A/N/A H R(K)</td><td></td><td>173.12</td><td>640.3668</td><td>1919.071</td><td>0.015</td><td>7.8</td><td>6.75</td><td>61224.7</td><td>5.7</td><td>HUMAN</td><td>P10809</td><td>60 kDa heat shock protein, mitochondrial</td></tr>
<tr><td>382</td><td>dirMS_041712_NAR_50mM_plus2.14402.14657.3</td><td>3</td><td>20.38</td><td>0</td><td>2.70E+06</td><td></td><td>(R)LSQYQE</td><td>(R)L/S|Q/Y/Q/E/P L/H L/P G V R(V)</td><td></td><td>144.57</td><td>546.2968</td><td>1636.881</td><td>-0.0047</td><td>-2.9</td><td>6.75</td><td>80685.1</td><td>7.31</td><td>HUMAN</td><td>P05165</td><td>Propionyl-CoA carboxylase alpha chain, mitochondrial</td></tr>
<tr><td>383</td><td>dirMS_041712_NAR_50mM_plus2.19996.20200.3</td><td>3</td><td>20.38</td><td>0</td><td>4.85E+05</td><td></td><td>(K)ALIEVLQ</td><td>(K)A L|I|E|V\L/Q/P/L/I/A E H Q A R(R)</td><td></td><td>174.2</td><td>601.0191</td><td>1801.033</td><td>0.0097</td><td>5.4</td><td>5.4</td><td>53506.8</td><td>5.83</td><td>HUMAN</td><td>P23381</td><td>Tryptophan--tRNA ligase, cytoplasmic</td></tr>
<tr><td>384</td><td>dirMS_041712_NAR_40mM_minus2.8183.8251.3</td><td>3</td><td>20.37</td><td>1</td><td>5.29E+05</td><td></td><td>(R)DVVICPI</td><td>(R)D V|V|I/C/P D A/S L/E D/A K K(E)</td><td>C:Carbamidomethylation</td><td>129.32</td><td>553.9467</td><td>1659.826</td><td>-0.0003</td><td>-0.2</td><td>4.23</td><td>18080.7</td><td>7.92</td><td>HUMAN</td><td>K7ELW0</td><td>Protein DJ-1</td></tr>
<tr><td>385</td><td>dirMS_041712_NAR_50mM_plus2.16989.17013.3</td><td>3</td><td>20.37</td><td>0</td><td>7.61E+05</td><td></td><td>(R)QYYTLLI</td><td>(R)Q Y/Y/T|L/L N Q/A/P/D|M L H/R(K)</td><td></td><td>156.97</td><td>621.6447</td><td>1862.922</td><td>-0.0022</td><td>-1.2</td><td>6.74</td><td>4335.1</td><td>9.82</td><td>HUMAN</td><td>E5RH00</td><td>Ras GTPase-activating protein-binding protein 1</td></tr>
<tr><td>386</td><td>dirMS_041712_NAR_120mM_minus.11654.11740.4</td><td>4</td><td>20.36</td><td>2</td><td>9.96E+05</td><td></td><td>(R)SGKYDLI</td><td>(R)S\G K Y D\L|D|F K/S/P D/D/P/S/R(Y)</td><td></td><td>117.92</td><td>457.4692</td><td>1826.855</td><td>-0.0006</td><td>-0.3</td><td>4.58</td><td>47510.4</td><td>7.04</td><td>HUMAN</td><td>P06733</td><td>Alpha-enolase</td></tr>
<tr><td>387</td><td>dirMS_041712_NAR_20mM_plus.10086.10327.2</td><td>2</td><td>20.36</td><td>0</td><td>1.43E+06</td><td></td><td>(K)LVSESSD</td><td>(K)L/V|S|E/S/S D|V/L/P K(-)</td><td></td><td>143.17</td><td>587.3192</td><td>1173.636</td><td>-0.005</td><td>-4.3</td><td>4.37</td><td>56607.4</td><td>5.37</td><td>HUMAN</td><td>F8VXB4</td><td>Keratin, type II cytoskeletal 8</td></tr>
<tr><td>388</td><td>dirMS_041712_NAR_50mM_plus2.15034.15460.3</td><td>3</td><td>20.36</td><td>1</td><td>1.02E+06</td><td></td><td>(R)GDVVPK</td><td>(R)G D/V|V|P/K/D V N/A/A/I/A T I/K(T)</td><td></td><td>147.9</td><td>537.6393</td><td>1610.911</td><td>-0.0079</td><td>-4.9</td><td>5.96</td><td>58642</td><td>4.95</td><td>HUMAN</td><td>F5H5D3</td><td>Tubulin alpha-1C chain</td></tr>
<tr><td>389</td><td>dirMS_041712_NAR_30mM_minus2.15454.15733.2</td><td>2</td><td>20.35</td><td>0</td><td>3.22E+05</td><td></td><td>(R)AAPFSLE</td><td>(R)A/A|P/F/S/L/E/Y/R(V)</td><td></td><td>138.3</td><td>527.2782</td><td>1053.536</td><td>0.0128</td><td>12.1</td><td>6.04</td><td>33115.9</td><td>5.54</td><td>HUMAN</td><td>Q15181</td><td>Inorganic pyrophosphatase</td></tr>
<tr><td>390</td><td>dirMS_041712_NAR_50mM_minus2.9026.9027.3</td><td>3</td><td>20.35</td><td>1</td><td>9.01E+05</td><td></td><td>(K)TLQEVTI</td><td>(K)T/L|Q E/V/T/E/M/D S V K/R(Y)</td><td></td><td>119.9</td><td>512.5962</td><td>1535.773</td><td>0.0007</td><td>0.4</td><td>4.68</td><td>558007.1</td><td>5.44</td><td>HUMAN</td><td>P58107</td><td>Epiplakin</td></tr>
<tr><td>391</td><td>dirMS_041712_NAR_60mM_plus.15572.15573.3</td><td>3</td><td>20.34</td><td>1</td><td>2.74E+05</td><td></td><td>(K)YYKVEPI</td><td>(K)Y Y/K V E|P|L|D|F/G G T Q K(Q)</td><td></td><td>138</td><td>548.9468</td><td>1644.827</td><td>-0.001</td><td>-0.6</td><td>6.07</td><td>23480.1</td><td>5.04</td><td>HUMAN</td><td>H0Y9B6</td><td>Beta-hexosaminidase subunit beta (Fragment)</td></tr>
<tr><td>392</td><td>dirMS_041712_NAR_40mM_plus2.11715.11715.3</td><td>3</td><td>20.33</td><td>1</td><td>2.82E+05</td><td></td><td>(R)IVAPGK</td><td>(R)I/V|A|P G K G I L/A A/D/E S T G S/I/A K(R)</td><td></td><td>135.38</td><td>633.3568</td><td>1898.059</td><td>-0.0035</td><td>-1.8</td><td>6.07</td><td>30711.4</td><td>7.15</td><td>HUMAN</td><td>H3BPS8</td><td>Fructose-bisphosphate aldolase A (Fragment)</td></tr>
<tr><td>393</td><td>dirMS_041712_NAR_50mM_minus2.16427.16699.3</td><td>3</td><td>20.32</td><td>1</td><td>1.07E+06</td><td></td><td>(R)VTFSGLI</td><td>(R)V/T|F/S/G/L R D T\V|T|P G E L L K(A)</td><td></td><td>162.5</td><td>611.672</td><td>1833.012</td><td>-0.0102</td><td>-5.6</td><td>6.04</td><td>266161</td><td>5.68</td><td>HUMAN</td><td>E9PPU0</td><td>Epiplakin</td></tr>
<tr><td>394</td><td>dirMS_041712_NAR_30mM_plus2.12906.13152.2</td><td>2</td><td>20.31</td><td>0</td><td>6.06E+05</td><td></td><td>(R)LIVENLS</td><td>(R)L/I|V|E/N/L/S S/R(V)</td><td></td><td>121.58</td><td>515.7979</td><td>1030.589</td><td>-0.0006</td><td>-0.6</td><td>6</td><td>14608.1</td><td>10.7</td><td>HUMAN</td><td>B4DJK0</td><td>Serine/arginine-rich-splicing factor 5</td></tr>
<tr><td>395</td><td>dirMS_041712_NAR_120mM_minus.12329.12647.3</td><td>3</td><td>20.3</td><td>0</td><td>1.12E+06</td><td></td><td>(K)TFTAWC</td><td>(K)T F|T/A/W/C/N S/H L/R(K)</td><td>C:Carbamidomethylation</td><td>121.85</td><td>464.8871</td><td>1392.648</td><td>-0.001</td><td>-0.7</td><td>9.44</td><td>104488.6</td><td>5.36</td><td>HUMAN</td><td>B2RCS5</td><td>Actinin, alpha 2, isoform CRA_b</td></tr>
<tr><td>396</td><td>dirMS_041712_NAR_50mM_minus2.12223.12223.3</td><td>3</td><td>20.3</td><td>0</td><td>2.59E+06</td><td>M240m</td><td>(K)mLDAED</td><td>(K)m L|D|A/E/D I/V N T A/R/P D|E K(A)</td><td>m:Oxidized methionine</td><td>137.85</td><td>611.6283</td><td>1816.875</td><td>15.9958</td><td>0.5</td><td>4.1</td><td>105308.6</td><td>5.27</td><td>HUMAN</td><td>O43707</td><td>Alpha-actinin-4</td></tr>
<tr><td>397</td><td>dirMS_041712_NAR_30mM_plus2.21017.21316.2</td><td>2</td><td>20.3</td><td>0</td><td>6.89E+05</td><td></td><td>(K)IPDWFLI</td><td>(K)I/P|D|W/F/L/N/R(Q)</td><td></td><td>165.78</td><td>530.785</td><td>1060.557</td><td>0.0053</td><td>5</td><td>5.84</td><td>9767.1</td><td>11.41</td><td>HUMAN</td><td>J3JS69</td><td>40S ribosomal protein S18</td></tr>
<tr><td>398</td><td>dirMS_041712_NAR_60mM_plus.12913.13042.3</td><td>3</td><td>20.3</td><td>1</td><td>8.37E+05</td><td></td><td>(R)EFEVYGI</td><td>(R)E F/E/V/Y/G/P I/K/R(I)</td><td></td><td>124.55</td><td>413.2246</td><td>1237.658</td><td>0.0017</td><td>1.4</td><td>6.24</td><td>51612.9</td><td>9.98</td><td>HUMAN</td><td>P08621</td><td>U1 small nuclear ribonucleoprotein 70 kDa</td></tr>
<tr><td>399</td><td>dirMS_041712_NAR_60mM_plus.8419.8738.3</td><td>3</td><td>20.3</td><td>0</td><td>1.91E+06</td><td></td><td>(K)VSYGIGI</td><td>(K)V/S|Y/G/I/G D E E/H/D/Q E/G R(V)</td><td></td><td>102.5</td><td>564.2485</td><td>1690.73</td><td>0.0006</td><td>0.4</td><td>4.17</td><td>19395.5</td><td>5.38</td><td>HUMAN</td><td>G3V359</td><td>DNA-(apurinic or apyrimidinic site) lyase (Fragment)</td></tr>
<tr><td>400</td><td>dirMS_041712_NAR_40mM_minus2.9611.9951.3</td><td>3</td><td>20.29</td><td>0</td><td>4.90E+05</td><td></td><td>(R)VVIIGAG</td><td>(R)V/V|I|I|G/A G K/P A A\V/V|L Q T K(G)</td><td></td><td>139.28</td><td>555.3529</td><td>1664.047</td><td>-0.0028</td><td>-1.7</td><td>10</td><td>96827.3</td><td>5.58</td><td>HUMAN</td><td>E9PKU7</td><td>Neutral alpha-glucosidase AB</td></tr>
<tr><td>401</td><td>dirMS_041712_NAR_60mM_plus.18721.18870.3</td><td>3</td><td>20.28</td><td>0</td><td>5.48E+05</td><td></td><td>(K)GISDLAC</td><td>(K)G I|S/D/L/A/Q/H/Y/L M/R(A)</td><td></td><td>155.78</td><td>468.5804</td><td>1403.71</td><td>0.0167</td><td>11.9</td><td>6.74</td><td>56186.7</td><td>5.47</td><td>HUMAN</td><td>B4DUR8</td><td>T-complex protein 1 subunit gamma</td></tr>
<tr><td>402</td><td>dirMS_041712_NAR_60mM_plus.8439.8440.3</td><td>3</td><td>20.28</td><td>0</td><td>6.06E+06</td><td></td><td>(R)FACHSA</td><td>(R)F/A/C/H/S/A/S/L/T\V/R(N)</td><td>C:Carbamidomethylation</td><td>102.83</td><td>416.8777</td><td>1248.615</td><td>0.0032</td><td>2.5</td><td>9.75</td><td>23729.5</td><td>9.89</td><td>HUMAN</td><td>C9IZL7</td><td>Non-POU domain-containing octamer-binding protein (Fragment)</td></tr>
<tr><td>403</td><td>dirMS_041712_NAR_50mM_plus2.16288.16540.3</td><td>3</td><td>20.27</td><td>1</td><td>4.20E+06</td><td></td><td>(K)LLQDFFI</td><td>(K)L/L|Q/D/F/F/N/G K E L N K(S)</td><td></td><td>154.6</td><td>522.6137</td><td>1565.832</td><td>-0.0057</td><td>-3.6</td><td>6.07</td><td>45508.6</td><td>5.5</td><td>HUMAN</td><td>A8K7Q2</td><td>Heat shock cognate 71 kDa protein</td></tr>
<tr><td>404</td><td>dirMS_041712_NAR_20mM_minus.13493.13603.3</td><td>3</td><td>20.26</td><td>0</td><td>8.96E+05</td><td>M221m</td><td>(R)ALTVPEI</td><td>(R)A L\T\V/P E/L/T/Q/Q/m F/D/A/K(N)</td><td>m:Oxidized methionine</td><td>186.35</td><td>569.9577</td><td>1691.867</td><td>15.9913</td><td>-2.1</td><td>4.37</td><td>42831.5</td><td>4.86</td><td>HUMAN</td><td>A8K854</td><td>HCG1983504, isoform CRA_f</td></tr>
<tr><td>405</td><td>dirMS_041712_NAR_40mM_minus2.12726.13015.3</td><td>3</td><td>20.26</td><td>0</td><td>3.18E+06</td><td></td><td>(R)VSLDVN</td><td>(R)V/S|L/D/V/N H/F/A/P D/E L T V K(T)</td><td></td><td>159.18</td><td>595.3178</td><td>1783.922</td><td>0.0164</td><td>9.2</td><td>4.54</td><td>20576.9</td><td>9.84</td><td>HUMAN</td><td>F8WE04</td><td>Heat shock protein beta-1</td></tr>
<tr><td>406</td><td>dirMS_041712_NAR_30mM_plus2.13295.13624.2</td><td>2</td><td>20.26</td><td>0</td><td>1.80E+07</td><td></td><td>(R)IVLQIDN</td><td>(R)I/V|L|Q|I|D/N A/R(L)</td><td></td><td>123.83</td><td>521.3067</td><td>1041.605</td><td>0.001</td><td>1</td><td>5.84</td><td>20842.9</td><td>5.32</td><td>HUMAN</td><td>C9JM50</td><td>Keratin, type I cytoskeletal 19 (Fragment)</td></tr>
<tr><td>407</td><td>dirMS_041712_NAR_30mM_plus2.6091.6130.2</td><td>2</td><td>20.26</td><td>0</td><td>4.04E+05</td><td></td><td>(R)FSSSSGY</td><td>(R)F/S|S/S/S|G/Y/G/G G/S S R(V)</td><td></td><td>86.35</td><td>618.2674</td><td>1235.529</td><td>-0.0012</td><td>-1</td><td>8.75</td><td>62291.5</td><td>5.13</td><td>HUMAN</td><td>P35527</td><td>Keratin, type I cytoskeletal 9</td></tr>
<tr><td>408</td><td>dirMS_041712_NAR_30mM_minus2.18932.19162.2</td><td>2</td><td>20.25</td><td>0</td><td>6.26E+05</td><td></td><td>(K)LALFQAI</td><td>(K)L/A|L|F/Q/A/I|G/K(E)</td><td></td><td>158.72</td><td>480.7964</td><td>960.588</td><td>-0.0022</td><td>-2.2</td><td>8.75</td><td>266161</td><td>5.68</td><td>HUMAN</td><td>E9PPU0</td><td>Epiplakin</td></tr>
<tr><td>409</td><td>dirMS_041712_NAR_50mM_minus2.17568.17568.3</td><td>3</td><td>20.25</td><td>0</td><td>4.20E+05</td><td></td><td>(K)VAHALA</td><td>(K)V A H A L A E G L|G\V|I/A/C I/G E/K(L)</td><td>C:Carbamidomethylation</td><td>168.75</td><td>603.3247</td><td>1807.974</td><td>-0.0139</td><td>-7.7</td><td>5.4</td><td>31075.7</td><td>5.65</td><td>HUMAN</td><td>P60174</td><td>Triosephosphate isomerase</td></tr>
<tr><td>410</td><td>dirMS_041712_NAR_120mM_plus.8294.8316.4</td><td>4</td><td>20.24</td><td>2</td><td>3.75E+06</td><td></td><td>(K)KGAVAE</td><td>(K)K\G A\V\A|E|D|G/D/E L R T E/P E/A/K(K)</td><td></td><td>97.05</td><td>479.4909</td><td>1914.94</td><td>0.0014</td><td>0.8</td><td>4.36</td><td>19284.3</td><td>5.23</td><td>HUMAN</td><td>G3V3C7</td><td>DNA-(apurinic or apyrimidinic site) lyase (Fragment)</td></tr>
<tr><td>411</td><td>dirMS_041712_NAR_20mM_plus.9933.10184.2</td><td>2</td><td>20.24</td><td>0</td><td>1.43E+06</td><td></td><td>(K)LVSESSD</td><td>(K)L V|S|E/S/S D|V/L/P/K(-)</td><td></td><td>143.17</td><td>587.3192</td><td>1173.636</td><td>-0.005</td><td>-4.3</td><td>4.37</td><td>56607.4</td><td>5.37</td><td>HUMAN</td><td>F8VXB4</td><td>Keratin, type II cytoskeletal 8</td></tr>
<tr><td>412</td><td>dirMS_041712_NAR_50mM_plus2.14640.14702.3</td><td>3</td><td>20.24</td><td>0</td><td>5.16E+05</td><td></td><td>(K)MLDAED</td><td>(K)M/L|D/A|E/D/I/V/N T A R/P D/E K(A)</td><td></td><td>144.95</td><td>606.2972</td><td>1816.875</td><td>0.0025</td><td>1.4</td><td>4.1</td><td>105308.6</td><td>5.27</td><td>HUMAN</td><td>O43707</td><td>Alpha-actinin-4</td></tr>
<tr><td>413</td><td>dirMS_041712_NAR_20mM_plus.14136.14136.2</td><td>2</td><td>20.23</td><td>0</td><td>1.57E+06</td><td>M313m</td><td>(R)TVQSLEI</td><td>(R)T V/Q|S/L|E|I D/L/D S m R(N)</td><td>m:Oxidized methionine</td><td>170.15</td><td>761.8738</td><td>1506.747</td><td>15.9935</td><td>-0.9</td><td>4.03</td><td>43773.3</td><td>5.26</td><td>HUMAN</td><td>F8VZY9</td><td>Keratin, type I cytoskeletal 18</td></tr>
<tr><td>414</td><td>dirMS_041712_NAR_30mM_plus2.17456.17857.2</td><td>2</td><td>20.23</td><td>0</td><td>1.26E+06</td><td></td><td>(K)FANPFP</td><td>(K)F/A|N|P|F/P A A V/R(G)</td><td></td><td>145.62</td><td>545.2953</td><td>1089.584</td><td>-0.0007</td><td>-0.6</td><td>9.75</td><td>61363</td><td>8.73</td><td>HUMAN</td><td>C9JQS9</td><td>Propionyl-CoA carboxylase beta chain, mitochondrial</td></tr>
<tr><td>415</td><td>dirMS_041712_NAR_60mM_minus.15624.15828.3</td><td>3</td><td>20.22</td><td>1</td><td>6.80E+05</td><td></td><td>(R)FLQDYFI</td><td>(R)F L|Q|D/Y/F/D/G N/L K/R(Y)</td><td></td><td>147.7</td><td>505.924</td><td>1515.759</td><td>-0.0016</td><td>-1.1</td><td>5.96</td><td>55361.8</td><td>6.42</td><td>HUMAN</td><td>G5EA52</td><td>Protein disulfide isomerase family A, member 3, isoform CRA_b</td></tr>
<tr><td>416</td><td>dirMS_041712_NAR_120mM_plus.9819.9832.3</td><td>3</td><td>20.22</td><td>1</td><td>2.43E+05</td><td></td><td>(K)DTGKTP</td><td>(K)D T/G K/T P V\E|P/E/V/A/I/H R(I)</td><td></td><td>104.82</td><td>550.2928</td><td>1648.865</td><td>-0.0015</td><td>-0.9</td><td>5.45</td><td>5429.1</td><td>9.6</td><td>HUMAN</td><td>E5RIP1</td><td>40S ribosomal protein S20</td></tr>
<tr><td>417</td><td>dirMS_041712_NAR_40mM_plus2.18067.18152.3</td><td>3</td><td>20.22</td><td>0</td><td>1.19E+05</td><td></td><td>(K)LVLIGSN</td><td>(K)L/V|L/I/G S/N/H/S/L/P F L/K(F)</td><td></td><td>172.83</td><td>513.3081</td><td>1537.91</td><td>-0.0003</td><td>-0.2</td><td>8.76</td><td>253486.5</td><td>5.51</td><td>HUMAN</td><td>J3KNL6</td><td>Protein transport protein Sec16A</td></tr>
<tr><td>418</td><td>dirMS_041712_NAR_120mM_minus.13808.13808.2</td><td>2</td><td>20.21</td><td>1</td><td>4.32E+06</td><td></td><td>(K)FASFIDK</td><td>(K)F/A|S/F/I/D/K/V R(F)</td><td></td><td>130.17</td><td>541.7968</td><td>1082.599</td><td>-0.013</td><td>-12</td><td>8.75</td><td>19569.8</td><td>9.88</td><td>HUMAN</td><td>F8VRG4</td><td>Keratin, type II cytoskeletal 8 (Fragment)</td></tr>
<tr><td>419</td><td>dirMS_041712_NAR_30mM_minus2.19439.19659.2</td><td>2</td><td>20.21</td><td>0</td><td>1.46E+06</td><td></td><td>(R)LFDQAF</td><td>(R)L/F|D|Q|A/F/G L|P R(L)</td><td></td><td>162.58</td><td>582.315</td><td>1163.621</td><td>0.002</td><td>1.7</td><td>5.84</td><td>20576.9</td><td>9.84</td><td>HUMAN</td><td>F8WE04</td><td>Heat shock protein beta-1</td></tr>
<tr><td>420</td><td>dirMS_041712_NAR_20mM_plus.15505.15505.2</td><td>2</td><td>20.21</td><td>0</td><td>4.20E+05</td><td></td><td>(R)GVVDSE</td><td>(R)G V/V/D/S|E\D|I|P L N/L/S/R(E)</td><td></td><td>179.18</td><td>757.3956</td><td>1513.786</td><td>-0.0017</td><td>-1.1</td><td>4.03</td><td>74494.3</td><td>7.79</td><td>HUMAN</td><td>F5H897</td><td>Heat shock protein 75 kDa, mitochondrial</td></tr>
<tr><td>421</td><td>dirMS_041712_NAR_50mM_plus2.5643.5736.3</td><td>3</td><td>20.21</td><td>1</td><td>1.06E+06</td><td></td><td>(K)TAGPIAS</td><td>(K)T/A|G/P/I/A/S/A Q K Q/P A/G K(V)</td><td></td><td>91.9</td><td>475.5987</td><td>1424.786</td><td>-0.0041</td><td>-2.8</td><td>10</td><td>75259.2</td><td>9.59</td><td>HUMAN</td><td>B5MEG9</td><td>Microtubule-associated protein 4 (Fragment)</td></tr>
<tr><td>422</td><td>dirMS_041712_NAR_50mM_minus2.13422.13423.3</td><td>3</td><td>20.19</td><td>0</td><td>5.13E+05</td><td></td><td>(K)SLTNDW</td><td>(K)S/L|T/N/D W/E/D H/L/A V/K(H)</td><td></td><td>144.2</td><td>509.9192</td><td>1527.744</td><td>-0.0008</td><td>-0.5</td><td>4.53</td><td>85057.6</td><td>4.94</td><td>HUMAN</td><td>P07900</td><td>Heat shock protein HSP 90-alpha</td></tr>
<tr><td>423</td><td>dirMS_041712_NAR_20mM_minus.10829.11097.2</td><td>2</td><td>20.17</td><td>0</td><td>1.62E+06</td><td>M92m</td><td>(R)ImNTFS</td><td>(R)I/m|N|T|F|S/V/V|P S/P K(V)</td><td>m:Oxidized methionine</td><td>157.15</td><td>668.3513</td><td>1319.703</td><td>15.9925</td><td>-1.8</td><td>8.75</td><td>42831.5</td><td>4.86</td><td>HUMAN</td><td>A8K854</td><td>HCG1983504, isoform CRA_f</td></tr>
<tr><td>424</td><td>dirMS_041712_NAR_40mM_minus2.14623.14851.3</td><td>3</td><td>20.16</td><td>1</td><td>1.45E+06</td><td></td><td>(K)TFVNITP</td><td>(K)T F|V|N|I/T/P A E/V/G V L/V/G K D R(S)</td><td></td><td>171.8</td><td>639.0252</td><td>1915.065</td><td>-0.0037</td><td>-1.9</td><td>5.74</td><td>17630.6</td><td>11.62</td><td>HUMAN</td><td>I3L3D5</td><td>Profilin-1 (Fragment)</td></tr>
<tr><td>425</td><td>dirMS_041712_NAR_20mM_plus.17495.17667.2</td><td>2</td><td>20.16</td><td>0</td><td>2.22E+05</td><td></td><td>(K)LLYAFAE</td><td>(K)L L|Y|A|F|A E/A/T|V/P K(V)</td><td></td><td>192.97</td><td>661.8712</td><td>1322.736</td><td>-0.0003</td><td>-0.3</td><td>6</td><td>61363</td><td>8.73</td><td>HUMAN</td><td>C9JQS9</td><td>Propionyl-CoA carboxylase beta chain, mitochondrial</td></tr>
<tr><td>426</td><td>dirMS_041712_NAR_40mM_minus2.15009.15151.3</td><td>3</td><td>20.15</td><td>0</td><td>2.68E+06</td><td></td><td>(K)ALESPEI</td><td>(K)A/L|E|S/P/E/R P/F L/A I/L/G G/A K(V)</td><td></td><td>174.77</td><td>590.3353</td><td>1768.996</td><td>-0.0042</td><td>-2.4</td><td>6.19</td><td>41827.5</td><td>7</td><td>HUMAN</td><td>B7Z7A9</td><td>Phosphoglycerate kinase</td></tr>
<tr><td>427</td><td>dirMS_041712_NAR_50mM_plus2.8925.9007.3</td><td>3</td><td>20.15</td><td>0</td><td>2.21E+06</td><td></td><td>(K)GIVEFSG</td><td>(K)G I|V/E/F/S|G K/P A A/R(K)</td><td></td><td>114.52</td><td>411.2309</td><td>1231.679</td><td>-0.0012</td><td>-1</td><td>8.75</td><td>23729.5</td><td>9.89</td><td>HUMAN</td><td>C9IZL7</td><td>Non-POU domain-containing octamer-binding protein (Fragment)</td></tr>
<tr><td>428</td><td>dirMS_041712_NAR_30mM_plus2.18568.18568.2</td><td>2</td><td>20.14</td><td>0</td><td>1.90E+05</td><td></td><td>(-)SDVLELT</td><td>(-)S D V|L|E|L/T/D D N F E S R(I)</td><td></td><td>150.83</td><td>820.3752</td><td>1639.745</td><td>-0.0015</td><td>-0.9</td><td>3.77</td><td>13747.3</td><td>6.8</td><td>HUMAN</td><td>H7BZJ3</td><td>Thioredoxin (Fragment)</td></tr>
<tr><td>429</td><td>dirMS_041712_NAR_50mM_plus2.18953.19379.3</td><td>3</td><td>20.13</td><td>1</td><td>1.72E+06</td><td></td><td>(K)LIQLMEI</td><td>(K)L/I|Q|L/M/E/E I/M/A E K E/N K(T)</td><td></td><td>169.7</td><td>606.9845</td><td>1818.934</td><td>0.005</td><td>2.7</td><td>4.49</td><td>73202</td><td>9.19</td><td>HUMAN</td><td>C9JMU5</td><td>Probable ATP-dependent RNA helicase DDX17</td></tr>
<tr><td>430</td><td>dirMS_041712_NAR_30mM_minus2.12397.12397.2</td><td>2</td><td>20.12</td><td>0</td><td>2.17E+05</td><td></td><td>(R)TLSGME</td><td>(R)T L/S/G/M/E/S/Y/C V R(A)</td><td>C:Carbamidomethylation</td><td>121.88</td><td>651.795</td><td>1302.582</td><td>0.001</td><td>0.8</td><td>5.66</td><td>42755</td><td>7.81</td><td>HUMAN</td><td>B7Z2F4</td><td>T-complex protein 1 subunit delta</td></tr>
<tr><td>431</td><td>dirMS_041712_NAR_30mM_minus2.14061.14421.2</td><td>2</td><td>20.12</td><td>0</td><td>1.16E+06</td><td></td><td>(R)GLLEDV</td><td>(R)G L|L|E|D|V|Q/E/G R(T)</td><td></td><td>130.88</td><td>558.2882</td><td>1115.569</td><td>0</td><td>0</td><td>4.14</td><td>266161</td><td>5.68</td><td>HUMAN</td><td>E9PPU0</td><td>Epiplakin</td></tr>
</table>

432	dirMS_041712_NAR_50mM_minus2.12140.12289.3	3	20.12	0	2.59E+06	M240m	(K)mLDAEC	(K)m/L|D|A/E/D/I/V/N T A R P D/E K(A)	m:Oxidized methionine	137.85	611.6283	1816.875	15.9958	0.5	4.1	105308.6	5.27	HUMAN	O43707	Alpha-actinin-4
433	dirMS_041712_NAR_60mM_plus.12325.12325.3	3	20.1	1	9.36E+05		(R)LSFDKA	(R)L/S|F/D K/D/A M/V/A/R(A)		121.18	418.2158	1252.635	-0.0026	-2.1	5.96	35556.4	9.3	HUMAN	F2Z393	Transaldolase
434	dirMS_041712_NAR_60mM_minus.17094.17178.3	3	20.09	1	9.85E+05		(K)SIYGEKF	(K)S I|Y|G/E/K/F E/D/E/N F/I L/K(H)		155.53	611.3103	1831.911	0.0051	2.8	4.41	13378.8	6.27	HUMAN	C9J5S7	Peptidyl-prolyl cis-trans isomerase
435	dirMS_041712_NAR_30mM_plus2.23481.23641.2	2	20.09	0	4.18E+06		(R)IAIYELLF	(R)I/A|I|Y/E|L|L/F/K(E)		191.57	555.3331	1109.661	-0.0016	-1.4	6	19924.8	9.99	HUMAN	F6U211	40S ribosomal protein S10
436	dirMS_041712_NAR_50mM_plus2.18208.18443.3	3	20.09	1	6.23E+05		(K)LLEPVLL	(K)L/L|E|P/V/L/L/L/G K E R(F)		164.48	460.6256	1379.862	0.0002	0.1	6.14	16559.1	10.36	HUMAN	P62249	40S ribosomal protein S16
437	dirMS_041712_NAR_60mM_plus2.17879.17930.3	3	20.09	1	3.01E+05		(R)KGFSEGI	(R)K G F S\E G\L\W\E\I/E\N/N\P\T V K(A)		150.67	650.0017	1947.981	0.0095	4.9	4.79	23020.5	4.53	HUMAN	A8K8G0	Hepatoma-derived growth factor
438	dirMS_041712_NAR_60mM_plus.10432.10898.3	3	20.08	1	1.15E+07		(R)VKYETEL	(R)V/K\Y E T\E|L/A/M/R(Q)		113.28	413.8859	1239.64	0.003	2.4	6.11	43773.3	5.26	HUMAN	F8VZY9	Keratin, type I cytoskeletal 18
439	dirMS_041712_NAR_30mM_minus2.11313.11583.2	2	20.05	0	9.49E+05		(K)LVSESSD	(K)L/V|S/E/S/S D|V|L/P/K(-)		115.78	587.3211	1173.636	-0.0012	-1	4.37	56607.4	5.37	HUMAN	F8VXB4	Keratin, type II cytoskeletal 8
440	dirMS_041712_NAR_60mM_minus.15810.15810.3	3	20.05	1	3.06E+05		(R)IDTRNEI	(R)I/D/T/R\N E L|E|S|Y/A Y/S/L/K(N)		148.1	601.3049	1801.897	0.0035	1.9	4.68	72445.7	5.07	HUMAN	P11021	78 kDa glucose-regulated protein
441	dirMS_041712_NAR_20mM_plus.10541.10541.2	2	20.05	0	3.12E+05		(K)VLQATV	(K)V L|Q/A|T/V/V/A/V/G/S G S K(G)		145.67	658.3823	1315.758	-0.0007	-0.5	8.72	10803.5	9.82	HUMAN	B8ZZL8	10 kDa heat shock protein, mitochondrial
442	dirMS_041712_NAR_50mM_plus2.19783.20199.3	3	20.05	0	4.80E+06		(K)ALESPEF	(K)A/L|E|S P E R P/F L|A/I/L/G/G A K(V)		173.65	590.3344	1768.996	-0.0069	-3.9	6.19	41827.5	7	HUMAN	B7Z7A9	Phosphoglycerate kinase
443	dirMS_041712_NAR_50mM_plus2.18223.18545.3	3	20.04	0	5.14E+05	M195m	(R)LHFFmP	(R)L H\F\F m|P G|F|A|P/L/T S R(G)	m:Oxidized methionine	164.23	546.2803	1620.835	15.9908	-2.5	9.75	42230.8	4.77	HUMAN	A6NKZ8	Putative tubulin beta chain-like protein ENSP00000290377
444	dirMS_041712_NAR_120mM_plus.9369.9407.4	4	20.03	2	1.18E+06		(K)LTDCVVI	(K)L/T/D/C/V/V/M R D|P A/S K/R(S)	C:Carbamidomethylation	102.65	412.7134	1647.831	0.0012	0.8	8.75	37486.1	9.03	HUMAN	P22626	Heterogeneous nuclear ribonucleoproteins A2/B1
445	dirMS_041712_NAR_40mM_plus2.18294.18294.3	3	20.01	0	3.29E+05		(R)EAVELPI	(R)E A/V/E/L/P L T/H F E/L V K(Q)		173.87	563.6336	1688.889	-0.0032	-1.9	4.75	47479.5	5.09	HUMAN	P43686	26S protease regulatory subunit 6B
446	dirMS_041712_NAR_50mM_plus2.17271.17453.3	3	20	0	6.18E+05		(K)VLISSLQ	(K)V/L|I|S/S/L/Q/D C L/H/G I E/S/K(S)	C:Carbamidomethylation	159.32	600.3189	1798.937	0.0054	3	5.32	52440.1	6	HUMAN	F8WJN3	Cleavage and polyadenylation-specificity factor subunit 6
447	dirMS_041712_NAR_60mM_minus.10360.10502.3	3	19.99	1	6.05E+05	M88m	(K)DVDEAY	(K)D V|D|E/A/Y/m/N/K/V/E L E/S R(L)	m:Oxidized methionine	118.37	605.2791	1797.832	15.9904	-2.5	4.18	13588.1	4.66	HUMAN	F8VP67	Keratin, type II cytoskeletal 8 (Fragment)
448	dirMS_041712_NAR_20mM_plus.8329.8362.2	2	19.99	0	8.56E+04		(R)LAPEYA	(R)L/A|P/E/Y/E/A/A/A T/R(L)		127.75	596.3035	1191.6	-0.0007	-0.6	4.53	55361.8	6.42	HUMAN	G5EA52	Protein disulfide isomerase family A, member 3, isoform CRA_b
449	dirMS_041712_NAR_50mM_plus2.12012.12160.3	3	19.99	0	2.97E+05		(R)VVVLMC	(R)V/V|V|L|M/G S T/S/D L/G H/C E K(I)	C:Carbamidomethylation	131.5	577.9512	1731.84	-0.0014	-0.8	5.32	37681.5	5.93	HUMAN	D6RF62	Phosphoribosylaminoimidazole carboxylase
450	dirMS_041712_NAR_30mM_minus2.16081.16323.2	2	19.98	0	3.06E+05		(R)GEVTAA	(R)G E|V|T|A|A|D\L/F/N\S R(V)		141.75	640.3185	1279.628	0.002	1.6	4.37	266161	5.68	HUMAN	E9PPU0	Epiplakin
451	dirMS_041712_NAR_30mM_plus2.18829.19255.2	2	19.98	0	3.29E+06		(R)QAQEYE	(R)Q A|Q|E/Y/E/A/L/L/N/I/K(V)		153.28	710.3766	1419.748	-0.0019	-1.3	4.53	43773.3	5.26	HUMAN	F8VZY9	Keratin, type I cytoskeletal 18
452	dirMS_041712_NAR_120mM_minus.13798.14077.2	2	19.97	1	4.39E+06		(K)FASFIDK	(K)F/A|S|F/I/D/K V/R(F)		130.18	541.7968	1082.599	-0.013	-12	8.75	19569.8	9.88	HUMAN	F8VRG4	Keratin, type II cytoskeletal 8 (Fragment)
453	dirMS_041712_NAR_50mM_minus2.15782.15782.3	3	19.97	0	1.29E+05		(K)FTISDHP	(K)F/T|I/S/D/H P Q P I D|P L L K(N)		157.77	574.3122	1720.927	-0.0048	-2.8	5.21	138084.9	5.52	HUMAN	Q86VP6	Cullin-associated NEDD8-dissociated protein 1
454	dirMS_041712_NAR_60mM_minus.16663.17032.3	3	19.97	0	2.45E+06		(R)DGLAFN	(R)D G|L/A/F/N/A/L/I/H/R(H)		154.08	409.5576	1226.664	-0.0058	-4.7	6.74	146974	5.55	HUMAN	C9JRP8	Spectrin beta chain, non-erythrocytic 4
455	dirMS_041712_NAR_20mM_plus.10864.10864.2	2	19.97	0	2.19E+05	M33m	(K)VAGmD	(K)V A|G/m/D|V|E L/T V/E/E/R(N)	m:Oxidized methionine	147.28	732.3555	1447.71	15.994	-0.6	4	10949.3	5.28	HUMAN	B4DJF2	14-3-3 protein epsilon
456	dirMS_041712_NAR_30mM_plus2.7466.7467.2	2	19.97	0	4.86E+05		(K)SLAAEEE	(K)S L|A|A/E|E/E/A A/R(Q)		93.58	523.7605	1046.511	0.0024	2.3	4.25	533778	5.74	HUMAN	Q15149	Plectin
457	dirMS_041712_NAR_60mM_plus.9986.9987.2	2	19.97	0	2.35E+05		(R)GCHLLV	(R)G C|H|L|L/V|A/T|P G R(L)	C:Carbamidomethylation	110.13	590.8148	1180.626	-0.0032	-2.7	9.75	73386.6	8.73	HUMAN	B4DXX7	ATP-dependent RNA helicase DDX3Y
458	dirMS_041712_NAR_20mM_plus.11939.12109.2	2	19.96	0	2.63E+05		(K)YGEPGE	(K)Y/G|E|P/G/E\V/F I/N K(G)		156.1	626.8223	1252.621	0.0165	13.2	4.53	76262.2	9.53	HUMAN	P23246	Splicing factor, proline- and glutamine-rich
459	dirMS_041712_NAR_30mM_plus2.15749.16120.2	2	19.96	0	2.79E+06		(R)VLQALE	(R)V/L|Q|A/L/E/G L/K(M)		134.72	485.7997	970.593	-0.001	-1.1	5.97	16060.2	10.31	HUMAN	P39019	40S ribosomal protein S19
460	dirMS_041712_NAR_40mM_plus2.18216.18216.3	3	19.96	0	1.04E+05		(K)VLSLPKP	(K)V/L|S L|P K P T I|I/L/S/A/Y Q R(K)		173.45	600.3613	1799.079	-0.0096	-5.3	9.99	80080.5	5.54	HUMAN	J3KMX1	Nuclear pore complex protein Nup88 (Fragment)
461	dirMS_041712_NAR_50mM_plus2.15980.16317.3	3	19.96	1	1.50E+06		(K)ILLAELEC	(K)I/L|L/A/E/L/E/Q L/K/G Q/G K(S)		152.23	513.9741	1539.911	-0.0027	-1.8	6.14	49709.6	5.19	HUMAN	B0YJC4	Vimentin
462	dirMS_041712_NAR_50mM_plus2.16192.16396.3	3	19.96	0	2.89E+05		(K)ACQSIYF	(K)A C|Q/S|I/Y/P/L/H D/V F V R(K)	C:Carbamidomethylation	154.4	568.9562	1704.853	0.0014	0.8	6.79	16650	9.47	HUMAN	D6R9B6	40S ribosomal protein S3a
463	dirMS_041712_NAR_30mM_plus2.14237.14259.2	2	19.95	0	9.98E+05		(R)MGQMA	(R)M/G Q|M|A|M|G/G/A M|G/I N N R(G)		127.32	769.8376	1538.67	-0.0015	-1	9.5	43979.5	8.92	HUMAN	F5GYZ3	Non-POU domain-containing octamer-binding protein
464	dirMS_041712_NAR_50mM_plus2.20887.21236.3	3	19.95	0	8.86E+06		(R)LHFFMP	(R)L H\F\F\M|P/G|F|A|P|L/T S R(G)		181.52	540.9498	1620.835	-0.0007	-0.4	9.75	42230.8	4.77	HUMAN	A6NKZ8	Putative tubulin beta chain-like protein ENSP00000290377
465	dirMS_041712_NAR_20mM_minus.8498.8498.2	2	19.94	0	7.57E+04		(R)IIYGGSV	(R)I I|Y/G G S/V/T/G A T C K(E)	C:Carbamidomethylation	134.28	663.8403	1326.672	0.0011	0.8	8.59	31075.7	5.65	HUMAN	P60174	Triosephosphate isomerase
466	dirMS_041712_NAR_30mM_minus2.9625.10182.2	2	19.94	0	7.15E+06		(K)TAVCDIF	(K)T A/V|C/D|I|P|P/R(G)	C:Carbamidomethylation	107.48	514.7641	1028.519	0.0016	1.5	5.5	50199.4	4.75	HUMAN	A6NNZ2	Tubulin beta-8 chain-like protein LOC260334
467	dirMS_041712_NAR_20mM_plus.18012.18012.2	2	19.94	0	1.91E+05		(K)GADFLV	(K)G A D|F|L|V|T E V\E/N G G S L/G S K(K)		195.25	890.4414	1779.876	-0.0004	-0.2	4.14	30947.9	6.47	HUMAN	H3BQ34	Pyruvate kinase
468	dirMS_041712_NAR_30mM_plus2.7333.7333.2	2	19.94	1	2.13E+05		(R)YSGGNY	(R)Y S|G/G/N/Y R D N|Y|D|N(-)		93.03	719.2864	1437.567	-0.001	-0.7	4.21	17170.1	8.86	HUMAN	P98179	Putative RNA-binding protein 3
469	dirMS_041712_NAR_60mM_plus.9269.9452.3	3	19.93	0	4.31E+06		(K)QEYDES	(K)Q E/Y/D E/S/G/P/S/I/V H/R(K)		107.12	506.2392	1516.703	0.0004	0.3	4.65	123097.1	5.83	HUMAN	A5A3E0	POTE ankyrin domain family member F
470	dirMS_041712_NAR_30mM_minus2.10678.10781.2	2	19.92	0	7.70E+05		(K)TVSPALI	(K)T V|S|P/A/L/I/S/R(F)		111.87	472.2815	943.557	-0.0014	-1.5	9.41	50425	5.53	HUMAN	H0YH27	Cullin-associated NEDD8-dissociated protein 1 (Fragment)
471	dirMS_041712_NAR_30mM_minus2.13154.13194.2	2	19.92	0	2.97E+05		(R)GATQQI	(R)G A/T|Q|Q|I|L|D|E/A/E R(S)		125.18	665.833	1330.66	-0.001	-0.8	4.14	57204.9	6.01	HUMAN	F5GWF6	T-complex protein 1 subunit beta
472	dirMS_041712_NAR_120mM_plus.16995.16995.3	3	19.92	1	5.91E+05		(K)YYVTIID	(K)Y/Y|V/T/I I/D/A/P G H R D F I K(N)		142.13	636.6716	1908.001	-0.0012	-0.6	6.75	12137.8	9.3	HUMAN	A6PW80	Elongation factor 1-alpha 1 (Fragment)
473	dirMS_041712_NAR_20mM_minus.12230.12536.2	2	19.91	0	9.01E+05	M185m	(K)LAVNm\	(K)L/A|V|N/m|V/P F/P/R(L)	m:Oxidized methionine	171.88	580.3173	1143.634	15.993	-1.6	9.75	42230.8	4.77	HUMAN	A6NKZ8	Putative tubulin beta chain-like protein ENSP00000290377
474	dirMS_041712_NAR_50mM_plus2.7088.7104.3	3	19.91	0	1.74E+05		(R)SAEEEA	(R)S A|E|E E/A/A/D/L/P T K/P T K(I)		106.92	529.6012	1586.791	-0.0018	-1.1	4.41	18924.5	6.86	HUMAN	Q8WW12	PEST proteolytic signal-containing nuclear protein
475	dirMS_041712_NAR_20mM_plus.10781.10971.2	2	19.9	0	7.16E+05		(K)IGGIGTV	(K)I/G|G|I|G|T/V|P V/G/R(V)		147.43	513.3057	1025.61	-0.0061	-5.9	9.75	50482.3	9.31	HUMAN	P68104	Elongation factor 1-alpha 1
476	dirMS_041712_NAR_50mM_plus2.14834.14954.3	3	19.9	0	5.02E+05		(K)TTISVAH	(K)T/T|I/S/V/A H/L/L/A A/R(Q)		146.45	418.2499	1252.737	-0.002	-1.6	9.44	34143.2	10.07	HUMAN	B4DT23	Protein FAM98A
477	dirMS_041712_NAR_30mM_minus2.7395.7395.2	2	19.88	0	1.14E+05		(K)EAEGAP	(K)E/A/E/G A|P Q/V/E/A/G\K(R)		94.65	593.2913	1185.575	0.0007	0.6	4.25	54219.9	5.66	HUMAN	C9J813	Caldesmon (Fragment)
478	dirMS_041712_NAR_60mM_plus.11647.11781.3	3	19.88	1	1.45E+06		(R)VLTVINC	(R)V/L|T|V/I/N Q/T/Q/K/E N L R(K)		118.62	552.6521	1655.944	-0.0021	-1.3	8.72	10815.7	10.72	HUMAN	F2Z388	60S ribosomal protein L35
479	dirMS_041712_NAR_120mM_minus.10163.10163.3	3	19.87	1	1.07E+06		(K)FRDGEE	(K)F R D G E E|A|G|A/Y/D/G P R(T)		110.25	551.5928	1652.766	-0.0025	-1.5	4.32	55361.8	6.42	HUMAN	G5EA52	Protein disulfide isomerase family A, member 3, isoform CRA_b
480	dirMS_041712_NAR_20mM_plus.15630.15630.2	2	19.87	0	3.47E+05		(K)VGINYQ	(K)V G I N Y|Q|P/P T V/V|P G G/D L/A/K(V)		179.05	912.9952	1824.985	-0.0023	-1.3	5.8	49012.3	4.9	HUMAN	A8MUB1	Tubulin alpha-4A chain
481	dirMS_041712_NAR_30mM_plus2.19925.19925.2	2	19.87	0	7.84E+05		(K)MSIYQA	(K)M/S|I|Y/Q A M W/K(G)		158.77	579.2783	1157.548	0.0011	1	8.34	266161	5.68	HUMAN	E9PPU0	Epiplakin
482	dirMS_041712_NAR_20mM_plus.10529.10603.2	2	19.86	0	3.12E+05		(K)VLQATV	(K)V L|Q|A|T|V/V/A/V|G S\G S K(G)		145.67	658.3823	1315.758	-0.0007	-0.5	8.72	10803.5	9.82	HUMAN	B8ZZL8	10 kDa heat shock protein, mitochondrial
483	dirMS_041712_NAR_50mM_plus2.9373.9476.3	3	19.86	1	5.41E+05		(K)GILAADE	(K)G I|L|A/A/D/E/S/T/G S I A K/R(L)		116.73	496.9389	1488.802	0.0005	0.3	6.07	30711.4	7.15	HUMAN	H3BPS8	Fructose-bisphosphate aldolase A (Fragment)
484	dirMS_041712_NAR_20mM_plus.8889.8890.2	2	19.85	0	1.30E+05		(K)VLEEAN	(K)V L|E|E|A|N/Q|A|I|N|P K(L)		131.6	663.353	1325.706	-0.0072	-5.5	4.53	73202	9.19	HUMAN	C9JMU5	Probable ATP-dependent RNA helicase DDX17
485	dirMS_041712_NAR_30mM_plus2.9056.9056.2	2	19.84	0	3.94E+05		(K)IYIDSNN	(K)I/Y|I/D/S/N N/N|P E R(F)		101.15	667.8205	1334.633	0.0002	0.2	4.37	193379.8	5.48	HUMAN	Q00610	Clathrin heavy chain 1
486	dirMS_041712_NAR_120mM_plus.10634.10783.3	3	19.83	1	7.19E+05		(R)FYKNEG	(R)F/Y/K N E/G/G|T/W/S/V E/K(V)		109.07	515.5841	1544.738	-0.0002	-0.2	6.14	39066	6.61	HUMAN	A6PVW9	Selenium binding protein 1 (Fragment)
487	dirMS_041712_NAR_60mM_minus.15091.15226.3	3	19.82	1	1.31E+06		(R)ELISNAS	(R)E L|I/S/N/A/S|D|A L/D/K I R(L)		144.2	515.6165	1544.828	0.0071	4.6	4.56	5582.4	4.81	HUMAN	F8W026	Endoplasmin (Fragment)
488	dirMS_041712_NAR_30mM_plus2.13709.13784.2	2	19.82	0	7.16E+05		(R)YALYDA	(R)Y/A|L|Y/D|A T/Y/E/T K(E)		125	669.317	1337.626	0.0007	0.6	4.37	22956	8.8	HUMAN	E9PK25	Cofilin-1
489	dirMS_041712_NAR_30mM_plus2.19579.19828.2	2	19.82	0	2.31E+05		(K)LLQDFFF	(K)L/L|Q/D/F|F/N/G R(D)		156.9	555.2902	1109.574	-0.0007	-0.6	5.84	60126	5.44	HUMAN	E7EP94	Heat shock 70 kDa protein 1A/1B
490	dirMS_041712_NAR_40mM_plus2.7744.7744.2	2	19.82	0	8.63E+04		(R)FGQGGA	(R)F G/Q/G/G/A|G|P V/G G Q G P R(G)		105.47	671.3368	1341.666	0.0005	0.4	9.75	76262.2	9.53	HUMAN	P23246	Splicing factor, proline- and glutamine-rich
491	dirMS_041712_NAR_60mM_plus.6446.6468.3	3	19.82	1	2.23E+05		(K)LEEAEKA	(K)L/E|E|A E/K/A/A/D/E|S E/R(G)		92.37	492.8967	1476.681	-0.0057	-3.9	4.14	31923.6	4.85	HUMAN	B7Z596	Tropomyosin alpha-1 chain
492	dirMS_041712_NAR_30mM_plus2.22641.22743.2	2	19.81	0	8.66E+05		(R)IDLAVLL	(R)I/D|L|A|V|L|L/G/K(T)		176.32	471.3029	941.603	-0.0045	-4.7	5.84	113668.7	6.2	HUMAN	F8W726	Ubiquitin-associated protein 2-like
493	dirMS_041712_NAR_20mM_plus.10586.10586.2	2	19.8	0	5.11E+04		(R)ELAEDG	(R)E L|A|E/D/G Y/S/G V/E V R(V)		145.7	712.3373	1423.67	-0.0026	-1.9	4	13054.7	9.59	HUMAN	E9PJH4	40S ribosomal protein S3
494	dirMS_041712_NAR_40mM_plus2.18486.18486.3	3	19.79	1	3.08E+04		(K)LLLEGVE	(K)L/L|L/E/G V E N K L I/P G L/K(I)		175.08	546.0066	1636.004	0.0009	0.5	6.14	59384	7.79	HUMAN	B7Z3Z9	Long-chain-fatty-acid--CoA ligase 1
495	dirMS_041712_NAR_60mM_plus.14451.14491.3	3	19.79	1	4.71E+05		(K)ISNYGW	(K)I/S|N/Y/G W/D/Q/S D/K F V/K(I)		132.32	562.9416	1686.812	-0.002	-1.2	5.96	21341.9	7.77	HUMAN	Q5R370	Calcyclin binding protein
496	dirMS_041712_NAR_120mM_minus.8814.8997.3	3	19.78	0	9.66E+05		(K)HTGPNS	(K)H T G\P N\S/P/D|T|A/N/D/G F V/R(L)		103.18	562.2599	1684.767	-0.0022	-1.3	5.21	18511.8	5.62	HUMAN	D6R9T0	Heterogeneous nuclear ribonucleoprotein H (Fragment)
497	dirMS_041712_NAR_30mM_plus2.21465.21465.2	2	19.78	0	1.58E+05		(R)VFIGNLN	(R)V F|I/G N/L N T/L/V V/K(K)		167.38	658.8983	1316.794	-0.0043	-3.3	8.72	15791.7	9.22	HUMAN	B4DSU6	Heterogeneous nuclear ribonucleoproteins C1/C2
498	dirMS_041712_NAR_30mM_plus2.8028.8028.2	2	19.78	0	2.20E+05		(K)IAVAAQ	(K)I/A|V|A|A Q N C Y/K(V)	C:Carbamidomethylation	96.05	569.2905	1137.572	0.0016	1.4	8.59	31075.7	5.65	HUMAN	P60174	Triosephosphate isomerase
499	dirMS_041712_NAR_50mM_plus2.15744.15766.3	3	19.78	1	3.70E+06		(K)ISDSEGF	(K)I/S|D/S|E/G F/K/A/N L/S/L L R(R)		151.2	550.6331	1649.886	-0.001	-0.6	6.07	76262.2	9.53	HUMAN	P23246	Splicing factor, proline- and glutamine-rich
500	dirMS_041712_NAR_50mM_plus2.9807.9881.3	3	19.78	1	2.67E+03		(R)TAGVKV	(R)T/A/G V/K V/E T/T E/D|L/V/A/K(L)		120.5	520.9473	1560.848	-0.0206	-13.2	4.68	28071.3	8.6	HUMAN	P38117	Electron transfer flavoprotein subunit beta
501	dirMS_041712_NAR_60mM_plus.13655.13655.3	3	19.78	1	5.75E+05		(R)ISVVYNE	(R)I/S|V/Y/Y N E A T G/G K Y V P R(A)		128.33	606.3113	1815.923	-0.0035	-1.9	8.43	57549.9	4.39	HUMAN	E9PBJ4	Tubulin beta chain
502	dirMS_041712_NAR_30mM_minus2.16093.16093.2	2	19.77	0	1.16E+05		(K)EQFLDG	(K)E/Q|F/L/D/G D G W/T S R(W)		141.08	705.8174	1410.628	-0.0009	-0.6	4.03	28545.1	4.5	HUMAN	K7EJB9	Calreticulin (Fragment)
503	dirMS_041712_NAR_50mM_minus2.8667.8971.3	3	19.77	1	3.08E+06	M88m	(K)DVDEAY	(K)D V|D|E|A/Y/m N K/V/E/L/E/S R(L)	m:Oxidized methionine	118	605.2717	1797.832	15.9682	-14.7	4.18	13588.1	4.66	HUMAN	F8VP67	Keratin, type II cytoskeletal 8 (Fragment)
504	dirMS_041712_NAR_40mM_plus2.12859.13027.3	3	19.77	0	3.08E+05		(K)VVEIAPA	(K)V/V|E|I/A/P/A A/H/L D/P Q L R(T)		142.65	543.6417	1628.912	-0.0013	-0.8	5.32	58266.4	9.46	HUMAN	B4DN00	Pyruvate carboxylase, mitochondrial
505	dirMS_041712_NAR_40mM_plus2.17146.17314.3	3	19.76	1	3.36E+05		(K)VIAATNI	(K)V/I|A/A/T/N R V/D|I|L/D/P A L L R(S)		168.1	617.3636	1850.086	-0.0096	-5.2	5.93	32827.5	5.92	HUMAN	E9PKD5	26S protease regulatory subunit 6A (Fragment)
506	dirMS_041712_NAR_20mM_plus.15481.15855.2	2	19.75	0	5.10E+05		(R)GVVDSE	(R)G V|V|D|S|E/D|I|P L N/L/S R(E)		179.4	757.3936	1513.786	-0.0017	-1.1	4.03	74494.3	7.79	HUMAN	F5H897	Heat shock protein 75 kDa, mitochondrial
507	dirMS_041712_NAR_20mM_plus.9334.9334.2	2	19.75	0	1.86E+05		(K)SVTNEI	(K)S V/T\N E/D\V/T Q/E E/L/G G A/K(T)		136	838.9014	1676.797	-0.0018	-1.1	4	61363	8.73	HUMAN	C9JQS9	Propionyl-CoA carboxylase beta chain, mitochondrial
508	dirMS_041712_NAR_40mM_plus2.12886.13195.3	3	19.75	0	9.15E+05		(R)VVIIGAC	(R)V/V|V/|I|I/G/A G K/P A A\V/V|L Q/T K(G)		143.22	555.3527	1664.047	-0.0034	-2	10	96827.3	5.58	HUMAN	E9PKU7	Neutral alpha-glucosidase AB
509	dirMS_041712_NAR_50mM_plus2.16024.16024.3	3	19.75	1	1.20E+06		(K)CEFQDA	(K)C E/F Q D A\Y/V/L/L/S E K K(I)	C:Carbamidomethylation	152.32	577.2866	1729.847	-0.0013	-0.8	4.68	61224.7	5.7	HUMAN	P10809	60 kDa heat shock protein, mitochondrial
510	dirMS_041712_NAR_20mM_plus2.6028.6044.2	2	19.74	0	4.93E+05		(R)QEMQE	(R)Q E|M|Q|E|V/Q/S/S R(S)		85.92	611.2799	1221.553	-0.0003	-0.2	4.53	37486.1	9.03	HUMAN	P22626	Heterogeneous nuclear ribonucleoproteins A2/B1
511	dirMS_041712_NAR_40mM_minus2.14295.14295.3	3	19.73	1	1.61E+05		(K)TGISDVF	(K)T G|I/S D V F A K/N D|L/A/V/V D/V R(I)		168.48	640.3454	1919.023	-0.0016	-0.8	4.43	76670.1	4.6	HUMAN	P19338	Nucleolin
512	dirMS_041712_NAR_50mM_plus2.20243.20243.3	3	19.73	1	1.43E+06		(R)TLAEIAK	(R)T L/A/E/I/A K/A/E L D G T/I/L/K(S)		175.75	562.6601	1685.968	-0.0026	-1.6	4.68	58390.7	6.26	HUMAN	Q8WXF1	Paraspeckle component 1
513	dirMS_041712_NAR_50mM_plus2.9854.9854.3	3	19.73	1	2.58E+05		(K)ALDLDS	(K)A L/D L/D/S S/C K E|A|A D/G/Y Q/R(C)	C:Carbamidomethylation	119.3	633.6225	1898.855	-0.0019	-1	4.23	60404.6	6.4	HUMAN	F5H0T1	Stress-induced-phosphoprotein 1
514	dirMS_041712_NAR_60mM_plus.16202.16383.3	3	19.73	0	5.95E+05		(K)WIGLDL	(K)W|I|G L/D/L/S/N/G K/P R(D)		142.1	452.5864	1355.743	0.0016	1.2	8.75	44227.3	7.81	HUMAN	E7EQR6	T-complex protein 1 subunit alpha
515	dirMS_041712_NAR_50mM_minus2.9551.9864.3	3	19.72	1	2.86E+06		(R)MQKEIT	(R)M Q\K\E I T/A/L|A|P S/T M/K(I)		123.52	516.9426	1548.812	0.0008	0.5	8.35	32616.5	5.18	HUMAN	A6NL76	Actin, alpha skeletal muscle
516	dirMS_041712_NAR_50mM_plus2.12452.12452.3	3	19.72	0	3.93E+05		(R)LDDTVH	(R)L/D/D T/V H V/V/I/A/T/P G/R(I)		133.35	498.2741	1492.812	-0.0041	-2.7	5.21	54815.2	9.17	HUMAN	P26196	Probable ATP-dependent RNA helicase DDX6
517	dirMS_041712_NAR_60mM_plus.9165.9204.3	3	19.71	1	2.99E+05		(R)FVKQDC	(R)F V/K Q D|Q|V/C/I|A/R(L)	C:Carbamidomethylation	106	455.2428	1363.715	-0.0012	-0.9	8.75	53458.1	5.47	HUMAN	H3BR35	Eukaryotic peptide chain release factor GTP-binding subunit ERF3A (Fragment)
518	dirMS_041712_NAR_30mM_plus2.10701.10909.2	2	19.7	0	8.88E+05		(R)AEAESM	(R)A E|A|E|S|M/Y/Q/I/K(Y)		109.97	585.2792	1169.551	0.0004	0.4	4.53	37296.3	5.3	HUMAN	F8VU64	Keratin, type II cytoskeletal 8
519	dirMS_041712_NAR_30mM_plus2.5469.5469.2	2	19.7	0	3.75E+04		(K)SAAQAA	(K)S A/A/Q/A/A/A|Q|T/N S N A A|G K(Q)		82.75	730.8568	1460.709	-0.0025	-1.7	8.47	45021.6	8.74	HUMAN	Q8NC51	Plasminogen activator inhibitor 1 RNA-binding protein
520	dirMS_041712_NAR_30mM_minus2.16236.16236.2	2	19.69	0	3.06E+05		(R)GEVTAA	(R)G E/V/T/A/A/D L|F N S R(V)		141.75	640.3185	1279.628	0.002	1.6	4.37	266161	5.68	HUMAN	E9PPU0	Epiplakin
521	dirMS_041712_NAR_40mM_minus2.14609.14863.3	3	19.68	1	1.45E+06		(K)TFVNITP	(K)T F|V|N|I/T/P A E V G V L/V/G\K D/R(S)		171.8	639.0252	1915.065	-0.0037	-1.9	5.74	17630.6	11.62	HUMAN	I3L3D5	Profilin-1 (Fragment)
522	dirMS_041712_NAR_50mM_minus2.5839.5843.3	3	19.68	0	1.31E+05		(R)AGAAG	(R)A/G|A|A|G|G/P E/E|E A E K/P V K(T)		95.32	513.9233	1539.765	-0.0096	-6.2	4.49	18924.5	6.86	HUMAN	Q8WW12	PEST proteolytic signal-containing nuclear protein
523	dirMS_041712_NAR_120mM_plus.20439.20673.3	3	19.68	1	9.13E+05		(R)RFDEILE	(R)R F D E|V|L|E|A|S/D/G|I/M/V/A R(G)		160.97	607.9762	1821.916	-0.0023	-1.3	4.32	18466.1	5.78	HUMAN	H3BSU3	Pyruvate kinase (Fragment)
524	dirMS_041712_NAR_120mM_plus.7483.7760.3	3	19.67	1	6.54E+05		(R)SGGYGC	(R)S/G|G|Y/G/G S R/D/Y/Y/S S/R(S)		93.37	504.5549	1511.651	-0.0008	-0.5	8.22	26585.7	11.25	HUMAN	B3KT17	Cold-inducible RNA-binding protein
525	dirMS_041712_NAR_50mM_plus2.8635.8970.3	3	19.67	0	1.55E+06		(R)LESGMC	(R)L/E|S G M/Q|N/M/S/I/H/T K(T)		113.23	492.5704	1475.698	-0.0015	-1	6.75	56607.4	5.37	HUMAN	F8VXB4	Keratin, type II cytoskeletal 8
526	dirMS_041712_NAR_30mM_minus2.12505.12908.2	2	19.66	0	7.93E+05		(R)GVVGPE	(R)G V|V|G|P/E/L/Y/G/R(L)		122.53	523.7834	1046.563	-0.0034	-3.2	6	266161	5.68	HUMAN	E9PPU0	Epiplakin
527	dirMS_041712_NAR_60mM_plus.7709.7728.3	3	19.66	1	6.81E+05		(K)AVPKED	(K)A V|P K E D I|Y|S/G/G/G/G G G\S R(S)		98.43	536.2643	1606.782	-0.0036	-2.3	6.11	31011.2	9.5	HUMAN	Q13151	Heterogeneous nuclear ribonucleoprotein A0
528	dirMS_041712_NAR_30mM_minus2.11608.11895.2	2	19.65	0	8.43E+06		(R)AQIFAN	(R)A Q|I|F|A|N/T/V/D/N A R(I)		117.53	660.3384	1319.67	-0.0007	-0.5	5.88	43773.3	5.26	HUMAN	F8VZY9	Keratin, type I cytoskeletal 18
529	dirMS_041712_NAR_20mM_plus.18029.18029.2	2	19.64	0	1.86E+05		(K)GADFLV	(K)G A D F L|V|T E/V/E/N/G G S L/G S\K(K)		195.27	890.4414	1779.876	-0.0004	-0.2	4.14	30947.9	6.47	HUMAN	H3BQ34	Pyruvate kinase
530	dirMS_041712_NAR_50mM_plus2.17679.17679.3	3	19.64	1	8.31E+05		(R)DSEQVA	(R)D S|E/Q/V/A E L/K Q E L A T L K(S)		161.07	601.322	1801.954	-0.0027	-1.5	4.41	109459.8	4.94	HUMAN	F5GYR8	General vesicular transport factor p115
531	dirMS_041712_NAR_60mM_plus.11701.11810.3	3	19.64	1	5.36E+05		(K)GVAINV	(K)G V|A|I/N|M/V/T E E D/K/R(T)		118.67	487.9162	1461.737	-0.0026	-1.7	4.68	46381.4	5.32	HUMAN	P60842	Eukaryotic initiation factor 4A-I
532	dirMS_041712_NAR_60mM_plus.18540.18540.3	3	19.64	1	2.60E+05		(R)RGDINLI	(R)R G D I\N/L|L|M/L/G D/P G T A/K(S)		154.52	557.6331	1670.889	-0.0047	-2.8	5.96	78390.7	9.27	HUMAN	B1AHB1	DNA replication licensing factor MCM5
533	dirMS_041712_NAR_50mM_minus2.17642.17712.3	3	19.63	1	2.12E+06		(R)GLFIIDD	(R)G L|F|I/I/D/D/K/G I L/R(Q)		170.03	453.9381	1359.8	0.0003	0.2	5.96	18498.6	5.82	HUMAN	H7C3T4	Peroxiredoxin-4 (Fragment)
534	dirMS_041712_NAR_120mM_plus.11362.11563.3	3	19.63	0	3.86E+06		(R)FAQHGT	(R)F A Q H G T\F\E|Y|E|Y/S Q R(W)		113.27	588.2657	1762.782	0.0006	0.3	5.4	26419.2	5.1	HUMAN	H0Y9K7	Splicing factor, proline- and glutamine-rich (Fragment)
535	dirMS_041712_NAR_50mM_plus2.17825.18326.3	3	19.63	0	3.82E+07		(R)IHFPLAT	(R)I H\F\P L A\T|Y|A|P/V/I/S A E K(A)		164.67	586.3252	1756.963	-0.0022	-1.2	6.75	49012.3	4.9	HUMAN	A8MUB1	Tubulin alpha-4A chain
536	dirMS_041712_NAR_20mM_minus.9802.10034.2	2	19.62	0	8.73E+05		(R)IVLQIDN	(R)I/V|L|Q|I/D/N A/R(L)		149.7	521.3059	1041.605	-0.0006	-0.6	5.84	20842.9	5.32	HUMAN	C9JM50	Keratin, type I cytoskeletal 19 (Fragment)
537	dirMS_041712_NAR_40mM_minus2.5861.6011.2	2	19.62	0	1.35E+06		(K)AGFAGC	(K)A/G/F|A|G/D/D/A/P/R(A)		98.18	488.7279	976.448	0.0002	0.2	4.21	123097.1	5.83	HUMAN	A5A3E0	POTE ankyrin domain family member F
538	dirMS_041712_NAR_60mM_minus.7198.7631.3	3	19.62	1	2.09E+06		(K)IQDKEGI	(K)I/Q|D K E G|I|P/P/D/Q Q R(L)		99.35	508.5978	1523.771	-0.0024	-1.6	4.56	17271.6	6.75	HUMAN	B4DV12	Ubiquitin
539	dirMS_041712_NAR_20mM_plus.12651.12651.2	2	19.62	0	1.21E+05		(K)GTIQVIT	(K)G T/I/Q|V|I T Q G T/S L K(N)		160.13	673.3872	1345.769	-0.0014	-1.1	8.75	57391.2	6.29	HUMAN	O43175	D-3-phosphoglycerate dehydrogenase
540	dirMS_041712_NAR_60mM_plus.11012.11192.3	3	19.62	1	1.31E+06		(K)GILAADE	(K)G I|L|A/A/D/E/S/T/G/S I A K/R(L)		115.78	496.9383	1488.802	-0.0013	-0.9	6.07	30711.4	7.15	HUMAN	H3BPS8	Fructose-bisphosphate aldolase A (Fragment)

541	dirMS_041712_NAR_30mM_minus2.10438.10644.2	2	19.61	0	9.33E+05		(K)GSDFDC	(K)G/S|D|F|D/C/E/L/R(L)	C:Carbamidomethylation	111.28	549.7307	1098.452	0.0021	1.9	4.03	51260.6	5.39	HUMAN	P61978	Heterogeneous nuclear ribonucleoprotein K
542	dirMS_041712_NAR_60mM_plus.6726.6757.3	3	19.61	1	1.28E+06		(K)SESPKEP	(K)S E|S/P/K/E/P E/Q L/R(K)		93.87	433.8892	1299.654	-0.0009	-0.7	4.78	33268.7	9.31	HUMAN	F8VRQ1	Heterogeneous nuclear ribonucleoprotein A1
543	dirMS_041712_NAR_30mM_minus2.14312.14849.2	2	19.6	0	1.12E+07		(R)FPGQLN	(R)F/P|G\Q|L/N/A D|L/R(K)		134.67	565.7971	1130.595	-0.0084	-7.4	5.84	42230.8	4.77	HUMAN	A6NKZ8	Putative tubulin beta chain-like protein ENSP00000290377
544	dirMS_041712_NAR_40mM_minus2.15621.15666.3	3	19.6	1	4.96E+05		(K)ALFEEVF	(K)A L|F/E/E/V/P E/L/L/T E/A E K K(E)		181.8	615.9988	1845.984	-0.0026	-1.4	4.32	65036	6.27	HUMAN	E9PBU3	Phosphoribosylaminoimidazolecarboxamide formyltransferase
545	dirMS_041712_NAR_30mM_plus2.20506.20914.2	2	19.6	0	2.26E+06		(R)LFDQAF	(R)L/F|D|Q/A/F/G L|P R(L)		162.9	582.3164	1163.621	0.0048	4.1	5.84	20576.9	9.84	HUMAN	F8WE04	Heat shock protein beta-1
546	dirMS_041712_NAR_50mM_plus2.14953.14953.3	3	19.6	0	5.02E+05		(K)TTISVAH	(K)T T/I/S/V/A/H/L|L/A/A/R(Q)		146.45	418.2499	1252.737	-0.002	-1.6	9.44	34143.2	10.07	HUMAN	B4DT23	Protein FAM98A
547	dirMS_041712_NAR_60mM_plus.11232.11232.2	2	19.6	0	2.12E+06		(K)GIVEFAS	(K)G I|V/E/F/A/S K/P A A R(K)		116.18	623.3504	1245.695	-0.0015	-1.2	8.75	76262.2	9.53	HUMAN	P23246	Splicing factor, proline- and glutamine-rich
548	dirMS_041712_NAR_30mM_plus2.7980.8001.2	2	19.59	0	1.46E+06		(K)SNVSDA	(K)S N|V|S|D/A/V|A/Q|S\T/R(I)		95.83	617.8044	1234.602	-0.0007	-0.6	5.55	31075.7	5.65	HUMAN	P60174	Triosephosphate isomerase
549	dirMS_041712_NAR_50mM_plus2.18695.18929.3	3	19.59	1	2.23E+05		(K)IGEEFLT	(K)I/G/E/E/F/L/T/D L S Q/L K K(L)		167.02	540.965	1620.884	-0.0039	-2.4	4.68	97378.8	6.4	HUMAN	P11216	Glycogen phosphorylase, brain form
550	dirMS_041712_NAR_20mM_plus.15278.15563.2	2	19.55	0	8.10E+05		(K)LLCGLLA	(K)L L|C|G/L|L/A/E/R(L)	C:Carbamidomethylation	177.65	522.7971	1044.587	-0.0001	-0.1	6	12647.2	8.07	HUMAN	P14174	Macrophage migration inhibitory factor
551	dirMS_041712_NAR_40mM_plus2.10934.11032.3	3	19.54	1	3.24E+05		(R)DVVICPI	(R)D V|V|I/C/P D A S L E D/A K K(E)	C:Carbamidomethylation	130.9	553.9465	1659.826	-0.0009	-0.5	4.23	18080.7	7.92	HUMAN	K7ELW0	Protein DJ-1
552	dirMS_041712_NAR_40mM_plus2.17402.17402.3	3	19.54	0	2.47E+04		(R)GTEDITS	(R)G T|E/D/I/T/S P/H G I/P L D\L\L D R(V)		169.42	650.3372	1948.997	-0.0004	-0.2	4.22	35067	5.55	HUMAN	E7ETR0	RuvB-like 1
553	dirMS_041712_NAR_50mM_plus2.9894.9902.3	3	19.54	1	1.55E+06	M88m	(K)DVDEAY	(K)D V|D|E/A/Y/m/N K/V/E L/E/S R(L)	m:Oxidized methionine	119.62	605.2794	1797.832	15.9913	-2	4.18	13588.1	4.66	HUMAN	F8VP67	Keratin, type II cytoskeletal 8 (Fragment)
554	dirMS_041712_NAR_120mM_minus.10434.10876.3	3	19.53	1	3.61E+06		(R)LQAEIEC	(R)L/Q|A/E/I/E/G L/K/G Q R(A)		111.42	447.9206	1341.748	-0.0012	-0.9	6.14	56607.4	5.37	HUMAN	F8VXB4	Keratin, type II cytoskeletal 8
555	dirMS_041712_NAR_50mM_plus2.15976.16046.3	3	19.53	1	1.11E+06		(R)GQFSTD	(R)G Q|F/S/T/D/E/L/V/A/E V/E/K R(N)		152.07	569.959	1707.855	0.0076	4.5	4.41	144109.9	6.01	HUMAN	J3KR87	Clathrin heavy chain 2
556	dirMS_041712_NAR_30mM_plus2.7498.7515.2	2	19.52	0	1.00E+06		(R)IQEAGTI	(R)I/Q|E|A/G/T/E/V/V/K(A)		93.7	537.2931	1073.584	-0.0048	-4.5	4.53	31330.8	9.26	HUMAN	E9PDB2	Malate dehydrogenase, mitochondrial
557	dirMS_041712_NAR_40mM_plus2.17015.17080.3	3	19.52	1	3.08E+05		(R)LFVGNLI	(R)L/F|V/G/N|L/P T D I/T/E E D F K R(L)		166.38	665.3502	1994.023	0.0131	6.6	4.32	58856.7	6.26	HUMAN	Q8WXF1	Paraspeckle component 1
558	dirMS_041712_NAR_30mM_plus2.12800.12800.2	2	19.51	0	3.70E+05		(R)ELAEDG	(R)E L/A/E/D G\Y/S|G/V/E V/R(V)		120.6	712.3382	1423.67	-0.0008	-0.6	4	13054.7	9.59	HUMAN	E9PJH4	40S ribosomal protein S3
559	dirMS_041712_NAR_120mM_plus.11532.11569.4	4	19.5	2	1.47E+06		(R)SGKYDLI	(R)S\G K Y D\L/D|F K/S/P D/D|P/S R(Y)		113.65	457.4697	1826.855	0.0014	0.8	4.58	47510.4	7.04	HUMAN	P06733	Alpha-enolase
560	dirMS_041712_NAR_20mM_plus.16103.16330.2	2	19.5	0	4.91E+05		(K)DAGTIA	(K)D A|G|T|I|A|G/L/N V L/R(I)		183.63	600.3422	1199.674	0.0029	2.4	5.84	17973	5.16	HUMAN	E9PI65	Heat shock cognate 71 kDa protein (Fragment)
561	dirMS_041712_NAR_20mM_minus.12178.12542.2	2	19.49	0	9.01E+05	M185m	(K)LAVNm	(K)L/A|V|N/m/V/P F/P R(L)	m:Oxidized methionine	171.88	580.3173	1143.634	15.993	-1.6	9.75	42230.8	4.77	HUMAN	A6NKZ8	Putative tubulin beta chain-like protein ENSP00000290377
562	dirMS_041712_NAR_30mM_minus2.13508.14029.2	2	19.49	0	3.96E+06		(K)ALDFIAS	(K)A/L|D|F|I/A/S/K(G)		128.55	432.7445	864.482	-0.0008	-1	5.88	107710.1	5.38	HUMAN	B7TY16	Actinin alpha 1 isoform 3
563	dirMS_041712_NAR_50mM_minus2.10991.11138.3	3	19.49	1	4.01E+05		(R)INVYYNI	(R)I/N|V|Y/Y|N E/A/T/G G K/Y/V P R(A)		131.28	615.3184	1843.934	0.0069	3.8	8.43	50286.6	4.79	HUMAN	P68371	Tubulin beta-4B chain
564	dirMS_041712_NAR_50mM_minus2.15418.15484.3	3	19.49	0	3.28E+05		(R)LSFQHD	(R)L/S|F Q H D|P E/T|S/V/L/V L R(K)		156.07	580.9793	1740.928	-0.0046	-2.6	5.32	96827.3	5.58	HUMAN	E9PKU7	Neutral alpha-glucosidase AB
565	dirMS_041712_NAR_50mM_plus2.18488.18652.3	3	19.49	0	4.12E+05		(R)VYAILTH	(R)V Y|A|I/L/T H/G I/F/S G/P A I S R(I)		165.83	601.3347	1801.996	-0.0064	-3.5	8.73	24482.9	7.96	HUMAN	B1ALA9	Phosphoribosyl pyrophosphate synthetase 1
566	dirMS_041712_NAR_60mM_minus.6105.6190.3	3	19.48	1	2.51E+06		(K)LEEAEKA	(K)L/E|E/A/E/K/A/A/D/E/S E R(G)		93.27	492.8981	1476.681	-0.0015	-1	4.14	31923.6	4.85	HUMAN	B7Z596	Tropomyosin alpha-1 chain
567	dirMS_041712_NAR_30mM_plus2.20539.20730.2	2	19.48	0	8.74E+05		(R)TVQSLEI	(R)T V|Q|S|L|E|I\D/L/D/S M R(N)		162.18	753.8768	1506.747	-0.0005	-0.3	4.03	43773.3	5.26	HUMAN	F8VZY9	Keratin, type I cytoskeletal 18
568	dirMS_041712_NAR_60mM_minus.18480.18887.3	3	19.47	0	9.16E+06	M195m	(R)LHFFmP	(R)L H\F\F m|P G|F|A|P/L/T S R(G)	m:Oxidized methionine	165.65	546.2843	1620.835	16.0028	4.8	9.75	42230.8	4.77	HUMAN	A6NKZ8	Putative tubulin beta chain-like protein ENSP00000290377
569	dirMS_041712_NAR_30mM_plus2.13173.13333.2	2	19.47	0	7.47E+05		(R)GYFDEEI	(R)G/Y|F|D/E/E/M/N/R(V)		122.82	580.7378	1160.468	0.0006	0.5	4.14	266161	5.68	HUMAN	E9PPU0	Epiplakin
570	dirMS_041712_NAR_30mM_plus2.8720.8894.2	2	19.47	0	1.01E+06		(K)VEIIAND	(K)V/E|I|I|A|N/D|Q/G N R(T)		99.7	614.8154	1228.628	-0.0045	-3.7	4.37	58502.6	6.09	HUMAN	B4DXY3	Heat shock 70 kDa protein 1-like
571	dirMS_041712_NAR_30mM_plus2.14186.14543.2	2	19.46	0	7.98E+05		(R)GLLEDV	(R)G L|L|E|D/V|Q/E/G\R(T)		128.15	558.2879	1115.569	-0.0006	-0.5	4.14	266161	5.68	HUMAN	E9PPU0	Epiplakin
572	dirMS_041712_NAR_60mM_plus.20120.20402.3	3	19.46	0	1.45E+06		(R)IHFPLAT	(R)I H F\P L A\T|Y|A|P/V/I/S A E K(A)		165.98	586.3246	1756.963	-0.004	-2.3	6.75	49012.3	4.9	HUMAN	A8MUB1	Tubulin alpha-4A chain
573	dirMS_041712_NAR_50mM_minus2.14352.14558.3	3	19.45	1	1.10E+06		(K)EAFSLFD	(K)E A|F/S/L/F/D/K D G D/G T/I/T T K(E)		149.92	615.6336	1844.891	-0.005	-2.7	4.23	21860.1	4.45	HUMAN	E7EMB3	Calmodulin
574	dirMS_041712_NAR_40mM_plus2.18496.18735.3	3	19.45	0	1.98E+06		(K)ALESPER	(K)A/L|E|S|P E R/P/F L|A/I/L/G/G A K(V)		175.55	590.3359	1768.996	-0.0024	-1.4	6.19	41827.5	7	HUMAN	B7Z7A9	Phosphoglycerate kinase
575	dirMS_041712_NAR_30mM_plus2.18230.18230.2	2	19.44	0	4.74E+05		(R)GYISPYF	(R)G Y\I|S|P Y/F/I\N T/S K(G)		149.62	695.3554	1389.705	-0.0014	-1	8.5	25225.7	9.52	HUMAN	E7ESH4	60 kDa heat shock protein, mitochondrial (Fragment)
576	dirMS_041712_NAR_50mM_plus2.9579.9832.3	3	19.44	1	1.99E+05		(R)AGTGVD	(R)A/G|T|G/V/D/N V/D L/E/A/A T R K(G)		118.75	539.6108	1616.824	-0.006	-3.7	4.56	57391.2	6.29	HUMAN	O43175	D-3-phosphoglycerate dehydrogenase
577	dirMS_041712_NAR_60mM_plus.11201.11230.3	3	19.44	1	1.81E+06		(K)ALIEMEI	(K)A L|I|E/M/E/K/Q Q Q/D/Q/V D/R(N)		116.28	610.971	1830.901	-0.003	-1.6	4.32	29678.2	9.57	HUMAN	C9JYS8	Non-POU domain-containing octamer-binding protein (Fragment)
578	dirMS_041712_NAR_40mM_plus2.7019.7160.2	2	19.43	0	7.71E+05		(K)AQYEDI	(K)A/Q|Y|E|D/I/A/N/R(S)		101.75	540.2589	1079.512	-0.0011	-1	4.37	37296.3	5.3	HUMAN	F8VU64	Keratin, type II cytoskeletal 8
579	dirMS_041712_NAR_60mM_plus.11213.11213.2	2	19.43	0	2.12E+06		(K)GIVEFAS	(K)G I/V|E/F/A S K/P A A/R(K)		116.18	623.3504	1245.695	-0.0015	-1.2	8.75	76262.2	9.53	HUMAN	P23246	Splicing factor, proline- and glutamine-rich
580	dirMS_041712_NAR_20mM_plus.18595.18595.2	2	19.42	0	1.70E+05	M332m	(R)IVSQLLT	(R)I V|S/Q\L|L|T/L/m D G L K(Q)	m:Oxidized methionine	199.42	723.9145	1430.829	15.993	-1.3	5.84	90004.9	5.14	HUMAN	P55072	Transitional endoplasmic reticulum ATPase
581	dirMS_041712_NAR_40mM_plus2.15526.15813.3	3	19.42	1	1.20E+06		(K)VLDSGA	(K)V/L\D|S G/A|P I\K I|P/V/G/P E T L G R(I)		159.17	640.3668	1919.096	-0.0102	-5.3	6.04	55307.5	5.25	HUMAN	F8VPV9	ATP synthase subunit beta
582	dirMS_041712_NAR_50mM_minus2.15234.15377.3	3	19.41	1	8.73E+05		(K)ILLAELE	(K)I/L|L/A/E L/E Q L/K/G Q/G K(S)		155.78	513.9739	1539.911	-0.0033	-2.2	6.14	49709.6	5.19	HUMAN	B0YJC4	Vimentin
583	dirMS_041712_NAR_120mM_plus.20210.20427.3	3	19.41	0	2.78E+05		(R)INLWHL	(R)I/N|L/W/H/L/E I/T D/R(S)		159.48	470.5889	1409.754	-0.0014	-1	5.32	52204.6	5.82	HUMAN	P63151	Serine/threonine-protein phosphatase 2A 55 kDa regulatory subunit B alpha isoform
584	dirMS_041712_NAR_30mM_plus2.17011.17056.2	2	19.41	0	1.58E+05		(R)IALTDN	(R)I/A|L|T|D/N/A L/I/A/R(S)		142.3	585.8454	1170.684	-0.0006	-0.5	5.84	24489.6	10.74	HUMAN	A8MUD9	60S ribosomal protein L7
585	dirMS_041712_NAR_40mM_plus2.18249.18455.3	3	19.41	1	1.59E+06		(R)GDLGIEI	(R)G D/L/G|I|E/I/P A E K V F L A Q K(M)		173.97	610.0115	1828.022	-0.0015	-0.8	4.68	18466.1	5.78	HUMAN	H3BSU3	Pyruvate kinase (Fragment)
586	dirMS_041712_NAR_50mM_plus2.19302.19591.3	3	19.41	1	9.05E+05		(R)ASLEAAI	(R)A S|L|E|A/A|I A/D/A E Q R G E L A I K(D)		170.58	652.6835	1956.04	-0.0037	-1.9	4.41	56607.4	5.37	HUMAN	F8VXB4	Keratin, type II cytoskeletal 8
587	dirMS_041712_NAR_60mM_minus.13278.13414.3	3	19.4	1	9.72E+05		(R)ELISNSSI	(R)E L|I/S/N/S/S/D/A\L D/K/I/R(Y)		134.78	520.9443	1560.823	-0.0044	-2.8	4.56	20151.2	4.58	HUMAN	G3V2J8	Heat shock protein HSP 90-alpha (Fragment)
588	dirMS_041712_NAR_60mM_minus.9525.9719.3	3	19.4	0	3.48E+05		(R)QLSQAG	(R)Q L|S Q/A/G/S/F/S/D|G T/H G G L R(Y)		113.6	573.2781	1717.825	-0.0055	-3.2	6.74	266161	5.68	HUMAN	E9PPU0	Epiplakin
589	dirMS_041712_NAR_30mM_plus2.21335.21498.2	2	19.39	0	3.90E+05		(R)VNQAIW	(R)V N\Q|A|I|W|L|L|C/T/G/A R(E)	C:Carbamidomethylation	166.88	751.3976	1501.794	-0.0065	-4.3	9.72	23047.2	9.95	HUMAN	P46782	40S ribosomal protein S5
590	dirMS_041712_NAR_40mM_plus2.16092.16092.3	3	19.39	0	8.57E+03		(K)TAMLLA	(K)T A|M|L/L|A L|Q R P E L V|E R(L)		161.05	580.663	1739.984	-0.0092	-5.3	5.81	34746.4	9.67	HUMAN	Q8NFV4	Alpha/beta hydrolase domain-containing protein 11
591	dirMS_041712_NAR_30mM_minus2.10439.10492.2	2	19.38	0	9.33E+05		(K)GSDFDC	(K)G S|D|F/D|C/E/L/R(L)	C:Carbamidomethylation	111.28	549.7307	1098.452	0.0021	1.9	4.03	51260.6	5.39	HUMAN	P61978	Heterogeneous nuclear ribonucleoprotein K
592	dirMS_041712_NAR_60mM_plus.6760.6804.3	3	19.38	1	1.08E+06		(K)TGQAPG	(K)T/G|Q|A|P G Y S Y/T/A/A N K/N K(G)		93.97	557.6087	1670.813	-0.0017	-1	9.52	11447.2	9.81	HUMAN	C9JFR7	Cytochrome c (Fragment)
593	dirMS_041712_NAR_30mM_plus2.13209.13290.2	2	19.37	0	1.08E+06		(R)IGLAEEII	(R)I/G|L|A|E/E/I/R(H)		122.77	450.7605	900.515	-0.0012	-1.3	4.53	26744.2	7.15	HUMAN	K7EK64	Acetyl-CoA carboxylase 1 (Fragment)
594	dirMS_041712_NAR_40mM_plus2.14789.14897.3	3	19.37	0	3.03E+05		(R)IEGDMI	(R)I/E|G/D/M/I V/C/A/A Y|A H E L/P K(Y)	C:Carbamidomethylation	153.93	639.6498	1916.925	0.0104	5.4	4.65	34590.2	9.84	HUMAN	P46777	60S ribosomal protein L5
595	dirMS_041712_NAR_50mM_plus2.16416.16575.3	3	19.37	1	2.02E+06		(K)QGGLGF	(K)Q G/G L/G/P M/N\I|P/L/V/S D/P K R(T)		154.68	593.6536	1778.958	-0.0119	-6.7	8.75	22338.2	8.65	HUMAN	Q06830	Peroxiredoxin-1
596	dirMS_041712_NAR_60mM_plus.18165.18437.3	3	19.37	1	9.69E+05		(K)EVNKFQ	(K)E V/N K F Q/M/A|Y/S/N/L L/R(A)		152.75	571.6293	1712.879	-0.0055	-3.2	8.69	14683.8	10.19	HUMAN	P37108	Signal recognition particle 14 kDa protein
597	dirMS_041712_NAR_120mM_minus.10471.10471.3	3	19.36	0	2.20E+05		(R)SSGPYG	(R)S/S/G/P/Y/G G G G Q Y F/A K P\R(N)		111.27	543.5972	1628.782	-0.0045	-2.8	9.7	33268.7	9.31	HUMAN	F8VRQ1	Heterogeneous nuclear ribonucleoprotein A1
598	dirMS_041712_NAR_50mM_plus2.16710.17196.3	3	19.36	1	1.65E+07		(R)VSFELFA	(R)V/S|F/E/L/F/A D K V/P K(T)		156.82	460.5906	1379.757	0.0003	0.2	6.04	13378.8	6.27	HUMAN	C9J5S7	Peptidyl-prolyl cis-trans isomerase
599	dirMS_041712_NAR_120mM_plus.19556.19595.3	3	19.35	1	7.12E+05		(R)DFNHIN	(R)D F/N H I\N\V|E|L/S/L L/G K K(K)		155.85	576.3192	1726.949	-0.0056	-3.2	6.75	20931.1	10.28	HUMAN	D6RAN4	60S ribosomal protein L9 (Fragment)
600	dirMS_041712_NAR_120mM_plus.19996.19996.4	4	19.35	1	6.21E+05		(K)AAIDWF	(K)A A/I/D/W/F/D G K E F H G N I I K(V)		158.17	491.0027	1960.992	-0.0026	-1.3	5.38	49181	4.93	HUMAN	K7EPT6	TATA-binding protein-associated factor 2N
601	dirMS_041712_NAR_60mM_minus.11644.11743.3	3	19.34	1	3.63E+05		(K)IAQLEEC	(K)I/A|Q|L/E/E Q/L/D/N E T K E R(Q)		125.08	605.9739	1815.908	-0.0012	-0.6	4.25	227783.5	5.5	HUMAN	P35579	Myosin-9
602	dirMS_041712_NAR_60mM_plus.11248.11249.3	3	19.34	1	1.87E+06		(K)LKGEAT	(K)L K G E A|T\V|S|F/D/D|P P S A K(A)		116.45	554.615	1661.838	-0.0076	-4.6	4.56	53724.2	9.54	HUMAN	H3BPE7	RNA-binding protein FUS
603	dirMS_041712_NAR_40mM_minus2.14855.15005.3	3	19.33	1	5.46E+05		(R)GDLGIEI	(R)G D/L/G|I|E/I/P A E K V F L/A Q K(M)		173.02	610.0109	1828.022	-0.0033	-1.8	4.68	18466.1	5.78	HUMAN	H3BSU3	Pyruvate kinase (Fragment)
604	dirMS_041712_NAR_30mM_plus2.12098.12159.2	2	19.33	0	4.46E+05		(R)GGAEGE	(R)G G A|E|G/E|L|Q/A/L/R(A)		116.58	550.7883	1100.57	-0.0001	-0.1	4.53	533778	5.74	HUMAN	Q15149	Plectin
605	dirMS_041712_NAR_50mM_plus2.9927.9929.3	3	19.33	0	1.61E+06		(R)VMLGET	(R)V/M|L|G|E/T/N/P A D/S K/P G T I R(G)		119.75	595.9774	1785.916	0.0013	0.7	6.04	15431.7	9	HUMAN	E7ERL0	Nucleoside diphosphate kinase A
606	dirMS_041712_NAR_60mM_plus.11016.11322.3	3	19.33	0	1.46E+07		(K)GIVEFAS	(K)G I|V/E/F/A/S/K P A A/R(K)		116.15	415.9031	1245.695	-0.0003	-0.2	8.75	76262.2	9.53	HUMAN	P23246	Splicing factor, proline- and glutamine-rich
607	dirMS_041712_NAR_20mM_plus.18373.18373.2	2	19.32	0	1.95E+05		(R)AAVEEG	(R)A A V/E\E G|I|V/L|G|G/G C A L L/R(C)	C:Carbamidomethylation	197.9	842.9549	1684.905	-0.0025	-1.5	4.53	61224.7	5.7	HUMAN	P10809	60 kDa heat shock protein, mitochondrial
608	dirMS_041712_NAR_40mM_plus2.17406.17644.3	3	19.32	0	4.48E+05		(R)DVCTELI	(R)D V|C|T|E L/L/P L I/K/P Q/G R(V)	C:Carbamidomethylation	169.65	580.3213	1738.952	-0.0027	-1.5	6.07	30659.7	9.02	HUMAN	P16152	Carbonyl reductase [NADPH] 1
609	dirMS_041712_NAR_60mM_plus.8469.8487.3	3	19.32	1	4.16E+05		(K)QGIETPE	(K)Q/G I/E/T/P E/D/Q N D/L R/K(M)		102.63	548.2719	1642.803	-0.002	-1.2	4.32	46648.4	9.67	HUMAN	B4DX42	Splicing factor 1
610	dirMS_041712_NAR_30mM_minus2.10650.10713.2	2	19.31	0	2.85E+05		(K)IINNTEN	(K)I/I|N|N|T/E/N L/V/R(E)		111.92	593.3316	1185.659	-0.0027	-2.3	6	40821.3	7.79	HUMAN	Q9Y617	Phosphoserine aminotransferase
611	dirMS_041712_NAR_60mM_minus.14506.14925.3	3	19.31	0	1.67E+06		(K)SLTNDW	(K)S L|T/N/D/W/E D H L/A V/K(H)		142.15	509.9191	1527.744	-0.0011	-0.7	4.53	85057.6	4.94	HUMAN	P07900	Heat shock protein HSP 90-alpha
612	dirMS_041712_NAR_30mM_plus2.19909.20377.2	2	19.31	0	3.32E+06		(K)LAVNM	(K)L/A|V|N|M|V|P F/P R(L)		159.35	572.32	1143.634	-0.0016	-1.4	9.75	42230.8	4.77	HUMAN	A6NKZ8	Putative tubulin beta chain-like protein ENSP00000290377
613	dirMS_041712_NAR_30mM_plus2.7494.7513.2	2	19.31	0	8.65E+05		(K)TPGPGA	(K)T/P|G|P/G/A/Q/S/A L/R(A)		93.72	527.785	1054.564	-0.0013	-1.2	9.41	14466.4	9.92	HUMAN	E5RH77	40S ribosomal protein S14
614	dirMS_041712_NAR_30mM_plus2.8215.8269.2	2	19.31	0	1.36E+06		(K)QGGGG	(K)Q G/G/G|G/G/G/G S|V|P/G I E/R(M)		97.17	642.8178	1284.629	-0.0008	-0.6	6	77799.7	9.09	HUMAN	P52272	Heterogeneous nuclear ribonucleoprotein M
615	dirMS_041712_NAR_60mM_plus.11479.11596.3	3	19.31	0	1.11E+06		(R)SIFQHIQ	(R)S I|F/Q/H/I/Q/S A Q/S Q/R(S)		117.93	510.6056	1529.782	0.0203	13.3	9.49	108720.9	10.17	HUMAN	Q9Y2W1	Thyroid hormone receptor-associated protein 3
616	dirMS_041712_NAR_30mM_plus2.16972.17014.2	2	19.3	0	2.53E+05	M313m	(R)TVQSLEI	(R)T V|Q|S/L|E|I\D/L D/S m/R(N)	m:Oxidized methionine	142.07	761.8741	1506.747	15.9941	-0.5	4.03	43773.3	5.26	HUMAN	F8VZY9	Keratin, type I cytoskeletal 18
617	dirMS_041712_NAR_50mM_plus2.17497.17551.3	3	19.3	0	5.30E+05		(R)GGYVLH	(R)G G|Y|V/L/H/I/G T I/Y/G D/L K(V)		160.33	535.9588	1605.863	-0.0017	-1	6.74	30427.2	7.21	HUMAN	H3BPK7	Alanine--tRNA ligase, cytoplasmic (Fragment)
618	dirMS_041712_NAR_30mM_plus2.6741.6802.2	2	19.29	0	2.92E+05		(R)EAGEQC	(R)E A/G E|Q/G/D|I/E/P R(R)		89.77	600.7772	1200.549	-0.002	-1.7	4	31450.7	5.73	HUMAN	F5H4D6	Ras GTPase-activating protein-binding protein 1
619	dirMS_041712_NAR_50mM_plus2.19906.19906.3	3	19.29	1	1.01E+06		(K)LKEAELL	(K)L K E A E L\L|E|P/L|M/P A I R(A)		172.58	574.9989	1722.982	-0.0001	-0.1	4.79	56164.6	6.01	HUMAN	E9PP73	Coatomer subunit beta (Fragment)
620	dirMS_041712_NAR_30mM_plus2.7349.7410.2	2	19.28	0	6.39E+05		(R)AMNGE	(R)A/M|N|G/E/S/L/D/G/R(Q)		93.25	525.238	1049.468	0.0007	0.7	4.37	17170.1	8.86	HUMAN	P98179	Putative RNA-binding protein 3
621	dirMS_041712_NAR_50mM_plus2.15464.15625.3	3	19.28	1	1.01E+06		(R)KVPGVT	(R)K\V/P G V T A\I|E|L|D/E/D|T/G/T F R(I)		149.87	650.0048	1948.002	-0.0024	-1.2	4.32	68668.3	5.77	HUMAN	B4DXZ6	Fragile X mental retardation syndrome-related protein 1
622	dirMS_041712_NAR_60mM_plus.18127.18143.3	3	19.28	1	1.18E+06		(K)SIYGEKF	(K)S I|Y/G/E/K/F/E/D E/N F I L/K(H)		152.82	611.3091	1831.911	0.0015	0.8	4.41	13378.8	6.27	HUMAN	C9J5S7	Peptidyl-prolyl cis-trans isomerase
623	dirMS_041712_NAR_20mM_plus.14000.14378.2	2	19.27	0	1.57E+06	M313m	(R)TVQSLEI	(R)T V|Q|S|L|E|I\D|L/D/S m R(N)	m:Oxidized methionine	170.15	761.8738	1506.747	15.9935	-0.9	4.03	43773.3	5.26	HUMAN	F8VZY9	Keratin, type I cytoskeletal 18
624	dirMS_041712_NAR_50mM_plus2.18735.18838.3	3	19.27	1	4.32E+05		(K)ITQDIFQ	(K)I/T|Q/D/I/F/Q Q/L L K R(G)		167.02	501.627	1502.869	-0.0025	-1.7	8.75	72796.9	6.75	HUMAN	A6NC17	Methionine--tRNA ligase, cytoplasmic
625	dirMS_041712_NAR_60mM_plus.17498.17655.3	3	19.27	1	6.27E+05		(R)IFDSEEIL	(R)I/F|D/S E/E/I/L/A/G Y K/R(E)		148.98	514.2744	1540.801	0.0081	5.2	4.68	35548.6	4.91	HUMAN	B4DN60	Asparagine--tRNA ligase, cytoplasmic
626	dirMS_041712_NAR_60mM_minus.15583.15807.3	3	19.26	1	1.58E+06		(R)KDDPVT	(R)K D D\P V\T N\L|N|N|A|F/E/V/A E/K(Y)		147.43	635.317	1903.94	-0.0032	-1.7	4.32	21601.4	6.27	HUMAN	K7EJH8	Alpha-actinin-4 (Fragment)
627	dirMS_041712_NAR_20mM_plus.12924.13145.2	2	19.26	0	4.19E+05		(K)LSELEAA	(K)L/S|E|L|E\A/A L/Q/R(A)		161.97	565.3132	1129.621	-0.002	-1.8	4.53	56607.4	5.37	HUMAN	F8VXB4	Keratin, type II cytoskeletal 8
628	dirMS_041712_NAR_20mM_plus.16627.16627.2	2	19.26	0	1.99E+05		(K)VQIAVA	(K)V Q|I/A|V/A\N/A/Q\E/L/L/Q R(M)		186.25	776.9419	1552.881	-0.004	-2.6	5.97	10728.1	9.15	HUMAN	Q9Y5L4	Mitochondrial import inner membrane translocase subunit Tim13
629	dirMS_041712_NAR_20mM_plus.7632.7632.2	2	19.26	0	1.57E+05		(K)IAVAAQ	(K)I/A|V/A/A Q N/C Y K(V)	C:Carbamidomethylation	122.43	569.2902	1137.572	0.001	0.9	8.59	31075.7	5.65	HUMAN	P60174	Triosephosphate isomerase
630	dirMS_041712_NAR_50mM_plus2.7689.7689.3	3	19.26	1	1.64E+05		(K)VDDSSEI	(K)V/D/D/S/S E D K T/E F T/V K(N)		106.45	533.9169	1599.738	-0.0023	-1.4	4.11	107550.2	5.34	HUMAN	P49588	Alanine--tRNA ligase, cytoplasmic
631	dirMS_041712_NAR_60mM_plus.5345.5392.3	3	19.26	1	1.02E+06		(K)CDSSPD	(K)C/D|S/S|P D|S/A/E/D V R K(V)	C:Carbamidomethylation	85.72	489.2128	1465.622	0.0015	1	4.23	40122.7	5.43	HUMAN	P02765	Alpha-2-HS-glycoprotein
632	dirMS_041712_NAR_30mM_minus2.14149.14400.2	2	19.25	0	4.17E+05		(K)MFLYAD	(K)M/F|L|Y|A/D/N E D/R(A)		131.08	637.2806	1273.552	0.0022	1.7	4.03	24724.1	5.17	HUMAN	E7ETQ7	Vacuolar protein sorting-associated protein VTA1 homolog
633	dirMS_041712_NAR_40mM_minus2.14042.14256.3	3	19.25	0	6.88E+05		(R)IHFPLAT	(R)I H F P L\A\T|Y|A|P/V/I/S A E K(A)		167.82	586.3246	1756.963	-0.004	-2.3	6.75	49012.3	4.9	HUMAN	A8MUB1	Tubulin alpha-4A chain
634	dirMS_041712_NAR_30mM_plus2.17600.17804.2	2	19.25	0	8.61E+05		(R)SIQEELC	(R)S I|Q|E|E|L/Q/Q/L/R(Q)		146.02	622.336	1243.664	0.0006	0.5	4.53	533778	5.74	HUMAN	Q15149	Plectin
635	dirMS_041712_NAR_30mM_plus2.18866.18926.2	2	19.25	0	4.85E+05		(K)ALQFLEE	(K)A L|Q|F|L/E/E/V/K(V)		152.2	538.8034	1076.599	0.0009	0.8	4.53	55323	6.02	HUMAN	B4DPJ8	T-complex protein 1 subunit zeta
636	dirMS_041712_NAR_50mM_plus2.11015.11076.3	3	19.25	0	1.32E+06		(K)VGSLDN	(K)V/G|S/L/D|N/V/G/H L/P A G G A V/K(I)		126.15	530.9567	1590.86	-0.0043	-2.7	6.71	26233.5	10.95	HUMAN	B4DSQ1	Microtubule-associated protein
637	dirMS_041712_NAR_50mM_plus2.19118.19420.3	3	19.25	0	1.09E+06		(K)LVLIGSN	(K)L/V|L/I/G/S N H S L/P F/L/K(F)		169.83	513.3073	1537.91	-0.0027	-1.8	8.76	253486.5	5.51	HUMAN	J3KNL6	Protein transport protein Sec16A
638	dirMS_041712_NAR_50mM_plus2.20152.20152.3	3	19.25	1	3.53E+06		(R)TLAEIAK	(R)T\L|A|E/I/A/K V E L D/N M P L R(G)		174.82	604.9965	1812.989	-0.0138	-7.6	4.68	23729.5	9.89	HUMAN	C9IZL7	Non-POU domain-containing octamer-binding protein (Fragment)
639	dirMS_041712_NAR_40mM_plus2.13150.13259.3	3	19.24	0	3.31E+05		(K)LQMEAF	(K)L/Q|M/E/A/P H I|I/V/G T/P G R(V)		144.07	540.2954	1618.873	-0.0017	-1.1	6.75	20859.5	4.85	HUMAN	J3KS25	Eukaryotic initiation factor 4A-I (Fragment)
640	dirMS_041712_NAR_50mM_plus2.11170.11273.3	3	19.24	1	3.14E+05		(R)DLDRIA	(R)D L/D R\I\A|G\Q|V/A/A/A/N K(K)		126.77	481.2661	1441.776	0.008	5.5	5.96	16060.2	10.31	HUMAN	P39019	40S ribosomal protein S19
641	dirMS_041712_NAR_60mM_minus.17881.17998.3	3	19.23	1	3.70E+05		(K)NLRDIDI	(K)N/L R D I\D|E|V/S|S/L/L/R(T)		160.93	510.6099	1529.828	-0.013	-8.5	4.56	58214.7	5.25	HUMAN	B4DEM7	T-complex protein 1 subunit theta
642	dirMS_041712_NAR_120mM_plus.11649.11780.3	3	19.23	1	8.12E+05		(R)ARQEEL	(R)A R Q E E\L|Y|S|E|L/Q/A/R(E)		114.23	531.6047	1592.803	-0.0032	-2	4.79	533778	5.74	HUMAN	Q15149	Plectin
643	dirMS_041712_NAR_60mM_plus.21519.21663.3	3	19.23	0	9.30E+06		(R)LHFFMP	(R)L H\F\F\M|P/G\F|A|P\L/T S R(G)		180.4	540.9492	1620.835	-0.0025	-1.5	9.75	42230.8	4.77	HUMAN	A6NKZ8	Putative tubulin beta chain-like protein ENSP00000290377
644	dirMS_041712_NAR_30mM_minus2.14816.15006.2	2	19.22	0	9.32E+05		(R)LSLYQA	(R)L/S|L/Y|Q/A/M/R(Q)		134.88	491.2611	981.519	-0.0037	-3.8	8.75	266161	5.68	HUMAN	E9PPU0	Epiplakin
645	dirMS_041712_NAR_20mM_plus.19016.19016.2	2	19.22	0	1.05E+05		(K)DTSYLF	(K)D T S/Y/L/F I/T|G P D/V/V/K(S)		201.92	777.9049	1554.805	-0.0025	-1.6	4.21	61363	8.73	HUMAN	C9JQS9	Propionyl-CoA carboxylase beta chain, mitochondrial
646	dirMS_041712_NAR_30mM_plus2.16226.16717.2	2	19.22	0	1.88E+06		(R)GGIVDE	(R)G G/I|V|D|E\G/A/L/L/R(A)		139.2	550.3082	1099.611	-0.0015	-1.3	4.37	57391.2	6.29	HUMAN	O43175	D-3-phosphoglycerate dehydrogenase
647	dirMS_041712_NAR_30mM_plus2.17316.17482.2	2	19.22	0	9.94E+05		(R)IMNTFS	(R)I/M|N|T/F|S/V/V/P S/P K(V)		145.17	660.3537	1319.703	-0.0027	-2	8.75	42831.5	4.86	HUMAN	A8K854	HCG1983504, isoform CRA_f
648	dirMS_041712_NAR_20mM_plus.13957.13957.2	2	19.21	0	1.97E+05		(R)AALQELI	(R)A A|L|Q/E L L/S K(G)		168.48	486.789	972.572	-0.0017	-1.7	6.05	13798.9	10.17	HUMAN	P62851	40S ribosomal protein S25
649	dirMS_041712_NAR_30mM_plus2.13241.13438.2	2	19.21	0	1.26E+06		(R)MVPAGI	(R)M V|P/A|G/M/G/A/G L E R(M)		123.2	594.797	1188.586	0.0003	0.3	5.75	77799.7	9.09	HUMAN	P52272	Heterogeneous nuclear ribonucleoprotein M

<table>
<tr><th></th><th></th><th></th><th></th><th></th><th></th><th></th><th></th><th></th><th></th><th></th><th></th><th></th><th></th><th></th><th></th><th></th><th></th><th></th><th></th><th></th></tr>
<tr><td>650</td><td>dirMS_041712_NAR_30mM_plus2.20290.20779.2</td><td>2</td><td>19.21</td><td>0</td><td>1.40E+06</td><td></td><td>(R)ILVATNL</td><td>(R)I/L|V|A/T/N/L/F/G R(G)</td><td></td><td>162.17</td><td>552.331</td><td>1103.657</td><td>-0.0024</td><td>-2.2</td><td>9.75</td><td>40918.8</td><td>9.33</td><td>HUMAN</td><td>B4DP52</td><td>HCG2005638, isoform CRA_c</td></tr>
<tr><td>651</td><td>dirMS_041712_NAR_50mM_plus2.22205.22205.3</td><td>3</td><td>19.21</td><td>1</td><td>1.35E+06</td><td></td><td>(K)EGGLGP</td><td>(K)E G|G L/G/P/L/N/I/P L L A D/V T R R(L)</td><td></td><td>191</td><td>631.029</td><td>1891.076</td><td>-0.0035</td><td>-1.9</td><td>6.17</td><td>15252.2</td><td>5.82</td><td>HUMAN</td><td>A6NIW5</td><td>Peroxiredoxin 2, isoform CRA_a</td></tr>
<tr><td>652</td><td>dirMS_041712_NAR_60mM_minus.13966.14069.3</td><td>3</td><td>19.2</td><td>0</td><td>2.71E+05</td><td></td><td>(K)YGGELV</td><td>(K)Y/G|G E L/V/P H/F/P A R(V)</td><td></td><td>138.02</td><td>448.2371</td><td>1342.69</td><td>0.0065</td><td>4.8</td><td>6.75</td><td>258215.9</td><td>5.48</td><td>HUMAN</td><td>E7EN95</td><td>Filamin-B</td></tr>
<tr><td>653</td><td>dirMS_041712_NAR_50mM_plus2.15749.15880.3</td><td>3</td><td>19.2</td><td>1</td><td>3.47E+06</td><td></td><td>(K)IIVDELK</td><td>(K)I/I|V/D/E L/K/Q E|V/I/S T S S K(A)</td><td></td><td>151.23</td><td>597.0024</td><td>1788.995</td><td>-0.0027</td><td>-1.5</td><td>4.68</td><td>30149.5</td><td>5.96</td><td>HUMAN</td><td>D6RAC2</td><td>Guanine nucleotide-binding protein subunit beta-2-like 1</td></tr>
<tr><td>654</td><td>dirMS_041712_NAR_50mM_plus2.18698.19170.3</td><td>3</td><td>19.2</td><td>0</td><td>1.48E+06</td><td></td><td>(R)LGFHLPI</td><td>(R)L G\F H L\P L|E|V|A/Y/Q/R(G)</td><td></td><td>167.67</td><td>514.9522</td><td>1542.843</td><td>-0.0007</td><td>-0.4</td><td>6.75</td><td>533778</td><td>5.74</td><td>HUMAN</td><td>Q15149</td><td>Plectin</td></tr>
<tr><td>655</td><td>dirMS_041712_NAR_30mM_plus2.16786.16786.2</td><td>2</td><td>19.19</td><td>0</td><td>2.84E+05</td><td></td><td>(R)SIQADG</td><td>(R)S I/Q|A/D/G L V|W/G S S K(L)</td><td></td><td>140.7</td><td>674.3475</td><td>1347.69</td><td>-0.0026</td><td>-1.9</td><td>5.55</td><td>24934.4</td><td>4.5</td><td>HUMAN</td><td>P24534</td><td>Elongation factor 1-beta</td></tr>
<tr><td>656</td><td>dirMS_041712_NAR_50mM_plus2.18481.18481.2</td><td>2</td><td>19.19</td><td>0</td><td>5.79E+05</td><td></td><td>(R)IHFPLAT</td><td>(R)I H|F|P\L/A\T/Y A P V I S A E K(A)</td><td></td><td>164.92</td><td>878.9842</td><td>1756.963</td><td>-0.0021</td><td>-1.2</td><td>6.75</td><td>49012.3</td><td>4.9</td><td>HUMAN</td><td>A8MUB1</td><td>Tubulin alpha-4A chain</td></tr>
<tr><td>657</td><td>dirMS_041712_NAR_50mM_plus2.5972.6087.3</td><td>3</td><td>19.19</td><td>1</td><td>2.51E+06</td><td></td><td>(R)AQLGGF</td><td>(R)A Q|L|G/G/P E A/A K/S D/E T A/A/K(-)</td><td></td><td>95.82</td><td>548.6114</td><td>1643.824</td><td>-0.0039</td><td>-2.3</td><td>4.68</td><td>3888.3</td><td>5</td><td>HUMAN</td><td>C9J3N8</td><td>Heat shock protein beta-1</td></tr>
<tr><td>658</td><td>dirMS_041712_NAR_60mM_minus.9546.9893.3</td><td>3</td><td>19.18</td><td>1</td><td>1.92E+07</td><td></td><td>(R)LQAEIEG</td><td>(R)L/Q|A/E/I/E/G L/K/G Q R(A)</td><td></td><td>114.12</td><td>447.9164</td><td>1341.748</td><td>-0.0138</td><td>-10.3</td><td>6.14</td><td>56607.4</td><td>5.37</td><td>HUMAN</td><td>F8VXB4</td><td>Keratin, type II cytoskeletal 8</td></tr>
<tr><td>659</td><td>dirMS_041712_NAR_120mM_plus.13419.13590.4</td><td>4</td><td>19.18</td><td>2</td><td>1.02E+06</td><td>M100m</td><td>(K)KQGGLG</td><td>(K)K\Q G G L G\P m/N\I/P/L/V/S D/P K R(T)</td><td>m:Oxidized methionine</td><td>123.85</td><td>481.5167</td><td>1907.053</td><td>15.9918</td><td>-1.6</td><td>9.99</td><td>22338.2</td><td>8.65</td><td>HUMAN</td><td>Q06830</td><td>Peroxiredoxin-1</td></tr>
<tr><td>660</td><td>dirMS_041712_NAR_30mM_plus2.12856.13261.2</td><td>2</td><td>19.18</td><td>0</td><td>2.83E+06</td><td></td><td>(R)EYQELM</td><td>(R)E Y|Q/E/L/M/N/V/K(L)</td><td></td><td>121.65</td><td>577.2809</td><td>1153.556</td><td>-0.0013</td><td>-1.1</td><td>4.53</td><td>56607.4</td><td>5.37</td><td>HUMAN</td><td>F8VXB4</td><td>Keratin, type II cytoskeletal 8</td></tr>
<tr><td>661</td><td>dirMS_041712_NAR_40mM_minus2.12773.13018.3</td><td>3</td><td>19.17</td><td>0</td><td>4.66E+05</td><td></td><td>(K)FTISDHP</td><td>(K)F/T|I/S/D/H|P Q/P I D/P L/L/K(N)</td><td></td><td>159.58</td><td>574.3124</td><td>1720.927</td><td>-0.0042</td><td>-2.4</td><td>5.21</td><td>138084.9</td><td>5.52</td><td>HUMAN</td><td>Q86VP6</td><td>Cullin-associated NEDD8-dissociated protein 1</td></tr>
<tr><td>662</td><td>dirMS_041712_NAR_50mM_plus2.15966.16063.3</td><td>3</td><td>19.17</td><td>1</td><td>1.50E+06</td><td></td><td>(K)VTADVI</td><td>(K)V/T|A/D|V/I/N A/A E K L/Q/V V G R(A)</td><td></td><td>152.23</td><td>595.0057</td><td>1783.007</td><td>-0.0047</td><td>-2.6</td><td>6.04</td><td>57391.2</td><td>6.29</td><td>HUMAN</td><td>O43175</td><td>D-3-phosphoglycerate dehydrogenase</td></tr>
<tr><td>663</td><td>dirMS_041712_NAR_60mM_minus.12002.12158.3</td><td>3</td><td>19.16</td><td>1</td><td>5.65E+05</td><td></td><td>(R)ISVYYNE</td><td>(R)I/S|V/Y/Y/N E/A/T/G G K Y/V/P R(A)</td><td></td><td>127.47</td><td>606.3103</td><td>1816.923</td><td>-0.0065</td><td>-3.6</td><td>8.43</td><td>27549.9</td><td>4.39</td><td>HUMAN</td><td>E9PBJ4</td><td>Tubulin beta chain</td></tr>
<tr><td>664</td><td>dirMS_041712_NAR_60mM_minus.6761.7216.3</td><td>3</td><td>19.16</td><td>1</td><td>1.58E+06</td><td></td><td>(K)IQDKEGI</td><td>(K)I/Q|D K E G|I|P/P/D/Q Q R(L)</td><td></td><td>95.47</td><td>508.5978</td><td>1523.781</td><td>-0.0024</td><td>-1.6</td><td>4.56</td><td>17271.6</td><td>6.75</td><td>HUMAN</td><td>B4DV12</td><td>Ubiquitin</td></tr>
<tr><td>665</td><td>dirMS_041712_NAR_20mM_plus.13976.13976.2</td><td>2</td><td>19.16</td><td>0</td><td>1.85E+05</td><td>M98m</td><td>(R)TLTIVDT</td><td>(R)T L|T I/V|D/T G/I/G/m T/K(A)</td><td>m:Oxidized methionine</td><td>167.67</td><td>683.3671</td><td>1349.735</td><td>15.9924</td><td>-1.8</td><td>5.5</td><td>20151.2</td><td>4.58</td><td>HUMAN</td><td>G3V2J8</td><td>Heat shock protein HSP 90-alpha (Fragment)</td></tr>
<tr><td>666</td><td>dirMS_041712_NAR_20mM_minus.14682.14682.2</td><td>2</td><td>19.15</td><td>0</td><td>2.10E+05</td><td></td><td>(K)LAVEALS</td><td>(K)L A/V E|A|L/S|S/L D|G D\L A/G R(Y)</td><td></td><td>201.28</td><td>793.922</td><td>1586.838</td><td>-0.0017</td><td>-1.1</td><td>4.03</td><td>24286.7</td><td>4.89</td><td>HUMAN</td><td>G3V4N7</td><td>Creatine kinase B-type (Fragment)</td></tr>
<tr><td>667</td><td>dirMS_041712_NAR_50mM_plus2.9514.9734.3</td><td>3</td><td>19.15</td><td>1</td><td>3.83E+05</td><td></td><td>(K)LTSDDVI</td><td>(K)L/T|S/D/D/V/K/E Q I/Y/K(L)</td><td></td><td>118.77</td><td>480.2521</td><td>1438.742</td><td>-0.0007</td><td>-0.5</td><td>4.56</td><td>12573.5</td><td>10.39</td><td>HUMAN</td><td>E9PS50</td><td>40S ribosomal protein S13 (Fragment)</td></tr>
<tr><td>668</td><td>dirMS_041712_NAR_30mM_minus2.10220.10242.2</td><td>2</td><td>19.14</td><td>0</td><td>3.59E+05</td><td></td><td>(R)EQAEAE</td><td>(R)E/Q|A|E/A/E V/A|S/L/N R(R)</td><td></td><td>109.7</td><td>658.8239</td><td>1316.644</td><td>-0.0036</td><td>-2.7</td><td>4.25</td><td>11017</td><td>4.8</td><td>HUMAN</td><td>D6R904</td><td>Tropomyosin alpha-3 chain</td></tr>
<tr><td>669</td><td>dirMS_041712_NAR_50mM_minus2.14673.14957.3</td><td>3</td><td>19.13</td><td>1</td><td>9.62E+05</td><td></td><td>(R)IINEPTA</td><td>(R)I/I|N|E/P T A/A/A/I/A/Y/G L D/K R(E)</td><td></td><td>152.6</td><td>606.0022</td><td>1815.996</td><td>-0.0043</td><td>-2.4</td><td>6.07</td><td>72445.7</td><td>5.07</td><td>HUMAN</td><td>P11021</td><td>78 kDa glucose-regulated protein</td></tr>
<tr><td>670</td><td>dirMS_041712_NAR_30mM_plus2.14536.14905.2</td><td>2</td><td>19.13</td><td>0</td><td>7.19E+06</td><td></td><td>(R)FPGQLN</td><td>(R)F/P/G|Q|L/N A D|L/R(K)</td><td></td><td>131.27</td><td>565.801</td><td>1130.595</td><td>-0.0006</td><td>-0.5</td><td>5.84</td><td>42230.8</td><td>4.77</td><td>HUMAN</td><td>A6NKZ8</td><td>Putative tubulin beta chain-like protein ENSP00000290377</td></tr>
<tr><td>671</td><td>dirMS_041712_NAR_60mM_plus.19768.19915.3</td><td>3</td><td>19.13</td><td>1</td><td>1.10E+06</td><td></td><td>(K)SLLGKD</td><td>(K)S L|L/G/K/D/V|L F L/K(D)</td><td></td><td>163.93</td><td>411.5915</td><td>1232.761</td><td>-0.0013</td><td>-1.1</td><td>8.31</td><td>41827.5</td><td>7</td><td>HUMAN</td><td>B7Z7A9</td><td>Phosphoglycerate kinase</td></tr>
<tr><td>672</td><td>dirMS_041712_NAR_30mM_minus2.18091.18091.2</td><td>2</td><td>19.12</td><td>0</td><td>2.57E+05</td><td></td><td>(K)EGMNIV</td><td>(K)E G\M|N/I|V|E/A/M/E/R(F)</td><td></td><td>152.28</td><td>639.7955</td><td>1278.582</td><td>0.002</td><td>1.6</td><td>4.25</td><td>18240.4</td><td>7.96</td><td>HUMAN</td><td>P62937</td><td>Peptidyl-prolyl cis-trans isomerase A</td></tr>
<tr><td>673</td><td>dirMS_041712_NAR_40mM_minus2.15115.15247.3</td><td>3</td><td>19.12</td><td>0</td><td>7.51E+05</td><td></td><td>(K)ALIEVLQ</td><td>(K)A L|I|E|V|L Q/P L/I/A E H Q A R(R)</td><td></td><td>175.3</td><td>601.0146</td><td>1801.033</td><td>-0.0038</td><td>-2.1</td><td>5.4</td><td>53506.8</td><td>5.83</td><td>HUMAN</td><td>P23381</td><td>Tryptophan--tRNA ligase, cytoplasmic</td></tr>
<tr><td>674</td><td>dirMS_041712_NAR_50mM_plus2.16569.16781.3</td><td>3</td><td>19.12</td><td>1</td><td>1.87E+05</td><td>M132m</td><td>(R)TLAEIAK</td><td>(R)T/L|A|E/I/A/K/V/E L/D/N m/P L R(G)</td><td>m:Oxidized methionine</td><td>155.92</td><td>610.3302</td><td>1812.989</td><td>15.9873</td><td>-4.2</td><td>4.68</td><td>23729.5</td><td>9.89</td><td>HUMAN</td><td>C9IZL7</td><td>Non-POU domain-containing octamer-binding protein (Fragment)</td></tr>
<tr><td>675</td><td>dirMS_041712_NAR_30mM_minus2.9300.9335.2</td><td>2</td><td>19.11</td><td>0</td><td>4.41E+05</td><td></td><td>(K)VTEDTS</td><td>(K)V/T|E|D|T/S/S/V/L/R(S)</td><td></td><td>104.87</td><td>553.7841</td><td>1106.569</td><td>-0.0079</td><td>-7.1</td><td>4.37</td><td>80685.1</td><td>7.31</td><td>HUMAN</td><td>P05165</td><td>Propionyl-CoA carboxylase alpha chain, mitochondrial</td></tr>
<tr><td>676</td><td>dirMS_041712_NAR_40mM_minus2.15017.15086.3</td><td>3</td><td>19.11</td><td>0</td><td>2.68E+06</td><td></td><td>(K)ALESPE</td><td>(K)A/L|E/S/P E/R P F\L/A I L/G G/A/K(V)</td><td></td><td>174.77</td><td>590.3353</td><td>1768.996</td><td>-0.0042</td><td>-2.4</td><td>6.19</td><td>41827.5</td><td>7</td><td>HUMAN</td><td>B7Z7A9</td><td>Phosphoglycerate kinase</td></tr>
<tr><td>677</td><td>dirMS_041712_NAR_60mM_minus.9385.9502.3</td><td>3</td><td>19.11</td><td>0</td><td>2.44E+06</td><td></td><td>(R)LESGM</td><td>(R)L/E|S/G M/Q/N/M/S I/H/T K(T)</td><td></td><td>112.38</td><td>492.571</td><td>1475.698</td><td>0.0003</td><td>0.2</td><td>6.75</td><td>56607.4</td><td>5.37</td><td>HUMAN</td><td>F8VXB4</td><td>Keratin, type II cytoskeletal 8</td></tr>
<tr><td>678</td><td>dirMS_041712_NAR_120mM_plus.11606.11632.3</td><td>3</td><td>19.1</td><td>2</td><td>7.09E+05</td><td></td><td>(R)TIEKFEK</td><td>(R)T/I|E/K/F E/K/E/A A E/M/G K(G)</td><td></td><td>113.92</td><td>537.6079</td><td>1610.809</td><td>-0.0003</td><td>-0.2</td><td>4.94</td><td>12137.8</td><td>9.3</td><td>HUMAN</td><td>A6PW80</td><td>Elongation factor 1-alpha 1 (Fragment)</td></tr>
<tr><td>679</td><td>dirMS_041712_NAR_50mM_plus2.15420.15670.3</td><td>3</td><td>19.1</td><td>0</td><td>1.21E+06</td><td></td><td>(K)LVEALC</td><td>(K)L/V|E/A/L/C/A E/H Q I/N L I/K(V)</td><td>C:Carbamidomethylation</td><td>149.78</td><td>584.3209</td><td>1750.952</td><td>-0.0039</td><td>-2.2</td><td>5.4</td><td>14914.1</td><td>6.84</td><td>HUMAN</td><td>P25398</td><td>40S ribosomal protein S12</td></tr>
<tr><td>680</td><td>dirMS_041712_NAR_60mM_plus.13658.13801.3</td><td>3</td><td>19.1</td><td>0</td><td>1.07E+06</td><td></td><td>(K)QADLYIS</td><td>(K)Q A D L/Y/I/S/E/G L/H/P/R(I)</td><td></td><td>128.62</td><td>500.2592</td><td>1498.765</td><td>-0.0018</td><td>-1.2</td><td>5.32</td><td>55323</td><td>6.02</td><td>HUMAN</td><td>B4DPJ8</td><td>T-complex protein 1 subunit zeta</td></tr>
<tr><td>681</td><td>dirMS_041712_NAR_120mM_minus.17365.17567.3</td><td>3</td><td>19.09</td><td>1</td><td>5.23E+05</td><td></td><td>(R)KVNNAD</td><td>(R)K V N N\A\D\D|F|P/N|L/F/R(Q)</td><td></td><td>150.12</td><td>517.2634</td><td>1549.776</td><td>-0.0001</td><td>-0.1</td><td>5.96</td><td>267260.7</td><td>5.95</td><td>HUMAN</td><td>Q13085</td><td>Acetyl-CoA carboxylase 1</td></tr>
<tr><td>682</td><td>dirMS_041712_NAR_30mM_minus2.15171.15171.2</td><td>2</td><td>19.09</td><td>0</td><td>1.56E+06</td><td></td><td>(R)QITVND</td><td>(R)Q/I/T/V|N D/L/P V/G R(S)</td><td></td><td>135.22</td><td>606.3404</td><td>1211.674</td><td>-0.0007</td><td>-0.6</td><td>5.84</td><td>22062.7</td><td>5.66</td><td>HUMAN</td><td>P32119</td><td>Peroxiredoxin-2</td></tr>
<tr><td>683</td><td>dirMS_041712_NAR_30mM_minus2.6899.6899.2</td><td>2</td><td>19.09</td><td>0</td><td>2.07E+05</td><td></td><td>(K)VTEGGE</td><td>(K)V/T|E/G G/E/P Y/R(L)</td><td></td><td>91.93</td><td>504.238</td><td>1007.479</td><td>-0.0105</td><td>-10.4</td><td>4.53</td><td>96827.3</td><td>5.58</td><td>HUMAN</td><td>E9PKU7</td><td>Neutral alpha-glucosidase AB</td></tr>
<tr><td>684</td><td>dirMS_041712_NAR_30mM_plus2.17289.17577.2</td><td>2</td><td>19.09</td><td>0</td><td>1.12E+06</td><td></td><td>(R)IMNTFS</td><td>(R)I/M|N|T|F|S V/V/P S/P K(V)</td><td></td><td>145.32</td><td>660.3537</td><td>1319.703</td><td>-0.0027</td><td>-2</td><td>8.75</td><td>42831.5</td><td>4.86</td><td>HUMAN</td><td>A8K854</td><td>HCG1983504, isoform CRA_f</td></tr>
<tr><td>685</td><td>dirMS_041712_NAR_60mM_plus.8471.8529.3</td><td>3</td><td>19.09</td><td>1</td><td>4.56E+05</td><td></td><td>(K)ALAAAG</td><td>(K)A L|A|A/A/G Y|D/V/E|K N N S/R(I)</td><td></td><td>102.5</td><td>526.9346</td><td>1578.787</td><td>0.0022</td><td>1.4</td><td>6.11</td><td>21864.9</td><td>11.03</td><td>HUMAN</td><td>P10412</td><td>Histone H1.4</td></tr>
<tr><td>686</td><td>dirMS_041712_NAR_50mM_minus2.8817.8945.3</td><td>3</td><td>19.08</td><td>1</td><td>2.50E+06</td><td>M227m</td><td>(R)mQKEIT</td><td>(R)m Q K E\I/T|A|L|A|P/S T M K(I)</td><td>m:Oxidized methionine</td><td>118.07</td><td>522.2727</td><td>1548.812</td><td>15.9911</td><td>-2.4</td><td>8.35</td><td>32616.5</td><td>5.18</td><td>HUMAN</td><td>A6NL76</td><td>Actin, alpha skeletal muscle</td></tr>
<tr><td>687</td><td>dirMS_041712_NAR_50mM_minus2.10663.11053.3</td><td>3</td><td>19.07</td><td>1</td><td>6.57E+05</td><td></td><td>(R)IAEFTTN</td><td>(R)I/A|E|F/T/T/N/L/T/E/E E E K/S K(S)</td><td></td><td>130.48</td><td>623.6418</td><td>1868.912</td><td>-0.0015</td><td>-0.8</td><td>4.32</td><td>227783.5</td><td>5.5</td><td>HUMAN</td><td>P35579</td><td>Myosin-9</td></tr>
<tr><td>688</td><td>dirMS_041712_NAR_50mM_minus2.9509.9958.3</td><td>3</td><td>19.06</td><td>1</td><td>2.86E+06</td><td></td><td>(R)MQKEIT</td><td>(R)M Q K E I T/A|L|A/P S/T M K(I)</td><td></td><td>123.52</td><td>516.9426</td><td>1548.812</td><td>0.0008</td><td>0.5</td><td>8.35</td><td>32616.5</td><td>5.18</td><td>HUMAN</td><td>A6NL76</td><td>Actin, alpha skeletal muscle</td></tr>
<tr><td>689</td><td>dirMS_041712_NAR_30mM_plus2.18738.18738.2</td><td>2</td><td>19.06</td><td>0</td><td>6.67E+05</td><td></td><td>(R)SYNDEL</td><td>(R)S/Y/N/D\E/L|Q/F/L E/K(I)</td><td></td><td>152.13</td><td>693.335</td><td>1385.658</td><td>0.0044</td><td>3.2</td><td>4.14</td><td>19338.9</td><td>5.33</td><td>HUMAN</td><td>E7EQS9</td><td>tRNA-splicing ligase RtcB homolog</td></tr>
<tr><td>690</td><td>dirMS_041712_NAR_50mM_plus2.19513.19756.3</td><td>3</td><td>19.06</td><td>0</td><td>6.79E+05</td><td></td><td>(R)EAVELP</td><td>(R)E A|V/E/L/P L/T/H F E L/Y/K(Q)</td><td></td><td>171.62</td><td>563.6357</td><td>1688.889</td><td>0.0031</td><td>1.9</td><td>4.75</td><td>47479.5</td><td>5.09</td><td>HUMAN</td><td>P43686</td><td>26S protease regulatory subunit 6B</td></tr>
<tr><td>691</td><td>dirMS_041712_NAR_20mM_plus.14211.14480.2</td><td>2</td><td>19.05</td><td>0</td><td>6.74E+05</td><td>M185m</td><td>(K)LAVNm</td><td>(K)L A|V/N/m|V|P F/P R(L)</td><td>m:Oxidized methionine</td><td>170.62</td><td>580.3169</td><td>1143.634</td><td>15.9922</td><td>-2.3</td><td>9.75</td><td>42230.8</td><td>4.77</td><td>HUMAN</td><td>A6NKZ8</td><td>Putative tubulin beta chain-like protein ENSP00000290377</td></tr>
<tr><td>692</td><td>dirMS_041712_NAR_30mM_plus2.22133.22133.2</td><td>2</td><td>19.05</td><td>0</td><td>2.08E+05</td><td></td><td>(K)NSSYFV</td><td>(K)N S\S Y F V/E/W|I|P N N V K(V)</td><td></td><td>171.85</td><td>848.9189</td><td>1696.833</td><td>-0.0024</td><td>-1.4</td><td>6</td><td>42831.5</td><td>4.86</td><td>HUMAN</td><td>A8K854</td><td>HCG1983504, isoform CRA_f</td></tr>
<tr><td>693</td><td>dirMS_041712_NAR_50mM_plus2.10047.10047.3</td><td>3</td><td>19.05</td><td>1</td><td>1.70E+06</td><td></td><td>(K)TLQEVT</td><td>(K)T L|Q/E/V/T/E M D/S/V/K R(Y)</td><td></td><td>120.07</td><td>512.5958</td><td>1535.773</td><td>-0.0005</td><td>-0.3</td><td>4.68</td><td>558007.1</td><td>5.44</td><td>HUMAN</td><td>P58107</td><td>Epiplakin</td></tr>
<tr><td>694</td><td>dirMS_041712_NAR_60mM_plus.11308.11642.2</td><td>2</td><td>19.05</td><td>1</td><td>1.49E+06</td><td></td><td>(R)ALAAFL</td><td>(R)A/L|A|A/F/L/K/K(S)</td><td></td><td>117.2</td><td>431.2814</td><td>861.556</td><td>-0.0001</td><td>-0.1</td><td>10</td><td>16060.2</td><td>10.31</td><td>HUMAN</td><td>P39019</td><td>40S ribosomal protein S19</td></tr>
<tr><td>695</td><td>dirMS_041712_NAR_120mM_plus.24592.24592.3</td><td>3</td><td>19.04</td><td>1</td><td>5.86E+05</td><td></td><td>(R)GHYTIG</td><td>(R)G H|Y/T I/G\K\E\I/I|D L/V L D/R(I)</td><td></td><td>184.32</td><td>614.6745</td><td>1842.012</td><td>-0.003</td><td>-1.6</td><td>5.38</td><td>58642</td><td>4.95</td><td>HUMAN</td><td>F5H5D3</td><td>Tubulin alpha-1C chain</td></tr>
<tr><td>696</td><td>dirMS_041712_NAR_60mM_plus.13823.13956.3</td><td>3</td><td>19.04</td><td>0</td><td>1.56E+06</td><td></td><td>(K)LAQAN</td><td>(K)L/A|Q/A N/G W/G V/M\V/S/H R(S)</td><td></td><td>129.07</td><td>509.2606</td><td>1525.769</td><td>-0.002</td><td>-1.3</td><td>9.75</td><td>47510.4</td><td>7.04</td><td>HUMAN</td><td>P06733</td><td>Alpha-enolase</td></tr>
<tr><td>697</td><td>dirMS_041712_NAR_50mM_minus2.6639.6669.3</td><td>3</td><td>19.03</td><td>1</td><td>2.15E+05</td><td></td><td>(K)IVADKD</td><td>(K)I/V|A/D/K/D/Y/S|V/T/A/N S K(I)</td><td></td><td>104.22</td><td>504.2623</td><td>1510.775</td><td>-0.0024</td><td>-1.6</td><td>5.96</td><td>25445.8</td><td>5.52</td><td>HUMAN</td><td>A8MW50</td><td>L-lactate dehydrogenase (Fragment)</td></tr>
<tr><td>698</td><td>dirMS_041712_NAR_60mM_minus.8539.8569.3</td><td>3</td><td>19.03</td><td>0</td><td>9.13E+04</td><td></td><td>(R)ISVYYNE</td><td>(R)I|S|V/Y/Y/N/E/A S/S H K(Y)</td><td></td><td>107.72</td><td>466.5604</td><td>1397.67</td><td>-0.0029</td><td>-2.1</td><td>6.75</td><td>18402.2</td><td>5.06</td><td>HUMAN</td><td>G3V2N6</td><td>HCG1983504, isoform CRA_d</td></tr>
<tr><td>699</td><td>dirMS_041712_NAR_50mM_plus2.9051.9301.3</td><td>3</td><td>19.03</td><td>1</td><td>1.70E+06</td><td></td><td>(R)LQAEIE</td><td>(R)L/Q|A/E/I/E/G L/K/G Q R(A)</td><td></td><td>115.58</td><td>447.921</td><td>1341.748</td><td>0</td><td>0</td><td>6.14</td><td>56607.4</td><td>5.37</td><td>HUMAN</td><td>F8VXB4</td><td>Keratin, type II cytoskeletal 8</td></tr>
<tr><td>700</td><td>dirMS_041712_NAR_60mM_minus.5544.5546.3</td><td>3</td><td>19.02</td><td>1</td><td>3.66E+07</td><td></td><td>(K)DSYVGD</td><td>(K)D S|Y/V/G/D/E/A/Q S K R(G)</td><td></td><td>87.63</td><td>452.2129</td><td>1354.623</td><td>0.0008</td><td>0.6</td><td>4.56</td><td>32616.5</td><td>5.18</td><td>HUMAN</td><td>A6NL76</td><td>Actin, alpha skeletal muscle</td></tr>
<tr><td>701</td><td>dirMS_041712_NAR_40mM_plus2.17020.17521.3</td><td>3</td><td>19.02</td><td>0</td><td>7.79E+05</td><td></td><td>(R)IHFPLAT</td><td>(R)I H F\P L A\T|Y|A|P/V/I/S A E K(A)</td><td></td><td>168.35</td><td>586.3246</td><td>1756.963</td><td>-0.004</td><td>-2.3</td><td>6.75</td><td>49012.3</td><td>4.9</td><td>HUMAN</td><td>A8MUB1</td><td>Tubulin alpha-4A chain</td></tr>
<tr><td>702</td><td>dirMS_041712_NAR_30mM_minus2.9007.9605.2</td><td>2</td><td>19.01</td><td>0</td><td>7.60E+06</td><td></td><td>(K)TAVCDI</td><td>(K)T A|V|C/D|I|P|P/R(G)</td><td>C:Carbamidomethylation</td><td>105.43</td><td>514.7641</td><td>1028.519</td><td>0.0016</td><td>1.5</td><td>5.5</td><td>50199.4</td><td>4.75</td><td>HUMAN</td><td>A6NNZ2</td><td>Tubulin beta-8 chain-like protein LOC260334</td></tr>
<tr><td>703</td><td>dirMS_041712_NAR_40mM_plus2.13550.13550.3</td><td>3</td><td>19.01</td><td>0</td><td>6.25E+05</td><td></td><td>(R)TIQVEN</td><td>(R)T I|Q|V/E N\S H L I L/T|G A G A L/N K(V)</td><td></td><td>146.55</td><td>660.3654</td><td>1979.092</td><td>-0.0104</td><td>-5.2</td><td>6.41</td><td>267260.7</td><td>5.95</td><td>HUMAN</td><td>Q13085</td><td>Acetyl-CoA carboxylase 1</td></tr>
<tr><td>704</td><td>dirMS_041712_NAR_60mM_plus.14404.14467.3</td><td>3</td><td>19.01</td><td>1</td><td>1.01E+06</td><td></td><td>(K)YGKIETI</td><td>(K)Y G K\I E T\I|E|V|M/E/D/R(Q)</td><td></td><td>132.13</td><td>528.2635</td><td>1582.778</td><td>-0.0022</td><td>-1.4</td><td>4.41</td><td>34329.8</td><td>8.74</td><td>HUMAN</td><td>E7EWI9</td><td>Heterogeneous nuclear ribonucleoprotein A3</td></tr>
<tr><td>705</td><td>dirMS_041712_NAR_30mM_plus2.21359.21564.2</td><td>2</td><td>19</td><td>0</td><td>3.50E+05</td><td></td><td>(K)FDGILG</td><td>(K)F D|G\I|L|G/M/A/Y/P R(I)</td><td></td><td>167.42</td><td>620.3199</td><td>1239.619</td><td>0.0135</td><td>10.9</td><td>5.83</td><td>32905.8</td><td>5.88</td><td>HUMAN</td><td>C9JH19</td><td>Cathepsin D light chain (Fragment)</td></tr>
<tr><td>706</td><td>dirMS_041712_NAR_50mM_minus2.16956.17380.3</td><td>3</td><td>18.99</td><td>0</td><td>2.11E+07</td><td></td><td>(R)IHFPLAT</td><td>(R)I H\F\P L A\T|Y|A|P/V/I/S A E K(A)</td><td></td><td>167.2</td><td>586.3248</td><td>1756.963</td><td>-0.0034</td><td>-1.9</td><td>6.75</td><td>49012.3</td><td>4.9</td><td>HUMAN</td><td>A8MUB1</td><td>Tubulin alpha-4A chain</td></tr>
<tr><td>707</td><td>dirMS_041712_NAR_20mM_plus.18770.18770.2</td><td>2</td><td>18.99</td><td>0</td><td>2.33E+05</td><td></td><td>(K)TAVETA</td><td>(K)T A\V|E|T/A/V L|L L R(I)</td><td></td><td>200.18</td><td>593.3625</td><td>1185.72</td><td>-0.0024</td><td>-2</td><td>5.66</td><td>56186.7</td><td>5.47</td><td>HUMAN</td><td>B4DUR8</td><td>T-complex protein 1 subunit gamma</td></tr>
<tr><td>708</td><td>dirMS_041712_NAR_60mM_plus.16348.16577.3</td><td>3</td><td>18.99</td><td>0</td><td>4.91E+05</td><td></td><td>(R)EAYPGD</td><td>(R)E A|Y/P/G D V/F/Y/L/H S R(L)</td><td></td><td>142.67</td><td>518.5855</td><td>1553.738</td><td>0.0036</td><td>2.3</td><td>5.32</td><td>48851.3</td><td>9.2</td><td>HUMAN</td><td>K7EK63</td><td>ATP synthase subunit alpha, mitochondrial</td></tr>
<tr><td>709</td><td>dirMS_041712_NAR_30mM_plus2.13187.13187.2</td><td>2</td><td>18.98</td><td>0</td><td>2.83E+05</td><td></td><td>(R)TNEGVI</td><td>(R)T/N|E|G/V/I/E/F R(S)</td><td></td><td>122.2</td><td>532.7727</td><td>1064.537</td><td>0.001</td><td>1</td><td>4.53</td><td>39700.2</td><td>11.49</td><td>HUMAN</td><td>Q13247</td><td>Serine/arginine-rich splicing factor 6</td></tr>
<tr><td>710</td><td>dirMS_041712_NAR_30mM_plus2.20720.20853.2</td><td>2</td><td>18.98</td><td>0</td><td>2.60E+05</td><td></td><td>(R)ASLSLW</td><td>(R)A/S/L/S/L|W/Q/A/M/E/K(G)</td><td></td><td>162.9</td><td>632.3231</td><td>1263.64</td><td>-0.0013</td><td>-1</td><td>6.05</td><td>266161</td><td>5.68</td><td>HUMAN</td><td>E9PPU0</td><td>Epiplakin</td></tr>
<tr><td>711</td><td>dirMS_041712_NAR_50mM_plus2.15368.15649.3</td><td>3</td><td>18.98</td><td>0</td><td>1.46E+06</td><td></td><td>(R)LASTLV</td><td>(R)L/A|S/T L/V/H L|G E\Y Q A|A/V/D/G A R(K)</td><td></td><td>149.63</td><td>657.6802</td><td>1971.029</td><td>-0.0034</td><td>-1.7</td><td>5.32</td><td>71165.3</td><td>5.57</td><td>HUMAN</td><td>J3KS13</td><td>Clathrin heavy chain 1</td></tr>
<tr><td>712</td><td>dirMS_041712_NAR_50mM_minus2.10016.10200.3</td><td>3</td><td>18.97</td><td>0</td><td>5.86E+05</td><td>M147m</td><td>(K)LQmEA</td><td>(K)L/Q|m/E A/P H|I|I/V/G T P G R(V)</td><td>m:Oxidized methionine</td><td>125.53</td><td>545.6267</td><td>1618.873</td><td>15.9922</td><td>-1.7</td><td>6.75</td><td>20859.5</td><td>4.85</td><td>HUMAN</td><td>J3KS25</td><td>Eukaryotic initiation factor 4A-I (Fragment)</td></tr>
<tr><td>713</td><td>dirMS_041712_NAR_30mM_plus2.17012.17115.2</td><td>2</td><td>18.97</td><td>0</td><td>3.76E+04</td><td>M185m</td><td>(K)LAVNm</td><td>(K)L A|V|N/m/V|P F/P/R(L)</td><td>m:Oxidized methionine</td><td>142.12</td><td>580.3176</td><td>1143.634</td><td>15.9936</td><td>-1.1</td><td>9.75</td><td>42230.8</td><td>4.77</td><td>HUMAN</td><td>A6NKZ8</td><td>Putative tubulin beta chain-like protein ENSP00000290377</td></tr>
<tr><td>714</td><td>dirMS_041712_NAR_50mM_plus2.12976.13247.3</td><td>3</td><td>18.97</td><td>1</td><td>1.45E+06</td><td></td><td>(R)VVDALG</td><td>(R)V/V|D|A/L/G N A/I/D/G K G P I/G S/K(T)</td><td></td><td>137.43</td><td>570.9829</td><td>1710.939</td><td>-0.0043</td><td>-2.5</td><td>5.93</td><td>15338.4</td><td>5.51</td><td>HUMAN</td><td>K7EJP1</td><td>ATP synthase subunit alpha, mitochondrial (Fragment)</td></tr>
<tr><td>715</td><td>dirMS_041712_NAR_60mM_minus.11186.11719.3</td><td>3</td><td>18.95</td><td>1</td><td>9.28E+05</td><td>M232m</td><td>(R)MSmKE</td><td>(R)M/S m K E V\D E\Q|M|L|N/V|Q/N K(N)</td><td>m:Oxidized methionine</td><td>122.78</td><td>647.3011</td><td>1923.897</td><td>15.9915</td><td>-1.8</td><td>4.68</td><td>40069.4</td><td>4.83</td><td>HUMAN</td><td>E7EWR1</td><td>Tubulin beta chain</td></tr>
<tr><td>716</td><td>dirMS_041712_NAR_60mM_minus.17319.17358.3</td><td>3</td><td>18.94</td><td>0</td><td>3.22E+05</td><td></td><td>(K)GSDHSA</td><td>(K)G S D H S A\S L E|P|G|E|L|A/E/L V R(S)</td><td></td><td>156.83</td><td>622.9764</td><td>1866.919</td><td>-0.0045</td><td>-2.4</td><td>4.4</td><td>12626.3</td><td>5.59</td><td>HUMAN</td><td>Q5TBR0</td><td>N-acetylneuraminic acid synthase (Fragment)</td></tr>
<tr><td>717</td><td>dirMS_041712_NAR_30mM_plus2.19850.19850.2</td><td>2</td><td>18.94</td><td>0</td><td>2.33E+05</td><td></td><td>(K)GFAFVQ</td><td>(K)G F A|F|V/Q/Y/V/N E/R(N)</td><td></td><td>157.58</td><td>665.3326</td><td>1329.659</td><td>-0.0007</td><td>-0.5</td><td>6</td><td>15791.7</td><td>9.22</td><td>HUMAN</td><td>B4DSU6</td><td>Heterogeneous nuclear ribonucleoproteins C1/C2</td></tr>
<tr><td>718</td><td>dirMS_041712_NAR_60mM_minus.6326.6366.3</td><td>3</td><td>18.93</td><td>0</td><td>2.63E+05</td><td></td><td>(K)TVEAEA</td><td>(K)T\V|E/A/E/A/A/H/G T V T/R(H)</td><td></td><td>94.07</td><td>447.8959</td><td>1341.676</td><td>-0.0026</td><td>-1.9</td><td>5.37</td><td>46943.7</td><td>6.54</td><td>HUMAN</td><td>O75874</td><td>Isocitrate dehydrogenase [NADP] cytoplasmic</td></tr>
<tr><td>719</td><td>dirMS_041712_NAR_60mM_minus.9420.9890.3</td><td>3</td><td>18.93</td><td>1</td><td>1.79E+06</td><td></td><td>(K)TAVCDI</td><td>(K)T/A|V/C/D/I/P/P R G L K(M)</td><td>C:Carbamidomethylation</td><td>114.42</td><td>442.9098</td><td>1326.72</td><td>-0.005</td><td>-3.8</td><td>8.41</td><td>50199.4</td><td>4.75</td><td>HUMAN</td><td>A6NNZ2</td><td>Tubulin beta-8 chain-like protein LOC260334</td></tr>
<tr><td>720</td><td>dirMS_041712_NAR_50mM_plus2.19955.20323.3</td><td>3</td><td>18.93</td><td>1</td><td>3.53E+06</td><td></td><td>(R)TLAEIAK</td><td>(R)T L|A|E/I/A/K/V/E L/D/N M/P L R(G)</td><td></td><td>174.82</td><td>604.9965</td><td>1812.989</td><td>-0.0138</td><td>-7.6</td><td>4.68</td><td>23729.5</td><td>9.89</td><td>HUMAN</td><td>C9IZL7</td><td>Non-POU domain-containing octamer-binding protein (Fragment)</td></tr>
<tr><td>721</td><td>dirMS_041712_NAR_30mM_minus2.12952.13095.2</td><td>2</td><td>18.92</td><td>0</td><td>2.92E+05</td><td></td><td>(K)LSELEPG</td><td>(K)L S|E|L|E|P/G T G D/L R(F)</td><td></td><td>124.57</td><td>643.8325</td><td>1286.659</td><td>-0.0009</td><td>-0.7</td><td>4.14</td><td>266161</td><td>5.68</td><td>HUMAN</td><td>E9PPU0</td><td>Epiplakin</td></tr>
<tr><td>722</td><td>dirMS_041712_NAR_120mM_plus.21127.21479.4</td><td>4</td><td>18.92</td><td>1</td><td>1.53E+06</td><td></td><td>(R)DYLHLP</td><td>(R)D Y/L H L|P|P/E\I/V/P A T L R/R(S)</td><td></td><td>163.77</td><td>473.2689</td><td>1890.06</td><td>-0.0058</td><td>-3.1</td><td>6.75</td><td>19924.8</td><td>9.99</td><td>HUMAN</td><td>F6U211</td><td>40S ribosomal protein S10</td></tr>
<tr><td>723</td><td>dirMS_041712_NAR_30mM_plus2.21853.22069.2</td><td>2</td><td>18.92</td><td>0</td><td>3.14E+05</td><td></td><td>(R)DLLFQA</td><td>(R)D L|L|F/Q/A|L/G/R(T)</td><td></td><td>170.52</td><td>516.7959</td><td>1032.584</td><td>0.0009</td><td>0.8</td><td>5.84</td><td>66742</td><td>6.4</td><td>HUMAN</td><td>Q9NSD9</td><td>Phenylalanine--tRNA ligase beta subunit</td></tr>
<tr><td>724</td><td>dirMS_041712_NAR_30mM_minus2.13575.14062.2</td><td>2</td><td>18.91</td><td>0</td><td>2.59E+06</td><td></td><td>(K)AVLSLY</td><td>(K)A V|L|S/L|Y|A/S/G R(T)</td><td></td><td>128.68</td><td>518.7898</td><td>1036.579</td><td>-0.0062</td><td>-6</td><td>8.79</td><td>14579.3</td><td>5.13</td><td>HUMAN</td><td>K7EM38</td><td>Actin, cytoplasmic 2, N-terminally processed (Fragment)</td></tr>
<tr><td>725</td><td>dirMS_041712_NAR_50mM_minus2.17934.17949.3</td><td>3</td><td>18.91</td><td>1</td><td>8.25E+05</td><td></td><td>(K)TFVNITP</td><td>(K)T F|V/N|I|T/P A/E V G V L/V G K D/R(S)</td><td></td><td>171.32</td><td>639.0247</td><td>1915.065</td><td>-0.0052</td><td>-2.7</td><td>5.74</td><td>17630.6</td><td>11.62</td><td>HUMAN</td><td>I3L3D5</td><td>Profilin-1 (Fragment)</td></tr>
<tr><td>726</td><td>dirMS_041712_NAR_60mM_minus.11272.11272.3</td><td>3</td><td>18.91</td><td>1</td><td>7.95E+05</td><td></td><td>(K)MKEIAE</td><td>(K)M/K/E I A|E|A/Y/L/G K(T)</td><td></td><td>122.48</td><td>418.226</td><td>1252.661</td><td>0.0029</td><td>2.3</td><td>5.9</td><td>17973</td><td>5.16</td><td>HUMAN</td><td>E9PI65</td><td>Heat shock cognate 71 kDa protein (Fragment)</td></tr>
<tr><td>727</td><td>dirMS_041712_NAR_30mM_plus2.18528.18605.2</td><td>2</td><td>18.91</td><td>0</td><td>5.62E+05</td><td></td><td>(R)GVVDSE</td><td>(R)G V|V/D|S|E/D|I|P L N/L|S\R(E)</td><td></td><td>150.85</td><td>757.3957</td><td>1513.786</td><td>-0.0015</td><td>-1</td><td>4.03</td><td>74494.3</td><td>7.79</td><td>HUMAN</td><td>F5H897</td><td>Heat shock protein 75 kDa, mitochondrial</td></tr>
<tr><td>728</td><td>dirMS_041712_NAR_60mM_minus.17151.17363.3</td><td>3</td><td>18.9</td><td>1</td><td>3.81E+05</td><td></td><td>(R)KAPSDL</td><td>(R)K\A P S D\L|Y|Q/I/I|L/K(A)</td><td></td><td>156.22</td><td>463.6095</td><td>1388.815</td><td>-0.0008</td><td>-0.6</td><td>8.5</td><td>21243.9</td><td>5.49</td><td>HUMAN</td><td>E9PL37</td><td>Calpain-1 catalytic subunit (Fragment)</td></tr>
<tr><td>729</td><td>dirMS_041712_NAR_30mM_plus2.8693.8927.2</td><td>2</td><td>18.9</td><td>0</td><td>5.33E+06</td><td></td><td>(R)FGQGG</td><td>(R)F/G|Q|G|G/A|G|P V|G G Q/G/P R(G)</td><td></td><td>99.67</td><td>671.3359</td><td>1341.666</td><td>-0.0013</td><td>-1</td><td>9.75</td><td>76262.2</td><td>9.53</td><td>HUMAN</td><td>P23246</td><td>Splicing factor, proline- and glutamine-rich</td></tr>
<tr><td>730</td><td>dirMS_041712_NAR_60mM_plus.15561.15602.3</td><td>3</td><td>18.9</td><td>0</td><td>7.49E+05</td><td></td><td>(K)DLAGCI</td><td>(K)D L|A/G/C I/H/G/L S N V/K(L)</td><td>C:Carbamidomethylation</td><td>138.1</td><td>461.9069</td><td>1383.705</td><td>0.0012</td><td>0.9</td><td>6.74</td><td>45578.2</td><td>7.83</td><td>HUMAN</td><td>B4DFL2</td><td>Isocitrate dehydrogenase [NADP]</td></tr>
<tr><td>731</td><td>dirMS_041712_NAR_30mM_minus2.16097.16097.2</td><td>2</td><td>18.89</td><td>0</td><td>2.90E+05</td><td></td><td>(R)GEVTAA</td><td>(R)G E|V/T|A/A|D L/F N S R(V)</td><td></td><td>141.68</td><td>640.3185</td><td>1279.628</td><td>0.002</td><td>1.6</td><td>4.37</td><td>266161</td><td>5.68</td><td>HUMAN</td><td>E9PPU0</td><td>Epiplakin</td></tr>
<tr><td>732</td><td>dirMS_041712_NAR_20mM_plus.18992.18992.2</td><td>2</td><td>18.89</td><td>0</td><td>1.91E+05</td><td></td><td>(R)STGEAF</td><td>(R)S T G E/A/F/V|Q/F/A|S Q E I/A E K(A)</td><td></td><td>202.2</td><td>921.4477</td><td>1841.892</td><td>-0.0035</td><td>-1.9</td><td>4.25</td><td>24249.1</td><td>6.99</td><td>HUMAN</td><td>D6RBM0</td><td>Heterogeneous nuclear ribonucleoprotein H (Fragment)</td></tr>
<tr><td>733</td><td>dirMS_041712_NAR_30mM_plus2.6933.6933.2</td><td>2</td><td>18.89</td><td>0</td><td>1.33E+05</td><td></td><td>(R)TAPAAN</td><td>(R)T/A|P/A/A/N L/A S R(I)</td><td></td><td>90.78</td><td>486.2674</td><td>971.527</td><td>0.0007</td><td>0.7</td><td>9.41</td><td>300351.7</td><td>12.1</td><td>HUMAN</td><td>Q9UQ35</td><td>Serine/arginine repetitive matrix protein 2</td></tr>
<tr><td>734</td><td>dirMS_041712_NAR_50mM_plus2.12470.12470.3</td><td>3</td><td>18.89</td><td>0</td><td>3.93E+05</td><td></td><td>(R)LDDTVH</td><td>(R)L/D/D\T V/H/V|V|I/A T/P G/R(I)</td><td></td><td>133.35</td><td>498.2741</td><td>1492.812</td><td>-0.0041</td><td>-2.7</td><td>5.21</td><td>54815.2</td><td>9.17</td><td>HUMAN</td><td>P26196</td><td>Probable ATP-dependent RNA helicase DDX6</td></tr>
<tr><td>735</td><td>dirMS_041712_NAR_30mM_plus2.22649.22750.2</td><td>2</td><td>18.88</td><td>0</td><td>5.16E+05</td><td></td><td>(K)LAAFWL</td><td>(K)L/A|A|F/W|L/M/K(A)</td><td></td><td>176.55</td><td>490.2728</td><td>979.543</td><td>-0.005</td><td>-5.2</td><td>8.75</td><td>32535.6</td><td>9.19</td><td>HUMAN</td><td>B4DZW6</td><td>Regulator of microtubule dynamics protein 1</td></tr>
<tr><td>736</td><td>dirMS_041712_NAR_50mM_plus2.18508.18631.3</td><td>3</td><td>18.88</td><td>0</td><td>4.12E+05</td><td></td><td>(R)VYAILTH</td><td>(R)V/Y|A\I/L T/H/G I/F/S G/P A I S R(I)</td><td></td><td>165.83</td><td>601.3347</td><td>1801.996</td><td>-0.0064</td><td>-3.5</td><td>8.73</td><td>24482.9</td><td>7.96</td><td>HUMAN</td><td>B1ALA9</td><td>Phosphoribosyl pyrophosphate synthetase 1</td></tr>
<tr><td>737</td><td>dirMS_041712_NAR_50mM_plus2.7409.7480.3</td><td>3</td><td>18.88</td><td>0</td><td>3.13E+05</td><td></td><td>(K)VSYGIG</td><td>(K)V/S|Y/G/I/G/D E/E/H/D/Q E G R(V)</td><td></td><td>105.08</td><td>564.2484</td><td>1690.73</td><td>0.0003</td><td>0.2</td><td>4.17</td><td>19395.5</td><td>5.38</td><td>HUMAN</td><td>G3V359</td><td>DNA-(apurinic or apyrimidinic site) lyase (Fragment)</td></tr>
<tr><td>738</td><td>dirMS_041712_NAR_60mM_minus.14235.14306.3</td><td>3</td><td>18.86</td><td>0</td><td>4.62E+05</td><td></td><td>(R)SDSFEN</td><td>(R)S D|S|F/E N/P V/L/Q/Q/H/F R(N)</td><td></td><td>139.27</td><td>568.6086</td><td>1703.814</td><td>-0.0024</td><td>-1.4</td><td>5.3</td><td>64302.5</td><td>6.4</td><td>HUMAN</td><td>B1AHC7</td><td>X-ray repair complementing defective repair in Chinese hamster cells 6 (Ku autoantigen, 70kDa)</td></tr>
<tr><td>739</td><td>dirMS_041712_NAR_20mM_plus.16889.17299.2</td><td>2</td><td>18.86</td><td>0</td><td>6.93E+05</td><td></td><td>(K)VLTPELY</td><td>(K)V L|T|P/E/L Y/A E/L/R(A)</td><td></td><td>189.62</td><td>652.3664</td><td>1303.726</td><td>-0.0001</td><td>-0.1</td><td>4.53</td><td>14391.8</td><td>4.99</td><td>HUMAN</td><td>G3V461</td><td>Creatine kinase B-type (Fragment)</td></tr>
<tr><td>740</td><td>dirMS_041712_NAR_30mM_plus2.10821.11021.2</td><td>2</td><td>18.86</td><td>0</td><td>1.39E+06</td><td></td><td>(K)GSDFDC</td><td>(K)G/S|D|F/D/C/E/L/R(L)</td><td>C:Carbamidomethylation</td><td>110.72</td><td>549.7297</td><td>1098.452</td><td>0.0001</td><td>0.1</td><td>4.03</td><td>51260.6</td><td>5.39</td><td>HUMAN</td><td>P61978</td><td>Heterogeneous nuclear ribonucleoprotein K</td></tr>
<tr><td>741</td><td>dirMS_041712_NAR_30mM_minus2.10811.10811.2</td><td>2</td><td>18.85</td><td>0</td><td>6.40E+05</td><td></td><td>(K)TVSPALI</td><td>(K)T V/S|P/A L/I S R(F)</td><td></td><td>112.1</td><td>472.2815</td><td>943.557</td><td>-0.0014</td><td>-1.5</td><td>9.41</td><td>50425</td><td>5.53</td><td>HUMAN</td><td>H0YH27</td><td>Cullin-associated NEDD8-dissociated protein 1 (Fragment)</td></tr>
<tr><td>742</td><td>dirMS_041712_NAR_30mM_minus2.13407.13481.2</td><td>2</td><td>18.85</td><td>0</td><td>5.61E+05</td><td></td><td>(K)VANVEL</td><td>(K)V/A|N|V|E|L/Y/Y/R(A)</td><td></td><td>125.93</td><td>563.7964</td><td>1126.589</td><td>-0.0036</td><td>-3.2</td><td>5.97</td><td>71165.3</td><td>5.57</td><td>HUMAN</td><td>J3KS13</td><td>Clathrin heavy chain 1</td></tr>
<tr><td>743</td><td>dirMS_041712_NAR_40mM_minus2.14316.14316.3</td><td>3</td><td>18.85</td><td>1</td><td>1.61E+05</td><td></td><td>(K)TGISDV</td><td>(K)T G I|S/D/V F A K N D L|A/V V/D/V/R(I)</td><td></td><td>168.48</td><td>640.3454</td><td>1919.023</td><td>-0.0016</td><td>-0.8</td><td>4.43</td><td>76670.1</td><td>4.6</td><td>HUMAN</td><td>P19338</td><td>Nucleolin</td></tr>
<tr><td>744</td><td>dirMS_041712_NAR_60mM_minus.10856.11043.3</td><td>3</td><td>18.85</td><td>1</td><td>4.59E+05</td><td></td><td>(K)GSITISA</td><td>(K)G S|I/T|I/S/A E E/I/K/D/N R(V)</td><td></td><td>121.13</td><td>511.6045</td><td>1532.792</td><td>0.0075</td><td>4.9</td><td>4.68</td><td>27142.4</td><td>5.34</td><td>HUMAN</td><td>E5RG68</td><td>Copine-3 (Fragment)</td></tr>
<tr><td>745</td><td>dirMS_041712_NAR_50mM_plus2.19450.19586.3</td><td>3</td><td>18.85</td><td>1</td><td>1.20E+06</td><td></td><td>(R)GDLGIEI</td><td>(R)G D/L/G|I|E/I/P A E K V F L/A Q K(M)</td><td></td><td>171.73</td><td>610.0104</td><td>1828.022</td><td>-0.0048</td><td>-2.6</td><td>4.68</td><td>18466.1</td><td>5.78</td><td>HUMAN</td><td>H3BSU3</td><td>Pyruvate kinase (Fragment)</td></tr>
<tr><td>746</td><td>dirMS_041712_NAR_60mM_plus.11214.11214.2</td><td>2</td><td>18.85</td><td>0</td><td>2.12E+06</td><td></td><td>(K)GIVEFAS</td><td>(K)G I/V|E/F/A/S\K/P A A/R(K)</td><td></td><td>116.18</td><td>623.3504</td><td>1245.695</td><td>-0.0015</td><td>-1.2</td><td>8.75</td><td>76262.2</td><td>9.53</td><td>HUMAN</td><td>P23246</td><td>Splicing factor, proline- and glutamine-rich</td></tr>
<tr><td>747</td><td>dirMS_041712_NAR_120mM_plus.18780.18947.3</td><td>3</td><td>18.84</td><td>2</td><td>5.39E+05</td><td></td><td>(R)KIEEIKD</td><td>(R)K|I/E\E/I|K|D|F/L/L/T A R(R)</td><td></td><td>152.18</td><td>525.9746</td><td>1575.911</td><td>-0.0012</td><td>-0.8</td><td>6.18</td><td>2559</td><td>9.99</td><td>HUMAN</td><td>J3KSP2</td><td>60S ribosomal protein L38 (Fragment)</td></tr>
<tr><td>748</td><td>dirMS_041712_NAR_20mM_plus.16456.16781.2</td><td>2</td><td>18.84</td><td>0</td><td>5.45E+05</td><td></td><td>(K)LGGSAV</td><td>(K)L G\G S|A|V|I|S L/E|G/K|P L(-)</td><td></td><td>185.73</td><td>670.8917</td><td>1340.778</td><td>-0.0023</td><td>-1.7</td><td>6</td><td>22956</td><td>8.8</td><td>HUMAN</td><td>E9PK25</td><td>Cofilin-1</td></tr>
<tr><td>749</td><td>dirMS_041712_NAR_30mM_plus2.13376.13545.2</td><td>2</td><td>18.84</td><td>0</td><td>1.36E+06</td><td></td><td>(K)ALMDEV</td><td>(K)A/L|M/D/E V/V/K(A)</td><td></td><td>123.52</td><td>452.7448</td><td>904.481</td><td>0.0015</td><td>1.7</td><td>4.37</td><td>41827.5</td><td>7</td><td>HUMAN</td><td>B7Z7A9</td><td>Phosphoglycerate kinase</td></tr>
<tr><td>750</td><td>dirMS_041712_NAR_50mM_plus2.13337.13654.3</td><td>3</td><td>18.82</td><td>1</td><td>2.13E+06</td><td>M357m</td><td>(R)ALAEIAK</td><td>(R)A L|A|E|I/A/K/A E L/D D/T/P m R(G)</td><td>m:Oxidized methionine</td><td>139.13</td><td>587.3005</td><td>1743.895</td><td>15.9924</td><td>-1.4</td><td>4.32</td><td>76262.2</td><td>9.53</td><td>HUMAN</td><td>P23246</td><td>Splicing factor, proline- and glutamine-rich</td></tr>
<tr><td>751</td><td>dirMS_041712_NAR_50mM_plus2.10242.10322.3</td><td>3</td><td>18.81</td><td>0</td><td>4.49E+05</td><td></td><td>(R)TIVITSH</td><td>(R)T I|V/I/T/S H/P/G Q I/V/K(H)</td><td></td><td>121.67</td><td>464.946</td><td>1392.821</td><td>0.0025</td><td>1.8</td><td>8.44</td><td>27999.4</td><td>5.61</td><td>HUMAN</td><td>B7Z5C0</td><td>DnaJ homolog subfamily A member 1</td></tr>
<tr><td>752</td><td>dirMS_041712_NAR_50mM_plus2.10335.10366.3</td><td>3</td><td>18.81</td><td>0</td><td>2.80E+05</td><td></td><td>(R)TPVIDA</td><td>(R)T/P|V|I/D/A D|K/P/V/S S Q L R(V)</td><td></td><td>121.88</td><td>542.6394</td><td>1625.886</td><td>0.0179</td><td>11</td><td>5.62</td><td>535136.6</td><td>6.01</td><td>HUMAN</td><td>Q14204</td><td>Cytoplasmic dynein 1 heavy chain 1</td></tr>
<tr><td>753</td><td>dirMS_041712_NAR_50mM_plus2.8894.9154.3</td><td>3</td><td>18.81</td><td>0</td><td>2.21E+06</td><td></td><td>(K)GIVEFS</td><td>(K)G I|V/E/F/S/G K/P A A R(K)</td><td></td><td>114.52</td><td>411.2309</td><td>1231.679</td><td>-0.0012</td><td>-1</td><td>8.75</td><td>23729.5</td><td>9.89</td><td>HUMAN</td><td>C9IZL7</td><td>Non-POU domain-containing octamer-binding protein (Fragment)</td></tr>
<tr><td>754</td><td>dirMS_041712_NAR_40mM_minus2.11082.11253.3</td><td>3</td><td>18.8</td><td>0</td><td>7.92E+05</td><td></td><td>(R)QVSASE</td><td>(R)Q V|S/A/S E L/H/T S G I/L/G/P E T L R(D)</td><td></td><td>148.75</td><td>665.6822</td><td>1995.051</td><td>-0.0185</td><td>-9.3</td><td>5.4</td><td>558007.1</td><td>5.44</td><td>HUMAN</td><td>P58107</td><td>Epiplakin</td></tr>
<tr><td>755</td><td>dirMS_041712_NAR_50mM_minus2.8723.8723.3</td><td>3</td><td>18.8</td><td>1</td><td>1.13E+05</td><td></td><td>(K)TAVCDI</td><td>(K)T A/V/C/D/I/P P R G L K(M)</td><td>C:Carbamidomethylation</td><td>117.28</td><td>442.9126</td><td>1326.72</td><td>0.0034</td><td>2.6</td><td>8.41</td><td>50199.4</td><td>4.75</td><td>HUMAN</td><td>A6NNZ2</td><td>Tubulin beta-8 chain-like protein LOC260334</td></tr>
<tr><td>756</td><td>dirMS_041712_NAR_30mM_plus2.8283.8511.2</td><td>2</td><td>18.8</td><td>0</td><td>4.04E+05</td><td></td><td>(K)IANPVE</td><td>(K)I/A|N|P/V/E/G/S T D/R(Q)</td><td></td><td>97.95</td><td>579.7908</td><td>1158.575</td><td>-0.0006</td><td>-0.5</td><td>4.37</td><td>34267.8</td><td>9.05</td><td>HUMAN</td><td>B4DXP5</td><td>Poly(rC)-binding protein 2</td></tr>
<tr><td>757</td><td>dirMS_041712_NAR_120mM_minus.10142.10404.3</td><td>3</td><td>18.79</td><td>1</td><td>1.07E+06</td><td></td><td>(K)IFRDGEE</td><td>(K)I/F/R D G E\E\A|G|A|Y/D/G P R(T)</td><td></td><td>110.25</td><td>551.5928</td><td>1652.766</td><td>-0.0025</td><td>-1.5</td><td>4.32</td><td>55361.8</td><td>6.42</td><td>HUMAN</td><td>G5EA52</td><td>Protein disulfide isomerase family A, member 3, isoform CRA_b</td></tr>
<tr><td>758</td><td>dirMS_041712_NAR_20mM_minus.12079.12079.2</td><td>2</td><td>18.79</td><td>0</td><td>8.51E+05</td><td>M313m</td><td>(R)TVQSLEI</td><td>(R)T V|Q|S|L/E/I D|L\D S m/R(N)</td><td>m:Oxidized methionine</td><td>170.4</td><td>761.8733</td><td>1506.747</td><td>15.9925</td><td>-1.6</td><td>4.03</td><td>43773.3</td><td>5.26</td><td>HUMAN</td><td>F8VZY9</td><td>Keratin, type I cytoskeletal 18</td></tr>
</table>

759	dirMS_041712_NAR_30mM_plus2.12019.12040.2	2	18.79	0	1.99E+05		(R)VFNETPI	(R)V/F|N|E|T/P I/N/P R(K)		116.15	593.8147	1186.622	0.0006	0.5	5.97	99028.8	5.32	HUMAN	Q9Y678	Coatomer subunit gamma-1
760	dirMS_041712_NAR_30mM_minus2.11822.12204.2	2	18.78	0	1.06E+06		(K)VLQLYPI	(K)V/L|Q|L|Y|P/N/N K(A)		118.48	544.8076	1088.61	-0.0019	-1.8	8.56	9740.1	10.08	HUMAN	H0YFG2	Peptidyl-prolyl cis-trans isomerase FKBP4, N-terminally processed (Fragment)
761	dirMS_041712_NAR_30mM_minus2.12685.12926.2	2	18.78	0	4.46E+06		(R)EYQELM	(R)E Y|Q/E/L/M/N/V/K(L)		122.25	577.2825	1153.556	0.0019	1.7	4.53	56607.4	5.37	HUMAN	F8VXB4	Keratin, type II cytoskeletal 8
762	dirMS_041712_NAR_60mM_minus.10837.11063.3	3	18.78	1	6.02E+05		(K)IQVLQQ	(K)I/Q|V|L/Q/Q/Q/A D/D A E E R A E R(L)		121.07	666.9983	1998.984	-0.0036	-1.8	4.18	26611.3	4.47	HUMAN	A6NL28	Putative tropomyosin alpha-3 chain-like protein
763	dirMS_041712_NAR_60mM_minus.15462.15462.3	3	18.78	1	4.49E+05		(R)LLDAQL	(R)L L/D|A/Q/L/A/T G G L V C P A R R(L)	C:Carbamidomethylation	146.02	604.3344	1810.996	-0.007	-3.8	9.6	266161	5.68	HUMAN	E9PPU0	Epiplakin
764	dirMS_041712_NAR_50mM_plus2.8654.8967.3	3	18.78	0	7.69E+05		(R)AALAHS	(R)A A L A H S/E E\V|T|A/S/Q/V A/A|T/K(T)		113.47	595.312	1783.919	0.003	1.7	5.4	23903	4.94	HUMAN	H0YDN1	Plectin (Fragment)
765	dirMS_041712_NAR_50mM_minus2.17504.17927.3	3	18.77	0	1.53E+07		(R)IHFPLAT	(R)I H\F\P L A\T|Y|A|P/V/I/S A E K(A)		167.6	586.3248	1756.963	-0.0034	-1.9	6.75	49012.3	4.9	HUMAN	A8MUB1	Tubulin alpha-4A chain
766	dirMS_041712_NAR_30mM_plus2.18764.19319.2	2	18.77	0	3.07E+06		(R)ADLIAYL	(R)A/D|L|I|A/Y/L/K(K)		153.18	453.7673	906.529	-0.0022	-2.4	5.88	11447.2	9.81	HUMAN	C9JFR7	Cytochrome c (Fragment)
767	dirMS_041712_NAR_30mM_plus2.8302.8302.2	2	18.77	0	2.13E+05		(R)VAIEPG	(R)V A|I/E/P/G A/P/R(E)		97.67	455.2602	909.515	-0.0021	-2.3	5.97	36856.1	8.55	HUMAN	H0YLA4	Sorbitol dehydrogenase
768	dirMS_041712_NAR_50mM_minus2.6763.6763.3	3	18.76	1	2.52E+05		(R)ASVDTK	(R)A S|V/D/T K E/A E G\A/P/Q V/E A G K(R)		104.8	596.2904	1786.882	-0.0251	-14	4.41	54214.9	5.66	HUMAN	C9J813	Caldesmon (Fragment)
769	dirMS_041712_NAR_30mM_plus2.12907.12907.2	2	18.76	0	3.70E+05		(R)ELAEDG	(R)E L/A|E/D/G Y/S/G/V E/V R(V)		120.6	712.3382	1423.67	-0.0008	-0.6	4	13054.7	9.59	HUMAN	E9PJH4	40S ribosomal protein S3
770	dirMS_041712_NAR_30mM_plus2.6103.6103.2	2	18.76	0	1.32E+05	M697m	(K)EGALCEI	(K)E/G A|L/C|E/E/N m/R(G)	C:Carbamidomethylation m:Oxid	86.15	612.7525	1208.503	15.9943	-0.5	4.25	96306.4	6.41	HUMAN	P13639	Elongation factor 2
771	dirMS_041712_NAR_30mM_plus2.8114.8280.2	2	18.76	0	3.43E+05		(K)EGALCEI	(K)E G|A|L/C|E|E/N M/R(G)	C:Carbamidomethylation	96.82	604.7561	1208.503	0.0015	1.2	4.25	96306.4	6.41	HUMAN	P13639	Elongation factor 2
772	dirMS_041712_NAR_50mM_plus2.15638.15660.3	3	18.76	1	9.32E+06		(R)IINEPTA	(R)I/I|N|E|P T A A/A/I A/Y G L D/K K(V)		149.67	596.6673	1787.99	-0.0028	-1.6	6.07	17973	5.16	HUMAN	E9PI65	Heat shock cognate 71 kDa protein (Fragment)
773	dirMS_041712_NAR_60mM_plus.20400.20400.3	3	18.76	1	4.21E+05		(K)NAAGAL	(K)N/A/A/G A L/D/L L/K E L/K(N)		166.93	452.6004	1355.789	-0.0026	-2	6.07	13786.8	9.33	HUMAN	B7Z4S1	Transcription elongation factor A protein 1
774	dirMS_041712_NAR_40mM_plus2.19790.19790.3	3	18.75	0	3.68E+05		(R)DLLDTVI	(R)D L|L|D T\V L/P H L Y N E T K(V)		185.23	590.9774	1770.927	-0.0096	-5.4	4.54	138084.9	5.52	HUMAN	Q86VP6	Cullin-associated NEDD8-dissociated protein 1
775	dirMS_041712_NAR_50mM_plus2.9837.9837.3	3	18.75	0	1.92E+06		(R)EIVHIQA	(R)E/I V H\I Q A|G/Q|C/G N Q I/G A/K(F)	C:Carbamidomethylation	119.02	608.3107	1822.923	-0.0053	-2.9	6.85	40069.4	4.83	HUMAN	E7EWR1	Tubulin beta chain
776	dirMS_041712_NAR_30mM_plus2.16796.16859.2	2	18.74	0	2.03E+06		(K)YMACCL	(K)Y/M|A|C/C/L L/Y/R(G)	C:Carbamidomethylation	140.15	625.2798	1249.553	-0.0003	-0.2	8.59	50706.6	5.68	HUMAN	A6NHL2	Tubulin alpha chain-like 3
777	dirMS_041712_NAR_50mM_plus2.14475.14708.3	3	18.74	0	1.61E+05		(K)TGEEIFG	(K)T|G|E|E|I|F/G T/I/G M R P N A K(N)		144.42	574.2925	1720.869	-0.0057	-3.3	5.81	38222.2	5.77	HUMAN	E9PKG1	Protein arginine N-methyltransferase 1
778	dirMS_041712_NAR_120mM_minus.15257.15446.3	3	18.73	2	9.57E+05		(R)ALKDEEI	(R)A L/K|D E\E|K/M/E/L/Q/E|I\Q|L/K(E)		137.72	649.0139	1945.031	-0.0039	-2	4.58	33450.2	4.73	HUMAN	J3KN67	Tropomyosin alpha-3 chain
779	dirMS_041712_NAR_120mM_minus.8075.8075.3	3	18.73	0	1.23E+06		(K)HSGPNS	(K)H S\G P N\S\A|D|S A/N/D/G/F V/R(L)		98.42	544.2477	1630.72	0.0081	5	5.21	46013.4	5.38	HUMAN	P52597	Heterogeneous nuclear ribonucleoprotein F
780	dirMS_041712_NAR_30mM_minus2.12047.12225.2	2	18.73	0	3.38E+05		(K)ILYSQCG	(K)I/L|Y|S|Q/C/G D/V M R(A)	C:Carbamidomethylation	119.22	671.3183	1341.629	0.0004	0.3	5.83	26991.7	5.01	HUMAN	B7Z6Z4	Myosin light polypeptide 6
781	dirMS_041712_NAR_30mM_minus2.15738.15941.2	2	18.73	0	8.39E+05		(R)GGIVDE	(R)G G/I|V|D|E\G A/L/L/R(A)		139.92	550.3083	1099.611	-0.0013	-1.2	4.37	57391.2	6.29	HUMAN	O43175	D-3-phosphoglycerate dehydrogenase
782	dirMS_041712_NAR_30mM_plus2.10917.11153.2	2	18.73	0	2.64E+06		(R)APIIAVT	(R)A/P|I|I|A/V/T/R(N)		111.08	420.7688	840.53	0.0002	0.2	9.79	53557.7	6.39	HUMAN	H3BTN5	Pyruvate kinase (Fragment)
783	dirMS_041712_NAR_30mM_plus2.6019.6033.2	2	18.73	0	1.18E+05		(R)YEVTSG	(R)Y/E|V|T|S/G/G/G G T S R(M)		85.87	585.7729	1170.539	0	0	6	334221.3	6.44	HUMAN	P15924	Desmoplakin
784	dirMS_041712_NAR_120mM_minus.11522.11537.3	3	18.72	1	2.55E+05		(R)VDWQE	(R)V/D|W/Q/E/N D F/S K/R(I)		117	475.225	1423.66	0.0004	0.3	4.56	267260.7	5.95	HUMAN	Q13085	Acetyl-CoA carboxylase 1
785	dirMS_041712_NAR_20mM_plus.11322.11410.2	2	18.72	0	3.86E+05		(K)LITPAVV	(K)L I|T|P/A/V/V/S\E/R(L)		150.87	542.8186	1084.636	-0.0062	-5.7	6	13798.9	10.17	HUMAN	P62851	40S ribosomal protein S25
786	dirMS_041712_NAR_30mM_plus2.13972.14040.2	2	18.72	0	6.56E+05		(R)IYVVDV	(R)I/Y|V|V|D|V/G|S E/P R(A)		126.27	617.3263	1233.647	-0.0021	-1.7	4.37	39066	6.61	HUMAN	A6PVW9	Selenium binding protein 1 (Fragment)
787	dirMS_041712_NAR_30mM_plus2.21207.21437.2	2	18.72	0	7.11E+05		(R)IINDLLQ	(R)I/I|N|D|L|L/Q/S L/R(S)		166.7	592.8453	1184.7	-0.0164	-13.9	5.84	73399.3	6.86	HUMAN	Q92945	Far upstream element-binding protein 2
788	dirMS_041712_NAR_50mM_plus2.12429.12762.3	3	18.72	1	1.68E+07		(K)KVPQVS	(K)K\V\P\Q V S\T|P/T|L|V/E/V S R(N)		134.13	547.3169	1639.938	-0.0016	-1	8.75	57751.3	6.9	HUMAN	B7WNR0	Serum albumin
789	dirMS_041712_NAR_30mM_minus2.12547.12939.2	2	18.71	0	2.40E+06		(R)GYFDEEI	(R)G/Y|F|D|E/E M/N/R(V)		123.02	580.7374	1160.468	-0.0002	-0.2	4.14	266161	5.68	HUMAN	E9PPU0	Epiplakin
790	dirMS_041712_NAR_20mM_plus.17539.17915.2	2	18.71	0	1.37E+06		(R)YLTVAA	(R)Y/L|T|V/A/A/V/F/R(G)		193.83	520.3009	1039.593	0.001	1	8.75	40069.4	4.83	HUMAN	E7EWR1	Tubulin beta chain
791	dirMS_041712_NAR_30mM_plus2.8963.9443.2	2	18.71	0	1.80E+06		(K)TAVCDIF	(K)T A|V|C/D/I|P|P/R(G)	C:Carbamidomethylation	100.42	514.7643	1028.519	0.002	1.9	5.5	50199.4	4.75	HUMAN	A6NNZ2	Tubulin beta-8 chain-like protein LOC260334
792	dirMS_041712_NAR_20mM_plus.15733.15733.2	2	18.7	0	5.04E+05		(R)GVVDSE	(R)G/V|V|D/S\E/D/I|P L\N L\S R(E)		179.42	757.3956	1513.786	-0.0017	-1.1	4.03	74494.3	7.79	HUMAN	F5H897	Heat shock protein 75 kDa, mitochondrial
793	dirMS_041712_NAR_50mM_minus2.14662.14810.3	3	18.69	1	1.72E+06		(K)AGTQIEI	(K)A/G|T|Q/I/E/N I D/E D/F R D G L K(L)		152.02	640.9832	1920.93	0.0053	2.8	4.11	59872.5	4.85	HUMAN	F5GXS2	Alpha-actinin-4
794	dirMS_041712_NAR_60mM_minus.14244.14541.3	3	18.69	1	1.30E+06		(R)MSMKE	(R)M/S M K E V D\E\Q|M|L/N/V/Q/N/K(N)		140.47	641.9701	1923.897	-0.0015	-0.8	4.68	40069.4	4.83	HUMAN	E7EWR1	Tubulin beta chain
795	dirMS_041712_NAR_120mM_plus.24541.24575.3	3	18.69	1	5.86E+05		(R)GHYTIGI	(R)G H|Y|T|I G K E|I|I|D L/V|L D/R(I)		184.32	614.6745	1842.012	-0.003	-1.6	5.38	58642	4.95	HUMAN	F5H5D3	Tubulin alpha-1C chain
796	dirMS_041712_NAR_120mM_plus.6478.6637.2	2	18.69	1	3.71E+06		(R)TKTEISEI	(R)T/K|T|E|I/S/E/M N/R(N)		88.15	604.8002	1208.594	-0.0008	-0.7	5.81	37296.3	5.3	HUMAN	F8VU64	Keratin, type II cytoskeletal 8
797	dirMS_041712_NAR_30mM_plus2.11494.11519.2	2	18.69	0	3.82E+05		(K)VAGQD	(K)V/A|G/Q|D/G S V V/Q|F/K(I)		113.82	617.8214	1234.643	-0.0071	-5.8	5.81	17127.4	10.05	HUMAN	A8MU27	Small ubiquitin-related modifier 3
798	dirMS_041712_NAR_40mM_plus2.17122.17333.3	3	18.69	1	3.36E+05		(K)VIAATNI	(K)V I|A|A/T/N R V D\I/L|D/P A L L/R(S)		168.1	617.3636	1850.086	-0.0096	-5.2	5.93	32827.5	5.92	HUMAN	E9PKD5	26S protease regulatory subunit 6A (Fragment)
799	dirMS_041712_NAR_20mM_plus.18760.18911.2	2	18.68	0	2.32E+05		(R)IITLAGP	(R)I I|T|L|A|G/P T N\A I F K(A)		200.97	679.9039	1358.804	-0.0037	-2.7	8.75	32621	8.75	HUMAN	B4DLC0	Poly(rC)-binding protein 2
800	dirMS_041712_NAR_30mM_minus2.15045.15045.2	2	18.67	0	7.03E+05		(K)SADTLW	(K)S/A|D|T|L|W/G I Q K(E)		135.23	559.8008	1118.584	0.0103	9.2	5.55	33940.9	9.27	HUMAN	F5H308	L-lactate dehydrogenase
801	dirMS_041712_NAR_50mM_minus2.11366.11388.3	3	18.67	1	9.59E+04		(R)LLIVSTTI	(R)L/L|I|V|S T T/P/Y/S E K D/T/K(Q)		133.12	565.6428	1694.921	-0.0072	-4.3	6.07	46476.4	5.33	HUMAN	B3KQ59	RuvB-like 2
802	dirMS_041712_NAR_60mM_minus.14232.14360.3	3	18.67	1	2.23E+06		(R)TEMENE	(R)T|E|M/E/N E|F/V/L/I/K K(D)		139.38	494.2594	1480.772	-0.0079	-5.4	4.78	13588.1	4.66	HUMAN	F8VP67	Keratin, type II cytoskeletal 8 (Fragment)
803	dirMS_041712_NAR_30mM_plus2.20197.20262.3	3	18.67	0	5.11E+05		(K)SYELPD	(K)S Y\E|L|P D G Q|V|I/T/I/G N E R(F)		159.87	597.6334	1790.892	-0.0063	-3.5	4.14	123097.1	5.83	HUMAN	A5A3E0	POTE ankyrin domain family member F
804	dirMS_041712_NAR_60mM_plus.11766.11791.3	3	18.67	0	9.46E+05	M271m	(R)DLNYCF	(R)D L|N/Y/C/F/S/G m S D/H R(Y)	C:Carbamidomethylation m:Oxid	118.98	539.886	1601.647	15.9963	0.9	5.21	47371.6	5.93	HUMAN	E9PCY7	Heterogeneous nuclear ribonucleoprotein H
805	dirMS_041712_NAR_40mM_minus2.5847.5974.2	2	18.66	0	1.35E+06		(K)AGFAGD	(K)A/G|F|A|G/D/D A/P R(A)		98.18	488.7279	976.448	0.0002	0.2	4.21	123097.1	5.83	HUMAN	A5A3E0	POTE ankyrin domain family member F
806	dirMS_041712_NAR_120mM_plus.10178.10566.3	3	18.66	1	1.42E+06		(R)YLAEFAT	(R)Y/L|A/E/F/A/T/G N D/R K(E)		107.77	462.2337	1384.686	0.001	0.7	6.07	12926.1	4.4	HUMAN	K7EM20	14-3-3 protein epsilon (Fragment)
807	dirMS_041712_NAR_50mM_plus2.12405.12405.3	3	18.66	1	7.86E+05		(K)IEVEKPF	(K)I/E|V E/K/P F A I A K E(-)		133.12	458.5944	1373.767	0.0012	0.8	5.01	23799.1	9.48	HUMAN	P23284	Peptidyl-prolyl cis-trans isomerase B
808	dirMS_041712_NAR_60mM_plus.9359.9487.3	3	18.66	0	7.99E+06		(K)STAGDT	(K)S T|A/G/D/T/H/L/G/G E/D F D/N R(M)		107.4	564.5799	1691.726	-0.0004	-0.3	4.22	69032.8	5.37	HUMAN	E9PKE3	Heat shock cognate 71 kDa protein
809	dirMS_041712_NAR_50mM_minus2.6142.6144.3	3	18.65	1	1.78E+05		(R)IVDGKV	(R)I/V|D/G K/V/V/S\E/T N D/T/K(V)		97.65	502.2662	1504.785	-0.0013	-0.9	4.56	48057	5.34	HUMAN	P05783	Keratin, type I cytoskeletal 18
810	dirMS_041712_NAR_120mM_plus.19581.19620.3	3	18.65	0	8.75E+05		(R)NSVSNF	(R)N|S|V/S/N/F/L/H S/L E/R(G)		156.15	468.2345	1402.707	-0.0184	-13.1	6.75	267260.7	5.95	HUMAN	Q13085	Acetyl-CoA carboxylase 1
811	dirMS_041712_NAR_30mM_plus2.8265.8279.2	2	18.65	0	1.96E+05		(R)TENCLS	(R)T/E|N|C|L/S/S/C V/D/R(F)	C:Carbamidomethylation	97.15	670.7815	1340.557	-0.0012	-0.9	4.37	11439.8	9.3	HUMAN	G3XAN8	Mitochondrial import inner membrane translocase subunit Tim8 B
812	dirMS_041712_NAR_40mM_plus2.14289.14289.3	3	18.65	1	9.15E+05		(R)IINEPTA	(R)I/I|N|E/P T A A/A I A Y G L D K K(V)		150.23	596.6671	1787.99	-0.0034	-1.9	6.07	17973	5.16	HUMAN	E9PI65	Heat shock cognate 71 kDa protein (Fragment)
813	dirMS_041712_NAR_50mM_plus2.18757.18757.3	3	18.65	1	4.36E+05		(K)AAIDWF	(K)A/A/I/D|W/F D G K\E F/S G N P I/K(V)		167.18	632.3186	1894.933	0.0079	4.1	4.56	53724.2	9.54	HUMAN	H3BPE7	RNA-binding protein FUS
814	dirMS_041712_NAR_30mM_plus2.21133.21133.2	2	18.64	0	6.89E+05		(K)IPDWFLI	(K)I/P/D/W/F L N R(Q)		165.78	530.785	1060.557	0.0053	5	5.84	9767.1	11.41	HUMAN	J3JS69	40S ribosomal protein S18
815	dirMS_041712_NAR_50mM_plus2.14589.14589.3	3	18.64	1	2.01E+06		(R)VAGGP	(R)V/A|G G/P Q/M/I Q L S L D/G K R(L)		144.47	557.3066	1669.905	-0.0002	-0.1	8.72	45924.5	5.29	HUMAN	B4E1F3	Selenium-binding protein 1
816	dirMS_041712_NAR_50mM_plus2.18228.18228.3	3	18.64	1	6.23E+05		(K)LLEPVLL	(K)L/L|E/P/V/L/L/L G K E/R(F)		164.48	460.6256	1379.862	0.0002	0.1	6.14	16559.1	10.36	HUMAN	P62249	40S ribosomal protein S16
817	dirMS_041712_NAR_50mM_plus2.9892.9892.3	3	18.64	1	1.74E+05		(R)LLDEEEA	(R)L/L|D/E E/E/A/T/D N D L R/A K(F)		119.38	577.9512	1731.84	-0.0005	-0.3	4.02	96649	6.13	HUMAN	Q8WUM4	Programmed cell death 6-interacting protein
818	dirMS_041712_NAR_20mM_minus.14447.14447.2	2	18.63	0	9.57E+04		(R)LTGQLFI	(R)L T/G/Q|L/F/L\G G S/I/V/K(G)		198.62	666.8967	1332.789	-0.0024	-1.8	8.75	45924.5	5.29	HUMAN	B4E1F3	Selenium-binding protein 1
819	dirMS_041712_NAR_120mM_plus.17058.17540.4	4	18.63	2	2.50E+06		(R)NLKASLI	(R)N/L K/A S\L|E/N/S L/R E V/E/A/R(Y)		143.6	458.0016	1828.988	-0.003	-1.6	6.23	43773.3	5.26	HUMAN	F8VZY9	Keratin, type I cytoskeletal 18
820	dirMS_041712_NAR_120mM_plus.8397.8400.3	3	18.63	1	3.91E+05		(K)NGRVEII	(K)N G R V\E\I\I|A|N/D/Q/G N/R(I)		97.38	519.2706	1555.794	0.0037	2.4	6.07	72445.7	5.07	HUMAN	P11021	78 kDa glucose-regulated protein
821	dirMS_041712_NAR_20mM_plus.14788.14788.2	2	18.63	0	1.98E+05	M179m M:	(K)mFVLDE	(K)m F|V|L|D E|A/D E m L/S R(G)	m:Oxidized methionine	173.6	794.3555	1555.713	31.9906	0.5	3.91	41517.9	5.45	HUMAN	E7EQG2	Eukaryotic initiation factor 4A-II
822	dirMS_041712_NAR_50mM_plus2.13243.13243.3	3	18.63	1	9.66E+05		(R)ELNITAA	(R)E L/N/I/T A/A K/E I/E/V/G G G R(K)		137.7	552.9671	1656.892	-0.0048	-2.9	4.79	21311.5	10.28	HUMAN	B5MCP9	40S ribosomal protein S7
823	dirMS_041712_NAR_50mM_minus2.8793.8903.3	3	18.62	0	2.36E+05		(R)VMLGET	(R)V/M|L\G/E/T N/P A/D/S K/P G T I R(G)		118.3	595.9744	1785.916	-0.0077	-4.3	6.04	15431.7	9	HUMAN	E7ERL0	Nucleoside diphosphate kinase A
824	dirMS_041712_NAR_50mM_minus2.9374.9409.3	3	18.62	0	6.84E+04		(R)AASIFG	(R)A/A|S/I/F/G/G A K/P V D/T A A R(E)		121.62	511.2773	1531.823	-0.0054	-3.5	8.79	64861.3	5.86	HUMAN	B4DS13	Eukaryotic translation initiation factor 4B
825	dirMS_041712_NAR_60mM_minus.13834.13965.3	3	18.62	0	1.38E+06		(K)LGDVYV	(K)L/G|D|V/Y V/N\D/A/F G T/A\H/R(A)		136.73	545.603	1634.792	0.0023	1.4	5.21	41827.5	7	HUMAN	B7Z7A9	Phosphoglycerate kinase
826	dirMS_041712_NAR_20mM_plus.10885.10885.2	2	18.62	0	2.19E+05	M33m	(K)VAGmD	(K)V/A/G/m/D V|E/L/T V E E R(N)	m:Oxidized methionine	147.28	732.3555	1447.71	15.994	-0.6	4	10949.3	5.28	HUMAN	B4DJF2	14-3-3 protein epsilon
827	dirMS_041712_NAR_20mM_plus.9639.9640.2	2	18.62	0	6.24E+05		(R)ISVYYNE	(R)I/S|V/Y|Y/N|E\A/T G G K(Y)		138.33	651.3211	1301.637	-0.0023	-1.8	6	27549.9	4.39	HUMAN	E9PBJ4	Tubulin beta chain
828	dirMS_041712_NAR_30mM_plus2.18267.18267.2	2	18.62	0	5.29E+05		(R)GYISPYF	(R)G Y\I/S|P Y F I N T/S K(G)		149.7	695.3554	1389.705	-0.0014	-1	8.5	25225.7	9.52	HUMAN	E7ESH4	60 kDa heat shock protein, mitochondrial (Fragment)
829	dirMS_041712_NAR_30mM_plus2.8198.8198.2	2	18.62	0	3.43E+05		(K)EGALCEI	(K)E/G/A|L/C/E/E/N/M R(G)	C:Carbamidomethylation	96.82	604.7561	1208.503	0.0015	1.2	4.25	96306.4	6.41	HUMAN	P13639	Elongation factor 2
830	dirMS_041712_NAR_30mM_plus2.8767.8810.2	2	18.62	0	6.99E+05		(R)AAPGAE	(R)A/A|P/G/A E/F/A|P N/K(R)		100.33	536.7755	1072.542	0.0015	1.4	6.05	43979.5	8.92	HUMAN	F5GYZ3	Non-POU domain-containing octamer-binding protein
831	dirMS_041712_NAR_50mM_plus2.21347.21427.3	3	18.62	1	5.13E+05	M145m	(R)GVmLA	(R)G V|m/L/A V/D/A V/I/A E/L K K(Q)	m:Oxidized methionine	182.28	524.9717	1556.908	15.9925	-1.5	6.07	25225.7	9.52	HUMAN	E7ESH4	60 kDa heat shock protein, mitochondrial (Fragment)
832	dirMS_041712_NAR_50mM_minus2.8715.8715.3	3	18.61	1	4.19E+04	M88m	(K)DVDEAY	(K)D V/D|E/A/Y m N K/V/E L E S R(L)	m:Oxidized methionine	117.5	605.2817	1797.832	15.9982	1.8	4.18	13588.1	4.66	HUMAN	F8VP67	Keratin, type II cytoskeletal 8 (Fragment)
833	dirMS_041712_NAR_20mM_plus.8087.8087.2	2	18.61	0	2.12E+05		(R)FGQGG	(R)F G/Q/G|G/A|G/P V/G G Q G P R(G)		125.2	671.3361	1341.666	-0.0009	-0.7	9.75	76262.2	9.53	HUMAN	P23246	Splicing factor, proline- and glutamine-rich
834	dirMS_041712_NAR_60mM_plus.12038.12171.3	3	18.61	0	1.36E+06		(K)VYNYNH	(K)V/Y|N/Y/N/H/L/M/P T|R(Y)		120.25	469.8993	1407.684	-0.0004	-0.3	8.57	16471.9	10.59	HUMAN	K7ELC7	60S ribosomal protein L27 (Fragment)
835	dirMS_041712_NAR_60mM_plus.12052.12052.2	2	18.61	1	1.52E+06		(R)DGKLVS	(R)D G K|L|V|S/E/S S D/V/L|P K(-)		120.25	737.3869	1473.78	-0.013	-8.8	4.56	56607.4	5.37	HUMAN	F8VXB4	Keratin, type II cytoskeletal 8
836	dirMS_041712_NAR_30mM_minus2.8053.8053.2	2	18.6	0	2.96E+05		(R)FLNAEN	(R)F/L|N|A/E/N A\Q/K(F)		98.62	517.7675	1034.526	0.0012	1.1	6	20592.3	9.75	HUMAN	B3KUP2	RAN binding protein 1, isoform CRA_f
837	dirMS_041712_NAR_60mM_minus.13647.13695.3	3	18.6	1	2.69E+05		(K)SLLSNLD	(K)S/L|L/S/N/L/D E V K K(E)		136	415.9055	1245.705	-0.0029	-2.4	5.79	96649	6.13	HUMAN	Q8WUM4	Programmed cell death 6-interacting protein
838	dirMS_041712_NAR_20mM_plus.14038.14253.2	2	18.6	0	1.57E+06	M313m	(R)TVQSLEI	(R)T V|Q|S|L|E|I\D/L/D/S m R(N)	m:Oxidized methionine	170.15	761.8738	1506.747	15.9935	-0.9	4.03	43773.3	5.26	HUMAN	F8VZY9	Keratin, type I cytoskeletal 18
839	dirMS_041712_NAR_30mM_plus2.12433.12433.2	2	18.6	0	7.27E+05		(K)NAGVEG	(K)N A|G|V|E/G/S L I/V/E K(I)		118.2	608.3313	1215.658	-0.0026	-2.2	4.53	61224.7	5.7	HUMAN	P10809	60 kDa heat shock protein, mitochondrial
840	dirMS_041712_NAR_30mM_plus2.19911.20112.2	2	18.6	0	8.41E+05		(K)MSIYQA	(K)M/S|I|Y/Q/A/M W/K(G)		158.83	579.2783	1157.548	0.0011	1	8.34	266161	5.68	HUMAN	E9PPU0	Epiplakin
841	dirMS_041712_NAR_40mM_plus2.9008.9008.3	3	18.6	0	5.80E+04		(R)AALAHS	(R)A A L A H S/E E\V|T|A/S|Q/V/A/A T K(T)		115.18	595.3161	1783.919	0.0153	8.6	5.4	23903	4.94	HUMAN	H0YDN1	Plectin (Fragment)
842	dirMS_041712_NAR_60mM_minus.19724.19900.3	3	18.59	0	6.61E+06		(R)LHFFMP	(R)L H F\F\M|P/G\F|A|P\L/T S R(G)		179.58	540.9491	1620.835	-0.0028	-1.7	9.75	42230.8	4.77	HUMAN	A6NKZ8	Putative tubulin beta chain-like protein ENSP00000290377
843	dirMS_041712_NAR_40mM_plus2.11314.11314.3	3	18.59	0	1.67E+05		(R)SVIDPVF	(R)S V\I/D|P/V/P/A/P V G D S H V D G A A K(S)		132.95	644.3327	1930.987	-0.0033	-1.7	4.41	58326.8	5.7	HUMAN	Q13177	Serine/threonine-protein kinase PAK 2
844	dirMS_041712_NAR_40mM_plus2.15106.15404.3	3	18.59	0	3.27E+05		(K)GVTIIGP	(K)G V|T|I/I|G/P A T V/G G I/K/P G C F K(I)	C:Carbamidomethylation	156.22	624.683	1872.041	-0.0067	-3.6	10	91839.6	9.22	HUMAN	B4E3P0	ATP-citrate synthase
845	dirMS_041712_NAR_120mM_minus.17941.18438.3	3	18.58	1	3.33E+06		(R)ARFEELN	(R)A R\F E\E\L|N|A|D/L/F/R(G)		154.37	494.2561	1480.754	-0.0006	-0.4	4.68	45508.6	5.5	HUMAN	A8K7Q2	Heat shock cognate 71 kDa protein
846	dirMS_041712_NAR_20mM_plus.16093.16353.2	2	18.58	0	4.91E+05		(K)DAGTIA	(K)D A|G T|I|A|G L/N/V L/R(I)		183.63	600.3422	1199.674	0.0029	2.4	5.84	17973	5.16	HUMAN	E9PI65	Heat shock cognate 71 kDa protein (Fragment)
847	dirMS_041712_NAR_30mM_plus2.6375.6391.2	2	18.58	0	1.01E+05		(R)SVSPCSI	(R)S V|S|P/C|S N/V E/S R(L)	C:Carbamidomethylation	87.83	611.2799	1221.553	-0.0003	-0.2	5.72	99885.2	12.36	HUMAN	I3L182	Serine/arginine repetitive matrix protein 2 (Fragment)
848	dirMS_041712_NAR_50mM_plus2.14344.14344.3	3	18.58	1	1.80E+05		(K)TITLEVEI	(K)T I|T|L/E/V E/P S D T/I/E N V K A K(I)		143.57	663.0232	1987.059	-0.0043	-2.2	4.41	17271.6	6.75	HUMAN	B4DV12	Ubiquitin
849	dirMS_041712_NAR_50mM_plus2.7499.7584.3	3	18.58	0	3.69E+05		(R)VGQAVI	(R)V/G|Q/A/V D\V/V/G Q/A/G K/P K(T)		105.72	484.9438	1452.817	-0.0001	0	8.56	86346.5	5.05	HUMAN	E7EW34	26S proteasome non-ATPase regulatory subunit 2
850	dirMS_041712_NAR_60mM_minus.6112.6112.3	3	18.57	1	2.64E+05		(K)LEEAEK	(K)L/E/E/A/E/K/A A D/E\S E R(G)		92.57	492.8981	1476.681	-0.0015	-1	4.14	31923.6	4.85	HUMAN	B7Z596	Tropomyosin alpha-1 chain
851	dirMS_041712_NAR_20mM_plus.14459.14499.2	2	18.57	0	6.32E+05	M185m	(K)LAVNm	(K)L/A|V/N/m|V|P/F P R(L)	m:Oxidized methionine	170.68	580.3169	1143.634	15.9922	-2.3	9.75	42230.8	4.77	HUMAN	A6NKZ8	Putative tubulin beta chain-like protein ENSP00000290377
852	dirMS_041712_NAR_30mM_plus2.13044.13426.2	2	18.56	0	6.09E+06		(K)IGGIGTV	(K)I/G|G|I|G/T/V|P V G R(V)		122.85	513.3084	1025.61	-0.0007	-0.7	9.75	50482.3	9.31	HUMAN	P68104	Elongation factor 1-alpha 1
853	dirMS_041712_NAR_60mM_plus.11843.11882.3	3	18.56	1	2.80E+06		(K)YGEPGE	(K)Y/G|E|P/G E V|F/I/N K G K(G)		119.42	479.9219	1437.737	0.0139	9.7	6.14	76262.2	9.53	HUMAN	P23246	Splicing factor, proline- and glutamine-rich
854	dirMS_041712_NAR_30mM_minus2.10250.10294.2	2	18.55	0	2.90E+05		(K)DQVANS	(K)D Q|V|A|N/S/A/F/V/E/R(L)		109.8	618.303	1235.602	-0.0028	-2.2	4.37	85057.6	4.94	HUMAN	P07900	Heat shock protein HSP 90-alpha
855	dirMS_041712_NAR_40mM_minus2.12486.12708.3	3	18.55	1	5.98E+05		(R)VCENIPI	(R)V/C|E|N/I|P I\V/L C G N K V D\I/K(D)	C:Carbamidomethylation	157.7	657.6842	1971.04	-0.0021	-1.1	6.04	14844.8	5.89	HUMAN	B4DV51	GTP-binding nuclear protein Ran
856	dirMS_041712_NAR_60mM_minus.6831.7286.3	3	18.55	1	2.64E+06		(K)IQDKEGI	(K)I/Q/D\K E G|I|P|P/D/Q Q R(L)		97.3	508.5978	1523.781	-0.0024	-1.6	4.56	17271.6	6.75	HUMAN	B4DV12	Ubiquitin
857	dirMS_041712_NAR_50mM_plus2.9942.9989.3	3	18.55	0	1.16E+06		(K)INEKPQ	(K)I/N/E K/P\Q\V|I|A/D/Y/E/S G R(A)		119.77	573.6314	1718.871	0.0089	5.2	4.68	16368.4	9.95	HUMAN	O60869	Endothelial differentiation-related factor 1
858	dirMS_041712_NAR_60mM_plus.11259.11452.3	3	18.55	0	4.00E+06		(K)NFYVEH	(K)N F/Y/V/E/H/P E V A/R(L)		116.57	454.2286	1360.664	0.0068	5	5.4	73202	9.19	HUMAN	C9JMU5	Probable ATP-dependent RNA helicase DDX17
859	dirMS_041712_NAR_60mM_plus.16220.16357.3	3	18.55	0	5.85E+05		(K)WIGLDL	(K)W/I|G/L/D/L/S/N/G K/P R(D)		142.08	452.5864	1355.743	0.0016	1.2	8.75	44227.3	7.81	HUMAN	E7EQR6	T-complex protein 1 subunit alpha
860	dirMS_041712_NAR_60mM_plus.8494.8718.3	3	18.55	0	6.18E+05		(K)GQVCVI	(K)G Q|V/C/V/M/I/H S G S R(G)	C:Carbamidomethylation	103.1	444.2219	1330.636	0.0157	11.8	9.75	55722.8	6.78	HUMAN	Q9Y3I0	tRNA-splicing ligase RtcB homolog
861	dirMS_041712_NAR_50mM_minus2.13786.14034.3	3	18.54	0	1.67E+06		(R)QVSASE	(R)Q V|S/A/S E/L/H T S G I/L/G/P E T L R(D)		147.53	665.688	1995.051	-0.0011	-0.6	5.4	558007.1	5.44	HUMAN	P58107	Epiplakin
862	dirMS_041712_NAR_60mM_minus.9217.9217.3	3	18.54	0	2.84E+05		(K)ATAGDT	(K)A/T|A/G D T H/L|G G E D F/D/N R(L)		111.4	559.2488	1675.731	0.0012	0.7	4.22	58502.6	6.09	HUMAN	B4DXY3	Heat shock 70 kDa protein 1-like
863	dirMS_041712_NAR_20mM_plus.8287.8287.2	2	18.54	0	9.05E+04		(K)GGEIQP	(K)G G\E/I|Q/P V/S/V/K(V)		127.42	507.2841	1013.563	-0.0017	-1.6	6	10803.5	9.82	HUMAN	B8ZZL8	10 kDa heat shock protein, mitochondrial
864	dirMS_041712_NAR_120mM_minus.13295.13295.4	4	18.53	1	8.85E+05		(K)SVHSSVI	(K)S V H S\S\V|P L/L/N S/K D/P I D\R(I)		127.12	466.7525	1863.992	-0.0041	-2.2	6.47	54427.4	5.96	HUMAN	K4DID9	Acetyl-CoA carboxylase 1
865	dirMS_041712_NAR_120mM_minus.14480.14500.4	4	18.53	1	5.55E+05		(R)ETNLDSI	(R)E T N L D S\L P/L/V/D/T/H S K/R(T)		133.47	456.992	1824.945	0.0012	0.6	5.38	53707.8	5.06	HUMAN	P08670	Vimentin
866	dirMS_041712_NAR_30mM_minus2.16876.17163.2	2	18.53	0	5.12E+05		(K)NSLESYA	(K)N S|L|E|S/Y/A/F/N/M K(A)		146.62	652.3031	1303.599	0.0002	0.2	6	45508.6	5.5	HUMAN	A8K7Q2	Heat shock cognate 71 kDa protein
867	dirMS_041712_NAR_40mM_minus2.12312.12312.3	3	18.53	0	4.42E+05		(K)IAILTCPI	(K)I A|I/L/T C/P F/E/P P K P K(T)	C:Carbamidomethylation	155.88	537.6382	1610.898	0.0026	1.6	8.59	49982	5.86	HUMAN	B4DYD8	T-complex protein 1 subunit epsilon

868	dirMS_041712_NAR_30mM_plus2.8400.8400.2	2	18.53	0	1.29E+06		(K)QGGGG	(K)Q G/G\G G/G G/G/S V/P G I/E R(M)		97.22	642.8178	1284.629	-0.0008	-0.6	6	77799.7	9.09	HUMAN	P52272	Heterogeneous nuclear ribonucleoprotein M
869	dirMS_041712_NAR_40mM_plus2.17055.17336.3	3	18.53	1	3.36E+05		(K)VIAATNI	(K)V/I|A|A/T/N R V/D\I/L/D/P A L L R(S)		168.1	617.3636	1850.086	-0.0096	-5.2	5.93	32827.5	5.92	HUMAN	E9PKD5	26S protease regulatory subunit 6A (Fragment)
870	dirMS_041712_NAR_50mM_plus2.15456.15458.3	3	18.53	1	8.05E+05		(K)EAFSLFD	(K)E A|F|S/L/F/D/K D G D G T I T/T/K(E)		149.45	615.6348	1844.891	-0.0014	-0.8	4.23	21860.1	4.45	HUMAN	E7EMB3	Calmodulin
871	dirMS_041712_NAR_30mM_minus2.15030.15420.2	2	18.51	0	1.29E+06		(K)FDLMYA	(K)F/D|L|M/Y/A/K(R)		136.1	444.2205	887.433	0.0006	0.6	5.83	50706.6	5.68	HUMAN	A6NHL2	Tubulin alpha chain-like 3
872	dirMS_041712_NAR_30mM_minus2.18176.18176.2	2	18.51	0	1.96E+05		(K)VADIGL	(K)V/A|D|I/G/L\A/A W G R(K)		152.82	564.8076	1128.616	-0.0081	-7.2	5.81	48285.8	5.93	HUMAN	P23526	Adenosylhomocysteinase
873	dirMS_041712_NAR_20mM_minus.14122.14122.3	3	18.5	0	1.04E+06		(K)SYELPDC	(K)S Y E\L/P D|G Q|V\I/T/I/G N E R(F)		194.2	597.6345	1790.892	-0.003	-1.7	4.14	123097.1	5.83	HUMAN	A5A3E0	POTE ankyrin domain family member F
874	dirMS_041712_NAR_50mM_minus2.7806.7806.3	3	18.5	1	5.68E+04		(R)LGAAPE	(R)L/G/A/A/P E E E S A Y V/A/G E K R(Q)		111.32	592.9614	1776.876	-0.0066	-3.7	4.5	30276.7	5	HUMAN	F2Z2K0	NSFL1 cofactor p47
875	dirMS_041712_NAR_60mM_minus.10174.10174.3	3	18.49	0	4.78E+06		(R)GSAVHC	(R)G S/A/V/H/Q/L|S/E/E L/R(C)		117.2	442.5648	1325.681	-0.001	-0.7	5.4	558007.1	5.44	HUMAN	P58107	Epiplakin
876	dirMS_041712_NAR_30mM_plus2.10927.11069.2	2	18.49	0	6.88E+05		(K)TGQEIP	(K)T/G|Q|E|I|P V/N V/R(F)		111	556.8073	1112.606	0.0015	1.3	5.66	28051.5	4.98	HUMAN	J3QKT2	Cytosolic non-specific dipeptidase (Fragment)
877	dirMS_041712_NAR_30mM_plus2.12226.12422.2	2	18.49	0	6.57E+05		(R)LAAEQE	(R)L/A|A|E|Q/E/L/I/R(L)		117.67	521.7961	1042.589	-0.0042	-4	4.53	533778	5.74	HUMAN	Q15149	Plectin
878	dirMS_041712_NAR_40mM_plus2.11399.11519.3	3	18.49	0	3.79E+05		(R)MPLPAP	(R)M/P/L|P/A/P S L S H\Q|P/P/P A P R(V)		133.63	598.3202	1792.953	-0.0066	-3.7	9.52	160853.1	6.11	HUMAN	F8VU51	YLP motif-containing protein 1
879	dirMS_041712_NAR_50mM_plus2.16549.16758.3	3	18.49	0	1.39E+06		(R)GFGHIG	(R)G F G H I\G I\A\V|P/D/V/Y/S A/C K(R)	C:Carbamidomethylation	155.57	597.634	1790.889	-0.002	-1.1	6.74	21005.6	5.12	HUMAN	Q04760	Lactoylglutathione lyase
880	dirMS_041712_NAR_50mM_minus2.12753.13155.3	3	18.48	1	3.42E+06		(R)TEMENE	(R)T|E|M/E/N E F/V/L/I/K/K(D)		141.77	494.2621	1480.772	0.0002	0.1	4.78	13588.1	4.66	HUMAN	F8VP67	Keratin, type II cytoskeletal 8 (Fragment)
881	dirMS_041712_NAR_50mM_minus2.15166.15420.3	3	18.47	0	9.01E+05		(R)LLIHQSL	(R)L L/I/H/Q\S\L|A/G/G I/I/G V/K(G)		154.83	506.9833	1518.937	-0.0013	-0.8	8.76	51260.6	5.39	HUMAN	P61978	Heterogeneous nuclear ribonucleoprotein K
882	dirMS_041712_NAR_120mM_plus.10758.10856.3	3	18.47	0	1.18E+06		(K)NFYQEH	(K)N/F/Y/Q E/H/P D/L A/R(R)		109.72	463.8906	1389.655	0.0027	1.9	5.32	61018.6	9.44	HUMAN	B4DLW8	Probable ATP-dependent RNA helicase DDX5
883	dirMS_041712_NAR_20mM_plus.15456.15810.2	2	18.47	0	1.28E+06	M73m	(R)AILVDLE	(R)A I|L|V|D|L|E|P G T/m D S V R(S)	m:Oxidized methionine	179.22	816.4181	1615.836	15.9929	-1.2	4.03	27549.9	4.39	HUMAN	E9PBJ4	Tubulin beta chain
884	dirMS_041712_NAR_30mM_plus2.8747.8928.2	2	18.47	0	8.74E+05		(R)VLNTNI	(R)V/L|N|T|N/I/D/G R(R)		100.02	501.2717	1001.537	-0.0013	-1.3	5.81	17718.4	10.99	HUMAN	P62269	40S ribosomal protein S18
885	dirMS_041712_NAR_30mM_plus2.9012.9012.2	2	18.47	0	6.30E+05		(R)SSQSSS	(R)S S/Q/S S/S Q/Q F|S|G/I/G\R(S)		101.03	728.345	1455.682	0.0005	0.3	9.47	73202	9.19	HUMAN	C9JMU5	Probable ATP-dependent RNA helicase DDX17
886	dirMS_041712_NAR_50mM_plus2.14408.14590.3	3	18.47	1	2.01E+06		(R)VAGGPC	(R)V/A/G/G/P Q M/I/Q/L/S L D/G K/R(L)		144.47	557.3066	1669.905	-0.0002	-0.1	8.72	45924.5	5.29	HUMAN	B4E1F3	Selenium-binding protein 1
887	dirMS_041712_NAR_50mM_plus2.8160.8206.3	3	18.47	0	2.70E+05		(K)STAGDT	(K)S|T|A/G D/T/H/L/G/G E D F D/N R(M)		109.7	564.5793	1691.726	-0.0022	-1.3	4.22	69032.8	5.37	HUMAN	E9PKE3	Heat shock cognate 71 kDa protein
888	dirMS_041712_NAR_30mM_minus2.15044.15108.2	2	18.46	0	7.03E+05		(K)SADTLW	(K)S A|D|T|L|W/G/I/Q/K(E)		135.23	559.8008	1118.584	0.0103	9.2	5.55	33940.9	9.27	HUMAN	F5H308	L-lactate dehydrogenase
889	dirMS_041712_NAR_60mM_plus.12901.12901.3	3	18.46	1	5.95E+05		(R)MAAPID	(R)M/A|A/P/I D R V G Q T I E/R(M)		124.35	519.6122	1556.821	0.0007	0.5	5.82	77799.7	9.09	HUMAN	P52272	Heterogeneous nuclear ribonucleoprotein M
890	dirMS_041712_NAR_60mM_plus.9218.9268.3	3	18.46	1	9.89E+05		(K)YAGKDG	(K)Y/A|G/K/D/G\Y N/Y T/L/S/K(T)		106.37	493.9098	1479.711	0.0034	2.3	8.38	11854.4	6.57	HUMAN	P31949	Protein S100-A11
891	dirMS_041712_NAR_60mM_plus.9975.10370.3	3	18.46	0	2.65E+07		(K)GIVEFSG	(K)G I|V/E/F/S/G K/P A A/R(K)		111.28	411.2315	1231.679	0.0006	0.5	8.75	23729.5	9.89	HUMAN	C9IZL7	Non-POU domain-containing octamer-binding protein (Fragment)
892	dirMS_041712_NAR_20mM_minus.13114.13259.2	2	18.45	0	1.21E+06	M73m	(R)AILVDLE	(R)A I|L|V|D|L|E|P G T/m D S V R(S)	m:Oxidized methionine	182.73	816.4184	1615.836	15.9935	-0.8	4.03	27549.9	4.39	HUMAN	E9PBJ4	Tubulin beta chain
893	dirMS_041712_NAR_30mM_minus2.10305.10559.2	2	18.45	0	1.45E+06		(R)AEAESM	(R)A E|A|E|S/M|Y/Q/I/K(Y)		110.47	585.2783	1169.551	-0.0014	-1.2	4.53	37296.3	5.3	HUMAN	F8VU64	Keratin, type II cytoskeletal 8
894	dirMS_041712_NAR_60mM_minus.6338.6844.3	3	18.45	1	3.02E+06		(K)IQDKEGI	(K)I Q|D K E G|I|P/P/D/Q Q R(L)		94.92	508.5978	1523.781	-0.0024	-1.6	4.56	17271.6	6.75	HUMAN	B4DV12	Ubiquitin
895	dirMS_041712_NAR_120mM_minus.13498.13623.4	4	18.44	2	8.32E+05	M100m	(K)KQGGLG	(K)K\Q G G L\G|P m|N\I/P/L/V/S D/P K R(T)	m:Oxidized methionine	128.55	481.5173	1907.053	15.9942	-0.3	9.99	22338.2	8.65	HUMAN	Q06830	Peroxiredoxin-1
896	dirMS_041712_NAR_120mM_minus.14864.14921.3	3	18.44	0	3.67E+05		(K)HLEINPD	(K)H/L|E|I\N\P D|H|P/I V/E T\L R(Q)		135.25	594.9872	1782.95	-0.0027	-1.5	5.27	83605	4.97	HUMAN	P08238	Heat shock protein HSP 90-beta
897	dirMS_041712_NAR_40mM_minus2.6432.6432.3	3	18.44	1	1.94E+05		(K)GAVAED	(K)G A|V/A|E/D/G D E L R T E P E A/K(K)		104.53	596.2893	1786.845	0.008	4.5	4.08	19284.3	5.23	HUMAN	G3V3C7	DNA-(apurinic or apyrimidinic site) lyase (Fragment)
898	dirMS_041712_NAR_40mM_plus2.14601.14684.3	3	18.44	0	5.43E+05		(K)IAILTCPF	(K)I/A|I|L/T/C/P F E/P P K P K(T)	C:Carbamidomethylation	153.55	537.6381	1610.898	0.0023	1.4	8.59	49982	5.86	HUMAN	B4DYD8	T-complex protein 1 subunit epsilon
899	dirMS_041712_NAR_60mM_plus.6792.6801.3	3	18.44	1	1.28E+06		(K)SLDEME	(K)S/L|D/E/M/E/K/Q/Q/R(E)		94.13	421.8717	1263.6	0.0008	0.6	4.68	26419.2	5.1	HUMAN	H0Y9K7	Splicing factor, proline- and glutamine-rich (Fragment)
900	dirMS_041712_NAR_50mM_minus2.10413.10880.3	3	18.43	1	7.18E+06	M72m	(R)TEmENE	(R)T|E|m/E N E|F/V L/I/K/K(D)	m:Oxidized methionine	129.07	499.5937	1480.772	15.995	0	4.78	13588.1	4.66	HUMAN	F8VP67	Keratin, type II cytoskeletal 8 (Fragment)
901	dirMS_041712_NAR_50mM_minus2.13801.13801.3	3	18.43	0	2.28E+06		(R)VAPDEH	(R)V A|P D E H P I L/L/T/E A/P L N P K(I)		146.07	652.0252	1954.064	-0.0034	-1.7	4.65	42344.8	5.39	HUMAN	Q562R1	Beta-actin-like protein 2
902	dirMS_041712_NAR_50mM_minus2.13951.14163.3	3	18.43	1	1.67E+06		(R)ELISNAS	(R)E L\I/S/N/A/S|D|A L/D/K I/R(L)		148.03	515.6179	1544.828	0.0113	7.3	4.56	5582.4	4.81	HUMAN	F8W026	Endoplasmin (Fragment)
903	dirMS_041712_NAR_20mM_plus.8127.8127.2	2	18.43	0	2.02E+05		(R)FGQGG	(R)F G/Q|G/G/A/G/P V/G G/Q/G P R(G)		125.25	671.3361	1341.666	-0.0009	-0.7	9.75	76262.2	9.53	HUMAN	P23246	Splicing factor, proline- and glutamine-rich
904	dirMS_041712_NAR_40mM_plus2.9869.9890.3	3	18.43	0	1.23E+05		(R)VMLGET	(R)V/M|L|G E T/N|P/A D/S K/P G T I R(G)		122.05	595.9778	1785.916	0.0025	1.4	6.04	15431.7	9	HUMAN	E7ERL0	Nucleoside diphosphate kinase A
905	dirMS_041712_NAR_30mM_plus2.12199.12199.2	2	18.42	0	9.10E+05		(K)EVDEQM	(K)E V|D E/Q/M L/N/V/Q/N K(N)		117.03	723.8478	1446.689	-0.001	-0.7	4.14	40069.4	4.83	HUMAN	E7EWR1	Tubulin beta chain
906	dirMS_041712_NAR_30mM_plus2.15986.16336.2	2	18.42	0	1.74E+06		(R)MGAGM	(R)M/G|A|G|M|G/F/G L/E/R(M)		137.58	563.2636	1125.518	0.002	1.7	5.75	77799.7	9.09	HUMAN	P52272	Heterogeneous nuclear ribonucleoprotein M
907	dirMS_041712_NAR_50mM_plus2.9972.10073.3	3	18.42	1	7.62E+05		(K)ITIKNDP	(K)I/T|I/K N D|P/S|L/P E/P K(C)		119.92	484.6079	1451.81	-0.0013	-0.9	6.07	20203.8	9.79	HUMAN	B4DG16	Asparagine--tRNA ligase, cytoplasmic
908	dirMS_041712_NAR_50mM_minus2.14854.14854.3	3	18.41	1	2.91E+05		(R)EAVTEIL	(R)E A V/T/E/I/L/G I/E/P D R E K(G)		152.33	566.9683	1698.891	-0.0005	-0.3	4.25	25685.9	6.4	HUMAN	E9PGT1	Translin
909	dirMS_041712_NAR_60mM_minus.16116.16361.3	3	18.41	1	5.82E+05		(K)LKLSILYF	(K)L K\L S I\L|Y|P/A/T T G R(N)		150.5	478.2883	1432.852	-0.0019	-1.3	9.99	25148.6	6	HUMAN	P30041	Peroxiredoxin-6
910	dirMS_041712_NAR_60mM_minus.18333.18333.3	3	18.41	1	4.80E+05		(K)TQLNLLI	(K)T/Q|L/N/L L/L/D/K L R(S)		163.37	442.9403	1326.81	-0.004	-3	8.41	150202.8	9.1	HUMAN	E7EW20	Unconventional myosin-VI
911	dirMS_041712_NAR_30mM_plus2.8964.8964.2	2	18.41	0	1.80E+06		(K)TAVCDIF	(K)T A|V/C/D/I|P\P/R(G)	C:Carbamidomethylation	100.42	514.7643	1028.519	0.002	1.9	5.5	50199.4	4.75	HUMAN	A6NNZ2	Tubulin beta-8 chain-like protein LOC260334
912	dirMS_041712_NAR_60mM_plus.6787.6787.3	3	18.41	1	7.29E+05		(R)AQLGGP	(R)A Q|L/G G/P E A/A K S D E T A\A K(-)		93.87	548.6115	1643.824	-0.0036	-2.2	4.68	3888.3	5	HUMAN	C9J3N8	Heat shock protein beta-1
913	dirMS_041712_NAR_50mM_plus2.15986.16170.3	3	18.4	1	1.29E+06		(K)GADFLV	(K)G A|D|F|L|V/T/E|V/E/N G G S L G S K K(G)		152.28	636.6618	1907.971	-0.0001	0	4.68	30947.9	6.47	HUMAN	H3BQ34	Pyruvate kinase
914	dirMS_041712_NAR_60mM_plus.7460.7684.3	3	18.4	1	7.87E+06		(R)VITEEEK	(R)V/I|T/E/E/E/K N F/K(A)		97.9	412.8904	1236.647	0.0096	7.8	4.79	18901.8	11.65	HUMAN	F5H1S2	60S ribosomal protein L13
915	dirMS_041712_NAR_50mM_minus2.11769.11950.3	3	18.39	1	6.11E+05		(R)ELISNSSI	(R)E L|I/S/N/S/S/D/A\L D/K I R(Y)		135.85	520.9445	1560.823	-0.0038	-2.5	4.56	20151.2	4.58	HUMAN	G3V2J8	Heat shock protein HSP 90-alpha (Fragment)
916	dirMS_041712_NAR_50mM_minus2.12818.12818.3	3	18.39	1	3.63E+05		(R)LSEDYG	(R)L/S/E/D Y/G V/L/K T/D E G I A Y R(G)		141.42	643.6561	1928.96	-0.0063	-3.2	4.32	22062.7	5.66	HUMAN	P32119	Peroxiredoxin-2
917	dirMS_041712_NAR_120mM_plus.8155.8171.3	3	18.39	0	1.78E+06		(K)HSGPNS	(K)H S G P N\S\A|D|S|A/N/D/G F/V/R(L)		96.53	544.2457	1630.72	0.0021	1.3	5.21	46013.4	5.38	HUMAN	P52597	Heterogeneous nuclear ribonucleoprotein F
918	dirMS_041712_NAR_30mM_plus2.17842.17889.2	2	18.39	0	3.47E+05		(R)MADALI	(R)M/A|D|A|L|D/N/Y\V/I/R(G)		147.02	640.8182	1280.63	-0.0012	-1	4.21	15838.9	7.03	HUMAN	H0Y5U0	Propionyl-CoA carboxylase alpha chain, mitochondrial (Fragment)
919	dirMS_041712_NAR_30mM_plus2.18982.18982.2	2	18.39	0	3.18E+06		(R)QAQEYE	(R)Q A Q|E/Y/E/A L/L/N\I/K(V)		153.23	710.3766	1419.748	-0.0019	-1.3	4.53	43773.3	5.26	HUMAN	F8VZY9	Keratin, type I cytoskeletal 18
920	dirMS_041712_NAR_50mM_plus2.10378.10406.3	3	18.39	0	5.32E+05		(R)LQAQQI	(R)L/Q|A\Q/Q D A\V/N/I/V/C H/S K(T)	C:Carbamidomethylation	122.2	570.9571	1710.859	-0.0024	-1.4	6.74	21953.7	6.6	HUMAN	A6PVX3	26S proteasome non-ATPase regulatory subunit 4 (Fragment)
921	dirMS_041712_NAR_50mM_plus2.11206.11350.3	3	18.39	0	1.71E+06		(R)GITINAA	(R)G I|T|I/N/A/A/H/V/E Y S T A A R(H)		127.07	558.6237	1673.861	-0.004	-2.4	6.75	49883	7.3	HUMAN	P49411	Elongation factor Tu, mitochondrial
922	dirMS_041712_NAR_50mM_plus2.20952.21302.3	3	18.39	0	8.90E+06		(R)LHFFMP	(R)L H\F\F\M\P G|F|A|P|L/T S/R(G)		181.53	540.9498	1620.835	-0.0007	-0.4	9.75	42230.8	4.77	HUMAN	A6NKZ8	Putative tubulin beta chain-like protein ENSP00000290377
923	dirMS_041712_NAR_60mM_plus.11217.11235.3	3	18.39	1	1.69E+06		(K)VIADNV	(K)V/I|A/D/N/V/K/D W S/K(V)		116.35	425.563	1274.674	0.0005	0.4	5.93	31075.7	5.65	HUMAN	P60174	Triosephosphate isomerase
924	dirMS_041712_NAR_30mM_minus2.10201.10788.2	2	18.38	0	3.78E+06		(K)TAVCDIF	(K)T A|V|C/D|I|P|P/R(G)	C:Carbamidomethylation	109.92	514.7641	1028.519	0.0016	1.5	5.5	50199.4	4.75	HUMAN	A6NNZ2	Tubulin beta-8 chain-like protein LOC260334
925	dirMS_041712_NAR_40mM_minus2.11962.11962.3	3	18.38	0	2.64E+05		(K)DAGEGL	(K)D A|G|E/G L/L A/V/Q I/T D/P E G K\P K(K)		153.95	646.6785	1938.018	0.0031	1.6	4.32	283472.4	5.71	HUMAN	P21333	Filamin-A
926	dirMS_041712_NAR_50mM_minus2.15712.15712.3	3	18.38	0	2.54E+05	M460m	(R)ISIEmNG	(R)I/S|I/E/m N G T L E/D Q L/S H L/K(Q)	m:Oxidized methionine	157.28	648.6635	1927.979	15.9966	0.9	4.65	80254.2	5.05	HUMAN	D6PXK4	Alpha actinin 4 short isoform
927	dirMS_041712_NAR_50mM_minus2.9254.9387.3	3	18.38	1	6.52E+05		(K)VTVAGL	(K)V/T|V|A|G/L/A|G K/D/P V Q/C S R(D)	C:Carbamidomethylation	121.4	553.2936	1657.869	-0.0028	-1.7	8.72	18080.7	7.92	HUMAN	K7ELW0	Protein DJ-1
928	dirMS_041712_NAR_30mM_plus2.11976.11976.2	2	18.38	0	4.31E+05		(K)AYGGAY	(K)A/Y|G/G|A|Y D/V M S S K(H)		115.77	624.7816	1248.556	-0.0006	-0.5	5.88	61363	8.73	HUMAN	C9JQS9	Propionyl-CoA carboxylase beta chain, mitochondrial
929	dirMS_041712_NAR_40mM_plus2.18490.18945.3	3	18.38	0	8.75E+05		(R)DYLHLPI	(R)D Y L H\L\P\P E\I|V|P/A/T L R(R)		176.45	578.6565	1733.959	-0.0035	-2	5.32	19924.8	9.99	HUMAN	F6U211	40S ribosomal protein S10
930	dirMS_041712_NAR_50mM_plus2.17429.17555.3	3	18.37	0	4.75E+05		(R)DLISHDE	(R)D L|I/S/H D E\M/F/S D I/Y K(I)		160.08	571.5992	1712.784	-0.0006	-0.3	4.22	19709.1	4.83	HUMAN	P13693	Translationally-controlled tumor protein
931	dirMS_041712_NAR_50mM_plus2.6002.6002.3	3	18.37	0	1.14E+05		(R)AGAAG	(R)A/G|A|A/G/G/P E E/E/A E K P V K(T)		95.73	513.9259	1539.765	-0.0018	-1.2	4.49	18924.5	6.86	HUMAN	Q8WW12	PEST proteolytic signal-containing nuclear protein
932	dirMS_041712_NAR_60mM_plus.7542.7542.2	2	18.37	1	7.47E+05		(R)VITEEEK	(R)V/I|T/E/E E K N F K(A)		97.92	618.8282	1236.647	0.0021	1.7	4.79	18901.8	11.65	HUMAN	F5H1S2	60S ribosomal protein L13
933	dirMS_041712_NAR_20mM_plus.18694.18694.2	2	18.36	0	2.87E+05		(K)LAVEALS	(K)L A V|E\A|L|S/S/L/D/G D L A G R(Y)		200.48	793.9214	1586.838	-0.0029	-1.8	4.03	24286.7	4.89	HUMAN	G3V4N7	Creatine kinase B-type (Fragment)
934	dirMS_041712_NAR_30mM_plus2.21270.21393.2	2	18.36	0	3.90E+05		(R)VNQAIW	(R)V N|Q|A|I|W/L|L|C/T G A R(E)	C:Carbamidomethylation	166.88	751.3976	1501.794	-0.0065	-4.3	9.72	23047.2	9.95	HUMAN	P46782	40S ribosomal protein S5
935	dirMS_041712_NAR_120mM_minus.7948.8178.2	2	18.35	0	1.65E+07		(R)HQGVM	(R)H|Q|G\V|M|V|G\M|G Q|K(D)		98.12	586.2907	1171.571	0.0031	2.6	8.76	32616.5	5.18	HUMAN	A6NL76	Actin, alpha skeletal muscle
936	dirMS_041712_NAR_40mM_plus2.7485.7910.2	2	18.35	0	6.83E+05		(K)ASLENSI	(K)A/S|L/E/N/S/L/R(E)		105.45	445.2395	889.474	-0.002	-2.3	6.05	43773.3	5.26	HUMAN	F8VZY9	Keratin, type I cytoskeletal 18
937	dirMS_041712_NAR_50mM_minus2.8902.8902.3	3	18.34	0	2.36E+05		(R)VMLGET	(R)V M/L/G/E T N/P A D S K/P G T I R(G)		118.3	595.9744	1785.916	-0.0077	-4.3	6.04	15431.7	9	HUMAN	E7ERL0	Nucleoside diphosphate kinase A
938	dirMS_041712_NAR_30mM_plus2.17985.18018.2	2	18.34	0	3.00E+05		(R)VNPALA	(R)V/N|P/A|L|A/E L/N L/R(S)		147.82	605.3509	1209.695	-0.0005	-0.4	5.97	20026.6	4.67	HUMAN	E9PIK5	Ribonuclease inhibitor (Fragment)
939	dirMS_041712_NAR_40mM_minus2.12094.12094.3	3	18.33	0	2.65E+05		(K)IAILTCPF	(K)I A|I\L T/C/P F/E/P P K/P K(T)	C:Carbamidomethylation	155.12	537.6382	1610.898	0.0026	1.6	8.59	49982	5.86	HUMAN	B4DYD8	T-complex protein 1 subunit epsilon
940	dirMS_041712_NAR_60mM_minus.14304.14549.3	3	18.33	0	3.65E+05		(R)VWYVSN	(R)V/W|Y/V/S/N I/D/G T H I/A/K(T)		140.38	534.9461	1602.828	-0.0037	-2.3	6.71	63374.2	8.64	HUMAN	P06744	Glucose-6-phosphate isomerase
941	dirMS_041712_NAR_20mM_plus.16614.16972.2	2	18.33	0	4.12E+05		(K)VQIAVA	(K)V Q|I|A|V|A/N/A Q E L/L Q/R(M)		187.28	776.9419	1552.881	-0.004	-2.6	5.97	10728.1	9.15	HUMAN	Q9Y5L4	Mitochondrial import inner membrane translocase subunit Tim13
942	dirMS_041712_NAR_30mM_plus2.22545.22643.3	3	18.33	0	1.19E+06		(R)LEGLTDE	(R)L E\G\L\T D\E|I/N/F/L/R(Q)		175.42	473.9205	1419.748	-0.0009	-0.6	4.14	13588.1	4.66	HUMAN	F8VP67	Keratin, type II cytoskeletal 8 (Fragment)
943	dirMS_041712_NAR_30mM_plus2.9547.10051.2	2	18.33	0	4.96E+05		(K)TAVCDIF	(K)T A|V|C|D|I|P|P/R(G)	C:Carbamidomethylation	105.07	514.7643	1028.519	0.002	1.9	5.5	50199.4	4.75	HUMAN	A6NNZ2	Tubulin beta-8 chain-like protein LOC260334
944	dirMS_041712_NAR_30mM_minus2.10957.10957.2	2	18.32	0	5.68E+05		(K)VAGQD	(K)V/A|G Q D/G S/V V/Q/F K(I)		113.77	617.824	1234.643	-0.0019	-1.5	5.81	17127.4	10.05	HUMAN	A8MU27	Small ubiquitin-related modifier 3
945	dirMS_041712_NAR_120mM_plus.15314.15459.3	3	18.32	0	9.71E+05		(K)FYVHND	(K)F/Y/V/H/N D/I/F/R(Y)		133.88	404.2038	1210.6	-0.0035	-2.9	6.74	17042.2	9.19	HUMAN	E5RIZ6	Ras GTPase-activating protein-binding protein 1 (Fragment)
946	dirMS_041712_NAR_20mM_minus.14662.14662.2	2	18.3	0	2.10E+05		(K)LAVEALS	(K)L A\V E/A|L|S/S L/D|G D L A G R(Y)		201.28	793.922	1586.838	-0.0017	-1.1	4.03	24286.7	4.89	HUMAN	G3V4N7	Creatine kinase B-type (Fragment)
947	dirMS_041712_NAR_30mM_minus2.9418.9499.2	2	18.3	0	1.28E+06		(K)SVEAAA	(K)S V|E|A|A|A/E|L/S A/K(D)		105.75	538.2838	1075.563	-0.0027	-2.5	4.53	12131.5	11.14	HUMAN	F5GXR3	Parathymosin
948	dirMS_041712_NAR_40mM_minus2.13737.13737.3	3	18.3	1	3.98E+05		(R)VTFSGLF	(R)V/T|F/S|G L R\D T V/T P G E L L K(A)		164.73	611.6742	1833.012	-0.0036	-2	6.04	266161	5.68	HUMAN	E9PPU0	Epiplakin
949	dirMS_041712_NAR_50mM_minus2.16863.16869.3	3	18.3	1	2.96E+05		(R)SELELTL	(R)S E|L/E/L/T/L/G/K L/E/Q V R(S)		163.73	538.9725	1614.906	-0.0032	-2	4.78	533778	5.74	HUMAN	Q15149	Plectin
950	dirMS_041712_NAR_60mM_minus.6708.7196.3	3	18.3	0	1.27E+06	M434m	(R)LESGmC	(R)L/E|S/G m Q/N/M/S I|H/T K(T)	m:Oxidized methionine	97.23	497.9023	1475.698	15.9942	-0.5	6.75	56607.4	5.37	HUMAN	F8VXB4	Keratin, type II cytoskeletal 8
951	dirMS_041712_NAR_120mM_plus.20098.20417.3	3	18.3	1	3.71E+06		(K)AAIDWF	(K)A A|I/D|W/F/D/G K E F/H/G N I I|K(V)		158.78	654.3334	1960.992	-0.0059	-3	5.38	49181	4.93	HUMAN	K7EPT6	TATA-binding protein-associated factor 2N
952	dirMS_041712_NAR_120mM_plus.6714.6765.3	3	18.3	0	4.95E+05		(K)NNRPSE	(K)N/N/R\P S E|G|P/L/Q/T R(L)		88.55	456.902	1368.698	-0.0064	-4.7	9.6	193379.8	5.48	HUMAN	Q00610	Clathrin heavy chain 1
953	dirMS_041712_NAR_60mM_plus.5734.5734.3	3	18.3	1	1.91E+05		(R)SSGGGY	(R)S/S/G/G G/Y/S G D R S G G G/Y G G D\R(S)		88.07	583.5777	1748.722	-0.0033	-1.9	5.68	21563.9	10.01	HUMAN	K7EKB2	TATA-binding protein-associated factor 2N (Fragment)
954	dirMS_041712_NAR_30mM_plus2.16969.16969.2	2	18.29	0	5.39E+05	M256m	(R)SLDmDS	(R)S L/D\m/D S/I I|A E/V/K(A)	m:Oxidized methionine	142.17	668.8384	1320.671	15.998	2.3	4.03	37296.3	5.3	HUMAN	F8VU64	Keratin, type II cytoskeletal 8
955	dirMS_041712_NAR_30mM_plus2.8630.8753.2	2	18.29	0	7.03E+06		(K)ASLENSI	(K)A/S|L/E|N/S/L/R(E)		99.25	445.2405	889.474	0	-0.1	6.05	43773.3	5.26	HUMAN	F8VZY9	Keratin, type I cytoskeletal 18
956	dirMS_041712_NAR_50mM_minus2.16446.16446.3	3	18.28	0	4.54E+05		(R)VLDPFTI	(R)V/L/D/P F/T/I K/P L D/R(K)		161.98	471.9411	1413.81	-0.0013	-0.9	5.93	63448.4	7.29	HUMAN	B4E022	Transketolase
957	dirMS_041712_NAR_60mM_minus.10683.10683.3	3	18.28	1	5.44E+05	M239m	(R)MSMKE	(R)M/S/M K E V D E Q m|L/N|V|Q N K(N)	m:Oxidized methionine	119.9	647.3011	1923.897	15.9915	-1.8	4.68	40069.4	4.83	HUMAN	E7EWR1	Tubulin beta chain
958	dirMS_041712_NAR_20mM_plus.10629.10629.3	3	18.28	0	8.07E+04		(K)TTTLSGT	(K)T T T L S G T A|P A/A/G V/V|P S R(V)		145.97	529.2872	1585.854	-0.0074	-4.6	9.41	26233.5	10.95	HUMAN	B4DSQ1	Microtubule-associated protein
959	dirMS_041712_NAR_50mM_plus2.12363.12551.3	3	18.28	1	1.70E+06		(R)SGKAPIL	(R)S G K A P I\L|I|A|T/D/V|A/S/R(G)		133.47	500.2911	1498.859	0	0	8.46	73202	9.19	HUMAN	C9JMU5	Probable ATP-dependent RNA helicase DDX17
960	dirMS_041712_NAR_60mM_plus.12653.12866.3	3	18.28	1	1.08E+06		(R)DLDRIA	(R)D L/D R\I\A|G\Q|V/A/A/A/N K(K)		123.85	481.263	1441.776	-0.0013	-0.9	5.96	16060.2	10.31	HUMAN	P39019	40S ribosomal protein S19
961	dirMS_041712_NAR_120mM_plus.5255.5378.3	3	18.27	0	4.05E+05		(R)YIDTEH	(R)Y/I|D/T/E/H/G/G/S Q A R(F)		80.98	445.2109	1333.613	0.005	3.8	5.32	63613.4	7.9	HUMAN	B3KXI2	Protein transport protein Sec23A
962	dirMS_041712_NAR_20mM_plus.9866.9866.2	2	18.27	0	2.24E+05		(K)IVVVTA	(K)I/V|V|V/T A G V R(Q)		139.22	457.2945	913.583	-0.0012	-1.3	9.75	25445.8	5.52	HUMAN	A8MW50	L-lactate dehydrogenase (Fragment)
963	dirMS_041712_NAR_20mM_minus.13222.13222.2	2	18.26	0	2.34E+05		(R)GVVDSE	(R)G V|V/D|S/E D|I|P\L N/L/S\R(E)		183.02	757.3944	1513.786	-0.0041	-2.7	4.03	74494.3	7.79	HUMAN	F5H897	Heat shock protein 75 kDa, mitochondrial
964	dirMS_041712_NAR_50mM_minus2.5832.5860.3	3	18.26	1	1.62E+06		(R)AQLGGP	(R)A Q|L|G/G/P E A/A/K/S D/E T A A/K(-)		95.6	548.6119	1643.824	-0.0024	-1.4	4.68	3888.3	5	HUMAN	C9J3N8	Heat shock protein beta-1
965	dirMS_041712_NAR_120mM_plus.13369.13557.3	3	18.26	0	1.45E+06		(K)YNPTWI	(K)Y/N|P/T/W/H/C I/V G/R(N)	C:Carbamidomethylation	123.37	468.2345	1402.669	0.0205	14.6	8.75	10536.9	6.9	HUMAN	P63167	Dynein light chain 1, cytoplasmic
966	dirMS_041712_NAR_30mM_plus2.15319.15464.2	2	18.26	0	4.57E+05		(R)MAGAIA	(R)M/A|G|A|I/A/Q/G L/I/R(A)		133.38	550.816	1100.624	0.0002	0.2	9.5	4449.1	11.42	HUMAN	F8WEI0	Pyrroline-5-carboxylate reductase 3
967	dirMS_041712_NAR_30mM_plus2.17062.17558.2	2	18.26	0	1.21E+06		(R)MNVLAI	(R)M/N|V|L/A/D/A/L/K(S)		143.63	487.7743	974.534	0.0074	7.6	5.59	27396	9.9	HUMAN	H3BN98	Uncharacterized protein (Fragment)
968	dirMS_041712_NAR_30mM_plus2.20053.20053.2	2	18.26	0	2.32E+05		(K)LALFQAI	(K)L/A|L/F/Q A/I/G K(E)		158.63	480.795	960.588	-0.005	-5.2	8.75	266161	5.68	HUMAN	E9PPU0	Epiplakin
969	dirMS_041712_NAR_30mM_plus2.22861.22959.2	2	18.26	0	1.84E+06		(R)GFGFVLI	(R)G/F|G|F/V/L|F/K(E)		179.85	457.76	914.514	-0.0007	-0.8	8.75	33782.7	8.78	HUMAN	B4DTC3	Heterogeneous nuclear ribonucleoprotein D0
970	dirMS_041712_NAR_50mM_plus2.18653.18653.3	3	18.26	0	3.54E+05		(R)LLLDGAI	(R)L L|L/D G A/P/L/I A I H K(A)		165.82	458.622	1373.852	-0.0001	0	6.74	16469.8	8	HUMAN	K7EMY7	Haloacid dehalogenase-like hydrolase domain-containing protein 2 (Fragment)
971	dirMS_041712_NAR_30mM_plus2.9565.9565.2	2	18.25	0	3.57E+05		(R)LLTYNA	(R)L/L/T/Y/N/A A/R(L)		104.33	461.2586	921.515	-0.0053	-5.8	8.75	36309.7	5.86	HUMAN	B4DQ51	Short/branched chain-specific acyl-CoA dehydrogenase, mitochondrial
972	dirMS_041712_NAR_50mM_plus2.9483.9862.3	3	18.25	0	2.45E+06		(K)GIVEFAS	(K)G I|V|E/F/A S/K P A A/R(K)		118.82	415.9028	1245.695	-0.0012	-0.9	8.75	76262.2	9.53	HUMAN	P23246	Splicing factor, proline- and glutamine-rich
973	dirMS_041712_NAR_20mM_minus.8911.9148.2	2	18.24	0	1.86E+06		(K)LVSESSD	(K)L/V|S|E/S/S D\V/L/P K(-)		139.67	587.321	1173.636	-0.0014	-1.2	4.37	56607.4	5.37	HUMAN	F8VXB4	Keratin, type II cytoskeletal 8
974	dirMS_041712_NAR_30mM_minus2.19883.20040.2	2	18.24	0	2.49E+06		(K)EIIDLVLI	(K)E I|I|D|L|V/L/D/R(I)		167.68	543.3132	1085.62	-0.001	-0.9	4.03	58642	4.95	HUMAN	F5H5D3	Tubulin alpha-1C chain
975	dirMS_041712_NAR_50mM_minus2.13163.13214.3	3	18.24	0	3.97E+05		(K)VTHAVV	(K)V T H\A V\V\T\V|P/A/Y/F/N D/A Q/R(Q)		143.42	629.9954	1887.971	0.0005	0.3	6.71	72445.7	5.07	HUMAN	P11021	78 kDa glucose-regulated protein
976	dirMS_041712_NAR_50mM_minus2.16807.16807.3	3	18.24	1	2.99E+05		(R)SELELTL	(R)S E|L/E/L/T/L/G K L E/Q V R(S)		163.72	538.9725	1614.906	-0.0032	-2	4.78	533778	5.74	HUMAN	Q15149	Plectin

977	dirMS_041712_NAR_20mM_plus.14128.14128.2	2	18.24	0	7.05E+04	M313m	(R)TVQSLEI	(R)T V|Q|S L/E|I D L/D S m R(N)	m:Oxidized methionine	169.18	761.8738	1506.747	15.9935	-0.9	4.03	43773.3	5.26	HUMAN	F8VZY9	Keratin, type I cytoskeletal 18
978	dirMS_041712_NAR_30mM_plus2.16361.16530.2	2	18.24	0	7.76E+04		(K)AELNEFL	(K)A E|L|N/E|F/L/T/R(E)		137.2	546.7844	1092.568	-0.0069	-6.3	4.53	13054.7	9.59	HUMAN	E9PJH4	40S ribosomal protein S3
979	dirMS_041712_NAR_30mM_plus2.7671.7718.2	2	18.24	0	9.82E+05		(R)VLEEGS\	(R)V/L|E|E/G S/V/E/A/R(T)		94.18	544.7829	1088.558	0.0003	0.3	4.25	25834.8	9.54	HUMAN	Q96CT7	Coiled-coil domain-containing protein 124
980	dirMS_041712_NAR_50mM_plus2.13226.13226.3	3	18.24	1	9.66E+05		(R)ELNITAA	(R)E L/N|I/T/A/A\K/E I E/V/G G G R(K)		137.7	552.9671	1656.892	-0.0048	-2.9	4.79	21311.5	10.28	HUMAN	B5MCP9	40S ribosomal protein S7
981	dirMS_041712_NAR_60mM_plus.20144.20144.3	3	18.24	0	2.36E+05		(K)ALSAIAD	(K)A L|S A/I A D/L/L/T/N E H E/R(V)		165.23	551.6246	1652.86	-0.001	-0.6	4.65	105417.2	6.41	HUMAN	C9JZR2	Catenin delta-1
982	dirMS_041712_NAR_20mM_plus.16162.16180.2	2	18.23	0	4.91E+05		(K)DAGTIA(	(K)D A|G\T|I/A|G/L/N/V/L/R(I)		183.63	600.3422	1199.674	0.0029	2.4	5.84	17973	5.16	HUMAN	E9PI65	Heat shock cognate 71 kDa protein (Fragment)
983	dirMS_041712_NAR_30mM_plus2.11385.11537.2	2	18.23	0	4.41E+05		(R)FASGGC	(R)F/A|S|G/G/C/D N L|I/K(L)	C:Carbamidomethylation	113.55	591.286	1181.562	0.0028	2.4	5.84	34524.7	5.4	HUMAN	A8MV37	Protein SEC13 homolog
984	dirMS_041712_NAR_30mM_plus2.7454.7455.2	2	18.23	0	1.02E+07		(K)AGFAGD	(K)A/G|F|A|G/D/D/A/P/R(A)		93.67	488.7269	976.448	-0.0018	-1.8	4.21	123097.1	5.83	HUMAN	A5A3E0	POTE ankyrin domain family member F
985	dirMS_041712_NAR_50mM_minus2.7106.7106.3	3	18.22	0	1.07E+05		(R)VGQAVD	(R)V/G|Q/A/V D V/V/G Q/A G K P K(T)		107.38	484.9436	1452.817	-0.0007	-0.5	8.56	86346.5	5.05	HUMAN	E7EW34	26S proteasome non-ATPase regulatory subunit 2
986	dirMS_041712_NAR_30mM_plus2.22026.22026.2	2	18.22	0	1.56E+05		(R)QSSFALI	(R)Q S/S F/A/L/L/G/D L T K(A)		170.63	640.3472	1279.689	-0.0021	-1.7	5.84	46579.3	4.96	HUMAN	E5RI07	Transportin-1
987	dirMS_041712_NAR_30mM_plus2.7504.7520.2	2	18.22	0	4.91E+05		(K)YQAECS	(K)Y/Q|A|E|C/S/Q/F K(A)	C:Carbamidomethylation	93.72	580.7556	1160.504	-0.0002	-0.1	6	334221.3	6.44	HUMAN	P15924	Desmoplakin
988	dirMS_041712_NAR_50mM_minus2.9057.9057.3	3	18.21	0	7.04E+04		(R)EANALA	(R)E A N/A/L/A/A G H/P A Q A V/A I N A R(L)		119.77	615.6611	1844.973	-0.0038	-2.1	6.85	272689.3	5.79	HUMAN	O15020	Spectrin beta chain, non-erythrocytic 2
989	dirMS_041712_NAR_20mM_minus.10099.10099.2	2	18.2	0	1.24E+05		(R)YALYDA	(R)Y/A|L|Y|D/A T/Y E\T K(E)		150.82	669.3156	1337.626	-0.0021	-1.5	4.37	22956	8.8	HUMAN	E9PK25	Cofilin-1
990	dirMS_041712_NAR_60mM_minus.9496.9496.3	3	18.2	0	3.19E+05		(R)QLSQAG	(R)Q L/S/Q/A/G S/F/S|D/G T H G G L R(Y)		113.52	573.2781	1717.825	-0.0055	-3.2	6.74	266161	5.68	HUMAN	E9PPU0	Epiplakin
991	dirMS_041712_NAR_50mM_plus2.17748.18021.3	3	18.2	0	2.13E+06		(K)LILPHVD	(K)L/I|L/P/H/V/D/I Q/L K(Y)		162.02	430.2706	1288.799	-0.0015	-1.2	6.74	45578.2	7.83	HUMAN	B4DFL2	Isocitrate dehydrogenase [NADP]
992	dirMS_041712_NAR_30mM_minus2.18425.18425.2	2	18.19	0	4.47E+05		(R)QAQEYE	(R)Q/A/Q E|Y/E/A L L/N I/K(V)		154.67	710.3759	1419.748	-0.0033	-2.3	4.53	43773.3	5.26	HUMAN	F8VZY9	Keratin, type I cytoskeletal 18
993	dirMS_041712_NAR_60mM_minus.15367.15367.3	3	18.19	0	1.09E+06		(R)LSDQFH	(R)L/S|D Q F/H/D/I/L/I R(K)		145.58	452.9148	1356.727	0.0028	2.1	5.21	12284.6	4.89	HUMAN	H0Y9X3	Programmed cell death protein 6 (Fragment)
994	dirMS_041712_NAR_60mM_minus.9279.9335.3	3	18.19	1	6.00E+05		(K)LEAEIAT	(K)L/E|A/E/I/A T Y R/R(L)		111.95	407.8897	1221.659	-0.0041	-3.3	6.14	43773.3	5.26	HUMAN	F8VZY9	Keratin, type I cytoskeletal 18
995	dirMS_041712_NAR_30mM_plus2.16008.16541.2	2	18.19	0	2.44E+06		(K)ICSWNV	(K)I/C|S|W/N/V/D/G L/R(A)	C:Carbamidomethylation	138.07	610.3003	1219.589	0.0045	3.7	5.84	19395.5	5.38	HUMAN	G3V359	DNA-(apurinic or apyrimidinic site) lyase (Fragment)
996	dirMS_041712_NAR_30mM_plus2.21421.21421.2	2	18.19	0	3.50E+05		(K)FDGILGN	(K)F D|G\I|L/G/M A Y P R(I)		167.42	620.3199	1239.619	0.0135	10.9	5.83	32905.8	5.88	HUMAN	C9JH19	Cathepsin D light chain (Fragment)
997	dirMS_041712_NAR_60mM_plus.19593.19593.3	3	18.19	1	6.22E+05		(K)TVTAMD	(K)T/V|T/A/M D V/V/Y A/L K\R(Q)		161.05	489.6071	1466.804	0.0032	2.2	8.25	11367.1	11.36	HUMAN	P62805	Histone H4
998	dirMS_041712_NAR_60mM_plus.9488.9488.3	3	18.19	0	1.08E+06		(R)IQFKPDD	(R)I/Q/F K/P D D/G T T/P/E/R(I)		107.58	501.923	1503.744	0.0106	7.1	4.56	70034.8	6.89	HUMAN	B4DT31	Far upstream element-binding protein 1
999	dirMS_041712_NAR_30mM_minus2.13876.14023.2	2	18.18	0	2.89E+05		(R)IYVVDV(	(R)I/Y|V|V/D|V|G\S E/P R(A)		129.45	617.3267	1233.647	-0.0013	-1	4.37	39066	6.61	HUMAN	A6PVW9	Selenium binding protein 1 (Fragment)
1000	dirMS_041712_NAR_30mM_minus2.19026.19044.2	2	18.18	0	9.29E+05		(K)MSIYQA	(K)M/S|I|Y/Q/A/M W/K(G)		158.9	579.2789	1157.548	0.0023	2	8.34	266161	5.68	HUMAN	E9PPU0	Epiplakin
1001	dirMS_041712_NAR_30mM_plus2.9019.9019.3	3	18.18	0	4.28E+05		(R)SSQSSS(	(R)S S\Q S S/S Q/Q|F/S/G/I G/R(S)		101.03	485.8992	1455.682	0.0008	0.5	9.47	73202	9.19	HUMAN	C9JMU5	Probable ATP-dependent RNA helicase DDX17
1002	dirMS_041712_NAR_50mM_plus2.18409.18752.3	3	18.18	0	3.64E+07		(R)IHFPLAT	(R)I H F\P L A\T|Y|A|P/V/I/S A E K(A)		164.83	586.3252	1756.963	-0.0022	-1.2	6.75	49012.3	4.9	HUMAN	A8MUB1	Tubulin alpha-4A chain
1003	dirMS_041712_NAR_60mM_plus.6540.6570.3	3	18.18	0	3.97E+05		(R)VEAKPE'	(R)V/E|A/K/P E V/Q/S/Q/P P R(V)		92.83	488.9311	1464.781	-0.0018	-1.2	6.11	54177.2	5.41	HUMAN	Q9UN86	Ras GTPase-activating protein-binding protein 2
1004	dirMS_041712_NAR_20mM_minus.12229.12535.2	2	18.17	0	9.01E+05	M185m	(K)LAVNm\	(K)L/A|V|N/m/V|P F/P R(L)	m:Oxidized methionine	171.88	580.3173	1143.634	15.993	-1.6	9.75	42230.8	4.77	HUMAN	A6NKZ8	Putative tubulin beta chain-like protein ENSP00000290377
1005	dirMS_041712_NAR_30mM_minus2.11371.11371.2	2	18.17	0	1.19E+06		(K)FSASGEI	(K)F S|A/S/G E/L/G/N G N I K(L)		114.97	647.3253	1293.643	0	0	6	29110.6	4.57	HUMAN	P12004	Proliferating cell nuclear antigen
1006	dirMS_041712_NAR_50mM_minus2.8453.8453.3	3	18.17	1	2.40E+05		(R)AGTGVD	(R)A G T/G/V/D N V D/L E/A/A/T R K(G)		116.53	539.6119	1616.824	-0.0027	-1.7	4.56	57391.2	6.29	HUMAN	O43175	D-3-phosphoglycerate dehydrogenase
1007	dirMS_041712_NAR_60mM_plus.19594.19594.3	3	18.17	0	1.33E+05		(R)NSVSNF	(R)N S|V|S N/F/L/H/S L E R(G)		161.23	468.2401	1402.707	-0.0016	-1.1	6.75	267260.7	5.95	HUMAN	Q13085	Acetyl-CoA carboxylase 1
1008	dirMS_041712_NAR_50mM_minus2.5320.5320.3	3	18.16	0	8.24E+05		(K)TATPQQ	(K)T A/T/P Q Q/A Q/E/V/H E K(L)		88.02	489.5768	1466.723	-0.0075	-5.1	5.37	31075.7	5.65	HUMAN	P60174	Triosephosphate isomerase
1009	dirMS_041712_NAR_30mM_plus2.18681.18681.2	2	18.15	0	1.67E+05		(K)TVLSNV(	(K)T/V|L|S|N V/Q\E E L D/R(M)		151	701.8633	1402.717	0.0021	1.5	4.14	45232	5.78	HUMAN	B4DZP4	Cytoplasmic dynein 1 light intermediate chain 2
1010	dirMS_041712_NAR_50mM_plus2.14390.14463.3	3	18.15	1	9.68E+05		(R)LAIKLPD	(R)L/A|I/K|L|P D D Q I/P K(T)		144.23	450.9373	1350.799	-0.0018	-1.3	5.96	26413	9.18	HUMAN	B1APP6	6-phosphofructokinase type C (Fragment)
1011	dirMS_041712_NAR_50mM_plus2.16471.16506.3	3	18.14	0	2.19E+05		(K)FTISDHP	(K)F/T|I/S D/H/P Q/P I D/P L L/K(N)		154.8	574.3119	1720.927	-0.0057	-3.3	5.21	138084.9	5.52	HUMAN	Q86VP6	Cullin-associated NEDD8-dissociated protein 1
1012	dirMS_041712_NAR_60mM_plus.14957.15120.3	3	18.14	0	7.90E+05		(R)SCTVNIN	(R)S C|T|V/N I|M/F/G/T/P Q E/H R(N)	C:Carbamidomethylation	135.17	592.9433	1776.816	-0.0003	-0.1	6.47	65763.5	9.81	HUMAN	Q9HCD5	Nuclear receptor coactivator 5
1013	dirMS_041712_NAR_30mM_minus2.15858.15963.2	2	18.13	0	3.84E+05		(R)FGLNVS:	(R)F/G|L|N|V/S/S/I/S/R(K)		139.93	540.2895	1079.584	-0.0127	-11.7	9.75	24161.9	5.27	HUMAN	F8VZQ9	SAP domain-containing ribonucleoprotein
1014	dirMS_041712_NAR_60mM_minus.16753.16951.3	3	18.13	1	3.66E+05		(K)ILLAELE(	(K)I/L|L/A/E L E/Q L/K/G Q/G K(S)		154.27	513.9734	1539.911	-0.0048	-3.1	6.14	49709.6	5.19	HUMAN	B0YJC4	Vimentin
1015	dirMS_041712_NAR_60mM_minus.16880.17038.3	3	18.13	1	2.55E+05		(R)YRVPDV	(R)Y R V P D\V\L\V|A/D/P/P I A/R(L)		154.5	560.9843	1680.943	-0.0048	-2.9	5.96	96499.9	5.43	HUMAN	F5H6X6	Neutral alpha-glucosidase AB
1016	dirMS_041712_NAR_60mM_minus.17122.17337.3	3	18.13	1	7.39E+05		(K)QLDDLK'	(K)Q/L|D/D/L K/V/E/L/S/Q/L/R(V)		156.18	519.6267	1556.864	0.0013	0.8	4.56	10815.7	10.72	HUMAN	F2Z388	60S ribosomal protein L35
1017	dirMS_041712_NAR_20mM_plus.15795.15795.2	2	18.13	0	4.27E+05		(R)GVVDSE	(R)G V|V|D S/E|D|I/P L N/L/S/R(E)		179.57	757.3956	1513.786	-0.0017	-1.1	4.03	74494.3	7.79	HUMAN	F5H897	Heat shock protein 75 kDa, mitochondrial
1018	dirMS_041712_NAR_30mM_plus2.18611.18611.2	2	18.13	0	1.90E+05		(-)SDVLELT	(-)S D V/L/E|L|T|D D N F E/S R(I)		150.83	820.3752	1639.745	-0.0015	-0.9	3.77	13747.3	6.8	HUMAN	H7BZJ3	Thioredoxin (Fragment)
1019	dirMS_041712_NAR_40mM_plus2.14909.14909.3	3	18.13	1	7.65E+05		(R)VCENIPI'	(R)V/C|E/N\I|P I V L C G N K V D|I K(D)	C:Carbamidomethylation	154.7	657.6841	1971.04	-0.0024	-1.2	6.04	14844.8	5.89	HUMAN	B4DV51	GTP-binding nuclear protein Ran
1020	dirMS_041712_NAR_50mM_plus2.21745.21761.3	3	18.13	1	2.38E+05		(K)LGFAGL\	(K)L/G|F/A/G/L/V Q/E I/S F G T T K D K(M)		184.83	637.6781	1911.022	-0.0025	-1.3	6.07	49982	5.86	HUMAN	B4DYD8	T-complex protein 1 subunit epsilon
1021	dirMS_041712_NAR_60mM_plus.16659.16865.3	3	18.13	1	1.13E+06		(R)ELISNAS	(R)E L|I/S/N/A/S/D|A L D/K I R(L)		144.67	515.6144	1544.828	0.0008	0.5	4.56	5582.4	4.81	HUMAN	F8W026	Endoplasmin (Fragment)
1022	dirMS_041712_NAR_30mM_minus2.7454.7510.2	2	18.12	0	4.30E+05		(R)VLADPSI	(R)V/L|A|D|P S D/D/T K(G)		95.23	530.7694	1060.516	0.0158	14.9	3.93	266161	5.68	HUMAN	E9PPU0	Epiplakin
1023	dirMS_041712_NAR_30mM_minus2.9745.9745.2	2	18.12	0	1.91E+05	M129m	(K)ILYSQCG	(K)I L|Y/S/Q/C G D/V/m R(A)	C:Carbamidomethylation m:Oxid	107.67	679.3145	1341.629	15.9928	-1.6	5.83	26991.7	5.01	HUMAN	B7Z6Z4	Myosin light polypeptide 6
1024	dirMS_041712_NAR_20mM_plus.10453.10527.2	2	18.12	0	2.32E+05		(R)AQIFAN'	(R)A Q|I|F|A|N/T V/D/N A R(I)		145.25	660.3343	1319.67	-0.0089	-6.8	5.88	43773.3	5.26	HUMAN	F8VZY9	Keratin, type I cytoskeletal 18
1025	dirMS_041712_NAR_20mM_plus.13661.13850.2	2	18.12	0	2.77E+05		(R)GDLGIEI	(R)G D/L|G/I|E|I|P/A E/K(V)		166.72	571.3075	1141.61	-0.0022	-1.9	4.14	18466.1	5.78	HUMAN	H3BSU3	Pyruvate kinase (Fragment)
1026	dirMS_041712_NAR_30mM_plus2.13439.13584.2	2	18.12	0	1.53E+06		(R)LSESQLS	(R)L/S|E/S/Q/L/S F R(R)		124.13	533.777	1066.553	-0.006	-5.7	6	69661.6	9.75	HUMAN	Q96PK6	RNA-binding protein 14
1027	dirMS_041712_NAR_30mM_plus2.17628.17852.2	2	18.12	0	6.11E+05		(K)NSLESYA	(K)N S|L|E|S|Y/A/F/N M K(A)		146.15	652.303	1303.599	0	0	6	45508.6	5.5	HUMAN	A8K7Q2	Heat shock cognate 71 kDa protein
1028	dirMS_041712_NAR_50mM_plus2.16208.16208.3	3	18.12	0	2.89E+05		(K)ACQSIYF	(K)A C|Q/S I/Y/P L/H/D V F V R(K)	C:Carbamidomethylation	154.4	568.9562	1704.853	0.0014	0.8	6.79	16650	9.47	HUMAN	D6R9B6	40S ribosomal protein S3a
1029	dirMS_041712_NAR_50mM_plus2.7423.7604.3	3	18.12	1	2.39E+06		(K)GAVAED	(K)G A/V|A|E/D/G D E/L R T E/P E/A K(K)		105.58	596.2908	1786.845	0.0125	7	4.08	19284.3	5.23	HUMAN	G3V3C7	DNA-(apurinic or apyrimidinic site) lyase (Fragment)
1030	dirMS_041712_NAR_60mM_plus.11513.11746.3	3	18.12	1	1.90E+06		(R)LMEEIM	(R)L/M|E/E/I/M/S E K/E/N K(T)		118.2	494.2424	1480.702	0.0105	7.1	4.49	61018.6	9.44	HUMAN	B4DLW8	Probable ATP-dependent RNA helicase DDX5
1031	dirMS_041712_NAR_60mM_plus.6514.7111.3	3	18.12	1	3.15E+06		(K)IQDKEGI	(K)I Q|D K E G|I|P/P/D/Q Q R(L)		94	508.5982	1523.781	-0.0012	-0.8	4.56	17271.6	6.75	HUMAN	B4DV12	Ubiquitin
1032	dirMS_041712_NAR_20mM_minus.10702.10888.2	2	18.11	0	4.20E+05		(K)DVNAAI.	(K)D V|N|A/A/I/A A/I/K(T)		155.98	493.287	985.568	-0.0009	-1	5.84	49012.3	4.9	HUMAN	A8MUB1	Tubulin alpha-4A chain
1033	dirMS_041712_NAR_50mM_minus2.9302.9407.3	3	18.11	1	3.41E+05		(K)LLEDKN(	(K)L/L|E/D/K/N/G E|V/Q N L A V K(C)		121.45	557.3071	1669.912	-0.0052	-3.1	4.68	6116.1	5.2	HUMAN	F5H6I6	Cullin-associated NEDD8-dissociated protein 1 (Fragment)
1034	dirMS_041712_NAR_120mM_plus.12294.12719.4	4	18.11	2	9.28E+05		(K)KGTVEG	(K)K\G T V\E|G\F\E|P/A/D/N K/C L L/R(A)	C:Carbamidomethylation	118.9	484.2468	1933.98	-0.0147	-7.6	6.18	13286.2	9.99	HUMAN	H0YLA2	Signal recognition particle 14 kDa protein
1035	dirMS_041712_NAR_60mM_plus.13078.13419.3	3	18.11	1	8.86E+05		(R)VIDRELY	(R)V/I|D/R E\L\Y|Q/Q|L/Q/R(G)		125.78	520.952	1560.849	-0.0078	-5	6.04	533778	5.74	HUMAN	Q15149	Plectin
1036	dirMS_041712_NAR_120mM_minus.14026.14527.4	4	18.1	2	5.90E+06		(K)VLAGDK	(K)V L|A/G/D/K/N/F/I/T A E E L R/R(E)		131.72	458.7498	1832.003	-0.0251	-13.7	6.15	80254.2	5.05	HUMAN	D6PXK4	Alpha actinin 4 short isoform
1037	dirMS_041712_NAR_50mM_minus2.12421.12940.3	3	18.1	1	7.56E+06		(R)IQLVEEE	(R)I/Q|L|V/E E/E/L D/R A Q E/R(L)		140.42	576.6348	1727.892	-0.0024	-1.4	4.25	31923.6	4.85	HUMAN	B7Z596	Tropomyosin alpha-1 chain
1038	dirMS_041712_NAR_40mM_plus2.7770.7770.2	2	18.1	0	5.58E+04		(R)VIGSGCI	(R)V/I|G S/G/C N L/D/S/A R(F)	C:Carbamidomethylation	105.37	624.8021	1248.6	-0.0032	-2.6	5.81	25445.8	5.52	HUMAN	A8MW50	L-lactate dehydrogenase (Fragment)
1039	dirMS_041712_NAR_60mM_plus.21515.21674.3	3	18.1	0	9.30E+06		(R)LHFFMP	(R)L H\F\F M|P G|F|A|P|L/T S R(G)		180.4	540.9492	1620.835	-0.0025	-1.5	9.75	42230.8	4.77	HUMAN	A6NKZ8	Putative tubulin beta chain-like protein ENSP00000290377
1040	dirMS_041712_NAR_30mM_minus2.19454.19730.2	2	18.09	0	2.68E+06		(K)EGLLLW	(K)E G\L|L|L|W/C/Q/R(K)	C:Carbamidomethylation	162.88	587.8058	1174.604	0.0006	0.5	6.1	104488.6	5.36	HUMAN	B2RCS5	Actinin, alpha 2, isoform CRA_b
1041	dirMS_041712_NAR_40mM_plus2.12292.12292.2	2	18.09	0	1.06E+05		(R)VLQALE(	(R)V/L|Q/A/L\E\G L/K(M)		138.8	485.8002	970.593	0	0	5.97	16060.2	10.31	HUMAN	P39019	40S ribosomal protein S19
1042	dirMS_041712_NAR_60mM_minus.11193.11333.3	3	18.08	1	3.02E+06		(R)MQKEIT	(R)M Q/K E I T/A|L|A/P/S T M K(I)		123.32	516.9421	1548.812	-0.0007	-0.4	8.35	32616.5	5.18	HUMAN	A6NL76	Actin, alpha skeletal muscle
1043	dirMS_041712_NAR_30mM_plus2.9689.9854.2	2	18.08	0	7.94E+05		(K)EAESSPF	(K)E\A|E|S/S/P/F/V/E/R(L)		105.18	575.7726	1150.537	0.0004	0.4	4.25	92752.5	4.76	HUMAN	P14625	Endoplasmin
1044	dirMS_041712_NAR_50mM_plus2.18467.18808.3	3	18.08	1	9.58E+06		(R)LFVGNLI	(R)L/F|V\G/N/L/P A D/I/T E D E F K R(L)		166.67	655.3417	1964.012	-0.0018	-0.9	4.32	76262.2	9.53	HUMAN	P23246	Splicing factor, proline- and glutamine-rich
1045	dirMS_041712_NAR_60mM_plus.6762.6788.3	3	18.08	1	7.29E+05		(R)AQLGGF	(R)A Q|L|G/G/P E A|A/K S D E/T A\A/K(-)		93.87	548.6115	1643.824	-0.0036	-2.2	4.68	3888.3	5	HUMAN	C9J3N8	Heat shock protein beta-1
1046	dirMS_041712_NAR_30mM_minus2.14066.14231.2	2	18.07	0	3.27E+05		(K)FGVYDIE	(K)F/G|V|Y/D/I/D|N K(T)		130.5	535.762	1070.515	0.0014	1.3	4.21	27142.4	5.34	HUMAN	E5RG68	Copine-3 (Fragment)
1047	dirMS_041712_NAR_60mM_minus.14940.15054.3	3	18.07	1	3.27E+05	M557m	(K)mFGIDR	(K)m/F|G/I/D/R D A\I/A/Q/A V/R(G)	m:Oxidized methionine	143.58	526.9353	1562.811	15.9806	-9.1	5.71	63448.4	7.29	HUMAN	B4E022	Transketolase
1048	dirMS_041712_NAR_30mM_plus2.11053.11176.2	2	18.07	0	7.00E+05		(K)TVSPALI	(K)T V|S|P/A/L/I/S/R(F)		111.05	472.2823	943.557	0.0002	0.2	9.41	50425	5.53	HUMAN	H0YH27	Cullin-associated NEDD8-dissociated protein 1 (Fragment)
1049	dirMS_041712_NAR_40mM_plus2.17423.17423.3	3	18.07	1	3.09E+05		(K)TGISDVF	(K)T/G\I/S/D\V/F/A K N/D L/A V V D|V R(I)		169.3	640.3451	1919.023	-0.0025	-1.3	4.43	76670.1	4.6	HUMAN	P19338	Nucleolin
1050	dirMS_041712_NAR_50mM_plus2.16121.16162.3	3	18.07	0	1.05E+06		(R)LSFQHD	(R)L/S/F Q H D\P E/T|S/V/L/V/L/R(K)		152.42	580.9801	1740.928	-0.0022	-1.2	5.32	96827.3	5.58	HUMAN	E9PKU7	Neutral alpha-glucosidase AB
1051	dirMS_041712_NAR_60mM_plus.12979.12979.3	3	18.07	1	1.36E+06		(K)NQVAM	(K)N Q|V|A\M|N/P T N T V/F D A K/R(L)		124.48	602.6367	1805.896	-0.0007	-0.4	8.75	20069.4	6.74	HUMAN	E9PK54	Heat shock cognate 71 kDa protein (Fragment)
1052	dirMS_041712_NAR_20mM_minus.12264.12387.2	2	18.06	0	9.88E+05	M313m	(R)TVQSLEI	(R)T V|Q|S/L|E|I D/L/D/S m R(N)	m:Oxidized methionine	171.08	761.8733	1506.747	15.9925	-1.6	4.03	43773.3	5.26	HUMAN	F8VZY9	Keratin, type I cytoskeletal 18
1053	dirMS_041712_NAR_60mM_minus.17179.17179.3	3	18.06	1	3.11E+05		(K)DANAKL	(K)D A/N/A\K L S E|L/E/A|A/L/Q R(A)		156.03	543.6239	1628.86	-0.0031	-1.9	4.68	56607.4	5.37	HUMAN	F8VXB4	Keratin, type II cytoskeletal 8
1054	dirMS_041712_NAR_120mM_plus.12737.13213.3	3	18.06	1	2.42E+06		(R)GYVKEQ	(R)G Y|V/K/E/Q|F/A/W/R(H)		121.9	428.5561	1283.653	0.0006	0.5	8.59	19924.8	9.99	HUMAN	F6U211	40S ribosomal protein S10
1055	dirMS_041712_NAR_20mM_plus.8083.8083.2	2	18.06	0	4.16E+05	M239m	(K)EVDEQn	(K)E V/D E/Q m L/N|V/Q/N/K(N)	m:Oxidized methionine	125.1	731.8435	1446.689	15.9904	-3.1	4.14	40069.4	4.83	HUMAN	E7EWR1	Tubulin beta chain
1056	dirMS_041712_NAR_30mM_plus2.11137.11563.2	2	18.06	0	1.70E+06		(K)LVLVGD(	(K)L/V|L|V|G/D/G G/T/G K(T)		112.93	508.2931	1015.578	0.0007	0.7	5.84	26394.9	6.5	HUMAN	B5MDF5	GTP-binding nuclear protein Ran
1057	dirMS_041712_NAR_30mM_plus2.16940.17116.2	2	18.06	0	5.93E+05	M256m	(R)SLDmDS	(R)S L|D\m/D|S/I/I\A E/V/K(A)	m:Oxidized methionine	142.28	668.8384	1320.671	15.998	2.3	4.03	37296.3	5.3	HUMAN	F8VU64	Keratin, type II cytoskeletal 8
1058	dirMS_041712_NAR_30mM_plus2.9840.9879.2	2	18.06	1	1.01E+05		(K)AIYKGP(	(K)A I|Y/K/G\P G S E|A|G|P(-)		105.18	573.7938	1146.579	0.0014	1.2	6.04	30492.9	5.26	HUMAN	P21964	Catechol O-methyltransferase
1059	dirMS_041712_NAR_50mM_minus2.7332.7393.3	3	18.05	1	1.43E+05		(R)VLVNDA	(R)V/L|V|N/D A Q/K|V/T/E/G Q Q E/R(L)		108.8	605.3171	1813.94	-0.0035	-1.9	4.68	90316.8	5.52	HUMAN	Q13263	Transcription intermediary factor 1-beta
1060	dirMS_041712_NAR_30mM_minus2.12049.12329.2	2	18.04	0	2.33E+05		(R)EYQELM	(R)E/Y/Q/E/L/M/N/V/K(L)		117.8	577.2825	1153.556	0.0019	1.7	4.53	56607.4	5.37	HUMAN	F8VXB4	Keratin, type II cytoskeletal 8
1061	dirMS_041712_NAR_20mM_plus.18093.18093.3	3	18.04	0	1.36E+05		(R)AILVDLE	(R)A I L V D L/E/P/G/T M/D S V R(S)		196.55	539.2833	1615.836	-0.0006	-0.4	4.03	27549.9	4.39	HUMAN	E9PBJ4	Tubulin beta chain
1062	dirMS_041712_NAR_30mM_plus2.18420.18420.2	2	18.04	0	1.65E+05		(K)QFLSQFI	(K)Q F\L|S/Q/F/E/M Q S R(K)		150.25	700.8358	1400.663	0.0016	1.2	6	52440.1	6	HUMAN	F8WJN3	Cleavage and polyadenylation-specificity factor subunit 6
1063	dirMS_041712_NAR_30mM_plus2.7430.7457.2	2	18.04	0	7.06E+05		(K)GPSSVEI	(K)G/P|S|S/V/E/D|I|K(A)		93.43	466.2425	931.473	0.0046	5	4.37	32745.6	4.64	HUMAN	P06748	Nucleophosmin
1064	dirMS_041712_NAR_50mM_plus2.16047.16047.3	3	18.04	1	7.23E+05		(R)EGLTSIE	(R)E G/L/T/S/I|E E/V T K N L Q/K(F)		152.15	563.6357	1688.907	-0.014	-8.3	4.79	533778	5.74	HUMAN	Q15149	Plectin
1065	dirMS_041712_NAR_50mM_plus2.9540.9540.3	3	18.04	1	1.58E+05		(R)LGGSPF(	(R)L/G/G S|P/F G|P A G/T G K T E S V K(A)		117.22	563.9657	1689.881	0.0019	1.1	8.59	535136.6	6.01	HUMAN	Q14204	Cytoplasmic dynein 1 heavy chain 1
1066	dirMS_041712_NAR_30mM_minus2.11101.11152.2	2	18.03	0	6.60E+05		(K)VAGQD(	(K)V/A|G|Q|D|G S V V/Q|F/K(I)		114.03	617.824	1234.643	-0.0019	-1.5	5.81	17127.4	10.05	HUMAN	A8MU27	Small ubiquitin-related modifier 3
1067	dirMS_041712_NAR_30mM_plus2.10629.10630.2	2	18.03	0	2.32E+05		(K)VLEEAN(	(K)V/L|E/E|A|N Q|A|I|N|P K(L)		109.18	663.3575	1325.706	0.0018	1.3	4.53	73202	9.19	HUMAN	C9JMU5	Probable ATP-dependent RNA helicase DDX17
1068	dirMS_041712_NAR_50mM_plus2.14284.14401.3	3	18.03	0	1.56E+06		(K)SLTNDW	(K)S L|T/N/D/W/E/D H L A V/K(H)		143.63	509.9193	1527.744	-0.0005	-0.3	4.53	85057.6	4.94	HUMAN	P07900	Heat shock protein HSP 90-alpha
1069	dirMS_041712_NAR_50mM_plus2.20512.20532.3	3	18.03	1	1.87E+05		(K)SYLNMD	(K)S Y|L/N/M/D/A I/M/E/A/I/K K(T)		176.7	542.9473	1626.823	0.0044	2.7	5.79	80685.1	7.31	HUMAN	P05165	Propionyl-CoA carboxylase alpha chain, mitochondrial
1070	dirMS_041712_NAR_20mM_minus.9640.9793.2	2	18.02	0	3.36E+05		(K)IGGIGTV	(K)I/G|G|I|G/T/V/P V G R(V)		147.47	513.3075	1025.61	-0.0025	-2.4	9.75	50482.3	9.31	HUMAN	P68104	Elongation factor 1-alpha 1
1071	dirMS_041712_NAR_30mM_minus2.9205.9205.2	2	18.02	0	2.85E+06		(R)VIGSGCI	(R)V/I|G\S/G/C/N L/D/S/A/R(F)	C:Carbamidomethylation	104.52	624.8035	1248.6	-0.0004	-0.3	5.81	25445.8	5.52	HUMAN	A8MW50	L-lactate dehydrogenase (Fragment)
1072	dirMS_041712_NAR_120mM_plus.20101.20528.3	3	18.02	1	3.71E+06		(K)AAIDWF	(K)A/A|I|D\W/F/D/G K E/F H G N\I I/K(V)		158.78	654.3334	1960.992	-0.0059	-3	5.38	49181	4.93	HUMAN	K7EPT6	TATA-binding protein-associated factor 2N
1073	dirMS_041712_NAR_60mM_plus.7790.7915.3	3	18.02	1	1.79E+06		(R)YLAEVA'	(R)Y/L|A/E/V/A/T/G E/K R(A)		99.27	412.8904	1236.658	-0.0016	-1.3	6.14	28473.3	4.8	HUMAN	P61981	14-3-3 protein gamma
1074	dirMS_041712_NAR_30mM_minus2.10934.11153.2	2	18.01	0	6.66E+05		(K)VAGQD(	(K)V A|G Q|D/G/S/V/V/Q/F/K(I)		114.02	617.824	1234.643	-0.0019	-1.5	5.81	17127.4	10.05	HUMAN	A8MU27	Small ubiquitin-related modifier 3
1075	dirMS_041712_NAR_40mM_plus2.9046.9046.2	2	18.01	0	8.93E+04		(K)GSDFDC	(K)G S/D/F/D|C E/L/R(L)	C:Carbamidomethylation	115.47	549.7296	1098.452	-0.0001	-0.1	4.03	51260.6	5.39	HUMAN	P61978	Heterogeneous nuclear ribonucleoprotein K
1076	dirMS_041712_NAR_60mM_plus.16822.16822.3	3	18.01	0	1.02E+06		(K)TTISVAH	(K)T T/I/S/V/A H/L/L A A/R(Q)		144.5	418.2478	1252.737	-0.0083	-6.7	9.44	34143.2	10.07	HUMAN	B4DT23	Protein FAM98A
1077	dirMS_041712_NAR_120mM_plus.7136.7265.3	3	18	1	2.69E+06		(K)SLDEME	(K)S/L|D/E/M/E/K/Q Q/R(E)		91.23	421.8744	1263.6	0.0089	7	4.68	26419.2	5.1	HUMAN	H0Y9K7	Splicing factor, proline- and glutamine-rich (Fragment)
1078	dirMS_041712_NAR_120mM_minus.14499.14545.4	4	17.99	1	5.55E+05		(R)ETNLDSI	(R)E T N L D S\L/P/L/V/D/T/H S K/R(T)		133.47	456.992	1824.945	0.0012	0.6	5.38	53707.8	5.06	HUMAN	P08670	Vimentin
1079	dirMS_041712_NAR_120mM_minus.6640.6647.3	3	17.99	1	2.91E+05		(K)DSYVGD	(K)D S|Y/V/G/D/E A Q/S K R(G)		90.67	452.2134	1354.623	0.0023	1.7	4.56	32616.5	5.18	HUMAN	A6NL76	Actin, alpha skeletal muscle
1080	dirMS_041712_NAR_30mM_minus2.18231.18553.2	2	17.99	0	4.47E+05		(R)QAQEYE	(R)Q/A/Q/E|Y/E/A/L/L N/I/K(V)		154.67	710.3759	1419.748	-0.0033	-2.3	4.53	43773.3	5.26	HUMAN	F8VZY9	Keratin, type I cytoskeletal 18
1081	dirMS_041712_NAR_20mM_plus.17980.17980.2	2	17.99	0	1.81E+05		(R)SLVPAAI	(R)S L|V|P A A/E/L/L E/S/R(V)		195.08	642.8613	1284.716	-0.0005	-0.4	4.53	533778	5.74	HUMAN	Q15149	Plectin
1082	dirMS_041712_NAR_30mM_plus2.8663.8728.2	2	17.99	0	3.15E+05		(K)TAEFQV.	(K)T|A|E|F/Q/V/A/R(T)		99.3	461.2413	921.479	-0.0035	-3.8	5.66	55373.1	7.25	HUMAN	F5H6Y1	Phenylalanine--tRNA ligase beta subunit
1083	dirMS_041712_NAR_50mM_plus2.20960.21310.3	3	17.99	0	8.90E+06		(R)LHFFMP	(R)L H F\F\M|P/G\F|A|P|L/T S/R(G)		181.53	540.9498	1620.835	-0.0007	-0.4	9.75	42230.8	4.77	HUMAN	A6NKZ8	Putative tubulin beta chain-like protein ENSP00000290377
1084	dirMS_041712_NAR_30mM_minus2.6484.6730.2	2	17.98	0	4.22E+05		(K)VLEEEEC	(K)V/L|E|E/E/E/Q/R(R)		90.3	516.2552	1031.5	0.0027	2.7	4.09	54214.9	5.66	HUMAN	C9J813	Caldesmon (Fragment)
1085	dirMS_041712_NAR_20mM_plus.19047.19047.2	2	17.98	0	1.91E+05		(R)STGEAF\	(R)S T G E A F/V/Q|F/A|S Q/E I/A/E K(A)		202.2	921.4477	1841.892	-0.0035	-1.9	4.25	24249.1	6.99	HUMAN	D6RBM0	Heterogeneous nuclear ribonucleoprotein H (Fragment)

1086	dirMS_041712_NAR_20mM_plus.8729.8729.2	2	17.98	0	2.45E+05		(R)VPPPPPI	(R)V/P|P/P/P P I A R(A)		130.62	472.2851	943.572	-0.0094	-10	9.72	32085.2	8.93	HUMAN	B4DY08	Heterogeneous nuclear ribonucleoproteins C1/C2
1087	dirMS_041712_NAR_60mM_plus.11254.11407.3	3	17.98	0	4.60E+06		(R)GSAVHC	(R)G S|A/V/H/Q/L/S/E/E/L R(C)		116.45	442.5666	1325.681	0.0044	3.4	5.4	558007.1	5.44	HUMAN	P58107	Epiplakin
1088	dirMS_041712_NAR_30mM_plus2.8424.8424.2	2	17.97	0	5.88E+05		(R)LGLGEG.	(R)L/G|L|G/E/G A E E K(S)		98.35	501.7639	1002.51	0.0103	10.3	4.25	40096.8	9.24	HUMAN	C9JW69	Regulator of chromosome condensation (Fragment)
1089	dirMS_041712_NAR_60mM_plus.10456.10456.2	2	17.97	1	4.41E+06		(R)LQAEIEC	(R)L/Q|A E/I/E/G L K G Q/R(A)		112.33	671.3774	1341.748	-0.001	-0.7	6.14	56607.4	5.37	HUMAN	F8VXB4	Keratin, type II cytoskeletal 8
1090	dirMS_041712_NAR_30mM_minus2.14650.14650.2	2	17.96	0	1.01E+05		(R)LDIDSPP	(R)L/D|I/D/S/P/P/I T A R(N)		133.55	599.3249	1197.647	-0.0049	-4.1	4.21	5755.5	5.75	HUMAN	H3BN34	Pyruvate kinase isozymes M1/M2 (Fragment)
1091	dirMS_041712_NAR_50mM_minus2.10567.10567.3	3	17.96	0	1.60E+05		(R)LLELGPK	(R)L L|E|L/G P/K/P E V|A\Q Q T R(K)		128.52	560.3182	1678.949	-0.0086	-5.1	6.14	139883.7	8.06	HUMAN	P53621	Coatomer subunit alpha
1092	dirMS_041712_NAR_50mM_minus2.5833.5833.3	3	17.96	0	1.31E+05		(R)AGAAGC	(R)A/G/A|A/G/G/P E/E E A E K P/V K(T)		95.32	513.9233	1539.765	-0.0096	-6.2	4.49	18924.5	6.86	HUMAN	Q8WW12	PEST proteolytic signal-containing nuclear protein
1093	dirMS_041712_NAR_50mM_plus2.13295.13558.3	3	17.95	0	1.58E+07		(R)LIDLHSP	(R)L/I|D/L H/S/P S E I/V/K(Q)		138.68	450.9258	1350.763	0.0001	0.1	5.32	7304.3	9.39	HUMAN	G3XAN0	40S ribosomal protein S20
1094	dirMS_041712_NAR_50mM_plus2.16567.16567.3	3	17.95	1	6.78E+06		(K)QLDDLK'	(K)Q\L/D\D/L/K|V/E L/S\Q/L R(V)		155.75	519.6261	1556.864	-0.0005	-0.3	4.56	10815.7	10.72	HUMAN	F2Z388	60S ribosomal protein L35
1095	dirMS_041712_NAR_50mM_plus2.19242.19419.3	3	17.95	0	8.48E+05		(R)DFNHIN'	(R)D F/N H\I\N|V|E|L/S L/L/G K(K)		170.1	533.6223	1598.854	-0.0013	-0.8	5.32	20931.1	10.28	HUMAN	D6RAN4	60S ribosomal protein L9 (Fragment)
1096	dirMS_041712_NAR_60mM_plus.14912.15009.3	3	17.95	1	1.52E+06		(R)VFDKDG	(R)V/F/D K D G N G\Y|I/S/A/A/E/L R(H)		134.8	585.6281	1754.871	-0.001	-0.6	4.56	21860.1	4.45	HUMAN	E7EMB3	Calmodulin
1097	dirMS_041712_NAR_60mM_plus.15519.15537.3	3	17.95	1	8.88E+05		(R)LSAEERI	(R)L/S|A/E/E R D Q|L|L|P N L R(A)		137.98	551.9672	1653.892	-0.0048	-2.9	4.68	12056.4	6.28	HUMAN	P61457	Pterin-4-alpha-carbinolamine dehydratase
1098	dirMS_041712_NAR_50mM_minus2.11744.12118.3	3	17.94	1	6.40E+06		(K)KVPQVS	(K)K\V\P\Q\V S T|P/T|L|V/E V S R(N)		136.33	547.3172	1639.938	-0.0007	-0.4	8.75	57751.3	6.9	HUMAN	B7WNR0	Serum albumin
1099	dirMS_041712_NAR_50mM_minus2.14571.14571.3	3	17.94	1	1.93E+05		(R)AEGAIA(	(R)A/E|G/A/I/A G F R/D/P F/S/G/K(Q)		151.2	508.2599	1522.765	0.0003	0.2	6.12	266161	5.68	HUMAN	E9PPU0	Epiplakin
1100	dirMS_041712_NAR_30mM_plus2.19013.19013.2	2	17.94	0	3.45E+05		(R)LTAEDLF	(R)L/T|A/E\D L/F/E/A R(I)		153.22	582.796	1164.59	-0.0048	-4.1	4.14	533778	5.74	HUMAN	Q15149	Plectin
1101	dirMS_041712_NAR_50mM_plus2.13658.13837.3	3	17.94	0	1.76E+06		(K)DLAGCII	(K)D L|A/G C I/H/G L S N V/K(L)	C:Carbamidomethylation	140.22	461.9061	1383.705	-0.0012	-0.8	6.74	45578.2	7.83	HUMAN	B4DFL2	Isocitrate dehydrogenase [NADP]
1102	dirMS_041712_NAR_50mM_plus2.16408.16548.3	3	17.94	1	7.41E+05		(R)TIAQDY(	(R)T I|A|Q/D/Y G V/L K/A/D/E G I S F R(G)		154.53	661.6783	1983.018	0.0022	1.1	4.56	22338.2	8.65	HUMAN	Q06830	Peroxiredoxin-1
1103	dirMS_041712_NAR_50mM_plus2.9952.10153.3	3	17.94	1	3.10E+06		(R)VLTVINC	(R)V/L|T|V/I/N Q/T Q K/E N L R(K)		119.9	552.6527	1655.944	-0.0003	-0.2	8.72	10815.7	10.72	HUMAN	F2Z388	60S ribosomal protein L35
1104	dirMS_041712_NAR_60mM_plus.13236.13719.3	3	17.94	1	9.94E+05		(R)INVYYNI	(R)I/N|V|Y/Y|N E A/T G G K/Y V/P R(A)		128.25	615.3146	1843.934	-0.0045	-2.4	8.43	50286.6	4.79	HUMAN	P68371	Tubulin beta-4B chain
1105	dirMS_041712_NAR_30mM_minus2.12076.12602.2	2	17.93	0	4.46E+06		(R)EYQELM	(R)E Y/Q/E/L/M/N/V/K(L)		122.25	577.2825	1153.556	0.0019	1.7	4.53	56607.4	5.37	HUMAN	F8VXB4	Keratin, type II cytoskeletal 8
1106	dirMS_041712_NAR_30mM_plus2.13931.14014.2	2	17.93	0	3.99E+05		(K)IGPLGLS	(K)I/G|P|L/G/L/S/P K(K)		126.17	441.2762	881.545	-0.0004	-0.4	8.75	17989.4	9.77	HUMAN	P30050	60S ribosomal protein L12
1107	dirMS_041712_NAR_30mM_plus2.8641.8641.2	2	17.93	1	2.10E+05		(R)GVEEEEI	(R)G V|E/E|E/E|E/D/G E/M/R\E(-)		98.92	769.301	1537.596	-0.0011	-0.7	3.88	9782.1	4.7	HUMAN	P62306	Small nuclear ribonucleoprotein F
1108	dirMS_041712_NAR_40mM_plus2.19232.19375.3	3	17.93	1	4.28E+05		(K)LLPLVSD	(K)L L|P/L/V/S/D/E V F I R D/V/A/K(V)		180.72	605.3503	1814.042	-0.0059	-3.2	4.56	97378.8	6.4	HUMAN	P11216	Glycogen phosphorylase, brain form
1109	dirMS_041712_NAR_50mM_plus2.5727.5727.3	3	17.93	0	1.00E+05		(K)ATCAPQ	(K)A T/C|A/P Q/H G A/P G/P G/P\A D A S K(V)	C:Carbamidomethylation	92.7	597.2829	1789.829	0.0055	3.1	6.79	83264.1	6.04	HUMAN	E9PHF0	Filamin-A
1110	dirMS_041712_NAR_50mM_plus2.9828.9865.3	3	17.93	1	2.54E+05		(K)TTDMAF	(K)T/T|D|M/A/P S K/E/T E/M A L A K(D)		119.22	575.2802	1723.824	0.0019	1.1	4.68	85679.3	7.65	HUMAN	B9ZVR1	Microtubule-associated protein
1111	dirMS_041712_NAR_50mM_plus2.9896.9896.3	3	17.93	1	1.41E+05		(K)TCVFEKE	(K)T C|V/F E K E/N D|P/S V M R(S)	C:Carbamidomethylation	119.48	571.2629	1711.778	-0.0037	-2.1	4.68	267260.7	5.95	HUMAN	Q13085	Acetyl-CoA carboxylase 1
1112	dirMS_041712_NAR_60mM_plus.9474.9495.3	3	17.93	0	4.79E+05		(K)ATAGDT	(K)A/T|A/G D/T/H/L/G/G E D F D/N R(L)		107.57	559.2488	1675.731	0.0012	0.7	4.22	58502.6	6.09	HUMAN	B4DXY3	Heat shock 70 kDa protein 1-like
1113	dirMS_041712_NAR_50mM_minus2.12759.12759.3	3	17.92	1	3.86E+05		(K)NTVELL\	(K)N T|V/E/L/L/V/E D K G N Q V Y R(C)		140.47	626.3309	1876.976	0.0018	1	4.68	258215.9	5.48	HUMAN	E7EN95	Filamin-B
1114	dirMS_041712_NAR_20mM_plus.16786.16786.2	2	17.92	0	3.08E+05		(K)VQIAVA	(K)V Q|I A|V/A/N/A Q E L/L/Q R(M)		187.02	776.9419	1552.881	-0.004	-2.6	5.97	10728.1	9.15	HUMAN	Q9Y5L4	Mitochondrial import inner membrane translocase subunit Tim13
1115	dirMS_041712_NAR_30mM_plus2.13387.13389.2	2	17.92	0	2.65E+05		(K)LSELEPG	(K)L/S\E|L|E|P G T\G D/L R(F)		123.58	643.8321	1286.659	-0.0017	-1.4	4.14	266161	5.68	HUMAN	E9PPU0	Epiplakin
1116	dirMS_041712_NAR_60mM_plus.10085.10360.3	3	17.92	1	1.48E+06		(K)IFRDGEE	(K)I/F R D G E\E\A|G|A|Y/D/G P R(T)		111.47	551.593	1652.766	-0.0019	-1.1	4.32	55361.8	6.42	HUMAN	G5EA52	Protein disulfide isomerase family A, member 3, isoform CRA_b
1117	dirMS_041712_NAR_40mM_minus2.15008.15222.3	3	17.91	0	2.68E+06		(K)ALESPEF	(K)A/L|E|S|P/E/R P/F L/A I/L/G G A/K(V)		174.77	590.3353	1768.996	-0.0042	-2.4	6.19	41827.5	7	HUMAN	B7Z7A9	Phosphoglycerate kinase
1118	dirMS_041712_NAR_60mM_plus.15545.15545.3	3	17.91	1	4.40E+05		(R)IQLVEEE	(R)I/Q|L/V/E E E/L D/R A Q E/R(L)		137.98	576.6345	1727.892	-0.0033	-1.9	4.25	31923.6	4.85	HUMAN	B7Z596	Tropomyosin alpha-1 chain
1119	dirMS_041712_NAR_120mM_plus.18196.18196.4	4	17.9	1	6.50E+05		(R)ILEFFGLI	(R)I/L/E/F/F/G L K/K E E C/P A V R(L)	C:Carbamidomethylation	148.63	484.7638	1936.036	-0.0027	-1.4	6.23	51524.1	4.82	HUMAN	F5H8J2	Protein disulfide-isomerase
1120	dirMS_041712_NAR_30mM_plus2.12180.12240.2	2	17.9	0	9.10E+05		(K)EVDEQM	(K)E V|D/E/Q\M\L|N/V|Q/N/K(N)		117.03	723.8478	1446.689	-0.001	-0.7	4.14	40069.4	4.83	HUMAN	E7EWR1	Tubulin beta chain
1121	dirMS_041712_NAR_40mM_plus2.19956.19979.3	3	17.9	1	1.55E+06		(R)ELAILLG	(R)E L/A|I/L/L/G/M L/D/P A E/K D/E K(G)		189.1	629.003	1884.999	-0.0042	-2.3	4.18	25148.6	6	HUMAN	P30041	Peroxiredoxin-6
1122	dirMS_041712_NAR_60mM_plus.18267.18392.3	3	17.9	1	9.23E+05		(K)EVNKFQ	(K)E V/N K F Q|M/A|Y/S N/L/L R(A)		152.83	571.6293	1712.879	-0.0055	-3.2	8.69	14683.8	10.19	HUMAN	P37108	Signal recognition particle 14 kDa protein
1123	dirMS_041712_NAR_120mM_minus.6262.6459.3	3	17.89	1	1.05E+06		(R)TEERAEI	(R)T/E|E/R A E|L|A/E/S/R(C)		89	430.8806	1290.628	-0.0012	-0.9	4.49	33450.2	4.73	HUMAN	J3KN67	Tropomyosin alpha-3 chain
1124	dirMS_041712_NAR_30mM_plus2.20391.20546.2	2	17.89	0	3.12E+05		(R)FEELNAI	(R)F/E|E|L|N A/D/L/F/R(G)		161.05	627.3106	1253.616	-0.0022	-1.7	4.14	45508.6	5.5	HUMAN	A8K7Q2	Heat shock cognate 71 kDa protein
1125	dirMS_041712_NAR_50mM_plus2.17344.17536.3	3	17.89	1	7.36E+05		(K)SELVAM	(K)S E|L|V/A/M/L/E/E E E L R K(A)		159.72	559.2899	1675.857	-0.002	-1.2	4.32	18679.2	5.61	HUMAN	B4DWW1	ADP-ribosylation factor-like protein 1
1126	dirMS_041712_NAR_60mM_plus.10416.10691.3	3	17.89	1	1.83E+06		(R)YLAEFAT	(R)Y/L|A/E/F/A/T/G N D R K(E)		112.82	462.2281	1384.686	-0.0158	-11.4	6.07	12926.1	4.4	HUMAN	K7EM20	14-3-3 protein epsilon (Fragment)
1127	dirMS_041712_NAR_40mM_minus2.11403.11497.3	3	17.88	0	8.05E+05		(R)TIQVEN!	(R)T I|Q|V/E N\S H L|I|L/T/G A G A L N K(V)		151	660.3663	1979.092	-0.0077	-3.9	6.41	267260.7	5.95	HUMAN	Q13085	Acetyl-CoA carboxylase 1
1128	dirMS_041712_NAR_50mM_minus2.13809.13885.3	3	17.88	0	1.67E+06		(R)QVSASE	(R)Q V|S A/S E L H/T/S/G I/L/G/P E/T L R(D)		147.53	665.688	1995.051	-0.0011	-0.6	5.4	558007.1	5.44	HUMAN	P58107	Epiplakin
1129	dirMS_041712_NAR_120mM_plus.8746.8858.3	3	17.88	0	5.55E+05		(R)FACHSA:	(R)F/A/C/H/S A/S/L/T\V/R(N)	C:Carbamidomethylation	99.62	416.8772	1248.615	0.0017	1.3	9.75	23729.5	9.89	HUMAN	C9IZL7	Non-POU domain-containing octamer-binding protein (Fragment)
1130	dirMS_041712_NAR_50mM_plus2.14467.14570.3	3	17.88	0	1.06E+06		(R)ETHSAL(	(R)E T/H S A L|G|P/A|L/Q/A/A/F K(L)		144.3	514.2745	1540.812	-0.0029	-1.9	6.85	141616.6	6.1	HUMAN	B7ZKM8	Protein transport protein Sec24B
1131	dirMS_041712_NAR_60mM_plus.17342.17361.3	3	17.88	1	1.26E+06		(R)FLQDYFI	(R)F/L|Q|D/Y/F/D/G N L K/R(Y)		148.32	505.9247	1515.759	0.0005	0.3	5.96	55361.8	6.42	HUMAN	G5EA52	Protein disulfide isomerase family A, member 3, isoform CRA_b
1132	dirMS_041712_NAR_60mM_plus.17392.17559.3	3	17.88	1	4.10E+05		(R)ISGLIYEE	(R)I/S|G|L|I/Y/E E T R G V/L/K(V)		148.38	526.634	1577.89	-0.0023	-1.4	6.14	11367.1	11.36	HUMAN	P62805	Histone H4
1133	dirMS_041712_NAR_30mM_minus2.19560.19560.2	2	17.87	0	4.67E+05		(R)ASLSLW	(R)A S/L/S/L|W Q/A/M/E K(G)		162.73	632.3234	1263.64	-0.0007	-0.5	6.05	266161	5.68	HUMAN	E9PPU0	Epiplakin
1134	dirMS_041712_NAR_50mM_minus2.15109.15109.3	3	17.87	1	2.85E+05		(K)VVEEAP!	(K)V V|E/E/A/P S I F L D/A/E T R R(A)		153.48	611.3243	1831.955	0.0035	1.9	4.41	80685.1	7.31	HUMAN	P05165	Propionyl-CoA carboxylase alpha chain, mitochondrial
1135	dirMS_041712_NAR_30mM_minus2.7367.7367.2	2	17.86	0	1.25E+05		(K)VQYPQS	(K)V Q|Y|P/Q S Q A/C K(M)	C:Carbamidomethylation	94.43	604.7902	1208.573	0.0003	0.2	8.56	535136.6	6.01	HUMAN	Q14204	Cytoplasmic dynein 1 heavy chain 1
1136	dirMS_041712_NAR_50mM_minus2.16990.17053.3	3	17.86	1	3.18E+05		(K)AILILDNI	(K)A I|L|I/L/D N D/G D/R L F/A K(Y)		165.27	558.6442	1673.922	-0.004	-2.4	4.43	18430.9	4.79	HUMAN	F8VS17	Coatomer subunit zeta-1
1137	dirMS_041712_NAR_20mM_plus.10967.11358.2	2	17.86	0	1.43E+06		(R)IVLQIDN	(R)I/V|L|Q|I|D/N A R(L)		149.23	521.3056	1041.605	-0.0012	-1.1	5.84	20842.9	5.32	HUMAN	C9JM50	Keratin, type I cytoskeletal 19 (Fragment)
1138	dirMS_041712_NAR_40mM_plus2.19948.19948.3	3	17.86	0	4.60E+05	M166m	(K)VLDFEHI	(K)V/L|D/F E H F\L|P m L Q T V A/K(N)	m:Oxidized methionine	187.63	635.3379	1888.004	15.9954	0.3	5.32	26991.7	5.01	HUMAN	B7Z6Z4	Myosin light polypeptide 6
1139	dirMS_041712_NAR_50mM_plus2.18797.18839.3	3	17.86	0	4.20E+05		(R)AGLGSG	(R)A G/L|G|S/G/L/S\L/S/G L V/H P/E\L S R(S)		167.15	617.3412	1850.013	-0.004	-2.2	6.79	73325.2	9.19	HUMAN	F5H2A9	Very long-chain-specific acyl-CoA dehydrogenase, mitochondrial
1140	dirMS_041712_NAR_120mM_minus.10480.10950.3	3	17.85	1	1.50E+07		(R)VKYETEL	(R)V/K Y E/T|E|L/A/M/R(Q)		113.32	413.885	1239.64	0.0003	0.2	6.11	43773.3	5.26	HUMAN	F8VZY9	Keratin, type I cytoskeletal 18
1141	dirMS_041712_NAR_60mM_minus.11900.12067.3	3	17.85	1	3.03E+06	M72m	(R)TEmENE	(R)T/E|m/E N\E|F/V L I/K K(D)	m:Oxidized methionine	126.87	499.5941	1480.772	15.9962	0.8	4.78	13588.1	4.66	HUMAN	F8VP67	Keratin, type II cytoskeletal 8 (Fragment)
1142	dirMS_041712_NAR_50mM_plus2.18274.18312.3	3	17.85	1	5.52E+05		(K)VIAATNI	(K)V/I|A/A/T N R\V D|I|L/D/P A L L R(S)		164.48	617.3645	1850.086	-0.0069	-3.7	5.93	32827.5	5.92	HUMAN	E9PKD5	26S protease regulatory subunit 6A (Fragment)
1143	dirMS_041712_NAR_120mM_minus.7696.7842.3	3	17.84	1	2.74E+06	M281m	(R)SRAEAE!	(R)S R\A E\A\E|S/m/Y/Q/I/K(Y)	m:Oxidized methionine	96.75	476.8951	1412.684	15.9869	-5.6	5.86	37296.3	5.3	HUMAN	F8VU64	Keratin, type II cytoskeletal 8
1144	dirMS_041712_NAR_30mM_minus2.11695.11753.2	2	17.84	0	9.21E+05		(K)EVDEQM	(K)E V|D/E|Q|M\L/N/V|Q/N K(N)		117.53	723.8476	1446.689	-0.0014	-1	4.14	40069.4	4.83	HUMAN	E7EWR1	Tubulin beta chain
1145	dirMS_041712_NAR_40mM_minus2.9627.9627.2	2	17.84	0	1.24E+06		(R)VAPEEH	(R)V A/P E/E/H/P V\L L/T E A/P L N|P K(A)		139.8	977.5343	1954.064	-0.0031	-1.6	4.75	18032.2	5.25	HUMAN	E7EVS6	Actin, cytoplasmic 1 (Fragment)
1146	dirMS_041712_NAR_50mM_minus2.16320.16342.3	3	17.84	1	1.47E+05		(R)LGLSTLC	(R)L/G|L|S|T/L/G E/L/K/Q N L S R(S)		160.87	543.6477	1628.933	-0.0044	-2.7	8.75	22294.4	5.26	HUMAN	O43399	Tumor protein D54
1147	dirMS_041712_NAR_30mM_plus2.6065.6065.2	2	17.84	0	8.14E+05		(R)NQGGY(	(R)N/Q|G G\Y|G/G|S S/S S/S S/Y/G S G R(R)		86.18	847.8524	1694.7	-0.0026	-1.5	8.59	33268.7	9.31	HUMAN	F8VRQ1	Heterogeneous nuclear ribonucleoprotein A1
1148	dirMS_041712_NAR_20mM_plus.17556.17556.2	2	17.83	0	1.61E+05	M74m	(R)mIAGQ\	(R)m I|A\G/Q|V/L D/I|N L/A/A E P K(V)	m:Oxidized methionine	192.85	849.9568	1682.915	15.9918	-1.9	4.37	15791.7	9.22	HUMAN	B4DSU6	Heterogeneous nuclear ribonucleoproteins C1/C2
1149	dirMS_041712_NAR_30mM_plus2.14063.14239.2	2	17.83	0	9.48E+05	M320m	(K)YmACCL	(K)Y/m|A|C/C/L/L|Y/R(G)	C:Carbamidomethylation m:Oxid	127.2	633.2777	1249.553	15.9955	0.5	8.59	50706.6	5.68	HUMAN	A6NHL2	Tubulin alpha chain-like 3
1150	dirMS_041712_NAR_40mM_plus2.9028.9047.2	2	17.83	0	8.93E+04		(K)GSDFDC	(K)G/S|D|F/D/C/E/L/R(L)	C:Carbamidomethylation	115.47	549.7296	1098.452	-0.0001	-0.1	4.03	51260.6	5.39	HUMAN	P61978	Heterogeneous nuclear ribonucleoprotein K
1151	dirMS_041712_NAR_50mM_plus2.6707.6707.3	3	17.83	0	4.53E+04		(K)ITPAHD(	(K)I/T|P/A/H D/Q N/D Y/E V/G Q R(H)		100.07	581.6074	1742.809	-0.0016	-0.9	4.54	141718.6	7.8	HUMAN	B0V043	Valine--tRNA ligase
1152	dirMS_041712_NAR_30mM_minus2.8502.8585.2	2	17.82	0	4.89E+05	M281m	(R)AEAESm	(R)A/E|A|E/S/m/Y Q/I/K(Y)	m:Oxidized methionine	101.12	593.2765	1169.551	15.995	0.1	4.53	37296.3	5.3	HUMAN	F8VU64	Keratin, type II cytoskeletal 8
1153	dirMS_041712_NAR_50mM_minus2.12670.12670.3	3	17.82	1	3.98E+05		(K)NTVELL\	(K)N T/V/E/L L|V/E D K G N Q V Y R(C)		140.43	626.3309	1876.976	0.0018	1	4.68	258215.9	5.48	HUMAN	E7EN95	Filamin-B
1154	dirMS_041712_NAR_50mM_minus2.7045.7045.3	3	17.82	1	2.04E+05		(K)GAVAED	(K)G A|V/A|E/D G D E L R T E P E/A K(K)		106.92	596.2904	1786.845	0.0113	6.3	4.08	19284.3	5.23	HUMAN	G3V3C7	DNA-(apurinic or apyrimidinic site) lyase (Fragment)
1155	dirMS_041712_NAR_40mM_plus2.16109.16109.3	3	17.82	0	4.11E+04		(K)VLISSLQ	(K)V L|I/S/S/L/Q D D C L/H G I E/S/K(S)	C:Carbamidomethylation	161.47	600.3172	1798.937	0.0003	0.2	5.32	52440.1	6	HUMAN	F8WJN3	Cleavage and polyadenylation-specificity factor subunit 6
1156	dirMS_041712_NAR_40mM_plus2.8978.8978.2	2	17.82	0	1.19E+05		(R)GYSFTTT	(R)G/Y|S\F/T/T/T/T A E/R(E)		114.9	566.7672	1132.527	0.0002	0.2	6	23901.9	5.28	HUMAN	I3L1U9	Actin, cytoplasmic 2, N-terminally processed (Fragment)
1157	dirMS_041712_NAR_50mM_plus2.13705.14147.3	3	17.82	1	1.07E+07		(R)TEMENE	(R)T|E|M/E/N E|F/V/L I/K K(D)		141.2	494.262	1480.772	-0.0001	-0.1	4.78	13588.1	4.66	HUMAN	F8VP67	Keratin, type II cytoskeletal 8 (Fragment)
1158	dirMS_041712_NAR_120mM_minus.10683.11147.3	3	17.81	0	9.56E+05		(R)GFCLPPI	(R)G F|C/L/P/P/H/C S/R(G)	C:Carbamidomethylation	113.55	410.858	1230.551	0.0088	7.1	9.75	14391.8	4.99	HUMAN	G3V461	Creatine kinase B-type (Fragment)
1159	dirMS_041712_NAR_120mM_minus.8138.8283.4	4	17.81	2	2.31E+06		(K)KGAVAE	(K)K\G A\V\A\E/D/G/D/E L R T/E/P E/A/K(K)		99.28	479.4901	1914.94	-0.0018	-0.9	4.36	19284.3	5.23	HUMAN	G3V3C7	DNA-(apurinic or apyrimidinic site) lyase (Fragment)
1160	dirMS_041712_NAR_30mM_plus2.7392.7419.2	2	17.81	0	1.53E+06		(R)FSTPEQ/	(R)F/S|T/P/E/Q A A/K(N)		93.63	489.7474	978.489	-0.0016	-1.6	6	64759.2	8.88	HUMAN	P14866	Heterogeneous nuclear ribonucleoprotein L
1161	dirMS_041712_NAR_50mM_plus2.8653.8655.3	3	17.81	0	4.30E+05		(R)AALAHS	(R)A A/L A H S E E\V|T|A/S Q V|A A|T K(T)		112.93	595.312	1783.919	0.003	1.7	5.4	23903	4.94	HUMAN	H0YDN1	Plectin (Fragment)
1162	dirMS_041712_NAR_30mM_minus2.9027.9088.2	2	17.8	0	3.74E+05		(K)IANPVE(	(K)I/A|N|P/V/E|G S T D/R(Q)		103.72	579.7876	1158.575	-0.007	-6.1	4.37	34267.8	9.05	HUMAN	B4DXP5	Poly(rC)-binding protein 2
1163	dirMS_041712_NAR_40mM_minus2.12871.13017.3	3	17.8	0	3.35E+05		(R)DVQIGD	(R)D V|Q|I|G/D/I/V/T/V/G E C R P L S K(T)	C:Carbamidomethylation	159.67	662.684	1986.033	0.005	2.5	4.56	18601.6	10.52	HUMAN	P62280	40S ribosomal protein S11
1164	dirMS_041712_NAR_20mM_plus.16338.16462.2	2	17.8	0	1.09E+05		(K)FIIPNVV	(K)F/I|I|P/N V/V K(Y)		184.45	465.2935	929.582	-0.0021	-2.3	8.75	17529.3	9.3	HUMAN	F5GXY2	L-lactate dehydrogenase A chain (Fragment)
1165	dirMS_041712_NAR_30mM_minus2.19034.19034.2	2	17.79	0	9.18E+05		(K)MSIYQA	(K)M/S/I/Y/Q A M|W/K(G)		158.88	579.2789	1157.548	0.0023	2	8.34	266161	5.68	HUMAN	E9PPU0	Epiplakin
1166	dirMS_041712_NAR_50mM_minus2.7123.7144.3	3	17.79	0	1.07E+05		(R)VGQAVI	(R)V/G/Q|A/V/D|V/V/G Q/A G K P K(T)		107.38	484.9436	1452.817	-0.0007	-0.5	8.56	86346.5	5.05	HUMAN	E7EW34	26S proteasome non-ATPase regulatory subunit 2
1167	dirMS_041712_NAR_30mM_plus2.8673.8740.2	2	17.79	0	1.26E+06		(R)INISEGN	(R)I/N|I|S|E/G N|C/P E/R(I)	C:Carbamidomethylation	99.47	644.8007	1288.595	-0.0009	-0.7	4.53	32621	8.75	HUMAN	B4DLC0	Poly(rC)-binding protein 2
1168	dirMS_041712_NAR_50mM_plus2.9944.9995.3	3	17.79	1	5.47E+05		(R)LMEEIM	(R)L/M|E/E/I/M/S E K/E/N K(T)		119.72	494.241	1480.702	0.0063	4.2	4.49	61018.6	9.44	HUMAN	B4DLW8	Probable ATP-dependent RNA helicase DDX5
1169	dirMS_041712_NAR_60mM_plus.15502.15524.3	3	17.79	0	3.36E+05		(K)SKPGAA	(K)S K|P G A A\M\V|E|M/A/D/G Y/A/V D R(A)		137.83	623.2943	1867.868	0.0007	0.3	4.56	64759.2	8.88	HUMAN	P14866	Heterogeneous nuclear ribonucleoprotein L
1170	dirMS_041712_NAR_20mM_minus.13375.13483.2	2	17.77	0	6.91E+05		(K)DAGTIA(	(K)D A\G/T|I|A|G/L/N V L/R(I)		184.93	600.3403	1199.674	-0.0009	-0.8	5.84	17973	5.16	HUMAN	E9PI65	Heat shock cognate 71 kDa protein (Fragment)
1171	dirMS_041712_NAR_40mM_minus2.11083.11127.3	3	17.77	0	7.92E+05		(R)QVSASE	(R)Q V|S/A/S E/L H T S G I/L/G/P E T L R(D)		148.75	665.6822	1995.051	-0.0185	-9.3	5.4	558007.1	5.44	HUMAN	P58107	Epiplakin
1172	dirMS_041712_NAR_30mM_plus2.13306.13409.2	2	17.77	0	4.02E+05		(K)AFCGFEI	(K)A F|C|G\F|E D|P/R(T)	C:Carbamidomethylation	123.03	549.7374	1098.467	0.0002	0.2	4.37	533778	5.74	HUMAN	Q15149	Plectin
1173	dirMS_041712_NAR_30mM_plus2.18610.18610.2	2	17.77	0	2.24E+05		(R)GNPTVE	(R)G N|P T/V/E/V D L F T S K(G)		150.92	703.8613	1406.716	-0.0009	-0.6	4.37	21077	9.05	HUMAN	K7EM90	Alpha-enolase (Fragment)
1174	dirMS_041712_NAR_30mM_plus2.8139.8139.2	2	17.77	0	3.24E+05		(K)IAVAAQ	(K)I A|V/A/A Q/N C Y K(V)	C:Carbamidomethylation	96	569.2905	1137.572	0.0016	1.4	8.59	31075.7	5.65	HUMAN	P60174	Triosephosphate isomerase
1175	dirMS_041712_NAR_40mM_minus2.15966.16010.3	3	17.76	0	1.60E+06	M166m	(K)VLDFEHI	(K)V L|D/F E H F\L|P m L Q/T V/A/K(N)	m:Oxidized methionine	187.72	635.3376	1888.004	15.9945	-0.2	5.32	26991.7	5.01	HUMAN	B7Z6Z4	Myosin light polypeptide 6
1176	dirMS_041712_NAR_60mM_plus.12884.13004.3	3	17.76	0	1.05E+06		(K)GFVEFA/	(K)G F|V/E/F/A/A/K/P P A|R(K)		124.37	430.5686	1289.7	-0.0088	-6.9	8.75	58856.7	6.26	HUMAN	Q8WXF1	Paraspeckle component 1
1177	dirMS_041712_NAR_60mM_plus.15038.15244.3	3	17.76	0	5.71E+05		(K)LSMKPE	(K)L/S|M/K/P E/P/L/F/R(S)		135.85	406.5616	1217.671	-0.0008	-0.7	8.75	75482.2	6.34	HUMAN	D6RA32	Microtubule-associated protein 1B (Fragment)
1178	dirMS_041712_NAR_30mM_minus2.10575.10661.2	2	17.75	0	8.47E+05		(K)TGQEIP\	(K)T G|Q|E|I|P V/N V R(F)		111.63	556.8062	1112.606	-0.0007	-0.7	5.66	28051.5	4.98	HUMAN	J3QKT2	Cytosolic non-specific dipeptidase (Fragment)
1179	dirMS_041712_NAR_30mM_minus2.11862.11862.2	2	17.75	0	9.21E+05		(K)EVDEQM	(K)E V/D/E/Q M L|N/V/Q N/K(N)		117.53	723.8476	1446.689	-0.0014	-1	4.14	40069.4	4.83	HUMAN	E7EWR1	Tubulin beta chain
1180	dirMS_041712_NAR_50mM_minus2.14688.14859.3	3	17.75	1	4.18E+05		(K)GCLELIK	(K)G C|L|E|L I\K E T/G\V|P/I/A/G R(H)	C:Carbamidomethylation	151.82	571.6494	1712.936	-0.0027	-1.6	6.14	111409.8	9.02	HUMAN	F5H2F4	C-1-tetrahydrofolate synthase, cytoplasmic
1181	dirMS_041712_NAR_40mM_plus2.11002.11038.3	3	17.75	1	6.32E+04		(K)VAVVAG	(K)V/A|V|V|A/G|Y G D/V G K G C A Q A L/R(G)	C:Carbamidomethylation	131.05	630.9981	1890.985	-0.0057	-3	8.56	45228.3	6.03	HUMAN	F5H737	Adenosylhomocysteinase
1182	dirMS_041712_NAR_50mM_plus2.9774.9880.3	3	17.75	0	1.92E+06		(R)EIVHIQA	(R)E/I V H I Q\A\G/Q/C|G/N Q I|G A/K(F)	C:Carbamidomethylation	119.02	608.3107	1822.923	-0.0053	-2.9	6.85	40069.4	4.83	HUMAN	E7EWR1	Tubulin beta chain
1183	dirMS_041712_NAR_50mM_minus2.5827.5939.3	3	17.74	1	1.62E+06		(R)AQLGGF	(R)A Q|L|G/G/P E/A/A/K S D E T A A/K(-)		95.6	548.6119	1643.824	-0.0024	-1.4	4.68	3888.3	5	HUMAN	C9J3N8	Heat shock protein beta-1
1184	dirMS_041712_NAR_20mM_plus.7655.7773.2	2	17.74	0	6.94E+05		(K)VIDDTNI	(K)V/I|D|D|T/N/I/T/R(L)		123.18	523.7766	1046.548	-0.0017	-1.7	4.21	43773.3	5.26	HUMAN	F8VZY9	Keratin, type I cytoskeletal 18
1185	dirMS_041712_NAR_30mM_plus2.16154.16154.2	2	17.74	0	2.79E+05		(K)MDQAII	(K)M/D/Q|A|I/I/F C R(T)	C:Carbamidomethylation	137.37	577.2809	1153.549	0.0053	4.6	5.59	83400.6	6.82	HUMAN	Q92499	ATP-dependent RNA helicase DDX1
1186	dirMS_041712_NAR_30mM_plus2.18712.18963.2	2	17.74	0	5.37E+05		(R)LIDFLES(	(R)L/I|D|F/L/E/S/G K(T)		152.02	511.2819	1021.556	0.0001	0.1	4.37	73202	9.19	HUMAN	C9JMU5	Probable ATP-dependent RNA helicase DDX17
1187	dirMS_041712_NAR_60mM_plus.9705.9887.3	3	17.74	1	1.31E+06		(K)LAELKQI	(K)L/A|E/L/K/Q/E/C L A/R(G)	C:Carbamidomethylation	109.57	444.2368	1330.715	-0.0189	-14.2	6.14	6106	9.22	HUMAN	F8VS12	SAP domain-containing ribonucleoprotein
1188	dirMS_041712_NAR_120mM_minus.10925.11027.3	3	17.73	1	6.07E+05		(R)FYKNEG	(R)F/Y|K N E G/G/T|W S/V/E K(V)		113.78	515.5834	1544.738	-0.0023	-1.5	6.14	39066	6.61	HUMAN	A6PVW9	Selenium binding protein 1 (Fragment)
1189	dirMS_041712_NAR_20mM_plus.18660.18660.2	2	17.73	0	2.87E+05		(K)LAVEALS	(K)L A V\E/A/L|S S L/D G D L/A/G R(Y)		200.48	793.9214	1586.838	-0.0029	-1.8	4.03	24286.7	4.89	HUMAN	G3V4N7	Creatine kinase B-type (Fragment)
1190	dirMS_041712_NAR_40mM_plus2.18357.18357.3	3	17.73	1	6.16E+04		(R)VLEALLP	(R)V/L|E/A L L/P L K/G L E E R(V)		174.47	527.3177	1579.942	-0.0032	-2	4.79	276046.1	6.01	HUMAN	P49327	Fatty acid synthase
1191	dirMS_041712_NAR_50mM_plus2.10309.10384.3	3	17.73	1	1.53E+06		(K)VTVAGL	(K)V/T|V|A/G/L/A/G K/D/P V Q/C S R(D)	C:Carbamidomethylation	121.92	553.2933	1657.869	-0.0037	-2.2	8.72	18080.7	7.92	HUMAN	K7ELW0	Protein DJ-1
1192	dirMS_041712_NAR_60mM_plus.10836.11129.3	3	17.73	1	6.41E+06		(R)QQEGFK	(R)Q Q/E/G F/K/G T/F/P/D/A R(E)		115.52	494.2424	1480.718	-0.0053	-3.6	6.07	43979.5	8.92	HUMAN	F5GYZ3	Non-POU domain-containing octamer-binding protein
1193	dirMS_041712_NAR_20mM_minus.12161.12161.2	2	17.72	0	1.06E+06	M313m	(R)TVQSLEI	(R)T V|Q S/L/E|I D L/D S m R(N)	m:Oxidized methionine	170.63	761.8733	1506.747	15.9925	-1.6	4.03	43773.3	5.26	HUMAN	F8VZY9	Keratin, type I cytoskeletal 18
1194	dirMS_041712_NAR_50mM_minus2.14931.15297.3	3	17.72	1	1.13E+06		(R)DVDFELI	(R)D V|D/F/E/L I/K V/E/G K(V)		154.02	464.5852	1391.742	-0.0006	-0.4	4.32	49982	5.86	HUMAN	B4DYD8	T-complex protein 1 subunit epsilon

<table>
<tr><td>1195</td><td>dirMS_041712_NAR_50mM_minus2.15612.15888.3</td><td>3</td><td>17.72</td><td>1</td><td>4.93E+05</td><td></td><td>(K)QGGLGF</td><td>(K)Q G G L/G/P\M N\I/P L/V/S/D/P K R(T)</td><td></td><td>157.5</td><td>593.6566</td><td>1778.958</td><td>-0.0029</td><td>-1.6</td><td>8.75</td><td>22338.2</td><td>8.65</td><td>HUMAN</td><td>Q06830</td><td>Peroxiredoxin-1</td></tr>
<tr><td>1196</td><td>dirMS_041712_NAR_50mM_minus2.16970.17157.3</td><td>3</td><td>17.72</td><td>0</td><td>1.33E+07</td><td></td><td>(R)IHFPLAT</td><td>(R)I H F\P L\A\T|Y|A|P/V/I/S A E K(A)</td><td></td><td>166.73</td><td>586.3248</td><td>1756.963</td><td>-0.0034</td><td>-1.9</td><td>6.75</td><td>49012.3</td><td>4.9</td><td>HUMAN</td><td>A8MUB1</td><td>Tubulin alpha-4A chain</td></tr>
<tr><td>1197</td><td>dirMS_041712_NAR_120mM_plus.10712.10817.3</td><td>3</td><td>17.72</td><td>0</td><td>4.24E+05</td><td></td><td>(K)GSYVSIH</td><td>(K)G S|Y/V/S I/H/S/S G\F/R(D)</td><td></td><td>109.6</td><td>432.8823</td><td>1296.633</td><td>-0.0008</td><td>-0.6</td><td>8.75</td><td>30944.2</td><td>6.52</td><td>HUMAN</td><td>B1Q2N1</td><td>ATP-dependent RNA helicase DDX39A</td></tr>
<tr><td>1198</td><td>dirMS_041712_NAR_50mM_plus2.17471.17576.3</td><td>3</td><td>17.72</td><td>1</td><td>7.15E+05</td><td></td><td>(R)CITDPQT</td><td>(R)C|I|T/D/P Q T/G/L/C L/L/P L K E K(K)</td><td>C:Carbamidomethylation</td><td>160.15</td><td>662.6825</td><td>1986.04</td><td>-0.0069</td><td>-3.5</td><td>6.07</td><td>533778</td><td>5.74</td><td>HUMAN</td><td>Q15149</td><td>Plectin</td></tr>
<tr><td>1199</td><td>dirMS_041712_NAR_120mM_minus.12286.12808.3</td><td>3</td><td>17.71</td><td>1</td><td>3.70E+06</td><td></td><td>(K)VKLEAEI</td><td>(K)V K L E\A\E|I|A/T/Y/R(R)</td><td></td><td>123.3</td><td>431.5783</td><td>1292.721</td><td>-0.0005</td><td>-0.4</td><td>6.11</td><td>43773.3</td><td>5.26</td><td>HUMAN</td><td>F8VZY9</td><td>Keratin, type I cytoskeletal 18</td></tr>
<tr><td>1200</td><td>dirMS_041712_NAR_120mM_minus.19581.19642.3</td><td>3</td><td>17.71</td><td>0</td><td>7.14E+05</td><td></td><td>(R)NSVSNF</td><td>(R)N/S|V/S N/F/L/H/S L E/R(G)</td><td></td><td>162.22</td><td>468.2346</td><td>1402.707</td><td>-0.0181</td><td>-12.9</td><td>6.75</td><td>267260.7</td><td>5.95</td><td>HUMAN</td><td>Q13085</td><td>Acetyl-CoA carboxylase 1</td></tr>
<tr><td>1201</td><td>dirMS_041712_NAR_30mM_minus2.12675.13014.2</td><td>2</td><td>17.71</td><td>0</td><td>7.94E+05</td><td></td><td>(K)LVIITAG</td><td>(K)L/V|I|I/T/A|G A/R(Q)</td><td></td><td>123.4</td><td>457.2948</td><td>913.583</td><td>-0.0006</td><td>-0.7</td><td>9.75</td><td>12539.4</td><td>5.78</td><td>HUMAN</td><td>F5GXH2</td><td>L-lactate dehydrogenase A chain (Fragment)</td></tr>
<tr><td>1202</td><td>dirMS_041712_NAR_40mM_minus2.11638.11940.3</td><td>3</td><td>17.71</td><td>1</td><td>2.86E+06</td><td></td><td>(R)IINEPTA</td><td>(R)I I\N|E/P T/A/A/A/I/A/Y G L D/K K(V)</td><td></td><td>153.08</td><td>596.6671</td><td>1787.99</td><td>-0.0034</td><td>-1.9</td><td>6.07</td><td>17973</td><td>5.16</td><td>HUMAN</td><td>E9PI65</td><td>Heat shock cognate 71 kDa protein (Fragment)</td></tr>
<tr><td>1203</td><td>dirMS_041712_NAR_50mM_minus2.17277.17289.3</td><td>3</td><td>17.71</td><td>1</td><td>8.80E+05</td><td></td><td>(K)IILDLISE</td><td>(K)I/I|L/D/L/I/S/E S P I K G/R(A)</td><td></td><td>167.65</td><td>518.6444</td><td>1553.926</td><td>-0.0075</td><td>-4.8</td><td>6.07</td><td>51260.6</td><td>5.39</td><td>HUMAN</td><td>P61978</td><td>Heterogeneous nuclear ribonucleoprotein K</td></tr>
<tr><td>1204</td><td>dirMS_041712_NAR_60mM_plus.17408.17595.3</td><td>3</td><td>17.71</td><td>0</td><td>1.38E+06</td><td></td><td>(R)MGPLGL</td><td>(R)M/G|P|L/G/L D/H/M/A/S S I E/R(M)</td><td></td><td>148.47</td><td>538.5969</td><td>1613.777</td><td>-0.0013</td><td>-0.8</td><td>5.3</td><td>77799.7</td><td>9.09</td><td>HUMAN</td><td>P52272</td><td>Heterogeneous nuclear ribonucleoprotein M</td></tr>
<tr><td>1205</td><td>dirMS_041712_NAR_30mM_minus2.18465.18465.2</td><td>2</td><td>17.7</td><td>0</td><td>1.20E+06</td><td></td><td>(R)DLTDYLI</td><td>(R)D L|T/D|Y/L M/K(I)</td><td></td><td>155.48</td><td>499.7499</td><td>998.486</td><td>0.0062</td><td>6.2</td><td>4.21</td><td>37482.1</td><td>5.36</td><td>HUMAN</td><td>E9PG30</td><td>Actin, gamma-enteric smooth muscle</td></tr>
<tr><td>1206</td><td>dirMS_041712_NAR_60mM_minus.15271.15542.3</td><td>3</td><td>17.7</td><td>1</td><td>4.74E+05</td><td></td><td>(R)LLDAQL</td><td>(R)L/L|D|A/Q|L|A T/G G L V C/P A R R(L)</td><td>C:Carbamidomethylation</td><td>146.02</td><td>604.3344</td><td>1810.996</td><td>-0.007</td><td>-3.8</td><td>9.6</td><td>266161</td><td>5.68</td><td>HUMAN</td><td>E9PPU0</td><td>Epiplakin</td></tr>
<tr><td>1207</td><td>dirMS_041712_NAR_30mM_plus2.22595.22612.2</td><td>2</td><td>17.7</td><td>0</td><td>1.01E+06</td><td></td><td>(R)FLTWILN</td><td>(R)F/L|T|W\I/L/N/R(Y)</td><td></td><td>175.88</td><td>531.8078</td><td>1062.61</td><td>-0.0012</td><td>-1.1</td><td>9.75</td><td>35548.6</td><td>4.91</td><td>HUMAN</td><td>B4DN60</td><td>Asparagine--tRNA ligase, cytoplasmic</td></tr>
<tr><td>1208</td><td>dirMS_041712_NAR_40mM_plus2.15142.15142.3</td><td>3</td><td>17.7</td><td>0</td><td>3.07E+05</td><td></td><td>(K)GVTIIGP</td><td>(K)G V|T|I/I/G/P A T V/G G I K P G C F K(I)</td><td>C:Carbamidomethylation</td><td>156.13</td><td>624.683</td><td>1872.041</td><td>-0.0067</td><td>-3.6</td><td>10</td><td>91839.6</td><td>9.22</td><td>HUMAN</td><td>B4E3P0</td><td>ATP-citrate synthase</td></tr>
<tr><td>1209</td><td>dirMS_041712_NAR_60mM_plus.13241.13241.3</td><td>3</td><td>17.7</td><td>1</td><td>2.80E+04</td><td></td><td>(K)AAAGKA</td><td>(K)A A/A/G/K A E L|E/L E\L/G R(I)</td><td></td><td>126.13</td><td>476.5985</td><td>1427.785</td><td>-0.0043</td><td>-3</td><td>4.79</td><td>533778</td><td>5.74</td><td>HUMAN</td><td>Q15149</td><td>Plectin</td></tr>
<tr><td>1210</td><td>dirMS_041712_NAR_20mM_plus.8342.8342.2</td><td>2</td><td>17.69</td><td>0</td><td>8.56E+04</td><td></td><td>(R)LAPEYEA</td><td>(R)L A|P/E/Y E/A A/A T/R(L)</td><td></td><td>127.75</td><td>596.3035</td><td>1191.6</td><td>-0.0007</td><td>-0.6</td><td>4.53</td><td>55361.8</td><td>6.42</td><td>HUMAN</td><td>G5EA52</td><td>Protein disulfide isomerase family A, member 3, isoform CRA_b</td></tr>
<tr><td>1211</td><td>dirMS_041712_NAR_40mM_plus2.18217.18412.3</td><td>3</td><td>17.69</td><td>0</td><td>3.43E+05</td><td></td><td>(R)EAVELPI</td><td>(R)E A|V/E/L/P L/T/H F E L/Y K(Q)</td><td></td><td>173.8</td><td>563.6336</td><td>1688.889</td><td>-0.0032</td><td>-1.9</td><td>4.75</td><td>47479.5</td><td>5.09</td><td>HUMAN</td><td>P43686</td><td>26S protease regulatory subunit 6B</td></tr>
<tr><td>1212</td><td>dirMS_041712_NAR_50mM_minus2.11307.11475.3</td><td>3</td><td>17.68</td><td>0</td><td>1.47E+05</td><td></td><td>(K)IVLEDGT</td><td>(K)I/V|L|E/D G T L/H/V/T E/G S G R(Y)</td><td></td><td>133.15</td><td>561.6271</td><td>1682.871</td><td>-0.004</td><td>-2.4</td><td>4.65</td><td>62748.9</td><td>5.95</td><td>HUMAN</td><td>Q16555</td><td>Dihydropyrimidinase-related protein 2</td></tr>
<tr><td>1213</td><td>dirMS_041712_NAR_50mM_minus2.12464.12464.3</td><td>3</td><td>17.68</td><td>0</td><td>2.90E+05</td><td></td><td>(K)AASDIAI</td><td>(K)A/A/S D\I/A|M T E L/P P T H/P I R(L)</td><td></td><td>139.23</td><td>607.3158</td><td>1819.937</td><td>-0.0042</td><td>-2.3</td><td>5.32</td><td>12926.1</td><td>4.4</td><td>HUMAN</td><td>K7EM20</td><td>14-3-3 protein epsilon (Fragment)</td></tr>
<tr><td>1214</td><td>dirMS_041712_NAR_50mM_minus2.12681.12736.3</td><td>3</td><td>17.68</td><td>0</td><td>2.42E+05</td><td></td><td>(K)FLSDPQ</td><td>(K)F/L|S/D/P Q/V/H/T V/L V E R(S)</td><td></td><td>140.57</td><td>547.2977</td><td>1639.88</td><td>-0.0017</td><td>-1</td><td>5.32</td><td>535136.6</td><td>6.01</td><td>HUMAN</td><td>Q14204</td><td>Cytoplasmic dynein 1 heavy chain 1</td></tr>
<tr><td>1215</td><td>dirMS_041712_NAR_50mM_minus2.8944.8944.3</td><td>3</td><td>17.68</td><td>1</td><td>1.16E+05</td><td>M88m</td><td>(K)DVDEAY</td><td>(K)D V|D E/A/Y m N K/V/E L E/S R(L)</td><td>m:Oxidized methionine</td><td>118.62</td><td>605.2817</td><td>1797.832</td><td>15.9982</td><td>1.8</td><td>4.18</td><td>13588.1</td><td>4.66</td><td>HUMAN</td><td>F8VP67</td><td>Keratin, type II cytoskeletal 8 (Fragment)</td></tr>
<tr><td>1216</td><td>dirMS_041712_NAR_120mM_plus.21148.21365.4</td><td>4</td><td>17.68</td><td>1</td><td>1.33E+06</td><td></td><td>(R)DYLHLPI</td><td>(R)D Y L H/L|P|P/E I/V/P A T L R/R(S)</td><td></td><td>163.95</td><td>473.2689</td><td>1890.06</td><td>-0.0058</td><td>-3.1</td><td>6.75</td><td>19924.8</td><td>9.99</td><td>HUMAN</td><td>F6U211</td><td>40S ribosomal protein S10</td></tr>
<tr><td>1217</td><td>dirMS_041712_NAR_30mM_plus2.15648.15648.2</td><td>2</td><td>17.68</td><td>0</td><td>1.34E+06</td><td></td><td>(R)QITVND</td><td>(R)Q I|T V/N/D/L|P V G/R(S)</td><td></td><td>133.82</td><td>606.3403</td><td>1211.674</td><td>-0.0009</td><td>-0.8</td><td>5.84</td><td>22062.7</td><td>5.66</td><td>HUMAN</td><td>P32119</td><td>Peroxiredoxin-2</td></tr>
<tr><td>1218</td><td>dirMS_041712_NAR_30mM_plus2.16488.16511.2</td><td>2</td><td>17.68</td><td>0</td><td>3.06E+05</td><td></td><td>(K)LLDLVQ(</td><td>(K)L L|D\L|V|Q|Q|S/C N/Y K(Q)</td><td>C:Carbamidomethylation</td><td>139.12</td><td>740.8751</td><td>1480.746</td><td>-0.0035</td><td>-2.4</td><td>5.83</td><td>14855.1</td><td>9.26</td><td>HUMAN</td><td>B1AHD1</td><td>NHP2 non-histone chromosome protein 2-like 1 (S. cerevisiae)</td></tr>
<tr><td>1219</td><td>dirMS_041712_NAR_50mM_plus2.18175.18446.3</td><td>3</td><td>17.68</td><td>1</td><td>5.52E+05</td><td></td><td>(K)VIAATNI</td><td>(K)V/I|A|A/T N R V D\I|L/D/P A L L/R(S)</td><td></td><td>164.48</td><td>617.3645</td><td>1850.086</td><td>-0.0069</td><td>-3.7</td><td>5.93</td><td>32827.5</td><td>5.92</td><td>HUMAN</td><td>E9PKD5</td><td>26S protease regulatory subunit 6A (Fragment)</td></tr>
<tr><td>1220</td><td>dirMS_041712_NAR_120mM_minus.13297.13297.3</td><td>3</td><td>17.67</td><td>2</td><td>2.61E+05</td><td></td><td>(R)AQKDEE</td><td>(R)A Q/K/D/E E/K/M E|I/Q/E/I\Q L K(E)</td><td></td><td>126.68</td><td>654.0058</td><td>1960.006</td><td>-0.0027</td><td>-1.4</td><td>4.58</td><td>31923.6</td><td>4.85</td><td>HUMAN</td><td>B7Z596</td><td>Tropomyosin alpha-1 chain</td></tr>
<tr><td>1221</td><td>dirMS_041712_NAR_30mM_minus2.12641.13113.2</td><td>2</td><td>17.67</td><td>0</td><td>2.03E+06</td><td></td><td>(K)IGGIGTV</td><td>(K)I/G|G|I|G/T/V|P V G R(V)</td><td></td><td>123.42</td><td>513.3086</td><td>1025.61</td><td>-0.0003</td><td>-0.3</td><td>9.75</td><td>50482.3</td><td>9.31</td><td>HUMAN</td><td>P68104</td><td>Elongation factor 1-alpha 1</td></tr>
<tr><td>1222</td><td>dirMS_041712_NAR_30mM_plus2.18129.18129.2</td><td>2</td><td>17.67</td><td>0</td><td>2.94E+05</td><td></td><td>(R)VNPALA</td><td>(R)V N/P A/L/A/E L/N L/R(S)</td><td></td><td>147.85</td><td>605.3509</td><td>1209.695</td><td>-0.0005</td><td>-0.4</td><td>5.97</td><td>20026.6</td><td>4.67</td><td>HUMAN</td><td>E9PIK5</td><td>Ribonuclease inhibitor (Fragment)</td></tr>
<tr><td>1223</td><td>dirMS_041712_NAR_30mM_plus2.23507.23551.2</td><td>2</td><td>17.67</td><td>0</td><td>4.18E+06</td><td></td><td>(R)IAIYELLF</td><td>(R)I/A|I|Y/E|L/L/F K(E)</td><td></td><td>191.57</td><td>555.3331</td><td>1109.661</td><td>-0.0016</td><td>-1.4</td><td>6</td><td>19924.8</td><td>9.99</td><td>HUMAN</td><td>F6U211</td><td>40S ribosomal protein S10</td></tr>
<tr><td>1224</td><td>dirMS_041712_NAR_40mM_plus2.12982.12982.3</td><td>3</td><td>17.67</td><td>1</td><td>9.95E+05</td><td></td><td>(K)TITLEVEI</td><td>(K)T I|T|L/E V/E/P S D T I E N V K A K(I)</td><td></td><td>143.45</td><td>663.0201</td><td>1987.059</td><td>-0.0136</td><td>-6.9</td><td>4.41</td><td>17271.6</td><td>6.75</td><td>HUMAN</td><td>B4DV12</td><td>Ubiquitin</td></tr>
<tr><td>1225</td><td>dirMS_041712_NAR_60mM_minus.10726.11191.3</td><td>3</td><td>17.66</td><td>1</td><td>2.04E+06</td><td></td><td>(R)FPGQLN</td><td>(R)F/P/G/Q|L/N/A/D/L R\K(L)</td><td></td><td>121.02</td><td>420.2346</td><td>1258.69</td><td>-0.001</td><td>-0.8</td><td>8.75</td><td>42230.8</td><td>4.77</td><td>HUMAN</td><td>A6NKZ8</td><td>Putative tubulin beta chain-like protein ENSP00000290377</td></tr>
<tr><td>1226</td><td>dirMS_041712_NAR_20mM_plus.14630.14843.2</td><td>2</td><td>17.66</td><td>0</td><td>9.93E+04</td><td></td><td>(K)FLDGIYV</td><td>(K)F/L|D|G/I|Y/V/S E/K(G)</td><td></td><td>173.5</td><td>585.8066</td><td>1170.604</td><td>0.0018</td><td>1.5</td><td>4.37</td><td>20931.1</td><td>10.28</td><td>HUMAN</td><td>D6RAN4</td><td>60S ribosomal protein L9 (Fragment)</td></tr>
<tr><td>1227</td><td>dirMS_041712_NAR_60mM_minus.13279.13481.3</td><td>3</td><td>17.65</td><td>1</td><td>5.13E+05</td><td>M313m</td><td>(R)RTVQSLI</td><td>(R)R T V\Q\S\L|E|I/D|L/D/S m R(N)</td><td>m:Oxidized methionine</td><td>134.52</td><td>560.2852</td><td>1662.848</td><td>15.9931</td><td>-1.1</td><td>4.56</td><td>43773.3</td><td>5.26</td><td>HUMAN</td><td>F8VZY9</td><td>Keratin, type I cytoskeletal 18</td></tr>
<tr><td>1228</td><td>dirMS_041712_NAR_20mM_plus.17111.17111.3</td><td>3</td><td>17.65</td><td>0</td><td>2.26E+05</td><td></td><td>(R)AIVAIEN</td><td>(R)A I\V/A I E/N/P A/D V/S/V/I/S S R(N)</td><td></td><td>188.93</td><td>580.9864</td><td>1740.949</td><td>-0.0044</td><td>-2.5</td><td>4.37</td><td>33022.6</td><td>4.78</td><td>HUMAN</td><td>A6NE09</td><td>Protein RPSAP58</td></tr>
<tr><td>1229</td><td>dirMS_041712_NAR_30mM_minus2.9362.9458.2</td><td>2</td><td>17.64</td><td>0</td><td>1.72E+06</td><td>M406m</td><td>(R)EYQELm</td><td>(R)E Y/Q|E/L/m/N V/K(L)</td><td>m:Oxidized methionine</td><td>105.6</td><td>585.2783</td><td>1153.556</td><td>15.9935</td><td>-1.2</td><td>4.53</td><td>56607.4</td><td>5.37</td><td>HUMAN</td><td>F8VXB4</td><td>Keratin, type II cytoskeletal 8</td></tr>
<tr><td>1230</td><td>dirMS_041712_NAR_50mM_minus2.15493.15547.3</td><td>3</td><td>17.64</td><td>1</td><td>1.73E+05</td><td></td><td>(K)DVLFLKI</td><td>(K)D V|L|F|L K/D C V/G/P/E/V E K(A)</td><td>C:Carbamidomethylation</td><td>156.42</td><td>583.3014</td><td>1747.893</td><td>-0.0039</td><td>-2.2</td><td>4.32</td><td>41827.5</td><td>7</td><td>HUMAN</td><td>B7Z7A9</td><td>Phosphoglycerate kinase</td></tr>
<tr><td>1231</td><td>dirMS_041712_NAR_20mM_plus.18652.18744.2</td><td>2</td><td>17.64</td><td>0</td><td>2.37E+05</td><td></td><td>(K)IYVDDGI</td><td>(K)I Y|V/D/D|G|L/I S/L/Q V K(Q)</td><td></td><td>200.67</td><td>731.9068</td><td>1462.815</td><td>-0.0088</td><td>-6</td><td>4.21</td><td>30947.9</td><td>6.47</td><td>HUMAN</td><td>H3BQ34</td><td>Pyruvate kinase</td></tr>
<tr><td>1232</td><td>dirMS_041712_NAR_50mM_plus2.12510.12510.3</td><td>3</td><td>17.64</td><td>1</td><td>3.06E+05</td><td></td><td>(R)SGVGLS</td><td>(R)S G V|G L/S/S Y/L/S T E K D A S|P K(T)</td><td></td><td>133.63</td><td>609.3102</td><td>1825.918</td><td>-0.0018</td><td>-1</td><td>5.79</td><td>64248.4</td><td>9.08</td><td>HUMAN</td><td>Q9BW04</td><td>Specifically androgen-regulated gene protein</td></tr>
<tr><td>1233</td><td>dirMS_041712_NAR_60mM_plus.15811.15877.3</td><td>3</td><td>17.64</td><td>0</td><td>4.58E+05</td><td></td><td>(R)VWYVSN</td><td>(R)V/W|Y V/S/N I/D/G T H I A/K(T)</td><td></td><td>139.78</td><td>534.9464</td><td>1602.828</td><td>-0.0028</td><td>-1.8</td><td>6.71</td><td>63374.2</td><td>8.64</td><td>HUMAN</td><td>P06744</td><td>Glucose-6-phosphate isomerase</td></tr>
<tr><td>1234</td><td>dirMS_041712_NAR_60mM_plus.16200.16318.3</td><td>3</td><td>17.64</td><td>0</td><td>6.45E+05</td><td></td><td>(R)ETHSAL(</td><td>(R)E T H S A|L|G/P|A|L|Q/A A/F K(L)</td><td></td><td>141.95</td><td>514.2744</td><td>1540.812</td><td>-0.0032</td><td>-2.1</td><td>6.85</td><td>141616.6</td><td>6.1</td><td>HUMAN</td><td>B7ZKM8</td><td>Protein transport protein Sec24B</td></tr>
<tr><td>1235</td><td>dirMS_041712_NAR_60mM_plus.20223.20223.3</td><td>3</td><td>17.64</td><td>1</td><td>3.02E+05</td><td></td><td>(K)IILDLISE</td><td>(K)I I/L/D/L I|S E S P/I K G\R(A)</td><td></td><td>166.2</td><td>518.6452</td><td>1553.926</td><td>-0.0051</td><td>-3.3</td><td>6.07</td><td>51260.6</td><td>5.39</td><td>HUMAN</td><td>P61978</td><td>Heterogeneous nuclear ribonucleoprotein K</td></tr>
<tr><td>1236</td><td>dirMS_041712_NAR_20mM_plus.17117.17452.2</td><td>2</td><td>17.63</td><td>0</td><td>1.44E+06</td><td></td><td>(R)VLITTDL</td><td>(R)V/L|I|T|T/D/L/L/A R(G)</td><td></td><td>191</td><td>557.8442</td><td>1114.683</td><td>-0.0019</td><td>-1.7</td><td>5.81</td><td>41517.9</td><td>5.45</td><td>HUMAN</td><td>E7EQG2</td><td>Eukaryotic initiation factor 4A-II</td></tr>
<tr><td>1237</td><td>dirMS_041712_NAR_40mM_plus2.12334.12334.2</td><td>2</td><td>17.63</td><td>0</td><td>1.06E+05</td><td></td><td>(R)VLQALE(</td><td>(R)V/L|Q|A L E/G/L K(M)</td><td></td><td>138.8</td><td>485.8002</td><td>970.593</td><td>0</td><td>0</td><td>5.97</td><td>16060.2</td><td>10.31</td><td>HUMAN</td><td>P39019</td><td>40S ribosomal protein S19</td></tr>
<tr><td>1238</td><td>dirMS_041712_NAR_40mM_plus2.13516.13717.3</td><td>3</td><td>17.63</td><td>0</td><td>6.25E+05</td><td></td><td>(R)TIQVEN</td><td>(R)T/I|Q|V/E N\S H L/I|L/T/G A/G A L N K(V)</td><td></td><td>146.55</td><td>660.3654</td><td>1979.092</td><td>-0.0104</td><td>-5.2</td><td>6.41</td><td>267260.7</td><td>5.95</td><td>HUMAN</td><td>Q13085</td><td>Acetyl-CoA carboxylase 1</td></tr>
<tr><td>1239</td><td>dirMS_041712_NAR_120mM_plus.11631.11631.2</td><td>2</td><td>17.62</td><td>1</td><td>8.55E+05</td><td></td><td>(R)NLKASLI</td><td>(R)N/L/K/A/S|L E/N S L R(E)</td><td></td><td>114.02</td><td>622.8511</td><td>1244.696</td><td>-0.0008</td><td>-0.6</td><td>8.75</td><td>43773.3</td><td>5.26</td><td>HUMAN</td><td>F8VZY9</td><td>Keratin, type I cytoskeletal 18</td></tr>
<tr><td>1240</td><td>dirMS_041712_NAR_30mM_plus2.15835.15835.2</td><td>2</td><td>17.62</td><td>0</td><td>5.00E+05</td><td></td><td>(K)LLYNNV</td><td>(K)L/L|Y|N/N V/S/N/F G R(L)</td><td></td><td>136.15</td><td>648.8354</td><td>1296.67</td><td>-0.006</td><td>-4.6</td><td>8.75</td><td>71165.3</td><td>5.57</td><td>HUMAN</td><td>J3KS13</td><td>Clathrin heavy chain 1</td></tr>
<tr><td>1241</td><td>dirMS_041712_NAR_50mM_plus2.14007.14007.3</td><td>3</td><td>17.62</td><td>1</td><td>7.05E+05</td><td></td><td>(R)VLELVSI</td><td>(R)V L|E\L|V/S/I/T A/N K N/T C P G D R(S)</td><td>C:Carbamidomethylation</td><td>141.97</td><td>663.0129</td><td>1987.028</td><td>-0.0036</td><td>-1.8</td><td>6.04</td><td>45186.1</td><td>9.89</td><td>HUMAN</td><td>B4DLV4</td><td>Serine hydroxymethyltransferase</td></tr>
<tr><td>1242</td><td>dirMS_041712_NAR_50mM_plus2.18245.18245.3</td><td>3</td><td>17.62</td><td>1</td><td>6.23E+05</td><td></td><td>(K)LLEPVLL</td><td>(K)L/L/E/P/V/L/L/L G K E R(F)</td><td></td><td>164.48</td><td>460.6256</td><td>1379.862</td><td>0.0002</td><td>0.1</td><td>6.14</td><td>16559.1</td><td>10.36</td><td>HUMAN</td><td>P62249</td><td>40S ribosomal protein S16</td></tr>
<tr><td>1243</td><td>dirMS_041712_NAR_60mM_minus.18772.18772.3</td><td>3</td><td>17.61</td><td>1</td><td>1.31E+05</td><td></td><td>(K)IILDLISE</td><td>(K)I/I/L/D\L/I/S E S P I K G R(A)</td><td></td><td>166.22</td><td>518.6442</td><td>1553.926</td><td>-0.0081</td><td>-5.2</td><td>6.07</td><td>51260.6</td><td>5.39</td><td>HUMAN</td><td>P61978</td><td>Heterogeneous nuclear ribonucleoprotein K</td></tr>
<tr><td>1244</td><td>dirMS_041712_NAR_120mM_plus.20802.21062.3</td><td>3</td><td>17.61</td><td>2</td><td>1.39E+06</td><td></td><td>(R)TLAEIAK</td><td>(R)T L|A|E|I|A K V E L D/N M/P L R G K(Q)</td><td></td><td>162.92</td><td>666.7047</td><td>1998.105</td><td>-0.0057</td><td>-2.8</td><td>5.85</td><td>23729.5</td><td>9.89</td><td>HUMAN</td><td>C9IZL7</td><td>Non-POU domain-containing octamer-binding protein (Fragment)</td></tr>
<tr><td>1245</td><td>dirMS_041712_NAR_30mM_plus2.13530.13637.2</td><td>2</td><td>17.61</td><td>0</td><td>5.55E+05</td><td></td><td>(K)VGLQVV</td><td>(K)V/G|L|Q|V/V/A|V/K(A)</td><td></td><td>124.08</td><td>456.7974</td><td>912.588</td><td>-0.0002</td><td>-0.2</td><td>8.72</td><td>61224.7</td><td>5.7</td><td>HUMAN</td><td>P10809</td><td>60 kDa heat shock protein, mitochondrial</td></tr>
<tr><td>1246</td><td>dirMS_041712_NAR_120mM_minus.9669.9789.3</td><td>3</td><td>17.6</td><td>1</td><td>6.41E+05</td><td></td><td>(R)AREQAE</td><td>(R)A R E Q A\E\A|E|V|A|S/L/N\R(R)</td><td></td><td>107.8</td><td>515.2649</td><td>1543.782</td><td>-0.0022</td><td>-1.4</td><td>4.79</td><td>11017</td><td>4.8</td><td>HUMAN</td><td>D6R904</td><td>Tropomyosin alpha-3 chain</td></tr>
<tr><td>1247</td><td>dirMS_041712_NAR_30mM_minus2.14972.14986.2</td><td>2</td><td>17.6</td><td>0</td><td>1.45E+05</td><td></td><td>(K)GFFEGTI</td><td>(K)G/F|F|E/G T I/A/S/K(G)</td><td></td><td>134.95</td><td>528.7721</td><td>1056.536</td><td>0.0009</td><td>0.8</td><td>6</td><td>139883.7</td><td>8.06</td><td>HUMAN</td><td>P53621</td><td>Coatomer subunit alpha</td></tr>
<tr><td>1248</td><td>dirMS_041712_NAR_50mM_minus2.6762.6762.3</td><td>3</td><td>17.6</td><td>1</td><td>2.52E+05</td><td></td><td>(R)ASVDTK</td><td>(R)A S|V/D T K E A E|G\A|P Q V E/A G K(R)</td><td></td><td>104.8</td><td>596.2904</td><td>1786.882</td><td>-0.0251</td><td>-14</td><td>4.41</td><td>54214.9</td><td>5.66</td><td>HUMAN</td><td>C9J813</td><td>Caldesmon (Fragment)</td></tr>
<tr><td>1249</td><td>dirMS_041712_NAR_120mM_plus.9633.9705.3</td><td>3</td><td>17.6</td><td>1</td><td>4.59E+05</td><td></td><td>(R)KAYGGA</td><td>(R)K\A Y G\G\A|Y|D|V/M/S/S/K(H)</td><td></td><td>104.08</td><td>459.5555</td><td>1376.651</td><td>0.0005</td><td>0.3</td><td>8.43</td><td>61363</td><td>8.73</td><td>HUMAN</td><td>C9JQS9</td><td>Propionyl-CoA carboxylase beta chain, mitochondrial</td></tr>
<tr><td>1250</td><td>dirMS_041712_NAR_30mM_minus2.19133.19133.2</td><td>2</td><td>17.59</td><td>0</td><td>2.61E+05</td><td></td><td>(K)SYELPD(</td><td>(K)S Y E/L|P D G Q/V I\T I G N E/R(F)</td><td></td><td>158.88</td><td>895.9492</td><td>1790.892</td><td>-0.0008</td><td>-0.4</td><td>4.14</td><td>123097.1</td><td>5.83</td><td>HUMAN</td><td>A5A3E0</td><td>POTE ankyrin domain family member F</td></tr>
<tr><td>1251</td><td>dirMS_041712_NAR_50mM_minus2.13298.13470.3</td><td>3</td><td>17.59</td><td>1</td><td>5.24E+05</td><td></td><td>(K)ATLPSPI</td><td>(K)A/T|L/P/S/P D/K L/P G F K(M)</td><td></td><td>144.18</td><td>457.5912</td><td>1370.768</td><td>-0.0088</td><td>-6.4</td><td>8.64</td><td>118930.9</td><td>5.49</td><td>HUMAN</td><td>P22314</td><td>Ubiquitin-like modifier-activating enzyme 1</td></tr>
<tr><td>1252</td><td>dirMS_041712_NAR_20mM_plus.14109.14109.2</td><td>2</td><td>17.59</td><td>0</td><td>2.29E+05</td><td></td><td>(R)AALQELI</td><td>(R)A/A/L/Q/E|L L/S K(G)</td><td></td><td>168.83</td><td>486.789</td><td>972.572</td><td>-0.0017</td><td>-1.7</td><td>6.05</td><td>13798.9</td><td>10.17</td><td>HUMAN</td><td>P62851</td><td>40S ribosomal protein S25</td></tr>
<tr><td>1253</td><td>dirMS_041712_NAR_20mM_plus.9356.9356.2</td><td>2</td><td>17.59</td><td>0</td><td>1.86E+05</td><td></td><td>(K)SVTNED</td><td>(K)S V\T N E/D/V/T Q/E/E/L/G G/A K(T)</td><td></td><td>136</td><td>838.9014</td><td>1676.797</td><td>-0.0018</td><td>-1.1</td><td>4</td><td>61363</td><td>8.73</td><td>HUMAN</td><td>C9JQS9</td><td>Propionyl-CoA carboxylase beta chain, mitochondrial</td></tr>
<tr><td>1254</td><td>dirMS_041712_NAR_30mM_plus2.21350.21481.3</td><td>3</td><td>17.59</td><td>0</td><td>5.51E+05</td><td></td><td>(R)VNQAIW</td><td>(R)V N Q\A\I\W|L|L/C/T/G A R(E)</td><td>C:Carbamidomethylation</td><td>166.88</td><td>501.2717</td><td>1501.794</td><td>0.0062</td><td>4.1</td><td>9.72</td><td>23047.2</td><td>9.95</td><td>HUMAN</td><td>P46782</td><td>40S ribosomal protein S5</td></tr>
<tr><td>1255</td><td>dirMS_041712_NAR_40mM_plus2.12377.12710.3</td><td>3</td><td>17.59</td><td>0</td><td>4.54E+05</td><td></td><td>(K)AASDIAI</td><td>(K)A A|S/D|I/A/M/T E/L/P P T H/P I R(L)</td><td></td><td>140.07</td><td>607.3167</td><td>1819.937</td><td>-0.0015</td><td>-0.9</td><td>5.32</td><td>12926.1</td><td>4.4</td><td>HUMAN</td><td>K7EM20</td><td>14-3-3 protein epsilon (Fragment)</td></tr>
<tr><td>1256</td><td>dirMS_041712_NAR_30mM_minus2.7281.7281.2</td><td>2</td><td>17.58</td><td>0</td><td>2.62E+05</td><td></td><td>(K)DAQGQI</td><td>(K)D A|Q/G/Q|P/G L/E R(A)</td><td></td><td>93.63</td><td>535.762</td><td>1070.523</td><td>-0.0058</td><td>-5.4</td><td>4.37</td><td>253486.5</td><td>5.51</td><td>HUMAN</td><td>J3KNL6</td><td>Protein transport protein Sec16A</td></tr>
<tr><td>1257</td><td>dirMS_041712_NAR_30mM_minus2.7580.7616.2</td><td>2</td><td>17.58</td><td>0</td><td>2.78E+05</td><td></td><td>(K)ITITNDQ</td><td>(K)I/T|I/T|N D/Q/N R(L)</td><td></td><td>95.85</td><td>537.7798</td><td>1074.554</td><td>-0.0015</td><td>-1.4</td><td>5.84</td><td>72445.7</td><td>5.07</td><td>HUMAN</td><td>P11021</td><td>78 kDa glucose-regulated protein</td></tr>
<tr><td>1258</td><td>dirMS_041712_NAR_50mM_minus2.17906.17977.3</td><td>3</td><td>17.58</td><td>1</td><td>8.25E+05</td><td></td><td>(K)TFVNITP</td><td>(K)T F|V|N/I|T/P A/E V G V/L V G K D R(S)</td><td></td><td>171.32</td><td>639.0247</td><td>1915.065</td><td>-0.0052</td><td>-2.7</td><td>5.74</td><td>17630.6</td><td>11.62</td><td>HUMAN</td><td>I3L3D5</td><td>Profilin-1 (Fragment)</td></tr>
<tr><td>1259</td><td>dirMS_041712_NAR_30mM_plus2.15895.15895.2</td><td>2</td><td>17.58</td><td>0</td><td>3.21E+05</td><td></td><td>(R)YSVDIPL</td><td>(R)Y/S|V/D/I/P L/D/K(T)</td><td></td><td>136.13</td><td>525.2819</td><td>1049.551</td><td>0.0052</td><td>4.9</td><td>4.21</td><td>16471.9</td><td>10.59</td><td>HUMAN</td><td>K7ELC7</td><td>60S ribosomal protein L27 (Fragment)</td></tr>
<tr><td>1260</td><td>dirMS_041712_NAR_60mM_plus.7874.7918.3</td><td>3</td><td>17.58</td><td>0</td><td>7.33E+05</td><td></td><td>(K)YKPESEE</td><td>(K)Y K|P E S E/E|L|T/A/E/R(I)</td><td></td><td>99.3</td><td>484.5717</td><td>1451.701</td><td>-0.0007</td><td>-0.5</td><td>4.5</td><td>51524.1</td><td>4.82</td><td>HUMAN</td><td>F5H8J2</td><td>Protein disulfide-isomerase</td></tr>
<tr><td>1261</td><td>dirMS_041712_NAR_50mM_minus2.16333.16333.3</td><td>3</td><td>17.57</td><td>1</td><td>1.91E+05</td><td></td><td>(R)EGDVLT</td><td>(R)E G\D V/L/T/L L/E/S E R E/A R(R)</td><td></td><td>160.42</td><td>572.9609</td><td>1716.876</td><td>-0.0081</td><td>-4.7</td><td>4.25</td><td>7898</td><td>11.1</td><td>HUMAN</td><td>P62857</td><td>40S ribosomal protein S28</td></tr>
<tr><td>1262</td><td>dirMS_041712_NAR_50mM_minus2.9034.9112.3</td><td>3</td><td>17.57</td><td>1</td><td>1.91E+05</td><td></td><td>(R)VLTVIN(</td><td>(R)V/L|T|V/I/N Q/T Q K/E N L R(K)</td><td></td><td>119.83</td><td>552.6528</td><td>1655.944</td><td>0</td><td>0</td><td>8.72</td><td>10815.7</td><td>10.72</td><td>HUMAN</td><td>F2Z388</td><td>60S ribosomal protein L35</td></tr>
<tr><td>1263</td><td>dirMS_041712_NAR_120mM_plus.13203.13412.4</td><td>4</td><td>17.57</td><td>0</td><td>1.76E+06</td><td></td><td>(R)VLSAPPI</td><td>(R)V L|S A/P/P/H/F/H/F/G Q T N R(T)</td><td></td><td>123.07</td><td>427.7235</td><td>1707.871</td><td>0.0008</td><td>0.5</td><td>9.74</td><td>56491.1</td><td>7.78</td><td>HUMAN</td><td>B4DTG2</td><td>Elongation factor 1-gamma</td></tr>
<tr><td>1264</td><td>dirMS_041712_NAR_20mM_plus.15856.15856.2</td><td>2</td><td>17.57</td><td>0</td><td>2.93E+05</td><td></td><td>(R)GVVDSE</td><td>(R)G V/V/D/S E/D\I|P L/N L S R(E)</td><td></td><td>179.8</td><td>757.3956</td><td>1513.786</td><td>-0.0017</td><td>-1.1</td><td>4.03</td><td>74494.3</td><td>7.79</td><td>HUMAN</td><td>F5H897</td><td>Heat shock protein 75 kDa, mitochondrial</td></tr>
<tr><td>1265</td><td>dirMS_041712_NAR_20mM_plus.16480.16480.3</td><td>3</td><td>17.57</td><td>0</td><td>2.24E+05</td><td></td><td>(K)TVTNAV</td><td>(K)T V\T N A V V T V|P/A Y/F/N D S Q R(Q)</td><td></td><td>184.53</td><td>661.3368</td><td>1981.998</td><td>-0.0019</td><td>-1</td><td>5.5</td><td>17973</td><td>5.16</td><td>HUMAN</td><td>E9PI65</td><td>Heat shock cognate 71 kDa protein (Fragment)</td></tr>
<tr><td>1266</td><td>dirMS_041712_NAR_40mM_plus2.10703.10703.3</td><td>3</td><td>17.57</td><td>0</td><td>1.85E+05</td><td></td><td>(K)VGSLDN</td><td>(K)V/G/S L/D N V/G H/L/P A/G G A V K(I)</td><td></td><td>128.15</td><td>530.9567</td><td>1590.86</td><td>-0.0043</td><td>-2.7</td><td>6.71</td><td>26233.5</td><td>10.95</td><td>HUMAN</td><td>B4DSQ1</td><td>Microtubule-associated protein</td></tr>
<tr><td>1267</td><td>dirMS_041712_NAR_40mM_plus2.6202.6202.3</td><td>3</td><td>17.57</td><td>1</td><td>3.14E+04</td><td></td><td>(R)AQLGGF</td><td>(R)A Q|L/G G/P E/A A K\S D E T A/A/K(-)</td><td></td><td>95.22</td><td>548.6136</td><td>1643.824</td><td>0.0027</td><td>1.7</td><td>4.68</td><td>3888.3</td><td>5</td><td>HUMAN</td><td>C9J3N8</td><td>Heat shock protein beta-1</td></tr>
<tr><td>1268</td><td>dirMS_041712_NAR_60mM_plus.14353.14486.3</td><td>3</td><td>17.57</td><td>0</td><td>3.25E+06</td><td></td><td>(R)DLNYCF</td><td>(R)D L|N|Y/C/F/S/G M/S D/H R(Y)</td><td>C:Carbamidomethylation</td><td>132.4</td><td>534.5555</td><td>1601.647</td><td>0.0048</td><td>3</td><td>5.21</td><td>47371.6</td><td>5.93</td><td>HUMAN</td><td>E9PCY7</td><td>Heterogeneous nuclear ribonucleoprotein H</td></tr>
<tr><td>1269</td><td>dirMS_041712_NAR_60mM_minus.5118.5328.3</td><td>3</td><td>17.56</td><td>0</td><td>2.04E+06</td><td></td><td>(K)TATPQQ</td><td>(K)T/A|T|P Q/Q/A/Q E/V/H E K(L)</td><td></td><td>84.4</td><td>489.5763</td><td>1466.723</td><td>-0.009</td><td>-6.2</td><td>5.37</td><td>31075.7</td><td>5.65</td><td>HUMAN</td><td>P60174</td><td>Triosephosphate isomerase</td></tr>
<tr><td>1270</td><td>dirMS_041712_NAR_120mM_plus.10620.10763.4</td><td>4</td><td>17.56</td><td>2</td><td>5.03E+05</td><td></td><td>(K)SKGCGV</td><td>(K)S K G C G\V V\K F E\S|P/E|V/A/E/R(A)</td><td>C:Carbamidomethylation</td><td>109.53</td><td>470.4895</td><td>1878.938</td><td>-0.0017</td><td>-0.9</td><td>5.95</td><td>77799.7</td><td>9.09</td><td>HUMAN</td><td>P52272</td><td>Heterogeneous nuclear ribonucleoprotein M</td></tr>
<tr><td>1271</td><td>dirMS_041712_NAR_120mM_plus.8742.8945.2</td><td>2</td><td>17.56</td><td>0</td><td>6.12E+05</td><td></td><td>(R)NGVMP</td><td>(R)N/G|V|M|P/S H|F|S R(G)</td><td></td><td>99.97</td><td>566.274</td><td>1131.536</td><td>0.0043</td><td>3.8</td><td>9.75</td><td>16060.2</td><td>10.31</td><td>HUMAN</td><td>P39019</td><td>40S ribosomal protein S19</td></tr>
<tr><td>1272</td><td>dirMS_041712_NAR_30mM_plus2.8967.9004.2</td><td>2</td><td>17.56</td><td>0</td><td>5.36E+05</td><td></td><td>(K)QADVNI</td><td>(K)Q/A|D|V/N/L|V/N/A/K(L)</td><td></td><td>100.82</td><td>536.2922</td><td>1071.579</td><td>-0.0022</td><td>-2</td><td>5.84</td><td>57391.2</td><td>6.29</td><td>HUMAN</td><td>O43175</td><td>D-3-phosphoglycerate dehydrogenase</td></tr>
<tr><td>1273</td><td>dirMS_041712_NAR_50mM_plus2.15340.15500.3</td><td>3</td><td>17.56</td><td>1</td><td>8.72E+05</td><td></td><td>(K)EAFSLFD</td><td>(K)E/A|F/S/L/F/D/K D/G D/G T I/T T K(E)</td><td></td><td>149.53</td><td>615.6348</td><td>1844.891</td><td>-0.0014</td><td>-0.8</td><td>4.23</td><td>21860.1</td><td>4.45</td><td>HUMAN</td><td>E7EMB3</td><td>Calmodulin</td></tr>
<tr><td>1274</td><td>dirMS_041712_NAR_50mM_plus2.17772.17876.3</td><td>3</td><td>17.56</td><td>1</td><td>3.77E+05</td><td></td><td>(K)IEEIKDFL</td><td>(K)I/E|E|I K/D\F L/L/T A/R(R)</td><td></td><td>161.72</td><td>483.2728</td><td>1447.815</td><td>-0.0117</td><td>-8.1</td><td>4.68</td><td>2559</td><td>9.99</td><td>HUMAN</td><td>J3KSP2</td><td>60S ribosomal protein L38 (Fragment)</td></tr>
<tr><td>1275</td><td>dirMS_041712_NAR_50mM_plus2.17833.17976.3</td><td>3</td><td>17.56</td><td>0</td><td>2.47E+05</td><td></td><td>(R)ILVTLLH</td><td>(R)I/L|V/T/L/L/H/T L E/R(M)</td><td></td><td>162.4</td><td>436.6064</td><td>1307.805</td><td>0.0001</td><td>0.1</td><td>6.75</td><td>45212.4</td><td>9.41</td><td>HUMAN</td><td>B7Z233</td><td>Acetyl-CoA acetyltransferase, cytosolic</td></tr>
<tr><td>1276</td><td>dirMS_041712_NAR_50mM_plus2.18740.19159.3</td><td>3</td><td>17.56</td><td>0</td><td>9.39E+05</td><td></td><td>(R)CRPDQL</td><td>(R)C R P D\Q\L|T|G\L|S|L/L/P L/S E K(A)</td><td>C:Carbamidomethylation</td><td>167.58</td><td>643.0148</td><td>1927.032</td><td>-0.0019</td><td>-1</td><td>6.07</td><td>533778</td><td>5.74</td><td>HUMAN</td><td>Q15149</td><td>Plectin</td></tr>
<tr><td>1277</td><td>dirMS_041712_NAR_50mM_plus2.21391.21420.3</td><td>3</td><td>17.56</td><td>1</td><td>1.64E+05</td><td></td><td>(K)ALFEEVF</td><td>(K)A L/F/E|E/V/P E L L/T E A E K K(E)</td><td></td><td>182.58</td><td>615.9987</td><td>1845.984</td><td>-0.0029</td><td>-1.6</td><td>4.32</td><td>65036</td><td>6.27</td><td>HUMAN</td><td>E9PBU3</td><td>Phosphoribosylaminoimidazolecarboxamide formyltransferase</td></tr>
<tr><td>1278</td><td>dirMS_041712_NAR_30mM_minus2.11058.11058.2</td><td>2</td><td>17.55</td><td>0</td><td>9.13E+05</td><td></td><td>(K)SSFLVD(</td><td>(K)S S|F/L/V/D C/S K(A)</td><td>C:Carbamidomethylation</td><td>113.6</td><td>521.7502</td><td>1042.487</td><td>0.0058</td><td>5.5</td><td>5.55</td><td>258215.9</td><td>5.48</td><td>HUMAN</td><td>E7EN95</td><td>Filamin-B</td></tr>
<tr><td>1279</td><td>dirMS_041712_NAR_50mM_minus2.14815.14815.3</td><td>3</td><td>17.55</td><td>1</td><td>3.01E+05</td><td></td><td>(R)EAVTEIL</td><td>(R)E A|V/T E/I/L/G\I/E/P D R E K(G)</td><td></td><td>152.28</td><td>566.9683</td><td>1698.891</td><td>-0.0005</td><td>-0.3</td><td>4.25</td><td>25685.9</td><td>6.4</td><td>HUMAN</td><td>E9PGT1</td><td>Translin</td></tr>
<tr><td>1280</td><td>dirMS_041712_NAR_20mM_plus.15172.15172.2</td><td>2</td><td>17.55</td><td>0</td><td>3.01E+05</td><td></td><td>(K)LLCGLLA</td><td>(K)L/L|C G|L/L/A E/R(L)</td><td>C:Carbamidomethylation</td><td>176.75</td><td>522.7971</td><td>1044.587</td><td>-0.0001</td><td>-0.1</td><td>6</td><td>12647.2</td><td>8.07</td><td>HUMAN</td><td>P14174</td><td>Macrophage migration inhibitory factor</td></tr>
<tr><td>1281</td><td>dirMS_041712_NAR_30mM_plus2.9880.9904.2</td><td>2</td><td>17.55</td><td>0</td><td>4.03E+05</td><td></td><td>(K)ILGPQGI</td><td>(K)I/L|G|P/Q/G N T I/K(R)</td><td></td><td>105.27</td><td>520.808</td><td>1040.61</td><td>-0.0011</td><td>-1.1</td><td>8.75</td><td>48340.6</td><td>8.84</td><td>HUMAN</td><td>Q07666</td><td>KH domain-containing, RNA-binding, signal transduction-associated protein 1</td></tr>
<tr><td>1282</td><td>dirMS_041712_NAR_50mM_plus2.14373.14404.3</td><td>3</td><td>17.55</td><td>0</td><td>5.99E+05</td><td></td><td>(R)AVAIDLF</td><td>(R)A V|A/I/D/L/P G L G H S K(E)</td><td></td><td>143.8</td><td>426.5786</td><td>1277.721</td><td>0</td><td>0</td><td>6.79</td><td>19983.6</td><td>8.14</td><td>HUMAN</td><td>B4DQI4</td><td>Alpha/beta hydrolase domain-containing protein 14B</td></tr>
<tr><td>1283</td><td>dirMS_041712_NAR_50mM_plus2.20548.20549.3</td><td>3</td><td>17.55</td><td>1</td><td>1.87E+05</td><td></td><td>(K)SYLNMD</td><td>(K)S/Y|L/N/M D A/I/M E A I/K/K(T)</td><td></td><td>176.7</td><td>542.9473</td><td>1626.823</td><td>0.0044</td><td>2.7</td><td>5.79</td><td>80685.1</td><td>7.31</td><td>HUMAN</td><td>P05165</td><td>Propionyl-CoA carboxylase alpha chain, mitochondrial</td></tr>
<tr><td>1284</td><td>dirMS_041712_NAR_60mM_plus.12750.12750.3</td><td>3</td><td>17.55</td><td>1</td><td>1.03E+06</td><td></td><td>(R)DLDRIA(</td><td>(R)D/L/D R I\A/G|Q|V/A/A A N/K(K)</td><td></td><td>123.8</td><td>481.263</td><td>1441.776</td><td>-0.0013</td><td>-0.9</td><td>5.96</td><td>16060.2</td><td>10.31</td><td>HUMAN</td><td>P39019</td><td>40S ribosomal protein S19</td></tr>
<tr><td>1285</td><td>dirMS_041712_NAR_50mM_plus2.16578.16704.3</td><td>3</td><td>17.54</td><td>1</td><td>5.82E+05</td><td></td><td>(K)DANAKL</td><td>(K)D A/N/A\K L\S E\L|E|A|A/L/Q R(A)</td><td></td><td>155.78</td><td>543.6256</td><td>1628.86</td><td>0.002</td><td>1.2</td><td>4.68</td><td>56607.4</td><td>5.37</td><td>HUMAN</td><td>F8VXB4</td><td>Keratin, type II cytoskeletal 8</td></tr>
<tr><td>1286</td><td>dirMS_041712_NAR_50mM_minus2.5627.5676.3</td><td>3</td><td>17.53</td><td>1</td><td>3.66E+05</td><td></td><td>(K)DSYVGD</td><td>(K)D S|Y|V/G D/E/A Q\S K R(G)</td><td></td><td>92.95</td><td>452.2119</td><td>1354.623</td><td>-0.0022</td><td>-1.6</td><td>4.56</td><td>32616.5</td><td>5.18</td><td>HUMAN</td><td>A6NL76</td><td>Actin, alpha skeletal muscle</td></tr>
<tr><td>1287</td><td>dirMS_041712_NAR_120mM_plus.8270.8283.3</td><td>3</td><td>17.53</td><td>1</td><td>3.10E+05</td><td></td><td>(K)KYEEIDN</td><td>(K)K\Y/E E\I D|N|A/P/E/E/R(A)</td><td></td><td>96.95</td><td>498.2388</td><td>1492.691</td><td>0.0104</td><td>7</td><td>4.25</td><td>49883</td><td>7.3</td><td>HUMAN</td><td>P49411</td><td>Elongation factor Tu, mitochondrial</td></tr>
<tr><td>1288</td><td>dirMS_041712_NAR_30mM_plus2.6013.6034.2</td><td>2</td><td>17.53</td><td>0</td><td>1.16E+05</td><td></td><td>(K)VLENAE(</td><td>(K)V/L|E/N/A/E/G A R(T)</td><td></td><td>85.83</td><td>479.7542</td><td>958.495</td><td>0.0059</td><td>6.1</td><td>4.53</td><td>6809.6</td><td>9.69</td><td>HUMAN</td><td>D6RA73</td><td>Stress-70 protein, mitochondrial (Fragment)</td></tr>
<tr><td>1289</td><td>dirMS_041712_NAR_60mM_plus.14954.15128.3</td><td>3</td><td>17.53</td><td>0</td><td>5.61E+05</td><td></td><td>(R)YLDLHD(</td><td>(R)Y/L/D/L/H/D/C Y L/K(Y)</td><td>C:Carbamidomethylation</td><td>135</td><td>447.2169</td><td>1339.635</td><td>0.001</td><td>0.8</td><td>5.21</td><td>52796.2</td><td>5.19</td><td>HUMAN</td><td>E7EUT8</td><td>Splicing factor 3A subunit 3</td></tr>
<tr><td>1290</td><td>dirMS_041712_NAR_20mM_plus.14500.14500.2</td><td>2</td><td>17.52</td><td>0</td><td>5.73E+05</td><td>M185m</td><td>(K)LAVNm\</td><td>(K)L A|V/N/m V/P F P R(L)</td><td>m:Oxidized methionine</td><td>170.77</td><td>580.3169</td><td>1143.634</td><td>15.9922</td><td>-2.3</td><td>9.75</td><td>42230.8</td><td>4.77</td><td>HUMAN</td><td>A6NKZ8</td><td>Putative tubulin beta chain-like protein ENSP00000290377</td></tr>
<tr><td>1291</td><td>dirMS_041712_NAR_20mM_plus.15313.15716.2</td><td>2</td><td>17.52</td><td>0</td><td>1.28E+06</td><td>M73m</td><td>(R)AILVDLE</td><td>(R)A I|L|V|D|L|E|P/G T/m D S V R(S)</td><td>m:Oxidized methionine</td><td>179.22</td><td>816.4181</td><td>1615.836</td><td>15.9929</td><td>-1.2</td><td>4.03</td><td>27549.9</td><td>4.39</td><td>HUMAN</td><td>E9PBJ4</td><td>Tubulin beta chain</td></tr>
<tr><td>1292</td><td>dirMS_041712_NAR_30mM_plus2.7226.7226.2</td><td>2</td><td>17.52</td><td>0</td><td>1.83E+04</td><td></td><td>(R)SGGGG(</td><td>(R)S/G G G/G G/G|G/L/G/S\G/G S I R(S)</td><td></td><td>92.03</td><td>616.8023</td><td>1232.598</td><td>-0.0005</td><td>-0.4</td><td>9.47</td><td>62291.5</td><td>5.13</td><td>HUMAN</td><td>P35527</td><td>Keratin, type I cytoskeletal 9</td></tr>
<tr><td>1293</td><td>dirMS_041712_NAR_30mM_minus2.14015.14413.2</td><td>2</td><td>17.51</td><td>0</td><td>3.71E+06</td><td>M320m</td><td>(K)YmACCL</td><td>(K)Y/m|A|C/C/L/L|Y/R(G)</td><td>C:Carbamidomethylation m:Oxid</td><td>130.8</td><td>633.2775</td><td>1249.553</td><td>15.9951</td><td>0.1</td><td>8.59</td><td>50706.6</td><td>5.68</td><td>HUMAN</td><td>A6NHL2</td><td>Tubulin alpha chain-like 3</td></tr>
<tr><td>1294</td><td>dirMS_041712_NAR_50mM_minus2.10095.10179.3</td><td>3</td><td>17.51</td><td>0</td><td>5.46E+05</td><td>M147m</td><td>(K)LQmEAF</td><td>(K)L Q|m/E/A/P H|I|I/V/G T P G R(V)</td><td>m:Oxidized methionine</td><td>125.63</td><td>545.6267</td><td>1618.873</td><td>15.9922</td><td>-1.7</td><td>6.75</td><td>20859.5</td><td>4.85</td><td>HUMAN</td><td>J3KS25</td><td>Eukaryotic initiation factor 4A-I (Fragment)</td></tr>
<tr><td>1295</td><td>dirMS_041712_NAR_50mM_minus2.12869.13235.3</td><td>3</td><td>17.51</td><td>1</td><td>1.62E+06</td><td></td><td>(K)ALQQGL</td><td>(K)A/L|Q/Q/G L/V/G L/E/L K E K(L)</td><td></td><td>142.47</td><td>509.3022</td><td>1525.895</td><td>-0.0028</td><td>-1.8</td><td>6.18</td><td>266161</td><td>5.68</td><td>HUMAN</td><td>E9PPU0</td><td>Epiplakin</td></tr>
<tr><td>1296</td><td>dirMS_041712_NAR_50mM_minus2.14918.14999.3</td><td>3</td><td>17.51</td><td>0</td><td>4.48E+05</td><td></td><td>(R)LQQELD</td><td>(R)L Q|Q|E/L/D D L/T/V/D/L/D H Q\R(Q)</td><td></td><td>153.03</td><td>646.6559</td><td>1937.956</td><td>-0.0032</td><td>-1.6</td><td>4.03</td><td>233493.8</td><td>5.45</td><td>HUMAN</td><td>F8VTL3</td><td>Myosin-10</td></tr>
<tr><td>1297</td><td>dirMS_041712_NAR_50mM_minus2.15545.15566.3</td><td>3</td><td>17.51</td><td>0</td><td>1.90E+06</td><td></td><td>(R)VSLDVN</td><td>(R)V/S|L/D V/N H/F|A/P D E L T V K(T)</td><td></td><td>157.4</td><td>595.3104</td><td>1783.922</td><td>-0.0058</td><td>-3.3</td><td>4.54</td><td>20576.9</td><td>9.84</td><td>HUMAN</td><td>F8WE04</td><td>Heat shock protein beta-1</td></tr>
<tr><td>1298</td><td>dirMS_041712_NAR_120mM_plus.14690.15128.3</td><td>3</td><td>17.51</td><td>0</td><td>1.55E+06</td><td></td><td>(-)MVNHFI</td><td>(-)M/V/N/H/F/I/A E/F/K(R)</td><td></td><td>131.67</td><td>412.5446</td><td>1235.624</td><td>-0.0049</td><td>-4</td><td>6.5</td><td>45508.6</td><td>5.5</td><td>HUMAN</td><td>A8K7Q2</td><td>Heat shock cognate 71 kDa protein</td></tr>
<tr><td>1299</td><td>dirMS_041712_NAR_20mM_plus.8427.8427.2</td><td>2</td><td>17.51</td><td>0</td><td>9.13E+04</td><td></td><td>(K)VVTDTD</td><td>(K)V/V|T/D|T|D E T/E L A R(Q)</td><td></td><td>128.17</td><td>674.8318</td><td>1348.659</td><td>-0.0027</td><td>-2</td><td>3.91</td><td>36396.8</td><td>5.02</td><td>HUMAN</td><td>P05198</td><td>Eukaryotic translation initiation factor 2 subunit 1</td></tr>
<tr><td>1300</td><td>dirMS_041712_NAR_50mM_plus2.15903.15923.3</td><td>3</td><td>17.51</td><td>0</td><td>6.05E+05</td><td></td><td>(R)MVILLCI</td><td>(R)M/V|I/L/L/C N L/K/P A/K(M)</td><td>C:Carbamidomethylation</td><td>151.52</td><td>467.2775</td><td>1399.816</td><td>0.0016</td><td>1.1</td><td>10</td><td>34637.3</td><td>8.91</td><td>HUMAN</td><td>Q12904</td><td>Aminoacyl tRNA synthase complex-interacting multifunctional protein 1</td></tr>
<tr><td>1301</td><td>dirMS_041712_NAR_50mM_plus2.19425.19821.3</td><td>3</td><td>17.51</td><td>1</td><td>1.20E+06</td><td></td><td>(R)GDLGIEI</td><td>(R)G D/L|G|I|E/I/P A E K V F L A Q K(M)</td><td></td><td>171.73</td><td>610.0104</td><td>1828.022</td><td>-0.0048</td><td>-2.6</td><td>4.68</td><td>18466.1</td><td>5.78</td><td>HUMAN</td><td>H3BSU3</td><td>Pyruvate kinase (Fragment)</td></tr>
<tr><td>1302</td><td>dirMS_041712_NAR_20mM_minus.12442.12442.2</td><td>2</td><td>17.5</td><td>0</td><td>6.00E+05</td><td>M185m</td><td>(K)LAVNm\</td><td>(K)L A/V/N/m/V/P F/P R(L)</td><td>m:Oxidized methionine</td><td>172.52</td><td>580.3173</td><td>1143.634</td><td>15.993</td><td>-1.6</td><td>9.75</td><td>42230.8</td><td>4.77</td><td>HUMAN</td><td>A6NKZ8</td><td>Putative tubulin beta chain-like protein ENSP00000290377</td></tr>
<tr><td>1303</td><td>dirMS_041712_NAR_30mM_plus2.17134.17134.2</td><td>2</td><td>17.5</td><td>0</td><td>7.63E+05</td><td></td><td>(R)MNVLAI</td><td>(R)M/N|V/L/A\D A L/K(S)</td><td></td><td>143.1</td><td>487.7743</td><td>974.534</td><td>0.0074</td><td>7.6</td><td>5.59</td><td>27396</td><td>9.9</td><td>HUMAN</td><td>H3BN98</td><td>Uncharacterized protein (Fragment)</td></tr>
</table>

1304	dirMS_041712_NAR_30mM_plus2.19614.19614.2	2	17.5	0	2.87E+04		(K)NQALFP	(K)N/Q|A|L/F/P A C V L K(N)	C:Carbamidomethylation	157.75	630.8399	1260.677	-0.0044	-3.5	8.75	83400.6	6.82	HUMAN	Q92499	ATP-dependent RNA helicase DDX1
1305	dirMS_041712_NAR_30mM_plus2.7526.7541.2	2	17.5	0	6.31E+05		(K)SFQQSSI	(K)S F|Q|Q|S/S/L/S/R(D)		93.8	520.2659	1039.517	0.0078	7.5	9.47	100478.9	5.71	HUMAN	A8MXP9	Matrin-3
1306	dirMS_041712_NAR_50mM_plus2.14819.14819.3	3	17.5	0	6.51E+05		(K)SPAPPLL	(K)S/P|A/P/P L L H V A A L/G Q K(Q)		145.78	500.2911	1498.874	-0.0153	-10.2	8.49	17549.1	10.39	HUMAN	E5RFJ3	DBIRD complex subunit KIAA1967 (Fragment)
1307	dirMS_041712_NAR_60mM_plus.17727.17899.3	3	17.5	1	4.14E+05		(R)RDDGTG	(R)R D D G T G Q\L|L|L|P L/S D/A R(K)		150.38	576.3069	1726.908	-0.0021	-1.2	4.43	533778	5.74	HUMAN	Q15149	Plectin
1308	dirMS_041712_NAR_20mM_minus.14653.14653.2	2	17.49	0	2.10E+05		(K)LAVEALS	(K)L A V/E|A|L|S|S/L D G D L A/G R(Y)		201.28	793.922	1586.838	-0.0017	-1.1	4.03	24286.7	4.89	HUMAN	G3V4N7	Creatine kinase B-type (Fragment)
1309	dirMS_041712_NAR_20mM_minus.7656.7656.2	2	17.49	0	6.65E+05	M239m	(K)EVDEQm	(K)E V|D E Q/m L/N|V/Q|N|K(N)	m:Oxidized methionine	125.12	731.843	1446.689	15.9894	-3.8	4.14	40069.4	4.83	HUMAN	E7EWR1	Tubulin beta chain
1310	dirMS_041712_NAR_50mM_plus2.22224.22224.3	3	17.49	1	1.35E+06		(K)EGGLGP	(K)E G G/L/G/P L/N I/P L L A D V T R R(L)		191	631.029	1891.076	-0.0035	-1.9	6.17	15252.2	5.82	HUMAN	A6NIW5	Peroxiredoxin 2, isoform CRA_a
1311	dirMS_041712_NAR_60mM_plus.18891.19267.3	3	17.49	1	1.21E+06		(R)EGDVLT	(R)E/G|D|V/L/T/L/L/E/S E R E/A R(R)		157.1	572.9638	1716.876	0.0006	0.3	4.25	7898	11.1	HUMAN	P62857	40S ribosomal protein S28
1312	dirMS_041712_NAR_20mM_plus.10867.11339.2	2	17.48	0	2.99E+06		(K)DVNAAI.	(K)D V|N|A|A/I/A/T/I/K(T)		150.08	508.292	1015.578	-0.0015	-1.5	5.84	58642	4.95	HUMAN	F5H5D3	Tubulin alpha-1C chain
1313	dirMS_041712_NAR_30mM_plus2.7254.7350.2	2	17.48	0	4.29E+05		(R)IYVSANS	(R)I/Y|V|S/A/N/S/G A/R(I)		92.9	519.2668	1037.537	-0.0111	-10.7	8.75	26744.2	7.15	HUMAN	K7EK64	Acetyl-CoA carboxylase 1 (Fragment)
1314	dirMS_041712_NAR_50mM_plus2.9840.9840.3	3	17.48	1	1.04E+06		(K)LKGEAT\	(K)L K G E\A|T\V|S/F/D/D|P/P S A K(A)		119.12	554.6155	1661.838	-0.0061	-3.7	4.56	53724.2	9.54	HUMAN	H3BPE7	RNA-binding protein FUS
1315	dirMS_041712_NAR_20mM_minus.13136.13136.3	3	17.47	0	4.00E+05		(K)TITLEVEI	(K)T I T L E V/E|P/S/D T/I E/N V K(A)		183.03	596.6453	1787.927	-0.006	-3.3	4	17271.6	6.75	HUMAN	B4DV12	Ubiquitin
1316	dirMS_041712_NAR_120mM_plus.5512.5550.3	3	17.47	0	7.03E+05		(R)STFETEH	(R)S/T|F/E/T/E/H/A G Q R(D)		82.03	421.5315	1262.576	0.0039	3.1	5.38	160853.1	6.11	HUMAN	F8VU51	YLP motif-containing protein 1
1317	dirMS_041712_NAR_20mM_plus.14768.14768.2	2	17.47	0	1.10E+05		(K)LLGELLQ	(K)L/L|G/E\L/L/Q D N/A K(L)		173.65	607.3421	1213.679	-0.0018	-1.4	4.37	35556.4	9.3	HUMAN	F2Z393	Transaldolase
1318	dirMS_041712_NAR_30mM_plus2.18871.18912.2	2	17.47	0	2.41E+05		(R)LLFNDV(	(R)L/L|F/N/D V/Q/T L K(D)		152.38	595.8347	1190.678	-0.0158	-13.3	5.84	81007.1	6.31	HUMAN	E9PMV1	Plectin (Fragment)
1319	dirMS_041712_NAR_30mM_plus2.5977.5990.2	2	17.47	0	5.16E+04		(K)SYSVGA!	(K)S Y|S|V/G/A/S/G|S/S S/R(K)		85.65	572.7644	1144.523	-0.0014	-1.2	8.46	26261.9	9.78	HUMAN	F5GWE6	Cleavage and polyadenylation-specificity factor subunit 7
1320	dirMS_041712_NAR_30mM_plus2.7684.7752.2	2	17.47	0	8.54E+05		(K)GANPVE	(K)G A|N|P/V/E/I/R(R)		94.42	428.241	855.468	0.0064	7.5	6	17213.7	10.05	HUMAN	B7Z712	60 kDa heat shock protein, mitochondrial
1321	dirMS_041712_NAR_30mM_plus2.8708.8926.2	2	17.47	0	2.54E+06		(K)YIDQEEL	(K)Y/I|D|Q/E E L|N/K(T)		99.85	576.2833	1151.558	0.0014	1.2	4.14	85057.6	4.94	HUMAN	P07900	Heat shock protein HSP 90-alpha
1322	dirMS_041712_NAR_40mM_plus2.12275.12275.3	3	17.47	0	2.19E+05		(K)AASDIAI	(K)A A/S/D/I/A/M T E L/P P T H/P I R(L)		139.18	607.3167	1819.937	-0.0015	-0.9	5.32	12926.1	4.4	HUMAN	K7EM20	14-3-3 protein epsilon (Fragment)
1323	dirMS_041712_NAR_40mM_plus2.19946.20032.3	3	17.47	1	1.56E+06		(R)ELAILLG	(R)E L|A|I/L/L/G M L/D/P A E K D/E K(G)		189.1	629.003	1884.999	-0.0042	-2.3	4.18	25148.6	6	HUMAN	P30041	Peroxiredoxin-6
1324	dirMS_041712_NAR_50mM_plus2.11371.11477.3	3	17.47	1	1.25E+06		(K)NQVAM	(K)N Q|V|A/M/N|P T/N/T V/F D A K/R(L)		127.8	602.6354	1805.896	-0.0046	-2.6	8.75	20069.4	6.74	HUMAN	E9PK54	Heat shock cognate 71 kDa protein (Fragment)
1325	dirMS_041712_NAR_50mM_minus2.18793.18879.3	3	17.46	1	2.24E+06		(R)LIALLEVI	(R)L/I|A|L/L/E/V L/S Q K/R(M)		183.72	461.6289	1382.873	-0.0008	-0.6	8.75	280326.9	5.48	HUMAN	O75369	Filamin-B
1326	dirMS_041712_NAR_20mM_plus.12262.12262.2	2	17.46	0	4.61E+04		(K)VALSSET	(K)V A|L/S/S/E/T E/V/A/L/A/R(D)		157.62	673.3693	1345.732	-0.0008	-0.6	4.53	246695.2	5.91	HUMAN	E7EVA0	Microtubule-associated protein
1327	dirMS_041712_NAR_30mM_plus2.12946.12946.2	2	17.46	0	3.14E+05		(K)QQIQSIC	(K)Q Q|I|Q/S I Q/Q S/I E/R(L)		120.75	729.3893	1457.771	0.0006	0.4	6	14855.1	9.26	HUMAN	B1AHD1	NHP2 non-histone chromosome protein 2-like 1 (S. cerevisiae)
1328	dirMS_041712_NAR_50mM_plus2.8198.8198.3	3	17.46	0	7.80E+04		(K)QEYDES(	(K)Q E/Y/D E/S/G/P/S/I V H/R(K)		109.4	506.2382	1516.703	-0.0026	-1.7	4.65	123097.1	5.83	HUMAN	A5A3E0	POTE ankyrin domain family member F
1329	dirMS_041712_NAR_30mM_minus2.19123.19123.2	2	17.45	0	2.61E+05		(K)SYELPDC	(K)S Y E\L|P/D G Q V I/T I/G N E R(F)		158.88	895.9492	1790.892	-0.0008	-0.4	4.14	123097.1	5.83	HUMAN	A5A3E0	POTE ankyrin domain family member F
1330	dirMS_041712_NAR_60mM_minus.18441.18594.3	3	17.45	0	2.26E+06		(R)IHFPLAT	(R)I H F\P L A\T|Y|A|P/V/I/S A E K(A)		165.67	586.3248	1756.963	-0.0034	-1.9	6.75	49012.3	4.9	HUMAN	A8MUB1	Tubulin alpha-4A chain
1331	dirMS_041712_NAR_60mM_minus.5071.5276.3	3	17.45	0	3.20E+06		(R)AEAEAG	(R)A E|A|E|A/G/S P R P D|P/R(E)		83.35	451.5562	1352.655	-0.0013	-0.9	4.68	558007.1	5.44	HUMAN	P58107	Epiplakin
1332	dirMS_041712_NAR_30mM_plus2.15103.15448.2	2	17.45	0	9.39E+05		(R)VWQVTI	(R)V/W|Q|V/T/I/G T/R(-)		132.57	530.3002	1059.595	-0.0014	-1.4	9.72	30149.5	5.96	HUMAN	D6RAC2	Guanine nucleotide-binding protein subunit beta-2-like 1
1333	dirMS_041712_NAR_30mM_plus2.18383.18417.2	2	17.45	0	2.22E+05		(K)GVEGLIC	(K)G V|E/G|L|I/D/I/E/N/P\N R(V)		149.45	713.3669	1425.733	-0.0067	-4.7	4.14	20629.7	8.84	HUMAN	Q13442	28 kDa heat- and acid-stable phosphoprotein
1334	dirMS_041712_NAR_30mM_plus2.9098.9629.2	2	17.45	0	1.04E+06		(R)ILSGVVT	(R)I/L|S/G V/V/T/K(M)		102.98	408.7614	816.519	-0.0034	-4.2	8.75	18601.6	10.52	HUMAN	P62280	40S ribosomal protein S11
1335	dirMS_041712_NAR_50mM_plus2.16336.16568.3	3	17.45	0	7.81E+06		(R)VSLDVN	(R)V/S|L/D/V N/H/F/A/P D E L T V K(T)		154.68	595.312	1783.922	-0.001	-0.6	4.54	20576.9	9.84	HUMAN	F8WE04	Heat shock protein beta-1
1336	dirMS_041712_NAR_60mM_minus.14053.14216.3	3	17.44	1	6.76E+05		(R)LSAEERE	(R)L/S|A/E/E R D Q|L|L|P N L R(A)		138.75	551.9683	1653.892	-0.0015	-0.9	4.68	12056.4	6.28	HUMAN	P61457	Pterin-4-alpha-carbinolamine dehydratase
1337	dirMS_041712_NAR_60mM_minus.19901.19901.3	3	17.44	1	1.39E+06		(K)EIIDLVLE	(K)E I/I/D/L/V/L D R I/R(K)		180.2	452.2739	1354.805	0.0019	1.4	4.56	58642	4.95	HUMAN	F5H5D3	Tubulin alpha-1C chain
1338	dirMS_041712_NAR_40mM_plus2.19253.19253.3	3	17.44	1	1.93E+05		(K)TSAQVV	(K)T S/A/Q/V/V/L D G L/V D K I G D/V K(C)		180.37	619.6811	1857.033	-0.004	-2.2	4.43	227202.2	8.26	HUMAN	Q14008	Cytoskeleton-associated protein 5
1339	dirMS_041712_NAR_50mM_plus2.13659.13660.3	3	17.44	0	1.68E+06		(K)DLAGCII	(K)D L|A/G/C/I/H/G L S N V/K(L)	C:Carbamidomethylation	140.15	461.9061	1383.705	-0.0012	-0.8	6.74	45578.2	7.83	HUMAN	B4DFL2	Isocitrate dehydrogenase [NADP]
1340	dirMS_041712_NAR_60mM_plus.11692.11840.3	3	17.44	0	9.22E+06		(R)FAQHGT	(R)F A Q H G T\F\E|Y|E|Y/S Q R(W)		118.63	588.265	1762.782	-0.0015	-0.9	5.4	26419.2	5.1	HUMAN	H0Y9K7	Splicing factor, proline- and glutamine-rich (Fragment)
1341	dirMS_041712_NAR_30mM_plus2.18308.18308.2	2	17.43	0	3.70E+05		(R)SQVEEEI	(R)S Q|V/E/E E/L/F S/V R(V)		149.38	661.8302	1322.659	-0.0055	-4.2	4.25	533778	5.74	HUMAN	Q15149	Plectin
1342	dirMS_041712_NAR_30mM_plus2.22006.22006.2	2	17.43	0	2.06E+05		(K)QSLGELI	(K)Q S\L|G/E|L/I|G T L N A A/K(V)		170.27	707.8889	1414.79	-0.0195	-13.8	6	31075.7	5.65	HUMAN	P60174	Triosephosphate isomerase
1343	dirMS_041712_NAR_30mM_plus2.22226.22226.2	2	17.43	0	9.21E+05		(K)IGFPWSI	(K)I G/F/P/W/S E I/R(N)		173	552.8032	1104.584	0.0155	14	6	71218.8	6.3	HUMAN	A7YIJ8	Radixin
1344	dirMS_041712_NAR_30mM_minus2.13195.13346.2	2	17.42	0	7.20E+05		(K)YGVSGY	(K)Y/G|V|S|G/Y/P T\L/K(I)		125.7	542.7876	1084.567	0.0006	0.5	8.5	55361.8	6.42	HUMAN	G5EA52	Protein disulfide isomerase family A, member 3, isoform CRA_b
1345	dirMS_041712_NAR_40mM_minus2.12104.12438.3	3	17.42	1	7.28E+05		(R)IIAFVGS	(R)I/I|A\F/V|G/S/P V E D N E K D L V/K(L)		156.48	658.3553	1973.059	-0.0076	-3.9	4.32	21953.7	6.6	HUMAN	A6PVX3	26S proteasome non-ATPase regulatory subunit 4 (Fragment)
1346	dirMS_041712_NAR_40mM_minus2.14275.14275.3	3	17.42	0	1.23E+05		(R)GTEDITS	(R)G T|E/D I/T/S/P H G I/P L D\L L D/R(V)		168.55	650.3366	1948.997	-0.0022	-1.1	4.22	35067	5.55	HUMAN	E7ETR0	RuvB-like 1
1347	dirMS_041712_NAR_50mM_minus2.10711.10711.3	3	17.42	0	1.79E+05		(R)LLELGPK	(R)L/L|E/L/G P K P E/V/A Q Q\T R(K)		128.88	560.3182	1678.949	-0.0086	-5.1	6.14	139883.7	8.06	HUMAN	P53621	Coatomer subunit alpha
1348	dirMS_041712_NAR_60mM_minus.9552.9589.3	3	17.42	0	2.61E+05		(K)GIVEFSG	(K)G I|V/E/F/S/G K/P/A A R(K)		113.3	411.2274	1231.679	-0.0117	-9.5	8.75	23729.5	9.89	HUMAN	C9IZL7	Non-POU domain-containing octamer-binding protein (Fragment)
1349	dirMS_041712_NAR_120mM_plus.20482.21019.4	4	17.42	1	3.77E+06		(R)DYLHLPI	(R)D Y\L H/L|P|P|E I/V/P A T L R R(S)		162.23	473.2689	1890.06	-0.0058	-3.1	6.75	19924.8	9.99	HUMAN	F6U211	40S ribosomal protein S10
1350	dirMS_041712_NAR_30mM_plus2.13763.13885.2	2	17.42	0	6.06E+05		(R)ISGLIYEE	(R)I/S|G|L|I|Y/E E/T/R(G)		125.55	590.8136	1180.621	-0.0009	-0.8	4.53	11367.1	11.36	HUMAN	P62805	Histone H4
1351	dirMS_041712_NAR_50mM_plus2.17754.17764.3	3	17.42	0	2.72E+05		(R)LGIEGLS	(R)L/G|I|E/G L/S L/H N V L K(L)		161.6	464.946	1392.821	0.0025	1.8	6.75	177928	5.42	HUMAN	Q9NYU2	UDP-glucose:glycoprotein glucosyltransferase 1
1352	dirMS_041712_NAR_50mM_minus2.8808.8808.3	3	17.41	0	2.36E+05		(R)VMLGET	(R)V/M/L/G/E|T N P A D S K P G T I R(G)		118.3	595.9744	1785.916	-0.0077	-4.3	6.04	15431.7	9	HUMAN	E7ERL0	Nucleoside diphosphate kinase A
1353	dirMS_041712_NAR_120mM_plus.19557.19573.3	3	17.41	1	7.12E+05		(R)DFNHIN	(R)D F/N H I N\V|E|L/S|L/L/G K/K(K)		155.85	576.3192	1726.949	-0.0056	-3.2	6.75	20931.1	10.28	HUMAN	D6RAN4	60S ribosomal protein L9 (Fragment)
1354	dirMS_041712_NAR_20mM_plus.11117.11458.2	2	17.41	0	2.99E+06		(K)DVNAAI.	(K)D V|N|A|A/I/A/T I/K(T)		150.08	508.292	1015.578	-0.0015	-1.5	5.84	58642	4.95	HUMAN	F5H5D3	Tubulin alpha-1C chain
1355	dirMS_041712_NAR_30mM_plus2.14029.14164.2	2	17.41	0	5.04E+05		(R)VLSIGDC	(R)V/L|S|I/G/D/G/I A R(V)		126.68	500.7889	1000.579	-0.008	-8	5.81	15338.4	5.51	HUMAN	K7EJP1	ATP synthase subunit alpha, mitochondrial (Fragment)
1356	dirMS_041712_NAR_30mM_plus2.18586.18607.2	2	17.41	0	3.64E+05		(R)SSFYVN(	(R)S S|F|Y|V|N/G L/T\L/G G Q K(C)		150.97	735.8825	1470.759	-0.001	-0.7	8.31	17630.6	11.62	HUMAN	I3L3D5	Profilin-1 (Fragment)
1357	dirMS_041712_NAR_20mM_minus.11251.11251.2	2	17.4	0	2.79E+05		(K)LSELEAA	(K)L/S|E|L/E A/A/L/Q R(A)		159.5	565.3134	1129.621	-0.0016	-1.5	4.53	56607.4	5.37	HUMAN	F8VXB4	Keratin, type II cytoskeletal 8
1358	dirMS_041712_NAR_50mM_minus2.12364.12396.3	3	17.4	0	6.53E+05		(K)AGVVGF	(K)A/G/V|V|G/P/E/L/H E Q L/L S A E K(A)		139.25	592.9866	1776.949	-0.0038	-2.1	4.75	533778	5.74	HUMAN	Q15149	Plectin
1359	dirMS_041712_NAR_50mM_minus2.14553.14624.3	3	17.4	1	1.93E+05		(R)AEGAIA(	(R)A/E|G A|I/A/G F R D/P F S/G K(Q)		151.2	508.2599	1522.765	0.0003	0.2	6.12	266161	5.68	HUMAN	E9PPU0	Epiplakin
1360	dirMS_041712_NAR_30mM_plus2.20195.20703.2	2	17.4	0	7.48E+06		(R)YLTVAA\	(R)Y/L|T|V/A/A/V/F/R(G)		160.82	520.2996	1039.593	-0.0016	-1.5	8.75	40069.4	4.83	HUMAN	E7EWR1	Tubulin beta chain
1361	dirMS_041712_NAR_30mM_plus2.20465.20465.2	2	17.4	0	6.53E+04		(K)SYELPDC	(K)S Y\E|L|P D G Q V|I/T\I/G N E R(F)		160.92	895.9487	1790.892	-0.0018	-1	4.14	123097.1	5.83	HUMAN	A5A3E0	POTE ankyrin domain family member F
1362	dirMS_041712_NAR_40mM_plus2.15087.15525.3	3	17.4	0	9.12E+05		(R)DVQIGD	(R)D V|Q|I|G/D/I/V/T V/G E C R P L S K(T)	C:Carbamidomethylation	157.25	662.6815	1986.033	-0.0025	-1.3	4.56	18601.6	10.52	HUMAN	P62280	40S ribosomal protein S11
1363	dirMS_041712_NAR_50mM_minus2.16106.16106.3	3	17.39	0	1.46E+05		(K)LDPHLVI	(K)L D/P H L/V|L/D/Q L/R(C)		160.2	440.2533	1318.748	-0.0024	-1.8	5.21	233493.8	5.45	HUMAN	F8VTL3	Myosin-10
1364	dirMS_041712_NAR_120mM_plus.13671.14030.2	2	17.39	1	5.06E+06		(K)FASFIDK	(K)F/A|S|F/I/D/K V/R(F)		125.95	541.7966	1082.599	-0.0134	-12.4	8.75	19569.8	9.88	HUMAN	F8VRG4	Keratin, type II cytoskeletal 8 (Fragment)
1365	dirMS_041712_NAR_20mM_plus.17540.17540.2	2	17.39	0	3.36E+04		(R)YLTVAA\	(R)Y L|T/V/A/A/V F|R(G)		192.65	520.3009	1039.593	0.001	1	8.75	40069.4	4.83	HUMAN	E7EWR1	Tubulin beta chain
1366	dirMS_041712_NAR_30mM_plus2.10188.10573.2	2	17.39	0	8.70E+05		(R)SPSTLLP	(R)S/P|S/T/L/L|P/K(K)		107.97	421.7559	842.498	0.0063	7.5	8.47	75259.2	9.59	HUMAN	B5MEG9	Microtubule-associated protein 4 (Fragment)
1367	dirMS_041712_NAR_30mM_plus2.10801.10860.2	2	17.39	0	6.16E+05		(K)EAAENS	(K)E A|A/E/N/S L/V/A/Y/K(A)		110.12	597.8036	1194.6	-0.0002	-0.1	4.53	12926.1	4.4	HUMAN	K7EM20	14-3-3 protein epsilon (Fragment)
1368	dirMS_041712_NAR_30mM_plus2.19109.19109.2	2	17.39	0	3.47E+05		(R)LTAEDLF	(R)L/T/A/E/D L/F E/A R(I)		153.45	582.796	1164.59	-0.0048	-4.1	4.14	533778	5.74	HUMAN	Q15149	Plectin
1369	dirMS_041712_NAR_50mM_plus2.14766.15033.3	3	17.39	1	1.14E+05		(R)LLDAQL/	(R)L/L|D|A/Q/L/A/T/G G L V C P A R/R(L)	C:Carbamidomethylation	147.2	604.3351	1810.996	-0.0049	-2.7	9.6	266161	5.68	HUMAN	E9PPU0	Epiplakin
1370	dirMS_041712_NAR_50mM_plus2.18107.18552.3	3	17.39	1	1.01E+06		(K)GYPPED	(K)G Y|P/P E D I/F/N L/V/G K/K(V)		164.65	526.2834	1576.837	-0.0013	-0.8	6.07	40763.2	6.29	HUMAN	Q9NR45	Sialic acid synthase
1371	dirMS_041712_NAR_120mM_minus.10681.11169.3	3	17.38	0	9.56E+05		(R)GFCLPPI	(R)G F|C/L/P/P/H C S/R(G)	C:Carbamidomethylation	113.55	410.858	1230.551	0.0088	7.1	9.75	14391.8	4.99	HUMAN	G3V461	Creatine kinase B-type (Fragment)
1372	dirMS_041712_NAR_120mM_minus.14965.15189.3	3	17.38	1	1.12E+06		(K)VKEGMI	(K)V K E G M\N\I|V|E/A/M/E/R(F)		136.7	502.5853	1505.745	-0.0037	-2.5	4.79	18240.4	7.96	HUMAN	P62937	Peptidyl-prolyl cis-trans isomerase A
1373	dirMS_041712_NAR_120mM_plus.11134.11339.2	2	17.38	1	8.18E+05		(R)ALAAFLI	(R)A/L|A|A/F/L/K/K(S)		112.15	431.2797	861.556	-0.0035	-4.1	10	16060.2	10.31	HUMAN	P39019	40S ribosomal protein S19
1374	dirMS_041712_NAR_40mM_plus2.16140.16140.3	3	17.38	0	4.91E+05		(R)FGVPVI/	(R)F/G V|P/V/I/A D G G I Q N V/G H I A/K(A)		162.27	631.3436	1892.039	-0.0226	-12	6.74	51583.4	8.8	HUMAN	H0Y4R1	Inosine-5'-monophosphate dehydrogenase 2 (Fragment)
1375	dirMS_041712_NAR_20mM_minus.10885.10885.2	2	17.37	0	1.53E+06	M92m	(R)ImNTFS\	(R)I m|N\T/F/S/V V/P S P K(V)	m:Oxidized methionine	157.12	668.3513	1319.703	15.9925	-1.8	8.75	42831.5	4.86	HUMAN	A8K854	HCG1983504, isoform CRA_f
1376	dirMS_041712_NAR_50mM_minus2.7873.7873.3	3	17.37	0	4.58E+04		(R)QLSQAG	(R)Q/L/S/Q/A G/S F/S D G T H/G G L R(Y)		111.92	573.2875	1717.825	0.0227	13.2	6.74	266161	5.68	HUMAN	E9PPU0	Epiplakin
1377	dirMS_041712_NAR_30mM_plus2.15301.15301.2	2	17.37	0	2.82E+05		(R)LLCNFM	(R)L/L|C/N F M/R(Q)	C:Carbamidomethylation	132.92	477.2409	953.47	0.005	5.2	9.75	18685.5	9.45	HUMAN	B4DEV8	Proteasome subunit alpha type
1378	dirMS_041712_NAR_60mM_plus.18308.18783.3	3	17.37	1	2.81E+06		(K)QLDDLK'	(K)Q L|D/D/L/K/V/E/L/S/Q L R(V)		154.67	519.6254	1556.864	-0.0026	-1.7	4.56	10815.7	10.72	HUMAN	F2Z388	60S ribosomal protein L35
1379	dirMS_041712_NAR_30mM_minus2.10292.10373.2	2	17.36	0	2.81E+05		(R)ETAEAD'	(R)E T/A/E/A/D/V|A|S/L/N|R(R)		110.08	638.3134	1275.618	0.002	1.6	4.14	31923.6	4.85	HUMAN	B7Z596	Tropomyosin alpha-1 chain
1380	dirMS_041712_NAR_50mM_minus2.14836.14836.3	3	17.36	1	2.96E+05		(R)EAVTEIL	(R)E/A/V/T/E/I/L/G I/E P D R E K(G)		152.32	566.9683	1698.891	-0.0005	-0.3	4.25	25685.9	6.4	HUMAN	E9PGT1	Translin
1381	dirMS_041712_NAR_30mM_plus2.12579.12579.2	2	17.36	0	7.73E+05		(R)YSGSYNI	(R)Y/S|G S/Y N/D/Y L/R(A)		119.15	619.2776	1237.548	-0.0005	-0.4	5.83	69661.6	9.75	HUMAN	Q96PK6	RNA-binding protein 14
1382	dirMS_041712_NAR_30mM_plus2.17087.17087.2	2	17.36	0	1.82E+05		(K)GVVDSD	(K)G V\V/D S D/D/L/P L/N V/S R(E)		142.53	743.3807	1485.754	-0.0002	-0.2	3.93	92752.5	4.76	HUMAN	P14625	Endoplasmin
1383	dirMS_041712_NAR_30mM_plus2.6360.6387.2	2	17.36	0	2.52E+05		(K)GEGQLG	(K)G E|G\Q/L|G/P/A/E/R(A)		87.53	507.2537	1013.501	-0.0009	-0.9	4.53	96306.4	6.41	HUMAN	P13639	Elongation factor 2
1384	dirMS_041712_NAR_40mM_plus2.13442.13442.3	3	17.36	0	6.01E+05		(R)QVSASE	(R)Q\V/S/A S E L H T S G I/L/G/P E T L R(D)		145.08	665.6807	1995.051	-0.023	-11.5	5.4	558007.1	5.44	HUMAN	P58107	Epiplakin
1385	dirMS_041712_NAR_50mM_plus2.11416.11436.3	3	17.36	1	6.51E+05		(R)DVVICPI	(R)D V|V|I/C/P D A/S L E D A K K(E)	C:Carbamidomethylation	127.98	553.9467	1659.826	-0.0003	-0.2	4.23	18080.7	7.92	HUMAN	K7ELW0	Protein DJ-1
1386	dirMS_041712_NAR_30mM_minus2.11561.11685.2	2	17.35	0	8.84E+05		(K)CGDLVF.	(K)C/G/D|L/V|F|A/K(M)	C:Carbamidomethylation	117	455.2306	909.45	0.0041	4.5	5.84	26901.9	4.7	HUMAN	P51858	Hepatoma-derived growth factor
1387	dirMS_041712_NAR_120mM_plus.9999.9999.2	2	17.35	0	4.61E+05		(R)VHIEIGP	(R)V/H|I|E\I|G|P D G R(V)		105.95	546.7931	1092.58	-0.0007	-0.6	5.32	47371.6	5.93	HUMAN	E9PCY7	Heterogeneous nuclear ribonucleoprotein H
1388	dirMS_041712_NAR_50mM_minus2.11749.12017.3	3	17.34	1	6.11E+05		(R)ELISNSSI	(R)E L/I/S/N/S/S/D/A\L/D/K I R(Y)		135.85	520.9445	1560.823	-0.0038	-2.5	4.56	20151.2	4.58	HUMAN	G3V2J8	Heat shock protein HSP 90-alpha (Fragment)
1389	dirMS_041712_NAR_20mM_plus.13700.13805.2	2	17.34	0	6.17E+05		(R)GALQNI	(R)G A/L|Q|N|I|I\P A/S T G A/A/K(A)		167.23	706.3975	1411.79	-0.0026	-1.9	8.75	31718.5	7.17	HUMAN	E7EUT4	Glyceraldehyde-3-phosphate dehydrogenase
1390	dirMS_041712_NAR_30mM_plus2.10567.10805.2	2	17.34	0	3.23E+06		(R)GYSFTTT	(R)G Y|S/F/T/T/T/A E/R(E)		109.3	566.7674	1132.527	0.0006	0.5	6	23901.9	5.28	HUMAN	I3L1U9	Actin, cytoplasmic 2, N-terminally processed (Fragment)
1391	dirMS_041712_NAR_30mM_plus2.17717.17776.2	2	17.34	0	3.19E+05		(K)SLEDQV	(K)S L|E|D|Q/V/E/M/L/R(T)		145.85	610.3003	1219.599	-0.0054	-4.4	4.14	61161.1	4.34	HUMAN	K7ELL7	Glucosidase 2 subunit beta
1392	dirMS_041712_NAR_40mM_plus2.13862.13862.3	3	17.34	0	2.23E+05		(K)VALLLLD	(K)V A|L/L/L L/D Q/G A/S/P H A/A A K(N)		148.13	558.9886	1674.954	-0.0025	-1.5	6.71	482683.6	6.07	HUMAN	Q12955	Ankyrin-3
1393	dirMS_041712_NAR_40mM_plus2.15014.15435.3	3	17.34	0	4.51E+06		(R)VSLDVN	(R)V/S|L/D/V/N H/F/A/P D E L T V/K(T)		156.22	595.3161	1783.922	0.0113	6.3	4.54	20576.9	9.84	HUMAN	F8WE04	Heat shock protein beta-1
1394	dirMS_041712_NAR_30mM_minus2.18226.18226.2	2	17.33	0	7.01E+04		(R)SYNDEL(	(R)S Y/N/D E/L/Q F L/E\K(I)		153.58	693.3327	1385.658	-0.0002	-0.2	4.14	19338.9	5.33	HUMAN	E7EQS9	tRNA-splicing ligase RtcB homolog
1395	dirMS_041712_NAR_120mM_plus.21211.21211.4	4	17.33	1	9.54E+05		(R)DYLHLPI	(R)D Y L H L/P|P E\I/V/P A T L R R(S)		164.53	473.2689	1890.06	-0.0058	-3.1	6.75	19924.8	9.99	HUMAN	F6U211	40S ribosomal protein S10
1396	dirMS_041712_NAR_30mM_plus2.12181.12201.2	2	17.33	0	3.82E+05		(R)QYFEEIC	(R)Q/Y|F/E/E/I/Q/R(R)		117.02	556.7725	1112.537	0.0006	0.6	4.53	13246.4	9.21	HUMAN	Q5JY17	Nuclear receptor coactivator 5 (Fragment)
1397	dirMS_041712_NAR_30mM_plus2.16885.16914.2	2	17.33	0	1.67E+05		(K)EQFLDG	(K)E Q|F|L/D/G/D/G W/T/S R(W)		141.32	705.8178	1410.628	-0.0001	-0.1	4.03	28545.1	4.5	HUMAN	K7EJB9	Calreticulin (Fragment)
1398	dirMS_041712_NAR_50mM_plus2.8630.8630.3	3	17.33	1	1.17E+05		(K)TENSGE.	(K)T/E|N/S/G E A L A K V E D/S N/P Q K(T)		112.38	639.6441	1916.92	-0.0018	-1	4.41	13579.2	9.99	HUMAN	E9PPQ6	Thymocyte nuclear protein 1 (Fragment)
1399	dirMS_041712_NAR_120mM_minus.13497.13795.4	4	17.32	2	8.32E+05	M100m	(K)KQGGLG	(K)K\Q G G L\G\P m\N I|P L/V/S D/P K/R(T)	m:Oxidized methionine	128.55	481.5173	1907.053	15.9942	-0.3	9.99	22338.2	8.65	HUMAN	Q06830	Peroxiredoxin-1
1400	dirMS_041712_NAR_30mM_minus2.19188.19188.2	2	17.32	0	1.31E+06		(K)LAVNM\	(K)L A/V/N/M/V|P/F/P/R(L)		159.15	572.3143	1143.634	-0.013	-11.4	9.75	42230.8	4.77	HUMAN	A6NKZ8	Putative tubulin beta chain-like protein ENSP00000290377
1401	dirMS_041712_NAR_50mM_minus2.9216.9217.3	3	17.32	0	1.99E+05		(K)VGQEIE\	(K)V/G|Q/E/I/E V R P G/I V/S/K(D)		120.43	504.2902	1510.859	-0.0027	-1.8	6.11	51679.1	9.11	HUMAN	P41091	Eukaryotic translation initiation factor 2 subunit 3
1402	dirMS_041712_NAR_60mM_minus.9960.9960.3	3	17.32	1	1.21E+05		(K)IFQKGES	(K)I/F/Q\K G E S/P|V/D/Y/D G G R(T)		115.3	556.6051	1667.802	-0.0016	-1	4.56	53659.5	5.35	HUMAN	B5MCQ5	Protein disulfide-isomerase A6
1403	dirMS_041712_NAR_20mM_plus.10585.10585.3	3	17.32	0	8.25E+04		(K)VLQATV	(K)V L\Q A T V/V/A/V G S/G/S/K(G)		145.63	439.2573	1315.758	-0.0006	-0.5	8.72	10803.5	9.82	HUMAN	B8ZZL8	10 kDa heat shock protein, mitochondrial
1404	dirMS_041712_NAR_20mM_plus.8294.8307.2	2	17.32	0	9.35E+04		(R)LATNTS/	(R)L A|T|N/T/S A/P D/L K(N)		127.33	565.806	1130.605	-0.0005	-0.4	5.84	26233.5	10.95	HUMAN	B4DSQ1	Microtubule-associated protein
1405	dirMS_041712_NAR_30mM_plus2.17870.17870.2	2	17.32	0	1.40E+05		(R)NELSGA	(R)N/E|L|S/G/A/L T/G/L T R(V)		146.73	616.3341	1231.664	-0.0032	-2.6	6	71231.7	9.22	HUMAN	E7ERI3	Threonine--tRNA ligase, cytoplasmic
1406	dirMS_041712_NAR_30mM_minus2.14904.14904.2	2	17.31	0	1.75E+05		(K)SLAEGYI	(K)S L|A|E/G Y/F D A A/G R(L)		134.77	628.7988	1256.591	-0.0003	-0.2	4.37	22270.1	8.67	HUMAN	E9PHS0	LanC-like protein 1 (Fragment)
1407	dirMS_041712_NAR_30mM_minus2.18864.18864.2	2	17.31	0	1.06E+05		(K)NLIDAG\	(K)N L|I|D/A G V D/A L/R(V)		157.17	578.811	1156.632	-0.0173	-15	4.21	51583.4	8.8	HUMAN	H0Y4R1	Inosine-5'-monophosphate dehydrogenase 2 (Fragment)
1408	dirMS_041712_NAR_120mM_plus.13642.13935.3	3	17.31	1	7.43E+05		(R)IAWDDE	(R)I/A|W/D/D/E E/T R D/G F/R(L)		125.52	537.248	1609.724	0.0053	3.3	4.11	80685.1	7.31	HUMAN	P05165	Propionyl-CoA carboxylase alpha chain, mitochondrial
1409	dirMS_041712_NAR_30mM_plus2.16759.16759.2	2	17.31	0	2.91E+05		(R)SIQADG	(R)S/I/Q|A|D G L/V/W/G S S K(L)		140.63	674.3475	1347.69	-0.0026	-1.9	5.55	24934.4	4.5	HUMAN	P24534	Elongation factor 1-beta
1410	dirMS_041712_NAR_50mM_plus2.13207.13207.3	3	17.31	0	1.63E+06		(R)GIYAYGI	(R)G I/Y|A/Y/G F E K/P S A/I Q\Q R(A)		136.78	609.9821	1827.939	-0.0071	-3.9	8.5	16071.1	4.8	HUMAN	B4E102	Eukaryotic initiation factor 4A-I
1411	dirMS_041712_NAR_60mM_minus.12230.12230.3	3	17.3	1	4.75E+05		(R)INVYYNI	(R)I/N/V/Y/Y N E/A/T/G/G K/Y V P R(A)		127.67	615.3145	1843.934	-0.0048	-2.6	8.43	50286.6	4.79	HUMAN	P68371	Tubulin beta-4B chain
1412	dirMS_041712_NAR_60mM_minus.7326.7758.3	3	17.3	1	1.38E+06		(K)IQDKEGI	(K)I Q|D K E G|I|P/P/D Q Q R(L)		100.07	508.5978	1523.781	-0.0024	-1.6	4.56	17271.6	6.75	HUMAN	B4DV12	Ubiquitin

1413	dirMS_041712_NAR_30mM_plus2.18533.18948.2	2	17.3	0	2.82E+06		(R)GLFIIDG	(R)G/L|F|I I D/G/K(G)		151.62	431.7495	862.503	-0.0116	-13.4	5.84	22062.7	5.66	HUMAN	P32119	Peroxiredoxin-2
1414	dirMS_041712_NAR_30mM_plus2.8237.8669.2	2	17.3	0	1.48E+07		(R)AQYDEL	(R)A/Q|Y|D/E/L/A/R(K)		99.1	483.2388	965.469	0.0016	1.7	4.37	43773.3	5.26	HUMAN	F8VZY9	Keratin, type I cytoskeletal 18
1415	dirMS_041712_NAR_40mM_plus2.13984.13984.3	3	17.3	0	2.92E+05		(R)LASTLVH	(R)L A|S T L/V H L G E/Y Q/A/A/V D/G A R(K)		148.43	657.6841	1971.029	0.0083	4.2	5.32	71165.3	5.57	HUMAN	J3KS13	Clathrin heavy chain 1
1416	dirMS_041712_NAR_50mM_plus2.19128.19236.3	3	17.3	0	5.12E+05		(R)GFGFITF	(R)G F|G|F|I|T/F/T/N/P E H A S V A M R(A)		168.95	661.3258	1981.959	0.004	2	6.75	17170.1	8.86	HUMAN	P98179	Putative RNA-binding protein 3
1417	dirMS_041712_NAR_60mM_plus.18328.18659.3	3	17.3	1	8.26E+05		(R)IRIDSLS	(R)I R I D S\L\S\A|Q|L|S/Q L Q/K(Q)		154.22	567.3254	1699.97	-0.0085	-5	8.75	63136.9	5.83	HUMAN	D6RAQ3	Prelamin-A/C
1418	dirMS_041712_NAR_60mM_plus.7954.7954.3	3	17.3	0	1.02E+06		(K)EAYPDH	(K)E A/Y/P D/H/T/Q/F/E K(N)		99.42	455.5423	1364.612	0.0006	0.5	4.65	25868	9.48	HUMAN	Q9P016	Thymocyte nuclear protein 1
1419	dirMS_041712_NAR_30mM_minus2.15751.15902.2	2	17.29	0	8.09E+05		(K)ICSWNV	(K)I/C|S|W/N/V|D/G L/R(A)	C:Carbamidomethylation	139.38	610.2986	1219.589	0.0011	0.9	5.84	19395.5	5.38	HUMAN	G3V359	DNA-(apurinic or apyrimidinic site) lyase (Fragment)
1420	dirMS_041712_NAR_50mM_minus2.14943.15317.3	3	17.29	1	1.13E+06		(R)DVDFELI	(R)D V|D/F/E/L/I/K V E/G K(V)		154.02	464.5852	1391.742	-0.0006	-0.4	4.32	49982	5.86	HUMAN	B4DYD8	T-complex protein 1 subunit epsilon
1421	dirMS_041712_NAR_50mM_minus2.7913.7950.3	3	17.29	1	3.13E+05		(R)LQAEIEG	(R)L/Q|A/E/I/E/G L/K/G Q R(A)		112.3	447.921	1341.748	0	0	6.14	56607.4	5.37	HUMAN	F8VXB4	Keratin, type II cytoskeletal 8
1422	dirMS_041712_NAR_60mM_minus.12057.12275.3	3	17.29	1	4.75E+05		(R)INVYYNE	(R)I/N|V/Y/Y/N E/A|T/G G K Y/V P R(A)		127.67	615.3145	1843.934	-0.0048	-2.6	8.43	50286.6	4.79	HUMAN	P68371	Tubulin beta-4B chain
1423	dirMS_041712_NAR_120mM_plus.16800.16841.3	3	17.29	0	2.20E+06		(R)EHALLAY	(R)E\H\A\L\L|A|Y/T/L/G V/K(Q)		141.53	438.9167	1314.742	-0.0061	-4.6	6.85	50482.3	9.31	HUMAN	P68104	Elongation factor 1-alpha 1
1424	dirMS_041712_NAR_30mM_plus2.21678.21678.2	2	17.29	0	4.75E+04		(K)EIIDLVLD	(K)E I|I|D/L/V/L D/R(I)		168.75	543.3133	1085.62	-0.0008	-0.7	4.03	58642	4.95	HUMAN	F5H5D3	Tubulin alpha-1C chain
1425	dirMS_041712_NAR_50mM_plus2.10927.10927.3	3	17.29	0	6.36E+04		(K)MVMIQI	(K)M V|M/I/Q/D|G P Q N T G/A D K P L/R(I)		125.3	657.663	1970.979	-0.0042	-2.1	5.71	70034.8	6.89	HUMAN	B4DT31	Far upstream element-binding protein 1
1426	dirMS_041712_NAR_50mM_plus2.18436.18436.3	3	17.29	0	5.12E+05		(K)VHLVGID	(K)V H L V G\I/D|I/F/T/G/K(K)		165.02	433.5865	1298.747	-0.0018	-1.3	6.71	13048.7	6.42	HUMAN	C9J4W5	Eukaryotic translation initiation factor 5A-2 (Fragment)
1427	dirMS_041712_NAR_60mM_plus.12364.12632.3	3	17.29	1	3.75E+06		(R)LTPYEVD	(R)L/T|P/Y E/V D/E/L R/R(K)		121.9	464.2489	1390.733	-0.0004	-0.3	4.68	73202	9.19	HUMAN	C9JMU5	Probable ATP-dependent RNA helicase DDX17
1428	dirMS_041712_NAR_30mM_minus2.15860.16068.2	2	17.28	0	3.66E+05		(R)STLNEIY	(R)S T|L/N|E|I|Y/F/G K(T)		140.15	586.3085	1171.599	0.0104	8.8	5.72	29580	6.46	HUMAN	B1AK87	Capping protein (Actin filament) muscle Z-line, beta
1429	dirMS_041712_NAR_30mM_minus2.18966.19150.2	2	17.28	0	9.54E+05		(K)MSIYQA	(K)M/S|I/Y/Q/A/M/W/K(G)		158.93	579.2789	1157.548	0.0023	2	8.34	266161	5.68	HUMAN	E9PPU0	Epiplakin
1430	dirMS_041712_NAR_50mM_minus2.11008.11008.3	3	17.28	1	5.59E+05		(K)QQEAAL	(K)Q\Q/E/A/A L/E/L/E E L|K/K(K)		131.1	510.2775	1528.822	-0.0038	-2.5	4.49	54214.9	5.66	HUMAN	C9J813	Caldesmon (Fragment)
1431	dirMS_041712_NAR_50mM_minus2.13614.13614.3	3	17.28	1	5.22E+05		(K)LTEDKAI	(K)L T|E D K A/D V Q|S/I|I/G/L Q/R(F)		144.82	595.9939	1785.97	-0.0034	-1.9	4.56	90316.8	5.52	HUMAN	Q13263	Transcription intermediary factor 1-beta
1432	dirMS_041712_NAR_60mM_minus.15621.15621.3	3	17.28	1	5.92E+05	M81m	(K)EVNKFQ	(K)E V/N K F/Q m/A/Y/S/N/L L/R(A)	m:Oxidized methionine	146.73	576.9619	1712.879	15.9923	-1.5	8.69	14683.8	10.19	HUMAN	P37108	Signal recognition particle 14 kDa protein
1433	dirMS_041712_NAR_30mM_plus2.14122.14283.2	2	17.28	0	1.13E+04		(R)QLLEEEL	(R)Q/L|L|E/E/E/L/A/R(L)		128.93	550.8009	1100.595	-0.0001	-0.1	4.25	533778	5.74	HUMAN	Q15149	Plectin
1434	dirMS_041712_NAR_20mM_minus.13035.13270.3	3	17.27	0	1.85E+06	M73m	(R)AILVDLE	(R)A I\L\V D\L E/P/G T/m/D/S V R(S)	m:Oxidized methionine	182.72	544.6138	1615.836	15.9909	-2.5	4.03	27549.9	4.39	HUMAN	E9PBJ4	Tubulin beta chain
1435	dirMS_041712_NAR_30mM_minus2.11287.11611.2	2	17.27	0	1.61E+06		(K)FYEAFSK	(K)F/Y|E/A/F/S/K(N)		115.67	446.2172	891.425	0.0024	2.7	6	20127.1	9.4	HUMAN	H0Y598	Heat shock protein HSP 90-beta (Fragment)
1436	dirMS_041712_NAR_30mM_minus2.7027.7109.2	2	17.27	0	2.76E+05		(R)CGCLDE	(R)C/G|C|L/D/E/D/T Q/R(Q)	C:Carbamidomethylation	93.07	627.2485	1253.488	0.0012	1	4.03	266161	5.68	HUMAN	E9PPU0	Epiplakin
1437	dirMS_041712_NAR_60mM_minus.15430.15638.3	3	17.27	0	2.06E+06		(R)QIFHPEC	(R)Q I/F H/P E/Q/L/I/T/G/K(E)		146.53	470.9285	1410.774	-0.003	-2.1	6.75	27836	8	HUMAN	Q9H853	Putative tubulin-like protein alpha-4B
1438	dirMS_041712_NAR_20mM_plus.11282.11282.2	2	17.27	0	1.40E+05		(K)STGGAP	(K)S T/G/G/A|P/T F N V T V T K(T)		150.18	690.3592	1379.717	-0.0054	-3.9	8.47	11504.1	9.57	HUMAN	K7EJ44	Profilin 1, isoform CRA_b
1439	dirMS_041712_NAR_50mM_plus2.10003.10030.3	3	17.27	1	2.08E+05		(K)GVAINM	(K)G V/A|I/N\M/V/T E E D/K R(T)		119.98	487.9171	1461.737	0.0001	0.1	4.68	46381.4	5.32	HUMAN	P60842	Eukaryotic initiation factor 4A-I
1440	dirMS_041712_NAR_50mM_plus2.6083.6083.3	3	17.27	0	1.85E+05		(R)ATAPQT	(R)A T/A/P Q/T/Q H/V S/P M\R(Q)		96.3	475.2418	1423.711	-0.0002	-0.1	9.8	29184.1	4.91	HUMAN	E9PBQ9	Elongation factor 1-delta
1441	dirMS_041712_NAR_30mM_plus2.16095.16587.2	2	17.26	0	2.35E+06		(R)AALQELI	(R)A A|L|Q/E/L/L|S/K(G)		138.78	486.7894	972.572	-0.0009	-0.9	6.05	13798.9	10.17	HUMAN	P62851	40S ribosomal protein S25
1442	dirMS_041712_NAR_30mM_plus2.16980.17520.2	2	17.26	0	1.59E+06		(R)LFEGNA	(R)L/F|E|G/N/A/L/L/R(R)		143.53	516.7959	1032.584	0.0009	0.8	6	16684.5	11.19	HUMAN	B5MCT8	40S ribosomal protein S9
1443	dirMS_041712_NAR_30mM_plus2.22646.22646.2	2	17.26	0	8.66E+05		(R)IDLAVLL	(R)I/D|L/A/V L/L G K(T)		176.32	471.3029	941.603	-0.0045	-4.7	5.84	113668.7	6.2	HUMAN	F8W726	Ubiquitin-associated protein 2-like
1444	dirMS_041712_NAR_30mM_plus2.6200.6237.2	2	17.26	0	3.45E+05		(R)GAGSIA	(R)G A|G\S|I|A/G/A/S A S/P K(E)		86.83	537.2812	1073.559	-0.0034	-3.2	8.75	334221.3	6.44	HUMAN	P15924	Desmoplakin
1445	dirMS_041712_NAR_50mM_plus2.17779.17845.3	3	17.26	1	6.96E+05		(K)SLLGKD	(K)S/L|L/G K/D\V/L F/L/K(D)		161.7	411.5915	1232.761	-0.0013	-1.1	8.31	41827.5	7	HUMAN	B7Z7A9	Phosphoglycerate kinase
1446	dirMS_041712_NAR_50mM_plus2.18318.18553.3	3	17.26	0	5.12E+05		(K)VHLVGID	(K)V H\L\V\G\I|D|I/F/T/G K(K)		165.02	433.5865	1298.747	-0.0018	-1.3	6.71	13048.7	6.42	HUMAN	C9J4W5	Eukaryotic translation initiation factor 5A-2 (Fragment)
1447	dirMS_041712_NAR_60mM_plus.14437.14579.3	3	17.26	1	7.09E+05		(R)ELISNSSI	(R)E L|I/S/N/S/S D/A\L D/K I/R(Y)		132.23	520.952	1560.823	0.0187	12	4.56	20151.2	4.58	HUMAN	G3V2J8	Heat shock protein HSP 90-alpha (Fragment)
1448	dirMS_041712_NAR_50mM_minus2.15187.15187.3	3	17.25	0	8.93E+05		(R)LLIHQSL	(R)L/L|I H Q\S\L/A/G/G I/I/G V K(G)		154.82	506.9833	1518.937	-0.0013	-0.8	8.76	51260.6	5.39	HUMAN	P61978	Heterogeneous nuclear ribonucleoprotein K
1449	dirMS_041712_NAR_50mM_minus2.16425.16593.3	3	17.25	0	4.54E+05		(R)VLDPFTI	(R)V/L|D|P/F/T I K/P L D/R(K)		161.98	471.9411	1413.81	-0.0013	-0.9	5.93	63448.4	7.29	HUMAN	B4E022	Transketolase
1450	dirMS_041712_NAR_60mM_minus.13653.13718.3	3	17.25	0	4.50E+05		(R)ANPQVC	(R)A N|P Q/V/G V/A/F/P H I K(H)		135.95	459.9265	1377.764	0.0012	0.9	8.8	83273.6	5.55	HUMAN	P13010	X-ray repair cross-complementing protein 5
1451	dirMS_041712_NAR_60mM_minus.9362.9405.3	3	17.25	1	2.35E+05		(R)ILKEEQE	(R)I/L|K/E E Q/E|L/Y Q/K(E)		112.08	474.2636	1420.768	0.008	5.6	4.79	150202.8	9.1	HUMAN	E7EW20	Unconventional myosin-VI
1452	dirMS_041712_NAR_30mM_plus2.7595.7612.2	2	17.25	0	2.89E+05	M35m	(R)VEFmDD	(R)V/E|F/m/D/D/T/S R(S)	m:Oxidized methionine	94.03	558.2383	1099.473	15.9969	1.8	4.03	7898	11.1	HUMAN	P62857	40S ribosomal protein S28
1453	dirMS_041712_NAR_50mM_plus2.14700.14818.3	3	17.25	0	1.17E+06		(R)EHALLAY	(R)E H\A\L\L|A|Y/T/L/G/V/K(Q)		145.75	438.9176	1314.742	-0.0034	-2.6	6.85	50482.3	9.31	HUMAN	P68104	Elongation factor 1-alpha 1
1454	dirMS_041712_NAR_50mM_plus2.15502.15578.3	3	17.25	1	8.65E+05		(R)AITGASL	(R)A I|T/G A/S/L/A/D I/M A K/R(N)		150.37	473.2658	1417.783	-0.0003	-0.2	8.79	17656.4	11.01	HUMAN	C9JNW5	60S ribosomal protein L24
1455	dirMS_041712_NAR_30mM_minus2.12077.12580.2	2	17.24	0	4.46E+06		(R)EYQELM	(R)E Y|Q/E/L/M/N/V/K(L)		122.25	577.2825	1153.556	0.0019	1.7	4.53	56607.4	5.37	HUMAN	F8VXB4	Keratin, type II cytoskeletal 8
1456	dirMS_041712_NAR_50mM_minus2.16777.16777.3	3	17.24	0	2.38E+05		(R)LGIEGLS	(R)L/G/I/E/G L/S L/H N V L K(L)		164.13	464.9452	1392.821	0.0001	0.1	6.75	177928	5.42	HUMAN	Q9NYU2	UDP-glucose:glycoprotein glucosyltransferase 1
1457	dirMS_041712_NAR_120mM_plus.5716.5716.4	4	17.24	2	4.78E+05		(R)KTGQAP	(R)K\T G Q\A/P/G/Y/S Y/T/A A N K N K(G)		83.5	450.4826	1798.908	0.0003	0.2	9.83	11447.2	9.81	HUMAN	C9JFR7	Cytochrome c (Fragment)
1458	dirMS_041712_NAR_30mM_plus2.8721.8791.2	2	17.24	0	3.36E+05		(R)SLETENA	(R)S L|E|T/E/N/A/G L/R(L)		99.57	545.28	1089.554	-0.0008	-0.7	4.53	74423.5	6.57	HUMAN	P02545	Prelamin-A/C
1459	dirMS_041712_NAR_50mM_plus2.17774.17875.3	3	17.24	1	4.80E+05		(R)LSCLPAF	(R)L/S|C/L/P A F/K D/L I/A/K(V)	C:Carbamidomethylation	162.05	492.6138	1475.829	-0.0022	-1.5	8.59	535136.6	6.01	HUMAN	Q14204	Cytoplasmic dynein 1 heavy chain 1
1460	dirMS_041712_NAR_20mM_minus.9898.10122.2	2	17.23	0	3.42E+06		(K)DVNAAL	(K)D V|N|A|A|I/A/T I/K(T)		150.18	508.2919	1015.578	-0.0017	-1.7	5.84	58642	4.95	HUMAN	F5H5D3	Tubulin alpha-1C chain
1461	dirMS_041712_NAR_30mM_minus2.16814.17102.2	2	17.23	0	3.39E+05		(K)FANPFP	(K)F A|N|P|F|P A A V R(G)		146.38	545.2937	1089.584	-0.0039	-3.5	9.75	61363	8.73	HUMAN	C9JQS9	Propionyl-CoA carboxylase beta chain, mitochondrial
1462	dirMS_041712_NAR_30mM_minus2.17164.17626.2	2	17.23	0	1.82E+06		(K)AILFVPR	(K)A I|L|F|V/P/R(R)		149.63	408.2606	815.514	0.0001	0.2	9.79	57660.2	6.38	HUMAN	Q9NQH7	Probable Xaa-Pro aminopeptidase 3
1463	dirMS_041712_NAR_60mM_minus.18426.18828.3	3	17.23	0	2.38E+06		(R)IHFPLAT	(R)I H F\P L A\T|Y|A|P/V/I/S A E K(A)		165.73	586.3248	1756.963	-0.0034	-1.9	6.75	49012.3	4.9	HUMAN	A8MUB1	Tubulin alpha-4A chain
1464	dirMS_041712_NAR_60mM_minus.8473.8473.3	3	17.23	0	1.38E+04		(R)SAEEEA	(R)S/A/E/E\E/A A/D L/P T K/P T K(I)		107.37	529.6002	1586.791	-0.0048	-3	4.41	18924.5	6.86	HUMAN	Q8WW12	PEST proteolytic signal-containing nuclear protein
1465	dirMS_041712_NAR_20mM_plus.16418.16418.2	2	17.23	0	4.06E+05		(K)LGGSAV	(K)L G\G S A|V/I/S L/E/G K/P L(-)		185.42	670.8917	1340.778	-0.0023	-1.7	6	22956	8.8	HUMAN	E9PK25	Cofilin-1
1466	dirMS_041712_NAR_30mM_plus2.11898.11898.2	2	17.23	0	4.07E+05		(K)GEATVSI	(K)G E|A T\V/S/F/D D|P P S/A K(A)		115.65	710.8312	1420.659	-0.0039	-2.8	4.03	53724.2	9.54	HUMAN	H3BPE7	RNA-binding protein FUS
1467	dirMS_041712_NAR_30mM_plus2.6702.6820.2	2	17.23	0	5.39E+05		(K)LMSPTG	(K)L/M|S|P/T/G G/R(V)		90.03	409.7135	818.419	0.0008	1	9.75	141616.6	6.1	HUMAN	B7ZKM8	Protein transport protein Sec24B
1468	dirMS_041712_NAR_30mM_plus2.8471.8471.2	2	17.23	0	4.04E+05		(K)IANPVEC	(K)I/A|N/P|V/E/G S\T D R(Q)		97.95	579.7908	1158.575	-0.0006	-0.5	4.37	34267.8	9.05	HUMAN	B4DXP5	Poly(rC)-binding protein 2
1469	dirMS_041712_NAR_40mM_plus2.12812.12812.3	3	17.23	1	3.87E+05		(R)VLELVSI	(R)V L|E|L/V/S I T A N K N T\C P G D/R(S)	C:Carbamidomethylation	142.58	663.0201	1987.028	0.018	9.1	6.04	45186.1	9.89	HUMAN	B4DLV4	Serine hydroxymethyltransferase
1470	dirMS_041712_NAR_50mM_plus2.12512.12677.3	3	17.23	1	8.84E+05		(R)ELISNSSI	(R)E L|I/S/N/S|S/D A|L/D K I/R(Y)		134.15	520.9473	1560.823	0.0046	2.9	4.56	20151.2	4.58	HUMAN	G3V2J8	Heat shock protein HSP 90-alpha (Fragment)
1471	dirMS_041712_NAR_20mM_plus.18935.18935.2	2	17.22	0	1.05E+05		(K)DTSYLFI	(K)D T S/Y/L/F/I/T/G|P D V/V K(S)		201.92	777.9049	1554.805	-0.0025	-1.6	4.21	61363	8.73	HUMAN	C9JQS9	Propionyl-CoA carboxylase beta chain, mitochondrial
1472	dirMS_041712_NAR_50mM_plus2.16946.16946.3	3	17.22	0	7.80E+05		(R)QYYTLLN	(R)Q Y/Y/T/L L/N\Q A/P D\M L H/R(K)		156.93	621.6447	1862.922	-0.0022	-1.2	6.74	4335.1	9.82	HUMAN	E5RH00	Ras GTPase-activating protein-binding protein 1
1473	dirMS_041712_NAR_30mM_minus2.7745.7767.2	2	17.21	0	1.61E+05		(K)IFAQDGI	(K)I/F|A/Q/D|G E/G Q R(I)		96.53	560.7727	1120.538	0	0	4.37	258215.9	5.48	HUMAN	E7EN95	Filamin-B
1474	dirMS_041712_NAR_120mM_plus.17422.17422.3	3	17.21	2	7.46E+05		(R)NLKASLE	(R)N/L|K/A/S/L E N S L/R E V E|A R(Y)		143.9	610.332	1828.988	-0.0061	-3.3	6.23	43773.3	5.26	HUMAN	F8VZY9	Keratin, type I cytoskeletal 18
1475	dirMS_041712_NAR_50mM_plus2.21077.21077.3	3	17.21	0	3.77E+06		(R)AGKPVIC	(R)A G K\P V I\C\A/T/Q/M/L/E S M I K(K)	C:Carbamidomethylation	180.08	626.3278	1876.969	-0.0005	-0.3	8.64	18466.1	5.78	HUMAN	H3BSU3	Pyruvate kinase (Fragment)
1476	dirMS_041712_NAR_120mM_minus.4747.4747.3	3	17.2	1	3.96E+05		(K)AGADEE	(K)A/G/A/D/E E R A E T A/R(L)		76.8	425.8687	1275.592	-0.0008	-0.7	4.41	57204.9	6.01	HUMAN	F5GWF6	T-complex protein 1 subunit beta
1477	dirMS_041712_NAR_120mM_minus.7382.7401.3	3	17.2	1	3.19E+05		(K)IYEDGDI	(K)I/Y/E/D/G D/D D M/K/R(T)		94.63	452.8645	1356.574	0.0053	3.9	4.03	21341.9	7.77	HUMAN	Q5R370	Calcyclin binding protein
1478	dirMS_041712_NAR_50mM_minus2.10263.10325.3	3	17.2	0	2.72E+05		(R)TFIAIKPI	(R)T/F|I/A I/K/P/D/G V Q/R(G)		126.83	448.923	1344.763	-0.009	-6.7	8.41	6640.6	8.25	HUMAN	C9K028	Nucleoside diphosphate kinase A (Fragment)
1479	dirMS_041712_NAR_120mM_plus.9773.10358.3	3	17.2	0	9.16E+05		(R)GFCLPPI	(R)G F|C/L/P/P/H/C S/R(G)	C:Carbamidomethylation	104.65	410.8593	1230.551	0.0127	10.3	9.75	14391.8	4.99	HUMAN	G3V461	Creatine kinase B-type (Fragment)
1480	dirMS_041712_NAR_20mM_minus.12201.12201.2	2	17.19	0	1.07E+06	M313m	(R)TVQSLEI	(R)T V/Q/S L/E|I\D/L D/S/m R(N)	m:Oxidized methionine	170.83	761.8733	1506.747	15.9925	-1.6	4.03	43773.3	5.26	HUMAN	F8VZY9	Keratin, type I cytoskeletal 18
1481	dirMS_041712_NAR_60mM_minus.13292.13292.3	3	17.19	1	2.81E+05	M194m	(R)KLQEEEI	(R)K\L Q E E E\L\L\A/T|Q/V|D/m R(A)	m:Oxidized methionine	133.93	606.9793	1802.932	15.9917	-1.8	4.41	35935.5	6.2	HUMAN	Q8IVM0	Coiled-coil domain-containing protein 50
1482	dirMS_041712_NAR_20mM_plus.12068.12068.2	2	17.19	0	1.20E+05		(R)IYVVDVC	(R)I/Y/V|V/D|V/G S E/P R(A)		156.17	617.3237	1233.647	-0.0073	-5.9	4.37	39066	6.61	HUMAN	A6PVW9	Selenium binding protein 1 (Fragment)
1483	dirMS_041712_NAR_30mM_plus2.13816.13816.2	2	17.19	0	7.43E+05		(R)GDFCIQ	(R)G D/F/C/I/Q V|G/R(-)	C:Carbamidomethylation	125.57	526.2542	1051.499	0.0022	2.1	5.84	15431.7	9	HUMAN	E7ERL0	Nucleoside diphosphate kinase A
1484	dirMS_041712_NAR_30mM_plus2.14833.14833.2	2	17.19	0	3.81E+05		(K)VELDNM	(K)V/E|L|D/N|M/P L/R(G)		130.8	543.7824	1086.561	-0.0037	-3.4	4.37	23729.5	9.89	HUMAN	C9IZL7	Non-POU domain-containing octamer-binding protein (Fragment)
1485	dirMS_041712_NAR_50mM_plus2.16541.16922.3	3	17.19	1	6.78E+06		(K)QLDDLK	(K)Q/L/D/D/L/K/V/E/L/S/Q L R(V)		155.75	519.6261	1556.864	-0.0005	-0.3	4.56	10815.7	10.72	HUMAN	F2Z388	60S ribosomal protein L35
1486	dirMS_041712_NAR_120mM_minus.8157.8241.4	4	17.18	2	2.31E+06		(K)KGAVAE	(K)K\G A\V\A|E|D/G/D/E L R T E/P E/A/K(K)		99.28	479.4901	1914.94	-0.0018	-0.9	4.36	19284.3	5.23	HUMAN	G3V3C7	DNA-(apurinic or apyrimidinic site) lyase (Fragment)
1487	dirMS_041712_NAR_50mM_minus2.9278.9450.3	3	17.18	1	1.55E+06		(K)IQVLQQ	(K)I/Q|V|L|Q/Q/Q/A D D/A E E R A E R(L)		121.5	666.9985	1998.984	-0.003	-1.5	4.18	26611.3	4.47	HUMAN	A6NL28	Putative tropomyosin alpha-3 chain-like protein
1488	dirMS_041712_NAR_60mM_minus.7884.8120.3	3	17.18	1	8.24E+05		(R)LREDEN	(R)L R E D\E\N\A\E|P|V/G T/T/Y Q K(T)		104.68	617.3015	1849.893	-0.0027	-1.5	4.41	76925.9	4.46	HUMAN	A8MV58	Drebrin
1489	dirMS_041712_NAR_120mM_plus.18704.18836.3	3	17.18	1	2.43E+05		(K)SIYGEKF	(K)S I|Y|G/E K/F/E D/E/N F I L/K(H)		151.63	611.3079	1831.911	-0.0021	-1.2	4.41	13378.8	6.27	HUMAN	C9J5S7	Peptidyl-prolyl cis-trans isomerase
1490	dirMS_041712_NAR_20mM_plus.12650.12671.2	2	17.18	0	1.21E+05		(K)GTIQVIT	(K)G T/I/Q|V|I|T/Q/G T/S L K(N)		160.13	673.3872	1345.769	-0.0014	-1.1	8.75	57391.2	6.29	HUMAN	O43175	D-3-phosphoglycerate dehydrogenase
1491	dirMS_041712_NAR_30mM_plus2.22865.22902.2	2	17.18	0	1.84E+06		(R)GFGFVLI	(R)G/F|G|F/V/L/F/K(E)		179.85	457.76	914.514	-0.0007	-0.8	8.75	33782.7	8.78	HUMAN	B4DTC3	Heterogeneous nuclear ribonucleoprotein D0
1492	dirMS_041712_NAR_40mM_plus2.6824.6824.2	2	17.18	0	4.44E+04		(K)SFQQSSI	(K)S F|Q/Q/S/S/L\S/R(D)		100.17	520.2619	1039.517	-0.0002	-0.2	9.47	100478.9	5.71	HUMAN	A8MXP9	Matrin-3
1493	dirMS_041712_NAR_60mM_plus.12956.13016.3	3	17.18	1	1.55E+06	M72m	(R)TEmENE	(R)T|E|m/E N E|F/V L/I/K/K(D)	m:Oxidized methionine	124.62	499.594	1480.772	15.9959	0.6	4.78	13588.1	4.66	HUMAN	F8VP67	Keratin, type II cytoskeletal 8 (Fragment)
1494	dirMS_041712_NAR_60mM_minus.15787.15787.3	3	17.17	1	2.97E+05		(R)IDTRNEI	(R)I/D T R N E\L/E|S/Y/A Y S L K(N)		148.05	601.3049	1801.897	0.0035	1.9	4.68	72445.7	5.07	HUMAN	P11021	78 kDa glucose-regulated protein
1495	dirMS_041712_NAR_60mM_minus.8964.8964.2	2	17.17	0	9.67E+05		(K)QEYDESI	(K)Q|E\Y\D/E S/G\P/S/I/V/H/R(K)		110.35	758.8539	1516.703	-0.0021	-1.4	4.65	123097.1	5.83	HUMAN	A5A3E0	POTE ankyrin domain family member F
1496	dirMS_041712_NAR_50mM_plus2.10846.10846.3	3	17.17	1	2.46E+06		(R)MQKEIT	(R)M/Q/K E I T A|L/A|P S T M K(I)		124.03	516.9418	1548.812	-0.0016	-1	8.35	32616.5	5.18	HUMAN	A6NL76	Actin, alpha skeletal muscle
1497	dirMS_041712_NAR_120mM_minus.9268.9390.3	3	17.16	1	7.92E+05		(K)EFFNGKI	(K)E F/F N/G K/E/P/S/R(G)		105.4	404.2008	1210.585	0.0027	2.3	6.24	72445.7	5.07	HUMAN	P11021	78 kDa glucose-regulated protein
1498	dirMS_041712_NAR_20mM_minus.13142.13271.2	2	17.16	0	4.63E+05		(K)TITLEVEI	(K)T I|T|L/E/V|E|P S D T I E N V/K(A)		183.05	894.4654	1787.927	-0.0038	-2.1	4	17271.6	6.75	HUMAN	B4DV12	Ubiquitin
1499	dirMS_041712_NAR_30mM_minus2.20094.20246.2	2	17.16	0	1.53E+06		(R)VIFGLFG	(R)V/I|F/G/L/F/G/K(T)		170.93	440.768	880.529	-0.0004	-0.4	8.72	23799.1	9.48	HUMAN	P23284	Peptidyl-prolyl cis-trans isomerase B
1500	dirMS_041712_NAR_40mM_minus2.12208.12396.3	3	17.16	1	6.50E+05		(R)IIAFVGS	(R)I/I|A|F/V/G/S/P V E D N E K D L V K(L)		156.3	658.3553	1973.059	-0.0076	-3.9	4.32	21953.7	6.6	HUMAN	A6PVX3	26S proteasome non-ATPase regulatory subunit 4 (Fragment)
1501	dirMS_041712_NAR_50mM_minus2.18083.18083.3	3	17.16	1	1.52E+05		(R)GDLGIEI	(R)G D L/G/I|E\I/P A E K V F/L A Q K(M)		172.1	610.0112	1828.022	-0.0024	-1.3	4.68	18466.1	5.78	HUMAN	H3BSU3	Pyruvate kinase (Fragment)
1502	dirMS_041712_NAR_60mM_minus.14270.14528.3	3	17.16	1	5.52E+05		(K)VLAGDK	(K)V/L|A/G D K/N/F|I/T/A E E/L R(R)		140.2	559.3035	1675.901	-0.0054	-3.2	4.68	80254.2	5.05	HUMAN	D6PXK4	Alpha actinin 4 short isoform
1503	dirMS_041712_NAR_20mM_plus.14237.14237.2	2	17.16	0	3.41E+04		(R)IALTDNA	(R)I/A|L/T/D/N/A/L/I A R(S)		170.65	585.8432	1170.684	-0.005	-4.2	5.84	24489.6	10.74	HUMAN	A8MUD9	60S ribosomal protein L7
1504	dirMS_041712_NAR_40mM_plus2.17549.17711.3	3	17.16	1	5.62E+05		(R)GLLPGLI	(R)G L|L|P\G L/L/P\A/P A D K L T/P K(G)		170.58	567.6811	1701.031	-0.0022	-1.3	8.59	153084.9	5.55	HUMAN	Q6P2E9	Enhancer of mRNA-decapping protein 4
1505	dirMS_041712_NAR_50mM_minus2.12728.13107.3	3	17.15	0	4.17E+06	M84m	(K)YPIEHGI	(K)Y/P/I E H G I I|T|N|W D D m E K(I)	m:Oxidized methionine	141.65	659.6398	1960.911	15.9939	-0.5	4.31	32616.5	5.18	HUMAN	A6NL76	Actin, alpha skeletal muscle
1506	dirMS_041712_NAR_120mM_plus.13261.13261.3	3	17.15	2	6.46E+05		(R)ELNITAA	(R)E L|N/I/T A/A K E I E/V/G G G R K(A)		123.02	595.6661	1784.987	-0.0027	-1.5	6.33	21311.5	10.28	HUMAN	B5MCP9	40S ribosomal protein S7
1507	dirMS_041712_NAR_20mM_plus.16440.16440.2	2	17.15	0	1.40E+05		(R)QAQEYE	(R)Q A/Q/E/Y/E/A L|L/N|I\K(V)		184.52	710.3754	1419.748	-0.0043	-3	4.53	43773.3	5.26	HUMAN	F8VZY9	Keratin, type I cytoskeletal 18
1508	dirMS_041712_NAR_50mM_plus2.11538.11997.3	3	17.15	1	1.27E+06		(R)INVYYNE	(R)I/N|V|Y/Y|N E/A/T/G G K Y V/P R(A)		128.47	615.3151	1843.934	-0.003	-1.6	8.43	50286.6	4.79	HUMAN	P68371	Tubulin beta-4B chain
1509	dirMS_041712_NAR_60mM_plus.11875.11888.3	3	17.15	0	4.38E+05		(R)MVQVH	(R)M/V|Q|V/H/E|L/S C E/G I/S K(S)	C:Carbamidomethylation	119.42	539.5969	1616.777	-0.0009	-0.6	5.38	87447.6	6.44	HUMAN	Q15437	Protein transport protein Sec23B
1510	dirMS_041712_NAR_30mM_minus2.14901.15325.2	2	17.14	0	1.56E+06		(R)QITVND	(R)Q I|T|V|N/D/L/P V/G R(S)		135.22	606.3404	1211.674	-0.0007	-0.6	5.84	22062.7	5.66	HUMAN	P32119	Peroxiredoxin-2
1511	dirMS_041712_NAR_50mM_minus2.16685.16870.3	3	17.13	1	2.99E+05		(R)SELELTL	(R)S E|L|E/L/T/L/G K L/E Q V R(S)		163.72	538.9725	1614.906	-0.0032	-2	4.78	533778	5.74	HUMAN	Q15149	Plectin
1512	dirMS_041712_NAR_50mM_minus2.17260.17598.3	3	17.13	0	1.05E+06	M195m	(R)LHFFmP	(R)L H F\F m|P G|F|A|P/L/T S R(G)	m:Oxidized methionine	167.33	546.2872	1620.835	16.0115	10.1	9.75	42230.8	4.77	HUMAN	A6NKZ8	Putative tubulin beta chain-like protein ENSP00000290377
1513	dirMS_041712_NAR_20mM_plus.17481.17925.2	2	17.13	0	1.37E+06		(R)YLTVAA	(R)Y/L|T|V/A/A/V/F/R(G)		193.83	520.3009	1039.593	0.001	1	8.75	40069.4	4.83	HUMAN	E7EWR1	Tubulin beta chain
1514	dirMS_041712_NAR_20mM_plus.18779.18779.2	2	17.13	0	2.87E+05		(K)LAVEALS	(K)L A V E\A|L|S/S L/D G D\L A/G R(Y)		200.48	793.9214	1586.838	-0.0029	-1.8	4.03	24286.7	4.89	HUMAN	G3V4N7	Creatine kinase B-type (Fragment)
1515	dirMS_041712_NAR_30mM_plus2.16868.16868.2	2	17.13	0	1.67E+05		(K)EQFLDG	(K)E Q/F|L/D/G D/G/W T S/R(W)		141.32	705.8178	1410.628	-0.0001	-0.1	4.03	28545.1	4.5	HUMAN	K7EJB9	Calreticulin (Fragment)
1516	dirMS_041712_NAR_50mM_plus2.12861.13214.3	3	17.13	1	1.61E+06		(K)LIADVAF	(K)L/I|A/D/V/A/P S A I R E N D/I/K(S)		136.73	575.6533	1724.954	-0.0088	-5.1	4.56	18388.8	6.85	HUMAN	F5H1Y3	Flap endonuclease 1 (Fragment)
1517	dirMS_041712_NAR_50mM_minus2.15278.15278.3	3	17.12	1	8.56E+05		(K)ILLAELE	(K)I/L|L/A/E L/E Q L K/G Q G K(S)		155.77	513.9739	1539.911	-0.0033	-2.2	6.14	49709.6	5.19	HUMAN	B0YJC4	Vimentin
1518	dirMS_041712_NAR_120mM_minus.10518.10827.3	3	17.11	1	6.68E+05		(R)RGVVGF	(R)R G V\V\G|P/E|L/Y/G/R(L)		113.2	401.559	1202.664	-0.0016	-1.3	8.75	266161	5.68	HUMAN	E9PPU0	Epiplakin
1519	dirMS_041712_NAR_30mM_minus2.10368.10514.2	2	17.11	0	6.05E+05		(K)EAAENS	(K)E A|A|E/N/S L/V/A/Y/K(A)		110.8	597.8033	1194.6	-0.0008	-0.6	4.53	12926.1	4.4	HUMAN	K7EM20	14-3-3 protein epsilon (Fragment)
1520	dirMS_041712_NAR_30mM_minus2.14927.15110.2	2	17.11	0	9.43E+05		(K)LYLADPI	(K)L/Y|L|A|D|P/M/K(A)		135.13	475.7541	950.502	-0.0006	-0.7	5.83	7781.8	6.82	HUMAN	E9PE20	Signal recognition particle 9 kDa protein
1521	dirMS_041712_NAR_50mM_minus2.6740.6761.3	3	17.11	1	1.91E+05	M24m M2	(K)LAEQAE	(K)L/A|E|Q/A/E R Y D/D m/A/A/A/m K(A)	m:Oxidized methionine	104.88	615.6099	1812.825	31.9897	-0.1	4.32	28196	4.76	HUMAN	P31946	14-3-3 protein beta/alpha

1522	dirMS_041712_NAR_60mM_minus.9506.9506.3	3	17.11	0	3.19E+05		(R)QLSQAG	(R)Q L/S\Q/A/G/S/F S/D G T H G G L R(Y)		113.52	573.2781	1717.825	-0.0055	-3.2	6.74	266161	5.68	HUMAN	E9PPU0	Epiplakin
1523	dirMS_041712_NAR_20mM_plus.15561.15561.2	2	17.11	0	2.37E+05		(K)TITLEVEI	(K)T I/T L E/V|E|P S D T I E N V K(A)		179.03	894.4658	1787.927	-0.003	-1.7	4	17271.6	6.75	HUMAN	B4DV12	Ubiquitin
1524	dirMS_041712_NAR_40mM_plus2.13006.13088.3	3	17.11	0	2.26E+05		(R)VVLSVSS	(R)V V|L/S/V/S S G G Q/H T V/L/L/V K(D)		143.62	575.0089	1723.011	0.0009	0.5	8.73	45424.7	7.24	HUMAN	P18754	Regulator of chromosome condensation
1525	dirMS_041712_NAR_40mM_plus2.12422.12422.3	3	17.1	1	1.73E+05		(K)IINADSE	(K)I/I/N A/D/S/E D/P K Y I I N V K(Q)		139.07	611.3255	1831.98	-0.0181	-9.9	4.56	14693.3	4.69	HUMAN	C9JLS9	26S protease regulatory subunit 7 (Fragment)
1526	dirMS_041712_NAR_50mM_plus2.11362.11362.3	3	17.1	1	1.25E+06		(K)NQVAM	(K)N\Q|V|A\M/N P/T/N T V/F/D A K/R(L)		127.8	602.6354	1805.896	-0.0046	-2.6	8.75	20069.4	6.74	HUMAN	E9PK54	Heat shock cognate 71 kDa protein (Fragment)
1527	dirMS_041712_NAR_60mM_plus.12356.12356.3	3	17.1	1	7.68E+05		(K)YGQISE\	(K)Y/G\Q/I/S E V/V/V/V K/D/R(E)		121.22	497.9427	1491.817	-0.003	-2	6.07	28815.3	9.52	HUMAN	B4E2X2	Cold-inducible RNA-binding protein
1528	dirMS_041712_NAR_20mM_minus.10608.10694.2	2	17.09	0	2.00E+05		(R)TTPSYV/	(R)T T|P S/Y/V|A/F/T D/T E/R(L)		154.63	744.3547	1487.701	0.0009	0.6	4.37	58502.6	6.09	HUMAN	B4DXY3	Heat shock 70 kDa protein 1-like
1529	dirMS_041712_NAR_30mM_minus2.10248.10248.2	2	17.09	0	3.99E+05		(K)ATAVVD	(K)A T|A/V|V D G A\F K(E)		109.93	489.7645	978.525	-0.0037	-3.8	5.88	15252.2	5.82	HUMAN	A6NIW5	Peroxiredoxin 2, isoform CRA_a
1530	dirMS_041712_NAR_30mM_minus2.7601.7601.2	2	17.09	0	8.10E+04		(R)SDEGQL	(R)S\D|E/G Q/L/S|P A T/R(G)		95.82	580.7788	1160.554	-0.0039	-3.3	4.37	81007.1	6.31	HUMAN	E9PMV1	Plectin (Fragment)
1531	dirMS_041712_NAR_120mM_plus.19549.19708.4	4	17.09	1	3.15E+06		(R)DFNHIN	(R)D F N H\I N|V E|L/S/L/L/G K K(K)		155.95	432.4918	1726.949	-0.0033	-1.9	6.75	20931.1	10.28	HUMAN	D6RAN4	60S ribosomal protein L9 (Fragment)
1532	dirMS_041712_NAR_30mM_plus2.17525.17570.2	2	17.09	0	1.64E+05		(R)ASLEAAI	(R)A S/L/E/A|A|I/A|D/A E Q/R(G)		145.03	672.8406	1344.675	-0.0015	-1.1	4.14	56607.4	5.37	HUMAN	F8VXB4	Keratin, type II cytoskeletal 8
1533	dirMS_041712_NAR_50mM_plus2.12434.12434.3	3	17.09	0	2.04E+05		(K)AIIKPQY	(K)A I|I/K/P Q Y V D/Q I/P K(A)		133.23	504.9637	1512.878	-0.0019	-1.3	8.54	47949	9.16	HUMAN	D6R9I9	ATP-binding cassette sub-family E member 1
1534	dirMS_041712_NAR_50mM_plus2.19845.19985.3	3	17.09	1	5.31E+05		(-)MDELQL	(-)M/D|E|L/Q L/F/R/G D T V/L/L/K(G)		173.25	593.3209	1777.952	-0.0035	-2	4.56	18601	5.1	HUMAN	C9IZA5	Transitional endoplasmic reticulum ATPase (Fragment)
1535	dirMS_041712_NAR_60mM_plus.5351.5538.3	3	17.09	0	1.54E+06		(R)AEAEAG	(R)A/E|A|E/A/G S P R P D|P/R(E)		85.88	451.5555	1352.655	-0.0034	-2.5	4.68	558007.1	5.44	HUMAN	P58107	Epiplakin
1536	dirMS_041712_NAR_30mM_plus2.14311.14311.2	2	17.08	0	9.71E+05		(R)MGQM/	(R)M\G Q|M|A|M/G/G/A M/G I N N R(G)		127.33	769.8376	1538.67	-0.0015	-1	9.5	43979.5	8.92	HUMAN	F5GYZ3	Non-POU domain-containing octamer-binding protein
1537	dirMS_041712_NAR_30mM_plus2.15092.15374.2	2	17.08	0	9.84E+05		(K)LWNTLG	(K)L/W|N|T/L/G V/C/K(Y)	C:Carbamidomethylation	132.65	545.7923	1090.571	0.0059	5.5	8.75	33029.4	9.62	HUMAN	D6R9L0	Guanine nucleotide-binding protein subunit beta-2-like 1 (Fragment)
1538	dirMS_041712_NAR_50mM_plus2.18985.19262.3	3	17.08	0	5.88E+05		(R)GFGFITF	(R)G F|G|F|I/T/F/T N/P E H A S V A M R(A)		168.7	661.3258	1981.959	0.004	2	6.75	17170.1	8.86	HUMAN	P98179	Putative RNA-binding protein 3
1539	dirMS_041712_NAR_20mM_minus.12249.12487.3	3	17.07	0	8.33E+05	M73m	(R)AVLVDLI	(R)A V|L\V D L E/P/G/T/m/D/S V R(S)	m:Oxidized methionine	172.57	539.9423	1601.82	15.992	-1.8	4.03	13112.4	5.23	HUMAN	F8W813	Tubulin beta-8 chain
1540	dirMS_041712_NAR_50mM_minus2.16032.16300.3	3	17.07	1	4.43E+06		(R)VSFELFA	(R)V/S|F/E/L/F/A D K V/P K(T)		159.72	460.5908	1379.757	0.0009	0.7	6.04	13378.8	6.27	HUMAN	C9J5S7	Peptidyl-prolyl cis-trans isomerase
1541	dirMS_041712_NAR_60mM_minus.6036.6047.3	3	17.07	1	2.71E+06		(K)DSYVGD	(K)D S/Y V/G/D/E/A/Q/S K R(G)		90.57	452.2129	1354.623	0.0008	0.6	4.56	32616.5	5.18	HUMAN	A6NL76	Actin, alpha skeletal muscle
1542	dirMS_041712_NAR_20mM_plus.18352.18352.2	2	17.07	0	1.95E+05		(R)AAVEEG	(R)A A V E E\G\I|V|L|G|G G C A L/L/R(C)	C:Carbamidomethylation	197.9	842.9549	1684.905	-0.0025	-1.5	4.53	61224.7	5.7	HUMAN	P10809	60 kDa heat shock protein, mitochondrial
1543	dirMS_041712_NAR_30mM_plus2.17036.17145.2	2	17.07	0	3.76E+04	M185m	(K)LAVNm\	(K)L/A|V|N/m|V/P F P R(L)	m:Oxidized methionine	142.12	580.3176	1143.634	15.9936	-1.1	9.75	42230.8	4.77	HUMAN	A6NKZ8	Putative tubulin beta chain-like protein ENSP00000290377
1544	dirMS_041712_NAR_50mM_minus2.14186.14245.3	3	17.06	0	2.61E+05		(R)TIQVENS	(R)T I|Q|V/E N\S H L/I/L/T/G A G A L N K(V)		149.02	660.367	1979.092	-0.0056	-2.8	6.41	267260.7	5.95	HUMAN	Q13085	Acetyl-CoA carboxylase 1
1545	dirMS_041712_NAR_50mM_minus2.8713.8713.3	3	17.06	1	2.14E+05		(R)VAIEPG/	(R)V/A|I|E/P/G A P R E/N D E F C K(M)	C:Carbamidomethylation	117.37	611.2923	1831.864	-0.002	-1.1	4.41	36856.1	8.55	HUMAN	H0YLA4	Sorbitol dehydrogenase
1546	dirMS_041712_NAR_60mM_minus.16774.16774.3	3	17.06	1	2.85E+05		(R)GQFSTD	(R)G Q|F/S T D E L/V/A/E V E/K R(N)		153.92	569.9569	1707.855	0.0013	0.8	4.41	144109.9	6.01	HUMAN	J3KR87	Clathrin heavy chain 2
1547	dirMS_041712_NAR_30mM_plus2.6894.6894.2	2	17.06	0	7.31E+04		(R)ENPYYD	(R)E N|P/Y/Y/D/S R(V)		90.4	522.2255	1043.443	0.0009	0.8	4.37	193379.8	5.48	HUMAN	Q00610	Clathrin heavy chain 1
1548	dirMS_041712_NAR_20mM_minus.13984.13984.2	2	17.05	0	6.81E+05		(R)VLITTDL	(R)V L|I/T|T D/L/L A R(G)		192.22	557.8438	1114.683	-0.0027	-2.4	5.81	41517.9	5.45	HUMAN	E7EQG2	Eukaryotic initiation factor 4A-II
1549	dirMS_041712_NAR_30mM_plus2.19782.19782.2	2	17.05	0	3.53E+05		(K)SNNVEN	(K)S N/N/V|E\M D W/V L\K(H)		156.98	667.8205	1334.641	-0.0072	-5.4	4.37	18511.8	5.62	HUMAN	D6R9T0	Heterogeneous nuclear ribonucleoprotein H (Fragment)
1550	dirMS_041712_NAR_40mM_plus2.17732.17732.3	3	17.05	1	1.96E+05		(R)LKLPSIPI	(R)L K\L P\S I\P L|V|P V/S A Q K(R)		170.73	530.671	1589.999	-0.0004	-0.3	10	34958.1	9.42	HUMAN	Q15785	Mitochondrial import receptor subunit TOM34
1551	dirMS_041712_NAR_60mM_plus.13079.13542.3	3	17.05	1	8.86E+05		(R)VIDRELY	(R)V/I|D R E L|Y\Q|Q/L/Q/R(G)		125.78	520.952	1560.849	-0.0078	-5	6.04	533778	5.74	HUMAN	Q15149	Plectin
1552	dirMS_041712_NAR_20mM_minus.12216.12552.3	3	17.04	0	8.33E+05	M73m	(R)AVLVDLI	(R)A V\L\V D\L E/P/G T|m/D S V R(S)	m:Oxidized methionine	172.57	539.9423	1601.82	15.992	-1.8	4.03	13112.4	5.23	HUMAN	F8W813	Tubulin beta-8 chain
1553	dirMS_041712_NAR_50mM_minus2.14263.14263.3	3	17.04	1	6.62E+05		(R)VEKIPGC	(R)V E/K\I\P G G\I|I|E/D/S/C V|L/R(G)	C:Carbamidomethylation	149.1	595.6534	1784.958	-0.0118	-6.6	4.68	56186.7	5.47	HUMAN	B4DUR8	T-complex protein 1 subunit gamma
1554	dirMS_041712_NAR_60mM_minus.8231.8231.3	3	17.04	1	1.42E+05		(R)LSQIGVE	(R)L/S/Q/I/G V/E N/T E E N R R(L)		106.07	548.9481	1644.83	-0.0002	-0.1	4.79	36636.9	7.84	HUMAN	A8MVZ9	Fructose-bisphosphate aldolase
1555	dirMS_041712_NAR_30mM_plus2.19187.19670.2	2	17.04	0	4.11E+06		(K)TLGILGL	(K)T/L|G|I|L|G/L|G R(I)		156.62	450.2857	899.567	-0.0032	-3.5	9.41	57391.2	6.29	HUMAN	O43175	D-3-phosphoglycerate dehydrogenase
1556	dirMS_041712_NAR_30mM_minus2.13847.13890.2	2	17.03	0	3.08E+05	M313m	(K)NSLESY/	(K)N S|L|E|S/Y/A/F/N m K(A)	m:Oxidized methionine	128.9	660.3011	1303.599	15.9962	1	6	45508.6	5.5	HUMAN	A8K7Q2	Heat shock cognate 71 kDa protein
1557	dirMS_041712_NAR_40mM_minus2.12336.12461.3	3	17.03	1	2.87E+05		(K)DVAIKEF	(K)D V|A|I|K E/P\L|V/D V|V D/P K(Q)		156.13	546.3068	1636.916	-0.0098	-6	4.23	24376.8	6.08	HUMAN	E9PIX6	Protein arginine N-methyltransferase 1 (Fragment)
1558	dirMS_041712_NAR_120mM_plus.20505.21040.4	4	17.03	1	3.77E+06		(R)DYLHLPI	(R)D Y\L H|L\P|P/E\I/V/P A T L R R(S)		162.23	473.2689	1890.06	-0.0058	-3.1	6.75	19924.8	9.99	HUMAN	F6U211	40S ribosomal protein S10
1559	dirMS_041712_NAR_50mM_plus2.14636.14797.3	3	17.03	1	4.12E+06		(R)ELISNAS	(R)E L|I/S/N/A/S|D|A L/D K I R(L)		145.58	515.6136	1544.828	-0.0016	-1	4.56	5582.4	4.81	HUMAN	F8W026	Endoplasmin (Fragment)
1560	dirMS_041712_NAR_60mM_plus.15773.16290.3	3	17.03	1	3.36E+06		(R)MSMKE'	(R)M/S M K E V\D\E\Q|M|L/N/V/Q/N K(N)		141.48	641.9696	1923.897	-0.003	-1.6	4.68	40069.4	4.83	HUMAN	E7EWR1	Tubulin beta chain
1561	dirMS_041712_NAR_30mM_minus2.14863.14863.2	2	17.02	0	2.59E+04		(R)LSLYQAI	(R)L/S|L/Y/Q A/M/R(Q)		134.33	491.2611	981.519	-0.0037	-3.8	8.75	266161	5.68	HUMAN	E9PPU0	Epiplakin
1562	dirMS_041712_NAR_50mM_minus2.10450.10963.3	3	17.02	1	7.18E+06	M72m	(R)TEmENE	(R)T\E|m/E N E|F/V L/I/K/K(D)	m:Oxidized methionine	129.07	499.5937	1480.772	15.995	0	4.78	13588.1	4.66	HUMAN	F8VP67	Keratin, type II cytoskeletal 8 (Fragment)
1563	dirMS_041712_NAR_50mM_minus2.15952.15952.3	3	17.02	1	8.21E+04		(K)DANAKL	(K)D A/N/A K L\S E\L|E/A/A|L/Q R(A)		158.97	543.6248	1628.86	-0.0004	-0.2	4.68	56607.4	5.37	HUMAN	F8VXB4	Keratin, type II cytoskeletal 8
1564	dirMS_041712_NAR_50mM_minus2.16991.16991.3	3	17.02	1	3.18E+05		(K)AILILDNI	(K)A I|L/I/L/D/N D/G D R L F A K(Y)		165.27	558.6442	1673.922	-0.004	-2.4	4.43	18430.9	4.79	HUMAN	F8VS17	Coatomer subunit zeta-1
1565	dirMS_041712_NAR_60mM_minus.9237.9237.3	3	17.02	0	2.84E+05		(K)ATAGDT	(K)A/T|A/G D/T/H|L G G E D/F D N R(L)		111.4	559.2488	1675.731	0.0012	0.7	4.22	58502.6	6.09	HUMAN	B4DXY3	Heat shock 70 kDa protein 1-like
1566	dirMS_041712_NAR_30mM_plus2.13940.14058.2	2	17.02	0	8.38E+05		(R)AFFSEVE	(R)A/F|F|S/E/V/E/R(R)		126.17	492.7436	984.479	0.0014	1.4	4.53	31751.6	6.33	HUMAN	F8VR77	Proliferation-associated protein 2G4
1567	dirMS_041712_NAR_30mM_plus2.7403.7423.2	2	17.02	0	1.76E+05		(K)TTTAAA'	(K)T T|T/A|A|A|V/A S/T/G|P S S R(S)		93.25	689.3507	1377.697	-0.0027	-2	9.41	75259.2	9.59	HUMAN	B5MEG9	Microtubule-associated protein 4 (Fragment)
1568	dirMS_041712_NAR_40mM_plus2.17456.17834.3	3	17.02	1	5.62E+05		(R)GLLPGLI	(R)G L|L|P|G L/L/P\A/P A D K L T P K(G)		170.58	567.6811	1701.031	-0.0022	-1.3	8.59	153084.9	5.55	HUMAN	Q6P2E9	Enhancer of mRNA-decapping protein 4
1569	dirMS_041712_NAR_40mM_plus2.18529.18788.3	3	17.02	0	4.34E+05		(K)ALIEVLQ	(K)A L|I/E|V/L/Q/P L I/A E H Q A R(R)		176.12	601.0146	1801.033	-0.0038	-2.1	5.4	53506.8	5.83	HUMAN	P23381	Tryptophan--tRNA ligase, cytoplasmic
1570	dirMS_041712_NAR_30mM_plus2.21715.21864.2	2	17.01	0	3.31E+05		(R)IIWELIK(	(R)I/I|W/E|L/I/K(E)		169.42	457.7881	914.571	-0.002	-2.2	6	17833.3	8.73	HUMAN	C9J4N6	Isocitrate dehydrogenase [NADP] cytoplasmic (Fragment)
1571	dirMS_041712_NAR_60mM_plus.6354.6354.3	3	17.01	0	9.24E+04		(R)HNGAAI	(R)H N G A A E|P E|P E/A E T A/R(G)		91.8	526.9106	1578.714	0.003	1.9	4.48	87537	9.67	HUMAN	Q9Y446	Plakophilin-3
1572	dirMS_041712_NAR_50mM_minus2.15713.15799.3	3	17	0	1.29E+05		(K)FTISDHP	(K)F/T|I/S D/H P Q/P I D/P L/L/K(N)		157.77	574.3122	1720.927	-0.0048	-2.8	5.21	138084.9	5.52	HUMAN	Q86VP6	Cullin-associated NEDD8-dissociated protein 1
1573	dirMS_041712_NAR_60mM_minus.6937.7030.3	3	17	1	2.74E+05		(R)IVDGKV'	(R)I/V|D/G K/V/V/S E/T N D\T K(V)		97.95	502.2642	1504.785	-0.0073	-4.8	4.56	48057	5.34	HUMAN	P05783	Keratin, type I cytoskeletal 18
1574	dirMS_041712_NAR_20mM_plus.16772.16772.2	2	17	0	3.05E+05		(K)VQIAVA	(K)V Q|I/A|V/A/N A/Q E L/L Q R(M)		186.97	776.9419	1552.881	-0.004	-2.6	5.97	10728.1	9.15	HUMAN	Q9Y5L4	Mitochondrial import inner membrane translocase subunit Tim13
1575	dirMS_041712_NAR_40mM_minus2.9011.9011.3	3	16.99	0	2.56E+04		(K)AASAGV	(K)A/A/S/A/G V/P Y H G E V/P V S L A R(A)		135.53	594.3141	1780.934	-0.0063	-3.5	6.79	272689.3	5.79	HUMAN	O15020	Spectrin beta chain, non-erythrocytic 2
1576	dirMS_041712_NAR_50mM_minus2.14841.14901.3	3	16.99	1	2.45E+05		(K)IIVDELK(	(K)I/I|V/D E/L K/Q E/V/I/S T/S S K(A)		152.52	597.0016	1788.995	-0.0051	-2.8	4.68	30149.5	5.96	HUMAN	D6RAC2	Guanine nucleotide-binding protein subunit beta-2-like 1
1577	dirMS_041712_NAR_50mM_minus2.8634.8716.3	3	16.99	0	1.65E+05	M180m	(R)VVVLmG	(R)V V|V|L/m/G S/T/S/D L/G H C E K(I)	C:Carbamidomethylation m:Oxid	117.55	583.2829	1731.84	15.9937	-0.7	5.32	37681.5	5.93	HUMAN	D6RF62	Phosphoribosylaminoimidazole carboxylase
1578	dirMS_041712_NAR_60mM_minus.13610.13610.3	3	16.99	0	3.60E+05		(K)ILQGGV(	(K)I/L|Q/G/G V/G/I/P H I/R(S)		136.02	420.5931	1259.758	0.0065	5.1	9.75	9772.1	9.6	HUMAN	D6RGE5	Casein kinase I isoform alpha
1579	dirMS_041712_NAR_120mM_plus.12512.12970.3	3	16.99	1	9.33E+05		(R)LTPYEVE	(R)L/T|P/Y E|V D/E/L R/R(K)		119.82	464.2488	1390.733	-0.0007	-0.5	4.68	73202	9.19	HUMAN	C9JMU5	Probable ATP-dependent RNA helicase DDX17
1580	dirMS_041712_NAR_120mM_plus.13692.14009.3	3	16.99	1	3.64E+06		(K)ASLENSI	(K)A/S|L/E/N S/L/R E V/E/A/R(Y)		125.98	491.9278	1473.766	0.0033	2.2	4.79	43773.3	5.26	HUMAN	F8VZY9	Keratin, type I cytoskeletal 18
1581	dirMS_041712_NAR_20mM_plus.11302.11302.2	2	16.99	0	2.56E+04		(R)IVLQIDN	(R)I/V|L|Q/I/D N A R(L)		150.32	521.3056	1041.605	-0.0012	-1.1	5.84	20842.9	5.32	HUMAN	C9JM50	Keratin, type I cytoskeletal 19 (Fragment)
1582	dirMS_041712_NAR_20mM_plus.15525.15608.2	2	16.99	0	2.37E+05		(K)TITLEVEI	(K)T I|T/L|E/V|E|P S D T I E/N V K(A)		179.03	894.4658	1787.927	-0.003	-1.7	4	17271.6	6.75	HUMAN	B4DV12	Ubiquitin
1583	dirMS_041712_NAR_20mM_plus.17082.17431.2	2	16.99	0	1.44E+06		(R)VLITTDL	(R)V/L|I|T|T/D/L L/A R(G)		191	557.8442	1114.683	-0.0019	-1.7	5.81	41517.9	5.45	HUMAN	E7EQG2	Eukaryotic initiation factor 4A-II
1584	dirMS_041712_NAR_30mM_plus2.16108.16255.2	2	16.99	0	5.14E+05		(R)ALQALEI	(R)A/L|Q|A|L/E/E/L/R(L)		137.4	521.7961	1042.589	-0.0042	-4	4.53	533778	5.74	HUMAN	Q15149	Plectin
1585	dirMS_041712_NAR_30mM_plus2.17016.17016.2	2	16.99	0	2.20E+05		(R)GLGLDD	(R)G L/G|L/D/D/A/L/E/P R(Q)		142.2	578.3042	1155.6	0.0007	0.6	4.03	51326.8	5.49	HUMAN	Q9Y230	RuvB-like 2
1586	dirMS_041712_NAR_50mM_plus2.15918.15935.3	3	16.99	1	1.60E+06		(R)DVDFELI	(R)D V|D/F/E/L/I/K V E G/K(V)		152	464.5859	1391.742	0.0015	1.1	4.32	49982	5.86	HUMAN	B4DYD8	T-complex protein 1 subunit epsilon
1587	dirMS_041712_NAR_60mM_plus.12503.12707.3	3	16.99	1	8.88E+05		(K)NKTGAA	(K)N K T G A A|P|I|I/D/V/V/R(S)		122.72	451.9377	1353.785	0.0137	10.1	8.75	10240.9	10.46	HUMAN	E9PJD9	60S ribosomal protein L27a
1588	dirMS_041712_NAR_50mM_minus2.13648.13648.3	3	16.98	0	4.59E+04		(K)MLDAEE	(K)M/L/D/A E/D I/V N T A R P D/E K(A)		144.98	606.2965	1816.875	0.0004	0.2	4.1	105308.6	5.27	HUMAN	O43707	Alpha-actinin-4
1589	dirMS_041712_NAR_60mM_minus.13309.13309.3	3	16.98	1	8.97E+05		(R)ELISNSSI	(R)E L/I/S/N/S/S D/A\L D K I R(Y)		134.7	520.9443	1560.823	-0.0044	-2.8	4.56	20151.2	4.58	HUMAN	G3V2J8	Heat shock protein HSP 90-alpha (Fragment)
1590	dirMS_041712_NAR_40mM_minus2.13145.13145.3	3	16.97	1	9.20E+04		(K)VLDSGA	(K)V L|D|S/G A/P I K/I/P V/G P E T L G R(I)		160.87	640.3618	1919.096	-0.0252	-13.1	6.04	55307.5	5.25	HUMAN	F8VPV9	ATP synthase subunit beta
1591	dirMS_041712_NAR_30mM_plus2.11036.11074.2	2	16.97	0	5.95E+04		(K)FAAATG	(K)F A|A|A|T|G/A|T/P I A G R(F)		111.33	602.3261	1203.648	-0.0031	-2.6	9.75	33022.6	4.78	HUMAN	A6NE09	Protein RPSAP58
1592	dirMS_041712_NAR_30mM_plus2.21540.21863.2	2	16.97	0	6.04E+05		(R)IIWQFIK	(R)I/I|W/Q/F/I/K(E)		169.2	474.2886	947.571	-0.0014	-1.5	8.75	45578.2	7.83	HUMAN	B4DFL2	Isocitrate dehydrogenase [NADP]
1593	dirMS_041712_NAR_50mM_plus2.10688.10688.3	3	16.97	1	2.00E+05		(K)GLESTTL	(K)G L/E/S T T L/A D K/D/G E I Y C/K(G)	C:Carbamidomethylation	123.87	633.9707	1899.9	-0.0029	-1.5	4.32	16685.6	9.7	HUMAN	E9PND2	Cysteine and glycine-rich protein 1 (Fragment)
1594	dirMS_041712_NAR_20mM_minus.13933.14086.2	2	16.96	0	6.81E+05		(R)VLITTDL	(R)V/L|I|T|T/D/L L/A R(G)		192.22	557.8438	1114.683	-0.0027	-2.4	5.81	41517.9	5.45	HUMAN	E7EQG2	Eukaryotic initiation factor 4A-II
1595	dirMS_041712_NAR_30mM_minus2.12031.12561.2	2	16.96	0	4.46E+06		(R)EYQELM	(R)E Y|Q/E/L/M/N V/K(L)		122.25	577.2825	1153.556	0.0019	1.7	4.53	56607.4	5.37	HUMAN	F8VXB4	Keratin, type II cytoskeletal 8
1596	dirMS_041712_NAR_30mM_minus2.12934.13436.2	2	16.96	0	1.86E+07		(R)IVLQIDN	(R)I/V|L|Q|I|D/N A R(L)		125.43	521.3062	1041.605	0	0	5.84	20842.9	5.32	HUMAN	C9JM50	Keratin, type I cytoskeletal 19 (Fragment)
1597	dirMS_041712_NAR_40mM_minus2.14645.14881.3	3	16.96	0	1.19E+06		(K)ISSIQSIV	(K)I/S|S I/Q/S I/V/P A L/E I/A/N/A H R(K)		172.1	640.3618	1919.071	0	0	6.75	61224.7	5.7	HUMAN	P10809	60 kDa heat shock protein, mitochondrial
1598	dirMS_041712_NAR_120mM_minus.7012.7340.2	2	16.95	1	3.10E+06		(K)IIAPPERI	(K)I/I|A|P/P E/R K(Y)		93.48	462.2871	923.567	-0.0004	-0.4	8.75	32616.5	5.18	HUMAN	A6NL76	Actin, alpha skeletal muscle
1599	dirMS_041712_NAR_40mM_minus2.9420.9420.3	3	16.95	1	2.72E+04		(K)IINADSE	(K)I/I/N/A/D S E D/P K/Y I I N V K(Q)		138.1	611.3244	1831.98	-0.0214	-11.7	4.56	14693.3	4.69	HUMAN	C9JLS9	26S protease regulatory subunit 7 (Fragment)
1600	dirMS_041712_NAR_60mM_minus.10046.10046.3	3	16.95	1	1.94E+05		(R)VEVGKD	(R)V/E|V G K D Q E F T/V/D/T R(G)		115.57	541.6051	1622.802	-0.0013	-0.8	4.32	258215.9	5.48	HUMAN	E7EN95	Filamin-B
1601	dirMS_041712_NAR_40mM_plus2.16561.16612.3	3	16.95	1	2.47E+05		(R)VTFSGLF	(R)V/T|F/S/G L/R D T\V T/P G E L/L/K(A)		163.87	611.6744	1833.012	-0.003	-1.6	6.04	266161	5.68	HUMAN	E9PPU0	Epiplakin
1602	dirMS_041712_NAR_50mM_plus2.10746.10746.3	3	16.95	1	8.49E+05		(R)DISTNK\	(R)D I|S/T/N K V E Q|I/P/Y/G\E R(I)		124.08	583.6304	1748.881	-0.0047	-2.7	4.68	160853.1	6.11	HUMAN	F8VU51	YLP motif-containing protein 1
1603	dirMS_041712_NAR_50mM_plus2.5182.5260.3	3	16.95	0	1.37E+06		(K)TATPQQ	(K)T A|T/P/Q|Q A/Q E/V/H E K(L)		86.38	489.5774	1466.723	-0.0057	-3.9	5.37	31075.7	5.65	HUMAN	P60174	Triosephosphate isomerase
1604	dirMS_041712_NAR_50mM_plus2.7203.7203.3	3	16.95	0	1.68E+05		(R)SGSSQEI	(R)S/G/S/S/Q/E L D/V K/P/S A S P Q E R(S)		103.85	634.6443	1901.92	-0.0016	-0.8	4.68	227779.5	12.08	HUMAN	I3L2L6	Serine/arginine repetitive matrix protein 2
1605	dirMS_041712_NAR_60mM_plus.11512.11802.3	3	16.95	1	7.92E+05		(K)TAVCDIF	(K)T|A|V/C D I/P P/R G L K(M)	C:Carbamidomethylation	119.07	442.9125	1326.72	0.0031	2.3	8.41	50199.4	4.75	HUMAN	A6NNZ2	Tubulin beta-8 chain-like protein LOC260334
1606	dirMS_041712_NAR_60mM_plus.6500.6501.3	3	16.95	0	7.31E+04		(K)TVEAEA	(K)T|V|E/A/E A/A/H/G T V T R(H)		92.38	447.892	1341.676	-0.0143	-10.6	5.37	46943.7	6.54	HUMAN	O75874	Isocitrate dehydrogenase [NADP] cytoplasmic
1607	dirMS_041712_NAR_50mM_minus2.15525.15796.3	3	16.94	0	1.90E+06		(R)VSLDVN	(R)V/S|L/D/V N/H/F|A/P D E L T V K(T)		157.4	595.3104	1783.922	-0.0058	-3.3	4.54	20576.9	9.84	HUMAN	F8WE04	Heat shock protein beta-1
1608	dirMS_041712_NAR_60mM_minus.11563.11564.3	3	16.94	0	7.51E+05		(K)TKPYIQ\	(K)T K|P Y\I Q|V/D|I/G/G G Q/T K(T)		124.67	535.6216	1604.864	-0.014	-8.7	8.17	72445.7	5.07	HUMAN	P11021	78 kDa glucose-regulated protein
1609	dirMS_041712_NAR_120mM_plus.18048.18396.3	3	16.94	1	4.47E+06		(R)ARFEELI	(R)A R\F E\E\L|N|A|D/L/F/R(G)		149.05	494.2571	1480.754	0.0024	1.7	4.68	45508.6	5.5	HUMAN	A8K7Q2	Heat shock cognate 71 kDa protein
1610	dirMS_041712_NAR_30mM_plus2.14346.14553.2	2	16.94	0	1.32E+07		(K)LLIEMEC	(K)L/L|I|E/M/E/Q/R(L)		130.35	516.2816	1031.555	0.0005	0.5	4.53	20203.9	6.24	HUMAN	H0YJG0	Creatine kinase B-type (Fragment)
1611	dirMS_041712_NAR_20mM_minus.11231.11231.2	2	16.93	0	2.79E+05		(K)LSELEAA	(K)L/S|E|L|E A/A\L Q R(A)		159.5	565.3134	1129.621	-0.0016	-1.5	4.53	56607.4	5.37	HUMAN	F8VXB4	Keratin, type II cytoskeletal 8
1612	dirMS_041712_NAR_30mM_minus2.13416.13501.2	2	16.93	0	2.86E+05		(R)VFNYNT	(R)V/F|N|Y/N/T/L/E|R(V)		126.75	578.299	1155.579	0.0114	9.9	5.97	99900.1	5.04	HUMAN	B4DZI8	Coatomer protein complex, subunit beta 2 (Beta prime), isoform CRA_b
1613	dirMS_041712_NAR_30mM_minus2.9489.9671.2	2	16.93	0	4.78E+05		(R)TSLGPN(	(R)T/S|L/G/P N/G L/D K(M)		106.28	501.2667	1001.526	-0.0001	-0.1	5.5	55804.4	5.33	HUMAN	B7ZAR1	T-complex protein 1 subunit epsilon
1614	dirMS_041712_NAR_120mM_plus.19980.20516.4	4	16.93	1	3.55E+07		(K)AAIDWF	(K)A/A\I D/W/F/D/G K E F/H/G N I I/K(V)		159.08	491.0027	1960.992	-0.0026	-1.3	5.38	49181	4.93	HUMAN	K7EPT6	TATA-binding protein-associated factor 2N
1615	dirMS_041712_NAR_50mM_plus2.15620.15704.3	3	16.93	1	7.69E+05		(R)AITGASL	(R)A I|T|G A/S L/A/D I/M/A K/R(N)		150.25	473.2658	1417.783	-0.0003	-0.2	8.79	17656.4	11.01	HUMAN	C9JNW5	60S ribosomal protein L24
1616	dirMS_041712_NAR_50mM_plus2.20815.21201.3	3	16.93	0	8.65E+06		(R)LHFFMP	(R)L H\F\F\M|P/G|F|A|P\L/T S R(G)		181.48	540.9498	1620.835	-0.0007	-0.4	9.75	42230.8	4.77	HUMAN	A6NKZ8	Putative tubulin beta chain-like protein ENSP00000290377
1617	dirMS_041712_NAR_120mM_minus.14609.14609.3	3	16.92	1	2.59E+05		(K)LDKSQIH	(K)L D/K/S Q I H\D\I|V|L\V|G\G S T R(I)		133.85	613.3393	1838.013	-0.0097	-5.3	6.75	45508.6	5.5	HUMAN	A8K7Q2	Heat shock cognate 71 kDa protein
1618	dirMS_041712_NAR_30mM_plus2.15023.15626.2	2	16.92	0	3.59E+06		(R)QITVND	(R)Q I|T|V/N/D/L/P V/G R(S)		133.02	606.3403	1211.674	-0.0009	-0.8	5.84	22062.7	5.66	HUMAN	P32119	Peroxiredoxin-2
1619	dirMS_041712_NAR_30mM_plus2.15873.15892.2	2	16.92	0	5.53E+05		(K)LLYNNV:	(K)L/L|Y|N/N/V|S/N F/G R(L)		136.27	648.8354	1296.67	-0.006	-4.6	8.75	71165.3	5.57	HUMAN	J3KS13	Clathrin heavy chain 1
1620	dirMS_041712_NAR_50mM_plus2.10681.10824.3	3	16.92	1	8.49E+05		(R)DISTNK\	(R)D I|S/T N K/V|E/Q|I|P Y G E R(I)		124.08	583.6304	1748.881	-0.0047	-2.7	4.68	160853.1	6.11	HUMAN	F8VU51	YLP motif-containing protein 1
1621	dirMS_041712_NAR_60mM_plus.10476.10476.2	2	16.92	1	4.41E+06		(R)LQAEIEC	(R)L Q|A/E|I/E|G L K/G Q R(A)		112.33	671.3774	1341.748	-0.001	-0.7	6.14	56607.4	5.37	HUMAN	F8VXB4	Keratin, type II cytoskeletal 8
1622	dirMS_041712_NAR_60mM_plus.13773.14096.2	2	16.92	1	2.17E+06		(K)FASFIDK	(K)F/A|S|F/I/D/K V/R(F)		129.18	541.8029	1082.599	-0.0008	-0.7	8.75	19569.8	9.88	HUMAN	F8VRG4	Keratin, type II cytoskeletal 8 (Fragment)
1623	dirMS_041712_NAR_60mM_plus.18288.18307.3	3	16.92	1	9.14E+05		(K)EVNKFQ	(K)E V|N K F Q/M/A|Y/S/N/L/L R(A)		152.85	571.6293	1712.879	-0.0055	-3.2	8.69	14683.8	10.19	HUMAN	P37108	Signal recognition particle 14 kDa protein
1624	dirMS_041712_NAR_120mM_minus.9832.9875.3	3	16.91	1	6.96E+05		(R)GSNKLV	(R)G S N K L\V|I|E/E/A/E/R(S)		108.1	448.9032	1344.712	-0.0167	-12.4	4.79	42755	7.81	HUMAN	B7Z2F4	T-complex protein 1 subunit delta
1625	dirMS_041712_NAR_50mM_minus2.10967.11007.3	3	16.91	1	6.21E+05		(R)ISVYYNE	(R)I/S|V|Y/Y N/E A/T/G G K Y V/P R(A)		131.08	606.3119	1816.923	-0.0017	-0.9	8.43	27549.9	4.39	HUMAN	E9PBJ4	Tubulin beta chain
1626	dirMS_041712_NAR_50mM_minus2.15019.15040.3	3	16.91	1	1.08E+06		(R)DVDFELI	(R)D V|D/F/E/L/I/K V E/G K(V)		153.95	464.5852	1391.742	-0.0006	-0.4	4.32	49982	5.86	HUMAN	B4DYD8	T-complex protein 1 subunit epsilon
1627	dirMS_041712_NAR_60mM_minus.13968.14218.3	3	16.91	1	1.28E+06		(K)VKEGMI	(K)V K E G\M N\I|V/E/A/M/E/R(F)		138.52	502.5858	1505.745	-0.0022	-1.5	4.79	18240.4	7.96	HUMAN	P62937	Peptidyl-prolyl cis-trans isomerase A
1628	dirMS_041712_NAR_30mM_plus2.18482.18482.2	2	16.91	0	2.36E+05		(K)GNFNYII	(K)G N|F|N/Y/I/E/F T R(I)		150.27	630.8035	1260.601	-0.001	-0.8	6	20513.6	4.62	HUMAN	J3QRS3	Myosin regulatory light chain 12A
1629	dirMS_041712_NAR_40mM_plus2.11418.11436.3	3	16.91	0	3.79E+05		(R)MPLPAP	(R)M/P|L|P A/P S L S H Q|P/P P A P R(V)		133.63	598.3202	1792.953	-0.0066	-3.7	9.52	160853.1	6.11	HUMAN	F8VU51	YLP motif-containing protein 1
1630	dirMS_041712_NAR_40mM_plus2.14730.14810.3	3	16.91	1	2.59E+05		(K)DVAIKEF	(K)D V|A|I/K E|P\L|V/D\V/V D/P K(Q)		153.82	546.3096	1636.916	-0.0014	-0.8	4.23	24376.8	6.08	HUMAN	E9PIX6	Protein arginine N-methyltransferase 1 (Fragment)

<table>
<tr><td>1631</td><td>dirMS_041712_NAR_50mM_minus2.13426.13510.3</td><td>3</td><td>16.9</td><td>1</td><td>1.91E+05</td><td></td><td>(K)TITLEVEI</td><td>(K)T I|T|L E/V/E/P S D T I/E N V K A K(I)</td><td></td><td>144.63</td><td>663.0226</td><td>1987.059</td><td>-0.0061</td><td>-3.1</td><td>4.41</td><td>17271.6</td><td>6.75</td><td>HUMAN</td><td>B4DV12</td><td>Ubiquitin</td></tr>
<tr><td>1632</td><td>dirMS_041712_NAR_50mM_minus2.8527.8570.3</td><td>3</td><td>16.9</td><td>1</td><td>2.43E+05</td><td></td><td>(R)AGTGVD</td><td>(R)A/G/T|G/V/D/N V/D L/E/A/A T R K(G)</td><td></td><td>116.55</td><td>539.6119</td><td>1616.824</td><td>-0.0027</td><td>-1.7</td><td>4.56</td><td>57391.2</td><td>6.29</td><td>HUMAN</td><td>O43175</td><td>D-3-phosphoglycerate dehydrogenase</td></tr>
<tr><td>1633</td><td>dirMS_041712_NAR_60mM_minus.16779.17051.3</td><td>3</td><td>16.9</td><td>0</td><td>1.02E+06</td><td></td><td>(K)ICDQWD</td><td>(K)I/C|D Q/W D A|L/G S L/T H S R(R)</td><td>C:Carbamidomethylation</td><td>154.87</td><td>586.9446</td><td>1758.823</td><td>-0.0035</td><td>-2</td><td>5.21</td><td>80254.2</td><td>5.05</td><td>HUMAN</td><td>D6PXK4</td><td>Alpha actinin 4 short isoform</td></tr>
<tr><td>1634</td><td>dirMS_041712_NAR_120mM_plus.20691.21229.4</td><td>4</td><td>16.9</td><td>2</td><td>1.12E+07</td><td></td><td>(R)TLAEIAK</td><td>(R)T/L\A E/I/A/K/V E L D N M/P L R G K(Q)</td><td></td><td>163.07</td><td>500.2814</td><td>1998.105</td><td>-0.0015</td><td>-0.7</td><td>5.85</td><td>23729.5</td><td>9.89</td><td>HUMAN</td><td>C9IZL7</td><td>Non-POU domain-containing octamer-binding protein (Fragment)</td></tr>
<tr><td>1635</td><td>dirMS_041712_NAR_50mM_plus2.20771.20807.3</td><td>3</td><td>16.9</td><td>1</td><td>2.60E+05</td><td></td><td>(R)NLQNLL</td><td>(R)N L|Q|N/L/L/I/L/T/A I K/A D R(T)</td><td></td><td>178.05</td><td>566.0067</td><td>1696.012</td><td>-0.006</td><td>-3.6</td><td>8.75</td><td>144109.9</td><td>6.01</td><td>HUMAN</td><td>J3KR87</td><td>Clathrin heavy chain 2</td></tr>
<tr><td>1636</td><td>dirMS_041712_NAR_60mM_plus.14448.14449.3</td><td>3</td><td>16.9</td><td>1</td><td>6.02E+05</td><td></td><td>(K)SLERAEA</td><td>(K)S L|E R A E\A\G D\N|L|G/A/L|V/R(G)</td><td></td><td>131.97</td><td>557.6331</td><td>1670.882</td><td>0.0027</td><td>1.6</td><td>4.68</td><td>49883</td><td>7.3</td><td>HUMAN</td><td>P49411</td><td>Elongation factor Tu, mitochondrial</td></tr>
<tr><td>1637</td><td>dirMS_041712_NAR_60mM_minus.6725.7214.3</td><td>3</td><td>16.89</td><td>0</td><td>1.27E+06</td><td>M437m</td><td>(R)LESGMC</td><td>(R)L/E|S G/M Q/N m/S I/H/T/K(T)</td><td>m:Oxidized methionine</td><td>97.23</td><td>497.9023</td><td>1475.698</td><td>15.9942</td><td>-0.5</td><td>6.75</td><td>56607.4</td><td>5.37</td><td>HUMAN</td><td>F8VXB4</td><td>Keratin, type II cytoskeletal 8</td></tr>
<tr><td>1638</td><td>dirMS_041712_NAR_50mM_plus2.10973.10995.3</td><td>3</td><td>16.89</td><td>1</td><td>3.72E+05</td><td></td><td>(K)LAEQAE</td><td>(K)L/A|E/Q/A/E R Y D/D\M|A/A C M K(S)</td><td>C:Carbamidomethylation</td><td>125.2</td><td>634.6126</td><td>1901.819</td><td>0.0042</td><td>2.2</td><td>4.32</td><td>5919.6</td><td>4.73</td><td>HUMAN</td><td>E5RGE1</td><td>14-3-3 protein zeta/delta (Fragment)</td></tr>
<tr><td>1639</td><td>dirMS_041712_NAR_120mM_minus.10984.11410.3</td><td>3</td><td>16.88</td><td>1</td><td>8.74E+05</td><td></td><td>(R)RFDDAV</td><td>(R)R F D D\A\V|V|Q/S/D/M/K(H)</td><td></td><td>115.2</td><td>470.8939</td><td>1410.668</td><td>-0.001</td><td>-0.7</td><td>4.43</td><td>20069.4</td><td>6.74</td><td>HUMAN</td><td>E9PK54</td><td>Heat shock cognate 71 kDa protein (Fragment)</td></tr>
<tr><td>1640</td><td>dirMS_041712_NAR_30mM_minus2.16571.16571.3</td><td>3</td><td>16.88</td><td>0</td><td>7.43E+05</td><td></td><td>(R)ASLEAAI</td><td>(R)A S\L E A A I/A/D/A E/Q R(G)</td><td></td><td>143.82</td><td>448.8965</td><td>1344.675</td><td>-0.0004</td><td>-0.3</td><td>4.14</td><td>56607.4</td><td>5.37</td><td>HUMAN</td><td>F8VXB4</td><td>Keratin, type II cytoskeletal 8</td></tr>
<tr><td>1641</td><td>dirMS_041712_NAR_30mM_minus2.7386.7386.2</td><td>2</td><td>16.88</td><td>0</td><td>1.44E+05</td><td></td><td>(R)FGGSGS</td><td>(R)F/G|G\S/G/S/Q/V/D/S A R(M)</td><td></td><td>94.65</td><td>584.2729</td><td>1167.539</td><td>-0.0004</td><td>-0.3</td><td>5.84</td><td>86346.5</td><td>5.05</td><td>HUMAN</td><td>E7EW34</td><td>26S proteasome non-ATPase regulatory subunit 2</td></tr>
<tr><td>1642</td><td>dirMS_041712_NAR_50mM_minus2.10622.10622.3</td><td>3</td><td>16.88</td><td>0</td><td>1.73E+05</td><td></td><td>(R)LLELGPK</td><td>(R)L/L/E|L/G/P K/P E V A Q Q T R(K)</td><td></td><td>128.68</td><td>560.3182</td><td>1678.949</td><td>-0.0086</td><td>-5.1</td><td>6.14</td><td>139883.7</td><td>8.06</td><td>HUMAN</td><td>P53621</td><td>Coatomer subunit alpha</td></tr>
<tr><td>1643</td><td>dirMS_041712_NAR_50mM_minus2.8432.8432.3</td><td>3</td><td>16.88</td><td>1</td><td>2.36E+05</td><td></td><td>(R)AGTGVD</td><td>(R)A G/T|G/V/D/N V D/L/E A/A T R\K(G)</td><td></td><td>116.53</td><td>539.6119</td><td>1616.824</td><td>-0.0027</td><td>-1.7</td><td>4.56</td><td>57391.2</td><td>6.29</td><td>HUMAN</td><td>O43175</td><td>D-3-phosphoglycerate dehydrogenase</td></tr>
<tr><td>1644</td><td>dirMS_041712_NAR_120mM_plus.7170.7233.3</td><td>3</td><td>16.88</td><td>1</td><td>4.42E+05</td><td></td><td>(K)AQLEPV.</td><td>(K)A Q|L/E/P V/A/S/P A K K/P K(V)</td><td></td><td>91.1</td><td>488.6234</td><td>1463.858</td><td>-0.0024</td><td>-1.6</td><td>9.7</td><td>533778</td><td>5.74</td><td>HUMAN</td><td>Q15149</td><td>Plectin</td></tr>
<tr><td>1645</td><td>dirMS_041712_NAR_50mM_plus2.12896.13141.3</td><td>3</td><td>16.88</td><td>1</td><td>1.61E+06</td><td></td><td>(K)LIADVAF</td><td>(K)L/I|A/D/V/A/P S A I R E N D/I/K(S)</td><td></td><td>136.73</td><td>575.6533</td><td>1724.954</td><td>-0.0088</td><td>-5.1</td><td>4.56</td><td>18388.8</td><td>6.85</td><td>HUMAN</td><td>F5H1Y3</td><td>Flap endonuclease 1 (Fragment)</td></tr>
<tr><td>1646</td><td>dirMS_041712_NAR_30mM_minus2.18843.18931.2</td><td>2</td><td>16.87</td><td>0</td><td>2.45E+05</td><td></td><td>(K)LLQDFFN</td><td>(K)L/L|Q|D/F/F/N G/R(D)</td><td></td><td>157.37</td><td>555.2906</td><td>1109.574</td><td>0.0001</td><td>0.1</td><td>5.84</td><td>60126</td><td>5.44</td><td>HUMAN</td><td>E7EP94</td><td>Heat shock 70 kDa protein 1A/1B</td></tr>
<tr><td>1647</td><td>dirMS_041712_NAR_50mM_plus2.7160.7223.3</td><td>3</td><td>16.87</td><td>0</td><td>1.04E+06</td><td></td><td>(R)EATNPP'</td><td>(R)E A|T/N/P/P V/I Q/E E K/P K(K)</td><td></td><td>103.55</td><td>527.282</td><td>1579.833</td><td>-0.0012</td><td>-0.7</td><td>4.79</td><td>55361.8</td><td>6.42</td><td>HUMAN</td><td>G5EA52</td><td>Protein disulfide isomerase family A, member 3, isoform CRA_b</td></tr>
<tr><td>1648</td><td>dirMS_041712_NAR_60mM_plus.10333.10507.3</td><td>3</td><td>16.87</td><td>0</td><td>4.39E+06</td><td></td><td>(R)SSGPYG</td><td>(R)S S|G/P/Y/G G/G G Q/Y/F/A/K/P\R(N)</td><td></td><td>112.17</td><td>543.6023</td><td>1628.782</td><td>0.0108</td><td>6.6</td><td>9.7</td><td>33268.7</td><td>9.31</td><td>HUMAN</td><td>F8VRQ1</td><td>Heterogeneous nuclear ribonucleoprotein A1</td></tr>
<tr><td>1649</td><td>dirMS_041712_NAR_120mM_minus.6846.7155.2</td><td>2</td><td>16.86</td><td>1</td><td>2.88E+06</td><td></td><td>(R)TKTEISEI</td><td>(R)T/K|T|E|I|S/E/M N/R(N)</td><td></td><td>92.53</td><td>604.8012</td><td>1208.594</td><td>0.0012</td><td>1</td><td>5.81</td><td>37296.3</td><td>5.3</td><td>HUMAN</td><td>F8VU64</td><td>Keratin, type II cytoskeletal 8</td></tr>
<tr><td>1650</td><td>dirMS_041712_NAR_30mM_minus2.10870.11050.2</td><td>2</td><td>16.86</td><td>0</td><td>5.46E+05</td><td></td><td>(R)FASGGC</td><td>(R)F/A|S|G/G/C/D N L/I/K(L)</td><td>C:Carbamidomethylation</td><td>113.6</td><td>591.2858</td><td>1181.562</td><td>0.0024</td><td>2</td><td>5.84</td><td>34524.7</td><td>5.4</td><td>HUMAN</td><td>A8MV37</td><td>Protein SEC13 homolog</td></tr>
<tr><td>1651</td><td>dirMS_041712_NAR_30mM_minus2.14109.14499.2</td><td>2</td><td>16.86</td><td>0</td><td>8.44E+05</td><td></td><td>(R)GLFDEY(</td><td>(R)G L|F|D/E/Y/G/S/K(K)</td><td></td><td>131.28</td><td>508.2442</td><td>1015.473</td><td>0.008</td><td>7.9</td><td>4.37</td><td>92752.5</td><td>4.76</td><td>HUMAN</td><td>P14625</td><td>Endoplasmin</td></tr>
<tr><td>1652</td><td>dirMS_041712_NAR_40mM_minus2.13608.13608.3</td><td>3</td><td>16.86</td><td>1</td><td>1.19E+05</td><td></td><td>(R)CITDPQT</td><td>(R)C/I|T/D/P Q T G L C L L/P L/K/E K(K)</td><td>C:Carbamidomethylation</td><td>164.02</td><td>662.684</td><td>1986.04</td><td>-0.0024</td><td>-1.2</td><td>6.07</td><td>533778</td><td>5.74</td><td>HUMAN</td><td>Q15149</td><td>Plectin</td></tr>
<tr><td>1653</td><td>dirMS_041712_NAR_60mM_minus.13268.13540.3</td><td>3</td><td>16.86</td><td>1</td><td>7.33E+06</td><td></td><td>(K)KVPQVS</td><td>(K)K\V\P\Q\V\S\T|P T|L|V/E V S R(N)</td><td></td><td>134.78</td><td>547.317</td><td>1639.938</td><td>-0.0013</td><td>-0.8</td><td>8.75</td><td>57751.3</td><td>6.9</td><td>HUMAN</td><td>B7WNR0</td><td>Serum albumin</td></tr>
<tr><td>1654</td><td>dirMS_041712_NAR_30mM_plus2.12350.12818.2</td><td>2</td><td>16.86</td><td>0</td><td>7.35E+05</td><td></td><td>(K)NAGVEG</td><td>(K)N/A|G|V|E|G S L/I/V/E K(I)</td><td></td><td>118.18</td><td>608.3313</td><td>1215.658</td><td>-0.0026</td><td>-2.2</td><td>4.53</td><td>61224.7</td><td>5.7</td><td>HUMAN</td><td>P10809</td><td>60 kDa heat shock protein, mitochondrial</td></tr>
<tr><td>1655</td><td>dirMS_041712_NAR_30mM_plus2.13055.13055.2</td><td>2</td><td>16.86</td><td>0</td><td>4.73E+05</td><td></td><td>(K)QSLPPG</td><td>(K)Q S L/P/P G L/A/V K(E)</td><td></td><td>121.9</td><td>505.3008</td><td>1009.604</td><td>-0.0097</td><td>-9.6</td><td>8.75</td><td>8274.8</td><td>10.19</td><td>HUMAN</td><td>P63173</td><td>60S ribosomal protein L38</td></tr>
<tr><td>1656</td><td>dirMS_041712_NAR_50mM_plus2.14641.14662.3</td><td>3</td><td>16.86</td><td>0</td><td>5.16E+05</td><td></td><td>(K)MLDAED</td><td>(K)M/L|D|A/E/D/I/V/N T A R P D/E K(A)</td><td></td><td>144.95</td><td>606.2972</td><td>1816.875</td><td>0.0025</td><td>1.4</td><td>4.1</td><td>105308.6</td><td>5.27</td><td>HUMAN</td><td>O43707</td><td>Alpha-actinin-4</td></tr>
<tr><td>1657</td><td>dirMS_041712_NAR_50mM_minus2.17010.17476.3</td><td>3</td><td>16.85</td><td>0</td><td>2.11E+07</td><td></td><td>(R)IHFPLAT</td><td>(R)I H F\P L\A\T\Y|A|P/V/I S A E K(A)</td><td></td><td>167.2</td><td>586.3248</td><td>1756.963</td><td>-0.0034</td><td>-1.9</td><td>6.75</td><td>49012.3</td><td>4.9</td><td>HUMAN</td><td>A8MUB1</td><td>Tubulin alpha-4A chain</td></tr>
<tr><td>1658</td><td>dirMS_041712_NAR_60mM_minus.16805.17138.3</td><td>3</td><td>16.85</td><td>1</td><td>2.04E+06</td><td></td><td>(K)LLQDFFN</td><td>(K)L/L|Q/D/F/F/N/G K/E L N K(S)</td><td></td><td>155.22</td><td>522.6138</td><td>1565.832</td><td>-0.0054</td><td>-3.4</td><td>6.07</td><td>45508.6</td><td>5.5</td><td>HUMAN</td><td>A8K7Q2</td><td>Heat shock cognate 71 kDa protein</td></tr>
<tr><td>1659</td><td>dirMS_041712_NAR_50mM_plus2.7651.7651.3</td><td>3</td><td>16.85</td><td>1</td><td>1.64E+05</td><td></td><td>(K)VDDSSEI</td><td>(K)V/D|D/S/S E D K T E F T V/K(N)</td><td></td><td>106.45</td><td>533.9169</td><td>1599.738</td><td>-0.0023</td><td>-1.4</td><td>4.11</td><td>107550.2</td><td>5.34</td><td>HUMAN</td><td>P49588</td><td>Alanine--tRNA ligase, cytoplasmic</td></tr>
<tr><td>1660</td><td>dirMS_041712_NAR_120mM_minus.11387.11485.3</td><td>3</td><td>16.84</td><td>0</td><td>5.06E+06</td><td></td><td>(R)GSAVHC</td><td>(R)G S|A/V/H/Q/L/S/E E/L/R(C)</td><td></td><td>116.67</td><td>442.565</td><td>1325.681</td><td>-0.0004</td><td>-0.3</td><td>5.4</td><td>558007.1</td><td>5.44</td><td>HUMAN</td><td>P58107</td><td>Epiplakin</td></tr>
<tr><td>1661</td><td>dirMS_041712_NAR_20mM_plus.12564.12629.2</td><td>2</td><td>16.84</td><td>0</td><td>2.52E+05</td><td></td><td>(K)DVNAAI.</td><td>(K)D V|N|A/A/I/A/A I/K(T)</td><td></td><td>159.55</td><td>493.2863</td><td>985.568</td><td>-0.0023</td><td>-2.4</td><td>5.84</td><td>49012.3</td><td>4.9</td><td>HUMAN</td><td>A8MUB1</td><td>Tubulin alpha-4A chain</td></tr>
<tr><td>1662</td><td>dirMS_041712_NAR_20mM_plus.14983.14984.2</td><td>2</td><td>16.84</td><td>0</td><td>1.45E+05</td><td></td><td>(K)VLEQLT(</td><td>(K)V L|E/Q|L|T|G\Q|T/P V/F/S K(A)</td><td></td><td>174.65</td><td>773.9244</td><td>1546.848</td><td>-0.006</td><td>-3.9</td><td>5.97</td><td>20480.2</td><td>9.91</td><td>HUMAN</td><td>P62913</td><td>60S ribosomal protein L11</td></tr>
<tr><td>1663</td><td>dirMS_041712_NAR_20mM_plus.16906.16906.2</td><td>2</td><td>16.84</td><td>0</td><td>3.11E+05</td><td></td><td>(K)VQIAVA</td><td>(K)V Q|I/A|V|A/N\A Q/E L L/Q R(M)</td><td></td><td>187.77</td><td>776.9419</td><td>1552.881</td><td>-0.004</td><td>-2.6</td><td>5.97</td><td>10728.1</td><td>9.15</td><td>HUMAN</td><td>Q9Y5L4</td><td>Mitochondrial import inner membrane translocase subunit Tim13</td></tr>
<tr><td>1664</td><td>dirMS_041712_NAR_30mM_plus2.19807.19923.2</td><td>2</td><td>16.84</td><td>0</td><td>5.28E+05</td><td></td><td>(K)MAILQIN</td><td>(K)M/A|I/L/Q|I/M/K(E)</td><td></td><td>158.22</td><td>474.2739</td><td>947.542</td><td>-0.0011</td><td>-1.2</td><td>8.5</td><td>90316.8</td><td>5.52</td><td>HUMAN</td><td>Q13263</td><td>Transcription intermediary factor 1-beta</td></tr>
<tr><td>1665</td><td>dirMS_041712_NAR_30mM_plus2.6326.6326.2</td><td>2</td><td>16.84</td><td>0</td><td>1.43E+05</td><td></td><td>(R)FGGSGS</td><td>(R)F/G/G/S/G|S Q V/D/S A/R(M)</td><td></td><td>87.52</td><td>584.2731</td><td>1167.539</td><td>0</td><td>0</td><td>5.84</td><td>86346.5</td><td>5.05</td><td>HUMAN</td><td>E7EW34</td><td>26S proteasome non-ATPase regulatory subunit 2</td></tr>
<tr><td>1666</td><td>dirMS_041712_NAR_30mM_minus2.17646.17852.2</td><td>2</td><td>16.83</td><td>0</td><td>1.51E+06</td><td></td><td>(K)AILFVPR</td><td>(K)A/I|L|F/V/P/R(R)</td><td></td><td>149.97</td><td>408.2606</td><td>815.514</td><td>0.0001</td><td>0.2</td><td>9.79</td><td>57660.2</td><td>6.38</td><td>HUMAN</td><td>Q9NQH7</td><td>Probable Xaa-Pro aminopeptidase 3</td></tr>
<tr><td>1667</td><td>dirMS_041712_NAR_50mM_minus2.15523.15761.3</td><td>3</td><td>16.83</td><td>1</td><td>6.45E+05</td><td></td><td>(K)LLQDFFN</td><td>(K)L/L|Q/D/F/F/N/G K E L/N/K(S)</td><td></td><td>157.4</td><td>522.6138</td><td>1565.832</td><td>-0.0054</td><td>-3.4</td><td>6.07</td><td>45508.6</td><td>5.5</td><td>HUMAN</td><td>A8K7Q2</td><td>Heat shock cognate 71 kDa protein</td></tr>
<tr><td>1668</td><td>dirMS_041712_NAR_60mM_minus.8304.8479.3</td><td>3</td><td>16.83</td><td>1</td><td>1.25E+06</td><td>M2747m</td><td>(K)TLQEVTI</td><td>(K)T L|Q/E/V/T E/m D/S V K/R(Y)</td><td>m:Oxidized methionine</td><td>106.78</td><td>517.927</td><td>1535.773</td><td>15.9931</td><td>-1.2</td><td>4.68</td><td>558007.1</td><td>5.44</td><td>HUMAN</td><td>P58107</td><td>Epiplakin</td></tr>
<tr><td>1669</td><td>dirMS_041712_NAR_120mM_plus.10377.10926.3</td><td>3</td><td>16.83</td><td>0</td><td>1.90E+06</td><td></td><td>(R)GFCLPPI</td><td>(R)G F|C\L/P/P/H C S R(G)</td><td>C:Carbamidomethylation</td><td>108.8</td><td>410.8593</td><td>1230.551</td><td>0.0127</td><td>10.3</td><td>9.75</td><td>14391.8</td><td>4.99</td><td>HUMAN</td><td>G3V461</td><td>Creatine kinase B-type (Fragment)</td></tr>
<tr><td>1670</td><td>dirMS_041712_NAR_20mM_plus.16773.16773.2</td><td>2</td><td>16.83</td><td>0</td><td>3.05E+05</td><td></td><td>(K)VQIAVA</td><td>(K)V Q|I A/V/A/N/A Q E/L/L Q R(M)</td><td></td><td>186.97</td><td>776.9419</td><td>1552.881</td><td>-0.004</td><td>-2.6</td><td>5.97</td><td>10728.1</td><td>9.15</td><td>HUMAN</td><td>Q9Y5L4</td><td>Mitochondrial import inner membrane translocase subunit Tim13</td></tr>
<tr><td>1671</td><td>dirMS_041712_NAR_50mM_plus2.18499.18829.3</td><td>3</td><td>16.83</td><td>1</td><td>9.58E+06</td><td></td><td>(R)LFVGNLI</td><td>(R)L F|V|G/N/L/P A/D I/T E D E F K R(L)</td><td></td><td>166.67</td><td>655.3417</td><td>1964.012</td><td>-0.0018</td><td>-0.9</td><td>4.32</td><td>76262.2</td><td>9.53</td><td>HUMAN</td><td>P23246</td><td>Splicing factor, proline- and glutamine-rich</td></tr>
<tr><td>1672</td><td>dirMS_041712_NAR_50mM_plus2.6022.6042.3</td><td>3</td><td>16.83</td><td>1</td><td>2.93E+05</td><td></td><td>(K)TGQAPG</td><td>(K)T/G|Q|A|P/G Y S Y/T/A A/N K N K(G)</td><td></td><td>95.93</td><td>557.6102</td><td>1670.813</td><td>0.0028</td><td>1.7</td><td>9.52</td><td>11447.2</td><td>9.81</td><td>HUMAN</td><td>C9JFR7</td><td>Cytochrome c (Fragment)</td></tr>
<tr><td>1673</td><td>dirMS_041712_NAR_30mM_minus2.18342.18342.2</td><td>2</td><td>16.82</td><td>0</td><td>4.39E+05</td><td></td><td>(R)QAQEYE</td><td>(R)Q A/Q E|Y|E/A L/L/N/I/K(V)</td><td></td><td>154.65</td><td>710.3759</td><td>1419.748</td><td>-0.0033</td><td>-2.3</td><td>4.53</td><td>43773.3</td><td>5.26</td><td>HUMAN</td><td>F8VZY9</td><td>Keratin, type I cytoskeletal 18</td></tr>
<tr><td>1674</td><td>dirMS_041712_NAR_50mM_minus2.9207.9207.3</td><td>3</td><td>16.82</td><td>0</td><td>1.89E+05</td><td>M778m</td><td>(R)mLTGPV</td><td>(R)m/L/T G/P V Y S Q/S T A L T H/K(Q)</td><td>m:Oxidized methionine</td><td>121.02</td><td>583.963</td><td>1733.889</td><td>15.9854</td><td>-5.5</td><td>8.37</td><td>138084.9</td><td>5.52</td><td>HUMAN</td><td>Q86VP6</td><td>Cullin-associated NEDD8-dissociated protein 1</td></tr>
<tr><td>1675</td><td>dirMS_041712_NAR_60mM_minus.13697.13792.3</td><td>3</td><td>16.82</td><td>0</td><td>4.30E+05</td><td></td><td>(K)IVLDNSV</td><td>(K)I/V|L/D\N/S/V/F/S/E/H R(N)</td><td></td><td>136.55</td><td>472.5814</td><td>1415.728</td><td>0.0019</td><td>1.3</td><td>5.32</td><td>144109.9</td><td>6.01</td><td>HUMAN</td><td>J3KR87</td><td>Clathrin heavy chain 2</td></tr>
<tr><td>1676</td><td>dirMS_041712_NAR_120mM_plus.8466.8466.3</td><td>3</td><td>16.82</td><td>1</td><td>3.33E+05</td><td></td><td>(R)LAEDEAI</td><td>(R)L/A|E/D/E A F Q R R(R)</td><td></td><td>97.8</td><td>412.2128</td><td>1234.618</td><td>0.0064</td><td>5.2</td><td>4.68</td><td>533778</td><td>5.74</td><td>HUMAN</td><td>Q15149</td><td>Plectin</td></tr>
<tr><td>1677</td><td>dirMS_041712_NAR_20mM_plus.12257.12257.2</td><td>2</td><td>16.82</td><td>0</td><td>1.17E+05</td><td></td><td>(K)VNQIGS'</td><td>(K)V N Q|I|G/S/V/T E S I/Q A C K(L)</td><td>C:Carbamidomethylation</td><td>157.67</td><td>817.4114</td><td>1633.821</td><td>-0.0059</td><td>-3.6</td><td>5.97</td><td>47328.4</td><td>7.76</td><td>HUMAN</td><td>P13929</td><td>Beta-enolase</td></tr>
<tr><td>1678</td><td>dirMS_041712_NAR_30mM_plus2.11042.11346.2</td><td>2</td><td>16.82</td><td>0</td><td>2.26E+06</td><td></td><td>(R)VEFMDD</td><td>(R)V/E|F|M/D/D/T S/R(S)</td><td></td><td>111.32</td><td>550.2401</td><td>1099.473</td><td>0.0005</td><td>0.4</td><td>4.03</td><td>7898</td><td>11.1</td><td>HUMAN</td><td>P62857</td><td>40S ribosomal protein S28</td></tr>
<tr><td>1679</td><td>dirMS_041712_NAR_60mM_plus.9291.9291.3</td><td>3</td><td>16.82</td><td>1</td><td>1.31E+06</td><td></td><td>(R)FNEVAA</td><td>(R)F/N|E/V|A A/Q/Y/S E\D K A R(Q)</td><td></td><td>106.1</td><td>543.2616</td><td>1627.771</td><td>-0.0008</td><td>-0.5</td><td>4.68</td><td>14236.2</td><td>10.25</td><td>HUMAN</td><td>F5H1P5</td><td>Peptidyl-prolyl cis-trans isomerase NIMA-interacting 4</td></tr>
<tr><td>1680</td><td>dirMS_041712_NAR_60mM_minus.11650.11650.3</td><td>3</td><td>16.81</td><td>1</td><td>2.96E+06</td><td></td><td>(K)VKLEAEI</td><td>(K)V/K L E A E|I/A/T/Y/R(R)</td><td></td><td>125.33</td><td>431.5779</td><td>1292.721</td><td>-0.0017</td><td>-1.3</td><td>6.11</td><td>43773.3</td><td>5.26</td><td>HUMAN</td><td>F8VZY9</td><td>Keratin, type I cytoskeletal 18</td></tr>
<tr><td>1681</td><td>dirMS_041712_NAR_60mM_minus.15793.16068.3</td><td>3</td><td>16.81</td><td>1</td><td>5.23E+05</td><td></td><td>(R)IFDSEEIL</td><td>(R)I/F\D/S/E E I/L/A/G Y K/R(E)</td><td></td><td>148.88</td><td>514.2713</td><td>1540.801</td><td>-0.0012</td><td>-0.8</td><td>4.68</td><td>35548.6</td><td>4.91</td><td>HUMAN</td><td>B4DN60</td><td>Asparagine--tRNA ligase, cytoplasmic</td></tr>
<tr><td>1682</td><td>dirMS_041712_NAR_20mM_plus.6462.6462.2</td><td>2</td><td>16.81</td><td>0</td><td>2.02E+05</td><td>M216m</td><td>(R)DLmACA</td><td>(R)D L/m|A/C A/Q T G S\G K(T)</td><td>C:Carbamidomethylation m:Oxid</td><td>107.72</td><td>627.776</td><td>1238.55</td><td>15.9943</td><td>-0.5</td><td>5.84</td><td>73386.6</td><td>8.73</td><td>HUMAN</td><td>B4DXX7</td><td>ATP-dependent RNA helicase DDX3Y</td></tr>
<tr><td>1683</td><td>dirMS_041712_NAR_30mM_plus2.13546.13691.2</td><td>2</td><td>16.81</td><td>0</td><td>1.10E+06</td><td></td><td>(R)ANTFVA</td><td>(R)A N|T|F/V/A/E/L/K(G)</td><td></td><td>124.65</td><td>496.774</td><td>992.541</td><td>-0.0004</td><td>-0.4</td><td>6.05</td><td>24936.4</td><td>8.67</td><td>HUMAN</td><td>G3XAL0</td><td>Malate dehydrogenase</td></tr>
<tr><td>1684</td><td>dirMS_041712_NAR_50mM_minus2.11513.11513.3</td><td>3</td><td>16.8</td><td>1</td><td>2.41E+04</td><td></td><td>(R)ESDVPLI</td><td>(R)E S D/V/P L/K/T/E E F E V/T K(T)</td><td></td><td>133.62</td><td>584.3049</td><td>1750.874</td><td>0.0256</td><td>14.6</td><td>4.25</td><td>18909</td><td>6.21</td><td>HUMAN</td><td>Q969H8</td><td>UPF0556 protein C19orf10</td></tr>
<tr><td>1685</td><td>dirMS_041712_NAR_20mM_plus.18729.18729.2</td><td>2</td><td>16.8</td><td>0</td><td>2.00E+05</td><td></td><td>(R)VFIGNLN</td><td>(R)V/F|I/G/N L|N T L V/V/K(K)</td><td></td><td>200.13</td><td>658.8999</td><td>1316.794</td><td>-0.0011</td><td>-0.9</td><td>8.72</td><td>15791.7</td><td>9.22</td><td>HUMAN</td><td>B4DSU6</td><td>Heterogeneous nuclear ribonucleoproteins C1/C2</td></tr>
<tr><td>1686</td><td>dirMS_041712_NAR_30mM_plus2.17969.18269.2</td><td>2</td><td>16.8</td><td>0</td><td>6.54E+05</td><td></td><td>(R)EQLAIAE</td><td>(R)E Q|L|A|I/A/E/F/A/R(S)</td><td></td><td>148.3</td><td>574.3083</td><td>1147.611</td><td>-0.0013</td><td>-1.1</td><td>4.53</td><td>44227.3</td><td>7.81</td><td>HUMAN</td><td>E7EQR6</td><td>T-complex protein 1 subunit alpha</td></tr>
<tr><td>1687</td><td>dirMS_041712_NAR_40mM_plus2.18107.18107.3</td><td>3</td><td>16.8</td><td>0</td><td>9.77E+04</td><td>M159m</td><td>(R)SVAAIHI</td><td>(R)S V|A\A I/H/P S L E\I P m L I/P K(E)</td><td>m:Oxidized methionine</td><td>172.8</td><td>611.3472</td><td>1816.04</td><td>15.9869</td><td>-4.4</td><td>6.47</td><td>31606.3</td><td>6.72</td><td>HUMAN</td><td>B4E329</td><td>Phosphoribosyl pyrophosphate synthase-associated protein 2</td></tr>
<tr><td>1688</td><td>dirMS_041712_NAR_50mM_plus2.19469.19469.3</td><td>3</td><td>16.8</td><td>1</td><td>6.92E+05</td><td></td><td>(K)AIDLFTD</td><td>(K)A I|D L/F T D/A I/K/L N/P R(L)</td><td></td><td>170.07</td><td>529.6346</td><td>1586.89</td><td>-0.0008</td><td>-0.5</td><td>6</td><td>18141.9</td><td>4.7</td><td>HUMAN</td><td>F6VDH7</td><td>Hsc70-interacting protein (Fragment)</td></tr>
<tr><td>1689</td><td>dirMS_041712_NAR_50mM_plus2.20968.21319.3</td><td>3</td><td>16.8</td><td>0</td><td>8.90E+06</td><td></td><td>(R)LHFFMP</td><td>(R)L H\F\F\M\P G\F|A|P|L/T S R(G)</td><td></td><td>181.53</td><td>540.9498</td><td>1620.835</td><td>-0.0007</td><td>-0.4</td><td>9.75</td><td>42230.8</td><td>4.77</td><td>HUMAN</td><td>A6NKZ8</td><td>Putative tubulin beta chain-like protein ENSP00000290377</td></tr>
<tr><td>1690</td><td>dirMS_041712_NAR_60mM_plus.17932.17997.3</td><td>3</td><td>16.8</td><td>1</td><td>4.10E+05</td><td></td><td>(K)ESQAKD</td><td>(K)E S Q/A K D\V|I/E/E/Y F/K(C)</td><td></td><td>151.17</td><td>529.2635</td><td>1585.774</td><td>0.0015</td><td>1</td><td>4.41</td><td>14914.1</td><td>6.84</td><td>HUMAN</td><td>P25398</td><td>40S ribosomal protein S12</td></tr>
<tr><td>1691</td><td>dirMS_041712_NAR_30mM_minus2.9449.9449.2</td><td>2</td><td>16.79</td><td>0</td><td>1.21E+06</td><td></td><td>(K)VFGPGV</td><td>(K)V/F|G/P/G V\E R(S)</td><td></td><td>105.38</td><td>430.7364</td><td>860.462</td><td>0.003</td><td>3.5</td><td>5.97</td><td>258215.9</td><td>5.48</td><td>HUMAN</td><td>E7EN95</td><td>Filamin-B</td></tr>
<tr><td>1692</td><td>dirMS_041712_NAR_60mM_minus.13519.13625.3</td><td>3</td><td>16.79</td><td>0</td><td>6.79E+05</td><td></td><td>(K)YYVTIID/</td><td>(K)Y Y|V/T/I/I/D/A P G H R(D)</td><td></td><td>135.67</td><td>468.9136</td><td>1404.727</td><td>-0.0008</td><td>-0.6</td><td>6.74</td><td>12137.8</td><td>9.3</td><td>HUMAN</td><td>A6PW80</td><td>Elongation factor 1-alpha 1 (Fragment)</td></tr>
<tr><td>1693</td><td>dirMS_041712_NAR_30mM_plus2.12834.12834.2</td><td>2</td><td>16.79</td><td>0</td><td>3.14E+05</td><td></td><td>(K)QQIQSI(</td><td>(K)Q Q|I/Q/S I/Q/Q S I/E/R(L)</td><td></td><td>120.75</td><td>729.3893</td><td>1457.771</td><td>0.0006</td><td>0.4</td><td>6</td><td>14855.1</td><td>9.26</td><td>HUMAN</td><td>B1AHD1</td><td>NHP2 non-histone chromosome protein 2-like 1 (S. cerevisiae)</td></tr>
<tr><td>1694</td><td>dirMS_041712_NAR_50mM_plus2.16993.16993.3</td><td>3</td><td>16.79</td><td>1</td><td>1.06E+06</td><td></td><td>(K)LFTAESL</td><td>(K)L/F/T/A E S L I/G L K/N P E K(S)</td><td></td><td>157.68</td><td>553.9791</td><td>1659.932</td><td>-0.0089</td><td>-5.3</td><td>6.14</td><td>62195.8</td><td>5.63</td><td>HUMAN</td><td>B0YIW6</td><td>Archain 1, isoform CRA_a</td></tr>
<tr><td>1695</td><td>dirMS_041712_NAR_60mM_plus.9537.9895.3</td><td>3</td><td>16.79</td><td>1</td><td>3.28E+06</td><td></td><td>(K)LEAEIAT</td><td>(K)L/E|A|E/I/A T Y R/R(L)</td><td></td><td>108.77</td><td>407.889</td><td>1221.659</td><td>-0.0062</td><td>-5</td><td>6.14</td><td>43773.3</td><td>5.26</td><td>HUMAN</td><td>F8VZY9</td><td>Keratin, type I cytoskeletal 18</td></tr>
<tr><td>1696</td><td>dirMS_041712_NAR_120mM_minus.5438.5554.2</td><td>2</td><td>16.78</td><td>0</td><td>5.16E+06</td><td>M49m</td><td>(R)HQGVM</td><td>(R)H/Q|G V|M|V|G m/G Q|K(D)</td><td>m:Oxidized methionine</td><td>83.28</td><td>594.2863</td><td>1171.571</td><td>15.9943</td><td>-0.5</td><td>8.76</td><td>32616.5</td><td>5.18</td><td>HUMAN</td><td>A6NL76</td><td>Actin, alpha skeletal muscle</td></tr>
<tr><td>1697</td><td>dirMS_041712_NAR_50mM_minus2.9303.9303.3</td><td>3</td><td>16.78</td><td>0</td><td>2.64E+05</td><td>M167m</td><td>(R)ImLFTNI</td><td>(R)I m/L\F/T/N E D N P H|G N D/S/A/K(A)</td><td>m:Oxidized methionine</td><td>121.37</td><td>640.2914</td><td>1902.865</td><td>15.9946</td><td>-0.2</td><td>4.54</td><td>64302.5</td><td>6.4</td><td>HUMAN</td><td>B1AHC7</td><td>X-ray repair complementing defective repair in Chinese hamster cells 6 (Ku autoantigen, 70kDa)</td></tr>
<tr><td>1698</td><td>dirMS_041712_NAR_60mM_minus.14428.14428.3</td><td>3</td><td>16.78</td><td>1</td><td>2.64E+05</td><td></td><td>(R)VEVERD</td><td>(R)V/E|V/E R D N\L|A/E D I M R(L)</td><td></td><td>139.98</td><td>563.6139</td><td>1688.827</td><td>-0.0001</td><td>0</td><td>4.18</td><td>49709.6</td><td>5.19</td><td>HUMAN</td><td>B0YJC4</td><td>Vimentin</td></tr>
<tr><td>1699</td><td>dirMS_041712_NAR_60mM_minus.17398.17625.3</td><td>3</td><td>16.78</td><td>1</td><td>4.55E+06</td><td></td><td>(R)VSFELFA</td><td>(R)V/S|F/E/L/F A D K\V/P K(T)</td><td></td><td>157.68</td><td>460.5911</td><td>1379.757</td><td>0.0018</td><td>1.3</td><td>6.04</td><td>13378.8</td><td>6.27</td><td>HUMAN</td><td>C9J5S7</td><td>Peptidyl-prolyl cis-trans isomerase</td></tr>
<tr><td>1700</td><td>dirMS_041712_NAR_20mM_plus.15649.15751.2</td><td>2</td><td>16.78</td><td>0</td><td>5.10E+05</td><td></td><td>(R)GVVDSE</td><td>(R)G V V/D|S|E|D|I|P L N/L/S R(E)</td><td></td><td>179.4</td><td>757.3956</td><td>1513.786</td><td>-0.0017</td><td>-1.1</td><td>4.03</td><td>74494.3</td><td>7.79</td><td>HUMAN</td><td>F5H897</td><td>Heat shock protein 75 kDa, mitochondrial</td></tr>
<tr><td>1701</td><td>dirMS_041712_NAR_20mM_plus.18631.18631.2</td><td>2</td><td>16.78</td><td>0</td><td>1.56E+05</td><td></td><td>(R)IITLTGPT</td><td>(R)I\I/T/L/T/G|P T N A I F K(A)</td><td></td><td>200.3</td><td>694.9091</td><td>1388.815</td><td>-0.0039</td><td>-2.8</td><td>8.75</td><td>38010.7</td><td>6.67</td><td>HUMAN</td><td>Q15365</td><td>Poly(rC)-binding protein 1</td></tr>
<tr><td>1702</td><td>dirMS_041712_NAR_20mM_plus.8910.8910.2</td><td>2</td><td>16.78</td><td>0</td><td>1.30E+05</td><td></td><td>(K)VLEEAN(</td><td>(K)V L|E/E|A/N Q\A|I/N/P/K(L)</td><td></td><td>131.6</td><td>663.353</td><td>1325.706</td><td>-0.0072</td><td>-5.5</td><td>4.53</td><td>73202</td><td>9.19</td><td>HUMAN</td><td>C9JMU5</td><td>Probable ATP-dependent RNA helicase DDX17</td></tr>
<tr><td>1703</td><td>dirMS_041712_NAR_50mM_plus2.17552.17552.3</td><td>3</td><td>16.78</td><td>1</td><td>5.87E+05</td><td></td><td>(R)LNRLPA/</td><td>(R)L N R\L P A A/G\V|G|D/M|V/M A T V K(K)</td><td></td><td>160.33</td><td>614.9984</td><td>1842.993</td><td>-0.0122</td><td>-6.6</td><td>8.75</td><td>12270.4</td><td>11.49</td><td>HUMAN</td><td>B9ZVP7</td><td>60S ribosomal protein L23</td></tr>
<tr><td>1704</td><td>dirMS_041712_NAR_60mM_plus.13954.13954.3</td><td>3</td><td>16.78</td><td>0</td><td>1.56E+06</td><td></td><td>(K)LAQANG</td><td>(K)L/A|Q/A/N/G W/G/V/M\V/S H R(S)</td><td></td><td>129.07</td><td>509.2606</td><td>1525.769</td><td>-0.002</td><td>-1.3</td><td>9.75</td><td>47510.4</td><td>7.04</td><td>HUMAN</td><td>P06733</td><td>Alpha-enolase</td></tr>
<tr><td>1705</td><td>dirMS_041712_NAR_120mM_minus.14585.14625.3</td><td>3</td><td>16.77</td><td>1</td><td>6.69E+05</td><td></td><td>(K)NKDQGT</td><td>(K)N K D Q G T\Y|E/D|Y|V/E/G L/R(V)</td><td></td><td>133.68</td><td>596.2795</td><td>1786.824</td><td>-0.0003</td><td>-0.2</td><td>4.32</td><td>26991.7</td><td>5.01</td><td>HUMAN</td><td>B7Z6Z4</td><td>Myosin light polypeptide 6</td></tr>
<tr><td>1706</td><td>dirMS_041712_NAR_120mM_minus.7924.7924.2</td><td>2</td><td>16.77</td><td>1</td><td>6.37E+04</td><td></td><td>(K)VAAYDK</td><td>(K)V/A|A/Y D K L E K(T)</td><td></td><td>97.5</td><td>518.7878</td><td>1036.567</td><td>0.001</td><td>1</td><td>6.04</td><td>227783.5</td><td>5.5</td><td>HUMAN</td><td>P35579</td><td>Myosin-9</td></tr>
<tr><td>1707</td><td>dirMS_041712_NAR_120mM_minus.9962.9999.3</td><td>3</td><td>16.77</td><td>1</td><td>1.83E+06</td><td></td><td>(K)LEAEIAT</td><td>(K)L/E|A/E/I/A T Y R/R(L)</td><td></td><td>109.02</td><td>407.8893</td><td>1221.659</td><td>-0.0053</td><td>-4.3</td><td>6.14</td><td>43773.3</td><td>5.26</td><td>HUMAN</td><td>F8VZY9</td><td>Keratin, type I cytoskeletal 18</td></tr>
<tr><td>1708</td><td>dirMS_041712_NAR_20mM_minus.14657.14657.2</td><td>2</td><td>16.77</td><td>0</td><td>2.10E+05</td><td></td><td>(K)LAVEALS</td><td>(K)L A V E|A|L/S/S L/D/G D/L A G/R(Y)</td><td></td><td>201.28</td><td>793.922</td><td>1586.838</td><td>-0.0017</td><td>-1.1</td><td>4.03</td><td>24286.7</td><td>4.89</td><td>HUMAN</td><td>G3V4N7</td><td>Creatine kinase B-type (Fragment)</td></tr>
<tr><td>1709</td><td>dirMS_041712_NAR_30mM_plus2.12173.12320.2</td><td>2</td><td>16.77</td><td>0</td><td>5.52E+05</td><td></td><td>(R)AQPSVS</td><td>(R)A Q|P/S/V|S/L|G A A Y/R(A)</td><td></td><td>117.42</td><td>610.3242</td><td>1219.643</td><td>-0.0018</td><td>-1.5</td><td>8.79</td><td>69661.6</td><td>9.75</td><td>HUMAN</td><td>Q96PK6</td><td>RNA-binding protein 14</td></tr>
<tr><td>1710</td><td>dirMS_041712_NAR_40mM_plus2.17737.17737.3</td><td>3</td><td>16.77</td><td>0</td><td>3.79E+04</td><td></td><td>(R)AGLGSG</td><td>(R)A G/L/G/S/G L/S L/S G L/V/H P E\L S R(S)</td><td></td><td>170.98</td><td>617.3431</td><td>1850.013</td><td>0.0017</td><td>0.9</td><td>6.79</td><td>73325.2</td><td>9.19</td><td>HUMAN</td><td>F5H2A9</td><td>Very long-chain-specific acyl-CoA dehydrogenase, mitochondrial</td></tr>
<tr><td>1711</td><td>dirMS_041712_NAR_40mM_plus2.17749.17749.3</td><td>3</td><td>16.77</td><td>1</td><td>1.96E+05</td><td></td><td>(R)LKLPSIPI</td><td>(R)L K L|P S I|P L\V/P V/S A Q K(R)</td><td></td><td>170.73</td><td>530.671</td><td>1589.999</td><td>-0.0004</td><td>-0.3</td><td>10</td><td>34958.1</td><td>9.42</td><td>HUMAN</td><td>Q15785</td><td>Mitochondrial import receptor subunit TOM34</td></tr>
<tr><td>1712</td><td>dirMS_041712_NAR_50mM_plus2.21906.22110.3</td><td>3</td><td>16.77</td><td>1</td><td>8.66E+06</td><td></td><td>(R)KEGGLG</td><td>(R)K\E G G L G\P L\N\I|P/L/L/A/D/V T R(R)</td><td></td><td>188.23</td><td>621.6925</td><td>1863.07</td><td>-0.0069</td><td>-3.7</td><td>6.07</td><td>15252.2</td><td>5.82</td><td>HUMAN</td><td>A6NIW5</td><td>Peroxiredoxin 2, isoform CRA_a</td></tr>
<tr><td>1713</td><td>dirMS_041712_NAR_30mM_minus2.5897.5897.2</td><td>2</td><td>16.76</td><td>0</td><td>5.74E+04</td><td></td><td>(R)LAEAEET</td><td>(R)L/A|E/A|E E T/A R(T)</td><td></td><td>86.62</td><td>495.2478</td><td>989.49</td><td>-0.0015</td><td>-1.5</td><td>4.25</td><td>67732.1</td><td>4.71</td><td>HUMAN</td><td>G3V119</td><td>DBIRD complex subunit KIAA1967</td></tr>
<tr><td>1714</td><td>dirMS_041712_NAR_120mM_plus.11467.11488.3</td><td>3</td><td>16.76</td><td>2</td><td>7.36E+05</td><td></td><td>(K)QAGPAS</td><td>(K)Q/A|G/P/A/S/V/P L R T E E E F\K K(F)</td><td></td><td>113.4</td><td>629.6702</td><td>1886.997</td><td>-0.001</td><td>-0.5</td><td>6.23</td><td>55361.8</td><td>6.42</td><td>HUMAN</td><td>G5EA52</td><td>Protein disulfide isomerase family A, member 3, isoform CRA_b</td></tr>
<tr><td>1715</td><td>dirMS_041712_NAR_30mM_plus2.19571.19873.2</td><td>2</td><td>16.76</td><td>0</td><td>8.13E+05</td><td></td><td>(-)MDELQL</td><td>(-)M/D|E|L|Q/L/F/R(G)</td><td></td><td>156.65</td><td>526.2726</td><td>1051.524</td><td>0.0138</td><td>13.2</td><td>4.37</td><td>18601</td><td>5.1</td><td>HUMAN</td><td>C9IZA5</td><td>Transitional endoplasmic reticulum ATPase (Fragment)</td></tr>
<tr><td>1716</td><td>dirMS_041712_NAR_60mM_minus.8963.8963.2</td><td>2</td><td>16.75</td><td>0</td><td>9.67E+05</td><td></td><td>(K)QEYDES(</td><td>(K)Q/E|Y|D|E S/G P S I/V/H|R(K)</td><td></td><td>110.35</td><td>758.8539</td><td>1516.703</td><td>-0.0021</td><td>-1.4</td><td>4.65</td><td>123097.1</td><td>5.83</td><td>HUMAN</td><td>A5A3E0</td><td>POTE ankyrin domain family member F</td></tr>
<tr><td>1717</td><td>dirMS_041712_NAR_20mM_plus.18645.18645.2</td><td>2</td><td>16.75</td><td>0</td><td>2.00E+05</td><td></td><td>(R)VFIGNLN</td><td>(R)V/F|I/G\N L/N T L\V V K(K)</td><td></td><td>200.13</td><td>658.8999</td><td>1316.794</td><td>-0.0011</td><td>-0.9</td><td>8.72</td><td>15791.7</td><td>9.22</td><td>HUMAN</td><td>B4DSU6</td><td>Heterogeneous nuclear ribonucleoproteins C1/C2</td></tr>
<tr><td>1718</td><td>dirMS_041712_NAR_50mM_plus2.11381.11399.3</td><td>3</td><td>16.75</td><td>0</td><td>4.19E+05</td><td></td><td>(K)GFVEFA/</td><td>(K)G F|V/E/F/A/A/K/P P A R(K)</td><td></td><td>127.83</td><td>430.5716</td><td>1289.7</td><td>0.0002</td><td>0.1</td><td>8.75</td><td>58856.7</td><td>6.26</td><td>HUMAN</td><td>Q8WXF1</td><td>Paraspeckle component 1</td></tr>
<tr><td>1719</td><td>dirMS_041712_NAR_50mM_plus2.16543.16924.3</td><td>3</td><td>16.75</td><td>1</td><td>6.78E+06</td><td></td><td>(K)QLDDLK'</td><td>(K)Q L/D/D/L/K/V/E/L/S/Q L R(V)</td><td></td><td>155.75</td><td>519.6261</td><td>1556.864</td><td>-0.0005</td><td>-0.3</td><td>4.56</td><td>10815.7</td><td>10.72</td><td>HUMAN</td><td>F2Z388</td><td>60S ribosomal protein L35</td></tr>
<tr><td>1720</td><td>dirMS_041712_NAR_60mM_plus.7196.7373.3</td><td>3</td><td>16.75</td><td>1</td><td>8.06E+05</td><td></td><td>(R)IQFKQD</td><td>(R)I/Q/F/K/Q/D D/G/T/G P E/K(I)</td><td></td><td>96.18</td><td>488.2441</td><td>1462.717</td><td>0.0005</td><td>0.3</td><td>4.56</td><td>73399.3</td><td>6.86</td><td>HUMAN</td><td>Q92945</td><td>Far upstream element-binding protein 2</td></tr>
<tr><td>1721</td><td>dirMS_041712_NAR_40mM_minus2.11103.11103.3</td><td>3</td><td>16.73</td><td>0</td><td>7.92E+05</td><td></td><td>(R)QVSASE</td><td>(R)Q V|S A/S E L H/T/S/G I L/G/P E T L R(D)</td><td></td><td>148.75</td><td>665.6822</td><td>1995.051</td><td>-0.0185</td><td>-9.3</td><td>5.4</td><td>558007.1</td><td>5.44</td><td>HUMAN</td><td>P58107</td><td>Epiplakin</td></tr>
<tr><td>1722</td><td>dirMS_041712_NAR_50mM_minus2.18081.18176.3</td><td>3</td><td>16.73</td><td>1</td><td>6.74E+05</td><td></td><td>(R)VLEALLP</td><td>(R)V/L|E/A/L L/P/L/K G L E E/R(V)</td><td></td><td>172.47</td><td>527.3171</td><td>1579.942</td><td>-0.005</td><td>-3.2</td><td>4.79</td><td>276046.1</td><td>6.01</td><td>HUMAN</td><td>P49327</td><td>Fatty acid synthase</td></tr>
<tr><td>1723</td><td>dirMS_041712_NAR_50mM_minus2.18373.18373.3</td><td>3</td><td>16.73</td><td>0</td><td>4.62E+05</td><td></td><td>(K)ALIEVLQ</td><td>(K)A L|I/E/V|L Q/P L I/A E/H Q A/R(R)</td><td></td><td>175.42</td><td>601.0139</td><td>1801.033</td><td>-0.0059</td><td>-3.3</td><td>5.4</td><td>53506.8</td><td>5.83</td><td>HUMAN</td><td>P23381</td><td>Tryptophan--tRNA ligase, cytoplasmic</td></tr>
<tr><td>1724</td><td>dirMS_041712_NAR_30mM_plus2.13814.13837.2</td><td>2</td><td>16.73</td><td>0</td><td>7.43E+05</td><td></td><td>(R)GDFCIQ'</td><td>(R)G D/F/C|I|Q/V|G R(-)</td><td>C:Carbamidomethylation</td><td>125.57</td><td>526.2542</td><td>1051.499</td><td>0.0022</td><td>2.1</td><td>5.84</td><td>15431.7</td><td>9</td><td>HUMAN</td><td>E7ERL0</td><td>Nucleoside diphosphate kinase A</td></tr>
<tr><td>1725</td><td>dirMS_041712_NAR_30mM_plus2.20504.20504.2</td><td>2</td><td>16.73</td><td>0</td><td>1.71E+05</td><td></td><td>(K)GSFSEQ(</td><td>(K)G S/F/S E/Q|G\I/N\E/F L/R(E)</td><td></td><td>161.05</td><td>742.362</td><td>1483.718</td><td>-0.0009</td><td>-0.6</td><td>4.53</td><td>53659.5</td><td>5.35</td><td>HUMAN</td><td>B5MCQ5</td><td>Protein disulfide-isomerase A6</td></tr>
<tr><td>1726</td><td>dirMS_041712_NAR_30mM_plus2.5695.5695.2</td><td>2</td><td>16.73</td><td>0</td><td>2.46E+05</td><td></td><td>(R)QSSGAS</td><td>(R)Q S\S/G/A|S/S/S S F/S S S R(A)</td><td></td><td>84.3</td><td>681.3013</td><td>1361.593</td><td>0.0025</td><td>1.9</td><td>9.75</td><td>73641.5</td><td>6.74</td><td>HUMAN</td><td>O00571</td><td>ATP-dependent RNA helicase DDX3X</td></tr>
<tr><td>1727</td><td>dirMS_041712_NAR_50mM_plus2.14703.14799.3</td><td>3</td><td>16.73</td><td>1</td><td>1.32E+06</td><td></td><td>(K)VIDPATA</td><td>(K)V/I|D|P/A/T A/T/S V D L R D/I/K(I)</td><td></td><td>145.65</td><td>571.9848</td><td>1713.938</td><td>0.0017</td><td>1</td><td>4.43</td><td>42755</td><td>7.81</td><td>HUMAN</td><td>B7Z2F4</td><td>T-complex protein 1 subunit delta</td></tr>
<tr><td>1728</td><td>dirMS_041712_NAR_120mM_minus.11966.12066.3</td><td>3</td><td>16.72</td><td>1</td><td>5.17E+05</td><td></td><td>(R)ARQEEL'</td><td>(R)A R Q E E\L|Y|S|E|L/Q/A R(E)</td><td></td><td>119.52</td><td>531.6052</td><td>1592.803</td><td>-0.0017</td><td>-1</td><td>4.79</td><td>533778</td><td>5.74</td><td>HUMAN</td><td>Q15149</td><td>Plectin</td></tr>
<tr><td>1729</td><td>dirMS_041712_NAR_30mM_minus2.16608.17015.2</td><td>2</td><td>16.72</td><td>0</td><td>1.63E+05</td><td></td><td>(R)FFMGN(</td><td>(R)F/F|M/G/N/Q V/L/K(V)</td><td></td><td>146.13</td><td>542.2866</td><td>1083.566</td><td>0.0004</td><td>0.3</td><td>8.75</td><td>83273.6</td><td>5.55</td><td>HUMAN</td><td>P13010</td><td>X-ray repair cross-complementing protein 5</td></tr>
<tr><td>1730</td><td>dirMS_041712_NAR_30mM_minus2.7410.7410.2</td><td>2</td><td>16.72</td><td>0</td><td>2.50E+03</td><td></td><td>(R)TPEEYPE</td><td>(R)T/P/E/E/Y/P E S/A/K(V)</td><td></td><td>93.4</td><td>575.7705</td><td>1150.526</td><td>0.0075</td><td>6.5</td><td>4.25</td><td>21580.5</td><td>5.38</td><td>HUMAN</td><td>B4DGT0</td><td>N(G),N(G)-dimethylarginine dimethylaminohydrolase 1</td></tr>
<tr><td>1731</td><td>dirMS_041712_NAR_40mM_minus2.16006.16013.3</td><td>3</td><td>16.72</td><td>0</td><td>1.60E+06</td><td>M166m</td><td>(K)VLDFEHI</td><td>(K)V/L|D\F E/H F\L|P m/L Q/T V A K(N)</td><td>m:Oxidized methionine</td><td>187.72</td><td>635.3376</td><td>1888.004</td><td>15.9945</td><td>-0.2</td><td>5.32</td><td>26991.7</td><td>5.01</td><td>HUMAN</td><td>B7Z6Z4</td><td>Myosin light polypeptide 6</td></tr>
<tr><td>1732</td><td>dirMS_041712_NAR_50mM_minus2.12163.12204.3</td><td>3</td><td>16.72</td><td>1</td><td>2.39E+05</td><td></td><td>(K)LIADVAF</td><td>(K)L/I|A/D/V/A/P S A I R E N D/I/K(S)</td><td></td><td>137.7</td><td>575.6545</td><td>1724.954</td><td>-0.0052</td><td>-3</td><td>4.56</td><td>18388.8</td><td>6.85</td><td>HUMAN</td><td>F5H1Y3</td><td>Flap endonuclease 1 (Fragment)</td></tr>
<tr><td>1733</td><td>dirMS_041712_NAR_50mM_minus2.9183.9345.3</td><td>3</td><td>16.72</td><td>0</td><td>3.48E+05</td><td></td><td>(R)TIVITSHI</td><td>(R)T I|V|I/T/S H/P G Q I V/K(H)</td><td></td><td>120.98</td><td>464.9452</td><td>1392.821</td><td>0.0001</td><td>0.1</td><td>8.44</td><td>27999.4</td><td>5.61</td><td>HUMAN</td><td>B7Z5C0</td><td>DnaJ homolog subfamily A member 1</td></tr>
<tr><td>1734</td><td>dirMS_041712_NAR_40mM_plus2.18608.18666.3</td><td>3</td><td>16.72</td><td>1</td><td>4.00E+05</td><td></td><td>(R)TLAEIAK</td><td>(R)T/L\A/E/I/A/K/V E\L/D/N M/P L R(G)</td><td></td><td>176.57</td><td>604.9967</td><td>1812.989</td><td>-0.0132</td><td>-7.3</td><td>4.68</td><td>23729.5</td><td>9.89</td><td>HUMAN</td><td>C9IZL7</td><td>Non-POU domain-containing octamer-binding protein (Fragment)</td></tr>
<tr><td>1735</td><td>dirMS_041712_NAR_60mM_plus.13848.13864.3</td><td>3</td><td>16.72</td><td>1</td><td>8.35E+05</td><td></td><td>(R)IAWDDE</td><td>(R)I/A|W|D/D/E E/T R D/G F/R(L)</td><td></td><td>129.13</td><td>537.2476</td><td>1609.724</td><td>0.0041</td><td>2.6</td><td>4.11</td><td>80685.1</td><td>7.31</td><td>HUMAN</td><td>P05165</td><td>Propionyl-CoA carboxylase alpha chain, mitochondrial</td></tr>
<tr><td>1736</td><td>dirMS_041712_NAR_120mM_minus.12140.12178.3</td><td>3</td><td>16.71</td><td>0</td><td>1.58E+06</td><td></td><td>(R)HVFGES</td><td>(R)H V\F\G E|S/D|E|L|I/G Q/K(V)</td><td></td><td>120.68</td><td>486.9119</td><td>1458.722</td><td>-0.0012</td><td>-0.8</td><td>4.65</td><td>31075.7</td><td>5.65</td><td>HUMAN</td><td>P60174</td><td>Triosephosphate isomerase</td></tr>
<tr><td>1737</td><td>dirMS_041712_NAR_30mM_minus2.20229.20242.2</td><td>2</td><td>16.71</td><td>0</td><td>6.49E+05</td><td></td><td>(K)IGFPWSI</td><td>(K)I/G|F|P/W/S E I/R(N)</td><td></td><td>171.88</td><td>552.7932</td><td>1104.584</td><td>-0.0045</td><td>-4.1</td><td>6</td><td>71218.8</td><td>6.3</td><td>HUMAN</td><td>A7YIJ8</td><td>Radixin</td></tr>
<tr><td>1738</td><td>dirMS_041712_NAR_40mM_minus2.11001.11001.3</td><td>3</td><td>16.71</td><td>0</td><td>4.81E+05</td><td></td><td>(R)DAGEGL</td><td>(R)D A|G\E/G L/L A V Q I/T/D Q E G K P K(R)</td><td></td><td>148</td><td>657.0157</td><td>1969.024</td><td>0.0089</td><td>4.5</td><td>4.32</td><td>258215.9</td><td>5.48</td><td>HUMAN</td><td>E7EN95</td><td>Filamin-B</td></tr>
<tr><td>1739</td><td>dirMS_041712_NAR_60mM_minus.12088.12088.3</td><td>3</td><td>16.71</td><td>1</td><td>3.03E+06</td><td>M72m</td><td>(R)TEmENE</td><td>(R)T/E/m/E N E F/V/L/I/K K(D)</td><td>m:Oxidized methionine</td><td>126.87</td><td>499.5941</td><td>1480.772</td><td>15.9962</td><td>0.8</td><td>4.78</td><td>13588.1</td><td>4.66</td><td>HUMAN</td><td>F8VP67</td><td>Keratin, type II cytoskeletal 8 (Fragment)</td></tr>
</table>

1740	dirMS_041712_NAR_60mM_minus.13253.13253.3	3	16.71	1	2.81E+05	M194m	(R)KLQEEEL	(R)K L Q E E E L L|A|T/Q/V/D|m R(A)	m:Oxidized methionine	133.93	606.9793	1802.932	15.9917	-1.8	4.41	35935.5	6.2	HUMAN	Q8IVM0	Coiled-coil domain-containing protein 50
1741	dirMS_041712_NAR_50mM_plus2.14846.14889.3	3	16.71	1	9.00E+05		(R)LYSILGT	(R)L/Y|S/I/L/G T T L/K D E/G K(L)		145.9	513.2859	1537.847	-0.0041	-2.6	6.07	66876.9	6.17	HUMAN	O75083	WD repeat-containing protein 1
1742	dirMS_041712_NAR_50mM_plus2.15994.16043.3	3	16.71	1	1.41E+06		(R)VCENIPI	(R)V/C/E|N|I|P I V L/C G N K V D|I/K(D)	C:Carbamidomethylation	152.08	657.6802	1971.04	-0.0141	-7.2	6.04	14844.8	5.89	HUMAN	B4DV51	GTP-binding nuclear protein Ran
1743	dirMS_041712_NAR_120mM_plus.10044.10539.3	3	16.7	1	1.60E+07		(R)VKYETEL	(R)V/K Y E T|E|L/A/M/R(Q)		107.58	413.8849	1239.64	0	0	6.11	43773.3	5.26	HUMAN	F8VZY9	Keratin, type I cytoskeletal 18
1744	dirMS_041712_NAR_60mM_plus.19435.19435.3	3	16.7	1	5.59E+05		(K)TVTAMD	(K)T/V|T/A/M D/V/V/Y A L K R(Q)		160.4	489.6071	1466.804	0.0032	2.2	8.25	11367.1	11.36	HUMAN	P62805	Histone H4
1745	dirMS_041712_NAR_120mM_minus.7830.8060.3	3	16.69	1	9.87E+05		(K)IQDKEGI	(K)I Q/D\K E G|I|P/P/D Q Q R(L)		97.6	508.5981	1523.781	-0.0015	-1	4.56	17271.6	6.75	HUMAN	B4DV12	Ubiquitin
1746	dirMS_041712_NAR_50mM_minus2.14963.14963.3	3	16.69	0	4.22E+05		(K)IAILTCPF	(K)I/A/I|L/T C\P F E/P P K/P K(T)	C:Carbamidomethylation	153.37	537.6375	1610.898	0.0005	0.3	8.59	49982	5.86	HUMAN	B4DYD8	T-complex protein 1 subunit epsilon
1747	dirMS_041712_NAR_50mM_plus2.19264.19400.3	3	16.69	1	3.94E+05		(K)ILDVASL	(K)I/L|D|V/A/S/L/E/V L N E V N R R(S)		169.97	614.0109	1840.029	-0.0105	-5.7	4.68	295149.3	7.4	HUMAN	Q92616	Translational activator GCN1
1748	dirMS_041712_NAR_50mM_plus2.20255.20368.3	3	16.69	0	1.29E+05		(R)AFIDPLG	(R)A F|I|D/P/L/G/L/P D|R P F Y R(H)		175.4	592.986	1776.943	0.0003	0.2	6	84557.3	6.5	HUMAN	Q04609	Glutamate carboxypeptidase 2
1749	dirMS_041712_NAR_30mM_minus2.7668.7668.2	2	16.68	0	9.48E+04		(R)FYQQVT	(R)F/Y|Q/Q|V T/K(D)		96.08	457.2426	913.478	0.0001	0.1	8.59	253486.5	5.51	HUMAN	J3KNL6	Protein transport protein Sec16A
1750	dirMS_041712_NAR_120mM_plus.8540.8943.3	3	16.68	0	2.74E+06		(K)HTGPNS	(K)H T G P N\S P/D|T|A/N/D/G/F V/R(L)		99.25	562.26	1684.767	-0.0019	-1.2	5.21	18511.8	5.62	HUMAN	D6R9T0	Heterogeneous nuclear ribonucleoprotein H (Fragment)
1751	dirMS_041712_NAR_20mM_plus.15501.15825.3	3	16.68	0	7.37E+05		(K)VGINYQ	(K)V G I N Y Q|P P T\V|V|P/G G D L A K(V)		179.22	608.9991	1824.985	-0.0027	-1.5	5.8	49012.3	4.9	HUMAN	A8MUB1	Tubulin alpha-4A chain
1752	dirMS_041712_NAR_20mM_plus.9664.9924.2	2	16.68	0	2.26E+05		(K)IVVVTA	(K)I/V|V|V|T/A/G/V/R(Q)		139.2	457.2945	913.583	-0.0012	-1.3	9.75	25445.8	5.52	HUMAN	A8MW50	L-lactate dehydrogenase (Fragment)
1753	dirMS_041712_NAR_30mM_plus2.18477.18497.2	2	16.68	0	5.13E+05		(R)GFVDDII	(R)G F|V/D|D|I|I|Q|P S S T R(A)		150.37	717.8617	1434.722	-0.0062	-4.3	4.21	61363	8.73	HUMAN	C9JQS9	Propionyl-CoA carboxylase beta chain, mitochondrial
1754	dirMS_041712_NAR_60mM_plus.11426.11577.3	3	16.68	1	1.25E+06		(K)FGQYNK	(K)F/G|Q|Y/N K/E D|P T/S F R(L)		117.23	530.2507	1588.739	-0.0015	-0.9	6.07	87447.6	6.44	HUMAN	Q15437	Protein transport protein Sec23B
1755	dirMS_041712_NAR_20mM_plus.18158.18158.2	2	16.67	0	1.70E+05		(R)LTGQLFI	(R)L T|G Q/L/F/L G G S I/V/K(G)		197.02	666.8962	1332.789	-0.0034	-2.6	8.75	45924.5	5.29	HUMAN	B4E1F3	Selenium-binding protein 1
1756	dirMS_041712_NAR_30mM_plus2.12257.12257.2	2	16.67	0	2.21E+05		(K)VLQATV	(K)V L/Q|A/T/V|V/A V/G S G S K(G)		117.57	658.3806	1315.758	-0.0041	-3.1	8.72	10803.5	9.82	HUMAN	B8ZZL8	10 kDa heat shock protein, mitochondrial
1757	dirMS_041712_NAR_30mM_plus2.13994.14284.2	2	16.67	0	1.37E+06		(R)NLVDNI	(R)N L|V|D|N/I|T/G Q/R(L)		127.05	565.3018	1129.596	0.0003	0.3	5.84	533778	5.74	HUMAN	Q15149	Plectin
1758	dirMS_041712_NAR_30mM_plus2.19827.19870.2	2	16.67	0	2.35E+05		(K)GFAFVQ	(K)G F|A|F|V|Q/Y V/N E R(N)		157.57	665.3326	1329.659	-0.0007	-0.5	6	15791.7	9.22	HUMAN	B4DSU6	Heterogeneous nuclear ribonucleoproteins C1/C2
1759	dirMS_041712_NAR_30mM_plus2.21773.21773.2	2	16.67	0	2.02E+05		(R)NSILAQ	(R)N S|I|L|A/Q\V L/D/Q S A R(A)		170.1	707.8889	1414.765	0.0057	4	5.84	15111	6.44	HUMAN	B4DE64	Programmed cell death protein 5
1760	dirMS_041712_NAR_50mM_plus2.8911.8911.3	3	16.67	0	1.99E+05		(K)VVQVSA	(K)V V|Q|V/S/A G D S H T A A L T\D D G R(V)		113.48	633.6509	1898.92	0.0179	9.4	4.41	29854.8	9.49	HUMAN	C9J3R0	Regulator of chromosome condensation (Fragment)
1761	dirMS_041712_NAR_30mM_minus2.15972.16283.2	2	16.66	0	5.16E+05		(K)LIINSLYK	(K)L/I|I|N/S/L/Y K(N)		141.27	482.2955	963.587	-0.0036	-3.8	8.59	5582.4	4.81	HUMAN	F8W026	Endoplasmin (Fragment)
1762	dirMS_041712_NAR_30mM_minus2.17025.17186.2	2	16.66	0	1.94E+05		(R)IYNVIGT	(R)I/Y|N|V|I|G/T L R(G)		146.9	524.8092	1048.615	-0.0038	-3.7	8.75	84557.3	6.5	HUMAN	Q04609	Glutamate carboxypeptidase 2
1763	dirMS_041712_NAR_30mM_minus2.18724.18901.2	2	16.66	0	2.79E+05		(R)LDFNLIR	(R)L/D|F/N/L/I/R(V)		157.08	445.759	890.509	0.0013	1.5	5.84	18956.4	6.97	HUMAN	C9JLK0	Phosphoribosylaminoimidazolecarboxamide formyltransferase (Fragment)
1764	dirMS_041712_NAR_60mM_minus.17201.17688.3	3	16.66	1	1.28E+07	M148m	(R)LDLAGR	(R)L/D|L|A/G R D L T/D/Y L/m K(I)	m:Oxidized methionine	157.95	547.2829	1623.841	15.9931	-1.1	4.43	37482.1	5.36	HUMAN	E9PG30	Actin, gamma-enteric smooth muscle
1765	dirMS_041712_NAR_120mM_plus.19016.19133.4	4	16.66	0	9.03E+05		(R)EVPIPEH	(R)E V P I/P/E/H\I/D/I/Y|H/L T/R(E)		153.55	458.7454	1831.97	-0.0103	-5.6	5.28	27043.5	6.34	HUMAN	C9JHK9	ATP-binding cassette sub-family F member 2 (Fragment)
1766	dirMS_041712_NAR_40mM_plus2.13530.13694.3	3	16.66	0	6.25E+05		(R)TIQVENS	(R)T/I|Q|V/E N\S H L/I/L/T/G A G A L N K(V)		146.55	660.3654	1979.092	-0.0104	-5.2	6.41	267260.7	5.95	HUMAN	Q13085	Acetyl-CoA carboxylase 1
1767	dirMS_041712_NAR_60mM_plus.6869.6869.3	3	16.66	1	7.28E+05		(R)SGAPAA	(R)S/G|A/P A/A E S/K E I V R(G)		94.78	438.904	1314.701	-0.0038	-2.9	5.87	14003.4	5.66	HUMAN	Q9NRX4	14 kDa phosphohistidine phosphatase
1768	dirMS_041712_NAR_120mM_minus.20139.20139.4	4	16.65	0	4.49E+05		(K)LAFPSPF	(K)L A|F|P/S/P F G H L S/E G F S/H S/R(W)		166.17	493.9968	1972.966	-0.0011	-0.5	6.92	16196.2	9.92	HUMAN	C9JRJ8	Sequestosome-1 (Fragment)
1769	dirMS_041712_NAR_30mM_minus2.18449.18556.2	2	16.65	0	2.28E+05		(K)YPQLLPC	(K)Y/P/Q/L|L|P G I R(G)		154.92	528.816	1056.62	0.0047	4.4	8.75	45228.3	6.03	HUMAN	F5H737	Adenosylhomocysteinase
1770	dirMS_041712_NAR_30mM_minus2.19113.19502.2	2	16.65	0	9.37E+06		(R)YLTVAA	(R)Y/L|T|V/A/A|V/F R(G)		161.02	520.2998	1039.593	-0.0012	-1.1	8.75	40069.4	4.83	HUMAN	E7EWR1	Tubulin beta chain
1771	dirMS_041712_NAR_20mM_plus.10907.10907.2	2	16.65	0	2.19E+05	M33m	(K)VAGmD	(K)V A/G|m|D/V/E L/T V E E R(N)	m:Oxidized methionine	147.28	732.3555	1447.71	15.994	-0.6	4	10949.3	5.28	HUMAN	B4DJF2	14-3-3 protein epsilon
1772	dirMS_041712_NAR_30mM_plus2.19593.19593.2	2	16.65	0	1.98E+04		(K)NQALFP	(K)N Q|A|L/F|P A C V L K(N)	C:Carbamidomethylation	157.7	630.8399	1260.677	-0.0044	-3.5	8.75	83400.6	6.82	HUMAN	Q92499	ATP-dependent RNA helicase DDX1
1773	dirMS_041712_NAR_30mM_plus2.21623.22005.2	2	16.65	0	1.49E+06		(K)EIIDLVLD	(K)E I|I/D|L|V/L/D/R(I)		169.95	543.3133	1085.62	-0.0008	-0.7	4.03	58642	4.95	HUMAN	F5H5D3	Tubulin alpha-1C chain
1774	dirMS_041712_NAR_40mM_plus2.10633.10772.3	3	16.65	0	1.77E+05		(K)MAPVPL	(K)M A|P/V/P/L D/D S N R P A S L T/K(D)		128.32	604.6467	1811.932	-0.0065	-3.6	5.71	53030.7	11.3	HUMAN	E9PJA7	Bcl-2-associated transcription factor 1
1775	dirMS_041712_NAR_50mM_plus2.20291.20567.3	3	16.65	1	4.12E+05		(R)VISGVLC	(R)V/I|S/G/V|L Q/L/G N I V/F K K(E)		176.1	539.0011	1614.994	-0.0054	-3.3	10	227783.5	5.5	HUMAN	P35579	Myosin-9
1776	dirMS_041712_NAR_60mM_plus.7442.7499.3	3	16.65	1	5.05E+05		(K)SSGGREI	(K)S/S|G/G R E D\L\E|S|S/G L Q\R(R)		97.3	526.5897	1577.751	0.0031	2	4.68	11625.5	8.01	HUMAN	B8ZZT7	Hematological and neurological-expressed 1 protein
1777	dirMS_041712_NAR_30mM_minus2.11952.12357.2	2	16.64	0	7.64E+05		(K)SIYYITGE	(K)S I|Y|Y/I/T/G E/S/K(E)		119.17	580.7949	1160.583	-0.0009	-0.7	5.72	16789.1	8.77	HUMAN	H0Y5S7	Heat shock protein HSP 90-beta (Fragment)
1778	dirMS_041712_NAR_30mM_plus2.21435.21565.2	2	16.64	0	3.50E+05		(K)FDGILGN	(K)F/D/G\I|L|G/M/A/Y|P R(I)		167.42	620.3199	1239.619	0.0135	10.9	5.83	32905.8	5.88	HUMAN	C9JH19	Cathepsin D light chain (Fragment)
1779	dirMS_041712_NAR_20mM_minus.13233.13233.2	2	16.63	0	2.34E+05		(R)GVVDSE	(R)G V/V|D/S|E/D I/P L/N L\S R(E)		183.02	757.3944	1513.786	-0.0041	-2.7	4.03	74494.3	7.79	HUMAN	F5H897	Heat shock protein 75 kDa, mitochondrial
1780	dirMS_041712_NAR_30mM_minus2.12658.12739.2	2	16.63	0	3.27E+05		(R)GGAEQF	(R)G G A|E|Q|F|M|E/E|T E/R(S)		122.7	692.2961	1383.584	0.0004	0.3	4.09	46550.7	7.81	HUMAN	B7Z1C9	Chaperonin containing TCP1, subunit 7 (Eta), isoform CRA_a
1781	dirMS_041712_NAR_30mM_minus2.13604.13643.2	2	16.63	0	3.31E+05		(R)ISGLIYEE	(R)I/S|G|L|I|Y/E E/T/R(G)		127.68	590.8127	1180.621	-0.0027	-2.3	4.53	11367.1	11.36	HUMAN	P62805	Histone H4
1782	dirMS_041712_NAR_30mM_plus2.18806.18868.2	2	16.63	0	1.53E+05		(K)FGAYIVD	(K)F/G|A|Y|I/V/D/G L/R(Q)		152.32	555.8011	1110.594	0.0007	0.6	5.83	269989.9	5.92	HUMAN	F8W8T8	Acetyl-CoA carboxylase 2
1783	dirMS_041712_NAR_60mM_plus.12101.12121.3	3	16.63	1	6.89E+05		(R)MGIVGP	(R)M/G I/V/G/P E/F K D/K(L)		120.63	407.5532	1220.634	0.0107	8.7	5.82	533778	5.74	HUMAN	Q15149	Plectin
1784	dirMS_041712_NAR_50mM_minus2.7190.7255.3	3	16.62	1	1.49E+05		(R)IAECSSQ	(R)I/A|E/C/S S Q L/A/E E/E E K/A K(N)	C:Carbamidomethylation	108.03	607.9557	1821.853	-0.0009	-0.5	4.32	233493.8	5.45	HUMAN	F8VTL3	Myosin-10
1785	dirMS_041712_NAR_60mM_minus.11637.11878.3	3	16.62	1	2.96E+06		(K)VKLEAEI	(K)V K L E A E|I|A/T/Y/R(R)		125.33	431.5779	1292.721	-0.0017	-1.3	6.11	43773.3	5.26	HUMAN	F8VZY9	Keratin, type I cytoskeletal 18
1786	dirMS_041712_NAR_50mM_plus2.12031.12161.3	3	16.62	0	2.97E+05		(R)VVVLMC	(R)V V|V|L/M/G S T S D L/G H/C E/K(I)	C:Carbamidomethylation	131.5	577.9512	1731.84	-0.0014	-0.8	5.32	37681.5	5.93	HUMAN	D6RF62	Phosphoribosylaminoimidazole carboxylase
1787	dirMS_041712_NAR_120mM_minus.18644.18666.3	3	16.61	1	3.30E+05		(R)ARFEELC	(R)A R F E E\L|C|S/D/L|F R(S)	C:Carbamidomethylation	156.88	514.9175	1542.737	0.001	0.7	4.68	25598.3	6.45	HUMAN	E7EP11	Heat shock 70 kDa protein 1A/1B
1788	dirMS_041712_NAR_50mM_minus2.15296.15462.3	3	16.61	0	5.45E+05		(R)DGLAFN	(R)D|G L|A/F/N/A/L/I/H/R(H)		155.45	409.5594	1226.664	-0.0004	-0.3	6.74	146974	5.55	HUMAN	C9JRP8	Spectrin beta chain, non-erythrocytic 4
1789	dirMS_041712_NAR_30mM_plus2.13563.13657.2	2	16.61	0	4.63E+05		(K)NLQYYD	(K)N/L|Q|Y|Y/D/I/S A K(S)		124.33	607.8058	1214.605	-0.0009	-0.7	5.83	14844.8	5.89	HUMAN	B4DV51	GTP-binding nuclear protein Ran
1790	dirMS_041712_NAR_50mM_plus2.13335.13336.3	3	16.61	0	6.82E+05		(K)AASDIAI	(K)A A|S D|I|A|M/T E L/P/P T H P I R(L)		138.7	607.3177	1819.937	0.0015	0.8	5.32	12926.1	4.4	HUMAN	K7EM20	14-3-3 protein epsilon (Fragment)
1791	dirMS_041712_NAR_60mM_minus.17165.17260.3	3	16.6	1	3.81E+05		(R)KAPSDL	(R)K\A/P S D\L|Y/Q I I/L/K(A)		156.22	463.6095	1388.815	-0.0008	-0.6	8.5	21243.9	5.49	HUMAN	E9PL37	Calpain-1 catalytic subunit (Fragment)
1792	dirMS_041712_NAR_60mM_minus.5107.5335.3	3	16.6	0	4.24E+06	M434m M4	(R)LESGmC	(R)L/E|S/G m Q/N m/S I/H/T/K(T)	m:Oxidized methionine	84.33	503.2336	1475.698	31.9881	-1.1	6.75	56607.4	5.37	HUMAN	F8VXB4	Keratin, type II cytoskeletal 8
1793	dirMS_041712_NAR_120mM_plus.8433.8433.3	3	16.6	1	1.83E+05		(K)ALSRQEI	(K)A L/S R Q E\M|Q|E|V/Q/S S/R(S)		97.45	550.2746	1648.807	0.0021	1.3	6.19	37486.1	9.03	HUMAN	P22626	Heterogeneous nuclear ribonucleoproteins A2/B1
1794	dirMS_041712_NAR_20mM_plus.13703.13851.2	2	16.6	0	2.08E+05		(K)APILIATI	(K)A/P|I/L|I|A/T/D V A S R(G)		166.65	613.8559	1226.71	-0.0058	-4.7	5.88	61018.6	9.44	HUMAN	B4DLW8	Probable ATP-dependent RNA helicase DDX5
1795	dirMS_041712_NAR_20mM_plus.16254.16499.2	2	16.6	0	2.45E+05		(R)ADLIAYL	(R)A/D|L|I|A/Y/L/K(K)		184.42	453.7678	906.529	-0.0012	-1.3	5.88	11447.2	9.81	HUMAN	C9JFR7	Cytochrome c (Fragment)
1796	dirMS_041712_NAR_20mM_plus.7627.7633.2	2	16.6	0	1.57E+05		(K)IAVAAQ	(K)I/A|V/A|A/Q/N C Y\K(V)	C:Carbamidomethylation	122.43	569.2902	1137.572	0.001	0.9	8.59	31075.7	5.65	HUMAN	P60174	Triosephosphate isomerase
1797	dirMS_041712_NAR_30mM_plus2.17548.17548.2	2	16.6	0	1.64E+05		(R)ASLEAAI	(R)A S L E/A/A|I/A|D/A E Q R(G)		145.03	672.8406	1344.675	-0.0015	-1.1	4.14	56607.4	5.37	HUMAN	F8VXB4	Keratin, type II cytoskeletal 8
1798	dirMS_041712_NAR_60mM_plus.11721.11721.3	3	16.6	0	2.19E+05		(K)TGNLGN	(K)T G|N|L/G N V/V/H/I E R(F)		118.37	436.9042	1308.702	-0.0038	-2.9	6.41	14478.1	9.37	HUMAN	C9JYQ9	60S ribosomal protein L22-like 1
1799	dirMS_041712_NAR_60mM_plus.12239.12296.3	3	16.6	1	8.16E+05		(K)NQLTSN	(K)N Q|L|T/S/N/P E/N T V/F D A K/R(L)		121.12	611.9737	1833.909	-0.0024	-1.3	6.07	72445.7	5.07	HUMAN	P11021	78 kDa glucose-regulated protein
1800	dirMS_041712_NAR_30mM_minus2.18426.18469.2	2	16.59	0	4.47E+05		(R)QAQEYE	(R)Q A|Q|E/Y/E/A/L/L/N I K(V)		154.67	710.3759	1419.748	-0.0033	-2.3	4.53	43773.3	5.26	HUMAN	F8VZY9	Keratin, type I cytoskeletal 18
1801	dirMS_041712_NAR_50mM_minus2.9448.9448.2	2	16.59	1	7.15E+05		(R)DGKLVS	(R)D/G K|L/V|S E S S D V L|P/K(-)		121.97	737.3927	1473.78	-0.0014	-0.9	4.56	56607.4	5.37	HUMAN	F8VXB4	Keratin, type II cytoskeletal 8
1802	dirMS_041712_NAR_30mM_plus2.16221.16221.2	2	16.59	0	7.14E+05		(R)GLCAIAC	(R)G L/C|A|I|A/Q/A/E/S L R(Y)	C:Carbamidomethylation	138.47	644.8372	1288.668	-0.0007	-0.5	6	18529.2	9.98	HUMAN	E9PL45	40S ribosomal protein S3
1803	dirMS_041712_NAR_30mM_plus2.16582.16880.2	2	16.59	0	5.48E+05		(R)FNVPVS	(R)F/N|V|P/V/S L E/S K(K)		140.52	560.3022	1119.604	-0.0073	-6.5	6	24161.9	5.27	HUMAN	F8VZQ9	SAP domain-containing ribonucleoprotein
1804	dirMS_041712_NAR_20mM_minus.12183.12189.2	2	16.58	0	5.41E+05	M185m	(K)LAVNm	(K)L/A|V/N|m|V/P F/P R(L)	m:Oxidized methionine	171.12	580.3173	1143.634	15.993	-1.6	9.75	42230.8	4.77	HUMAN	A6NKZ8	Putative tubulin beta chain-like protein ENSP00000290377
1805	dirMS_041712_NAR_50mM_minus2.14855.14855.3	3	16.58	1	2.91E+05		(R)EAVTEIL	(R)E A/V/T/E/I/L/G I/E/P D R E K(G)		152.33	566.9683	1698.891	-0.0005	-0.3	4.25	25685.9	6.4	HUMAN	E9PGT1	Translin
1806	dirMS_041712_NAR_20mM_plus.17624.17624.2	2	16.58	0	1.55E+05		(K)DVDLEFI	(K)D V|D|L|E F L A\K(M)		192.7	525.2795	1049.551	0.0004	0.4	4.03	90004.9	5.14	HUMAN	P55072	Transitional endoplasmic reticulum ATPase
1807	dirMS_041712_NAR_20mM_plus.9312.9529.2	2	16.58	0	5.64E+05		(K)TIAPALV	(K)T I|A|P/A L/V/S K(K)		136.25	450.2814	899.556	-0.0005	-0.6	8.41	21077	9.05	HUMAN	K7EM90	Alpha-enolase (Fragment)
1808	dirMS_041712_NAR_30mM_plus2.16224.16390.2	2	16.58	0	1.72E+05		(K)YIQELWI	(K)Y/I|Q/E/L/W/R(K)		138.08	504.2687	1007.531	-0.0008	-0.8	6	20608.6	11.48	HUMAN	E7ENU7	Ribosomal protein L15 (Fragment)
1809	dirMS_041712_NAR_50mM_plus2.13400.13400.3	3	16.58	0	6.77E+05		(R)VLVVHD	(R)V/L|V/V/H/D G F E G L/A/K(G)		139.17	461.9229	1383.763	-0.0089	-6.5	5.32	18719.9	6.91	HUMAN	F8VP00	6-phosphofructokinase, muscle type (Fragment)
1810	dirMS_041712_NAR_50mM_plus2.18522.18641.3	3	16.58	0	9.87E+05		(K)VAHALA	(K)V A H A L\A E\G\L|G|V|I/A/C/I G E K(L)	C:Carbamidomethylation	166.15	603.3265	1807.974	-0.0085	-4.7	5.4	31075.7	5.65	HUMAN	P60174	Triosephosphate isomerase
1811	dirMS_041712_NAR_60mM_minus.9607.9607.3	3	16.57	1	4.95E+05		(R)LQAEIEG	(R)L Q|A/E/I E/G L/K G Q R(A)		113.32	447.9164	1341.748	-0.0138	-10.3	6.14	56607.4	5.37	HUMAN	F8VXB4	Keratin, type II cytoskeletal 8
1812	dirMS_041712_NAR_20mM_plus.15350.15769.3	3	16.57	0	1.92E+06	M73m	(R)AILVDLE	(R)A I\L\V D\L E/P/G T/m/D S V R(S)	m:Oxidized methionine	179.15	544.6139	1615.836	15.9912	-2.3	4.03	27549.9	4.39	HUMAN	E9PBJ4	Tubulin beta chain
1813	dirMS_041712_NAR_30mM_plus2.17064.17064.2	2	16.57	0	1.82E+05		(K)GVVDSD	(K)G V|V|D|S/D D L|P L N V/S R(E)		142.53	743.3807	1485.754	-0.0002	-0.2	3.93	92752.5	4.76	HUMAN	P14625	Endoplasmin
1814	dirMS_041712_NAR_50mM_plus2.14739.14739.3	3	16.57	1	1.88E+05		(R)ALSNLES	(R)A L|S|N/L/E/S I/P G G Y N A L R\R(M)		146.72	610.9972	1830.982	-0.005	-2.7	8.79	16443.8	5	HUMAN	H0YDS0	Ubiquilin-1 (Fragment)
1815	dirMS_041712_NAR_50mM_plus2.15943.15963.3	3	16.57	1	3.72E+05		(K)TQLLQD	(K)T Q|L|L/Q/D V/Q/D E N K/L/F/K(S)		151.8	606.9845	1818.96	-0.0207	-11.4	4.55	69285	5.2	HUMAN	G3V4Y7	Kinectin
1816	dirMS_041712_NAR_60mM_plus.19366.19556.3	3	16.57	1	6.73E+05		(R)TRIEGLL	(R)T R I E G\L|L|A/A/F/P/K(L)		159.8	439.262	1315.773	-0.0018	-1.4	8.41	62195.8	5.63	HUMAN	B0YIW6	Archain 1, isoform CRA_a
1817	dirMS_041712_NAR_20mM_minus.7866.7866.2	2	16.56	0	1.69E+05		(K)SVEAAA	(K)S V|E/A/A/A/E L S A K(D)		128.45	538.2838	1075.563	-0.0027	-2.5	4.53	12131.5	11.14	HUMAN	F5GXR3	Parathymosin
1818	dirMS_041712_NAR_30mM_plus2.19581.19581.2	2	16.56	0	2.14E+05		(K)TVDNFV	(K)T V|D|N/F|V/A L/A T G E K(G)		155.58	682.8539	1364.706	-0.0051	-3.7	4.37	23799.1	9.48	HUMAN	P23284	Peptidyl-prolyl cis-trans isomerase B
1819	dirMS_041712_NAR_40mM_minus2.12009.12090.3	3	16.55	1	9.22E+04		(K)IIVDELK	(K)I/I|V|D/E L K/Q E\V/I/S T S S/K(A)		154.43	597.0023	1788.995	-0.003	-1.7	4.68	30149.5	5.96	HUMAN	D6RAC2	Guanine nucleotide-binding protein subunit beta-2-like 1
1820	dirMS_041712_NAR_30mM_plus2.16492.16492.2	2	16.55	0	1.38E+05		(R)STLNEIY	(R)S T/L/N/E/I|Y/F G K(T)		139.07	586.3039	1171.599	0.0012	1	5.72	29580	6.46	HUMAN	B1AK87	Capping protein (Actin filament) muscle Z-line, beta
1821	dirMS_041712_NAR_30mM_plus2.17843.17860.2	2	16.55	0	1.29E+05		(K)FAEAFE	(K)F/A|E|A|F/E/A/I/P R(A)		146.62	575.798	1150.589	-0.0004	-0.4	4.53	58214.7	5.25	HUMAN	B4DEM7	T-complex protein 1 subunit theta
1822	dirMS_041712_NAR_60mM_plus.17088.17088.3	3	16.55	1	7.26E+06		(K)LKVPEW	(K)L/K V\P E/W|V/D/T V/K(L)		145.72	438.5872	1313.746	0.0007	0.5	6.07	16060.2	10.31	HUMAN	P39019	40S ribosomal protein S19
1823	dirMS_041712_NAR_60mM_plus.5154.5154.3	3	16.55	1	4.46E+04		(K)GNSPPS	(K)G N/S|P/P/S S G E A C R E E K(G)	C:Carbamidomethylation	84.52	535.5678	1604.697	-0.0081	-5	4.79	24954.4	5.33	HUMAN	A6NM98	ARD1 homolog A, N-acetyltransferase (S. cerevisiae), isoform CRA_b
1824	dirMS_041712_NAR_30mM_minus2.8998.9018.2	2	16.54	0	2.70E+05		(K)TNEAQA	(K)T/N|E|A|Q/A|I|E/T A R(A)		103.52	602.3015	1203.596	-0.0007	-0.6	4.53	10145.6	9.15	HUMAN	F8VQQ8	Ubiquitin-conjugating enzyme E2 N
1825	dirMS_041712_NAR_50mM_minus2.12363.12541.3	3	16.54	0	6.64E+05		(K)AGVVGF	(K)A/G\V|V|G/P E/L/H/E Q L L/S A E K(A)		139.27	592.9866	1776.949	-0.0038	-2.1	4.75	533778	5.74	HUMAN	Q15149	Plectin
1826	dirMS_041712_NAR_50mM_minus2.12471.12524.3	3	16.54	0	1.98E+05		(R)VLVVHD	(R)V/L/V/V/H/D/G F E G L/A K(G)		139.7	461.9238	1383.763	-0.0062	-4.5	5.32	18719.9	6.91	HUMAN	F8VP00	6-phosphofructokinase, muscle type (Fragment)
1827	dirMS_041712_NAR_50mM_minus2.8472.8673.3	3	16.54	0	1.58E+06		(R)EIVHIQA	(R)E/I\V H I Q\A G/Q|C/G N Q I/G A/K(F)	C:Carbamidomethylation	116.8	608.3087	1822.923	-0.0113	-6.2	6.85	40069.4	4.83	HUMAN	E7EWR1	Tubulin beta chain
1828	dirMS_041712_NAR_20mM_plus.15454.15790.3	3	16.54	0	1.92E+06	M73m	(R)AILVDLE	(R)A I|L\V D L E|P/G T m/D/S V R(S)	m:Oxidized methionine	179.15	544.6139	1615.836	15.9912	-2.3	4.03	27549.9	4.39	HUMAN	E9PBJ4	Tubulin beta chain
1829	dirMS_041712_NAR_20mM_plus.16449.16449.2	2	16.54	0	1.66E+05		(R)LLEAQIA	(R)L L E/A/Q I|A|T G G I/I\D|P K(E)		185.07	769.9362	1538.879	-0.0137	-8.9	4.37	334221.3	6.44	HUMAN	P15924	Desmoplakin
1830	dirMS_041712_NAR_20mM_plus.19355.19355.2	2	16.54	0	8.40E+04		(R)ALIAGG	(R)A/L/I|A|G G G\A|P E I E\L A L R(L)		205.1	775.9467	1550.89	-0.0039	-2.5	4.53	42755	7.81	HUMAN	B7Z2F4	T-complex protein 1 subunit delta
1831	dirMS_041712_NAR_30mM_plus2.12078.12110.2	2	16.54	0	2.57E+05		(R)ENVNLA	(R)E N|V|N/L/A/Q/I/R(T)		116.4	528.7932	1056.58	-0.0005	-0.5	6.1	62195.8	5.63	HUMAN	B0YIW6	Archain 1, isoform CRA_a
1832	dirMS_041712_NAR_30mM_plus2.12353.12425.2	2	16.54	0	8.33E+04		(K)MADEAI	(K)M/A|D|E|A|I|L/Q E/R(E)		116.57	588.2906	1175.573	0.0014	1.2	4.14	65763.5	9.81	HUMAN	Q9HCD5	Nuclear receptor coactivator 5
1833	dirMS_041712_NAR_60mM_plus.21571.21571.3	3	16.54	1	5.98E+05		(K)EIIDLVLD	(K)E I/I/D/L V L/D/R I/R(K)		180.57	452.2733	1354.805	0.0001	0.1	4.56	58642	4.95	HUMAN	F5H5D3	Tubulin alpha-1C chain
1834	dirMS_041712_NAR_60mM_minus.14146.14243.3	3	16.53	0	1.14E+05		(K)GVGISVI	(K)G V|G I/S/V/L/E/M S H/R(S)		139.12	428.9004	1284.673	0.0138	10.7	6.75	40821.3	7.79	HUMAN	Q9Y617	Phosphoserine aminotransferase
1835	dirMS_041712_NAR_20mM_plus.13897.13897.2	2	16.53	0	6.90E+04		(K)LLDLVQ	(K)L/L\D\L/V/Q/Q/S C N Y K(Q)	C:Carbamidomethylation	167.73	740.8757	1480.746	-0.0023	-1.6	5.83	14855.1	9.26	HUMAN	B1AHD1	NHP2 non-histone chromosome protein 2-like 1 (S. cerevisiae)
1836	dirMS_041712_NAR_30mM_plus2.9737.9775.2	2	16.53	0	5.42E+05		(K)FESPEVA	(K)F/E|S|P/E/V/A E R(A)		104.78	532.2579	1063.506	0.0031	2.9	4.25	77799.7	9.09	HUMAN	P52272	Heterogeneous nuclear ribonucleoprotein M
1837	dirMS_041712_NAR_60mM_plus.14919.14951.3	3	16.53	1	7.41E+05		(R)ELAFQIS	(R)E L/A/F/Q/I/S K/E/Y E R(F)		134.78	504.9258	1512.769	-0.0064	-4.3	4.79	30944.2	6.52	HUMAN	B1Q2N1	ATP-dependent RNA helicase DDX39A
1838	dirMS_041712_NAR_60mM_plus.5085.5085.3	3	16.53	1	3.95E+05		(R)AAEDDE	(R)A/A|E D/D E/D/D D V/D T K K(Q)		83.53	522.5529	1565.645	-0.0008	-0.5	3.76	14366.2	3.71	HUMAN	B8ZZA1	Thymosin alpha-1
1839	dirMS_041712_NAR_60mM_minus.10597.10597.3	3	16.52	0	5.02E+05	M368m	(K)LAQANG	(K)L/A|Q\A N/G W/G V/m\V/S H R(S)	m:Oxidized methionine	119	514.591	1525.769	15.9892	-3.7	9.75	47510.4	7.04	HUMAN	P06733	Alpha-enolase
1840	dirMS_041712_NAR_20mM_plus.18693.18693.2	2	16.52	0	2.74E+05		(K)TAVETA	(K)T A|V/E/T/A/V L\L/L R(I)		200	593.3625	1185.72	-0.0024	-2	5.66	56186.7	5.47	HUMAN	B4DUR8	T-complex protein 1 subunit gamma
1841	dirMS_041712_NAR_30mM_plus2.16018.16039.2	2	16.52	0	2.68E+06		(R)INEILSN	(R)I/N|E|I|L/S N/A L K(R)		137.82	557.8307	1114.647	0.0075	6.7	6	77799.7	9.09	HUMAN	P52272	Heterogeneous nuclear ribonucleoprotein M
1842	dirMS_041712_NAR_30mM_plus2.17129.17318.2	2	16.52	0	3.45E+05		(K)TVFGVEI	(K)T V|F/G|V/E/P\D L T R(E)		143.35	617.3263	1233.647	-0.0021	-1.7	4.37	53219.8	5.66	HUMAN	Q96KP4	Cytosolic non-specific dipeptidase
1843	dirMS_041712_NAR_40mM_plus2.16880.16880.3	3	16.52	0	8.98E+04		(R)LLEVLSG	(R)L/L/E V/L/S G E/I/L/P K P T K(G)		166	546.3323	1636.988	-0.006	-3.7	6.14	13611	6.13	HUMAN	E9PJZ2	Spectrin beta chain, non-erythrocytic 2 (Fragment)
1844	dirMS_041712_NAR_50mM_plus2.21733.21849.3	3	16.52	0	9.49E+05		(K)GLAPDLI	(K)G L|A|P/D/L/P E\D L/Y/H L I/K(K)		185.65	565.3101	1693.916	-0.0002	-0.1	4.54	16789.5	10.63	HUMAN	J3KMX5	40S ribosomal protein S13
1845	dirMS_041712_NAR_60mM_plus.9449.9450.3	3	16.52	0	2.84E+06		(K)SYCAEIA	(K)S Y|C/A E/I/A H N V S/S/K(N)	C:Carbamidomethylation	107.38	489.235	1465.674	0.0164	11.2	6.47	15730.4	11.67	HUMAN	D3YTB1	60S ribosomal protein L32 (Fragment)
1846	dirMS_041712_NAR_30mM_minus2.18036.18280.2	2	16.51	0	4.30E+05		(R)GLFIIDD	(R)G L|F|I|I/D/D/K(G)		153.47	460.7585	920.509	0.001	1	4.21	18498.6	5.82	HUMAN	H7C3T4	Peroxiredoxin-4 (Fragment)
1847	dirMS_041712_NAR_30mM_minus2.18323.18323.2	2	16.51	0	4.28E+05		(R)QAQEYE	(R)Q A/Q/E|Y/E/A/L/L/N I K(V)		154.63	710.3759	1419.748	-0.0033	-2.3	4.53	43773.3	5.26	HUMAN	F8VZY9	Keratin, type I cytoskeletal 18
1848	dirMS_041712_NAR_30mM_minus2.7471.7471.2	2	16.51	0	5.49E+04		(R)GAEQLA	(R)G A|E|Q/L A|E/G G R(M)		95.05	494.2466	987.485	0.0005	0.5	4.53	59953.2	6.14	HUMAN	G3V1R4	NOL1/NOP2/Sun domain family, member 2, isoform CRA_b

1849	dirMS_041712_NAR_60mM_minus.10287.10543.3	3	16.51	1	6.05E+05	M88m	(K)DVDEAY	(K)D V\D|E/A/Y m N K/V/E/L/E/S R(L)	m:Oxidized methionine	118.37	605.2791	1797.832	15.9904	-2.5	4.18	13588.1	4.66	HUMAN	F8VP67	Keratin, type II cytoskeletal 8 (Fragment)
1850	dirMS_041712_NAR_60mM_minus.11906.11906.3	3	16.51	1	3.74E+05		(K)NQVAM	(K)N Q|V/A|M/N/P T N T V F/D A K R(L)		126.55	602.6357	1805.896	-0.0037	-2.1	8.75	20069.4	6.74	HUMAN	E9PK54	Heat shock cognate 71 kDa protein (Fragment)
1851	dirMS_041712_NAR_30mM_plus2.22336.22336.3	3	16.51	0	5.20E+05		(R)VGNLGL	(R)V G N L\G L\A\T/S/F/F|N E R(N)		172.8	508.9306	1524.781	-0.0033	-2.1	5.97	73641.5	6.74	HUMAN	O00571	ATP-dependent RNA helicase DDX3X
1852	dirMS_041712_NAR_40mM_plus2.16866.16866.3	3	16.51	1	1.02E+05		(R)ALGTEVI	(R)A L|G T/E/V/I Q L/F/P E K G N M G/K(I)		165.6	644.6779	1932.026	-0.0068	-3.5	6.18	67184.7	9.24	HUMAN	O95831	Apoptosis-inducing factor 1, mitochondrial
1853	dirMS_041712_NAR_40mM_plus2.19240.19240.3	3	16.51	1	1.93E+05		(K)TSAQVV	(K)T S/A/Q/V/V/L|D G L V D K I G D/V/K(C)		180.37	619.6811	1857.033	-0.004	-2.2	4.43	227202.2	8.26	HUMAN	Q14008	Cytoskeleton-associated protein 5
1854	dirMS_041712_NAR_50mM_plus2.6005.6005.3	3	16.51	1	3.38E+04		(K)SAVEAQ	(K)S A/V|E/A/Q/N E V T E N/P K/Q K(I)		95.67	591.3073	1771.882	0.0253	14.3	4.78	89879.1	4.58	HUMAN	Q32MZ4	Leucine-rich repeat flightless-interacting protein 1
1855	dirMS_041712_NAR_120mM_minus.8095.8115.3	3	16.5	0	1.23E+06		(K)HSGPNS	(K)H S G P N\S\A/D|S|A|N/D/G F V/R(L)		98.42	544.2477	1630.72	0.0081	5	5.21	46013.4	5.38	HUMAN	P52597	Heterogeneous nuclear ribonucleoprotein F
1856	dirMS_041712_NAR_120mM_minus.8350.8473.3	3	16.5	1	1.00E+06		(K)KGTVEG	(K)K\G T V E\G\F|E|P/A/D/N K(C)		100.1	464.5648	1391.68	-0.0003	-0.2	4.68	13286.2	9.99	HUMAN	H0YLA2	Signal recognition particle 14 kDa protein
1857	dirMS_041712_NAR_20mM_plus.6868.6868.2	2	16.5	0	4.23E+05	M132m	(R)DmVGIA	(R)D m|V|G/I|A Q/T/G S G K(T)	m:Oxidized methionine	112.85	590.2864	1163.573	15.993	-1.6	5.84	73202	9.19	HUMAN	C9JMU5	Probable ATP-dependent RNA helicase DDX17
1858	dirMS_041712_NAR_40mM_plus2.10727.10727.3	3	16.5	0	1.85E+05		(K)VGSLDN	(K)V/G/S\L D N V/G H/L P/A G G A V/K(I)		128.15	530.9567	1590.86	-0.0043	-2.7	6.71	26233.5	10.95	HUMAN	B4DSQ1	Microtubule-associated protein
1859	dirMS_041712_NAR_40mM_plus2.13783.13783.3	3	16.5	1	2.45E+05		(R)GDVVPK	(R)G D/V|V|P/K/D\V N/A A I A/T/I K(T)		147.4	537.6381	1610.911	-0.0115	-7.1	5.96	58642	4.95	HUMAN	F5H5D3	Tubulin alpha-1C chain
1860	dirMS_041712_NAR_120mM_plus.13414.13435.3	3	16.49	1	4.93E+05		(R)VIDRELY	(R)V/I/D/R E L\Y|Q/Q|L Q/R(G)		123.58	520.9543	1560.849	-0.0009	-0.6	6.04	533778	5.74	HUMAN	Q15149	Plectin
1861	dirMS_041712_NAR_20mM_plus.18612.18772.2	2	16.49	0	2.00E+05		(R)VFIGNLN	(R)V/F|I|G/N/L/N T/L/V\V/K(K)		200.13	658.8999	1316.794	-0.0011	-0.9	8.72	15791.7	9.22	HUMAN	B4DSU6	Heterogeneous nuclear ribonucleoproteins C1/C2
1862	dirMS_041712_NAR_30mM_plus2.11204.11509.2	2	16.49	0	8.39E+05		(K)LTVDSAI	(K)L/T|V/D/S/A/I A R(D)		112.58	473.2687	945.536	-0.0062	-6.6	5.84	334221.3	6.44	HUMAN	P15924	Desmoplakin
1863	dirMS_041712_NAR_120mM_minus.6664.6667.2	2	16.48	1	8.10E+04		(K)DSYVGD	(K)D/S/Y|V/G/D/E A Q S K/R(G)		90.67	677.8145	1354.623	-0.0016	-1.2	4.56	32616.5	5.18	HUMAN	A6NL76	Actin, alpha skeletal muscle
1864	dirMS_041712_NAR_30mM_minus2.16073.16073.2	2	16.48	0	1.16E+05		(K)EQFLDG	(K)E Q|F|L/D/G D/G W/T|S R(W)		141.08	705.8174	1410.628	-0.0009	-0.6	4.03	28545.1	4.5	HUMAN	K7EJB9	Calreticulin (Fragment)
1865	dirMS_041712_NAR_60mM_minus.14221.14222.3	3	16.48	0	1.14E+05		(K)GVGISVI	(K)G V|G I/S V/L/E/M/S H R(S)		139.12	428.9004	1284.673	0.0138	10.7	6.75	40821.3	7.79	HUMAN	Q9Y617	Phosphoserine aminotransferase
1866	dirMS_041712_NAR_20mM_plus.14298.14298.2	2	16.48	0	3.41E+04		(R)IALTDN/	(R)I/A|L\T/D/N/A L I/A R(S)		170.65	585.8432	1170.684	-0.005	-4.2	5.84	24489.6	10.74	HUMAN	A8MUD9	60S ribosomal protein L7
1867	dirMS_041712_NAR_20mM_plus.8335.8335.2	2	16.48	0	1.32E+04		(R)LAAVDV	(R)L/A|A|V/D\V S A/R(A)		127.57	451.2557	901.51	-0.006	-6.7	5.84	266161	5.68	HUMAN	E9PPU0	Epiplakin
1868	dirMS_041712_NAR_20mM_plus.10616.10616.2	2	16.47	0	7.50E+04		(K)TTTLSGT	(K)T T T|L/S/G/T/A|P A A/G V V|P S R(V)		145.97	793.4284	1585.854	-0.0049	-3.1	9.41	26233.5	10.95	HUMAN	B4DSQ1	Microtubule-associated protein
1869	dirMS_041712_NAR_30mM_plus2.14555.14760.2	2	16.47	0	4.73E+06		(R)FPGQLN	(R)F/P|G/Q/L/N/A|D|L R(K)		130.65	565.801	1130.595	-0.0006	-0.5	5.84	42230.8	4.77	HUMAN	A6NKZ8	Putative tubulin beta chain-like protein ENSP00000290377
1870	dirMS_041712_NAR_40mM_plus2.15007.15402.3	3	16.47	0	6.93E+05		(K)IEVIKPG	(K)I/E|V I/K/P G D|L/G V D/L T S K(L)		156.1	561.9876	1683.953	-0.0045	-2.7	4.56	28071.3	8.6	HUMAN	P38117	Electron transfer flavoprotein subunit beta
1871	dirMS_041712_NAR_40mM_plus2.16567.16593.3	3	16.47	0	1.95E+05		(R)VAAGLP	(R)V A|A|G/L|P/P I L V H/T D A A/Q A L G K(Q)		164.1	648.041	1942.112	-0.0036	-1.8	6.71	17974.2	4.97	HUMAN	C9JNL5	Selenocysteine lyase (Fragment)
1872	dirMS_041712_NAR_40mM_plus2.6678.6678.2	2	16.47	0	4.08E+05		(K)AGFAGD	(K)A G/F/A/G/D/D/A/P R(A)		99.45	488.728	976.448	0.0004	0.4	4.21	123097.1	5.83	HUMAN	A5A3E0	POTE ankyrin domain family member F
1873	dirMS_041712_NAR_50mM_plus2.14466.14598.3	3	16.47	0	1.06E+06		(R)ETHSAL(	(R)E T H S A\L|G|P|A|L|Q/A A F/K(L)		144.3	514.2745	1540.812	-0.0029	-1.9	6.85	141616.6	6.1	HUMAN	B7ZKM8	Protein transport protein Sec24B
1874	dirMS_041712_NAR_120mM_minus.15982.16391.4	4	16.46	2	1.46E+06		(K)KQGGLG	(K)K Q G G\L G\P M|N\I/P/L/V/S D/P K R(T)		143.48	477.5179	1907.053	-0.0034	-1.8	9.99	22338.2	8.65	HUMAN	Q06830	Peroxiredoxin-1
1875	dirMS_041712_NAR_20mM_minus.11224.11224.2	2	16.46	0	2.79E+05		(K)LSELEAA	(K)L/S/E/L|E A A|L/Q R(A)		159.5	565.3134	1129.621	-0.0016	-1.5	4.53	56607.4	5.37	HUMAN	F8VXB4	Keratin, type II cytoskeletal 8
1876	dirMS_041712_NAR_20mM_minus.8134.8445.2	2	16.46	0	6.77E+06	M239m	(K)EITALAP	(K)E I|T/A|L|A|P/S T m K(I)	m:Oxidized methionine	132.6	589.3094	1161.618	15.9931	-1.5	6.1	32616.5	5.18	HUMAN	A6NL76	Actin, alpha skeletal muscle
1877	dirMS_041712_NAR_50mM_minus2.5675.5675.3	3	16.46	1	3.66E+05		(K)DSYVGD	(K)D/S|Y/V/G D E/A/Q S K/R(G)		92.95	452.2119	1354.623	-0.0022	-1.6	4.56	32616.5	5.18	HUMAN	A6NL76	Actin, alpha skeletal muscle
1878	dirMS_041712_NAR_60mM_minus.18853.19112.3	3	16.46	1	4.12E+06		(R)LDLAGR	(R)L/D|L/A/G R D L/T D/Y L/M/K(I)		167.95	541.9507	1623.841	-0.0035	-2.2	4.43	37482.1	5.36	HUMAN	E9PG30	Actin, gamma-enteric smooth muscle
1879	dirMS_041712_NAR_60mM_minus.8253.8253.3	3	16.46	1	1.45E+05		(R)LSQIGVE	(R)L/S/Q/I/G/V E N T E E N R R(L)		106.1	548.9481	1644.83	-0.0002	-0.1	4.79	36636.9	7.84	HUMAN	A8MVZ9	Fructose-bisphosphate aldolase
1880	dirMS_041712_NAR_120mM_plus.16292.16763.4	4	16.46	2	4.59E+06		(K)KQGGLG	(K)K Q G G\L G\P M|N\I/P/L/V/S D/P K R(T)		140.23	477.5186	1907.053	-0.0006	-0.3	9.99	22338.2	8.65	HUMAN	Q06830	Peroxiredoxin-1
1881	dirMS_041712_NAR_30mM_plus2.13928.14038.2	2	16.46	0	3.99E+05		(R)LLQLQE(	(R)L L|Q|L/Q\E/Q/M/R(A)		126.32	579.8193	1158.63	0.0014	1.2	6	334221.3	6.44	HUMAN	P15924	Desmoplakin
1882	dirMS_041712_NAR_50mM_plus2.11437.11437.3	3	16.46	1	7.45E+05		(K)LAEQAE	(K)L/A|E/Q/A/E R Y D D/M A A A M K(A)		127.98	604.9469	1812.825	0.0007	0.4	4.32	28196	4.76	HUMAN	P31946	14-3-3 protein beta/alpha
1883	dirMS_041712_NAR_40mM_minus2.13812.13812.3	3	16.45	1	4.48E+04		(K)FGEVVD	(K)F/G/E/V/V/D C T L K/L D/P I\T G R(S)	C:Carbamidomethylation	165.3	640.6656	1919.989	-0.0073	-3.8	4.56	33782.7	8.78	HUMAN	B4DTC3	Heterogeneous nuclear ribonucleoprotein D0
1884	dirMS_041712_NAR_60mM_minus.12725.13235.3	3	16.45	0	1.39E+07		(R)NLDIERF	(R)N/L|D|I/E R/P T Y/T/N/L N R(L)		130.23	573.6304	1718.882	-0.0054	-3.1	6.07	49012.3	4.9	HUMAN	A8MUB1	Tubulin alpha-4A chain
1885	dirMS_041712_NAR_20mM_plus.18802.19183.2	2	16.45	0	1.21E+06		(K)EIIDLVLI	(K)E I|I/D|L/V/L/D/R(I)		202.38	543.313	1085.62	-0.0014	-1.3	4.03	58642	4.95	HUMAN	F5H5D3	Tubulin alpha-1C chain
1886	dirMS_041712_NAR_30mM_plus2.19929.20072.2	2	16.45	0	3.53E+05		(R)LNILDTL	(R)L/N|I|L/D/T/L/S/K(F)		158.72	508.8023	1016.599	-0.0013	-1.3	5.84	86346.5	5.05	HUMAN	E7EW34	26S proteasome non-ATPase regulatory subunit 2
1887	dirMS_041712_NAR_50mM_plus2.16044.16044.3	3	16.45	0	1.05E+06		(R)LSFQHD	(R)L/S/F Q H/D|P E/T S V/L/V L/R(K)		152.42	580.9801	1740.928	-0.0022	-1.2	5.32	96827.3	5.58	HUMAN	E9PKU7	Neutral alpha-glucosidase AB
1888	dirMS_041712_NAR_60mM_plus.11495.11495.3	3	16.45	0	1.11E+06		(R)SIFQHIQ	(R)S I/F Q/H I/Q/S/A/Q/S Q/R(S)		117.93	510.6056	1529.782	0.0203	13.3	9.49	108720.9	10.17	HUMAN	Q9Y2W1	Thyroid hormone receptor-associated protein 3
1889	dirMS_041712_NAR_60mM_minus.13591.13918.3	3	16.44	1	2.50E+07		(R)GYSFTTT	(R)G Y|S|F/T T/T/A E R E I V/R(D)		136.28	543.9458	1629.823	-0.0003	-0.2	6.14	23901.9	5.28	HUMAN	I3L1U9	Actin, cytoplasmic 2, N-terminally processed (Fragment)
1890	dirMS_041712_NAR_30mM_plus2.13778.13793.2	2	16.44	0	8.29E+05		(R)GIVDSIT	(R)G I|V|D/S/I/T/G Q/R(L)		125.5	523.2797	1045.564	-0.0115	-11	5.84	334221.3	6.44	HUMAN	P15924	Desmoplakin
1891	dirMS_041712_NAR_30mM_plus2.9894.9910.2	2	16.44	0	1.51E+06		(R)LTVDEA\	(R)L/T|V/D/E/A V/R(A)		105.73	451.7509	902.494	0.0004	0.4	4.37	533778	5.74	HUMAN	Q15149	Plectin
1892	dirMS_041712_NAR_30mM_minus2.15071.15198.2	2	16.43	0	7.18E+05	M2249m	(K)MSIYQA	(K)M/S|I|Y/Q/A/m W/K(G)	m:Oxidized methionine	135.72	587.2756	1157.548	15.9957	0.7	8.34	266161	5.68	HUMAN	E9PPU0	Epiplakin
1893	dirMS_041712_NAR_40mM_minus2.14514.14781.3	3	16.43	1	1.50E+06	M116m	(R)ELAILLG	(R)E L|A|I/L/L/G m L/D/P A/E K D E K(G)	m:Oxidized methionine	171.22	634.338	1884.999	16.0008	3.1	4.18	25148.6	6	HUMAN	P30041	Peroxiredoxin-6
1894	dirMS_041712_NAR_60mM_minus.11523.11523.3	3	16.43	1	2.94E+05		(R)VFDKEG	(R)V/F|D K E G\N G\T\V/M/G A E/I R(H)		124.35	574.9539	1722.848	-0.0008	-0.5	4.68	26991.7	5.01	HUMAN	B7Z6Z4	Myosin light polypeptide 6
1895	dirMS_041712_NAR_60mM_minus.12199.12724.3	3	16.43	1	3.18E+06		(K)ASLENSI	(K)A/S|L/E/N S/L/R E V E/A/R(Y)		128.3	491.927	1473.766	0.0009	0.6	4.79	43773.3	5.26	HUMAN	F8VZY9	Keratin, type I cytoskeletal 18
1896	dirMS_041712_NAR_40mM_plus2.18201.18391.3	3	16.43	0	3.43E+05		(R)EAVELPI	(R)E A|V/E/L/P/L/T/H F E/L/Y/K(Q)		173.8	563.6336	1688.889	-0.0032	-1.9	4.75	47479.5	5.09	HUMAN	P43686	26S protease regulatory subunit 6B
1897	dirMS_041712_NAR_50mM_plus2.10638.10806.3	3	16.43	1	6.61E+05		(K)GCGVVK	(K)G C|G V|V K/F E/S/P/E/V A E R(A)	C:Carbamidomethylation	123.88	555.2753	1663.811	0.0005	0.3	4.79	77799.7	9.09	HUMAN	P52272	Heterogeneous nuclear ribonucleoprotein M
1898	dirMS_041712_NAR_120mM_minus.11584.12119.3	3	16.42	1	5.12E+06		(R)FPGQLN	(R)F/P/G Q|L/N/A/D/L R K(L)		118.65	420.2348	1258.69	-0.0004	-0.3	8.75	42230.8	4.77	HUMAN	A6NKZ8	Putative tubulin beta chain-like protein ENSP00000290377
1899	dirMS_041712_NAR_20mM_minus.13093.13151.3	3	16.42	0	1.85E+06	M73m	(R)AILVDLE	(R)A I\L\V D\L E|P/G T/m D/S V R(S)	m:Oxidized methionine	182.72	544.6138	1615.836	15.9909	-2.5	4.03	27549.9	4.39	HUMAN	E9PBJ4	Tubulin beta chain
1900	dirMS_041712_NAR_120mM_plus.11430.11448.3	3	16.42	0	7.46E+05		(K)TGNLGN	(K)T/G|N|L/G N V/V/H/I/E R(F)		113.42	436.9054	1308.702	-0.0002	-0.2	6.41	14478.1	9.37	HUMAN	C9JYQ9	60S ribosomal protein L22-like 1
1901	dirMS_041712_NAR_20mM_plus.15524.15524.2	2	16.42	0	4.33E+05		(R)GVVDSE	(R)G V\V/D/S/E/D/I|P L N/L/S/R(E)		179.2	757.3956	1513.786	-0.0017	-1.1	4.03	74494.3	7.79	HUMAN	F5H897	Heat shock protein 75 kDa, mitochondrial
1902	dirMS_041712_NAR_20mM_plus.18677.18677.2	2	16.42	0	2.87E+05		(K)LAVEALS	(K)L A V/E A|L|S/S L|D|G D L/A/G R(Y)		200.48	793.9214	1586.838	-0.0029	-1.8	4.03	24286.7	4.89	HUMAN	G3V4N7	Creatine kinase B-type (Fragment)
1903	dirMS_041712_NAR_30mM_plus2.19467.19529.2	2	16.42	0	7.16E+05		(R)LDFNLIR	(R)L/D|F|N|L/I/R(V)		156.18	445.759	890.509	0.0013	1.5	5.84	18956.4	6.97	HUMAN	C9JLK0	Phosphoribosylaminoimidazolecarboxamide formyltransferase (Fragment)
1904	dirMS_041712_NAR_50mM_plus2.14343.14343.3	3	16.42	1	1.80E+05		(K)TITLEVEI	(K)T I|T L/E/V/E/P S/D T I E/N V K/A K(I)		143.57	663.0232	1987.059	-0.0043	-2.2	4.41	17271.6	6.75	HUMAN	B4DV12	Ubiquitin
1905	dirMS_041712_NAR_120mM_minus.10767.10788.3	3	16.41	0	9.41E+05		(R)GFCLPPI	(R)G F|C/L/P/P/H C S R(G)	C:Carbamidomethylation	113.53	410.858	1230.551	0.0088	7.1	9.75	14391.8	4.99	HUMAN	G3V461	Creatine kinase B-type (Fragment)
1906	dirMS_041712_NAR_30mM_minus2.18980.19254.2	2	16.41	0	1.32E+06		(K)LAVNM\	(K)L A|V/N|M|V|P F/P R(L)		159.15	572.3143	1143.634	-0.013	-11.4	9.75	42230.8	4.77	HUMAN	A6NKZ8	Putative tubulin beta chain-like protein ENSP00000290377
1907	dirMS_041712_NAR_60mM_minus.11928.12008.3	3	16.41	0	3.17E+05		(K)VFEGNR	(K)V/F|E/G N R P T N S I/V/F/T/K(L)		126.65	570.304	1708.902	-0.0042	-2.5	8.72	63374.2	8.64	HUMAN	P06744	Glucose-6-phosphate isomerase
1908	dirMS_041712_NAR_20mM_plus.13915.13915.2	2	16.41	0	6.90E+04		(K)LLDLVQ(	(K)L L|D|L/V/Q/Q/S/C/N Y K(Q)	C:Carbamidomethylation	167.73	740.8757	1480.746	-0.0023	-1.6	5.83	14855.1	9.26	HUMAN	B1AHD1	NHP2 non-histone chromosome protein 2-like 1 (S. cerevisiae)
1909	dirMS_041712_NAR_20mM_plus.18331.18331.2	2	16.41	0	1.95E+05		(R)AAVEEG	(R)A A V E\E\G/I|V|L/G G/G C/A L L R(C)	C:Carbamidomethylation	197.9	842.9549	1684.905	-0.0025	-1.5	4.53	61224.7	5.7	HUMAN	P10809	60 kDa heat shock protein, mitochondrial
1910	dirMS_041712_NAR_30mM_plus2.11479.11529.2	2	16.41	0	4.70E+05		(K)IVVVTA(	(K)I/V|V|V/T/A G V/R(Q)		113.82	457.2931	913.583	-0.004	-4.4	9.75	25445.8	5.52	HUMAN	A8MW50	L-lactate dehydrogenase (Fragment)
1911	dirMS_041712_NAR_30mM_plus2.13171.13478.2	2	16.41	0	5.84E+06		(R)YLYEIAR	(R)Y/L|Y/E/I/A/R(R)		123.2	464.2507	927.493	0.0007	0.7	6	53112	6.64	HUMAN	H0YA55	Serum albumin (Fragment)
1912	dirMS_041712_NAR_30mM_minus2.16596.16596.2	2	16.4	0	2.83E+05		(R)ASLEAAI	(R)A S/L E/A/A|I/A/D/A\E Q/R(G)		143.85	672.8404	1344.675	-0.0019	-1.4	4.14	56607.4	5.37	HUMAN	F8VXB4	Keratin, type II cytoskeletal 8
1913	dirMS_041712_NAR_30mM_minus2.18749.19104.2	2	16.4	0	1.14E+06		(R)ALLFIPR	(R)A/L|L|F|I/P/R(R)		157.52	415.2685	829.529	0.0003	0.3	9.79	83605	4.97	HUMAN	P08238	Heat shock protein HSP 90-beta
1914	dirMS_041712_NAR_50mM_minus2.6231.6231.3	3	16.4	0	2.41E+05	M434m	(R)LESGmC	(R)L/E|S\G m/Q N/M/S\I H/T/K(T)	m:Oxidized methionine	98.83	497.9018	1475.698	15.9927	-1.5	6.75	56607.4	5.37	HUMAN	F8VXB4	Keratin, type II cytoskeletal 8
1915	dirMS_041712_NAR_60mM_minus.5063.5314.3	3	16.4	0	3.20E+06		(R)AEAEAG	(R)A E|A|E|A/G S P R P D|P/R(E)		83.35	451.5562	1352.655	-0.0013	-0.9	4.68	558007.1	5.44	HUMAN	P58107	Epiplakin
1916	dirMS_041712_NAR_120mM_plus.9732.9761.3	3	16.4	0	2.73E+06		(R)QSVEND	(R)Q S/V/E N/D/I/H/G L/R(K)		104.47	423.2188	1267.639	0.0029	2.3	5.32	43773.3	5.26	HUMAN	F8VZY9	Keratin, type I cytoskeletal 18
1917	dirMS_041712_NAR_20mM_plus.17590.17590.2	2	16.4	0	8.72E+04	M148m	(K)TFAPEEI	(K)T F|A/P E E I/S A m/V L\T K(M)	m:Oxidized methionine	193.43	776.8996	1536.798	15.9941	-0.5	4.53	72445.7	5.07	HUMAN	P11021	78 kDa glucose-regulated protein
1918	dirMS_041712_NAR_20mM_plus.18813.19032.2	2	16.4	0	1.21E+06		(K)EIIDLVLI	(K)E I|I|D|L/V/L/D/R(I)		202.38	543.313	1085.62	-0.0014	-1.3	4.03	58642	4.95	HUMAN	F5H5D3	Tubulin alpha-1C chain
1919	dirMS_041712_NAR_50mM_plus2.12287.12287.3	3	16.4	1	3.20E+05		(K)LSAEKVE	(K)L/S\A/E K/V E/I/A/T/L/T R(E)		132.33	477.6113	1430.821	-0.002	-1.4	6.14	26661.1	7.81	HUMAN	H0YL69	Proteasome subunit alpha type (Fragment)
1920	dirMS_041712_NAR_50mM_plus2.16428.16428.3	3	16.4	0	5.57E+05		(R)QVVQGI	(R)Q V|V/Q|G|L\L S/E T/Y/L|E/A H R(I)		154.53	614.9984	1842.971	0.0098	5.3	5.4	81934.1	6.08	HUMAN	P33993	DNA replication licensing factor MCM7
1921	dirMS_041712_NAR_50mM_plus2.8224.8224.3	3	16.4	1	3.71E+05		(R)VLVNDA	(R)V/L/V/N/D A Q/K V/T E\G Q Q E R(L)		109.88	605.3151	1813.94	-0.0095	-5.2	4.68	90316.8	5.52	HUMAN	Q13263	Transcription intermediary factor 1-beta
1922	dirMS_041712_NAR_60mM_plus.9167.9167.3	3	16.4	1	8.85E+05		(K)AQAEVE	(K)A Q|A|E|V/E/G/L G K/G V A R(L)		105.75	462.2549	1384.754	-0.0042	-3	6.19	533778	5.74	HUMAN	Q15149	Plectin
1923	dirMS_041712_NAR_20mM_minus.14706.14880.2	2	16.39	0	3.01E+06		(K)EIIDLVLI	(K)E I|I|D|L/V/L/D/R(I)		203.67	543.3133	1085.62	-0.0008	-0.7	4.03	58642	4.95	HUMAN	F5H5D3	Tubulin alpha-1C chain
1924	dirMS_041712_NAR_30mM_minus2.10956.11131.2	2	16.39	0	6.58E+05		(K)VAGQD(	(K)V/A|G\Q/D|G/S V/V/Q\F K(I)		114	617.824	1234.643	-0.0019	-1.5	5.81	17127.4	10.05	HUMAN	A8MU27	Small ubiquitin-related modifier 3
1925	dirMS_041712_NAR_30mM_minus2.12237.12720.2	2	16.39	0	5.64E+05		(K)GDYPLE/	(K)G D/Y|P/L/E/A/V/R(M)		122.22	510.2598	1019.516	-0.0033	-3.2	4.37	18466.1	5.78	HUMAN	H3BSU3	Pyruvate kinase (Fragment)
1926	dirMS_041712_NAR_30mM_minus2.13683.13683.2	2	16.39	0	6.08E+05		(K)NLVTED'	(K)N L|V|T/E D/V M\R(M)		127.03	538.7747	1076.541	0.0017	1.5	4.37	12797.5	10.58	HUMAN	K7EIL4	Glucose-6-phosphate isomerase (Fragment)
1927	dirMS_041712_NAR_30mM_minus2.9615.9795.2	2	16.39	1	9.31E+05		(K)AIYKGP(	(K)A I|Y/K/G P G S E|A|G|P(-)		107.28	573.7932	1146.579	0.0002	0.1	6.04	30492.9	5.26	HUMAN	P21964	Catechol O-methyltransferase
1928	dirMS_041712_NAR_120mM_plus.10866.11191.3	3	16.39	0	1.51E+06		(R)GSAVHC	(R)G S|A/V/H/Q/L/S/E/E/L/R(C)		111.13	442.5657	1325.681	0.0017	1.3	5.4	558007.1	5.44	HUMAN	P58107	Epiplakin
1929	dirMS_041712_NAR_120mM_plus.9689.9729.3	3	16.39	1	4.94E+05		(K)LTRDETI	(K)L T R D E/T\N|Y/G|I|P Q/R(A)		104.2	521.6023	1562.792	0.0002	0.1	6.07	13083.6	6.26	HUMAN	D6R909	Guanine nucleotide-binding protein subunit beta-2-like 1
1930	dirMS_041712_NAR_20mM_plus.10627.10708.2	2	16.39	0	2.04E+05		(K)NAGVEC	(K)N A|G|V|E|G S L/I/V/E/K(I)		146.07	608.3304	1215.658	-0.0044	-3.6	4.53	61224.7	5.7	HUMAN	P10809	60 kDa heat shock protein, mitochondrial
1931	dirMS_041712_NAR_30mM_plus2.10697.10698.2	2	16.39	0	2.62E+05		(K)DQVANS	(K)D Q|V|A|N/S/A/F/V/E/R(L)		109.43	618.3057	1235.602	0.0026	2.1	4.37	85057.6	4.94	HUMAN	P07900	Heat shock protein HSP 90-alpha
1932	dirMS_041712_NAR_30mM_plus2.8255.8569.2	2	16.39	0	1.33E+06		(K)YICENQI	(K)Y/I|C|E N Q D S/I|S/S K(L)	C:Carbamidomethylation	97.88	722.3239	1443.642	-0.0015	-1	4.37	57751.3	6.9	HUMAN	B7WNR0	Serum albumin
1933	dirMS_041712_NAR_50mM_plus2.17639.17639.3	3	16.39	1	8.31E+05		(R)DSEQVA	(R)D S|E/Q/V/A E/L K Q E L A T L K(S)		161.07	601.322	1801.954	-0.0027	-1.5	4.41	109459.8	4.94	HUMAN	F5GYR8	General vesicular transport factor p115
1934	dirMS_041712_NAR_60mM_plus.18347.18410.3	3	16.39	1	1.76E+06		(K)QLDDLK'	(K)Q L/D/D/L/K/V/E/L/S Q/L R(V)		154.27	519.6254	1556.864	-0.0026	-1.7	4.56	10815.7	10.72	HUMAN	F2Z388	60S ribosomal protein L35
1935	dirMS_041712_NAR_60mM_plus.9791.9791.3	3	16.39	0	7.66E+05		(R)QQEGFK	(R)Q Q/E/G/F K/P N/Y M E/N/R(E)		109.3	547.5879	1640.749	0.0006	0.4	6.14	58856.7	6.26	HUMAN	Q8WXF1	Paraspeckle component 1
1936	dirMS_041712_NAR_50mM_minus2.16353.16353.3	3	16.38	1	5.41E+04		(K)SELVAM	(K)S E|L|V/A M/L E/E E E L/R K(A)		161.32	559.2917	1675.857	0.0034	2	4.32	18679.2	5.61	HUMAN	B4DWW1	ADP-ribosylation factor-like protein 1
1937	dirMS_041712_NAR_20mM_plus.18330.18446.3	3	16.38	0	3.15E+05		(R)AAVEEG	(R)A A\V E E\G I\V|L/G|G/G/C/A L L R(C)	C:Carbamidomethylation	198.02	562.3058	1684.905	-0.0022	-1.3	4.53	61224.7	5.7	HUMAN	P10809	60 kDa heat shock protein, mitochondrial
1938	dirMS_041712_NAR_30mM_plus2.8094.8384.2	2	16.38	0	1.36E+06		(K)QGGGG(	(K)Q G\G G/G|G/G/G/S/V|P G I/E/R(M)		97.17	642.8178	1284.629	-0.0008	-0.6	6	77799.7	9.09	HUMAN	P52272	Heterogeneous nuclear ribonucleoprotein M
1939	dirMS_041712_NAR_40mM_plus2.11892.12028.3	3	16.38	1	2.81E+05		(K)LIADVAF	(K)L/I|A|D|V|A/P S A I R E N D/I/K(S)		136.47	575.6528	1724.954	-0.0103	-6	4.56	18388.8	6.85	HUMAN	F5H1Y3	Flap endonuclease 1 (Fragment)
1940	dirMS_041712_NAR_50mM_plus2.10749.10749.3	3	16.38	1	5.26E+05		(R)VAVEEV	(R)V/A|V/E/E/V/D E E/G K F V/R(L)		124.4	535.9422	1605.812	0.0002	0.1	4.25	63136.9	5.83	HUMAN	D6RAQ3	Prelamin-A/C
1941	dirMS_041712_NAR_50mM_plus2.11372.11372.3	3	16.38	1	1.25E+06		(K)NQVAM	(K)N Q|V|A M/N/P T/N T V/F D A K R(L)		127.8	602.6354	1805.896	-0.0046	-2.6	8.75	20069.4	6.74	HUMAN	E9PK54	Heat shock cognate 71 kDa protein (Fragment)
1942	dirMS_041712_NAR_50mM_minus2.11477.11477.3	3	16.37	0	2.35E+04		(R)LIAEQPF	(R)L/I/A E Q/P P H L/T P/G I R(E)		133.68	514.6309	1541.88	-0.0017	-1.1	6.75	20915.7	6.45	HUMAN	C9JBI3	Phosphoserine phosphatase (Fragment)
1943	dirMS_041712_NAR_50mM_minus2.17753.17753.3	3	16.37	1	3.97E+05		(R)LKLPSIPI	(R)L K L P S I|P|L|V/P V/S A Q K(R)		169.73	530.669	1589.999	-0.0064	-4.1	10	34958.1	9.42	HUMAN	Q15785	Mitochondrial import receptor subunit TOM34
1944	dirMS_041712_NAR_50mM_minus2.9052.9090.3	3	16.37	0	2.39E+05		(R)GISEETT	(R)G I|S/E/E/T/T/T/G V/H N L Y K(M)		119.9	550.2763	1648.818	-0.0033	-2	5.4	45228.3	6.03	HUMAN	F5H737	Adenosylhomocysteinase
1945	dirMS_041712_NAR_20mM_plus.16612.16905.2	2	16.37	0	4.12E+05		(K)VQIAVA	(K)V Q|I\A/V|A|N A/Q/E/L L/Q R(M)		187.28	776.9419	1552.881	-0.004	-2.6	5.97	10728.1	9.15	HUMAN	Q9Y5L4	Mitochondrial import inner membrane translocase subunit Tim13
1946	dirMS_041712_NAR_30mM_plus2.14075.14325.3	3	16.37	0	1.32E+06		(R)MGQM/	(R)M/G\Q\M A\M/G G\A|M/G|I/N/N R(G)		127.32	513.5611	1538.67	-0.0007	-0.5	9.5	43979.5	8.92	HUMAN	F5GYZ3	Non-POU domain-containing octamer-binding protein
1947	dirMS_041712_NAR_50mM_plus2.16209.16209.3	3	16.37	0	2.89E+05		(K)ACQSIYF	(K)A C/Q S/I/Y/P L/H/D V F V R(K)	C:Carbamidomethylation	154.4	568.9562	1704.853	0.0014	0.8	6.79	16650	9.47	HUMAN	D6R9B6	40S ribosomal protein S3a
1948	dirMS_041712_NAR_20mM_minus.13918.13952.2	2	16.36	0	8.14E+05		(K)VLTPELY	(K)V L|T|P/E/L Y A E/L R(A)		191.32	652.3653	1303.726	-0.0023	-1.8	4.53	14391.8	4.99	HUMAN	G3V461	Creatine kinase B-type (Fragment)
1949	dirMS_041712_NAR_20mM_plus.14296.14643.3	3	16.36	0	8.76E+05	M73m	(R)AVLVDLI	(R)A V|L\V D L\E/P/G/T/m/D S V R(S)	m:Oxidized methionine	171.03	539.9421	1601.82	15.9914	-2.2	4.03	13112.4	5.23	HUMAN	F8W813	Tubulin beta-8 chain
1950	dirMS_041712_NAR_50mM_plus2.12011.12046.3	3	16.36	0	1.29E+06		(R)VYNVTQ	(R)V/Y/N/V/T/Q/H A\V/G I V/V/N K(Q)		131.48	547.6436	1640.912	0.0044	2.7	8.57	10000.5	10.56	HUMAN	G3V1B3	60S ribosomal protein L21
1951	dirMS_041712_NAR_50mM_plus2.16888.16888.3	3	16.36	1	8.57E+05		(K)LFTAESL	(K)L/F|T\A/E S L/I/G L K/N P E/K(S)		157.48	553.9791	1659.932	-0.0089	-5.3	6.14	62195.8	5.63	HUMAN	B0YIW6	Archain 1, isoform CRA_a
1952	dirMS_041712_NAR_50mM_plus2.19676.20162.3	3	16.35	1	3.18E+06		(R)KAIIIFVP	(R)K\A I I\I\F|V|P/V P Q/L K(S)		173.15	489.3212	1465.95	-0.0014	-1	10	21311.5	10.28	HUMAN	B5MCP9	40S ribosomal protein S7
1953	dirMS_041712_NAR_60mM_plus.13044.13045.3	3	16.35	1	1.81E+05		(R)GLAGLG	(R)G L|A/G/L/G D/V/A E/V R/K(S)		124.92	428.9129	1284.727	-0.0029	-2.2	6.07	97378.8	6.4	HUMAN	P11216	Glycogen phosphorylase, brain form
1954	dirMS_041712_NAR_60mM_plus.6820.6820.3	3	16.35	0	5.16E+05		(R)ATAPQT	(R)A T|A/P Q T Q/H/V/S/P M R(Q)		94.17	475.2423	1423.711	0.0013	0.9	9.8	29184.1	4.91	HUMAN	E9PBQ9	Elongation factor 1-delta
1955	dirMS_041712_NAR_20mM_minus.12302.12511.2	2	16.34	0	5.88E+05	M73m	(R)AVLVDLI	(R)A V/L|V/D/L|E|P/G T\m D S V R(S)	m:Oxidized methionine	172.52	809.4088	1601.82	15.99	-3	4.03	13112.4	5.23	HUMAN	F8W813	Tubulin beta-8 chain
1956	dirMS_041712_NAR_30mM_minus2.17806.17945.2	2	16.34	0	2.89E+05		(R)NNLAGA	(R)N N|L|A|G/A/E E/L/F A/R(K)		151.62	652.8358	1304.659	0.005	3.8	4.53	193379.8	5.48	HUMAN	Q00610	Clathrin heavy chain 1
1957	dirMS_041712_NAR_40mM_minus2.14772.14772.3	3	16.34	1	1.48E+06	M116m	(R)ELAILLG	(R)E L/A/I/L L/G m L D/P A E/K D/E K(G)	m:Oxidized methionine	171.23	634.338	1884.999	16.0008	3.1	4.18	25148.6	6	HUMAN	P30041	Peroxiredoxin-6

<table>
<tr><th></th><th></th><th></th><th></th><th></th><th></th><th></th><th></th><th></th><th></th><th></th><th></th><th></th><th></th><th></th><th></th><th></th><th></th><th></th><th></th><th></th></tr>
<tr><td>1958</td><td>dirMS_041712_NAR_50mM_minus2.17273.17273.3</td><td>3</td><td>16.34</td><td>0</td><td>1.05E+06</td><td>M195m</td><td>(R)LHFFmP</td><td>(R)L H F F m|P G|F|A/P L T S R(G)</td><td>m:Oxidized methionine</td><td>167.33</td><td>546.2872</td><td>1620.835</td><td>16.0115</td><td>10.1</td><td>9.75</td><td>42230.8</td><td>4.77</td><td>HUMAN</td><td>A6NKZ8</td><td>Putative tubulin beta chain-like protein ENSP00000290377</td></tr>
<tr><td>1959</td><td>dirMS_041712_NAR_60mM_minus.11605.11605.3</td><td>3</td><td>16.34</td><td>0</td><td>7.51E+05</td><td></td><td>(K)TKPYIQV</td><td>(K)T K\P Y I Q\V|D|I/G G G Q T K(T)</td><td></td><td>124.67</td><td>535.6216</td><td>1604.864</td><td>-0.014</td><td>-8.7</td><td>8.17</td><td>72445.7</td><td>5.07</td><td>HUMAN</td><td>P11021</td><td>78 kDa glucose-regulated protein</td></tr>
<tr><td>1960</td><td>dirMS_041712_NAR_60mM_minus.18465.18465.3</td><td>3</td><td>16.34</td><td>0</td><td>5.02E+04</td><td>M195m</td><td>(R)LHFFmP</td><td>(R)L H\F F m/P G|F/A|P L/T S R(G)</td><td>m:Oxidized methionine</td><td>164.37</td><td>546.2843</td><td>1620.835</td><td>16.0028</td><td>4.8</td><td>9.75</td><td>42230.8</td><td>4.77</td><td>HUMAN</td><td>A6NKZ8</td><td>Putative tubulin beta chain-like protein ENSP00000290377</td></tr>
<tr><td>1961</td><td>dirMS_041712_NAR_20mM_plus.9325.9325.2</td><td>2</td><td>16.34</td><td>0</td><td>1.86E+05</td><td></td><td>(K)SVTNED</td><td>(K)S V/T/N|E|D V/T Q/E E/L/G G A K(T)</td><td></td><td>136</td><td>838.9014</td><td>1676.797</td><td>-0.0018</td><td>-1.1</td><td>4</td><td>61363</td><td>8.73</td><td>HUMAN</td><td>C9JQS9</td><td>Propionyl-CoA carboxylase beta chain, mitochondrial</td></tr>
<tr><td>1962</td><td>dirMS_041712_NAR_40mM_plus2.7506.7506.2</td><td>2</td><td>16.34</td><td>0</td><td>3.99E+05</td><td></td><td>(K)ASLENSL</td><td>(K)A S/L/E/N/S L/R(E)</td><td></td><td>105.08</td><td>445.2395</td><td>889.474</td><td>-0.002</td><td>-2.3</td><td>6.05</td><td>43773.3</td><td>5.26</td><td>HUMAN</td><td>F8VZY9</td><td>Keratin, type I cytoskeletal 18</td></tr>
<tr><td>1963</td><td>dirMS_041712_NAR_50mM_plus2.15498.15498.3</td><td>3</td><td>16.34</td><td>1</td><td>9.99E+05</td><td></td><td>(R)KVPGVT</td><td>(R)K\V\P G V T A I\E\L/D/E/D/T/G T F R(I)</td><td></td><td>149.85</td><td>650.0048</td><td>1948.002</td><td>-0.0024</td><td>-1.2</td><td>4.32</td><td>68668.3</td><td>5.77</td><td>HUMAN</td><td>B4DXZ6</td><td>Fragile X mental retardation syndrome-related protein 1</td></tr>
<tr><td>1964</td><td>dirMS_041712_NAR_60mM_plus.12958.13020.3</td><td>3</td><td>16.34</td><td>1</td><td>1.36E+06</td><td></td><td>(K)NQVAM</td><td>(K)N Q|V|A/M/N/P T/N/T/V/F D A K R(L)</td><td></td><td>124.48</td><td>602.6367</td><td>1805.896</td><td>-0.0007</td><td>-0.4</td><td>8.75</td><td>20069.4</td><td>6.74</td><td>HUMAN</td><td>E9PK54</td><td>Heat shock cognate 71 kDa protein (Fragment)</td></tr>
<tr><td>1965</td><td>dirMS_041712_NAR_60mM_plus.16347.16465.3</td><td>3</td><td>16.34</td><td>0</td><td>4.79E+05</td><td></td><td>(R)EAYPGD</td><td>(R)E A/Y/P/G D V/F Y L/H/S R(L)</td><td></td><td>142.62</td><td>518.5855</td><td>1553.738</td><td>0.0036</td><td>2.3</td><td>5.32</td><td>48851.3</td><td>9.2</td><td>HUMAN</td><td>K7EK63</td><td>ATP synthase subunit alpha, mitochondrial</td></tr>
<tr><td>1966</td><td>dirMS_041712_NAR_30mM_minus2.19569.19627.2</td><td>2</td><td>16.33</td><td>0</td><td>4.70E+05</td><td></td><td>(R)ASLSLW</td><td>(R)A S/L/S/L|W/Q/A/M E K(G)</td><td></td><td>162.75</td><td>632.3234</td><td>1263.64</td><td>-0.0007</td><td>-0.5</td><td>6.05</td><td>266161</td><td>5.68</td><td>HUMAN</td><td>E9PPU0</td><td>Epiplakin</td></tr>
<tr><td>1967</td><td>dirMS_041712_NAR_30mM_plus2.17524.17549.2</td><td>2</td><td>16.33</td><td>0</td><td>1.64E+05</td><td></td><td>(R)ASLEAAI</td><td>(R)A S L|E/A|A|I/A|D\A/E Q R(G)</td><td></td><td>145.03</td><td>672.8406</td><td>1344.675</td><td>-0.0015</td><td>-1.1</td><td>4.14</td><td>56607.4</td><td>5.37</td><td>HUMAN</td><td>F8VXB4</td><td>Keratin, type II cytoskeletal 8</td></tr>
<tr><td>1968</td><td>dirMS_041712_NAR_30mM_plus2.7606.7646.2</td><td>2</td><td>16.33</td><td>0</td><td>9.52E+05</td><td></td><td>(K)STCIYGG</td><td>(K)S/T|C|I/Y/G G A/P/K(G)</td><td>C:Carbamidomethylation</td><td>94.2</td><td>527.2559</td><td>1053.503</td><td>0.0012</td><td>1.1</td><td>8.31</td><td>61018.6</td><td>9.44</td><td>HUMAN</td><td>B4DLW8</td><td>Probable ATP-dependent RNA helicase DDX5</td></tr>
<tr><td>1969</td><td>dirMS_041712_NAR_50mM_plus2.15971.16027.3</td><td>3</td><td>16.33</td><td>1</td><td>7.23E+05</td><td></td><td>(R)EGLTSIE</td><td>(R)E/G/L|T/S/I/E E/V/T K/N L Q/K(F)</td><td></td><td>152.15</td><td>563.6357</td><td>1688.907</td><td>-0.014</td><td>-8.3</td><td>4.79</td><td>533778</td><td>5.74</td><td>HUMAN</td><td>Q15149</td><td>Plectin</td></tr>
<tr><td>1970</td><td>dirMS_041712_NAR_50mM_plus2.21792.21792.3</td><td>3</td><td>16.33</td><td>1</td><td>1.26E+05</td><td></td><td>(K)TENPLIL</td><td>(K)T E|N/P L/I/L I/D E V/D/K\I/G R(G)</td><td></td><td>185.57</td><td>609.0045</td><td>1825.007</td><td>-0.0076</td><td>-4.2</td><td>4.32</td><td>86039.3</td><td>5.74</td><td>HUMAN</td><td>F5GZ27</td><td>Lon protease homolog</td></tr>
<tr><td>1971</td><td>dirMS_041712_NAR_60mM_plus.18327.18552.3</td><td>3</td><td>16.33</td><td>1</td><td>2.81E+06</td><td></td><td>(K)QLDDLK</td><td>(K)Q L|D/D/L/K/V/E/L/S/Q L R(V)</td><td></td><td>154.67</td><td>519.6254</td><td>1556.864</td><td>-0.0026</td><td>-1.7</td><td>4.56</td><td>10815.7</td><td>10.72</td><td>HUMAN</td><td>F2Z388</td><td>60S ribosomal protein L35</td></tr>
<tr><td>1972</td><td>dirMS_041712_NAR_50mM_minus2.6821.6822.3</td><td>3</td><td>16.32</td><td>0</td><td>5.92E+05</td><td></td><td>(R)EATNPP</td><td>(R)E A|T/N/P/P V/I Q E/E K/P K(K)</td><td></td><td>105.52</td><td>527.2846</td><td>1579.833</td><td>0.0066</td><td>4.2</td><td>4.79</td><td>55361.8</td><td>6.42</td><td>HUMAN</td><td>G5EA52</td><td>Protein disulfide isomerase family A, member 3, isoform CRA_b</td></tr>
<tr><td>1973</td><td>dirMS_041712_NAR_30mM_plus2.8034.8610.2</td><td>2</td><td>16.32</td><td>0</td><td>7.03E+06</td><td></td><td>(K)ASLENSL</td><td>(K)A/S|L/E/N/S/L/R(E)</td><td></td><td>99.25</td><td>445.2405</td><td>889.474</td><td>0</td><td>-0.1</td><td>6.05</td><td>43773.3</td><td>5.26</td><td>HUMAN</td><td>F8VZY9</td><td>Keratin, type I cytoskeletal 18</td></tr>
<tr><td>1974</td><td>dirMS_041712_NAR_40mM_plus2.13963.13963.3</td><td>3</td><td>16.32</td><td>0</td><td>2.92E+05</td><td></td><td>(R)LASTLVH</td><td>(R)L/A|S/T L/V/H L G E|Y Q A|A|V\D G A R(K)</td><td></td><td>148.43</td><td>657.6841</td><td>1971.029</td><td>0.0083</td><td>4.2</td><td>5.32</td><td>71165.3</td><td>5.57</td><td>HUMAN</td><td>J3KS13</td><td>Clathrin heavy chain 1</td></tr>
<tr><td>1975</td><td>dirMS_041712_NAR_50mM_plus2.20117.20118.3</td><td>3</td><td>16.32</td><td>0</td><td>3.51E+05</td><td></td><td>(R)NEVSFVI</td><td>(R)N E|V/S/F V/I/H|N L P\V/L A K(M)</td><td></td><td>174.32</td><td>560.6531</td><td>1679.948</td><td>-0.0032</td><td>-1.9</td><td>6.75</td><td>35663.7</td><td>5.91</td><td>HUMAN</td><td>B4DWK0</td><td>Selenide, water dikinase 1</td></tr>
<tr><td>1976</td><td>dirMS_041712_NAR_40mM_minus2.11652.11652.3</td><td>3</td><td>16.31</td><td>1</td><td>1.06E+05</td><td></td><td>(R)IINEPTA</td><td>(R)I I/N\E/P T/A A/A I A Y/G L/D/K K(V)</td><td></td><td>152.32</td><td>596.6671</td><td>1787.99</td><td>-0.0034</td><td>-1.9</td><td>6.07</td><td>17973</td><td>5.16</td><td>HUMAN</td><td>E9PI65</td><td>Heat shock cognate 71 kDa protein (Fragment)</td></tr>
<tr><td>1977</td><td>dirMS_041712_NAR_50mM_minus2.13447.13447.3</td><td>3</td><td>16.31</td><td>0</td><td>3.52E+05</td><td></td><td>(R)AVAIDLF</td><td>(R)A V\A/I/D L/P/G L G/H/S K(E)</td><td></td><td>144.57</td><td>426.5785</td><td>1277.721</td><td>-0.0003</td><td>-0.2</td><td>6.79</td><td>19983.6</td><td>8.14</td><td>HUMAN</td><td>B4DQI4</td><td>Alpha/beta hydrolase domain-containing protein 14B</td></tr>
<tr><td>1978</td><td>dirMS_041712_NAR_50mM_minus2.14267.14267.3</td><td>3</td><td>16.31</td><td>0</td><td>2.61E+05</td><td></td><td>(R)TIQVENS</td><td>(R)T I|Q/V/E N S H L I L/T/G A G A L N K(V)</td><td></td><td>149.02</td><td>660.367</td><td>1979.092</td><td>-0.0056</td><td>-2.8</td><td>6.41</td><td>267260.7</td><td>5.95</td><td>HUMAN</td><td>Q13085</td><td>Acetyl-CoA carboxylase 1</td></tr>
<tr><td>1979</td><td>dirMS_041712_NAR_60mM_minus.10027.10069.3</td><td>3</td><td>16.31</td><td>0</td><td>1.56E+06</td><td></td><td>(R)EIVHIQA</td><td>(R)E/I\V H I Q A G/Q|C|G N Q I/G A/K(F)</td><td>C:Carbamidomethylation</td><td>115.57</td><td>608.3099</td><td>1822.923</td><td>-0.0077</td><td>-4.2</td><td>6.85</td><td>40069.4</td><td>4.83</td><td>HUMAN</td><td>E7EWR1</td><td>Tubulin beta chain</td></tr>
<tr><td>1980</td><td>dirMS_041712_NAR_120mM_plus.10407.10407.3</td><td>3</td><td>16.31</td><td>0</td><td>4.82E+05</td><td></td><td>(K)NLPLAD</td><td>(K)N L/P/L/A D/Q/G S/S H H I T V K(I)</td><td></td><td>107.58</td><td>572.9712</td><td>1716.903</td><td>-0.0037</td><td>-2.2</td><td>6.92</td><td>334221.3</td><td>6.44</td><td>HUMAN</td><td>P15924</td><td>Desmoplakin</td></tr>
<tr><td>1981</td><td>dirMS_041712_NAR_60mM_plus.6427.6444.3</td><td>3</td><td>16.31</td><td>1</td><td>2.97E+05</td><td></td><td>(K)VATQEG</td><td>(K)V/A|T/Q/E/G K/E I/T/C R(S)</td><td>C:Carbamidomethylation</td><td>92.3</td><td>464.5649</td><td>1391.695</td><td>-0.0146</td><td>-10.5</td><td>6.11</td><td>18626.5</td><td>5.64</td><td>HUMAN</td><td>B8ZZK2</td><td>Gamma-glutamylcyclotransferase</td></tr>
<tr><td>1982</td><td>dirMS_041712_NAR_20mM_minus.13261.13261.3</td><td>3</td><td>16.3</td><td>0</td><td>2.65E+05</td><td></td><td>(K)VGINYQ</td><td>(K)V G I N Y Q|P P T\V/V/P/G/G D L A K(V)</td><td></td><td>183.15</td><td>608.9971</td><td>1824.985</td><td>-0.0087</td><td>-4.8</td><td>5.8</td><td>49012.3</td><td>4.9</td><td>HUMAN</td><td>A8MUB1</td><td>Tubulin alpha-4A chain</td></tr>
<tr><td>1983</td><td>dirMS_041712_NAR_50mM_minus2.12995.13237.3</td><td>3</td><td>16.3</td><td>1</td><td>3.35E+05</td><td></td><td>(R)ALPAVQ</td><td>(R)A L|P/A/V/Q/Q N N L/D/E D/L I R K(L)</td><td></td><td>142.8</td><td>646.3522</td><td>1937.045</td><td>-0.003</td><td>-1.6</td><td>4.56</td><td>118930.9</td><td>5.49</td><td>HUMAN</td><td>P22314</td><td>Ubiquitin-like modifier-activating enzyme 1</td></tr>
<tr><td>1984</td><td>dirMS_041712_NAR_50mM_minus2.8670.8670.3</td><td>3</td><td>16.3</td><td>0</td><td>1.55E+06</td><td></td><td>(R)EIVHIQA</td><td>(R)E/I/V H I Q A G\Q/C|G N Q I/G A K(F)</td><td>C:Carbamidomethylation</td><td>116.82</td><td>608.3087</td><td>1822.923</td><td>-0.0113</td><td>-6.2</td><td>6.85</td><td>40069.4</td><td>4.83</td><td>HUMAN</td><td>E7EWR1</td><td>Tubulin beta chain</td></tr>
<tr><td>1985</td><td>dirMS_041712_NAR_20mM_plus.10512.10626.2</td><td>2</td><td>16.3</td><td>0</td><td>3.12E+05</td><td></td><td>(K)VLQATV</td><td>(K)V/L/Q|A|T|V/V/A V/G S\G S K(G)</td><td></td><td>145.67</td><td>658.3823</td><td>1315.758</td><td>-0.0007</td><td>-0.5</td><td>8.72</td><td>10803.5</td><td>9.82</td><td>HUMAN</td><td>B8ZZL8</td><td>10 kDa heat shock protein, mitochondrial</td></tr>
<tr><td>1986</td><td>dirMS_041712_NAR_20mM_plus.18841.18841.2</td><td>2</td><td>16.3</td><td>0</td><td>1.01E+05</td><td></td><td>(K)LTVYTTL</td><td>(K)L T/V|Y/T/T L\I/D/V T K(G)</td><td></td><td>200.87</td><td>683.8948</td><td>1366.783</td><td>-0.0005</td><td>-0.4</td><td>5.83</td><td>42194.9</td><td>8.75</td><td>HUMAN</td><td>E7EMM4</td><td>Acid ceramidase</td></tr>
<tr><td>1987</td><td>dirMS_041712_NAR_50mM_minus2.14835.14835.3</td><td>3</td><td>16.29</td><td>1</td><td>2.96E+05</td><td></td><td>(R)EAVTEIL</td><td>(R)E/A|V/T/E/I L G I/E/P D R E K(G)</td><td></td><td>152.32</td><td>566.9683</td><td>1698.891</td><td>-0.0005</td><td>-0.3</td><td>4.25</td><td>25685.9</td><td>6.4</td><td>HUMAN</td><td>E9PGT1</td><td>Translin</td></tr>
<tr><td>1988</td><td>dirMS_041712_NAR_120mM_plus.5719.6114.3</td><td>3</td><td>16.29</td><td>1</td><td>1.19E+06</td><td></td><td>(R)TAQEVE</td><td>(R)T/A|Q/E V/E/T Y R/R(S)</td><td></td><td>84.27</td><td>418.2137</td><td>1252.628</td><td>-0.0015</td><td>-1.2</td><td>5.81</td><td>61018.6</td><td>9.44</td><td>HUMAN</td><td>B4DLW8</td><td>Probable ATP-dependent RNA helicase DDX5</td></tr>
<tr><td>1989</td><td>dirMS_041712_NAR_120mM_plus.9444.9476.3</td><td>3</td><td>16.29</td><td>1</td><td>3.69E+05</td><td></td><td>(R)SRSGEG</td><td>(R)S R S G E\G\E|V|S/G/L/M/R(K)</td><td></td><td>102.8</td><td>455.5574</td><td>1364.659</td><td>-0.001</td><td>-0.8</td><td>5.87</td><td>90316.8</td><td>5.52</td><td>HUMAN</td><td>Q13263</td><td>Transcription intermediary factor 1-beta</td></tr>
<tr><td>1990</td><td>dirMS_041712_NAR_20mM_plus.6946.6946.2</td><td>2</td><td>16.29</td><td>0</td><td>8.44E+04</td><td>M29m</td><td>(K)IVQmTE</td><td>(K)I/V|Q|m/T E/A/E V R(G)</td><td>m:Oxidized methionine</td><td>114.35</td><td>596.3035</td><td>1175.609</td><td>15.9908</td><td>-3.4</td><td>4.53</td><td>14493.5</td><td>5.23</td><td>HUMAN</td><td>C9J9S3</td><td>Serine/threonine-protein phosphatase (Fragment)</td></tr>
<tr><td>1991</td><td>dirMS_041712_NAR_30mM_plus2.9548.9559.2</td><td>2</td><td>16.29</td><td>0</td><td>4.96E+05</td><td></td><td>(K)TAVCDIF</td><td>(K)T A|V|C/D|I|P|P/R(G)</td><td>C:Carbamidomethylation</td><td>105.07</td><td>514.7643</td><td>1028.519</td><td>0.002</td><td>1.9</td><td>5.5</td><td>50199.4</td><td>4.75</td><td>HUMAN</td><td>A6NNZ2</td><td>Tubulin beta-8 chain-like protein LOC260334</td></tr>
<tr><td>1992</td><td>dirMS_041712_NAR_50mM_plus2.10075.10075.3</td><td>3</td><td>16.29</td><td>1</td><td>5.94E+04</td><td></td><td>(K)ITIKNDP</td><td>(K)I/T|I/K\N D P S L P/E/P K(C)</td><td></td><td>120.53</td><td>484.6079</td><td>1451.81</td><td>-0.0013</td><td>-0.9</td><td>6.07</td><td>20203.8</td><td>9.79</td><td>HUMAN</td><td>B4DG16</td><td>Asparagine--tRNA ligase, cytoplasmic</td></tr>
<tr><td>1993</td><td>dirMS_041712_NAR_60mM_plus.17903.18000.3</td><td>3</td><td>16.29</td><td>1</td><td>3.14E+04</td><td></td><td>(K)MFGIDR</td><td>(K)M/F|G/I/D/R D A\I/A/Q/A V R(G)</td><td></td><td>152.45</td><td>521.6038</td><td>1562.811</td><td>-0.0139</td><td>-8.9</td><td>5.71</td><td>63448.4</td><td>7.29</td><td>HUMAN</td><td>B4E022</td><td>Transketolase</td></tr>
<tr><td>1994</td><td>dirMS_041712_NAR_20mM_minus.9260.9260.2</td><td>2</td><td>16.28</td><td>0</td><td>2.79E+05</td><td></td><td>(R)AQIFAN</td><td>(R)A Q|I|F/A/N/T\V D N/A R(I)</td><td></td><td>142.32</td><td>660.3388</td><td>1319.67</td><td>0.0001</td><td>0.1</td><td>5.88</td><td>43773.3</td><td>5.26</td><td>HUMAN</td><td>F8VZY9</td><td>Keratin, type I cytoskeletal 18</td></tr>
<tr><td>1995</td><td>dirMS_041712_NAR_50mM_minus2.9723.9742.3</td><td>3</td><td>16.28</td><td>0</td><td>3.73E+05</td><td></td><td>(R)EALLSSA</td><td>(R)E A|L/L/S/S A\V\D/H G S D E/V/K(F)</td><td></td><td>123.88</td><td>552.9403</td><td>1656.807</td><td>-0.0012</td><td>-0.7</td><td>4.31</td><td>57204.9</td><td>6.01</td><td>HUMAN</td><td>F5GWF6</td><td>T-complex protein 1 subunit beta</td></tr>
<tr><td>1996</td><td>dirMS_041712_NAR_50mM_plus2.11438.11438.3</td><td>3</td><td>16.28</td><td>1</td><td>7.45E+05</td><td></td><td>(K)LAEQAE</td><td>(K)L/A|E/Q/A/E R Y D D M/A A A M/K(A)</td><td></td><td>127.98</td><td>604.9469</td><td>1812.825</td><td>0.0007</td><td>0.4</td><td>4.32</td><td>28196</td><td>4.76</td><td>HUMAN</td><td>P31946</td><td>14-3-3 protein beta/alpha</td></tr>
<tr><td>1997</td><td>dirMS_041712_NAR_20mM_plus.14233.14379.3</td><td>3</td><td>16.27</td><td>0</td><td>6.16E+05</td><td>M256m</td><td>(R)SLDmDS</td><td>(R)S L\D\m\D S|I|I A E/V/K(A)</td><td>m:Oxidized methionine</td><td>170.42</td><td>446.2266</td><td>1320.671</td><td>15.9937</td><td>-0.9</td><td>4.03</td><td>37296.3</td><td>5.3</td><td>HUMAN</td><td>F8VU64</td><td>Keratin, type II cytoskeletal 8</td></tr>
<tr><td>1998</td><td>dirMS_041712_NAR_30mM_plus2.16578.16764.2</td><td>2</td><td>16.27</td><td>0</td><td>3.64E+05</td><td></td><td>(R)FLICTDV</td><td>(R)F/L|I|C/T D/V A A/R(G)</td><td>C:Carbamidomethylation</td><td>140.02</td><td>583.3055</td><td>1165.603</td><td>0.0003</td><td>0.3</td><td>5.84</td><td>83400.6</td><td>6.82</td><td>HUMAN</td><td>Q92499</td><td>ATP-dependent RNA helicase DDX1</td></tr>
<tr><td>1999</td><td>dirMS_041712_NAR_30mM_plus2.23357.23527.2</td><td>2</td><td>16.27</td><td>0</td><td>2.11E+06</td><td></td><td>(R)DLPLLLF</td><td>(R)D L|P|L|L|L/F/R(T)</td><td></td><td>189.25</td><td>493.8049</td><td>986.603</td><td>-0.0008</td><td>-0.8</td><td>5.84</td><td>57391.2</td><td>6.29</td><td>HUMAN</td><td>O43175</td><td>D-3-phosphoglycerate dehydrogenase</td></tr>
<tr><td>2000</td><td>dirMS_041712_NAR_40mM_plus2.12921.13214.3</td><td>3</td><td>16.27</td><td>1</td><td>9.95E+05</td><td></td><td>(K)TITLEVEI</td><td>(K)T I|T|L/E/V/E/P S D T I/E N V K A K(I)</td><td></td><td>143.45</td><td>663.0201</td><td>1987.059</td><td>-0.0136</td><td>-6.9</td><td>4.41</td><td>17271.6</td><td>6.75</td><td>HUMAN</td><td>B4DV12</td><td>Ubiquitin</td></tr>
<tr><td>2001</td><td>dirMS_041712_NAR_50mM_plus2.18614.18749.3</td><td>3</td><td>16.27</td><td>1</td><td>9.79E+05</td><td></td><td>(R)LKLPSIPI</td><td>(R)L K L/P S I|P\L\V|P V/S A Q K(R)</td><td></td><td>166.8</td><td>530.6702</td><td>1589.999</td><td>-0.0028</td><td>-1.8</td><td>10</td><td>34958.1</td><td>9.42</td><td>HUMAN</td><td>Q15785</td><td>Mitochondrial import receptor subunit TOM34</td></tr>
<tr><td>2002</td><td>dirMS_041712_NAR_50mM_plus2.9775.9775.3</td><td>3</td><td>16.27</td><td>1</td><td>1.99E+05</td><td></td><td>(R)AGTGVD</td><td>(R)A/G/T|G/V/D N V D/L E/A A/T R K(G)</td><td></td><td>118.75</td><td>539.6108</td><td>1616.824</td><td>-0.006</td><td>-3.7</td><td>4.56</td><td>57391.2</td><td>6.29</td><td>HUMAN</td><td>O43175</td><td>D-3-phosphoglycerate dehydrogenase</td></tr>
<tr><td>2003</td><td>dirMS_041712_NAR_60mM_plus.7130.7130.3</td><td>3</td><td>16.27</td><td>0</td><td>3.22E+05</td><td></td><td>(K)IFSGSSH</td><td>(K)I/F|S/G/S S H/Q D L/S Q K(I)</td><td></td><td>95.45</td><td>478.5718</td><td>1433.702</td><td>-0.0011</td><td>-0.8</td><td>6.74</td><td>18741.7</td><td>9.49</td><td>HUMAN</td><td>B1ALA7</td><td>Phosphoribosyl pyrophosphate synthetase 1</td></tr>
<tr><td>2004</td><td>dirMS_041712_NAR_50mM_minus2.16845.16948.3</td><td>3</td><td>16.26</td><td>0</td><td>4.60E+05</td><td></td><td>(K)NILFVITI</td><td>(K)N I|L|F/V/I T/K/P D V/Y K(S)</td><td></td><td>164.12</td><td>517.2991</td><td>1549.899</td><td>-0.0161</td><td>-10.4</td><td>8.5</td><td>206102.3</td><td>9.72</td><td>HUMAN</td><td>E9PAV3</td><td>Nascent polypeptide-associated complex subunit alpha</td></tr>
<tr><td>2005</td><td>dirMS_041712_NAR_20mM_plus.10467.10486.2</td><td>2</td><td>16.26</td><td>0</td><td>2.32E+05</td><td></td><td>(R)AQIFAN</td><td>(R)A Q|I|F/A|N/T/V/D N A R(I)</td><td></td><td>145.25</td><td>660.3343</td><td>1319.67</td><td>-0.0089</td><td>-6.8</td><td>5.88</td><td>43773.3</td><td>5.26</td><td>HUMAN</td><td>F8VZY9</td><td>Keratin, type I cytoskeletal 18</td></tr>
<tr><td>2006</td><td>dirMS_041712_NAR_30mM_plus2.16021.16299.2</td><td>2</td><td>16.26</td><td>0</td><td>1.14E+06</td><td></td><td>(R)GDLGIEI</td><td>(R)G D/L|G|I|E|I|P A E K(V)</td><td></td><td>137.55</td><td>571.3087</td><td>1141.61</td><td>0.0002</td><td>0.2</td><td>4.14</td><td>18466.1</td><td>5.78</td><td>HUMAN</td><td>H3BSU3</td><td>Pyruvate kinase (Fragment)</td></tr>
<tr><td>2007</td><td>dirMS_041712_NAR_20mM_minus.12520.12520.2</td><td>2</td><td>16.25</td><td>0</td><td>4.31E+05</td><td>M185m</td><td>(K)LAVNm</td><td>(K)L/A/V/N/m/V/P F P/R(L)</td><td>m:Oxidized methionine</td><td>172.88</td><td>580.3173</td><td>1143.634</td><td>15.993</td><td>-1.6</td><td>9.75</td><td>42230.8</td><td>4.77</td><td>HUMAN</td><td>A6NKZ8</td><td>Putative tubulin beta chain-like protein ENSP00000290377</td></tr>
<tr><td>2008</td><td>dirMS_041712_NAR_30mM_minus2.14121.14121.2</td><td>2</td><td>16.25</td><td>0</td><td>3.83E+05</td><td></td><td>(R)NLVDNI</td><td>(R)N L|V|D N/I/T/G Q/R(L)</td><td></td><td>129.95</td><td>565.3015</td><td>1129.596</td><td>-0.0003</td><td>-0.3</td><td>5.84</td><td>533778</td><td>5.74</td><td>HUMAN</td><td>Q15149</td><td>Plectin</td></tr>
<tr><td>2009</td><td>dirMS_041712_NAR_30mM_plus2.20271.20766.2</td><td>2</td><td>16.25</td><td>0</td><td>1.40E+06</td><td></td><td>(R)ILVATNL</td><td>(R)I/L|V|A/T/N/L F/G R(G)</td><td></td><td>162.17</td><td>552.331</td><td>1103.657</td><td>-0.0024</td><td>-2.2</td><td>9.75</td><td>40918.8</td><td>9.33</td><td>HUMAN</td><td>B4DP52</td><td>HCG2005638, isoform CRA_c</td></tr>
<tr><td>2010</td><td>dirMS_041712_NAR_50mM_plus2.14422.14422.3</td><td>3</td><td>16.25</td><td>1</td><td>9.68E+05</td><td></td><td>(R)LAIKLPD</td><td>(R)L/A/I/K\L/P D D Q I P K(T)</td><td></td><td>144.23</td><td>450.9373</td><td>1350.799</td><td>-0.0018</td><td>-1.3</td><td>5.96</td><td>26413</td><td>9.18</td><td>HUMAN</td><td>B1APP6</td><td>6-phosphofructokinase type C (Fragment)</td></tr>
<tr><td>2011</td><td>dirMS_041712_NAR_60mM_plus.18372.18372.3</td><td>3</td><td>16.25</td><td>1</td><td>5.97E+05</td><td></td><td>(R)IRIDSLS</td><td>(R)I R I D S L\S A|Q\L|S Q L/Q/K(Q)</td><td></td><td>153.73</td><td>567.3254</td><td>1699.97</td><td>-0.0085</td><td>-5</td><td>8.75</td><td>63136.9</td><td>5.83</td><td>HUMAN</td><td>D6RAQ3</td><td>Prelamin-A/C</td></tr>
<tr><td>2012</td><td>dirMS_041712_NAR_30mM_minus2.14401.14851.2</td><td>2</td><td>16.24</td><td>0</td><td>4.42E+06</td><td></td><td>(R)NLLSVA</td><td>(R)N/L|L|S/V/A/Y|K(N)</td><td></td><td>133.37</td><td>454.2604</td><td>907.525</td><td>-0.0112</td><td>-12.4</td><td>8.59</td><td>18789.1</td><td>6.77</td><td>HUMAN</td><td>A2IDB2</td><td>14-3-3 protein eta (Fragment)</td></tr>
<tr><td>2013</td><td>dirMS_041712_NAR_50mM_minus2.15535.15779.3</td><td>3</td><td>16.24</td><td>1</td><td>4.93E+05</td><td></td><td>(K)QGGLGF</td><td>(K)Q/G/G L/G/P M N\I/P/L/V/S D/P K R(T)</td><td></td><td>157.5</td><td>593.6566</td><td>1778.958</td><td>-0.0029</td><td>-1.6</td><td>8.75</td><td>22338.2</td><td>8.65</td><td>HUMAN</td><td>Q06830</td><td>Peroxiredoxin-1</td></tr>
<tr><td>2014</td><td>dirMS_041712_NAR_60mM_minus.9834.10013.3</td><td>3</td><td>16.24</td><td>0</td><td>1.71E+06</td><td></td><td>(R)ISNRPAF</td><td>(R)I/S/N R P A\F\M|P/S/E/G K(M)</td><td></td><td>115.08</td><td>478.5785</td><td>1433.721</td><td>0.0004</td><td>0.3</td><td>8.75</td><td>104488.6</td><td>5.36</td><td>HUMAN</td><td>B2RCS5</td><td>Actinin, alpha 2, isoform CRA_b</td></tr>
<tr><td>2015</td><td>dirMS_041712_NAR_120mM_plus.17377.17678.4</td><td>4</td><td>16.24</td><td>1</td><td>1.48E+05</td><td></td><td>(R)GFGHIG</td><td>(R)G F G H I G I|A\V|P/D/V/Y/S A C K R(F)</td><td>C:Carbamidomethylation</td><td>146.95</td><td>487.5024</td><td>1946.991</td><td>-0.0028</td><td>-1.4</td><td>8.6</td><td>21005.6</td><td>5.12</td><td>HUMAN</td><td>Q04760</td><td>Lactoylglutathione lyase</td></tr>
<tr><td>2016</td><td>dirMS_041712_NAR_20mM_plus.16439.16439.2</td><td>2</td><td>16.24</td><td>0</td><td>1.40E+05</td><td></td><td>(R)QAQEYE</td><td>(R)Q A/Q|E Y/E/A L/L/N I/K(V)</td><td></td><td>184.52</td><td>710.3754</td><td>1419.748</td><td>-0.0043</td><td>-3</td><td>4.53</td><td>43773.3</td><td>5.26</td><td>HUMAN</td><td>F8VZY9</td><td>Keratin, type I cytoskeletal 18</td></tr>
<tr><td>2017</td><td>dirMS_041712_NAR_30mM_plus2.22606.22606.2</td><td>2</td><td>16.24</td><td>0</td><td>1.01E+06</td><td></td><td>(R)FLTWILN</td><td>(R)F L|T/W I L/N R(Y)</td><td></td><td>175.88</td><td>531.8078</td><td>1062.61</td><td>-0.0012</td><td>-1.1</td><td>9.75</td><td>35548.6</td><td>4.91</td><td>HUMAN</td><td>B4DN60</td><td>Asparagine--tRNA ligase, cytoplasmic</td></tr>
<tr><td>2018</td><td>dirMS_041712_NAR_60mM_plus.5622.5622.3</td><td>3</td><td>16.24</td><td>1</td><td>1.91E+05</td><td></td><td>(R)SGGGYG</td><td>(R)S G G G\Y/G/G D R S S G/G G/Y|S G D/R(S)</td><td></td><td>88.07</td><td>583.5777</td><td>1748.722</td><td>-0.0033</td><td>-1.9</td><td>5.68</td><td>21563.9</td><td>10.01</td><td>HUMAN</td><td>K7EKB2</td><td>TATA-binding protein-associated factor 2N (Fragment)</td></tr>
<tr><td>2019</td><td>dirMS_041712_NAR_60mM_plus.8339.8374.3</td><td>3</td><td>16.24</td><td>0</td><td>2.22E+06</td><td></td><td>(K)HTGPNS</td><td>(K)H T G\P N\S P/D|T|A/N/D/G F/V/R(L)</td><td></td><td>102.07</td><td>562.2601</td><td>1684.767</td><td>-0.0016</td><td>-1</td><td>5.21</td><td>18511.8</td><td>5.62</td><td>HUMAN</td><td>D6R9T0</td><td>Heterogeneous nuclear ribonucleoprotein H (Fragment)</td></tr>
<tr><td>2020</td><td>dirMS_041712_NAR_30mM_minus2.12971.13439.2</td><td>2</td><td>16.23</td><td>0</td><td>5.54E+06</td><td></td><td>(K)FASFIDK</td><td>(K)F/A|S|F/I/D|K(V)</td><td></td><td>125.32</td><td>414.2189</td><td>827.43</td><td>0.0007</td><td>0.9</td><td>5.84</td><td>54364</td><td>5.73</td><td>HUMAN</td><td>F5H8K9</td><td>Keratin, type II cytoskeletal 4</td></tr>
<tr><td>2021</td><td>dirMS_041712_NAR_30mM_minus2.15879.15879.2</td><td>2</td><td>16.23</td><td>0</td><td>3.79E+05</td><td></td><td>(R)FGLNVS</td><td>(R)F/G\L|N|V/S/S I S R(K)</td><td></td><td>139.97</td><td>540.2895</td><td>1079.584</td><td>-0.0127</td><td>-11.7</td><td>9.75</td><td>24161.9</td><td>5.27</td><td>HUMAN</td><td>F8VZQ9</td><td>SAP domain-containing ribonucleoprotein</td></tr>
<tr><td>2022</td><td>dirMS_041712_NAR_30mM_plus2.17533.17533.3</td><td>3</td><td>16.23</td><td>0</td><td>8.69E+05</td><td></td><td>(R)ASLEAAI</td><td>(R)A S L E A\A|I/A/D/A/E/Q R(G)</td><td></td><td>144.75</td><td>448.8963</td><td>1344.675</td><td>-0.001</td><td>-0.8</td><td>4.14</td><td>56607.4</td><td>5.37</td><td>HUMAN</td><td>F8VXB4</td><td>Keratin, type II cytoskeletal 8</td></tr>
<tr><td>2023</td><td>dirMS_041712_NAR_30mM_plus2.6102.6102.2</td><td>2</td><td>16.23</td><td>0</td><td>7.31E+04</td><td></td><td>(K)SCCSCC</td><td>(K)S C|C/S\C|C/P V/G\C A K(C)</td><td>C:Carbamidomethylation</td><td>86.22</td><td>723.2586</td><td>1445.51</td><td>0.0001</td><td>0</td><td>8.47</td><td>7183.2</td><td>9.78</td><td>HUMAN</td><td>P02795</td><td>Metallothionein-2</td></tr>
<tr><td>2024</td><td>dirMS_041712_NAR_30mM_plus2.6342.6342.2</td><td>2</td><td>16.23</td><td>0</td><td>1.43E+05</td><td></td><td>(R)FGGSGS</td><td>(R)F/G|G/S/G S Q/V/D|S A R(M)</td><td></td><td>87.52</td><td>584.2731</td><td>1167.539</td><td>0</td><td>0</td><td>5.84</td><td>86346.5</td><td>5.05</td><td>HUMAN</td><td>E7EW34</td><td>26S proteasome non-ATPase regulatory subunit 2</td></tr>
<tr><td>2025</td><td>dirMS_041712_NAR_50mM_plus2.15390.15441.3</td><td>3</td><td>16.23</td><td>1</td><td>4.11E+05</td><td></td><td>(R)LAAELNI</td><td>(R)L/A|A/E/L N/K F/M L E/K(V)</td><td></td><td>149.03</td><td>469.5948</td><td>1406.771</td><td>-0.0014</td><td>-1</td><td>6.14</td><td>80685.1</td><td>7.31</td><td>HUMAN</td><td>P05165</td><td>Propionyl-CoA carboxylase alpha chain, mitochondrial</td></tr>
<tr><td>2026</td><td>dirMS_041712_NAR_50mM_plus2.6754.6754.3</td><td>3</td><td>16.23</td><td>1</td><td>1.72E+05</td><td></td><td>(R)EGVLTG</td><td>(R)E G\V/L/T/G S/P E Q K E E A/A K(A)</td><td></td><td>100.87</td><td>558.2837</td><td>1672.839</td><td>-0.0023</td><td>-1.4</td><td>4.49</td><td>295149.3</td><td>7.4</td><td>HUMAN</td><td>Q92616</td><td>Translational activator GCN1</td></tr>
<tr><td>2027</td><td>dirMS_041712_NAR_20mM_minus.10619.10619.2</td><td>2</td><td>16.22</td><td>0</td><td>2.00E+05</td><td></td><td>(R)TTPSYVA</td><td>(R)T T|P S Y/V|A/F T D/T E/R(L)</td><td></td><td>154.63</td><td>744.3547</td><td>1487.701</td><td>0.0009</td><td>0.6</td><td>4.37</td><td>58502.6</td><td>6.09</td><td>HUMAN</td><td>B4DXY3</td><td>Heat shock 70 kDa protein 1-like</td></tr>
<tr><td>2028</td><td>dirMS_041712_NAR_40mM_minus2.13511.13511.3</td><td>3</td><td>16.22</td><td>0</td><td>3.00E+04</td><td></td><td>(K)YVDTPF</td><td>(K)Y V|D T/P F G K/P S D/A L/I/L G K(I)</td><td></td><td>163.33</td><td>607.6637</td><td>1820.979</td><td>-0.0027</td><td>-1.5</td><td>5.96</td><td>33699.1</td><td>7.18</td><td>HUMAN</td><td>B4DUC8</td><td>S-methyl-5'-thioadenosine phosphorylase</td></tr>
<tr><td>2029</td><td>dirMS_041712_NAR_50mM_minus2.16098.16303.3</td><td>3</td><td>16.22</td><td>1</td><td>4.74E+05</td><td></td><td>(K)LFTAESL</td><td>(K)L/F|T/A/E S L I/G/L K N/P E/K(S)</td><td></td><td>160.67</td><td>553.9759</td><td>1659.932</td><td>-0.0185</td><td>-11.1</td><td>6.14</td><td>62195.8</td><td>5.63</td><td>HUMAN</td><td>B0YIW6</td><td>Archain 1, isoform CRA_a</td></tr>
<tr><td>2030</td><td>dirMS_041712_NAR_50mM_minus2.7668.7668.3</td><td>3</td><td>16.22</td><td>0</td><td>5.25E+04</td><td>M151m</td><td>(R)AYADSY</td><td>(R)A/Y|A/D/S Y/Y Y/E D/G G m K P R(V)</td><td>m:Oxidized methionine</td><td>110.73</td><td>634.6139</td><td>1885.806</td><td>16.021</td><td>13.7</td><td>4.56</td><td>35935.5</td><td>6.2</td><td>HUMAN</td><td>Q8IVM0</td><td>Coiled-coil domain-containing protein 50</td></tr>
<tr><td>2031</td><td>dirMS_041712_NAR_50mM_minus2.9323.9344.3</td><td>3</td><td>16.22</td><td>0</td><td>1.54E+05</td><td></td><td>(R)TPVIDAI</td><td>(R)T/P|V|I/D\A D K|P/V/S S Q L/R(V)</td><td></td><td>121.28</td><td>542.6354</td><td>1625.886</td><td>0.0059</td><td>3.7</td><td>5.62</td><td>535136.6</td><td>6.01</td><td>HUMAN</td><td>Q14204</td><td>Cytoplasmic dynein 1 heavy chain 1</td></tr>
<tr><td>2032</td><td>dirMS_041712_NAR_30mM_minus2.7659.7718.2</td><td>2</td><td>16.21</td><td>0</td><td>2.91E+06</td><td></td><td>(K)TEISEMI</td><td>(K)T/E|I|S/E/M/N/R(N)</td><td></td><td>96.23</td><td>490.2292</td><td>979.451</td><td>-0.0002</td><td>-0.2</td><td>4.53</td><td>37296.3</td><td>5.3</td><td>HUMAN</td><td>F8VU64</td><td>Keratin, type II cytoskeletal 8</td></tr>
<tr><td>2033</td><td>dirMS_041712_NAR_50mM_minus2.18041.18041.3</td><td>3</td><td>16.21</td><td>1</td><td>1.56E+04</td><td></td><td>(K)TFVNITP</td><td>(K)T F|V/N I/T/P A E V G V L\V/G\K D R(S)</td><td></td><td>171.78</td><td>639.0247</td><td>1915.065</td><td>-0.0052</td><td>-2.7</td><td>5.74</td><td>17630.6</td><td>11.62</td><td>HUMAN</td><td>I3L3D5</td><td>Profilin-1 (Fragment)</td></tr>
<tr><td>2034</td><td>dirMS_041712_NAR_20mM_plus.10797.10797.2</td><td>2</td><td>16.21</td><td>0</td><td>1.18E+05</td><td></td><td>(R)AGTGVD</td><td>(R)A G T G V/D/N/V|D|L E A/A/T R(K)</td><td></td><td>146.88</td><td>744.8731</td><td>1488.729</td><td>0.01</td><td>6.7</td><td>4.03</td><td>57391.2</td><td>6.29</td><td>HUMAN</td><td>O43175</td><td>D-3-phosphoglycerate dehydrogenase</td></tr>
<tr><td>2035</td><td>dirMS_041712_NAR_30mM_plus2.11513.11557.2</td><td>2</td><td>16.21</td><td>0</td><td>5.15E+05</td><td></td><td>(R)SGEGEV</td><td>(R)S G|E|G|E|V S|G L/M/R(K)</td><td></td><td>114.07</td><td>561.2662</td><td>1121.526</td><td>-0.0004</td><td>-0.4</td><td>4.53</td><td>90316.8</td><td>5.52</td><td>HUMAN</td><td>Q13263</td><td>Transcription intermediary factor 1-beta</td></tr>
<tr><td>2036</td><td>dirMS_041712_NAR_30mM_plus2.16012.16256.2</td><td>2</td><td>16.21</td><td>0</td><td>7.42E+05</td><td></td><td>(R)GALQNI</td><td>(R)G A/L|Q|N|I|I\P A/S T G\A/A K(A)</td><td></td><td>137.53</td><td>706.3979</td><td>1411.79</td><td>-0.0018</td><td>-1.3</td><td>8.75</td><td>31718.5</td><td>7.17</td><td>HUMAN</td><td>E7EUT4</td><td>Glyceraldehyde-3-phosphate dehydrogenase</td></tr>
<tr><td>2037</td><td>dirMS_041712_NAR_30mM_plus2.17772.17772.2</td><td>2</td><td>16.21</td><td>0</td><td>4.92E+05</td><td></td><td>(K)LLGELLQ</td><td>(K)L/L|G\E/L/L/Q D N/A K(L)</td><td></td><td>145.8</td><td>607.3423</td><td>1213.679</td><td>-0.0014</td><td>-1.1</td><td>4.37</td><td>35556.4</td><td>9.3</td><td>HUMAN</td><td>F2Z393</td><td>Transaldolase</td></tr>
<tr><td>2038</td><td>dirMS_041712_NAR_30mM_plus2.20438.20438.2</td><td>2</td><td>16.21</td><td>0</td><td>3.06E+05</td><td></td><td>(R)YISPDQL</td><td>(R)Y I|S|P D Q L A D L Y K(S)</td><td></td><td>161.52</td><td>713.3669</td><td>1425.726</td><td>0.0005</td><td>0.4</td><td>4.21</td><td>47510.4</td><td>7.04</td><td>HUMAN</td><td>P06733</td><td>Alpha-enolase</td></tr>
<tr><td>2039</td><td>dirMS_041712_NAR_30mM_plus2.9010.9553.2</td><td>2</td><td>16.21</td><td>0</td><td>3.27E+06</td><td></td><td>(R)MGYMD</td><td>(R)M/G|Y|M/D|P/R(E)</td><td></td><td>101.28</td><td>435.186</td><td>869.364</td><td>0.0003</td><td>0.3</td><td>5.59</td><td>26419.2</td><td>5.1</td><td>HUMAN</td><td>H0Y9K7</td><td>Splicing factor, proline- and glutamine-rich (Fragment)</td></tr>
<tr><td>2040</td><td>dirMS_041712_NAR_50mM_plus2.15077.15618.3</td><td>3</td><td>16.21</td><td>1</td><td>9.32E+06</td><td></td><td>(R)IINEPTA</td><td>(R)I/I|N\E|P T A A/A/I/A/Y G L D K K(V)</td><td></td><td>149.67</td><td>596.6673</td><td>1787.99</td><td>-0.0028</td><td>-1.6</td><td>6.07</td><td>17973</td><td>5.16</td><td>HUMAN</td><td>E9PI65</td><td>Heat shock cognate 71 kDa protein (Fragment)</td></tr>
<tr><td>2041</td><td>dirMS_041712_NAR_60mM_plus.16344.16344.3</td><td>3</td><td>16.21</td><td>1</td><td>1.84E+05</td><td></td><td>(K)KAPDFV</td><td>(K)K A P D\F V|F/Y/A/P/R(L)</td><td></td><td>141.8</td><td>437.5685</td><td>1310.689</td><td>0.0018</td><td>1.3</td><td>8.59</td><td>71218.8</td><td>6.3</td><td>HUMAN</td><td>A7YIJ8</td><td>Radixin</td></tr>
<tr><td>2042</td><td>dirMS_041712_NAR_60mM_plus.9825.9825.3</td><td>3</td><td>16.21</td><td>1</td><td>1.31E+06</td><td></td><td>(K)LAELKQE</td><td>(K)L/A/E/L/K/Q/E C/L A R(G)</td><td>C:Carbamidomethylation</td><td>109.57</td><td>444.2368</td><td>1330.715</td><td>-0.0189</td><td>-14.2</td><td>6.14</td><td>6106</td><td>9.22</td><td>HUMAN</td><td>F8VS12</td><td>SAP domain-containing ribonucleoprotein</td></tr>
<tr><td>2043</td><td>dirMS_041712_NAR_30mM_minus2.10473.10473.2</td><td>2</td><td>16.2</td><td>0</td><td>1.68E+06</td><td></td><td>(K)TAVCDIF</td><td>(K)T A|V|C/D/I/P|P R(G)</td><td>C:Carbamidomethylation</td><td>110.87</td><td>514.7641</td><td>1028.519</td><td>0.0016</td><td>1.5</td><td>5.5</td><td>50199.4</td><td>4.75</td><td>HUMAN</td><td>A6NNZ2</td><td>Tubulin beta-8 chain-like protein LOC260334</td></tr>
<tr><td>2044</td><td>dirMS_041712_NAR_50mM_minus2.12725.12902.3</td><td>3</td><td>16.2</td><td>1</td><td>3.91E+05</td><td></td><td>(R)LSEDYG</td><td>(R)L/S|E/D/Y/G V/L K/T D/E/G I A Y R(G)</td><td></td><td>141.52</td><td>643.6561</td><td>1928.96</td><td>-0.0063</td><td>-3.2</td><td>4.32</td><td>22062.7</td><td>5.66</td><td>HUMAN</td><td>P32119</td><td>Peroxiredoxin-2</td></tr>
<tr><td>2045</td><td>dirMS_041712_NAR_120mM_plus.7940.8118.4</td><td>4</td><td>16.2</td><td>1</td><td>6.41E+05</td><td></td><td>(K)VLATVTI</td><td>(K)V/L\A/T/V/T K/P V G G D/K/N G G T R(V)</td><td></td><td>95.57</td><td>443.251</td><td>1769.987</td><td>-0.0047</td><td>-2.6</td><td>9.99</td><td>12738</td><td>10.72</td><td>HUMAN</td><td>F8VWR1</td><td>60S ribosomal protein L6 (Fragment)</td></tr>
<tr><td>2046</td><td>dirMS_041712_NAR_20mM_plus.10796.10796.2</td><td>2</td><td>16.2</td><td>0</td><td>3.14E+04</td><td></td><td>(K)IGGIGTV</td><td>(K)I/G/G\I/G/T/V/P V G R(V)</td><td></td><td>146.8</td><td>513.3057</td><td>1025.61</td><td>-0.0061</td><td>-5.9</td><td>9.75</td><td>50482.3</td><td>9.31</td><td>HUMAN</td><td>P68104</td><td>Elongation factor 1-alpha 1</td></tr>
<tr><td>2047</td><td>dirMS_041712_NAR_120mM_minus.5124.5147.3</td><td>3</td><td>16.19</td><td>0</td><td>1.86E+05</td><td></td><td>(R)YINTEH</td><td>(R)Y/I/N/T/E/H/G G S Q A/R(F)</td><td></td><td>80.6</td><td>444.8813</td><td>1332.629</td><td>0.0002</td><td>0.2</td><td>6.75</td><td>87447.6</td><td>6.44</td><td>HUMAN</td><td>Q15437</td><td>Protein transport protein Sec23B</td></tr>
<tr><td>2048</td><td>dirMS_041712_NAR_20mM_minus.14676.14676.2</td><td>2</td><td>16.19</td><td>0</td><td>2.10E+05</td><td></td><td>(K)LAVEALS</td><td>(K)L A/V|E/A|L/S/S L|D\G D L A/G R(Y)</td><td></td><td>201.28</td><td>793.922</td><td>1586.838</td><td>-0.0017</td><td>-1.1</td><td>4.03</td><td>24286.7</td><td>4.89</td><td>HUMAN</td><td>G3V4N7</td><td>Creatine kinase B-type (Fragment)</td></tr>
<tr><td>2049</td><td>dirMS_041712_NAR_30mM_minus2.10933.10933.2</td><td>2</td><td>16.19</td><td>0</td><td>5.26E+05</td><td></td><td>(K)VAGQD</td><td>(K)V/A|G/Q/D|G S/V/V Q|F K(I)</td><td></td><td>113.68</td><td>617.824</td><td>1234.643</td><td>-0.0019</td><td>-1.5</td><td>5.81</td><td>17127.4</td><td>10.05</td><td>HUMAN</td><td>A8MU27</td><td>Small ubiquitin-related modifier 3</td></tr>
<tr><td>2050</td><td>dirMS_041712_NAR_30mM_minus2.18340.18467.3</td><td>3</td><td>16.19</td><td>0</td><td>5.33E+05</td><td></td><td>(R)QAQEYE</td><td>(R)Q A Q\E\Y|E|A/L/L/N/I K(V)</td><td></td><td>154.7</td><td>473.9196</td><td>1419.748</td><td>-0.0036</td><td>-2.5</td><td>4.53</td><td>43773.3</td><td>5.26</td><td>HUMAN</td><td>F8VZY9</td><td>Keratin, type I cytoskeletal 18</td></tr>
<tr><td>2051</td><td>dirMS_041712_NAR_50mM_minus2.7914.7930.3</td><td>3</td><td>16.19</td><td>1</td><td>3.13E+05</td><td></td><td>(R)LQAEIEG</td><td>(R)L/Q|A/E/I/E/G L/K/G Q R(A)</td><td></td><td>112.3</td><td>447.921</td><td>1341.748</td><td>0</td><td>0</td><td>6.14</td><td>56607.4</td><td>5.37</td><td>HUMAN</td><td>F8VXB4</td><td>Keratin, type II cytoskeletal 8</td></tr>
<tr><td>2052</td><td>dirMS_041712_NAR_20mM_plus.16674.16674.2</td><td>2</td><td>16.19</td><td>0</td><td>5.10E+05</td><td></td><td>(K)LGGSAV</td><td>(K)L G G S A/V|I/S L/E/G\K|P L(-)</td><td></td><td>185.82</td><td>670.8917</td><td>1340.778</td><td>-0.0023</td><td>-1.7</td><td>6</td><td>22956</td><td>8.8</td><td>HUMAN</td><td>E9PK25</td><td>Cofilin-1</td></tr>
<tr><td>2053</td><td>dirMS_041712_NAR_30mM_plus2.19892.20176.2</td><td>2</td><td>16.19</td><td>0</td><td>8.41E+05</td><td></td><td>(K)MSIYQA</td><td>(K)M/S|I/Y/Q/A/M/W/K(G)</td><td></td><td>158.83</td><td>579.2783</td><td>1157.548</td><td>0.0011</td><td>1</td><td>8.34</td><td>266161</td><td>5.68</td><td>HUMAN</td><td>E9PPU0</td><td>Epiplakin</td></tr>
<tr><td>2054</td><td>dirMS_041712_NAR_60mM_plus.7525.7730.3</td><td>3</td><td>16.19</td><td>1</td><td>1.56E+06</td><td></td><td>(K)IVADKD</td><td>(K)I/V|A D/K/D/Y/S V/T/A N S K(I)</td><td></td><td>97.95</td><td>504.2625</td><td>1510.775</td><td>-0.0018</td><td>-1.2</td><td>5.96</td><td>25445.8</td><td>5.52</td><td>HUMAN</td><td>A8MW50</td><td>L-lactate dehydrogenase (Fragment)</td></tr>
<tr><td>2055</td><td>dirMS_041712_NAR_20mM_minus.12232.12473.3</td><td>3</td><td>16.18</td><td>0</td><td>8.33E+05</td><td>M73m</td><td>(R)AVLVDLI</td><td>(R)A V L\V D\L\E/P G T|m/D S V R(S)</td><td>m:Oxidized methionine</td><td>172.57</td><td>539.9423</td><td>1601.82</td><td>15.992</td><td>-1.8</td><td>4.03</td><td>13112.4</td><td>5.23</td><td>HUMAN</td><td>F8W813</td><td>Tubulin beta-8 chain</td></tr>
<tr><td>2056</td><td>dirMS_041712_NAR_30mM_minus2.13306.13558.2</td><td>2</td><td>16.18</td><td>0</td><td>9.82E+05</td><td></td><td>(K)DVNAAL</td><td>(K)D V|N/A|A/I/A/T/I/K(T)</td><td></td><td>126.43</td><td>508.2928</td><td>1015.578</td><td>0.0001</td><td>0.1</td><td>5.84</td><td>58642</td><td>4.95</td><td>HUMAN</td><td>F5H5D3</td><td>Tubulin alpha-1C chain</td></tr>
<tr><td>2057</td><td>dirMS_041712_NAR_30mM_minus2.6842.6898.2</td><td>2</td><td>16.18</td><td>0</td><td>3.65E+05</td><td></td><td>(K)AMEAV</td><td>(K)A M|E|A|V/A|A|Q/G K(A)</td><td></td><td>91.98</td><td>488.2495</td><td>975.493</td><td>-0.0011</td><td>-1.1</td><td>6.05</td><td>28936.8</td><td>9.22</td><td>HUMAN</td><td>P15259</td><td>Phosphoglycerate mutase 2</td></tr>
<tr><td>2058</td><td>dirMS_041712_NAR_50mM_plus2.19281.19281.3</td><td>3</td><td>16.18</td><td>0</td><td>4.12E+05</td><td></td><td>(R)GFGFITF</td><td>(R)G F G\F/I/T/F T/N/P E H A/S V/A M R(A)</td><td></td><td>169.22</td><td>661.3258</td><td>1981.959</td><td>0.004</td><td>2</td><td>6.75</td><td>17170.1</td><td>8.86</td><td>HUMAN</td><td>P98179</td><td>Putative RNA-binding protein 3</td></tr>
<tr><td>2059</td><td>dirMS_041712_NAR_60mM_plus.7880.7880.3</td><td>3</td><td>16.18</td><td>1</td><td>5.18E+05</td><td></td><td>(K)FAAKGE</td><td>(K)F/A|A/K/G E G Q|L/G|P/A E R(A)</td><td></td><td>99.2</td><td>477.5865</td><td>1430.739</td><td>0.0063</td><td>4.4</td><td>6.14</td><td>96306.4</td><td>6.41</td><td>HUMAN</td><td>P13639</td><td>Elongation factor 2</td></tr>
<tr><td>2060</td><td>dirMS_041712_NAR_50mM_minus2.10513.10844.3</td><td>3</td><td>16.17</td><td>1</td><td>4.28E+05</td><td></td><td>(R)DVVICPI</td><td>(R)D V|V|I/C/P D/A S L E D A K K(E)</td><td>C:Carbamidomethylation</td><td>128.55</td><td>553.9474</td><td>1659.826</td><td>0.0018</td><td>1.1</td><td>4.23</td><td>18080.7</td><td>7.92</td><td>HUMAN</td><td>K7ELW0</td><td>Protein DJ-1</td></tr>
<tr><td>2061</td><td>dirMS_041712_NAR_120mM_plus.18692.18815.3</td><td>3</td><td>16.17</td><td>1</td><td>6.76E+05</td><td></td><td>(R)ARFEEL</td><td>(R)A R F E E\L|C/S/D/L/F R(S)</td><td>C:Carbamidomethylation</td><td>151.33</td><td>514.9165</td><td>1542.737</td><td>-0.002</td><td>-1.3</td><td>4.68</td><td>25598.3</td><td>6.45</td><td>HUMAN</td><td>E7EP11</td><td>Heat shock 70 kDa protein 1A/1B</td></tr>
<tr><td>2062</td><td>dirMS_041712_NAR_120mM_plus.6761.6806.3</td><td>3</td><td>16.17</td><td>1</td><td>6.52E+05</td><td></td><td>(R)NKQTYS</td><td>(R)N K Q T\Y|S|T/E/P/N N L K(A)</td><td></td><td>88.83</td><td>512.9261</td><td>1536.765</td><td>-0.0015</td><td>-1</td><td>8.5</td><td>7945.1</td><td>11.03</td><td>HUMAN</td><td>C9JB50</td><td>60S ribosomal protein L28</td></tr>
<tr><td>2063</td><td>dirMS_041712_NAR_30mM_plus2.11626.11923.2</td><td>2</td><td>16.17</td><td>0</td><td>2.78E+06</td><td></td><td>(K)LSDGVA</td><td>(K)L/S|D|G V A/V|L/K(V)</td><td></td><td>115.52</td><td>451.2712</td><td>901.535</td><td>-0.0002</td><td>-0.2</td><td>5.84</td><td>61224.7</td><td>5.7</td><td>HUMAN</td><td>P10809</td><td>60 kDa heat shock protein, mitochondrial</td></tr>
<tr><td>2064</td><td>dirMS_041712_NAR_30mM_plus2.18758.18942.2</td><td>2</td><td>16.17</td><td>0</td><td>1.19E+06</td><td></td><td>(R)LSNIFVI</td><td>(R)L/S|N|I|F/V|I/G/K(G)</td><td></td><td>151.42</td><td>495.8019</td><td>990.598</td><td>-0.0017</td><td>-1.7</td><td>8.75</td><td>29825.4</td><td>10.37</td><td>HUMAN</td><td>P62701</td><td>40S ribosomal protein S4, X isoform</td></tr>
<tr><td>2065</td><td>dirMS_041712_NAR_30mM_minus2.11002.11002.2</td><td>2</td><td>16.16</td><td>0</td><td>5.91E+05</td><td></td><td>(K)VAGQD</td><td>(K)V A|G|Q/D|G S/V V Q|F K(I)</td><td></td><td>113.82</td><td>617.824</td><td>1234.643</td><td>-0.0019</td><td>-1.5</td><td>5.81</td><td>17127.4</td><td>10.05</td><td>HUMAN</td><td>A8MU27</td><td>Small ubiquitin-related modifier 3</td></tr>
<tr><td>2066</td><td>dirMS_041712_NAR_30mM_plus2.14608.14910.2</td><td>2</td><td>16.16</td><td>0</td><td>2.26E+06</td><td></td><td>(K)DLAGSII</td><td>(K)D/L|A|G/S I/I/G/K(G)</td><td></td><td>130.5</td><td>437.2557</td><td>873.504</td><td>0.0001</td><td>0.1</td><td>5.84</td><td>51260.6</td><td>5.39</td><td>HUMAN</td><td>P61978</td><td>Heterogeneous nuclear ribonucleoprotein K</td></tr>
</table>

<table>
<tr><td>2067</td><td>dirMS_041712_NAR_60mM_plus.14390.14468.3</td><td>3</td><td>16.16</td><td>1</td><td>9.50E+05</td><td></td><td>(K)TKEVYEL</td><td>(K)T K/E V Y\E|L|L/D/S/P G/K(V)</td><td></td><td>131.9</td><td>493.5959</td><td>1478.774</td><td>-0.0006</td><td>-0.4</td><td>4.68</td><td>46335.4</td><td>6.29</td><td>HUMAN</td><td>E9PBS1</td><td>Phosphoribosylaminoimidazole carboxylase (Fragment)</td></tr>
<tr><td>2068</td><td>dirMS_041712_NAR_60mM_plus.16256.16564.3</td><td>3</td><td>16.16</td><td>1</td><td>3.69E+05</td><td></td><td>(-)MKLNISF</td><td>(-)M K L N I S\F|P/A/T/G C Q K(L)</td><td>C:Carbamidomethylation</td><td>142.85</td><td>532.2733</td><td>1594.808</td><td>-0.0026</td><td>-1.7</td><td>10</td><td>10471.9</td><td>5.4</td><td>HUMAN</td><td>A2A3R7</td><td>40S ribosomal protein S6</td></tr>
<tr><td>2069</td><td>dirMS_041712_NAR_60mM_plus.21545.21709.3</td><td>3</td><td>16.16</td><td>0</td><td>9.30E+06</td><td></td><td>(R)LHFFMP</td><td>(R)L H\F\F\M|P/G\F|A|P\L/T S R(G)</td><td></td><td>180.4</td><td>540.9492</td><td>1620.835</td><td>-0.0025</td><td>-1.5</td><td>9.75</td><td>42230.8</td><td>4.77</td><td>HUMAN</td><td>A6NKZ8</td><td>Putative tubulin beta chain-like protein ENSP00000290377</td></tr>
<tr><td>2070</td><td>dirMS_041712_NAR_60mM_plus.7177.7408.3</td><td>3</td><td>16.16</td><td>1</td><td>1.57E+07</td><td></td><td>(R)AAPGAE</td><td>(R)A A|P/G A E/F A/P N K/R(R)</td><td></td><td>96.3</td><td>410.2195</td><td>1228.643</td><td>0.0007</td><td>0.5</td><td>8.79</td><td>43979.5</td><td>8.92</td><td>HUMAN</td><td>F5GYZ3</td><td>Non-POU domain-containing octamer-binding protein</td></tr>
<tr><td>2071</td><td>dirMS_041712_NAR_120mM_minus.5379.5379.3</td><td>3</td><td>16.15</td><td>1</td><td>9.04E+05</td><td></td><td>(R)NDDDEE</td><td>(R)N/D|D/D/E/E/E A A\R E R(R)</td><td></td><td>82.93</td><td>483.5345</td><td>1448.588</td><td>0.0005</td><td>0.4</td><td>3.95</td><td>54214.9</td><td>5.66</td><td>HUMAN</td><td>C9J813</td><td>Caldesmon (Fragment)</td></tr>
<tr><td>2072</td><td>dirMS_041712_NAR_30mM_minus2.10523.10566.2</td><td>2</td><td>16.15</td><td>0</td><td>3.17E+05</td><td></td><td>(R)SGSYSYL</td><td>(R)S G|S|Y/S/Y/L/E/E/R(K)</td><td></td><td>111.13</td><td>595.7696</td><td>1190.532</td><td>-0.0005</td><td>-0.4</td><td>4.53</td><td>22046.7</td><td>5.8</td><td>HUMAN</td><td>H0YIM2</td><td>Protein phosphatase 1 regulatory subunit 12A (Fragment)</td></tr>
<tr><td>2073</td><td>dirMS_041712_NAR_50mM_minus2.8555.8756.3</td><td>3</td><td>16.15</td><td>1</td><td>4.24E+05</td><td>M244m</td><td>(K)ILIANTG</td><td>(K)I/L|I/A/N/T/G m D T D/K I/K(I)</td><td>m:Oxidized methionine</td><td>117.38</td><td>516.9426</td><td>1532.835</td><td>15.978</td><td>-10.9</td><td>5.96</td><td>57204.9</td><td>6.01</td><td>HUMAN</td><td>F5GWF6</td><td>T-complex protein 1 subunit beta</td></tr>
<tr><td>2074</td><td>dirMS_041712_NAR_60mM_minus.18628.18755.3</td><td>3</td><td>16.15</td><td>1</td><td>1.31E+05</td><td></td><td>(K)IILDLISE</td><td>(K)I I|L D/L/I/S E/S/P I K G R(A)</td><td></td><td>166.17</td><td>518.6442</td><td>1553.926</td><td>-0.0081</td><td>-5.2</td><td>6.07</td><td>51260.6</td><td>5.39</td><td>HUMAN</td><td>P61978</td><td>Heterogeneous nuclear ribonucleoprotein K</td></tr>
<tr><td>2075</td><td>dirMS_041712_NAR_20mM_plus.13793.13793.2</td><td>2</td><td>16.15</td><td>0</td><td>5.65E+04</td><td>M33m</td><td>(R)LVQSPN</td><td>(R)L V|Q|S|P N S/Y/F m D V/K(C)</td><td>m:Oxidized methionine</td><td>167.07</td><td>772.3772</td><td>1527.751</td><td>15.9959</td><td>0.7</td><td>5.83</td><td>11227.9</td><td>9.22</td><td>HUMAN</td><td>C9JLI6</td><td>40S ribosomal protein S27</td></tr>
<tr><td>2076</td><td>dirMS_041712_NAR_30mM_plus2.11949.12010.2</td><td>2</td><td>16.15</td><td>0</td><td>6.83E+05</td><td></td><td>(K)CGDLVF</td><td>(K)C/G/D|L/V/F|A/K(M)</td><td>C:Carbamidomethylation</td><td>116.12</td><td>455.2298</td><td>909.45</td><td>0.0025</td><td>2.7</td><td>5.84</td><td>26901.9</td><td>4.7</td><td>HUMAN</td><td>P51858</td><td>Hepatoma-derived growth factor</td></tr>
<tr><td>2077</td><td>dirMS_041712_NAR_30mM_plus2.8624.8920.2</td><td>2</td><td>16.15</td><td>0</td><td>4.02E+06</td><td></td><td>(R)ELNITAA</td><td>(R)E L/N/I/T/A/A/K(E)</td><td></td><td>100.15</td><td>430.2487</td><td>859.488</td><td>0.0018</td><td>2.1</td><td>6.1</td><td>21311.5</td><td>10.28</td><td>HUMAN</td><td>B5MCP9</td><td>40S ribosomal protein S7</td></tr>
<tr><td>2078</td><td>dirMS_041712_NAR_20mM_minus.10615.10615.2</td><td>2</td><td>16.14</td><td>0</td><td>2.00E+05</td><td></td><td>(R)TTPSYV</td><td>(R)T T/P S Y/V/A/F T D/T E/R(L)</td><td></td><td>154.63</td><td>744.3547</td><td>1487.701</td><td>0.0009</td><td>0.6</td><td>4.37</td><td>58502.6</td><td>6.09</td><td>HUMAN</td><td>B4DXY3</td><td>Heat shock 70 kDa protein 1-like</td></tr>
<tr><td>2079</td><td>dirMS_041712_NAR_40mM_minus2.14534.14534.3</td><td>3</td><td>16.14</td><td>1</td><td>1.17E+06</td><td>M116m</td><td>(R)ELAILLG</td><td>(R)E L/A I/L/L/G m L D/P A E K D E/K(G)</td><td>m:Oxidized methionine</td><td>171.02</td><td>634.338</td><td>1884.999</td><td>16.0008</td><td>3.1</td><td>4.18</td><td>25148.6</td><td>6</td><td>HUMAN</td><td>P30041</td><td>Peroxiredoxin-6</td></tr>
<tr><td>2080</td><td>dirMS_041712_NAR_20mM_plus.18175.18175.2</td><td>2</td><td>16.14</td><td>0</td><td>1.13E+05</td><td></td><td>(K)LVLPSLIS</td><td>(K)L V|L/P S L/I S S R(I)</td><td></td><td>196.6</td><td>542.8388</td><td>1084.672</td><td>-0.0021</td><td>-2</td><td>9.75</td><td>39066</td><td>6.61</td><td>HUMAN</td><td>A6PVW9</td><td>Selenium binding protein 1 (Fragment)</td></tr>
<tr><td>2081</td><td>dirMS_041712_NAR_30mM_plus2.17864.17868.2</td><td>2</td><td>16.14</td><td>0</td><td>2.68E+05</td><td></td><td>(K)FEDENFI</td><td>(K)F/E|D E/N|F/I/L/K(H)</td><td></td><td>147.02</td><td>577.7904</td><td>1154.573</td><td>0.0007</td><td>0.6</td><td>4.14</td><td>13378.8</td><td>6.27</td><td>HUMAN</td><td>C9J5S7</td><td>Peptidyl-prolyl cis-trans isomerase</td></tr>
<tr><td>2082</td><td>dirMS_041712_NAR_60mM_plus.12668.12668.3</td><td>3</td><td>16.14</td><td>1</td><td>8.35E+05</td><td></td><td>(R)DLDRIA</td><td>(R)D L/D R\I\A/G Q\V/A|A/A N K(K)</td><td></td><td>123.38</td><td>481.263</td><td>1441.776</td><td>-0.0013</td><td>-0.9</td><td>5.96</td><td>16060.2</td><td>10.31</td><td>HUMAN</td><td>P39019</td><td>40S ribosomal protein S19</td></tr>
<tr><td>2083</td><td>dirMS_041712_NAR_30mM_minus2.10563.10588.2</td><td>2</td><td>16.13</td><td>0</td><td>3.30E+05</td><td></td><td>(K)FADLSE</td><td>(K)F/A|D|L|S/E/A/A N R(N)</td><td></td><td>111.48</td><td>547.265</td><td>1093.527</td><td>-0.0045</td><td>-4.2</td><td>4.37</td><td>49709.6</td><td>5.19</td><td>HUMAN</td><td>B0YJC4</td><td>Vimentin</td></tr>
<tr><td>2084</td><td>dirMS_041712_NAR_50mM_minus2.12273.12349.3</td><td>3</td><td>16.13</td><td>0</td><td>1.08E+06</td><td></td><td>(K)LGDVYV</td><td>(K)L/G/D|V/Y V/N/D A/F G T A\H/R(A)</td><td></td><td>138.38</td><td>545.6023</td><td>1634.792</td><td>0.0002</td><td>0.1</td><td>5.21</td><td>41827.5</td><td>7</td><td>HUMAN</td><td>B7Z7A9</td><td>Phosphoglycerate kinase</td></tr>
<tr><td>2085</td><td>dirMS_041712_NAR_60mM_minus.9391.9403.3</td><td>3</td><td>16.13</td><td>1</td><td>1.78E+05</td><td></td><td>(K)IFRDGEE</td><td>(K)I/F/R D G E\E\A|G|A|Y/D/G P R(T)</td><td></td><td>112.25</td><td>551.5934</td><td>1652.766</td><td>-0.0007</td><td>-0.4</td><td>4.32</td><td>55361.8</td><td>6.42</td><td>HUMAN</td><td>G5EA52</td><td>Protein disulfide isomerase family A, member 3, isoform CRA_b</td></tr>
<tr><td>2086</td><td>dirMS_041712_NAR_30mM_plus2.6095.6118.2</td><td>2</td><td>16.13</td><td>0</td><td>3.26E+05</td><td></td><td>(K)ISSPTETI</td><td>(K)I/S|S|P/T E T E R(C)</td><td></td><td>86.23</td><td>510.2497</td><td>1019.5</td><td>-0.0083</td><td>-8.1</td><td>4.53</td><td>11854.4</td><td>6.57</td><td>HUMAN</td><td>P31949</td><td>Protein S100-A11</td></tr>
<tr><td>2087</td><td>dirMS_041712_NAR_40mM_plus2.19128.19128.3</td><td>3</td><td>16.13</td><td>1</td><td>3.14E+05</td><td></td><td>(R)GGPNIIT</td><td>(R)G G/P\N/I|I/T/L A D I/V/K D P V S R(T)</td><td></td><td>178.97</td><td>622.3522</td><td>1865.049</td><td>-0.007</td><td>-3.8</td><td>5.96</td><td>45481.2</td><td>7.85</td><td>HUMAN</td><td>E7EU96</td><td>Casein kinase II subunit alpha</td></tr>
<tr><td>2088</td><td>dirMS_041712_NAR_50mM_plus2.6020.6020.3</td><td>3</td><td>16.13</td><td>0</td><td>1.14E+05</td><td></td><td>(R)AGAAG</td><td>(R)A/G\A|A/G|G/P E E E A E/K P V\K(T)</td><td></td><td>95.73</td><td>513.9259</td><td>1539.765</td><td>-0.0018</td><td>-1.2</td><td>4.49</td><td>18924.5</td><td>6.86</td><td>HUMAN</td><td>Q8WW12</td><td>PEST proteolytic signal-containing nuclear protein</td></tr>
<tr><td>2089</td><td>dirMS_041712_NAR_30mM_minus2.13259.13259.2</td><td>2</td><td>16.12</td><td>0</td><td>3.00E+05</td><td></td><td>(K)STYGWT</td><td>(K)S T|Y/G|W/T A/N M E/R(I)</td><td></td><td>125.7</td><td>658.2918</td><td>1315.574</td><td>0.0028</td><td>2.1</td><td>5.72</td><td>47825.1</td><td>5.07</td><td>HUMAN</td><td>Q58FG1</td><td>Putative heat shock protein HSP 90-alpha A4</td></tr>
<tr><td>2090</td><td>dirMS_041712_NAR_50mM_minus2.14392.14539.3</td><td>3</td><td>16.12</td><td>1</td><td>1.07E+06</td><td></td><td>(K)EAFSLFD</td><td>(K)E A|F|S/L F/D/K/D/G D/G T I T T K(E)</td><td></td><td>149.97</td><td>615.6336</td><td>1844.891</td><td>-0.005</td><td>-2.7</td><td>4.23</td><td>21860.1</td><td>4.45</td><td>HUMAN</td><td>E7EMB3</td><td>Calmodulin</td></tr>
<tr><td>2091</td><td>dirMS_041712_NAR_30mM_plus2.21467.21467.2</td><td>2</td><td>16.12</td><td>0</td><td>3.90E+05</td><td></td><td>(R)VNQAIW</td><td>(R)V N Q A/I/W|L|L/C/T/G A R(E)</td><td>C:Carbamidomethylation</td><td>166.88</td><td>751.3976</td><td>1501.794</td><td>-0.0065</td><td>-4.3</td><td>9.72</td><td>23047.2</td><td>9.95</td><td>HUMAN</td><td>P46782</td><td>40S ribosomal protein S5</td></tr>
<tr><td>2092</td><td>dirMS_041712_NAR_30mM_plus2.7182.7182.2</td><td>2</td><td>16.12</td><td>0</td><td>1.83E+04</td><td></td><td>(R)SGGGG</td><td>(R)S G|G G G/G/G G L/G S G G/S I/R(S)</td><td></td><td>92.03</td><td>616.8023</td><td>1232.598</td><td>-0.0005</td><td>-0.4</td><td>9.47</td><td>62291.5</td><td>5.13</td><td>HUMAN</td><td>P35527</td><td>Keratin, type I cytoskeletal 9</td></tr>
<tr><td>2093</td><td>dirMS_041712_NAR_40mM_plus2.6150.6192.2</td><td>2</td><td>16.12</td><td>0</td><td>2.76E+05</td><td></td><td>(R)MGANSI</td><td>(R)M/G|A|N/S L/E/R(M)</td><td></td><td>95.23</td><td>439.2183</td><td>877.42</td><td>0.0097</td><td>11.1</td><td>5.75</td><td>77799.7</td><td>9.09</td><td>HUMAN</td><td>P52272</td><td>Heterogeneous nuclear ribonucleoprotein M</td></tr>
<tr><td>2094</td><td>dirMS_041712_NAR_40mM_minus2.8617.8630.3</td><td>3</td><td>16.11</td><td>0</td><td>2.84E+05</td><td></td><td>(R)SVIDPVF</td><td>(R)S V\I D/P V/P A/P V G/D/S H V D G A A K(S)</td><td></td><td>132.52</td><td>644.3329</td><td>1930.987</td><td>-0.0027</td><td>-1.4</td><td>4.41</td><td>58326.8</td><td>5.7</td><td>HUMAN</td><td>Q13177</td><td>Serine/threonine-protein kinase PAK 2</td></tr>
<tr><td>2095</td><td>dirMS_041712_NAR_50mM_minus2.8265.8280.3</td><td>3</td><td>16.11</td><td>1</td><td>2.19E+05</td><td></td><td>(K)GILAADE</td><td>(K)G I|L/A/A/D/E/S T/G S I A K/R(L)</td><td></td><td>114.78</td><td>496.9372</td><td>1488.802</td><td>-0.0046</td><td>-3.1</td><td>6.07</td><td>30711.4</td><td>7.15</td><td>HUMAN</td><td>H3BPS8</td><td>Fructose-bisphosphate aldolase A (Fragment)</td></tr>
<tr><td>2096</td><td>dirMS_041712_NAR_120mM_plus.20630.20630.4</td><td>4</td><td>16.11</td><td>2</td><td>6.22E+05</td><td></td><td>(K)LTVEDLE</td><td>(K)L/T/V/E/D|L/E/K E R D F Y F G K(L)</td><td></td><td>160.9</td><td>498.0043</td><td>1988.996</td><td>-0.001</td><td>-0.5</td><td>4.51</td><td>30169.7</td><td>5.02</td><td>HUMAN</td><td>Q15691</td><td>Microtubule-associated protein RP/EB family member 1</td></tr>
<tr><td>2097</td><td>dirMS_041712_NAR_30mM_plus2.10950.11051.2</td><td>2</td><td>16.11</td><td>0</td><td>5.82E+05</td><td></td><td>(R)QLYEEEI</td><td>(R)Q/L|Y/E/E/E/I/R(E)</td><td></td><td>110.97</td><td>540.2736</td><td>1079.537</td><td>0.0032</td><td>2.9</td><td>4.25</td><td>37296.3</td><td>5.3</td><td>HUMAN</td><td>F8VU64</td><td>Keratin, type II cytoskeletal 8</td></tr>
<tr><td>2098</td><td>dirMS_041712_NAR_30mM_plus2.20756.20756.2</td><td>2</td><td>16.11</td><td>0</td><td>2.60E+05</td><td></td><td>(R)ASLSLW</td><td>(R)A S/L/S/L/W\Q A/M E K(G)</td><td></td><td>162.9</td><td>632.3231</td><td>1263.64</td><td>-0.0013</td><td>-1</td><td>6.05</td><td>266161</td><td>5.68</td><td>HUMAN</td><td>E9PPU0</td><td>Epiplakin</td></tr>
<tr><td>2099</td><td>dirMS_041712_NAR_30mM_plus2.9980.10438.2</td><td>2</td><td>16.11</td><td>0</td><td>3.14E+06</td><td></td><td>(K)DVNQQI</td><td>(K)D V|N|Q|Q E/F/V/R(A)</td><td></td><td>106.65</td><td>567.7813</td><td>1134.554</td><td>0.0015</td><td>1.3</td><td>4.37</td><td>16060.2</td><td>10.31</td><td>HUMAN</td><td>P39019</td><td>40S ribosomal protein S19</td></tr>
<tr><td>2100</td><td>dirMS_041712_NAR_40mM_plus2.12271.12447.2</td><td>2</td><td>16.11</td><td>0</td><td>1.06E+05</td><td></td><td>(R)VLQALE</td><td>(R)V/L|Q|A/L E/G L/K(M)</td><td></td><td>138.8</td><td>485.8002</td><td>970.593</td><td>0</td><td>0</td><td>5.97</td><td>16060.2</td><td>10.31</td><td>HUMAN</td><td>P39019</td><td>40S ribosomal protein S19</td></tr>
<tr><td>2101</td><td>dirMS_041712_NAR_50mM_plus2.14002.14171.3</td><td>3</td><td>16.11</td><td>1</td><td>1.21E+06</td><td></td><td>(K)ALQQGL</td><td>(K)A L|Q/Q/G L/V/G L/E L K\E K(L)</td><td></td><td>142.02</td><td>509.3022</td><td>1525.895</td><td>-0.0028</td><td>-1.8</td><td>6.18</td><td>266161</td><td>5.68</td><td>HUMAN</td><td>E9PPU0</td><td>Epiplakin</td></tr>
<tr><td>2102</td><td>dirMS_041712_NAR_50mM_plus2.17856.17900.3</td><td>3</td><td>16.11</td><td>0</td><td>2.57E+05</td><td></td><td>(K)YAPLHL</td><td>(K)Y A|P L/H/L|V|P/L I E R(L)</td><td></td><td>162.08</td><td>474.2819</td><td>1420.831</td><td>0.0001</td><td>0</td><td>6.75</td><td>147553.6</td><td>6.73</td><td>HUMAN</td><td>E7EVF4</td><td>Cytoplasmic FMR1-interacting protein 2</td></tr>
<tr><td>2103</td><td>dirMS_041712_NAR_120mM_minus.18058.18210.3</td><td>3</td><td>16.1</td><td>1</td><td>3.33E+06</td><td></td><td>(R)ARFEELN</td><td>(R)A R\F E\E\L|N|A|D/L/F/R(G)</td><td></td><td>154.37</td><td>494.2561</td><td>1480.754</td><td>-0.0006</td><td>-0.4</td><td>4.68</td><td>45508.6</td><td>5.5</td><td>HUMAN</td><td>A8K7Q2</td><td>Heat shock cognate 71 kDa protein</td></tr>
<tr><td>2104</td><td>dirMS_041712_NAR_60mM_minus.10011.10011.3</td><td>3</td><td>16.1</td><td>0</td><td>2.95E+04</td><td></td><td>(R)ISNRPAF</td><td>(R)I/S|N R P A F|M/P S/E G/K(M)</td><td></td><td>115.67</td><td>478.5785</td><td>1433.721</td><td>0.0004</td><td>0.3</td><td>8.75</td><td>104488.6</td><td>5.36</td><td>HUMAN</td><td>B2RCS5</td><td>Actinin, alpha 2, isoform CRA_b</td></tr>
<tr><td>2105</td><td>dirMS_041712_NAR_60mM_minus.15202.15202.2</td><td>2</td><td>16.1</td><td>0</td><td>4.20E+05</td><td></td><td>(R)EHALLA</td><td>(R)E/H/A|L|L/A/Y T L G V K(Q)</td><td></td><td>144.65</td><td>657.873</td><td>1314.742</td><td>-0.0029</td><td>-2.2</td><td>6.85</td><td>50482.3</td><td>9.31</td><td>HUMAN</td><td>P68104</td><td>Elongation factor 1-alpha 1</td></tr>
<tr><td>2106</td><td>dirMS_041712_NAR_60mM_minus.7338.7744.3</td><td>3</td><td>16.1</td><td>1</td><td>1.38E+06</td><td></td><td>(K)IQDKEGI</td><td>(K)I Q D K E G\I|P/P/D/Q Q R(L)</td><td></td><td>100.07</td><td>508.5978</td><td>1523.781</td><td>-0.0024</td><td>-1.6</td><td>4.56</td><td>17271.6</td><td>6.75</td><td>HUMAN</td><td>B4DV12</td><td>Ubiquitin</td></tr>
<tr><td>2107</td><td>dirMS_041712_NAR_120mM_plus.8216.8254.3</td><td>3</td><td>16.1</td><td>1</td><td>8.15E+05</td><td></td><td>(R)AVVIVDI</td><td>(R)A/V|V|I/V/D/D/R G/R P S G K(G)</td><td></td><td>96.73</td><td>490.2792</td><td>1468.823</td><td>0</td><td>0</td><td>8.79</td><td>23729.5</td><td>9.89</td><td>HUMAN</td><td>C9IZL7</td><td>Non-POU domain-containing octamer-binding protein (Fragment)</td></tr>
<tr><td>2108</td><td>dirMS_041712_NAR_20mM_plus.11303.11558.2</td><td>2</td><td>16.1</td><td>0</td><td>2.93E+05</td><td></td><td>(K)LITPAVV</td><td>(K)L I|T|P/A/V/V S E/R(L)</td><td></td><td>150.83</td><td>542.8186</td><td>1084.636</td><td>-0.0062</td><td>-5.7</td><td>6</td><td>13798.9</td><td>10.17</td><td>HUMAN</td><td>P62851</td><td>40S ribosomal protein S25</td></tr>
<tr><td>2109</td><td>dirMS_041712_NAR_30mM_plus2.8235.8533.2</td><td>2</td><td>16.1</td><td>0</td><td>1.06E+06</td><td></td><td>(R)ALANSL</td><td>(R)A L|A|N/S|L A/C/Q/G K(Y)</td><td>C:Carbamidomethylation</td><td>97.87</td><td>566.7922</td><td>1132.578</td><td>-0.0008</td><td>-0.7</td><td>8.8</td><td>13951.6</td><td>9.48</td><td>HUMAN</td><td>H3BR68</td><td>Fructose-bisphosphate aldolase A (Fragment)</td></tr>
<tr><td>2110</td><td>dirMS_041712_NAR_40mM_plus2.19392.19532.3</td><td>3</td><td>16.1</td><td>1</td><td>4.58E+05</td><td></td><td>(K)ALFEEVF</td><td>(K)A L|F/E/E/V/P E L L/T E A E K K(E)</td><td></td><td>181.77</td><td>615.9991</td><td>1845.984</td><td>-0.0017</td><td>-0.9</td><td>4.32</td><td>65036</td><td>6.27</td><td>HUMAN</td><td>E9PBU3</td><td>Phosphoribosylaminoimidazolecarboxamide formyltransferase</td></tr>
<tr><td>2111</td><td>dirMS_041712_NAR_50mM_plus2.14829.14829.3</td><td>3</td><td>16.1</td><td>1</td><td>4.71E+05</td><td>M93m</td><td>(K)SELVAm</td><td>(K)S E|L|V/A/m L E/E E E/L R/K(A)</td><td>m:Oxidized methionine</td><td>146.08</td><td>564.6211</td><td>1675.857</td><td>15.9916</td><td>-1.9</td><td>4.32</td><td>18679.2</td><td>5.61</td><td>HUMAN</td><td>B4DWW1</td><td>ADP-ribosylation factor-like protein 1</td></tr>
<tr><td>2112</td><td>dirMS_041712_NAR_60mM_plus.15623.15623.3</td><td>3</td><td>16.1</td><td>0</td><td>1.71E+05</td><td></td><td>(R)ILHGLGF</td><td>(R)I L H G L G F|T|P/A M Q R(K)</td><td></td><td>138.35</td><td>480.9305</td><td>1440.778</td><td>-0.0011</td><td>-0.7</td><td>9.75</td><td>27043.5</td><td>6.34</td><td>HUMAN</td><td>C9JHK9</td><td>ATP-binding cassette sub-family F member 2 (Fragment)</td></tr>
<tr><td>2113</td><td>dirMS_041712_NAR_60mM_plus.7115.7115.2</td><td>2</td><td>16.1</td><td>0</td><td>1.72E+06</td><td></td><td>(K)STTTGHI</td><td>(K)S/T|T/T/G/H L\I/Y/K(C)</td><td></td><td>95.58</td><td>560.8031</td><td>1120.6</td><td>-0.0008</td><td>-0.7</td><td>8.34</td><td>12137.8</td><td>9.3</td><td>HUMAN</td><td>A6PW80</td><td>Elongation factor 1-alpha 1 (Fragment)</td></tr>
<tr><td>2114</td><td>dirMS_041712_NAR_120mM_minus.12126.12437.3</td><td>3</td><td>16.09</td><td>0</td><td>1.58E+06</td><td></td><td>(R)HVFGES</td><td>(R)H V\F G\E|S|D|E|L|I/G Q/K(V)</td><td></td><td>120.68</td><td>486.9119</td><td>1458.722</td><td>-0.0012</td><td>-0.8</td><td>4.65</td><td>31075.7</td><td>5.65</td><td>HUMAN</td><td>P60174</td><td>Triosephosphate isomerase</td></tr>
<tr><td>2115</td><td>dirMS_041712_NAR_30mM_minus2.9172.9172.2</td><td>2</td><td>16.09</td><td>0</td><td>4.11E+04</td><td></td><td>(R)TYQASS</td><td>(R)T Y|Q/A/S S\A\A F/R(Y)</td><td></td><td>104.48</td><td>551.2778</td><td>1101.532</td><td>0.016</td><td>14.5</td><td>8.41</td><td>26240.9</td><td>9.1</td><td>HUMAN</td><td>Q92567</td><td>Protein FAM168A</td></tr>
<tr><td>2116</td><td>dirMS_041712_NAR_120mM_plus.20441.20958.4</td><td>4</td><td>16.09</td><td>1</td><td>3.77E+06</td><td></td><td>(R)DYLHLPI</td><td>(R)D Y L H L|P\P/E I/V/P A T L R/R(S)</td><td></td><td>162.23</td><td>473.2689</td><td>1890.06</td><td>-0.0058</td><td>-3.1</td><td>6.75</td><td>19924.8</td><td>9.99</td><td>HUMAN</td><td>F6U211</td><td>40S ribosomal protein S10</td></tr>
<tr><td>2117</td><td>dirMS_041712_NAR_20mM_plus.18627.18793.2</td><td>2</td><td>16.09</td><td>0</td><td>3.00E+05</td><td></td><td>(K)TAVETA</td><td>(K)T\A\V|E/T/A/V/L|L L R(I)</td><td></td><td>199.87</td><td>593.3625</td><td>1185.72</td><td>-0.0024</td><td>-2</td><td>5.66</td><td>56186.7</td><td>5.47</td><td>HUMAN</td><td>B4DUR8</td><td>T-complex protein 1 subunit gamma</td></tr>
<tr><td>2118</td><td>dirMS_041712_NAR_30mM_plus2.14339.14339.2</td><td>2</td><td>16.09</td><td>0</td><td>5.73E+05</td><td></td><td>(K)CCSGAII</td><td>(K)C C|S/G A I|I/V|L\T K(S)</td><td>C:Carbamidomethylation</td><td>128.2</td><td>611.3189</td><td>1221.633</td><td>-0.0025</td><td>-2</td><td>8.75</td><td>58506.4</td><td>8.38</td><td>HUMAN</td><td>P14618</td><td>Pyruvate kinase isozymes M1/M2</td></tr>
<tr><td>2119</td><td>dirMS_041712_NAR_30mM_minus2.12766.12766.2</td><td>2</td><td>16.08</td><td>0</td><td>3.27E+05</td><td></td><td>(R)GGAEQF</td><td>(R)G G A\E/Q|F|M/E E/T/E R(S)</td><td></td><td>122.7</td><td>692.2961</td><td>1383.584</td><td>0.0004</td><td>0.3</td><td>4.09</td><td>46550.7</td><td>7.81</td><td>HUMAN</td><td>B7Z1C9</td><td>Chaperonin containing TCP1, subunit 7 (Eta), isoform CRA_a</td></tr>
<tr><td>2120</td><td>dirMS_041712_NAR_30mM_minus2.18870.18870.2</td><td>2</td><td>16.08</td><td>0</td><td>5.41E+04</td><td></td><td>(R)CGGIQY</td><td>(R)C G/G\I|Q|Y/L\Q\N/A I/E/S/R(Q)</td><td>C:Carbamidomethylation</td><td>157.12</td><td>804.8951</td><td>1608.78</td><td>0.0031</td><td>1.9</td><td>6</td><td>150202.8</td><td>9.1</td><td>HUMAN</td><td>E7EW20</td><td>Unconventional myosin-VI</td></tr>
<tr><td>2121</td><td>dirMS_041712_NAR_30mM_minus2.7406.7552.2</td><td>2</td><td>16.08</td><td>0</td><td>9.71E+05</td><td></td><td>(R)EMDEQI</td><td>(R)E/M/D/E/Q/I/R(L)</td><td></td><td>95.1</td><td>460.7171</td><td>920.414</td><td>0.0127</td><td>13.8</td><td>4.14</td><td>33450.2</td><td>4.73</td><td>HUMAN</td><td>J3KN67</td><td>Tropomyosin alpha-3 chain</td></tr>
<tr><td>2122</td><td>dirMS_041712_NAR_60mM_minus.15853.16095.3</td><td>3</td><td>16.08</td><td>1</td><td>5.23E+05</td><td></td><td>(R)IFDSEEIL</td><td>(R)I/F\D/S E/E/I/L/A/G Y K/R(E)</td><td></td><td>148.88</td><td>514.2713</td><td>1540.801</td><td>-0.0012</td><td>-0.8</td><td>4.68</td><td>35548.6</td><td>4.91</td><td>HUMAN</td><td>B4DN60</td><td>Asparagine--tRNA ligase, cytoplasmic</td></tr>
<tr><td>2123</td><td>dirMS_041712_NAR_20mM_plus.13230.13644.2</td><td>2</td><td>16.08</td><td>0</td><td>4.95E+05</td><td>M148m</td><td>(R)DLTDYL</td><td>(R)D/L|T/D/Y/L/m/K(I)</td><td>m:Oxidized methionine</td><td>164.77</td><td>507.7439</td><td>998.486</td><td>15.9942</td><td>-0.7</td><td>4.21</td><td>37482.1</td><td>5.36</td><td>HUMAN</td><td>E9PG30</td><td>Actin, gamma-enteric smooth muscle</td></tr>
<tr><td>2124</td><td>dirMS_041712_NAR_20mM_plus.16417.16417.2</td><td>2</td><td>16.08</td><td>0</td><td>1.40E+05</td><td></td><td>(R)QAQEYE</td><td>(R)Q A|Q/E Y/E/A L/L/N/I/K(V)</td><td></td><td>184.52</td><td>710.3754</td><td>1419.748</td><td>-0.0043</td><td>-3</td><td>4.53</td><td>43773.3</td><td>5.26</td><td>HUMAN</td><td>F8VZY9</td><td>Keratin, type I cytoskeletal 18</td></tr>
<tr><td>2125</td><td>dirMS_041712_NAR_30mM_plus2.12798.13079.2</td><td>2</td><td>16.08</td><td>0</td><td>1.75E+06</td><td></td><td>(R)LQAEIEG</td><td>(R)L/Q|A|E/I/E/G/L/K(G)</td><td></td><td>121.42</td><td>500.7889</td><td>1000.567</td><td>0.0032</td><td>3.2</td><td>4.53</td><td>37296.3</td><td>5.3</td><td>HUMAN</td><td>F8VU64</td><td>Keratin, type II cytoskeletal 8</td></tr>
<tr><td>2126</td><td>dirMS_041712_NAR_50mM_plus2.14407.14407.3</td><td>3</td><td>16.08</td><td>1</td><td>9.68E+05</td><td></td><td>(R)LAIKLPD</td><td>(R)L/A/I K L/P D D/Q I/P K(T)</td><td></td><td>144.23</td><td>450.9373</td><td>1350.799</td><td>-0.0018</td><td>-1.3</td><td>5.96</td><td>26413</td><td>9.18</td><td>HUMAN</td><td>B1APP6</td><td>6-phosphofructokinase type C (Fragment)</td></tr>
<tr><td>2127</td><td>dirMS_041712_NAR_60mM_plus.14918.15248.3</td><td>3</td><td>16.08</td><td>0</td><td>2.92E+06</td><td></td><td>(-)MVNHFI</td><td>(-)M/V/N/H/F/I A E/F/K(R)</td><td></td><td>135.18</td><td>412.5464</td><td>1235.624</td><td>0.0005</td><td>0.4</td><td>6.5</td><td>45508.6</td><td>5.5</td><td>HUMAN</td><td>A8K7Q2</td><td>Heat shock cognate 71 kDa protein</td></tr>
<tr><td>2128</td><td>dirMS_041712_NAR_120mM_minus.20119.20119.3</td><td>3</td><td>16.07</td><td>0</td><td>5.07E+05</td><td>M195m</td><td>(R)LHFFmP</td><td>(R)L H F F m\P G|F/A/P/L/T S R(G)</td><td>m:Oxidized methionine</td><td>166.18</td><td>546.2809</td><td>1620.835</td><td>15.9926</td><td>-1.4</td><td>9.75</td><td>42230.8</td><td>4.77</td><td>HUMAN</td><td>A6NKZ8</td><td>Putative tubulin beta chain-like protein ENSP00000290377</td></tr>
<tr><td>2129</td><td>dirMS_041712_NAR_120mM_plus.7602.7602.2</td><td>2</td><td>16.07</td><td>1</td><td>1.19E+06</td><td></td><td>(R)AQYDEL</td><td>(R)A/Q|Y/D/E/L A/R K(N)</td><td></td><td>93.32</td><td>547.2874</td><td>1093.564</td><td>0.0039</td><td>3.5</td><td>6.11</td><td>43773.3</td><td>5.26</td><td>HUMAN</td><td>F8VZY9</td><td>Keratin, type I cytoskeletal 18</td></tr>
<tr><td>2130</td><td>dirMS_041712_NAR_30mM_plus2.19075.19616.2</td><td>2</td><td>16.07</td><td>0</td><td>1.20E+06</td><td></td><td>(R)LIDFLEC</td><td>(R)L/I|D|F|L/E/C/G/K(T)</td><td>C:Carbamidomethylation</td><td>155.15</td><td>547.7808</td><td>1094.555</td><td>-0.0007</td><td>-0.7</td><td>4.37</td><td>61018.6</td><td>9.44</td><td>HUMAN</td><td>B4DLW8</td><td>Probable ATP-dependent RNA helicase DDX5</td></tr>
<tr><td>2131</td><td>dirMS_041712_NAR_30mM_plus2.19535.19535.2</td><td>2</td><td>16.07</td><td>0</td><td>1.85E+04</td><td></td><td>(K)MNEFLE</td><td>(K)M/N/E|F|L/E N F E K(N)</td><td></td><td>155.87</td><td>650.7974</td><td>1300.588</td><td>-0.0003</td><td>-0.2</td><td>4.25</td><td>28580.2</td><td>9.35</td><td>HUMAN</td><td>Q9UFN0</td><td>Protein NipSnap homolog 3A</td></tr>
<tr><td>2132</td><td>dirMS_041712_NAR_30mM_plus2.19653.19868.2</td><td>2</td><td>16.07</td><td>0</td><td>7.76E+05</td><td></td><td>(K)VLTPELY</td><td>(K)V L|T|P/E/L Y/A E/L R(A)</td><td></td><td>157.2</td><td>652.3617</td><td>1303.726</td><td>-0.0095</td><td>-7.3</td><td>4.53</td><td>14391.8</td><td>4.99</td><td>HUMAN</td><td>G3V461</td><td>Creatine kinase B-type (Fragment)</td></tr>
<tr><td>2133</td><td>dirMS_041712_NAR_40mM_plus2.12440.12440.3</td><td>3</td><td>16.07</td><td>0</td><td>3.39E+05</td><td></td><td>(K)AASDIAI</td><td>(K)A A\S D\I/A|M/T E L/P/P/T H P I R(L)</td><td></td><td>139.6</td><td>607.3167</td><td>1819.937</td><td>-0.0015</td><td>-0.9</td><td>5.32</td><td>12926.1</td><td>4.4</td><td>HUMAN</td><td>K7EM20</td><td>14-3-3 protein epsilon (Fragment)</td></tr>
<tr><td>2134</td><td>dirMS_041712_NAR_40mM_plus2.5423.5458.2</td><td>2</td><td>16.07</td><td>0</td><td>2.45E+05</td><td>M300m</td><td>(K)NMmAA</td><td>(K)N|M|m|A|A/C/D|P/R(H)</td><td>C:Carbamidomethylation m:Oxid</td><td>89.2</td><td>541.2145</td><td>1065.427</td><td>15.9943</td><td>-0.6</td><td>5.84</td><td>50199.4</td><td>4.75</td><td>HUMAN</td><td>A6NNZ2</td><td>Tubulin beta-8 chain-like protein LOC260334</td></tr>
<tr><td>2135</td><td>dirMS_041712_NAR_50mM_plus2.11628.11988.3</td><td>3</td><td>16.07</td><td>1</td><td>1.78E+06</td><td></td><td>(K)TGQPMI</td><td>(K)T G\Q|P/M/I/N L Y/T/D R E|T G K(L)</td><td></td><td>130.72</td><td>608.6361</td><td>1823.896</td><td>-0.0019</td><td>-1</td><td>5.73</td><td>53724.2</td><td>9.54</td><td>HUMAN</td><td>H3BPE7</td><td>RNA-binding protein FUS</td></tr>
<tr><td>2136</td><td>dirMS_041712_NAR_120mM_minus.5401.5423.3</td><td>3</td><td>16.06</td><td>1</td><td>2.17E+05</td><td></td><td>(R)KQQTLE</td><td>(R)K\Q Q\T\L|E/A/E E/A/K(R)</td><td></td><td>82.88</td><td>425.5623</td><td>1274.659</td><td>0.0137</td><td>10.7</td><td>4.79</td><td>76925.9</td><td>4.46</td><td>HUMAN</td><td>A8MV58</td><td>Drebrin</td></tr>
<tr><td>2137</td><td>dirMS_041712_NAR_50mM_minus2.8545.8652.3</td><td>3</td><td>16.06</td><td>0</td><td>1.58E+06</td><td></td><td>(R)EIVHIQA</td><td>(R)E/I|V H I Q\A\G/Q|C/G N Q\I/G A/K(F)</td><td>C:Carbamidomethylation</td><td>116.8</td><td>608.3087</td><td>1822.923</td><td>-0.0113</td><td>-6.2</td><td>6.85</td><td>40069.4</td><td>4.83</td><td>HUMAN</td><td>E7EWR1</td><td>Tubulin beta chain</td></tr>
<tr><td>2138</td><td>dirMS_041712_NAR_20mM_plus.17952.17969.2</td><td>2</td><td>16.06</td><td>0</td><td>1.63E+05</td><td></td><td>(R)TVQSLEI</td><td>(R)T V|Q|S/L/E|I\D L D/S M R(N)</td><td></td><td>195.05</td><td>753.8776</td><td>1506.747</td><td>0.0011</td><td>0.7</td><td>4.03</td><td>43773.3</td><td>5.26</td><td>HUMAN</td><td>F8VZY9</td><td>Keratin, type I cytoskeletal 18</td></tr>
<tr><td>2139</td><td>dirMS_041712_NAR_30mM_plus2.11112.11174.2</td><td>2</td><td>16.06</td><td>0</td><td>6.16E+05</td><td></td><td>(R)AQPSAS</td><td>(R)A Q|P/S/A/S/L/G V/G Y R(T)</td><td></td><td>111.58</td><td>603.3192</td><td>1205.627</td><td>0.0038</td><td>3.2</td><td>8.79</td><td>69661.6</td><td>9.75</td><td>HUMAN</td><td>Q96PK6</td><td>RNA-binding protein 14</td></tr>
<tr><td>2140</td><td>dirMS_041712_NAR_30mM_plus2.18436.18585.2</td><td>2</td><td>16.06</td><td>0</td><td>5.82E+05</td><td></td><td>(R)NNLAGA</td><td>(R)N N|L|A|G/A/E E/L/F/A R(K)</td><td></td><td>150.42</td><td>652.8322</td><td>1304.659</td><td>-0.0022</td><td>-1.7</td><td>4.53</td><td>193379.8</td><td>5.48</td><td>HUMAN</td><td>Q00610</td><td>Clathrin heavy chain 1</td></tr>
<tr><td>2141</td><td>dirMS_041712_NAR_30mM_plus2.9578.9701.2</td><td>2</td><td>16.06</td><td>0</td><td>4.97E+05</td><td></td><td>(R)LAPEYEA</td><td>(R)L/A|P/E/Y/E/A/A A T R(L)</td><td></td><td>104.48</td><td>596.3026</td><td>1191.6</td><td>-0.0025</td><td>-2.1</td><td>4.53</td><td>55361.8</td><td>6.42</td><td>HUMAN</td><td>G5EA52</td><td>Protein disulfide isomerase family A, member 3, isoform CRA_b</td></tr>
<tr><td>2142</td><td>dirMS_041712_NAR_40mM_plus2.14722.14868.3</td><td>3</td><td>16.06</td><td>1</td><td>1.15E+06</td><td></td><td>(R)IIAFVGS</td><td>(R)I/I|A|F/V/G/S/P V E D N E K D L V/K(L)</td><td></td><td>153.98</td><td>658.3499</td><td>1973.059</td><td>-0.0238</td><td>-12.1</td><td>4.32</td><td>21953.7</td><td>6.6</td><td>HUMAN</td><td>A6PVX3</td><td>26S proteasome non-ATPase regulatory subunit 4 (Fragment)</td></tr>
<tr><td>2143</td><td>dirMS_041712_NAR_20mM_minus.11273.11479.2</td><td>2</td><td>16.05</td><td>0</td><td>1.17E+07</td><td>M148m</td><td>(R)DLTDYL</td><td>(R)D L|T|D/Y/L/m/K(I)</td><td>m:Oxidized methionine</td><td>162.17</td><td>507.7443</td><td>998.486</td><td>15.995</td><td>0.1</td><td>4.21</td><td>37482.1</td><td>5.36</td><td>HUMAN</td><td>E9PG30</td><td>Actin, gamma-enteric smooth muscle</td></tr>
<tr><td>2144</td><td>dirMS_041712_NAR_30mM_minus2.17120.17120.2</td><td>2</td><td>16.05</td><td>0</td><td>1.88E+05</td><td></td><td>(R)SIQEELC</td><td>(R)S I|Q/E/E L/Q Q/L/R(Q)</td><td></td><td>146.55</td><td>622.3359</td><td>1243.664</td><td>0.0004</td><td>0.3</td><td>4.53</td><td>533778</td><td>5.74</td><td>HUMAN</td><td>Q15149</td><td>Plectin</td></tr>
<tr><td>2145</td><td>dirMS_041712_NAR_30mM_minus2.18180.18198.2</td><td>2</td><td>16.05</td><td>0</td><td>1.57E+05</td><td></td><td>(K)FGAYIVD</td><td>(K)F/G|A|Y|I/V/D G L/R(Q)</td><td></td><td>153.6</td><td>555.7984</td><td>1110.594</td><td>-0.0047</td><td>-4.2</td><td>5.83</td><td>269989.9</td><td>5.92</td><td>HUMAN</td><td>F8W8T8</td><td>Acetyl-CoA carboxylase 2</td></tr>
<tr><td>2146</td><td>dirMS_041712_NAR_50mM_minus2.16754.16754.3</td><td>3</td><td>16.05</td><td>1</td><td>2.96E+05</td><td></td><td>(R)SELELTL</td><td>(R)S E|L|E L T L/G K L E/Q V R(S)</td><td></td><td>163.72</td><td>538.9725</td><td>1614.906</td><td>-0.0032</td><td>-2</td><td>4.78</td><td>533778</td><td>5.74</td><td>HUMAN</td><td>Q15149</td><td>Plectin</td></tr>
<tr><td>2147</td><td>dirMS_041712_NAR_50mM_minus2.8993.8993.3</td><td>3</td><td>16.05</td><td>0</td><td>2.22E+05</td><td></td><td>(R)VMLGET</td><td>(R)V M|L/G E/T/N/P A D S K/P G T/I R(G)</td><td></td><td>118.33</td><td>595.9744</td><td>1785.916</td><td>-0.0077</td><td>-4.3</td><td>6.04</td><td>15431.7</td><td>9</td><td>HUMAN</td><td>E7ERL0</td><td>Nucleoside diphosphate kinase A</td></tr>
<tr><td>2148</td><td>dirMS_041712_NAR_60mM_minus.10790.10790.3</td><td>3</td><td>16.05</td><td>1</td><td>5.44E+05</td><td>M239m</td><td>(R)MSMKE</td><td>(R)M/S|M K E V D E|Q|m|L N/V/Q N K(N)</td><td>m:Oxidized methionine</td><td>119.9</td><td>647.3011</td><td>1923.897</td><td>15.9915</td><td>-1.8</td><td>4.68</td><td>40069.4</td><td>4.83</td><td>HUMAN</td><td>E7EWR1</td><td>Tubulin beta chain</td></tr>
<tr><td>2149</td><td>dirMS_041712_NAR_40mM_plus2.17752.17752.3</td><td>3</td><td>16.05</td><td>0</td><td>3.79E+04</td><td></td><td>(R)AGLGSG</td><td>(R)A G L|G/S/G L/S L/S G L V/H P E\L S\R(S)</td><td></td><td>170.98</td><td>617.3431</td><td>1850.013</td><td>0.0017</td><td>0.9</td><td>6.79</td><td>73325.2</td><td>9.19</td><td>HUMAN</td><td>F5H2A9</td><td>Very long-chain-specific acyl-CoA dehydrogenase, mitochondrial</td></tr>
<tr><td>2150</td><td>dirMS_041712_NAR_50mM_plus2.13312.13600.3</td><td>3</td><td>16.05</td><td>0</td><td>2.57E+06</td><td></td><td>(K)AGVVGF</td><td>(K)A/G V|V|G/P E L/H/E Q L L/S A E K(A)</td><td></td><td>138.58</td><td>592.986</td><td>1776.949</td><td>-0.0056</td><td>-3.1</td><td>4.75</td><td>533778</td><td>5.74</td><td>HUMAN</td><td>Q15149</td><td>Plectin</td></tr>
<tr><td>2151</td><td>dirMS_041712_NAR_50mM_plus2.15907.15954.3</td><td>3</td><td>16.05</td><td>1</td><td>1.60E+06</td><td></td><td>(R)DVDFELI</td><td>(R)D V|D/F/E/L/I K/V E/G K(V)</td><td></td><td>152</td><td>464.5859</td><td>1391.742</td><td>0.0015</td><td>1.1</td><td>4.32</td><td>49982</td><td>5.86</td><td>HUMAN</td><td>B4DYD8</td><td>T-complex protein 1 subunit epsilon</td></tr>
<tr><td>2152</td><td>dirMS_041712_NAR_50mM_plus2.5647.5730.3</td><td>3</td><td>16.05</td><td>1</td><td>1.06E+06</td><td></td><td>(K)TAGPIAS</td><td>(K)T/A|G/P I/A/S\A/Q\K Q/P A/G K(V)</td><td></td><td>91.9</td><td>475.5987</td><td>1424.786</td><td>-0.0041</td><td>-2.8</td><td>10</td><td>75259.2</td><td>9.59</td><td>HUMAN</td><td>B5MEG9</td><td>Microtubule-associated protein 4 (Fragment)</td></tr>
<tr><td>2153</td><td>dirMS_041712_NAR_30mM_minus2.7672.7680.2</td><td>2</td><td>16.04</td><td>0</td><td>2.56E+05</td><td></td><td>(K)LAGESES</td><td>(K)L/A|G|E|S/E/S N/L/R(K)</td><td></td><td>96.3</td><td>538.273</td><td>1075.538</td><td>0.0009</td><td>0.8</td><td>4.53</td><td>90004.9</td><td>5.14</td><td>HUMAN</td><td>P55072</td><td>Transitional endoplasmic reticulum ATPase</td></tr>
<tr><td>2154</td><td>dirMS_041712_NAR_50mM_minus2.15439.15439.3</td><td>3</td><td>16.04</td><td>0</td><td>3.28E+05</td><td></td><td>(R)LSFQHD</td><td>(R)L/S/F Q/H D P E|T S/V/L/V/L R(K)</td><td></td><td>156.07</td><td>580.9793</td><td>1740.928</td><td>-0.0046</td><td>-2.6</td><td>5.32</td><td>96827.3</td><td>5.58</td><td>HUMAN</td><td>E9PKU7</td><td>Neutral alpha-glucosidase AB</td></tr>
<tr><td>2155</td><td>dirMS_041712_NAR_20mM_plus.13810.13810.2</td><td>2</td><td>16.04</td><td>0</td><td>6.96E+04</td><td></td><td>(R)VEVTEFE</td><td>(R)V E|V/T E F E D/I/K(S)</td><td></td><td>167.18</td><td>604.8056</td><td>1208.604</td><td>-0.0006</td><td>-0.5</td><td>4</td><td>30992.3</td><td>4.14</td><td>HUMAN</td><td>B2REB8</td><td>Protein SET</td></tr>
<tr><td>2156</td><td>dirMS_041712_NAR_30mM_plus2.11766.11766.2</td><td>2</td><td>16.04</td><td>0</td><td>5.40E+05</td><td></td><td>(K)VASGCLI</td><td>(K)V A|S/G/C/L/D I N S S V/K(G)</td><td>C:Carbamidomethylation</td><td>114.82</td><td>675.3393</td><td>1349.673</td><td>-0.0016</td><td>-1.2</td><td>5.81</td><td>56837</td><td>7.19</td><td>HUMAN</td><td>E7ETT1</td><td>Propionyl-CoA carboxylase beta chain, mitochondrial</td></tr>
<tr><td>2157</td><td>dirMS_041712_NAR_20mM_minus.10042.10042.2</td><td>2</td><td>16.03</td><td>0</td><td>8.73E+05</td><td></td><td>(R)IVLQIDN</td><td>(R)I/V|L/Q/I|D/N/A R(L)</td><td></td><td>149.7</td><td>521.3059</td><td>1041.605</td><td>-0.0006</td><td>-0.6</td><td>5.84</td><td>20842.9</td><td>5.32</td><td>HUMAN</td><td>C9JM50</td><td>Keratin, type I cytoskeletal 19 (Fragment)</td></tr>
<tr><td>2158</td><td>dirMS_041712_NAR_50mM_minus2.15319.15465.3</td><td>3</td><td>16.03</td><td>1</td><td>4.51E+05</td><td></td><td>(K)GADFLV</td><td>(K)G A\D|F|L|V/T/E V E/N G G S L G S K K(G)</td><td></td><td>155.55</td><td>636.662</td><td>1907.971</td><td>0.0005</td><td>0.3</td><td>4.68</td><td>30947.9</td><td>6.47</td><td>HUMAN</td><td>H3BQ34</td><td>Pyruvate kinase</td></tr>
<tr><td>2159</td><td>dirMS_041712_NAR_60mM_minus.7087.7148.3</td><td>3</td><td>16.03</td><td>1</td><td>3.04E+05</td><td></td><td>(R)ELFESST</td><td>(R)E L|F/E/S/S T N N N K D/T/K(Q)</td><td></td><td>98.72</td><td>542.9247</td><td>1626.761</td><td>-0.001</td><td>-0.6</td><td>4.68</td><td>150202.8</td><td>9.1</td><td>HUMAN</td><td>E7EW20</td><td>Unconventional myosin-VI</td></tr>
<tr><td>2160</td><td>dirMS_041712_NAR_30mM_plus2.10005.10024.2</td><td>2</td><td>16.03</td><td>0</td><td>2.67E+05</td><td></td><td>(R)ELSEQLC</td><td>(R)E L|S E|Q/L|G/Q/A E/R(A)</td><td></td><td>105.82</td><td>630.3143</td><td>1259.623</td><td>-0.0013</td><td>-1</td><td>4.25</td><td>266161</td><td>5.68</td><td>HUMAN</td><td>E9PPU0</td><td>Epiplakin</td></tr>
<tr><td>2161</td><td>dirMS_041712_NAR_50mM_plus2.20408.20551.3</td><td>3</td><td>16.03</td><td>1</td><td>4.12E+05</td><td></td><td>(R)VISGVLC</td><td>(R)V/I|S G/V/L/Q/L/G N I|V F K K(E)</td><td></td><td>176.1</td><td>539.0011</td><td>1614.994</td><td>-0.0054</td><td>-3.3</td><td>10</td><td>227783.5</td><td>5.5</td><td>HUMAN</td><td>P35579</td><td>Myosin-9</td></tr>
<tr><td>2162</td><td>dirMS_041712_NAR_50mM_plus2.7501.7501.3</td><td>3</td><td>16.03</td><td>0</td><td>2.12E+05</td><td></td><td>(R)SAEEEA</td><td>(R)S A|E/E/E A A/D/L P T K P T K(I)</td><td></td><td>105.3</td><td>529.5984</td><td>1586.791</td><td>-0.0102</td><td>-6.4</td><td>4.41</td><td>18924.5</td><td>6.86</td><td>HUMAN</td><td>Q8WW12</td><td>PEST proteolytic signal-containing nuclear protein</td></tr>
<tr><td>2163</td><td>dirMS_041712_NAR_120mM_minus.10608.10643.3</td><td>3</td><td>16.02</td><td>1</td><td>2.46E+05</td><td></td><td>(R)YLAEFAT</td><td>(R)Y/L|A E/F/A/T/G N D R K(E)</td><td></td><td>111.73</td><td>462.2338</td><td>1384.686</td><td>0.0013</td><td>0.9</td><td>6.07</td><td>12926.1</td><td>4.4</td><td>HUMAN</td><td>K7EM20</td><td>14-3-3 protein epsilon (Fragment)</td></tr>
<tr><td>2164</td><td>dirMS_041712_NAR_30mM_minus2.16902.16902.2</td><td>2</td><td>16.02</td><td>0</td><td>1.48E+05</td><td></td><td>(R)ASLEAAI</td><td>(R)A S/L E/A|A/I/A/D A/E Q R(G)</td><td></td><td>145.03</td><td>672.8404</td><td>1344.675</td><td>-0.0019</td><td>-1.4</td><td>4.14</td><td>56607.4</td><td>5.37</td><td>HUMAN</td><td>F8VXB4</td><td>Keratin, type II cytoskeletal 8</td></tr>
<tr><td>2165</td><td>dirMS_041712_NAR_30mM_minus2.5645.5686.2</td><td>2</td><td>16.02</td><td>0</td><td>1.73E+05</td><td></td><td>(R)LYQAGE</td><td>(R)L/Y|Q|A/G E/G R(L)</td><td></td><td>84.73</td><td>447.2217</td><td>893.448</td><td>-0.0114</td><td>-12.8</td><td>6</td><td>38089.6</td><td>6.79</td><td>HUMAN</td><td>B4DT77</td><td>Annexin</td></tr>
<tr><td>2166</td><td>dirMS_041712_NAR_50mM_minus2.14781.14781.3</td><td>3</td><td>16.02</td><td>0</td><td>3.07E+04</td><td></td><td>(K)GQYFGE</td><td>(K)G Q/Y/F/G E|L A L/V T/N K P R(A)</td><td></td><td>152.52</td><td>564.9709</td><td>1692.907</td><td>-0.0086</td><td>-5.1</td><td>8.59</td><td>10305.6</td><td>6.94</td><td>HUMAN</td><td>H7C330</td><td>cAMP-dependent protein kinase type II-alpha regulatory subunit (Fragment)</td></tr>
<tr><td>2167</td><td>dirMS_041712_NAR_50mM_minus2.15148.15341.3</td><td>3</td><td>16.02</td><td>1</td><td>3.90E+05</td><td></td><td>(R)VVVDAL</td><td>(R)V/V|V/D/A/L/S/G L K/G D L A G R(Y)</td><td></td><td>154.87</td><td>523.9677</td><td>1569.896</td><td>-0.0073</td><td>-4.7</td><td>5.93</td><td>26949.2</td><td>7.99</td><td>HUMAN</td><td>C9JSQ1</td><td>Creatine kinase U-type, mitochondrial (Fragment)</td></tr>
<tr><td>2168</td><td>dirMS_041712_NAR_20mM_plus.13936.13936.2</td><td>2</td><td>16.02</td><td>0</td><td>6.12E+04</td><td></td><td>(K)IVPNVLL</td><td>(K)I/V|P N V/L/L/E Q/G K(A)</td><td></td><td>167.72</td><td>605.3616</td><td>1209.72</td><td>-0.0042</td><td>-3.5</td><td>6</td><td>50659.2</td><td>7.87</td><td>HUMAN</td><td>B4DJV2</td><td>Citrate synthase</td></tr>
<tr><td>2169</td><td>dirMS_041712_NAR_30mM_plus2.20844.21209.2</td><td>2</td><td>16.02</td><td>0</td><td>3.43E+05</td><td></td><td>(K)FIIPQIVK</td><td>(K)F/I|I|P Q/I\V/K(Y)</td><td></td><td>164.53</td><td>479.3092</td><td>957.613</td><td>-0.002</td><td>-2.1</td><td>8.75</td><td>25445.8</td><td>5.52</td><td>HUMAN</td><td>A8MW50</td><td>L-lactate dehydrogenase (Fragment)</td></tr>
<tr><td>2170</td><td>dirMS_041712_NAR_30mM_plus2.22352.22352.2</td><td>2</td><td>16.02</td><td>0</td><td>3.76E+05</td><td></td><td>(R)VGNLGL</td><td>(R)V G N|L|G/L/A/T/S F F N/E R(N)</td><td></td><td>172.82</td><td>762.8926</td><td>1524.781</td><td>-0.0026</td><td>-1.7</td><td>5.97</td><td>73641.5</td><td>6.74</td><td>HUMAN</td><td>O00571</td><td>ATP-dependent RNA helicase DDX3X</td></tr>
<tr><td>2171</td><td>dirMS_041712_NAR_30mM_plus2.6845.6970.2</td><td>2</td><td>16.02</td><td>0</td><td>4.77E+05</td><td></td><td>(R)VISSIEQI</td><td>(R)V/I|S/S/I/E/Q/K(T)</td><td></td><td>90.68</td><td>452.2602</td><td>903.515</td><td>-0.0014</td><td>-1.6</td><td>5.97</td><td>18789.1</td><td>6.77</td><td>HUMAN</td><td>A2IDB2</td><td>14-3-3 protein eta (Fragment)</td></tr>
<tr><td>2172</td><td>dirMS_041712_NAR_40mM_plus2.15544.15584.3</td><td>3</td><td>16.02</td><td>1</td><td>3.51E+04</td><td></td><td>(R)IILPPEKE</td><td>(R)I I|L|P/P E K E L|A|M/P G E D/L/K(F)</td><td></td><td>158.55</td><td>631.6874</td><td>1893.04</td><td>0.0075</td><td>4</td><td>4.41</td><td>49883</td><td>7.3</td><td>HUMAN</td><td>P49411</td><td>Elongation factor Tu, mitochondrial</td></tr>
<tr><td>2173</td><td>dirMS_041712_NAR_50mM_plus2.18796.18796.3</td><td>3</td><td>16.02</td><td>1</td><td>4.32E+05</td><td></td><td>(K)ITQDIFQ</td><td>(K)I/T/Q/D|I/F/Q Q L/L K/R(G)</td><td></td><td>167.02</td><td>501.627</td><td>1502.869</td><td>-0.0025</td><td>-1.7</td><td>8.75</td><td>72796.9</td><td>6.75</td><td>HUMAN</td><td>A6NC17</td><td>Methionine--tRNA ligase, cytoplasmic</td></tr>
<tr><td>2174</td><td>dirMS_041712_NAR_50mM_minus2.14331.14665.3</td><td>3</td><td>16.01</td><td>1</td><td>3.52E+06</td><td></td><td>(R)IINEPTA</td><td>(R)I/I|N|E|P T A A/A/I/A Y G L D K K(V)</td><td></td><td>151.02</td><td>596.6655</td><td>1787.99</td><td>-0.0082</td><td>-4.6</td><td>6.07</td><td>17973</td><td>5.16</td><td>HUMAN</td><td>E9PI65</td><td>Heat shock cognate 71 kDa protein (Fragment)</td></tr>
<tr><td>2175</td><td>dirMS_041712_NAR_50mM_minus2.14474.14474.3</td><td>3</td><td>16.01</td><td>1</td><td>3.52E+06</td><td></td><td>(R)IINEPTA</td><td>(R)I/I|N\E/P T A A A I/A/Y/G L D/K K(V)</td><td></td><td>151.02</td><td>596.6655</td><td>1787.99</td><td>-0.0082</td><td>-4.6</td><td>6.07</td><td>17973</td><td>5.16</td><td>HUMAN</td><td>E9PI65</td><td>Heat shock cognate 71 kDa protein (Fragment)</td></tr>
</table>

<table>
<tr><td>2176</td><td>dirMS_041712_NAR_60mM_minus.15625.15806.3</td><td>3</td><td>16.01</td><td>1</td><td>6.80E+05</td><td></td><td>(R)FLQDYFI</td><td>(R)F/L|Q/D Y/F/D/G N L K R(Y)</td><td></td><td>147.7</td><td>505.924</td><td>1515.759</td><td>-0.0016</td><td>-1.1</td><td>5.96</td><td>55361.8</td><td>6.42</td><td>HUMAN</td><td>G5EA52</td><td>Protein disulfide isomerase family A, member 3, isoform CRA_b</td></tr>
<tr><td>2177</td><td>dirMS_041712_NAR_30mM_plus2.6193.6194.2</td><td>2</td><td>16.01</td><td>0</td><td>1.67E+05</td><td></td><td>(K)VQEQEL</td><td>(K)V/Q|E|Q/E/L/T/R(L)</td><td></td><td>86.78</td><td>501.7639</td><td>1002.521</td><td>-0.0009</td><td>-0.9</td><td>4.53</td><td>334221.3</td><td>6.44</td><td>HUMAN</td><td>P15924</td><td>Desmoplakin</td></tr>
<tr><td>2178</td><td>dirMS_041712_NAR_50mM_plus2.18292.18292.3</td><td>3</td><td>16.01</td><td>0</td><td>1.35E+06</td><td></td><td>(K)ALSAIAD</td><td>(K)A L/S\A/I/A D/L/L T N E H E R(V)</td><td></td><td>163.85</td><td>551.6254</td><td>1652.86</td><td>0.0014</td><td>0.9</td><td>4.65</td><td>105417.2</td><td>6.41</td><td>HUMAN</td><td>C9JZR2</td><td>Catenin delta-1</td></tr>
<tr><td>2179</td><td>dirMS_041712_NAR_120mM_minus.17700.18190.3</td><td>3</td><td>16</td><td>1</td><td>1.19E+06</td><td></td><td>(R)RAPFDLI</td><td>(R)R A\P\F|D|L|F E/N/R(K)</td><td></td><td>153.2</td><td>422.2193</td><td>1264.643</td><td>0.0001</td><td>0</td><td>6.07</td><td>85057.6</td><td>4.94</td><td>HUMAN</td><td>P07900</td><td>Heat shock protein HSP 90-alpha</td></tr>
<tr><td>2180</td><td>dirMS_041712_NAR_20mM_minus.13751.13751.2</td><td>2</td><td>16</td><td>0</td><td>3.99E+05</td><td></td><td>(K)LGGSAV</td><td>(K)L G\G S\A V|I/S L E G|K|P L(-)</td><td></td><td>188.68</td><td>670.8912</td><td>1340.778</td><td>-0.0033</td><td>-2.4</td><td>6</td><td>22956</td><td>8.8</td><td>HUMAN</td><td>E9PK25</td><td>Cofilin-1</td></tr>
<tr><td>2181</td><td>dirMS_041712_NAR_50mM_minus2.8266.8324.3</td><td>3</td><td>16</td><td>1</td><td>2.27E+05</td><td></td><td>(K)GILAADE</td><td>(K)G I|L/A/A/D E/S T/G S I/A/K R(L)</td><td></td><td>114.85</td><td>496.9372</td><td>1488.802</td><td>-0.0046</td><td>-3.1</td><td>6.07</td><td>30711.4</td><td>7.15</td><td>HUMAN</td><td>H3BPS8</td><td>Fructose-bisphosphate aldolase A (Fragment)</td></tr>
<tr><td>2182</td><td>dirMS_041712_NAR_120mM_plus.10877.11044.4</td><td>4</td><td>16</td><td>1</td><td>1.33E+06</td><td></td><td>(K)VVQHLG</td><td>(K)V/V Q H L G/P/A T/D/F/Y K K(L)</td><td></td><td>110.45</td><td>401.4712</td><td>1602.864</td><td>-0.0009</td><td>-0.6</td><td>8.48</td><td>141616.6</td><td>6.1</td><td>HUMAN</td><td>B7ZKM8</td><td>Protein transport protein Sec24B</td></tr>
<tr><td>2183</td><td>dirMS_041712_NAR_20mM_plus.9952.10195.2</td><td>2</td><td>16</td><td>0</td><td>1.43E+06</td><td></td><td>(K)LVSESSD</td><td>(K)L/V|S/E|S/S D\V L/P K(-)</td><td></td><td>143.17</td><td>587.3192</td><td>1173.636</td><td>-0.005</td><td>-4.3</td><td>4.37</td><td>56607.4</td><td>5.37</td><td>HUMAN</td><td>F8VXB4</td><td>Keratin, type II cytoskeletal 8</td></tr>
<tr><td>2184</td><td>dirMS_041712_NAR_30mM_plus2.12440.12602.2</td><td>2</td><td>16</td><td>0</td><td>9.27E+05</td><td></td><td>(R)YSGSYNI</td><td>(R)Y/S|G S|Y/N/D/Y/L/R(A)</td><td></td><td>119.1</td><td>619.2776</td><td>1237.548</td><td>-0.0005</td><td>-0.4</td><td>5.83</td><td>69661.6</td><td>9.75</td><td>HUMAN</td><td>Q96PK6</td><td>RNA-binding protein 14</td></tr>
<tr><td>2185</td><td>dirMS_041712_NAR_30mM_plus2.13174.13307.2</td><td>2</td><td>16</td><td>0</td><td>1.30E+06</td><td></td><td>(K)NDLAVV</td><td>(K)N/D|L|A/V/V/D/V/R(I)</td><td></td><td>121.68</td><td>500.7769</td><td>1000.542</td><td>0.0043</td><td>4.3</td><td>4.21</td><td>76670.1</td><td>4.6</td><td>HUMAN</td><td>P19338</td><td>Nucleolin</td></tr>
<tr><td>2186</td><td>dirMS_041712_NAR_40mM_plus2.12539.12559.3</td><td>3</td><td>16</td><td>0</td><td>3.96E+05</td><td></td><td>(K)AASDIAI</td><td>(K)A A|S/D I/A/M T/E/L/P P T H P I R(L)</td><td></td><td>140</td><td>607.3167</td><td>1819.937</td><td>-0.0015</td><td>-0.9</td><td>5.32</td><td>12926.1</td><td>4.4</td><td>HUMAN</td><td>K7EM20</td><td>14-3-3 protein epsilon (Fragment)</td></tr>
<tr><td>2187</td><td>dirMS_041712_NAR_50mM_plus2.13638.13732.3</td><td>3</td><td>16</td><td>0</td><td>1.81E+06</td><td></td><td>(K)DLAGCII</td><td>(K)D L|A/G/C/I H/G L S N V/K(L)</td><td>C:Carbamidomethylation</td><td>140.17</td><td>461.9121</td><td>1383.705</td><td>0.0168</td><td>12.2</td><td>6.74</td><td>45578.2</td><td>7.83</td><td>HUMAN</td><td>B4DFL2</td><td>Isocitrate dehydrogenase [NADP]</td></tr>
<tr><td>2188</td><td>dirMS_041712_NAR_50mM_plus2.18594.18814.3</td><td>3</td><td>16</td><td>1</td><td>9.79E+05</td><td></td><td>(R)LKLPSIPI</td><td>(R)L K L P S I|P\L|V|P V/S A Q K(R)</td><td></td><td>166.8</td><td>530.6702</td><td>1589.999</td><td>-0.0028</td><td>-1.8</td><td>10</td><td>34958.1</td><td>9.42</td><td>HUMAN</td><td>Q15785</td><td>Mitochondrial import receptor subunit TOM34</td></tr>
<tr><td>2189</td><td>dirMS_041712_NAR_60mM_plus.12229.12229.3</td><td>3</td><td>16</td><td>1</td><td>1.21E+06</td><td></td><td>(R)LNDSILC</td><td>(R)L/N/D/S|I/L Q A T E Q R R(R)</td><td></td><td>120.48</td><td>515.2771</td><td>1543.819</td><td>-0.0019</td><td>-1.3</td><td>6.07</td><td>334221.3</td><td>6.44</td><td>HUMAN</td><td>P15924</td><td>Desmoplakin</td></tr>
<tr><td>2190</td><td>dirMS_041712_NAR_120mM_minus.20169.20169.3</td><td>3</td><td>15.99</td><td>0</td><td>3.74E+05</td><td></td><td>(K)VHLVGII</td><td>(K)V H L\V G\I/D|I/F T/G/K(K)</td><td></td><td>166.4</td><td>433.5866</td><td>1298.747</td><td>-0.0015</td><td>-1.1</td><td>6.71</td><td>13048.7</td><td>6.42</td><td>HUMAN</td><td>C9J4W5</td><td>Eukaryotic translation initiation factor 5A-2 (Fragment)</td></tr>
<tr><td>2191</td><td>dirMS_041712_NAR_120mM_minus.5198.5198.3</td><td>3</td><td>15.99</td><td>1</td><td>3.17E+05</td><td></td><td>(R)LRAETEC</td><td>(R)L R\A E T\E|Q|G/E/Q/Q R(Q)</td><td></td><td>81.13</td><td>482.2432</td><td>1444.714</td><td>0.0012</td><td>0.8</td><td>4.79</td><td>533778</td><td>5.74</td><td>HUMAN</td><td>Q15149</td><td>Plectin</td></tr>
<tr><td>2192</td><td>dirMS_041712_NAR_50mM_minus2.13381.13509.3</td><td>3</td><td>15.99</td><td>0</td><td>2.46E+05</td><td></td><td>(K)VIQYLAY</td><td>(K)V/I/Q|Y/L/A|Y V/A/S S/H K(S)</td><td></td><td>144.78</td><td>493.6043</td><td>1478.8</td><td>-0.0018</td><td>-1.2</td><td>8.48</td><td>227783.5</td><td>5.5</td><td>HUMAN</td><td>P35579</td><td>Myosin-9</td></tr>
<tr><td>2193</td><td>dirMS_041712_NAR_20mM_plus.15506.15506.2</td><td>2</td><td>15.99</td><td>0</td><td>4.20E+05</td><td></td><td>(R)GVVDSE</td><td>(R)G/V\V D/S|E/D/I|P L/N/L\S R(E)</td><td></td><td>179.18</td><td>757.3956</td><td>1513.786</td><td>-0.0017</td><td>-1.1</td><td>4.03</td><td>74494.3</td><td>7.79</td><td>HUMAN</td><td>F5H897</td><td>Heat shock protein 75 kDa, mitochondrial</td></tr>
<tr><td>2194</td><td>dirMS_041712_NAR_20mM_plus.7264.7264.2</td><td>2</td><td>15.99</td><td>0</td><td>2.85E+04</td><td></td><td>(R)VLEEGS\</td><td>(R)V/L|E/E/G/S V E/A R(T)</td><td></td><td>118.03</td><td>544.7829</td><td>1088.558</td><td>0.0003</td><td>0.3</td><td>4.25</td><td>25834.8</td><td>9.54</td><td>HUMAN</td><td>Q96CT7</td><td>Coiled-coil domain-containing protein 124</td></tr>
<tr><td>2195</td><td>dirMS_041712_NAR_30mM_plus2.10023.10292.2</td><td>2</td><td>15.99</td><td>0</td><td>7.41E+05</td><td></td><td>(K)EQVANS</td><td>(K)E Q|V|A/N/S/A/F/V/E R(C)</td><td></td><td>106.63</td><td>625.311</td><td>1249.617</td><td>-0.0024</td><td>-1.9</td><td>4.53</td><td>17664</td><td>8.77</td><td>HUMAN</td><td>H0Y6E4</td><td>Heat shock protein HSP 90-beta (Fragment)</td></tr>
<tr><td>2196</td><td>dirMS_041712_NAR_30mM_plus2.7444.7474.2</td><td>2</td><td>15.99</td><td>0</td><td>5.37E+05</td><td></td><td>(K)TQLAVC</td><td>(K)T Q|L|A|V/C/Q/Q/R(I)</td><td>C:Carbamidomethylation</td><td>93.53</td><td>552.2842</td><td>1103.563</td><td>-0.0015</td><td>-1.3</td><td>9.41</td><td>9740.1</td><td>10.08</td><td>HUMAN</td><td>H0YFG2</td><td>Peptidyl-prolyl cis-trans isomerase FKBP4, N-terminally processed (Fragment)</td></tr>
<tr><td>2197</td><td>dirMS_041712_NAR_50mM_plus2.19891.20031.3</td><td>3</td><td>15.99</td><td>0</td><td>4.71E+05</td><td></td><td>(K)HSQFIG\</td><td>(K)H S Q\F\I|G\Y|P/I/T L/Y L E/K(E)</td><td></td><td>173.82</td><td>603.6541</td><td>1808.958</td><td>-0.0104</td><td>-5.7</td><td>6.75</td><td>83605</td><td>4.97</td><td>HUMAN</td><td>P08238</td><td>Heat shock protein HSP 90-beta</td></tr>
<tr><td>2198</td><td>dirMS_041712_NAR_60mM_plus.11216.11216.3</td><td>3</td><td>15.99</td><td>0</td><td>1.50E+06</td><td></td><td>(K)YIQQTKI</td><td>(K)Y/I|Q Q T/K/P L T/L E/R(T)</td><td></td><td>115.95</td><td>497.2818</td><td>1489.837</td><td>-0.0065</td><td>-4.3</td><td>8.59</td><td>26283.8</td><td>8.96</td><td>HUMAN</td><td>O43809</td><td>Cleavage and polyadenylation specificity factor subunit 5</td></tr>
<tr><td>2199</td><td>dirMS_041712_NAR_20mM_minus.12289.12537.2</td><td>2</td><td>15.98</td><td>0</td><td>5.88E+05</td><td>M73m</td><td>(R)AVLVDLI</td><td>(R)A V\L|V|D|L|E|P G T\m D S V R(S)</td><td>m:Oxidized methionine</td><td>172.52</td><td>809.4088</td><td>1601.82</td><td>15.99</td><td>-3</td><td>4.03</td><td>13112.4</td><td>5.23</td><td>HUMAN</td><td>F8W813</td><td>Tubulin beta-8 chain</td></tr>
<tr><td>2200</td><td>dirMS_041712_NAR_20mM_minus.14714.14884.2</td><td>2</td><td>15.98</td><td>0</td><td>3.01E+06</td><td></td><td>(K)EIIDLVLI</td><td>(K)E I|I/D|L/V/L/D/R(I)</td><td></td><td>203.67</td><td>543.3133</td><td>1085.62</td><td>-0.0008</td><td>-0.7</td><td>4.03</td><td>58642</td><td>4.95</td><td>HUMAN</td><td>F5H5D3</td><td>Tubulin alpha-1C chain</td></tr>
<tr><td>2201</td><td>dirMS_041712_NAR_50mM_minus2.9044.9044.3</td><td>3</td><td>15.98</td><td>0</td><td>7.04E+04</td><td></td><td>(R)EANALA</td><td>(R)E A\N\A/L/A A G H/P A Q A V A I N A R(L)</td><td></td><td>119.77</td><td>615.6611</td><td>1844.973</td><td>-0.0038</td><td>-2.1</td><td>6.85</td><td>272689.3</td><td>5.79</td><td>HUMAN</td><td>O15020</td><td>Spectrin beta chain, non-erythrocytic 2</td></tr>
<tr><td>2202</td><td>dirMS_041712_NAR_30mM_plus2.21319.21319.2</td><td>2</td><td>15.98</td><td>0</td><td>2.71E+05</td><td></td><td>(R)GVVQEL</td><td>(R)G V|V/Q/E|L/Q/Q A/I/S/K(L)</td><td></td><td>166.02</td><td>650.3632</td><td>1299.727</td><td>-0.0076</td><td>-5.8</td><td>6</td><td>22489.6</td><td>5.38</td><td>HUMAN</td><td>E9PI39</td><td>Elongation factor 1-delta (Fragment)</td></tr>
<tr><td>2203</td><td>dirMS_041712_NAR_30mM_plus2.6022.6025.2</td><td>2</td><td>15.98</td><td>0</td><td>2.12E+06</td><td>M300m</td><td>(K)NMmAA</td><td>(K)N/M|m|A|A/C\D|P R(H)</td><td>C:Carbamidomethylation m:Oxid</td><td>86.02</td><td>541.2163</td><td>1065.427</td><td>15.9979</td><td>2.8</td><td>5.84</td><td>50199.4</td><td>4.75</td><td>HUMAN</td><td>A6NNZ2</td><td>Tubulin beta-8 chain-like protein LOC260334</td></tr>
<tr><td>2204</td><td>dirMS_041712_NAR_50mM_plus2.13260.13285.3</td><td>3</td><td>15.98</td><td>0</td><td>2.96E+06</td><td></td><td>(K)LGDVYV</td><td>(K)L/G|D\V/Y V/N D/A/F G T A\H/R(A)</td><td></td><td>138.15</td><td>545.6029</td><td>1634.792</td><td>0.002</td><td>1.2</td><td>5.21</td><td>41827.5</td><td>7</td><td>HUMAN</td><td>B7Z7A9</td><td>Phosphoglycerate kinase</td></tr>
<tr><td>2205</td><td>dirMS_041712_NAR_50mM_plus2.20290.20606.3</td><td>3</td><td>15.98</td><td>1</td><td>4.12E+05</td><td></td><td>(R)VISGVLC</td><td>(R)V/I|S/G V/L Q/L/G N I V F/K/K(E)</td><td></td><td>176.1</td><td>539.0011</td><td>1614.994</td><td>-0.0054</td><td>-3.3</td><td>10</td><td>227783.5</td><td>5.5</td><td>HUMAN</td><td>P35579</td><td>Myosin-9</td></tr>
<tr><td>2206</td><td>dirMS_041712_NAR_120mM_minus.10135.10298.3</td><td>3</td><td>15.97</td><td>1</td><td>6.25E+05</td><td>M142m</td><td>(K)VKEGMI</td><td>(K)V K E G M N|I|V/E/A m E/R(F)</td><td>m:Oxidized methionine</td><td>110.08</td><td>507.9186</td><td>1505.745</td><td>15.9962</td><td>0.8</td><td>4.79</td><td>18240.4</td><td>7.96</td><td>HUMAN</td><td>P62937</td><td>Peptidyl-prolyl cis-trans isomerase A</td></tr>
<tr><td>2207</td><td>dirMS_041712_NAR_30mM_minus2.17317.17317.2</td><td>2</td><td>15.97</td><td>0</td><td>2.42E+05</td><td></td><td>(R)NGGQIL</td><td>(R)N/G\G Q|I/L I\A/D L R(G)</td><td></td><td>148.4</td><td>585.3352</td><td>1169.664</td><td>-0.0006</td><td>-0.5</td><td>5.84</td><td>34524.7</td><td>5.4</td><td>HUMAN</td><td>A8MV37</td><td>Protein SEC13 homolog</td></tr>
<tr><td>2208</td><td>dirMS_041712_NAR_20mM_plus.15054.15054.2</td><td>2</td><td>15.97</td><td>0</td><td>1.40E+05</td><td></td><td>(K)VLEQLT(</td><td>(K)V/L|E/Q\L|T/G/Q/T/P V/F\S K(A)</td><td></td><td>174.68</td><td>773.9244</td><td>1546.848</td><td>-0.006</td><td>-3.9</td><td>5.97</td><td>20480.2</td><td>9.91</td><td>HUMAN</td><td>P62913</td><td>60S ribosomal protein L11</td></tr>
<tr><td>2209</td><td>dirMS_041712_NAR_20mM_plus.16649.16649.2</td><td>2</td><td>15.97</td><td>0</td><td>1.58E+05</td><td></td><td>(R)LLEAQIA</td><td>(R)L/L/E\A/Q|I\A|T G G I I D|P K(E)</td><td></td><td>185.68</td><td>769.9362</td><td>1538.879</td><td>-0.0137</td><td>-8.9</td><td>4.37</td><td>334221.3</td><td>6.44</td><td>HUMAN</td><td>P15924</td><td>Desmoplakin</td></tr>
<tr><td>2210</td><td>dirMS_041712_NAR_20mM_plus.18757.18757.2</td><td>2</td><td>15.97</td><td>0</td><td>2.40E+05</td><td></td><td>(K)IYVDDGI</td><td>(K)I Y|V/D/D G/L/I/S L/Q V K(Q)</td><td></td><td>200.68</td><td>731.9068</td><td>1462.815</td><td>-0.0088</td><td>-6</td><td>4.21</td><td>30947.9</td><td>6.47</td><td>HUMAN</td><td>H3BQ34</td><td>Pyruvate kinase</td></tr>
<tr><td>2211</td><td>dirMS_041712_NAR_50mM_plus2.11270.11270.3</td><td>3</td><td>15.97</td><td>0</td><td>9.26E+05</td><td></td><td>(R)TFIAIKPI</td><td>(R)T F/I A I/K/P/D/G V Q R(G)</td><td></td><td>126.32</td><td>448.9241</td><td>1344.763</td><td>-0.0057</td><td>-4.2</td><td>8.41</td><td>6640.6</td><td>8.25</td><td>HUMAN</td><td>C9K028</td><td>Nucleoside diphosphate kinase A (Fragment)</td></tr>
<tr><td>2212</td><td>dirMS_041712_NAR_60mM_plus.16395.16772.3</td><td>3</td><td>15.97</td><td>0</td><td>1.39E+07</td><td></td><td>(R)EHALLA\</td><td>(R)E H\A\L\L|A|Y|T/L/G V/K(Q)</td><td></td><td>144.08</td><td>438.9179</td><td>1314.742</td><td>-0.0025</td><td>-1.9</td><td>6.85</td><td>50482.3</td><td>9.31</td><td>HUMAN</td><td>P68104</td><td>Elongation factor 1-alpha 1</td></tr>
<tr><td>2213</td><td>dirMS_041712_NAR_60mM_minus.15408.15408.3</td><td>3</td><td>15.96</td><td>0</td><td>1.03E+06</td><td></td><td>(R)LSDQFH</td><td>(R)L/S/D/Q/F/H/D I L I/R(K)</td><td></td><td>145.65</td><td>452.9148</td><td>1356.727</td><td>0.0028</td><td>2.1</td><td>5.21</td><td>12284.6</td><td>4.89</td><td>HUMAN</td><td>H0Y9X3</td><td>Programmed cell death protein 6 (Fragment)</td></tr>
<tr><td>2214</td><td>dirMS_041712_NAR_60mM_minus.9526.9526.3</td><td>3</td><td>15.96</td><td>0</td><td>3.33E+05</td><td></td><td>(R)QLSQAG</td><td>(R)Q\L/S Q/A/G/S F/S D/G T H G\G L/R(Y)</td><td></td><td>113.55</td><td>573.2781</td><td>1717.825</td><td>-0.0055</td><td>-3.2</td><td>6.74</td><td>266161</td><td>5.68</td><td>HUMAN</td><td>E9PPU0</td><td>Epiplakin</td></tr>
<tr><td>2215</td><td>dirMS_041712_NAR_120mM_plus.10867.11152.3</td><td>3</td><td>15.96</td><td>0</td><td>1.47E+06</td><td></td><td>(R)GSAVHC</td><td>(R)G S|A/V/H/Q/L/S/E E/L/R(C)</td><td></td><td>111.08</td><td>442.5657</td><td>1325.681</td><td>0.0017</td><td>1.3</td><td>5.4</td><td>558007.1</td><td>5.44</td><td>HUMAN</td><td>P58107</td><td>Epiplakin</td></tr>
<tr><td>2216</td><td>dirMS_041712_NAR_30mM_plus2.21716.21716.2</td><td>2</td><td>15.96</td><td>0</td><td>3.41E+05</td><td></td><td>(R)FSWNV\</td><td>(R)F/S\W/N/V W/P S S R(L)</td><td></td><td>169.22</td><td>633.3085</td><td>1265.606</td><td>0.0035</td><td>2.8</td><td>9.75</td><td>83880.6</td><td>7.33</td><td>HUMAN</td><td>F5H365</td><td>Protein transport protein Sec23A</td></tr>
<tr><td>2217</td><td>dirMS_041712_NAR_40mM_plus2.13540.13798.3</td><td>3</td><td>15.96</td><td>1</td><td>1.93E+06</td><td></td><td>(K)TVGVEP.</td><td>(K)T V\G|V/E/P A A D G K/G V V V V/I/K(R)</td><td></td><td>147.37</td><td>580.0077</td><td>1738.011</td><td>-0.0024</td><td>-1.4</td><td>5.73</td><td>7945.1</td><td>11.03</td><td>HUMAN</td><td>C9JB50</td><td>60S ribosomal protein L28</td></tr>
<tr><td>2218</td><td>dirMS_041712_NAR_120mM_minus.11894.12056.3</td><td>3</td><td>15.95</td><td>1</td><td>2.55E+06</td><td></td><td>(R)NLKASLI</td><td>(R)N L/K A S\L|E/N/S/L/R(E)</td><td></td><td>119.45</td><td>415.5698</td><td>1244.696</td><td>-0.0009</td><td>-0.7</td><td>8.75</td><td>43773.3</td><td>5.26</td><td>HUMAN</td><td>F8VZY9</td><td>Keratin, type I cytoskeletal 18</td></tr>
<tr><td>2219</td><td>dirMS_041712_NAR_120mM_minus.14729.14791.3</td><td>3</td><td>15.95</td><td>1</td><td>1.31E+06</td><td></td><td>(R)GYSFTTI</td><td>(R)G Y|S|F/T T/T A E/R E/I V/R(D)</td><td></td><td>134.73</td><td>543.9428</td><td>1629.823</td><td>-0.0093</td><td>-5.7</td><td>6.14</td><td>23901.9</td><td>5.28</td><td>HUMAN</td><td>I3L1U9</td><td>Actin, cytoplasmic 2, N-terminally processed (Fragment)</td></tr>
<tr><td>2220</td><td>dirMS_041712_NAR_120mM_minus.14793.14860.4</td><td>4</td><td>15.95</td><td>2</td><td>6.23E+05</td><td></td><td>(R)KLVIIESI</td><td>(R)K L V\I\I|E/S/D L/E R/A E/E/R(A)</td><td></td><td>134.88</td><td>450.7512</td><td>1799.986</td><td>-0.0032</td><td>-1.8</td><td>4.58</td><td>31923.6</td><td>4.85</td><td>HUMAN</td><td>B7Z596</td><td>Tropomyosin alpha-1 chain</td></tr>
<tr><td>2221</td><td>dirMS_041712_NAR_60mM_minus.13716.13716.3</td><td>3</td><td>15.95</td><td>0</td><td>3.78E+05</td><td></td><td>(-)MVNHFI.</td><td>(-)M/V|N H|F\I/A E/F K(R)</td><td></td><td>136.52</td><td>412.5461</td><td>1235.624</td><td>-0.0004</td><td>-0.3</td><td>6.5</td><td>45508.6</td><td>5.5</td><td>HUMAN</td><td>A8K7Q2</td><td>Heat shock cognate 71 kDa protein</td></tr>
<tr><td>2222</td><td>dirMS_041712_NAR_30mM_plus2.18895.19194.2</td><td>2</td><td>15.95</td><td>0</td><td>1.39E+06</td><td></td><td>(K)FIIPNVV</td><td>(K)F/I|I|P N V V/K(Y)</td><td></td><td>153.13</td><td>465.2943</td><td>929.582</td><td>-0.0005</td><td>-0.6</td><td>8.75</td><td>17529.3</td><td>9.3</td><td>HUMAN</td><td>F5GXY2</td><td>L-lactate dehydrogenase A chain (Fragment)</td></tr>
<tr><td>2223</td><td>dirMS_041712_NAR_60mM_plus.7086.7086.3</td><td>3</td><td>15.95</td><td>1</td><td>2.94E+05</td><td></td><td>(R)ARQENC</td><td>(R)A R Q\E N G\M|P E E/P/A/T T A R(G)</td><td></td><td>95.2</td><td>586.6118</td><td>1757.824</td><td>-0.0027</td><td>-1.5</td><td>4.79</td><td>64356.2</td><td>9.96</td><td>HUMAN</td><td>O96013</td><td>Serine/threonine-protein kinase PAK 4</td></tr>
<tr><td>2224</td><td>dirMS_041712_NAR_120mM_minus.13668.13984.3</td><td>3</td><td>15.94</td><td>0</td><td>2.04E+06</td><td></td><td>(R)NLDIERI</td><td>(R)N/L|D|I/E R/P T Y/T/N L N R(L)</td><td></td><td>129.73</td><td>573.6313</td><td>1718.882</td><td>-0.0027</td><td>-1.6</td><td>6.07</td><td>49012.3</td><td>4.9</td><td>HUMAN</td><td>A8MUB1</td><td>Tubulin alpha-4A chain</td></tr>
<tr><td>2225</td><td>dirMS_041712_NAR_30mM_minus2.11003.11003.2</td><td>2</td><td>15.94</td><td>0</td><td>5.91E+05</td><td></td><td>(K)VAGQD(</td><td>(K)V A|G/Q/D|G S/V V Q\F K(I)</td><td></td><td>113.82</td><td>617.824</td><td>1234.643</td><td>-0.0019</td><td>-1.5</td><td>5.81</td><td>17127.4</td><td>10.05</td><td>HUMAN</td><td>A8MU27</td><td>Small ubiquitin-related modifier 3</td></tr>
<tr><td>2226</td><td>dirMS_041712_NAR_50mM_minus2.10392.10392.3</td><td>3</td><td>15.94</td><td>0</td><td>3.38E+04</td><td></td><td>(R)GITINAA</td><td>(R)G I/T/I/N A A H/V/E/Y S T A/A R(H)</td><td></td><td>127.28</td><td>558.6229</td><td>1673.861</td><td>-0.0064</td><td>-3.8</td><td>6.75</td><td>49883</td><td>7.3</td><td>HUMAN</td><td>P49411</td><td>Elongation factor Tu, mitochondrial</td></tr>
<tr><td>2227</td><td>dirMS_041712_NAR_50mM_minus2.13471.13828.3</td><td>3</td><td>15.94</td><td>0</td><td>3.04E+05</td><td></td><td>(R)DLSHIGI</td><td>(R)D L S H\I G D\A\V|V|I/S/C/A K(D)</td><td>C:Carbamidomethylation</td><td>146.72</td><td>528.9397</td><td>1584.805</td><td>-0.0005</td><td>-0.3</td><td>5.21</td><td>29110.6</td><td>4.57</td><td>HUMAN</td><td>P12004</td><td>Proliferating cell nuclear antigen</td></tr>
<tr><td>2228</td><td>dirMS_041712_NAR_50mM_minus2.9510.9945.3</td><td>3</td><td>15.94</td><td>1</td><td>2.86E+06</td><td></td><td>(R)MQKEIT</td><td>(R)M Q|K E I T/A|L|A|P/S T M K(I)</td><td></td><td>123.52</td><td>516.9426</td><td>1548.812</td><td>0.0008</td><td>0.5</td><td>8.35</td><td>32616.5</td><td>5.18</td><td>HUMAN</td><td>A6NL76</td><td>Actin, alpha skeletal muscle</td></tr>
<tr><td>2229</td><td>dirMS_041712_NAR_20mM_plus.18207.18207.2</td><td>2</td><td>15.94</td><td>0</td><td>1.70E+05</td><td></td><td>(R)LTGQLFI</td><td>(R)L T|G/Q|L|F L\G/G S I/V\K(G)</td><td></td><td>197.02</td><td>666.8962</td><td>1332.789</td><td>-0.0034</td><td>-2.6</td><td>8.75</td><td>45924.5</td><td>5.29</td><td>HUMAN</td><td>B4E1F3</td><td>Selenium-binding protein 1</td></tr>
<tr><td>2230</td><td>dirMS_041712_NAR_30mM_plus2.12478.12478.2</td><td>2</td><td>15.94</td><td>0</td><td>5.26E+05</td><td></td><td>(R)LLVVTDI</td><td>(R)L L|V/V/T D/P R(A)</td><td></td><td>118.67</td><td>456.779</td><td>912.551</td><td>-0.0006</td><td>-0.6</td><td>5.84</td><td>33022.6</td><td>4.78</td><td>HUMAN</td><td>A6NE09</td><td>Protein RPSAP58</td></tr>
<tr><td>2231</td><td>dirMS_041712_NAR_40mM_plus2.13722.13925.3</td><td>3</td><td>15.94</td><td>1</td><td>2.67E+05</td><td></td><td>(R)GDVVPK</td><td>(R)G D|V|V|P/K/D\V N/A|A/I A T I K(T)</td><td></td><td>147.25</td><td>537.6381</td><td>1610.911</td><td>-0.0115</td><td>-7.1</td><td>5.96</td><td>58642</td><td>4.95</td><td>HUMAN</td><td>F5H5D3</td><td>Tubulin alpha-1C chain</td></tr>
<tr><td>2232</td><td>dirMS_041712_NAR_50mM_plus2.16309.16478.3</td><td>3</td><td>15.94</td><td>1</td><td>4.20E+06</td><td></td><td>(K)LLQDFFI</td><td>(K)L/L|Q/D|F/F/N G K E L N/K(S)</td><td></td><td>154.6</td><td>522.6137</td><td>1565.832</td><td>-0.0057</td><td>-3.6</td><td>6.07</td><td>45508.6</td><td>5.5</td><td>HUMAN</td><td>A8K7Q2</td><td>Heat shock cognate 71 kDa protein</td></tr>
<tr><td>2233</td><td>dirMS_041712_NAR_50mM_plus2.19803.19864.3</td><td>3</td><td>15.94</td><td>0</td><td>1.72E+05</td><td></td><td>(R)QLPAMT</td><td>(R)Q L|P/A/M T A/L/Q/T L/H/L R(S)</td><td></td><td>172.75</td><td>531.635</td><td>1592.894</td><td>-0.0037</td><td>-2.3</td><td>9.75</td><td>78827.3</td><td>5.92</td><td>HUMAN</td><td>J3KS54</td><td>Protein flightless-1 homolog</td></tr>
<tr><td>2234</td><td>dirMS_041712_NAR_120mM_minus.6321.6321.3</td><td>3</td><td>15.93</td><td>1</td><td>9.86E+05</td><td></td><td>(R)TEERAEI</td><td>(R)T|E/E/R A E/L/A E/S R(C)</td><td></td><td>88.93</td><td>430.8806</td><td>1290.628</td><td>-0.0012</td><td>-0.9</td><td>4.49</td><td>33450.2</td><td>4.73</td><td>HUMAN</td><td>J3KN67</td><td>Tropomyosin alpha-3 chain</td></tr>
<tr><td>2235</td><td>dirMS_041712_NAR_30mM_minus2.18072.18302.2</td><td>2</td><td>15.93</td><td>0</td><td>3.80E+05</td><td></td><td>(K)GLIPWE(</td><td>(K)G/L|I|P/W/E Q/A A R(L)</td><td></td><td>153.42</td><td>570.8106</td><td>1140.616</td><td>-0.0021</td><td>-1.8</td><td>6</td><td>266161</td><td>5.68</td><td>HUMAN</td><td>E9PPU0</td><td>Epiplakin</td></tr>
<tr><td>2236</td><td>dirMS_041712_NAR_50mM_minus2.14125.14125.3</td><td>3</td><td>15.93</td><td>1</td><td>1.24E+06</td><td></td><td>(R)LLDAQL.</td><td>(R)L/L/D/A/Q/L/A T G G L V C P A R R(L)</td><td>C:Carbamidomethylation</td><td>149.03</td><td>604.3359</td><td>1810.996</td><td>-0.0025</td><td>-1.4</td><td>9.6</td><td>266161</td><td>5.68</td><td>HUMAN</td><td>E9PPU0</td><td>Epiplakin</td></tr>
<tr><td>2237</td><td>dirMS_041712_NAR_40mM_plus2.11540.11540.3</td><td>3</td><td>15.93</td><td>0</td><td>3.79E+05</td><td></td><td>(R)MPLPAP</td><td>(R)M/P L/P/A/P S L S H Q|P/P P A P R(V)</td><td></td><td>133.63</td><td>598.3202</td><td>1792.953</td><td>-0.0066</td><td>-3.7</td><td>9.52</td><td>160853.1</td><td>6.11</td><td>HUMAN</td><td>F8VU51</td><td>YLP motif-containing protein 1</td></tr>
<tr><td>2238</td><td>dirMS_041712_NAR_60mM_plus.15620.15620.2</td><td>2</td><td>15.93</td><td>1</td><td>4.11E+05</td><td></td><td>(R)TEMENE</td><td>(R)T/E|M/E/N E/F V/L I/K K(D)</td><td></td><td>138.27</td><td>740.8875</td><td>1480.772</td><td>-0.0039</td><td>-2.6</td><td>4.78</td><td>13588.1</td><td>4.66</td><td>HUMAN</td><td>F8VP67</td><td>Keratin, type II cytoskeletal 8 (Fragment)</td></tr>
<tr><td>2239</td><td>dirMS_041712_NAR_20mM_minus.7692.7692.2</td><td>2</td><td>15.92</td><td>0</td><td>5.17E+05</td><td>M239m</td><td>(K)EVDEQm</td><td>(K)E V/D/E Q m L/N|V Q/N K(N)</td><td>m:Oxidized methionine</td><td>125.37</td><td>731.843</td><td>1446.689</td><td>15.9894</td><td>-3.8</td><td>4.14</td><td>40069.4</td><td>4.83</td><td>HUMAN</td><td>E7EWR1</td><td>Tubulin beta chain</td></tr>
<tr><td>2240</td><td>dirMS_041712_NAR_30mM_minus2.10941.11254.2</td><td>2</td><td>15.92</td><td>0</td><td>5.89E+05</td><td></td><td>(K)IVVVTA(</td><td>(K)I/V|V|V|T/A G V/R(Q)</td><td></td><td>114.07</td><td>457.2948</td><td>913.583</td><td>-0.0006</td><td>-0.7</td><td>9.75</td><td>25445.8</td><td>5.52</td><td>HUMAN</td><td>A8MW50</td><td>L-lactate dehydrogenase (Fragment)</td></tr>
<tr><td>2241</td><td>dirMS_041712_NAR_30mM_minus2.16670.16670.2</td><td>2</td><td>15.92</td><td>0</td><td>8.67E+04</td><td></td><td>(R)IMNTFS\</td><td>(R)I M/N\T|F/S V/V/P S/P K(V)</td><td></td><td>144.65</td><td>660.3534</td><td>1319.703</td><td>-0.0033</td><td>-2.5</td><td>8.75</td><td>42831.5</td><td>4.86</td><td>HUMAN</td><td>A8K854</td><td>HCG1983504, isoform CRA_f</td></tr>
<tr><td>2242</td><td>dirMS_041712_NAR_30mM_minus2.18263.18344.2</td><td>2</td><td>15.92</td><td>0</td><td>2.33E+05</td><td></td><td>(K)ALQFLEI</td><td>(K)A/L|Q/F/L E/E V/K(V)</td><td></td><td>153.82</td><td>538.7985</td><td>1076.599</td><td>-0.0089</td><td>-8.3</td><td>4.53</td><td>55323</td><td>6.02</td><td>HUMAN</td><td>B4DPJ8</td><td>T-complex protein 1 subunit zeta</td></tr>
<tr><td>2243</td><td>dirMS_041712_NAR_50mM_minus2.12749.12749.3</td><td>3</td><td>15.92</td><td>0</td><td>3.41E+06</td><td>M84m</td><td>(K)YPIEHGI</td><td>(K)Y/P/I E H G I I/T/N/W/D D m E K(I)</td><td>m:Oxidized methionine</td><td>141.47</td><td>659.6398</td><td>1960.911</td><td>15.9939</td><td>-0.5</td><td>4.31</td><td>32616.5</td><td>5.18</td><td>HUMAN</td><td>A6NL76</td><td>Actin, alpha skeletal muscle</td></tr>
<tr><td>2244</td><td>dirMS_041712_NAR_120mM_plus.21491.21594.4</td><td>4</td><td>15.92</td><td>2</td><td>4.53E+05</td><td>M145m</td><td>(R)RGVmL/</td><td>(R)R G V\m|L\A|V/D A V I/A/E L/K K(Q)</td><td>m:Oxidized methionine</td><td>166.22</td><td>433.0059</td><td>1713.009</td><td>15.9926</td><td>-1.3</td><td>8.59</td><td>25225.7</td><td>9.52</td><td>HUMAN</td><td>E7ESH4</td><td>60 kDa heat shock protein, mitochondrial (Fragment)</td></tr>
<tr><td>2245</td><td>dirMS_041712_NAR_50mM_plus2.17936.18222.3</td><td>3</td><td>15.92</td><td>1</td><td>1.81E+06</td><td></td><td>(R)ELIKVLEI</td><td>(R)E L\I K V L E E A/N/Q|A/I/N/P K(L)</td><td></td><td>163.53</td><td>603.6741</td><td>1809.012</td><td>-0.0039</td><td>-2.2</td><td>4.79</td><td>73202</td><td>9.19</td><td>HUMAN</td><td>C9JMU5</td><td>Probable ATP-dependent RNA helicase DDX17</td></tr>
<tr><td>2246</td><td>dirMS_041712_NAR_20mM_minus.10714.10714.2</td><td>2</td><td>15.91</td><td>0</td><td>1.92E+05</td><td></td><td>(R)TTPSYV/</td><td>(R)T T|P/S Y/V/A/F T D/T E R(L)</td><td></td><td>154.67</td><td>744.3547</td><td>1487.701</td><td>0.0009</td><td>0.6</td><td>4.37</td><td>58502.6</td><td>6.09</td><td>HUMAN</td><td>B4DXY3</td><td>Heat shock 70 kDa protein 1-like</td></tr>
<tr><td>2247</td><td>dirMS_041712_NAR_60mM_minus.7586.7630.3</td><td>3</td><td>15.91</td><td>1</td><td>6.66E+05</td><td></td><td>(K)IVADKD\</td><td>(K)I/V|A D/K/D Y/S/V T/A/N S K(I)</td><td></td><td>102.58</td><td>504.2624</td><td>1510.775</td><td>-0.0021</td><td>-1.4</td><td>5.96</td><td>25445.8</td><td>5.52</td><td>HUMAN</td><td>A8MW50</td><td>L-lactate dehydrogenase (Fragment)</td></tr>
<tr><td>2248</td><td>dirMS_041712_NAR_60mM_minus.9384.9401.3</td><td>3</td><td>15.91</td><td>1</td><td>2.76E+05</td><td>M198m</td><td>(R)VFDKEG</td><td>(R)V/F|D K E G N\G T|V/m/G A/E/I R(H)</td><td>m:Oxidized methionine</td><td>112.15</td><td>580.2853</td><td>1722.848</td><td>15.9934</td><td>-0.9</td><td>4.68</td><td>26991.7</td><td>5.01</td><td>HUMAN</td><td>B7Z6Z4</td><td>Myosin light polypeptide 6</td></tr>
<tr><td>2249</td><td>dirMS_041712_NAR_20mM_plus.10668.10668.2</td><td>2</td><td>15.91</td><td>0</td><td>6.90E+04</td><td>M61m</td><td>(K)NQVAm</td><td>(K)N Q/V|A/m|N/P T N T/V F D/A K(R)</td><td>m:Oxidized methionine</td><td>146.22</td><td>833.3977</td><td>1649.795</td><td>15.9929</td><td>-1.2</td><td>5.84</td><td>20069.4</td><td>6.74</td><td>HUMAN</td><td>E9PK54</td><td>Heat shock cognate 71 kDa protein (Fragment)</td></tr>
<tr><td>2250</td><td>dirMS_041712_NAR_40mM_plus2.15955.15955.3</td><td>3</td><td>15.91</td><td>0</td><td>2.95E+05</td><td></td><td>(K)VAIVKP(</td><td>(K)V/A\I/V K/P G|V|P/M E I V L N K(E)</td><td></td><td>160.92</td><td>569.6778</td><td>1707.024</td><td>-0.0049</td><td>-2.9</td><td>8.56</td><td>73146.9</td><td>5.45</td><td>HUMAN</td><td>E9PJ21</td><td>Hypoxia up-regulated protein 1 (Fragment)</td></tr>
<tr><td>2251</td><td>dirMS_041712_NAR_60mM_plus.7455.7477.3</td><td>3</td><td>15.91</td><td>1</td><td>5.05E+05</td><td></td><td>(K)SSGGREI</td><td>(K)S/S/G G R E D\L|E|S/S/G L Q|R(R)</td><td></td><td>97.3</td><td>526.5897</td><td>1577.751</td><td>0.0031</td><td>2</td><td>4.68</td><td>11625.5</td><td>8.01</td><td>HUMAN</td><td>B8ZZT7</td><td>Hematological and neurological-expressed 1 protein</td></tr>
<tr><td>2252</td><td>dirMS_041712_NAR_30mM_minus2.19043.19043.2</td><td>2</td><td>15.9</td><td>0</td><td>1.18E+06</td><td></td><td>(K)LAVNM\</td><td>(K)L A|V/N|M|V/P F P R(L)</td><td></td><td>159.03</td><td>572.3143</td><td>1143.634</td><td>-0.013</td><td>-11.4</td><td>9.75</td><td>42230.8</td><td>4.77</td><td>HUMAN</td><td>A6NKZ8</td><td>Putative tubulin beta chain-like protein ENSP00000290377</td></tr>
<tr><td>2253</td><td>dirMS_041712_NAR_50mM_minus2.15906.15906.3</td><td>3</td><td>15.9</td><td>0</td><td>3.64E+05</td><td></td><td>(K)LYGPTNI</td><td>(K)L/Y/G/P T N|F/S/P\I I N H V A R(F)</td><td></td><td>158.22</td><td>600.3221</td><td>1798.96</td><td>-0.0081</td><td>-4.5</td><td>8.75</td><td>60985.3</td><td>5.6</td><td>HUMAN</td><td>O75131</td><td>Copine-3</td></tr>
<tr><td>2254</td><td>dirMS_041712_NAR_60mM_minus.7330.7759.3</td><td>3</td><td>15.9</td><td>1</td><td>1.38E+06</td><td></td><td>(K)IQDKEGI</td><td>(K)I/Q\D K E G|I|P/P D/Q Q/R(L)</td><td></td><td>100.07</td><td>508.5978</td><td>1523.781</td><td>-0.0024</td><td>-1.6</td><td>4.56</td><td>17271.6</td><td>6.75</td><td>HUMAN</td><td>B4DV12</td><td>Ubiquitin</td></tr>
<tr><td>2255</td><td>dirMS_041712_NAR_20mM_plus.16379.16461.3</td><td>3</td><td>15.9</td><td>0</td><td>1.68E+05</td><td></td><td>(K)TVTNAV</td><td>(K)T V\T N A V\V T V/P/A/Y/F/N D S Q R(Q)</td><td></td><td>184.78</td><td>661.3368</td><td>1981.998</td><td>-0.0019</td><td>-1</td><td>5.5</td><td>17973</td><td>5.16</td><td>HUMAN</td><td>E9PI65</td><td>Heat shock cognate 71 kDa protein (Fragment)</td></tr>
<tr><td>2256</td><td>dirMS_041712_NAR_30mM_plus2.7530.7530.2</td><td>2</td><td>15.9</td><td>0</td><td>3.61E+05</td><td></td><td>(R)MNEAF(</td><td>(R)M/N|E|A/F/G D/T K(F)</td><td></td><td>93.75</td><td>506.7256</td><td>1012.44</td><td>0.0035</td><td>3.5</td><td>4.37</td><td>90316.8</td><td>5.52</td><td>HUMAN</td><td>Q13263</td><td>Transcription intermediary factor 1-beta</td></tr>
<tr><td>2257</td><td>dirMS_041712_NAR_40mM_plus2.16896.16918.3</td><td>3</td><td>15.9</td><td>0</td><td>8.98E+04</td><td></td><td>(R)LLEVLSG</td><td>(R)L/L|E|V\L S/G E/I/L/P K/P T K(G)</td><td></td><td>166</td><td>546.3323</td><td>1636.988</td><td>-0.006</td><td>-3.7</td><td>6.14</td><td>13611</td><td>6.13</td><td>HUMAN</td><td>E9PJZ2</td><td>Spectrin beta chain, non-erythrocytic 2 (Fragment)</td></tr>
<tr><td>2258</td><td>dirMS_041712_NAR_50mM_plus2.18484.18650.3</td><td>3</td><td>15.9</td><td>1</td><td>3.94E+05</td><td></td><td>(K)VCSTND</td><td>(K)V/C|S T N D/L K/E L L/I/F/N/K(Q)</td><td>C:Carbamidomethylation</td><td>166.02</td><td>598.6527</td><td>1793.947</td><td>-0.0031</td><td>-1.7</td><td>6.04</td><td>113880.5</td><td>9.18</td><td>HUMAN</td><td>P09874</td><td>Poly [ADP-ribose] polymerase 1</td></tr>
<tr><td>2259</td><td>dirMS_041712_NAR_60mM_plus.14465.14613.3</td><td>3</td><td>15.9</td><td>1</td><td>1.26E+06</td><td></td><td>(K)FSIEDLK.</td><td>(K)F/S|I/E/D/L K/A Q/P K(Q)</td><td></td><td>132.38</td><td>425.898</td><td>1275.694</td><td>-0.0149</td><td>-11.7</td><td>6.07</td><td>13579.2</td><td>9.99</td><td>HUMAN</td><td>E9PPQ6</td><td>Thymocyte nuclear protein 1 (Fragment)</td></tr>
<tr><td>2260</td><td>dirMS_041712_NAR_50mM_minus2.12922.12922.3</td><td>3</td><td>15.89</td><td>0</td><td>3.06E+05</td><td></td><td>(R)ETNLDSI</td><td>(R)E T N/L/D/S L/P/L/V/D T H S K(R)</td><td></td><td>142.27</td><td>556.9529</td><td>1668.844</td><td>0.0002</td><td>0.1</td><td>4.53</td><td>53707.8</td><td>5.06</td><td>HUMAN</td><td>P08670</td><td>Vimentin</td></tr>
<tr><td>2261</td><td>dirMS_041712_NAR_120mM_plus.11511.11511.3</td><td>3</td><td>15.89</td><td>2</td><td>7.36E+05</td><td></td><td>(K)QAGPAS</td><td>(K)Q A|G/P/A/S/V/P L R T E E E F K/K(F)</td><td></td><td>113.4</td><td>629.6702</td><td>1886.997</td><td>-0.001</td><td>-0.5</td><td>6.23</td><td>55361.8</td><td>6.42</td><td>HUMAN</td><td>G5EA52</td><td>Protein disulfide isomerase family A, member 3, isoform CRA_b</td></tr>
<tr><td>2262</td><td>dirMS_041712_NAR_30mM_plus2.21984.21984.2</td><td>2</td><td>15.89</td><td>0</td><td>2.09E+05</td><td></td><td>(K)QSLGELI</td><td>(K)Q S|L/G|E|L/I|G/T L N A/A K(V)</td><td></td><td>170.25</td><td>707.8889</td><td>1414.79</td><td>-0.0195</td><td>-13.8</td><td>6</td><td>31075.7</td><td>5.65</td><td>HUMAN</td><td>P60174</td><td>Triosephosphate isomerase</td></tr>
<tr><td>2263</td><td>dirMS_041712_NAR_30mM_plus2.22156.22156.2</td><td>2</td><td>15.89</td><td>0</td><td>2.10E+05</td><td></td><td>(K)NSSYFVI</td><td>(K)N S S Y/F/V/E/W/I|P N N V/K(V)</td><td></td><td>171.87</td><td>848.9189</td><td>1696.833</td><td>-0.0024</td><td>-1.4</td><td>6</td><td>42831.5</td><td>4.86</td><td>HUMAN</td><td>A8K854</td><td>HCG1983504, isoform CRA_f</td></tr>
<tr><td>2264</td><td>dirMS_041712_NAR_30mM_plus2.22577.22703.2</td><td>2</td><td>15.89</td><td>0</td><td>1.01E+06</td><td></td><td>(R)FLTWILN</td><td>(R)F/L|T|W I/L/N/R(Y)</td><td></td><td>175.88</td><td>531.8078</td><td>1062.61</td><td>-0.0012</td><td>-1.1</td><td>9.75</td><td>35548.6</td><td>4.91</td><td>HUMAN</td><td>B4DN60</td><td>Asparagine--tRNA ligase, cytoplasmic</td></tr>
<tr><td>2265</td><td>dirMS_041712_NAR_30mM_minus2.12598.12598.2</td><td>2</td><td>15.88</td><td>0</td><td>2.70E+05</td><td></td><td>(R)TLSGME</td><td>(R)T L|S/G M/E/S Y C V/R(A)</td><td>C:Carbamidomethylation</td><td>122.03</td><td>651.795</td><td>1302.582</td><td>0.001</td><td>0.8</td><td>5.66</td><td>42755</td><td>7.81</td><td>HUMAN</td><td>B7Z2F4</td><td>T-complex protein 1 subunit delta</td></tr>
<tr><td>2266</td><td>dirMS_041712_NAR_20mM_plus.13244.13665.2</td><td>2</td><td>15.88</td><td>0</td><td>8.91E+05</td><td></td><td>(R)VLQALE(</td><td>(R)V/L|Q/A/L/E|G L K(M)</td><td></td><td>164.68</td><td>485.8004</td><td>970.593</td><td>0.0004</td><td>0.4</td><td>5.97</td><td>16060.2</td><td>10.31</td><td>HUMAN</td><td>P39019</td><td>40S ribosomal protein S19</td></tr>
<tr><td>2267</td><td>dirMS_041712_NAR_30mM_plus2.11794.11794.2</td><td>2</td><td>15.88</td><td>0</td><td>4.85E+05</td><td></td><td>(R)AMGEQ.</td><td>(R)A M/G E|Q/A/V|A L A/R(A)</td><td></td><td>114.22</td><td>558.7944</td><td>1116.583</td><td>-0.0015</td><td>-1.3</td><td>6.05</td><td>80685.1</td><td>7.31</td><td>HUMAN</td><td>P05165</td><td>Propionyl-CoA carboxylase alpha chain, mitochondrial</td></tr>
<tr><td>2268</td><td>dirMS_041712_NAR_40mM_plus2.13130.13278.3</td><td>3</td><td>15.88</td><td>0</td><td>3.31E+05</td><td></td><td>(K)LQMEAI</td><td>(K)L/Q|M E A/P H/I/I/V/G T/P G R(V)</td><td></td><td>144.07</td><td>540.2954</td><td>1618.873</td><td>-0.0017</td><td>-1.1</td><td>6.75</td><td>20859.5</td><td>4.85</td><td>HUMAN</td><td>J3KS25</td><td>Eukaryotic initiation factor 4A-I (Fragment)</td></tr>
<tr><td>2269</td><td>dirMS_041712_NAR_50mM_plus2.19370.19370.3</td><td>3</td><td>15.88</td><td>0</td><td>3.55E+05</td><td></td><td>(-)MKPLVV</td><td>(-)M K P L V V F V|L/G/G/P G A G|K(G)</td><td></td><td>169.73</td><td>523.9715</td><td>1569.919</td><td>-0.0186</td><td>-11.8</td><td>10</td><td>22450.2</td><td>5.44</td><td>HUMAN</td><td>P30085</td><td>UMP-CMP kinase</td></tr>
<tr><td>2270</td><td>dirMS_041712_NAR_30mM_minus2.11584.11733.2</td><td>2</td><td>15.87</td><td>0</td><td>9.25E+05</td><td></td><td>(K)SSLGPV(</td><td>(K)S S|L/G|P V/G L/D K(M)</td><td></td><td>117.13</td><td>486.7751</td><td>972.536</td><td>0.0069</td><td>7.1</td><td>5.55</td><td>47423</td><td>5.43</td><td>HUMAN</td><td>E7ERF2</td><td>T-complex protein 1 subunit alpha</td></tr>
<tr><td>2271</td><td>dirMS_041712_NAR_30mM_minus2.11632.11667.2</td><td>2</td><td>15.87</td><td>0</td><td>5.59E+05</td><td>M64m</td><td>(K)VmYGF(</td><td>(K)V/m|Y|G/F/C S V/K(D)</td><td>C:Carbamidomethylation m:Oxid</td><td>117.33</td><td>553.7611</td><td>1090.506</td><td>16.0089</td><td>12.7</td><td>8.56</td><td>76925.9</td><td>4.46</td><td>HUMAN</td><td>A8MV58</td><td>Drebrin</td></tr>
<tr><td>2272</td><td>dirMS_041712_NAR_120mM_plus.9417.9417.4</td><td>4</td><td>15.87</td><td>1</td><td>2.90E+05</td><td></td><td>(K)MCKQDI</td><td>(K)M/C K Q D|P/S V L H/T E E|M/R(F)</td><td>C:Carbamidomethylation</td><td>102.68</td><td>465.966</td><td>1860.84</td><td>0.0021</td><td>1.1</td><td>5.43</td><td>4776.5</td><td>8.27</td><td>HUMAN</td><td>F8WAQ7</td><td>Hsc70-interacting protein</td></tr>
<tr><td>2273</td><td>dirMS_041712_NAR_20mM_plus.7900.7900.3</td><td>3</td><td>15.87</td><td>0</td><td>1.18E+05</td><td></td><td>(K)VEIIAND</td><td>(K)V E\I\I A/N|D/Q/G N/R(T)</td><td></td><td>124.5</td><td>410.2139</td><td>1228.628</td><td>-0.0009</td><td>-0.7</td><td>4.37</td><td>58502.6</td><td>6.09</td><td>HUMAN</td><td>B4DXY3</td><td>Heat shock 70 kDa protein 1-like</td></tr>
<tr><td>2274</td><td>dirMS_041712_NAR_30mM_plus2.19998.20177.2</td><td>2</td><td>15.87</td><td>0</td><td>2.43E+05</td><td></td><td>(K)LYNLFLK</td><td>(K)L/Y/N|L|F/L K(I)</td><td></td><td>159.32</td><td>455.7738</td><td>910.54</td><td>0.0007</td><td>0.7</td><td>8.59</td><td>41666</td><td>7.73</td><td>HUMAN</td><td>E9PDQ8</td><td>Succinyl-CoA ligase [GDP-forming] subunit beta, mitochondrial</td></tr>
<tr><td>2275</td><td>dirMS_041712_NAR_50mM_plus2.18773.18835.3</td><td>3</td><td>15.87</td><td>1</td><td>2.23E+05</td><td></td><td>(K)IGEEFLT</td><td>(K)I/G|E/E/F L/T/D L/S Q/L K/K(L)</td><td></td><td>167.02</td><td>540.965</td><td>1620.884</td><td>-0.0039</td><td>-2.4</td><td>4.68</td><td>97378.8</td><td>6.4</td><td>HUMAN</td><td>P11216</td><td>Glycogen phosphorylase, brain form</td></tr>
<tr><td>2276</td><td>dirMS_041712_NAR_30mM_minus2.18028.18028.2</td><td>2</td><td>15.86</td><td>0</td><td>4.12E+05</td><td></td><td>(R)LLLNNDI</td><td>(R)L L|L/N/N D N L/L/R(E)</td><td></td><td>153.13</td><td>599.3487</td><td>1197.695</td><td>-0.0049</td><td>-4.1</td><td>5.84</td><td>19945.8</td><td>4.97</td><td>HUMAN</td><td>B4DG50</td><td>F-actin-capping protein subunit alpha-2</td></tr>
<tr><td>2277</td><td>dirMS_041712_NAR_50mM_minus2.16852.16852.3</td><td>3</td><td>15.86</td><td>0</td><td>4.60E+05</td><td></td><td>(K)NILFVITI</td><td>(K)N\I/L/F/V/I/T K/P D V/Y K(S)</td><td></td><td>164.12</td><td>517.2991</td><td>1549.899</td><td>-0.0161</td><td>-10.4</td><td>8.5</td><td>206102.3</td><td>9.72</td><td>HUMAN</td><td>E9PAV3</td><td>Nascent polypeptide-associated complex subunit alpha</td></tr>
<tr><td>2278</td><td>dirMS_041712_NAR_20mM_plus.6022.6022.3</td><td>3</td><td>15.86</td><td>0</td><td>1.02E+06</td><td>M295m M</td><td>(R)mGQmA</td><td>(R)m G Q m A\m/G\G|A|m/G I N N R(G)</td><td>m:Oxidized methionine</td><td>100.27</td><td>534.8861</td><td>1538.67</td><td>63.9743</td><td>-3.4</td><td>9.5</td><td>43979.5</td><td>8.92</td><td>HUMAN</td><td>F5GYZ3</td><td>Non-POU domain-containing octamer-binding protein</td></tr>
<tr><td>2279</td><td>dirMS_041712_NAR_30mM_plus2.20250.20583.2</td><td>2</td><td>15.86</td><td>0</td><td>2.89E+05</td><td></td><td>(R)FLIQNVL</td><td>(R)F/L|I|Q N/V L/R(L)</td><td></td><td>160.97</td><td>501.8112</td><td>1002.61</td><td>0.0056</td><td>5.6</td><td>9.75</td><td>132902.3</td><td>5.82</td><td>HUMAN</td><td>J3KR24</td><td>Isoleucine--tRNA ligase, cytoplasmic</td></tr>
<tr><td>2280</td><td>dirMS_041712_NAR_30mM_plus2.21455.21862.2</td><td>2</td><td>15.86</td><td>0</td><td>8.57E+05</td><td></td><td>(R)LFFLQVI</td><td>(R)L/F|F|L/Q/V/K(E)</td><td></td><td>169.12</td><td>447.7756</td><td>894.545</td><td>-0.0008</td><td>-0.9</td><td>8.75</td><td>71218.8</td><td>6.3</td><td>HUMAN</td><td>A7YIJ8</td><td>Radixin</td></tr>
<tr><td>2281</td><td>dirMS_041712_NAR_50mM_plus2.15945.15978.3</td><td>3</td><td>15.86</td><td>1</td><td>7.30E+05</td><td></td><td>(R)LKLPAV\</td><td>(R)L K L P A\V|V|T|A/D/L/R(L)</td><td></td><td>152.03</td><td>432.6039</td><td>1295.805</td><td>-0.0074</td><td>-5.7</td><td>8.75</td><td>28071.3</td><td>8.6</td><td>HUMAN</td><td>P38117</td><td>Electron transfer flavoprotein subunit beta</td></tr>
<tr><td>2282</td><td>dirMS_041712_NAR_50mM_plus2.17493.17493.3</td><td>3</td><td>15.86</td><td>1</td><td>7.15E+05</td><td></td><td>(R)CITDPQ</td><td>(R)C/I|T|D/P Q\T G L C L/L P L K E K(K)</td><td>C:Carbamidomethylation</td><td>160.15</td><td>662.6825</td><td>1986.04</td><td>-0.0069</td><td>-3.5</td><td>6.07</td><td>533778</td><td>5.74</td><td>HUMAN</td><td>Q15149</td><td>Plectin</td></tr>
<tr><td>2283</td><td>dirMS_041712_NAR_50mM_plus2.19630.19630.3</td><td>3</td><td>15.86</td><td>1</td><td>7.20E+05</td><td></td><td>(R)VLEALLP</td><td>(R)V/L|E/A L L/P/L K G L E E R(V)</td><td></td><td>171.8</td><td>527.318</td><td>1579.942</td><td>-0.0023</td><td>-1.5</td><td>4.79</td><td>276046.1</td><td>6.01</td><td>HUMAN</td><td>P49327</td><td>Fatty acid synthase</td></tr>
<tr><td>2284</td><td>dirMS_041712_NAR_50mM_plus2.20180.20201.3</td><td>3</td><td>15.86</td><td>0</td><td>4.67E+05</td><td></td><td>(K)ALIEVLQ</td><td>(K)A L\I|E/V/L Q/P L/I/A/E H Q A R(R)</td><td></td><td>174.23</td><td>601.0191</td><td>1801.033</td><td>0.0097</td><td>5.4</td><td>5.4</td><td>53506.8</td><td>5.83</td><td>HUMAN</td><td>P23381</td><td>Tryptophan--tRNA ligase, cytoplasmic</td></tr>
</table>

2285	dirMS_041712_NAR_30mM_minus2.7747.7850.2	2	15.85	0	4.23E+05		(K)TDEGIA\	(K)T/D|E|G|I|A/Y/R(G)		96.7	462.7203	924.442	-0.0088	-9.5	4.37	22062.7	5.66	HUMAN	P32119	Peroxiredoxin-2
2286	dirMS_041712_NAR_60mM_minus.8979.8979.2	2	15.85	0	9.67E+05		(K)QEYDES	(K)Q\E|Y D E/S/G/P S I/V/H/R(K)		110.35	758.8539	1516.703	-0.0021	-1.4	4.65	123097.1	5.83	HUMAN	A5A3E0	POTE ankyrin domain family member F
2287	dirMS_041712_NAR_20mM_plus.8314.8361.2	2	15.85	0	1.23E+05		(K)SVEAAA	(K)S V|E|A|A/A/E/L/S A K(D)		127.72	538.2834	1075.563	-0.0035	-3.2	4.53	12131.5	11.14	HUMAN	F5GXR3	Parathymosin
2288	dirMS_041712_NAR_30mM_plus2.13303.13401.2	2	15.85	0	4.96E+05		(K)SITEGIE	(K)I/S|I/T/E/G I E/R(G)		123.28	509.2827	1017.558	0.0006	0.6	4.53	334221.3	6.44	HUMAN	P15924	Desmoplakin
2289	dirMS_041712_NAR_50mM_plus2.21947.22124.3	3	15.85	1	8.66E+06		(R)KEGGLG	(R)K\E G G L\G P\L\N\I|P|L/L/A/D V T R(R)		185.23	621.6925	1863.07	-0.0069	-3.7	6.07	15252.2	5.82	HUMAN	A6NIW5	Peroxiredoxin 2, isoform CRA_a
2290	dirMS_041712_NAR_60mM_plus.12175.12217.3	3	15.85	1	1.99E+06		(R)MQKEIT	(R)M Q K E I T/A|L/A/P S/T/M K(I)		120.7	516.9402	1548.812	-0.0064	-4.1	8.35	32616.5	5.18	HUMAN	A6NL76	Actin, alpha skeletal muscle
2291	dirMS_041712_NAR_60mM_plus.20058.20138.3	3	15.85	1	5.36E+05		(R)AKFEELN	(R)A K\F E E\L/N|M/D/L/F/R(S)		165.08	504.9258	1512.752	0.0113	7.5	4.68	72445.7	5.07	HUMAN	P11021	78 kDa glucose-regulated protein
2292	dirMS_041712_NAR_120mM_minus.13538.13538.4	4	15.84	1	7.85E+05		(K)SVHSSVI	(K)S V H S S V|P L/L/N S K D/P I D|R(I)		127.8	466.7525	1863.992	-0.0041	-2.2	6.47	54427.4	5.96	HUMAN	K4DID9	Acetyl-CoA carboxylase 1
2293	dirMS_041712_NAR_120mM_minus.7490.7512.3	3	15.84	1	2.23E+05		(R)KTGQAP	(R)K\T G Q A|P/G\Y|S|Y\T/A A/N K(N)		95.17	519.5935	1556.77	-0.0044	-2.8	9.53	11447.2	9.81	HUMAN	C9JFR7	Cytochrome c (Fragment)
2294	dirMS_041712_NAR_20mM_minus.13199.13274.3	3	15.84	0	4.00E+05		(K)TITLEVEI	(K)T\I\T L E\V E/P/S/D/T I/E N V\K(A)		183.03	596.6453	1787.927	-0.006	-3.3	4	17271.6	6.75	HUMAN	B4DV12	Ubiquitin
2295	dirMS_041712_NAR_30mM_minus2.16115.16115.2	2	15.84	0	1.16E+05		(K)EQFLDG	(K)E Q|F|L/D/G D G W T S R(W)		141.08	705.8174	1410.628	-0.0009	-0.6	4.03	28545.1	4.5	HUMAN	K7EJB9	Calreticulin (Fragment)
2296	dirMS_041712_NAR_30mM_minus2.7596.7596.2	2	15.84	0	1.75E+05		(K)YTQSNS	(K)Y/T|Q/S/N/S V C/Y/A/K(N)	C:Carbamidomethylation	95.73	660.7969	1320.589	-0.0024	-1.8	8.5	65036	6.27	HUMAN	E9PBU3	Phosphoribosylaminoimidazolecarboxamide formyltransferase
2297	dirMS_041712_NAR_50mM_minus2.13364.13364.3	3	15.84	1	1.91E+05		(K)TITLEVEI	(K)T I/T/L/E V E/P S/D/T I/E N V K A K(I)		144.63	663.0226	1987.059	-0.0061	-3.1	4.41	17271.6	6.75	HUMAN	B4DV12	Ubiquitin
2298	dirMS_041712_NAR_120mM_plus.10633.10837.3	3	15.84	1	1.21E+06		(R)QQEGFK	(R)Q Q/E/G F/K/G T/F/P/D/A R(E)		109.43	494.2435	1480.718	-0.002	-1.3	6.07	43979.5	8.92	HUMAN	F5GYZ3	Non-POU domain-containing octamer-binding protein
2299	dirMS_041712_NAR_30mM_plus2.13704.13761.2	2	15.84	0	2.11E+05		(R)VFNYNT	(R)V/F|N|Y/N/T L/E/R(V)		124.98	578.2929	1155.579	-0.0008	-0.7	5.97	99900.1	5.04	HUMAN	B4DZI8	Coatomer protein complex, subunit beta 2 (Beta prime), isoform CRA_b
2300	dirMS_041712_NAR_30mM_plus2.16477.16968.2	2	15.84	0	1.10E+06		(K)LIINSLYK	(K)L/I|I|N/S L/Y/K(N)		140.43	482.2971	963.587	-0.0004	-0.4	8.59	5582.4	4.81	HUMAN	F8W026	Endoplasmin (Fragment)
2301	dirMS_041712_NAR_40mM_plus2.19639.19639.3	3	15.84	1	1.67E+05		(K)LGFAGL	(K)L G|F/A/G L/V Q\E/I/S F/G T T K D K(M)		183.78	637.673	1911.022	-0.0178	-9.3	6.07	49982	5.86	HUMAN	B4DYD8	T-complex protein 1 subunit epsilon
2302	dirMS_041712_NAR_30mM_minus2.13012.13238.2	2	15.83	0	5.66E+05		(K)ALMDEV	(K)A/L|M/D/E/V/K(A)		125.2	452.7446	904.481	0.0011	1.2	4.37	41827.5	7	HUMAN	B7Z7A9	Phosphoglycerate kinase
2303	dirMS_041712_NAR_50mM_minus2.14460.14460.3	3	15.83	1	1.40E+05		(R)GDVVPK	(R)G D/V|V/P/K D\V/N A\A I/A T I K(T)		150.42	537.6375	1610.911	-0.0133	-8.2	5.96	58642	4.95	HUMAN	F5H5D3	Tubulin alpha-1C chain
2304	dirMS_041712_NAR_60mM_minus.9349.9350.3	3	15.83	1	1.46E+05		(R)KIQVLQ	(R)K\I Q\V L\Q\Q|Q|A/D/D/A/E\E R(A)		112	590.9696	1770.898	-0.0038	-2.2	4.32	11017	4.8	HUMAN	D6R904	Tropomyosin alpha-3 chain
2305	dirMS_041712_NAR_120mM_plus.17568.18101.3	3	15.83	1	2.34E+06		(R)RAPFDLI	(R)R\A\P\F\D\L\F E/N/R(K)		146.97	422.22	1264.643	0.0022	1.7	6.07	85057.6	4.94	HUMAN	P07900	Heat shock protein HSP 90-alpha
2306	dirMS_041712_NAR_20mM_plus.14264.14665.2	2	15.83	0	6.81E+05	M73m	(R)AVLVDLI	(R)A V|L|V|D|L|E|P G/T\m D S V R(S)	m:Oxidized methionine	171.03	809.4082	1601.82	15.9888	-3.8	4.03	13112.4	5.23	HUMAN	F8W813	Tubulin beta-8 chain
2307	dirMS_041712_NAR_20mM_plus.7429.7429.2	2	15.83	0	7.63E+04		(K)SNVSDA	(K)S N|V/S D A/V/A Q/S|T/R(I)		119.48	617.8019	1234.602	-0.0057	-4.6	5.55	31075.7	5.65	HUMAN	P60174	Triosephosphate isomerase
2308	dirMS_041712_NAR_40mM_plus2.11872.11872.3	3	15.83	0	4.24E+04		(K)AASAGV	(K)A A|A S A/G/V/P Y/H G E V/P V S L A R(A)		136.07	594.314	1780.934	-0.0066	-3.7	6.79	272689.3	5.79	HUMAN	O15020	Spectrin beta chain, non-erythrocytic 2
2309	dirMS_041712_NAR_40mM_plus2.15562.15562.3	3	15.83	1	7.33E+04		(K)VLDSGA	(K)V L|D\S/G A/P I K/I|P V G/P E T L/G R(I)		158.12	640.3668	1919.096	-0.0102	-5.3	6.04	55307.5	5.25	HUMAN	F8VPV9	ATP synthase subunit beta
2310	dirMS_041712_NAR_20mM_minus.8145.8281.2	2	15.82	0	6.77E+06	M239m	(K)EITALAP	(K)E I|T|A|L|A/P/S T m K(I)	m:Oxidized methionine	132.6	589.3094	1161.618	15.9931	-1.5	6.1	32616.5	5.18	HUMAN	A6NL76	Actin, alpha skeletal muscle
2311	dirMS_041712_NAR_30mM_minus2.8095.8095.2	2	15.82	0	1.83E+05		(K)GSGTAE	(K)G S|G T|A/E V/E/L K(K)		98.32	495.7528	990.51	-0.0119	-12	4.53	30947.9	6.47	HUMAN	H3BQ34	Pyruvate kinase
2312	dirMS_041712_NAR_40mM_minus2.14317.14317.3	3	15.82	1	1.61E+05		(K)TGISDVF	(K)T G/I/S D/V F A K\N D L/A/V V D/V R(I)		168.48	640.3454	1919.023	-0.0016	-0.8	4.43	76670.1	4.6	HUMAN	P19338	Nucleolin
2313	dirMS_041712_NAR_60mM_minus.9737.9737.2	2	15.82	1	4.41E+05		(R)LQAEIEC	(R)L/Q|A|E|I/E G L K/G Q R(A)		114.13	671.3768	1341.748	-0.0022	-1.6	6.14	56607.4	5.37	HUMAN	F8VXB4	Keratin, type II cytoskeletal 8
2314	dirMS_041712_NAR_20mM_plus.18728.18728.3	3	15.82	0	5.22E+05		(K)LAVEALS	(K)L A V E A L\S\S/L/D/G D/L/A/G R(Y)		200.47	529.6164	1586.838	-0.0038	-2.4	4.03	24286.7	4.89	HUMAN	G3V4N7	Creatine kinase B-type (Fragment)
2315	dirMS_041712_NAR_40mM_plus2.15332.15332.3	3	15.82	0	6.25E+05		(K)IEVIKPG	(K)I/E|V I/K P/G D\L G V D L T S K(L)		156.2	561.9876	1683.953	-0.0045	-2.7	4.56	28071.3	8.6	HUMAN	P38117	Electron transfer flavoprotein subunit beta
2316	dirMS_041712_NAR_30mM_minus2.18391.18795.2	2	15.81	0	1.87E+06		(R)DLTDYLI	(R)D L|T|D/Y/L/M/K(I)		155.93	499.7499	998.486	0.0062	6.2	4.21	37482.1	5.36	HUMAN	E9PG30	Actin, gamma-enteric smooth muscle
2317	dirMS_041712_NAR_50mM_minus2.13141.13141.3	3	15.81	0	3.75E+05		(K)VTHAVV	(K)V T H A\V V\T\V/P\A/Y/F/N D A Q R(Q)		143.35	629.9954	1887.971	0.0005	0.3	6.71	72445.7	5.07	HUMAN	P11021	78 kDa glucose-regulated protein
2318	dirMS_041712_NAR_30mM_plus2.7037.7179.2	2	15.81	0	2.83E+05		(K)ALLQSSA	(K)A/L|L|Q/S/S\A/S/R(K)		91.82	466.7618	932.516	0.0004	0.4	9.79	44128.4	6.13	HUMAN	Q9UQ80	Proliferation-associated protein 2G4
2319	dirMS_041712_NAR_20mM_plus2.9432.9555.2	2	15.81	0	8.52E+04		(K)VCNPIIT	(K)V/C|N/P I/I/T/K(L)	C:Carbamidomethylation	104.22	472.7711	944.523	0.0116	12.2	8.72	45508.6	5.5	HUMAN	A8K7Q2	Heat shock cognate 71 kDa protein
2320	dirMS_041712_NAR_40mM_plus2.18885.18885.3	3	15.81	1	4.04E+05		(R)TLAEIAK	(R)T L/A/E I/A K V|E L/D N M/P L R(G)		176.78	604.9967	1812.989	-0.0132	-7.3	4.68	23729.5	9.89	HUMAN	C9JZL7	Non-POU domain-containing octamer-binding protein (Fragment)
2321	dirMS_041712_NAR_30mM_minus2.10166.10166.2	2	15.8	0	5.85E+05		(K)FGVQMI	(K)F/G/V/Q M D/R(V)		109.88	426.7012	852.403	-0.0081	-9.5	5.84	25153.2	6.6	HUMAN	H7C314	Src substrate cortactin (Fragment)
2322	dirMS_041712_NAR_30mM_minus2.18343.18448.2	2	15.8	0	4.47E+05		(R)QAQEYE	(R)Q A|Q/E/Y/E/A/L/L/N I K(V)		154.67	710.3759	1419.748	-0.0033	-2.3	4.53	43773.3	5.26	HUMAN	F8VZY9	Keratin, type I cytoskeletal 18
2323	dirMS_041712_NAR_30mM_minus2.7754.7754.2	2	15.8	0	1.29E+05		(K)VTQVDC	(K)V/T|Q/V/D/G N/S P V R(F)		96.45	586.3085	1171.607	0.0031	2.7	5.81	62195.8	5.63	HUMAN	B0YIW6	Archain 1, isoform CRA_a
2324	dirMS_041712_NAR_40mM_minus2.13124.13144.3	3	15.8	1	9.20E+04		(K)VLDSGA	(K)V/L|D|S/G A/P I K I/P/V G/P E T L G R(I)		160.87	640.3618	1919.096	-0.0252	-13.1	6.04	55307.5	5.25	HUMAN	F8VPV9	ATP synthase subunit beta
2325	dirMS_041712_NAR_50mM_minus2.11257.11257.3	3	15.8	0	1.44E+06		(R)NLDIERF	(R)N/L|D|I/E R P T Y T N L N R(L)		131.95	573.6313	1718.882	-0.0027	-1.6	6.07	49012.3	4.9	HUMAN	A8MUB1	Tubulin alpha-4A chain
2326	dirMS_041712_NAR_60mM_minus.15159.15159.2	2	15.8	0	4.20E+05		(R)EHALLA\	(R)E H|A/L/L/A Y/T\L G V K(Q)		144.65	657.873	1314.742	-0.0029	-2.2	6.85	50482.3	9.31	HUMAN	P68104	Elongation factor 1-alpha 1
2327	dirMS_041712_NAR_60mM_minus.17181.17181.3	3	15.8	1	6.23E+05		(K)EVNKFQ	(K)E V/N K F Q M/A/Y/S/N/L/L R(A)		155.47	571.6279	1712.879	-0.0097	-5.7	8.69	14683.8	10.19	HUMAN	P37108	Signal recognition particle 14 kDa protein
2328	dirMS_041712_NAR_120mM_plus.21471.21538.3	3	15.8	1	2.33E+05		(R)GLFIIDD	(R)G L|F/I/I/D/D K/G I L R(Q)		165.98	453.9377	1359.8	-0.0009	-0.7	5.96	18498.6	5.82	HUMAN	H7C3T4	Peroxiredoxin-4 (Fragment)
2329	dirMS_041712_NAR_20mM_plus.17520.17520.2	2	15.8	0	3.08E+05		(K)SYELPDC	(K)S Y\E|L|P D G Q V/I|T I/G N E R(F)		193.17	895.9492	1790.892	-0.0008	-0.4	4.14	123097.1	5.83	HUMAN	A5A3E0	POTE ankyrin domain family member F
2330	dirMS_041712_NAR_60mM_plus.7439.7530.3	3	15.8	0	5.01E+05		(R)SSGHSS!	(R)S/S G H S S S\E|L|S|P D A/V/E/K(A)		96.95	539.584	1616.74	-0.0024	-1.5	4.65	227779.5	12.08	HUMAN	I3L2L6	Serine/arginine repetitive matrix protein 2
2331	dirMS_041712_NAR_60mM_plus.9511.9878.3	3	15.8	1	1.21E+06		(K)ILVANR(	(K)I/L|V/A/N R/G E I/A C/R(V)	C:Carbamidomethylation	107.78	457.9222	1371.753	-0.0005	-0.4	9.6	80685.1	7.31	HUMAN	P05165	Propionyl-CoA carboxylase alpha chain, mitochondrial
2332	dirMS_041712_NAR_120mM_minus.8006.8101.2	2	15.79	0	5.03E+05		(K)GHPAPI	(K)I/G/H|P/A/P N\F/K(A)		98.13	490.7694	980.531	0.0003	0.3	8.76	22338.2	8.65	HUMAN	Q06830	Peroxiredoxin-1
2333	dirMS_041712_NAR_20mM_minus.10350.10350.2	2	15.79	0	4.24E+04		(K)AVLSLYA	(K)A V/L|S L/Y/A S G/R(T)		152.4	518.7929	1036.579	0	0	8.79	14579.3	5.13	HUMAN	K7EM38	Actin, cytoplasmic 2, N-terminally processed (Fragment)
2334	dirMS_041712_NAR_50mM_minus2.10395.10395.3	3	15.79	0	7.23E+04		(R)LTSPIVS	(R)L\T|S/P I/V/S/T/H L D/T R(N)		127.37	480.6	1439.785	0.0002	0.1	6.74	58760.2	5.26	HUMAN	Q6ZTN6	Ankyrin repeat domain-containing protein 13D
2335	dirMS_041712_NAR_50mM_minus2.10568.10568.3	3	15.79	0	1.60E+05		(R)LLELGPK	(R)L L|E|L/G P K/P E V A|Q Q T R(K)		128.52	560.3182	1678.949	-0.0086	-5.1	6.14	139883.7	8.06	HUMAN	P53621	Coatomer subunit alpha
2336	dirMS_041712_NAR_20mM_plus.16611.16611.2	2	15.79	0	1.91E+05		(K)VQIAVA	(K)V Q|I/A/V/A/N A Q E L/L\Q|R(M)		186.22	776.9419	1552.881	-0.004	-2.6	5.97	10728.1	9.15	HUMAN	Q9Y5L4	Mitochondrial import inner membrane translocase subunit Tim13
2337	dirMS_041712_NAR_30mM_plus2.12388.12908.2	2	15.79	0	2.48E+06		(R)LQIDEQI	(R)L/Q|I|D/E/Q L/R(Q)		118.98	507.7822	1014.558	-0.0007	-0.7	4.37	66105.7	7.3	HUMAN	A8MQB8	Fragile X mental retardation protein 1
2338	dirMS_041712_NAR_30mM_plus2.21157.21398.2	2	15.79	0	2.85E+05		(R)FDAMPF	(R)F/D|A|M|P/F/T L/R(A)		166.32	549.276	1097.545	-0.0001	-0.1	5.84	42088	5.39	HUMAN	F8VTY8	Proliferation-associated protein 2G4
2339	dirMS_041712_NAR_50mM_plus2.14381.14403.3	3	15.79	0	5.99E+05		(R)AVAIDLF	(R)A V|A I/D L/P/G L G H S K(E)		143.8	426.5786	1277.721	0	0	6.79	19983.6	8.14	HUMAN	B4DQI4	Alpha/beta hydrolase domain-containing protein 14B
2340	dirMS_041712_NAR_50mM_plus2.15238.15238.3	3	15.79	1	6.82E+05		(K)TVGVEP	(K)T V|G V/E/P A A D/G K G V V V V I K(R)		147.9	580.0056	1738.011	-0.0087	-5	5.73	7945.1	11.03	HUMAN	C9JB50	60S ribosomal protein L28
2341	dirMS_041712_NAR_50mM_plus2.17722.17872.3	3	15.79	0	8.69E+05		(K)NILFVTH	(K)N I|I|L/F/V/I/T K/P D V Y K(S)		161.55	517.3015	1549.899	-0.0089	-5.7	8.5	206112.9	9.72	HUMAN	E9PAV3	Nascent polypeptide-associated complex subunit alpha
2342	dirMS_041712_NAR_60mM_plus.14375.14425.3	3	15.79	1	8.33E+05		(R)TIRYPDF	(R)T/I/R|Y P D|P/L/I/K(V)		131.87	405.9078	1215.71	-0.0007	-0.6	8.25	29825.4	10.37	HUMAN	P62701	40S ribosomal protein S4, X isoform
2343	dirMS_041712_NAR_20mM_plus.7173.7173.2	2	15.78	0	3.06E+05	M532m M!	(R)mVPAGI	(R)m V|P/A/G m G A G L E R(M)	m:Oxidized methionine	117.37	610.7907	1188.586	31.9877	-1.7	5.75	77799.7	9.09	HUMAN	P52272	Heterogeneous nuclear ribonucleoprotein M
2344	dirMS_041712_NAR_30mM_plus2.10513.10513.2	2	15.78	0	1.78E+05		(R)DGYDYC	(R)D G Y/D/Y/D/G Y R(L)		108.43	562.2211	1123.433	0.0022	2	3.93	28442.8	10.22	HUMAN	J3KTL2	Serine/arginine-rich-splicing factor 1
2345	dirMS_041712_NAR_30mM_plus2.14272.14272.2	2	15.78	0	2.49E+05		(R)IYGFYDE	(R)I/Y|G F Y D E/C K(R)	C:Carbamidomethylation	127.52	597.761	1194.514	0.0011	1	4.37	33222.3	5.74	HUMAN	A6NNR3	Serine/threonine-protein phosphatase
2346	dirMS_041712_NAR_50mM_plus2.13401.13526.3	3	15.78	0	1.54E+06		(R)VLVVHD	(R)V/L|V/V/H/D G F E G L A/K(G)		139.83	461.9229	1383.763	-0.0089	-6.5	5.32	18719.9	6.91	HUMAN	F8VP00	6-phosphofructokinase, muscle type (Fragment)
2347	dirMS_041712_NAR_40mM_minus2.13615.13753.3	3	15.77	1	3.98E+05		(R)VTFSGLI	(R)V T|F/S/G L R D T\V/T/P G E L L K(A)		164.73	611.6742	1833.012	-0.0036	-2	6.04	266161	5.68	HUMAN	E9PPU0	Epiplakin
2348	dirMS_041712_NAR_50mM_minus2.12436.12436.3	3	15.77	0	2.71E+05		(K)AASDIAI	(K)A A/S D I/A/M/T E L/P/P T H/P I R(L)		139.15	607.3158	1819.937	-0.0042	-2.3	5.32	12926.1	4.4	HUMAN	K7EM20	14-3-3 protein epsilon (Fragment)
2349	dirMS_041712_NAR_50mM_minus2.15419.15419.3	3	15.77	0	3.28E+05		(R)LSFQHD	(R)L/S/F Q H D\P\E\T\S/V/L/V L/R(K)		156.07	580.9793	1740.928	-0.0046	-2.6	5.32	96827.3	5.58	HUMAN	E9PKU7	Neutral alpha-glucosidase AB
2350	dirMS_041712_NAR_30mM_plus2.15018.15018.2	2	15.77	0	7.43E+05		(R)LSLYQAI	(R)L/S|L/Y/Q A/M R(Q)		131.28	491.2638	981.519	0.0017	1.7	8.75	266161	5.68	HUMAN	E9PPU0	Epiplakin
2351	dirMS_041712_NAR_50mM_plus2.10370.10371.3	3	15.77	1	2.76E+05		(K)IQVLQQ	(K)I/Q|V|L/Q/Q/Q/A D/D/A E E R A E R(L)		121.95	666.999	1998.984	-0.0015	-0.7	4.18	26611.3	4.47	HUMAN	A6NL28	Putative tropomyosin alpha-3 chain-like protein
2352	dirMS_041712_NAR_50mM_plus2.11351.11370.3	3	15.77	0	5.94E+05		(R)ITLRPDF	(R)I/T/L/R P D|P\L/P E/R(S)		127.35	436.2535	1306.748	-0.0018	-1.4	6.07	160853.1	6.11	HUMAN	F8VU51	YLP motif-containing protein 1
2353	dirMS_041712_NAR_50mM_plus2.13324.13476.3	3	15.77	0	9.05E+05		(R)TVVTGIE	(R)T/V|V/T/G I/E M/F/H K(S)		138.55	421.225	1261.661	-0.0005	-0.4	6.41	49883	7.3	HUMAN	P49411	Elongation factor Tu, mitochondrial
2354	dirMS_041712_NAR_60mM_plus.8093.8235.3	3	15.77	1	6.15E+05		(R)KQSTDE	(R)K\Q S T D\E|E\V/T|S L/A/K(S)		100.87	479.2451	1434.728	-0.0037	-2.6	4.68	12611.4	8.8	HUMAN	E5RIS7	Transcription elongation factor A protein 1
2355	dirMS_041712_NAR_30mM_minus2.16414.16851.2	2	15.76	0	3.42E+05		(R)ASLEAAI	(R)A S/L/E/A|A|I/D/A/E Q R(G)		144.08	672.8404	1344.675	-0.0019	-1.4	4.14	56607.4	5.37	HUMAN	F8VXB4	Keratin, type II cytoskeletal 8
2356	dirMS_041712_NAR_50mM_minus2.13489.13719.3	3	15.76	0	3.04E+05		(R)DLSHIGI	(R)D L S H\\G D\A\V|V|I/S/C/A K(D)	C:Carbamidomethylation	146.72	528.9397	1584.805	-0.0005	-0.3	5.21	29110.6	4.57	HUMAN	P12004	Proliferating cell nuclear antigen
2357	dirMS_041712_NAR_40mM_plus2.17300.17300.3	3	15.76	1	1.93E+05		(R)GDSVIV\	(R)G D/S/V/I|V V L R N/P L I A\G K(-)		168.72	551.0007	1650.99	-0.0026	-1.6	8.75	6144.3	10.23	HUMAN	K7EJB5	Small nuclear ribonucleoprotein Sm D2
2358	dirMS_041712_NAR_120mM_minus.13514.13514.3	3	15.75	2	9.83E+05		(R)AQKDEE	(R)A Q/K/D E E K|M E/I/Q\E I Q\L K(E)		128.13	654.0058	1960.006	-0.0027	-1.4	4.58	31923.6	4.85	HUMAN	B7Z596	Tropomyosin alpha-1 chain
2359	dirMS_041712_NAR_30mM_plus2.17161.17161.2	2	15.75	0	2.76E+05		(R)LLNTFLE	(R)L/L|N/T/F L E/R(L)		146.9	503.2871	1005.573	-0.0058	-5.8	6	535136.6	6.01	HUMAN	Q14204	Cytoplasmic dynein 1 heavy chain 1
2360	dirMS_041712_NAR_20mM_minus.10632.10662.3	3	15.75	1	1.21E+05		(R)VLTVINC	(R)V/L|T/V/I/N Q T Q K/E/N L R(K)		119.27	552.6501	1655.944	-0.0081	-4.9	8.72	10815.7	10.72	HUMAN	F2Z388	60S ribosomal protein L35
2361	dirMS_041712_NAR_60mM_minus.7060.7224.3	3	15.75	1	3.04E+05		(R)ELFESST	(R)E L|F/E/S S/T N N N K D/T/K(Q)		98.72	542.9247	1626.761	-0.001	-0.6	4.68	150202.8	9.1	HUMAN	E7EW20	Unconventional myosin-VI
2362	dirMS_041712_NAR_120mM_plus.5514.5553.3	3	15.75	1	6.20E+05		(R)GGGYG(	(R)G G|G|Y/G/G D R\G/G G Y/G G D/R(G)		82.03	486.5431	1457.615	-0.0005	-0.3	5.96	21563.9	10.01	HUMAN	K7EKB2	TATA-binding protein-associated factor 2N (Fragment)
2363	dirMS_041712_NAR_30mM_plus2.12278.12542.2	2	15.75	0	1.00E+06		(R)SGYLLPE	(R)S G/Y|L|L|P D/T/K(A)		117.57	497.2641	993.525	-0.0042	-4.2	5.55	17045.4	4.25	HUMAN	H0YIV0	Endoplasmin (Fragment)
2364	dirMS_041712_NAR_40mM_plus2.8220.8270.2	2	15.75	0	2.86E+05		(R)FGSGMI	(R)F/G/S/G/M N/M|G/R(I)		109.45	478.706	956.408	-0.003	-3.1	9.75	77799.7	9.09	HUMAN	P52272	Heterogeneous nuclear ribonucleoprotein M
2365	dirMS_041712_NAR_60mM_plus.11936.11949.3	3	15.75	1	3.80E+05		(R)VTVAGL	(R)V/T|V/A/G/L/A/G K/D/P V Q C S R(D)	C:Carbamidomethylation	119.65	553.2934	1657.869	-0.0034	-2	8.72	18080.7	7.92	HUMAN	K7ELW0	Protein DJ-1
2366	dirMS_041712_NAR_60mM_plus.16486.16486.2	2	15.75	0	6.93E+05		(R)EHALLA\	(R)E/H|A/L|L/A Y/T/L G V K(Q)		143.63	657.8735	1314.742	-0.0019	-1.4	6.85	50482.3	9.31	HUMAN	P68104	Elongation factor 1-alpha 1
2367	dirMS_041712_NAR_20mM_minus.11225.11468.2	2	15.74	0	1.17E+07	M148m	(R)DLTDYL	(R)D L|T|D|Y/L/m/K(I)	m:Oxidized methionine	162.17	507.7443	998.486	15.995	0.1	4.21	37482.1	5.36	HUMAN	E9PG30	Actin, gamma-enteric smooth muscle
2368	dirMS_041712_NAR_30mM_minus2.14569.14665.2	2	15.74	0	2.99E+05		(R)SLSAIYE	(R)S L|S|A|I/Y/L/E/K(L)		133.4	512.2885	1023.572	-0.0024	-2.3	5.72	533778	5.74	HUMAN	Q15149	Plectin
2369	dirMS_041712_NAR_50mM_minus2.13261.13378.3	3	15.74	1	5.24E+05		(K)ATLPSP(	(K)A/T|L/P/S/P D K L/P G F/K(M)		144.18	457.5912	1370.768	-0.0088	-6.4	8.64	118930.9	5.49	HUMAN	P22314	Ubiquitin-like modifier-activating enzyme 1
2370	dirMS_041712_NAR_60mM_minus.7691.7691.3	3	15.74	1	6.25E+05		(K)IQDKEGI	(K)I Q D K E/G I|P/P/D/Q Q/R(L)		102.15	508.5978	1523.781	-0.0024	-1.6	4.56	17271.6	6.75	HUMAN	B4DV12	Ubiquitin
2371	dirMS_041712_NAR_60mM_minus.8289.8354.3	3	15.74	1	2.41E+05		(K)MIYASSI	(K)M/I/Y/A/S/S K D/A I/K(K)		106.4	409.5576	1226.645	0.0133	10.9	8.26	22956	8.8	HUMAN	E9PK25	Cofilin-1
2372	dirMS_041712_NAR_20mM_plus.10801.10801.2	2	15.74	0	8.63E+04		(R)FLEQQN	(R)F L|E\Q/Q N Q/V/L|Q/T\K(W)		146.73	738.3942	1475.785	-0.0041	-2.8	6	66208.7	8.32	HUMAN	P04264	Keratin, type II cytoskeletal 1
2373	dirMS_041712_NAR_20mM_plus.17535.17535.2	2	15.74	0	2.22E+05		(K)LLYAFAE	(K)L L|Y/A/F A E/A\T V/P K(V)		192.97	661.8712	1322.736	-0.0003	-0.3	6	61363	8.73	HUMAN	C9JQS9	Propionyl-CoA carboxylase beta chain, mitochondrial
2374	dirMS_041712_NAR_30mM_plus2.6659.6659.2	2	15.74	0	2.92E+05		(R)EAGEQC	(R)E\A/G|E/Q\G D/I|E/P R(R)		89.77	600.7772	1200.549	-0.002	-1.7	4	31450.7	5.73	HUMAN	F5H4D6	Ras GTPase-activating protein-binding protein 1
2375	dirMS_041712_NAR_30mM_plus2.9303.9891.2	2	15.74	0	9.85E+06		(R)LASYLDI	(R)L/A|S|Y L/D|R(V)		104.55	419.2286	837.447	0.0034	4.1	5.83	43773.3	5.26	HUMAN	F8VZY9	Keratin, type I cytoskeletal 18
2376	dirMS_041712_NAR_50mM_plus2.8689.8689.3	3	15.74	0	1.95E+04		(R)TVAIYSE	(R)T V|A|I/Y S E Q/D T/G Q/M/H R(Q)		113.87	579.2737	1735.807	-0.0003	-0.2	5.29	58266.4	9.46	HUMAN	B4DN00	Pyruvate carboxylase, mitochondrial
2377	dirMS_041712_NAR_60mM_plus.15640.15683.3	3	15.74	1	3.43E+06		(R)TEMENE	(R)T|E|M/E N E|F/V/L/I/K K(D)		138.23	494.2622	1480.772	0.0005	0.3	4.78	13588.1	4.66	HUMAN	F8VP67	Keratin, type II cytoskeletal 8 (Fragment)
2378	dirMS_041712_NAR_60mM_plus.20147.20161.3	3	15.74	0	2.10E+06		(K)VHLVGII	(K)V H\L\V G|I/D|I/F/T/G K(K)		166.1	433.5839	1298.747	-0.0096	-7.4	6.71	13048.7	6.42	HUMAN	C9J4W5	Eukaryotic translation initiation factor 5A-2 (Fragment)
2379	dirMS_041712_NAR_120mM_plus.10363.10740.3	3	15.73	1	1.62E+06		(R)FLRENP\	(R)F L/R E N|P|Y|Y/D/S|R(V)		108.55	487.239	1459.697	0.006	4.1	6.07	193379.8	5.48	HUMAN	Q00610	Clathrin heavy chain 1
2380	dirMS_041712_NAR_20mM_plus.14479.14479.2	2	15.73	0	6.07E+05	M185m	(K)LAVNm\	(K)L/A/V|N m/V|P F\P R(L)	m:Oxidized methionine	170.72	580.3169	1143.634	15.9922	-2.3	9.75	42230.8	4.77	HUMAN	A6NKZ8	Putative tubulin beta chain-like protein ENSP00000290377
2381	dirMS_041712_NAR_20mM_plus.16203.16496.3	3	15.73	0	1.17E+06	M221m	(R)ALTVPEI	(R)A L\T|V P E/L/T/Q/Q/m/F D/A K(N)	m:Oxidized methionine	184.35	569.9583	1691.867	15.9931	-1.1	4.37	42831.5	4.86	HUMAN	A8K854	HCG1983504, isoform CRA_f
2382	dirMS_041712_NAR_20mM_plus.16477.16477.2	2	15.73	0	4.51E+05		(K)LGGSAV	(K)L/G\G S A\V|I/S L E/G K|P L(-)		185.52	670.8917	1340.778	-0.0023	-1.7	6	22956	8.8	HUMAN	E9PK25	Cofilin-1
2383	dirMS_041712_NAR_50mM_plus2.13332.13332.3	3	15.73	0	4.70E+05		(K)QLTEED(	(K)Q L/T/E/E/D/G V/H S V/I E E N I K(C)		138.32	647.3311	1939.961	0.018	9.3	4.25	54427.4	5.96	HUMAN	K4DID9	Acetyl-CoA carboxylase 1
2384	dirMS_041712_NAR_50mM_plus2.18603.18603.3	3	15.73	1	5.52E+05		(K)NAAGAL	(K)N A|A|G/A/L/D L/L K/E L/K(N)		165.92	452.601	1355.789	-0.0008	-0.6	6.07	13786.8	9.33	HUMAN	B7Z4S1	Transcription elongation factor A protein 1
2385	dirMS_041712_NAR_120mM_minus.19502.19502.3	3	15.72	1	3.72E+05		(K)NLRDIDI	(K)N L R\ D I D/E|V|S/S/L L R(T)		161.42	510.614	1529.828	-0.0007	-0.5	4.56	58214.7	5.25	HUMAN	B4DEM7	T-complex protein 1 subunit theta
2386	dirMS_041712_NAR_50mM_minus2.16891.16891.3	3	15.72	1	2.84E+05		(R)SELELTI	(R)S E|L/E L T/L/G K L E Q V R(S)		163.78	538.9725	1614.906	-0.0032	-2	4.78	533778	5.74	HUMAN	Q15149	Plectin
2387	dirMS_041712_NAR_20mM_plus.15400.15400.2	2	15.72	0	7.60E+05		(K)LCGLLA	(K)L L/C/G L L/A E K(T)	C:Carbamidomethylation	177.75	522.7971	1044.587	-0.0001	-0.1	6	12647.2	8.07	HUMAN	P14174	Macrophage migration inhibitory factor
2388	dirMS_041712_NAR_30mM_plus2.16147.16147.2	2	15.72	0	7.42E+05		(R)GALQNI	(R)G A L\Q/N|I|I\P A S/T/G A A K(A)		137.53	706.3979	1411.79	-0.0018	-1.3	8.75	31718.5	7.17	HUMAN	E7EUT4	Glyceraldehyde-3-phosphate dehydrogenase
2389	dirMS_041712_NAR_30mM_plus2.6277.6277.2	2	15.72	0	3.51E+05		(K)NQCTQ\	(K)N Q|C|T/Q V/V/Q E R(E)	C:Carbamidomethylation	87	631.3039	1261.595	0.0052	4.1	6	334221.3	6.44	HUMAN	P15924	Desmoplakin
2390	dirMS_041712_NAR_60mM_plus.17743.17743.3	3	15.72	1	2.42E+05		(K)IIVDELKI	(K)I/I|V/D E/L K Q|E/V\I S\T S S K(A)		149.47	597.0009	1788.995	-0.0072	-4	4.68	30149.5	5.96	HUMAN	D6RAC2	Guanine nucleotide-binding protein subunit beta-2-like 1
2391	dirMS_041712_NAR_30mM_minus2.9610.9610.2	2	15.71	0	8.22E+05		(K)EAESSPI	(K)E A|E/S/S/P F V/E R(L)		107.22	575.7705	1150.537	-0.0038	-3.3	4.25	92752.5	4.76	HUMAN	P14625	Endoplasmin
2392	dirMS_041712_NAR_40mM_minus2.11984.11984.3	3	15.71	1	1.80E+05		(K)AGTQIEI	(K)A/G T|Q/I/E N I/D E D/F R D/G L/K(L)		153.87	640.9843	1920.93	0.0086	4.5	4.11	59872.5	4.85	HUMAN	F5GXS2	Alpha-actinin-4
2393	dirMS_041712_NAR_60mM_minus.11484.11630.3	3	15.71	0	7.51E+05		(K)TKPYIQ\	(K)T K|P Y\I\Q\V/D|I/G/G G Q/T K(T)		124.67	535.6216	1604.864	-0.014	-8.7	8.17	72445.7	5.07	HUMAN	P11021	78 kDa glucose-regulated protein

<table>
<tr><td></td><td></td><td></td><td></td><td></td><td></td><td></td><td></td><td></td><td></td><td></td><td></td><td></td><td></td><td></td><td></td><td></td><td></td><td></td><td></td><td></td></tr>
<tr><td>2394</td><td>dirMS_041712_NAR_60mM_minus.6102.6102.3</td><td>3</td><td>15.71</td><td>1</td><td>1.53E+06</td><td></td><td>(K)DSYVGD</td><td>(K)D S/Y|V/G D/E/A Q S K/R(G)</td><td></td><td>90.97</td><td>452.2129</td><td>1354.623</td><td>0.0008</td><td>0.6</td><td>4.56</td><td>32616.5</td><td>5.18</td><td>HUMAN</td><td>A6NL76</td><td>Actin, alpha skeletal muscle</td></tr>
<tr><td>2395</td><td>dirMS_041712_NAR_30mM_plus2.8326.8885.2</td><td>2</td><td>15.71</td><td>0</td><td>2.01E+06</td><td></td><td>(K)TAVCDIF</td><td>(K)T A|V|C/D/I|P|P/R(G)</td><td>C:Carbamidomethylation</td><td>100.2</td><td>514.7643</td><td>1028.519</td><td>0.002</td><td>1.9</td><td>5.5</td><td>50199.4</td><td>4.75</td><td>HUMAN</td><td>A6NNZ2</td><td>Tubulin beta-8 chain-like protein LOC260334</td></tr>
<tr><td>2396</td><td>dirMS_041712_NAR_50mM_plus2.14115.14115.3</td><td>3</td><td>15.71</td><td>1</td><td>7.81E+05</td><td></td><td>(R)MSMKE</td><td>(R)M/S/M K E V D E Q/M/L N|V|Q N K(N)</td><td></td><td>142.88</td><td>641.9706</td><td>1923.897</td><td>0</td><td>0</td><td>4.68</td><td>40069.4</td><td>4.83</td><td>HUMAN</td><td>E7EWR1</td><td>Tubulin beta chain</td></tr>
<tr><td>2397</td><td>dirMS_041712_NAR_50mM_plus2.16417.16417.3</td><td>3</td><td>15.71</td><td>1</td><td>2.02E+06</td><td></td><td>(K)QGGLGF</td><td>(K)Q G G L/G/P M/N\I|P L V/S D/P K R(T)</td><td></td><td>154.68</td><td>593.6536</td><td>1778.958</td><td>-0.0119</td><td>-6.7</td><td>8.75</td><td>22338.2</td><td>8.65</td><td>HUMAN</td><td>Q06830</td><td>Peroxiredoxin-1</td></tr>
<tr><td>2398</td><td>dirMS_041712_NAR_60mM_plus.18266.18266.3</td><td>3</td><td>15.71</td><td>1</td><td>9.23E+05</td><td></td><td>(K)EVNKFQ</td><td>(K)E V N K F Q\M|A|Y/S/N/L L R(A)</td><td></td><td>152.83</td><td>571.6293</td><td>1712.879</td><td>-0.0055</td><td>-3.2</td><td>8.69</td><td>14683.8</td><td>10.19</td><td>HUMAN</td><td>P37108</td><td>Signal recognition particle 14 kDa protein</td></tr>
<tr><td>2399</td><td>dirMS_041712_NAR_120mM_minus.8998.8998.3</td><td>3</td><td>15.7</td><td>0</td><td>9.66E+05</td><td></td><td>(K)HTGPNS</td><td>(K)H T G P N S P D\T|A/N/D G F V/R(L)</td><td></td><td>103.18</td><td>562.2599</td><td>1684.767</td><td>-0.0022</td><td>-1.3</td><td>5.21</td><td>18511.8</td><td>5.62</td><td>HUMAN</td><td>D6R9T0</td><td>Heterogeneous nuclear ribonucleoprotein H (Fragment)</td></tr>
<tr><td>2400</td><td>dirMS_041712_NAR_30mM_minus2.10500.10609.2</td><td>2</td><td>15.7</td><td>0</td><td>3.43E+05</td><td></td><td>(K)SAVGFD</td><td>(K)S A|V|G|F/D/Y/Q/G K(T)</td><td></td><td>111.15</td><td>536.2608</td><td>1071.511</td><td>0.0038</td><td>3.5</td><td>5.55</td><td>25153.2</td><td>6.6</td><td>HUMAN</td><td>H7C314</td><td>Src substrate cortactin (Fragment)</td></tr>
<tr><td>2401</td><td>dirMS_041712_NAR_30mM_minus2.18138.18138.2</td><td>2</td><td>15.7</td><td>0</td><td>2.25E+05</td><td></td><td>(K)EGMNIV</td><td>(K)E G\M/N|I/V E/A/M E R(F)</td><td></td><td>152.4</td><td>639.7955</td><td>1278.582</td><td>0.002</td><td>1.6</td><td>4.25</td><td>18240.4</td><td>7.96</td><td>HUMAN</td><td>P62937</td><td>Peptidyl-prolyl cis-trans isomerase A</td></tr>
<tr><td>2402</td><td>dirMS_041712_NAR_50mM_minus2.15570.15570.3</td><td>3</td><td>15.7</td><td>0</td><td>2.05E+05</td><td></td><td>(R)QVVQGI</td><td>(R)Q V\V/Q|G\L\L/S\E T Y/L/E A H R(I)</td><td></td><td>156.73</td><td>614.9953</td><td>1842.971</td><td>0.0005</td><td>0.3</td><td>5.4</td><td>81934.1</td><td>6.08</td><td>HUMAN</td><td>P33993</td><td>DNA replication licensing factor MCM7</td></tr>
<tr><td>2403</td><td>dirMS_041712_NAR_20mM_plus.18814.18930.2</td><td>2</td><td>15.7</td><td>0</td><td>1.09E+06</td><td></td><td>(K)EIIDLVLI</td><td>(K)E I|I/D|L/V/L/D/R(I)</td><td></td><td>202.28</td><td>543.313</td><td>1085.62</td><td>-0.0014</td><td>-1.3</td><td>4.03</td><td>58642</td><td>4.95</td><td>HUMAN</td><td>F5H5D3</td><td>Tubulin alpha-1C chain</td></tr>
<tr><td>2404</td><td>dirMS_041712_NAR_30mM_plus2.16266.16266.2</td><td>2</td><td>15.7</td><td>0</td><td>2.25E+05</td><td></td><td>(K)IIIPEIQK</td><td>(K)I I|I/P/E/I/Q/K(V)</td><td></td><td>137.62</td><td>477.3031</td><td>953.603</td><td>-0.0041</td><td>-4.3</td><td>6</td><td>86098.8</td><td>5.31</td><td>HUMAN</td><td>B4DUC5</td><td>Exportin-2</td></tr>
<tr><td>2405</td><td>dirMS_041712_NAR_30mM_plus2.16302.16302.2</td><td>2</td><td>15.7</td><td>0</td><td>1.18E+06</td><td></td><td>(R)FGLNVS</td><td>(R)F G|L/N/V/S/S I S/R(K)</td><td></td><td>139.02</td><td>540.3016</td><td>1079.584</td><td>0.0115</td><td>10.7</td><td>9.75</td><td>24161.9</td><td>5.27</td><td>HUMAN</td><td>F8VZQ9</td><td>SAP domain-containing ribonucleoprotein</td></tr>
<tr><td>2406</td><td>dirMS_041712_NAR_30mM_plus2.16766.16793.2</td><td>2</td><td>15.7</td><td>0</td><td>4.37E+04</td><td></td><td>(R)AALQELI</td><td>(R)A/A/L/Q/E/L L|S/K(G)</td><td></td><td>140.85</td><td>486.7894</td><td>972.572</td><td>-0.0009</td><td>-0.9</td><td>6.05</td><td>13798.9</td><td>10.17</td><td>HUMAN</td><td>P62851</td><td>40S ribosomal protein S25</td></tr>
<tr><td>2407</td><td>dirMS_041712_NAR_60mM_plus.16698.16800.3</td><td>3</td><td>15.7</td><td>1</td><td>5.39E+05</td><td></td><td>(K)AKLEQLF</td><td>(K)A K L E Q\L|F|Q/D E\V/A/K(A)</td><td></td><td>144.23</td><td>506.9426</td><td>1518.816</td><td>-0.003</td><td>-2</td><td>4.68</td><td>23903</td><td>4.94</td><td>HUMAN</td><td>H0YDN1</td><td>Plectin (Fragment)</td></tr>
<tr><td>2408</td><td>dirMS_041712_NAR_60mM_plus.8313.8313.3</td><td>3</td><td>15.7</td><td>1</td><td>7.22E+05</td><td></td><td>(R)DSFDGR</td><td>(R)D S|F/D/G R G P/P G P\E/S Q S R(A)</td><td></td><td>101.7</td><td>563.5923</td><td>1688.762</td><td>0</td><td>0</td><td>4.56</td><td>13246.4</td><td>9.21</td><td>HUMAN</td><td>Q5JY17</td><td>Nuclear receptor coactivator 5 (Fragment)</td></tr>
<tr><td>2409</td><td>dirMS_041712_NAR_60mM_minus.14874.14874.3</td><td>3</td><td>15.69</td><td>1</td><td>2.10E+05</td><td></td><td>(K)IDSRLEQ</td><td>(K)I/D/S R L E Q/Y\T|S/A/I/E G T K(S)</td><td></td><td>142.88</td><td>604.3095</td><td>1810.918</td><td>-0.0042</td><td>-2.3</td><td>4.68</td><td>54214.9</td><td>5.66</td><td>HUMAN</td><td>C9J813</td><td>Caldesmon (Fragment)</td></tr>
<tr><td>2410</td><td>dirMS_041712_NAR_30mM_plus2.18588.18588.2</td><td>2</td><td>15.69</td><td>0</td><td>1.90E+05</td><td></td><td>(-)SDVLELT</td><td>(-)S D V/L/E|L/T/D D\N F/E S R(I)</td><td></td><td>150.83</td><td>820.3752</td><td>1639.745</td><td>-0.0015</td><td>-0.9</td><td>3.77</td><td>13747.3</td><td>6.8</td><td>HUMAN</td><td>H7BZJ3</td><td>Thioredoxin (Fragment)</td></tr>
<tr><td>2411</td><td>dirMS_041712_NAR_30mM_plus2.7276.7291.2</td><td>2</td><td>15.69</td><td>0</td><td>1.84E+05</td><td></td><td>(K)AAAAAA</td><td>(K)A A|A|A|A|A|A L Q/A K(S)</td><td></td><td>92.88</td><td>478.7844</td><td>956.552</td><td>0.0092</td><td>9.6</td><td>8.8</td><td>37884.5</td><td>11.27</td><td>HUMAN</td><td>H3BM89</td><td>60S ribosomal protein L4</td></tr>
<tr><td>2412</td><td>dirMS_041712_NAR_50mM_plus2.11443.11443.3</td><td>3</td><td>15.69</td><td>1</td><td>2.61E+05</td><td></td><td>(K)CATITPD</td><td>(K)C/A|T I/T/P D/E A R V E E/F/K(L)</td><td>C:Carbamidomethylation</td><td>127.75</td><td>589.2842</td><td>1765.843</td><td>-0.0045</td><td>-2.5</td><td>4.41</td><td>45578.2</td><td>7.83</td><td>HUMAN</td><td>B4DFL2</td><td>Isocitrate dehydrogenase [NADP]</td></tr>
<tr><td>2413</td><td>dirMS_041712_NAR_40mM_minus2.13831.13831.3</td><td>3</td><td>15.68</td><td>0</td><td>1.24E+05</td><td></td><td>(R)VAAGLP</td><td>(R)V A|A/G L|P P I L V H/T D A A Q A/L G K(Q)</td><td></td><td>165.12</td><td>648.0401</td><td>1942.112</td><td>-0.0063</td><td>-3.2</td><td>6.71</td><td>17974.2</td><td>4.97</td><td>HUMAN</td><td>C9JNL5</td><td>Selenocysteine lyase (Fragment)</td></tr>
<tr><td>2414</td><td>dirMS_041712_NAR_120mM_plus.4091.4306.3</td><td>3</td><td>15.68</td><td>1</td><td>2.05E+06</td><td>M54m</td><td>(K)SLDEmE</td><td>(K)S/L|D/E/m/E/K/Q/Q R(E)</td><td>m:Oxidized methionine</td><td>70.2</td><td>427.2032</td><td>1263.6</td><td>15.9953</td><td>0.3</td><td>4.68</td><td>26419.2</td><td>5.1</td><td>HUMAN</td><td>H0Y9K7</td><td>Splicing factor, proline- and glutamine-rich (Fragment)</td></tr>
<tr><td>2415</td><td>dirMS_041712_NAR_50mM_plus2.15514.15514.3</td><td>3</td><td>15.68</td><td>0</td><td>1.51E+06</td><td></td><td>(R)LASTLVH</td><td>(R)L A|S/T L V/H\L|G E Y Q A\A/V/D G A R(K)</td><td></td><td>149.68</td><td>657.6802</td><td>1971.029</td><td>-0.0034</td><td>-1.7</td><td>5.32</td><td>71165.3</td><td>5.57</td><td>HUMAN</td><td>J3KS13</td><td>Clathrin heavy chain 1</td></tr>
<tr><td>2416</td><td>dirMS_041712_NAR_50mM_minus2.10984.10985.3</td><td>3</td><td>15.67</td><td>1</td><td>2.77E+06</td><td>M72m</td><td>(R)TEmENE</td><td>(R)T|E|m E N E\F|V/L/I/K K(D)</td><td>m:Oxidized methionine</td><td>129.93</td><td>499.5937</td><td>1480.772</td><td>15.995</td><td>0</td><td>4.78</td><td>13588.1</td><td>4.66</td><td>HUMAN</td><td>F8VP67</td><td>Keratin, type II cytoskeletal 8 (Fragment)</td></tr>
<tr><td>2417</td><td>dirMS_041712_NAR_20mM_plus.17541.17558.2</td><td>2</td><td>15.67</td><td>0</td><td>3.08E+05</td><td></td><td>(K)SYELPDC</td><td>(K)S Y E/L|P D\G Q/V|I|T/I/G N E R(F)</td><td></td><td>193.17</td><td>895.9492</td><td>1790.892</td><td>-0.0008</td><td>-0.4</td><td>4.14</td><td>123097.1</td><td>5.83</td><td>HUMAN</td><td>A5A3E0</td><td>POTE ankyrin domain family member F</td></tr>
<tr><td>2418</td><td>dirMS_041712_NAR_40mM_plus2.13928.13946.3</td><td>3</td><td>15.67</td><td>0</td><td>3.53E+05</td><td></td><td>(K)LVEALCA</td><td>(K)L/V|E|A/L/C A E/H Q I N L I/K(V)</td><td>C:Carbamidomethylation</td><td>148.62</td><td>584.3205</td><td>1750.952</td><td>-0.0051</td><td>-2.9</td><td>5.4</td><td>14914.1</td><td>6.84</td><td>HUMAN</td><td>P25398</td><td>40S ribosomal protein S12</td></tr>
<tr><td>2419</td><td>dirMS_041712_NAR_50mM_plus2.15734.15734.3</td><td>3</td><td>15.67</td><td>0</td><td>1.18E+05</td><td></td><td>(K)NLDYVA</td><td>(K)N L|D|Y V/A/T/S I/H E A V/T K(I)</td><td></td><td>150.87</td><td>554.2887</td><td>1660.854</td><td>-0.0025</td><td>-1.5</td><td>5.32</td><td>44431.6</td><td>8.52</td><td>HUMAN</td><td>B7Z7E9</td><td>Aspartate aminotransferase</td></tr>
<tr><td>2420</td><td>dirMS_041712_NAR_60mM_plus.11804.11804.3</td><td>3</td><td>15.67</td><td>1</td><td>3.83E+05</td><td></td><td>(R)LAEVEA</td><td>(R)L/A|E/V/E/A A L E|K Q R(Q)</td><td></td><td>118.88</td><td>452.9164</td><td>1356.748</td><td>-0.0135</td><td>-10</td><td>4.79</td><td>533778</td><td>5.74</td><td>HUMAN</td><td>Q15149</td><td>Plectin</td></tr>
<tr><td>2421</td><td>dirMS_041712_NAR_120mM_minus.13545.13579.3</td><td>3</td><td>15.66</td><td>1</td><td>2.25E+05</td><td></td><td>(R)VIDRELY</td><td>(R)V/I/D/R E L|Y|Q/Q/L Q R(G)</td><td></td><td>128.15</td><td>520.955</td><td>1560.849</td><td>0.0012</td><td>0.8</td><td>6.04</td><td>533778</td><td>5.74</td><td>HUMAN</td><td>Q15149</td><td>Plectin</td></tr>
<tr><td>2422</td><td>dirMS_041712_NAR_20mM_minus.8320.8320.2</td><td>2</td><td>15.66</td><td>0</td><td>1.25E+05</td><td></td><td>(K)VTADVII</td><td>(K)V/T|A/D\V/I/N A A E/K(L)</td><td></td><td>132.47</td><td>565.8065</td><td>1130.605</td><td>0.0005</td><td>0.5</td><td>4.37</td><td>57391.2</td><td>6.29</td><td>HUMAN</td><td>O43175</td><td>D-3-phosphoglycerate dehydrogenase</td></tr>
<tr><td>2423</td><td>dirMS_041712_NAR_50mM_minus2.17714.17770.3</td><td>3</td><td>15.66</td><td>1</td><td>3.97E+05</td><td></td><td>(R)LKLPSIPI</td><td>(R)L K L/P S I|P\L\V|P V/S A Q K(R)</td><td></td><td>169.73</td><td>530.669</td><td>1589.999</td><td>-0.0064</td><td>-4.1</td><td>10</td><td>34958.1</td><td>9.42</td><td>HUMAN</td><td>Q15785</td><td>Mitochondrial import receptor subunit TOM34</td></tr>
<tr><td>2424</td><td>dirMS_041712_NAR_60mM_minus.14913.14913.3</td><td>3</td><td>15.66</td><td>0</td><td>4.33E+05</td><td></td><td>(K)WIGLDL</td><td>(K)W\I|G L D/L/S/N/G K/P R(D)</td><td></td><td>143.53</td><td>452.5849</td><td>1355.743</td><td>-0.0029</td><td>-2.1</td><td>8.75</td><td>44227.3</td><td>7.81</td><td>HUMAN</td><td>E7EQR6</td><td>T-complex protein 1 subunit alpha</td></tr>
<tr><td>2425</td><td>dirMS_041712_NAR_60mM_minus.15742.15742.3</td><td>3</td><td>15.66</td><td>1</td><td>1.58E+06</td><td></td><td>(R)KDDPVT</td><td>(R)K D D\P\V\T\N\L N/N/A/F E V/A E K(Y)</td><td></td><td>147.43</td><td>635.317</td><td>1903.94</td><td>-0.0032</td><td>-1.7</td><td>4.32</td><td>21601.4</td><td>6.27</td><td>HUMAN</td><td>K7EJH8</td><td>Alpha-actinin-4 (Fragment)</td></tr>
<tr><td>2426</td><td>dirMS_041712_NAR_60mM_minus.9452.9452.3</td><td>3</td><td>15.66</td><td>1</td><td>2.04E+05</td><td>M118m</td><td>(R)LSFDKD</td><td>(R)L/S/F/D K/D A m/V A R(A)</td><td>m:Oxidized methionine</td><td>112.4</td><td>423.5497</td><td>1252.635</td><td>15.9991</td><td>3.3</td><td>5.96</td><td>35556.4</td><td>9.3</td><td>HUMAN</td><td>F2Z393</td><td>Transaldolase</td></tr>
<tr><td>2427</td><td>dirMS_041712_NAR_120mM_plus.12464.12908.3</td><td>3</td><td>15.66</td><td>1</td><td>1.16E+06</td><td></td><td>(K)LFNLSKE</td><td>(K)L/F|N/L/S/K/E D D/V/R(Q)</td><td></td><td>119.35</td><td>445.9007</td><td>1335.69</td><td>-0.0028</td><td>-2.1</td><td>4.56</td><td>25082</td><td>11.29</td><td>HUMAN</td><td>A2A3R5</td><td>40S ribosomal protein S6</td></tr>
<tr><td>2428</td><td>dirMS_041712_NAR_120mM_plus.21512.21533.4</td><td>4</td><td>15.66</td><td>2</td><td>4.53E+05</td><td>M145m</td><td>(R)RGVmLA</td><td>(R)R G V\m\L|A|V D/A V/I/A E L K/K(Q)</td><td>m:Oxidized methionine</td><td>166.22</td><td>433.0059</td><td>1713.009</td><td>15.9926</td><td>-1.3</td><td>8.59</td><td>25225.7</td><td>9.52</td><td>HUMAN</td><td>E7ESH4</td><td>60 kDa heat shock protein, mitochondrial (Fragment)</td></tr>
<tr><td>2429</td><td>dirMS_041712_NAR_30mM_plus2.19472.19874.2</td><td>2</td><td>15.66</td><td>0</td><td>8.20E+05</td><td></td><td>(-)MDELQL</td><td>(-)M/D|E|L|Q/L/F/R(G)</td><td></td><td>156.63</td><td>526.2726</td><td>1051.524</td><td>0.0138</td><td>13.2</td><td>4.37</td><td>18601</td><td>5.1</td><td>HUMAN</td><td>C9IZA5</td><td>Transitional endoplasmic reticulum ATPase (Fragment)</td></tr>
<tr><td>2430</td><td>dirMS_041712_NAR_30mM_plus2.19956.19956.2</td><td>2</td><td>15.66</td><td>0</td><td>8.31E+04</td><td></td><td>(R)FEELTNL</td><td>(R)F/E|E|L/T N L\I R(T)</td><td></td><td>158.62</td><td>567.8152</td><td>1134.615</td><td>0.0078</td><td>6.9</td><td>4.53</td><td>106155.7</td><td>5.45</td><td>HUMAN</td><td>B5ME19</td><td>Eukaryotic translation initiation factor 3 subunit C</td></tr>
<tr><td>2431</td><td>dirMS_041712_NAR_50mM_plus2.5963.5963.3</td><td>3</td><td>15.66</td><td>0</td><td>3.79E+04</td><td></td><td>(R)ITAAQH</td><td>(R)I/T|A/A Q H\S V T G S A V S K(T)</td><td></td><td>95.48</td><td>486.2644</td><td>1456.775</td><td>0.0032</td><td>2.2</td><td>8.76</td><td>5282</td><td>9.4</td><td>HUMAN</td><td>E9PJT1</td><td>Phosphatidylinositol-binding clathrin assembly protein</td></tr>
<tr><td>2432</td><td>dirMS_041712_NAR_50mM_plus2.7946.7946.3</td><td>3</td><td>15.66</td><td>1</td><td>5.05E+04</td><td></td><td>(K)ELQAAG</td><td>(K)E L/Q|A/A G/K S/P E/D/L E R(L)</td><td></td><td>108.27</td><td>514.9321</td><td>1542.776</td><td>0.0059</td><td>3.8</td><td>4.41</td><td>63374.2</td><td>8.64</td><td>HUMAN</td><td>P06744</td><td>Glucose-6-phosphate isomerase</td></tr>
<tr><td>2433</td><td>dirMS_041712_NAR_50mM_plus2.9304.9304.3</td><td>3</td><td>15.66</td><td>1</td><td>2.75E+05</td><td></td><td>(R)TNAENE</td><td>(R)T|N/A/E N E F/V/T I/K K(D)</td><td></td><td>115.83</td><td>465.2518</td><td>1393.732</td><td>0.0087</td><td>6.2</td><td>5.81</td><td>66208.7</td><td>8.32</td><td>HUMAN</td><td>P04264</td><td>Keratin, type II cytoskeletal 1</td></tr>
<tr><td>2434</td><td>dirMS_041712_NAR_50mM_minus2.13882.13882.3</td><td>3</td><td>15.65</td><td>0</td><td>3.00E+04</td><td></td><td>(K)TGEEIFG</td><td>(K)T/G|E/E|I/F G T I/G M R P N A K(N)</td><td></td><td>146.93</td><td>574.2947</td><td>1720.869</td><td>0.0009</td><td>0.5</td><td>5.81</td><td>38222.2</td><td>5.77</td><td>HUMAN</td><td>E9PKG1</td><td>Protein arginine N-methyltransferase 1</td></tr>
<tr><td>2435</td><td>dirMS_041712_NAR_120mM_plus.7268.7310.3</td><td>3</td><td>15.65</td><td>1</td><td>5.17E+05</td><td></td><td>(K)SKAEAE</td><td>(K)S K\A E A\E|S|L/Y/Q/S/K(Y)</td><td></td><td>91.33</td><td>447.5614</td><td>1340.669</td><td>0.0004</td><td>0.3</td><td>5.86</td><td>66208.7</td><td>8.32</td><td>HUMAN</td><td>P04264</td><td>Keratin, type II cytoskeletal 1</td></tr>
<tr><td>2436</td><td>dirMS_041712_NAR_60mM_plus.8292.8332.3</td><td>3</td><td>15.65</td><td>1</td><td>1.52E+06</td><td></td><td>(K)MIYASSI</td><td>(K)M/I/Y/A/S/S K/D A I/K(K)</td><td></td><td>101.88</td><td>409.553</td><td>1226.645</td><td>-0.0005</td><td>-0.4</td><td>8.26</td><td>22956</td><td>8.8</td><td>HUMAN</td><td>E9PK25</td><td>Cofilin-1</td></tr>
<tr><td>2437</td><td>dirMS_041712_NAR_30mM_minus2.12330.12623.2</td><td>2</td><td>15.64</td><td>0</td><td>1.76E+06</td><td></td><td>(R)LQAEIEC</td><td>(R)L/Q|A|E/I E/G L/K(G)</td><td></td><td>121.93</td><td>500.7842</td><td>1000.567</td><td>-0.0062</td><td>-6.2</td><td>4.53</td><td>37296.3</td><td>5.3</td><td>HUMAN</td><td>F8VU64</td><td>Keratin, type II cytoskeletal 8</td></tr>
<tr><td>2438</td><td>dirMS_041712_NAR_60mM_minus.6666.6884.3</td><td>3</td><td>15.64</td><td>1</td><td>4.60E+05</td><td></td><td>(R)LQSIGTE</td><td>(R)L/Q|S|I/G T/E N T/E E/N R/R(F)</td><td></td><td>96.57</td><td>549.6071</td><td>1646.809</td><td>-0.0025</td><td>-1.5</td><td>4.79</td><td>15633.5</td><td>9.15</td><td>HUMAN</td><td>H3BMQ8</td><td>Fructose-bisphosphate aldolase A (Fragment)</td></tr>
<tr><td>2439</td><td>dirMS_041712_NAR_120mM_plus.20656.20736.3</td><td>3</td><td>15.64</td><td>0</td><td>3.63E+05</td><td></td><td>(K)VHLVGII</td><td>(K)V H\L\V\G\I|D|I/F/T/G K(K)</td><td></td><td>161.62</td><td>433.5874</td><td>1298.747</td><td>0.0009</td><td>0.7</td><td>6.71</td><td>13048.7</td><td>6.42</td><td>HUMAN</td><td>C9J4W5</td><td>Eukaryotic translation initiation factor 5A-2 (Fragment)</td></tr>
<tr><td>2440</td><td>dirMS_041712_NAR_50mM_plus2.11394.11394.3</td><td>3</td><td>15.64</td><td>1</td><td>9.91E+05</td><td></td><td>(K)VIQEIVD</td><td>(K)V I/Q/E/I V D K/S G V V R(V)</td><td></td><td>127.82</td><td>481.2834</td><td>1441.837</td><td>-0.0017</td><td>-1.1</td><td>6.04</td><td>68668.3</td><td>5.77</td><td>HUMAN</td><td>B4DXZ6</td><td>Fragile X mental retardation syndrome-related protein 1</td></tr>
<tr><td>2441</td><td>dirMS_041712_NAR_50mM_minus2.17715.17715.3</td><td>3</td><td>15.63</td><td>0</td><td>6.40E+04</td><td></td><td>(R)IHFPLAT</td><td>(R)I H F P L\A\T\Y A/P/V/I S A E K(A)</td><td></td><td>169.52</td><td>586.3248</td><td>1756.963</td><td>-0.0034</td><td>-1.9</td><td>6.75</td><td>49012.3</td><td>4.9</td><td>HUMAN</td><td>A8MUB1</td><td>Tubulin alpha-4A chain</td></tr>
<tr><td>2442</td><td>dirMS_041712_NAR_50mM_minus2.9056.9056.3</td><td>3</td><td>15.63</td><td>0</td><td>7.04E+04</td><td></td><td>(R)EANALA</td><td>(R)E A/N A/L/A/A G H/P A Q A/V/A I N A R(L)</td><td></td><td>119.77</td><td>615.6611</td><td>1844.973</td><td>-0.0038</td><td>-2.1</td><td>6.85</td><td>272689.3</td><td>5.79</td><td>HUMAN</td><td>O15020</td><td>Spectrin beta chain, non-erythrocytic 2</td></tr>
<tr><td>2443</td><td>dirMS_041712_NAR_60mM_minus.9980.10285.3</td><td>3</td><td>15.63</td><td>1</td><td>6.51E+05</td><td></td><td>(K)VIADNV</td><td>(K)V/I|A/D/N V/K D W S/K(V)</td><td></td><td>116.02</td><td>425.5679</td><td>1274.674</td><td>0.0152</td><td>11.9</td><td>5.93</td><td>31075.7</td><td>5.65</td><td>HUMAN</td><td>P60174</td><td>Triosephosphate isomerase</td></tr>
<tr><td>2444</td><td>dirMS_041712_NAR_120mM_plus.7475.7688.3</td><td>3</td><td>15.63</td><td>1</td><td>2.02E+06</td><td>M281m</td><td>(R)SRAEAE</td><td>(R)S R\A E\A\E|S/m/Y Q/I/K(Y)</td><td>m:Oxidized methionine</td><td>93.02</td><td>476.8977</td><td>1412.684</td><td>15.9947</td><td>-0.1</td><td>5.86</td><td>37296.3</td><td>5.3</td><td>HUMAN</td><td>F8VU64</td><td>Keratin, type II cytoskeletal 8</td></tr>
<tr><td>2445</td><td>dirMS_041712_NAR_20mM_plus.15764.15764.2</td><td>2</td><td>15.63</td><td>0</td><td>2.05E+05</td><td></td><td>(K)TITLEVEI</td><td>(K)T I/T|L E/V/E|P S D T I/E N V K(A)</td><td></td><td>179.13</td><td>894.4658</td><td>1787.927</td><td>-0.003</td><td>-1.7</td><td>4</td><td>17271.6</td><td>6.75</td><td>HUMAN</td><td>B4DV12</td><td>Ubiquitin</td></tr>
<tr><td>2446</td><td>dirMS_041712_NAR_30mM_plus2.18017.18017.2</td><td>2</td><td>15.63</td><td>0</td><td>6.21E+05</td><td></td><td>(R)EQLAIAE</td><td>(R)E Q L/A/I/A E/F A R(S)</td><td></td><td>148.23</td><td>574.3083</td><td>1147.611</td><td>-0.0013</td><td>-1.1</td><td>4.53</td><td>44227.3</td><td>7.81</td><td>HUMAN</td><td>E7EQR6</td><td>T-complex protein 1 subunit alpha</td></tr>
<tr><td>2447</td><td>dirMS_041712_NAR_30mM_plus2.6153.6169.2</td><td>2</td><td>15.63</td><td>0</td><td>2.13E+05</td><td></td><td>(R)ETQSQL</td><td>(R)E T|Q/S/Q/L/E/T/E/R(S)</td><td></td><td>86.58</td><td>610.8002</td><td>1220.575</td><td>0.0178</td><td>14.6</td><td>4.25</td><td>334221.3</td><td>6.44</td><td>HUMAN</td><td>P15924</td><td>Desmoplakin</td></tr>
<tr><td>2448</td><td>dirMS_041712_NAR_30mM_plus2.7246.7287.2</td><td>2</td><td>15.63</td><td>0</td><td>1.31E+06</td><td></td><td>(R)VTDALN</td><td>(R)V/T|D|A|L/N/A T/R(A)</td><td></td><td>92.9</td><td>480.759</td><td>960.511</td><td>-0.0002</td><td>-0.2</td><td>5.81</td><td>61224.7</td><td>5.7</td><td>HUMAN</td><td>P10809</td><td>60 kDa heat shock protein, mitochondrial</td></tr>
<tr><td>2449</td><td>dirMS_041712_NAR_30mM_plus2.9598.9804.2</td><td>2</td><td>15.63</td><td>0</td><td>1.23E+06</td><td></td><td>(R)FGSGMI</td><td>(R)F/G|S|G/M N/M|G R(I)</td><td></td><td>104.72</td><td>478.7075</td><td>956.408</td><td>0</td><td>0</td><td>9.75</td><td>77799.7</td><td>9.09</td><td>HUMAN</td><td>P52272</td><td>Heterogeneous nuclear ribonucleoprotein M</td></tr>
<tr><td>2450</td><td>dirMS_041712_NAR_50mM_plus2.10972.10972.3</td><td>3</td><td>15.63</td><td>1</td><td>3.72E+05</td><td></td><td>(K)LAEQAE</td><td>(K)L A|E/Q/A E R Y D|D M/A|A C M K(S)</td><td>C:Carbamidomethylation</td><td>125.2</td><td>634.6126</td><td>1901.819</td><td>0.0042</td><td>2.2</td><td>4.32</td><td>5919.6</td><td>4.73</td><td>HUMAN</td><td>E5RGE1</td><td>14-3-3 protein zeta/delta (Fragment)</td></tr>
<tr><td>2451</td><td>dirMS_041712_NAR_120mM_minus.10006.10091.2</td><td>2</td><td>15.62</td><td>0</td><td>5.19E+05</td><td></td><td>(K)HIYYITG</td><td>(K)H|I|Y|Y|I|T\G E|T/K(D)</td><td></td><td>109.03</td><td>612.8167</td><td>1224.626</td><td>0.0002</td><td>0.2</td><td>6.75</td><td>85057.6</td><td>4.94</td><td>HUMAN</td><td>P07900</td><td>Heat shock protein HSP 90-alpha</td></tr>
<tr><td>2452</td><td>dirMS_041712_NAR_120mM_minus.13577.13597.4</td><td>4</td><td>15.62</td><td>2</td><td>8.32E+05</td><td>M100m</td><td>(K)KQGGLC</td><td>(K)K\Q G G L\G P\m/N\I/P L/V/S D/P K R(T)</td><td>m:Oxidized methionine</td><td>128.55</td><td>481.5173</td><td>1907.053</td><td>15.9942</td><td>-0.3</td><td>9.99</td><td>22338.2</td><td>8.65</td><td>HUMAN</td><td>Q06830</td><td>Peroxiredoxin-1</td></tr>
<tr><td>2453</td><td>dirMS_041712_NAR_30mM_minus2.10408.10408.2</td><td>2</td><td>15.62</td><td>0</td><td>1.88E+06</td><td></td><td>(K)TAVCDIF</td><td>(K)T/A|V/C D/I|P\P R(G)</td><td>C:Carbamidomethylation</td><td>110.4</td><td>514.7641</td><td>1028.519</td><td>0.0016</td><td>1.5</td><td>5.5</td><td>50199.4</td><td>4.75</td><td>HUMAN</td><td>A6NNZ2</td><td>Tubulin beta-8 chain-like protein LOC260334</td></tr>
<tr><td>2454</td><td>dirMS_041712_NAR_60mM_minus.11174.11479.3</td><td>3</td><td>15.62</td><td>1</td><td>3.02E+06</td><td></td><td>(R)MQKEIT</td><td>(R)M Q\K E I T/A|L|A/P S/T M/K(I)</td><td></td><td>123.32</td><td>516.9421</td><td>1548.812</td><td>-0.0007</td><td>-0.4</td><td>8.35</td><td>32616.5</td><td>5.18</td><td>HUMAN</td><td>A6NL76</td><td>Actin, alpha skeletal muscle</td></tr>
<tr><td>2455</td><td>dirMS_041712_NAR_60mM_minus.8508.8508.3</td><td>3</td><td>15.62</td><td>1</td><td>5.78E+04</td><td></td><td>(K)GAVAED</td><td>(K)G A/V|A/E|D G D E L R T E P E/A K(K)</td><td></td><td>107.4</td><td>596.2923</td><td>1786.845</td><td>0.017</td><td>9.5</td><td>4.08</td><td>19284.3</td><td>5.23</td><td>HUMAN</td><td>G3V3C7</td><td>DNA-(apurinic or apyrimidinic site) lyase (Fragment)</td></tr>
<tr><td>2456</td><td>dirMS_041712_NAR_30mM_plus2.9877.9916.2</td><td>2</td><td>15.62</td><td>0</td><td>6.65E+05</td><td></td><td>(K)NNTQVL</td><td>(K)N N|T/Q|V|L|I/N\C/R(N)</td><td>C:Carbamidomethylation</td><td>105.42</td><td>616.3143</td><td>1231.621</td><td>0.0001</td><td>0.1</td><td>9.75</td><td>8948</td><td>9.6</td><td>HUMAN</td><td>K7ERG4</td><td>Small nuclear ribonucleoprotein Sm D2</td></tr>
<tr><td>2457</td><td>dirMS_041712_NAR_50mM_plus2.11446.11483.3</td><td>3</td><td>15.62</td><td>1</td><td>3.27E+05</td><td></td><td>(K)ELALQPI</td><td>(K)E/L/A L Q/P/K/D D I/V/D/R(A)</td><td></td><td>128.03</td><td>504.606</td><td>1511.806</td><td>-0.003</td><td>-2</td><td>4.23</td><td>83905.8</td><td>6.62</td><td>HUMAN</td><td>P41250</td><td>Glycine--tRNA ligase</td></tr>
<tr><td>2458</td><td>dirMS_041712_NAR_60mM_plus.12936.12936.3</td><td>3</td><td>15.62</td><td>0</td><td>1.26E+06</td><td></td><td>(K)VNGRPL</td><td>(K)V/N/G R P L E/M|I/E/P/R(T)</td><td></td><td>124.65</td><td>470.9225</td><td>1410.752</td><td>0.0008</td><td>0.5</td><td>6.11</td><td>16559.1</td><td>10.36</td><td>HUMAN</td><td>P62249</td><td>40S ribosomal protein S16</td></tr>
<tr><td>2459</td><td>dirMS_041712_NAR_30mM_minus2.10551.10614.2</td><td>2</td><td>15.61</td><td>0</td><td>1.33E+06</td><td></td><td>(R)QLYEEEI</td><td>(R)Q L|Y/E/E/E/I/R(E)</td><td></td><td>111.58</td><td>540.2725</td><td>1079.537</td><td>0.001</td><td>0.9</td><td>4.25</td><td>37296.3</td><td>5.3</td><td>HUMAN</td><td>F8VU64</td><td>Keratin, type II cytoskeletal 8</td></tr>
<tr><td>2460</td><td>dirMS_041712_NAR_40mM_minus2.14870.14870.3</td><td>3</td><td>15.61</td><td>0</td><td>1.16E+06</td><td></td><td>(K)ISSIQSIV</td><td>(K)I S\S I/Q/S I/V/P A L E/I/A N/A H R(K)</td><td></td><td>172.13</td><td>640.3618</td><td>1919.071</td><td>0</td><td>0</td><td>6.75</td><td>61224.7</td><td>5.7</td><td>HUMAN</td><td>P10809</td><td>60 kDa heat shock protein, mitochondrial</td></tr>
<tr><td>2461</td><td>dirMS_041712_NAR_60mM_minus.11082.11082.3</td><td>3</td><td>15.61</td><td>1</td><td>6.02E+05</td><td></td><td>(K)IQVLQQ</td><td>(K)I Q|V/L/Q/Q Q A D D A E E/R A E R(L)</td><td></td><td>121.07</td><td>666.9983</td><td>1998.984</td><td>-0.0036</td><td>-1.8</td><td>4.18</td><td>26611.3</td><td>4.47</td><td>HUMAN</td><td>A6NL28</td><td>Putative tropomyosin alpha-3 chain-like protein</td></tr>
<tr><td>2462</td><td>dirMS_041712_NAR_60mM_minus.18417.18460.3</td><td>3</td><td>15.61</td><td>1</td><td>3.37E+05</td><td></td><td>(R)GFKDQI</td><td>(R)G F\K D\Q\I|Y/D/I/F/Q/K(L)</td><td></td><td>164.1</td><td>501.2584</td><td>1501.769</td><td>-0.0079</td><td>-5.3</td><td>5.96</td><td>26945.8</td><td>8.07</td><td>HUMAN</td><td>J3KSZ0</td><td>Eukaryotic initiation factor 4A-I (Fragment)</td></tr>
<tr><td>2463</td><td>dirMS_041712_NAR_120mM_plus.17297.17297.3</td><td>3</td><td>15.61</td><td>2</td><td>9.50E+05</td><td></td><td>(R)NLKASLI</td><td>(R)N L/K/A/S L/E N S L R E V E|A R(Y)</td><td></td><td>143.52</td><td>610.332</td><td>1828.988</td><td>-0.0061</td><td>-3.3</td><td>6.23</td><td>43773.3</td><td>5.26</td><td>HUMAN</td><td>F8VZY9</td><td>Keratin, type I cytoskeletal 18</td></tr>
<tr><td>2464</td><td>dirMS_041712_NAR_30mM_plus2.11962.11962.2</td><td>2</td><td>15.61</td><td>0</td><td>8.69E+04</td><td></td><td>(K)VGDPQE</td><td>(K)V/G/D|P Q/E|L/N G I T R(A)</td><td></td><td>115.82</td><td>649.8373</td><td>1298.67</td><td>-0.0026</td><td>-2</td><td>4.37</td><td>276046.1</td><td>6.01</td><td>HUMAN</td><td>P49327</td><td>Fatty acid synthase</td></tr>
<tr><td>2465</td><td>dirMS_041712_NAR_30mM_plus2.19784.20117.2</td><td>2</td><td>15.61</td><td>0</td><td>5.28E+05</td><td></td><td>(K)MAILQI</td><td>(K)M/A|I|L/Q/I M/K(E)</td><td></td><td>158.22</td><td>474.2739</td><td>947.542</td><td>-0.0011</td><td>-1.2</td><td>8.5</td><td>90316.8</td><td>5.52</td><td>HUMAN</td><td>Q13263</td><td>Transcription intermediary factor 1-beta</td></tr>
<tr><td>2466</td><td>dirMS_041712_NAR_30mM_plus2.8558.8558.2</td><td>2</td><td>15.61</td><td>0</td><td>8.96E+05</td><td></td><td>(R)LGLGEG.</td><td>(R)L/G/L/G E G/A E E|K(S)</td><td></td><td>99.07</td><td>501.7639</td><td>1002.51</td><td>0.0103</td><td>10.3</td><td>4.25</td><td>40096.8</td><td>9.24</td><td>HUMAN</td><td>C9JW69</td><td>Regulator of chromosome condensation (Fragment)</td></tr>
<tr><td>2467</td><td>dirMS_041712_NAR_40mM_plus2.11024.11024.3</td><td>3</td><td>15.61</td><td>1</td><td>6.32E+04</td><td></td><td>(K)VAVVAG</td><td>(K)V A|V/V/A/G/Y G D V/G/K G C A Q A L R(G)</td><td>C:Carbamidomethylation</td><td>131.05</td><td>630.9981</td><td>1890.985</td><td>-0.0057</td><td>-3</td><td>8.56</td><td>45228.3</td><td>6.03</td><td>HUMAN</td><td>F5H737</td><td>Adenosylhomocysteinase</td></tr>
<tr><td>2468</td><td>dirMS_041712_NAR_40mM_plus2.11226.11226.3</td><td>3</td><td>15.61</td><td>0</td><td>1.03E+05</td><td></td><td>(R)VLVASS</td><td>(R)V L|V/A/S/S V V A G E S Q K P E/L K(A)</td><td></td><td>132.08</td><td>614.3443</td><td>1841.038</td><td>-0.0195</td><td>-10.6</td><td>6.11</td><td>61441.6</td><td>5.93</td><td>HUMAN</td><td>G8JLE9</td><td>Fragile X mental retardation protein 1</td></tr>
<tr><td>2469</td><td>dirMS_041712_NAR_120mM_minus.11964.11974.3</td><td>3</td><td>15.6</td><td>0</td><td>3.85E+05</td><td></td><td>(R)HSSLAG</td><td>(R)H S\S L\A G|C|Q|I/I/N\Y R(T)</td><td>C:Carbamidomethylation</td><td>119.48</td><td>506.9192</td><td>1518.748</td><td>-0.0051</td><td>-3.4</td><td>8.75</td><td>193379.8</td><td>5.48</td><td>HUMAN</td><td>Q00610</td><td>Clathrin heavy chain 1</td></tr>
<tr><td>2470</td><td>dirMS_041712_NAR_120mM_minus.8225.8305.3</td><td>3</td><td>15.6</td><td>1</td><td>5.91E+05</td><td></td><td>(K)SSGGREI</td><td>(K)S/S|G G R E D\L\E|S/S/G/L/Q\R(R)</td><td></td><td>99.45</td><td>526.5882</td><td>1577.751</td><td>-0.0014</td><td>-0.9</td><td>4.68</td><td>11625.5</td><td>8.01</td><td>HUMAN</td><td>B8ZZT7</td><td>Hematological and neurological-expressed 1 protein</td></tr>
<tr><td>2471</td><td>dirMS_041712_NAR_60mM_minus.15075.15075.3</td><td>3</td><td>15.6</td><td>0</td><td>2.16E+05</td><td></td><td>(K)MLDAED</td><td>(K)M/L/D/A E D/I/V/N T/A R P D/E K(A)</td><td></td><td>143.72</td><td>606.2986</td><td>1816.875</td><td>0.0067</td><td>3.7</td><td>4.1</td><td>105308.6</td><td>5.27</td><td>HUMAN</td><td>O43707</td><td>Alpha-actinin-4</td></tr>
<tr><td>2472</td><td>dirMS_041712_NAR_30mM_plus2.13839.13903.2</td><td>2</td><td>15.6</td><td>0</td><td>5.28E+05</td><td></td><td>(K)YLVLDEA</td><td>(K)Y/L|V|L/D/E/A D/R(M)</td><td></td><td>125.72</td><td>547.2819</td><td>1093.552</td><td>0.0041</td><td>3.8</td><td>4.03</td><td>73386.6</td><td>8.73</td><td>HUMAN</td><td>B4DXX7</td><td>ATP-dependent RNA helicase DDX3Y</td></tr>
<tr><td>2473</td><td>dirMS_041712_NAR_30mM_plus2.21018.21018.2</td><td>2</td><td>15.6</td><td>0</td><td>2.03E+05</td><td></td><td>(R)GVVQEL</td><td>(R)G V/V/Q/E L|Q/Q A I/S\K(L)</td><td></td><td>165.2</td><td>650.3632</td><td>1299.727</td><td>-0.0076</td><td>-5.8</td><td>6</td><td>22489.6</td><td>5.38</td><td>HUMAN</td><td>E9PI39</td><td>Elongation factor 1-delta (Fragment)</td></tr>
<tr><td>2474</td><td>dirMS_041712_NAR_50mM_plus2.15068.15068.3</td><td>3</td><td>15.6</td><td>0</td><td>4.74E+05</td><td></td><td>(K)TTISVAH</td><td>(K)T T|I/S V/A/H L\L A A/R(Q)</td><td></td><td>146.48</td><td>418.2499</td><td>1252.737</td><td>-0.002</td><td>-1.6</td><td>9.44</td><td>34143.2</td><td>10.07</td><td>HUMAN</td><td>B4DT23</td><td>Protein FAM98A</td></tr>
<tr><td>2475</td><td>dirMS_041712_NAR_30mM_minus2.16476.16476.2</td><td>2</td><td>15.59</td><td>0</td><td>2.61E+05</td><td></td><td>(R)ASLEAAI</td><td>(R)A S L E/A\A/I/A/D/A E Q/R(G)</td><td></td><td>143.62</td><td>672.8404</td><td>1344.675</td><td>-0.0019</td><td>-1.4</td><td>4.14</td><td>56607.4</td><td>5.37</td><td>HUMAN</td><td>F8VXB4</td><td>Keratin, type II cytoskeletal 8</td></tr>
<tr><td>2476</td><td>dirMS_041712_NAR_30mM_minus2.19142.19142.2</td><td>2</td><td>15.59</td><td>0</td><td>2.58E+05</td><td></td><td>(K)SYELPDC</td><td>(K)S Y\E|L/P/D G Q/V|I/T I G N E R(F)</td><td></td><td>158.9</td><td>895.9492</td><td>1790.892</td><td>-0.0008</td><td>-0.4</td><td>4.14</td><td>123097.1</td><td>5.83</td><td>HUMAN</td><td>A5A3E0</td><td>POTE ankyrin domain family member F</td></tr>
<tr><td>2477</td><td>dirMS_041712_NAR_50mM_minus2.8682.8714.3</td><td>3</td><td>15.59</td><td>1</td><td>4.19E+04</td><td>M88m</td><td>(K)DVDEAY</td><td>(K)D V|D/E/A|Y/m N K V/E L E/S R(L)</td><td>m:Oxidized methionine</td><td>117.5</td><td>605.2817</td><td>1797.832</td><td>15.9982</td><td>1.8</td><td>4.18</td><td>13588.1</td><td>4.66</td><td>HUMAN</td><td>F8VP67</td><td>Keratin, type II cytoskeletal 8 (Fragment)</td></tr>
<tr><td>2478</td><td>dirMS_041712_NAR_20mM_plus.6866.6866.2</td><td>2</td><td>15.59</td><td>0</td><td>4.25E+05</td><td>M132m</td><td>(R)DmVGIA</td><td>(R)D m|V/G/I|A/Q T\G S G K(T)</td><td>m:Oxidized methionine</td><td>112.83</td><td>590.2864</td><td>1163.573</td><td>15.993</td><td>-1.6</td><td>5.84</td><td>73202</td><td>9.19</td><td>HUMAN</td><td>C9JMU5</td><td>Probable ATP-dependent RNA helicase DDX17</td></tr>
<tr><td>2479</td><td>dirMS_041712_NAR_120mM_minus.5618.5618.3</td><td>3</td><td>15.58</td><td>1</td><td>1.23E+05</td><td></td><td>(K)CDSSPD</td><td>(K)C D/S S/P D/S/A E D/V R K(V)</td><td>C:Carbamidomethylation</td><td>83.98</td><td>489.2132</td><td>1465.622</td><td>0.0027</td><td>1.8</td><td>4.23</td><td>40122.7</td><td>5.43</td><td>HUMAN</td><td>P02765</td><td>Alpha-2-HS-glycoprotein</td></tr>
<tr><td>2480</td><td>dirMS_041712_NAR_120mM_minus.8432.8515.3</td><td>3</td><td>15.58</td><td>1</td><td>4.37E+05</td><td></td><td>(K)NGRVEII</td><td>(K)N G R V E\I|I|A|N/D/Q/G N/R(I)</td><td></td><td>100.53</td><td>519.269</td><td>1555.794</td><td>-0.0011</td><td>-0.7</td><td>6.07</td><td>72445.7</td><td>5.07</td><td>HUMAN</td><td>P11021</td><td>78 kDa glucose-regulated protein</td></tr>
<tr><td>2481</td><td>dirMS_041712_NAR_30mM_minus2.10094.10291.2</td><td>2</td><td>15.58</td><td>0</td><td>6.09E+05</td><td>M239m</td><td>(K)EITALAP</td><td>(K)E I|T|A|L/A|P/S T m K(I)</td><td>m:Oxidized methionine</td><td>109.48</td><td>589.3092</td><td>1161.618</td><td>15.9927</td><td>-1.8</td><td>6.1</td><td>32616.5</td><td>5.18</td><td>HUMAN</td><td>A6NL76</td><td>Actin, alpha skeletal muscle</td></tr>
<tr><td>2482</td><td>dirMS_041712_NAR_30mM_minus2.17946.18008.2</td><td>2</td><td>15.58</td><td>0</td><td>2.20E+05</td><td></td><td>(K)EGMNIV</td><td>(K)E G/M/N\I|V\E/A/M/E/R(F)</td><td></td><td>152.3</td><td>639.7955</td><td>1278.582</td><td>0.002</td><td>1.6</td><td>4.25</td><td>18240.4</td><td>7.96</td><td>HUMAN</td><td>P62937</td><td>Peptidyl-prolyl cis-trans isomerase A</td></tr>
<tr><td>2483</td><td>dirMS_041712_NAR_50mM_minus2.13531.13531.3</td><td>3</td><td>15.58</td><td>0</td><td>3.38E+05</td><td></td><td>(K)MLDAED</td><td>(K)M/L|D|A/E|D\I/V N T A R P D E K(A)</td><td></td><td>145.72</td><td>606.2965</td><td>1816.875</td><td>0.0004</td><td>0.2</td><td>4.1</td><td>105308.6</td><td>5.27</td><td>HUMAN</td><td>O43707</td><td>Alpha-actinin-4</td></tr>
<tr><td>2484</td><td>dirMS_041712_NAR_60mM_minus.14267.14267.3</td><td>3</td><td>15.58</td><td>1</td><td>2.87E+05</td><td></td><td>(K)VFGPGIE</td><td>(K)V/F/G/P G/I E/G K D V F R(E)</td><td></td><td>139.95</td><td>474.2636</td><td>1420.758</td><td>0.0179</td><td>12.6</td><td>6.04</td><td>258215.9</td><td>5.48</td><td>HUMAN</td><td>E7EN95</td><td>Filamin-B</td></tr>
<tr><td>2485</td><td>dirMS_041712_NAR_60mM_minus.17868.18131.3</td><td>3</td><td>15.58</td><td>0</td><td>1.33E+06</td><td></td><td>(K)VSHLLGI</td><td>(K)V S H\L\L\G\I|N|V|T/D/F T R(G)</td><td></td><td>161.62</td><td>524.623</td><td>1571.854</td><td>0.0004</td><td>0.3</td><td>6.71</td><td>227783.5</td><td>5.5</td><td>HUMAN</td><td>P35579</td><td>Myosin-9</td></tr>
<tr><td>2486</td><td>dirMS_041712_NAR_20mM_plus.8364.8364.2</td><td>2</td><td>15.58</td><td>0</td><td>4.09E+04</td><td>M105m</td><td>(K)mADEA</td><td>(K)m A\D/E/A|V/C V/G P A/P T S K(S)</td><td>C:Carbamidomethylation m:Oxid</td><td>127.8</td><td>774.8589</td><td>1532.708</td><td>16.0022</td><td>4.7</td><td>4.37</td><td>80685.1</td><td>7.31</td><td>HUMAN</td><td>P05165</td><td>Propionyl-CoA carboxylase alpha chain, mitochondrial</td></tr>
<tr><td>2487</td><td>dirMS_041712_NAR_30mM_plus2.13358.13358.2</td><td>2</td><td>15.58</td><td>0</td><td>5.70E+05</td><td></td><td>(K)CPFTGN</td><td>(K)C/P/F|T/G/N V S I/R(G)</td><td>C:Carbamidomethylation</td><td>122.88</td><td>575.7876</td><td>1150.567</td><td>0.0006</td><td>0.5</td><td>9.75</td><td>18601.6</td><td>10.52</td><td>HUMAN</td><td>P62280</td><td>40S ribosomal protein S11</td></tr>
<tr><td>2488</td><td>dirMS_041712_NAR_30mM_plus2.23513.23513.2</td><td>2</td><td>15.58</td><td>0</td><td>1.80E+05</td><td></td><td>(R)IAIYELLF</td><td>(R)I/A/I\Y/E\L|L F K(E)</td><td></td><td>190.25</td><td>555.3331</td><td>1109.661</td><td>-0.0016</td><td>-1.4</td><td>6</td><td>19924.8</td><td>9.99</td><td>HUMAN</td><td>F6U211</td><td>40S ribosomal protein S10</td></tr>
<tr><td>2489</td><td>dirMS_041712_NAR_30mM_plus2.6114.6114.2</td><td>2</td><td>15.58</td><td>0</td><td>8.14E+05</td><td></td><td>(R)NQGGY</td><td>(R)N/Q|G/G Y\G/G S/S S S S/S Y/G S G R(R)</td><td></td><td>86.18</td><td>847.8524</td><td>1694.7</td><td>-0.0026</td><td>-1.5</td><td>8.59</td><td>33268.7</td><td>9.31</td><td>HUMAN</td><td>F8VRQ1</td><td>Heterogeneous nuclear ribonucleoprotein A1</td></tr>
<tr><td>2490</td><td>dirMS_041712_NAR_30mM_plus2.9987.10063.2</td><td>2</td><td>15.58</td><td>0</td><td>3.63E+05</td><td></td><td>(R)GNIPTLN</td><td>(R)G N|I|P/T/L/N/R(M)</td><td></td><td>105.65</td><td>442.7515</td><td>884.495</td><td>0.0009</td><td>1</td><td>9.75</td><td>267260.7</td><td>5.95</td><td>HUMAN</td><td>Q13085</td><td>Acetyl-CoA carboxylase 1</td></tr>
<tr><td>2491</td><td>dirMS_041712_NAR_40mM_plus2.12896.12916.3</td><td>3</td><td>15.58</td><td>0</td><td>4.96E+05</td><td></td><td>(R)VAPDEH</td><td>(R)V/A|P D E H\P I L/L/T/E A/P L N/P K(I)</td><td></td><td>142.93</td><td>652.0252</td><td>1954.064</td><td>-0.0034</td><td>-1.7</td><td>4.65</td><td>42344.8</td><td>5.39</td><td>HUMAN</td><td>Q562R1</td><td>Beta-actin-like protein 2</td></tr>
<tr><td>2492</td><td>dirMS_041712_NAR_20mM_minus.7310.7310.2</td><td>2</td><td>15.57</td><td>0</td><td>3.99E+04</td><td></td><td>(K)AVTEQG</td><td>(K)A V/T E/Q G/A|E L|S/N E/E/R(N)</td><td></td><td>120.98</td><td>766.8612</td><td>1532.719</td><td>-0.0036</td><td>-2.3</td><td>4.09</td><td>17219.2</td><td>5.69</td><td>HUMAN</td><td>E9PG15</td><td>14-3-3 protein theta (Fragment)</td></tr>
<tr><td>2493</td><td>dirMS_041712_NAR_30mM_minus2.18228.18532.3</td><td>3</td><td>15.57</td><td>0</td><td>5.33E+05</td><td></td><td>(R)QAQEYE</td><td>(R)Q A\Q\E\Y|E|A|L/L/N I K(V)</td><td></td><td>154.7</td><td>473.9196</td><td>1419.748</td><td>-0.0036</td><td>-2.5</td><td>4.53</td><td>43773.3</td><td>5.26</td><td>HUMAN</td><td>F8VZY9</td><td>Keratin, type I cytoskeletal 18</td></tr>
<tr><td>2494</td><td>dirMS_041712_NAR_30mM_minus2.7922.8099.2</td><td>2</td><td>15.57</td><td>0</td><td>1.14E+06</td><td></td><td>(R)SLQAVP</td><td>(R)S/L|Q|A/V/P/G\A K(D)</td><td></td><td>97.65</td><td>435.7524</td><td>870.504</td><td>-0.0068</td><td>-7.8</td><td>8.47</td><td>266161</td><td>5.68</td><td>HUMAN</td><td>E9PPU0</td><td>Epiplakin</td></tr>
<tr><td>2495</td><td>dirMS_041712_NAR_60mM_minus.14783.14783.3</td><td>3</td><td>15.57</td><td>0</td><td>2.66E+05</td><td></td><td>(K)YYITIIDA</td><td>(K)Y Y/I/T I/I/D/A/P/G H R(D)</td><td></td><td>142.87</td><td>473.5815</td><td>1418.743</td><td>-0.0127</td><td>-9</td><td>6.74</td><td>50811.6</td><td>9.32</td><td>HUMAN</td><td>Q05639</td><td>Elongation factor 1-alpha 2</td></tr>
<tr><td>2496</td><td>dirMS_041712_NAR_20mM_plus.10647.10647.2</td><td>2</td><td>15.57</td><td>0</td><td>7.50E+04</td><td></td><td>(K)TTTLSGT</td><td>(K)T T/T L/S/G/T A/P A A G V/V/P S R(V)</td><td></td><td>145.97</td><td>793.4284</td><td>1585.854</td><td>-0.0049</td><td>-3.1</td><td>9.41</td><td>26233.5</td><td>10.95</td><td>HUMAN</td><td>B4DSQ1</td><td>Microtubule-associated protein</td></tr>
<tr><td>2497</td><td>dirMS_041712_NAR_30mM_plus2.9952.9979.2</td><td>2</td><td>15.57</td><td>0</td><td>1.48E+05</td><td></td><td>(R)LTPEEIEI</td><td>(R)L/T|P/E/E/I E/R(M)</td><td></td><td>105.78</td><td>493.7641</td><td>986.515</td><td>0.0056</td><td>5.7</td><td>4.25</td><td>72445.7</td><td>5.07</td><td>HUMAN</td><td>P11021</td><td>78 kDa glucose-regulated protein</td></tr>
<tr><td>2498</td><td>dirMS_041712_NAR_30mM_minus2.6544.6544.2</td><td>2</td><td>15.56</td><td>0</td><td>4.22E+05</td><td></td><td>(K)VLEEEEC</td><td>(K)V/L|E/E/E E Q/R(R)</td><td></td><td>90.3</td><td>516.2552</td><td>1031.5</td><td>0.0027</td><td>2.7</td><td>4.09</td><td>54214.9</td><td>5.66</td><td>HUMAN</td><td>C9J813</td><td>Caldesmon (Fragment)</td></tr>
<tr><td>2499</td><td>dirMS_041712_NAR_40mM_minus2.10966.11252.3</td><td>3</td><td>15.56</td><td>0</td><td>7.92E+05</td><td></td><td>(R)QVSASE</td><td>(R)Q V/S/A/S E L H/T/S/G I/L/G P E/T L R(D)</td><td></td><td>148.75</td><td>665.6822</td><td>1995.051</td><td>-0.0185</td><td>-9.3</td><td>5.4</td><td>558007.1</td><td>5.44</td><td>HUMAN</td><td>P58107</td><td>Epiplakin</td></tr>
<tr><td>2500</td><td>dirMS_041712_NAR_50mM_minus2.10945.11068.3</td><td>3</td><td>15.56</td><td>1</td><td>6.21E+05</td><td></td><td>(R)ISVYYNE</td><td>(R)I S|V/Y/Y/N E A/T/G G K Y V P R(A)</td><td></td><td>131.08</td><td>606.3119</td><td>1816.923</td><td>-0.0017</td><td>-0.9</td><td>8.43</td><td>27549.9</td><td>4.39</td><td>HUMAN</td><td>E9PBJ4</td><td>Tubulin beta chain</td></tr>
<tr><td>2501</td><td>dirMS_041712_NAR_50mM_minus2.8925.8925.3</td><td>3</td><td>15.56</td><td>1</td><td>1.31E+05</td><td>M227m</td><td>(R)mQKEIT.</td><td>(R)m Q K E I T|A|L/A/P S T M K(I)</td><td>m:Oxidized methionine</td><td>119.48</td><td>522.2727</td><td>1548.812</td><td>15.9911</td><td>-2.4</td><td>8.35</td><td>32616.5</td><td>5.18</td><td>HUMAN</td><td>A6NL76</td><td>Actin, alpha skeletal muscle</td></tr>
<tr><td>2502</td><td>dirMS_041712_NAR_120mM_plus.17339.17613.3</td><td>3</td><td>15.56</td><td>1</td><td>6.08E+05</td><td></td><td>(R)RAPFDLI</td><td>(R)R A\P\F|D|L|F/E/N K(K)</td><td></td><td>144.5</td><td>412.8845</td><td>1236.637</td><td>0.0018</td><td>1.5</td><td>6.07</td><td>83605</td><td>4.97</td><td>HUMAN</td><td>P08238</td><td>Heat shock protein HSP 90-beta</td></tr>
</table>

2503	dirMS_041712_NAR_30mM_plus2.16665.17073.2	2	15.56	0	8.08E+05		(K)FSIEDLK	(K)F/S|I/E/D|L/K(A)		141.68	426.2291	851.451	0	0	4.37	13579.2	9.99	HUMAN	E9PPQ6	Thymocyte nuclear protein 1 (Fragment)
2504	dirMS_041712_NAR_30mM_plus2.7673.7804.2	2	15.56	0	1.67E+06		(K)VTLTSEE	(K)V/T|L|T/S/E E E/A R(L)		94.47	567.7813	1134.564	-0.0084	-7.4	4.25	33940.9	9.27	HUMAN	F5H308	L-lactate dehydrogenase
2505	dirMS_041712_NAR_50mM_plus2.19277.19277.3	3	15.56	1	3.94E+05		(K)ILDVASL	(K)I/L|D|V/A/S L/E V L N E V N R R(S)		169.97	614.0109	1840.029	-0.0105	-5.7	4.68	295149.3	7.4	HUMAN	Q92616	Translational activator GCN1
2506	dirMS_041712_NAR_60mM_plus.6676.6741.3	3	15.56	1	4.94E+05		(K)IYEDGD	(K)I/Y|E/D/G D/D D/M/K/R(T)		93.68	452.8641	1356.574	0.0041	3	4.03	21341.9	7.77	HUMAN	Q5R370	Calcyclin binding protein
2507	dirMS_041712_NAR_60mM_plus.7475.7476.3	3	15.56	1	7.87E+06		(R)VITEEEK	(R)V/I/T/E E E/K N F/K(A)		97.9	412.8904	1236.647	0.0096	7.8	4.79	18901.8	11.65	HUMAN	F5H1S2	60S ribosomal protein L13
2508	dirMS_041712_NAR_60mM_minus.11995.12037.3	3	15.55	1	2.62E+05		(R)GLAGLG	(R)G L|A|G/L/G/D/V/A/E V R K(S)		126.95	428.9126	1284.727	-0.0038	-2.9	6.07	97378.8	6.4	HUMAN	P11216	Glycogen phosphorylase, brain form
2509	dirMS_041712_NAR_50mM_plus2.19301.19324.3	3	15.55	0	3.59E+05		(-)MKPLVV	(-)M K P L V V|F|V|L|G/G/P G A G K(G)		169.73	523.9715	1569.919	-0.0186	-11.8	10	22450.2	5.44	HUMAN	P30085	UMP-CMP kinase
2510	dirMS_041712_NAR_50mM_plus2.9884.9884.3	3	15.55	1	1.04E+06		(K)LKGEAT	(K)L K G E A\T\V|S|F D/D/P/P S A K(A)		119.12	554.6155	1661.838	-0.0061	-3.7	4.56	53724.2	9.54	HUMAN	H3BPE7	RNA-binding protein FUS
2511	dirMS_041712_NAR_60mM_plus.11051.11099.3	3	15.55	1	9.67E+05		(R)KTEELEE	(R)K\T E E\L E|E|E/S/F/P E/R(S)		115.75	541.5897	1622.754	0.0001	0.1	4.2	108720.9	10.17	HUMAN	Q9Y2W1	Thyroid hormone receptor-associated protein 3
2512	dirMS_041712_NAR_60mM_plus.15044.15044.3	3	15.55	1	2.99E+04		(R)ELAFQIS	(R)E L/A/F/Q/I/S K E/Y E/R(F)		135.27	504.9258	1512.769	-0.0064	-4.3	4.79	30944.2	6.52	HUMAN	B1Q2N1	ATP-dependent RNA helicase DDX39A
2513	dirMS_041712_NAR_60mM_plus.7002.7017.3	3	15.55	1	2.95E+05		(R)DSGRGD	(R)D S\G R G D S\V|S|D|S/G S/D/A L R(S)		95	560.9181	1680.742	-0.0022	-1.3	4.14	50974.6	5.31	HUMAN	B5ME91	Programmed cell death protein 4
2514	dirMS_041712_NAR_120mM_minus.12012.12073.3	3	15.54	1	2.38E+06		(K)MKEIAE	(K)M/K E I A\E|A/Y/L/G/K(T)		119.63	418.2252	1252.661	0.0005	0.4	5.9	17973	5.16	HUMAN	E9PI65	Heat shock cognate 71 kDa protein (Fragment)
2515	dirMS_041712_NAR_20mM_minus.12366.12386.2	2	15.54	0	5.77E+05	M73m	(R)AVLVDL	(R)A V|L\V|D|L|E|P G T|m D S V R(S)	m:Oxidized methionine	172.48	809.4088	1601.82	15.99	-3	4.03	13112.4	5.23	HUMAN	F8W813	Tubulin beta-8 chain
2516	dirMS_041712_NAR_30mM_minus2.10120.10187.2	2	15.54	0	5.79E+05		(K)TGTVSLE	(K)T/G|T|V/S/L E/V/R(L)		109.52	481.2669	961.531	-0.0048	-5	5.66	276046.1	6.01	HUMAN	P49327	Fatty acid synthase
2517	dirMS_041712_NAR_50mM_minus2.17775.17775.3	3	15.54	0	1.26E+05		(R)CRPDQL	(R)C R\P D Q\L T/G|L/S L/L/P L S E K(A)	C:Carbamidomethylation	170.07	643.0129	1927.032	-0.0076	-3.9	6.07	533778	5.74	HUMAN	Q15149	Plectin
2518	dirMS_041712_NAR_120mM_plus.17857.17857.3	3	15.54	0	7.30E+05		(R)HLLQAP	(R)H L|L Q\A|P V/D D A/Q E/I L/H\S R(F)		146.82	648.0088	1942.014	-0.0023	-1.2	5.21	63613.4	7.9	HUMAN	B3KXI2	Protein transport protein Sec23A
2519	dirMS_041712_NAR_20mM_plus.18934.18934.2	2	15.54	0	2.17E+05		(R)IITLAGP	(R)I/I|T L/A/G/P T N A I F K(A)		201.03	679.9039	1358.804	-0.0037	-2.7	8.75	32621	8.75	HUMAN	B4DLC0	Poly(rC)-binding protein 2
2520	dirMS_041712_NAR_30mM_plus2.18244.18512.2	2	15.54	0	2.76E+05		(R)TEMDW	(R)T/E|M/D/W/V/L/K(H)		149.6	511.2577	1021.502	0.0058	5.7	4.37	46013.4	5.38	HUMAN	P52597	Heterogeneous nuclear ribonucleoprotein F
2521	dirMS_041712_NAR_40mM_plus2.13757.13757.3	3	15.54	1	3.43E+05		(R)QVFGEA	(R)Q V|F/G/E A T K Q/P G I T F I A A K(F)		147.45	636.0176	1906.043	-0.005	-2.6	8.59	32905.8	5.88	HUMAN	C9JH19	Cathepsin D light chain (Fragment)
2522	dirMS_041712_NAR_30mM_minus2.11322.11322.2	2	15.53	0	1.40E+05		(R)AATSPA	(R)A A|T\S/P/A L F N R(C)		115.4	524.2789	1047.558	-0.0076	-7.3	9.79	535136.6	6.01	HUMAN	Q14204	Cytoplasmic dynein 1 heavy chain 1
2523	dirMS_041712_NAR_50mM_minus2.15934.16407.3	3	15.53	1	3.24E+07	M148m	(R)LDLAGR	(R)L/D|L|A/G R D L/T/D/Y L/m K(I)	m:Oxidized methionine	160.03	547.2838	1623.841	15.9958	0.5	4.43	37482.1	5.36	HUMAN	E9PG30	Actin, gamma-enteric smooth muscle
2524	dirMS_041712_NAR_120mM_plus.7648.7817.3	3	15.53	0	5.16E+05		(R)SGSMDF	(R)S G|S|M/D/P/S G A/H P S V/R(Q)		94.08	462.218	1384.627	0.0121	8.7	6.46	48340.6	8.84	HUMAN	Q07666	KH domain-containing, RNA-binding, signal transduction-associated protein 1
2525	dirMS_041712_NAR_30mM_plus2.8262.8262.2	2	15.53	0	2.73E+05		(K)EIAENAL	(K)E I|A/E/N A/L/G/K(H)		96.92	472.755	944.505	-0.002	-2.1	4.53	36982.9	6.37	HUMAN	P31942	Heterogeneous nuclear ribonucleoprotein H3
2526	dirMS_041712_NAR_50mM_plus2.10365.10677.3	3	15.53	1	2.20E+07		(R)DGKLVS	(R)D G K\L\V\S\E|S/S/D/V/L/P/K(-)		122.83	491.9313	1473.78	-0.0002	-0.1	4.56	56607.4	5.37	HUMAN	F8VXB4	Keratin, type II cytoskeletal 8
2527	dirMS_041712_NAR_60mM_plus.13209.13605.3	3	15.53	1	1.02E+06		(R)ISVYYNE	(R)I/S|V/Y/Y N/E A/T/G G K/Y V/P R(A)		125.95	606.3113	1816.923	-0.0035	-1.9	8.43	27549.9	4.39	HUMAN	E9PBJ4	Tubulin beta chain
2528	dirMS_041712_NAR_50mM_minus2.18364.18364.3	3	15.52	0	4.62E+05		(K)ALIEVLQ	(K)A L|I/E/V L Q/P L/I A E H Q A R(R)		175.42	601.0139	1801.033	-0.0059	-3.3	5.4	53506.8	5.83	HUMAN	P23381	Tryptophan--tRNA ligase, cytoplasmic
2529	dirMS_041712_NAR_20mM_plus.18985.19149.3	3	15.52	0	1.11E+05		(R)VCTLAII	(R)V C T L\A I\I/D|P/G/D/S D I I R(S)	C:Carbamidomethylation	202.28	586.641	1757.91	-0.0018	-1	3.93	12826.8	9.82	HUMAN	E5RI99	60S ribosomal protein L30 (Fragment)
2530	dirMS_041712_NAR_30mM_plus2.16988.17489.2	2	15.52	0	3.76E+06		(K)MIFVGI	(K)M/I|F/V/G/I/K(K)		144.32	404.2433	807.48	-0.0004	-0.5	8.5	11447.2	9.81	HUMAN	C9JFR7	Cytochrome c (Fragment)
2531	dirMS_041712_NAR_60mM_plus.17877.17971.3	3	15.52	1	1.04E+06		(R)ARFEEL	(R)A R F E\E\L|N|A/D/L/F/R(G)		150.85	494.2554	1480.754	-0.0027	-1.8	4.68	45508.6	5.5	HUMAN	A8K7Q2	Heat shock cognate 71 kDa protein
2532	dirMS_041712_NAR_60mM_plus.20562.20664.3	3	15.52	1	7.14E+05		(R)LDLAGR	(R)L/D|L|A/G R D L/T/D/Y L/M/K(I)		168.28	541.9516	1623.841	-0.0008	-0.5	4.43	37482.1	5.36	HUMAN	E9PG30	Actin, gamma-enteric smooth muscle
2533	dirMS_041712_NAR_60mM_plus.7940.8003.3	3	15.52	1	3.68E+05		(K)DISTNYY	(K)D I|S|T/N/Y/Y/A S Q K K(T)		99.77	473.2367	1417.696	-0.0002	-0.2	8.43	17045.4	4.25	HUMAN	H0YIV0	Endoplasmin (Fragment)
2534	dirMS_041712_NAR_20mM_minus.13781.13781.2	2	15.51	0	2.36E+05		(R)IVLLDSS	(R)I V|L/L/D/S S L\E Y K(K)		189.15	640.3594	1279.714	-0.0029	-2.2	4.37	56186.7	5.47	HUMAN	B4DUR8	T-complex protein 1 subunit gamma
2535	dirMS_041712_NAR_20mM_plus.18969.18969.2	2	15.51	0	2.13E+04		(K)LLVYEAL	(K)L/L|V/Y/E A/L E|Y A/K(R)		201.67	656.3664	1311.719	0.006	4.6	4.53	32535.6	9.19	HUMAN	B4DZW6	Regulator of microtubule dynamics protein 1
2536	dirMS_041712_NAR_30mM_plus2.21578.21578.2	2	15.51	0	3.32E+05		(K)FDGILG	(K)F/D|G I/L|G/M A\Y P R(I)		167.47	620.3199	1239.619	0.0135	10.9	5.83	32905.8	5.88	HUMAN	C9JH19	Cathepsin D light chain (Fragment)
2537	dirMS_041712_NAR_40mM_plus2.18463.18463.3	3	15.51	1	2.56E+04		(R)IAEVGG	(R)I A/E/V/G/G V/P Y L L P L V N Q K K(V)		174.58	646.716	1938.142	-0.0088	-4.5	8.5	18803.3	5.55	HUMAN	E9PIP1	Ester hydrolase C11orf54 (Fragment)
2538	dirMS_041712_NAR_60mM_plus.10080.10330.2	2	15.51	0	2.03E+06		(K)GIVEFSG	(K)G I|V/E/F/S G/K/P A A/R(K)		111.3	616.3425	1231.679	-0.0016	-1.3	8.75	23729.5	9.89	HUMAN	C9IZL7	Non-POU domain-containing octamer-binding protein (Fragment)
2539	dirMS_041712_NAR_120mM_minus.10162.10162.3	3	15.5	1	1.07E+06		(K)IFRDGEE	(K)I/F R D G E E\A|G|A/Y/D/G P R(T)		110.25	551.5928	1652.766	-0.0025	-1.5	4.32	55361.8	6.42	HUMAN	G5EA52	Protein disulfide isomerase family A, member 3, isoform CRA_b
2540	dirMS_041712_NAR_120mM_minus.11184.11184.3	3	15.5	0	2.93E+05		(K)SAKPTK	(K)S A/K/P T K\P A A/S D|L|P V|P A E G V R(N)		115.03	664.3662	1991.092	-0.008	-4	8.31	54214.9	5.66	HUMAN	C9J813	Caldesmon (Fragment)
2541	dirMS_041712_NAR_30mM_minus2.6878.6902.2	2	15.5	0	1.55E+05	M697m	(K)EGALCE	(K)E G A|L/C|E/E/N\m R(G)	C:Carbamidomethylation m:Oxid	91.9	612.7538	1208.503	15.9969	1.6	4.25	96306.4	6.41	HUMAN	P13639	Elongation factor 2
2542	dirMS_041712_NAR_30mM_plus2.14243.14466.2	2	15.5	0	5.73E+05		(K)CCSGAI	(K)C C|S|G/A|I/I V|L/T/K(S)	C:Carbamidomethylation	128.2	611.3189	1221.633	-0.0025	-2	8.75	58506.4	8.38	HUMAN	P14618	Pyruvate kinase isozymes M1/M2
2543	dirMS_041712_NAR_30mM_plus2.16007.16007.2	2	15.5	0	5.88E+05		(R)YLCGLG	(R)Y L/C/G/L/G A V G G V/R(L)	C:Carbamidomethylation	137.22	611.3189	1221.641	-0.0103	-8.5	8.75	266161	5.68	HUMAN	E9PPU0	Epiplakin
2544	dirMS_041712_NAR_40mM_plus2.11023.11023.3	3	15.5	1	6.32E+04		(K)VAVVAG	(K)V A|V/V/A|G Y G D V/G K G C A Q A L R(G)	C:Carbamidomethylation	131.05	630.9981	1890.985	-0.0057	-3	8.56	45228.3	6.03	HUMAN	F5H737	Adenosylhomocysteinase
2545	dirMS_041712_NAR_60mM_plus.12372.12372.3	3	15.5	1	9.36E+05		(R)LSFDKD	(R)L/S/F D/K D A/M/V A R(A)		121.18	418.2158	1252.635	-0.0026	-2.1	5.96	35556.4	9.3	HUMAN	F2Z393	Transaldolase
2546	dirMS_041712_NAR_120mM_minus.17386.17443.3	3	15.49	1	3.94E+05		(R)RTVQSLI	(R)R T V Q\S\L|E|I|D|L/D/S M R(N)		149.48	554.9552	1662.848	0.0031	1.9	4.56	43773.3	5.26	HUMAN	F8VZY9	Keratin, type I cytoskeletal 18
2547	dirMS_041712_NAR_20mM_minus.9816.9887.2	2	15.49	0	8.73E+05		(R)IVLQIDN	(R)I V|L|Q/I/D/N/A R(L)		149.7	521.3059	1041.605	-0.0006	-0.6	5.84	20842.9	5.32	HUMAN	C9JM50	Keratin, type I cytoskeletal 19 (Fragment)
2548	dirMS_041712_NAR_50mM_minus2.14501.14501.3	3	15.49	1	2.03E+05		(R)IIDSLFN	(R)I/I/D S L/F/N T V/T D K K(I)		150.6	498.612	1493.821	0.0005	0.3	5.96	44813.4	7.79	HUMAN	B4DN25	UDP-glucose 6-dehydrogenase
2549	dirMS_041712_NAR_120mM_plus.6259.6364.3	3	15.49	1	5.04E+05		(R)ARSDEG	(R)A R S D E/G\Q|L|S|P/A T/R(G)		86.7	463.2395	1387.692	0.0115	8.3	6.12	81007.1	6.31	HUMAN	E9PMV1	Plectin (Fragment)
2550	dirMS_041712_NAR_20mM_plus.14735.14735.2	2	15.49	0	1.06E+05		(K)LLGELLQ	(K)L L/G E|L/L Q/D N A K(L)		173.62	607.3421	1213.679	-0.0018	-1.4	4.37	35556.4	9.3	HUMAN	F2Z393	Transaldolase
2551	dirMS_041712_NAR_30mM_plus2.18953.18953.2	2	15.49	0	3.33E+05		(R)LTAEDLF	(R)L/T|A/E|D\L/F/E A R(I)		153.07	582.796	1164.59	-0.0048	-4.1	4.14	533778	5.74	HUMAN	Q15149	Plectin
2552	dirMS_041712_NAR_40mM_plus2.10704.10704.3	3	15.49	0	1.85E+05		(K)VGSLDN	(K)V/G|S L/D\N V G/H L/P A G G A V K(I)		128.15	530.9567	1590.86	-0.0043	-2.7	6.71	26233.5	10.95	HUMAN	B4DSQ1	Microtubule-associated protein
2553	dirMS_041712_NAR_50mM_plus2.15478.15478.3	3	15.49	1	8.15E+05		(K)EAFSLFD	(K)E A|F S/L/F/D/K D G D G/T I/T T K(E)		149.52	615.6348	1844.891	-0.0014	-0.8	4.23	21860.1	4.45	HUMAN	E7EMB3	Calmodulin
2554	dirMS_041712_NAR_30mM_minus2.14878.14924.2	2	15.48	0	1.75E+05		(K)SLAEGY	(K)S L|A|E|G/Y/F/D/A A G R(L)		134.77	628.7988	1256.591	-0.0003	-0.2	4.37	22270.1	8.67	HUMAN	E9PHS0	LanC-like protein 1 (Fragment)
2555	dirMS_041712_NAR_30mM_minus2.7345.7368.2	2	15.48	0	1.25E+05		(K)VQYPQS	(K)V Q|Y|P Q/S Q/A/C/K(M)	C:Carbamidomethylation	94.43	604.7902	1208.573	0.0003	0.2	8.56	535136.6	6.01	HUMAN	Q14204	Cytoplasmic dynein 1 heavy chain 1
2556	dirMS_041712_NAR_120mM_plus.8887.8887.2	2	15.48	0	5.69E+05		(R)NGVMP	(R)N G V|M/P S H F S R(G)		99.85	566.274	1131.536	0.0043	3.8	9.75	16060.2	10.31	HUMAN	P39019	40S ribosomal protein S19
2557	dirMS_041712_NAR_30mM_plus2.16303.16303.2	2	15.48	0	1.18E+06		(R)FGLNVS	(R)F/G/L|N/V/S/S I S R(K)		139.02	540.3016	1079.584	0.0115	10.7	9.75	24161.9	5.27	HUMAN	F8VZQ9	SAP domain-containing ribonucleoprotein
2558	dirMS_041712_NAR_30mM_plus2.17254.17798.2	2	15.48	0	1.17E+06		(R)IMNTFS	(R)I M|N|T|F|S V/V/P S/P K(V)		145.38	660.3537	1319.703	-0.0027	-2	8.75	42831.5	4.86	HUMAN	A8K854	HCG1983504, isoform CRA_f
2559	dirMS_041712_NAR_120mM_minus.19438.19439.3	3	15.47	1	3.72E+05		(K)NLRDID	(K)N L/R D I D\E|V|S/S/L/L R(T)		161.42	510.614	1529.828	-0.0007	-0.5	4.56	58214.7	5.25	HUMAN	B4DEM7	T-complex protein 1 subunit theta
2560	dirMS_041712_NAR_20mM_minus.10310.10310.2	2	15.47	0	7.05E+04		(K)AVLSLY	(K)A V|L/S|L/Y A/S G R(T)		152.45	518.7929	1036.579	0	0	8.79	14579.3	5.13	HUMAN	K7EM38	Actin, cytoplasmic 2, N-terminally processed (Fragment)
2561	dirMS_041712_NAR_30mM_minus2.17821.17821.2	2	15.47	0	3.07E+05		(K)EAFQLF	(K)E A/F|Q/L/F D/R(T)		151.65	513.2571	1025.505	0.0019	1.8	4.37	26991.7	5.01	HUMAN	B7Z6Z4	Myosin light polypeptide 6
2562	dirMS_041712_NAR_60mM_minus.14849.14887.3	3	15.47	1	2.72E+05		(R)KLQEEE	(R)K L Q E/E E\L\L|A|T/Q/V/D\M R(A)		142.8	601.6438	1802.932	-0.0148	-8.2	4.41	35935.5	6.2	HUMAN	Q8IVM0	Coiled-coil domain-containing protein 50
2563	dirMS_041712_NAR_120mM_plus.12384.12541.4	4	15.47	2	9.28E+05		(K)KGTVEG	(K)K G T V E G\F\E|P/A/D/N K/C/L L R(A)	C:Carbamidomethylation	118.9	484.2468	1933.98	-0.0147	-7.6	6.18	13286.2	9.99	HUMAN	H0YLA2	Signal recognition particle 14 kDa protein
2564	dirMS_041712_NAR_20mM_plus.16430.16430.2	2	15.47	0	1.63E+05		(R)LLEAQIA	(R)L L|E\A Q/I|A T/G/G/I I/D/P K(E)		185	769.9362	1538.879	-0.0137	-8.9	4.37	334221.3	6.44	HUMAN	P15924	Desmoplakin
2565	dirMS_041712_NAR_40mM_plus2.14766.14874.3	3	15.47	0	3.03E+05		(R)IEGDMI	(R)I/E|G D\M I/V/C/A A Y A H E L/P K(Y)	C:Carbamidomethylation	153.93	639.6498	1916.925	0.0104	5.4	4.65	34590.2	9.84	HUMAN	P46777	60S ribosomal protein L5
2566	dirMS_041712_NAR_60mM_plus.10477.10477.2	2	15.47	1	4.41E+06		(R)LQAEIEG	(R)L/Q|A/E/I\E/G\L K/G Q/R(A)		112.33	671.3774	1341.748	-0.001	-0.7	6.14	56607.4	5.37	HUMAN	F8VXB4	Keratin, type II cytoskeletal 8
2567	dirMS_041712_NAR_30mM_minus2.11353.11353.2	2	15.46	0	1.22E+06		(K)FSASGE	(K)F S|A/S|G E|L/G N/G N I/K(L)		114.95	647.3253	1293.643	0	0	6	29110.6	4.57	HUMAN	P12004	Proliferating cell nuclear antigen
2568	dirMS_041712_NAR_50mM_minus2.14289.14481.3	3	15.46	0	3.62E+05		(K)LHIIEVG	(K)L H I\I E V\G\T|P/P/T/G N Q/P F/P K(K)		150.02	649.0215	1945.054	-0.0042	-2.2	6.75	193379.8	5.48	HUMAN	Q00610	Clathrin heavy chain 1
2569	dirMS_041712_NAR_60mM_minus.10091.10091.3	3	15.46	0	1.56E+06		(R)EIVHIQA	(R)E/I V H I Q\A\G/Q/C/G/N Q\I/G A/K(F)	C:Carbamidomethylation	115.57	608.3099	1822.923	-0.0077	-4.2	6.85	40069.4	4.83	HUMAN	E7EWR1	Tubulin beta chain
2570	dirMS_041712_NAR_60mM_minus.6559.6559.3	3	15.46	1	9.46E+04	M436m	(R)TPGKEA	(R)T/P/G K E A V A m/E/S Y/A/K(A)	m:Oxidized methionine	95.08	499.9146	1481.73	15.9988	2.6	5.81	57204.9	6.01	HUMAN	F5GWF6	T-complex protein 1 subunit beta
2571	dirMS_041712_NAR_120mM_plus.11710.12026.3	3	15.46	0	2.30E+05		(K)VYNYNH	(K)V/Y/N/Y/N/H/L/M/P T|R(Y)		114.35	469.8997	1407.684	0.0008	0.5	8.57	16471.9	10.59	HUMAN	K7ELC7	60S ribosomal protein L27 (Fragment)
2572	dirMS_041712_NAR_20mM_plus.11340.11340.2	2	15.46	0	2.96E+05		(K)LITPAVV	(K)L I/T|P A V V/S\E R(L)		150.53	542.8186	1084.636	-0.0062	-5.7	6	13798.9	10.17	HUMAN	P62851	40S ribosomal protein S25
2573	dirMS_041712_NAR_30mM_plus2.6215.6256.2	2	15.46	0	3.51E+05		(K)NQCTQ	(K)N|Q|C|T|Q V/V/Q/E/R(E)	C:Carbamidomethylation	87	631.3039	1261.595	0.0052	4.1	6	334221.3	6.44	HUMAN	P15924	Desmoplakin
2574	dirMS_041712_NAR_40mM_plus2.10387.10558.2	2	15.46	0	4.25E+05		(R)YLYEIAR	(R)Y/L|Y/E/I/A/R(R)		126.83	464.2502	927.493	-0.0003	-0.3	6	53112	6.64	HUMAN	H0YA55	Serum albumin (Fragment)
2575	dirMS_041712_NAR_40mM_plus2.9027.9027.2	2	15.46	0	8.93E+04		(K)GSDFDC	(K)G S|D/F/D/C E/L/R(L)	C:Carbamidomethylation	115.47	549.7296	1098.452	-0.0001	-0.1	4.03	51260.6	5.39	HUMAN	P61978	Heterogeneous nuclear ribonucleoprotein K
2576	dirMS_041712_NAR_50mM_plus2.13234.13254.3	3	15.46	0	2.27E+06		(R)SCTVNI	(R)S C|T|V/N I/M|F|G/T/P\Q E H R(N)	C:Carbamidomethylation	138.43	592.9438	1776.816	0.0012	0.7	6.47	65763.5	9.81	HUMAN	Q9HCD5	Nuclear receptor coactivator 5
2577	dirMS_041712_NAR_50mM_plus2.8947.8947.3	3	15.46	0	6.04E+05		(R)AALAHS	(R)A A|L A H S/E E\V\T|A/S/Q/V/A A/T K(T)		113.75	595.312	1783.919	0.003	1.7	5.4	23903	4.94	HUMAN	H0YDN1	Plectin (Fragment)
2578	dirMS_041712_NAR_60mM_plus.9665.9952.3	3	15.46	0	4.99E+06		(R)QSVEND	(R)Q/S/V/E N D/I/H/G L/R(K)		109.7	423.2195	1267.639	0.005	4	5.32	43773.3	5.26	HUMAN	F8VZY9	Keratin, type I cytoskeletal 18
2579	dirMS_041712_NAR_120mM_minus.11929.11947.4	4	15.45	2	6.76E+05		(K)GTVIKVF	(K)G T|V I\K V/P L/K V/E Q/A/N/N A R(D)		119.27	460.021	1837.065	-0.0032	-1.8	9.99	150202.8	9.1	HUMAN	E7EW20	Unconventional myosin-VI
2580	dirMS_041712_NAR_120mM_minus.14733.14900.3	3	15.45	0	1.01E+06		(-)MVNHFI	(-)M/V/N/H/F|I A E/F/K(R)		134.9	412.5458	1235.624	-0.0013	-1	6.5	45508.6	5.5	HUMAN	A8K7Q2	Heat shock cognate 71 kDa protein
2581	dirMS_041712_NAR_30mM_minus2.13577.13909.2	2	15.45	0	1.22E+06		(K)VMYGF	(K)V/M|Y/G/F/C/S V/K(D)	C:Carbamidomethylation	127.8	545.7611	1090.506	0.0089	8.2	8.56	76925.9	4.46	HUMAN	A8MV58	Drebrin
2582	dirMS_041712_NAR_30mM_minus2.13976.13976.2	2	15.45	0	1.66E+05		(K)NVIGLQ	(K)N V|I|G\L/Q\M G T N R(G)		129.38	601.8183	1202.631	-0.0017	-1.4	9.75	22562.2	8.8	HUMAN	P37802	Transgelin-2
2583	dirMS_041712_NAR_60mM_minus.14288.14529.3	3	15.45	0	3.65E+05		(R)VWYVSN	(R)V W|Y|V/S N I/D\G T H I A K(T)		140.38	534.9461	1602.828	-0.0037	-2.3	6.71	63374.2	8.64	HUMAN	P06744	Glucose-6-phosphate isomerase
2584	dirMS_041712_NAR_120mM_plus.23690.23690.3	3	15.45	0	7.83E+05		(R)LHFFMP	(R)L H F F\M\P G\F|A/P\L/T S R(G)		179.12	540.9492	1620.835	-0.0025	-1.5	9.75	42230.8	4.77	HUMAN	A6NKZ8	Putative tubulin beta chain-like protein ENSP00000290377
2585	dirMS_041712_NAR_30mM_plus2.13433.13433.2	2	15.45	0	2.78E+05		(K)SDFQVN	(K)S D F Q|V|N L/N N/A S R(S)		123.47	682.8318	1364.655	0.001	0.7	5.55	105417.2	6.41	HUMAN	C9JZR2	Catenin delta-1
2586	dirMS_041712_NAR_50mM_plus2.10850.10872.3	3	15.45	0	4.89E+05		(R)EALLSSA	(R)E A\L/L/S/S/A|V|D|H G S D E/V K(F)		124.7	552.9403	1656.807	-0.0012	-0.7	4.31	57204.9	6.01	HUMAN	F5GWF6	T-complex protein 1 subunit beta
2587	dirMS_041712_NAR_20mM_minus.13206.13206.3	3	15.44	0	4.00E+05		(K)TITLEVE	(K)T\I\T L E V E/P S/D/T I E/N V K(A)		183.03	596.6453	1787.927	-0.006	-3.3	4	17271.6	6.75	HUMAN	B4DV12	Ubiquitin
2588	dirMS_041712_NAR_20mM_minus.8147.8188.2	2	15.44	0	6.38E+06	M239m	(K)EITALAP	(K)E I|T/A|L/A|P/S T m K(I)	m:Oxidized methionine	132.57	589.3094	1161.618	15.9931	-1.5	6.1	32616.5	5.18	HUMAN	A6NL76	Actin, alpha skeletal muscle
2589	dirMS_041712_NAR_40mM_minus2.8850.8850.3	3	15.44	1	2.33E+05		(R)IVLAND	(R)I/V|L/A/N D P D A D R L A V A E K(Q)		134.3	603.9966	1809.97	0.0047	2.6	4.23	32837.5	6.16	HUMAN	B4E0G8	Phosphoglucomutase-2
2590	dirMS_041712_NAR_50mM_minus2.12270.12330.3	3	15.44	1	3.32E+05		(R)DGRGAL	(R)D G R G A L Q\N\I|I|P/A/S T G A A K(A)		138.33	580.6487	1739.94	-0.0083	-4.8	8.75	31718.5	7.17	HUMAN	E7EUT4	Glyceraldehyde-3-phosphate dehydrogenase
2591	dirMS_041712_NAR_60mM_minus.10397.10397.3	3	15.44	1	6.05E+05	M88m	(K)DVDEAY	(K)D V|D/E/A|Y m N/K V E L E S R(L)	m:Oxidized methionine	118.37	605.2791	1797.832	15.9904	-2.5	4.18	13588.1	4.66	HUMAN	F8VP67	Keratin, type II cytoskeletal 8 (Fragment)
2592	dirMS_041712_NAR_60mM_minus.18962.18962.3	3	15.44	1	1.98E+05		(K)ITQDIFQ	(K)I/T|Q/D I/F/Q Q L L K R(G)		167.82	501.6268	1502.869	-0.0031	-2.1	8.75	72796.9	6.75	HUMAN	A6NC17	Methionine--tRNA ligase, cytoplasmic
2593	dirMS_041712_NAR_120mM_plus.6453.6592.3	3	15.44	1	1.74E+07		(R)TKTEISE	(R)T\K\T E\I/S/E/M/N/R(N)		88.12	403.5365	1208.594	0.001	0.8	5.81	37296.3	5.3	HUMAN	F8VU64	Keratin, type II cytoskeletal 8
2594	dirMS_041712_NAR_120mM_plus.9686.9686.3	3	15.44	0	6.06E+05		(K)STAGDT	(K)S T|A/G D/T H L/G G E|D F D/N R(M)		104.03	564.579	1691.726	-0.0031	-1.9	4.22	69032.8	5.37	HUMAN	E9PKE3	Heat shock cognate 71 kDa protein
2595	dirMS_041712_NAR_20mM_plus.18808.18808.2	2	15.44	0	2.08E+05		(K)TAVETA	(K)T\A|V E|T/A/V/L\L/L/R(I)		200.28	593.3625	1185.72	-0.0024	-2	5.66	56186.7	5.47	HUMAN	B4DUR8	T-complex protein 1 subunit gamma
2596	dirMS_041712_NAR_30mM_plus2.19531.19531.2	2	15.44	0	2.30E+04		(K)NQALFP	(K)N Q|A|L|F/P A C V L K(N)	C:Carbamidomethylation	155.17	630.8399	1260.677	-0.0044	-3.5	8.75	83400.6	6.82	HUMAN	Q92499	ATP-dependent RNA helicase DDX1
2597	dirMS_041712_NAR_60mM_plus.12065.12220.3	3	15.44	1	2.25E+06		(K)GCGVVK	(K)G C|G V/V/K/F E\S|P/E/V/A E R(A)	C:Carbamidomethylation	120.45	555.2752	1663.811	0.0002	0.1	4.79	77799.7	9.09	HUMAN	P52272	Heterogeneous nuclear ribonucleoprotein M
2598	dirMS_041712_NAR_60mM_plus.5672.5672.3	3	15.44	0	1.11E+06		(K)TATPQQ	(K)T/A|T|P/Q Q A/Q E V H E K(L)		87.6	489.5727	1466.723	-0.0198	-13.5	5.37	31075.7	5.65	HUMAN	P60174	Triosephosphate isomerase
2599	dirMS_041712_NAR_120mM_plus.16796.16943.4	4	15.43	1	3.13E+05		(R)GHYTIGI	(R)G H\Y\T I G/K E I\I\D|P|V/L/D/R(I)		141.77	457.2493	1825.981	-0.0053	-2.9	5.38	49012.3	4.9	HUMAN	A8MUB1	Tubulin alpha-4A chain
2600	dirMS_041712_NAR_30mM_plus2.18637.18881.2	2	15.43	0	5.93E+05		(R)GLFIIDD	(R)G L|F|I|I/D/D/K(G)		151.82	460.7596	920.509	0.0032	3.4	4.21	18498.6	5.82	HUMAN	H7C3T4	Peroxiredoxin-4 (Fragment)
2601	dirMS_041712_NAR_30mM_plus2.20368.20368.2	2	15.43	0	1.80E+05		(K)GSFSEQ	(K)G S/F/S/E/Q/G I|N E F\L R(E)		161	742.362	1483.718	-0.0009	-0.6	4.53	53659.5	5.35	HUMAN	B5MCQ5	Protein disulfide-isomerase A6
2602	dirMS_041712_NAR_50mM_plus2.9503.9799.3	3	15.43	1	1.99E+05		(R)AGTGVD	(R)A G/T/G/V/D N V/D L/E/A A/T R K(G)		118.75	539.6108	1616.824	-0.006	-3.7	4.56	57391.2	6.29	HUMAN	O43175	D-3-phosphoglycerate dehydrogenase
2603	dirMS_041712_NAR_30mM_minus2.16043.16492.2	2	15.42	0	6.11E+05		(R)DFFLAN	(R)D F|F|L\A|N/A|S/R(A)		142.12	520.763	1040.516	0.0028	2.6	5.84	82516.1	5.49	HUMAN	P07384	Calpain-1 catalytic subunit
2604	dirMS_041712_NAR_50mM_minus2.12161.12208.3	3	15.42	1	2.66E+05		(K)LAEIGA	(K)L/A\E|I|G/A/P I/Q/G N/R E E L V E R(L)		137.77	665.3584	1994.066	-0.0059	-2.9	4.49	38572.2	6.31	HUMAN	E9PJ04	Splicing factor 3B subunit 2 (Fragment)
2605	dirMS_041712_NAR_50mM_minus2.6705.6705.3	3	15.42	1	2.52E+05		(R)ASVDTK	(R)A S/V D/T/K E A E/G A/P Q/V E|A G K(R)		104.8	596.2904	1786.882	-0.0251	-14	4.41	54214.9	5.66	HUMAN	C9J813	Caldesmon (Fragment)
2606	dirMS_041712_NAR_60mM_minus.15260.15282.3	3	15.42	1	1.56E+06		(R)TIPWLE	(R)T I|P/W/L/E/D R V/P Q/K(T)		145.47	494.6084	1481.811	-0.0004	-0.3	5.74	80254.2	5.05	HUMAN	D6PXK4	Alpha actinin 4 short isoform
2607	dirMS_041712_NAR_20mM_plus.15584.15584.3	3	15.42	0	2.07E+05		(K)TITLEVE	(K)T I\T L E\V\E/P S/D/T I/E N V K(A)		179.07	596.6463	1787.927	-0.003	-1.7	4	17271.6	6.75	HUMAN	B4DV12	Ubiquitin
2608	dirMS_041712_NAR_30mM_plus2.20352.20584.2	2	15.42	0	3.06E+05		(R)YISPDQL	(R)Y I|S|P/D Q/L/A D L/Y K(S)		161.52	713.3669	1425.726	0.0005	0.4	4.21	47510.4	7.04	HUMAN	P06733	Alpha-enolase
2609	dirMS_041712_NAR_50mM_plus2.12491.12491.3	3	15.42	0	3.93E+05		(R)LDDTVH	(R)L/D/D T V/H/V/V/I/A T\P G R(I)		133.35	498.2741	1492.812	-0.0041	-2.7	5.21	54815.2	9.17	HUMAN	P26196	Probable ATP-dependent RNA helicase DDX6
2610	dirMS_041712_NAR_20mM_minus.10600.10600.2	2	15.41	0	2.00E+05		(R)TTPSYV	(R)T T/P S/Y V/A/F T D/T E R(L)		154.63	744.3547	1487.701	0.0009	0.6	4.37	58502.6	6.09	HUMAN	B4DXY3	Heat shock 70 kDa protein 1-like
2611	dirMS_041712_NAR_20mM_minus.11232.11473.2	2	15.41	0	1.17E+07	M148m	(R)DLTDYL	(R)D/L|T|D|Y/L/m/K(I)	m:Oxidized methionine	162.17	507.7443	998.486	15.995	0.1	4.21	37482.1	5.36	HUMAN	E9PG30	Actin, gamma-enteric smooth muscle

2612	dirMS_041712_NAR_20mM_plus.12490.12490.2	2	15.41	0	1.78E+05		(R)GVEICIA	(R)G V E|I/C/I A/T/P/G R(L)	C:Carbamidomethylation	158.8	586.8084	1172.609	0.0003	0.3	6	61018.6	9.44	HUMAN	B4DLW8	Probable ATP-dependent RNA helicase DDX5
2613	dirMS_041712_NAR_30mM_plus2.19264.19756.2	2	15.41	0	2.25E+06		(R)ALLFIPR	(R)A L|L|F|I/P/R(R)		156.72	415.2703	829.529	0.0039	4.7	9.79	83605	4.97	HUMAN	P08238	Heat shock protein HSP 90-beta
2614	dirMS_041712_NAR_30mM_plus2.6885.6927.2	2	15.41	0	3.28E+05		(R)FSNISAA	(R)F/S|N/I/S/A/A|K(A)		90.63	419.2286	837.447	0.0034	4.1	8.75	55232.1	8.4	HUMAN	F5H5W3	T-complex protein 1 subunit delta
2615	dirMS_041712_NAR_50mM_plus2.14992.14992.3	3	15.41	1	1.14E+05		(R)LLDAQL	(R)L/L|D A/Q|L|A T G G L V C/P A/R R(L)	C:Carbamidomethylation	147.2	604.3351	1810.996	-0.0049	-2.7	9.6	266161	5.68	HUMAN	E9PPU0	Epiplakin
2616	dirMS_041712_NAR_50mM_plus2.18688.18688.3	3	15.41	1	5.80E+05		(R)YLTAEET	(R)Y/L|T/A/E/E T D K I/I N Y L R(E)		166.65	614.662	1841.964	0.0071	3.8	4.68	65763.5	9.81	HUMAN	Q9HCD5	Nuclear receptor coactivator 5
2617	dirMS_041712_NAR_60mM_plus.10838.10960.3	3	15.41	1	6.41E+06		(R)QQEGFK	(R)Q Q/E G F K/G T/F/P/D|A/R(E)		115.52	494.2424	1480.718	-0.0053	-3.6	6.07	43979.5	8.92	HUMAN	F5GYZ3	Non-POU domain-containing octamer-binding protein
2618	dirMS_041712_NAR_60mM_plus.6130.6130.3	3	15.41	1	4.67E+04		(R)ALCDYK	(R)A/L/C D/Y K/Q D Q/K(I)	C:Carbamidomethylation	90.68	423.5361	1268.594	-0.0002	-0.2	6.01	334221.3	6.44	HUMAN	P15924	Desmoplakin
2619	dirMS_041712_NAR_60mM_plus.8351.8351.3	3	15.41	1	2.74E+05		(R)NVDSSG	(R)N/V|D/S/S/G N K S V/L/M E R(L)		102.1	512.5887	1535.748	0.0033	2.2	6.07	103309.5	5.34	HUMAN	A0AV56	SAFB protein
2620	dirMS_041712_NAR_120mM_minus.18180.18295.4	4	15.4	1	4.84E+05		(K)EMVELP	(K)E M V/E/L/P L R H/P A L/F/K(A)		154.6	420.737	1679.93	-0.004	-2.4	6.86	90004.9	5.14	HUMAN	P55072	Transitional endoplasmic reticulum ATPase
2621	dirMS_041712_NAR_120mM_minus.8136.8136.3	3	15.4	0	1.23E+06		(K)HSGPNS	(K)H S G\P N\S A|D\S\A/N/D/G F/V/R(L)		98.42	544.2477	1630.72	0.0081	5	5.21	46013.4	5.38	HUMAN	P52597	Heterogeneous nuclear ribonucleoprotein F
2622	dirMS_041712_NAR_20mM_minus.12188.12188.2	2	15.4	0	1.08E+06	M313m	(R)TVQSLEI	(R)T V/Q|S L|E|I\D L D S m R(N)	m:Oxidized methionine	170.73	761.8733	1506.747	15.9925	-1.6	4.03	43773.3	5.26	HUMAN	F8VZY9	Keratin, type I cytoskeletal 18
2623	dirMS_041712_NAR_50mM_minus2.9378.9378.3	3	15.4	0	2.53E+05		(K)TGVAVN	(K)T/G|V/A V/N K/P A/E F T V D/A K(H)		121.95	549.6316	1646.875	0.0054	3.3	5.73	283472.4	5.71	HUMAN	P21333	Filamin-A
2624	dirMS_041712_NAR_120mM_plus.16287.16424.3	3	15.4	1	6.86E+05		(K)STFVLDE	(K)S T|F/V/L/D/E/F K/R(K)		138.87	414.5556	1241.652	-0.0002	-0.2	5.79	56491.1	7.78	HUMAN	B4DTG2	Elongation factor 1-gamma
2625	dirMS_041712_NAR_20mM_plus.16610.16907.2	2	15.4	0	2.65E+05		(R)IVLLDSS	(R)I/V|L|L|D/S S L\E/Y K(K)		186.97	640.36	1279.714	-0.0017	-1.3	4.37	56186.7	5.47	HUMAN	B4DUR8	T-complex protein 1 subunit gamma
2626	dirMS_041712_NAR_20mM_plus.17051.17051.2	2	15.4	0	2.06E+05		(K)VQIAVA	(K)V Q|I|A|V/A/N A Q E\L/L\Q\R(M)		188.23	776.9419	1552.881	-0.004	-2.6	5.97	10728.1	9.15	HUMAN	Q9Y5L4	Mitochondrial import inner membrane translocase subunit Tim13
2627	dirMS_041712_NAR_30mM_plus2.11583.11584.2	2	15.4	0	3.50E+05		(K)YQAVTA	(K)Y/Q|A|V|T/A T L/E/E/K(R)		114.32	626.8237	1252.642	-0.0018	-1.5	4.53	23633.9	11.02	HUMAN	P40429	60S ribosomal protein L13a
2628	dirMS_041712_NAR_30mM_plus2.18491.18673.2	2	15.4	0	6.99E+05		(K)EGMNIV	(K)E G\M|N/I|V|E/A M/E/R(F)		150.78	639.7958	1278.582	0.0026	2.1	4.25	18240.4	7.96	HUMAN	P62937	Peptidyl-prolyl cis-trans isomerase A
2629	dirMS_041712_NAR_30mM_minus2.20098.20137.2	2	15.39	0	1.49E+06		(R)VIFGLFG	(R)V/I|F/G L/F/G/K(T)		170.9	440.768	880.529	-0.0004	-0.4	8.72	23799.1	9.48	HUMAN	P23284	Peptidyl-prolyl cis-trans isomerase B
2630	dirMS_041712_NAR_50mM_minus2.12132.12132.3	3	15.39	0	9.82E+04	M240m	(K)mLDAED	(K)m L|D/A/E D I V/N T A R P D|E K(A)	m:Oxidized methionine	137.35	611.6283	1816.875	15.9958	0.5	4.1	105308.6	5.27	HUMAN	O43707	Alpha-actinin-4
2631	dirMS_041712_NAR_30mM_plus2.7657.7691.2	2	15.39	0	4.27E+05		(R)VGQDP\	(R)V/G|Q|D|P V L|R(Q)		94.27	442.2536	883.5	0.0003	0.4	5.81	14130.9	9.56	HUMAN	C9K0U8	Single-stranded DNA-binding protein (Fragment)
2632	dirMS_041712_NAR_40mM_plus2.16063.16063.3	3	15.39	1	1.85E+05		(K)LGVQVV	(K)L G V|Q/V/V|I/T D/P E K L D Q\I R(Q)		161.38	641.701	1923.091	-0.0025	-1.3	4.56	44227.3	7.81	HUMAN	E7EQR6	T-complex protein 1 subunit alpha
2633	dirMS_041712_NAR_30mM_minus2.11305.11378.2	2	15.38	0	3.66E+05		(K)TDGFGI	(K)T D|G|F|G/I/D/T C/R(S)	C:Carbamidomethylation	115.32	571.2521	1141.494	0.0027	2.3	4.21	21983.3	4.68	HUMAN	K7EIV0	Calpain small subunit 1
2634	dirMS_041712_NAR_50mM_minus2.11351.11351.3	3	15.38	1	9.59E+04		(R)LLIVSTTI	(R)L L|I/V|S T T/P Y/S E K D T K(Q)		133.12	565.6428	1694.921	-0.0072	-4.3	6.07	46476.4	5.33	HUMAN	B3KQ59	RuvB-like 2
2635	dirMS_041712_NAR_50mM_minus2.12360.12360.3	3	15.38	1	8.70E+03	M124m M:	(K)SYLNmD	(K)S/Y|L/N m/D A I m E A I K/K(T)	m:Oxidized methionine	137.73	553.6091	1626.823	31.9898	0	5.79	80685.1	7.31	HUMAN	P05165	Propionyl-CoA carboxylase alpha chain, mitochondrial
2636	dirMS_041712_NAR_50mM_minus2.16033.16033.3	3	15.38	1	4.43E+06		(R)VSFELFA	(R)V/S/F E L/F/A D K|V P K(T)		159.72	460.5908	1379.757	0.0009	0.7	6.04	13378.8	6.27	HUMAN	C9J5S7	Peptidyl-prolyl cis-trans isomerase
2637	dirMS_041712_NAR_30mM_plus2.20346.20496.2	2	15.38	0	1.13E+06		(R)ILVATNL	(R)I/L|V|A|T/N/L/F G R(G)		161.92	552.331	1103.657	-0.0024	-2.2	9.75	40918.8	9.33	HUMAN	B4DP52	HCG2005638, isoform CRA_c
2638	dirMS_041712_NAR_50mM_plus2.14970.15142.3	3	15.38	0	9.16E+05		(K)LHIIEVG	(K)L H\I\I E\V\G\T|P/P/T/G N Q P F/P K(K)		147.17	649.0216	1945.054	-0.0039	-2	6.75	193379.8	5.48	HUMAN	Q00610	Clathrin heavy chain 1
2639	dirMS_041712_NAR_60mM_plus.11414.11519.3	3	15.38	1	4.52E+05		(K)TCVFEKE	(K)T/C|V|F/E/K/E N D|P/S V M/R(S)	C:Carbamidomethylation	117.33	571.2635	1711.778	-0.0019	-1.1	4.68	267260.7	5.95	HUMAN	Q13085	Acetyl-CoA carboxylase 1
2640	dirMS_041712_NAR_30mM_minus2.12849.12967.2	2	15.37	0	5.00E+05		(R)GGEPQC	(R)G G\E|P/Q|G/P P F I|K(Y)		124.6	563.7964	1126.589	-0.0036	-3.2	6	266161	5.68	HUMAN	E9PPU0	Epiplakin
2641	dirMS_041712_NAR_30mM_minus2.18297.18600.2	2	15.37	0	6.71E+05		(K)FIIPNVV	(K)F/I|I|P N V V/K(Y)		154.63	465.2877	929.582	-0.0137	-14.8	8.75	17529.3	9.3	HUMAN	F5GXY2	L-lactate dehydrogenase A chain (Fragment)
2642	dirMS_041712_NAR_40mM_minus2.11821.11821.3	3	15.37	0	1.02E+04		(K)LVEALC	(K)L/V/E\A L C A/E/H Q I N L I K(V)	C:Carbamidomethylation	152.93	584.3199	1750.952	-0.0069	-3.9	5.4	14914.1	6.84	HUMAN	P25398	40S ribosomal protein S12
2643	dirMS_041712_NAR_60mM_plus.14407.14840.3	3	15.37	1	7.26E+06		(R)QLREYQ	(R)Q L/R E Y\Q/E/L/M/N V/K(L)		132.48	517.6044	1550.8	-0.0009	-0.6	6.14	56607.4	5.37	HUMAN	F8VXB4	Keratin, type II cytoskeletal 8
2644	dirMS_041712_NAR_30mM_minus2.13121.13121.2	2	15.36	0	3.22E+05		(K)LSELEPG	(K)L S/E/L/E/P G T\G D\L/R(F)		124.73	643.8325	1286.659	-0.0009	-0.7	4.14	266161	5.68	HUMAN	E9PPU0	Epiplakin
2645	dirMS_041712_NAR_30mM_minus2.18427.18427.2	2	15.36	0	4.47E+05		(R)QAQEYE	(R)Q A Q|E/Y/E/A L/L|N/I K(V)		154.67	710.3759	1419.748	-0.0033	-2.3	4.53	43773.3	5.26	HUMAN	F8VZY9	Keratin, type I cytoskeletal 18
2646	dirMS_041712_NAR_40mM_minus2.9153.9189.3	3	15.36	1	6.96E+05	M356m	(K)VGEVIV	(K)V G|E|V|I|V/T K D D A m L/L K(G)	m:Oxidized methionine	136.93	549.6354	1630.908	15.9832	-7.1	4.56	61224.7	5.7	HUMAN	P10809	60 kDa heat shock protein, mitochondrial
2647	dirMS_041712_NAR_60mM_minus.5108.5206.3	3	15.36	0	4.24E+06	M434m M4	(R)LESGmC	(R)L/E|S/G m Q/N m/S I/H/T K(T)	m:Oxidized methionine	84.33	503.2336	1475.698	31.9881	-1.1	6.75	56607.4	5.37	HUMAN	F8VXB4	Keratin, type II cytoskeletal 8
2648	dirMS_041712_NAR_30mM_plus2.13677.13697.2	2	15.36	0	3.10E+05		(R)IASLEVE	(R)I A|S\L|E/V|E N Q S L/R(G)		124.87	679.867	1358.727	-0.0007	-0.5	4.53	22489.6	5.38	HUMAN	E9PI39	Elongation factor 1-delta (Fragment)
2649	dirMS_041712_NAR_30mM_plus2.16027.16125.2	2	15.36	0	7.35E+05		(R)GALQNI	(R)G A/L|Q|N|I|I\P A S T G\A A\K(A)		137.52	706.3979	1411.79	-0.0018	-1.3	8.75	31718.5	7.17	HUMAN	E7EUT4	Glyceraldehyde-3-phosphate dehydrogenase
2650	dirMS_041712_NAR_30mM_plus2.19882.19882.2	2	15.36	0	5.28E+05		(K)MAILQII	(K)M/A|I|L Q I/M|K(E)		158.22	474.2739	947.542	-0.0011	-1.2	8.5	90316.8	5.52	HUMAN	Q13263	Transcription intermediary factor 1-beta
2651	dirMS_041712_NAR_30mM_plus2.21942.21942.2	2	15.36	0	1.55E+05		(K)ANFLQN	(K)A N|F\L/Q|N M/I/E/D/R(T)		170.25	675.8282	1350.647	0.0021	1.5	4.37	120546	7.82	HUMAN	O95486	Protein transport protein Sec24A
2652	dirMS_041712_NAR_120mM_minus.10768.11144.3	3	15.35	0	1.08E+06		(R)GFCLPPI	(R)G F|C L/P/P H/C S/R(G)	C:Carbamidomethylation	113.72	410.858	1230.551	0.0088	7.1	9.75	14391.8	4.99	HUMAN	G3V461	Creatine kinase B-type (Fragment)
2653	dirMS_041712_NAR_120mM_minus.19212.19212.3	3	15.35	1	2.76E+05	M148m	(R)LDLAGR	(R)L/D|L|A/G R D L T D/Y L m K(I)	m:Oxidized methionine	159.78	547.2876	1623.841	16.0072	7.5	4.43	37482.1	5.36	HUMAN	E9PG30	Actin, gamma-enteric smooth muscle
2654	dirMS_041712_NAR_40mM_minus2.12893.12893.3	3	15.35	0	3.35E+05		(R)DVQIGD	(R)D V|Q|I/G/D/I/V/T V G E C R P L S K(T)	C:Carbamidomethylation	159.67	662.684	1986.033	0.005	2.5	4.56	18601.6	10.52	HUMAN	P62280	40S ribosomal protein S11
2655	dirMS_041712_NAR_40mM_minus2.15432.15432.3	3	15.35	1	3.01E+05		(R)GGPNIIT	(R)G G/P N I|I/T L/A D I V K D/P V S R(T)		178.45	622.3523	1865.049	-0.0067	-3.6	5.96	45481.2	7.85	HUMAN	E7EU96	Casein kinase II subunit alpha
2656	dirMS_041712_NAR_50mM_minus2.17479.17660.3	3	15.35	0	4.20E+05		(K)VAHALA	(K)V A H A L A E G\L|G\V|I|A/C/I/G E K(L)	C:Carbamidomethylation	168.75	603.3247	1807.974	-0.0139	-7.7	5.4	31075.7	5.65	HUMAN	P60174	Triosephosphate isomerase
2657	dirMS_041712_NAR_50mM_minus2.9996.9996.3	3	15.35	0	5.74E+05	M147m	(K)LQmEAF	(K)L Q/m\E A/P H\I|I/V G T P G R(V)	m:Oxidized methionine	125.45	545.6267	1618.873	15.9922	-1.7	6.75	20859.5	4.85	HUMAN	J3KS25	Eukaryotic initiation factor 4A-I (Fragment)
2658	dirMS_041712_NAR_60mM_minus.9967.10009.3	3	15.35	0	1.64E+06		(R)DNIQGI	(R)D N|I|Q/G I/T K/P A/I/R(R)		116.53	442.5878	1325.754	-0.0047	-3.6	8.75	11367.1	11.36	HUMAN	P62805	Histone H4
2659	dirMS_041712_NAR_20mM_plus.15483.15483.2	2	15.35	0	7.27E+04		(R)AVLNPL	(R)A V\L/N|P\L C/Q V D/Y/R(A)	C:Carbamidomethylation	178.62	724.3735	1447.736	0.0035	2.4	5.88	83880.6	7.33	HUMAN	F5H365	Protein transport protein Sec23A
2660	dirMS_041712_NAR_30mM_plus2.7741.7773.2	2	15.35	0	3.78E+05		(R)VGFAEA	(R)V/G|F/A/E/A A/R(L)		94.65	410.7181	820.431	-0.0023	-2.7	5.97	20599.3	9.19	HUMAN	H7C1V0	Cathepsin D light chain (Fragment)
2661	dirMS_041712_NAR_50mM_plus2.14769.14813.3	3	15.35	1	1.58E+04		(K)KGVNLP	(K)K G V|N L P G\A\A|V/D|L|P A V/S E K(D)		145.08	588.999	1764.985	-0.003	-1.7	6.07	30947.9	6.47	HUMAN	H3BQ34	Pyruvate kinase
2662	dirMS_041712_NAR_50mM_plus2.18540.18560.3	3	15.35	0	9.87E+05		(K)VAHALA	(K)V A H A\L A E G|L/G\V|I/A/C/I G E K(L)	C:Carbamidomethylation	166.15	603.3265	1807.974	-0.0085	-4.7	5.4	31075.7	5.65	HUMAN	P60174	Triosephosphate isomerase
2663	dirMS_041712_NAR_60mM_plus.16088.16088.3	3	15.35	0	1.09E+06		(K)SLTNDW	(K)S L/T/N/D/W/E D H/L/A/V K(H)		141.68	509.919	1527.744	-0.0014	-0.9	4.53	85057.6	4.94	HUMAN	P07900	Heat shock protein HSP 90-alpha
2664	dirMS_041712_NAR_120mM_minus.9371.9601.3	3	15.34	0	1.70E+06		(K)EFHLNE	(K)E\F H\L N\E/S/G/D/P/S/S K(S)		106.18	482.8881	1446.65	0.0002	0.1	4.65	30992.3	4.14	HUMAN	B2REB8	Protein SET
2665	dirMS_041712_NAR_30mM_minus2.14145.14349.2	2	15.34	0	4.40E+05		(R)SVPTWL	(R)S V|P/T|W/L/K(L)		131.07	415.7421	830.477	-0.0001	-0.2	8.47	12573.5	10.39	HUMAN	E9PS50	40S ribosomal protein S13 (Fragment)
2666	dirMS_041712_NAR_30mM_minus2.15675.15731.2	2	15.34	0	2.02E+05		(K)ALELDSI	(K)A/L|E|L|D/S N/L/Y/R(I)		138.85	597.3118	1193.616	0.0002	0.2	4.37	227783.5	5.5	HUMAN	P35579	Myosin-9
2667	dirMS_041712_NAR_60mM_minus.10259.10484.3	3	15.34	0	5.97E+05		(K)AQVARP	(K)A/Q|V/A/R P G G D/T I/F/G/K(I)		117.88	472.9235	1416.759	-0.0034	-2.4	8.79	7323.5	9.4	HUMAN	D6RC06	Histidine triad nucleotide-binding protein 1
2668	dirMS_041712_NAR_120mM_plus.19629.19688.3	3	15.34	1	2.18E+05		(K)RSIQFVD	(K)R S I Q\F\V|D|W/C/P T G F K(V)	C:Carbamidomethylation	156.15	580.9585	1740.853	0.0083	4.8	8.75	49012.3	4.9	HUMAN	A8MUB1	Tubulin alpha-4A chain
2669	dirMS_041712_NAR_20mM_plus.11005.11005.2	2	15.34	0	2.60E+04		(R)IVLQIDN	(R)I V|L/Q/I/D N/A R(L)		148.23	521.3056	1041.605	-0.0012	-1.1	5.84	20842.9	5.32	HUMAN	C9JM50	Keratin, type I cytoskeletal 19 (Fragment)
2670	dirMS_041712_NAR_30mM_plus2.17624.17684.2	2	15.34	0	4.73E+05		(R)LSYTQLL	(R)L/S|Y/T/Q/L/L/R(R)		145.87	497.2902	993.573	0.0004	0.4	8.75	533778	5.74	HUMAN	Q15149	Plectin
2671	dirMS_041712_NAR_40mM_plus2.13298.13400.3	3	15.34	0	6.01E+05		(R)QVSASE	(R)Q V\S/A/S E/L H T/S G I/L/G/P E T L R(D)		145.08	665.6807	1995.051	-0.023	-11.5	5.4	558007.1	5.44	HUMAN	P58107	Epiplakin
2672	dirMS_041712_NAR_60mM_plus.11514.11592.3	3	15.34	1	1.90E+06		(R)LMEEIM	(R)L/M|E/E I/M/S E K E N K(T)		118.2	494.2424	1480.702	0.0105	7.1	4.49	61018.6	9.44	HUMAN	B4DLW8	Probable ATP-dependent RNA helicase DDX5
2673	dirMS_041712_NAR_60mM_plus.12298.12371.3	3	15.34	1	9.36E+05		(R)LSFDKD	(R)L/S|F D/K/D/A/M/V A/R(A)		121.18	418.2158	1252.635	-0.0026	-2.1	5.96	35556.4	9.3	HUMAN	F2Z393	Transaldolase
2674	dirMS_041712_NAR_50mM_minus2.15780.15780.3	3	15.33	0	3.64E+05		(K)LYGPTNI	(K)L Y|G/P/T N F/S P I I N/H V A R(F)		158.22	600.3221	1798.96	-0.0081	-4.5	8.75	60985.3	5.6	HUMAN	O75131	Copine-3
2675	dirMS_041712_NAR_50mM_minus2.18183.18183.3	3	15.33	1	2.42E+05		(K)LKEAELL	(K)L/K\E A E\L\L|E|P\L M/P A I R(A)		172.72	574.9957	1722.982	-0.0097	-5.6	4.79	56164.6	6.01	HUMAN	E9PP73	Coatomer subunit beta (Fragment)
2676	dirMS_041712_NAR_120mM_plus.18686.19009.3	3	15.33	1	2.43E+05		(K)SIYGEKF	(K)S I|Y/G E/K/F/E/D E N F I/L/K(H)		151.63	611.3079	1831.911	-0.0021	-1.2	4.41	13378.8	6.27	HUMAN	C9J5S7	Peptidyl-prolyl cis-trans isomerase
2677	dirMS_041712_NAR_20mM_plus.14468.14468.3	3	15.33	0	1.30E+05		(R)LQEAGIL	(R)L Q E\A G\I\L|S A/E/E L/Q R(L)		171.58	519.6131	1556.828	-0.0031	-2	4.25	533778	5.74	HUMAN	Q15149	Plectin
2678	dirMS_041712_NAR_20mM_plus.15189.15564.2	2	15.33	0	8.10E+05		(K)LLCGLLA	(K)L/L|C|G/L/L/A E/R(L)	C:Carbamidomethylation	177.65	522.7971	1044.587	-0.0001	-0.1	6	12647.2	8.07	HUMAN	P14174	Macrophage migration inhibitory factor
2679	dirMS_041712_NAR_30mM_plus2.10865.11120.2	2	15.33	0	1.56E+06		(K)TIAPALV	(K)T I|A|P/A L/V S K(K)		110.98	450.2857	899.556	0.0081	9	8.41	21077	9.05	HUMAN	K7EM90	Alpha-enolase (Fragment)
2680	dirMS_041712_NAR_30mM_plus2.16993.16993.2	2	15.33	0	2.53E+05	M313m	(R)TVQSLEI	(R)T V/Q/S L/E|I D L/D S m R(N)	m:Oxidized methionine	142.07	761.8741	1506.747	15.9941	-0.5	4.03	43773.3	5.26	HUMAN	F8VZY9	Keratin, type I cytoskeletal 18
2681	dirMS_041712_NAR_30mM_plus2.6898.6958.2	2	15.33	0	1.33E+05		(R)TAPAAN	(R)T/A|P|A/A/N/L A S/R(I)		90.78	486.2674	971.527	0.0007	0.7	9.41	300351.7	12.1	HUMAN	Q9UQ35	Serine/arginine repetitive matrix protein 2
2682	dirMS_041712_NAR_20mM_minus.7535.7535.2	2	15.32	0	6.83E+05	M239m	(K)EVDEQm	(K)E V/D/E/Q/m\L/N|V/Q/N K(N)	m:Oxidized methionine	123.77	731.843	1446.689	15.9894	-3.8	4.14	40069.4	4.83	HUMAN	E7EWR1	Tubulin beta chain
2683	dirMS_041712_NAR_30mM_minus2.7665.7665.2	2	15.32	0	1.21E+05		(R)SGGGG(	(R)S G/G/G/G/G/G G L/G S G/G S I R(S)		96.1	616.8021	1232.598	-0.0009	-0.7	9.47	62291.5	5.13	HUMAN	P35527	Keratin, type I cytoskeletal 9
2684	dirMS_041712_NAR_60mM_minus.9338.9338.3	3	15.32	1	2.25E+05	M232m M:	(R)MSmKE\	(R)M S m K E V\D\E Q|m|L/N V/Q/N K(N)	m:Oxidized methionine	111.92	652.6318	1923.897	31.9836	-3.2	4.68	40069.4	4.83	HUMAN	E7EWR1	Tubulin beta chain
2685	dirMS_041712_NAR_60mM_minus.9351.9361.3	3	15.32	1	1.67E+05		(K)LAELKQI	(K)L/A|E/L/K/Q/E/C/L A R(G)	C:Carbamidomethylation	112.02	444.2455	1330.715	0.0072	5.4	6.14	6106	9.22	HUMAN	F8VS12	SAP domain-containing ribonucleoprotein
2686	dirMS_041712_NAR_120mM_plus.9473.9473.3	3	15.32	0	3.32E+05		(K)QEYDES	(K)Q E Y/D E S/G/P/S I/V/H/R(K)		102.88	506.2393	1516.703	0.0007	0.5	4.65	123097.1	5.83	HUMAN	A5A3E0	POTE ankyrin domain family member F
2687	dirMS_041712_NAR_20mM_plus.15706.15706.2	2	15.32	0	1.28E+06	M73m	(R)AILVDLE	(R)A I L V|D|L|E/P G T m D S V R(S)	m:Oxidized methionine	179.22	816.4181	1615.836	15.9929	-1.2	4.03	27549.9	4.39	HUMAN	E9PBJ4	Tubulin beta chain
2688	dirMS_041712_NAR_20mM_plus.16031.16378.2	2	15.32	0	4.91E+05		(K)DAGTIA	(K)D A\G\T|I|A|G/L/N/V L R(I)		183.63	600.3422	1199.674	0.0029	2.4	5.84	17973	5.16	HUMAN	E9PI65	Heat shock cognate 71 kDa protein (Fragment)
2689	dirMS_041712_NAR_20mM_plus.18241.18241.2	2	15.32	0	1.70E+05		(R)LTGQLFI	(R)L/T|G Q|L|F/L/G G S I V K(G)		197.02	666.8962	1332.789	-0.0034	-2.6	8.75	45924.5	5.29	HUMAN	B4E1F3	Selenium-binding protein 1
2690	dirMS_041712_NAR_30mM_plus2.6384.6384.2	2	15.32	0	3.31E+05	M367m	(R)FGSGmI	(R)F/G|S|G m N M/G R(I)	m:Oxidized methionine	87.53	486.7048	956.408	15.9946	-0.3	9.75	77799.7	9.09	HUMAN	P52272	Heterogeneous nuclear ribonucleoprotein M
2691	dirMS_041712_NAR_40mM_plus2.11915.11915.3	3	15.32	1	6.43E+04		(R)FGEVVD	(R)F/G|E/V/V/D C T I/K T D P/V T G R(S)	C:Carbamidomethylation	136.33	631.9821	1893.938	-0.0057	-3	4.56	46607.8	9.68	HUMAN	O14979	Heterogeneous nuclear ribonucleoprotein D-like
2692	dirMS_041712_NAR_30mM_minus2.7370.7404.2	2	15.31	0	5.41E+05		(K)AMDVY(	(K)A/M|D|V/Y/Q/K(A)		94.8	427.7069	854.408	-0.0011	-1.3	5.88	60404.6	6.4	HUMAN	F5H0T1	Stress-induced-phosphoprotein 1
2693	dirMS_041712_NAR_50mM_minus2.9363.9702.3	3	15.31	1	1.07E+07		(R)DGKLVS	(R)D G K\L\V\S\E|S/S/D/V/L/P/K(-)		121.97	491.9322	1473.78	0.0025	1.7	4.56	56607.4	5.37	HUMAN	F8VXB4	Keratin, type II cytoskeletal 8
2694	dirMS_041712_NAR_60mM_minus.10048.10048.3	3	15.31	0	1.56E+06		(R)EIVHIQA	(R)E/I V H I Q A G Q/C/G N Q I|G A K(F)	C:Carbamidomethylation	115.57	608.3099	1822.923	-0.0077	-4.2	6.85	40069.4	4.83	HUMAN	E7EWR1	Tubulin beta chain
2695	dirMS_041712_NAR_30mM_plus2.16032.16032.2	2	15.31	0	4.04E+05		(K)MDQAII	(K)M D|Q/A|I/I/F/C R(T)	C:Carbamidomethylation	137.1	577.2809	1153.549	0.0053	4.6	5.59	83400.6	6.82	HUMAN	Q92499	ATP-dependent RNA helicase DDX1
2696	dirMS_041712_NAR_30mM_plus2.16890.17177.2	2	15.31	0	7.05E+05		(K)FFVGGN	(K)F/F|V/G/G N/W K(M)		142.57	477.7447	954.483	-0.0011	-1.1	8.75	31075.7	5.65	HUMAN	P60174	Triosephosphate isomerase
2697	dirMS_041712_NAR_30mM_plus2.19404.19554.2	2	15.31	0	2.95E+05		(K)TVDNFV	(K)T V|D|N|F/V/A L/A/T/G E K(G)		155.22	682.8539	1364.706	-0.0051	-3.7	4.37	23799.1	9.48	HUMAN	P23284	Peptidyl-prolyl cis-trans isomerase B
2698	dirMS_041712_NAR_30mM_plus2.20798.20992.2	2	15.31	0	3.07E+05		(K)LVLPSLI	(K)L/V|L/P/S/L/I/S S/R(I)		163.97	542.8382	1084.672	-0.0033	-3.1	9.75	39066	6.61	HUMAN	A6PVW9	Selenium binding protein 1 (Fragment)
2699	dirMS_041712_NAR_50mM_plus2.20345.20345.3	3	15.31	1	3.35E+06		(R)TLAEIAK	(R)T L|A E/I/A/K/V/E\L/D N M P L R(G)		174.87	604.9965	1812.989	-0.0138	-7.6	4.68	23729.5	9.89	HUMAN	C9IZL7	Non-POU domain-containing octamer-binding protein (Fragment)
2700	dirMS_041712_NAR_50mM_minus2.16905.16905.3	3	15.3	0	2.41E+05		(R)LGIEGLS	(R)L/G\I|E/G L/S L H N V L/K(L)		164.2	464.9452	1392.821	0.0001	0.1	6.75	177928	5.42	HUMAN	Q9NYU2	UDP-glucose:glycoprotein glucosyltransferase 1
2701	dirMS_041712_NAR_60mM_minus.10114.10114.3	3	15.3	0	3.39E+05		(K)LTPLSHE	(K)L/T/P L/S H/E V I/S R(Q)		115.77	417.9007	1251.706	-0.018	-14.4	6.75	51099.4	8.43	HUMAN	F8W810	Uncharacterized protein
2702	dirMS_041712_NAR_120mM_plus.10719.10719.3	3	15.3	0	8.43E+05		(R)YYDSRP(	(R)Y/Y/D/S\R P/G G Y G Y/G Y G R(S)		108.75	577.5892	1730.756	-0.0027	-1.6	8.38	17170.1	8.86	HUMAN	P98179	Putative RNA-binding protein 3
2703	dirMS_041712_NAR_30mM_plus2.13475.13496.2	2	15.3	0	1.03E+05		(R)TIYAGN	(R)T I|Y|A|G/N|A|L C T/V/K(C)	C:Carbamidomethylation	123.85	655.8414	1310.677	-0.0018	-1.4	8.26	24273.5	8.98	HUMAN	H0YK49	Electron transfer flavoprotein subunit alpha, mitochondrial
2704	dirMS_041712_NAR_30mM_plus2.8995.8995.2	2	15.3	0	6.30E+05		(R)SSQSSS(	(R)S S/Q|S S|S Q Q/F/S/G I|G\R(S)		101.03	728.345	1455.682	0.0005	0.3	9.47	73202	9.19	HUMAN	C9JMU5	Probable ATP-dependent RNA helicase DDX17
2705	dirMS_041712_NAR_40mM_plus2.7020.7057.2	2	15.3	0	1.61E+05		(R)QLETLG(	(R)Q/L/E/T/L/G Q/E/K(L)		101.52	523.282	1045.552	0.0043	4.1	4.53	13588.1	4.66	HUMAN	F8VP67	Keratin, type II cytoskeletal 8 (Fragment)
2706	dirMS_041712_NAR_50mM_plus2.9533.9533.3	3	15.3	1	4.76E+05		(K)LTSDDVI	(K)L/T|S D/D V K/E Q I/Y/K(L)		117.47	480.2521	1438.742	-0.0007	-0.5	4.56	12573.5	10.39	HUMAN	E9PS50	40S ribosomal protein S13 (Fragment)
2707	dirMS_041712_NAR_60mM_plus.15578.15579.3	3	15.3	1	4.36E+05		(K)TLNNKF	(K)T/L|N N K/F/A/S/F I/D K(V)		138.13	466.5862	1397.742	0.0017	1.2	8.26	19569.8	9.88	HUMAN	F8VRG4	Keratin, type II cytoskeletal 8 (Fragment)
2708	dirMS_041712_NAR_60mM_plus.18454.18454.3	3	15.3	1	7.09E+05		(R)IRIDSLS	(R)I R I D S\L\S\A\Q|L/S/Q/L Q K(Q)		153.97	567.3254	1699.97	-0.0085	-5	8.75	63136.9	5.83	HUMAN	D6RAQ3	Prelamin-A/C
2709	dirMS_041712_NAR_50mM_minus2.16428.16720.3	3	15.29	1	1.07E+06		(R)VTFSGLF	(R)V/T|F/S/G L R D T V/T/P G E L/L/K(A)		162.5	611.672	1833.012	-0.0102	-5.6	6.04	266161	5.68	HUMAN	E9PPU0	Epiplakin
2710	dirMS_041712_NAR_50mM_minus2.18067.18182.3	3	15.29	1	6.74E+05		(R)VLEALLP	(R)V/L|E|A/L L/P L/K G L E/E R(V)		172.47	527.3171	1579.942	-0.005	-3.2	4.79	276046.1	6.01	HUMAN	P49327	Fatty acid synthase
2711	dirMS_041712_NAR_30mM_plus2.17038.17085.2	2	15.29	0	2.91E+05		(R)GLGLDD	(R)G L|G|L/D/D/A|L/E|P R(Q)		142.02	578.3042	1155.6	0.0007	0.6	4.03	51326.8	5.49	HUMAN	Q9Y230	RuvB-like 2
2712	dirMS_041712_NAR_30mM_plus2.20390.20390.2	2	15.29	0	6.53E+04		(K)SYELPD(	(K)S Y\E|L/P D/G Q V I|T I/G N E R(F)		160.92	895.9487	1790.892	-0.0018	-1	4.14	123097.1	5.83	HUMAN	A5A3E0	POTE ankyrin domain family member F
2713	dirMS_041712_NAR_30mM_plus2.22258.22258.2	2	15.29	0	1.03E+06		(K)IGFPWSI	(K)I G/F|P/W/S E I R(N)		173.07	552.8032	1104.584	0.0155	14	6	71218.8	6.3	HUMAN	A7YIJ8	Radixin
2714	dirMS_041712_NAR_50mM_plus2.18186.18186.3	3	15.29	1	1.08E+05		(K)DLEIKIP\	(K)D L\E I K/I/P\V/V/V/R(L)		163.52	427.6021	1280.794	-0.0019	-1.5	6.07	44183.5	5.64	HUMAN	F5GXC8	Succinyl-CoA ligase [ADP-forming] subunit beta, mitochondrial
2715	dirMS_041712_NAR_60mM_plus.5038.5038.3	3	15.29	1	3.95E+05		(R)AAEDDE	(R)A A|E/D D/E/D D D/V D T K K(Q)		83.53	522.5529	1565.645	-0.0008	-0.5	3.76	14366.2	3.71	HUMAN	B8ZZA1	Thymosin alpha-1
2716	dirMS_041712_NAR_60mM_plus.5301.5301.3	3	15.29	1	1.18E+04	M117m	(K)EmQLRC	(K)E\m/Q/L/R Q E/E/E/R(R)	m:Oxidized methionine	85.13	455.2157	1347.632	16.0004	4	4.49	26419.2	5.1	HUMAN	H0Y9K7	Splicing factor, proline- and glutamine-rich (Fragment)
2717	dirMS_041712_NAR_120mM_minus.11369.11369.3	3	15.28	0	2.31E+05		(R)GFCLPPI	(R)G F|C/L/P/P/H C S R(G)	C:Carbamidomethylation	116.42	410.858	1230.551	0.0088	7.1	9.75	14391.8	4.99	HUMAN	G3V461	Creatine kinase B-type (Fragment)
2718	dirMS_041712_NAR_120mM_minus.20102.20250.3	3	15.28	0	1.72E+05	M195m	(R)LHFFmP	(R)L H F F m|P G|F|A|P/L T S/R(G)	m:Oxidized methionine	165.67	546.2809	1620.835	15.9926	-1.4	9.75	42230.8	4.77	HUMAN	A6NKZ8	Putative tubulin beta chain-like protein ENSP00000290377
2719	dirMS_041712_NAR_120mM_minus.9837.9894.3	3	15.28	0	8.69E+05		(K)STAGDT	(K)S/T|A/G D/T H/L/G G E D F D/N R(M)		108.4	564.5795	1691.726	-0.0016	-1	4.22	69032.8	5.37	HUMAN	E9PKE3	Heat shock cognate 71 kDa protein
2720	dirMS_041712_NAR_20mM_plus.6956.6956.2	2	15.28	0	8.44E+04	M29m	(K)IVQmTE.	(K)I/V|Q m|T/E A E/V R(G)	m:Oxidized methionine	114.35	596.3035	1175.609	15.9908	-3.4	4.53	14493.5	5.23	HUMAN	C9J9S3	Serine/threonine-protein phosphatase (Fragment)

2721	dirMS_041712_NAR_30mM_plus2.8833.8833.2	2	15.28	0	3.99E+05		(K)ITSEIPQ1	(K)I T|S/E|I/P Q T E R(M)		100.2	587.3094	1173.611	0.0005	0.5	4.53	24161.9	5.27	HUMAN	F8VZQ9	SAP domain-containing ribonucleoprotein
2722	dirMS_041712_NAR_50mM_plus2.22484.22484.3	3	15.28	1	1.26E+06		(R)GVMLA\	(R)G V/M/L/A V/D A V I A E/L K K(Q)		195.7	519.6393	1556.908	-0.0047	-3	6.07	25225.7	9.52	HUMAN	E7ESH4	60 kDa heat shock protein, mitochondrial (Fragment)
2723	dirMS_041712_NAR_60mM_plus.7898.7933.3	3	15.28	0	1.02E+06		(K)EAYPDH	(K)E A|Y/P/D/H T Q F E/K(N)		99.42	455.5423	1364.612	0.0006	0.5	4.65	25868	9.48	HUMAN	Q9P016	Thymocyte nuclear protein 1
2724	dirMS_041712_NAR_30mM_minus2.10689.10840.2	2	15.27	0	5.83E+05		(R)GYSFTT1	(R)G/Y|S|F/T/T/T/A E R(E)		112.3	566.7699	1132.527	0.0056	4.9	6	23901.9	5.28	HUMAN	I3L1U9	Actin, cytoplasmic 2, N-terminally processed (Fragment)
2725	dirMS_041712_NAR_30mM_minus2.12569.13070.2	2	15.27	1	3.16E+06		(K)KITIADC	(K)K\I\T\I|A|D\C\G\Q|L|E(-)	C:Carbamidomethylation	123.22	624.3176	1247.63	-0.0021	-1.7	4.75	18240.4	7.96	HUMAN	P62937	Peptidyl-prolyl cis-trans isomerase A
2726	dirMS_041712_NAR_30mM_minus2.14875.14875.2	2	15.27	0	1.40E+05		(R)WALSQS	(R)W/A|L|S/Q/S N/P S A L R(E)		134.55	665.3481	1329.691	-0.0021	-1.5	9.75	90004.9	5.14	HUMAN	P55072	Transitional endoplasmic reticulum ATPase
2727	dirMS_041712_NAR_30mM_plus2.14583.14773.2	2	15.27	0	4.71E+05		(K)VPEWVI	(K)V/P|E/W/V/D/T V/K(L)		129.9	536.7859	1072.567	-0.0028	-2.6	4.37	16060.2	10.31	HUMAN	P39019	40S ribosomal protein S19
2728	dirMS_041712_NAR_30mM_plus2.19089.19089.2	2	15.27	0	9.29E+05		(K)DAGTIA	(K)D A G/T|I/A/G L|N V L R(I)		152.63	600.3411	1199.674	0.0007	0.6	5.84	17973	5.16	HUMAN	E9PI65	Heat shock cognate 71 kDa protein (Fragment)
2729	dirMS_041712_NAR_30mM_plus2.8792.8931.2	2	15.27	0	7.50E+05		(R)GMGPG	(R)G M/G/P/G/T|P A/G Y/G R(G)		100.25	560.7657	1120.52	0.0037	3.3	8.75	76262.2	9.53	HUMAN	P23246	Splicing factor, proline- and glutamine-rich
2730	dirMS_041712_NAR_60mM_plus.16743.16761.3	3	15.27	1	8.72E+05		(R)STNKGT.	(R)S T N K\G T A\Y\T/F/F/T|P G N/L K(Q)		144.73	616.3117	1846.933	-0.0128	-7	9.7	73202	9.19	HUMAN	C9JMU5	Probable ATP-dependent RNA helicase DDX17
2731	dirMS_041712_NAR_120mM_minus.17474.17760.3	3	15.26	1	3.32E+05		(R)RAPFDLI	(R)R A\P F|D|L|F/E/N/K(K)		150.82	412.8849	1236.637	0.003	2.4	6.07	83605	4.97	HUMAN	P08238	Heat shock protein HSP 90-beta
2732	dirMS_041712_NAR_40mM_minus2.14113.14201.3	3	15.26	0	6.88E+05		(R)IHFPLAT	(R)I H F\P L A\T|Y|A|P V/I/S A E K(A)		167.82	586.3246	1756.963	-0.004	-2.3	6.75	49012.3	4.9	HUMAN	A8MUB1	Tubulin alpha-4A chain
2733	dirMS_041712_NAR_40mM_minus2.14162.14277.3	3	15.26	1	2.95E+05		(K)VIAATNI	(K)V/I|A|A/T N R V D I|L/D/P A L L R(S)		167.82	617.3653	1850.086	-0.0045	-2.4	5.93	32827.5	5.92	HUMAN	E9PKD5	26S protease regulatory subunit 6A (Fragment)
2734	dirMS_041712_NAR_50mM_minus2.17073.17073.3	3	15.26	0	1.55E+05		(R)LLEVLSG	(R)L/L|E|V/L/S G E I L/P/K/P T K(G)		165.35	546.3329	1636.988	-0.0042	-2.6	6.14	13611	6.13	HUMAN	E9PJZ2	Spectrin beta chain, non-erythrocytic 2 (Fragment)
2735	dirMS_041712_NAR_60mM_minus.15320.15320.3	3	15.26	1	3.71E+05		(R)LLDAQL/	(R)L L/D/A/Q L/A|T G/G L V C P A R R(L)	C:Carbamidomethylation	145.7	604.3344	1810.996	-0.007	-3.8	9.6	266161	5.68	HUMAN	E9PPU0	Epiplakin
2736	dirMS_041712_NAR_120mM_plus.10966.11258.3	3	15.26	0	1.15E+06		(K)NFYVEH	(K)N F/Y/V/E H/P E/V A/R(L)		111.23	454.2277	1360.664	0.0041	3	5.4	73202	9.19	HUMAN	C9JMU5	Probable ATP-dependent RNA helicase DDX17
2737	dirMS_041712_NAR_20mM_plus.12409.12409.3	3	15.26	0	3.15E+04		(K)IISNASC	(K)I I\S N\A|S/C T T N/C L|A/P L A K(V)	C:Carbamidomethylation	157.65	611.9768	1833.92	-0.0039	-2.1	8.75	31718.5	7.17	HUMAN	E7EUT4	Glyceraldehyde-3-phosphate dehydrogenase
2738	dirMS_041712_NAR_30mM_plus2.12103.12104.2	2	15.26	0	3.92E+05		(R)GYFSEEI	(R)G Y|F|S/E E/M N/R(V)		116.33	566.7397	1132.473	-0.0007	-0.6	4.53	533778	5.74	HUMAN	Q15149	Plectin
2739	dirMS_041712_NAR_50mM_plus2.12236.12299.3	3	15.26	1	1.69E+06		(K)ILIANTG	(K)I/L|I|A N/T/G/M D T D K I K(I)		132.52	511.6172	1532.835	0.0018	1.2	5.96	57204.9	6.01	HUMAN	F5GWF6	T-complex protein 1 subunit beta
2740	dirMS_041712_NAR_60mM_plus.8565.8565.3	3	15.26	1	1.32E+06		(K)MQASIE	(K)M/Q|A S I/E K/G G S L/P K(V)		103.15	449.2449	1345.714	0.0057	4.3	8.35	32745.6	4.64	HUMAN	P06748	Nucleophosmin
2741	dirMS_041712_NAR_60mM_minus.10051.10479.3	3	15.25	0	5.90E+06		(R)GSAVHC	(R)G S|A/V/H/Q/L/S/E/E/L/R(C)		117.4	442.5648	1325.681	-0.001	-0.7	5.4	558007.1	5.44	HUMAN	P58107	Epiplakin
2742	dirMS_041712_NAR_60mM_minus.11315.11315.3	3	15.25	1	1.43E+05		(R)DGKLVS	(R)D G K L V\S E/S/S/D V/L/P K(-)		123.12	491.927	1473.78	-0.0131	-8.9	4.56	56607.4	5.37	HUMAN	F8VXB4	Keratin, type II cytoskeletal 8
2743	dirMS_041712_NAR_120mM_plus.19474.19474.3	3	15.25	1	6.90E+05		(K)YNQLLRI	(K)Y/N/Q/L/L/R I E E E L G S K(A)		155.23	564.6353	1691.896	-0.0049	-2.9	4.79	47510.4	7.04	HUMAN	P06733	Alpha-enolase
2744	dirMS_041712_NAR_30mM_plus2.17667.18204.2	2	15.25	0	2.38E+06		(K)AILFVPR	(K)A I|L|F/V/P/R(R)		147.43	408.2604	815.514	-0.0003	-0.3	9.79	57660.2	6.38	HUMAN	Q9NQH7	Probable Xaa-Pro aminopeptidase 3
2745	dirMS_041712_NAR_40mM_plus2.11775.11816.3	3	15.25	1	2.80E+05		(R)IVAPGK(	(R)I V|A|P G K G I L A A/D/E S T/G S I/A K(R)		135.4	633.3568	1898.059	-0.0035	-1.8	6.07	30711.4	7.15	HUMAN	H3BPS8	Fructose-bisphosphate aldolase A (Fragment)
2746	dirMS_041712_NAR_60mM_minus.8540.8625.3	3	15.24	0	2.74E+05		(R)LAQGHT	(R)L/A|Q G/H/T/T V/D/E/L A R(R)		107.93	470.9153	1410.734	-0.0022	-1.6	5.32	533778	5.74	HUMAN	Q15149	Plectin
2747	dirMS_041712_NAR_120mM_plus.18407.18464.3	3	15.24	1	6.09E+04	M75m	(R)RFDEILE	(R)R F D E\I\L\E|A|S/D G/I/m/V A R(G)	m:Oxidized methionine	149.78	613.3075	1821.916	15.9916	-1.8	4.32	18466.1	5.78	HUMAN	H3BSU3	Pyruvate kinase (Fragment)
2748	dirMS_041712_NAR_20mM_plus.12670.12670.2	2	15.24	0	1.21E+05		(K)GTIQVIT	(K)G T I/Q/V/I/T\Q/G T/S/L K(N)		160.13	673.3872	1345.769	-0.0014	-1.1	8.75	57391.2	6.29	HUMAN	O43175	D-3-phosphoglycerate dehydrogenase
2749	dirMS_041712_NAR_30mM_plus2.6009.6009.2	2	15.24	0	9.97E+04		(R)QLAEED	(R)Q L/A/E/E/D/A A R(Q)		85.77	501.7464	1002.485	0.0005	0.5	4.14	533778	5.74	HUMAN	Q15149	Plectin
2750	dirMS_041712_NAR_50mM_plus2.16362.16499.3	3	15.24	0	1.45E+06		(K)IEVIKPG	(K)I/E|V|I/K/P G D L/G V D/L T S K(L)		154.62	561.9883	1683.953	-0.0024	-1.4	4.56	28071.3	8.6	HUMAN	P38117	Electron transfer flavoprotein subunit beta
2751	dirMS_041712_NAR_50mM_plus2.5726.5726.3	3	15.24	1	1.05E+06		(K)TAGPIAS	(K)T A/G/P I/A S\A Q K Q P A G K(V)		91.9	475.5987	1424.786	-0.0041	-2.8	10	75259.2	9.59	HUMAN	B5MEG9	Microtubule-associated protein 4 (Fragment)
2752	dirMS_041712_NAR_50mM_plus2.9501.9501.3	3	15.24	1	3.39E+05		(R)AGTGVI	(R)A/G T/G/V/D N V/D/L/E/A A T R K(G)		117.4	539.6108	1616.824	-0.006	-3.7	4.56	57391.2	6.29	HUMAN	O43175	D-3-phosphoglycerate dehydrogenase
2753	dirMS_041712_NAR_60mM_plus.11742.11742.2	2	15.24	0	1.31E+06		(R)FAQHGT	(R)F A/Q/H G\T|F E/Y\E/Y/S Q\R(W)		118.65	881.8928	1762.782	-0.0037	-2.1	5.4	26419.2	5.1	HUMAN	H0Y9K7	Splicing factor, proline- and glutamine-rich (Fragment)
2754	dirMS_041712_NAR_60mM_plus.12409.12534.3	3	15.24	1	3.85E+06		(K)LFNLSKE	(K)L/F|N L/S K/E D D/V/R(Q)		121.6	445.9046	1335.69	0.0089	6.7	4.56	25082	11.29	HUMAN	A2A3R5	40S ribosomal protein S6
2755	dirMS_041712_NAR_60mM_plus.12935.12982.3	3	15.24	0	1.26E+06		(K)VNGRPL	(K)V/N|G\R\P L E|M/I/E/P/R(T)		124.65	470.9225	1410.752	0.0008	0.5	6.11	16559.1	10.36	HUMAN	P62249	40S ribosomal protein S16
2756	dirMS_041712_NAR_60mM_plus.17087.17087.3	3	15.24	1	7.26E+06		(K)LKVPEW	(K)L/K/V/P E\W/V/D/T V K(L)		145.72	438.5872	1313.746	0.0007	0.5	6.07	16060.2	10.31	HUMAN	P39019	40S ribosomal protein S19
2757	dirMS_041712_NAR_60mM_plus.9917.9918.3	3	15.24	1	1.03E+06		(R)SLNESKI	(R)S L|N E/S/K|I/E/I E|R(L)		109.93	439.9069	1317.701	0.0053	4	4.78	334221.3	6.44	HUMAN	P15924	Desmoplakin
2758	dirMS_041712_NAR_120mM_minus.14365.14529.3	3	15.23	1	1.43E+05	M313m	(R)RTVQSLI	(R)R T V Q\S\L/E|I|D|L/D/S m R(N)	m:Oxidized methionine	133.25	560.2849	1662.848	15.9922	-1.6	4.56	43773.3	5.26	HUMAN	F8VZY9	Keratin, type I cytoskeletal 18
2759	dirMS_041712_NAR_120mM_minus.5611.5769.3	3	15.23	0	1.21E+06		(R)AEAEAG	(R)A E|A|E|A/G S P R P D|P/R(E)		84.47	451.5563	1352.655	-0.001	-0.7	4.68	558007.1	5.44	HUMAN	P58107	Epiplakin
2760	dirMS_041712_NAR_30mM_minus2.15286.15286.2	2	15.23	0	4.79E+04		(K)LCDFGF/	(K)L/C|D/F G\F/A R(A)	C:Carbamidomethylation	135.8	493.2343	985.456	0.0053	5.4	5.84	143554.7	5.57	HUMAN	A8MU99	Serine/threonine-protein kinase 36
2761	dirMS_041712_NAR_30mM_plus2.9020.9020.2	2	15.23	0	5.79E+05		(R)LATNTS/	(R)L A|T/N T/S A/P D/L/K(N)		100.57	565.801	1130.605	-0.0105	-9.2	5.84	26233.5	10.95	HUMAN	B4DSQ1	Microtubule-associated protein
2762	dirMS_041712_NAR_40mM_plus2.19022.19110.3	3	15.23	1	3.19E+05		(R)GGPNIIT	(R)G G P|N|I|I|T L/A D I V/K/D/P V S R(T)		178.95	622.3522	1865.049	-0.007	-3.8	5.96	45481.2	7.85	HUMAN	E7EU96	Casein kinase II subunit alpha
2763	dirMS_041712_NAR_60mM_plus.13790.14340.3	3	15.23	1	2.88E+07		(K)ASLENSL	(K)A/S|L/E/N S/L R E V E/A/R(Y)		129.92	491.928	1473.766	0.0039	2.6	4.79	43773.3	5.26	HUMAN	F8VZY9	Keratin, type I cytoskeletal 18
2764	dirMS_041712_NAR_60mM_plus.17760.17760.3	3	15.23	0	3.72E+05		(R)SLESLHS	(R)S L|E/S/L H S F V A A/A T K(E)		149.9	487.5963	1460.774	0	0	6.47	81007.1	6.31	HUMAN	E9PMV1	Plectin (Fragment)
2765	dirMS_041712_NAR_60mM_minus.17101.17101.3	3	15.22	0	8.20E+04		(K)LLDSSTV	(K)L/L|D/S/S T/V T H L F K(I)		155.38	454.2519	1360.747	-0.0059	-4.4	6.74	25746.2	6.77	HUMAN	B4DXJ9	Proteasome (Prosome, macropain) subunit, alpha type, 6, isoform CRA_a
2766	dirMS_041712_NAR_120mM_plus.9709.9726.3	3	15.22	1	1.26E+06		(K)LEAEIAT	(K)L/E|A|E/I A T Y R/R(L)		104.37	407.8899	1221.659	-0.0035	-2.8	6.14	43773.3	5.26	HUMAN	F8VZY9	Keratin, type I cytoskeletal 18
2767	dirMS_041712_NAR_20mM_plus.15672.15672.3	3	15.22	0	7.37E+05		(K)VGINYQ	(K)V G I N Y Q|P P/T/V\V/P G G D L A K(V)		179.22	608.9991	1824.985	-0.0027	-1.5	5.8	49012.3	4.9	HUMAN	A8MUB1	Tubulin alpha-4A chain
2768	dirMS_041712_NAR_20mM_plus.16433.16706.2	2	15.22	0	5.45E+05		(K)LGGSAV	(K)L G G\S A/V|I|S L|E/G/K|P L(-)		185.73	670.8917	1340.778	-0.0023	-1.7	6	22956	8.8	HUMAN	E9PK25	Cofilin-1
2769	dirMS_041712_NAR_40mM_plus2.13987.13987.3	3	15.22	0	2.12E+05		(K)VALLLLD	(K)V A|L|L/L/L/D|Q/G A S/P H A A/A K(N)		148.2	558.9886	1674.954	-0.0025	-1.5	6.71	482683.6	6.07	HUMAN	Q12955	Ankyrin-3
2770	dirMS_041712_NAR_50mM_plus2.6129.6129.3	3	15.22	0	1.25E+05		(R)SGSMDI	(R)S/G|S|M/D/P S/G A H P S V R(Q)		96.5	462.214	1384.627	0.0001	0	6.46	48340.6	8.84	HUMAN	Q07666	KH domain-containing, RNA-binding, signal transduction-associated protein 1
2771	dirMS_041712_NAR_60mM_plus.14508.14508.3	3	15.22	1	7.09E+05		(R)ELISNSSI	(R)E L|I/S/N/S/S/D A\L D K I R(Y)		132.23	520.952	1560.823	0.0187	12	4.56	20151.2	4.58	HUMAN	G3V2J8	Heat shock protein HSP 90-alpha (Fragment)
2772	dirMS_041712_NAR_60mM_plus.17768.17768.3	3	15.22	1	1.13E+05		(K)WVAIQS	(K)W/V/A/I Q S V S A/W/P E K R(G)		150.05	552.9655	1656.886	-0.0037	-2.2	8.75	6412.4	9.52	HUMAN	J3KSS4	Cytosolic non-specific dipeptidase (Fragment)
2773	dirMS_041712_NAR_20mM_minus.11193.11429.2	2	15.21	0	1.17E+07	M148m	(R)DLTDYLr	(R)D L|T|D/Y/L/m/K(I)	m:Oxidized methionine	162.17	507.7443	998.486	15.995	0.1	4.21	37482.1	5.36	HUMAN	E9PG30	Actin, gamma-enteric smooth muscle
2774	dirMS_041712_NAR_30mM_minus2.11048.11048.2	2	15.21	0	5.11E+05		(K)IVVVTA(	(K)I V|V/V/T\A G V/R(Q)		114.15	457.2948	913.583	-0.0006	-0.7	9.75	25445.8	5.52	HUMAN	A8MW50	L-lactate dehydrogenase (Fragment)
2775	dirMS_041712_NAR_50mM_minus2.12774.12774.3	3	15.21	1	1.29E+05		(R)TEMENE	(R)T/E|M E N E/F|V/L I K K(D)		140.62	494.2621	1480.772	0.0002	0.1	4.78	13588.1	4.66	HUMAN	F8VP67	Keratin, type II cytoskeletal 8 (Fragment)
2776	dirMS_041712_NAR_50mM_minus2.7053.7053.3	3	15.21	1	2.04E+05		(K)GAVAED	(K)G A V|A|E D/G D/E L/R T E P E/A/K(K)		106.92	596.2904	1786.845	0.0113	6.3	4.08	19284.3	5.23	HUMAN	G3V3C7	DNA-(apurinic or apyrimidinic site) lyase (Fragment)
2777	dirMS_041712_NAR_50mM_minus2.9684.9684.3	3	15.21	1	1.97E+05		(R)ALEEAM	(R)A L|E|E A/M E/Q K A E L E/R(L)		122.57	549.6072	1646.805	0.0016	1	4.33	227783.5	5.5	HUMAN	P35579	Myosin-9
2778	dirMS_041712_NAR_60mM_minus.11188.11189.3	3	15.21	1	8.90E+05	M232m	(R)MSmKE\	(R)M/S/m K E V D E Q|M|L/N|V|Q N K(N)	m:Oxidized methionine	122.73	647.3011	1923.897	15.9915	-1.8	4.68	40069.4	4.83	HUMAN	E7EWR1	Tubulin beta chain
2779	dirMS_041712_NAR_20mM_plus.8097.8114.2	2	15.21	0	8.65E+04		(R)AAPGAE	(R)A/A/P/G/A E/F A|P/N K(R)		125.65	536.7749	1072.542	0.0003	0.3	6.05	43979.5	8.92	HUMAN	F5GYZ3	Non-POU domain-containing octamer-binding protein
2780	dirMS_041712_NAR_30mM_plus2.11347.11364.2	2	15.21	0	6.30E+05		(R)MGESDI	(R)M/G|E|S/D/D/S I L/R(L)		113.68	561.7585	1122.51	0.0002	0.1	4.03	9225.3	9.16	HUMAN	P63220	40S ribosomal protein S21
2781	dirMS_041712_NAR_30mM_plus2.14333.14333.2	2	15.21	0	9.53E+05		(R)MGQM/	(R)M/G Q/M A|M|G/G A/M|G\I N N R(G)		127.35	769.8376	1538.67	-0.0015	-1	9.5	43979.5	8.92	HUMAN	F5GYZ3	Non-POU domain-containing octamer-binding protein
2782	dirMS_041712_NAR_60mM_plus.11262.11262.2	2	15.21	0	2.12E+06		(K)GIVEFAS	(K)G I|V/E/F/A S K P A A R(K)		116.18	623.3504	1245.695	-0.0015	-1.2	8.75	76262.2	9.53	HUMAN	P23246	Splicing factor, proline- and glutamine-rich
2783	dirMS_041712_NAR_60mM_plus.14804.14867.3	3	15.21	1	4.49E+05		(R)ELAVQI\	(R)E L/A/V/Q/I/Y/E E/A R K(F)		133.9	483.5949	1448.774	-0.0042	-2.9	4.79	73386.6	8.73	HUMAN	B4DXX7	ATP-dependent RNA helicase DDX3Y
2784	dirMS_041712_NAR_50mM_minus2.12131.12131.3	3	15.2	0	9.09E+03		(K)AASAGV	(K)A A|S/A/G V/P Y H/G E V/P V S L A R(A)		135.98	594.315	1780.934	-0.0036	-2	6.79	272689.3	5.79	HUMAN	O15020	Spectrin beta chain, non-erythrocytic 2
2785	dirMS_041712_NAR_50mM_minus2.8927.8927.3	3	15.2	0	2.36E+05		(R)VMLGET	(R)V M|L/G|E T N P A D/S K/P G T I R(G)		118.3	595.9744	1785.916	-0.0077	-4.3	6.04	15431.7	9	HUMAN	E7ERL0	Nucleoside diphosphate kinase A
2786	dirMS_041712_NAR_60mM_minus.12074.12130.3	3	15.2	1	3.97E+05		(K)QQEAAL	(K)Q Q/E/A/A/L/E/L E E/L\K|K(K)		127.52	510.2782	1528.822	-0.0017	-1.1	4.49	54214.9	5.66	HUMAN	C9J813	Caldesmon (Fragment)
2787	dirMS_041712_NAR_20mM_plus.16625.16625.2	2	15.2	0	1.99E+05		(K)VQIAVA	(K)V Q|I|A/V/A/N A Q\E/L\L/Q/R(M)		186.25	776.9419	1552.881	-0.004	-2.6	5.97	10728.1	9.15	HUMAN	Q9Y5L4	Mitochondrial import inner membrane translocase subunit Tim13
2788	dirMS_041712_NAR_50mM_plus2.12219.12219.3	3	15.2	0	3.98E+05		(K)IVLEDGT	(K)I V|L/E/D G T/L/H V T E G S G R(Y)		132.23	561.6272	1682.871	-0.0037	-2.2	4.65	62748.9	5.95	HUMAN	Q16555	Dihydropyrimidinase-related protein 2
2789	dirMS_041712_NAR_60mM_plus.10750.11170.3	3	15.2	1	6.13E+06		(K)TAVCDIF	(K)T/A|V/C D/I/P P R G L K(M)	C:Carbamidomethylation	115.45	442.9125	1326.72	0.0031	2.3	8.41	50199.4	4.75	HUMAN	A6NNZ2	Tubulin beta-8 chain-like protein LOC260334
2790	dirMS_041712_NAR_120mM_minus.7446.7510.3	3	15.19	1	7.50E+05		(K)EAETRAI	(K)E/A|E/T/R A E/F/A/E/R(S)		94.98	436.8772	1308.618	-0.0008	-0.6	4.49	31923.6	4.85	HUMAN	B7Z596	Tropomyosin alpha-1 chain
2791	dirMS_041712_NAR_30mM_minus2.15455.15565.2	2	15.19	0	3.22E+05		(R)AAPFSLE	(R)A A|P/F/S|L E/Y R(V)		138.3	527.2782	1053.536	0.0128	12.1	6.04	33115.9	5.54	HUMAN	Q15181	Inorganic pyrophosphatase
2792	dirMS_041712_NAR_50mM_minus2.16936.16936.3	3	15.19	1	9.17E+04		(K)AAILPTS	(K)A A/I/L/P T/S I/F L T N K K(G)		164.28	506.3077	1516.91	-0.0012	-0.8	10	61676.9	5.5	HUMAN	B4DV00	Protein arginine N-methyltransferase 5
2793	dirMS_041712_NAR_60mM_minus.19745.19893.3	3	15.19	1	1.81E+06		(K)EIIDLVLI	(K)E I/I/D/L/V/L/D/R I R(K)		179.88	452.2739	1354.805	0.0019	1.4	4.56	58642	4.95	HUMAN	F5H5D3	Tubulin alpha-1C chain
2794	dirMS_041712_NAR_30mM_plus2.12160.12160.2	2	15.19	0	4.46E+05		(R)GGAEGE	(R)G G\A|E/G E/L|Q/A L R(A)		116.58	550.7883	1100.57	-0.0001	-0.1	4.53	533778	5.74	HUMAN	Q15149	Plectin
2795	dirMS_041712_NAR_30mM_plus2.19785.19785.2	2	15.19	0	2.33E+05		(K)GFAFVQ	(K)G F\A|F/V|Q/Y|V/N E R(N)		157.57	665.3326	1329.659	-0.0007	-0.5	6	15791.7	9.22	HUMAN	B4DSU6	Heterogeneous nuclear ribonucleoproteins C1/C2
2796	dirMS_041712_NAR_30mM_plus2.19959.19959.2	2	15.19	0	5.04E+04		(K)TFESLVI	(K)T/F|E|S L/V D/F S K(A)		158.22	586.793	1172.583	-0.0047	-4	4.37	35407.1	6.73	HUMAN	E9PF18	Hydroxyacyl-coenzyme A dehydrogenase, mitochondrial
2797	dirMS_041712_NAR_60mM_plus.6934.6974.3	3	15.19	1	2.81E+05		(R)VISSIEQI	(R)V/I|S S I/E/Q/K T E/R(N)		94.73	430.5686	1289.706	-0.0147	-11.4	6.11	28196	4.76	HUMAN	P31946	14-3-3 protein beta/alpha
2798	dirMS_041712_NAR_50mM_minus2.16418.16418.3	3	15.18	0	1.24E+05		(R)IMEVID/	(R)I/M|E/V/I/D A I T/T T/A/Q S H/Q R(T)		161.6	638.6598	1913.975	-0.0101	-5.3	5.32	85815.7	7.35	HUMAN	P17858	6-phosphofructokinase, liver type
2799	dirMS_041712_NAR_50mM_minus2.17018.17038.3	3	15.18	0	1.55E+05		(R)LLEVLSG	(R)L L|E|V/L\S/G E I L/P K/P\T K(G)		165.35	546.3329	1636.988	-0.0042	-2.6	6.14	13611	6.13	HUMAN	E9PJZ2	Spectrin beta chain, non-erythrocytic 2 (Fragment)
2800	dirMS_041712_NAR_50mM_minus2.7016.7166.3	3	15.18	1	7.15E+05		(K)GAVAED	(K)G A|V|A|E/D/G D E L R T E/P E/A K(K)		107.32	596.2904	1786.845	0.0113	6.3	4.08	19284.3	5.23	HUMAN	G3V3C7	DNA-(apurinic or apyrimidinic site) lyase (Fragment)
2801	dirMS_041712_NAR_60mM_minus.13595.13626.3	3	15.18	0	2.18E+05		(K)FADEHV	(K)F/A|D E H\V|P G S/P F T V K(I)		135.83	510.9286	1530.759	0.0125	8.2	5.32	258215.9	5.48	HUMAN	E7EN95	Filamin-B
2802	dirMS_041712_NAR_60mM_minus.13829.14285.3	3	15.18	1	9.82E+06		(R)IQLVEEE	(R)I/Q\L|V/E E/E/L D/R A Q E/R(L)		138.48	576.6347	1727.892	-0.0027	-1.6	4.25	31923.6	4.85	HUMAN	B7Z596	Tropomyosin alpha-1 chain
2803	dirMS_041712_NAR_60mM_minus.6491.6491.3	3	15.18	1	1.71E+05		(R)AQLGGF	(R)A Q|L|G G/P E A A/K S D E T A A K(-)		94.9	548.6116	1643.824	-0.0033	-2	4.68	3888.3	5	HUMAN	C9J3N8	Heat shock protein beta-1
2804	dirMS_041712_NAR_120mM_plus.14159.14159.4	4	15.18	1	5.85E+05		(R)LEAPELP	(R)L E|A/P E/L/P V/K V/N P/T K P R(R)		127.43	447.7616	1788.038	-0.0132	-7.4	8.59	10558	9.4	HUMAN	H0YE28	Protein C11orf31 (Fragment)
2805	dirMS_041712_NAR_120mM_plus.15296.15441.3	3	15.18	0	1.10E+06		(K)HLEINPI	(K)H/L|E I N\P D|H|P/I/V E T L R(Q)		133.9	594.9869	1782.95	-0.0036	-2	5.27	83605	4.97	HUMAN	P08238	Heat shock protein HSP 90-beta
2806	dirMS_041712_NAR_20mM_plus.15105.15105.2	2	15.18	0	2.17E+05		(K)GLAITFV	(K)G L A\I|T|F/V|S/D E N D A K(I)		175.3	740.3693	1479.733	-0.0012	-0.8	4.03	40918.8	9.33	HUMAN	B4DP52	HCG2005638, isoform CRA_c
2807	dirMS_041712_NAR_30mM_plus2.21121.21121.2	2	15.18	0	6.77E+05		(K)IPDWFLI	(K)I/P/D/W F L N R(Q)		165.77	530.785	1060.557	0.0053	5	5.84	9767.1	11.41	HUMAN	J3JS69	40S ribosomal protein S18
2808	dirMS_041712_NAR_30mM_minus2.17127.17127.2	2	15.17	0	4.44E+04		(K)LGINSLC	(K)L/G/I/N/S/L/Q E L/K(Q)		146.95	557.8242	1114.647	-0.0055	-5	6	25978.5	4.91	HUMAN	C9J502	Prostate leucine zipper variant 2
2809	dirMS_041712_NAR_30mM_minus2.19620.19620.2	2	15.17	0	4.38E+05		(R)ASLSLW	(R)A S/L/S\L/W/Q/A M E/K(G)		162.85	632.3234	1263.64	-0.0007	-0.5	6.05	266161	5.68	HUMAN	E9PPU0	Epiplakin
2810	dirMS_041712_NAR_40mM_minus2.12770.12835.3	3	15.17	0	1.42E+05		(K)IEVIKPG	(K)I/E|V|I/K/P/G D/L/G V D L T S K(L)		159.03	561.989	1683.953	-0.0003	-0.2	4.56	28071.3	8.6	HUMAN	P38117	Electron transfer flavoprotein subunit beta
2811	dirMS_041712_NAR_50mM_minus2.10559.10561.3	3	15.17	1	2.88E+05		(K)NQVAM	(K)N Q|V|A/M/N/P T N/T V/F/D A K R(L)		128.55	602.6379	1805.896	0.0029	1.6	8.75	20069.4	6.74	HUMAN	E9PK54	Heat shock cognate 71 kDa protein (Fragment)
2812	dirMS_041712_NAR_50mM_minus2.13380.13380.3	3	15.17	0	3.49E+05		(R)EQTLSP1	(R)E Q/T L/S/P T/I/T/S/G L H N I A R(S)		143.7	613.3274	1837.977	-0.009	-4.9	6.85	128523.4	7.06	HUMAN	B7ZL00	Protein transport protein Sec31A
2813	dirMS_041712_NAR_60mM_minus.10376.10376.3	3	15.17	1	6.05E+05	M88m	(K)DVDEAY	(K)D V|D/E|A Y\m N/K V E L E/S R(L)	m:Oxidized methionine	118.37	605.2791	1797.832	15.9904	-2.5	4.18	13588.1	4.66	HUMAN	F8VP67	Keratin, type II cytoskeletal 8 (Fragment)
2814	dirMS_041712_NAR_120mM_plus.13278.13324.3	3	15.17	2	6.46E+05		(R)ELNITAA	(R)E L/N|I/T/A/A\K E I/E/V/G G G R K(A)		123.02	595.6661	1784.987	-0.0027	-1.5	6.33	21311.5	10.28	HUMAN	B5MCP9	40S ribosomal protein S7
2815	dirMS_041712_NAR_120mM_plus.17586.18122.3	3	15.17	1	2.34E+06		(R)RAPFDLI	(R)R A\P F\D|L|F E/N/R(K)		146.97	422.22	1264.643	0.0022	1.7	6.07	85057.6	4.94	HUMAN	P07900	Heat shock protein HSP 90-alpha
2816	dirMS_041712_NAR_30mM_plus2.20824.20913.2	2	15.17	0	3.07E+05		(K)LVLPSLIS	(K)L V|L|P/S L/I/S S/R(I)		163.97	542.8382	1084.672	-0.0033	-3.1	9.75	39066	6.61	HUMAN	A6PVW9	Selenium binding protein 1 (Fragment)
2817	dirMS_041712_NAR_60mM_plus.12115.12115.2	2	15.17	1	1.52E+06		(R)DGKLVS	(R)D/G K/L|V/S\E S S D V/L/P K(-)		120.25	737.3869	1473.78	-0.013	-8.8	4.56	56607.4	5.37	HUMAN	F8VXB4	Keratin, type II cytoskeletal 8
2818	dirMS_041712_NAR_60mM_plus.12339.12339.3	3	15.17	1	4.98E+05		(R)TSDVGG	(R)T/S/D/V/G/G Y Y Y E/K I\E R(T)		121.18	560.6017	1679.791	-0.0006	-0.4	4.68	271827.6	4.73	HUMAN	P46821	Microtubule-associated protein 1B
2819	dirMS_041712_NAR_120mM_minus.16063.16063.4	4	15.16	2	2.10E+05		(K)KQGGLG	(K)K Q G G L/G|P M N\I/P L|V S D/P K R(T)		141.62	477.5179	1907.053	-0.0034	-1.8	9.99	22338.2	8.65	HUMAN	Q06830	Peroxiredoxin-1
2820	dirMS_041712_NAR_30mM_minus2.14980.14980.2	2	15.16	0	5.54E+05	M316m	(R)ISEQFTA	(R)I/S\E/Q/F/T A/m F R(R)	m:Oxidized methionine	135.18	623.3014	1229.598	15.9972	1.8	6	42831.5	4.86	HUMAN	A8K854	HCG1983504, isoform CRA_f
2821	dirMS_041712_NAR_50mM_minus2.11493.11493.3	3	15.16	0	1.45E+05		(K)VSQPIEC	(K)V/S Q/P I|E G H A/A S F A/Q F K(M)		133.52	572.9609	1716.87	-0.0022	-1.3	6.72	193379.8	5.48	HUMAN	Q00610	Clathrin heavy chain 1
2822	dirMS_041712_NAR_60mM_minus.10515.10515.3	3	15.16	1	1.64E+06		(K)TLQEVTI	(K)T L/Q/E V/T/E|M D/S V/K R(Y)		119.37	512.5957	1535.773	-0.0008	-0.5	4.68	558007.1	5.44	HUMAN	P58107	Epiplakin
2823	dirMS_041712_NAR_60mM_minus.13694.13694.3	3	15.16	1	2.69E+05		(K)SLLSNLD	(K)S L/L/S/N L\D E V/K K(E)		136	415.9055	1245.705	-0.0029	-2.4	5.79	96649	6.13	HUMAN	Q8WUM4	Programmed cell death 6-interacting protein
2824	dirMS_041712_NAR_30mM_plus2.11938.11973.2	2	15.16	0	6.57E+05		(K)LEAEIAT	(K)L/E|A|E|I\A|T Y R(R)		116.08	533.2814	1065.557	-0.002	-1.9	4.53	43773.3	5.26	HUMAN	F8VZY9	Keratin, type I cytoskeletal 18
2825	dirMS_041712_NAR_50mM_plus2.19780.19780.3	3	15.16	0	3.09E+05		(K)ILFIFIDSI	(K)I L|F|I/F I/D S D H T D N Q/R(I)		172.6	611.9758	1833.913	-0.0001	-0.1	4.41	51524.1	4.82	HUMAN	F5H8J2	Protein disulfide-isomerase
2826	dirMS_041712_NAR_60mM_plus.16493.16946.3	3	15.16	0	1.18E+06		(K)TTISVAH	(K)T T|I/S/V/A/H/L/L A A/R(Q)		144.27	418.2478	1252.737	-0.0083	-6.7	9.44	34143.2	10.07	HUMAN	B4DT23	Protein FAM98A
2827	dirMS_041712_NAR_60mM_plus.5762.5762.3	3	15.16	1	8.61E+04	M117m	(R)ARQENC	(R)A R Q\E N G\m|P E/E/P A T T A R(G)	m:Oxidized methionine	88.33	591.9441	1757.824	15.9942	-0.4	4.79	64356.2	9.96	HUMAN	O96013	Serine/threonine-protein kinase PAK 4
2828	dirMS_041712_NAR_30mM_minus2.7408.7408.2	2	15.15	0	1.44E+05		(R)FGGSGS	(R)F/G/G\S/G/S Q/V/D/S A/R(M)		94.65	584.2729	1167.539	-0.0004	-0.3	5.84	86346.5	5.05	HUMAN	E7EW34	26S proteasome non-ATPase regulatory subunit 2
2829	dirMS_041712_NAR_40mM_minus2.11961.11961.3	3	15.15	0	2.64E+05		(K)DAGEGL	(K)D A|G|E|G/L/L A V Q/I T D P E G K\P K(K)		153.95	646.6785	1938.018	0.0031	1.6	4.32	283472.4	5.71	HUMAN	P21333	Filamin-A

2830	dirMS_041712_NAR_50mM_minus2.7744.7765.3	3	15.15	0	2.44E+05		(R)LESGMC	(R)L/E|S G M Q/N/M/S I/H/T/K(T)		111.27	492.5712	1475.698	0.0009	0.6	6.75	56607.4	5.37	HUMAN	F8VXB4	Keratin, type II cytoskeletal 8
2831	dirMS_041712_NAR_60mM_minus.5713.5713.2	2	15.15	1	4.42E+06		(K)DSYVGD	(K)D S/Y/V/G/D/E A Q S/K/R(G)		87.58	677.8144	1354.623	-0.0018	-1.3	4.56	32616.5	5.18	HUMAN	A6NL76	Actin, alpha skeletal muscle
2832	dirMS_041712_NAR_120mM_plus.11645.11645.4	4	15.15	1	2.63E+05		(R)LQQLGE	(R)L Q\Q/L|G/E/A H/Q A E T E V L R R(E)		114.02	495.2678	1978.046	0.0029	1.5	5.5	97620.5	5.02	HUMAN	F5H4J1	Nuclear mitotic apparatus protein 1 (Fragment)
2833	dirMS_041712_NAR_30mM_plus2.17148.17290.2	2	15.15	0	3.36E+05		(K)TVFGVEI	(K)T V|F/G|V|E P|D L T R(E)		143.33	617.3263	1233.647	-0.0021	-1.7	4.37	53219.8	5.66	HUMAN	Q96KP4	Cytosolic non-specific dipeptidase
2834	dirMS_041712_NAR_30mM_plus2.6622.6717.2	2	15.15	0	1.10E+06		(K)SLDEME	(K)S/L|D|E M/E/K(Q)		90	426.1971	851.381	0.0054	6.4	4.14	26419.2	5.1	HUMAN	H0Y9K7	Splicing factor, proline- and glutamine-rich (Fragment)
2835	dirMS_041712_NAR_50mM_plus2.14006.14006.3	3	15.15	1	6.07E+05		(K)GGELVY	(K)G G\E/L/V/Y/T/D S E A R D V/F E K(A)		141.53	638.9735	1914.908	-0.002	-1.1	4.18	533778	5.74	HUMAN	Q15149	Plectin
2836	dirMS_041712_NAR_50mM_plus2.19975.20249.3	3	15.15	0	2.96E+05		(R)DYLHLPI	(R)D Y L H L\P\P E\I|V|P/A T L R(R)		173.92	578.658	1733.959	0.001	0.6	5.32	19924.8	9.99	HUMAN	F6U211	40S ribosomal protein S10
2837	dirMS_041712_NAR_60mM_plus.17492.17492.3	3	15.15	1	3.74E+05		(R)KVPGVT	(R)K\V P G V\T A\I\E|L|D/E/D/T/G T F R(I)		148.18	650.0017	1948.002	-0.0117	-6	4.32	68668.3	5.77	HUMAN	B4DXZ6	Fragile X mental retardation syndrome-related protein 1
2838	dirMS_041712_NAR_120mM_minus.4243.4243.3	3	15.14	1	6.83E+05		(R)EPGPAG	(R)E P/G/P/A G R G D G/D S/G R(S)		70.47	443.2041	1327.599	-0.0008	-0.6	4.56	558007.1	5.44	HUMAN	P58107	Epiplakin
2839	dirMS_041712_NAR_50mM_minus2.14124.14124.3	3	15.14	0	3.40E+05		(K)SPAPPLL	(K)S/P/A/P/P L L H V/A A L G Q/K(Q)		148.45	500.2911	1498.874	-0.0153	-10.2	8.49	17549.1	10.39	HUMAN	E5RFJ3	DBIRD complex subunit KIAA1967 (Fragment)
2840	dirMS_041712_NAR_50mM_minus2.16703.16703.3	3	15.14	1	9.23E+04		(R)DSEQVA	(R)D/S/E/Q/V/A E/L K/Q E L A T L K(S)		163.05	601.3217	1801.954	-0.0036	-2	4.41	109459.8	4.94	HUMAN	F5GYR8	General vesicular transport factor p115
2841	dirMS_041712_NAR_50mM_minus2.8818.8839.3	3	15.14	1	2.50E+06	M227m	(R)mQKEIT.	(R)m Q K E\I|T|A|L|A|P S T M K(I)	m:Oxidized methionine	118.07	522.2727	1548.812	15.9911	-2.4	8.35	32616.5	5.18	HUMAN	A6NL76	Actin, alpha skeletal muscle
2842	dirMS_041712_NAR_60mM_minus.13847.14329.3	3	15.14	1	9.82E+06		(R)IQLVEEE	(R)I/Q|L|V/E E/E L D/R A Q E/R(L)		138.48	576.6347	1727.892	-0.0027	-1.6	4.25	31923.6	4.85	HUMAN	B7Z596	Tropomyosin alpha-1 chain
2843	dirMS_041712_NAR_60mM_minus.15008.15029.3	3	15.14	0	4.49E+06		(R)EHALLA'	(R)E H A\L\L|A|Y/T/L/G V/K(Q)		144.57	438.9202	1314.742	0.0044	3.4	6.85	50482.3	9.31	HUMAN	P68104	Elongation factor 1-alpha 1
2844	dirMS_041712_NAR_20mM_plus.10634.10634.2	2	15.14	0	4.78E+04		(K)GILAADI	(K)G I|L|A|A|D E\S T\G S I/A/K(R)		145.93	666.8531	1332.7	-0.0016	-1.2	4.37	30711.4	7.15	HUMAN	H3BPS8	Fructose-bisphosphate aldolase A (Fragment)
2845	dirMS_041712_NAR_20mM_plus.12947.12947.2	2	15.14	0	4.05E+05		(K)LSELEAA	(K)L/S|E|L/E A/A\L Q R(A)		161.95	565.3132	1129.621	-0.002	-1.8	4.53	56607.4	5.37	HUMAN	F8VXB4	Keratin, type II cytoskeletal 8
2846	dirMS_041712_NAR_50mM_plus2.11167.11167.3	3	15.14	0	3.91E+05		(K)LQAEAP	(K)L/Q/A E/A P/H I V V/G T P G R(V)		126.6	515.6223	1544.854	-0.002	-1.3	6.75	41517.9	5.45	HUMAN	E7EQG2	Eukaryotic initiation factor 4A-II
2847	dirMS_041712_NAR_120mM_minus.20018.20018.3	3	15.13	0	7.83E+04		(R)INLWHL	(R)I/N|L\W/H L/E/I T D R(S)		165.22	470.5897	1409.754	0.001	0.7	5.32	52204.6	5.82	HUMAN	P63151	Serine/threonine-protein phosphatase 2A 55 kDa regulatory subunit B alpha isoform
2848	dirMS_041712_NAR_30mM_minus2.17811.17901.2	2	15.13	0	1.96E+05		(K)FFFDPSA	(K)F/F|F/D|P/S A/R(D)		151.65	493.7405	986.473	0.0007	0.7	5.84	266161	5.68	HUMAN	E9PPU0	Epiplakin
2849	dirMS_041712_NAR_30mM_minus2.18339.18573.2	2	15.13	0	4.14E+05		(R)ADLIAYL	(R)A/D|L|I|A/Y/L/K(K)		154.65	453.7668	906.529	-0.0032	-3.5	5.88	11447.2	9.81	HUMAN	C9JFR7	Cytochrome c (Fragment)
2850	dirMS_041712_NAR_50mM_minus2.15210.15210.3	3	15.13	0	4.10E+05		(K)AETLASS	(K)A E|T/L/A/S S/L Q E/D L/A H T R(N)		154.98	581.2998	1741.872	0.0133	7.7	4.65	88624.6	5.09	HUMAN	C9J8U1	Cytospin-A (Fragment)
2851	dirMS_041712_NAR_120mM_plus.8321.8337.3	3	15.13	1	9.59E+05		(R)VITEEEK	(R)V/I|T/E/E/E/K N F K(A)		97.13	412.8845	1236.647	-0.0081	-6.5	4.79	18901.8	11.65	HUMAN	F5H1S2	60S ribosomal protein L13
2852	dirMS_041712_NAR_20mM_plus.9468.9641.2	2	15.13	0	3.16E+05		(K)LVLVGD	(K)L/V|L|V/G/D/G G T G K(T)		137.4	508.292	1015.578	-0.0015	-1.5	5.84	26394.9	6.5	HUMAN	B5MDF5	GTP-binding nuclear protein Ran
2853	dirMS_041712_NAR_20mM_plus.9802.9802.2	2	15.13	0	1.46E+05		(K)YQAVTA	(K)Y Q|A|V/T/A T/L E/E K(R)		139.28	626.8223	1252.642	-0.0046	-3.7	4.53	23633.9	11.02	HUMAN	P40429	60S ribosomal protein L13a
2854	dirMS_041712_NAR_50mM_plus2.15515.15515.3	3	15.13	0	1.51E+06		(R)LASTLVH	(R)L/A|S T L V H L\G E Y/Q A|A\V/D G A R(K)		149.68	657.6802	1971.029	-0.0034	-1.7	5.32	71165.3	5.57	HUMAN	J3KS13	Clathrin heavy chain 1
2855	dirMS_041712_NAR_50mM_plus2.20652.20652.3	3	15.13	1	1.70E+05		(R)VNSKFD'	(R)V N/S/K F D\T\I/Y\Q/I/L/L K(K)		177.62	561.3209	1681.952	-0.0042	-2.5	8.47	72626.7	9.13	HUMAN	Q14258	E3 ubiquitin/ISG15 ligase TRIM25
2856	dirMS_041712_NAR_60mM_plus.10013.10013.2	2	15.13	0	9.64E+05		(R)LESGMC	(R)L E|S G M\Q/N M S I/H/T|K(T)		110.1	738.3522	1475.698	-0.001	-0.7	6.75	56607.4	5.37	HUMAN	F8VXB4	Keratin, type II cytoskeletal 8
2857	dirMS_041712_NAR_120mM_minus.18191.18191.4	4	15.12	1	3.15E+05		(K)ILGSGIS:	(K)I L G S G\I/S/S/S/S V/L/H G M V F K K(E)		154.12	487.5223	1947.073	-0.0058	-3	10	58214.7	5.25	HUMAN	B4DEM7	T-complex protein 1 subunit theta
2858	dirMS_041712_NAR_120mM_minus.7300.7300.3	3	15.12	0	4.15E+05		(K)NNRPSE	(K)N/N|R P S E|G|P/L/Q/T/R(L)		94.1	456.9036	1368.698	-0.0016	-1.2	9.6	193379.8	5.48	HUMAN	Q00610	Clathrin heavy chain 1
2859	dirMS_041712_NAR_30mM_minus2.15304.15609.2	2	15.12	0	3.50E+05		(R)IVFQEFR	(R)I/V|F/Q E/F/R(Q)		137.48	469.7582	938.509	-0.0003	-0.3	6	233493.8	5.45	HUMAN	F8VTL3	Myosin-10
2860	dirMS_041712_NAR_50mM_minus2.11973.11973.3	3	15.12	1	3.56E+05		(K)AVLFCLS	(K)A/V|L F C/L/S/E D K/K(N)	C:Carbamidomethylation	136.05	437.2326	1309.682	0.0012	0.9	6.11	22956	8.8	HUMAN	E9PK25	Cofilin-1
2861	dirMS_041712_NAR_50mM_minus2.17426.17426.3	3	15.12	1	3.59E+05		(K)GYPPED	(K)G Y|P P E/D I/F N\L V G K K(V)		167.22	526.2832	1576.837	-0.0019	-1.2	6.07	40763.2	6.29	HUMAN	Q9NR45	Sialic acid synthase
2862	dirMS_041712_NAR_50mM_minus2.17921.17921.3	3	15.12	1	8.25E+05		(K)TFVNITP	(K)T F/V/N/I|T/P/A E V/G V L V G K D R(S)		171.32	639.0247	1915.065	-0.0052	-2.7	5.74	17630.6	11.62	HUMAN	I3L3D5	Profilin-1 (Fragment)
2863	dirMS_041712_NAR_60mM_minus.7132.7132.3	3	15.12	1	3.04E+05		(R)ELFESST	(R)E L|F/E/S/S T N N N K D/T K(Q)		98.72	542.9247	1626.761	-0.001	-0.6	4.68	150202.8	9.1	HUMAN	E7EW20	Unconventional myosin-VI
2864	dirMS_041712_NAR_60mM_minus.9298.9298.3	3	15.12	1	2.37E+05		(R)QDSAAV	(R)Q D/S/A/A/V/G F/D Y K/E K(L)		111.87	486.5639	1457.691	-0.0136	-9.3	4.56	61756.2	5.24	HUMAN	Q14247	Src substrate cortactin
2865	dirMS_041712_NAR_120mM_plus.11651.11651.2	2	15.12	1	8.55E+05		(R)NLKASLI	(R)N/L|K/A/S/L E N S L R(E)		114.02	622.8511	1244.696	-0.0008	-0.6	8.75	43773.3	5.26	HUMAN	F8VZY9	Keratin, type I cytoskeletal 18
2866	dirMS_041712_NAR_20mM_plus.19128.19128.3	3	15.12	0	1.99E+05		(K)TLETQS/	(K)T L E\T Q S/A L G/P/A L Q/A A F K(L)		202.6	582.6516	1745.943	-0.003	-1.7	5.66	120546	7.82	HUMAN	O95486	Protein transport protein Sec24A
2867	dirMS_041712_NAR_50mM_plus2.10975.10975.3	3	15.12	0	1.94E+05		(K)ADDGRI	(K)A/D|D/G R P F/P Q V\I/K(S)		125.1	448.2419	1342.711	-0.0002	-0.2	6	15633.5	9.15	HUMAN	H3BMQ8	Fructose-bisphosphate aldolase A (Fragment)
2868	dirMS_041712_NAR_50mM_plus2.14296.14315.3	3	15.12	0	1.11E+06		(R)IVATKPL	(R)I/V|A/T K/P L/Y/V/A/L/A Q R(K)		143.7	514.9831	1542.937	-0.0019	-1.2	9.99	65918.2	9.74	HUMAN	E7EQV3	Polyadenylate-binding protein 1
2869	dirMS_041712_NAR_50mM_plus2.16908.17051.3	3	15.12	1	6.11E+05		(K)ISMPDIE	(K)I/S\M/P|D I/D L/N L|K/G P K(V)		157.08	514.2847	1540.84	-0.0008	-0.5	5.96	629603.7	5.8	HUMAN	Q09666	Neuroblast differentiation-associated protein AHNAK
2870	dirMS_041712_NAR_50mM_plus2.19871.20032.3	3	15.12	0	4.71E+05		(K)HSQFIG'	(K)H S Q F\I G|Y|P/I/T/L/Y L\E K(E)		173.82	603.6541	1808.958	-0.0104	-5.7	6.75	83605	4.97	HUMAN	P08238	Heat shock protein HSP 90-beta
2871	dirMS_041712_NAR_60mM_plus.12326.12601.3	3	15.12	0	1.52E+06		(K)ADDGRI	(K)A/D|D/G R P/F|P Q V/I/K(S)		121.6	448.2414	1342.711	-0.0017	-1.3	6	15633.5	9.15	HUMAN	H3BMQ8	Fructose-bisphosphate aldolase A (Fragment)
2872	dirMS_041712_NAR_120mM_minus.14076.14076.2	2	15.11	1	4.39E+06		(K)FASFIDK	(K)F A|S/F|I/D K V/R(F)		130.18	541.7968	1082.599	-0.013	-12	8.75	19569.8	9.88	HUMAN	F8VRG4	Keratin, type II cytoskeletal 8 (Fragment)
2873	dirMS_041712_NAR_30mM_minus2.14298.14597.2	2	15.11	0	5.88E+06		(K)LLIEMEC	(K)L/L|I|E/M/E/Q/R(L)		133.73	516.281	1031.555	-0.0007	-0.6	4.53	20203.9	6.24	HUMAN	H0YJG0	Creatine kinase B-type (Fragment)
2874	dirMS_041712_NAR_30mM_minus2.18046.18066.2	2	15.11	0	4.12E+05		(R)LLLNNDI	(R)L/L|L|N/N/D/N L L/R(E)		153.13	599.3487	1197.695	-0.0049	-4.1	5.84	19945.8	4.97	HUMAN	B4DG50	F-actin-capping protein subunit alpha-2
2875	dirMS_041712_NAR_30mM_plus2.12527.12527.2	2	15.11	0	2.66E+05		(R)ELAEDG'	(R)E L|A|E/D G Y/S\G\V|E V R(V)		118.92	712.3382	1423.67	-0.0008	-0.6	4	13054.7	9.59	HUMAN	E9PJH4	40S ribosomal protein S3
2876	dirMS_041712_NAR_30mM_plus2.16992.16992.2	2	15.11	0	5.45E+05	M256m	(R)SLDmDS	(R)S/L/D m/D|S/I/I|A E/V\K(A)	m:Oxidized methionine	142.22	668.8384	1320.671	15.998	2.3	4.03	37296.3	5.3	HUMAN	F8VU64	Keratin, type II cytoskeletal 8
2877	dirMS_041712_NAR_30mM_plus2.21352.21375.2	2	15.11	0	2.35E+04		(R)GAFQNL	(R)G A F|Q/N/L/F/Q|S/V/R(E)		167.87	633.8315	1266.659	-0.0032	-2.5	9.75	68629.9	5.59	HUMAN	E7EVX6	Androgen receptor
2878	dirMS_041712_NAR_30mM_plus2.7713.8154.2	2	15.11	0	1.42E+06		(K)IAVAAQ	(K)I/A|V|A|A/Q/N/C Y K(V)	C:Carbamidomethylation	94.57	569.2905	1137.572	0.0016	1.4	8.59	31075.7	5.65	HUMAN	P60174	Triosephosphate isomerase
2879	dirMS_041712_NAR_60mM_plus.20646.20646.3	3	15.11	1	7.14E+05		(R)LDLAGR	(R)L D|L/A G R D L T D/Y/L/M/K(I)		168.28	541.9516	1623.841	-0.0008	-0.5	4.43	37482.1	5.36	HUMAN	E9PG30	Actin, gamma-enteric smooth muscle
2880	dirMS_041712_NAR_60mM_plus.5234.5234.3	3	15.11	0	2.64E+04		(R)TATEKP(	(R)T A|T E/K/P G/P A E A A/R(Q)		84.77	433.5671	1298.67	0.0168	13	5.81	49823.3	9.39	HUMAN	B4DQQ4	NADPH:adrenodoxin oxidoreductase, mitochondrial
2881	dirMS_041712_NAR_120mM_minus.6752.7046.2	2	15.1	0	6.08E+06	M46m	(R)HQGVm	(R)H|Q|G V|m\V/G M/G Q|K(D)	m:Oxidized methionine	91.72	594.2863	1171.571	15.9943	-0.5	8.76	32616.5	5.18	HUMAN	A6NL76	Actin, alpha skeletal muscle
2882	dirMS_041712_NAR_40mM_minus2.11383.11646.3	3	15.1	0	8.05E+05		(R)TIQVEN:	(R)T I|Q|V/E/N\S H L/I/L/T/G A/G A L N K(V)		151	660.3663	1979.092	-0.0077	-3.9	6.41	267260.7	5.95	HUMAN	Q13085	Acetyl-CoA carboxylase 1
2883	dirMS_041712_NAR_40mM_minus2.8603.8603.3	3	15.1	0	2.87E+05		(R)SVIDPVI	(R)S V I|D|P/V/P A/P V G D S H V D G A A K(S)		132.52	644.3329	1930.987	-0.0027	-1.4	4.41	58326.8	5.7	HUMAN	Q13177	Serine/threonine-protein kinase PAK 2
2884	dirMS_041712_NAR_120mM_plus.8135.8135.3	3	15.1	0	7.47E+05		(K)LFNHAN	(K)L/F N/H A N/Q E V/Q/R(H)		96.6	452.5653	1355.681	-0.0001	-0.1	6.75	87537	9.67	HUMAN	Q9Y446	Plakophilin-3
2885	dirMS_041712_NAR_30mM_plus2.10614.10628.2	2	15.1	0	3.95E+05		(K)TGTVSLI	(K)T G|T|V/S/L/E/V/R(L)		109.33	481.2676	961.531	-0.0034	-3.5	5.66	276046.1	6.01	HUMAN	P49327	Fatty acid synthase
2886	dirMS_041712_NAR_30mM_plus2.7652.8180.2	2	15.1	0	1.35E+07		(R)STFSTNY	(R)S T|F/S/T/N/Y/R(S)		94.53	488.2306	975.453	0.0009	0.9	8.46	43773.3	5.26	HUMAN	F8VZY9	Keratin, type I cytoskeletal 18
2887	dirMS_041712_NAR_40mM_plus2.17814.17814.3	3	15.1	0	1.42E+05		(R)DIKPQN	(R)D I K P Q N\L\L|L/D/P D T A V L K(L)		170.92	631.6874	1893.069	-0.0215	-11.4	4.43	47257	9.38	HUMAN	P49841	Glycogen synthase kinase-3 beta
2888	dirMS_041712_NAR_50mM_plus2.16612.16736.3	3	15.1	0	5.93E+05		(K)AVDIPHI	(K)A/V|D/I/P H/M/D I E A L/K(K)		155.63	484.589	1451.756	-0.0038	-2.6	4.54	25002	10.06	HUMAN	P62906	60S ribosomal protein L10a
2889	dirMS_041712_NAR_60mM_plus.13118.13118.3	3	15.1	0	5.35E+04		(K)VNGRPL	(K)V/N/G R P L E M/I/E/P/R(T)		125.57	470.9225	1410.752	0.0008	0.5	6.11	16559.1	10.36	HUMAN	P62249	40S ribosomal protein S16
2890	dirMS_041712_NAR_60mM_plus.13606.14176.3	3	15.1	0	2.61E+07		(R)NLDIERI	(R)N/L|D|I/E R/P T Y/T/N L N/R(L)		129.02	573.6312	1718.882	-0.003	-1.7	6.07	49012.3	4.9	HUMAN	A8MUB1	Tubulin alpha-4A chain
2891	dirMS_041712_NAR_60mM_plus.14913.15180.3	3	15.1	1	5.39E+06		(R)GYSFTT1	(R)G Y|S|F/T T/T A E R E I V/R(D)		134.97	543.9389	1629.823	-0.021	-12.9	6.14	23901.9	5.28	HUMAN	I3L1U9	Actin, cytoplasmic 2, N-terminally processed (Fragment)
2892	dirMS_041712_NAR_60mM_plus.7178.7178.2	2	15.1	0	1.72E+06		(K)STTTGHI	(K)S T|T/T/G H L/I Y K(C)		95.58	560.8031	1120.6	-0.0008	-0.7	8.34	12137.8	9.3	HUMAN	A6PW80	Elongation factor 1-alpha 1 (Fragment)
2893	dirMS_041712_NAR_120mM_minus.14478.14623.3	3	15.09	1	1.99E+06		(R)QLREYQ	(R)Q L/R E Y\Q/E/L/M N/V/K(L)		133.6	517.6038	1550.8	-0.0027	-1.7	6.14	56607.4	5.37	HUMAN	F8VXB4	Keratin, type II cytoskeletal 8
2894	dirMS_041712_NAR_30mM_minus2.9905.9928.2	2	15.09	0	8.17E+04		(R)LEDAAD	(R)L/E|D|A|A D/V/Y/R(G)		108.18	526.2618	1051.506	0.0109	10.3	4.03	102525.8	7.33	HUMAN	Q9BXJ9	N-alpha-acetyltransferase 15, NatA auxiliary subunit
2895	dirMS_041712_NAR_40mM_minus2.10357.10750.3	3	15.09	1	3.05E+06		(K)TITLEVEI	(K)T I|T|L/E/V/E/P S D T I E N V K A K(I)		145.98	663.0226	1987.059	-0.0061	-3.1	4.41	17271.6	6.75	HUMAN	B4DV12	Ubiquitin
2896	dirMS_041712_NAR_50mM_minus2.10744.10744.3	3	15.09	0	2.68E+05		(K)VFEGNR	(K)V/F/E/G N R P T N S I\V/F/T K(L)		129.25	570.3032	1708.902	-0.0066	-3.9	8.72	63374.2	8.64	HUMAN	P06744	Glucose-6-phosphate isomerase
2897	dirMS_041712_NAR_30mM_plus2.12002.12002.2	2	15.09	0	1.59E+05		(K)GYDVIA(	(K)G Y D\V|I|A|Q/A Q/S G T G K(T)		115.98	697.848	1394.691	-0.0023	-1.7	5.83	16071.1	4.8	HUMAN	B4E102	Eukaryotic initiation factor 4A-I
2898	dirMS_041712_NAR_30mM_plus2.19060.19060.2	2	15.09	0	1.50E+05		(R)VYSLFLD	(R)V/Y/S L/F/L D/E S R(S)		153.13	614.8154	1228.621	0.0027	2.2	4.37	46476.4	5.33	HUMAN	B3KQ59	RuvB-like 2
2899	dirMS_041712_NAR_30mM_plus2.8775.8934.2	2	15.09	0	2.43E+06		(R)VIGSGCI	(R)V/I|G/S/G C/N L/D S A/R(F)	C:Carbamidomethylation	99.68	624.802	1248.6	-0.0034	-2.7	5.81	25445.8	5.52	HUMAN	A8MW50	L-lactate dehydrogenase (Fragment)
2900	dirMS_041712_NAR_50mM_plus2.13203.13203.3	3	15.09	1	9.66E+05		(R)ELNITAA	(R)E L/N I/T A A K E\I/E|V/G G/G R(K)		137.7	552.9671	1656.892	-0.0048	-2.9	4.79	21311.5	10.28	HUMAN	B5MCP9	40S ribosomal protein S7
2901	dirMS_041712_NAR_50mM_plus2.17798.17852.3	3	15.09	0	4.45E+05	M64m	(R)GFGFITF	(R)G F|G\F|I|T/F T N/P E H A S V/A m R(A)	m:Oxidized methionine	161.67	666.658	1981.959	16.0006	2.8	6.75	17170.1	8.86	HUMAN	P98179	Putative RNA-binding protein 3
2902	dirMS_041712_NAR_60mM_plus.9406.9447.3	3	15.09	1	1.94E+06		(K)VVWVEI	(K)V/V|W/V/E/E S D/K R(S)		107.43	416.221	1246.643	0.0058	4.7	4.68	73641.5	6.74	HUMAN	O00571	ATP-dependent RNA helicase DDX3X
2903	dirMS_041712_NAR_60mM_minus.12256.12282.3	3	15.08	1	7.93E+05		(K)AVIFCLS.	(K)A V|I/F/C/L/S A D/K K(C)	C:Carbamidomethylation	127.92	417.9007	1251.677	0.011	8.8	8.64	15739.2	9.3	HUMAN	F6RFD5	Destrin
2904	dirMS_041712_NAR_60mM_minus.12541.12952.3	3	15.08	1	1.26E+06		(K)AVLFCLS	(K)A/V|L/F/C/L/S E D K/K(N)	C:Carbamidomethylation	131.25	437.2334	1309.682	0.0036	2.7	6.11	22956	8.8	HUMAN	E9PK25	Cofilin-1
2905	dirMS_041712_NAR_50mM_plus2.14794.14934.3	3	15.08	0	8.03E+05		(R)TIQVEN:	(R)T I|Q|V/E N\S H L/I/L/T/G A/G A L N K(V)		146.07	660.3668	1979.092	-0.0062	-3.1	6.41	267260.7	5.95	HUMAN	Q13085	Acetyl-CoA carboxylase 1
2906	dirMS_041712_NAR_60mM_plus.11245.11246.3	3	15.08	1	3.12E+05		(R)AGTGVD	(R)A/G/T|G/V/D N V D L E/A/A T R K(G)		116.3	539.6102	1616.824	-0.0078	-4.8	4.56	57391.2	6.29	HUMAN	O43175	D-3-phosphoglycerate dehydrogenase
2907	dirMS_041712_NAR_60mM_plus.6488.6488.3	3	15.08	1	2.39E+05		(R)LTQEPES	(R)L/T|Q/E/P E S I R K(W)		92.45	400.8875	1200.658	-0.0103	-8.6	6.14	8409.9	4.52	HUMAN	H0Y9Q6	Clathrin light chain B (Fragment)
2908	dirMS_041712_NAR_20mM_minus.7205.7205.2	2	15.07	0	1.49E+04	M33m	(R)IDEmPE/	(R)I/D|E m/P E/A A V K(S)	m:Oxidized methionine	120.27	559.7733	1102.545	15.9944	-0.4	4.14	26901.9	4.7	HUMAN	P51858	Hepatoma-derived growth factor
2909	dirMS_041712_NAR_30mM_minus2.19507.19580.2	2	15.07	0	7.67E+06		(R)YLTVAA'	(R)Y/L|T|V/A/A V/F R(G)		161.2	520.2998	1039.593	-0.0012	-1.1	8.75	40069.4	4.83	HUMAN	E7EWR1	Tubulin beta chain
2910	dirMS_041712_NAR_40mM_minus2.13736.13826.3	3	15.07	1	3.98E+05		(R)VTFSGLI	(R)V T|F/S G L/R D T|V/T/P G E/L L/K(A)		164.73	611.6742	1833.012	-0.0036	-2	6.04	266161	5.68	HUMAN	E9PPU0	Epiplakin
2911	dirMS_041712_NAR_50mM_minus2.10986.11110.3	3	15.07	1	5.59E+05		(K)QQEAAL	(K)Q\Q/E/A/A\L/E/L/E E L\K K(K)		131.1	510.2775	1528.822	-0.0038	-2.5	4.49	54214.9	5.66	HUMAN	C9J813	Caldesmon (Fragment)
2912	dirMS_041712_NAR_50mM_minus2.11061.11258.3	3	15.07	0	1.44E+06		(R)NLDIERI	(R)N/L|D|I/E R/P T Y/T/N L N R(L)		131.95	573.6313	1718.882	-0.0027	-1.6	6.07	49012.3	4.9	HUMAN	A8MUB1	Tubulin alpha-4A chain
2913	dirMS_041712_NAR_50mM_minus2.16892.16892.3	3	15.07	1	2.84E+05		(R)SELELTL(	(R)S/E|L/E/L/T L G K L E/Q V R(S)		163.78	538.9725	1614.906	-0.0032	-2	4.78	533778	5.74	HUMAN	Q15149	Plectin
2914	dirMS_041712_NAR_50mM_minus2.8695.8695.3	3	15.07	0	1.65E+05	M180m	(R)VVVLmC	(R)V/V|V|L m/G S/T S D/L G H C E K(I)	C:Carbamidomethylation m:Oxid	117.55	583.2829	1731.84	15.9937	-0.7	5.32	37681.5	5.93	HUMAN	D6RF62	Phosphoribosylaminoimidazole carboxylase
2915	dirMS_041712_NAR_30mM_plus2.11535.11535.2	2	15.07	0	5.15E+05		(R)SGEGEV	(R)S/G\E|G/E/V S G L\M/R(K)		114.07	561.2662	1121.526	-0.0004	-0.4	4.53	90316.8	5.52	HUMAN	Q13263	Transcription intermediary factor 1-beta
2916	dirMS_041712_NAR_30mM_plus2.14632.15016.2	2	15.07	0	3.35E+07		(K)WSLLQC	(K)W/S|L/L/Q/Q/Q/K(T)		130.53	515.7855	1030.568	-0.0043	-4.2	8.75	13588.1	4.66	HUMAN	F8VP67	Keratin, type II cytoskeletal 8 (Fragment)
2917	dirMS_041712_NAR_50mM_plus2.13232.13232.3	3	15.07	0	2.15E+06		(R)SCTVNII	(R)S C|T/V/N/I/M/F/G T/P Q E/H R(N)	C:Carbamidomethylation	138.4	592.9438	1776.816	0.0012	0.7	6.47	65763.5	9.81	HUMAN	Q9HCD5	Nuclear receptor coactivator 5
2918	dirMS_041712_NAR_60mM_plus.14463.14463.3	3	15.07	0	3.18E+06		(R)DLNYCF:	(R)D L/N|Y/C/F/S G M S D H/R(Y)	C:Carbamidomethylation	132.38	534.5555	1601.647	0.0048	3	5.21	47371.6	5.93	HUMAN	E9PCY7	Heterogeneous nuclear ribonucleoprotein H
2919	dirMS_041712_NAR_60mM_plus.9853.9879.3	3	15.07	1	2.90E+05		(K)SEEQLKI	(K)S/E|E/Q/L/K E E/G I E/Y K(V)		109.28	527.9264	1581.764	0.0004	0.2	4.32	43928.7	6.58	HUMAN	B4DHG0	Dihydrolipoyl dehydrogenase, mitochondrial
2920	dirMS_041712_NAR_120mM_minus.7467.7591.3	3	15.06	1	1.42E+06		(R)RQLETL(	(R)R Q\L E|T|L/G Q/E/K(L)		95.2	401.2208	1201.653	-0.0057	-4.7	6.14	13588.1	4.66	HUMAN	F8VP67	Keratin, type II cytoskeletal 8 (Fragment)
2921	dirMS_041712_NAR_30mM_minus2.15769.15769.2	2	15.06	0	1.23E+05		(R)TINEVEN	(R)T I/N|E/V/E/N Q/I/L T R(D)		139.58	715.3834	1429.765	-0.005	-3.5	4.53	107710.1	5.38	HUMAN	B7TY16	Actinin alpha 1 isoform 3
2922	dirMS_041712_NAR_50mM_minus2.15063.15063.3	3	15.06	0	4.19E+05		(K)IAILTCPI	(K)I A|I L T/C|P/F/E/P/P K P K(T)	C:Carbamidomethylation	153.57	537.6375	1610.898	0.0005	0.3	8.59	49982	5.86	HUMAN	B4DYD8	T-complex protein 1 subunit epsilon
2923	dirMS_041712_NAR_20mM_plus.18567.18611.3	3	15.06	0	1.88E+04		(R)APVPAS	(R)A P\V\P\A S E\L\L|A|S/G/V/L/S R(A)		199.92	522.9655	1566.885	-0.003	-1.9	6.05	533778	5.74	HUMAN	Q15149	Plectin
2924	dirMS_041712_NAR_20mM_plus.8406.8406.2	2	15.06	0	1.04E+05	M607m M(	(R)mGLAm	(R)m G\L/A m|G/G G G\G A S F/D R(A)	m:Oxidized methionine	128.17	708.3061	1383.614	31.9905	0.5	5.59	77799.7	9.09	HUMAN	P52272	Heterogeneous nuclear ribonucleoprotein M
2925	dirMS_041712_NAR_30mM_plus2.20116.20444.3	3	15.06	0	5.11E+05		(K)SYELPD(	(K)S Y\E|L/P D G Q|V|I/T I/G N E R(F)		159.87	597.6334	1790.892	-0.0063	-3.5	4.14	123097.1	5.83	HUMAN	A5A3E0	POTE ankyrin domain family member F
2926	dirMS_041712_NAR_30mM_plus2.5794.5794.2	2	15.06	0	2.62E+05		(K)TSEVNC'	(K)T|S|E|V/N C/Y/R(C)	C:Carbamidomethylation	85.08	514.7261	1028.447	-0.0016	-1.6	5.66	18946.7	9.13	HUMAN	B4DP17	Cellular nucleic acid-binding protein
2927	dirMS_041712_NAR_30mM_plus2.8660.8660.2	2	15.06	1	2.10E+05		(R)GVEEEEI	(R)G V/E/E/E\E/E D/G/E M/R E(-)		98.92	769.301	1537.596	-0.0011	-0.7	3.88	9782.1	4.7	HUMAN	P62306	Small nuclear ribonucleoprotein F
2928	dirMS_041712_NAR_50mM_plus2.20077.20077.3	3	15.06	1	1.08E+05		(K)SVISGGL	(K)S V|I/S G G L/D/A L E F I G K K(T)		174.18	545.3096	1633.916	-0.0017	-1	5.79	22133.2	4.82	HUMAN	E5RGF9	Protein FAM114A2 (Fragment)
2929	dirMS_041712_NAR_50mM_plus2.7205.7205.3	3	15.06	1	1.94E+05		(R)DSFDGR	(R)D S|F/D/G R/G P/P G\P/E S Q S R(A)		103.85	563.592	1688.762	-0.0009	-0.5	4.56	13246.4	9.21	HUMAN	Q5JY17	Nuclear receptor coactivator 5 (Fragment)
2930	dirMS_041712_NAR_50mM_plus2.9830.9830.3	3	15.06	1	2.54E+05		(K)TTDMAI	(K)T/T/D M/A/P S K/E T E M A L A K(D)		119.22	575.2802	1723.824	0.0019	1.1	4.68	85679.3	7.65	HUMAN	B9ZVR1	Microtubule-associated protein
2931	dirMS_041712_NAR_60mM_plus.13065.13585.3	3	15.06	1	1.02E+06		(R)ISVYYNE	(R)I/S|V|Y/Y N/E A/T/G G K Y V/P R(A)		125.95	606.3113	1816.923	-0.0035	-1.9	8.43	27549.9	4.39	HUMAN	E9PBJ4	Tubulin beta chain
2932	dirMS_041712_NAR_120mM_minus.8757.8757.3	3	15.05	0	5.76E+05		(R)THYSNIE	(R)T\H Y S N\I|E|A|N E/S/E E V/R(Q)		102.13	593.2723	1777.799	0.0036	2	4.48	21983.3	4.68	HUMAN	K7EIV0	Calpain small subunit 1
2933	dirMS_041712_NAR_30mM_minus2.17549.17549.2	2	15.05	0	4.37E+05		(R)EQLAIAE	(R)E Q|L/A/I/A E F A\R(S)		150.25	574.3084	1147.611	-0.0011	-0.9	4.53	44227.3	7.81	HUMAN	E7EQR6	T-complex protein 1 subunit alpha
2934	dirMS_041712_NAR_30mM_minus2.8132.8525.2	2	15.05	0	3.66E+06		(R)GTLDPV	(R)G T|L/D/P V/E/K(A)		100.18	429.7348	858.457	0.0056	6.5	4.37	45508.6	5.5	HUMAN	A8K7Q2	Heat shock cognate 71 kDa protein
2935	dirMS_041712_NAR_120mM_plus.6452.6635.3	3	15.05	1	1.74E+07		(R)TKTEISEI	(R)T\K\T E\I|S/E/M/N/R(N)		88.12	403.5365	1208.594	0.001	0.8	5.81	37296.3	5.3	HUMAN	F8VU64	Keratin, type II cytoskeletal 8
2936	dirMS_041712_NAR_20mM_plus.6994.7014.2	2	15.05	0	2.19E+05		(R)LLEAAA(	(R)L/L|E|A|A/A Q/S T K(G)		114.93	516.2866	1031.573	-0.0072	-7	6	533778	5.74	HUMAN	Q15149	Plectin
2937	dirMS_041712_NAR_40mM_plus2.17486.17486.3	3	15.05	1	3.17E+05		(K)TGISDVI	(K)T G I/S D\V/F A K/N D/L/A\V/V D/V R(I)		169.33	640.3451	1919.023	-0.0025	-1.3	4.43	76670.1	4.6	HUMAN	P19338	Nucleolin
2938	dirMS_041712_NAR_60mM_plus.12037.12098.3	3	15.05	1	7.57E+05		(R)ARQEEL'	(R)A R Q\E E\L|Y|S/E/L/Q/A R(E)		120.1	531.6013	1592.803	-0.0134	-8.4	4.79	533778	5.74	HUMAN	Q15149	Plectin

<table>
<tr><td>2939</td><td>dirMS_041712_NAR_40mM_minus2.11002.11021.3</td><td>3</td><td>15.04</td><td>0</td><td>4.86E+05</td><td></td><td>(R)DAGEGL</td><td>(R)D A|G|E G/L/L A/V Q I/T D Q E/G\K\P K(R)</td><td></td><td>148.02</td><td>657.0157</td><td>1969.024</td><td>0.0089</td><td>4.5</td><td>4.32</td><td>258215.9</td><td>5.48</td><td>HUMAN</td><td>E7EN95</td><td>Filamin-B</td></tr>
<tr><td>2940</td><td>dirMS_041712_NAR_50mM_minus2.18368.18368.3</td><td>3</td><td>15.04</td><td>0</td><td>4.62E+05</td><td></td><td>(K)ALIEVLQ</td><td>(K)A L\/E/V/L Q/P L I A/E H Q A R(R)</td><td></td><td>175.42</td><td>601.0139</td><td>1801.033</td><td>-0.0059</td><td>-3.3</td><td>5.4</td><td>53506.8</td><td>5.83</td><td>HUMAN</td><td>P23381</td><td>Tryptophan--tRNA ligase, cytoplasmic</td></tr>
<tr><td>2941</td><td>dirMS_041712_NAR_50mM_minus2.9195.9195.3</td><td>3</td><td>15.04</td><td>0</td><td>3.48E+05</td><td></td><td>(R)TIVITSHI</td><td>(R)T I|V|I/T S H/P G Q I V K(H)</td><td></td><td>120.98</td><td>464.9452</td><td>1392.821</td><td>0.0001</td><td>0.1</td><td>8.44</td><td>27999.4</td><td>5.61</td><td>HUMAN</td><td>B7Z5C0</td><td>DnaJ homolog subfamily A member 1</td></tr>
<tr><td>2942</td><td>dirMS_041712_NAR_20mM_plus.13905.13905.2</td><td>2</td><td>15.04</td><td>0</td><td>2.12E+05</td><td>M98m</td><td>(R)TLTIVDT</td><td>(R)T L|T/I/V/D\T G I G/m/T K(A)</td><td>m:Oxidized methionine</td><td>167.5</td><td>683.3671</td><td>1349.735</td><td>15.9924</td><td>-1.8</td><td>5.5</td><td>20151.2</td><td>4.58</td><td>HUMAN</td><td>G3V2J8</td><td>Heat shock protein HSP 90-alpha (Fragment)</td></tr>
<tr><td>2943</td><td>dirMS_041712_NAR_50mM_plus2.14393.14574.3</td><td>3</td><td>15.04</td><td>1</td><td>1.20E+06</td><td></td><td>(K)ITDIIGKE</td><td>(K)I/T|D\|I/G K E E G\/G/P E N L R(G)</td><td></td><td>144.28</td><td>618.6652</td><td>1853.997</td><td>-0.0157</td><td>-8.5</td><td>4.41</td><td>267260.7</td><td>5.95</td><td>HUMAN</td><td>Q13085</td><td>Acetyl-CoA carboxylase 1</td></tr>
<tr><td>2944</td><td>dirMS_041712_NAR_30mM_minus2.12953.13079.2</td><td>2</td><td>15.03</td><td>0</td><td>2.84E+05</td><td></td><td>(K)LSELEPG</td><td>(K)L S E|L|E|P/G T G D/L R(F)</td><td></td><td>124.53</td><td>643.8325</td><td>1286.659</td><td>-0.0009</td><td>-0.7</td><td>4.14</td><td>266161</td><td>5.68</td><td>HUMAN</td><td>E9PPU0</td><td>Epiplakin</td></tr>
<tr><td>2945</td><td>dirMS_041712_NAR_60mM_minus.7674.7674.3</td><td>3</td><td>15.03</td><td>1</td><td>6.66E+05</td><td></td><td>(K)IVADKD</td><td>(K)I/V/A D K D|Y/S/V/T A N S K(I)</td><td></td><td>102.58</td><td>504.2624</td><td>1510.775</td><td>-0.0021</td><td>-1.4</td><td>5.96</td><td>25445.8</td><td>5.52</td><td>HUMAN</td><td>A8MW50</td><td>L-lactate dehydrogenase (Fragment)</td></tr>
<tr><td>2946</td><td>dirMS_041712_NAR_20mM_plus.12632.12632.2</td><td>2</td><td>15.03</td><td>0</td><td>9.34E+04</td><td>M91m</td><td>(R)AITGASL</td><td>(R)A I|T|G/A/S|L A D I m A K(R)</td><td>m:Oxidized methionine</td><td>159.93</td><td>639.3398</td><td>1261.682</td><td>15.9903</td><td>-3.6</td><td>5.88</td><td>17656.4</td><td>11.01</td><td>HUMAN</td><td>C9JNW5</td><td>60S ribosomal protein L24</td></tr>
<tr><td>2947</td><td>dirMS_041712_NAR_20mM_plus.13538.13538.2</td><td>2</td><td>15.03</td><td>0</td><td>1.60E+05</td><td>M98m</td><td>(R)TLTIVDT</td><td>(R)T L|T\/V/D T G I/G\m/T K(A)</td><td>m:Oxidized methionine</td><td>165.8</td><td>683.3671</td><td>1349.735</td><td>15.9924</td><td>-1.8</td><td>5.5</td><td>20151.2</td><td>4.58</td><td>HUMAN</td><td>G3V2J8</td><td>Heat shock protein HSP 90-alpha (Fragment)</td></tr>
<tr><td>2948</td><td>dirMS_041712_NAR_50mM_plus2.19863.19863.3</td><td>3</td><td>15.03</td><td>0</td><td>2.50E+05</td><td></td><td>(K)LFLFHTS</td><td>(K)L/F|L F H T S\L|P/I/A E|A/P G K(L)</td><td></td><td>172.83</td><td>580.9943</td><td>1740.968</td><td>0</td><td>0</td><td>6.75</td><td>108105.4</td><td>6.57</td><td>HUMAN</td><td>E7EP00</td><td>Protein transport protein Sec24C</td></tr>
<tr><td>2949</td><td>dirMS_041712_NAR_120mM_minus.9163.9163.2</td><td>2</td><td>15.02</td><td>0</td><td>4.74E+05</td><td></td><td>(K)HFVALS</td><td>(K)H/F|V|A\L/S T N T/T K(V)</td><td></td><td>103.53</td><td>609.8267</td><td>1218.648</td><td>-0.0016</td><td>-1.3</td><td>8.76</td><td>63374.2</td><td>8.64</td><td>HUMAN</td><td>P06744</td><td>Glucose-6-phosphate isomerase</td></tr>
<tr><td>2950</td><td>dirMS_041712_NAR_30mM_minus2.12950.12950.2</td><td>2</td><td>15.02</td><td>0</td><td>2.77E+04</td><td></td><td>(K)LYLPVQ</td><td>(K)L Y|L|P V Q N K(A)</td><td></td><td>124.5</td><td>487.788</td><td>974.567</td><td>0.0018</td><td>1.8</td><td>8.59</td><td>22945.2</td><td>9.96</td><td>HUMAN</td><td>Q9Y6A4</td><td>UPF0468 protein C16orf80</td></tr>
<tr><td>2951</td><td>dirMS_041712_NAR_50mM_minus2.10818.10818.3</td><td>3</td><td>15.02</td><td>1</td><td>6.04E+05</td><td></td><td>(R)IAEFTTN</td><td>(R)I/A|E\F T T N L/T/E E E E K\S K(S)</td><td></td><td>130.4</td><td>623.6418</td><td>1868.912</td><td>-0.0015</td><td>-0.8</td><td>4.32</td><td>227783.5</td><td>5.5</td><td>HUMAN</td><td>P35579</td><td>Myosin-9</td></tr>
<tr><td>2952</td><td>dirMS_041712_NAR_50mM_minus2.13676.13676.3</td><td>3</td><td>15.02</td><td>0</td><td>7.43E+04</td><td></td><td>(K)MLDAEL</td><td>(K)M\L/D/A E/D I V/N T A R/P D E/K(A)</td><td></td><td>146.2</td><td>606.2965</td><td>1816.875</td><td>0.0004</td><td>0.2</td><td>4.1</td><td>105308.6</td><td>5.27</td><td>HUMAN</td><td>O43707</td><td>Alpha-actinin-4</td></tr>
<tr><td>2953</td><td>dirMS_041712_NAR_50mM_minus2.15992.15992.3</td><td>3</td><td>15.02</td><td>0</td><td>2.36E+05</td><td></td><td>(K)AQDGSI</td><td>(K)A/Q/D G S H P L/S/L Q D/L I E K(V)</td><td></td><td>159.08</td><td>584.3049</td><td>1750.897</td><td>0.0031</td><td>1.8</td><td>4.54</td><td>128523.4</td><td>7.06</td><td>HUMAN</td><td>B7ZL00</td><td>Protein transport protein Sec31A</td></tr>
<tr><td>2954</td><td>dirMS_041712_NAR_60mM_minus.17913.18151.3</td><td>3</td><td>15.02</td><td>0</td><td>1.33E+06</td><td></td><td>(K)VSHLLGI</td><td>(K)V S H\L\L\G\I|N|V|T|D/F T R(G)</td><td></td><td>161.62</td><td>524.623</td><td>1571.854</td><td>0.0004</td><td>0.3</td><td>6.71</td><td>227783.5</td><td>5.5</td><td>HUMAN</td><td>P35579</td><td>Myosin-9</td></tr>
<tr><td>2955</td><td>dirMS_041712_NAR_20mM_plus.12513.12513.2</td><td>2</td><td>15.02</td><td>0</td><td>1.19E+05</td><td></td><td>(K)IISNASC</td><td>(K)I I/S/N|A S C T T/N C L A|P L A K(V)</td><td>C:Carbamidomethylation</td><td>159.55</td><td>917.4626</td><td>1833.92</td><td>-0.0018</td><td>-1</td><td>8.75</td><td>31718.5</td><td>7.17</td><td>HUMAN</td><td>E7EUT4</td><td>Glyceraldehyde-3-phosphate dehydrogenase</td></tr>
<tr><td>2956</td><td>dirMS_041712_NAR_20mM_plus.13704.13764.2</td><td>2</td><td>15.02</td><td>0</td><td>2.08E+05</td><td></td><td>(K)APILIAT</td><td>(K)A P|I|L|I|A/T D/V A S R(G)</td><td></td><td>166.65</td><td>613.8559</td><td>1226.71</td><td>-0.0058</td><td>-4.7</td><td>5.88</td><td>61018.6</td><td>9.44</td><td>HUMAN</td><td>B4DLW8</td><td>Probable ATP-dependent RNA helicase DDX5</td></tr>
<tr><td>2957</td><td>dirMS_041712_NAR_20mM_plus.18421.18555.2</td><td>2</td><td>15.02</td><td>0</td><td>1.78E+05</td><td></td><td>(R)NIPGITL</td><td>(R)N/I|P/G/I/T L L/N V/S K(L)</td><td></td><td>198.83</td><td>634.8801</td><td>1268.757</td><td>-0.0043</td><td>-3.4</td><td>8.75</td><td>37884.5</td><td>11.27</td><td>HUMAN</td><td>H3BM89</td><td>60S ribosomal protein L4</td></tr>
<tr><td>2958</td><td>dirMS_041712_NAR_30mM_plus2.10588.10588.2</td><td>2</td><td>15.02</td><td>0</td><td>3.47E+05</td><td></td><td>(K)VSYVQLI</td><td>(K)V/S|Y/V Q L/K(E)</td><td></td><td>108.8</td><td>418.7442</td><td>836.488</td><td>-0.0065</td><td>-7.8</td><td>8.56</td><td>334221.3</td><td>6.44</td><td>HUMAN</td><td>P15924</td><td>Desmoplakin</td></tr>
<tr><td>2959</td><td>dirMS_041712_NAR_30mM_plus2.13359.13359.2</td><td>2</td><td>15.02</td><td>0</td><td>5.70E+05</td><td></td><td>(K)CPFTGN</td><td>(K)C/P|F|T/G N V/S I/R(G)</td><td>C:Carbamidomethylation</td><td>122.88</td><td>575.7876</td><td>1150.567</td><td>0.0006</td><td>0.5</td><td>9.75</td><td>18601.6</td><td>10.52</td><td>HUMAN</td><td>P62280</td><td>40S ribosomal protein S11</td></tr>
<tr><td>2960</td><td>dirMS_041712_NAR_30mM_plus2.22647.22659.3</td><td>3</td><td>15.02</td><td>0</td><td>5.00E+05</td><td></td><td>(R)ALTVPEI</td><td>(R)A L\T V/P E\L/T/Q Q V/F/D/A K(N)</td><td></td><td>175.77</td><td>553.9697</td><td>1659.895</td><td>-0.0007</td><td>-0.4</td><td>4.37</td><td>40069.4</td><td>4.83</td><td>HUMAN</td><td>E7EWR1</td><td>Tubulin beta chain</td></tr>
<tr><td>2961</td><td>dirMS_041712_NAR_30mM_plus2.7539.7559.2</td><td>2</td><td>15.02</td><td>0</td><td>8.08E+05</td><td></td><td>(R)LSVAAQ</td><td>(R)L/S|V/A|A/Q/E/A A/R(L)</td><td></td><td>93.83</td><td>508.2801</td><td>1015.553</td><td>-0.0002</td><td>-0.2</td><td>6</td><td>533778</td><td>5.74</td><td>HUMAN</td><td>Q15149</td><td>Plectin</td></tr>
<tr><td>2962</td><td>dirMS_041712_NAR_120mM_minus.14084.14374.3</td><td>3</td><td>15.01</td><td>1</td><td>1.40E+06</td><td></td><td>(R)RLEAAYI</td><td>(R)R L E\A\A|Y|L/D|L/Q/R(I)</td><td></td><td>132.12</td><td>449.9126</td><td>1347.738</td><td>-0.0147</td><td>-10.9</td><td>6.07</td><td>15962.5</td><td>9.61</td><td>HUMAN</td><td>B4DT30</td><td>Tubulin-specific chaperone A</td></tr>
<tr><td>2963</td><td>dirMS_041712_NAR_20mM_minus.9696.9716.2</td><td>2</td><td>15.01</td><td>0</td><td>3.36E+05</td><td></td><td>(K)IGGIGTV</td><td>(K)I/G|G|I|G|T/V/P V G R(V)</td><td></td><td>147.47</td><td>513.3075</td><td>1025.61</td><td>-0.0025</td><td>-2.4</td><td>9.75</td><td>50482.3</td><td>9.31</td><td>HUMAN</td><td>P68104</td><td>Elongation factor 1-alpha 1</td></tr>
<tr><td>2964</td><td>dirMS_041712_NAR_30mM_minus2.15572.15572.2</td><td>2</td><td>15.01</td><td>0</td><td>1.45E+05</td><td></td><td>(K)AMTGVI</td><td>(K)A M/T/G/V/E Q/W/P Y/R(A)</td><td></td><td>138.32</td><td>669.3172</td><td>1337.631</td><td>-0.0036</td><td>-2.7</td><td>6.04</td><td>56186.7</td><td>5.47</td><td>HUMAN</td><td>B4DUR8</td><td>T-complex protein 1 subunit gamma</td></tr>
<tr><td>2965</td><td>dirMS_041712_NAR_30mM_minus2.19359.19505.2</td><td>2</td><td>15.01</td><td>0</td><td>2.42E+05</td><td></td><td>(R)FEELNAI</td><td>(R)F/E|E|L|N A/D/L/F/R(G)</td><td></td><td>160.88</td><td>627.3141</td><td>1253.616</td><td>0.0048</td><td>3.9</td><td>4.14</td><td>45508.6</td><td>5.5</td><td>HUMAN</td><td>A8K7Q2</td><td>Heat shock cognate 71 kDa protein</td></tr>
<tr><td>2966</td><td>dirMS_041712_NAR_40mM_minus2.12399.12399.3</td><td>3</td><td>15.01</td><td>0</td><td>1.70E+05</td><td></td><td>(R)GAPPSSI</td><td>(R)G A/P/P S S N I/E D/F H G L L/P K(G)</td><td></td><td>155.92</td><td>593.6401</td><td>1778.907</td><td>-0.0014</td><td>-0.8</td><td>5.32</td><td>100478.9</td><td>5.71</td><td>HUMAN</td><td>A8MXP9</td><td>Matrin-3</td></tr>
<tr><td>2967</td><td>dirMS_041712_NAR_40mM_minus2.9212.9234.3</td><td>3</td><td>15.01</td><td>0</td><td>2.52E+05</td><td>M240m</td><td>(K)mLDAEI</td><td>(K)m L|D|A/E/D/I/V/N T A R P D/E K(A)</td><td>m:Oxidized methionine</td><td>136.73</td><td>611.627</td><td>1816.875</td><td>15.9919</td><td>-1.6</td><td>4.1</td><td>105308.6</td><td>5.27</td><td>HUMAN</td><td>O43707</td><td>Alpha-actinin-4</td></tr>
<tr><td>2968</td><td>dirMS_041712_NAR_50mM_minus2.11664.11664.3</td><td>3</td><td>15.01</td><td>0</td><td>1.12E+05</td><td></td><td>(R)VTCPNH</td><td>(R)V/T/C/P N H|P D A\I L V/E D Y R(A)</td><td>C:Carbamidomethylation</td><td>134.77</td><td>633.641</td><td>1898.907</td><td>0.0019</td><td>1</td><td>4.54</td><td>25558.6</td><td>9.3</td><td>HUMAN</td><td>B1APE1</td><td>General transcription factor IIB (Fragment)</td></tr>
<tr><td>2969</td><td>dirMS_041712_NAR_50mM_minus2.14082.14082.3</td><td>3</td><td>15.01</td><td>0</td><td>3.40E+05</td><td></td><td>(K)SPAPPLL</td><td>(K)S/P/A|P/P L L H/V A A L/G Q K(Q)</td><td></td><td>148.45</td><td>500.2911</td><td>1498.874</td><td>-0.0153</td><td>-10.2</td><td>8.49</td><td>17549.1</td><td>10.39</td><td>HUMAN</td><td>E5RFJ3</td><td>DBIRD complex subunit KIAA1967 (Fragment)</td></tr>
<tr><td>2970</td><td>dirMS_041712_NAR_50mM_minus2.8922.8924.3</td><td>3</td><td>15.01</td><td>1</td><td>1.31E+05</td><td>M227m</td><td>(R)mQKEIT</td><td>(R)m Q K E I\T|A|L|A|P S T M/K(I)</td><td>m:Oxidized methionine</td><td>119.48</td><td>522.2727</td><td>1548.812</td><td>15.9911</td><td>-2.4</td><td>8.35</td><td>32616.5</td><td>5.18</td><td>HUMAN</td><td>A6NL76</td><td>Actin, alpha skeletal muscle</td></tr>
<tr><td>2971</td><td>dirMS_041712_NAR_120mM_plus.4103.4203.3</td><td>3</td><td>15.01</td><td>1</td><td>2.05E+06</td><td>M54m</td><td>(K)SLDEmE</td><td>(K)S/L|D/E/m/E/K Q/Q/R(E)</td><td>m:Oxidized methionine</td><td>70.2</td><td>427.2032</td><td>1263.6</td><td>15.9953</td><td>0.3</td><td>4.68</td><td>26419.2</td><td>5.1</td><td>HUMAN</td><td>H0Y9K7</td><td>Splicing factor, proline- and glutamine-rich (Fragment)</td></tr>
<tr><td>2972</td><td>dirMS_041712_NAR_20mM_plus.13157.13258.2</td><td>2</td><td>15.01</td><td>0</td><td>6.67E+05</td><td></td><td>(R)VLQALE</td><td>(R)V/L|Q|A/L E/G L/K(M)</td><td></td><td>164.38</td><td>485.8004</td><td>970.593</td><td>0.0004</td><td>0.4</td><td>5.97</td><td>16060.2</td><td>10.31</td><td>HUMAN</td><td>P39019</td><td>40S ribosomal protein S19</td></tr>
<tr><td>2973</td><td>dirMS_041712_NAR_20mM_plus.18875.18875.2</td><td>2</td><td>15.01</td><td>0</td><td>2.32E+05</td><td></td><td>(R)IITLAGP</td><td>(R)I I|T L/A|G/P T N A I F K(A)</td><td></td><td>200.97</td><td>679.9039</td><td>1358.804</td><td>-0.0037</td><td>-2.7</td><td>8.75</td><td>32621</td><td>8.75</td><td>HUMAN</td><td>B4DLC0</td><td>Poly(rC)-binding protein 2</td></tr>
<tr><td>2974</td><td>dirMS_041712_NAR_30mM_plus2.17947.18108.2</td><td>2</td><td>15.01</td><td>0</td><td>3.00E+05</td><td></td><td>(R)VNPALA</td><td>(R)V N|P/A/L|A/E L/N L R(S)</td><td></td><td>147.82</td><td>605.3509</td><td>1209.695</td><td>-0.0005</td><td>-0.4</td><td>5.97</td><td>20026.6</td><td>4.67</td><td>HUMAN</td><td>E9PIK5</td><td>Ribonuclease inhibitor (Fragment)</td></tr>
<tr><td>2975</td><td>dirMS_041712_NAR_40mM_plus2.18159.18409.3</td><td>3</td><td>15.01</td><td>1</td><td>1.59E+06</td><td></td><td>(R)GDLGIEI</td><td>(R)G D L|G|I\E/I/P A E K V F L A Q K(M)</td><td></td><td>173.97</td><td>610.0115</td><td>1828.022</td><td>-0.0015</td><td>-0.8</td><td>4.68</td><td>18466.1</td><td>5.78</td><td>HUMAN</td><td>H3BSU3</td><td>Pyruvate kinase (Fragment)</td></tr>
<tr><td>2976</td><td>dirMS_041712_NAR_50mM_plus2.13117.13117.3</td><td>3</td><td>15.01</td><td>0</td><td>7.93E+05</td><td></td><td>(R)VLHVSEI</td><td>(R)V L H V S E N\P\V|P/L T V R(V)</td><td></td><td>137.38</td><td>520.6339</td><td>1559.89</td><td>-0.0033</td><td>-2.1</td><td>6.72</td><td>64042.3</td><td>9.02</td><td>HUMAN</td><td>Q8N1G4</td><td>Leucine-rich repeat-containing protein 47</td></tr>
<tr><td>2977</td><td>dirMS_041712_NAR_50mM_plus2.9307.9307.3</td><td>3</td><td>15.01</td><td>0</td><td>1.33E+05</td><td></td><td>(K)AEITLVA</td><td>(K)A E|I\T L/V/A T K/P E K(L)</td><td></td><td>116.23</td><td>433.922</td><td>1299.752</td><td>-0.0004</td><td>-0.3</td><td>6.18</td><td>98761.7</td><td>5.56</td><td>HUMAN</td><td>Q9UBF2</td><td>Coatomer subunit gamma-2</td></tr>
<tr><td>2978</td><td>dirMS_041712_NAR_60mM_plus.5212.5212.3</td><td>3</td><td>15.01</td><td>1</td><td>4.08E+05</td><td></td><td>(K)VSSAEG</td><td>(K)V/S|S A/E/G/A\A/K E/E P K(R)</td><td></td><td>85.05</td><td>434.8867</td><td>1302.654</td><td>-0.008</td><td>-6.2</td><td>4.79</td><td>4801.6</td><td>11.17</td><td>HUMAN</td><td>F2Z2W6</td><td>Non-histone chromosomal protein HMG-14</td></tr>
<tr><td>2979</td><td>dirMS_041712_NAR_20mM_minus.10877.10877.2</td><td>2</td><td>15</td><td>0</td><td>1.50E+06</td><td>M92m</td><td>(R)ImNTFS</td><td>(R)I m|N T/F/S V V/P/S/P K(V)</td><td>m:Oxidized methionine</td><td>157.1</td><td>668.3513</td><td>1319.703</td><td>15.9925</td><td>-1.8</td><td>8.75</td><td>42831.5</td><td>4.86</td><td>HUMAN</td><td>A8K854</td><td>HCG1983504, isoform CRA_f</td></tr>
<tr><td>2980</td><td>dirMS_041712_NAR_50mM_minus2.13359.13359.3</td><td>3</td><td>15</td><td>0</td><td>3.68E+05</td><td></td><td>(R)EQTLSP</td><td>(R)E Q|T/L/S/P T/I/T/S G L H N\/A R(S)</td><td></td><td>143.63</td><td>613.3274</td><td>1837.977</td><td>-0.009</td><td>-4.9</td><td>6.85</td><td>128523.4</td><td>7.06</td><td>HUMAN</td><td>B7ZL00</td><td>Protein transport protein Sec31A</td></tr>
<tr><td>2981</td><td>dirMS_041712_NAR_50mM_minus2.9055.9055.3</td><td>3</td><td>15</td><td>0</td><td>7.04E+04</td><td></td><td>(R)EANALA</td><td>(R)E A N|A/L/A/A G H/P A Q A V A/I N A R(L)</td><td></td><td>119.77</td><td>615.6611</td><td>1844.973</td><td>-0.0038</td><td>-2.1</td><td>6.85</td><td>272689.3</td><td>5.79</td><td>HUMAN</td><td>O15020</td><td>Spectrin beta chain, non-erythrocytic 2</td></tr>
<tr><td>2982</td><td>dirMS_041712_NAR_120mM_plus.16387.16387.4</td><td>4</td><td>15</td><td>1</td><td>1.30E+06</td><td></td><td>(R)YAEIVHL</td><td>(R)Y/A E/I V H L/T L/P/D/G/T K/R(S)</td><td></td><td>138.97</td><td>428.9885</td><td>1712.933</td><td>-0.0008</td><td>-0.5</td><td>6.75</td><td>21230</td><td>6.36</td><td>HUMAN</td><td>H0YC04</td><td>V-type proton ATPase subunit B, brain isoform (Fragment)</td></tr>
<tr><td>2983</td><td>dirMS_041712_NAR_120mM_plus.22933.22933.3</td><td>3</td><td>15</td><td>1</td><td>4.81E+05</td><td></td><td>(K)GLAPDLI</td><td>(K)G L|A/P/D/L/P E D L Y H L I K K(A)</td><td></td><td>174</td><td>608.0076</td><td>1822.011</td><td>-0.0027</td><td>-1.5</td><td>5.38</td><td>16789.5</td><td>10.63</td><td>HUMAN</td><td>J3KMX5</td><td>40S ribosomal protein S13</td></tr>
<tr><td>2984</td><td>dirMS_041712_NAR_50mM_plus2.9532.9532.3</td><td>3</td><td>15</td><td>0</td><td>5.99E+05</td><td></td><td>(K)GAVDG</td><td>(K)G A V/D G/G L/S/I/P H S T\K(R)</td><td></td><td>117.25</td><td>446.9057</td><td>1338.701</td><td>0.0013</td><td>1</td><td>6.74</td><td>34590.2</td><td>9.84</td><td>HUMAN</td><td>P46777</td><td>60S ribosomal protein L5</td></tr>
<tr><td>2985</td><td>dirMS_041712_NAR_120mM_minus.9020.9020.2</td><td>2</td><td>14.99</td><td>0</td><td>5.98E+05</td><td></td><td>(K)HFVALS</td><td>(K)H/F|V|A/L S T N/T T K(V)</td><td></td><td>103.33</td><td>609.8267</td><td>1218.648</td><td>-0.0016</td><td>-1.3</td><td>8.76</td><td>63374.2</td><td>8.64</td><td>HUMAN</td><td>P06744</td><td>Glucose-6-phosphate isomerase</td></tr>
<tr><td>2986</td><td>dirMS_041712_NAR_30mM_minus2.10859.10859.2</td><td>2</td><td>14.99</td><td>0</td><td>1.05E+06</td><td></td><td>(R)GYSFTT</td><td>(R)G Y|S|F/T/T T/A E R(E)</td><td></td><td>111.93</td><td>566.7699</td><td>1132.527</td><td>0.0056</td><td>4.9</td><td>6</td><td>23901.9</td><td>5.28</td><td>HUMAN</td><td>I3L1U9</td><td>Actin, cytoplasmic 2, N-terminally processed (Fragment)</td></tr>
<tr><td>2987</td><td>dirMS_041712_NAR_30mM_minus2.14619.15007.2</td><td>2</td><td>14.99</td><td>0</td><td>1.64E+07</td><td></td><td>(K)WSLLQC</td><td>(K)W/S|L/L/Q/Q/Q/K(T)</td><td></td><td>134.22</td><td>515.7847</td><td>1030.568</td><td>-0.0059</td><td>-5.7</td><td>8.75</td><td>13588.1</td><td>4.66</td><td>HUMAN</td><td>F8VP67</td><td>Keratin, type II cytoskeletal 8 (Fragment)</td></tr>
<tr><td>2988</td><td>dirMS_041712_NAR_30mM_minus2.18847.18882.2</td><td>2</td><td>14.99</td><td>0</td><td>1.91E+05</td><td></td><td>(K)CFSVLGI</td><td>(K)C|F|S|V/L/G F/C K(S)</td><td>C:Carbamidomethylation</td><td>157.42</td><td>559.2673</td><td>1117.517</td><td>0.0104</td><td>9.3</td><td>8.75</td><td>83273.6</td><td>5.55</td><td>HUMAN</td><td>P13010</td><td>X-ray repair cross-complementing protein 5</td></tr>
<tr><td>2989</td><td>dirMS_041712_NAR_40mM_minus2.14385.14413.3</td><td>3</td><td>14.99</td><td>1</td><td>2.00E+05</td><td></td><td>(R)GLLPGLI</td><td>(R)G L|L|P\G L L/P\A/P A/D K L T P K(G)</td><td></td><td>169.47</td><td>567.6813</td><td>1701.031</td><td>-0.0016</td><td>-0.9</td><td>8.59</td><td>153084.9</td><td>5.55</td><td>HUMAN</td><td>Q6P2E9</td><td>Enhancer of mRNA-decapping protein 4</td></tr>
<tr><td>2990</td><td>dirMS_041712_NAR_120mM_plus.20604.20624.4</td><td>4</td><td>14.99</td><td>1</td><td>2.15E+05</td><td></td><td>(K)AAIDWF</td><td>(K)A A|I/D/W/F D/G K E F H/G N I I/K(V)</td><td></td><td>161.23</td><td>491.0027</td><td>1960.992</td><td>-0.0026</td><td>-1.3</td><td>5.38</td><td>49181</td><td>4.93</td><td>HUMAN</td><td>K7EPT6</td><td>TATA-binding protein-associated factor 2N</td></tr>
<tr><td>2991</td><td>dirMS_041712_NAR_20mM_plus.15466.15542.2</td><td>2</td><td>14.99</td><td>0</td><td>2.37E+05</td><td></td><td>(K)TITLEVEI</td><td>(K)T I T\L/E|V|E|P S D T/I/E N V K(A)</td><td></td><td>179.03</td><td>894.4658</td><td>1787.927</td><td>-0.003</td><td>-1.7</td><td>4</td><td>17271.6</td><td>6.75</td><td>HUMAN</td><td>B4DV12</td><td>Ubiquitin</td></tr>
<tr><td>2992</td><td>dirMS_041712_NAR_30mM_plus2.16622.16814.2</td><td>2</td><td>14.99</td><td>0</td><td>3.51E+05</td><td></td><td>(R)SIQADG</td><td>(R)S I|Q|A/D/G L/V/W/G S S K(L)</td><td></td><td>140.48</td><td>674.3475</td><td>1347.69</td><td>-0.0026</td><td>-1.9</td><td>5.55</td><td>24934.4</td><td>4.5</td><td>HUMAN</td><td>P24534</td><td>Elongation factor 1-beta</td></tr>
<tr><td>2993</td><td>dirMS_041712_NAR_30mM_plus2.9587.9587.2</td><td>2</td><td>14.99</td><td>0</td><td>2.50E+05</td><td></td><td>(K)NMGLY</td><td>(K)N/M|G|L/Y/G E/R(V)</td><td></td><td>104.62</td><td>470.2224</td><td>939.435</td><td>0.0022</td><td>2.4</td><td>6</td><td>43371.1</td><td>9.64</td><td>HUMAN</td><td>E7ERW2</td><td>Aspartate aminotransferase</td></tr>
<tr><td>2994</td><td>dirMS_041712_NAR_40mM_plus2.13738.13758.3</td><td>3</td><td>14.99</td><td>1</td><td>3.43E+05</td><td></td><td>(R)QVFGEA</td><td>(R)Q V F/G E/A T/K Q/P/G I/T F I/A A K(F)</td><td></td><td>147.45</td><td>636.0176</td><td>1906.043</td><td>-0.005</td><td>-2.6</td><td>8.59</td><td>32905.8</td><td>5.88</td><td>HUMAN</td><td>C9JH19</td><td>Cathepsin D light chain (Fragment)</td></tr>
<tr><td>2995</td><td>dirMS_041712_NAR_50mM_plus2.14421.14421.3</td><td>3</td><td>14.99</td><td>0</td><td>2.70E+06</td><td></td><td>(R)LSQYQE</td><td>(R)L/S|Q/Y Q E/P L H L P G V R(V)</td><td></td><td>144.57</td><td>546.2968</td><td>1636.881</td><td>-0.0047</td><td>-2.9</td><td>6.75</td><td>80685.1</td><td>7.31</td><td>HUMAN</td><td>P05165</td><td>Propionyl-CoA carboxylase alpha chain, mitochondrial</td></tr>
<tr><td>2996</td><td>dirMS_041712_NAR_50mM_plus2.15742.15743.3</td><td>3</td><td>14.99</td><td>1</td><td>6.78E+05</td><td></td><td>(K)GCLELIK</td><td>(K)G C|L/E/L I K E T G\V|P/I/A G R(H)</td><td>C:Carbamidomethylation</td><td>151.05</td><td>571.6495</td><td>1712.936</td><td>-0.0024</td><td>-1.4</td><td>6.14</td><td>111409.8</td><td>9.02</td><td>HUMAN</td><td>F5H2F4</td><td>C-1-tetrahydrofolate synthase, cytoplasmic</td></tr>
<tr><td>2997</td><td>dirMS_041712_NAR_50mM_plus2.16579.16682.3</td><td>3</td><td>14.99</td><td>1</td><td>5.82E+05</td><td></td><td>(K)DANAKL</td><td>(K)D A/N|A K L\S E\L|E|A/A/L/Q R(A)</td><td></td><td>155.78</td><td>543.6256</td><td>1628.86</td><td>0.002</td><td>1.2</td><td>4.68</td><td>56607.4</td><td>5.37</td><td>HUMAN</td><td>F8VXB4</td><td>Keratin, type II cytoskeletal 8</td></tr>
<tr><td>2998</td><td>dirMS_041712_NAR_60mM_plus.12164.12164.3</td><td>3</td><td>14.99</td><td>1</td><td>1.01E+06</td><td>M232m</td><td>(R)MSmKE</td><td>(R)M/S m K E\V D\E Q|M/L/N/V/Q/N K(N)</td><td>m:Oxidized methionine</td><td>120.57</td><td>647.3021</td><td>1923.897</td><td>15.9945</td><td>-0.2</td><td>4.68</td><td>40069.4</td><td>4.83</td><td>HUMAN</td><td>E7EWR1</td><td>Tubulin beta chain</td></tr>
<tr><td>2999</td><td>dirMS_041712_NAR_60mM_minus.7747.7857.3</td><td>3</td><td>14.98</td><td>1</td><td>4.17E+05</td><td></td><td>(K)KGTVEG</td><td>(K)K G T V E G\F\E|P/A/D/N K(C)</td><td></td><td>103.35</td><td>464.5665</td><td>1391.68</td><td>0.0048</td><td>3.5</td><td>4.68</td><td>13286.2</td><td>9.99</td><td>HUMAN</td><td>H0YLA2</td><td>Signal recognition particle 14 kDa protein</td></tr>
<tr><td>3000</td><td>dirMS_041712_NAR_20mM_plus.8308.8308.2</td><td>2</td><td>14.98</td><td>0</td><td>9.05E+04</td><td></td><td>(K)GGEIQP</td><td>(K)G G\E|I|Q/P/V/S\V K(V)</td><td></td><td>127.42</td><td>507.2841</td><td>1013.563</td><td>-0.0017</td><td>-1.6</td><td>6</td><td>10803.5</td><td>9.82</td><td>HUMAN</td><td>B8ZZL8</td><td>10 kDa heat shock protein, mitochondrial</td></tr>
<tr><td>3001</td><td>dirMS_041712_NAR_30mM_plus2.19078.19078.2</td><td>2</td><td>14.98</td><td>0</td><td>1.48E+05</td><td></td><td>(R)VYSLFLD</td><td>(R)V/Y/S/L/F L/D E S R(S)</td><td></td><td>153.18</td><td>614.8154</td><td>1228.621</td><td>0.0027</td><td>2.2</td><td>4.37</td><td>46476.4</td><td>5.33</td><td>HUMAN</td><td>B3KQ59</td><td>RuvB-like 2</td></tr>
<tr><td>3002</td><td>dirMS_041712_NAR_40mM_plus2.10241.10456.2</td><td>2</td><td>14.98</td><td>1</td><td>8.78E+05</td><td></td><td>(K)KITIADC</td><td>(K)K\I T\I|A|D\C\G\Q|L|E(-)</td><td>C:Carbamidomethylation</td><td>125.52</td><td>624.3146</td><td>1247.63</td><td>-0.0081</td><td>-6.5</td><td>4.75</td><td>18240.4</td><td>7.96</td><td>HUMAN</td><td>P62937</td><td>Peptidyl-prolyl cis-trans isomerase A</td></tr>
<tr><td>3003</td><td>dirMS_041712_NAR_50mM_plus2.5143.5143.3</td><td>3</td><td>14.98</td><td>0</td><td>7.90E+04</td><td></td><td>(R)AEAEAG</td><td>(R)A E|A|E A/G S P R P D|P R(E)</td><td></td><td>85.77</td><td>451.5555</td><td>1352.655</td><td>-0.0034</td><td>-2.5</td><td>4.68</td><td>558007.1</td><td>5.44</td><td>HUMAN</td><td>P58107</td><td>Epiplakin</td></tr>
<tr><td>3004</td><td>dirMS_041712_NAR_60mM_plus.8513.8513.3</td><td>3</td><td>14.98</td><td>1</td><td>4.16E+05</td><td></td><td>(K)QGIETP</td><td>(K)Q G/I/E/T|P E\D Q/N D/L R K(M)</td><td></td><td>102.63</td><td>548.2719</td><td>1642.803</td><td>-0.002</td><td>-1.2</td><td>4.32</td><td>46648.4</td><td>9.67</td><td>HUMAN</td><td>B4DX42</td><td>Splicing factor 1</td></tr>
<tr><td>3005</td><td>dirMS_041712_NAR_30mM_minus2.14735.14896.2</td><td>2</td><td>14.97</td><td>0</td><td>4.17E+05</td><td></td><td>(R)LWVDE</td><td>(R)L/W|V/D/E/A V/R(A)</td><td></td><td>134.55</td><td>494.2641</td><td>987.526</td><td>-0.0049</td><td>-4.9</td><td>4.37</td><td>266161</td><td>5.68</td><td>HUMAN</td><td>E9PPU0</td><td>Epiplakin</td></tr>
<tr><td>3006</td><td>dirMS_041712_NAR_40mM_minus2.13115.13115.3</td><td>3</td><td>14.97</td><td>1</td><td>9.20E+04</td><td></td><td>(K)VLDSGA</td><td>(K)V L/D/S/G A/P I K/I/P V/G P E/T L G R(I)</td><td></td><td>160.87</td><td>640.3618</td><td>1919.096</td><td>-0.0252</td><td>-13.1</td><td>6.04</td><td>55307.5</td><td>5.25</td><td>HUMAN</td><td>F8VPV9</td><td>ATP synthase subunit beta</td></tr>
<tr><td>3007</td><td>dirMS_041712_NAR_50mM_minus2.7786.7786.3</td><td>3</td><td>14.97</td><td>0</td><td>2.44E+05</td><td></td><td>(R)LESGMC</td><td>(R)L E|S G|M Q/N|M/S I H T K(T)</td><td></td><td>111.27</td><td>492.5712</td><td>1475.698</td><td>0.0009</td><td>0.6</td><td>6.75</td><td>56607.4</td><td>5.37</td><td>HUMAN</td><td>F8VXB4</td><td>Keratin, type II cytoskeletal 8</td></tr>
<tr><td>3008</td><td>dirMS_041712_NAR_60mM_minus.9565.9565.3</td><td>3</td><td>14.97</td><td>1</td><td>4.95E+05</td><td></td><td>(R)LQAEIE</td><td>(R)L/Q|A/E/I/E/G L K G Q R(A)</td><td></td><td>113.32</td><td>447.9164</td><td>1341.748</td><td>-0.0138</td><td>-10.3</td><td>6.14</td><td>56607.4</td><td>5.37</td><td>HUMAN</td><td>F8VXB4</td><td>Keratin, type II cytoskeletal 8</td></tr>
<tr><td>3009</td><td>dirMS_041712_NAR_120mM_plus.8299.8300.3</td><td>3</td><td>14.97</td><td>2</td><td>1.65E+06</td><td></td><td>(K)KGAVAE</td><td>(K)K|G A|V/A E D G D/E/L R T E|P E|A\K(K)</td><td></td><td>97.05</td><td>638.9851</td><td>1914.94</td><td>0.0004</td><td>0.2</td><td>4.36</td><td>19284.3</td><td>5.23</td><td>HUMAN</td><td>G3V3C7</td><td>DNA-(apurinic or apyrimidinic site) lyase (Fragment)</td></tr>
<tr><td>3010</td><td>dirMS_041712_NAR_120mM_plus.9219.9343.3</td><td>3</td><td>14.97</td><td>1</td><td>3.55E+06</td><td></td><td>(R)SRAEAE</td><td>(R)S R A E\A\E|S|M/Y/Q/I/K(Y)</td><td></td><td>102.38</td><td>471.5662</td><td>1412.684</td><td>0.0002</td><td>0.1</td><td>5.86</td><td>37296.3</td><td>5.3</td><td>HUMAN</td><td>F8VU64</td><td>Keratin, type II cytoskeletal 8</td></tr>
<tr><td>3011</td><td>dirMS_041712_NAR_30mM_plus2.22031.22031.2</td><td>2</td><td>14.97</td><td>0</td><td>2.62E+05</td><td></td><td>(K)LISLFQA</td><td>(K)L/I/S|L F Q A\M K(K)</td><td></td><td>171.07</td><td>525.8042</td><td>1050.602</td><td>-0.0005</td><td>-0.5</td><td>8.75</td><td>533778</td><td>5.74</td><td>HUMAN</td><td>Q15149</td><td>Plectin</td></tr>
<tr><td>3012</td><td>dirMS_041712_NAR_50mM_plus2.9541.9541.3</td><td>3</td><td>14.97</td><td>1</td><td>1.58E+05</td><td></td><td>(R)LGGSPF</td><td>(R)L/G|G/S/P F G/P A G T G K T E/S V\K(A)</td><td></td><td>117.22</td><td>563.9657</td><td>1689.881</td><td>0.0019</td><td>1.1</td><td>8.59</td><td>535136.6</td><td>6.01</td><td>HUMAN</td><td>Q14204</td><td>Cytoplasmic dynein 1 heavy chain 1</td></tr>
<tr><td>3013</td><td>dirMS_041712_NAR_60mM_plus.9142.9180.3</td><td>3</td><td>14.97</td><td>1</td><td>9.47E+05</td><td></td><td>(K)AQAEVE</td><td>(K)A Q|A|E/V/E/G L/G K G V A R(L)</td><td></td><td>105.8</td><td>462.2549</td><td>1384.754</td><td>-0.0042</td><td>-3</td><td>6.19</td><td>533778</td><td>5.74</td><td>HUMAN</td><td>Q15149</td><td>Plectin</td></tr>
<tr><td>3014</td><td>dirMS_041712_NAR_60mM_plus.9340.9340.3</td><td>3</td><td>14.97</td><td>1</td><td>2.84E+06</td><td></td><td>(R)AYQKQF</td><td>(R)A Y/Q|K Q|P T|I/F Q N K(K)</td><td></td><td>107.38</td><td>489.2643</td><td>1465.78</td><td>-0.0014</td><td>-1</td><td>9.7</td><td>18601.6</td><td>10.52</td><td>HUMAN</td><td>P62280</td><td>40S ribosomal protein S11</td></tr>
<tr><td>3015</td><td>dirMS_041712_NAR_40mM_minus2.12688.12688.3</td><td>3</td><td>14.96</td><td>1</td><td>2.01E+05</td><td></td><td>(K)DVLFLK</td><td>(K)D V|L/F/L\K D C V/G P E V E K(A)</td><td>C:Carbamidomethylation</td><td>158.43</td><td>583.3021</td><td>1747.893</td><td>-0.0018</td><td>-1</td><td>4.32</td><td>41827.5</td><td>7</td><td>HUMAN</td><td>B7Z7A9</td><td>Phosphoglycerate kinase</td></tr>
<tr><td>3016</td><td>dirMS_041712_NAR_50mM_minus2.6924.7103.3</td><td>3</td><td>14.96</td><td>1</td><td>7.83E+05</td><td>M2747m</td><td>(K)TLQEVT</td><td>(K)T L|Q/E V/T E m/D/S V K/R(Y)</td><td>m:Oxidized methionine</td><td>106.27</td><td>517.9271</td><td>1535.773</td><td>15.9934</td><td>-1</td><td>4.68</td><td>558007.1</td><td>5.44</td><td>HUMAN</td><td>P58107</td><td>Epiplakin</td></tr>
<tr><td>3017</td><td>dirMS_041712_NAR_60mM_minus.10071.10279.3</td><td>3</td><td>14.96</td><td>0</td><td>5.90E+06</td><td></td><td>(R)GSAVHC</td><td>(R)G S/A/V/H/Q/L/S/E/E/L R(C)</td><td></td><td>117.4</td><td>442.5648</td><td>1325.681</td><td>-0.001</td><td>-0.7</td><td>5.4</td><td>558007.1</td><td>5.44</td><td>HUMAN</td><td>P58107</td><td>Epiplakin</td></tr>
<tr><td>3018</td><td>dirMS_041712_NAR_60mM_minus.9677.9677.3</td><td>3</td><td>14.96</td><td>0</td><td>3.48E+05</td><td></td><td>(R)QLSQAG</td><td>(R)Q L/S Q/A/G/S/F/S D|G T H/G G L R(Y)</td><td></td><td>113.6</td><td>573.2781</td><td>1717.825</td><td>-0.0055</td><td>-3.2</td><td>6.74</td><td>266161</td><td>5.68</td><td>HUMAN</td><td>E9PPU0</td><td>Epiplakin</td></tr>
<tr><td>3019</td><td>dirMS_041712_NAR_20mM_plus.9953.9953.2</td><td>2</td><td>14.96</td><td>0</td><td>1.64E+05</td><td></td><td>(K)YQAVTA</td><td>(K)Y/Q|A|V|T A T L E E K(R)</td><td></td><td>139.75</td><td>626.8223</td><td>1252.642</td><td>-0.0046</td><td>-3.7</td><td>4.53</td><td>23633.9</td><td>11.02</td><td>HUMAN</td><td>P40429</td><td>60S ribosomal protein L13a</td></tr>
<tr><td>3020</td><td>dirMS_041712_NAR_30mM_plus2.18765.18932.2</td><td>2</td><td>14.96</td><td>0</td><td>3.63E+05</td><td></td><td>(R)NLVCTD</td><td>(R)N L|V|C|T/D/L F T/R(G)</td><td>C:Carbamidomethylation</td><td>152.18</td><td>619.8128</td><td>1238.62</td><td>-0.0015</td><td>-1.2</td><td>5.84</td><td>54815.2</td><td>9.17</td><td>HUMAN</td><td>P26196</td><td>Probable ATP-dependent RNA helicase DDX6</td></tr>
<tr><td>3021</td><td>dirMS_041712_NAR_30mM_plus2.21961.21961.2</td><td>2</td><td>14.96</td><td>0</td><td>1.87E+05</td><td></td><td>(R)EGDVLT</td><td>(R)E G D\V/L/T|L/L/E S E R(E)</td><td></td><td>170.55</td><td>680.8538</td><td>1360.696</td><td>0.0049</td><td>3.6</td><td>4</td><td>7898</td><td>11.1</td><td>HUMAN</td><td>P62857</td><td>40S ribosomal protein S28</td></tr>
<tr><td>3022</td><td>dirMS_041712_NAR_40mM_plus2.10726.10726.3</td><td>3</td><td>14.96</td><td>0</td><td>1.85E+05</td><td></td><td>(K)VGSLDN</td><td>(K)V/G|S L D/N V/G H L/P A G G A V K(I)</td><td></td><td>128.15</td><td>530.9567</td><td>1590.86</td><td>-0.0043</td><td>-2.7</td><td>6.71</td><td>26233.5</td><td>10.95</td><td>HUMAN</td><td>B4DSQ1</td><td>Microtubule-associated protein</td></tr>
<tr><td>3023</td><td>dirMS_041712_NAR_40mM_plus2.5875.5895.2</td><td>2</td><td>14.96</td><td>0</td><td>1.61E+05</td><td>M369m</td><td>(R)FGSGMI</td><td>(R)F/G|S|G/M/N m/G R(I)</td><td>m:Oxidized methionine</td><td>92.73</td><td>486.7054</td><td>956.408</td><td>15.9958</td><td>1</td><td>9.75</td><td>77799.7</td><td>9.09</td><td>HUMAN</td><td>P52272</td><td>Heterogeneous nuclear ribonucleoprotein M</td></tr>
<tr><td>3024</td><td>dirMS_041712_NAR_30mM_minus2.12358.12358.2</td><td>2</td><td>14.95</td><td>0</td><td>1.26E+05</td><td></td><td>(K)SIYYITGI</td><td>(K)S I|Y/Y/I/T G/E/S/K(E)</td><td></td><td>121.13</td><td>580.7949</td><td>1160.583</td><td>-0.0009</td><td>-0.7</td><td>5.72</td><td>16789.1</td><td>8.77</td><td>HUMAN</td><td>H0Y5S7</td><td>Heat shock protein HSP 90-beta (Fragment)</td></tr>
<tr><td>3025</td><td>dirMS_041712_NAR_30mM_minus2.15726.16004.2</td><td>2</td><td>14.95</td><td>0</td><td>8.39E+05</td><td></td><td>(R)GGIVDE</td><td>(R)G G/I|V|D|E G/A\L L/R(A)</td><td></td><td>139.92</td><td>550.3083</td><td>1099.611</td><td>-0.0013</td><td>-1.2</td><td>4.37</td><td>57391.2</td><td>6.29</td><td>HUMAN</td><td>O43175</td><td>D-3-phosphoglycerate dehydrogenase</td></tr>
<tr><td>3026</td><td>dirMS_041712_NAR_20mM_plus.15508.15508.3</td><td>3</td><td>14.95</td><td>0</td><td>1.97E+05</td><td></td><td>(K)TITLEVEI</td><td>(K)T I\T L E V E/P S/D/T/I/E N V K(A)</td><td></td><td>179.02</td><td>596.6463</td><td>1787.927</td><td>-0.003</td><td>-1.7</td><td>4</td><td>17271.6</td><td>6.75</td><td>HUMAN</td><td>B4DV12</td><td>Ubiquitin</td></tr>
<tr><td>3027</td><td>dirMS_041712_NAR_30mM_plus2.20148.20148.2</td><td>2</td><td>14.95</td><td>0</td><td>1.34E+05</td><td></td><td>(K)NIEDVIA</td><td>(K)N I/E|D V/I/A Q G I|G/K(L)</td><td></td><td>160.27</td><td>628.8445</td><td>1256.685</td><td>-0.0028</td><td>-2.2</td><td>4.37</td><td>9098.8</td><td>4.38</td><td>HUMAN</td><td>H0YDD8</td><td>60S acidic ribosomal protein P2 (Fragment)</td></tr>
<tr><td>3028</td><td>dirMS_041712_NAR_50mM_plus2.17745.17915.3</td><td>3</td><td>14.95</td><td>0</td><td>5.68E+05</td><td></td><td>(K)TVSGVN</td><td>(K)T V|S/G V/N/G/P L/V/I/L/D H V K(V)</td><td></td><td>162.05</td><td>549.9834</td><td>1647.943</td><td>-0.0072</td><td>-4.4</td><td>6.41</td><td>6939</td><td>10.41</td><td>HUMAN</td><td>E5RGH6</td><td>V-type proton ATPase subunit B, brain isoform</td></tr>
<tr><td>3029</td><td>dirMS_041712_NAR_50mM_plus2.17749.17884.3</td><td>3</td><td>14.95</td><td>0</td><td>2.13E+06</td><td></td><td>(K)LILPHVD</td><td>(K)L/I|L/P H/V D I Q L/K(Y)</td><td></td><td>162.02</td><td>430.2706</td><td>1288.799</td><td>-0.0015</td><td>-1.2</td><td>6.74</td><td>45578.2</td><td>7.83</td><td>HUMAN</td><td>B4DFL2</td><td>Isocitrate dehydrogenase [NADP]</td></tr>
<tr><td>3030</td><td>dirMS_041712_NAR_50mM_plus2.21192.21192.3</td><td>3</td><td>14.95</td><td>0</td><td>2.41E+06</td><td></td><td>(R)AGKPVI</td><td>(R)A G K\P V\I\C A\T Q/M|L/E S M/I/K(K)</td><td>C:Carbamidomethylation</td><td>180.53</td><td>626.3278</td><td>1876.969</td><td>-0.0005</td><td>-0.3</td><td>8.64</td><td>18466.1</td><td>5.78</td><td>HUMAN</td><td>H3BSU3</td><td>Pyruvate kinase (Fragment)</td></tr>
<tr><td>3031</td><td>dirMS_041712_NAR_60mM_plus.8495.8495.3</td><td>3</td><td>14.95</td><td>0</td><td>5.94E+05</td><td></td><td>(K)GQVCVI</td><td>(K)G Q|V|C V/M/I/H S/G S/R(G)</td><td>C:Carbamidomethylation</td><td>103.07</td><td>444.2219</td><td>1330.636</td><td>0.0157</td><td>11.8</td><td>9.75</td><td>55722.8</td><td>6.78</td><td>HUMAN</td><td>Q9Y3I0</td><td>tRNA-splicing ligase RtcB homolog</td></tr>
<tr><td>3032</td><td>dirMS_041712_NAR_120mM_minus.4713.4885.3</td><td>3</td><td>14.94</td><td>0</td><td>1.09E+07</td><td>M46m M4</td><td>(R)HQGVm</td><td>(R)H Q\G\V\m|V/G/m/G Q/K(D)</td><td>m:Oxidized methionine</td><td>75.72</td><td>401.8604</td><td>1171.571</td><td>31.9956</td><td>4.8</td><td>8.76</td><td>32616.5</td><td>5.18</td><td>HUMAN</td><td>A6NL76</td><td>Actin, alpha skeletal muscle</td></tr>
<tr><td>3033</td><td>dirMS_041712_NAR_120mM_minus.6610.6610.2</td><td>2</td><td>14.94</td><td>1</td><td>1.91E+05</td><td></td><td>(K)SYKVSTS</td><td>(K)S Y/K/V/S/T/S\G P/R(A)</td><td></td><td>90.57</td><td>541.2833</td><td>1081.564</td><td>-0.0043</td><td>-4</td><td>9.99</td><td>19288.4</td><td>10.19</td><td>HUMAN</td><td>F8VQY3</td><td>Keratin, type II cytoskeletal 8 (Fragment)</td></tr>
<tr><td>3034</td><td>dirMS_041712_NAR_30mM_minus2.10107.10151.2</td><td>2</td><td>14.94</td><td>0</td><td>2.88E+06</td><td></td><td>(K)VIPELNC</td><td>(K)V I|P/E L/N/G/K(L)</td><td></td><td>109.28</td><td>435.2563</td><td>869.509</td><td>-0.0038</td><td>-4.3</td><td>5.97</td><td>31718.5</td><td>7.17</td><td>HUMAN</td><td>E7EUT4</td><td>Glyceraldehyde-3-phosphate dehydrogenase</td></tr>
<tr><td>3035</td><td>dirMS_041712_NAR_30mM_minus2.17788.17788.2</td><td>2</td><td>14.94</td><td>0</td><td>1.59E+04</td><td></td><td>(R)SLAALSC</td><td>(R)S L|A|A|L|S/Q I A|Y/Q R(N)</td><td></td><td>150.98</td><td>660.8642</td><td>1320.727</td><td>-0.0059</td><td>-4.5</td><td>8.46</td><td>64225</td><td>6.66</td><td>HUMAN</td><td>E7EX44</td><td>Caldesmon</td></tr>
<tr><td>3036</td><td>dirMS_041712_NAR_60mM_minus.9341.9378.3</td><td>3</td><td>14.94</td><td>0</td><td>1.14E+06</td><td></td><td>(R)QSVENI</td><td>(R)Q/S/V|E N D/I/H/G L/R(K)</td><td></td><td>112.07</td><td>423.218</td><td>1267.639</td><td>0.0005</td><td>0.4</td><td>5.32</td><td>43773.3</td><td>5.26</td><td>HUMAN</td><td>F8VZY9</td><td>Keratin, type I cytoskeletal 18</td></tr>
<tr><td>3037</td><td>dirMS_041712_NAR_20mM_plus.11476.11476.2</td><td>2</td><td>14.94</td><td>0</td><td>2.08E+05</td><td></td><td>(K)VGLQVV</td><td>(K)V/G/L/Q/V V/A V K(A)</td><td></td><td>151.53</td><td>456.7977</td><td>912.588</td><td>0.0004</td><td>0.5</td><td>8.72</td><td>61224.7</td><td>5.7</td><td>HUMAN</td><td>P10809</td><td>60 kDa heat shock protein, mitochondrial</td></tr>
<tr><td>3038</td><td>dirMS_041712_NAR_20mM_plus.16626.16626.2</td><td>2</td><td>14.94</td><td>0</td><td>1.99E+05</td><td></td><td>(K)VQIAVA</td><td>(K)V Q|I/A V/A|N A/Q\E L|L Q R(M)</td><td></td><td>186.25</td><td>776.9419</td><td>1552.881</td><td>-0.004</td><td>-2.6</td><td>5.97</td><td>10728.1</td><td>9.15</td><td>HUMAN</td><td>Q9Y5L4</td><td>Mitochondrial import inner membrane translocase subunit Tim13</td></tr>
<tr><td>3039</td><td>dirMS_041712_NAR_20mM_plus.18713.18731.2</td><td>2</td><td>14.94</td><td>0</td><td>2.37E+05</td><td></td><td>(K)IYVDDG</td><td>(K)I/Y|V/D/D G L|I|S L Q V K(Q)</td><td></td><td>200.67</td><td>731.9068</td><td>1462.815</td><td>-0.0088</td><td>-6</td><td>4.21</td><td>30947.9</td><td>6.47</td><td>HUMAN</td><td>H3BQ34</td><td>Pyruvate kinase</td></tr>
<tr><td>3040</td><td>dirMS_041712_NAR_40mM_plus2.16308.16308.2</td><td>2</td><td>14.94</td><td>0</td><td>1.50E+05</td><td></td><td>(R)YLTVAA</td><td>(R)Y/L|T\V/A/A/V/F/R(G)</td><td></td><td>162.03</td><td>520.2997</td><td>1039.593</td><td>-0.0014</td><td>-1.3</td><td>8.75</td><td>40069.4</td><td>4.83</td><td>HUMAN</td><td>E7EWR1</td><td>Tubulin beta chain</td></tr>
<tr><td>3041</td><td>dirMS_041712_NAR_60mM_plus.20201.20202.3</td><td>3</td><td>14.94</td><td>1</td><td>3.02E+05</td><td></td><td>(K)IILDLISE</td><td>(K)I I|L/D|L/I/S E/S P I K/G R(A)</td><td></td><td>166.2</td><td>518.6452</td><td>1553.926</td><td>-0.0051</td><td>-3.3</td><td>6.07</td><td>51260.6</td><td>5.39</td><td>HUMAN</td><td>P61978</td><td>Heterogeneous nuclear ribonucleoprotein K</td></tr>
<tr><td>3042</td><td>dirMS_041712_NAR_40mM_minus2.13543.13543.3</td><td>3</td><td>14.93</td><td>1</td><td>1.96E+05</td><td></td><td>(K)LGVQVV</td><td>(K)L/G V/Q/V/V/I/T/D P E K L D Q I R(Q)</td><td></td><td>163.38</td><td>641.7007</td><td>1923.091</td><td>-0.0034</td><td>-1.8</td><td>4.56</td><td>44227.3</td><td>7.81</td><td>HUMAN</td><td>E7EQR6</td><td>T-complex protein 1 subunit alpha</td></tr>
<tr><td>3043</td><td>dirMS_041712_NAR_120mM_plus.13476.13476.3</td><td>3</td><td>14.93</td><td>1</td><td>2.61E+05</td><td></td><td>(R)AVVGVV</td><td>(R)A V|V/G V/V/A G G G R I\D K P I L K(A)</td><td></td><td>124.08</td><td>583.6945</td><td>1749.074</td><td>-0.0056</td><td>-3.2</td><td>9.99</td><td>24115.6</td><td>11.59</td><td>HUMAN</td><td>E9PIZ3</td><td>60S ribosomal protein L8</td></tr>
<tr><td>3044</td><td>dirMS_041712_NAR_30mM_plus2.13663.13701.2</td><td>2</td><td>14.93</td><td>0</td><td>9.41E+04</td><td></td><td>(K)GLGLDE</td><td>(K)G L|G|L/D|E\S/G L/A/K(Q)</td><td></td><td>124.92</td><td>530.2889</td><td>1059.568</td><td>0.0025</td><td>2.3</td><td>4.37</td><td>50569.4</td><td>6.03</td><td>HUMAN</td><td>Q9Y265</td><td>RuvB-like 1</td></tr>
<tr><td>3045</td><td>dirMS_041712_NAR_30mM_plus2.16829.17007.2</td><td>2</td><td>14.93</td><td>0</td><td>4.15E+05</td><td></td><td>(K)FVADGII</td><td>(K)F/V|A/D|G I/F/K(A)</td><td></td><td>141.65</td><td>448.7485</td><td>896.488</td><td>0.0021</td><td>2.3</td><td>5.84</td><td>13054.7</td><td>9.59</td><td>HUMAN</td><td>E9PJH4</td><td>40S ribosomal protein S3</td></tr>
<tr><td>3046</td><td>dirMS_041712_NAR_30mM_minus2.18866.18866.2</td><td>2</td><td>14.92</td><td>0</td><td>1.91E+05</td><td></td><td>(K)CFSVLGI</td><td>(K)C\F/S\V/L/G F/C K(S)</td><td>C:Carbamidomethylation</td><td>157.42</td><td>559.2673</td><td>1117.517</td><td>0.0104</td><td>9.3</td><td>8.75</td><td>83273.6</td><td>5.55</td><td>HUMAN</td><td>P13010</td><td>X-ray repair cross-complementing protein 5</td></tr>
<tr><td>3047</td><td>dirMS_041712_NAR_50mM_minus2.14601.14601.3</td><td>3</td><td>14.92</td><td>0</td><td>3.68E+05</td><td></td><td>(R)LASTLVI</td><td>(R)L/A/S T L V H L|G E Y/Q/A|A\V/D/G A R(K)</td><td></td><td>150.95</td><td>657.6803</td><td>1971.029</td><td>-0.0031</td><td>-1.6</td><td>5.32</td><td>71165.3</td><td>5.57</td><td>HUMAN</td><td>J3KS13</td><td>Clathrin heavy chain 1</td></tr>
</table>

<table>
<tr><td></td><td></td><td></td><td></td><td></td><td></td><td></td><td></td><td></td><td></td><td></td><td></td><td></td><td></td><td></td><td></td><td></td><td></td><td></td><td></td><td></td></tr>
<tr><td>3048</td><td>dirMS_041712_NAR_60mM_minus.8214.8214.3</td><td>3</td><td>14.92</td><td>1</td><td>1.40E+05</td><td></td><td>(R)LSQIGVE</td><td>(R)L/S|Q/I/G V/E N T E E N R R(L)</td><td></td><td>106.05</td><td>548.9481</td><td>1644.83</td><td>-0.0002</td><td>-0.1</td><td>4.79</td><td>36636.9</td><td>7.84</td><td>HUMAN</td><td>A8MVZ9</td><td>Fructose-bisphosphate aldolase</td></tr>
<tr><td>3049</td><td>dirMS_041712_NAR_60mM_minus.9884.9912.3</td><td>3</td><td>14.92</td><td>0</td><td>6.26E+05</td><td></td><td>(R)AHSSM\</td><td>(R)A H\S S\M|V|G|V/N/L/P Q K(A)</td><td></td><td>114.95</td><td>456.576</td><td>1367.71</td><td>0.0035</td><td>2.5</td><td>8.8</td><td>41827.5</td><td>7</td><td>HUMAN</td><td>B7Z7A9</td><td>Phosphoglycerate kinase</td></tr>
<tr><td>3050</td><td>dirMS_041712_NAR_20mM_plus.14417.14417.2</td><td>2</td><td>14.92</td><td>0</td><td>1.59E+04</td><td></td><td>(K)TVFGVEI</td><td>(K)T V|F/G V/E P\D L/T R(E)</td><td></td><td>171.18</td><td>617.3237</td><td>1233.647</td><td>-0.0073</td><td>-5.9</td><td>4.37</td><td>53219.8</td><td>5.66</td><td>HUMAN</td><td>Q96KP4</td><td>Cytosolic non-specific dipeptidase</td></tr>
<tr><td>3051</td><td>dirMS_041712_NAR_50mM_minus2.11597.11597.3</td><td>3</td><td>14.91</td><td>0</td><td>2.80E+05</td><td>M291m</td><td>(K)TGEEIFG</td><td>(K)T/G E/E/I/F/G T I G m R P N A K(N)</td><td>m:Oxidized methionine</td><td>134.18</td><td>579.6239</td><td>1720.869</td><td>15.9885</td><td>-3.7</td><td>5.81</td><td>38222.2</td><td>5.77</td><td>HUMAN</td><td>E9PKG1</td><td>Protein arginine N-methyltransferase 1</td></tr>
<tr><td>3052</td><td>dirMS_041712_NAR_50mM_minus2.18184.18388.3</td><td>3</td><td>14.91</td><td>0</td><td>4.52E+06</td><td></td><td>(K)ALESPER</td><td>(K)A/L|E|S/P E R/P F\L\A I L|G G A K(V)</td><td></td><td>174.72</td><td>590.3356</td><td>1768.996</td><td>-0.0033</td><td>-1.9</td><td>6.19</td><td>41827.5</td><td>7</td><td>HUMAN</td><td>B7Z7A9</td><td>Phosphoglycerate kinase</td></tr>
<tr><td>3053</td><td>dirMS_041712_NAR_60mM_minus.18675.18797.3</td><td>3</td><td>14.91</td><td>0</td><td>2.97E+05</td><td></td><td>(K)LHDNLII</td><td>(K)L H D\N\L\I|I/S|D/L E/N T V/K(K)</td><td></td><td>165.95</td><td>575.3117</td><td>1723.922</td><td>-0.0019</td><td>-1.1</td><td>4.54</td><td>88624.6</td><td>5.09</td><td>HUMAN</td><td>C9J8U1</td><td>Cytospin-A (Fragment)</td></tr>
<tr><td>3054</td><td>dirMS_041712_NAR_120mM_plus.11184.11348.3</td><td>3</td><td>14.91</td><td>1</td><td>5.89E+05</td><td></td><td>(R)NREEEW</td><td>(R)N R E E\E\W|D|P/E/Y T/P K(S)</td><td></td><td>112.4</td><td>564.9219</td><td>1692.75</td><td>0.0011</td><td>0.7</td><td>4.25</td><td>108720.9</td><td>10.17</td><td>HUMAN</td><td>Q9Y2W1</td><td>Thyroid hormone receptor-associated protein 3</td></tr>
<tr><td>3055</td><td>dirMS_041712_NAR_120mM_plus.11589.11717.3</td><td>3</td><td>14.91</td><td>1</td><td>4.16E+06</td><td></td><td>(R)NLKASLE</td><td>(R)N L|K A S L|E/N/S|L/R(E)</td><td></td><td>114.02</td><td>415.5688</td><td>1244.696</td><td>-0.0039</td><td>-3.1</td><td>8.75</td><td>43773.3</td><td>5.26</td><td>HUMAN</td><td>F8VZY9</td><td>Keratin, type I cytoskeletal 18</td></tr>
<tr><td>3056</td><td>dirMS_041712_NAR_120mM_plus.8174.8174.2</td><td>2</td><td>14.91</td><td>0</td><td>5.56E+05</td><td></td><td>(K)HSGPNS</td><td>(K)H/S|G|P N S A D/S A N\D/G F V R(L)</td><td></td><td>96.55</td><td>815.8631</td><td>1630.72</td><td>-0.0015</td><td>-0.9</td><td>5.21</td><td>46013.4</td><td>5.38</td><td>HUMAN</td><td>P52597</td><td>Heterogeneous nuclear ribonucleoprotein F</td></tr>
<tr><td>3057</td><td>dirMS_041712_NAR_20mM_plus.8299.8341.2</td><td>2</td><td>14.91</td><td>0</td><td>1.06E+05</td><td>M406m</td><td>(R)EYQELm</td><td>(R)E Y|Q/E/L/m/N V/K(L)</td><td>m:Oxidized methionine</td><td>127.52</td><td>585.2801</td><td>1153.556</td><td>15.9971</td><td>1.9</td><td>4.53</td><td>56607.4</td><td>5.37</td><td>HUMAN</td><td>F8VXB4</td><td>Keratin, type II cytoskeletal 8</td></tr>
<tr><td>3058</td><td>dirMS_041712_NAR_30mM_plus2.20223.20223.2</td><td>2</td><td>14.91</td><td>0</td><td>1.79E+05</td><td></td><td>(R)FLGTEVL</td><td>(R)F/L/G/T/E/V L L/R(L)</td><td></td><td>160.75</td><td>524.3115</td><td>1047.62</td><td>-0.004</td><td>-3.8</td><td>6</td><td>103045.5</td><td>7.1</td><td>HUMAN</td><td>B9ZVT1</td><td>RNA-binding protein 12B</td></tr>
<tr><td>3059</td><td>dirMS_041712_NAR_30mM_plus2.9002.9002.2</td><td>2</td><td>14.91</td><td>0</td><td>2.40E+05</td><td></td><td>(R)SGQGAF</td><td>(R)S G|Q|G/A|F|G N/M/C R(G)</td><td>C:Carbamidomethylation</td><td>100.87</td><td>592.7504</td><td>1184.494</td><td>0</td><td>0</td><td>9.47</td><td>18988.7</td><td>11.2</td><td>HUMAN</td><td>H3BTP7</td><td>60S ribosomal protein L4 (Fragment)</td></tr>
<tr><td>3060</td><td>dirMS_041712_NAR_40mM_plus2.18369.18369.3</td><td>3</td><td>14.91</td><td>1</td><td>2.86E+05</td><td></td><td>(R)VAAKLE\</td><td>(R)V A/A K L E V\A|P/I/S D I I A I K(S)</td><td></td><td>174.02</td><td>584.3591</td><td>1751.068</td><td>-0.005</td><td>-2.8</td><td>6.04</td><td>24273.5</td><td>8.98</td><td>HUMAN</td><td>H0YK49</td><td>Electron transfer flavoprotein subunit alpha, mitochondrial</td></tr>
<tr><td>3061</td><td>dirMS_041712_NAR_50mM_plus2.10725.10725.3</td><td>3</td><td>14.91</td><td>1</td><td>8.49E+05</td><td></td><td>(R)DISTNK\</td><td>(R)D I/S T N K V E Q|I|P Y/G E R(I)</td><td></td><td>124.08</td><td>583.6304</td><td>1748.881</td><td>-0.0047</td><td>-2.7</td><td>4.68</td><td>160853.1</td><td>6.11</td><td>HUMAN</td><td>F8VU51</td><td>YLP motif-containing protein 1</td></tr>
<tr><td>3062</td><td>dirMS_041712_NAR_20mM_minus.6166.6174.2</td><td>2</td><td>14.9</td><td>0</td><td>3.76E+06</td><td></td><td>(K)DSYVGD</td><td>(K)D S/Y|V|G/D E/A/Q/S K(R)</td><td></td><td>103.87</td><td>599.7642</td><td>1198.522</td><td>-0.0011</td><td>-0.9</td><td>4.03</td><td>32616.5</td><td>5.18</td><td>HUMAN</td><td>A6NL76</td><td>Actin, alpha skeletal muscle</td></tr>
<tr><td>3063</td><td>dirMS_041712_NAR_30mM_minus2.10495.10634.2</td><td>2</td><td>14.9</td><td>0</td><td>1.09E+06</td><td></td><td>(K)TIAPALV</td><td>(K)T I|A|P A L V/S K(K)</td><td></td><td>111.75</td><td>450.2803</td><td>899.556</td><td>-0.0027</td><td>-3</td><td>8.41</td><td>21077</td><td>9.05</td><td>HUMAN</td><td>K7EM90</td><td>Alpha-enolase (Fragment)</td></tr>
<tr><td>3064</td><td>dirMS_041712_NAR_40mM_minus2.11171.11171.3</td><td>3</td><td>14.9</td><td>0</td><td>8.43E+04</td><td></td><td>(K)MLDAEC</td><td>(K)M/L/D/A E D/I V N/T A R P D/E|K(A)</td><td></td><td>148.55</td><td>606.297</td><td>1816.875</td><td>0.0019</td><td>1</td><td>4.1</td><td>105308.6</td><td>5.27</td><td>HUMAN</td><td>O43707</td><td>Alpha-actinin-4</td></tr>
<tr><td>3065</td><td>dirMS_041712_NAR_20mM_plus.17953.17953.2</td><td>2</td><td>14.9</td><td>0</td><td>1.97E+05</td><td></td><td>(K)GADFLV</td><td>(K)G A D F L|V|T E|V/E|N G G/S L G S K(K)</td><td></td><td>195.2</td><td>890.4414</td><td>1779.876</td><td>-0.0004</td><td>-0.2</td><td>4.14</td><td>30947.9</td><td>6.47</td><td>HUMAN</td><td>H3BQ34</td><td>Pyruvate kinase</td></tr>
<tr><td>3066</td><td>dirMS_041712_NAR_50mM_plus2.18319.18462.3</td><td>3</td><td>14.9</td><td>1</td><td>4.07E+05</td><td></td><td>(R)IAKEEIF(</td><td>(R)I A K E E I|F|G|P V/Q/P L/F K(F)</td><td></td><td>164.98</td><td>572.6586</td><td>1715.973</td><td>-0.0118</td><td>-6.9</td><td>6.14</td><td>57661.7</td><td>6.36</td><td>HUMAN</td><td>P30837</td><td>Aldehyde dehydrogenase X, mitochondrial</td></tr>
<tr><td>3067</td><td>dirMS_041712_NAR_50mM_plus2.7315.7315.3</td><td>3</td><td>14.9</td><td>1</td><td>1.93E+05</td><td></td><td>(R)IVEQDTI</td><td>(R)I/V/E Q/D T M/P/P K G V/R(H)</td><td></td><td>104.88</td><td>490.5973</td><td>1469.778</td><td>-0.0007</td><td>-0.5</td><td>6.07</td><td>73386.6</td><td>8.73</td><td>HUMAN</td><td>B4DXX7</td><td>ATP-dependent RNA helicase DDX3Y</td></tr>
<tr><td>3068</td><td>dirMS_041712_NAR_120mM_minus.8074.8096.4</td><td>4</td><td>14.89</td><td>2</td><td>6.38E+05</td><td></td><td>(R)ASVDTK</td><td>(R)A S\V D T K E/A E/G A/P/Q/V/E A G K/R(L)</td><td></td><td>98.37</td><td>486.5008</td><td>1942.983</td><td>-0.0015</td><td>-0.8</td><td>4.87</td><td>54214.9</td><td>5.66</td><td>HUMAN</td><td>C9J813</td><td>Caldesmon (Fragment)</td></tr>
<tr><td>3069</td><td>dirMS_041712_NAR_120mM_minus.9989.10132.2</td><td>2</td><td>14.89</td><td>0</td><td>9.76E+05</td><td></td><td>(R)HVLVTL(</td><td>(R)H|V|L|V/T L\G E|K(M)</td><td></td><td>109.08</td><td>498.298</td><td>995.588</td><td>0.0003</td><td>0.3</td><td>6.75</td><td>26991.7</td><td>5.01</td><td>HUMAN</td><td>B7Z6Z4</td><td>Myosin light polypeptide 6</td></tr>
<tr><td>3070</td><td>dirMS_041712_NAR_30mM_minus2.16662.16662.2</td><td>2</td><td>14.89</td><td>0</td><td>1.61E+05</td><td></td><td>(R)NPSAAF</td><td>(R)N/P S/A A/F|F/C V A/R(L)</td><td>C:Carbamidomethylation</td><td>144.18</td><td>620.3013</td><td>1239.594</td><td>0.0014</td><td>1.1</td><td>9.75</td><td>18626.5</td><td>5.64</td><td>HUMAN</td><td>B8ZZK2</td><td>Gamma-glutamylcyclotransferase</td></tr>
<tr><td>3071</td><td>dirMS_041712_NAR_30mM_minus2.18825.18825.2</td><td>2</td><td>14.89</td><td>0</td><td>2.45E+05</td><td></td><td>(K)LLQDFFI</td><td>(K)L/L|Q/D/F/F/N G R(D)</td><td></td><td>157.37</td><td>555.2906</td><td>1109.574</td><td>0.0001</td><td>0.1</td><td>5.84</td><td>60126</td><td>5.44</td><td>HUMAN</td><td>E7EP94</td><td>Heat shock 70 kDa protein 1A/1B</td></tr>
<tr><td>3072</td><td>dirMS_041712_NAR_30mM_minus2.8947.9315.2</td><td>2</td><td>14.89</td><td>0</td><td>1.60E+07</td><td></td><td>(R)AQYDEL</td><td>(R)A/Q|Y/D/E/L/A/R(K)</td><td></td><td>104.02</td><td>483.2423</td><td>965.469</td><td>0.0086</td><td>8.9</td><td>4.37</td><td>43773.3</td><td>5.26</td><td>HUMAN</td><td>F8VZY9</td><td>Keratin, type I cytoskeletal 18</td></tr>
<tr><td>3073</td><td>dirMS_041712_NAR_50mM_minus2.13340.13573.3</td><td>3</td><td>14.89</td><td>0</td><td>3.52E+05</td><td></td><td>(R)AVAIDLF</td><td>(R)A V|A\I/D L/P/G L G/H S K(E)</td><td></td><td>144.57</td><td>426.5785</td><td>1277.721</td><td>-0.0003</td><td>-0.2</td><td>6.79</td><td>19983.6</td><td>8.14</td><td>HUMAN</td><td>B4DQI4</td><td>Alpha/beta hydrolase domain-containing protein 14B</td></tr>
<tr><td>3074</td><td>dirMS_041712_NAR_60mM_minus.8646.9072.3</td><td>3</td><td>14.89</td><td>1</td><td>2.06E+06</td><td>M227m M:</td><td>(R)mQKEIT.</td><td>(R)m Q K E I T A|L|A|P/S/T m K(I)</td><td>m:Oxidized methionine</td><td>109.67</td><td>527.6044</td><td>1548.812</td><td>31.9862</td><td>-2.3</td><td>8.35</td><td>32616.5</td><td>5.18</td><td>HUMAN</td><td>A6NL76</td><td>Actin, alpha skeletal muscle</td></tr>
<tr><td>3075</td><td>dirMS_041712_NAR_60mM_minus.9430.9463.3</td><td>3</td><td>14.89</td><td>1</td><td>2.46E+05</td><td></td><td>(R)QERYEIE</td><td>(R)Q E/R Y\E\I|E|E/T/E T V/T K(S)</td><td></td><td>112.57</td><td>585.6185</td><td>1754.844</td><td>-0.0034</td><td>-1.9</td><td>4.32</td><td>54214.9</td><td>5.66</td><td>HUMAN</td><td>C9J813</td><td>Caldesmon (Fragment)</td></tr>
<tr><td>3076</td><td>dirMS_041712_NAR_120mM_plus.16919.16920.3</td><td>3</td><td>14.89</td><td>1</td><td>1.40E+06</td><td></td><td>(K)RISEQFT</td><td>(K)R I S\E\Q|F|T|A/M/F/R(R)</td><td></td><td>141.67</td><td>462.5709</td><td>1385.699</td><td>-0.0013</td><td>-0.9</td><td>9.6</td><td>42831.5</td><td>4.86</td><td>HUMAN</td><td>A8K854</td><td>HCG1983504, isoform CRA_f</td></tr>
<tr><td>3077</td><td>dirMS_041712_NAR_20mM_plus.14731.14731.2</td><td>2</td><td>14.89</td><td>0</td><td>6.83E+04</td><td></td><td>(R)IMNTFS\</td><td>(R)I M/N T|F/S/V/V/P S P K(V)</td><td></td><td>173.27</td><td>660.3527</td><td>1319.703</td><td>-0.0047</td><td>-3.5</td><td>8.75</td><td>42831.5</td><td>4.86</td><td>HUMAN</td><td>A8K854</td><td>HCG1983504, isoform CRA_f</td></tr>
<tr><td>3078</td><td>dirMS_041712_NAR_20mM_plus.15612.15612.2</td><td>2</td><td>14.89</td><td>0</td><td>8.46E+04</td><td></td><td>(-)SDVLELT</td><td>(-)S D V/L/E/L|T/D/D N F E/S R(I)</td><td></td><td>179.27</td><td>820.3754</td><td>1639.745</td><td>-0.0011</td><td>-0.6</td><td>3.77</td><td>13747.3</td><td>6.8</td><td>HUMAN</td><td>H7BZJ3</td><td>Thioredoxin (Fragment)</td></tr>
<tr><td>3079</td><td>dirMS_041712_NAR_30mM_plus2.18779.19066.2</td><td>2</td><td>14.89</td><td>0</td><td>1.04E+06</td><td></td><td>(K)DAGTIA(</td><td>(K)D A|G\T|I|A|G/L/N V L/R(I)</td><td></td><td>152.53</td><td>600.3411</td><td>1199.674</td><td>0.0007</td><td>0.6</td><td>5.84</td><td>17973</td><td>5.16</td><td>HUMAN</td><td>E9PI65</td><td>Heat shock cognate 71 kDa protein (Fragment)</td></tr>
<tr><td>3080</td><td>dirMS_041712_NAR_120mM_minus.5001.5010.3</td><td>3</td><td>14.88</td><td>1</td><td>5.75E+05</td><td></td><td>(R)AAEEED</td><td>(R)A/A|E/E/E D/E/A/D/P K/R(Q)</td><td></td><td>79.35</td><td>453.8716</td><td>1359.602</td><td>-0.002</td><td>-1.5</td><td>4.08</td><td>11529.7</td><td>4.14</td><td>HUMAN</td><td>P20962</td><td>Parathymosin</td></tr>
<tr><td>3081</td><td>dirMS_041712_NAR_40mM_minus2.11244.11244.3</td><td>3</td><td>14.88</td><td>0</td><td>4.48E+04</td><td></td><td>(R)EALAEA</td><td>(R)E A\L/A/E/A/A L E/S P R P A L V R(S)</td><td></td><td>149.25</td><td>598.3347</td><td>1792.992</td><td>-0.002</td><td>-1.1</td><td>4.79</td><td>22148.2</td><td>5.87</td><td>HUMAN</td><td>B3KY21</td><td>Na(+)/H(+) exchange regulatory cofactor NHE-RF1</td></tr>
<tr><td>3082</td><td>dirMS_041712_NAR_60mM_minus.10625.10625.3</td><td>3</td><td>14.88</td><td>0</td><td>2.54E+05</td><td></td><td>(R)GISEETT</td><td>(R)G I|S/E/E/T T/T G V H N L/Y K(M)</td><td></td><td>119.3</td><td>550.2838</td><td>1648.818</td><td>0.0192</td><td>11.6</td><td>5.4</td><td>45228.3</td><td>6.03</td><td>HUMAN</td><td>F5H737</td><td>Adenosylhomocysteinase</td></tr>
<tr><td>3083</td><td>dirMS_041712_NAR_60mM_minus.12258.12258.3</td><td>3</td><td>14.88</td><td>0</td><td>2.05E+05</td><td></td><td>(K)LAQANG</td><td>(K)L/A\Q/A N/G W/G V/M\V/S H R(S)</td><td></td><td>128.17</td><td>509.2631</td><td>1525.769</td><td>0.0055</td><td>3.6</td><td>9.75</td><td>47510.4</td><td>7.04</td><td>HUMAN</td><td>P06733</td><td>Alpha-enolase</td></tr>
<tr><td>3084</td><td>dirMS_041712_NAR_20mM_plus.16648.16648.2</td><td>2</td><td>14.88</td><td>0</td><td>1.58E+05</td><td></td><td>(R)LLEAQIA</td><td>(R)L L/E A|Q\I|A/T G/G I|I/D\P K(E)</td><td></td><td>185.68</td><td>769.9362</td><td>1538.879</td><td>-0.0137</td><td>-8.9</td><td>4.37</td><td>334221.3</td><td>6.44</td><td>HUMAN</td><td>P15924</td><td>Desmoplakin</td></tr>
<tr><td>3085</td><td>dirMS_041712_NAR_20mM_plus.8278.8405.2</td><td>2</td><td>14.88</td><td>0</td><td>5.53E+05</td><td></td><td>(R)AVVIVDI</td><td>(R)A V|V|I|V|D/D/R(G)</td><td></td><td>127.7</td><td>443.7527</td><td>886.499</td><td>-0.0011</td><td>-1.3</td><td>4.21</td><td>23729.5</td><td>9.89</td><td>HUMAN</td><td>C9IZL7</td><td>Non-POU domain-containing octamer-binding protein (Fragment)</td></tr>
<tr><td>3086</td><td>dirMS_041712_NAR_30mM_plus2.14467.14467.2</td><td>2</td><td>14.88</td><td>0</td><td>3.41E+05</td><td></td><td>(K)TLPQMF</td><td>(K)T L|P Q/M/F T/K(T)</td><td></td><td>129.05</td><td>483.2645</td><td>965.513</td><td>0.0093</td><td>9.6</td><td>8.41</td><td>120546</td><td>7.82</td><td>HUMAN</td><td>O95486</td><td>Protein transport protein Sec24A</td></tr>
<tr><td>3087</td><td>dirMS_041712_NAR_40mM_plus2.7947.8097.2</td><td>2</td><td>14.88</td><td>0</td><td>3.26E+05</td><td></td><td>(R)MGYMD</td><td>(R)M/G|Y|M D|P/R(E)</td><td></td><td>107.5</td><td>435.1865</td><td>869.364</td><td>0.0013</td><td>1.5</td><td>5.59</td><td>26419.2</td><td>5.1</td><td>HUMAN</td><td>H0Y9K7</td><td>Splicing factor, proline- and glutamine-rich (Fragment)</td></tr>
<tr><td>3088</td><td>dirMS_041712_NAR_50mM_plus2.15477.15477.3</td><td>3</td><td>14.88</td><td>1</td><td>8.15E+05</td><td></td><td>(K)EAFSLFD</td><td>(K)E A/F|S/L/F/D K D G D G T I T T K(E)</td><td></td><td>149.52</td><td>615.6348</td><td>1844.891</td><td>-0.0014</td><td>-0.8</td><td>4.23</td><td>21860.1</td><td>4.45</td><td>HUMAN</td><td>E7EMB3</td><td>Calmodulin</td></tr>
<tr><td>3089</td><td>dirMS_041712_NAR_50mM_plus2.18836.18836.3</td><td>3</td><td>14.88</td><td>1</td><td>4.36E+05</td><td></td><td>(K)AAIDWF</td><td>(K)A/A|I/D/W/F/D G K E/F S G N P I K(V)</td><td></td><td>167.18</td><td>632.3186</td><td>1894.933</td><td>0.0079</td><td>4.1</td><td>4.56</td><td>53724.2</td><td>9.54</td><td>HUMAN</td><td>H3BPE7</td><td>RNA-binding protein FUS</td></tr>
<tr><td>3090</td><td>dirMS_041712_NAR_50mM_plus2.22478.22478.3</td><td>3</td><td>14.88</td><td>1</td><td>1.26E+06</td><td></td><td>(R)GVMLA\</td><td>(R)G V|M/L/A V/D A/V I A E L K K(Q)</td><td></td><td>195.7</td><td>519.6393</td><td>1556.908</td><td>-0.0047</td><td>-3</td><td>6.07</td><td>25225.7</td><td>9.52</td><td>HUMAN</td><td>E7ESH4</td><td>60 kDa heat shock protein, mitochondrial (Fragment)</td></tr>
<tr><td>3091</td><td>dirMS_041712_NAR_60mM_plus.11885.11885.3</td><td>3</td><td>14.88</td><td>0</td><td>8.64E+05</td><td></td><td>(R)NSNLVG</td><td>(R)N S/N|L/V/G A A/H E E/L Q Q S R(I)</td><td></td><td>119.28</td><td>584.9576</td><td>1752.862</td><td>-0.0041</td><td>-2.3</td><td>5.4</td><td>63136.9</td><td>5.83</td><td>HUMAN</td><td>D6RAQ3</td><td>Prelamin-A/C</td></tr>
<tr><td>3092</td><td>dirMS_041712_NAR_120mM_minus.17125.17188.3</td><td>3</td><td>14.87</td><td>1</td><td>4.49E+05</td><td></td><td>(K)RISEQFT</td><td>(K)R I S\E\Q|F|T|A|M/F/R(R)</td><td></td><td>147.98</td><td>462.5714</td><td>1385.699</td><td>0.0002</td><td>0.2</td><td>9.6</td><td>42831.5</td><td>4.86</td><td>HUMAN</td><td>A8K854</td><td>HCG1983504, isoform CRA_f</td></tr>
<tr><td>3093</td><td>dirMS_041712_NAR_30mM_minus2.14188.14230.2</td><td>2</td><td>14.87</td><td>0</td><td>4.17E+05</td><td></td><td>(K)MFLYAD</td><td>(K)M F|L|Y/A/D/N/E D R(A)</td><td></td><td>131.08</td><td>637.2806</td><td>1273.552</td><td>0.0022</td><td>1.7</td><td>4.03</td><td>24724.1</td><td>5.17</td><td>HUMAN</td><td>E7ETQ7</td><td>Vacuolar protein sorting-associated protein VTA1 homolog</td></tr>
<tr><td>3094</td><td>dirMS_041712_NAR_30mM_minus2.16935.16935.2</td><td>2</td><td>14.87</td><td>0</td><td>3.36E+05</td><td></td><td>(K)FANPFP.</td><td>(K)F A|N|P/F/P A A V R(G)</td><td></td><td>146.37</td><td>545.2937</td><td>1089.584</td><td>-0.0039</td><td>-3.5</td><td>9.75</td><td>61363</td><td>8.73</td><td>HUMAN</td><td>C9JQS9</td><td>Propionyl-CoA carboxylase beta chain, mitochondrial</td></tr>
<tr><td>3095</td><td>dirMS_041712_NAR_30mM_minus2.17804.18048.2</td><td>2</td><td>14.87</td><td>0</td><td>3.35E+05</td><td></td><td>(K)EAFQLFI</td><td>(K)E A|F/Q/L/F/D/R(T)</td><td></td><td>151.78</td><td>513.2571</td><td>1025.505</td><td>0.0019</td><td>1.8</td><td>4.37</td><td>26991.7</td><td>5.01</td><td>HUMAN</td><td>B7Z6Z4</td><td>Myosin light polypeptide 6</td></tr>
<tr><td>3096</td><td>dirMS_041712_NAR_30mM_minus2.8569.8569.2</td><td>2</td><td>14.87</td><td>0</td><td>9.67E+04</td><td></td><td>(R)VGQDP\</td><td>(R)V/G/Q/D/P/V L\R(Q)</td><td></td><td>101.38</td><td>442.2532</td><td>883.5</td><td>-0.0005</td><td>-0.5</td><td>5.81</td><td>14130.9</td><td>9.56</td><td>HUMAN</td><td>C9K0U8</td><td>Single-stranded DNA-binding protein (Fragment)</td></tr>
<tr><td>3097</td><td>dirMS_041712_NAR_50mM_minus2.13085.13085.3</td><td>3</td><td>14.87</td><td>0</td><td>3.53E+05</td><td></td><td>(R)ETNLDSI</td><td>(R)E T/N L/D/S L/P/L/V D/T H S K(R)</td><td></td><td>142.43</td><td>556.9529</td><td>1668.844</td><td>0.0002</td><td>0.1</td><td>4.53</td><td>53707.8</td><td>5.06</td><td>HUMAN</td><td>P08670</td><td>Vimentin</td></tr>
<tr><td>3098</td><td>dirMS_041712_NAR_60mM_minus.10038.10038.3</td><td>3</td><td>14.87</td><td>1</td><td>1.25E+05</td><td></td><td>(R)AGTGVD</td><td>(R)A G/T/G V/D/N V D L E/A/A T R\K(G)</td><td></td><td>115.55</td><td>539.6117</td><td>1616.824</td><td>-0.0033</td><td>-2</td><td>4.56</td><td>57391.2</td><td>6.29</td><td>HUMAN</td><td>O43175</td><td>D-3-phosphoglycerate dehydrogenase</td></tr>
<tr><td>3099</td><td>dirMS_041712_NAR_60mM_minus.10682.10810.3</td><td>3</td><td>14.87</td><td>1</td><td>5.44E+05</td><td>M239m</td><td>(R)MSMKE\</td><td>(R)M S|M K E V\D E\Q|m|L/N\V/Q N K(N)</td><td>m:Oxidized methionine</td><td>119.9</td><td>647.3011</td><td>1923.897</td><td>15.9915</td><td>-1.8</td><td>4.68</td><td>40069.4</td><td>4.83</td><td>HUMAN</td><td>E7EWR1</td><td>Tubulin beta chain</td></tr>
<tr><td>3100</td><td>dirMS_041712_NAR_120mM_plus.9452.9594.3</td><td>3</td><td>14.87</td><td>1</td><td>6.52E+05</td><td></td><td>(R)GSNKLV</td><td>(R)G S N K L\V|I|E/E/A/E/R(S)</td><td></td><td>102.85</td><td>448.9084</td><td>1344.712</td><td>-0.0011</td><td>-0.8</td><td>4.79</td><td>42755</td><td>7.81</td><td>HUMAN</td><td>B7Z2F4</td><td>T-complex protein 1 subunit delta</td></tr>
<tr><td>3101</td><td>dirMS_041712_NAR_120mM_plus.9727.9727.2</td><td>2</td><td>14.87</td><td>0</td><td>8.09E+05</td><td></td><td>(R)FATHAA</td><td>(R)F A/T/H|A|A\A\L S V/R(N)</td><td></td><td>104.23</td><td>572.3112</td><td>1143.627</td><td>-0.0118</td><td>-10.3</td><td>9.75</td><td>76262.2</td><td>9.53</td><td>HUMAN</td><td>P23246</td><td>Splicing factor, proline- and glutamine-rich</td></tr>
<tr><td>3102</td><td>dirMS_041712_NAR_20mM_plus.12259.12366.2</td><td>2</td><td>14.87</td><td>0</td><td>2.65E+05</td><td></td><td>(K)LLQLVEC</td><td>(K)L/L|Q|L|V/E D/R(G)</td><td></td><td>158.32</td><td>493.2863</td><td>985.568</td><td>-0.0023</td><td>-2.4</td><td>4.37</td><td>61018.6</td><td>9.44</td><td>HUMAN</td><td>B4DLW8</td><td>Probable ATP-dependent RNA helicase DDX5</td></tr>
<tr><td>3103</td><td>dirMS_041712_NAR_30mM_plus2.13726.13809.2</td><td>2</td><td>14.87</td><td>0</td><td>1.74E+05</td><td></td><td>(K)FAFQAE'</td><td>(K)F/A|F/Q|A/E/V N/R(M)</td><td></td><td>125.3</td><td>541.2764</td><td>1081.543</td><td>0.003</td><td>2.8</td><td>6</td><td>92752.5</td><td>4.76</td><td>HUMAN</td><td>P14625</td><td>Endoplasmin</td></tr>
<tr><td>3104</td><td>dirMS_041712_NAR_30mM_plus2.14285.14580.2</td><td>2</td><td>14.87</td><td>0</td><td>7.81E+05</td><td></td><td>(R)GLFDEY(</td><td>(R)G L|F|D/E/Y/G/S K(K)</td><td></td><td>128.55</td><td>508.2403</td><td>1015.473</td><td>0.0002</td><td>0.2</td><td>4.37</td><td>92752.5</td><td>4.76</td><td>HUMAN</td><td>P14625</td><td>Endoplasmin</td></tr>
<tr><td>3105</td><td>dirMS_041712_NAR_30mM_plus2.19214.19570.2</td><td>2</td><td>14.87</td><td>0</td><td>8.31E+05</td><td></td><td>(R)DLTDYLI</td><td>(R)D L|T/D/Y/L/M/K(I)</td><td></td><td>155.33</td><td>499.7467</td><td>998.486</td><td>-0.0002</td><td>-0.2</td><td>4.21</td><td>37482.1</td><td>5.36</td><td>HUMAN</td><td>E9PG30</td><td>Actin, gamma-enteric smooth muscle</td></tr>
<tr><td>3106</td><td>dirMS_041712_NAR_50mM_plus2.8912.8912.3</td><td>3</td><td>14.87</td><td>0</td><td>1.99E+05</td><td></td><td>(K)VVQVSA</td><td>(K)V V/Q V/S/A G D/S/H T A A L/T D D G\R(V)</td><td></td><td>113.48</td><td>633.6509</td><td>1898.92</td><td>0.0179</td><td>9.4</td><td>4.41</td><td>29854.8</td><td>9.49</td><td>HUMAN</td><td>C9J3R0</td><td>Regulator of chromosome condensation (Fragment)</td></tr>
<tr><td>3107</td><td>dirMS_041712_NAR_20mM_minus.14647.14647.2</td><td>2</td><td>14.86</td><td>0</td><td>2.10E+05</td><td></td><td>(K)LAVEALS</td><td>(K)L A|V/E A|L/S|S L D/G D/L A/G R(Y)</td><td></td><td>201.28</td><td>793.922</td><td>1586.838</td><td>-0.0017</td><td>-1.1</td><td>4.03</td><td>24286.7</td><td>4.89</td><td>HUMAN</td><td>G3V4N7</td><td>Creatine kinase B-type (Fragment)</td></tr>
<tr><td>3108</td><td>dirMS_041712_NAR_50mM_minus2.13485.13485.3</td><td>3</td><td>14.86</td><td>0</td><td>2.98E+05</td><td></td><td>(K)VIQYLAY</td><td>(K)V I|Q/Y L A/Y/V/A|S S/H K(S)</td><td></td><td>144.62</td><td>493.6043</td><td>1478.8</td><td>-0.0018</td><td>-1.2</td><td>8.48</td><td>227783.5</td><td>5.5</td><td>HUMAN</td><td>P35579</td><td>Myosin-9</td></tr>
<tr><td>3109</td><td>dirMS_041712_NAR_20mM_plus.18625.18782.2</td><td>2</td><td>14.86</td><td>0</td><td>2.00E+05</td><td></td><td>(R)VFIGNLI</td><td>(R)V F|I|G|N/L N\T L/V V/K(K)</td><td></td><td>200.13</td><td>658.8999</td><td>1316.794</td><td>-0.0011</td><td>-0.9</td><td>8.72</td><td>15791.7</td><td>9.22</td><td>HUMAN</td><td>B4DSU6</td><td>Heterogeneous nuclear ribonucleoproteins C1/C2</td></tr>
<tr><td>3110</td><td>dirMS_041712_NAR_20mM_plus.9355.9355.2</td><td>2</td><td>14.86</td><td>0</td><td>1.86E+05</td><td></td><td>(K)SVTNED'</td><td>(K)S V|T|N/E/D V|T Q E E/L/G G A/K(T)</td><td></td><td>136</td><td>838.9014</td><td>1676.797</td><td>-0.0018</td><td>-1.1</td><td>4</td><td>61363</td><td>8.73</td><td>HUMAN</td><td>C9JQS9</td><td>Propionyl-CoA carboxylase beta chain, mitochondrial</td></tr>
<tr><td>3111</td><td>dirMS_041712_NAR_30mM_plus2.13474.13474.2</td><td>2</td><td>14.86</td><td>0</td><td>1.03E+05</td><td></td><td>(R)TIYAGN.</td><td>(R)T\I/Y/A|G N/A\L/C T V/K(C)</td><td>C:Carbamidomethylation</td><td>123.85</td><td>655.8414</td><td>1310.677</td><td>-0.0018</td><td>-1.4</td><td>8.26</td><td>24273.5</td><td>8.98</td><td>HUMAN</td><td>H0YK49</td><td>Electron transfer flavoprotein subunit alpha, mitochondrial</td></tr>
<tr><td>3112</td><td>dirMS_041712_NAR_30mM_plus2.19307.19787.2</td><td>2</td><td>14.86</td><td>0</td><td>7.16E+05</td><td></td><td>(R)LDFNLIR</td><td>(R)L/D|F/N/L/I/R(V)</td><td></td><td>156.18</td><td>445.759</td><td>890.509</td><td>0.0013</td><td>1.5</td><td>5.84</td><td>18956.4</td><td>6.97</td><td>HUMAN</td><td>C9JLK0</td><td>Phosphoribosylaminoimidazolecarboxamide formyltransferase (Fragment)</td></tr>
<tr><td>3113</td><td>dirMS_041712_NAR_30mM_plus2.22589.22589.2</td><td>2</td><td>14.86</td><td>0</td><td>1.01E+06</td><td></td><td>(R)FLTWILI</td><td>(R)F/L|T|W I L/N/R(Y)</td><td></td><td>175.88</td><td>531.8078</td><td>1062.61</td><td>-0.0012</td><td>-1.1</td><td>9.75</td><td>35548.6</td><td>4.91</td><td>HUMAN</td><td>B4DN60</td><td>Asparagine--tRNA ligase, cytoplasmic</td></tr>
<tr><td>3114</td><td>dirMS_041712_NAR_50mM_plus2.20623.20625.3</td><td>3</td><td>14.86</td><td>1</td><td>8.20E+05</td><td></td><td>(R)TLAEIAK</td><td>(R)T/L\A|E/I A/K A E L/D G T/I/L K(S)</td><td></td><td>176.45</td><td>562.6601</td><td>1685.968</td><td>-0.0026</td><td>-1.6</td><td>4.68</td><td>58856.7</td><td>6.26</td><td>HUMAN</td><td>Q8WXF1</td><td>Paraspeckle component 1</td></tr>
<tr><td>3115</td><td>dirMS_041712_NAR_30mM_minus2.10286.10617.2</td><td>2</td><td>14.85</td><td>0</td><td>2.26E+06</td><td></td><td>(R)LSIYEAR</td><td>(R)L/S/I/Y/E/A/R(C)</td><td></td><td>111.07</td><td>426.2334</td><td>851.462</td><td>-0.0026</td><td>-3.1</td><td>6</td><td>266161</td><td>5.68</td><td>HUMAN</td><td>E9PPU0</td><td>Epiplakin</td></tr>
<tr><td>3116</td><td>dirMS_041712_NAR_50mM_minus2.12569.12569.3</td><td>3</td><td>14.85</td><td>0</td><td>3.06E+05</td><td></td><td>(K)AASDIAI</td><td>(K)A/A/S D|I/A M T E/L/P P T H P I/R(L)</td><td></td><td>139.55</td><td>607.3158</td><td>1819.937</td><td>-0.0042</td><td>-2.3</td><td>5.32</td><td>12926.1</td><td>4.4</td><td>HUMAN</td><td>K7EM20</td><td>14-3-3 protein epsilon (Fragment)</td></tr>
<tr><td>3117</td><td>dirMS_041712_NAR_50mM_minus2.15990.16128.3</td><td>3</td><td>14.85</td><td>0</td><td>5.46E+05</td><td></td><td>(K)GSDHSA</td><td>(K)G S D H S A S L\E|P|G E|L|A/E/L V R(S)</td><td></td><td>159.35</td><td>622.978</td><td>1866.919</td><td>0.0003</td><td>0.1</td><td>4.4</td><td>12626.3</td><td>5.59</td><td>HUMAN</td><td>Q5TBR0</td><td>N-acetylneuraminic acid synthase (Fragment)</td></tr>
<tr><td>3118</td><td>dirMS_041712_NAR_50mM_minus2.17979.17999.3</td><td>3</td><td>14.85</td><td>1</td><td>1.70E+05</td><td></td><td>(K)AIDLFTD</td><td>(K)A I/D|L/F/T D|A/I/K L N P R(L)</td><td></td><td>171.33</td><td>529.6358</td><td>1586.89</td><td>0.0028</td><td>1.8</td><td>6</td><td>18141.9</td><td>4.7</td><td>HUMAN</td><td>F6VDH7</td><td>Hsc70-interacting protein (Fragment)</td></tr>
<tr><td>3119</td><td>dirMS_041712_NAR_60mM_minus.4991.4991.3</td><td>3</td><td>14.85</td><td>1</td><td>8.21E+04</td><td></td><td>(K)VSSAEG.</td><td>(K)V/S/S/A E/G A A/K\E E P/K(R)</td><td></td><td>82.42</td><td>434.8904</td><td>1302.654</td><td>0.0031</td><td>2.3</td><td>4.79</td><td>4801.6</td><td>11.17</td><td>HUMAN</td><td>F2Z2W6</td><td>Non-histone chromosomal protein HMG-14</td></tr>
<tr><td>3120</td><td>dirMS_041712_NAR_120mM_plus.15280.15380.3</td><td>3</td><td>14.85</td><td>1</td><td>6.06E+05</td><td></td><td>(K)NREPVQ</td><td>(K)N R E P\V\Q|L|E|T/L/S I/R(G)</td><td></td><td>133.35</td><td>518.9599</td><td>1554.86</td><td>0.0053</td><td>3.4</td><td>6.14</td><td>8392.6</td><td>11.84</td><td>HUMAN</td><td>J3QLI9</td><td>Small nuclear ribonucleoprotein Sm D1</td></tr>
<tr><td>3121</td><td>dirMS_041712_NAR_20mM_plus.11969.12067.2</td><td>2</td><td>14.85</td><td>0</td><td>1.20E+05</td><td></td><td>(R)IYVVDV(</td><td>(R)I/Y|V|V/D/V|G S E\P R(A)</td><td></td><td>156.17</td><td>617.3237</td><td>1233.647</td><td>-0.0073</td><td>-5.9</td><td>4.37</td><td>39066</td><td>6.61</td><td>HUMAN</td><td>A6PVW9</td><td>Selenium binding protein 1 (Fragment)</td></tr>
<tr><td>3122</td><td>dirMS_041712_NAR_20mM_plus.6014.6014.3</td><td>3</td><td>14.85</td><td>0</td><td>1.02E+06</td><td>M295m M:</td><td>(R)mGQmA</td><td>(R)m G Q\m\A m\G\G\A/m/G/I/N N R(G)</td><td>m:Oxidized methionine</td><td>100.27</td><td>534.8861</td><td>1538.67</td><td>63.9743</td><td>-3.4</td><td>9.5</td><td>43979.5</td><td>8.92</td><td>HUMAN</td><td>F5GYZ3</td><td>Non-POU domain-containing octamer-binding protein</td></tr>
<tr><td>3123</td><td>dirMS_041712_NAR_30mM_plus2.11729.11903.2</td><td>2</td><td>14.85</td><td>0</td><td>1.54E+06</td><td></td><td>(K)FYEAFSK</td><td>(K)F/Y|E/A/F/S/K(N)</td><td></td><td>115.28</td><td>446.2164</td><td>891.425</td><td>0.0008</td><td>0.9</td><td>6</td><td>20127.1</td><td>9.4</td><td>HUMAN</td><td>H0Y598</td><td>Heat shock protein HSP 90-beta (Fragment)</td></tr>
<tr><td>3124</td><td>dirMS_041712_NAR_30mM_plus2.12270.12270.2</td><td>2</td><td>14.85</td><td>0</td><td>9.10E+05</td><td></td><td>(K)EVDEQN</td><td>(K)E V|D E/Q|M L\N V/Q/N K(N)</td><td></td><td>117.03</td><td>723.8478</td><td>1446.689</td><td>-0.001</td><td>-0.7</td><td>4.14</td><td>40069.4</td><td>4.83</td><td>HUMAN</td><td>E7EWR1</td><td>Tubulin beta chain</td></tr>
<tr><td>3125</td><td>dirMS_041712_NAR_30mM_plus2.7300.7301.2</td><td>2</td><td>14.85</td><td>0</td><td>7.08E+04</td><td></td><td>(R)IVAECN.</td><td>(R)I/V|A|E/C N/A V R(Q)</td><td>C:Carbamidomethylation</td><td>92.8</td><td>516.2677</td><td>1031.53</td><td>-0.0021</td><td>-2.1</td><td>6</td><td>89360.3</td><td>5.63</td><td>HUMAN</td><td>F8W845</td><td>Catenin alpha-1</td></tr>
<tr><td>3126</td><td>dirMS_041712_NAR_30mM_plus2.8539.8539.2</td><td>2</td><td>14.85</td><td>0</td><td>8.74E+05</td><td></td><td>(R)LGLGEG.</td><td>(R)L/G/L/G E/G A/E E K(S)</td><td></td><td>98.95</td><td>501.7639</td><td>1002.51</td><td>0.0103</td><td>10.3</td><td>4.25</td><td>40096.8</td><td>9.24</td><td>HUMAN</td><td>C9JW69</td><td>Regulator of chromosome condensation (Fragment)</td></tr>
<tr><td>3127</td><td>dirMS_041712_NAR_40mM_plus2.13965.14010.3</td><td>3</td><td>14.85</td><td>0</td><td>2.17E+05</td><td></td><td>(K)VALLLLD</td><td>(K)V A|L/L|L/L/D Q/G A/S P H A A A K(N)</td><td></td><td>148.17</td><td>558.9886</td><td>1674.954</td><td>-0.0025</td><td>-1.5</td><td>6.71</td><td>482683.6</td><td>6.07</td><td>HUMAN</td><td>Q12955</td><td>Ankyrin-3</td></tr>
<tr><td>3128</td><td>dirMS_041712_NAR_50mM_plus2.10702.10847.3</td><td>3</td><td>14.85</td><td>1</td><td>8.49E+05</td><td></td><td>(R)DISTNK\</td><td>(R)D I/S T N K V/E/Q|I|P/Y/G E R(I)</td><td></td><td>124.08</td><td>583.6304</td><td>1748.881</td><td>-0.0047</td><td>-2.7</td><td>4.68</td><td>160853.1</td><td>6.11</td><td>HUMAN</td><td>F8VU51</td><td>YLP motif-containing protein 1</td></tr>
<tr><td>3129</td><td>dirMS_041712_NAR_60mM_plus.17897.17897.3</td><td>3</td><td>14.85</td><td>1</td><td>3.01E+05</td><td></td><td>(R)KGFSEGI</td><td>(R)K G F S E G L\W|E|I/E/N N P\T V/K(A)</td><td></td><td>150.67</td><td>650.0017</td><td>1947.981</td><td>0.0095</td><td>4.9</td><td>4.79</td><td>23020.5</td><td>4.53</td><td>HUMAN</td><td>A8K8G0</td><td>Hepatoma-derived growth factor</td></tr>
<tr><td>3130</td><td>dirMS_041712_NAR_120mM_minus.13153.13432.4</td><td>4</td><td>14.84</td><td>2</td><td>3.61E+06</td><td></td><td>(R)AQKDEE</td><td>(R)A Q\K D E E|K M E\I|Q/E/I/Q/L/K(E)</td><td></td><td>128.07</td><td>490.7539</td><td>1960.006</td><td>-0.0118</td><td>-6</td><td>4.58</td><td>31923.6</td><td>4.85</td><td>HUMAN</td><td>B7Z596</td><td>Tropomyosin alpha-1 chain</td></tr>
<tr><td>3131</td><td>dirMS_041712_NAR_120mM_minus.14840.14938.4</td><td>4</td><td>14.84</td><td>0</td><td>1.33E+06</td><td></td><td>(K)HLEINPC</td><td>(K)H L\E\I\N/P D/H/P I/V/E/T L R(Q)</td><td></td><td>135.22</td><td>446.4924</td><td>1782.95</td><td>-0.0019</td><td>-1.1</td><td>5.27</td><td>83605</td><td>4.97</td><td>HUMAN</td><td>P08238</td><td>Heat shock protein HSP 90-beta</td></tr>
<tr><td>3132</td><td>dirMS_041712_NAR_20mM_minus.14049.14049.2</td><td>2</td><td>14.84</td><td>0</td><td>6.94E+05</td><td></td><td>(K)SYELPDC</td><td>(K)S Y E|L/P D/G Q V|I|T I/G N E R(F)</td><td></td><td>193.68</td><td>895.9481</td><td>1790.892</td><td>-0.003</td><td>-1.7</td><td>4.14</td><td>123097.1</td><td>5.83</td><td>HUMAN</td><td>A5A3E0</td><td>POTE ankyrin domain family member F</td></tr>
<tr><td>3133</td><td>dirMS_041712_NAR_30mM_minus2.15012.15261.2</td><td>2</td><td>14.84</td><td>0</td><td>3.89E+05</td><td></td><td>(R)IAAYLFK</td><td>(R)I/A|A/Y L/F/K(G)</td><td></td><td>135.42</td><td>413.2472</td><td>825.487</td><td>0.0002</td><td>0.3</td><td>8.59</td><td>15830.9</td><td>5.16</td><td>HUMAN</td><td>J3KRF5</td><td>Clathrin heavy chain 1 (Fragment)</td></tr>
<tr><td>3134</td><td>dirMS_041712_NAR_50mM_minus2.13987.13987.3</td><td>3</td><td>14.84</td><td>0</td><td>1.88E+04</td><td></td><td>(R)LTEDLEY</td><td>(R)L/T|E/D L E/Y/H E L L D R(A)</td><td></td><td>147.45</td><td>549.2731</td><td>1645.807</td><td>-0.002</td><td>-1.2</td><td>4.17</td><td>19946.4</td><td>5.79</td><td>HUMAN</td><td>H0YCV6</td><td>Oxysterol-binding protein 1 (Fragment)</td></tr>
<tr><td>3135</td><td>dirMS_041712_NAR_20mM_plus.18714.18781.2</td><td>2</td><td>14.84</td><td>0</td><td>1.68E+05</td><td></td><td>(R)IITLTGP1</td><td>(R)I/I|T\L|T|G/P T N A I F K(A)</td><td></td><td>200.4</td><td>694.9091</td><td>1388.815</td><td>-0.0039</td><td>-2.8</td><td>8.75</td><td>38010.7</td><td>6.67</td><td>HUMAN</td><td>Q15365</td><td>Poly(rC)-binding protein 1</td></tr>
<tr><td>3136</td><td>dirMS_041712_NAR_20mM_plus.7313.7313.2</td><td>2</td><td>14.84</td><td>0</td><td>3.27E+05</td><td>M281m</td><td>(R)AEAESm</td><td>(R)A E|A/E/S m Y Q/I/K(Y)</td><td>m:Oxidized methionine</td><td>118.63</td><td>593.2759</td><td>1169.551</td><td>15.9938</td><td>-0.9</td><td>4.53</td><td>37296.3</td><td>5.3</td><td>HUMAN</td><td>F8VU64</td><td>Keratin, type II cytoskeletal 8</td></tr>
<tr><td>3137</td><td>dirMS_041712_NAR_30mM_plus2.7623.7730.2</td><td>2</td><td>14.84</td><td>0</td><td>1.29E+06</td><td></td><td>(R)TVGIVGI</td><td>(R)T V|G|I|V/G N Q/P K(V)</td><td></td><td>94.25</td><td>506.7931</td><td>1012.579</td><td>0.0004</td><td>0.3</td><td>8.41</td><td>61363</td><td>8.73</td><td>HUMAN</td><td>C9JQS9</td><td>Propionyl-CoA carboxylase beta chain, mitochondrial</td></tr>
<tr><td>3138</td><td>dirMS_041712_NAR_40mM_plus2.14726.14796.3</td><td>3</td><td>14.84</td><td>1</td><td>1.18E+05</td><td></td><td>(K)VVEEAP:</td><td>(K)V/V|E/E/A/P S I F L D/A E T R/R(A)</td><td></td><td>153.57</td><td>611.3255</td><td>1831.955</td><td>0.0071</td><td>3.9</td><td>4.41</td><td>80685.1</td><td>7.31</td><td>HUMAN</td><td>P05165</td><td>Propionyl-CoA carboxylase alpha chain, mitochondrial</td></tr>
<tr><td>3139</td><td>dirMS_041712_NAR_40mM_plus2.6994.6994.2</td><td>2</td><td>14.84</td><td>0</td><td>1.27E+05</td><td></td><td>(R)MGPVM</td><td>(R)M/G/P/V/M D/R(M)</td><td></td><td>100.62</td><td>403.1892</td><td>805.37</td><td>0.0016</td><td>2</td><td>5.59</td><td>77799.7</td><td>9.09</td><td>HUMAN</td><td>P52272</td><td>Heterogeneous nuclear ribonucleoprotein M</td></tr>
<tr><td>3140</td><td>dirMS_041712_NAR_50mM_plus2.16969.16969.3</td><td>3</td><td>14.84</td><td>0</td><td>7.73E+05</td><td></td><td>(R)QYYTLLI</td><td>(R)Q Y|Y T/L/L N Q/A/P/D\M L H R(K)</td><td></td><td>156.93</td><td>621.6447</td><td>1862.922</td><td>-0.0022</td><td>-1.2</td><td>6.74</td><td>4335.1</td><td>9.82</td><td>HUMAN</td><td>E5RH00</td><td>Ras GTPase-activating protein-binding protein 1</td></tr>
<tr><td>3141</td><td>dirMS_041712_NAR_50mM_minus2.15091.15091.3</td><td>3</td><td>14.83</td><td>1</td><td>2.94E+05</td><td></td><td>(K)VVEEAP:</td><td>(K)V/V/E/E/A/P S I F L D A E T R R(A)</td><td></td><td>153.45</td><td>611.3243</td><td>1831.955</td><td>0.0035</td><td>1.9</td><td>4.41</td><td>80685.1</td><td>7.31</td><td>HUMAN</td><td>P05165</td><td>Propionyl-CoA carboxylase alpha chain, mitochondrial</td></tr>
<tr><td>3142</td><td>dirMS_041712_NAR_60mM_minus.13397.13585.3</td><td>3</td><td>14.83</td><td>1</td><td>1.40E+06</td><td></td><td>(R)QLREYQ</td><td>(R)Q L/R E Y\Q/E/L/M N/V/K(L)</td><td></td><td>135.27</td><td>517.6033</td><td>1550.8</td><td>-0.0042</td><td>-2.7</td><td>6.14</td><td>56607.4</td><td>5.37</td><td>HUMAN</td><td>F8VXB4</td><td>Keratin, type II cytoskeletal 8</td></tr>
<tr><td>3143</td><td>dirMS_041712_NAR_60mM_minus.15457.15457.3</td><td>3</td><td>14.83</td><td>0</td><td>2.13E+05</td><td></td><td>(K)EGHVEV</td><td>(K)E/G H V E\V|V|S|E/L/L Q/R(E)</td><td></td><td>145.9</td><td>498.9349</td><td>1494.791</td><td>-0.0009</td><td>-0.6</td><td>4.75</td><td>48586.6</td><td>9.32</td><td>HUMAN</td><td>D6RCB2</td><td>Ankyrin-3</td></tr>
<tr><td>3144</td><td>dirMS_041712_NAR_60mM_minus.8171.8171.3</td><td>3</td><td>14.83</td><td>1</td><td>1.32E+05</td><td></td><td>(R)KQSTDE</td><td>(R)K\Q S T D\E\E/V/T/S L/A/K(S)</td><td></td><td>105.82</td><td>479.2451</td><td>1435.728</td><td>-0.0067</td><td>-4.7</td><td>4.68</td><td>12611.4</td><td>8.8</td><td>HUMAN</td><td>E5RIS7</td><td>Transcription elongation factor A protein 1</td></tr>
<tr><td>3145</td><td>dirMS_041712_NAR_120mM_plus.15887.15907.4</td><td>4</td><td>14.83</td><td>0</td><td>6.37E+05</td><td></td><td>(R)GISTKPV</td><td>(R)G I S T K/P V Y I/P/E/V/E/L N H K(L)</td><td></td><td>136.73</td><td>481.7682</td><td>1924.054</td><td>-0.0029</td><td>-1.5</td><td>6.75</td><td>72626.7</td><td>9.13</td><td>HUMAN</td><td>Q14258</td><td>E3 ubiquitin/ISG15 ligase TRIM25</td></tr>
<tr><td>3146</td><td>dirMS_041712_NAR_120mM_plus.7400.7687.3</td><td>3</td><td>14.83</td><td>1</td><td>2.02E+06</td><td>M281m</td><td>(R)SRAEAE:</td><td>(R)S R\A E\A\E|S/m/Y Q/I/K(Y)</td><td>m:Oxidized methionine</td><td>93.02</td><td>476.8977</td><td>1412.684</td><td>15.9947</td><td>-0.1</td><td>5.86</td><td>37296.3</td><td>5.3</td><td>HUMAN</td><td>F8VU64</td><td>Keratin, type II cytoskeletal 8</td></tr>
<tr><td>3147</td><td>dirMS_041712_NAR_30mM_plus2.13153.13189.2</td><td>2</td><td>14.83</td><td>0</td><td>7.55E+05</td><td></td><td>(R)GVVGPE</td><td>(R)G V|V|G/P E/L/Y G/R(L)</td><td></td><td>122.62</td><td>523.7794</td><td>1046.563</td><td>-0.0114</td><td>-10.9</td><td>6</td><td>266161</td><td>5.68</td><td>HUMAN</td><td>E9PPU0</td><td>Epiplakin</td></tr>
<tr><td>3148</td><td>dirMS_041712_NAR_30mM_plus2.14644.14644.2</td><td>2</td><td>14.83</td><td>0</td><td>7.63E+05</td><td></td><td>(R)GVEICIA</td><td>(R)G V\E\I/C|I/A/T/P G R(L)</td><td>C:Carbamidomethylation</td><td>130.1</td><td>586.8051</td><td>1172.609</td><td>-0.0063</td><td>-5.4</td><td>6</td><td>61018.6</td><td>9.44</td><td>HUMAN</td><td>B4DLW8</td><td>Probable ATP-dependent RNA helicase DDX5</td></tr>
<tr><td>3149</td><td>dirMS_041712_NAR_30mM_plus2.8337.8689.2</td><td>2</td><td>14.83</td><td>0</td><td>1.28E+07</td><td></td><td>(K)VIDDTNI</td><td>(K)V/I|D|D/T/N I/T/R(L)</td><td></td><td>99.22</td><td>523.7794</td><td>1046.548</td><td>0.0039</td><td>3.7</td><td>4.21</td><td>43773.3</td><td>5.26</td><td>HUMAN</td><td>F8VZY9</td><td>Keratin, type I cytoskeletal 18</td></tr>
<tr><td>3150</td><td>dirMS_041712_NAR_60mM_plus.12291.12346.3</td><td>3</td><td>14.83</td><td>1</td><td>1.09E+06</td><td></td><td>(R)VCEEIAII</td><td>(R)V/C|E E I A|I|I/P/S K K(L)</td><td>C:Carbamidomethylation</td><td>121.32</td><td>462.9253</td><td>1386.766</td><td>-0.0048</td><td>-3.4</td><td>6.11</td><td>21927.8</td><td>9.55</td><td>HUMAN</td><td>H0YK46</td><td>40S ribosomal protein S17 (Fragment)</td></tr>
<tr><td>3151</td><td>dirMS_041712_NAR_30mM_minus2.12540.12540.2</td><td>2</td><td>14.82</td><td>0</td><td>7.79E+05</td><td></td><td>(R)GVVGPE</td><td>(R)G V/V/G/P/E L/Y/G/R(L)</td><td></td><td>122.52</td><td>523.7834</td><td>1046.563</td><td>-0.0034</td><td>-3.2</td><td>6</td><td>266161</td><td>5.68</td><td>HUMAN</td><td>E9PPU0</td><td>Epiplakin</td></tr>
<tr><td>3152</td><td>dirMS_041712_NAR_30mM_minus2.12607.12708.2</td><td>2</td><td>14.82</td><td>1</td><td>3.06E+06</td><td></td><td>(K)KITIADC</td><td>(K)K\I\T I|A|D\C\G\Q|L|E(-)</td><td>C:Carbamidomethylation</td><td>123.18</td><td>624.3176</td><td>1247.63</td><td>-0.0021</td><td>-1.7</td><td>4.75</td><td>18240.4</td><td>7.96</td><td>HUMAN</td><td>P62937</td><td>Peptidyl-prolyl cis-trans isomerase A</td></tr>
<tr><td>3153</td><td>dirMS_041712_NAR_40mM_minus2.14124.14137.3</td><td>3</td><td>14.82</td><td>0</td><td>6.82E+05</td><td></td><td>(R)IHFPLAT</td><td>(R)I H F\P L A\T|Y/A/P/V/I/S A E K(A)</td><td></td><td>167.8</td><td>586.3246</td><td>1756.963</td><td>-0.004</td><td>-2.3</td><td>6.75</td><td>49012.3</td><td>4.9</td><td>HUMAN</td><td>A8MUB1</td><td>Tubulin alpha-4A chain</td></tr>
<tr><td>3154</td><td>dirMS_041712_NAR_60mM_minus.11879.11879.2</td><td>2</td><td>14.82</td><td>1</td><td>3.16E+05</td><td></td><td>(K)TIDDLEC</td><td>(K)T/I/D/D|L/E D K L/K(C)</td><td></td><td>126.35</td><td>595.3185</td><td>1189.631</td><td>-0.0013</td><td>-1.1</td><td>4.22</td><td>33450.2</td><td>4.73</td><td>HUMAN</td><td>J3KN67</td><td>Tropomyosin alpha-3 chain</td></tr>
<tr><td>3155</td><td>dirMS_041712_NAR_30mM_plus2.16661.16936.2</td><td>2</td><td>14.82</td><td>0</td><td>2.37E+05</td><td></td><td>(R)EIGYPVI</td><td>(R)E I|G Y/P/V/M I/K(A)</td><td></td><td>141.15</td><td>525.2819</td><td>1049.57</td><td>-0.0135</td><td>-12.8</td><td>6.1</td><td>80685.1</td><td>7.31</td><td>HUMAN</td><td>P05165</td><td>Propionyl-CoA carboxylase alpha chain, mitochondrial</td></tr>
<tr><td>3156</td><td>dirMS_041712_NAR_40mM_plus2.7903.7903.2</td><td>2</td><td>14.82</td><td>0</td><td>1.43E+05</td><td></td><td>(K)ASLENSI</td><td>(K)A/S|L/E/N S L R(E)</td><td></td><td>106.82</td><td>445.2395</td><td>889.474</td><td>-0.002</td><td>-2.3</td><td>6.05</td><td>43773.3</td><td>5.26</td><td>HUMAN</td><td>F8VZY9</td><td>Keratin, type I cytoskeletal 18</td></tr>
</table>

3157	dirMS_041712_NAR_50mM_plus2.12261.12261.3	3	14.82	0	3.98E+05		(K)IVLEDGT	(K)I/V|L/E/D G T L|H V T E G S G R(Y)		132.23	561.6272	1682.871	-0.0037	-2.2	4.65	62748.9	5.95	HUMAN	Q16555	Dihydropyrimidinase-related protein 2
3158	dirMS_041712_NAR_50mM_plus2.13475.13475.3	3	14.82	0	2.57E+06		(K)AGVVGF	(K)A G/V/V/G/P/E/L H E Q L L S A E K(A)		138.58	592.986	1776.949	-0.0056	-3.1	4.75	533778	5.74	HUMAN	Q15149	Plectin
3159	dirMS_041712_NAR_30mM_minus2.16239.16708.2	2	14.81	0	7.48E+05		(K)FFVGGN	(K)F/F|V|G/G N/W/K(M)		143.3	477.745	954.483	-0.0005	-0.5	8.75	31075.7	5.65	HUMAN	P60174	Triosephosphate isomerase
3160	dirMS_041712_NAR_50mM_minus2.6900.6900.3	3	14.81	0	5.92E+05		(R)EATNPP	(R)E A T N/P/P V/I Q/E E K/P K(K)		105.52	527.2846	1579.833	0.0066	4.2	4.79	55361.8	6.42	HUMAN	G5EA52	Protein disulfide isomerase family A, member 3, isoform CRA_b
3161	dirMS_041712_NAR_50mM_minus2.9355.9406.3	3	14.81	0	2.72E+05		(K)TGVAVN	(K)T/G\V|A/V/N/K/P A E F T V D/A/K(H)		122.03	549.6316	1646.875	0.0054	3.3	5.73	283472.4	5.71	HUMAN	P21333	Filamin-A
3162	dirMS_041712_NAR_60mM_minus.18440.18440.3	3	14.81	1	1.29E+05		(R)RFDEILE	(R)R F D\E\I\L\E\A|S/D/G|I|M/V/A R(G)		164.42	607.9739	1821.916	-0.0092	-5.1	4.32	18466.1	5.78	HUMAN	H3BSU3	Pyruvate kinase (Fragment)
3163	dirMS_041712_NAR_120mM_plus.12180.12222.4	4	14.81	1	2.72E+06		(K)TLTTVQ	(K)T L\T T V/Q/G I/A/D/D Y D K K/K(L)		117.32	449.7412	1795.944	-0.0006	-0.4	5.71	13778.5	7.96	HUMAN	K7EM18	Eukaryotic translation initiation factor 1
3164	dirMS_041712_NAR_30mM_plus2.20747.20747.2	2	14.81	0	2.72E+05		(K)GGIVGM	(K)G G I|V/G/M/T/L/P I A/R(D)		162.32	592.8453	1184.682	0.0013	1.1	9.75	27150.8	7.95	HUMAN	Q99714	3-hydroxyacyl-CoA dehydrogenase type-2
3165	dirMS_041712_NAR_40mM_plus2.11950.12007.3	3	14.81	1	2.28E+05		(R)VVDALG	(R)V/V|D A/L/G/N A I/D/G K G P I G S K(T)		137.08	570.9838	1710.939	-0.0016	-1	5.93	15338.4	5.51	HUMAN	K7EJP1	ATP synthase subunit alpha, mitochondrial (Fragment)
3166	dirMS_041712_NAR_40mM_plus2.8169.8486.2	2	14.81	0	1.55E+06		(R)LASYLDF	(R)L/A|S|Y L/D/R(V)		109.68	419.2273	837.447	0.0008	1	5.83	43773.3	5.26	HUMAN	F8VZY9	Keratin, type I cytoskeletal 18
3167	dirMS_041712_NAR_50mM_plus2.12660.12660.3	3	14.81	1	3.83E+05		(K)LPGGELI	(K)L/P G G E L/N|P/G E/D/E V E/G L K/R(L)		134.28	636.9936	1908.966	0.0001	0.1	4.25	17283.8	5.95	HUMAN	H3BND3	Cleavage and polyadenylation-specificity factor subunit 5 (Fragment)
3168	dirMS_041712_NAR_50mM_plus2.15042.15042.3	3	14.81	0	4.96E+05		(K)TTISVAH	(K)T T|I/S V A/H/L/L A A/R(Q)		146.45	418.2499	1252.737	-0.002	-1.6	9.44	34143.2	10.07	HUMAN	B4DT23	Protein FAM98A
3169	dirMS_041712_NAR_40mM_minus2.14420.14420.3	3	14.8	0	2.72E+05		(R)DVCTELI	(R)D V|C/T/E L L/P L I K P Q G R(V)	C:Carbamidomethylation	168.95	580.3211	1738.952	-0.0033	-1.9	6.07	30659.7	9.02	HUMAN	P16152	Carbonyl reductase [NADPH] 1
3170	dirMS_041712_NAR_50mM_minus2.16691.16814.3	3	14.8	1	1.60E+05		(K)NIPIPTLI	(K)N I/P I/P/T/L K D/F A/K(A)		163.38	452.9385	1356.789	0.0124	9.1	8.59	20960.6	7.05	HUMAN	H0YKU1	Tropomodulin-3 (Fragment)
3171	dirMS_041712_NAR_50mM_minus2.9045.9045.3	3	14.8	0	7.04E+04		(R)EANALA	(R)E A N/A/L/A/A G H/P A Q/A V A/I N A R(L)		119.77	615.6611	1844.973	-0.0038	-2.1	6.85	272689.3	5.79	HUMAN	O15020	Spectrin beta chain, non-erythrocytic 2
3172	dirMS_041712_NAR_60mM_minus.15007.15365.3	3	14.8	0	5.04E+06		(R)EHALLA	(R)E H\A\L\L|A|Y/T/L/G V/K(Q)		144.68	438.9202	1314.742	0.0044	3.4	6.85	50482.3	9.31	HUMAN	P68104	Elongation factor 1-alpha 1
3173	dirMS_041712_NAR_20mM_plus.16346.16346.2	2	14.8	0	1.40E+05		(R)QAQEYE	(R)Q A/Q E|Y/E/A/L/L N I K(V)		184.52	710.3754	1419.748	-0.0043	-3	4.53	43773.3	5.26	HUMAN	F8VZY9	Keratin, type I cytoskeletal 18
3174	dirMS_041712_NAR_30mM_plus2.22091.22091.2	2	14.8	0	1.92E+05		(K)NSSYFVE	(K)N S/S Y/F/V|E W/I/P N/N V K(V)		171.77	848.9189	1696.833	-0.0024	-1.4	6	42831.5	4.86	HUMAN	A8K854	HCG1983504, isoform CRA_f
3175	dirMS_041712_NAR_30mM_minus2.10052.10072.2	2	14.79	0	1.12E+05		(K)CLDAVV	(K)C/L|D|A|V/V S T/R(H)	C:Carbamidomethylation	108.93	510.7635	1020.514	0.0055	5.4	5.84	50425	5.53	HUMAN	H0YH27	Cullin-associated NEDD8-dissociated protein 1 (Fragment)
3176	dirMS_041712_NAR_30mM_minus2.9763.9763.2	2	14.79	0	6.20E+04		(R)ELSEQLC	(R)E L/S E Q/L/G/Q/A E/R(A)		107.38	630.312	1259.623	-0.0059	-4.7	4.25	266161	5.68	HUMAN	E9PPU0	Epiplakin
3177	dirMS_041712_NAR_60mM_minus.15439.15439.3	3	14.79	1	1.77E+05		(R)ASLISAV	(R)A/S/L/I/S/A V/S/D K L/R(W)		146.23	420.5811	1259.732	-0.003	-2.4	8.79	41088.1	5.18	HUMAN	F5H442	Tumor susceptibility gene 101 protein
3178	dirMS_041712_NAR_30mM_plus2.10824.10824.2	2	14.79	0	5.58E+05		(R)LASFYER	(R)L/A|S|F/Y/E/R(A)		110.48	443.2301	885.447	0.0064	7.3	6	65077.2	5.47	HUMAN	B7Z1R5	V-type proton ATPase catalytic subunit A
3179	dirMS_041712_NAR_30mM_plus2.11559.11559.2	2	14.79	0	1.26E+06		(R)GGGGNI	(R)G G/G G N|F|G|P G/P G S N/F R(G)		114.38	689.3175	1377.629	-0.0017	-1.2	9.75	37486.1	9.03	HUMAN	P22626	Heterogeneous nuclear ribonucleoproteins A2/B1
3180	dirMS_041712_NAR_30mM_plus2.17754.17754.2	2	14.79	0	3.10E+05		(K)SLEDQV	(K)S L|E/D/Q/V/E M L R(T)		145.88	610.3003	1219.599	-0.0054	-4.4	4.14	61161.1	4.34	HUMAN	K7ELL7	Glucosidase 2 subunit beta
3181	dirMS_041712_NAR_40mM_plus2.14271.14271.3	3	14.79	1	7.99E+05		(R)IINEPTA	(R)I I|N E|P T A/A A I\A Y G L D K K(V)		149.77	596.6671	1787.99	-0.0034	-1.9	6.07	17973	5.16	HUMAN	E9PI65	Heat shock cognate 71 kDa protein (Fragment)
3182	dirMS_041712_NAR_120mM_minus.6097.6097.3	3	14.78	1	5.64E+05		(K)TCEESSF	(K)T/C/E/E/S/S/F C/K/R(Q)	C:Carbamidomethylation	86.8	435.1849	1303.541	-0.0004	-0.3	5.81	10576.1	11.44	HUMAN	E9PRF8	Neutral alpha-glucosidase AB
3183	dirMS_041712_NAR_60mM_minus.14088.14088.3	3	14.78	0	3.65E+05		(K)QLPLVKI	(K)Q L/P L/V/K/P Y L R(S)		138.22	409.59	1226.762	-0.0065	-5.3	9.99	71165.3	5.57	HUMAN	J3KS13	Clathrin heavy chain 1
3184	dirMS_041712_NAR_60mM_minus.4806.4806.3	3	14.78	1	7.13E+05	M137m	(R)mQNDT.	(R)m Q|N/D T A/E/N E/T T E K/E|E K(S)	m:Oxidized methionine	79.37	638.2735	1896.813	15.9932	-0.9	4.14	54214.9	5.66	HUMAN	C9J813	Caldesmon (Fragment)
3185	dirMS_041712_NAR_60mM_minus.9088.9088.2	2	14.78	0	9.67E+05		(K)QEYDES	(K)Q E|Y/D/E/S/G/P S I/V/H/R(K)		110.35	758.8539	1516.703	-0.0021	-1.4	4.65	123097.1	5.83	HUMAN	A5A3E0	POTE ankyrin domain family member F
3186	dirMS_041712_NAR_30mM_plus2.13638.13820.2	2	14.78	0	1.91E+06		(K)VLEGME	(K)V/L|E|G M/E V/V/R(K)		125.05	516.2816	1031.555	0.0005	0.5	4.53	23799.1	9.48	HUMAN	P23284	Peptidyl-prolyl cis-trans isomerase B
3187	dirMS_041712_NAR_40mM_plus2.11313.11313.3	3	14.78	0	1.67E+05		(R)SVIDPVF	(R)S V/I/D/P\V/P A/P V G D S H V D G A A K(S)		132.95	644.3327	1930.987	-0.0033	-1.7	4.41	58326.8	5.7	HUMAN	Q13177	Serine/threonine-protein kinase PAK 2
3188	dirMS_041712_NAR_50mM_plus2.20884.20884.3	3	14.78	0	3.32E+06		(R)AGKPVI(	(R)A G K\P V I C A|T/Q/M/L/E/S M/I/K(K)	C:Carbamidomethylation	179.12	626.3278	1876.969	-0.0005	-0.3	8.64	18466.1	5.78	HUMAN	H3BSU3	Pyruvate kinase (Fragment)
3189	dirMS_041712_NAR_50mM_plus2.22294.22294.3	3	14.78	0	1.53E+06		(R)DALSDL	(R)D A/L/S D L A/L/H F L N K(M)		191.78	486.2644	1456.78	-0.0008	-0.6	5.21	42755	7.81	HUMAN	B7Z2F4	T-complex protein 1 subunit delta
3190	dirMS_041712_NAR_120mM_minus.21420.21434.3	3	14.77	0	8.12E+05		(R)LHFFMP	(R)L H F F M|P G\F|A|P\L/T S R(G)		182.17	540.9497	1620.835	-0.001	-0.6	9.75	42230.8	4.77	HUMAN	A6NKZ8	Putative tubulin beta chain-like protein ENSP00000290377
3191	dirMS_041712_NAR_20mM_minus.7303.7316.2	2	14.77	0	1.32E+05	M374m	(R)mAPTPII	(R)m|A|P/T|P I/P T R(S)	m:Oxidized methionine	121.02	500.2679	983.534	15.9943	-0.7	9.5	76925.9	4.46	HUMAN	A8MV58	Drebrin
3192	dirMS_041712_NAR_40mM_minus2.15777.15829.3	3	14.77	0	7.17E+05		(K)GLAPDLI	(K)G L|A/P/D/L/P/E D L Y/H L I K(K)		184.48	565.3111	1693.916	0.0028	1.7	4.54	16789.5	10.63	HUMAN	J3KMX5	40S ribosomal protein S13
3193	dirMS_041712_NAR_50mM_minus2.10374.10374.3	3	14.77	0	1.46E+05		(K)VGSLDN	(K)V/G|S/L D N/V G H L/P A/G G A\V K(I)		126.9	530.9592	1590.86	0.0032	2	6.71	26233.5	10.95	HUMAN	B4DSQ1	Microtubule-associated protein
3194	dirMS_041712_NAR_50mM_minus2.14561.14623.3	3	14.77	1	6.71E+05		(K)VALNVS	(K)V/A|L|N|V/S/C A N L L D K D/I G S K(S)	C:Carbamidomethylation	151.22	639.6751	1917.011	-0.0002	-0.1	5.93	9239.5	7.98	HUMAN	E5RFT7	Copine-3 (Fragment)
3195	dirMS_041712_NAR_40mM_plus2.17503.17608.3	3	14.77	1	3.17E+05		(K)TGISDVF	(K)T G/I/S D V|F A/K N D|L/A|V/V D/V R(I)		169.33	640.3451	1919.023	-0.0025	-1.3	4.43	76670.1	4.6	HUMAN	P19338	Nucleolin
3196	dirMS_041712_NAR_50mM_plus2.14419.14492.3	3	14.77	1	1.75E+06		(K)LTEDKAI	(K)L/T|E D K A D V\Q/S|I/I|G/L Q R(F)		143.9	595.9941	1785.97	-0.0028	-1.5	4.56	90316.8	5.52	HUMAN	Q13263	Transcription intermediary factor 1-beta
3197	dirMS_041712_NAR_60mM_plus.7500.7500.3	3	14.77	1	5.05E+05		(K)SSGGREI	(K)S S|G G R E D\L|E/S\S/G L/Q|R(R)		97.3	526.5897	1577.751	0.0031	2	4.68	11625.5	8.01	HUMAN	B8ZZT7	Hematological and neurological-expressed 1 protein
3198	dirMS_041712_NAR_30mM_minus2.18136.18136.2	2	14.76	0	1.03E+05		(K)LLCGLLA	(K)L L|C|G/L L/A/E R(L)	C:Carbamidomethylation	152.35	522.7963	1044.587	-0.0017	-1.6	6	12647.2	8.07	HUMAN	P14174	Macrophage migration inhibitory factor
3199	dirMS_041712_NAR_40mM_minus2.14846.14846.3	3	14.76	1	1.64E+04		(R)GDLGIEI	(R)G D/L/G\I/E I/P A E K V F L A Q K(M)		172.22	610.0109	1828.022	-0.0033	-1.8	4.68	18466.1	5.78	HUMAN	H3BSU3	Pyruvate kinase (Fragment)
3200	dirMS_041712_NAR_40mM_minus2.9403.9404.3	3	14.76	0	1.42E+05		(K)QLTEED(	(K)Q L/T E/E/D/G V/H/S V/I E E N\I K(C)		137.88	647.3235	1939.961	-0.0048	-2.5	4.25	54427.4	5.96	HUMAN	K4DID9	Acetyl-CoA carboxylase 1
3201	dirMS_041712_NAR_50mM_minus2.11448.11471.3	3	14.76	0	1.45E+05		(K)VSQPIEC	(K)V/S\Q/P I/E G H A/A/S F A Q/F/K(M)		133.52	572.9609	1716.87	-0.0022	-1.3	6.72	193379.8	5.48	HUMAN	Q00610	Clathrin heavy chain 1
3202	dirMS_041712_NAR_120mM_plus.5479.5480.3	3	14.76	1	6.39E+05		(R)YYGGGS	(R)Y/Y|G/G G/S|E|G G R A P K(R)		81.9	433.5423	1298.612	0	0	8.5	64759.2	8.88	HUMAN	P14866	Heterogeneous nuclear ribonucleoprotein L
3203	dirMS_041712_NAR_50mM_plus2.12894.12894.3	3	14.76	1	3.37E+05		(R)ELAVQIY	(R)E L/A/V/Q/I/Y/E E A R K(F)		135.53	483.5916	1448.774	-0.0141	-9.7	4.79	73386.6	8.73	HUMAN	B4DXX7	ATP-dependent RNA helicase DDX3Y
3204	dirMS_041712_NAR_50mM_plus2.19278.19349.3	3	14.76	0	3.59E+05		(-)MKPLVV	(-)M K P L V V\F|V|L/G G/P G A G K(G)		169.73	523.9715	1569.919	-0.0186	-11.8	10	22450.2	5.44	HUMAN	P30085	UMP-CMP kinase
3205	dirMS_041712_NAR_60mM_plus.15641.15663.3	3	14.76	1	3.43E+06		(R)TEMENE	(R)T\E|M/E/N E/F/V/L I/K K(D)		138.23	494.2622	1480.772	0.0005	0.3	4.78	13588.1	4.66	HUMAN	F8VP67	Keratin, type II cytoskeletal 8 (Fragment)
3206	dirMS_041712_NAR_20mM_minus.14688.14688.3	3	14.75	0	3.68E+05		(K)LAVEALS	(K)L A V\E A\L|S S/L D/G D/L/A/G R(Y)		201.3	529.6163	1586.838	-0.0041	-2.6	4.03	24286.7	4.89	HUMAN	G3V4N7	Creatine kinase B-type (Fragment)
3207	dirMS_041712_NAR_50mM_minus2.12962.12962.3	3	14.75	1	3.89E+05		(R)LSEDYG	(R)L/S/E/D/Y/G V L K T/D E G I A Y R(G)		141.73	643.6561	1928.96	-0.0063	-3.2	4.32	22062.7	5.66	HUMAN	P32119	Peroxiredoxin-2
3208	dirMS_041712_NAR_50mM_minus2.15088.15107.3	3	14.75	1	2.54E+05		(K)LALDIEI	(K)L/A|L D/I E/I/A/T Y R/K(L)		154.13	469.2729	1405.805	-0.0008	-0.6	6.07	49564	5.84	HUMAN	E9PAX3	Glial fibrillary acidic protein
3209	dirMS_041712_NAR_50mM_minus2.15737.15737.3	3	14.75	1	4.93E+05		(K)QGGLGF	(K)Q G\G L/G/P M N I/P L V S D/P K R(T)		157.5	593.6566	1778.958	-0.0029	-1.6	8.75	22338.2	8.65	HUMAN	Q06830	Peroxiredoxin-1
3210	dirMS_041712_NAR_60mM_minus.11172.11400.3	3	14.75	1	5.95E+05	M136m	(K)VKEGmN	(K)V/K\E G m N\I|V|E A/M/E/R(F)	m:Oxidized methionine	122.87	507.9181	1505.745	15.9947	-0.1	4.79	18240.4	7.96	HUMAN	P62937	Peptidyl-prolyl cis-trans isomerase A
3211	dirMS_041712_NAR_60mM_minus.12310.12310.3	3	14.75	1	4.35E+05		(R)INVYYNI	(R)I N/V|Y/Y\N E/A T/G G K Y/V P R(A)		127.72	615.3145	1843.934	-0.0048	-2.6	8.43	50286.6	4.79	HUMAN	P68371	Tubulin beta-4B chain
3212	dirMS_041712_NAR_20mM_plus.17079.17079.2	2	14.75	0	1.91E+05		(K)VQIAVA	(K)V Q I/A/V/A N/A/Q E/L\L/Q R(M)		188.3	776.9419	1552.881	-0.004	-2.6	5.97	10728.1	9.15	HUMAN	Q9Y5L4	Mitochondrial import inner membrane translocase subunit Tim13
3213	dirMS_041712_NAR_30mM_plus2.13495.13534.2	2	14.75	0	1.03E+05		(R)TIYAGN	(R)T/I|Y|A/G N|A|L/C T V K(C)	C:Carbamidomethylation	123.85	655.8414	1310.677	-0.0018	-1.4	8.26	24273.5	8.98	HUMAN	H0YK49	Electron transfer flavoprotein subunit alpha, mitochondrial
3214	dirMS_041712_NAR_30mM_plus2.8714.8845.3	3	14.75	0	1.43E+06		(R)FGQGG	(R)F G\Q G G A G|P|V/G/G/Q/G P/R(G)		99.68	447.893	1341.666	-0.0014	-1	9.75	76262.2	9.53	HUMAN	P23246	Splicing factor, proline- and glutamine-rich
3215	dirMS_041712_NAR_40mM_plus2.17751.17751.3	3	14.75	0	3.79E+04		(R)AGLGSG	(R)A G L/G/S/G/L/S L/S/G L V/H P/E L S R(S)		170.98	617.3431	1850.013	0.0017	0.9	6.79	73325.2	9.19	HUMAN	F5H2A9	Very long-chain-specific acyl-CoA dehydrogenase, mitochondrial
3216	dirMS_041712_NAR_50mM_plus2.18656.19187.3	3	14.75	1	6.52E+06		(R)LDLAGR	(R)L/D|L|A/G R D L/T D/Y L/M/K(I)		167.65	541.9524	1623.841	0.0016	1	4.43	37482.1	5.36	HUMAN	E9PG30	Actin, gamma-enteric smooth muscle
3217	dirMS_041712_NAR_60mM_plus.11480.11497.3	3	14.75	0	1.11E+06		(R)SIFQHIQ	(R)S I|F Q/H/I/Q S/A Q/S Q R(S)		117.93	510.6056	1529.782	0.0203	13.3	9.49	108720.9	10.17	HUMAN	Q9Y2W1	Thyroid hormone receptor-associated protein 3
3218	dirMS_041712_NAR_60mM_plus.15420.15420.3	3	14.75	1	3.85E+05		(R)SLIANLA	(R)S L/I/A/N/L/A A A\N C Y/K K(E)	C:Carbamidomethylation	137.43	512.9411	1536.82	-0.0115	-7.5	9.7	36978.6	6.33	HUMAN	B7Z800	Adenosine kinase
3219	dirMS_041712_NAR_60mM_plus.5566.5587.3	3	14.75	0	6.14E+05	M434m M	(R)LESGmC	(R)L/E|S G m Q N m/S I/H/T/K(T)	m:Oxidized methionine	87.57	503.2333	1475.698	31.9872	-1.7	6.75	56607.4	5.37	HUMAN	F8VXB4	Keratin, type II cytoskeletal 8
3220	dirMS_041712_NAR_30mM_minus2.16082.16082.2	2	14.74	0	2.79E+05		(R)GEVTAA	(R)G E|V/T/A/A/D L/F N S R(V)		141.65	640.3185	1279.628	0.002	1.6	4.37	266161	5.68	HUMAN	E9PPU0	Epiplakin
3221	dirMS_041712_NAR_30mM_minus2.20017.20077.2	2	14.74	0	2.44E+04		(K)SLNILTA	(K)S L|N|I|L/T/A/F Q/K(K)		168.82	567.8283	1134.652	-0.0024	-2.1	8.47	18172.6	7.77	HUMAN	F8VY02	Endoplasmic reticulum resident protein 29
3222	dirMS_041712_NAR_50mM_minus2.10229.10329.3	3	14.74	0	1.46E+05		(K)VGSLDN	(K)V/G|S/L/D N/V G H\L/P A G G A V/K(I)		126.9	530.9592	1590.86	0.0032	2	6.71	26233.5	10.95	HUMAN	B4DSQ1	Microtubule-associated protein
3223	dirMS_041712_NAR_60mM_minus.11899.11899.2	2	14.74	1	3.16E+05		(K)TIDDLEC	(K)T I|D|D/L E D K L/K(C)		126.35	595.3185	1189.631	-0.0013	-1.1	4.22	33450.2	4.73	HUMAN	J3KN67	Tropomyosin alpha-3 chain
3224	dirMS_041712_NAR_120mM_plus.11653.11989.3	3	14.74	1	1.18E+06		(K)MKEIAE	(K)M/K E I A|E|A/Y/L/G/K(T)		114.3	418.2253	1252.661	0.0008	0.6	5.9	17973	5.16	HUMAN	E9PI65	Heat shock cognate 71 kDa protein (Fragment)
3225	dirMS_041712_NAR_120mM_plus.15275.15300.3	3	14.74	1	5.50E+05		(K)KKELEEI	(K)K\K E L\E E\I|V|Q/P I/I/S K(L)		133.28	551.998	1653.978	0.0009	0.5	6.23	72445.7	5.07	HUMAN	P11021	78 kDa glucose-regulated protein
3226	dirMS_041712_NAR_20mM_plus.10906.10906.2	2	14.74	0	2.19E+05	M33m	(K)VAGmD	(K)V A/G\m D|V|E/L/T V/E/E R(N)	m:Oxidized methionine	147.28	732.3555	1447.71	15.994	-0.6	4	10949.3	5.28	HUMAN	B4DJF2	14-3-3 protein epsilon
3227	dirMS_041712_NAR_20mM_plus.11106.11106.2	2	14.74	0	9.73E+03		(R)TIYAGN	(R)T I|Y/A/G N/A\L C T V K(C)	C:Carbamidomethylation	148.83	655.8428	1310.677	0.001	0.8	8.26	24273.5	8.98	HUMAN	H0YK49	Electron transfer flavoprotein subunit alpha, mitochondrial
3228	dirMS_041712_NAR_20mM_plus.18015.18015.2	2	14.74	0	1.71E+05		(R)SLVPAAI	(R)S L\V|P A/A/E|L L E S R(V)		195.18	642.8613	1284.716	-0.0005	-0.4	4.53	533778	5.74	HUMAN	Q15149	Plectin
3229	dirMS_041712_NAR_20mM_plus.7425.7472.2	2	14.74	0	2.40E+05		(K)VTLTSEE	(K)V/T|L/T|S/E E E A R(L)		119.92	567.7865	1134.564	0.002	1.8	4.25	33940.9	9.27	HUMAN	F5H308	L-lactate dehydrogenase
3230	dirMS_041712_NAR_30mM_plus2.12710.12710.2	2	14.74	0	3.16E+05		(K)LVPVLS	(K)L V/P/V/L S\A K(A)		119.62	413.7745	826.54	0.0021	2.5	8.75	37710.6	6.36	HUMAN	P37837	Transaldolase
3231	dirMS_041712_NAR_30mM_plus2.7070.7269.2	2	14.74	0	6.70E+05		(K)AQCPIVI	(K)A/Q|C|P/I V/E/R(L)	C:Carbamidomethylation	92.52	486.7542	972.493	0.008	8.2	6.05	23047.2	9.95	HUMAN	P46782	40S ribosomal protein S5
3232	dirMS_041712_NAR_30mM_plus2.9521.9521.2	2	14.74	0	6.85E+05		(R)GGGQIII	(R)G G/G Q|I|I|P T A/R(R)		104.17	485.2772	969.548	-0.0005	-0.5	9.75	15036.9	7.16	HUMAN	K7EJ74	116 kDa U5 small nuclear ribonucleoprotein component (Fragment)
3233	dirMS_041712_NAR_40mM_plus2.7198.7480.2	2	14.74	0	3.09E+05		(K)VIDDTNI	(K)V/I|D|D|T/N I/T/R(L)		103.53	523.7788	1046.548	0.0027	2.5	4.21	43773.3	5.26	HUMAN	F8VZY9	Keratin, type I cytoskeletal 18
3234	dirMS_041712_NAR_50mM_plus2.21443.21443.3	3	14.74	1	1.51E+05		(K)ALFEEVF	(K)A L/F/E/E/V/P E L L T E A E K/K(E)		182.68	615.9987	1845.984	-0.0029	-1.6	4.32	65036	6.27	HUMAN	E9PBU3	Phosphoribosylaminoimidazolecarboxamide formyltransferase
3235	dirMS_041712_NAR_60mM_plus.15619.15619.2	2	14.74	1	4.11E+05		(R)TEMENE	(R)T/E|M|E/N E F V/L I/K K(D)		138.27	740.8875	1480.772	-0.0039	-2.6	4.78	13588.1	4.66	HUMAN	F8VP67	Keratin, type II cytoskeletal 8 (Fragment)
3236	dirMS_041712_NAR_60mM_minus.13632.13633.3	3	14.73	1	1.80E+05		(K)QMADT	(K)Q/M/A/D/T G/K/L/N/T L/L Q R(A)		135.92	530.2846	1588.848	-0.0083	-5.2	8.75	91324.5	5.75	HUMAN	Q00839	Heterogeneous nuclear ribonucleoprotein U
3237	dirMS_041712_NAR_120mM_plus.10981.11111.3	3	14.73	1	5.81E+05		(K)VIADNV	(K)V/I/A/D/N V/K D W/S/K(V)		110.67	425.5621	1274.674	-0.0022	-1.7	5.93	31075.7	5.65	HUMAN	P60174	Triosephosphate isomerase
3238	dirMS_041712_NAR_120mM_plus.14419.14917.4	4	14.73	1	2.10E+06		(K)GLDPAR	(K)G L\D\P A R V N V/P/V/I/G G H A G K(T)		130.67	439.9998	1756.982	-0.0043	-2.5	8.76	24936.4	8.67	HUMAN	G3XAL0	Malate dehydrogenase
3239	dirMS_041712_NAR_20mM_plus.18857.18857.2	2	14.73	0	2.72E+05		(K)LAVEALS	(K)L A/V/E|A/L|S/S L/D G D/L A/G R(Y)		200.53	793.9214	1586.838	-0.0029	-1.8	4.03	24286.7	4.89	HUMAN	G3V4N7	Creatine kinase B-type (Fragment)
3240	dirMS_041712_NAR_40mM_plus2.14948.14948.3	3	14.73	0	6.60E+04		(R)QAQEYE	(R)Q A Q E\Y\E/A|L|L N/I K(V)		154.73	473.9204	1419.748	-0.0012	-0.8	4.53	43773.3	5.26	HUMAN	F8VZY9	Keratin, type I cytoskeletal 18
3241	dirMS_041712_NAR_40mM_plus2.19355.19355.3	3	14.73	0	1.61E+06		(R)AGKPVI(	(R)A G K\P V I\C A\T/Q/M/L/E S M I K(K)	C:Carbamidomethylation	180.63	626.3273	1876.969	-0.002	-1.1	8.64	18466.1	5.78	HUMAN	H3BSU3	Pyruvate kinase (Fragment)
3242	dirMS_041712_NAR_50mM_plus2.14176.14176.3	3	14.73	0	5.08E+05		(K)VTHAVV	(K)V T H A V V/T V|P A/Y/F/N/D/A Q/R(Q)		142.7	629.9955	1887.971	0.0008	0.4	6.71	72445.7	5.07	HUMAN	P11021	78 kDa glucose-regulated protein
3243	dirMS_041712_NAR_50mM_plus2.8776.8776.3	3	14.73	0	1.55E+06		(R)LESGMC	(R)L E/S G M Q/N M/S/I/H T K(T)		113.23	492.5704	1475.698	-0.0015	-1	6.75	56607.4	5.37	HUMAN	F8VXB4	Keratin, type II cytoskeletal 8
3244	dirMS_041712_NAR_60mM_plus.10282.10282.3	3	14.73	0	2.57E+05		(K)TVFAEHI	(K)T/V|F A E/H I/S D E C K(R)	C:Carbamidomethylation	111.73	479.2229	1435.652	0.0019	1.4	4.65	31471	10.11	HUMAN	B5MCW2	60S ribosomal protein L3 (Fragment)
3245	dirMS_041712_NAR_30mM_minus2.12624.13069.2	2	14.72	1	3.16E+06		(K)KITIADC	(K)K\I T I|A|D\C|G Q|L|E(-)	C:Carbamidomethylation	123.22	624.3176	1247.63	-0.0021	-1.7	4.75	18240.4	7.96	HUMAN	P62937	Peptidyl-prolyl cis-trans isomerase A
3246	dirMS_041712_NAR_30mM_minus2.13603.13809.2	2	14.72	0	3.31E+05		(R)ISGLIYEE	(R)I/S|G|L|I|Y/E E/T R(G)		127.68	590.8127	1180.621	-0.0027	-2.3	4.53	11367.1	11.36	HUMAN	P62805	Histone H4
3247	dirMS_041712_NAR_60mM_minus.14177.14177.3	3	14.72	1	2.27E+05		(R)TFNTST(	(R)T F/N/T/S T G G L/L/L/P S D/T K R(S)		138.82	603.3262	1807.955	0.0092	5.1	8.41	64302.5	6.4	HUMAN	B1AHC7	X-ray repair complementing defective repair in Chinese hamster cells 6 (Ku autoantigen, 70kDa)
3248	dirMS_041712_NAR_120mM_plus.14840.14884.3	3	14.72	1	5.29E+05		(R)KLDELYC	(R)K L D E\L|Y/G/T/W/R(K)		131.2	427.5607	1280.663	0.0042	3.3	6.07	37884.5	11.27	HUMAN	H3BM89	60S ribosomal protein L4
3249	dirMS_041712_NAR_30mM_plus2.18570.18570.2	2	14.72	0	2.24E+05		(R)GNPTVE	(R)G N|P T/V|E V D L F/T S K(G)		150.92	703.8613	1406.716	-0.0009	-0.6	4.37	21077	9.05	HUMAN	K7EM90	Alpha-enolase (Fragment)
3250	dirMS_041712_NAR_30mM_plus2.19597.19597.2	2	14.72	0	3.99E+05		(K)LLQDFFI	(K)L/L|Q|D/F F N/G R(D)		156.8	555.2902	1109.574	-0.0007	-0.6	5.84	60126	5.44	HUMAN	E7EP94	Heat shock 70 kDa protein 1A/1B
3251	dirMS_041712_NAR_40mM_plus2.14348.14436.3	3	14.72	1	1.80E+05		(K)IMASSPI	(K)I/M|A/S S/P D|M/D L/A T V S A L R K(M)		151.18	635.9975	1905.977	0.0007	0.4	5.96	36208.1	7.21	HUMAN	B4DMU0	Pyrroline-5-carboxylate reductase
3252	dirMS_041712_NAR_50mM_plus2.18170.18170.3	3	14.72	1	1.09E+05		(K)DLEIKIP	(K)D/L\E/I\K/I/P V V V R(L)		163.5	427.6021	1280.794	-0.0019	-1.5	6.07	44183.5	5.64	HUMAN	F5GXC8	Succinyl-CoA ligase [ADP-forming] subunit beta, mitochondrial
3253	dirMS_041712_NAR_60mM_plus.15308.15554.3	3	14.72	1	7.13E+06		(K)VKEGMI	(K)V K\E G M N\I|V|E/A/M/E/R(F)		137.73	502.5849	1505.745	-0.0049	-3.3	4.79	18240.4	7.96	HUMAN	P62937	Peptidyl-prolyl cis-trans isomerase A
3254	dirMS_041712_NAR_60mM_plus.17677.17677.3	3	14.72	1	4.28E+05		(R)LMDLLG	(R)L/M|D\L/L/G E\G L K/R(S)		149.27	415.5712	1244.703	-0.0041	-3.3	6.07	52817.9	6.2	HUMAN	F5H3T8	Arginine--tRNA ligase, cytoplasmic
3255	dirMS_041712_NAR_50mM_minus2.13410.13410.3	3	14.71	0	1.48E+05		(R)IVATKPL	(R)I V/A T K/P L/Y V/A/L A/Q/R(K)		144.15	514.9821	1542.937	-0.0049	-3.2	9.99	65918.2	9.74	HUMAN	E7EQV3	Polyadenylate-binding protein 1
3256	dirMS_041712_NAR_60mM_minus.10704.10704.3	3	14.71	1	5.44E+05	M239m	(R)MSMKE	(R)M/S\M K E V D E Q|m|L/N/V/Q/N K(N)	m:Oxidized methionine	119.9	647.3011	1923.897	15.9915	-1.8	4.68	40069.4	4.83	HUMAN	E7EWR1	Tubulin beta chain
3257	dirMS_041712_NAR_60mM_minus.18683.18683.3	3	14.71	0	2.97E+05		(K)LHDNLII	(K)L H D N L\I|I/S D/L/E N T V K(K)		165.95	575.3117	1723.922	-0.0019	-1.1	4.54	88624.6	5.09	HUMAN	C9J8U1	Cytospin-A (Fragment)
3258	dirMS_041712_NAR_120mM_plus.12297.12707.3	3	14.71	0	1.51E+06		(R)HVFGES	(R)H V\F G\E|S/D|E|L|I/G Q/K(V)		119.02	486.9124	1458.722	0.0003	0.2	4.65	31075.7	5.65	HUMAN	P60174	Triosephosphate isomerase
3259	dirMS_041712_NAR_20mM_plus.6694.6694.2	2	14.71	0	2.51E+04		(R)AAPTAA	(R)A A/P/T/A/A/S/D/Q/P D S A A T T E K(A)		110.9	866.4036	1731.803	-0.0032	-1.9	4.03	22655.1	9.45	HUMAN	Q9BRP8	Partner of Y14 and mago
3260	dirMS_041712_NAR_30mM_plus2.18989.18989.2	2	14.71	0	2.07E+05		(K)YPQLLP(	(K)Y/P|Q|L/L/P G I R(G)		153.25	528.814	1056.62	0.0007	0.6	8.75	45228.3	6.03	HUMAN	F5H737	Adenosylhomocysteinase
3261	dirMS_041712_NAR_30mM_plus2.8362.8633.2	2	14.71	0	1.11E+06		(R)SLQSVAI	(R)S L|Q|S/V A/E/E R(A)		98.42	509.7627	1018.516	0.0018	1.7	4.53	20623.6	11.46	HUMAN	E7EQV9	Ribosomal protein L15 (Fragment)
3262	dirMS_041712_NAR_30mM_plus2.8560.8702.2	2	14.71	0	3.58E+05		(R)AVVGV	(R)A V|V/G|V|V/A/G\G\G R(I)		99.07	471.2779	941.553	-0.0042	-4.4	9.79	24115.6	11.59	HUMAN	E9PIZ3	60S ribosomal protein L8
3263	dirMS_041712_NAR_40mM_plus2.20060.20060.3	3	14.71	1	1.43E+06		(R)ELAILLG	(R)E L/A I/L/L G/M L D/P/A E K D E K(G)		189.2	629.003	1884.999	-0.0042	-2.3	4.18	25148.6	6	HUMAN	P30041	Peroxiredoxin-6
3264	dirMS_041712_NAR_20mM_plus.9051.9051.2	2	14.7	0	2.63E+05		(R)VLTVINC	(R)V L|T/V|I/N Q T Q K(E)		133.05	572.3399	1143.673	-0.0007	-0.6	8.72	10815.7	10.72	HUMAN	F2Z388	60S ribosomal protein L35
3265	dirMS_041712_NAR_30mM_plus2.20035.20035.2	2	14.7	0	8.31E+04		(R)FEELTNL	(R)F/E|E/L/T/N L/I R(T)		158.62	567.8152	1134.615	0.0078	6.9	4.53	106155.7	5.45	HUMAN	B5ME19	Eukaryotic translation initiation factor 3 subunit C

<table>
<tr><td>3266</td><td>dirMS_041712_NAR_60mM_plus.16618.16618.3</td><td>3</td><td>14.7</td><td>1</td><td>5.49E+05</td><td></td><td>(K)AAVEWI</td><td>(K)A A|V/E/W/F D/G K D F Q G S K(L)</td><td></td><td>143.2</td><td>562.2762</td><td>1684.797</td><td>0.0175</td><td>10.4</td><td>4.56</td><td>65213.5</td><td>9.57</td><td>HUMAN</td><td>B0QYK0</td><td>Ewing sarcoma breakpoint region 1</td></tr>
<tr><td>3267</td><td>dirMS_041712_NAR_120mM_minus.7815.7905.3</td><td>3</td><td>14.69</td><td>1</td><td>5.78E+05</td><td></td><td>(R)LQSIGTE</td><td>(R)L/Q|S|I/G/T E/N T/E E N R R(F)</td><td></td><td>97.25</td><td>549.607</td><td>1646.809</td><td>-0.0028</td><td>-1.7</td><td>4.79</td><td>15633.5</td><td>9.15</td><td>HUMAN</td><td>H3BMQ8</td><td>Fructose-bisphosphate aldolase A (Fragment)</td></tr>
<tr><td>3268</td><td>dirMS_041712_NAR_120mM_minus.8411.8411.3</td><td>3</td><td>14.69</td><td>0</td><td>1.85E+05</td><td></td><td>(K)LFQECCI</td><td>(K)L/F/Q/E C/C/P/H/S T D R(V)</td><td>C:Carbamidomethylation</td><td>99.98</td><td>517.2227</td><td>1549.652</td><td>0.0013</td><td>0.9</td><td>5.32</td><td>51260.6</td><td>5.39</td><td>HUMAN</td><td>P61978</td><td>Heterogeneous nuclear ribonucleoprotein K</td></tr>
<tr><td>3269</td><td>dirMS_041712_NAR_20mM_minus.14668.14668.2</td><td>2</td><td>14.69</td><td>0</td><td>2.10E+05</td><td></td><td>(K)LAVEALS</td><td>(K)L A|V E/A|L|S/S L/D\G D L A G R(Y)</td><td></td><td>201.28</td><td>793.922</td><td>1586.838</td><td>-0.0017</td><td>-1.1</td><td>4.03</td><td>24286.7</td><td>4.89</td><td>HUMAN</td><td>G3V4N7</td><td>Creatine kinase B-type (Fragment)</td></tr>
<tr><td>3270</td><td>dirMS_041712_NAR_30mM_minus2.10487.10487.2</td><td>2</td><td>14.69</td><td>0</td><td>5.97E+05</td><td></td><td>(K)EAAENS</td><td>(K)E A|A/E/N S L V/A/Y/K(A)</td><td></td><td>110.83</td><td>597.8033</td><td>1194.6</td><td>-0.0008</td><td>-0.6</td><td>4.53</td><td>12926.1</td><td>4.4</td><td>HUMAN</td><td>K7EM20</td><td>14-3-3 protein epsilon (Fragment)</td></tr>
<tr><td>3271</td><td>dirMS_041712_NAR_40mM_minus2.10349.10764.3</td><td>3</td><td>14.69</td><td>1</td><td>3.05E+06</td><td></td><td>(K)TITLEVEI</td><td>(K)T I\T|L/E/V/E/P S D T I E N V K A K(I)</td><td></td><td>145.98</td><td>663.0226</td><td>1987.059</td><td>-0.0061</td><td>-3.1</td><td>4.41</td><td>17271.6</td><td>6.75</td><td>HUMAN</td><td>B4DV12</td><td>Ubiquitin</td></tr>
<tr><td>3272</td><td>dirMS_041712_NAR_50mM_minus2.16186.16186.3</td><td>3</td><td>14.69</td><td>0</td><td>1.46E+05</td><td></td><td>(K)LDPHLVI</td><td>(K)L/D/P H/L V/L D/Q|L R(C)</td><td></td><td>160.2</td><td>440.2533</td><td>1318.748</td><td>-0.0024</td><td>-1.8</td><td>5.21</td><td>233493.8</td><td>5.45</td><td>HUMAN</td><td>F8VTL3</td><td>Myosin-10</td></tr>
<tr><td>3273</td><td>dirMS_041712_NAR_120mM_plus.7403.7438.3</td><td>3</td><td>14.69</td><td>2</td><td>2.39E+05</td><td></td><td>(K)VVSETN</td><td>(K)V/V|S E/T N D T/K/V/L R/H(-)</td><td></td><td>92.38</td><td>499.9371</td><td>1497.802</td><td>-0.0052</td><td>-3.5</td><td>6.72</td><td>48057</td><td>5.34</td><td>HUMAN</td><td>P05783</td><td>Keratin, type I cytoskeletal 18</td></tr>
<tr><td>3274</td><td>dirMS_041712_NAR_30mM_plus2.12131.12131.2</td><td>2</td><td>14.69</td><td>0</td><td>1.34E+06</td><td></td><td>(K)GIYAVGI</td><td>(K)G I/Y|A/V|G D V/C G K(A)</td><td>C:Carbamidomethylation</td><td>117.58</td><td>569.7798</td><td>1138.556</td><td>-0.0038</td><td>-3.3</td><td>5.83</td><td>56826.6</td><td>9.16</td><td>HUMAN</td><td>P00390</td><td>Glutathione reductase, mitochondrial</td></tr>
<tr><td>3275</td><td>dirMS_041712_NAR_30mM_plus2.8771.8828.2</td><td>2</td><td>14.69</td><td>0</td><td>7.16E+04</td><td></td><td>(K)FGDSNT</td><td>(K)F/G|D|S|N T/V/M/R(F)</td><td></td><td>99.8</td><td>513.7373</td><td>1026.467</td><td>0</td><td>0</td><td>5.84</td><td>334221.3</td><td>6.44</td><td>HUMAN</td><td>P15924</td><td>Desmoplakin</td></tr>
<tr><td>3276</td><td>dirMS_041712_NAR_50mM_plus2.15011.15202.3</td><td>3</td><td>14.69</td><td>0</td><td>7.94E+05</td><td></td><td>(R)QIFHPEC</td><td>(R)Q I/F H/P E Q/L/I/T G K(E)</td><td></td><td>147.53</td><td>470.9293</td><td>1410.774</td><td>-0.0006</td><td>-0.4</td><td>6.75</td><td>27836</td><td>8</td><td>HUMAN</td><td>Q9H853</td><td>Putative tubulin-like protein alpha-4B</td></tr>
<tr><td>3277</td><td>dirMS_041712_NAR_60mM_plus.10819.11162.3</td><td>3</td><td>14.69</td><td>1</td><td>1.34E+06</td><td></td><td>(R)KAGNFY</td><td>(R)K\A G\N F\Y|V/P/A E/P/K(L)</td><td></td><td>115.4</td><td>440.9074</td><td>1320.695</td><td>0.013</td><td>9.8</td><td>8.5</td><td>24489.6</td><td>10.74</td><td>HUMAN</td><td>A8MUD9</td><td>60S ribosomal protein L7</td></tr>
<tr><td>3278</td><td>dirMS_041712_NAR_60mM_plus.16092.16234.3</td><td>3</td><td>14.69</td><td>1</td><td>6.29E+05</td><td></td><td>(R)IKIGDPL</td><td>(R)I K I G D/P\L|L/E/D/T/R(M)</td><td></td><td>141.63</td><td>457.2595</td><td>1369.769</td><td>-0.0046</td><td>-3.4</td><td>4.56</td><td>47309.6</td><td>5.42</td><td>HUMAN</td><td>B4DXY7</td><td>4-trimethylaminobutyraldehyde dehydrogenase</td></tr>
<tr><td>3279</td><td>dirMS_041712_NAR_60mM_plus.16293.16293.3</td><td>3</td><td>14.69</td><td>0</td><td>6.39E+05</td><td></td><td>(R)ETHSALC</td><td>(R)E T H S\A\L/G|P\A/L/Q/A A/F K(L)</td><td></td><td>141.93</td><td>514.2744</td><td>1540.812</td><td>-0.0032</td><td>-2.1</td><td>6.85</td><td>141616.6</td><td>6.1</td><td>HUMAN</td><td>B7ZKM8</td><td>Protein transport protein Sec24B</td></tr>
<tr><td>3280</td><td>dirMS_041712_NAR_60mM_plus.18245.18245.3</td><td>3</td><td>14.69</td><td>1</td><td>9.27E+05</td><td></td><td>(K)EVNKFQ</td><td>(K)E V N K F Q M|A/Y/S/N/L/L/R(A)</td><td></td><td>152.8</td><td>571.6293</td><td>1712.879</td><td>-0.0055</td><td>-3.2</td><td>8.69</td><td>14683.8</td><td>10.19</td><td>HUMAN</td><td>P37108</td><td>Signal recognition particle 14 kDa protein</td></tr>
<tr><td>3281</td><td>dirMS_041712_NAR_50mM_minus2.14951.15090.3</td><td>3</td><td>14.68</td><td>1</td><td>2.09E+05</td><td></td><td>(K)VVEEAPS</td><td>(K)V/V|E/E/A|P S I F L D/A E T R R(A)</td><td></td><td>153.72</td><td>611.3243</td><td>1831.955</td><td>0.0035</td><td>1.9</td><td>4.41</td><td>80685.1</td><td>7.31</td><td>HUMAN</td><td>P05165</td><td>Propionyl-CoA carboxylase alpha chain, mitochondrial</td></tr>
<tr><td>3282</td><td>dirMS_041712_NAR_120mM_plus.4205.4205.2</td><td>2</td><td>14.68</td><td>1</td><td>1.40E+06</td><td>M310m</td><td>(R)TKTEISEI</td><td>(R)T/K|T|E|I|S E m N R(N)</td><td>m:Oxidized methionine</td><td>69.85</td><td>612.7976</td><td>1208.594</td><td>15.994</td><td>-0.8</td><td>5.81</td><td>37296.3</td><td>5.3</td><td>HUMAN</td><td>F8VU64</td><td>Keratin, type II cytoskeletal 8</td></tr>
<tr><td>3283</td><td>dirMS_041712_NAR_120mM_plus.8497.8497.3</td><td>3</td><td>14.68</td><td>1</td><td>2.94E+05</td><td></td><td>(R)ASGNYA</td><td>(R)A/S|G/N/Y/A/T V I S H N P E T K K(T)</td><td></td><td>97.62</td><td>606.3102</td><td>1816.919</td><td>-0.0028</td><td>-1.5</td><td>8.55</td><td>24115.6</td><td>11.59</td><td>HUMAN</td><td>E9PIZ3</td><td>60S ribosomal protein L8</td></tr>
<tr><td>3284</td><td>dirMS_041712_NAR_30mM_plus2.8432.8632.2</td><td>2</td><td>14.68</td><td>0</td><td>9.63E+05</td><td></td><td>(K)LYEQLSC</td><td>(K)L/Y|E/Q/L/S/G/K(-)</td><td></td><td>99.03</td><td>469.2488</td><td>937.499</td><td>-0.0086</td><td>-9.2</td><td>6</td><td>21170.5</td><td>7.02</td><td>HUMAN</td><td>P30086</td><td>Phosphatidylethanolamine-binding protein 1</td></tr>
<tr><td>3285</td><td>dirMS_041712_NAR_50mM_plus2.19650.19883.3</td><td>3</td><td>14.68</td><td>1</td><td>1.01E+06</td><td></td><td>(K)LKEAELL</td><td>(K)L K E A E L\L|E|P|L/M/P A I R(A)</td><td></td><td>172.58</td><td>574.9989</td><td>1722.982</td><td>-0.0001</td><td>-0.1</td><td>4.79</td><td>56164.6</td><td>6.01</td><td>HUMAN</td><td>E9PP73</td><td>Coatomer subunit beta (Fragment)</td></tr>
<tr><td>3286</td><td>dirMS_041712_NAR_60mM_plus.20561.20561.3</td><td>3</td><td>14.68</td><td>1</td><td>7.00E+05</td><td></td><td>(K)IKYPENF</td><td>(K)I/K Y P E N/F/F/L/L/R(G)</td><td></td><td>167.98</td><td>480.6056</td><td>1439.805</td><td>-0.0023</td><td>-1.6</td><td>8.59</td><td>33222.3</td><td>5.74</td><td>HUMAN</td><td>A6NNR3</td><td>Serine/threonine-protein phosphatase</td></tr>
<tr><td>3287</td><td>dirMS_041712_NAR_50mM_minus2.7699.7699.3</td><td>3</td><td>14.67</td><td>1</td><td>1.00E+05</td><td></td><td>(K)TAVCDIF</td><td>(K)T/A|V/C D I/P/P R G L K(M)</td><td>C:Carbamidomethylation</td><td>111.52</td><td>442.9126</td><td>1326.72</td><td>0.0034</td><td>2.6</td><td>8.41</td><td>50199.4</td><td>4.75</td><td>HUMAN</td><td>A6NNZ2</td><td>Tubulin beta-8 chain-like protein LOC260334</td></tr>
<tr><td>3288</td><td>dirMS_041712_NAR_20mM_plus.10550.10550.2</td><td>2</td><td>14.67</td><td>0</td><td>4.53E+04</td><td></td><td>(R)AQPSVS</td><td>(R)A Q|P S/V\S/L/G A A Y R(A)</td><td></td><td>145.72</td><td>610.3183</td><td>1219.643</td><td>-0.0136</td><td>-11.2</td><td>8.79</td><td>69661.6</td><td>9.75</td><td>HUMAN</td><td>Q96PK6</td><td>RNA-binding protein 14</td></tr>
<tr><td>3289</td><td>dirMS_041712_NAR_20mM_plus.19015.19015.2</td><td>2</td><td>14.67</td><td>0</td><td>1.90E+05</td><td></td><td>(R)VCTLAIII</td><td>(R)V C T\L|A|I|I|D P G D S D I I R(S)</td><td>C:Carbamidomethylation</td><td>202.68</td><td>879.4578</td><td>1757.91</td><td>-0.0019</td><td>-1.1</td><td>3.93</td><td>12826.8</td><td>9.82</td><td>HUMAN</td><td>E5RI99</td><td>60S ribosomal protein L30 (Fragment)</td></tr>
<tr><td>3290</td><td>dirMS_041712_NAR_30mM_plus2.18402.18441.2</td><td>2</td><td>14.67</td><td>0</td><td>7.81E+04</td><td></td><td>(R)VVNVSS</td><td>(R)V V|N\V|S/S I/M/S V/R(A)</td><td></td><td>149.77</td><td>595.8347</td><td>1190.656</td><td>0.006</td><td>5</td><td>9.72</td><td>30659.7</td><td>9.02</td><td>HUMAN</td><td>P16152</td><td>Carbonyl reductase [NADPH] 1</td></tr>
<tr><td>3291</td><td>dirMS_041712_NAR_30mM_plus2.22052.22052.2</td><td>2</td><td>14.67</td><td>0</td><td>1.87E+05</td><td></td><td>(R)EGDVLT</td><td>(R)E G D\V/L/T/L/L|E/S E/R(E)</td><td></td><td>170.55</td><td>680.8538</td><td>1360.696</td><td>0.0049</td><td>3.6</td><td>4</td><td>7898</td><td>11.1</td><td>HUMAN</td><td>P62857</td><td>40S ribosomal protein S28</td></tr>
<tr><td>3292</td><td>dirMS_041712_NAR_30mM_plus2.7468.7469.2</td><td>2</td><td>14.67</td><td>0</td><td>5.73E+05</td><td></td><td>(R)DGYQQI</td><td>(R)D G|Y|Q/Q/N/F/K(R)</td><td></td><td>93.6</td><td>500.2305</td><td>999.453</td><td>0.0007</td><td>0.7</td><td>5.83</td><td>70523.3</td><td>4.94</td><td>HUMAN</td><td>G3V153</td><td>Caprin-1</td></tr>
<tr><td>3293</td><td>dirMS_041712_NAR_60mM_plus.14679.14911.3</td><td>3</td><td>14.67</td><td>0</td><td>8.30E+05</td><td></td><td>(R)LYQQHG</td><td>(R)L/Y/Q Q H/G A G L/F/D/V T R(G)</td><td></td><td>134.47</td><td>535.6064</td><td>1604.818</td><td>-0.0133</td><td>-8.3</td><td>6.74</td><td>61760.3</td><td>6.01</td><td>HUMAN</td><td>O14773</td><td>Tripeptidyl-peptidase 1</td></tr>
<tr><td>3294</td><td>dirMS_041712_NAR_50mM_minus2.6185.6185.3</td><td>3</td><td>14.66</td><td>0</td><td>5.69E+04</td><td></td><td>(R)ASAAPK</td><td>(R)A S/A|A/P K/P E/P V/P/V Q K(V)</td><td></td><td>97.78</td><td>473.6033</td><td>1418.8</td><td>-0.0048</td><td>-3.4</td><td>8.64</td><td>23930.8</td><td>10.06</td><td>HUMAN</td><td>F5H0X8</td><td>PDZ and LIM domain protein 5</td></tr>
<tr><td>3295</td><td>dirMS_041712_NAR_50mM_minus2.8717.8717.3</td><td>3</td><td>14.66</td><td>1</td><td>8.70E+04</td><td></td><td>(R)LLDEEEA</td><td>(R)L/L|D/E E E A/T D N D L R A K(F)</td><td></td><td>117.43</td><td>577.9535</td><td>1731.84</td><td>0.0064</td><td>3.7</td><td>4.02</td><td>96649</td><td>6.13</td><td>HUMAN</td><td>Q8WUM4</td><td>Programmed cell death 6-interacting protein</td></tr>
<tr><td>3296</td><td>dirMS_041712_NAR_50mM_minus2.8947.8947.3</td><td>3</td><td>14.66</td><td>1</td><td>1.31E+05</td><td>M227m</td><td>(R)mQKEIT.</td><td>(R)m Q K E I|T|A|L/A/P S/T M K(I)</td><td>m:Oxidized methionine</td><td>119.48</td><td>522.2727</td><td>1548.812</td><td>15.9911</td><td>-2.4</td><td>8.35</td><td>32616.5</td><td>5.18</td><td>HUMAN</td><td>A6NL76</td><td>Actin, alpha skeletal muscle</td></tr>
<tr><td>3297</td><td>dirMS_041712_NAR_60mM_minus.16933.16933.3</td><td>3</td><td>14.66</td><td>1</td><td>2.65E+05</td><td></td><td>(R)GQFSTD</td><td>(R)G Q|F/S T D/E L/V/A/E V E K R(N)</td><td></td><td>154</td><td>569.9569</td><td>1707.855</td><td>0.0013</td><td>0.8</td><td>4.41</td><td>144109.9</td><td>6.01</td><td>HUMAN</td><td>J3KR87</td><td>Clathrin heavy chain 2</td></tr>
<tr><td>3298</td><td>dirMS_041712_NAR_60mM_minus.9218.9218.3</td><td>3</td><td>14.66</td><td>0</td><td>2.84E+05</td><td></td><td>(K)ATAGDT</td><td>(K)A T|A/G D/T H\L/G G E D/F D/N R(L)</td><td></td><td>111.4</td><td>559.2488</td><td>1675.731</td><td>0.0012</td><td>0.7</td><td>4.22</td><td>58502.6</td><td>6.09</td><td>HUMAN</td><td>B4DXY3</td><td>Heat shock 70 kDa protein 1-like</td></tr>
<tr><td>3299</td><td>dirMS_041712_NAR_30mM_plus2.20365.20850.2</td><td>2</td><td>14.66</td><td>0</td><td>1.40E+06</td><td></td><td>(R)ILVATNL</td><td>(R)I/L|V|A|T/N L/F G R(G)</td><td></td><td>162.17</td><td>552.331</td><td>1103.657</td><td>-0.0024</td><td>-2.2</td><td>9.75</td><td>40918.8</td><td>9.33</td><td>HUMAN</td><td>B4DP52</td><td>HCG2005638, isoform CRA_c</td></tr>
<tr><td>3300</td><td>dirMS_041712_NAR_50mM_plus2.11060.11060.3</td><td>3</td><td>14.66</td><td>1</td><td>2.67E+04</td><td></td><td>(R)SAAETV</td><td>(R)S A|A/E T V T K/G G I/M L/P/E/K(S)</td><td></td><td>125.88</td><td>544.624</td><td>1631.867</td><td>-0.0098</td><td>-6</td><td>5.86</td><td>10803.5</td><td>9.82</td><td>HUMAN</td><td>B8ZZL8</td><td>10 kDa heat shock protein, mitochondrial</td></tr>
<tr><td>3301</td><td>dirMS_041712_NAR_120mM_minus.11163.11404.3</td><td>3</td><td>14.65</td><td>0</td><td>4.42E+05</td><td></td><td>(K)SAKPTKF</td><td>(K)S A K|P T K P A A/S D|L|P\V|P A E G V R(N)</td><td></td><td>116.37</td><td>664.3662</td><td>1991.092</td><td>-0.008</td><td>-4</td><td>8.31</td><td>54214.9</td><td>5.66</td><td>HUMAN</td><td>C9J813</td><td>Caldesmon (Fragment)</td></tr>
<tr><td>3302</td><td>dirMS_041712_NAR_30mM_minus2.11341.11603.2</td><td>2</td><td>14.65</td><td>0</td><td>1.18E+06</td><td></td><td>(K)LEAEIAT</td><td>(K)L/E|A|E|I A/T Y/R(R)</td><td></td><td>116.22</td><td>533.2797</td><td>1065.557</td><td>-0.0054</td><td>-5</td><td>4.53</td><td>43773.3</td><td>5.26</td><td>HUMAN</td><td>F8VZY9</td><td>Keratin, type I cytoskeletal 18</td></tr>
<tr><td>3303</td><td>dirMS_041712_NAR_30mM_minus2.12201.12201.2</td><td>2</td><td>14.65</td><td>0</td><td>3.38E+05</td><td></td><td>(K)ILYSQCG</td><td>(K)I L|Y|S/Q/C/G D V M\R(A)</td><td>C:Carbamidomethylation</td><td>119.22</td><td>671.3183</td><td>1341.629</td><td>0.0004</td><td>0.3</td><td>5.83</td><td>26991.7</td><td>5.01</td><td>HUMAN</td><td>B7Z6Z4</td><td>Myosin light polypeptide 6</td></tr>
<tr><td>3304</td><td>dirMS_041712_NAR_40mM_minus2.9045.9045.3</td><td>3</td><td>14.65</td><td>1</td><td>9.47E+04</td><td></td><td>(K)LAEIGAF</td><td>(K)L/A|E|I/G|A/P I Q/G N R E E L V E R(L)</td><td></td><td>136.07</td><td>665.3586</td><td>1994.066</td><td>-0.0053</td><td>-2.6</td><td>4.49</td><td>38572.2</td><td>6.31</td><td>HUMAN</td><td>E9PJ04</td><td>Splicing factor 3B subunit 2 (Fragment)</td></tr>
<tr><td>3305</td><td>dirMS_041712_NAR_60mM_minus.11159.11215.3</td><td>3</td><td>14.65</td><td>1</td><td>2.07E+05</td><td></td><td>(K)FADLSEA</td><td>(K)F A|D|L/S/E/A/A N R N N D A L/R(Q)</td><td></td><td>122.5</td><td>592.9581</td><td>1776.862</td><td>-0.0026</td><td>-1.5</td><td>4.56</td><td>49709.6</td><td>5.19</td><td>HUMAN</td><td>B0YJC4</td><td>Vimentin</td></tr>
<tr><td>3306</td><td>dirMS_041712_NAR_30mM_plus2.14645.14645.2</td><td>2</td><td>14.65</td><td>0</td><td>7.63E+05</td><td></td><td>(R)GVEICIA</td><td>(R)G V|E|I/C/I A/T/P G R(L)</td><td>C:Carbamidomethylation</td><td>130.1</td><td>586.8051</td><td>1172.609</td><td>-0.0063</td><td>-5.4</td><td>6</td><td>61018.6</td><td>9.44</td><td>HUMAN</td><td>B4DLW8</td><td>Probable ATP-dependent RNA helicase DDX5</td></tr>
<tr><td>3307</td><td>dirMS_041712_NAR_40mM_plus2.10790.10790.3</td><td>3</td><td>14.65</td><td>0</td><td>6.60E+05</td><td></td><td>(R)LLEAQA</td><td>(R)L/L|E/A/Q/A/C/T/G G I I H P T T G Q K(L)</td><td>C:Carbamidomethylation</td><td>129.48</td><td>665.6807</td><td>1995.033</td><td>-0.0052</td><td>-2.6</td><td>6.75</td><td>334221.3</td><td>6.44</td><td>HUMAN</td><td>P15924</td><td>Desmoplakin</td></tr>
<tr><td>3308</td><td>dirMS_041712_NAR_50mM_plus2.12001.12001.3</td><td>3</td><td>14.65</td><td>1</td><td>4.97E+05</td><td>M327m</td><td>(R)VAGGPC</td><td>(R)V/A/G G/P Q m I/Q|L/S L D G K/R(L)</td><td>m:Oxidized methionine</td><td>131.13</td><td>562.6377</td><td>1669.905</td><td>15.9931</td><td>-1</td><td>8.72</td><td>45924.5</td><td>5.29</td><td>HUMAN</td><td>B4E1F3</td><td>Selenium-binding protein 1</td></tr>
<tr><td>3309</td><td>dirMS_041712_NAR_50mM_plus2.19155.19155.3</td><td>3</td><td>14.65</td><td>0</td><td>4.98E+05</td><td></td><td>(R)GFGFITF</td><td>(R)G/F/G\F|I/T/F/T N/P E H A S V A M R(A)</td><td></td><td>168.98</td><td>661.3258</td><td>1981.959</td><td>0.004</td><td>2</td><td>6.75</td><td>17170.1</td><td>8.86</td><td>HUMAN</td><td>P98179</td><td>Putative RNA-binding protein 3</td></tr>
<tr><td>3310</td><td>dirMS_041712_NAR_60mM_plus.15380.15443.3</td><td>3</td><td>14.65</td><td>1</td><td>4.00E+05</td><td></td><td>(R)SLIANLA</td><td>(R)S L|I/A/N L/A/A/A N/C Y K K(E)</td><td>C:Carbamidomethylation</td><td>137.37</td><td>512.9411</td><td>1536.82</td><td>-0.0115</td><td>-7.5</td><td>9.7</td><td>36978.6</td><td>6.33</td><td>HUMAN</td><td>B7Z800</td><td>Adenosine kinase</td></tr>
<tr><td>3311</td><td>dirMS_041712_NAR_60mM_plus.19565.19607.3</td><td>3</td><td>14.65</td><td>1</td><td>6.66E+05</td><td></td><td>(K)TVTAMD</td><td>(K)T V|T A/M/D/V/V/Y A L K/R(Q)</td><td></td><td>161</td><td>489.6071</td><td>1466.804</td><td>0.0032</td><td>2.2</td><td>8.25</td><td>11367.1</td><td>11.36</td><td>HUMAN</td><td>P62805</td><td>Histone H4</td></tr>
<tr><td>3312</td><td>dirMS_041712_NAR_60mM_plus.9788.9788.3</td><td>3</td><td>14.65</td><td>1</td><td>1.29E+06</td><td></td><td>(K)EAALSTA</td><td>(K)E A A/L/S/T A/L/S E K/R(T)</td><td></td><td>109.78</td><td>425.898</td><td>1275.69</td><td>-0.0109</td><td>-8.5</td><td>6.24</td><td>63136.9</td><td>5.83</td><td>HUMAN</td><td>D6RAQ3</td><td>Prelamin-A/C</td></tr>
<tr><td>3313</td><td>dirMS_041712_NAR_120mM_minus.6847.6981.2</td><td>2</td><td>14.64</td><td>1</td><td>2.88E+06</td><td></td><td>(R)TKTEISEI</td><td>(R)T/K|T|E|I|S/E M N/R(N)</td><td></td><td>92.53</td><td>604.8012</td><td>1208.594</td><td>0.0012</td><td>1</td><td>5.81</td><td>37296.3</td><td>5.3</td><td>HUMAN</td><td>F8VU64</td><td>Keratin, type II cytoskeletal 8</td></tr>
<tr><td>3314</td><td>dirMS_041712_NAR_20mM_minus.12323.12323.2</td><td>2</td><td>14.64</td><td>0</td><td>1.61E+05</td><td></td><td>(R)AAVIGPI</td><td>(R)A A/V|I/G/P D V F A K(L)</td><td></td><td>171.58</td><td>544.3103</td><td>1087.615</td><td>-0.0013</td><td>-1.2</td><td>5.88</td><td>266161</td><td>5.68</td><td>HUMAN</td><td>E9PPU0</td><td>Epiplakin</td></tr>
<tr><td>3315</td><td>dirMS_041712_NAR_30mM_minus2.17776.17974.2</td><td>2</td><td>14.64</td><td>0</td><td>3.94E+05</td><td></td><td>(K)GNFNYII</td><td>(K)G N|F/N/Y|I/E/F T/R(I)</td><td></td><td>151.3</td><td>630.8052</td><td>1260.601</td><td>0.0024</td><td>1.9</td><td>6</td><td>20513.6</td><td>4.62</td><td>HUMAN</td><td>J3QRS3</td><td>Myosin regulatory light chain 12A</td></tr>
<tr><td>3316</td><td>dirMS_041712_NAR_30mM_minus2.7769.7769.2</td><td>2</td><td>14.64</td><td>0</td><td>3.47E+05</td><td></td><td>(K)SLAAEEE</td><td>(K)S L|A/A|E/E|E A A R(Q)</td><td></td><td>96.33</td><td>523.7597</td><td>1046.511</td><td>0.0008</td><td>0.8</td><td>4.25</td><td>533778</td><td>5.74</td><td>HUMAN</td><td>Q15149</td><td>Plectin</td></tr>
<tr><td>3317</td><td>dirMS_041712_NAR_60mM_minus.12183.12183.3</td><td>3</td><td>14.64</td><td>0</td><td>3.77E+05</td><td></td><td>(K)VTNEFV</td><td>(K)V/T/N E/F V/H/I N/N L/K(L)</td><td></td><td>127.53</td><td>476.5974</td><td>1427.764</td><td>0.0135</td><td>9.5</td><td>6.72</td><td>18317.8</td><td>6.19</td><td>HUMAN</td><td>B7Z3E2</td><td>Serine/threonine-protein phosphatase 6 catalytic subunit</td></tr>
<tr><td>3318</td><td>dirMS_041712_NAR_20mM_plus.14297.14435.2</td><td>2</td><td>14.64</td><td>0</td><td>6.81E+05</td><td>M73m</td><td>(R)AVLVDLI</td><td>(R)A V|L|V|D|L|E|P G T\m D S V R(S)</td><td>m:Oxidized methionine</td><td>171.03</td><td>809.4082</td><td>1601.82</td><td>15.9888</td><td>-3.8</td><td>4.03</td><td>13112.4</td><td>5.23</td><td>HUMAN</td><td>F8W813</td><td>Tubulin beta-8 chain</td></tr>
<tr><td>3319</td><td>dirMS_041712_NAR_30mM_plus2.19038.19038.2</td><td>2</td><td>14.64</td><td>0</td><td>1.51E+05</td><td></td><td>(R)VYSLFLD</td><td>(R)V/Y|S L/F/L D E S R(S)</td><td></td><td>153.08</td><td>614.8154</td><td>1228.621</td><td>0.0027</td><td>2.2</td><td>4.37</td><td>46476.4</td><td>5.33</td><td>HUMAN</td><td>B3KQ59</td><td>RuvB-like 2</td></tr>
<tr><td>3320</td><td>dirMS_041712_NAR_30mM_plus2.5728.5770.2</td><td>2</td><td>14.64</td><td>0</td><td>2.46E+05</td><td></td><td>(R)QSSGAS</td><td>(R)Q S|S/G/A|S/S/S/S/F/S S S R(A)</td><td></td><td>84.3</td><td>681.3013</td><td>1361.593</td><td>0.0025</td><td>1.9</td><td>9.75</td><td>73641.5</td><td>6.74</td><td>HUMAN</td><td>O00571</td><td>ATP-dependent RNA helicase DDX3X</td></tr>
<tr><td>3321</td><td>dirMS_041712_NAR_50mM_plus2.20121.20121.3</td><td>3</td><td>14.64</td><td>0</td><td>2.56E+05</td><td></td><td>(R)LQSLPFA</td><td>(R)L/Q/S L/P F/A/L T H L N\L K(A)</td><td></td><td>173.95</td><td>532.3178</td><td>1594.931</td><td>0.0073</td><td>4.6</td><td>8.76</td><td>175851.7</td><td>5.01</td><td>HUMAN</td><td>Q14160</td><td>Protein scribble homolog</td></tr>
<tr><td>3322</td><td>dirMS_041712_NAR_60mM_plus.12410.12516.3</td><td>3</td><td>14.64</td><td>1</td><td>3.85E+06</td><td></td><td>(K)LFNLSKE</td><td>(K)L/F|N/L/S K/E D D/V/R(Q)</td><td></td><td>121.6</td><td>445.9046</td><td>1335.69</td><td>0.0089</td><td>6.7</td><td>4.56</td><td>25082</td><td>11.29</td><td>HUMAN</td><td>A2A3R5</td><td>40S ribosomal protein S6</td></tr>
<tr><td>3323</td><td>dirMS_041712_NAR_20mM_minus.10869.10869.2</td><td>2</td><td>14.63</td><td>0</td><td>1.46E+06</td><td>M92m</td><td>(R)ImNTFS</td><td>(R)I m/N T\F\S V/V|P/S P K(V)</td><td>m:Oxidized methionine</td><td>157.08</td><td>668.3513</td><td>1319.703</td><td>15.9925</td><td>-1.8</td><td>8.75</td><td>42831.5</td><td>4.86</td><td>HUMAN</td><td>A8K854</td><td>HCG1983504, isoform CRA_f</td></tr>
<tr><td>3324</td><td>dirMS_041712_NAR_30mM_minus2.8390.8390.2</td><td>2</td><td>14.63</td><td>0</td><td>5.11E+05</td><td></td><td>(K)SFQQSSI</td><td>(K)S F|Q Q/S/S L/S R(D)</td><td></td><td>100.55</td><td>520.2591</td><td>1039.517</td><td>-0.0058</td><td>-5.6</td><td>9.47</td><td>100478.9</td><td>5.71</td><td>HUMAN</td><td>A8MXP9</td><td>Matrin-3</td></tr>
<tr><td>3325</td><td>dirMS_041712_NAR_50mM_minus2.10601.10601.3</td><td>3</td><td>14.63</td><td>0</td><td>1.70E+05</td><td></td><td>(R)LLELGPK</td><td>(R)L L|E|L|G P K/P E/V A Q Q\T R(K)</td><td></td><td>128.62</td><td>560.3182</td><td>1678.949</td><td>-0.0086</td><td>-5.1</td><td>6.14</td><td>139883.7</td><td>8.06</td><td>HUMAN</td><td>P53621</td><td>Coatomer subunit alpha</td></tr>
<tr><td>3326</td><td>dirMS_041712_NAR_60mM_minus.9129.9166.3</td><td>3</td><td>14.63</td><td>1</td><td>1.02E+06</td><td></td><td>(R)ETAEAD</td><td>(R)E/T/A/E/A D/V|A S L N R R(I)</td><td></td><td>111.37</td><td>477.9107</td><td>1431.719</td><td>-0.0011</td><td>-0.8</td><td>4.68</td><td>31923.6</td><td>4.85</td><td>HUMAN</td><td>B7Z596</td><td>Tropomyosin alpha-1 chain</td></tr>
<tr><td>3327</td><td>dirMS_041712_NAR_20mM_plus.17571.17571.2</td><td>2</td><td>14.63</td><td>0</td><td>3.20E+04</td><td></td><td>(K)LFVTND</td><td>(K)L F|V|T/N/D A/A T I L R(E)</td><td></td><td>192.63</td><td>667.3752</td><td>1333.747</td><td>-0.0043</td><td>-3.2</td><td>5.84</td><td>58214.7</td><td>5.25</td><td>HUMAN</td><td>B4DEM7</td><td>T-complex protein 1 subunit theta</td></tr>
<tr><td>3328</td><td>dirMS_041712_NAR_30mM_plus2.9052.9596.2</td><td>2</td><td>14.63</td><td>0</td><td>7.51E+06</td><td></td><td>(R)AVVIVDI</td><td>(R)A V|V|I|V/D/D/R(G)</td><td></td><td>102.47</td><td>443.7534</td><td>886.499</td><td>0.0003</td><td>0.3</td><td>4.21</td><td>23729.5</td><td>9.89</td><td>HUMAN</td><td>C9IZL7</td><td>Non-POU domain-containing octamer-binding protein (Fragment)</td></tr>
<tr><td>3329</td><td>dirMS_041712_NAR_40mM_plus2.16055.16055.3</td><td>3</td><td>14.63</td><td>0</td><td>3.19E+05</td><td></td><td>(K)VAIVKPC</td><td>(K)V/A|I/V K/P G V|P M E I V L N K(E)</td><td></td><td>161.2</td><td>569.6778</td><td>1707.024</td><td>-0.0049</td><td>-2.9</td><td>8.56</td><td>73146.9</td><td>5.45</td><td>HUMAN</td><td>E9PJ21</td><td>Hypoxia up-regulated protein 1 (Fragment)</td></tr>
<tr><td>3330</td><td>dirMS_041712_NAR_50mM_plus2.17899.17899.3</td><td>3</td><td>14.63</td><td>0</td><td>2.57E+05</td><td></td><td>(K)YAPLHL</td><td>(K)Y/A/P L/H/L/V/P L I E R(L)</td><td></td><td>162.08</td><td>474.2819</td><td>1420.831</td><td>0.0001</td><td>0</td><td>6.75</td><td>147553.6</td><td>6.73</td><td>HUMAN</td><td>E7EVF4</td><td>Cytoplasmic FMR1-interacting protein 2</td></tr>
<tr><td>3331</td><td>dirMS_041712_NAR_50mM_plus2.5216.5216.3</td><td>3</td><td>14.63</td><td>0</td><td>1.37E+06</td><td></td><td>(K)TATPQQ</td><td>(K)T/A|T/P Q Q/A Q E/V H E K(L)</td><td></td><td>86.38</td><td>489.5774</td><td>1466.723</td><td>-0.0057</td><td>-3.9</td><td>5.37</td><td>31075.7</td><td>5.65</td><td>HUMAN</td><td>P60174</td><td>Triosephosphate isomerase</td></tr>
<tr><td>3332</td><td>dirMS_041712_NAR_60mM_plus.14361.14591.3</td><td>3</td><td>14.63</td><td>1</td><td>8.43E+06</td><td></td><td>(K)KVPQVS</td><td>(K)K\V P\Q\V S T|P/T|L|V/E V S R(N)</td><td></td><td>132.15</td><td>547.3173</td><td>1639.938</td><td>-0.0004</td><td>-0.2</td><td>8.75</td><td>57751.3</td><td>6.9</td><td>HUMAN</td><td>B7WNR0</td><td>Serum albumin</td></tr>
<tr><td>3333</td><td>dirMS_041712_NAR_30mM_minus2.14918.15043.2</td><td>2</td><td>14.62</td><td>0</td><td>9.09E+05</td><td></td><td>(R)NFDEILR</td><td>(R)N/F|D/E/I/L/R(V)</td><td></td><td>135.07</td><td>453.7335</td><td>906.468</td><td>-0.0082</td><td>-9.1</td><td>4.37</td><td>25148.6</td><td>6</td><td>HUMAN</td><td>P30041</td><td>Peroxiredoxin-6</td></tr>
<tr><td>3334</td><td>dirMS_041712_NAR_60mM_minus.17139.17139.3</td><td>3</td><td>14.62</td><td>1</td><td>7.39E+05</td><td></td><td>(K)QLDDLK</td><td>(K)Q/L/D D L/K/V/E L/S Q L R(V)</td><td></td><td>156.18</td><td>519.6267</td><td>1556.864</td><td>0.0013</td><td>0.8</td><td>4.56</td><td>10815.7</td><td>10.72</td><td>HUMAN</td><td>F2Z388</td><td>60S ribosomal protein L35</td></tr>
<tr><td>3335</td><td>dirMS_041712_NAR_120mM_plus.20960.20960.3</td><td>3</td><td>14.62</td><td>2</td><td>1.39E+06</td><td></td><td>(R)TLAEIAK</td><td>(R)T L|A\E/I/A K V E L/D N\M/P L R G K(Q)</td><td></td><td>162.92</td><td>666.7047</td><td>1998.105</td><td>-0.0057</td><td>-2.8</td><td>5.85</td><td>23729.5</td><td>9.89</td><td>HUMAN</td><td>C9IZL7</td><td>Non-POU domain-containing octamer-binding protein (Fragment)</td></tr>
<tr><td>3336</td><td>dirMS_041712_NAR_120mM_plus.7413.7454.3</td><td>3</td><td>14.62</td><td>1</td><td>1.14E+06</td><td></td><td>(R)EKVPGG</td><td>(R)E K V/P G G L|Q/G/S Q D/R(G)</td><td></td><td>92.32</td><td>457.5737</td><td>1370.702</td><td>0.0043</td><td>3.1</td><td>6.17</td><td>180453.7</td><td>6.04</td><td>HUMAN</td><td>H0YIQ2</td><td>YLP motif-containing protein 1 (Fragment)</td></tr>
<tr><td>3337</td><td>dirMS_041712_NAR_120mM_plus.9842.9919.3</td><td>3</td><td>14.62</td><td>1</td><td>7.96E+05</td><td>M142m</td><td>(K)VKEGMI</td><td>(K)V K E G M N|I|V/E/A m/E R(F)</td><td>m:Oxidized methionine</td><td>104.72</td><td>507.918</td><td>1505.745</td><td>15.9944</td><td>-0.3</td><td>4.79</td><td>18240.4</td><td>7.96</td><td>HUMAN</td><td>P62937</td><td>Peptidyl-prolyl cis-trans isomerase A</td></tr>
<tr><td>3338</td><td>dirMS_041712_NAR_30mM_plus2.18593.18678.2</td><td>2</td><td>14.62</td><td>0</td><td>2.03E+05</td><td></td><td>(K)SILFVPT</td><td>(K)S I|L/F|V|P/T S A P/R(G)</td><td></td><td>151.1</td><td>594.3409</td><td>1187.678</td><td>-0.0038</td><td>-3.2</td><td>9.47</td><td>92752.5</td><td>4.76</td><td>HUMAN</td><td>P14625</td><td>Endoplasmin</td></tr>
<tr><td>3339</td><td>dirMS_041712_NAR_40mM_plus2.18895.18895.3</td><td>3</td><td>14.62</td><td>1</td><td>3.85E+05</td><td></td><td>(R)TLAEIAK</td><td>(R)T L|A\E|I/A/K V E L/D/N M/P L R(G)</td><td></td><td>176.82</td><td>604.9967</td><td>1812.989</td><td>-0.0132</td><td>-7.3</td><td>4.68</td><td>23729.5</td><td>9.89</td><td>HUMAN</td><td>C9IZL7</td><td>Non-POU domain-containing octamer-binding protein (Fragment)</td></tr>
<tr><td>3340</td><td>dirMS_041712_NAR_30mM_minus2.12954.13235.2</td><td>2</td><td>14.61</td><td>0</td><td>1.09E+06</td><td></td><td>(R)LTPEDFA</td><td>(R)L/T|P/E/D/F A/R(A)</td><td></td><td>124.93</td><td>474.7377</td><td>948.479</td><td>-0.0104</td><td>-11</td><td>4.37</td><td>24724.1</td><td>5.17</td><td>HUMAN</td><td>E7ETQ7</td><td>Vacuolar protein sorting-associated protein VTA1 homolog</td></tr>
<tr><td>3341</td><td>dirMS_041712_NAR_30mM_minus2.19551.19551.2</td><td>2</td><td>14.61</td><td>0</td><td>4.63E+05</td><td></td><td>(R)ASLSLW</td><td>(R)A S/L|S|L/W/Q A/M E K(G)</td><td></td><td>162.7</td><td>632.3234</td><td>1263.64</td><td>-0.0007</td><td>-0.5</td><td>6.05</td><td>266161</td><td>5.68</td><td>HUMAN</td><td>E9PPU0</td><td>Epiplakin</td></tr>
<tr><td>3342</td><td>dirMS_041712_NAR_30mM_minus2.9480.9647.2</td><td>2</td><td>14.61</td><td>0</td><td>4.44E+05</td><td></td><td>(K)VCNPIIT</td><td>(K)V/C|N|P I|I/T K(L)</td><td>C:Carbamidomethylation</td><td>106.6</td><td>472.7669</td><td>944.523</td><td>0.0032</td><td>3.3</td><td>8.72</td><td>45508.6</td><td>5.5</td><td>HUMAN</td><td>A8K7Q2</td><td>Heat shock cognate 71 kDa protein</td></tr>
<tr><td>3343</td><td>dirMS_041712_NAR_60mM_minus.14891.14891.3</td><td>3</td><td>14.61</td><td>1</td><td>2.10E+05</td><td></td><td>(K)IDSRLEC</td><td>(K)I/D S R L E Q|Y|T|S/A I E G T K(S)</td><td></td><td>142.88</td><td>604.3095</td><td>1810.918</td><td>-0.0042</td><td>-2.3</td><td>4.68</td><td>54214.9</td><td>5.66</td><td>HUMAN</td><td>C9J813</td><td>Caldesmon (Fragment)</td></tr>
<tr><td>3344</td><td>dirMS_041712_NAR_50mM_plus2.11629.11839.3</td><td>3</td><td>14.61</td><td>1</td><td>1.78E+06</td><td></td><td>(K)TGQPMI</td><td>(K)T/G|Q|P M/I/N L Y/T/D R E T G K(L)</td><td></td><td>130.72</td><td>608.6361</td><td>1823.896</td><td>-0.0019</td><td>-1</td><td>5.73</td><td>53724.2</td><td>9.54</td><td>HUMAN</td><td>H3BPE7</td><td>RNA-binding protein FUS</td></tr>
<tr><td>3345</td><td>dirMS_041712_NAR_60mM_minus.16797.16813.3</td><td>3</td><td>14.6</td><td>1</td><td>2.46E+05</td><td></td><td>(R)KGFSEGI</td><td>(R)K G F S E G L\W|E|I|E/N/N P\T V K(A)</td><td></td><td>153.75</td><td>649.9986</td><td>1947.981</td><td>0.0002</td><td>0.1</td><td>4.79</td><td>23020.5</td><td>4.53</td><td>HUMAN</td><td>A8K8G0</td><td>Hepatoma-derived growth factor</td></tr>
<tr><td>3346</td><td>dirMS_041712_NAR_60mM_minus.9964.9985.3</td><td>3</td><td>14.6</td><td>0</td><td>1.56E+06</td><td></td><td>(R)EIVHIQA</td><td>(R)E/I\V\H\I Q A/G/Q|C/G N Q I/G A K(F)</td><td>C:Carbamidomethylation</td><td>115.57</td><td>608.3099</td><td>1822.923</td><td>-0.0077</td><td>-4.2</td><td>6.85</td><td>40069.4</td><td>4.83</td><td>HUMAN</td><td>E7EWR1</td><td>Tubulin beta chain</td></tr>
<tr><td>3347</td><td>dirMS_041712_NAR_20mM_plus.16904.16904.2</td><td>2</td><td>14.6</td><td>0</td><td>3.11E+05</td><td></td><td>(K)VQIAVA</td><td>(K)V Q\I/A/V|A/N/A/Q E|L L Q/R(M)</td><td></td><td>187.77</td><td>776.9419</td><td>1552.881</td><td>-0.004</td><td>-2.6</td><td>5.97</td><td>10728.1</td><td>9.15</td><td>HUMAN</td><td>Q9Y5L4</td><td>Mitochondrial import inner membrane translocase subunit Tim13</td></tr>
<tr><td>3348</td><td>dirMS_041712_NAR_30mM_plus2.20743.20743.2</td><td>2</td><td>14.6</td><td>0</td><td>4.46E+05</td><td></td><td>(R)SLVPAAI</td><td>(R)S L|V/P A A E L L E/S R(V)</td><td></td><td>162.23</td><td>642.8602</td><td>1284.716</td><td>-0.0027</td><td>-2.1</td><td>4.53</td><td>533778</td><td>5.74</td><td>HUMAN</td><td>Q15149</td><td>Plectin</td></tr>
<tr><td>3349</td><td>dirMS_041712_NAR_30mM_plus2.21302.21302.2</td><td>2</td><td>14.6</td><td>0</td><td>2.83E+05</td><td></td><td>(R)GVVQEL</td><td>(R)G V\V/Q/E/L/Q Q\A|I/S K(L)</td><td></td><td>165.97</td><td>650.3632</td><td>1299.727</td><td>-0.0076</td><td>-5.8</td><td>6</td><td>22489.6</td><td>5.38</td><td>HUMAN</td><td>E9PI39</td><td>Elongation factor 1-delta (Fragment)</td></tr>
<tr><td>3350</td><td>dirMS_041712_NAR_30mM_plus2.22193.22395.2</td><td>2</td><td>14.6</td><td>1</td><td>6.77E+05</td><td></td><td>(K)ATCKDL</td><td>(K)A T|C K D L\V|M|F|I(-)</td><td>C:Carbamidomethylation</td><td>172.75</td><td>599.3037</td><td>1197.601</td><td>-0.0005</td><td>-0.4</td><td>5.88</td><td>26705.5</td><td>7.89</td><td>HUMAN</td><td>P54819</td><td>Adenylate kinase 2, mitochondrial</td></tr>
<tr><td>3351</td><td>dirMS_041712_NAR_30mM_plus2.8261.8308.2</td><td>2</td><td>14.6</td><td>0</td><td>2.73E+05</td><td></td><td>(K)EIAENAL</td><td>(K)E I/A|E/N A/L/G K(H)</td><td></td><td>96.92</td><td>472.755</td><td>944.505</td><td>-0.002</td><td>-2.1</td><td>4.53</td><td>36982.9</td><td>6.37</td><td>HUMAN</td><td>P31942</td><td>Heterogeneous nuclear ribonucleoprotein H3</td></tr>
<tr><td>3352</td><td>dirMS_041712_NAR_50mM_plus2.18738.18738.3</td><td>3</td><td>14.6</td><td>1</td><td>4.36E+05</td><td></td><td>(K)AAIDWF</td><td>(K)A A|I/D|W/F/D G K E F S G N\P I K(V)</td><td></td><td>167.18</td><td>632.3186</td><td>1894.933</td><td>0.0079</td><td>4.1</td><td>4.56</td><td>53724.2</td><td>9.54</td><td>HUMAN</td><td>H3BPE7</td><td>RNA-binding protein FUS</td></tr>
<tr><td>3353</td><td>dirMS_041712_NAR_50mM_plus2.9938.9947.3</td><td>3</td><td>14.6</td><td>1</td><td>3.08E+05</td><td></td><td>(R)SATKVT</td><td>(R)S A|T K V|T A D|V|I/N/A/A E K(L)</td><td></td><td>119.68</td><td>506.6094</td><td>1517.817</td><td>-0.0033</td><td>-2.2</td><td>5.79</td><td>57391.2</td><td>6.29</td><td>HUMAN</td><td>O43175</td><td>D-3-phosphoglycerate dehydrogenase</td></tr>
<tr><td>3354</td><td>dirMS_041712_NAR_20mM_plus.8104.8104.2</td><td>2</td><td>14.59</td><td>0</td><td>4.05E+05</td><td>M239m</td><td>(K)EVDEQm</td><td>(K)E V/D E Q m L\N|V/Q/N K(N)</td><td>m:Oxidized methionine</td><td>125.13</td><td>731.8435</td><td>1446.689</td><td>15.9904</td><td>-3.1</td><td>4.14</td><td>40069.4</td><td>4.83</td><td>HUMAN</td><td>E7EWR1</td><td>Tubulin beta chain</td></tr>
<tr><td>3355</td><td>dirMS_041712_NAR_50mM_minus2.13285.13285.3</td><td>3</td><td>14.58</td><td>0</td><td>1.68E+05</td><td></td><td>(K)MLDAED</td><td>(K)M/L/D/A/E/D/I V G T A R P D E K(A)</td><td></td><td>143.55</td><td>587.2894</td><td>1759.853</td><td>0.0006</td><td>0.3</td><td>4.1</td><td>107710.1</td><td>5.38</td><td>HUMAN</td><td>B7TY16</td><td>Actinin alpha 1 isoform 3</td></tr>
<tr><td>3356</td><td>dirMS_041712_NAR_50mM_minus2.15931.15931.3</td><td>3</td><td>14.58</td><td>0</td><td>9.69E+04</td><td></td><td>(K)INLEAAE</td><td>(K)I N|L/E/A A E|L/G/E I S D I H/T K(L)</td><td></td><td>158.72</td><td>618.326</td><td>1852.965</td><td>-0.0016</td><td>-0.9</td><td>4.4</td><td>84268.2</td><td>6.32</td><td>HUMAN</td><td>Q9UHD2</td><td>Serine/threonine-protein kinase TBK1</td></tr>
<tr><td>3357</td><td>dirMS_041712_NAR_50mM_minus2.7200.7392.3</td><td>3</td><td>14.58</td><td>1</td><td>4.76E+06</td><td>M227m M</td><td>(R)mQKEIT.</td><td>(R)m Q K E I T\A|L|A|P/S T m K(I)</td><td>m:Oxidized methionine</td><td>108.5</td><td>527.6049</td><td>1548.812</td><td>31.9877</td><td>-1.3</td><td>8.35</td><td>32616.5</td><td>5.18</td><td>HUMAN</td><td>A6NL76</td><td>Actin, alpha skeletal muscle</td></tr>
<tr><td>3358</td><td>dirMS_041712_NAR_60mM_minus.13284.13567.3</td><td>3</td><td>14.58</td><td>1</td><td>5.68E+05</td><td></td><td>(R)KLVIIEGI</td><td>(R)K\L V\I\I|E|G/D L/E/R(T)</td><td></td><td>135.13</td><td>428.9221</td><td>1284.752</td><td>-0.0004</td><td>-0.3</td><td>4.68</td><td>33450.2</td><td>4.73</td><td>HUMAN</td><td>J3KN67</td><td>Tropomyosin alpha-3 chain</td></tr>
<tr><td>3359</td><td>dirMS_041712_NAR_20mM_plus.7410.7410.2</td><td>2</td><td>14.58</td><td>0</td><td>2.86E+05</td><td></td><td>(K)TGQAPG</td><td>(K)T G/Q|A|P/G Y S Y T/A/A N K(N)</td><td></td><td>119.52</td><td>714.8394</td><td>1428.675</td><td>-0.0039</td><td>-2.7</td><td>8.16</td><td>11447.2</td><td>9.81</td><td>HUMAN</td><td>C9JFR7</td><td>Cytochrome c (Fragment)</td></tr>
<tr><td>3360</td><td>dirMS_041712_NAR_60mM_plus.8343.8343.3</td><td>3</td><td>14.58</td><td>1</td><td>6.44E+05</td><td></td><td>(K)EPLYEKD</td><td>(K)E P L/Y\E K/D S/S/V/A A/R(F)</td><td></td><td>102.25</td><td>488.9148</td><td>1464.733</td><td>-0.0031</td><td>-2.1</td><td>4.68</td><td>26283.8</td><td>8.96</td><td>HUMAN</td><td>O43809</td><td>Cleavage and polyadenylation specificity factor subunit 5</td></tr>
<tr><td>3361</td><td>dirMS_041712_NAR_60mM_plus.8432.8432.3</td><td>3</td><td>14.58</td><td>1</td><td>1.94E+05</td><td></td><td>(R)TKSQGG</td><td>(R)T K\S Q G G E|P T/Y/N\V|A V/G R(A)</td><td></td><td>102.22</td><td>555.2829</td><td>1663.84</td><td>-0.0057</td><td>-3.4</td><td>8.26</td><td>9954.3</td><td>9.57</td><td>HUMAN</td><td>C9J0J7</td><td>Profilin-2</td></tr>
<tr><td>3362</td><td>dirMS_041712_NAR_120mM_minus.11364.11431.3</td><td>3</td><td>14.57</td><td>1</td><td>4.08E+05</td><td></td><td>(K)VIADNV</td><td>(K)V/I|A/D/N V/K/D W/S K(V)</td><td></td><td>116.48</td><td>425.5623</td><td>1274.674</td><td>-0.0016</td><td>-1.2</td><td>5.93</td><td>31075.7</td><td>5.65</td><td>HUMAN</td><td>P60174</td><td>Triosephosphate isomerase</td></tr>
<tr><td>3363</td><td>dirMS_041712_NAR_50mM_minus2.7165.7165.3</td><td>3</td><td>14.57</td><td>0</td><td>3.33E+04</td><td></td><td>(K)IITGKPSC</td><td>(K)I/I|T/G/K/P S G A I/S G V Q K(A)</td><td></td><td>107.57</td><td>485.9536</td><td>1455.853</td><td>-0.0067</td><td>-4.6</td><td>10</td><td>84268.2</td><td>6.32</td><td>HUMAN</td><td>Q9UHD2</td><td>Serine/threonine-protein kinase TBK1</td></tr>
<tr><td>3364</td><td>dirMS_041712_NAR_60mM_minus.12011.12011.3</td><td>3</td><td>14.57</td><td>1</td><td>2.51E+05</td><td></td><td>(R)GLAGLG</td><td>(R)G L|A G L/G D/V A E V R K(S)</td><td></td><td>127.02</td><td>428.9126</td><td>1284.727</td><td>-0.0038</td><td>-2.9</td><td>6.07</td><td>97378.8</td><td>6.4</td><td>HUMAN</td><td>P11216</td><td>Glycogen phosphorylase, brain form</td></tr>
<tr><td>3365</td><td>dirMS_041712_NAR_30mM_plus2.18509.18509.2</td><td>2</td><td>14.57</td><td>0</td><td>1.54E+05</td><td></td><td>(R)AMLDQI</td><td>(R)A M|L|D/Q/L/M/G/T S/R(D)</td><td></td><td>150.35</td><td>611.8</td><td>1222.592</td><td>0.0009</td><td>0.7</td><td>5.88</td><td>5509.4</td><td>10.84</td><td>HUMAN</td><td>F8WB41</td><td>Putative RNA-binding protein Luc7-like 2</td></tr>
<tr><td>3366</td><td>dirMS_041712_NAR_30mM_plus2.9044.9074.2</td><td>2</td><td>14.57</td><td>0</td><td>1.78E+05</td><td></td><td>(R)LELQGPI</td><td>(R)L/E|L|Q/G/P/R(G)</td><td></td><td>100.93</td><td>406.7314</td><td>812.462</td><td>-0.007</td><td>-8.6</td><td>6</td><td>32503.1</td><td>5.29</td><td>HUMAN</td><td>H7BY16</td><td>Nucleolin (Fragment)</td></tr>
<tr><td>3367</td><td>dirMS_041712_NAR_120mM_minus.13892.13892.4</td><td>4</td><td>14.56</td><td>1</td><td>1.62E+06</td><td></td><td>(K)ASIHEAV</td><td>(K)A/S|I/H/E A W T D/G K E/A M/L K(H)</td><td></td><td>129.97</td><td>447.476</td><td>1786.879</td><td>0.0029</td><td>1.6</td><td>5.45</td><td>80254.2</td><td>5.05</td><td>HUMAN</td><td>D6PXK4</td><td>Alpha actinin 4 short isoform</td></tr>
<tr><td>3368</td><td>dirMS_041712_NAR_20mM_minus.14070.14070.3</td><td>3</td><td>14.56</td><td>0</td><td>6.38E+05</td><td></td><td>(K)SYELPDC</td><td>(K)S Y E\L|P\D G Q V/I|T/I/G N E R(F)</td><td></td><td>193.82</td><td>597.6345</td><td>1790.892</td><td>-0.003</td><td>-1.7</td><td>4.14</td><td>123097.1</td><td>5.83</td><td>HUMAN</td><td>A5A3E0</td><td>POTE ankyrin domain family member F</td></tr>
<tr><td>3369</td><td>dirMS_041712_NAR_40mM_minus2.14262.14262.3</td><td>3</td><td>14.56</td><td>0</td><td>1.90E+04</td><td></td><td>(R)VSAAVLI</td><td>(R)V/S/A/A\V/L/P L V/H/P L P E G L R(A)</td><td></td><td>167.98</td><td>590.0205</td><td>1768.048</td><td>-0.001</td><td>-0.6</td><td>6.72</td><td>63124.2</td><td>9.53</td><td>HUMAN</td><td>E7EVU5</td><td>Nuclear receptor corepressor 1 (Fragment)</td></tr>
<tr><td>3370</td><td>dirMS_041712_NAR_60mM_minus.10735.10735.3</td><td>3</td><td>14.56</td><td>0</td><td>5.06E+04</td><td></td><td>(R)AYADSY</td><td>(R)A Y|A/D/S/Y/Y Y/E D G G M K P R(V)</td><td></td><td>119.78</td><td>629.2712</td><td>1885.806</td><td>-0.0071</td><td>-3.8</td><td>4.56</td><td>35935.5</td><td>6.2</td><td>HUMAN</td><td>Q8IVM0</td><td>Coiled-coil domain-containing protein 50</td></tr>
<tr><td>3371</td><td>dirMS_041712_NAR_60mM_minus.10782.10782.3</td><td>3</td><td>14.56</td><td>0</td><td>5.06E+04</td><td></td><td>(R)AYADSY</td><td>(R)A Y|A/D/S Y/Y Y/E/D/G G M K P R(V)</td><td></td><td>119.78</td><td>629.2712</td><td>1885.806</td><td>-0.0071</td><td>-3.8</td><td>4.56</td><td>35935.5</td><td>6.2</td><td>HUMAN</td><td>Q8IVM0</td><td>Coiled-coil domain-containing protein 50</td></tr>
<tr><td>3372</td><td>dirMS_041712_NAR_120mM_plus.8348.8348.2</td><td>2</td><td>14.56</td><td>1</td><td>3.54E+05</td><td></td><td>(K)YEKDIAA</td><td>(K)Y|E/K/D/I A A Y R(A)</td><td></td><td>97.17</td><td>564.7869</td><td>1128.568</td><td>-0.0019</td><td>-1.7</td><td>6.07</td><td>25064.5</td><td>5.62</td><td>HUMAN</td><td>P09429</td><td>High mobility group protein B1</td></tr>
<tr><td>3373</td><td>dirMS_041712_NAR_20mM_plus.9447.9447.2</td><td>2</td><td>14.56</td><td>0</td><td>1.42E+05</td><td></td><td>(R)GTGIVSA</td><td>(R)G T G I/V|S A P V/P/K(K)</td><td></td><td>136.92</td><td>513.3057</td><td>1025.599</td><td>0.0052</td><td>5</td><td>8.75</td><td>21898</td><td>11.33</td><td>HUMAN</td><td>E9PM36</td><td>40S ribosomal protein S2</td></tr>
<tr><td>3374</td><td>dirMS_041712_NAR_30mM_plus2.13734.13824.2</td><td>2</td><td>14.56</td><td>0</td><td>6.83E+05</td><td></td><td>(K)TCTFILRI</td><td>(K)T/C|T/F/I/L/R(G)</td><td>C:Carbamidomethylation</td><td>125.53</td><td>455.7451</td><td>910.481</td><td>0.0014</td><td>1.6</td><td>9.41</td><td>46550.7</td><td>7.81</td><td>HUMAN</td><td>B7Z1C9</td><td>Chaperonin containing TCP1, subunit 7 (Eta), isoform CRA_a</td></tr>
</table>

<table>
<tr><th></th><th></th><th></th><th></th><th></th><th></th><th></th><th></th><th></th><th></th><th></th><th></th><th></th><th></th><th></th><th></th><th></th><th></th><th></th><th></th><th></th></tr>
<tr><td>3375</td><td>dirMS_041712_NAR_30mM_plus2.17780.18166.2</td><td>2</td><td>14.56</td><td>0</td><td>2.92E+06</td><td></td><td>(R)GFSLEEL</td><td>(R)G F|S|L/E/E/L/R(V)</td><td></td><td>147.47</td><td>475.7563</td><td>950.494</td><td>0.0112</td><td>11.7</td><td>4.53</td><td>18901.8</td><td>11.65</td><td>HUMAN</td><td>F5H1S2</td><td>60S ribosomal protein L13</td></tr>
<tr><td>3376</td><td>dirMS_041712_NAR_30mM_plus2.18458.18458.2</td><td>2</td><td>14.56</td><td>0</td><td>2.36E+05</td><td></td><td>(K)GNFNYII</td><td>(K)G N|F|N/Y|I/E/F/T/R(I)</td><td></td><td>150.27</td><td>630.8035</td><td>1260.601</td><td>-0.001</td><td>-0.8</td><td>6</td><td>20513.6</td><td>4.62</td><td>HUMAN</td><td>J3QRS3</td><td>Myosin regulatory light chain 12A</td></tr>
<tr><td>3377</td><td>dirMS_041712_NAR_30mM_plus2.18515.18515.2</td><td>2</td><td>14.56</td><td>0</td><td>6.31E+04</td><td></td><td>(R)FAQPGS</td><td>(R)F A/Q|P/G S F E Y E Y A M R(W)</td><td></td><td>150.32</td><td>848.3763</td><td>1695.747</td><td>-0.0018</td><td>-1.1</td><td>4.53</td><td>29678.2</td><td>9.57</td><td>HUMAN</td><td>C9JYS8</td><td>Non-POU domain-containing octamer-binding protein (Fragment)</td></tr>
<tr><td>3378</td><td>dirMS_041712_NAR_30mM_minus2.13828.13828.2</td><td>2</td><td>14.55</td><td>0</td><td>2.26E+05</td><td></td><td>(K)TYSVEYL</td><td>(K)T/Y|S/V/E Y L/P K(V)</td><td></td><td>128.3</td><td>550.2855</td><td>1099.567</td><td>-0.0033</td><td>-3</td><td>5.66</td><td>258215.9</td><td>5.48</td><td>HUMAN</td><td>E7EN95</td><td>Filamin-B</td></tr>
<tr><td>3379</td><td>dirMS_041712_NAR_50mM_minus2.7424.7424.3</td><td>3</td><td>14.55</td><td>0</td><td>1.91E+04</td><td></td><td>(R)AVEVQG</td><td>(R)A V|E|V Q/G P S L/E S G D/H G K(I)</td><td></td><td>109.23</td><td>537.2631</td><td>1609.782</td><td>-0.0069</td><td>-4.3</td><td>4.65</td><td>629603.7</td><td>5.8</td><td>HUMAN</td><td>Q09666</td><td>Neuroblast differentiation-associated protein AHNAK</td></tr>
<tr><td>3380</td><td>dirMS_041712_NAR_60mM_minus.14245.14479.3</td><td>3</td><td>14.55</td><td>1</td><td>1.30E+06</td><td></td><td>(R)MSMKE</td><td>(R)M/S\M K E V\D E\Q|M|L/N V/Q/N K(N)</td><td></td><td>140.47</td><td>641.9701</td><td>1923.897</td><td>-0.0015</td><td>-0.8</td><td>4.68</td><td>40069.4</td><td>4.83</td><td>HUMAN</td><td>E7EWR1</td><td>Tubulin beta chain</td></tr>
<tr><td>3381</td><td>dirMS_041712_NAR_20mM_plus.13567.13567.2</td><td>2</td><td>14.55</td><td>0</td><td>1.45E+05</td><td>M273m</td><td>(K)mISDAIF</td><td>(K)m I/S/D A/I/P/E/L K(A)</td><td>m:Oxidized methionine</td><td>165.55</td><td>566.7967</td><td>1116.597</td><td>15.9892</td><td>-5</td><td>4.37</td><td>31330.8</td><td>9.26</td><td>HUMAN</td><td>E9PDB2</td><td>Malate dehydrogenase, mitochondrial</td></tr>
<tr><td>3382</td><td>dirMS_041712_NAR_30mM_plus2.11539.11578.2</td><td>2</td><td>14.55</td><td>0</td><td>1.45E+05</td><td></td><td>(K)MLVIEQ</td><td>(K)M/L|V|I/E/Q/C K(N)</td><td>C:Carbamidomethylation</td><td>113.97</td><td>510.764</td><td>1020.522</td><td>-0.0009</td><td>-0.9</td><td>5.75</td><td>49982</td><td>5.86</td><td>HUMAN</td><td>B4DYD8</td><td>T-complex protein 1 subunit epsilon</td></tr>
<tr><td>3383</td><td>dirMS_041712_NAR_30mM_plus2.12967.13278.2</td><td>2</td><td>14.55</td><td>0</td><td>1.15E+06</td><td></td><td>(K)LVIITAG</td><td>(K)L/V|I|I/T/A/G A/R(Q)</td><td></td><td>122.35</td><td>457.2931</td><td>913.583</td><td>-0.004</td><td>-4.4</td><td>9.75</td><td>12539.4</td><td>5.78</td><td>HUMAN</td><td>F5GXH2</td><td>L-lactate dehydrogenase A chain (Fragment)</td></tr>
<tr><td>3384</td><td>dirMS_041712_NAR_30mM_plus2.14481.14752.2</td><td>2</td><td>14.55</td><td>0</td><td>6.95E+05</td><td></td><td>(R)SLSAIYLI</td><td>(R)S L|S|A|I/Y/L E/K(L)</td><td></td><td>129.82</td><td>512.2888</td><td>1023.572</td><td>-0.0018</td><td>-1.7</td><td>5.72</td><td>533778</td><td>5.74</td><td>HUMAN</td><td>Q15149</td><td>Plectin</td></tr>
<tr><td>3385</td><td>dirMS_041712_NAR_30mM_plus2.14567.14735.2</td><td>2</td><td>14.55</td><td>0</td><td>2.53E+05</td><td></td><td>(R)SIGEYDV</td><td>(R)S I|G|E/Y/D|V L/R(G)</td><td></td><td>129.85</td><td>526.2726</td><td>1051.542</td><td>-0.0039</td><td>-3.7</td><td>4.37</td><td>470342.8</td><td>6.83</td><td>HUMAN</td><td>E7EUY0</td><td>DNA-dependent protein kinase catalytic subunit</td></tr>
<tr><td>3386</td><td>dirMS_041712_NAR_30mM_plus2.18527.18527.2</td><td>2</td><td>14.55</td><td>0</td><td>2.03E+05</td><td></td><td>(R)AVLNPL</td><td>(R)A V|L/N|P L/C Q V/D/Y R(A)</td><td>C:Carbamidomethylation</td><td>150.18</td><td>724.3703</td><td>1447.736</td><td>-0.0029</td><td>-2</td><td>5.88</td><td>83880.6</td><td>7.33</td><td>HUMAN</td><td>F5H365</td><td>Protein transport protein Sec23A</td></tr>
<tr><td>3387</td><td>dirMS_041712_NAR_30mM_plus2.7432.7453.2</td><td>2</td><td>14.55</td><td>0</td><td>1.06E+06</td><td></td><td>(R)IADGYE</td><td>(R)I/A|D|G/Y/E/Q/A A/R(V)</td><td></td><td>93.37</td><td>547.2595</td><td>1093.527</td><td>-0.0155</td><td>-14.2</td><td>4.37</td><td>49982</td><td>5.86</td><td>HUMAN</td><td>B4DYD8</td><td>T-complex protein 1 subunit epsilon</td></tr>
<tr><td>3388</td><td>dirMS_041712_NAR_40mM_plus2.16881.17057.3</td><td>3</td><td>14.55</td><td>1</td><td>3.08E+05</td><td></td><td>(R)LFVGNLI</td><td>(R)L/F|V/G/N/L/P/T D I T E E D F K R(L)</td><td></td><td>166.38</td><td>665.3502</td><td>1994.023</td><td>0.0131</td><td>6.6</td><td>4.32</td><td>58856.7</td><td>6.26</td><td>HUMAN</td><td>Q8WXF1</td><td>Paraspeckle component 1</td></tr>
<tr><td>3389</td><td>dirMS_041712_NAR_60mM_plus.13187.13630.3</td><td>3</td><td>14.55</td><td>1</td><td>1.02E+06</td><td></td><td>(R)ISVYYNE</td><td>(R)I/S|V/Y/Y N E\A T/G G K Y V/P R(A)</td><td></td><td>125.95</td><td>606.3113</td><td>1816.923</td><td>-0.0035</td><td>-1.9</td><td>8.43</td><td>27549.9</td><td>4.39</td><td>HUMAN</td><td>E9PBJ4</td><td>Tubulin beta chain</td></tr>
<tr><td>3390</td><td>dirMS_041712_NAR_60mM_plus.17557.17557.3</td><td>3</td><td>14.55</td><td>1</td><td>1.76E+05</td><td></td><td>(R)RTVQSLI</td><td>(R)R T V Q S\L|E|I/D|L D S M/R(N)</td><td></td><td>148.8</td><td>554.954</td><td>1662.848</td><td>-0.0005</td><td>-0.3</td><td>4.56</td><td>43773.3</td><td>5.26</td><td>HUMAN</td><td>F8VZY9</td><td>Keratin, type I cytoskeletal 18</td></tr>
<tr><td>3391</td><td>dirMS_041712_NAR_120mM_minus.17743.17743.3</td><td>3</td><td>14.54</td><td>1</td><td>1.85E+05</td><td></td><td>(R)AGKLDP</td><td>(R)A G K/L D|P H/L/V L D Q L/R(C)</td><td></td><td>151.48</td><td>525.6385</td><td>1574.901</td><td>-0.0004</td><td>-0.2</td><td>6.79</td><td>233493.8</td><td>5.45</td><td>HUMAN</td><td>F8VTL3</td><td>Myosin-10</td></tr>
<tr><td>3392</td><td>dirMS_041712_NAR_50mM_minus2.14877.14877.3</td><td>3</td><td>14.54</td><td>1</td><td>2.84E+05</td><td></td><td>(R)EAVTEIL</td><td>(R)E A\V/T/E I/L/G I/E P D R E K(G)</td><td></td><td>152.37</td><td>566.9683</td><td>1698.891</td><td>-0.0005</td><td>-0.3</td><td>4.25</td><td>25685.9</td><td>6.4</td><td>HUMAN</td><td>E9PGT1</td><td>Translin</td></tr>
<tr><td>3393</td><td>dirMS_041712_NAR_120mM_plus.14519.15064.3</td><td>3</td><td>14.54</td><td>1</td><td>3.52E+06</td><td></td><td>(R)KFLDGIY</td><td>(R)K\F\L\D\G\I|Y|V/S/E/K(G)</td><td></td><td>131.25</td><td>433.5709</td><td>1298.699</td><td>-0.0009</td><td>-0.7</td><td>6.07</td><td>20931.1</td><td>10.28</td><td>HUMAN</td><td>D6RAN4</td><td>60S ribosomal protein L9 (Fragment)</td></tr>
<tr><td>3394</td><td>dirMS_041712_NAR_30mM_plus2.12376.12885.2</td><td>2</td><td>14.54</td><td>0</td><td>7.81E+05</td><td></td><td>(K)SIYYITGI</td><td>(K)S I|Y|Y|I/T/G E/S K(E)</td><td></td><td>119.27</td><td>580.796</td><td>1160.583</td><td>0.0013</td><td>1.2</td><td>5.72</td><td>16789.1</td><td>8.77</td><td>HUMAN</td><td>H0Y5S7</td><td>Heat shock protein HSP 90-beta (Fragment)</td></tr>
<tr><td>3395</td><td>dirMS_041712_NAR_30mM_plus2.7580.7602.2</td><td>2</td><td>14.54</td><td>0</td><td>1.24E+05</td><td></td><td>(K)SAAMTL</td><td>(K)S A|A|M/T/L/N E/R(F)</td><td></td><td>93.52</td><td>496.7467</td><td>992.483</td><td>0.0032</td><td>3.2</td><td>5.72</td><td>83344.9</td><td>10.18</td><td>HUMAN</td><td>E9PK09</td><td>Bcl-2-associated transcription factor 1 (Fragment)</td></tr>
<tr><td>3396</td><td>dirMS_041712_NAR_50mM_plus2.10119.10236.3</td><td>3</td><td>14.54</td><td>0</td><td>6.15E+05</td><td></td><td>(K)VGQEIE</td><td>(K)V/G/Q/E/I E/V/R P G I V/S/K(D)</td><td></td><td>121.05</td><td>504.2907</td><td>1510.859</td><td>-0.0012</td><td>-0.8</td><td>6.11</td><td>51679.1</td><td>9.11</td><td>HUMAN</td><td>P41091</td><td>Eukaryotic translation initiation factor 2 subunit 3</td></tr>
<tr><td>3397</td><td>dirMS_041712_NAR_50mM_plus2.18520.18627.3</td><td>3</td><td>14.54</td><td>1</td><td>5.52E+05</td><td></td><td>(K)NAAGAL</td><td>(K)N A|A G/A/L/D L L/K E/L/K(N)</td><td></td><td>165.92</td><td>452.601</td><td>1355.789</td><td>-0.0008</td><td>-0.6</td><td>6.07</td><td>13786.8</td><td>9.33</td><td>HUMAN</td><td>B7Z4S1</td><td>Transcription elongation factor A protein 1</td></tr>
<tr><td>3398</td><td>dirMS_041712_NAR_60mM_plus.12059.12138.3</td><td>3</td><td>14.54</td><td>1</td><td>1.53E+06</td><td>M136m</td><td>(K)VKEGm</td><td>(K)V K E G m\N\I|V|E\A/M/E/R(F)</td><td>m:Oxidized methionine</td><td>120.35</td><td>507.9187</td><td>1505.745</td><td>15.9965</td><td>1</td><td>4.79</td><td>18240.4</td><td>7.96</td><td>HUMAN</td><td>P62937</td><td>Peptidyl-prolyl cis-trans isomerase A</td></tr>
<tr><td>3399</td><td>dirMS_041712_NAR_60mM_plus.17939.17940.3</td><td>3</td><td>14.54</td><td>1</td><td>1.60E+05</td><td></td><td>(K)ILLAELE</td><td>(K)I/L|L/A E L/E Q L/K/G Q G K(S)</td><td></td><td>150.9</td><td>513.9717</td><td>1539.911</td><td>-0.0099</td><td>-6.4</td><td>6.14</td><td>49709.6</td><td>5.19</td><td>HUMAN</td><td>B0YJC4</td><td>Vimentin</td></tr>
<tr><td>3400</td><td>dirMS_041712_NAR_120mM_minus.11078.11140.3</td><td>3</td><td>14.53</td><td>1</td><td>7.77E+05</td><td></td><td>(R)RFDDAV</td><td>(R)R F D D\A|V|V|Q/S D/M/K(H)</td><td></td><td>115.05</td><td>470.8939</td><td>1410.668</td><td>-0.001</td><td>-0.7</td><td>4.43</td><td>20069.4</td><td>6.74</td><td>HUMAN</td><td>E9PK54</td><td>Heat shock cognate 71 kDa protein (Fragment)</td></tr>
<tr><td>3401</td><td>dirMS_041712_NAR_30mM_minus2.10978.10978.2</td><td>2</td><td>14.53</td><td>0</td><td>5.82E+05</td><td></td><td>(K)VAGQD</td><td>(K)V/A|G/Q/D/G S V/V/Q/F K(I)</td><td></td><td>113.8</td><td>617.824</td><td>1234.643</td><td>-0.0019</td><td>-1.5</td><td>5.81</td><td>17127.4</td><td>10.05</td><td>HUMAN</td><td>A8MU27</td><td>Small ubiquitin-related modifier 3</td></tr>
<tr><td>3402</td><td>dirMS_041712_NAR_120mM_plus.10368.10662.3</td><td>3</td><td>14.53</td><td>1</td><td>3.83E+05</td><td></td><td>(K)KGENYV</td><td>(K)K\G E N Y\V|Y|I|N/E/A T/R(H)</td><td></td><td>108.4</td><td>519.5943</td><td>1556.77</td><td>-0.002</td><td>-1.3</td><td>6.14</td><td>266161</td><td>5.68</td><td>HUMAN</td><td>E9PPU0</td><td>Epiplakin</td></tr>
<tr><td>3403</td><td>dirMS_041712_NAR_20mM_plus.18630.18630.2</td><td>2</td><td>14.53</td><td>0</td><td>2.78E+05</td><td></td><td>(K)LAVEALS</td><td>(K)L A/V/E A\L|S/S L/D/G D L A/G R(Y)</td><td></td><td>200.45</td><td>793.9214</td><td>1586.838</td><td>-0.0029</td><td>-1.8</td><td>4.03</td><td>24286.7</td><td>4.89</td><td>HUMAN</td><td>G3V4N7</td><td>Creatine kinase B-type (Fragment)</td></tr>
<tr><td>3404</td><td>dirMS_041712_NAR_30mM_plus2.5791.5854.2</td><td>2</td><td>14.53</td><td>0</td><td>3.52E+05</td><td></td><td>(K)YAEAVTI</td><td>(K)Y/A|E|A/V/T/R(A)</td><td></td><td>84.63</td><td>405.2125</td><td>809.415</td><td>0.0025</td><td>3.1</td><td>6</td><td>41827.5</td><td>7</td><td>HUMAN</td><td>B7Z7A9</td><td>Phosphoglycerate kinase</td></tr>
<tr><td>3405</td><td>dirMS_041712_NAR_40mM_plus2.18220.18411.3</td><td>3</td><td>14.53</td><td>1</td><td>2.86E+05</td><td></td><td>(R)VAAKLE</td><td>(R)V A A K L E\V\A|P|I/S D/I I A I K(S)</td><td></td><td>174.02</td><td>584.3591</td><td>1751.068</td><td>-0.005</td><td>-2.8</td><td>6.04</td><td>24273.5</td><td>8.98</td><td>HUMAN</td><td>H0YK49</td><td>Electron transfer flavoprotein subunit alpha, mitochondrial</td></tr>
<tr><td>3406</td><td>dirMS_041712_NAR_50mM_plus2.16005.16005.3</td><td>3</td><td>14.53</td><td>0</td><td>1.05E+06</td><td></td><td>(R)LSFQHD</td><td>(R)L/S/F Q H D|P E|T/S|V/L/V L R(K)</td><td></td><td>152.42</td><td>580.9801</td><td>1740.928</td><td>-0.0022</td><td>-1.2</td><td>5.32</td><td>96827.3</td><td>5.58</td><td>HUMAN</td><td>E9PKU7</td><td>Neutral alpha-glucosidase AB</td></tr>
<tr><td>3407</td><td>dirMS_041712_NAR_20mM_minus.12346.12346.2</td><td>2</td><td>14.52</td><td>0</td><td>7.12E+05</td><td>M185m</td><td>(K)LAVNm</td><td>(K)L A|V/N/m/V/P/F P R(L)</td><td>m:Oxidized methionine</td><td>172</td><td>580.3173</td><td>1143.634</td><td>15.993</td><td>-1.6</td><td>9.75</td><td>42230.8</td><td>4.77</td><td>HUMAN</td><td>A6NKZ8</td><td>Putative tubulin beta chain-like protein ENSP00000290377</td></tr>
<tr><td>3408</td><td>dirMS_041712_NAR_60mM_minus.7860.7943.3</td><td>3</td><td>14.52</td><td>1</td><td>5.22E+05</td><td></td><td>(K)IQDKEGI</td><td>(K)I Q\D/K E G/I|P/P D/Q Q R(L)</td><td></td><td>103.63</td><td>508.5978</td><td>1523.781</td><td>-0.0024</td><td>-1.6</td><td>4.56</td><td>17271.6</td><td>6.75</td><td>HUMAN</td><td>B4DV12</td><td>Ubiquitin</td></tr>
<tr><td>3409</td><td>dirMS_041712_NAR_20mM_plus.18720.18720.2</td><td>2</td><td>14.52</td><td>0</td><td>1.01E+05</td><td></td><td>(K)LTVYTTL</td><td>(K)L/T|V/Y/T/T/L I/D/V T K(G)</td><td></td><td>200.8</td><td>683.8948</td><td>1366.783</td><td>-0.0005</td><td>-0.4</td><td>5.83</td><td>42194.9</td><td>8.75</td><td>HUMAN</td><td>E7EMM4</td><td>Acid ceramidase</td></tr>
<tr><td>3410</td><td>dirMS_041712_NAR_30mM_plus2.20041.20041.2</td><td>2</td><td>14.52</td><td>0</td><td>6.71E+05</td><td></td><td>(K)SYELPD</td><td>(K)S Y E|L/P D/G Q/V I|T I|G N E/R(F)</td><td></td><td>159.7</td><td>895.9487</td><td>1790.892</td><td>-0.0018</td><td>-1</td><td>4.14</td><td>123097.1</td><td>5.83</td><td>HUMAN</td><td>A5A3E0</td><td>POTE ankyrin domain family member F</td></tr>
<tr><td>3411</td><td>dirMS_041712_NAR_30mM_plus2.6303.6303.2</td><td>2</td><td>14.52</td><td>0</td><td>2.56E+05</td><td></td><td>(K)SAVLQE</td><td>(K)S A/V/L|Q E A/R(I)</td><td></td><td>87.2</td><td>437.2414</td><td>873.479</td><td>-0.0033</td><td>-3.8</td><td>5.72</td><td>28218.2</td><td>9.35</td><td>HUMAN</td><td>F6X838</td><td>Coatomer subunit gamma-2</td></tr>
<tr><td>3412</td><td>dirMS_041712_NAR_60mM_plus.9432.9432.2</td><td>2</td><td>14.52</td><td>0</td><td>1.27E+06</td><td></td><td>(K)STAGDT</td><td>(K)S T/A/G D|T H L|G G E/D/F D|N/R(M)</td><td></td><td>107.4</td><td>846.3654</td><td>1691.726</td><td>-0.0021</td><td>-1.2</td><td>4.22</td><td>69032.8</td><td>5.37</td><td>HUMAN</td><td>E9PKE3</td><td>Heat shock cognate 71 kDa protein</td></tr>
<tr><td>3413</td><td>dirMS_041712_NAR_30mM_minus2.12905.13137.2</td><td>2</td><td>14.51</td><td>0</td><td>7.12E+05</td><td></td><td>(R)VNFAMI</td><td>(R)V/N|F|A/M/N V/G\K(A)</td><td></td><td>124.37</td><td>490.2513</td><td>979.503</td><td>-0.0076</td><td>-7.8</td><td>8.72</td><td>58506.4</td><td>8.38</td><td>HUMAN</td><td>P14618</td><td>Pyruvate kinase isozymes M1/M2</td></tr>
<tr><td>3414</td><td>dirMS_041712_NAR_50mM_minus2.18357.18357.3</td><td>3</td><td>14.51</td><td>0</td><td>4.62E+05</td><td></td><td>(K)ALIEVLQ</td><td>(K)A L|I/E/V L Q/P L I/A E H Q A R(R)</td><td></td><td>175.42</td><td>601.0139</td><td>1801.033</td><td>-0.0059</td><td>-3.3</td><td>5.4</td><td>53506.8</td><td>5.83</td><td>HUMAN</td><td>P23381</td><td>Tryptophan--tRNA ligase, cytoplasmic</td></tr>
<tr><td>3415</td><td>dirMS_041712_NAR_120mM_plus.19580.19728.3</td><td>3</td><td>14.51</td><td>0</td><td>8.75E+05</td><td></td><td>(R)NSVSNF</td><td>(R)N/S|V/S N F/L/H/S L E/R(G)</td><td></td><td>156.15</td><td>468.2345</td><td>1402.707</td><td>-0.0184</td><td>-13.1</td><td>6.75</td><td>267260.7</td><td>5.95</td><td>HUMAN</td><td>Q13085</td><td>Acetyl-CoA carboxylase 1</td></tr>
<tr><td>3416</td><td>dirMS_041712_NAR_30mM_plus2.12011.12011.2</td><td>2</td><td>14.51</td><td>0</td><td>6.83E+05</td><td></td><td>(K)CGDLVF</td><td>(K)C/G|D|L/V/F\A/K(M)</td><td>C:Carbamidomethylation</td><td>116.12</td><td>455.2298</td><td>909.45</td><td>0.0025</td><td>2.7</td><td>5.84</td><td>26901.9</td><td>4.7</td><td>HUMAN</td><td>P51858</td><td>Hepatoma-derived growth factor</td></tr>
<tr><td>3417</td><td>dirMS_041712_NAR_30mM_plus2.12242.12391.2</td><td>2</td><td>14.51</td><td>0</td><td>8.43E+05</td><td></td><td>(K)GLLSAE</td><td>(K)G L|L|S/A/E/V A/R(L)</td><td></td><td>117.6</td><td>458.2693</td><td>915.526</td><td>0.0055</td><td>6</td><td>6</td><td>533778</td><td>5.74</td><td>HUMAN</td><td>Q15149</td><td>Plectin</td></tr>
<tr><td>3418</td><td>dirMS_041712_NAR_30mM_plus2.19225.19754.2</td><td>2</td><td>14.51</td><td>0</td><td>4.11E+06</td><td></td><td>(K)TLGILGL</td><td>(K)T/L|G|I|L/G/L/G R(I)</td><td></td><td>156.62</td><td>450.2857</td><td>899.567</td><td>-0.0032</td><td>-3.5</td><td>9.41</td><td>57391.2</td><td>6.29</td><td>HUMAN</td><td>O43175</td><td>D-3-phosphoglycerate dehydrogenase</td></tr>
<tr><td>3419</td><td>dirMS_041712_NAR_30mM_plus2.6964.6964.2</td><td>2</td><td>14.51</td><td>0</td><td>1.34E+05</td><td></td><td>(K)YTQSNS</td><td>(K)Y/T|Q S|N/S V/C Y A K(N)</td><td>C:Carbamidomethylation</td><td>91.15</td><td>660.7963</td><td>1320.589</td><td>-0.0036</td><td>-2.7</td><td>8.5</td><td>65036</td><td>6.27</td><td>HUMAN</td><td>E9PBU3</td><td>Phosphoribosylaminoimidazolecarboxamide formyltransferase</td></tr>
<tr><td>3420</td><td>dirMS_041712_NAR_60mM_plus.7904.7938.3</td><td>3</td><td>14.51</td><td>1</td><td>1.08E+05</td><td></td><td>(K)ALAAGG</td><td>(K)A/L|A|A|G/G/Y/D V/E K N N S R(I)</td><td></td><td>99.43</td><td>522.2648</td><td>1564.771</td><td>0.0084</td><td>5.4</td><td>6.11</td><td>22579.8</td><td>10.91</td><td>HUMAN</td><td>P16401</td><td>Histone H1.5</td></tr>
<tr><td>3421</td><td>dirMS_041712_NAR_30mM_minus2.18575.18575.2</td><td>2</td><td>14.5</td><td>0</td><td>3.87E+05</td><td></td><td>(R)QAQEYE</td><td>(R)Q A/Q|E|Y/E A/L/L/N I K(V)</td><td></td><td>154.83</td><td>710.3759</td><td>1419.748</td><td>-0.0033</td><td>-2.3</td><td>4.53</td><td>43773.3</td><td>5.26</td><td>HUMAN</td><td>F8VZY9</td><td>Keratin, type I cytoskeletal 18</td></tr>
<tr><td>3422</td><td>dirMS_041712_NAR_40mM_minus2.8727.8820.3</td><td>3</td><td>14.5</td><td>1</td><td>2.33E+05</td><td></td><td>(R)IVLANDI</td><td>(R)I V|L/A/N/D P D/A D/R L A V/A E/K(Q)</td><td></td><td>134.3</td><td>603.9966</td><td>1809.97</td><td>0.0047</td><td>2.6</td><td>4.23</td><td>32837.5</td><td>6.16</td><td>HUMAN</td><td>B4E0G8</td><td>Phosphoglucomutase-2</td></tr>
<tr><td>3423</td><td>dirMS_041712_NAR_50mM_minus2.13258.13258.3</td><td>3</td><td>14.5</td><td>0</td><td>2.61E+05</td><td></td><td>(K)VTHAVV</td><td>(K)V T H A V\V T\V/P A|Y/F/N D/A Q R(Q)</td><td></td><td>143.18</td><td>629.9954</td><td>1887.971</td><td>0.0005</td><td>0.3</td><td>6.71</td><td>72445.7</td><td>5.07</td><td>HUMAN</td><td>P11021</td><td>78 kDa glucose-regulated protein</td></tr>
<tr><td>3424</td><td>dirMS_041712_NAR_50mM_minus2.7898.7898.3</td><td>3</td><td>14.5</td><td>1</td><td>1.50E+05</td><td></td><td>(K)TAVCDIF</td><td>(K)T/A|V C D/I/P/P R G L K(M)</td><td>C:Carbamidomethylation</td><td>111.93</td><td>442.9126</td><td>1326.72</td><td>0.0034</td><td>2.6</td><td>8.41</td><td>50199.4</td><td>4.75</td><td>HUMAN</td><td>A6NNZ2</td><td>Tubulin beta-8 chain-like protein LOC260334</td></tr>
<tr><td>3425</td><td>dirMS_041712_NAR_60mM_minus.15988.16407.3</td><td>3</td><td>14.5</td><td>1</td><td>5.82E+05</td><td></td><td>(K)LKLSILYF</td><td>(K)L K L S I\L|Y|P/A/T T G/R(N)</td><td></td><td>150.5</td><td>478.2883</td><td>1432.852</td><td>-0.0019</td><td>-1.3</td><td>9.99</td><td>25148.6</td><td>6</td><td>HUMAN</td><td>P30041</td><td>Peroxiredoxin-6</td></tr>
<tr><td>3426</td><td>dirMS_041712_NAR_60mM_minus.16939.16939.3</td><td>3</td><td>14.5</td><td>1</td><td>2.24E+05</td><td></td><td>(R)KGFSEGI</td><td>(R)K G F S E G L\W/E|I|E N N P\T V|K(A)</td><td></td><td>153.83</td><td>649.9986</td><td>1947.981</td><td>0.0002</td><td>0.1</td><td>4.79</td><td>23020.5</td><td>4.53</td><td>HUMAN</td><td>A8K8G0</td><td>Hepatoma-derived growth factor</td></tr>
<tr><td>3427</td><td>dirMS_041712_NAR_60mM_minus.18252.18252.3</td><td>3</td><td>14.5</td><td>1</td><td>1.37E+05</td><td></td><td>(R)SELELTL</td><td>(R)S E|L/E L/T L/G K L/E Q V/R(S)</td><td></td><td>163.05</td><td>538.9724</td><td>1614.906</td><td>-0.0035</td><td>-2.1</td><td>4.78</td><td>533778</td><td>5.74</td><td>HUMAN</td><td>Q15149</td><td>Plectin</td></tr>
<tr><td>3428</td><td>dirMS_041712_NAR_120mM_plus.11500.12033.3</td><td>3</td><td>14.5</td><td>1</td><td>2.54E+06</td><td></td><td>(R)FPGQLN</td><td>(R)F/P/G Q/L/N/A/D/L R K(L)</td><td></td><td>113.82</td><td>420.2353</td><td>1258.69</td><td>0.0011</td><td>0.9</td><td>8.75</td><td>42230.8</td><td>4.77</td><td>HUMAN</td><td>A6NKZ8</td><td>Putative tubulin beta chain-like protein ENSP00000290377</td></tr>
<tr><td>3429</td><td>dirMS_041712_NAR_60mM_plus.9508.9683.3</td><td>3</td><td>14.5</td><td>1</td><td>1.92E+06</td><td></td><td>(K)LTRDET</td><td>(K)L T R D E T\N|Y|G|I/P Q/R(A)</td><td></td><td>107.8</td><td>521.6038</td><td>1562.792</td><td>0.0047</td><td>3</td><td>6.07</td><td>13083.6</td><td>6.26</td><td>HUMAN</td><td>D6R909</td><td>Guanine nucleotide-binding protein subunit beta-2-like 1</td></tr>
<tr><td>3430</td><td>dirMS_041712_NAR_20mM_minus.7047.7047.2</td><td>2</td><td>14.49</td><td>0</td><td>4.77E+05</td><td>M281m</td><td>(R)AEAESm</td><td>(R)A E|A/E/S m/Y Q I/K(Y)</td><td>m:Oxidized methionine</td><td>117.9</td><td>593.2759</td><td>1169.551</td><td>15.9938</td><td>-0.9</td><td>4.53</td><td>37296.3</td><td>5.3</td><td>HUMAN</td><td>F8VU64</td><td>Keratin, type II cytoskeletal 8</td></tr>
<tr><td>3431</td><td>dirMS_041712_NAR_30mM_minus2.18953.19212.2</td><td>2</td><td>14.49</td><td>0</td><td>9.54E+05</td><td></td><td>(K)MSIYQA</td><td>(K)M/S|I/Y/Q/A M W/K(G)</td><td></td><td>158.93</td><td>579.2789</td><td>1157.548</td><td>0.0023</td><td>2</td><td>8.34</td><td>266161</td><td>5.68</td><td>HUMAN</td><td>E9PPU0</td><td>Epiplakin</td></tr>
<tr><td>3432</td><td>dirMS_041712_NAR_30mM_minus2.8479.8499.2</td><td>2</td><td>14.49</td><td>0</td><td>2.60E+05</td><td></td><td>(R)YLSEVAS</td><td>(R)Y/L|S|E/V A|S G D/N K(Q)</td><td></td><td>100.93</td><td>591.7852</td><td>1182.564</td><td>-0.0006</td><td>-0.5</td><td>4.37</td><td>28196</td><td>4.76</td><td>HUMAN</td><td>P31946</td><td>14-3-3 protein beta/alpha</td></tr>
<tr><td>3433</td><td>dirMS_041712_NAR_60mM_minus.14928.14944.3</td><td>3</td><td>14.49</td><td>0</td><td>2.96E+05</td><td></td><td>(K)WIGLDL</td><td>(K)W/I|G L D L/S/N/G K/P R(D)</td><td></td><td>143.43</td><td>452.5849</td><td>1355.743</td><td>-0.0029</td><td>-2.1</td><td>8.75</td><td>44227.3</td><td>7.81</td><td>HUMAN</td><td>E7EQR6</td><td>T-complex protein 1 subunit alpha</td></tr>
<tr><td>3434</td><td>dirMS_041712_NAR_120mM_plus.11449.11513.4</td><td>4</td><td>14.49</td><td>2</td><td>9.39E+05</td><td></td><td>(K)KTGQPN</td><td>(K)K\T G Q|P\M|I/N L/Y/T D/R E T G K(L)</td><td></td><td>113.32</td><td>488.7518</td><td>1951.991</td><td>-0.0052</td><td>-2.7</td><td>8.5</td><td>53724.2</td><td>9.54</td><td>HUMAN</td><td>H3BPE7</td><td>RNA-binding protein FUS</td></tr>
<tr><td>3435</td><td>dirMS_041712_NAR_120mM_plus.23622.23915.3</td><td>3</td><td>14.49</td><td>0</td><td>8.56E+05</td><td></td><td>(R)LHFFMP</td><td>(R)L H\F\F\M|P G\F|A|P\L/T S R(G)</td><td></td><td>179.22</td><td>540.9492</td><td>1620.835</td><td>-0.0025</td><td>-1.5</td><td>9.75</td><td>42230.8</td><td>4.77</td><td>HUMAN</td><td>A6NKZ8</td><td>Putative tubulin beta chain-like protein ENSP00000290377</td></tr>
<tr><td>3436</td><td>dirMS_041712_NAR_30mM_plus2.17265.17265.2</td><td>2</td><td>14.49</td><td>0</td><td>3.05E+05</td><td></td><td>(K)TVFGVEI</td><td>(K)T V|F/G V|E P|D L T/R(E)</td><td></td><td>143.42</td><td>617.3263</td><td>1233.647</td><td>-0.0021</td><td>-1.7</td><td>4.37</td><td>53219.8</td><td>5.66</td><td>HUMAN</td><td>Q96KP4</td><td>Cytosolic non-specific dipeptidase</td></tr>
<tr><td>3437</td><td>dirMS_041712_NAR_50mM_plus2.11330.11448.3</td><td>3</td><td>14.49</td><td>0</td><td>5.94E+05</td><td></td><td>(R)ITLRPDP</td><td>(R)I/T|L R P D|P\L/P E/R(S)</td><td></td><td>127.35</td><td>436.2535</td><td>1306.748</td><td>-0.0018</td><td>-1.4</td><td>6.07</td><td>160853.1</td><td>6.11</td><td>HUMAN</td><td>F8VU51</td><td>YLP motif-containing protein 1</td></tr>
<tr><td>3438</td><td>dirMS_041712_NAR_60mM_plus.11263.11263.2</td><td>2</td><td>14.49</td><td>0</td><td>2.12E+06</td><td></td><td>(K)GIVEFAS</td><td>(K)G I|V|E|F/A/S/K/P/A A R(K)</td><td></td><td>116.18</td><td>623.3504</td><td>1245.695</td><td>-0.0015</td><td>-1.2</td><td>8.75</td><td>76262.2</td><td>9.53</td><td>HUMAN</td><td>P23246</td><td>Splicing factor, proline- and glutamine-rich</td></tr>
<tr><td>3439</td><td>dirMS_041712_NAR_40mM_minus2.11472.11472.3</td><td>3</td><td>14.48</td><td>0</td><td>1.87E+05</td><td></td><td>(K)LVTSIGD</td><td>(K)L V/T/S|I/G D V V/N H/D P V V G D R(L)</td><td></td><td>150.45</td><td>631.3335</td><td>1891.987</td><td>-0.0013</td><td>-0.7</td><td>4.41</td><td>27786.2</td><td>5.93</td><td>HUMAN</td><td>H0Y4Z6</td><td>Phosphorylase (Fragment)</td></tr>
<tr><td>3440</td><td>dirMS_041712_NAR_20mM_plus.10549.10549.2</td><td>2</td><td>14.48</td><td>0</td><td>2.94E+04</td><td></td><td>(R)SSSVGSS</td><td>(R)S S S|V/G S|S/S S/Y|P I S/P A\V|S/R(T)</td><td></td><td>145.53</td><td>877.9279</td><td>1754.855</td><td>-0.007</td><td>-4</td><td>8.46</td><td>533778</td><td>5.74</td><td>HUMAN</td><td>Q15149</td><td>Plectin</td></tr>
<tr><td>3441</td><td>dirMS_041712_NAR_30mM_plus2.8328.8594.2</td><td>2</td><td>14.48</td><td>0</td><td>1.08E+06</td><td></td><td>(K)TAVCDIF</td><td>(K)T A|V|C/D/I/P|P R(G)</td><td>C:Carbamidomethylation</td><td>99.48</td><td>514.7643</td><td>1028.519</td><td>0.002</td><td>1.9</td><td>5.5</td><td>50199.4</td><td>4.75</td><td>HUMAN</td><td>A6NNZ2</td><td>Tubulin beta-8 chain-like protein LOC260334</td></tr>
<tr><td>3442</td><td>dirMS_041712_NAR_40mM_plus2.5714.5714.2</td><td>2</td><td>14.48</td><td>0</td><td>1.12E+05</td><td></td><td>(R)FSSSSGY</td><td>(R)F/S|S/S S/G/Y/G/G/G S S R(V)</td><td></td><td>90.98</td><td>618.2686</td><td>1235.529</td><td>0.0012</td><td>1</td><td>8.75</td><td>62291.5</td><td>5.13</td><td>HUMAN</td><td>P35527</td><td>Keratin, type I cytoskeletal 9</td></tr>
<tr><td>3443</td><td>dirMS_041712_NAR_50mM_plus2.16853.16853.3</td><td>3</td><td>14.48</td><td>1</td><td>4.74E+05</td><td></td><td>(K)VLDSGA</td><td>(K)V L/D|S/G A/P I/K I/P V G P E T L G R(I)</td><td></td><td>156.7</td><td>640.3623</td><td>1919.096</td><td>-0.0237</td><td>-12.3</td><td>6.04</td><td>55307.5</td><td>5.25</td><td>HUMAN</td><td>F8VPV9</td><td>ATP synthase subunit beta</td></tr>
<tr><td>3444</td><td>dirMS_041712_NAR_60mM_plus.12082.12176.3</td><td>3</td><td>14.48</td><td>1</td><td>1.99E+06</td><td></td><td>(R)MQKEIT</td><td>(R)M Q K E I T A|L/A/P/S T M/K(I)</td><td></td><td>120.7</td><td>516.9402</td><td>1548.812</td><td>-0.0064</td><td>-4.1</td><td>8.35</td><td>32616.5</td><td>5.18</td><td>HUMAN</td><td>A6NL76</td><td>Actin, alpha skeletal muscle</td></tr>
<tr><td>3445</td><td>dirMS_041712_NAR_60mM_plus.7494.7535.3</td><td>3</td><td>14.48</td><td>1</td><td>3.06E+06</td><td></td><td>(K)QLELRQ</td><td>(K)Q/L/E/L/R Q/E E/E/R(R)</td><td></td><td>97.9</td><td>443.8995</td><td>1329.676</td><td>0.0082</td><td>6.2</td><td>4.49</td><td>43979.5</td><td>8.92</td><td>HUMAN</td><td>F5GYZ3</td><td>Non-POU domain-containing octamer-binding protein</td></tr>
<tr><td>3446</td><td>dirMS_041712_NAR_120mM_minus.13315.13315.4</td><td>4</td><td>14.47</td><td>1</td><td>9.47E+05</td><td></td><td>(K)SVHSSVI</td><td>(K)S V H S S V/P|L/L/N S K D/P I D R(I)</td><td></td><td>127.18</td><td>466.7525</td><td>1863.992</td><td>-0.0041</td><td>-2.2</td><td>6.47</td><td>54427.4</td><td>5.96</td><td>HUMAN</td><td>K4DID9</td><td>Acetyl-CoA carboxylase 1</td></tr>
<tr><td>3447</td><td>dirMS_041712_NAR_30mM_minus2.14632.14651.2</td><td>2</td><td>14.47</td><td>0</td><td>1.01E+05</td><td></td><td>(R)LDIDSPP</td><td>(R)L/D|I|D|S|P/P I T A R(N)</td><td></td><td>133.55</td><td>599.3249</td><td>1197.647</td><td>-0.0049</td><td>-4.1</td><td>4.21</td><td>5755.5</td><td>5.75</td><td>HUMAN</td><td>H3BN34</td><td>Pyruvate kinase isozymes M1/M2 (Fragment)</td></tr>
<tr><td>3448</td><td>dirMS_041712_NAR_50mM_minus2.10283.10303.3</td><td>3</td><td>14.47</td><td>1</td><td>5.22E+05</td><td></td><td>(K)IAQLEEC</td><td>(K)I/A|Q|L/E/E Q L/D N E T K/E R(Q)</td><td></td><td>127.12</td><td>605.9737</td><td>1815.908</td><td>-0.0018</td><td>-1</td><td>4.25</td><td>227783.5</td><td>5.5</td><td>HUMAN</td><td>P35579</td><td>Myosin-9</td></tr>
<tr><td>3449</td><td>dirMS_041712_NAR_60mM_minus.14525.14546.3</td><td>3</td><td>14.47</td><td>0</td><td>1.00E+06</td><td></td><td>(K)SLTNDW</td><td>(K)S L|T/N D/W/E/D H L/A/V K(H)</td><td></td><td>141.7</td><td>509.9191</td><td>1527.744</td><td>-0.0011</td><td>-0.7</td><td>4.53</td><td>85057.6</td><td>4.94</td><td>HUMAN</td><td>P07900</td><td>Heat shock protein HSP 90-alpha</td></tr>
<tr><td>3450</td><td>dirMS_041712_NAR_20mM_plus.17884.17884.2</td><td>2</td><td>14.47</td><td>0</td><td>1.27E+06</td><td></td><td>(R)YLTVAA</td><td>(R)Y/L/T/V/A A V F/R(G)</td><td></td><td>193.88</td><td>520.3009</td><td>1039.593</td><td>0.001</td><td>1</td><td>8.75</td><td>40069.4</td><td>4.83</td><td>HUMAN</td><td>E7EWR1</td><td>Tubulin beta chain</td></tr>
<tr><td>3451</td><td>dirMS_041712_NAR_30mM_plus2.10594.10627.2</td><td>2</td><td>14.47</td><td>0</td><td>3.95E+05</td><td></td><td>(K)TGTVSLI</td><td>(K)T G|T|V/S/L/E/V/R(L)</td><td></td><td>109.33</td><td>481.2676</td><td>961.531</td><td>-0.0034</td><td>-3.5</td><td>5.66</td><td>276046.1</td><td>6.01</td><td>HUMAN</td><td>P49327</td><td>Fatty acid synthase</td></tr>
<tr><td>3452</td><td>dirMS_041712_NAR_30mM_plus2.18231.18231.2</td><td>2</td><td>14.47</td><td>0</td><td>4.74E+05</td><td></td><td>(R)GYISPYF</td><td>(R)G Y|I|S|P Y F I N T S K(G)</td><td></td><td>149.62</td><td>695.3554</td><td>1389.705</td><td>-0.0014</td><td>-1</td><td>8.5</td><td>25225.7</td><td>9.52</td><td>HUMAN</td><td>E7ESH4</td><td>60 kDa heat shock protein, mitochondrial (Fragment)</td></tr>
<tr><td>3453</td><td>dirMS_041712_NAR_30mM_plus2.21971.22380.2</td><td>2</td><td>14.47</td><td>0</td><td>2.46E+06</td><td></td><td>(R)VIFGLFG</td><td>(R)V/I|F/G/L/F/G/K(T)</td><td></td><td>172.28</td><td>440.7682</td><td>880.529</td><td>0</td><td>0</td><td>8.72</td><td>23799.1</td><td>9.48</td><td>HUMAN</td><td>P23284</td><td>Peptidyl-prolyl cis-trans isomerase B</td></tr>
<tr><td>3454</td><td>dirMS_041712_NAR_30mM_plus2.7296.7314.2</td><td>2</td><td>14.47</td><td>0</td><td>7.19E+05</td><td></td><td>(K)STMQEL</td><td>(K)S T|M/Q|E|L/N/S R(L)</td><td></td><td>93.8</td><td>533.2595</td><td>1065.499</td><td>0.0124</td><td>11.6</td><td>5.72</td><td>62291.5</td><td>5.13</td><td>HUMAN</td><td>P35527</td><td>Keratin, type I cytoskeletal 9</td></tr>
<tr><td>3455</td><td>dirMS_041712_NAR_40mM_plus2.17588.17686.3</td><td>3</td><td>14.47</td><td>1</td><td>5.62E+05</td><td></td><td>(R)GLLPGLI</td><td>(R)G L|L\P|G/L L/P\A/P A D K L T/P K(G)</td><td></td><td>170.58</td><td>567.6811</td><td>1701.031</td><td>-0.0022</td><td>-1.3</td><td>8.59</td><td>153084.9</td><td>5.55</td><td>HUMAN</td><td>Q6P2E9</td><td>Enhancer of mRNA-decapping protein 4</td></tr>
<tr><td>3456</td><td>dirMS_041712_NAR_50mM_plus2.17645.17645.3</td><td>3</td><td>14.47</td><td>1</td><td>2.09E+05</td><td></td><td>(R)EVQLEEI</td><td>(R)E/V/Q/L/E/E L E A A R D L V S K(E)</td><td></td><td>160.73</td><td>610.3302</td><td>1828.965</td><td>0.011</td><td>6</td><td>4.25</td><td>45984.6</td><td>6.24</td><td>HUMAN</td><td>B4E1G6</td><td>Galactokinase</td></tr>
<tr><td>3457</td><td>dirMS_041712_NAR_50mM_plus2.7080.7080.3</td><td>3</td><td>14.47</td><td>1</td><td>3.75E+04</td><td></td><td>(R)ASVDTK</td><td>(R)A/S/V/D\T K E A\E G A/P Q V E\A G K(R)</td><td></td><td>103.02</td><td>596.2908</td><td>1786.882</td><td>-0.0239</td><td>-13.4</td><td>4.41</td><td>54214.9</td><td>5.66</td><td>HUMAN</td><td>C9J813</td><td>Caldesmon (Fragment)</td></tr>
<tr><td>3458</td><td>dirMS_041712_NAR_50mM_plus2.8769.8769.3</td><td>3</td><td>14.47</td><td>1</td><td>2.21E+05</td><td></td><td>(K)TTSPPE</td><td>(K)T T|S/P P E V/S G Y S Y E K/T E/R(S)</td><td></td><td>113.33</td><td>644.3093</td><td>1930.903</td><td>0.0105</td><td>5.4</td><td>4.78</td><td>271827.6</td><td>4.73</td><td>HUMAN</td><td>P46821</td><td>Microtubule-associated protein 1B</td></tr>
<tr><td>3459</td><td>dirMS_041712_NAR_60mM_plus.17185.17300.3</td><td>3</td><td>14.47</td><td>0</td><td>2.04E+05</td><td></td><td>(K)THLPGF</td><td>(K)T\H L\P G\F\V|E|Q/A E/A L/K(A)</td><td></td><td>147.35</td><td>513.9442</td><td>1539.817</td><td>0.0015</td><td>1</td><td>5.37</td><td>22314.2</td><td>9.39</td><td>HUMAN</td><td>P30044</td><td>Peroxiredoxin-5, mitochondrial</td></tr>
<tr><td>3460</td><td>dirMS_041712_NAR_120mM_minus.6799.7173.3</td><td>3</td><td>14.46</td><td>1</td><td>2.14E+07</td><td></td><td>(R)TKTEISEI</td><td>(R)T\K\T E\I|S/E/M/N/R(N)</td><td></td><td>92.57</td><td>403.5363</td><td>1208.594</td><td>0.0004</td><td>0.3</td><td>5.81</td><td>37296.3</td><td>5.3</td><td>HUMAN</td><td>F8VU64</td><td>Keratin, type II cytoskeletal 8</td></tr>
<tr><td>3461</td><td>dirMS_041712_NAR_120mM_minus.8100.8100.3</td><td>3</td><td>14.46</td><td>2</td><td>1.87E+05</td><td></td><td>(R)ASVDTK</td><td>(R)A S V/D T\K/E A E G A/P Q/V/E/A G K R(L)</td><td></td><td>98.33</td><td>648.3315</td><td>1942.983</td><td>-0.0029</td><td>-1.5</td><td>4.87</td><td>54214.9</td><td>5.66</td><td>HUMAN</td><td>C9J813</td><td>Caldesmon (Fragment)</td></tr>
<tr><td>3462</td><td>dirMS_041712_NAR_20mM_minus.13750.13750.2</td><td>2</td><td>14.46</td><td>0</td><td>3.99E+05</td><td></td><td>(K)LGGSAV</td><td>(K)L G\G S A|V/I/S L|E/G K|P L(-)</td><td></td><td>188.68</td><td>670.8912</td><td>1340.778</td><td>-0.0033</td><td>-2.4</td><td>6</td><td>22956</td><td>8.8</td><td>HUMAN</td><td>E9PK25</td><td>Cofilin-1</td></tr>
<tr><td>3463</td><td>dirMS_041712_NAR_30mM_minus2.16452.16452.2</td><td>2</td><td>14.46</td><td>0</td><td>2.53E+05</td><td></td><td>(R)ASLEAAI</td><td>(R)A S L\E/A|A/I/A/D A/E Q R(G)</td><td></td><td>143.58</td><td>672.8404</td><td>1344.675</td><td>-0.0019</td><td>-1.4</td><td>4.14</td><td>56607.4</td><td>5.37</td><td>HUMAN</td><td>F8VXB4</td><td>Keratin, type II cytoskeletal 8</td></tr>
<tr><td>3464</td><td>dirMS_041712_NAR_60mM_minus.10422.10422.3</td><td>3</td><td>14.46</td><td>1</td><td>2.88E+04</td><td></td><td>(K)SPFEVS</td><td>(K)S/P|F/E/V S V D K A/Q\G D/A S K(V)</td><td></td><td>117.73</td><td>555.6083</td><td>1664.813</td><td>-0.0023</td><td>-1.4</td><td>4.56</td><td>258215.9</td><td>5.48</td><td>HUMAN</td><td>E7EN95</td><td>Filamin-B</td></tr>
<tr><td>3465</td><td>dirMS_041712_NAR_30mM_plus2.14749.14811.2</td><td>2</td><td>14.46</td><td>0</td><td>7.63E+05</td><td></td><td>(K)FVMQEE</td><td>(K)F V|M|Q/E/E/F/S R(D)</td><td></td><td>130.1</td><td>586.778</td><td>1172.541</td><td>0.0083</td><td>7</td><td>4.53</td><td>55361.8</td><td>6.42</td><td>HUMAN</td><td>G5EA52</td><td>Protein disulfide isomerase family A, member 3, isoform CRA_b</td></tr>
<tr><td>3466</td><td>dirMS_041712_NAR_60mM_plus.13138.13138.3</td><td>3</td><td>14.46</td><td>1</td><td>7.13E+05</td><td></td><td>(R)INVYYNI</td><td>(R)I/N/V/Y/Y N/E A T G G K Y/V P R(A)</td><td></td><td>126</td><td>615.3146</td><td>1843.934</td><td>-0.0045</td><td>-2.4</td><td>8.43</td><td>50286.6</td><td>4.79</td><td>HUMAN</td><td>P68371</td><td>Tubulin beta-4B chain</td></tr>
<tr><td>3467</td><td>dirMS_041712_NAR_60mM_plus.21561.21679.3</td><td>3</td><td>14.46</td><td>1</td><td>7.16E+05</td><td></td><td>(K)EIIDLVLI</td><td>(K)E I/I/D/L/V/L D/R I R(K)</td><td></td><td>180.75</td><td>452.2733</td><td>1354.805</td><td>0.0001</td><td>0.1</td><td>4.56</td><td>58642</td><td>4.95</td><td>HUMAN</td><td>F5H5D3</td><td>Tubulin alpha-1C chain</td></tr>
<tr><td>3468</td><td>dirMS_041712_NAR_50mM_minus2.10539.10683.3</td><td>3</td><td>14.45</td><td>1</td><td>3.44E+05</td><td></td><td>(K)NQVAM</td><td>(K)N Q|V|A|M/N/P/T/N T/V F D A K R(L)</td><td></td><td>128.8</td><td>602.6379</td><td>1805.896</td><td>0.0029</td><td>1.6</td><td>8.75</td><td>20069.4</td><td>6.74</td><td>HUMAN</td><td>E9PK54</td><td>Heat shock cognate 71 kDa protein (Fragment)</td></tr>
<tr><td>3469</td><td>dirMS_041712_NAR_50mM_minus2.14498.14559.3</td><td>3</td><td>14.45</td><td>0</td><td>4.40E+05</td><td></td><td>(R)QVLIASH</td><td>(R)Q/V|L/I/A S/H|L/P S Y E L R(H)</td><td></td><td>150.95</td><td>542.6354</td><td>1625.901</td><td>-0.0093</td><td>-5.7</td><td>6.75</td><td>267260.7</td><td>5.95</td><td>HUMAN</td><td>Q13085</td><td>Acetyl-CoA carboxylase 1</td></tr>
<tr><td>3470</td><td>dirMS_041712_NAR_50mM_minus2.14687.14687.3</td><td>3</td><td>14.45</td><td>0</td><td>6.40E+04</td><td></td><td>(K)VYTDVQ</td><td>(K)V Y|T/D/V/Q Q/V/A S S L T H P R(F)</td><td></td><td>151.52</td><td>600.976</td><td>1800.924</td><td>-0.0104</td><td>-5.8</td><td>6.71</td><td>74230.8</td><td>5.34</td><td>HUMAN</td><td>B1AH89</td><td>Tubulin tyrosine ligase-like family, member 12</td></tr>
<tr><td>3471</td><td>dirMS_041712_NAR_60mM_minus.7117.7117.3</td><td>3</td><td>14.45</td><td>1</td><td>3.04E+05</td><td></td><td>(R)ELFESST</td><td>(R)E L|F/E/S S T N N N K D/T K(Q)</td><td></td><td>98.72</td><td>542.9247</td><td>1626.761</td><td>-0.001</td><td>-0.6</td><td>4.68</td><td>150202.8</td><td>9.1</td><td>HUMAN</td><td>E7EW20</td><td>Unconventional myosin-VI</td></tr>
<tr><td>3472</td><td>dirMS_041712_NAR_120mM_plus.15911.15911.4</td><td>4</td><td>14.45</td><td>1</td><td>8.06E+05</td><td></td><td>(K)THLPGF</td><td>(K)T H\L P G\F|V/E Q/A E/A L K A/K(G)</td><td></td><td>137.05</td><td>435.4926</td><td>1738.949</td><td>-0.0001</td><td>0</td><td>6.42</td><td>22314.2</td><td>9.39</td><td>HUMAN</td><td>P30044</td><td>Peroxiredoxin-5, mitochondrial</td></tr>
<tr><td>3473</td><td>dirMS_041712_NAR_20mM_plus.13896.13896.2</td><td>2</td><td>14.45</td><td>0</td><td>6.90E+04</td><td></td><td>(K)LLDLVQ</td><td>(K)L L|D|L\V|Q Q/S C N Y/K(Q)</td><td>C:Carbamidomethylation</td><td>167.73</td><td>740.8757</td><td>1480.746</td><td>-0.0023</td><td>-1.6</td><td>5.83</td><td>14855.1</td><td>9.26</td><td>HUMAN</td><td>B1AHD1</td><td>NHP2 non-histone chromosome protein 2-like 1 (S. cerevisiae)</td></tr>
<tr><td>3474</td><td>dirMS_041712_NAR_20mM_plus.15021.15021.2</td><td>2</td><td>14.45</td><td>0</td><td>1.07E+05</td><td></td><td>(R)ILNVPQI</td><td>(R)I L/N|V/P Q/E L Y E/K(G)</td><td></td><td>174.67</td><td>673.3693</td><td>1345.736</td><td>-0.0049</td><td>-3.6</td><td>4.53</td><td>267260.7</td><td>5.95</td><td>HUMAN</td><td>Q13085</td><td>Acetyl-CoA carboxylase 1</td></tr>
<tr><td>3475</td><td>dirMS_041712_NAR_50mM_plus2.6104.6104.3</td><td>3</td><td>14.45</td><td>0</td><td>1.85E+05</td><td></td><td>(R)ATAPQT</td><td>(R)A/T|A/P Q T Q/H/V/S P M R(Q)</td><td></td><td>96.3</td><td>475.2418</td><td>1423.711</td><td>-0.0002</td><td>-0.1</td><td>9.8</td><td>29184.1</td><td>4.91</td><td>HUMAN</td><td>E9PBQ9</td><td>Elongation factor 1-delta</td></tr>
<tr><td>3476</td><td>dirMS_041712_NAR_20mM_minus.7854.7854.2</td><td>2</td><td>14.44</td><td>0</td><td>7.81E+04</td><td>M406m</td><td>(R)EYQELm</td><td>(R)E/Y/Q/E/L/m N V/K(L)</td><td>m:Oxidized methionine</td><td>128.13</td><td>585.2788</td><td>1153.556</td><td>15.9945</td><td>-0.3</td><td>4.53</td><td>56607.4</td><td>5.37</td><td>HUMAN</td><td>F8VXB4</td><td>Keratin, type II cytoskeletal 8</td></tr>
<tr><td>3477</td><td>dirMS_041712_NAR_60mM_minus.10613.10613.3</td><td>3</td><td>14.44</td><td>0</td><td>1.44E+05</td><td>M271m</td><td>(R)DLNYCF</td><td>(R)D L|N/Y/C/F S G m S/D/H R(Y)</td><td>C:Carbamidomethylation m:Oxid</td><td>119.2</td><td>539.8863</td><td>1601.647</td><td>15.9972</td><td>1.4</td><td>5.21</td><td>47371.6</td><td>5.93</td><td>HUMAN</td><td>E9PCY7</td><td>Heterogeneous nuclear ribonucleoprotein H</td></tr>
<tr><td>3478</td><td>dirMS_041712_NAR_120mM_plus.4550.4660.3</td><td>3</td><td>14.44</td><td>0</td><td>8.70E+05</td><td></td><td>(R)YINTEH</td><td>(R)Y/I/N/T E/H/G G S Q A R(F)</td><td></td><td>74.22</td><td>444.8857</td><td>1332.629</td><td>0.0134</td><td>10.1</td><td>6.75</td><td>87447.6</td><td>6.44</td><td>HUMAN</td><td>Q15437</td><td>Protein transport protein Sec23B</td></tr>
<tr><td>3479</td><td>dirMS_041712_NAR_120mM_plus.9978.10127.2</td><td>2</td><td>14.44</td><td>0</td><td>5.50E+05</td><td></td><td>(K)HLTDAY</td><td>(K)H|L|T D|A\Y|F/K(K)</td><td></td><td>105.57</td><td>497.7567</td><td>994.499</td><td>0.0069</td><td>6.9</td><td>6.74</td><td>25891.4</td><td>10.67</td><td>HUMAN</td><td>F8W181</td><td>60S ribosomal protein L6 (Fragment)</td></tr>
<tr><td>3480</td><td>dirMS_041712_NAR_30mM_plus2.10258.10558.2</td><td>2</td><td>14.44</td><td>0</td><td>1.57E+06</td><td></td><td>(K)VIPELNG</td><td>(K)V I|P/E L/N/G/K(L)</td><td></td><td>108.53</td><td>435.2581</td><td>869.509</td><td>-0.0002</td><td>-0.2</td><td>5.97</td><td>31718.5</td><td>7.17</td><td>HUMAN</td><td>E7EUT4</td><td>Glyceraldehyde-3-phosphate dehydrogenase</td></tr>
<tr><td>3481</td><td>dirMS_041712_NAR_30mM_plus2.9400.9807.2</td><td>2</td><td>14.44</td><td>0</td><td>1.62E+06</td><td></td><td>(K)LIEVDDE</td><td>(K)L/I|E|V/D D/E/R(K)</td><td></td><td>105.03</td><td>494.7532</td><td>988.495</td><td>0.0046</td><td>4.6</td><td>3.91</td><td>10471.9</td><td>5.4</td><td>HUMAN</td><td>A2A3R7</td><td>40S ribosomal protein S6</td></tr>
<tr><td>3482</td><td>dirMS_041712_NAR_40mM_plus2.10941.10992.3</td><td>3</td><td>14.44</td><td>1</td><td>3.24E+05</td><td></td><td>(R)DVVICP</td><td>(R)D V|V|I/C/P D A S L E D A K K(E)</td><td>C:Carbamidomethylation</td><td>130.9</td><td>553.9465</td><td>1659.826</td><td>-0.0009</td><td>-0.5</td><td>4.23</td><td>18080.7</td><td>7.92</td><td>HUMAN</td><td>K7ELW0</td><td>Protein DJ-1</td></tr>
<tr><td>3483</td><td>dirMS_041712_NAR_50mM_plus2.12618.12618.3</td><td>3</td><td>14.44</td><td>1</td><td>5.35E+05</td><td></td><td>(K)FSIEDLK</td><td>(K)F/S|I/E D/L/K/A Q P K(Q)</td><td></td><td>134.4</td><td>425.8966</td><td>1275.694</td><td>-0.0191</td><td>-15</td><td>6.07</td><td>13579.2</td><td>9.99</td><td>HUMAN</td><td>E9PPQ6</td><td>Thymocyte nuclear protein 1 (Fragment)</td></tr>
</table>

<table>
<tr><th></th><th></th><th></th><th></th><th></th><th></th><th></th><th></th><th></th><th></th><th></th><th></th><th></th><th></th><th></th><th></th><th></th><th></th><th></th><th></th><th></th></tr>
<tr><td>3484</td><td>dirMS_041712_NAR_120mM_minus.13316.13316.4</td><td>4</td><td>14.43</td><td>1</td><td>9.47E+05</td><td></td><td>(K)SVHSSVI</td><td>(K)S\V H S\S V/P L/L/N S K D/P I D R(I)</td><td></td><td>127.18</td><td>466.7525</td><td>1863.992</td><td>-0.0041</td><td>-2.2</td><td>6.47</td><td>54427.4</td><td>5.96</td><td>HUMAN</td><td>K4DID9</td><td>Acetyl-CoA carboxylase 1</td></tr>
<tr><td>3485</td><td>dirMS_041712_NAR_120mM_minus.8377.8394.3</td><td>3</td><td>14.43</td><td>0</td><td>1.85E+05</td><td></td><td>(K)LFQECCI</td><td>(K)L/F|Q/E/C C/P H S T D/R(V)</td><td>C:Carbamidomethylation</td><td>99.98</td><td>517.2227</td><td>1549.652</td><td>0.0013</td><td>0.9</td><td>5.32</td><td>51260.6</td><td>5.39</td><td>HUMAN</td><td>P61978</td><td>Heterogeneous nuclear ribonucleoprotein K</td></tr>
<tr><td>3486</td><td>dirMS_041712_NAR_30mM_minus2.12471.12638.2</td><td>2</td><td>14.43</td><td>0</td><td>2.70E+05</td><td></td><td>(R)TLSGME</td><td>(R)T L|S|G/M/E/S/Y C V/R(A)</td><td>C:Carbamidomethylation</td><td>122.03</td><td>651.795</td><td>1302.582</td><td>0.001</td><td>0.8</td><td>5.66</td><td>42755</td><td>7.81</td><td>HUMAN</td><td>B7Z2F4</td><td>T-complex protein 1 subunit delta</td></tr>
<tr><td>3487</td><td>dirMS_041712_NAR_30mM_minus2.16157.16157.2</td><td>2</td><td>14.43</td><td>0</td><td>1.21E+05</td><td></td><td>(K)SSEELLS</td><td>(K)S S E/E/L|L|S/A/L Q/K(K)</td><td></td><td>141.47</td><td>602.816</td><td>1204.642</td><td>-0.0172</td><td>-14.3</td><td>4.53</td><td>150202.8</td><td>9.1</td><td>HUMAN</td><td>E7EW20</td><td>Unconventional myosin-VI</td></tr>
<tr><td>3488</td><td>dirMS_041712_NAR_30mM_minus2.19598.19598.2</td><td>2</td><td>14.43</td><td>0</td><td>2.54E+05</td><td></td><td>(R)YLTVAA</td><td>(R)Y L/T|V/A A/V F/R(G)</td><td></td><td>162.82</td><td>520.2998</td><td>1039.593</td><td>-0.0012</td><td>-1.1</td><td>8.75</td><td>40069.4</td><td>4.83</td><td>HUMAN</td><td>E7EWR1</td><td>Tubulin beta chain</td></tr>
<tr><td>3489</td><td>dirMS_041712_NAR_40mM_minus2.14320.14410.3</td><td>3</td><td>14.43</td><td>0</td><td>2.72E+05</td><td></td><td>(R)DVCTELI</td><td>(R)D V|C|T/E L/L/P L I K/P Q/G R(V)</td><td>C:Carbamidomethylation</td><td>168.95</td><td>580.3211</td><td>1738.952</td><td>-0.0033</td><td>-1.9</td><td>6.07</td><td>30659.7</td><td>9.02</td><td>HUMAN</td><td>P16152</td><td>Carbonyl reductase [NADPH] 1</td></tr>
<tr><td>3490</td><td>dirMS_041712_NAR_40mM_minus2.8129.8142.3</td><td>3</td><td>14.43</td><td>1</td><td>3.18E+05</td><td></td><td>(R)DGKLVS</td><td>(R)D/G K L V\S\E|S/S D|V/L/P K(-)</td><td></td><td>128.2</td><td>491.9311</td><td>1473.78</td><td>-0.0008</td><td>-0.5</td><td>4.56</td><td>56607.4</td><td>5.37</td><td>HUMAN</td><td>F8VXB4</td><td>Keratin, type II cytoskeletal 8</td></tr>
<tr><td>3491</td><td>dirMS_041712_NAR_50mM_minus2.16531.16578.3</td><td>3</td><td>14.43</td><td>1</td><td>1.60E+05</td><td></td><td>(K)VVSSVL(</td><td>(K)V/V|S/S V L\Q/F G N I/S/F/K K(E)</td><td></td><td>162</td><td>551.6488</td><td>1652.937</td><td>-0.0052</td><td>-3.1</td><td>10</td><td>233493.8</td><td>5.45</td><td>HUMAN</td><td>F8VTL3</td><td>Myosin-10</td></tr>
<tr><td>3492</td><td>dirMS_041712_NAR_50mM_minus2.19068.19068.3</td><td>3</td><td>14.43</td><td>1</td><td>1.34E+06</td><td></td><td>(R)KEGGLG</td><td>(R)K\E G G L G P L N|I|P/L/L A\D V T R(R)</td><td></td><td>187.5</td><td>621.6941</td><td>1863.07</td><td>-0.0021</td><td>-1.1</td><td>6.07</td><td>15252.2</td><td>5.82</td><td>HUMAN</td><td>A6NIW5</td><td>Peroxiredoxin 2, isoform CRA_a</td></tr>
<tr><td>3493</td><td>dirMS_041712_NAR_20mM_plus.10450.10450.2</td><td>2</td><td>14.43</td><td>0</td><td>1.88E+05</td><td>M592m M</td><td>(R)mGPAm</td><td>(R)m G/P A/m/G/P A/L G A G I E/R(M)</td><td>m:Oxidized methionine</td><td>144.65</td><td>730.3568</td><td>1427.713</td><td>31.993</td><td>2.1</td><td>5.75</td><td>77799.7</td><td>9.09</td><td>HUMAN</td><td>P52272</td><td>Heterogeneous nuclear ribonucleoprotein M</td></tr>
<tr><td>3494</td><td>dirMS_041712_NAR_30mM_plus2.18567.18567.2</td><td>2</td><td>14.43</td><td>0</td><td>3.64E+05</td><td></td><td>(R)SSFYVN(</td><td>(R)S S F|Y/V/N G L/T\L/G G Q K(C)</td><td></td><td>150.97</td><td>735.8825</td><td>1470.759</td><td>-0.001</td><td>-0.7</td><td>8.31</td><td>17630.6</td><td>11.62</td><td>HUMAN</td><td>I3L3D5</td><td>Profilin-1 (Fragment)</td></tr>
<tr><td>3495</td><td>dirMS_041712_NAR_60mM_plus.6263.6263.3</td><td>3</td><td>14.43</td><td>1</td><td>3.09E+05</td><td></td><td>(R)GSCSTE</td><td>(R)G/S|C|S/T E V E K E T Q E K(M)</td><td>C:Carbamidomethylation</td><td>91.08</td><td>537.9088</td><td>1611.717</td><td>-0.0048</td><td>-3</td><td>4.49</td><td>13871.4</td><td>9.22</td><td>HUMAN</td><td>O75348</td><td>V-type proton ATPase subunit G 1</td></tr>
<tr><td>3496</td><td>dirMS_041712_NAR_120mM_minus.10031.10155.3</td><td>3</td><td>14.42</td><td>0</td><td>5.96E+05</td><td></td><td>(R)HGESAV</td><td>(R)H G E\S\A\W|N|L/E/N/R(F)</td><td></td><td>109.4</td><td>438.2098</td><td>1312.603</td><td>0.012</td><td>9.1</td><td>5.4</td><td>28917.5</td><td>6.68</td><td>HUMAN</td><td>P18669</td><td>Phosphoglycerate mutase 1</td></tr>
<tr><td>3497</td><td>dirMS_041712_NAR_20mM_minus.12054.12401.3</td><td>3</td><td>14.42</td><td>0</td><td>2.99E+06</td><td>M313m</td><td>(R)TVQSLEI</td><td>(R)T\V\Q\S L\E|I/D/L/D/S m/R(N)</td><td>m:Oxidized methionine</td><td>170.78</td><td>508.2517</td><td>1506.747</td><td>15.9937</td><td>-0.8</td><td>4.03</td><td>43773.3</td><td>5.26</td><td>HUMAN</td><td>F8VZY9</td><td>Keratin, type I cytoskeletal 18</td></tr>
<tr><td>3498</td><td>dirMS_041712_NAR_50mM_minus2.15925.15925.3</td><td>3</td><td>14.42</td><td>0</td><td>3.64E+05</td><td></td><td>(K)LYGPTNI</td><td>(K)L Y|G/P/T N F S/P I/I N H V A R(F)</td><td></td><td>158.22</td><td>600.3221</td><td>1798.96</td><td>-0.0081</td><td>-4.5</td><td>8.75</td><td>60985.3</td><td>5.6</td><td>HUMAN</td><td>O75131</td><td>Copine-3</td></tr>
<tr><td>3499</td><td>dirMS_041712_NAR_60mM_minus.5572.5572.3</td><td>3</td><td>14.42</td><td>1</td><td>1.23E+04</td><td></td><td>(K)LAGESES</td><td>(K)L/A/G|E/S E/S N L R/K(A)</td><td></td><td>87.08</td><td>401.8822</td><td>1203.633</td><td>-0.0007</td><td>-0.6</td><td>6.14</td><td>90004.9</td><td>5.14</td><td>HUMAN</td><td>P55072</td><td>Transitional endoplasmic reticulum ATPase</td></tr>
<tr><td>3500</td><td>dirMS_041712_NAR_120mM_plus.11105.11105.3</td><td>3</td><td>14.42</td><td>0</td><td>1.04E+06</td><td></td><td>(K)NFYVEH</td><td>(K)N F/Y V/E/H/P E V A R(L)</td><td></td><td>111.43</td><td>454.2277</td><td>1360.664</td><td>0.0041</td><td>3</td><td>5.4</td><td>73202</td><td>9.19</td><td>HUMAN</td><td>C9JMU5</td><td>Probable ATP-dependent RNA helicase DDX17</td></tr>
<tr><td>3501</td><td>dirMS_041712_NAR_30mM_plus2.19195.19641.2</td><td>2</td><td>14.42</td><td>0</td><td>1.20E+06</td><td></td><td>(R)LIDFLEC</td><td>(R)L/I|D|F|L/E/C G/K(T)</td><td>C:Carbamidomethylation</td><td>155.15</td><td>547.7808</td><td>1094.555</td><td>-0.0007</td><td>-0.7</td><td>4.37</td><td>61018.6</td><td>9.44</td><td>HUMAN</td><td>B4DLW8</td><td>Probable ATP-dependent RNA helicase DDX5</td></tr>
<tr><td>3502</td><td>dirMS_041712_NAR_30mM_plus2.5532.5590.2</td><td>2</td><td>14.42</td><td>0</td><td>2.38E+05</td><td>M154m M</td><td>(R)mGYmD</td><td>(R)m/G/Y|m|D|P/R(E)</td><td>m:Oxidized methionine</td><td>83.4</td><td>451.1814</td><td>869.364</td><td>31.9911</td><td>1.4</td><td>5.59</td><td>26419.2</td><td>5.1</td><td>HUMAN</td><td>H0Y9K7</td><td>Splicing factor, proline- and glutamine-rich (Fragment)</td></tr>
<tr><td>3503</td><td>dirMS_041712_NAR_40mM_plus2.12152.12152.3</td><td>3</td><td>14.42</td><td>1</td><td>2.35E+05</td><td></td><td>(R)VVDALG</td><td>(R)V/V/D A/L/G N A/I D G K G P I G S K(T)</td><td></td><td>137.17</td><td>570.9838</td><td>1710.939</td><td>-0.0016</td><td>-1</td><td>5.93</td><td>15338.4</td><td>5.51</td><td>HUMAN</td><td>K7EJP1</td><td>ATP synthase subunit alpha, mitochondrial (Fragment)</td></tr>
<tr><td>3504</td><td>dirMS_041712_NAR_40mM_plus2.14370.14370.3</td><td>3</td><td>14.42</td><td>1</td><td>4.53E+05</td><td></td><td>(R)IINEPTA</td><td>(R)I I|N/E/P T/A A A I A/Y G L D K/K(V)</td><td></td><td>150.6</td><td>596.6671</td><td>1787.99</td><td>-0.0034</td><td>-1.9</td><td>6.07</td><td>17973</td><td>5.16</td><td>HUMAN</td><td>E9PI65</td><td>Heat shock cognate 71 kDa protein (Fragment)</td></tr>
<tr><td>3505</td><td>dirMS_041712_NAR_60mM_plus.6445.6463.2</td><td>2</td><td>14.42</td><td>1</td><td>3.50E+05</td><td></td><td>(K)VQVEYK</td><td>(K)V/Q|V/E/Y K G E|T/K(S)</td><td></td><td>92.37</td><td>590.8148</td><td>1180.621</td><td>0.0015</td><td>1.3</td><td>6.11</td><td>20069.4</td><td>6.74</td><td>HUMAN</td><td>E9PK54</td><td>Heat shock cognate 71 kDa protein (Fragment)</td></tr>
<tr><td>3506</td><td>dirMS_041712_NAR_30mM_minus2.13245.13245.2</td><td>2</td><td>14.41</td><td>0</td><td>3.00E+05</td><td></td><td>(K)STYGWT</td><td>(K)S T|Y/G|W/T/A/N M E R(I)</td><td></td><td>125.7</td><td>658.2918</td><td>1315.574</td><td>0.0028</td><td>2.1</td><td>5.72</td><td>47825.1</td><td>5.07</td><td>HUMAN</td><td>Q58FG1</td><td>Putative heat shock protein HSP 90-alpha A4</td></tr>
<tr><td>3507</td><td>dirMS_041712_NAR_30mM_minus2.18823.18902.2</td><td>2</td><td>14.41</td><td>0</td><td>2.04E+05</td><td></td><td>(R)AIGIGAY</td><td>(R)A I|G|I|G/A/Y L V|R(L)</td><td></td><td>157.57</td><td>516.8122</td><td>1032.62</td><td>-0.0029</td><td>-2.8</td><td>8.79</td><td>9731</td><td>5.19</td><td>HUMAN</td><td>E9PEW7</td><td>Acetyl-CoA carboxylase 2</td></tr>
<tr><td>3508</td><td>dirMS_041712_NAR_50mM_minus2.12769.13106.3</td><td>3</td><td>14.41</td><td>0</td><td>4.17E+06</td><td>M84m</td><td>(K)YPIEHGI</td><td>(K)Y/P/I E H G I I|T/N/W|D/D m E K(I)</td><td>m:Oxidized methionine</td><td>141.65</td><td>659.6398</td><td>1960.911</td><td>15.9939</td><td>-0.5</td><td>4.31</td><td>32616.5</td><td>5.18</td><td>HUMAN</td><td>A6NL76</td><td>Actin, alpha skeletal muscle</td></tr>
<tr><td>3509</td><td>dirMS_041712_NAR_50mM_minus2.12837.12882.3</td><td>3</td><td>14.41</td><td>1</td><td>3.84E+05</td><td></td><td>(R)LSEDYG</td><td>(R)L/S|E/D/Y G V/L K/T D E G/I A/Y R(G)</td><td></td><td>141.48</td><td>643.6561</td><td>1928.96</td><td>-0.0063</td><td>-3.2</td><td>4.32</td><td>22062.7</td><td>5.66</td><td>HUMAN</td><td>P32119</td><td>Peroxiredoxin-2</td></tr>
<tr><td>3510</td><td>dirMS_041712_NAR_50mM_minus2.16836.16836.3</td><td>3</td><td>14.41</td><td>0</td><td>4.56E+05</td><td></td><td>(K)NILFVIT</td><td>(K)N I/L/F V/I/T K/P D V/Y/K(S)</td><td></td><td>164.1</td><td>517.2991</td><td>1549.899</td><td>-0.0161</td><td>-10.4</td><td>8.5</td><td>206102.3</td><td>9.72</td><td>HUMAN</td><td>E9PAV3</td><td>Nascent polypeptide-associated complex subunit alpha</td></tr>
<tr><td>3511</td><td>dirMS_041712_NAR_50mM_minus2.18163.18171.3</td><td>3</td><td>14.41</td><td>0</td><td>2.31E+05</td><td></td><td>(K)ILFIFIDSI</td><td>(K)I/L\F|I/F I/D/S D H T D N/Q R(I)</td><td></td><td>172.78</td><td>611.9745</td><td>1833.913</td><td>-0.004</td><td>-2.2</td><td>4.41</td><td>51524.1</td><td>4.82</td><td>HUMAN</td><td>F5H8J2</td><td>Protein disulfide-isomerase</td></tr>
<tr><td>3512</td><td>dirMS_041712_NAR_120mM_plus.6571.6571.3</td><td>3</td><td>14.41</td><td>1</td><td>1.74E+07</td><td></td><td>(R)TKTEISEI</td><td>(R)T K\T\E\I/S/E/M/N/R(N)</td><td></td><td>88.12</td><td>403.5365</td><td>1208.594</td><td>0.001</td><td>0.8</td><td>5.81</td><td>37296.3</td><td>5.3</td><td>HUMAN</td><td>F8VU64</td><td>Keratin, type II cytoskeletal 8</td></tr>
<tr><td>3513</td><td>dirMS_041712_NAR_30mM_plus2.20716.20716.2</td><td>2</td><td>14.41</td><td>0</td><td>8.74E+05</td><td></td><td>(R)TVQSLEI</td><td>(R)T V|Q/S/L/E/I\D L D S M R(N)</td><td></td><td>162.18</td><td>753.8768</td><td>1506.747</td><td>-0.0005</td><td>-0.3</td><td>4.03</td><td>43773.3</td><td>5.26</td><td>HUMAN</td><td>F8VZY9</td><td>Keratin, type I cytoskeletal 18</td></tr>
<tr><td>3514</td><td>dirMS_041712_NAR_30mM_plus2.6410.6410.2</td><td>2</td><td>14.41</td><td>0</td><td>2.52E+05</td><td></td><td>(K)GEGQLG</td><td>(K)G E/G/Q|L/G/P A E R(A)</td><td></td><td>87.53</td><td>507.2537</td><td>1013.501</td><td>-0.0009</td><td>-0.9</td><td>4.53</td><td>96306.4</td><td>6.41</td><td>HUMAN</td><td>P13639</td><td>Elongation factor 2</td></tr>
<tr><td>3515</td><td>dirMS_041712_NAR_30mM_plus2.7553.7553.2</td><td>2</td><td>14.41</td><td>0</td><td>2.40E+05</td><td></td><td>(R)VLANPG</td><td>(R)V L|A/N|P/G N S Q V A R(V)</td><td></td><td>93.82</td><td>613.3361</td><td>1225.665</td><td>0.0002</td><td>0.1</td><td>9.72</td><td>16598.6</td><td>5.48</td><td>HUMAN</td><td>J3QR48</td><td>Importin subunit beta-1 (Fragment)</td></tr>
<tr><td>3516</td><td>dirMS_041712_NAR_40mM_plus2.10748.11012.3</td><td>3</td><td>14.41</td><td>0</td><td>9.41E+05</td><td></td><td>(R)LLEAQA(</td><td>(R)L/L|E|A/Q A/C/T G G I/I/H/P T T G Q K(L)</td><td>C:Carbamidomethylation</td><td>130.33</td><td>665.6807</td><td>1995.033</td><td>-0.0052</td><td>-2.6</td><td>6.75</td><td>334221.3</td><td>6.44</td><td>HUMAN</td><td>P15924</td><td>Desmoplakin</td></tr>
<tr><td>3517</td><td>dirMS_041712_NAR_40mM_plus2.11309.11309.3</td><td>3</td><td>14.41</td><td>0</td><td>1.21E+05</td><td></td><td>(K)EAGELKI</td><td>(K)E A G E L/K P E E E I|T|V/G P V Q K(L)</td><td></td><td>132.8</td><td>651.6782</td><td>1953.017</td><td>0.0025</td><td>1.3</td><td>4.32</td><td>108281.4</td><td>5.72</td><td>HUMAN</td><td>P53618</td><td>Coatomer subunit beta</td></tr>
<tr><td>3518</td><td>dirMS_041712_NAR_40mM_plus2.11636.11636.3</td><td>3</td><td>14.41</td><td>1</td><td>2.52E+05</td><td></td><td>(R)IVAPGK(</td><td>(R)I/V|A|P G K G I L/A A D E S T G S I A K(R)</td><td></td><td>135.27</td><td>633.3568</td><td>1898.059</td><td>-0.0035</td><td>-1.8</td><td>6.07</td><td>30711.4</td><td>7.15</td><td>HUMAN</td><td>H3BPS8</td><td>Fructose-bisphosphate aldolase A (Fragment)</td></tr>
<tr><td>3519</td><td>dirMS_041712_NAR_50mM_plus2.7506.7647.3</td><td>3</td><td>14.41</td><td>1</td><td>3.52E+05</td><td></td><td>(R)VVASDK</td><td>(R)V/V|A/S D K/E/T Y E/L R(Y)</td><td></td><td>105.87</td><td>470.5836</td><td>1409.727</td><td>0.0092</td><td>6.5</td><td>4.68</td><td>14478.1</td><td>9.37</td><td>HUMAN</td><td>C9JYQ9</td><td>60S ribosomal protein L22-like 1</td></tr>
<tr><td>3520</td><td>dirMS_041712_NAR_60mM_plus.17857.17894.3</td><td>3</td><td>14.41</td><td>1</td><td>3.70E+05</td><td></td><td>(R)VVVDAL</td><td>(R)V/V|V/D/A/L/S G L K G D L A G/R(Y)</td><td></td><td>150.23</td><td>523.9678</td><td>1569.896</td><td>-0.007</td><td>-4.5</td><td>5.93</td><td>26949.2</td><td>7.99</td><td>HUMAN</td><td>C9JSQ1</td><td>Creatine kinase U-type, mitochondrial (Fragment)</td></tr>
<tr><td>3521</td><td>dirMS_041712_NAR_60mM_plus.20098.20118.3</td><td>3</td><td>14.41</td><td>1</td><td>3.34E+05</td><td></td><td>(K)GPVREG</td><td>(K)G P V R\E G D\V\L|T|L|L|E S E R(E)</td><td></td><td>165.02</td><td>590.6485</td><td>1769.939</td><td>-0.0083</td><td>-4.7</td><td>4.41</td><td>7898</td><td>11.1</td><td>HUMAN</td><td>P62857</td><td>40S ribosomal protein S28</td></tr>
<tr><td>3522</td><td>dirMS_041712_NAR_40mM_minus2.14843.14843.3</td><td>3</td><td>14.4</td><td>1</td><td>1.22E+06</td><td>M116m</td><td>(R)ELAILLG</td><td>(R)E L/A I/L|L/G m L D/P A E K D/E K(G)</td><td>m:Oxidized methionine</td><td>171.37</td><td>634.338</td><td>1884.999</td><td>16.0008</td><td>3.1</td><td>4.18</td><td>25148.6</td><td>6</td><td>HUMAN</td><td>P30041</td><td>Peroxiredoxin-6</td></tr>
<tr><td>3523</td><td>dirMS_041712_NAR_60mM_minus.7760.7838.3</td><td>3</td><td>14.4</td><td>1</td><td>4.17E+05</td><td></td><td>(K)KGTVEG</td><td>(K)K\G T V E G\F\E|P/A/D/N K(C)</td><td></td><td>103.35</td><td>464.5665</td><td>1391.68</td><td>0.0048</td><td>3.5</td><td>4.68</td><td>13286.2</td><td>9.99</td><td>HUMAN</td><td>H0YLA2</td><td>Signal recognition particle 14 kDa protein</td></tr>
<tr><td>3524</td><td>dirMS_041712_NAR_120mM_plus.19049.19049.4</td><td>4</td><td>14.4</td><td>0</td><td>9.03E+05</td><td></td><td>(R)EVPIPEH</td><td>(R)E V/P I/P E/H|I/D|I/Y\H L T/R(E)</td><td></td><td>153.55</td><td>458.7454</td><td>1831.97</td><td>-0.0103</td><td>-5.6</td><td>5.28</td><td>27043.5</td><td>6.34</td><td>HUMAN</td><td>C9JHK9</td><td>ATP-binding cassette sub-family F member 2 (Fragment)</td></tr>
<tr><td>3525</td><td>dirMS_041712_NAR_120mM_plus.23106.23106.3</td><td>3</td><td>14.4</td><td>1</td><td>4.13E+05</td><td></td><td>(K)GLAPDLI</td><td>(K)G L\A|P/D L/P E D L Y H/L I K/K(A)</td><td></td><td>174.18</td><td>608.0076</td><td>1822.011</td><td>-0.0027</td><td>-1.5</td><td>5.38</td><td>16789.5</td><td>10.63</td><td>HUMAN</td><td>J3KMX5</td><td>40S ribosomal protein S13</td></tr>
<tr><td>3526</td><td>dirMS_041712_NAR_120mM_plus.7624.7624.2</td><td>2</td><td>14.4</td><td>1</td><td>1.19E+06</td><td></td><td>(R)AQYDEL</td><td>(R)A/Q|Y/D/E L/A/R K(N)</td><td></td><td>93.32</td><td>547.2874</td><td>1093.564</td><td>0.0039</td><td>3.5</td><td>6.11</td><td>43773.3</td><td>5.26</td><td>HUMAN</td><td>F8VZY9</td><td>Keratin, type I cytoskeletal 18</td></tr>
<tr><td>3527</td><td>dirMS_041712_NAR_120mM_plus.9979.10174.2</td><td>2</td><td>14.4</td><td>0</td><td>4.61E+05</td><td></td><td>(R)VHIEIGP</td><td>(R)V/H|I|E|I|G/P D|G R(V)</td><td></td><td>105.95</td><td>546.7931</td><td>1092.58</td><td>-0.0007</td><td>-0.6</td><td>5.32</td><td>47371.6</td><td>5.93</td><td>HUMAN</td><td>E9PCY7</td><td>Heterogeneous nuclear ribonucleoprotein H</td></tr>
<tr><td>3528</td><td>dirMS_041712_NAR_20mM_plus.18821.18821.2</td><td>2</td><td>14.4</td><td>0</td><td>2.84E+05</td><td></td><td>(K)LAVEALS</td><td>(K)L A V|E A\L|S/S/L|D/G D L A G R(Y)</td><td></td><td>200.5</td><td>793.9214</td><td>1586.838</td><td>-0.0029</td><td>-1.8</td><td>4.03</td><td>24286.7</td><td>4.89</td><td>HUMAN</td><td>G3V4N7</td><td>Creatine kinase B-type (Fragment)</td></tr>
<tr><td>3529</td><td>dirMS_041712_NAR_30mM_plus2.10509.10547.2</td><td>2</td><td>14.4</td><td>0</td><td>3.85E+05</td><td></td><td>(R)DPYGFG</td><td>(R)D/P|Y|G|F G D/S R(D)</td><td></td><td>109.02</td><td>507.2274</td><td>1013.432</td><td>0.0152</td><td>15</td><td>4.21</td><td>65763.5</td><td>9.81</td><td>HUMAN</td><td>Q9HCD5</td><td>Nuclear receptor coactivator 5</td></tr>
<tr><td>3530</td><td>dirMS_041712_NAR_30mM_plus2.17911.18212.2</td><td>2</td><td>14.4</td><td>0</td><td>5.51E+05</td><td></td><td>(K)SFGLPSI</td><td>(K)S F|G|L|P/S/I G R(L)</td><td></td><td>147.78</td><td>467.2628</td><td>933.515</td><td>0.0031</td><td>3.3</td><td>9.47</td><td>80685.1</td><td>7.31</td><td>HUMAN</td><td>P05165</td><td>Propionyl-CoA carboxylase alpha chain, mitochondrial</td></tr>
<tr><td>3531</td><td>dirMS_041712_NAR_40mM_plus2.12832.12832.3</td><td>3</td><td>14.4</td><td>1</td><td>4.30E+05</td><td></td><td>(R)VLELVSI</td><td>(R)V L|E/L/V|S I T A N K N T\C P G D R(S)</td><td>C:Carbamidomethylation</td><td>142.67</td><td>663.0201</td><td>1987.028</td><td>0.018</td><td>9.1</td><td>6.04</td><td>45186.1</td><td>9.89</td><td>HUMAN</td><td>B4DLV4</td><td>Serine hydroxymethyltransferase</td></tr>
<tr><td>3532</td><td>dirMS_041712_NAR_40mM_plus2.7767.7767.2</td><td>2</td><td>14.4</td><td>0</td><td>8.63E+04</td><td></td><td>(R)FGQGG</td><td>(R)F G Q|G/G/A/G/P V|G G Q G P R(G)</td><td></td><td>105.47</td><td>671.3368</td><td>1341.666</td><td>0.0005</td><td>0.4</td><td>9.75</td><td>76262.2</td><td>9.53</td><td>HUMAN</td><td>P23246</td><td>Splicing factor, proline- and glutamine-rich</td></tr>
<tr><td>3533</td><td>dirMS_041712_NAR_60mM_plus.16565.16565.3</td><td>3</td><td>14.4</td><td>1</td><td>3.69E+05</td><td></td><td>(-)MKLNISF</td><td>(-)M K/L N I S/F|P/A|T G C Q K(L)</td><td>C:Carbamidomethylation</td><td>142.85</td><td>532.2733</td><td>1594.808</td><td>-0.0026</td><td>-1.7</td><td>10</td><td>10471.9</td><td>5.4</td><td>HUMAN</td><td>A2A3R7</td><td>40S ribosomal protein S6</td></tr>
<tr><td>3534</td><td>dirMS_041712_NAR_120mM_minus.4529.4529.2</td><td>2</td><td>14.39</td><td>0</td><td>7.49E+05</td><td></td><td>(R)HSDGNL</td><td>(R)H/S|D G/N/L|C V|K(V)</td><td>C:Carbamidomethylation</td><td>73.97</td><td>515.2423</td><td>1029.478</td><td>-0.0009</td><td>-0.9</td><td>6.74</td><td>7781.8</td><td>6.82</td><td>HUMAN</td><td>E9PE20</td><td>Signal recognition particle 9 kDa protein</td></tr>
<tr><td>3535</td><td>dirMS_041712_NAR_50mM_minus2.19054.19060.3</td><td>3</td><td>14.39</td><td>1</td><td>1.34E+06</td><td></td><td>(R)KEGGLG</td><td>(R)K\E G G L G P\L|N\I|P/L/L/A D V T R(R)</td><td></td><td>187.5</td><td>621.6941</td><td>1863.07</td><td>-0.0021</td><td>-1.1</td><td>6.07</td><td>15252.2</td><td>5.82</td><td>HUMAN</td><td>A6NIW5</td><td>Peroxiredoxin 2, isoform CRA_a</td></tr>
<tr><td>3536</td><td>dirMS_041712_NAR_20mM_plus.17725.17919.2</td><td>2</td><td>14.39</td><td>0</td><td>2.00E+05</td><td></td><td>(R)YISPDQL</td><td>(R)Y/I|S|P D Q L/A D/L Y K(S)</td><td></td><td>194.23</td><td>713.3667</td><td>1425.726</td><td>0.0001</td><td>0.1</td><td>4.21</td><td>47510.4</td><td>7.04</td><td>HUMAN</td><td>P06733</td><td>Alpha-enolase</td></tr>
<tr><td>3537</td><td>dirMS_041712_NAR_30mM_plus2.7924.7924.2</td><td>2</td><td>14.39</td><td>0</td><td>9.94E+05</td><td></td><td>(R)DYDDM</td><td>(R)D Y|D|D M/S/P/R(R)</td><td></td><td>96.1</td><td>499.6972</td><td>998.388</td><td>-0.0013</td><td>-1.3</td><td>3.93</td><td>51260.6</td><td>5.39</td><td>HUMAN</td><td>P61978</td><td>Heterogeneous nuclear ribonucleoprotein K</td></tr>
<tr><td>3538</td><td>dirMS_041712_NAR_50mM_plus2.18338.18563.3</td><td>3</td><td>14.39</td><td>1</td><td>3.49E+05</td><td></td><td>(R)GDSVIV</td><td>(R)G D/S/V/I|V V/L/R N P L I/A/G K(-)</td><td></td><td>165.37</td><td>550.9999</td><td>1650.99</td><td>-0.005</td><td>-3</td><td>8.75</td><td>6144.3</td><td>10.23</td><td>HUMAN</td><td>K7EJB5</td><td>Small nuclear ribonucleoprotein Sm D2</td></tr>
<tr><td>3539</td><td>dirMS_041712_NAR_60mM_plus.10318.10362.3</td><td>3</td><td>14.39</td><td>0</td><td>1.35E+04</td><td></td><td>(K)AFFESHI</td><td>(K)A/F|F/E/S H/P A/P S A E/R(T)</td><td></td><td>113.22</td><td>482.5659</td><td>1445.681</td><td>0.0023</td><td>1.6</td><td>5.4</td><td>95141.7</td><td>5.22</td><td>HUMAN</td><td>B7Z463</td><td>Puromycin-sensitive aminopeptidase</td></tr>
<tr><td>3540</td><td>dirMS_041712_NAR_60mM_plus.18613.18613.3</td><td>3</td><td>14.39</td><td>1</td><td>1.09E+05</td><td></td><td>(K)MFIGGL</td><td>(K)M F/I/G G L/S/W D T T K K(D)</td><td></td><td>154.55</td><td>495.2592</td><td>1483.761</td><td>0.0017</td><td>1.1</td><td>8.35</td><td>33782.7</td><td>8.78</td><td>HUMAN</td><td>B4DTC3</td><td>Heterogeneous nuclear ribonucleoprotein D0</td></tr>
<tr><td>3541</td><td>dirMS_041712_NAR_20mM_plus.15789.15789.3</td><td>3</td><td>14.38</td><td>0</td><td>1.53E+06</td><td>M73m</td><td>(R)AILVDLE</td><td>(R)A I|L|V D L E/P/G T m D S V R(S)</td><td>m:Oxidized methionine</td><td>179.32</td><td>544.6139</td><td>1615.836</td><td>15.9912</td><td>-2.3</td><td>4.03</td><td>27549.9</td><td>4.39</td><td>HUMAN</td><td>E9PBJ4</td><td>Tubulin beta chain</td></tr>
<tr><td>3542</td><td>dirMS_041712_NAR_20mM_plus.18994.18994.3</td><td>3</td><td>14.38</td><td>0</td><td>1.11E+05</td><td></td><td>(R)VCTLAII</td><td>(R)V C T L A I\I|D/P/G D S/D I I R(S)</td><td>C:Carbamidomethylation</td><td>202.28</td><td>586.641</td><td>1757.91</td><td>-0.0018</td><td>-1</td><td>3.93</td><td>12826.8</td><td>9.82</td><td>HUMAN</td><td>E5RI99</td><td>60S ribosomal protein L30 (Fragment)</td></tr>
<tr><td>3543</td><td>dirMS_041712_NAR_30mM_plus2.18606.18647.3</td><td>3</td><td>14.38</td><td>0</td><td>4.97E+05</td><td></td><td>(R)SSFYVN(</td><td>(R)S S\F Y V N/G\L/T/L/G/G Q K(C)</td><td></td><td>150.95</td><td>490.9251</td><td>1470.759</td><td>0.002</td><td>1.4</td><td>8.31</td><td>17630.6</td><td>11.62</td><td>HUMAN</td><td>I3L3D5</td><td>Profilin-1 (Fragment)</td></tr>
<tr><td>3544</td><td>dirMS_041712_NAR_30mM_plus2.22059.22059.2</td><td>2</td><td>14.38</td><td>0</td><td>1.78E+05</td><td></td><td>(K)NSSYFV</td><td>(K)N S/S Y/F V/E/W/I|P N N/V K(V)</td><td></td><td>171.7</td><td>848.9189</td><td>1696.833</td><td>-0.0024</td><td>-1.4</td><td>6</td><td>42831.5</td><td>4.86</td><td>HUMAN</td><td>A8K854</td><td>HCG1983504, isoform CRA_f</td></tr>
<tr><td>3545</td><td>dirMS_041712_NAR_30mM_plus2.22345.22345.2</td><td>2</td><td>14.38</td><td>0</td><td>3.79E+05</td><td></td><td>(R)VGNLGL</td><td>(R)V/G N\L/G/L|A/T S/F F N E R(N)</td><td></td><td>172.8</td><td>762.8926</td><td>1524.781</td><td>-0.0026</td><td>-1.7</td><td>5.97</td><td>73641.5</td><td>6.74</td><td>HUMAN</td><td>O00571</td><td>ATP-dependent RNA helicase DDX3X</td></tr>
<tr><td>3546</td><td>dirMS_041712_NAR_50mM_minus2.10310.10310.3</td><td>3</td><td>14.37</td><td>0</td><td>1.46E+05</td><td></td><td>(K)VGSLDN</td><td>(K)V/G|S/L|D/N V/G H L/P A G G A V K(I)</td><td></td><td>126.9</td><td>530.9592</td><td>1590.86</td><td>0.0032</td><td>2</td><td>6.71</td><td>26233.5</td><td>10.95</td><td>HUMAN</td><td>B4DSQ1</td><td>Microtubule-associated protein</td></tr>
<tr><td>3547</td><td>dirMS_041712_NAR_50mM_minus2.7908.7994.3</td><td>3</td><td>14.37</td><td>1</td><td>3.94E+06</td><td>M239m</td><td>(R)MQKEIT</td><td>(R)M Q K E I T A|L|A|P/S/T m K(I)</td><td>m:Oxidized methionine</td><td>112.32</td><td>522.2727</td><td>1548.812</td><td>15.9911</td><td>-2.4</td><td>8.35</td><td>32616.5</td><td>5.18</td><td>HUMAN</td><td>A6NL76</td><td>Actin, alpha skeletal muscle</td></tr>
<tr><td>3548</td><td>dirMS_041712_NAR_60mM_minus.9933.9933.3</td><td>3</td><td>14.37</td><td>1</td><td>5.63E+05</td><td>M230m M</td><td>(R)mSmKE</td><td>(R)m S m K E V D E\Q|M|L/N/V\Q/N K(N)</td><td>m:Oxidized methionine</td><td>114.97</td><td>652.6318</td><td>1923.897</td><td>31.9836</td><td>-3.2</td><td>4.68</td><td>40069.4</td><td>4.83</td><td>HUMAN</td><td>E7EWR1</td><td>Tubulin beta chain</td></tr>
<tr><td>3549</td><td>dirMS_041712_NAR_20mM_plus.18780.18780.2</td><td>2</td><td>14.37</td><td>0</td><td>2.25E+05</td><td></td><td>(K)TAVETA</td><td>(K)T A\V|E/T|A/V L|L L R(I)</td><td></td><td>200.22</td><td>593.3625</td><td>1185.72</td><td>-0.0024</td><td>-2</td><td>5.66</td><td>56186.7</td><td>5.47</td><td>HUMAN</td><td>B4DUR8</td><td>T-complex protein 1 subunit gamma</td></tr>
<tr><td>3550</td><td>dirMS_041712_NAR_20mM_plus.6410.6410.2</td><td>2</td><td>14.37</td><td>0</td><td>1.60E+05</td><td></td><td>(K)DSYVGD</td><td>(K)D S Y|V/G/D/E A Q S K(R)</td><td></td><td>107.17</td><td>599.7645</td><td>1198.522</td><td>-0.0005</td><td>-0.4</td><td>4.03</td><td>32616.5</td><td>5.18</td><td>HUMAN</td><td>A6NL76</td><td>Actin, alpha skeletal muscle</td></tr>
<tr><td>3551</td><td>dirMS_041712_NAR_30mM_plus2.11977.11977.2</td><td>2</td><td>14.37</td><td>0</td><td>4.31E+05</td><td></td><td>(K)AYGGAY</td><td>(K)A Y/G|G A/Y/D V\M S/S K(H)</td><td></td><td>115.77</td><td>624.7816</td><td>1248.556</td><td>-0.0006</td><td>-0.5</td><td>5.88</td><td>61363</td><td>8.73</td><td>HUMAN</td><td>C9JQS9</td><td>Propionyl-CoA carboxylase beta chain, mitochondrial</td></tr>
<tr><td>3552</td><td>dirMS_041712_NAR_50mM_plus2.16657.16657.3</td><td>3</td><td>14.37</td><td>0</td><td>1.44E+05</td><td></td><td>(R)DVQIGD</td><td>(R)D V|Q/I/G D I/V T V G E C R P L S K(T)</td><td>C:Carbamidomethylation</td><td>155.08</td><td>662.6825</td><td>1986.033</td><td>0.0005</td><td>0.2</td><td>4.56</td><td>18601.6</td><td>10.52</td><td>HUMAN</td><td>P62280</td><td>40S ribosomal protein S11</td></tr>
<tr><td>3553</td><td>dirMS_041712_NAR_50mM_plus2.16971.16971.3</td><td>3</td><td>14.37</td><td>1</td><td>6.05E+05</td><td></td><td>(K)ISMPDIL</td><td>(K)I/S/M/P D/I D L N L K G P K(V)</td><td></td><td>157.1</td><td>514.2847</td><td>1540.84</td><td>-0.0008</td><td>-0.5</td><td>5.96</td><td>629603.7</td><td>5.8</td><td>HUMAN</td><td>Q09666</td><td>Neuroblast differentiation-associated protein AHNAK</td></tr>
<tr><td>3554</td><td>dirMS_041712_NAR_30mM_minus2.5847.5847.2</td><td>2</td><td>14.36</td><td>0</td><td>5.74E+04</td><td></td><td>(R)LAEAEE</td><td>(R)L/A/E|A/E E/T/A R(T)</td><td></td><td>86.62</td><td>495.2478</td><td>989.49</td><td>-0.0015</td><td>-1.5</td><td>4.25</td><td>67732.1</td><td>4.71</td><td>HUMAN</td><td>G3V119</td><td>DBIRD complex subunit KIAA1967</td></tr>
<tr><td>3555</td><td>dirMS_041712_NAR_40mM_minus2.12317.12489.3</td><td>3</td><td>14.36</td><td>1</td><td>2.92E+05</td><td></td><td>(K)DVAIKEI</td><td>(K)D V|A\I\K E/P\L\V/D/V\V D/P/K(Q)</td><td></td><td>156.1</td><td>546.3068</td><td>1636.916</td><td>-0.0098</td><td>-6</td><td>4.23</td><td>24376.8</td><td>6.08</td><td>HUMAN</td><td>E9PIX6</td><td>Protein arginine N-methyltransferase 1 (Fragment)</td></tr>
<tr><td>3556</td><td>dirMS_041712_NAR_50mM_minus2.12780.12780.3</td><td>3</td><td>14.36</td><td>1</td><td>3.77E+05</td><td></td><td>(K)NTVELL</td><td>(K)N T|V/E/L/L/V E D K G N Q V Y R(C)</td><td></td><td>140.5</td><td>626.3309</td><td>1876.976</td><td>0.0018</td><td>1</td><td>4.68</td><td>258215.9</td><td>5.48</td><td>HUMAN</td><td>E7EN95</td><td>Filamin-B</td></tr>
<tr><td>3557</td><td>dirMS_041712_NAR_20mM_plus.15513.15691.3</td><td>3</td><td>14.36</td><td>0</td><td>2.11E+05</td><td></td><td>(R)GVVDSE</td><td>(R)G V\V D S E D/I/P/L N/L S/R(E)</td><td></td><td>179.38</td><td>505.2667</td><td>1513.786</td><td>-0.0001</td><td>-0.1</td><td>4.03</td><td>74494.3</td><td>7.79</td><td>HUMAN</td><td>F5H897</td><td>Heat shock protein 75 kDa, mitochondrial</td></tr>
<tr><td>3558</td><td>dirMS_041712_NAR_20mM_plus.18626.18805.3</td><td>3</td><td>14.36</td><td>0</td><td>5.22E+05</td><td></td><td>(K)LAVEALS</td><td>(K)L A\V E A\L|S S L/D/G D/L/A/G R(Y)</td><td></td><td>200.47</td><td>529.6164</td><td>1586.838</td><td>-0.0038</td><td>-2.4</td><td>4.03</td><td>24286.7</td><td>4.89</td><td>HUMAN</td><td>G3V4N7</td><td>Creatine kinase B-type (Fragment)</td></tr>
<tr><td>3559</td><td>dirMS_041712_NAR_20mM_plus.8912.9064.2</td><td>2</td><td>14.36</td><td>0</td><td>4.48E+05</td><td>M161m</td><td>(K)LLIEmEC</td><td>(K)L/L|I/E/m/E/Q/R(L)</td><td>m:Oxidized methionine</td><td>132.82</td><td>524.278</td><td>1031.555</td><td>15.9933</td><td>-1.5</td><td>4.53</td><td>20203.9</td><td>6.24</td><td>HUMAN</td><td>H0YJG0</td><td>Creatine kinase B-type (Fragment)</td></tr>
<tr><td>3560</td><td>dirMS_041712_NAR_30mM_plus2.13235.13235.2</td><td>2</td><td>14.36</td><td>0</td><td>5.70E+05</td><td></td><td>(K)CPFTGN</td><td>(K)C/P|F|T/G N V/S I R(G)</td><td>C:Carbamidomethylation</td><td>122.88</td><td>575.7876</td><td>1150.567</td><td>0.0006</td><td>0.5</td><td>9.75</td><td>18601.6</td><td>10.52</td><td>HUMAN</td><td>P62280</td><td>40S ribosomal protein S11</td></tr>
<tr><td>3561</td><td>dirMS_041712_NAR_30mM_plus2.19982.19982.2</td><td>2</td><td>14.36</td><td>0</td><td>2.64E+04</td><td></td><td>(R)ALMDSL</td><td>(R)A L|M/D/S/L/G/P E W R(L)</td><td></td><td>158.47</td><td>637.8131</td><td>1274.62</td><td>-0.0009</td><td>-0.7</td><td>4.37</td><td>34958.1</td><td>9.42</td><td>HUMAN</td><td>Q15785</td><td>Mitochondrial import receptor subunit TOM34</td></tr>
<tr><td>3562</td><td>dirMS_041712_NAR_50mM_plus2.10809.10809.3</td><td>3</td><td>14.36</td><td>1</td><td>4.28E+05</td><td></td><td>(K)NQLTSN</td><td>(K)N Q|L/T/S N/P E N T V/F D A K R(L)</td><td></td><td>124.88</td><td>611.9758</td><td>1833.909</td><td>0.0039</td><td>2.1</td><td>6.07</td><td>72445.7</td><td>5.07</td><td>HUMAN</td><td>P11021</td><td>78 kDa glucose-regulated protein</td></tr>
<tr><td>3563</td><td>dirMS_041712_NAR_60mM_plus.17937.17937.3</td><td>3</td><td>14.36</td><td>1</td><td>1.75E+05</td><td></td><td>(K)FNADEF</td><td>(K)F N|A/D E F/E D M/V/A E K R(L)</td><td></td><td>150.68</td><td>567.5906</td><td>1700.758</td><td>-0.0012</td><td>-0.7</td><td>4.18</td><td>18819.6</td><td>10.31</td><td>HUMAN</td><td>F8W7C6</td><td>60S ribosomal protein L10</td></tr>
<tr><td>3564</td><td>dirMS_041712_NAR_120mM_minus.11732.11888.3</td><td>3</td><td>14.35</td><td>1</td><td>9.68E+05</td><td></td><td>(R)KFDQLL</td><td>(R)K\F\D\Q\L|L|A E/E/K(A)</td><td></td><td>118.67</td><td>407.5551</td><td>1220.652</td><td>-0.0014</td><td>-1.1</td><td>4.68</td><td>232239.1</td><td>5.52</td><td>HUMAN</td><td>F2Z2U8</td><td>Myosin-14</td></tr>
<tr><td>3565</td><td>dirMS_041712_NAR_120mM_minus.4414.4676.3</td><td>3</td><td>14.35</td><td>0</td><td>3.14E+07</td><td>M46m M4</td><td>(R)HQGVm</td><td>(R)H Q\G\V\m|V/G/m/G Q/K(D)</td><td>m:Oxidized methionine</td><td>74.18</td><td>401.8604</td><td>1171.571</td><td>31.9956</td><td>4.8</td><td>8.76</td><td>32616.5</td><td>5.18</td><td>HUMAN</td><td>A6NL76</td><td>Actin, alpha skeletal muscle</td></tr>
<tr><td>3566</td><td>dirMS_041712_NAR_20mM_minus.12303.12303.2</td><td>2</td><td>14.35</td><td>0</td><td>1.70E+05</td><td></td><td>(R)AAVIGPI</td><td>(R)A/A/V|I|G|P D/V F A K(L)</td><td></td><td>171.52</td><td>544.3103</td><td>1087.615</td><td>-0.0013</td><td>-1.2</td><td>5.88</td><td>266161</td><td>5.68</td><td>HUMAN</td><td>E9PPU0</td><td>Epiplakin</td></tr>
<tr><td>3567</td><td>dirMS_041712_NAR_30mM_minus2.11572.11741.2</td><td>2</td><td>14.35</td><td>0</td><td>5.18E+05</td><td></td><td>(R)DVCLYPI</td><td>(R)D V|C|L/Y/P/R(F)</td><td>C:Carbamidomethylation</td><td>117.17</td><td>461.7315</td><td>922.445</td><td>0.0106</td><td>11.5</td><td>5.83</td><td>35548.6</td><td>4.91</td><td>HUMAN</td><td>B4DN60</td><td>Asparagine--tRNA ligase, cytoplasmic</td></tr>
<tr><td>3568</td><td>dirMS_041712_NAR_30mM_minus2.11672.11721.2</td><td>2</td><td>14.35</td><td>0</td><td>1.84E+05</td><td></td><td>(R)ENVNLA</td><td>(R)E N|V|N/L|A/Q/I/R(T)</td><td></td><td>117.28</td><td>528.7959</td><td>1056.58</td><td>0.0049</td><td>4.6</td><td>6.1</td><td>62195.8</td><td>5.63</td><td>HUMAN</td><td>B0YIW6</td><td>Archain 1, isoform CRA_a</td></tr>
<tr><td>3569</td><td>dirMS_041712_NAR_30mM_minus2.17450.17671.2</td><td>2</td><td>14.35</td><td>0</td><td>4.37E+05</td><td></td><td>(R)EQLAIAE</td><td>(R)E Q|L|A|I/A/E/F A R(S)</td><td></td><td>150.25</td><td>574.3084</td><td>1147.611</td><td>-0.0011</td><td>-0.9</td><td>4.53</td><td>44227.3</td><td>7.81</td><td>HUMAN</td><td>E7EQR6</td><td>T-complex protein 1 subunit alpha</td></tr>
<tr><td>3570</td><td>dirMS_041712_NAR_60mM_minus.7977.7977.3</td><td>3</td><td>14.35</td><td>1</td><td>4.69E+05</td><td></td><td>(R)YLAEVA</td><td>(R)Y/L/A/E V A/T G E/K/R(A)</td><td></td><td>104.32</td><td>412.8902</td><td>1236.658</td><td>-0.0022</td><td>-1.8</td><td>6.14</td><td>28473.3</td><td>4.8</td><td>HUMAN</td><td>P61981</td><td>14-3-3 protein gamma</td></tr>
<tr><td>3571</td><td>dirMS_041712_NAR_120mM_plus.12522.12715.3</td><td>3</td><td>14.35</td><td>0</td><td>6.26E+05</td><td></td><td>(K)HLSVND</td><td>(K)H L\S\V N\D|L/P/V/G/R(S)</td><td></td><td>119.52</td><td>402.8906</td><td>1206.659</td><td>-0.0017</td><td>-1.4</td><td>6.74</td><td>26123.4</td><td>7.07</td><td>HUMAN</td><td>E9PH29</td><td>Thioredoxin-dependent peroxide reductase, mitochondrial</td></tr>
<tr><td>3572</td><td>dirMS_041712_NAR_20mM_plus.19365.19365.3</td><td>3</td><td>14.35</td><td>0</td><td>2.22E+05</td><td></td><td>(R)LAGTQP</td><td>(R)L A G T\Q/P L|E\V\L/E/A/V Q R(S)</td><td></td><td>204.52</td><td>541.9731</td><td>1623.906</td><td>-0.0017</td><td>-1</td><td>4.53</td><td>118930.9</td><td>5.49</td><td>HUMAN</td><td>P22314</td><td>Ubiquitin-like modifier-activating enzyme 1</td></tr>
<tr><td>3573</td><td>dirMS_041712_NAR_20mM_plus.8117.8117.2</td><td>2</td><td>14.35</td><td>0</td><td>6.42E+03</td><td></td><td>(K)TAVCDII</td><td>(K)T A/V|C/D/I|P|P R(G)</td><td>C:Carbamidomethylation</td><td>127.22</td><td>514.7664</td><td>1028.519</td><td>0.0062</td><td>6</td><td>5.5</td><td>50199.4</td><td>4.75</td><td>HUMAN</td><td>A6NNZ2</td><td>Tubulin beta-8 chain-like protein LOC260334</td></tr>
<tr><td>3574</td><td>dirMS_041712_NAR_30mM_plus2.15883.16354.2</td><td>2</td><td>14.35</td><td>0</td><td>3.72E+06</td><td></td><td>(R)INEILSN</td><td>(R)I/N|E|I|L|S/N A L/K(R)</td><td></td><td>138.07</td><td>557.8307</td><td>1114.647</td><td>0.0075</td><td>6.7</td><td>6</td><td>77799.7</td><td>9.09</td><td>HUMAN</td><td>P52272</td><td>Heterogeneous nuclear ribonucleoprotein M</td></tr>
<tr><td>3575</td><td>dirMS_041712_NAR_40mM_plus2.18627.18627.3</td><td>3</td><td>14.35</td><td>1</td><td>3.56E+05</td><td></td><td>(R)TLAEIAK</td><td>(R)T L/A/E/I/A K V/E L D/N M/P L R(G)</td><td></td><td>176.47</td><td>604.9967</td><td>1812.989</td><td>-0.0132</td><td>-7.3</td><td>4.68</td><td>23729.5</td><td>9.89</td><td>HUMAN</td><td>C9IZL7</td><td>Non-POU domain-containing octamer-binding protein (Fragment)</td></tr>
<tr><td>3576</td><td>dirMS_041712_NAR_40mM_plus2.6213.6213.2</td><td>2</td><td>14.35</td><td>1</td><td>2.84E+05</td><td></td><td>(R)YSGGNY</td><td>(R)Y/S|G/G/N Y R D N\Y|D|N(-)</td><td></td><td>95.58</td><td>719.2857</td><td>1437.567</td><td>-0.0024</td><td>-1.7</td><td>4.21</td><td>17170.1</td><td>8.86</td><td>HUMAN</td><td>P98179</td><td>Putative RNA-binding protein 3</td></tr>
<tr><td>3577</td><td>dirMS_041712_NAR_50mM_plus2.10704.10704.3</td><td>3</td><td>14.35</td><td>0</td><td>4.20E+05</td><td></td><td>(K)TSEVDL</td><td>(K)T/S|E|V D\L A K P L/V/K(F)</td><td></td><td>123.78</td><td>433.922</td><td>1299.752</td><td>-0.0004</td><td>-0.3</td><td>5.73</td><td>17178.3</td><td>5.63</td><td>HUMAN</td><td>C9IZF9</td><td>Programmed cell death 6-interacting protein (Fragment)</td></tr>
<tr><td>3578</td><td>dirMS_041712_NAR_60mM_plus.11280.11280.3</td><td>3</td><td>14.35</td><td>0</td><td>1.93E+06</td><td></td><td>(R)EIVHIQA</td><td>(R)E/I/V H I Q A G/Q/C/G/N Q I|G A/K(F)</td><td>C:Carbamidomethylation</td><td>116.42</td><td>608.311</td><td>1822.923</td><td>-0.0044</td><td>-2.4</td><td>6.85</td><td>40069.4</td><td>4.83</td><td>HUMAN</td><td>E7EWR1</td><td>Tubulin beta chain</td></tr>
<tr><td>3579</td><td>dirMS_041712_NAR_60mM_plus.7456.7456.3</td><td>3</td><td>14.35</td><td>1</td><td>5.05E+05</td><td></td><td>(K)SSGGREI</td><td>(K)S/S/G G R E D L\E|S/S/G L Q|R(R)</td><td></td><td>97.3</td><td>526.5897</td><td>1577.751</td><td>0.0031</td><td>2</td><td>4.68</td><td>11625.5</td><td>8.01</td><td>HUMAN</td><td>B8ZZT7</td><td>Hematological and neurological-expressed 1 protein</td></tr>
<tr><td>3580</td><td>dirMS_041712_NAR_60mM_plus.8976.8976.2</td><td>2</td><td>14.35</td><td>0</td><td>1.94E+05</td><td></td><td>(R)VLIAAH(</td><td>(R)V/L|I|A|A/H G N S L/R(G)</td><td></td><td>105.23</td><td>575.8377</td><td>1150.669</td><td>-0.001</td><td>-0.9</td><td>9.73</td><td>28936.8</td><td>9.22</td><td>HUMAN</td><td>P15259</td><td>Phosphoglycerate mutase 2</td></tr>
<tr><td>3581</td><td>dirMS_041712_NAR_30mM_minus2.7571.7656.2</td><td>2</td><td>14.34</td><td>0</td><td>1.29E+06</td><td></td><td>(R)FVDPNT</td><td>(R)F/V|D|P/N/T Q E K(V)</td><td></td><td>95.88</td><td>539.2647</td><td>1077.521</td><td>0.001</td><td>0.9</td><td>4.37</td><td>266161</td><td>5.68</td><td>HUMAN</td><td>E9PPU0</td><td>Epiplakin</td></tr>
<tr><td>3582</td><td>dirMS_041712_NAR_30mM_minus2.8079.8079.2</td><td>2</td><td>14.34</td><td>0</td><td>8.16E+04</td><td></td><td>(K)SLAAEEE</td><td>(K)S L|A/A/E/E E A/A/R(Q)</td><td></td><td>98.37</td><td>523.7597</td><td>1046.511</td><td>0.0008</td><td>0.8</td><td>4.25</td><td>533778</td><td>5.74</td><td>HUMAN</td><td>Q15149</td><td>Plectin</td></tr>
<tr><td>3583</td><td>dirMS_041712_NAR_40mM_minus2.8606.8607.3</td><td>3</td><td>14.34</td><td>0</td><td>2.85E+05</td><td></td><td>(R)SVIDPVF</td><td>(R)S V|I/D|P|V/P A/P V G D S H V D G A A K(S)</td><td></td><td>132.52</td><td>644.3329</td><td>1930.987</td><td>-0.0027</td><td>-1.4</td><td>4.41</td><td>58326.8</td><td>5.7</td><td>HUMAN</td><td>Q13177</td><td>Serine/threonine-protein kinase PAK 2</td></tr>
<tr><td>3584</td><td>dirMS_041712_NAR_60mM_minus.9321.9321.3</td><td>3</td><td>14.34</td><td>1</td><td>2.37E+05</td><td></td><td>(R)QDSAAV</td><td>(R)Q D/S A/A V/G/F D Y K E/K(L)</td><td></td><td>111.87</td><td>486.5639</td><td>1457.691</td><td>-0.0136</td><td>-9.3</td><td>4.56</td><td>61756.2</td><td>5.24</td><td>HUMAN</td><td>Q14247</td><td>Src substrate cortactin</td></tr>
<tr><td>3585</td><td>dirMS_041712_NAR_30mM_plus2.21241.21468.2</td><td>2</td><td>14.34</td><td>0</td><td>5.32E+05</td><td></td><td>(K)STLINTLI</td><td>(K)S/T|L|I/N/T L/F/K(S)</td><td></td><td>166.63</td><td>518.799</td><td>1036.604</td><td>-0.013</td><td>-12.5</td><td>8.47</td><td>18010.4</td><td>9.42</td><td>HUMAN</td><td>K7EIE4</td><td>Septin-9 (Fragment)</td></tr>
<tr><td>3586</td><td>dirMS_041712_NAR_30mM_plus2.6070.6088.2</td><td>2</td><td>14.34</td><td>0</td><td>2.40E+05</td><td></td><td>(R)IGDEDV(</td><td>(R)I/G|D|E/D/V/G/R(V)</td><td></td><td>86.23</td><td>430.7093</td><td>860.411</td><td>0.0005</td><td>0.6</td><td>4.03</td><td>23799.1</td><td>9.48</td><td>HUMAN</td><td>P23284</td><td>Peptidyl-prolyl cis-trans isomerase B</td></tr>
<tr><td>3587</td><td>dirMS_041712_NAR_30mM_plus2.7054.7262.2</td><td>2</td><td>14.34</td><td>0</td><td>3.13E+06</td><td></td><td>(K)NAGFTP</td><td>(K)N A|G|F|T/P Q E/R(Q)</td><td></td><td>92.33</td><td>510.2497</td><td>1019.491</td><td>0.0016</td><td>1.6</td><td>6</td><td>29474.8</td><td>6.94</td><td>HUMAN</td><td>G3V3M6</td><td>DNA-(apurinic or apyrimidinic site) lyase (Fragment)</td></tr>
<tr><td>3588</td><td>dirMS_041712_NAR_60mM_plus.15126.15289.3</td><td>3</td><td>14.34</td><td>1</td><td>8.60E+05</td><td></td><td>(R)MTGSEF</td><td>(R)M/T|G S E/F/D/F/E E M K R(K)</td><td></td><td>135.73</td><td>536.2355</td><td>1606.688</td><td>0.0043</td><td>2.7</td><td>4.41</td><td>51636.5</td><td>8.68</td><td>HUMAN</td><td>E7EU23</td><td>Rab GDP dissociation inhibitor beta</td></tr>
<tr><td>3589</td><td>dirMS_041712_NAR_60mM_plus.6453.6453.3</td><td>3</td><td>14.34</td><td>1</td><td>1.35E+05</td><td></td><td>(R)SIPAGAE</td><td>(R)S I/P/A G A E/P G E K G S A R(K)</td><td></td><td>92.35</td><td>476.2505</td><td>1426.728</td><td>0.0085</td><td>5.9</td><td>5.87</td><td>18962.9</td><td>9.6</td><td>HUMAN</td><td>A6NGP5</td><td>Hematological and neurological-expressed 1-like protein</td></tr>
<tr><td>3590</td><td>dirMS_041712_NAR_30mM_minus2.16912.16912.2</td><td>2</td><td>14.33</td><td>0</td><td>4.99E+05</td><td></td><td>(K)NSLESYA</td><td>(K)N S|L|E/S Y/A/F/N M K(A)</td><td></td><td>146.6</td><td>652.3031</td><td>1303.599</td><td>0.0002</td><td>0.2</td><td>6</td><td>45508.6</td><td>5.5</td><td>HUMAN</td><td>A8K7Q2</td><td>Heat shock cognate 71 kDa protein</td></tr>
<tr><td>3591</td><td>dirMS_041712_NAR_60mM_minus.7902.8018.3</td><td>3</td><td>14.33</td><td>1</td><td>8.24E+05</td><td></td><td>(R)LREDEN</td><td>(R)L R E D E N\A|E|P/V/G T T Y Q K(T)</td><td></td><td>104.68</td><td>617.3015</td><td>1849.893</td><td>-0.0027</td><td>-1.5</td><td>4.41</td><td>76925.9</td><td>4.46</td><td>HUMAN</td><td>A8MV58</td><td>Drebrin</td></tr>
<tr><td>3592</td><td>dirMS_041712_NAR_30mM_plus2.14774.14966.2</td><td>2</td><td>14.33</td><td>0</td><td>6.29E+05</td><td></td><td>(K)YDGIILP(</td><td>(K)Y/D|G\I|I/L/P|G/K(-)</td><td></td><td>130.85</td><td>488.28</td><td>975.551</td><td>0.0018</td><td>1.8</td><td>5.83</td><td>20480.2</td><td>9.91</td><td>HUMAN</td><td>P62913</td><td>60S ribosomal protein L11</td></tr>
</table>

3593	dirMS_041712_NAR_50mM_plus2.18670.18800.3	3	14.33	1	5.80E+05		(R)YLTAEET	(R)Y/L|T/A/E/E/T D K/I/I/N Y L R(E)		166.65	614.662	1841.964	0.0071	3.8	4.68	65763.5	9.81	HUMAN	Q9HCD5	Nuclear receptor coactivator 5
3594	dirMS_041712_NAR_50mM_plus2.6067.6146.3	3	14.33	0	1.85E+05		(R)ATAPQT	(R)A/T/A P/Q|T Q/H/V/S P M R(Q)		96.3	475.2418	1423.711	-0.0002	-0.1	9.8	29184.1	4.91	HUMAN	E9PBQ9	Elongation factor 1-delta
3595	dirMS_041712_NAR_50mM_plus2.9308.9308.3	3	14.33	0	1.33E+05		(K)AEITLVA	(K)A E|I/T L/V/A T/K/P E K(L)		116.23	433.922	1299.752	-0.0004	-0.3	6.18	98761.7	5.56	HUMAN	Q9UBF2	Coatomer subunit gamma-2
3596	dirMS_041712_NAR_120mM_minus.10985.11425.4	4	14.32	0	1.57E+06		(K)SAKPTKF	(K)S A K\P T K P A A/S\D\L\P\V|P/A/E/G V R(N)		116.33	498.5274	1991.092	-0.0042	-2.1	8.31	54214.9	5.66	HUMAN	C9J813	Caldesmon (Fragment)
3597	dirMS_041712_NAR_120mM_minus.5446.5446.3	3	14.32	0	1.24E+05		(R)YIDTEHC	(R)Y/I/D T E/H/G G S Q A R(F)		82.93	445.2092	1333.613	-0.0001	-0.1	5.32	63613.4	7.9	HUMAN	B3KXI2	Protein transport protein Sec23A
3598	dirMS_041712_NAR_30mM_minus2.16333.16333.2	2	14.32	0	1.29E+05	M256m	(R)SLDmDS	(R)S L/D\m/D\S I/I A/E V K(A)	m:Oxidized methionine	142	668.8368	1320.671	15.9948	-0.1	4.03	37296.3	5.3	HUMAN	F8VU64	Keratin, type II cytoskeletal 8
3599	dirMS_041712_NAR_60mM_minus.14894.14894.3	3	14.32	0	4.07E+05		(K)WIGLDL	(K)W/I/G/L D/L S/N/G K/P R(D)		143.47	452.5849	1355.743	-0.0029	-2.1	8.75	44227.3	7.81	HUMAN	E7EQR6	T-complex protein 1 subunit alpha
3600	dirMS_041712_NAR_60mM_minus.16393.16393.3	3	14.32	0	2.04E+05		(R)HDPTSE	(R)H D P T S E\N|I|L Q/L V/K(A)		150.92	498.6051	1493.796	0.0049	3.3	5.32	13564.4	5.9	HUMAN	F8VZ98	Serine/threonine-protein kinase D1 (Fragment)
3601	dirMS_041712_NAR_120mM_plus.21623.21623.4	4	14.32	1	2.53E+05		(R)KVDIIAD	(R)K\V D\I I|A/D/A/A Y/S/I/F Q/K/P K(S)		166.88	477.5186	1907.064	-0.0111	-5.8	8.43	23634.7	4.93	HUMAN	B4DWC7	Hydroxysteroid dehydrogenase-like protein 2
3602	dirMS_041712_NAR_20mM_plus.12284.12568.2	2	14.32	0	1.98E+05		(R)GVEICIA	(R)G V|E|I/C I/A|T|P G R(L)	C:Carbamidomethylation	158.67	586.8084	1172.609	0.0003	0.3	6	61018.6	9.44	HUMAN	B4DLW8	Probable ATP-dependent RNA helicase DDX5
3603	dirMS_041712_NAR_30mM_plus2.10341.10520.2	2	14.32	0	9.85E+05		(R)FAQMLE	(R)F/A|Q|M/L/E/K(V)		108.48	433.7317	866.444	0.0121	13.9	6	45578.2	7.83	HUMAN	B4DFL2	Isocitrate dehydrogenase [NADP]
3604	dirMS_041712_NAR_30mM_plus2.16785.16785.2	2	14.32	0	2.84E+05		(R)SIQADG	(R)S I|Q/A|D G L/V W/G/S S K(L)		140.7	674.3475	1347.69	-0.0026	-1.9	5.55	24934.4	4.5	HUMAN	P24534	Elongation factor 1-beta
3605	dirMS_041712_NAR_40mM_plus2.10247.10247.2	2	14.32	0	6.93E+04		(R)EYQELM	(R)E Y\Q/E|L/M/N/V K(L)		125.03	577.2823	1153.556	0.0015	1.3	4.53	56607.4	5.37	HUMAN	F8VXB4	Keratin, type II cytoskeletal 8
3606	dirMS_041712_NAR_40mM_plus2.12281.12281.3	3	14.32	0	3.63E+04		(R)VLTEDEI	(R)V/L|T\E/D E M G H/P E I/G D A I A R(L)		138.75	651.6493	1952.938	-0.0049	-2.5	4.17	40874	5.06	HUMAN	E9PCI9	Farnesyl pyrophosphate synthase
3607	dirMS_041712_NAR_40mM_plus2.14642.14642.3	3	14.32	0	5.34E+05		(K)IAILTCPF	(K)I/A\I|L T/C/P F E/P P K P K(T)	C:Carbamidomethylation	153.53	537.6381	1610.898	0.0023	1.4	8.59	49982	5.86	HUMAN	B4DYD8	T-complex protein 1 subunit epsilon
3608	dirMS_041712_NAR_50mM_minus2.19064.19064.3	3	14.31	1	1.34E+06		(R)KEGGLG	(R)K E G G L G\P L\N|I|P L/L A D V T R(R)		187.5	621.6941	1863.07	-0.0021	-1.1	6.07	15252.2	5.82	HUMAN	A6NIW5	Peroxiredoxin 2, isoform CRA_a
3609	dirMS_041712_NAR_120mM_plus.5571.5571.3	3	14.31	1	7.62E+04		(R)YGEDNR	(R)Y/G|E/D N R G Y|G G S Q/G G G R(G)		82.97	543.9055	1629.7	0.0019	1.2	6.07	26157.9	4.57	HUMAN	K7EL85	TATA-binding protein-associated factor 2N (Fragment)
3610	dirMS_041712_NAR_20mM_plus.6932.6932.2	2	14.31	0	1.59E+05		(K)TTTAAA'	(K)T T T A/A/A|V/A S/T/G\P/S S/R(S)		114.18	689.3492	1377.697	-0.0057	-4.2	9.41	75259.2	9.59	HUMAN	B5MEG9	Microtubule-associated protein 4 (Fragment)
3611	dirMS_041712_NAR_30mM_plus2.12023.12024.2	2	14.31	0	4.33E+05		(R)LYDVPAI	(R)L/Y|D|V|P A N S M|R(L)		115.97	583.2879	1165.567	0.0015	1.3	5.83	16508.2	5.16	HUMAN	J3QSX4	Mitotic checkpoint protein BUB3
3612	dirMS_041712_NAR_30mM_plus2.7146.7304.2	2	14.31	0	1.70E+06		(K)TEISEMI	(K)T E|I|S|E/M/N|R(N)		92.85	490.2294	979.451	0.0002	0.2	4.53	37296.3	5.3	HUMAN	F8VU64	Keratin, type II cytoskeletal 8
3613	dirMS_041712_NAR_30mM_plus2.9922.9922.2	2	14.31	0	1.65E+06		(K)GSFSDT(	(K)G S/F/S/D/T/G L/G/D/G K(M)		106	570.7598	1140.517	-0.0044	-3.9	4.21	108720.9	10.17	HUMAN	Q9Y2W1	Thyroid hormone receptor-associated protein 3
3614	dirMS_041712_NAR_40mM_plus2.10695.10695.3	3	14.31	0	1.85E+05		(K)VGSLDN	(K)V/G/S|L/D N V G H L/P A G G A V K(I)		128.15	530.9567	1590.86	-0.0043	-2.7	6.71	26233.5	10.95	HUMAN	B4DSQ1	Microtubule-associated protein
3615	dirMS_041712_NAR_60mM_plus.11896.11953.3	3	14.31	1	8.29E+05		(R)QSVEAD	(R)Q/S/V/E/A/D/I/N G\L R R(V)		119.35	453.2446	1357.718	0.001	0.7	6.07	19213.2	4.66	HUMAN	K7EMD9	Keratin, type I cytoskeletal 13 (Fragment)
3616	dirMS_041712_NAR_30mM_minus2.13678.13678.2	2	14.3	0	2.27E+05		(K)LTVEEAF	(K)L/T|V/E/E A/F K(A)		127.68	468.7559	936.504	0.0008	0.9	4.53	266161	5.68	HUMAN	E9PPU0	Epiplakin
3617	dirMS_041712_NAR_60mM_minus.11921.11921.3	3	14.3	1	3.03E+06	M72m	(R)TEmENE	(R)T E|m/E N E/F/V L/I/K K(D)	m:Oxidized methionine	126.87	499.5941	1480.772	15.9962	0.8	4.78	13588.1	4.66	HUMAN	F8VP67	Keratin, type II cytoskeletal 8 (Fragment)
3618	dirMS_041712_NAR_20mM_plus.10530.10530.2	2	14.3	0	1.07E+05		(R)AVLLGPI	(R)A V|L|L|G P P G A G K(G)		144.9	490.2992	979.593	-0.0024	-2.4	8.8	4460.2	8.14	HUMAN	F8VPP1	Adenylate kinase 2, mitochondrial
3619	dirMS_041712_NAR_20mM_plus.17804.17938.2	2	14.3	0	4.16E+05		(R)ILVATNL	(R)I/L|V|A/T/N L F/G R(G)		194.68	552.3309	1103.657	-0.0026	-2.4	9.75	40918.8	9.33	HUMAN	B4DP52	HCG2005638, isoform CRA_c
3620	dirMS_041712_NAR_20mM_plus.8088.8176.2	2	14.3	0	1.02E+05		(R)LLEAASV	(R)L/L|E|A/A/S V/S/S K(G)		125.87	502.7839	1004.562	-0.0017	-1.7	6	334221.3	6.44	HUMAN	P15924	Desmoplakin
3621	dirMS_041712_NAR_30mM_plus2.12299.12299.2	2	14.3	0	7.33E+04		(R)AQIFAN'	(R)A Q|I|F|A/N/T V D N/A R(I)		117.52	660.338	1319.67	-0.0015	-1.1	5.88	43773.3	5.26	HUMAN	F8VZY9	Keratin, type I cytoskeletal 18
3622	dirMS_041712_NAR_40mM_plus2.15016.15016.3	3	14.3	0	2.29E+05		(R)EVIDLLK	(R)E V/I/D/L/L/K P D Q V E G I Q K(S)		155.13	608.6749	1824.011	-0.0012	-0.6	4.32	156557.7	5.52	HUMAN	Q86UP2	Kinectin
3623	dirMS_041712_NAR_50mM_plus2.10124.10124.3	3	14.3	0	2.23E+05		(R)TITVALA	(R)T I|T V/A/L A D/G G R P D N T G R(G)		120.23	571.9661	1713.888	-0.0041	-2.4	5.63	107550.2	5.34	HUMAN	P49588	Alanine--tRNA ligase, cytoplasmic
3624	dirMS_041712_NAR_60mM_plus.7543.7543.2	2	14.3	1	7.47E+05		(R)VITEEEK	(R)V I|T/E/E/E/K N F K(A)		97.92	618.8282	1236.647	0.0021	1.7	4.79	18901.8	11.65	HUMAN	F5H1S2	60S ribosomal protein L13
3625	dirMS_041712_NAR_60mM_plus.9114.9304.3	3	14.3	0	2.11E+06		(K)NCPHIV'	(K)N C|P H|I|V/V/G T/P G/R(I)	C:Carbamidomethylation	106.15	436.2272	1306.669	-0.0014	-1.1	9.75	28185.1	10.76	HUMAN	B4DIJ6	Spliceosome RNA helicase DDX39B
3626	dirMS_041712_NAR_120mM_minus.9835.9893.3	3	14.29	1	1.52E+06		(K)AADAEA	(K)A A D|A/E/A/E/V/A S L N R/R(I)		108.45	491.5865	1472.745	-0.0002	-0.2	4.68	33450.2	4.73	HUMAN	J3KN67	Tropomyosin alpha-3 chain
3627	dirMS_041712_NAR_30mM_minus2.10330.10350.2	2	14.29	0	6.12E+05		(K)AVGQDT	(K)A/V|G|Q/D/T L L/R(S)		110.63	486.7751	972.547	-0.0043	-4.5	5.88	72873.4	5.73	HUMAN	Q96S55	ATPase WRNIP1
3628	dirMS_041712_NAR_60mM_plus.6843.6887.2	2	14.29	1	5.64E+05		(R)LSVDYGI	(R)L/S|V/D|Y|G K K(S)		94.35	455.257	909.504	0.0027	3	8.5	49012.3	4.9	HUMAN	A8MUB1	Tubulin alpha-4A chain
3629	dirMS_041712_NAR_120mM_minus.11469.11469.2	2	14.28	0	7.46E+05		(R)GSAVHC	(R)G S A/V/H|Q|L S/E/E\L R(C)		116.68	663.3434	1325.681	-0.0013	-1	5.4	558007.1	5.44	HUMAN	P58107	Epiplakin
3630	dirMS_041712_NAR_60mM_minus.17746.17746.3	3	14.28	1	4.16E+05	M148m	(R)LDLAGR	(R)L/D|L/A G R D L/T/D Y/L m K(I)	m:Oxidized methionine	159.32	547.2829	1623.841	15.9931	-1.1	4.43	37482.1	5.36	HUMAN	E9PG30	Actin, gamma-enteric smooth muscle
3631	dirMS_041712_NAR_120mM_plus.19626.19640.3	3	14.28	1	4.65E+05		(K)NLRDIDI	(K)N L\R D I\D\E|V|S|S L/L R(T)		156.13	510.6138	1529.828	-0.0013	-0.9	4.56	58214.7	5.25	HUMAN	B4DEM7	T-complex protein 1 subunit theta
3632	dirMS_041712_NAR_20mM_plus.17665.17665.3	3	14.28	0	1.23E+05		(K)LATQSN	(K)L A\T Q S N E\I T\I/P\V/T F E S R(A)		193.2	636.0003	1905.992	-0.0053	-2.8	4.53	3888.3	5	HUMAN	C9J3N8	Heat shock protein beta-1
3633	dirMS_041712_NAR_30mM_plus2.13463.13589.2	2	14.28	0	1.05E+06		(K)YGVSGY	(K)Y/G|V|S/G/Y/P/T L/K(I)		124.33	542.7882	1084.567	0.0018	1.6	8.5	55361.8	6.42	HUMAN	G5EA52	Protein disulfide isomerase family A, member 3, isoform CRA_b
3634	dirMS_041712_NAR_30mM_plus2.16197.16197.2	2	14.28	0	2.79E+05		(K)MDQAII	(K)M D/Q|A/I/I F/C R(T)	C:Carbamidomethylation	137.37	577.2809	1153.549	0.0053	4.6	5.59	83400.6	6.82	HUMAN	Q92499	ATP-dependent RNA helicase DDX1
3635	dirMS_041712_NAR_30mM_plus2.19722.19722.2	2	14.28	0	3.60E+05		(K)SNNVEM	(K)S N\N/V|E|M D W V/L K(H)		156.88	667.8205	1334.641	-0.0072	-5.4	4.37	18511.8	5.62	HUMAN	D6R9T0	Heterogeneous nuclear ribonucleoprotein H (Fragment)
3636	dirMS_041712_NAR_30mM_minus2.11717.11925.2	2	14.27	0	7.98E+05		(R)LPLETAY	(R)L/P|L|E|T A Y/R(R)		117.87	481.7625	962.531	-0.0128	-13.3	6	266161	5.68	HUMAN	E9PPU0	Epiplakin
3637	dirMS_041712_NAR_30mM_minus2.17195.17195.2	2	14.27	0	1.03E+05		(R)NGGQIL	(R)N G G/Q|I/L I/A D/L/R(G)		147.92	585.3352	1169.664	-0.0006	-0.5	5.84	34524.7	5.4	HUMAN	A8MV37	Protein SEC13 homolog
3638	dirMS_041712_NAR_50mM_minus2.9763.9763.3	3	14.27	0	2.46E+05		(K)TSEVDL/	(K)T/S|E V/D/L A K/P L V/K(F)		123.2	433.9205	1299.752	-0.0049	-3.8	5.73	17178.3	5.63	HUMAN	C9IZF9	Programmed cell death 6-interacting protein (Fragment)
3639	dirMS_041712_NAR_60mM_minus.17756.17756.3	3	14.27	1	2.33E+05		(R)LGLSTLG	(R)L/G/L/S\T L/G E/L/K Q N L S R(S)		159.42	543.6476	1628.933	-0.0047	-2.9	8.75	22294.4	5.26	HUMAN	O43399	Tumor protein D54
3640	dirMS_041712_NAR_120mM_plus.13268.13277.4	4	14.27	1	1.43E+06		(K)SVHSSVI	(K)S V H S\S\V|P L/L/N S K/D/P I/D/R(I)		123.27	466.7533	1863.992	-0.0009	-0.5	6.47	54427.4	5.96	HUMAN	K4DID9	Acetyl-CoA carboxylase 1
3641	dirMS_041712_NAR_20mM_plus.16613.16613.2	2	14.27	0	1.91E+05		(K)VQIAVA	(K)V Q|I|A\V|A N/A/Q/E L/L Q R(M)		186.22	776.9419	1552.881	-0.004	-2.6	5.97	10728.1	9.15	HUMAN	Q9Y5L4	Mitochondrial import inner membrane translocase subunit Tim13
3642	dirMS_041712_NAR_30mM_plus2.20863.21330.2	2	14.27	0	4.30E+06		(K)FLPLFDR	(K)F/L|P|L F D/R(V)		165.65	454.2621	907.504	0.0133	14.7	5.84	10803.5	9.82	HUMAN	B8ZZL8	10 kDa heat shock protein, mitochondrial
3643	dirMS_041712_NAR_30mM_plus2.8349.8349.2	2	14.27	0	4.11E+05		(K)TAVCDIF	(K)T A|V/C/D I|P\P R(G)	C:Carbamidomethylation	98	514.7643	1028.519	0.002	1.9	5.5	50199.4	4.75	HUMAN	A6NNZ2	Tubulin beta-8 chain-like protein LOC260334
3644	dirMS_041712_NAR_50mM_plus2.16929.16929.3	3	14.27	1	6.11E+05		(K)ISMPDIL	(K)I/S M/P/D I D|L N L\K/G P/K(V)		157.08	514.2847	1540.84	-0.0008	-0.5	5.96	629603.7	5.8	HUMAN	Q09666	Neuroblast differentiation-associated protein AHNAK
3645	dirMS_041712_NAR_60mM_plus.14394.14394.3	3	14.27	0	2.46E+05		(R)VNAIEH'	(R)V/N|A/I/E H/V/I I P R(I)		132	420.9182	1260.742	-0.0022	-1.8	6.72	21920.7	5.63	HUMAN	G3V2S6	V-type proton ATPase subunit D
3646	dirMS_041712_NAR_60mM_plus.20145.20183.3	3	14.27	0	4.68E+05		(R)DFLLKPE	(R)D F|L/L/K/P E L L/R(A)		165.73	415.2483	1243.741	-0.0105	-8.5	6.07	30944.2	6.52	HUMAN	B1Q2N1	ATP-dependent RNA helicase DDX39A
3647	dirMS_041712_NAR_60mM_plus.9541.9541.3	3	14.27	0	1.08E+06		(K)GHENVE	(K)G H E\N V\E\A\A|Q/A/E Y I E/K(F)		108.1	563.2692	1687.792	0.0008	0.5	4.48	61363	8.73	HUMAN	C9JQS9	Propionyl-CoA carboxylase beta chain, mitochondrial
3648	dirMS_041712_NAR_20mM_minus.11508.11553.2	2	14.26	0	1.03E+07	M148m	(R)DLTDYLr	(R)D L|T|D|Y/L/m/K(I)	m:Oxidized methionine	162.28	507.7443	998.486	15.995	0.1	4.21	37482.1	5.36	HUMAN	E9PG30	Actin, gamma-enteric smooth muscle
3649	dirMS_041712_NAR_30mM_minus2.11825.12161.2	2	14.26	0	5.97E+05		(R)SGYLLPL	(R)S/G|Y|L|L|P D/T K(A)		118.77	497.265	993.525	-0.0024	-2.4	5.55	17045.4	4.25	HUMAN	H0YIV0	Endoplasmin (Fragment)
3650	dirMS_041712_NAR_60mM_minus.7285.7692.3	3	14.26	1	1.70E+06		(K)IQDKEGI	(K)I Q/D K E G|I|P/P D/Q Q R(L)		99.7	508.5978	1523.781	-0.0024	-1.6	4.56	17271.6	6.75	HUMAN	B4DV12	Ubiquitin
3651	dirMS_041712_NAR_60mM_minus.8076.8094.3	3	14.26	1	1.61E+05		(K)DISTNYY	(K)D I/S/T/N Y/Y/A/S/Q/K K(T)		105.07	473.231	1417.696	-0.0173	-12.2	8.43	17045.4	4.25	HUMAN	H0YIV0	Endoplasmin (Fragment)
3652	dirMS_041712_NAR_120mM_plus.6133.6300.3	3	14.26	0	7.89E+05		(K)QRPQAT	(K)Q/R/P Q\A|T/A/E/Q/I/R(L)		86.08	433.2367	1297.697	-0.0016	-1.2	9.6	113668.7	6.2	HUMAN	F8W726	Ubiquitin-associated protein 2-like
3653	dirMS_041712_NAR_20mM_plus.9055.9065.2	2	14.26	0	2.66E+05		(R)VLTVINC	(R)V L|T\V/I/N/Q/T Q|K(E)		133.07	572.3399	1143.673	-0.0007	-0.6	8.72	10815.7	10.72	HUMAN	F2Z388	60S ribosomal protein L35
3654	dirMS_041712_NAR_20mM_plus.9333.9375.2	2	14.26	0	5.64E+05		(K)TIAPALV	(K)T I|A|P A L/V S K(K)		136.25	450.2814	899.556	-0.0005	-0.6	8.41	21077	9.05	HUMAN	K7EM90	Alpha-enolase (Fragment)
3655	dirMS_041712_NAR_40mM_plus2.13161.13300.3	3	14.26	0	1.97E+05		(R)DLSHIGI	(R)D L S H I\G D\A\V|V|I/S/C/A K(D)	C:Carbamidomethylation	144.35	528.9395	1584.805	-0.0011	-0.7	5.21	29110.6	4.57	HUMAN	P12004	Proliferating cell nuclear antigen
3656	dirMS_041712_NAR_40mM_plus2.16019.16285.2	2	14.26	0	1.50E+05		(R)YLTVAA'	(R)Y L|T|V/A/A V/F/R(G)		162.03	520.2997	1039.593	-0.0014	-1.3	8.75	40069.4	4.83	HUMAN	E7EWR1	Tubulin beta chain
3657	dirMS_041712_NAR_60mM_plus.15998.15998.3	3	14.26	1	2.98E+05		(R)MSMKE'	(R)M/S M K\E V D\E Q|M/L/N V Q N K(N)		139.27	641.9696	1923.897	-0.003	-1.6	4.68	40069.4	4.83	HUMAN	E7EWR1	Tubulin beta chain
3658	dirMS_041712_NAR_120mM_minus.8475.8579.3	3	14.25	0	6.90E+05		(K)YKPESEE	(K)Y K|P E S E E|L/T/A/E/R(I)		100.73	484.5714	1451.701	-0.0016	-1.1	4.5	51524.1	4.82	HUMAN	F5H8J2	Protein disulfide-isomerase
3659	dirMS_041712_NAR_20mM_minus.11736.11736.2	2	14.25	0	3.20E+05		(R)GALQNI	(R)G A L\Q|N|I|I\P A S/T G A A K(A)		165.35	706.3976	1411.79	-0.0024	-1.7	8.75	31718.5	7.17	HUMAN	E7EUT4	Glyceraldehyde-3-phosphate dehydrogenase
3660	dirMS_041712_NAR_50mM_minus2.10796.10819.3	3	14.25	1	6.04E+05		(R)IAEFTTN	(R)I/A|E/F/T/T|N L/T/E E E E K S K(S)		130.4	623.6418	1868.912	-0.0015	-0.8	4.32	227783.5	5.5	HUMAN	P35579	Myosin-9
3661	dirMS_041712_NAR_20mM_plus.13770.13952.2	2	14.25	0	2.82E+05		(R)INEILSN/	(R)I/N|E|I|L/S N A L/K(R)		167.63	557.8267	1114.647	-0.0005	-0.5	6	77799.7	9.09	HUMAN	P52272	Heterogeneous nuclear ribonucleoprotein M
3662	dirMS_041712_NAR_30mM_plus2.14308.14308.2	2	14.25	0	1.13E+04		(R)QLLEEEL	(R)Q L/L/E/E/E L/A R(L)		128.93	550.8009	1100.595	-0.0001	-0.1	4.25	533778	5.74	HUMAN	Q15149	Plectin
3663	dirMS_041712_NAR_30mM_plus2.16512.16512.2	2	14.25	0	3.03E+05		(K)LLDLVQ(	(K)L L\D|L/V/Q/Q\S|C N Y K(Q)	C:Carbamidomethylation	139.13	740.8751	1480.746	-0.0035	-2.4	5.83	14855.1	9.26	HUMAN	B1AHD1	NHP2 non-histone chromosome protein 2-like 1 (S. cerevisiae)
3664	dirMS_041712_NAR_30mM_plus2.21643.21643.2	2	14.25	0	3.07E+05		(R)FSWNV\	(R)F S/W/N V W/P S S R(L)		169.08	633.3085	1265.606	0.0035	2.8	9.75	83880.6	7.33	HUMAN	F5H365	Protein transport protein Sec23A
3665	dirMS_041712_NAR_40mM_plus2.15107.15500.3	3	14.25	0	9.12E+05		(R)DVQIGD	(R)D V\Q|I/G/D/I/V/T V/G E/C R P L S K(T)	C:Carbamidomethylation	157.25	662.6815	1986.033	-0.0025	-1.3	4.56	18601.6	10.52	HUMAN	P62280	40S ribosomal protein S11
3666	dirMS_041712_NAR_50mM_plus2.15925.15925.3	3	14.25	1	1.41E+05		(K)VSEQGL	(K)V/S|E Q/G L I E I L K/K(V)		151.65	452.944	1356.81	0.0078	5.7	6.11	12899.6	6.59	HUMAN	K7EL31	Programmed cell death protein 5
3667	dirMS_041712_NAR_50mM_plus2.16450.16450.3	3	14.25	0	4.72E+05		(R)IETMLGI	(R)I/E\T M L G D|V A/V/A V/H|P K(D)		154.65	527.282	1579.851	-0.0198	-12.5	5.32	141718.6	7.8	HUMAN	B0V043	Valine--tRNA ligase
3668	dirMS_041712_NAR_60mM_plus.15419.15419.3	3	14.25	1	3.85E+05		(R)SLIANLA	(R)S L/I/A N/L/A A A/N C/Y K K(E)	C:Carbamidomethylation	137.43	512.9411	1536.82	-0.0115	-7.5	9.7	36978.6	6.33	HUMAN	B7Z800	Adenosine kinase
3669	dirMS_041712_NAR_60mM_plus.4797.4797.3	3	14.25	1	3.22E+05		(K)AGADEE	(K)A/G/A|D/E/E R A E/T A R(L)		79.42	425.868	1275.592	-0.0029	-2.3	4.41	57204.9	6.01	HUMAN	F5GWF6	T-complex protein 1 subunit beta
3670	dirMS_041712_NAR_20mM_minus.14648.14648.2	2	14.24	0	2.02E+05		(K)TAVETA'	(K)T A|V/E/T/A/V L\L L/R(I)		201.2	593.3613	1185.72	-0.0048	-4.1	5.66	56186.7	5.47	HUMAN	B4DUR8	T-complex protein 1 subunit gamma
3671	dirMS_041712_NAR_40mM_minus2.16101.16101.3	3	14.24	1	6.73E+05		(R)ELAILLG	(R)E L|A/I/L/L/G M/L/D/P A E K D E K(G)		189.58	629.0034	1884.999	-0.003	-1.6	4.18	25148.6	6	HUMAN	P30041	Peroxiredoxin-6
3672	dirMS_041712_NAR_50mM_minus2.17031.17491.3	3	14.24	0	2.11E+07		(R)IHFPLAT	(R)I H\F\P L A\T\Y|A|P V/I/S A E K(A)		167.2	586.3248	1756.963	-0.0034	-1.9	6.75	49012.3	4.9	HUMAN	A8MUB1	Tubulin alpha-4A chain
3673	dirMS_041712_NAR_50mM_minus2.7416.7416.3	3	14.24	0	6.77E+04		(K)STAGDT	(K)S/T|A/G D T H L/G G E D F D/N R(M)		108.82	564.5808	1691.726	0.0023	1.3	4.22	69032.8	5.37	HUMAN	E9PKE3	Heat shock cognate 71 kDa protein
3674	dirMS_041712_NAR_120mM_plus.7912.8022.4	4	14.24	1	6.41E+05		(K)VLATVTI	(K)V L\A T/V/T/K/P V G G D K/N/G G T R(V)		95.57	443.251	1769.987	-0.0047	-2.6	9.99	12738	10.72	HUMAN	F8VWR1	60S ribosomal protein L6 (Fragment)
3675	dirMS_041712_NAR_20mM_plus.12531.12607.3	3	14.24	0	3.48E+05		(K)IISNASC'	(K)I I|S N\A\S C T T/N/C L/A/P L A K(V)	C:Carbamidomethylation	159.53	611.9768	1833.92	-0.0039	-2.1	8.75	31718.5	7.17	HUMAN	E7EUT4	Glyceraldehyde-3-phosphate dehydrogenase
3676	dirMS_041712_NAR_20mM_plus.13791.13791.2	2	14.24	0	6.96E+04		(R)VEVTEFI	(R)V/E|V/T E/F/E D/I K(S)		167.18	604.8056	1208.604	-0.0006	-0.5	4	30992.3	4.14	HUMAN	B2REB8	Protein SET
3677	dirMS_041712_NAR_20mM_plus.9068.9068.2	2	14.24	0	2.65E+05		(R)VLTVINC	(R)V L/T/V/I/N Q/T Q K(E)		133.08	572.3399	1143.673	-0.0007	-0.6	8.72	10815.7	10.72	HUMAN	F2Z388	60S ribosomal protein L35
3678	dirMS_041712_NAR_50mM_plus2.17853.17855.3	3	14.24	0	2.58E+05		(R)IGRPSET	(R)I G R P S E T G\I|I G\I|I|D/P E C R(M)	C:Carbamidomethylation	161.9	661.6783	1983.033	-0.0124	-6.3	4.68	16255.6	5.76	HUMAN	F5GWI0	DNA damage-binding protein 1 (Fragment)
3679	dirMS_041712_NAR_60mM_plus.16676.16883.3	3	14.24	1	1.13E+06		(R)ELISNAS	(R)E L|I/S/N/A/S|D|A L D K I R(L)		144.67	515.6144	1544.828	0.0008	0.5	4.56	5582.4	4.81	HUMAN	F8W026	Endoplasmin (Fragment)
3680	dirMS_041712_NAR_60mM_plus.18889.18889.3	3	14.24	0	3.09E+05		(R)GGHVVE	(R)G G H V V E G L|A|G E/L E Q/L/R(A)		156.17	555.2949	1663.876	-0.0061	-3.6	4.75	106088.3	5.78	HUMAN	P10275	Androgen receptor
3681	dirMS_041712_NAR_60mM_plus.20611.20611.3	3	14.24	0	7.27E+05		(R)LGFHLPI	(R)L/G\F H\L/P L E|V/A/Y/Q R(G)		168.62	514.9568	1542.843	0.0131	8.5	6.75	533778	5.74	HUMAN	Q15149	Plectin
3682	dirMS_041712_NAR_30mM_minus2.17509.17509.2	2	14.23	0	2.23E+05		(R)NGGQIL	(R)N G/G Q/I|L I/A D/L R(G)		149.25	585.3352	1169.664	-0.0006	-0.5	5.84	34524.7	5.4	HUMAN	A8MV37	Protein SEC13 homolog
3683	dirMS_041712_NAR_60mM_minus.6579.6579.3	3	14.23	0	5.72E+04		(K)ELVSDAI	(K)E L/V/S\D/A/N Q|H/V/K(S)		95.22	413.8839	1239.633	0.0044	3.5	5.32	57818.1	5.5	HUMAN	E9PH38	Serine/threonine-protein phosphatase 2A 65 kDa regulatory subunit A alpha isoform
3684	dirMS_041712_NAR_20mM_plus.8012.8012.2	2	14.23	0	4.21E+05	M239m	(K)EVDEQm	(K)E V/D E Q/m L/N|V|Q\N/K(N)	m:Oxidized methionine	125.1	731.8435	1446.689	15.9904	-3.1	4.14	40069.4	4.83	HUMAN	E7EWR1	Tubulin beta chain
3685	dirMS_041712_NAR_30mM_plus2.14793.14840.2	2	14.23	0	1.83E+05		(R)NWADV	(R)N/W|A|D|V/Q/S\Q L/R(H)		130.72	608.8101	1216.607	0.006	4.9	5.84	87447.6	6.44	HUMAN	Q15437	Protein transport protein Sec23B
3686	dirMS_041712_NAR_40mM_plus2.10769.10909.3	3	14.23	0	9.41E+05		(R)LLEAQA(	(R)L L|E|A/Q/A/C/T G G I/I H/P T T G Q K(L)	C:Carbamidomethylation	130.33	665.6807	1995.033	-0.0052	-2.6	6.75	334221.3	6.44	HUMAN	P15924	Desmoplakin
3687	dirMS_041712_NAR_40mM_plus2.7869.7869.2	2	14.23	0	5.86E+05		(K)ASLENSI	(K)A/S/L/E|N S L\R(E)		105.63	445.2395	889.474	-0.002	-2.3	6.05	43773.3	5.26	HUMAN	F8VZY9	Keratin, type I cytoskeletal 18
3688	dirMS_041712_NAR_50mM_plus2.13465.13465.3	3	14.23	0	1.05E+06		(K)AASDIAI	(K)A A\S D/I/A/M T/E/L/P P T H/P I R(L)		139.18	607.3177	1819.937	0.0015	0.8	5.32	12926.1	4.4	HUMAN	K7EM20	14-3-3 protein epsilon (Fragment)
3689	dirMS_041712_NAR_50mM_plus2.14623.14623.3	3	14.23	0	1.85E+05		(R)ISTLTIEE	(R)I/S/T/L/T/I E/E G/N L D I Q R P K(R)		144.78	643.0148	1927.05	-0.0196	-10.2	4.68	38649.2	6.88	HUMAN	Q12972	Nuclear inhibitor of protein phosphatase 1
3690	dirMS_041712_NAR_50mM_plus2.17919.18204.3	3	14.23	1	1.01E+06		(K)GPVREG	(K)G P V R E G D\V\L|T|L|L/E S E/R(E)		162.88	590.6519	1769.939	0.0019	1.1	4.41	7898	11.1	HUMAN	P62857	40S ribosomal protein S28
3691	dirMS_041712_NAR_50mM_plus2.9860.9860.3	3	14.23	1	1.04E+06		(K)LKGEAT\	(K)L K\G E A\T V|S/F/D D/P P S A K(A)		119.12	554.6155	1661.838	-0.0061	-3.7	4.56	53724.2	9.54	HUMAN	H3BPE7	RNA-binding protein FUS
3692	dirMS_041712_NAR_60mM_plus.6538.6538.3	3	14.23	1	6.89E+05		(R)NKQTYS	(R)N K Q T Y S|T E/P N/N L K(A)		92.93	512.9257	1536.765	-0.0027	-1.8	8.5	7945.1	11.03	HUMAN	C9JB50	60S ribosomal protein L28
3693	dirMS_041712_NAR_120mM_minus.9915.9978.3	3	14.22	1	7.79E+05		(R)ETAEAD'	(R)E/T/A/E/A D/V|A S L N R R(I)		108.67	477.9106	1431.719	-0.0014	-1	4.68	31923.6	4.85	HUMAN	B7Z596	Tropomyosin alpha-1 chain
3694	dirMS_041712_NAR_30mM_minus2.14466.14684.2	2	14.22	0	5.00E+05		(R)TFEDFVI	(R)T/F|E|D/F/V/R(I)		133.23	457.2246	913.441	0.0005	0.6	4.37	267260.7	5.95	HUMAN	Q13085	Acetyl-CoA carboxylase 1
3695	dirMS_041712_NAR_30mM_minus2.7394.7394.2	2	14.22	0	2.50E+03		(R)TPEEYPE	(R)T/P|E|E Y/P E/S A K(V)		93.4	575.7705	1150.526	0.0075	6.5	4.25	21580.5	5.38	HUMAN	B4DGT0	N(G),N(G)-dimethylarginine dimethylaminohydrolase 1
3696	dirMS_041712_NAR_30mM_minus2.8498.8542.2	2	14.22	0	6.85E+05		(R)QAEVEL	(R)Q/A\E/V/E/L/A/S/R(V)		100.67	501.7573	1002.521	-0.0141	-14.1	4.53	533778	5.74	HUMAN	Q15149	Plectin
3697	dirMS_041712_NAR_120mM_plus.7220.7332.3	3	14.22	1	7.86E+05		(R)KTGQAP	(R)K\T G Q A|P G\Y|S|Y\T/A A/N K(N)		91.62	519.5943	1556.77	-0.002	-1.3	9.53	11447.2	9.81	HUMAN	C9JFR7	Cytochrome c (Fragment)
3698	dirMS_041712_NAR_20mM_plus.17850.17850.2	2	14.22	0	1.59E+05		(R)TVQSLEI	(R)T V/Q|S/L/E|I D L D/S M R(N)		194.85	753.8776	1506.747	0.0011	0.7	4.03	43773.3	5.26	HUMAN	F8VZY9	Keratin, type I cytoskeletal 18
3699	dirMS_041712_NAR_30mM_plus2.14459.14660.2	2	14.22	0	5.85E+05		(K)IGLDFCK	(K)I/G|L/D|F/C/K(Y)	C:Carbamidomethylation	129.58	426.7164	852.428	-0.0029	-3.4	5.84	73386.6	8.73	HUMAN	B4DXX7	ATP-dependent RNA helicase DDX3Y
3700	dirMS_041712_NAR_40mM_plus2.20059.20059.3	3	14.22	1	5.88E+05		(K)EGGLGP	(K)E G G L/G/P/L/N/I/P L L A D V T R R(L)		189.8	631.0288	1891.076	-0.0041	-2.2	6.17	15252.2	5.82	HUMAN	A6NIW5	Peroxiredoxin 2, isoform CRA_a
3701	dirMS_041712_NAR_50mM_plus2.14994.14994.3	3	14.22	1	1.14E+05		(R)LLDAQL/	(R)L L|D/A/Q L/A T/G G L|V C P A R R(L)	C:Carbamidomethylation	147.2	604.3351	1810.996	-0.0049	-2.7	9.6	266161	5.68	HUMAN	E9PPU0	Epiplakin

<table>
<tr><td>3702</td><td>dirMS_041712_NAR_50mM_plus2.17527.17527.3</td><td>3</td><td>14.22</td><td>0</td><td>9.60E+05</td><td></td><td>(R)VLDPFTI</td><td>(R)V/L/D/P/F/T I/K P L D R(K)</td><td></td><td>160.52</td><td>471.9408</td><td>1413.81</td><td>-0.0022</td><td>-1.5</td><td>5.93</td><td>63448.4</td><td>7.29</td><td>HUMAN</td><td>B4E022</td><td>Transketolase</td></tr>
<tr><td>3703</td><td>dirMS_041712_NAR_30mM_minus2.17986.17986.2</td><td>2</td><td>14.21</td><td>0</td><td>2.20E+05</td><td></td><td>(K)EGMNIV</td><td>(K)E G M/N/I|V E A M E/R(F)</td><td></td><td>152.3</td><td>639.7955</td><td>1278.582</td><td>0.002</td><td>1.6</td><td>4.25</td><td>18240.4</td><td>7.96</td><td>HUMAN</td><td>P62937</td><td>Peptidyl-prolyl cis-trans isomerase A</td></tr>
<tr><td>3704</td><td>dirMS_041712_NAR_50mM_minus2.12779.12779.3</td><td>3</td><td>14.21</td><td>1</td><td>3.77E+05</td><td></td><td>(K)NTVELL</td><td>(K)N T/V/E/L/L V E D/K G N Q V Y R(C)</td><td></td><td>140.5</td><td>626.3309</td><td>1876.976</td><td>0.0018</td><td>1</td><td>4.68</td><td>258215.9</td><td>5.48</td><td>HUMAN</td><td>E7EN95</td><td>Filamin-B</td></tr>
<tr><td>3705</td><td>dirMS_041712_NAR_50mM_minus2.5850.5850.3</td><td>3</td><td>14.21</td><td>0</td><td>1.31E+05</td><td></td><td>(R)AGAAG</td><td>(R)A G\A|A/G/G P E/E E A E/K P V K(T)</td><td></td><td>95.32</td><td>513.9233</td><td>1539.765</td><td>-0.0096</td><td>-6.2</td><td>4.49</td><td>18924.5</td><td>6.86</td><td>HUMAN</td><td>Q8WW12</td><td>PEST proteolytic signal-containing nuclear protein</td></tr>
<tr><td>3706</td><td>dirMS_041712_NAR_120mM_plus.11006.11006.4</td><td>4</td><td>14.21</td><td>0</td><td>8.17E+05</td><td></td><td>(R)GAIQFV</td><td>(R)G A|I\Q/F/V/T H/Y Q H/S S T Q R(R)</td><td></td><td>111.07</td><td>465.7339</td><td>1859.915</td><td>-0.0009</td><td>-0.5</td><td>8.76</td><td>87447.6</td><td>6.44</td><td>HUMAN</td><td>Q15437</td><td>Protein transport protein Sec23B</td></tr>
<tr><td>3707</td><td>dirMS_041712_NAR_20mM_plus.12007.12024.2</td><td>2</td><td>14.21</td><td>0</td><td>6.74E+04</td><td></td><td>(K)VLIANN</td><td>(K)V L|I|A|N/N G I A/A/V K(C)</td><td></td><td>155.83</td><td>591.8632</td><td>1182.72</td><td>-0.0014</td><td>-1.2</td><td>8.72</td><td>269989.9</td><td>5.92</td><td>HUMAN</td><td>F8W8T8</td><td>Acetyl-CoA carboxylase 2</td></tr>
<tr><td>3708</td><td>dirMS_041712_NAR_30mM_plus2.10320.10320.2</td><td>2</td><td>14.21</td><td>0</td><td>5.06E+05</td><td></td><td>(K)EQVANS</td><td>(K)E Q/V/A/N/S/A F V E/R(C)</td><td></td><td>107.17</td><td>625.311</td><td>1249.617</td><td>-0.0024</td><td>-1.9</td><td>4.53</td><td>17664</td><td>8.77</td><td>HUMAN</td><td>H0Y6E4</td><td>Heat shock protein HSP 90-beta (Fragment)</td></tr>
<tr><td>3709</td><td>dirMS_041712_NAR_50mM_plus2.15516.15516.3</td><td>3</td><td>14.21</td><td>0</td><td>1.51E+06</td><td></td><td>(R)LASTLV</td><td>(R)L A|S T L|V/H L\G E/Y Q A/A/V/D G A R(K)</td><td></td><td>149.68</td><td>657.6802</td><td>1971.029</td><td>-0.0034</td><td>-1.7</td><td>5.32</td><td>71165.3</td><td>5.57</td><td>HUMAN</td><td>J3KS13</td><td>Clathrin heavy chain 1</td></tr>
<tr><td>3710</td><td>dirMS_041712_NAR_50mM_plus2.6030.6030.3</td><td>3</td><td>14.21</td><td>0</td><td>8.84E+04</td><td></td><td>(R)ITPPAA</td><td>(R)I T|P/P A A\K/P G S P Q A K(S)</td><td></td><td>95.85</td><td>454.9298</td><td>1362.774</td><td>0.0009</td><td>0.6</td><td>10</td><td>214357.5</td><td>7.19</td><td>HUMAN</td><td>P35658</td><td>Nuclear pore complex protein Nup214</td></tr>
<tr><td>3711</td><td>dirMS_041712_NAR_60mM_plus.12970.12992.3</td><td>3</td><td>14.21</td><td>1</td><td>4.93E+05</td><td></td><td>(K)FMLGK</td><td>(K)F/M/L/G/K/Q/E V/I/R(G)</td><td></td><td>124.43</td><td>407.5638</td><td>1220.682</td><td>-0.0051</td><td>-4.2</td><td>8.75</td><td>10155.5</td><td>5.89</td><td>HUMAN</td><td>A8MSS1</td><td>Peptidyl-prolyl cis-trans isomerase</td></tr>
<tr><td>3712</td><td>dirMS_041712_NAR_60mM_plus.6889.6957.3</td><td>3</td><td>14.21</td><td>1</td><td>5.25E+05</td><td></td><td>(K)LADLEQ</td><td>(K)L/A|D|L/E/Q/E/Q/S S K R(L)</td><td></td><td>94.5</td><td>468.5804</td><td>1403.713</td><td>0.0141</td><td>10.1</td><td>4.68</td><td>64248.4</td><td>9.08</td><td>HUMAN</td><td>Q9BW04</td><td>Specifically androgen-regulated gene protein</td></tr>
<tr><td>3713</td><td>dirMS_041712_NAR_30mM_minus2.13509.13654.2</td><td>2</td><td>14.2</td><td>0</td><td>6.19E+05</td><td></td><td>(K)TCTFILR</td><td>(K)T/C|T/F/I/L/R(G)</td><td>C:Carbamidomethylation</td><td>127.5</td><td>455.7458</td><td>910.481</td><td>0.0028</td><td>3.1</td><td>9.41</td><td>46550.7</td><td>7.81</td><td>HUMAN</td><td>B7Z1C9</td><td>Chaperonin containing TCP1, subunit 7 (Eta), isoform CRA_a</td></tr>
<tr><td>3714</td><td>dirMS_041712_NAR_30mM_minus2.13556.13556.2</td><td>2</td><td>14.2</td><td>0</td><td>1.78E+05</td><td>M357m</td><td>(R)FFmGN</td><td>(R)F/F|m|G N/Q V L/K(V)</td><td>m:Oxidized methionine</td><td>128.15</td><td>550.2855</td><td>1083.566</td><td>15.9982</td><td>3</td><td>8.75</td><td>83273.6</td><td>5.55</td><td>HUMAN</td><td>P13010</td><td>X-ray repair cross-complementing protein 5</td></tr>
<tr><td>3715</td><td>dirMS_041712_NAR_30mM_minus2.18852.18852.2</td><td>2</td><td>14.2</td><td>0</td><td>5.41E+04</td><td></td><td>(R)CGGIQY</td><td>(R)C G G\I/Q|Y|L/Q|N A/I E S R(Q)</td><td>C:Carbamidomethylation</td><td>157.12</td><td>804.8951</td><td>1608.78</td><td>0.0031</td><td>1.9</td><td>6</td><td>150202.8</td><td>9.1</td><td>HUMAN</td><td>E7EW20</td><td>Unconventional myosin-VI</td></tr>
<tr><td>3716</td><td>dirMS_041712_NAR_50mM_minus2.17698.17815.3</td><td>3</td><td>14.2</td><td>1</td><td>3.97E+05</td><td></td><td>(R)LKLPSIP</td><td>(R)L K L P S I|P\L|V|P V/S A Q K(R)</td><td></td><td>169.73</td><td>530.669</td><td>1589.999</td><td>-0.0064</td><td>-4.1</td><td>10</td><td>34958.1</td><td>9.42</td><td>HUMAN</td><td>Q15785</td><td>Mitochondrial import receptor subunit TOM34</td></tr>
<tr><td>3717</td><td>dirMS_041712_NAR_60mM_minus.8998.8998.2</td><td>2</td><td>14.2</td><td>0</td><td>9.67E+05</td><td></td><td>(K)QEYDES</td><td>(K)Q E|Y/D/E/S/G|P S I/V H R(K)</td><td></td><td>110.35</td><td>758.8539</td><td>1516.703</td><td>-0.0021</td><td>-1.4</td><td>4.65</td><td>123097.1</td><td>5.83</td><td>HUMAN</td><td>A5A3E0</td><td>POTE ankyrin domain family member F</td></tr>
<tr><td>3718</td><td>dirMS_041712_NAR_30mM_plus2.6298.6298.2</td><td>2</td><td>14.2</td><td>0</td><td>3.31E+05</td><td>M369m</td><td>(R)FGSGM</td><td>(R)F/G|S/G M N m/G R(I)</td><td>m:Oxidized methionine</td><td>87.53</td><td>486.7048</td><td>956.408</td><td>15.9946</td><td>-0.3</td><td>9.75</td><td>77799.7</td><td>9.09</td><td>HUMAN</td><td>P52272</td><td>Heterogeneous nuclear ribonucleoprotein M</td></tr>
<tr><td>3719</td><td>dirMS_041712_NAR_30mM_plus2.8303.8679.2</td><td>2</td><td>14.2</td><td>0</td><td>1.26E+06</td><td></td><td>(R)TLLEGEE</td><td>(R)T L|L|E|G/E E/S/R(M)</td><td></td><td>98.95</td><td>517.2613</td><td>1033.516</td><td>-0.0007</td><td>-0.7</td><td>4.25</td><td>66208.7</td><td>8.32</td><td>HUMAN</td><td>P04264</td><td>Keratin, type II cytoskeletal 1</td></tr>
<tr><td>3720</td><td>dirMS_041712_NAR_40mM_plus2.11505.11505.3</td><td>3</td><td>14.2</td><td>0</td><td>5.67E+04</td><td></td><td>(R)LDDTVH</td><td>(R)L/D\D/T V/H V/V/I/A T/P G R(I)</td><td></td><td>134.08</td><td>498.2739</td><td>1492.812</td><td>-0.0047</td><td>-3.1</td><td>5.21</td><td>54815.2</td><td>9.17</td><td>HUMAN</td><td>P26196</td><td>Probable ATP-dependent RNA helicase DDX6</td></tr>
<tr><td>3721</td><td>dirMS_041712_NAR_50mM_plus2.12914.12914.3</td><td>3</td><td>14.2</td><td>1</td><td>3.26E+05</td><td></td><td>(R)ELAVQI</td><td>(R)E L/A/V/Q/I Y E/E A R K(F)</td><td></td><td>135.57</td><td>483.5916</td><td>1448.774</td><td>-0.0141</td><td>-9.7</td><td>4.79</td><td>73386.6</td><td>8.73</td><td>HUMAN</td><td>B4DXX7</td><td>ATP-dependent RNA helicase DDX3Y</td></tr>
<tr><td>3722</td><td>dirMS_041712_NAR_50mM_plus2.17612.17676.3</td><td>3</td><td>14.2</td><td>1</td><td>1.27E+06</td><td></td><td>(R)VTFSGL</td><td>(R)V/T|F/S/G L R D T\V|T/P G E L L K(A)</td><td></td><td>160.82</td><td>611.6724</td><td>1833.012</td><td>-0.009</td><td>-4.9</td><td>6.04</td><td>266161</td><td>5.68</td><td>HUMAN</td><td>E9PPU0</td><td>Epiplakin</td></tr>
<tr><td>3723</td><td>dirMS_041712_NAR_120mM_minus.12496.13020.3</td><td>3</td><td>14.19</td><td>1</td><td>3.70E+06</td><td></td><td>(K)VKLEAE</td><td>(K)V K L E A E|I|A/T/Y/R(R)</td><td></td><td>123.3</td><td>431.5783</td><td>1292.721</td><td>-0.0005</td><td>-0.4</td><td>6.11</td><td>43773.3</td><td>5.26</td><td>HUMAN</td><td>F8VZY9</td><td>Keratin, type I cytoskeletal 18</td></tr>
<tr><td>3724</td><td>dirMS_041712_NAR_20mM_minus.9272.9280.3</td><td>3</td><td>14.19</td><td>0</td><td>3.43E+05</td><td></td><td>(R)AQIFAN</td><td>(R)A Q\I\F A/N|T|V/D/N A/R(I)</td><td></td><td>142.32</td><td>440.5612</td><td>1319.67</td><td>-0.0012</td><td>-0.9</td><td>5.88</td><td>43773.3</td><td>5.26</td><td>HUMAN</td><td>F8VZY9</td><td>Keratin, type I cytoskeletal 18</td></tr>
<tr><td>3725</td><td>dirMS_041712_NAR_30mM_minus2.7300.7446.2</td><td>2</td><td>14.19</td><td>0</td><td>5.44E+06</td><td></td><td>(K)LLEGEES</td><td>(K)L/L|E|G E/E/S/R(I)</td><td></td><td>94.55</td><td>466.7386</td><td>932.468</td><td>0.0016</td><td>1.7</td><td>4.25</td><td>49709.6</td><td>5.19</td><td>HUMAN</td><td>B0YJC4</td><td>Vimentin</td></tr>
<tr><td>3726</td><td>dirMS_041712_NAR_50mM_minus2.11313.11313.3</td><td>3</td><td>14.19</td><td>1</td><td>2.04E+05</td><td></td><td>(K)MLVSG</td><td>(K)M/L\V/S/G A G D I/K/L T/K(D)</td><td></td><td>132.4</td><td>444.9227</td><td>1332.756</td><td>-0.002</td><td>-1.5</td><td>8.35</td><td>55323</td><td>6.02</td><td>HUMAN</td><td>B4DPJ8</td><td>T-complex protein 1 subunit zeta</td></tr>
<tr><td>3727</td><td>dirMS_041712_NAR_50mM_minus2.12907.12907.3</td><td>3</td><td>14.19</td><td>1</td><td>1.79E+05</td><td></td><td>(K)GGELVY</td><td>(K)G G\E|L/V/Y/T D/S E A R D/V F E K(A)</td><td></td><td>141.53</td><td>638.9739</td><td>1914.908</td><td>-0.0008</td><td>-0.4</td><td>4.18</td><td>533778</td><td>5.74</td><td>HUMAN</td><td>Q15149</td><td>Plectin</td></tr>
<tr><td>3728</td><td>dirMS_041712_NAR_60mM_minus.17438.17438.3</td><td>3</td><td>14.19</td><td>0</td><td>2.98E+05</td><td></td><td>(K)GSDHSA</td><td>(K)G S D H S A S L E|P G E|L A/E L V R(S)</td><td></td><td>156.93</td><td>622.9764</td><td>1866.919</td><td>-0.0045</td><td>-2.4</td><td>4.4</td><td>12626.3</td><td>5.59</td><td>HUMAN</td><td>Q5TBR0</td><td>N-acetylneuraminic acid synthase (Fragment)</td></tr>
<tr><td>3729</td><td>dirMS_041712_NAR_30mM_plus2.20820.20820.2</td><td>2</td><td>14.19</td><td>0</td><td>2.76E+05</td><td></td><td>(R)ASLSLW</td><td>(R)A S/L/S/L|W Q A\M/E K(G)</td><td></td><td>163.05</td><td>632.3231</td><td>1263.64</td><td>-0.0013</td><td>-1</td><td>6.05</td><td>266161</td><td>5.68</td><td>HUMAN</td><td>E9PPU0</td><td>Epiplakin</td></tr>
<tr><td>3730</td><td>dirMS_041712_NAR_50mM_plus2.16049.16131.3</td><td>3</td><td>14.19</td><td>1</td><td>5.32E+05</td><td></td><td>(K)YAVLYQ</td><td>(K)Y/A|V|L/Y Q/P L F D/K R(F)</td><td></td><td>152.5</td><td>504.9436</td><td>1512.821</td><td>-0.0047</td><td>-3.1</td><td>8.49</td><td>42989.1</td><td>4.45</td><td>HUMAN</td><td>B3KNT8</td><td>Nucleosome assembly protein 1-like 1</td></tr>
<tr><td>3731</td><td>dirMS_041712_NAR_50mM_plus2.16840.16840.3</td><td>3</td><td>14.19</td><td>1</td><td>2.93E+05</td><td>M148m</td><td>(R)LDLAGR</td><td>(R)L/D/L/A/G R D L/T D Y\L m K(I)</td><td>m:Oxidized methionine</td><td>156.47</td><td>547.2842</td><td>1623.841</td><td>15.997</td><td>1.3</td><td>4.43</td><td>37482.1</td><td>5.36</td><td>HUMAN</td><td>E9PG30</td><td>Actin, gamma-enteric smooth muscle</td></tr>
<tr><td>3732</td><td>dirMS_041712_NAR_50mM_plus2.18987.18987.3</td><td>3</td><td>14.19</td><td>1</td><td>2.90E+05</td><td></td><td>(K)LALFQA</td><td>(K)L/A|L/F/Q A I G K E V V D/R(A)</td><td></td><td>168.05</td><td>520.3019</td><td>1558.895</td><td>-0.004</td><td>-2.6</td><td>6.07</td><td>266161</td><td>5.68</td><td>HUMAN</td><td>E9PPU0</td><td>Epiplakin</td></tr>
<tr><td>3733</td><td>dirMS_041712_NAR_20mM_minus.11219.11464.2</td><td>2</td><td>14.18</td><td>0</td><td>1.17E+07</td><td>M148m</td><td>(R)DLTDYL</td><td>(R)D L|T|D/Y/L/m/K(I)</td><td>m:Oxidized methionine</td><td>162.17</td><td>507.7443</td><td>998.486</td><td>15.995</td><td>0.1</td><td>4.21</td><td>37482.1</td><td>5.36</td><td>HUMAN</td><td>E9PG30</td><td>Actin, gamma-enteric smooth muscle</td></tr>
<tr><td>3734</td><td>dirMS_041712_NAR_20mM_minus.14469.14469.3</td><td>3</td><td>14.18</td><td>0</td><td>2.19E+05</td><td></td><td>(K)LAAELS</td><td>(K)L A A E\L S A T\L|E/Q A/A/A/T A R(V)</td><td></td><td>198.9</td><td>562.9713</td><td>1686.902</td><td>-0.0027</td><td>-1.6</td><td>4.53</td><td>558007.1</td><td>5.44</td><td>HUMAN</td><td>P58107</td><td>Epiplakin</td></tr>
<tr><td>3735</td><td>dirMS_041712_NAR_50mM_minus2.16950.16950.3</td><td>3</td><td>14.18</td><td>1</td><td>2.20E+05</td><td></td><td>(K)SLLGKD</td><td>(K)S L|L/G/K D V L F/L K(D)</td><td></td><td>164.27</td><td>411.5911</td><td>1232.761</td><td>-0.0025</td><td>-2.1</td><td>8.31</td><td>41827.5</td><td>7</td><td>HUMAN</td><td>B7Z7A9</td><td>Phosphoglycerate kinase</td></tr>
<tr><td>3736</td><td>dirMS_041712_NAR_50mM_minus2.9075.9093.3</td><td>3</td><td>14.18</td><td>0</td><td>1.48E+05</td><td></td><td>(R)TITVALA</td><td>(R)T I|T/V/A|L A/D/G G R/P D N T G R(G)</td><td></td><td>120.08</td><td>571.9667</td><td>1713.888</td><td>-0.0023</td><td>-1.3</td><td>5.63</td><td>107550.2</td><td>5.34</td><td>HUMAN</td><td>P49588</td><td>Alanine--tRNA ligase, cytoplasmic</td></tr>
<tr><td>3737</td><td>dirMS_041712_NAR_60mM_minus.14433.14433.3</td><td>3</td><td>14.18</td><td>1</td><td>1.62E+05</td><td></td><td>(R)IQLVEEE</td><td>(R)I Q|L/V/E E E/L D R A Q E R(L)</td><td></td><td>139.57</td><td>576.6347</td><td>1727.892</td><td>-0.0027</td><td>-1.6</td><td>4.25</td><td>31923.6</td><td>4.85</td><td>HUMAN</td><td>B7Z596</td><td>Tropomyosin alpha-1 chain</td></tr>
<tr><td>3738</td><td>dirMS_041712_NAR_20mM_plus.16240.16332.2</td><td>2</td><td>14.18</td><td>0</td><td>3.80E+05</td><td>M221m</td><td>(R)ALTVPEL</td><td>(R)A L|T|V|P E L T/Q Q/m F D\A K(N)</td><td>m:Oxidized methionine</td><td>184.3</td><td>854.4341</td><td>1691.867</td><td>15.9936</td><td>-0.7</td><td>4.37</td><td>42831.5</td><td>4.86</td><td>HUMAN</td><td>A8K854</td><td>HCG1983504, isoform CRA_f</td></tr>
<tr><td>3739</td><td>dirMS_041712_NAR_30mM_plus2.5774.5774.2</td><td>2</td><td>14.18</td><td>0</td><td>2.51E+05</td><td></td><td>(K)TSEVNC</td><td>(K)T|S|E|V/N C/Y R(C)</td><td>C:Carbamidomethylation</td><td>85.05</td><td>514.7261</td><td>1028.447</td><td>-0.0016</td><td>-1.6</td><td>5.66</td><td>18946.7</td><td>9.13</td><td>HUMAN</td><td>B4DP17</td><td>Cellular nucleic acid-binding protein</td></tr>
<tr><td>3740</td><td>dirMS_041712_NAR_50mM_plus2.19152.19152.3</td><td>3</td><td>14.18</td><td>1</td><td>1.72E+06</td><td></td><td>(K)LIQLME</td><td>(K)L I|Q/L M/E/E I M A E K E/N K(T)</td><td></td><td>169.7</td><td>606.9845</td><td>1818.934</td><td>0.005</td><td>2.7</td><td>4.49</td><td>73202</td><td>9.19</td><td>HUMAN</td><td>C9JMU5</td><td>Probable ATP-dependent RNA helicase DDX17</td></tr>
<tr><td>3741</td><td>dirMS_041712_NAR_50mM_plus2.6561.6561.3</td><td>3</td><td>14.18</td><td>0</td><td>6.49E+05</td><td>M434m</td><td>(R)LESGmC</td><td>(R)L/E|S G/m Q N/M S I/H\T K(T)</td><td>m:Oxidized methionine</td><td>98.9</td><td>497.9026</td><td>1475.698</td><td>15.9951</td><td>0.1</td><td>6.75</td><td>56607.4</td><td>5.37</td><td>HUMAN</td><td>F8VXB4</td><td>Keratin, type II cytoskeletal 8</td></tr>
<tr><td>3742</td><td>dirMS_041712_NAR_60mM_plus.15213.15482.3</td><td>3</td><td>14.18</td><td>0</td><td>9.78E+05</td><td></td><td>(R)TVVTGIE</td><td>(R)T V|V/T|G I/E M/F/H K(S)</td><td></td><td>137</td><td>421.2292</td><td>1261.661</td><td>0.0121</td><td>9.6</td><td>6.41</td><td>49883</td><td>7.3</td><td>HUMAN</td><td>P49411</td><td>Elongation factor Tu, mitochondrial</td></tr>
<tr><td>3743</td><td>dirMS_041712_NAR_60mM_plus.17637.17637.3</td><td>3</td><td>14.18</td><td>1</td><td>4.81E+05</td><td></td><td>(R)LMDLLG</td><td>(R)L/M/D\L/L/G E\G L K/R(S)</td><td></td><td>149.12</td><td>415.5712</td><td>1244.703</td><td>-0.0041</td><td>-3.3</td><td>6.07</td><td>52817.9</td><td>6.2</td><td>HUMAN</td><td>F5H3T8</td><td>Arginine--tRNA ligase, cytoplasmic</td></tr>
<tr><td>3744</td><td>dirMS_041712_NAR_60mM_plus.7158.7158.2</td><td>2</td><td>14.18</td><td>0</td><td>1.72E+06</td><td></td><td>(K)STTTGH</td><td>(K)S T/T/T/G H L I|Y/K(C)</td><td></td><td>95.58</td><td>560.8031</td><td>1120.6</td><td>-0.0008</td><td>-0.7</td><td>8.34</td><td>12137.8</td><td>9.3</td><td>HUMAN</td><td>A6PW80</td><td>Elongation factor 1-alpha 1 (Fragment)</td></tr>
<tr><td>3745</td><td>dirMS_041712_NAR_30mM_minus2.13974.13974.2</td><td>2</td><td>14.17</td><td>0</td><td>3.15E+05</td><td></td><td>(R)LAGLFN</td><td>(R)L A|G\L/F N E/Q R(K)</td><td></td><td>129.43</td><td>524.2789</td><td>1047.558</td><td>-0.0076</td><td>-7.3</td><td>6</td><td>63098.1</td><td>7.83</td><td>HUMAN</td><td>B7Z909</td><td>Hypoxia up-regulated protein 1</td></tr>
<tr><td>3746</td><td>dirMS_041712_NAR_40mM_minus2.11311.11370.3</td><td>3</td><td>14.17</td><td>0</td><td>1.95E+05</td><td></td><td>(K)LVTSIGD</td><td>(K)L V|T/S/I/G D V/V N H D/P V V G D R(L)</td><td></td><td>150.32</td><td>631.3335</td><td>1891.987</td><td>-0.0013</td><td>-0.7</td><td>4.41</td><td>27786.2</td><td>5.93</td><td>HUMAN</td><td>H0Y4Z6</td><td>Phosphorylase (Fragment)</td></tr>
<tr><td>3747</td><td>dirMS_041712_NAR_50mM_minus2.9405.9405.3</td><td>3</td><td>14.17</td><td>0</td><td>2.72E+05</td><td></td><td>(K)TGVAVN</td><td>(K)T\G/V|A V/N K/P A E\F T V D/A K(H)</td><td></td><td>122.03</td><td>549.6316</td><td>1646.875</td><td>0.0054</td><td>3.3</td><td>5.73</td><td>283472.4</td><td>5.71</td><td>HUMAN</td><td>P21333</td><td>Filamin-A</td></tr>
<tr><td>3748</td><td>dirMS_041712_NAR_60mM_minus.4809.4809.3</td><td>3</td><td>14.17</td><td>1</td><td>7.13E+05</td><td>M137m</td><td>(R)mQNDT</td><td>(R)m Q/N D/T A E N E T/T E/K E|E K(S)</td><td>m:Oxidized methionine</td><td>79.37</td><td>638.2735</td><td>1896.813</td><td>15.9932</td><td>-0.9</td><td>4.14</td><td>54214.9</td><td>5.66</td><td>HUMAN</td><td>C9J813</td><td>Caldesmon (Fragment)</td></tr>
<tr><td>3749</td><td>dirMS_041712_NAR_60mM_minus.5863.5863.2</td><td>2</td><td>14.17</td><td>1</td><td>2.81E+06</td><td></td><td>(K)DSYVGD</td><td>(K)D S|Y|V/G D|E A/Q S K R(G)</td><td></td><td>88.47</td><td>677.8144</td><td>1354.623</td><td>-0.0018</td><td>-1.3</td><td>4.56</td><td>32616.5</td><td>5.18</td><td>HUMAN</td><td>A6NL76</td><td>Actin, alpha skeletal muscle</td></tr>
<tr><td>3750</td><td>dirMS_041712_NAR_30mM_plus2.12016.12115.2</td><td>2</td><td>14.17</td><td>0</td><td>6.51E+05</td><td></td><td>(K)SSLGPV</td><td>(K)S S|L/G|P V/G L/D K(M)</td><td></td><td>116.38</td><td>486.775</td><td>972.536</td><td>0.0067</td><td>6.9</td><td>5.55</td><td>47423</td><td>5.43</td><td>HUMAN</td><td>E7ERF2</td><td>T-complex protein 1 subunit alpha</td></tr>
<tr><td>3751</td><td>dirMS_041712_NAR_40mM_plus2.17048.17048.3</td><td>3</td><td>14.17</td><td>0</td><td>4.41E+05</td><td></td><td>(R)IHFPLAT</td><td>(R)I H/F P L A T Y/A/P V I/S A E K(A)</td><td></td><td>167.57</td><td>586.3246</td><td>1756.963</td><td>-0.004</td><td>-2.3</td><td>6.75</td><td>49012.3</td><td>4.9</td><td>HUMAN</td><td>A8MUB1</td><td>Tubulin alpha-4A chain</td></tr>
<tr><td>3752</td><td>dirMS_041712_NAR_40mM_plus2.9024.9024.3</td><td>3</td><td>14.17</td><td>0</td><td>5.80E+04</td><td></td><td>(R)AALAHS</td><td>(R)A A L A H S E E\V|T|A/S Q/V/A A T K(T)</td><td></td><td>115.18</td><td>595.3161</td><td>1783.919</td><td>0.0153</td><td>8.6</td><td>5.4</td><td>23903</td><td>4.94</td><td>HUMAN</td><td>H0YDN1</td><td>Plectin (Fragment)</td></tr>
<tr><td>3753</td><td>dirMS_041712_NAR_60mM_plus.8369.8371.3</td><td>3</td><td>14.17</td><td>1</td><td>6.44E+05</td><td></td><td>(K)EPLYEK</td><td>(K)E P/L/Y/E/K/D S\S/V/A A/R(F)</td><td></td><td>102.25</td><td>488.9148</td><td>1464.733</td><td>-0.0031</td><td>-2.1</td><td>4.68</td><td>26283.8</td><td>8.96</td><td>HUMAN</td><td>O43809</td><td>Cleavage and polyadenylation specificity factor subunit 5</td></tr>
<tr><td>3754</td><td>dirMS_041712_NAR_120mM_minus.8483.8483.3</td><td>3</td><td>14.16</td><td>1</td><td>4.31E+05</td><td></td><td>(R)YLAEVA</td><td>(R)Y/L/A/E V A/T/G E/K/R(A)</td><td></td><td>100.85</td><td>412.8849</td><td>1236.658</td><td>-0.0181</td><td>-14.7</td><td>6.14</td><td>28473.3</td><td>4.8</td><td>HUMAN</td><td>P61981</td><td>14-3-3 protein gamma</td></tr>
<tr><td>3755</td><td>dirMS_041712_NAR_30mM_minus2.15680.15680.2</td><td>2</td><td>14.16</td><td>0</td><td>9.77E+04</td><td></td><td>(R)GSTDNL</td><td>(R)G S T D/N/L|M/D/D I E/R(A)</td><td></td><td>138.77</td><td>683.3009</td><td>1365.595</td><td>-0.0006</td><td>-0.4</td><td>3.84</td><td>58214.7</td><td>5.25</td><td>HUMAN</td><td>B4DEM7</td><td>T-complex protein 1 subunit theta</td></tr>
<tr><td>3756</td><td>dirMS_041712_NAR_30mM_minus2.8605.8704.2</td><td>2</td><td>14.16</td><td>0</td><td>8.94E+05</td><td></td><td>(K)VLTPTQ</td><td>(K)V L|T|P/T Q V K(N)</td><td></td><td>101.9</td><td>443.2733</td><td>885.54</td><td>-0.0011</td><td>-1.2</td><td>8.72</td><td>21170.5</td><td>7.02</td><td>HUMAN</td><td>P30086</td><td>Phosphatidylethanolamine-binding protein 1</td></tr>
<tr><td>3757</td><td>dirMS_041712_NAR_40mM_minus2.11322.11322.3</td><td>3</td><td>14.16</td><td>0</td><td>2.05E+04</td><td></td><td>(K)VALLLLD</td><td>(K)V A/L L|L/L D\Q/G A S P|H A A/A K(N)</td><td></td><td>150</td><td>558.9882</td><td>1674.954</td><td>-0.0037</td><td>-2.2</td><td>6.71</td><td>482683.6</td><td>6.07</td><td>HUMAN</td><td>Q12955</td><td>Ankyrin-3</td></tr>
<tr><td>3758</td><td>dirMS_041712_NAR_40mM_minus2.14260.14260.3</td><td>3</td><td>14.16</td><td>1</td><td>1.72E+05</td><td></td><td>(K)ITSDEPL</td><td>(K)I/T|S D E/P L T K D D I/P V F L R(H)</td><td></td><td>168.37</td><td>653.6833</td><td>1959.043</td><td>-0.008</td><td>-4.1</td><td>4.23</td><td>22313.1</td><td>4.9</td><td>HUMAN</td><td>B1AHE4</td><td>Ataxin 10 (Fragment)</td></tr>
<tr><td>3759</td><td>dirMS_041712_NAR_50mM_minus2.10500.10500.3</td><td>3</td><td>14.16</td><td>0</td><td>2.85E+05</td><td>M35m</td><td>(K)AASDIA</td><td>(K)A A|S D I/A m/T E/L|P/P T/H/P I R(L)</td><td>m:Oxidized methionine</td><td>127.88</td><td>612.6486</td><td>1819.937</td><td>15.9942</td><td>-0.4</td><td>5.32</td><td>12926.1</td><td>4.4</td><td>HUMAN</td><td>K7EM20</td><td>14-3-3 protein epsilon (Fragment)</td></tr>
<tr><td>3760</td><td>dirMS_041712_NAR_60mM_minus.17285.17448.3</td><td>3</td><td>14.16</td><td>0</td><td>3.22E+05</td><td></td><td>(K)GSDHSA</td><td>(K)G S D H\S A S L E|P G/E|L/A/E L V R(S)</td><td></td><td>156.83</td><td>622.9764</td><td>1866.919</td><td>-0.0045</td><td>-2.4</td><td>4.4</td><td>12626.3</td><td>5.59</td><td>HUMAN</td><td>Q5TBR0</td><td>N-acetylneuraminic acid synthase (Fragment)</td></tr>
<tr><td>3761</td><td>dirMS_041712_NAR_60mM_minus.6811.7266.3</td><td>3</td><td>14.16</td><td>0</td><td>1.27E+06</td><td>M434m</td><td>(R)LESGmC</td><td>(R)L/E|S/G m\Q/N M/S I/H/T K(T)</td><td>m:Oxidized methionine</td><td>97.23</td><td>497.9023</td><td>1475.698</td><td>15.9942</td><td>-0.5</td><td>6.75</td><td>56607.4</td><td>5.37</td><td>HUMAN</td><td>F8VXB4</td><td>Keratin, type II cytoskeletal 8</td></tr>
<tr><td>3762</td><td>dirMS_041712_NAR_60mM_minus.9089.9089.2</td><td>2</td><td>14.16</td><td>0</td><td>9.67E+05</td><td></td><td>(K)QEYDES</td><td>(K)Q E|Y D/E/S/G P S/I V/H/R(K)</td><td></td><td>110.35</td><td>758.8539</td><td>1516.703</td><td>-0.0021</td><td>-1.4</td><td>4.65</td><td>123097.1</td><td>5.83</td><td>HUMAN</td><td>A5A3E0</td><td>POTE ankyrin domain family member F</td></tr>
<tr><td>3763</td><td>dirMS_041712_NAR_50mM_plus2.16314.16314.3</td><td>3</td><td>14.16</td><td>1</td><td>7.02E+05</td><td></td><td>(K)CEFQDA</td><td>(K)C E/F/Q D A|Y\V/L/L S E K K(I)</td><td>C:Carbamidomethylation</td><td>152.62</td><td>577.2866</td><td>1729.847</td><td>-0.0013</td><td>-0.8</td><td>4.68</td><td>61224.7</td><td>5.7</td><td>HUMAN</td><td>P10809</td><td>60 kDa heat shock protein, mitochondrial</td></tr>
<tr><td>3764</td><td>dirMS_041712_NAR_30mM_minus2.8001.8001.2</td><td>2</td><td>14.15</td><td>0</td><td>1.36E+05</td><td></td><td>(K)SLAAEEE</td><td>(K)S L|A|A/E/E E/A A/R(Q)</td><td></td><td>96.82</td><td>523.7597</td><td>1046.511</td><td>0.0008</td><td>0.8</td><td>4.25</td><td>533778</td><td>5.74</td><td>HUMAN</td><td>Q15149</td><td>Plectin</td></tr>
<tr><td>3765</td><td>dirMS_041712_NAR_50mM_minus2.15892.15892.3</td><td>3</td><td>14.15</td><td>0</td><td>9.69E+04</td><td></td><td>(K)INLEAAE</td><td>(K)I/N|L E/A A E L G E/I/S D I H/T K(L)</td><td></td><td>158.72</td><td>618.326</td><td>1852.965</td><td>-0.0016</td><td>-0.9</td><td>4.4</td><td>84268.2</td><td>6.32</td><td>HUMAN</td><td>Q9UHD2</td><td>Serine/threonine-protein kinase TBK1</td></tr>
<tr><td>3766</td><td>dirMS_041712_NAR_60mM_minus.12240.12240.3</td><td>3</td><td>14.15</td><td>0</td><td>2.05E+05</td><td></td><td>(K)LAQAN</td><td>(K)L A/Q|A N G W/G V/M V/S H/R(S)</td><td></td><td>128.17</td><td>509.2631</td><td>1525.769</td><td>0.0055</td><td>3.6</td><td>9.75</td><td>47510.4</td><td>7.04</td><td>HUMAN</td><td>P06733</td><td>Alpha-enolase</td></tr>
<tr><td>3767</td><td>dirMS_041712_NAR_120mM_plus.12619.13106.3</td><td>3</td><td>14.15</td><td>1</td><td>3.54E+06</td><td></td><td>(K)VKLEAE</td><td>(K)V K L E\A\E|I|A/T/Y/R(R)</td><td></td><td>121.43</td><td>431.5774</td><td>1292.721</td><td>-0.0032</td><td>-2.5</td><td>6.11</td><td>43773.3</td><td>5.26</td><td>HUMAN</td><td>F8VZY9</td><td>Keratin, type I cytoskeletal 18</td></tr>
<tr><td>3768</td><td>dirMS_041712_NAR_30mM_plus2.9838.9838.2</td><td>2</td><td>14.15</td><td>0</td><td>2.05E+05</td><td></td><td>(K)LNCSPD</td><td>(K)L/N|C|S|P D/S F R(C)</td><td>C:Carbamidomethylation</td><td>104.85</td><td>548.2495</td><td>1095.489</td><td>0.003</td><td>2.7</td><td>5.84</td><td>141616.6</td><td>6.1</td><td>HUMAN</td><td>B7ZKM8</td><td>Protein transport protein Sec24B</td></tr>
<tr><td>3769</td><td>dirMS_041712_NAR_50mM_plus2.13165.13165.3</td><td>3</td><td>14.15</td><td>1</td><td>9.66E+05</td><td></td><td>(R)ELNITA</td><td>(R)E L/N I/T\A/A K E/I/E/V/G G G R(K)</td><td></td><td>137.7</td><td>552.9671</td><td>1656.892</td><td>-0.0048</td><td>-2.9</td><td>4.79</td><td>21311.5</td><td>10.28</td><td>HUMAN</td><td>B5MCP9</td><td>40S ribosomal protein S7</td></tr>
<tr><td>3770</td><td>dirMS_041712_NAR_50mM_plus2.17479.17479.3</td><td>3</td><td>14.15</td><td>0</td><td>6.37E+05</td><td></td><td>(R)IMEVID</td><td>(R)I/M|E V/I D/A I/T T T A Q S H Q R(T)</td><td></td><td>160.35</td><td>638.6636</td><td>1913.975</td><td>0.0013</td><td>0.7</td><td>5.32</td><td>85815.7</td><td>7.35</td><td>HUMAN</td><td>P17858</td><td>6-phosphofructokinase, liver type</td></tr>
<tr><td>3771</td><td>dirMS_041712_NAR_50mM_plus2.19270.19270.3</td><td>3</td><td>14.15</td><td>1</td><td>4.32E+05</td><td></td><td>(R)LKVPPA</td><td>(R)L K/V/P P A I\N Q/F/T Q A L D R(Q)</td><td></td><td>169.82</td><td>604.3351</td><td>1811.017</td><td>-0.0266</td><td>-14.7</td><td>8.75</td><td>30166.2</td><td>10.79</td><td>HUMAN</td><td>P62424</td><td>60S ribosomal protein L7a</td></tr>
<tr><td>3772</td><td>dirMS_041712_NAR_50mM_plus2.19657.19657.3</td><td>3</td><td>14.15</td><td>0</td><td>4.09E+04</td><td></td><td>(K)IFTPIVE</td><td>(K)I F/T/P I/V/E H L G L Q/I R(F)</td><td></td><td>173.07</td><td>545.9893</td><td>1635.958</td><td>-0.0047</td><td>-2.9</td><td>6.75</td><td>15289.3</td><td>10.14</td><td>HUMAN</td><td>F8WBJ6</td><td>RNA-binding protein PNO1</td></tr>
<tr><td>3773</td><td>dirMS_041712_NAR_50mM_plus2.19896.19896.3</td><td>3</td><td>14.15</td><td>1</td><td>8.17E+04</td><td></td><td>(R)ILTEAEI</td><td>(R)I/L|T E A/E I/D A/H L V A L A E R D(-)</td><td></td><td>172.9</td><td>660.3539</td><td>1979.044</td><td>0.0028</td><td>1.4</td><td>4.33</td><td>19157.6</td><td>5.18</td><td>HUMAN</td><td>B4DQR4</td><td>Proteasome subunit alpha type-6</td></tr>
<tr><td>3774</td><td>dirMS_041712_NAR_60mM_plus.11672.11672.3</td><td>3</td><td>14.15</td><td>1</td><td>2.93E+06</td><td></td><td>(K)KAEAGA</td><td>(K)K A E A G A G S\A/T/E/F/Q/F/R(G)</td><td></td><td>118.23</td><td>523.9277</td><td>1569.766</td><td>0.003</td><td>1.9</td><td>6.14</td><td>18897.4</td><td>10.15</td><td>HUMAN</td><td>P46783</td><td>40S ribosomal protein S10</td></tr>
<tr><td>3775</td><td>dirMS_041712_NAR_60mM_plus.9421.9421.3</td><td>3</td><td>14.15</td><td>1</td><td>4.42E+05</td><td></td><td>(K)CLDENL</td><td>(K)C/L|D|E/N L E/D A S Q C K K(L)</td><td>C:Carbamidomethylation</td><td>107.22</td><td>570.5876</td><td>1709.747</td><td>0.0013</td><td>0.8</td><td>4.32</td><td>14458.2</td><td>9.22</td><td>HUMAN</td><td>Q5JTJ3</td><td>Cytochrome c oxidase assembly factor 6 homolog</td></tr>
<tr><td>3776</td><td>dirMS_041712_NAR_20mM_minus.9727.9738.2</td><td>2</td><td>14.14</td><td>0</td><td>2.44E+05</td><td></td><td>(K)LVIITAG</td><td>(K)L V|I|I/T/A G/A R(Q)</td><td></td><td>147.62</td><td>457.2947</td><td>913.583</td><td>-0.0008</td><td>-0.9</td><td>9.75</td><td>12539.4</td><td>5.78</td><td>HUMAN</td><td>F5GXH2</td><td>L-lactate dehydrogenase A chain (Fragment)</td></tr>
<tr><td>3777</td><td>dirMS_041712_NAR_60mM_minus.15709.15849.3</td><td>3</td><td>14.14</td><td>1</td><td>3.91E+05</td><td></td><td>(K)KANLQI</td><td>(K)K A N L Q\I D\Q\I|N|T|D/L/N L E R(S)</td><td></td><td>148.05</td><td>666.6911</td><td>1998.061</td><td>-0.0027</td><td>-1.3</td><td>4.56</td><td>227783.5</td><td>5.5</td><td>HUMAN</td><td>P35579</td><td>Myosin-9</td></tr>
<tr><td>3778</td><td>dirMS_041712_NAR_60mM_minus.9149.9149.3</td><td>3</td><td>14.14</td><td>1</td><td>5.12E+04</td><td></td><td>(R)QLEEAE</td><td>(R)Q L/E/E/A/E E E/A Q/R A N\A S R(R)</td><td></td><td>110.88</td><td>610.9566</td><td>1830.858</td><td>-0.0024</td><td>-1.3</td><td>4.33</td><td>227783.5</td><td>5.5</td><td>HUMAN</td><td>P35579</td><td>Myosin-9</td></tr>
<tr><td>3779</td><td>dirMS_041712_NAR_40mM_plus2.11789.11789.3</td><td>3</td><td>14.14</td><td>1</td><td>2.76E+05</td><td></td><td>(R)IVAPGK</td><td>(R)I V|A/P G\K G/I L A A D\E/S T G S I A K(R)</td><td></td><td>135.42</td><td>633.3568</td><td>1898.059</td><td>-0.0035</td><td>-1.8</td><td>6.07</td><td>30711.4</td><td>7.15</td><td>HUMAN</td><td>H3BPS8</td><td>Fructose-bisphosphate aldolase A (Fragment)</td></tr>
<tr><td>3780</td><td>dirMS_041712_NAR_50mM_plus2.15958.15958.3</td><td>3</td><td>14.14</td><td>1</td><td>7.30E+05</td><td></td><td>(R)LKLPAV</td><td>(R)L K\L P A\V|V/T/A/D/L/R(L)</td><td></td><td>152.03</td><td>432.6039</td><td>1295.805</td><td>-0.0074</td><td>-5.7</td><td>8.75</td><td>28071.3</td><td>8.6</td><td>HUMAN</td><td>P38117</td><td>Electron transfer flavoprotein subunit beta</td></tr>
<tr><td>3781</td><td>dirMS_041712_NAR_60mM_plus.13334.13562.3</td><td>3</td><td>14.14</td><td>1</td><td>5.66E+05</td><td></td><td>(R)ISVYYNE</td><td>(R)I/S|V/Y/Y N E/A/T G G K Y V P R(A)</td><td></td><td>128.32</td><td>606.3113</td><td>1816.923</td><td>-0.0035</td><td>-1.9</td><td>8.43</td><td>27549.9</td><td>4.39</td><td>HUMAN</td><td>E9PBJ4</td><td>Tubulin beta chain</td></tr>
<tr><td>3782</td><td>dirMS_041712_NAR_60mM_plus.9405.9405.2</td><td>2</td><td>14.14</td><td>0</td><td>1.98E+06</td><td></td><td>(R)IGAEVY</td><td>(R)I/G|A|E V/Y/H N L/K(N)</td><td></td><td>107.55</td><td>572.3143</td><td>1143.616</td><td>0.0056</td><td>4.9</td><td>6.75</td><td>21077</td><td>9.05</td><td>HUMAN</td><td>K7EM90</td><td>Alpha-enolase (Fragment)</td></tr>
<tr><td>3783</td><td>dirMS_041712_NAR_50mM_minus2.17262.17431.3</td><td>3</td><td>14.13</td><td>0</td><td>2.00E+05</td><td></td><td>(R)DFLLKP</td><td>(R)D F|L|L/K/P E L/L R(A)</td><td></td><td>167.07</td><td>415.255</td><td>1243.741</td><td>0.0096</td><td>7.7</td><td>6.07</td><td>30944.2</td><td>6.52</td><td>HUMAN</td><td>B1Q2N1</td><td>ATP-dependent RNA helicase DDX39A</td></tr>
<tr><td>3784</td><td>dirMS_041712_NAR_50mM_minus2.9377.9394.3</td><td>3</td><td>14.13</td><td>0</td><td>2.61E+05</td><td></td><td>(K)TGVAVN</td><td>(K)T/G\V|A|V/N K/P A E F/T V D/A K(H)</td><td></td><td>121.98</td><td>549.6316</td><td>1646.875</td><td>0.0054</td><td>3.3</td><td>5.73</td><td>283472.4</td><td>5.71</td><td>HUMAN</td><td>P21333</td><td>Filamin-A</td></tr>
<tr><td>3785</td><td>dirMS_041712_NAR_20mM_plus.12987.12987.2</td><td>2</td><td>14.13</td><td>0</td><td>1.49E+05</td><td></td><td>(R)LVVPAS</td><td>(R)L V|V|P A S Q C G S L I/G K(G)</td><td>C:Carbamidomethylation</td><td>161.83</td><td>714.8991</td><td>1428.788</td><td>0.003</td><td>2.1</td><td>8.75</td><td>32621</td><td>8.75</td><td>HUMAN</td><td>B4DLC0</td><td>Poly(rC)-binding protein 2</td></tr>
<tr><td>3786</td><td>dirMS_041712_NAR_20mM_plus.16767.16767.2</td><td>2</td><td>14.13</td><td>0</td><td>4.11E+05</td><td></td><td>(K)LGGSAV</td><td>(K)L G G/S|A/V|I/S L/E\G\K|P L(-)</td><td></td><td>186.02</td><td>670.8917</td><td>1340.778</td><td>-0.0023</td><td>-1.7</td><td>6</td><td>22956</td><td>8.8</td><td>HUMAN</td><td>E9PK25</td><td>Cofilin-1</td></tr>
<tr><td>3787</td><td>dirMS_041712_NAR_40mM_plus2.13255.13255.3</td><td>3</td><td>14.13</td><td>1</td><td>2.42E+06</td><td></td><td>(K)VGEVIV</td><td>(K)V/G|E/V I V/T K D D A M L L/K(G)</td><td></td><td>145.27</td><td>544.307</td><td>1630.908</td><td>-0.002</td><td>-1.2</td><td>4.56</td><td>61224.7</td><td>5.7</td><td>HUMAN</td><td>P10809</td><td>60 kDa heat shock protein, mitochondrial</td></tr>
<tr><td>3788</td><td>dirMS_041712_NAR_50mM_plus2.14453.14538.3</td><td>3</td><td>14.13</td><td>1</td><td>1.66E+04</td><td></td><td>(K)NLKVTA</td><td>(K)N L K V T A\V/P/T/L L/K(Y)</td><td></td><td>143.12</td><td>432.9459</td><td>1296.825</td><td>-0.0018</td><td>-1.4</td><td>10</td><td>11462.8</td><td>5.18</td><td>HUMAN</td><td>I3L2R6</td><td>Thioredoxin domain-containing protein 17</td></tr>
<tr><td>3789</td><td>dirMS_041712_NAR_50mM_plus2.16274.16274.3</td><td>3</td><td>14.13</td><td>1</td><td>1.28E+05</td><td></td><td>(K)ILLAELE</td><td>(K)I/L/L/A|E L/E Q L K G Q G K(S)</td><td></td><td>153.83</td><td>513.9741</td><td>1539.911</td><td>-0.0027</td><td>-1.8</td><td>6.14</td><td>49709.6</td><td>5.19</td><td>HUMAN</td><td>B0YJC4</td><td>Vimentin</td></tr>
<tr><td>3790</td><td>dirMS_041712_NAR_50mM_plus2.21004.21345.3</td><td>3</td><td>14.13</td><td>0</td><td>8.90E+06</td><td></td><td>(R)LHFFMP</td><td>(R)L H\F\F\M\P/G\F|A|P\L/T S R(G)</td><td></td><td>181.53</td><td>540.9498</td><td>1620.835</td><td>-0.0007</td><td>-0.4</td><td>9.75</td><td>42230.8</td><td>4.77</td><td>HUMAN</td><td>A6NKZ8</td><td>Putative tubulin beta chain-like protein ENSP00000290377</td></tr>
<tr><td>3791</td><td>dirMS_041712_NAR_50mM_plus2.6622.6622.3</td><td>3</td><td>14.13</td><td>1</td><td>1.11E+05</td><td></td><td>(R)ITGDPY</td><td>(R)I/T|G D/P/Y/K/V Q/Q A K(E)</td><td></td><td>99.47</td><td>449.9102</td><td>1347.727</td><td>-0.0106</td><td>-7.9</td><td>8.5</td><td>70034.8</td><td>6.89</td><td>HUMAN</td><td>B4DT31</td><td>Far upstream element-binding protein 1</td></tr>
<tr><td>3792</td><td>dirMS_041712_NAR_60mM_plus.20121.20121.3</td><td>3</td><td>14.13</td><td>0</td><td>1.33E+06</td><td></td><td>(R)IHFPLAT</td><td>(R)I H F P L A\T Y/A/P/V I/S A E K(A)</td><td></td><td>165.92</td><td>586.3246</td><td>1756.963</td><td>-0.004</td><td>-2.3</td><td>6.75</td><td>49012.3</td><td>4.9</td><td>HUMAN</td><td>A8MUB1</td><td>Tubulin alpha-4A chain</td></tr>
<tr><td>3793</td><td>dirMS_041712_NAR_30mM_minus2.15942.15942.2</td><td>2</td><td>14.12</td><td>0</td><td>3.59E+05</td><td></td><td>(R)FGLNVS</td><td>(R)F/G|L/N/V/S S I S R(K)</td><td></td><td>140.05</td><td>540.2895</td><td>1079.584</td><td>-0.0127</td><td>-11.7</td><td>9.75</td><td>24161.9</td><td>5.27</td><td>HUMAN</td><td>F8VZQ9</td><td>SAP domain-containing ribonucleoprotein</td></tr>
<tr><td>3794</td><td>dirMS_041712_NAR_40mM_minus2.11209.11209.3</td><td>3</td><td>14.12</td><td>1</td><td>1.92E+04</td><td>M250m</td><td>(R)DSDIIED</td><td>(R)D S/D|I/I/E D/V m V K/D L Q K(L)</td><td>m:Oxidized methionine</td><td>149.23</td><td>588.6311</td><td>1747.878</td><td>16.0005</td><td>3.2</td><td>4.04</td><td>55757.3</td><td>5.55</td><td>HUMAN</td><td>O94919</td><td>Endonuclease domain-containing 1 protein</td></tr>
<tr><td>3795</td><td>dirMS_041712_NAR_60mM_minus.14911.14911.3</td><td>3</td><td>14.12</td><td>1</td><td>3.69E+05</td><td></td><td>(R)IKIGDPL</td><td>(R)I K I G D\P\L|L/E/D/T R(M)</td><td></td><td>142.82</td><td>457.2595</td><td>1369.769</td><td>-0.0046</td><td>-3.4</td><td>4.56</td><td>47309.6</td><td>5.42</td><td>HUMAN</td><td>B4DXY7</td><td>4-trimethylaminobutyraldehyde dehydrogenase</td></tr>
<tr><td>3796</td><td>dirMS_041712_NAR_120mM_plus.23964.24093.3</td><td>3</td><td>14.12</td><td>1</td><td>1.51E+05</td><td></td><td>(K)EIIDLVL</td><td>(K)E I/I/D/L/V/L D/R I/R(K)</td><td></td><td>180.03</td><td>452.2725</td><td>1354.805</td><td>-0.0023</td><td>-1.7</td><td>4.56</td><td>58642</td><td>4.95</td><td>HUMAN</td><td>F5H5D3</td><td>Tubulin alpha-1C chain</td></tr>
<tr><td>3797</td><td>dirMS_041712_NAR_30mM_plus2.11689.11689.2</td><td>2</td><td>14.12</td><td>0</td><td>5.34E+05</td><td></td><td>(K)VASGCL</td><td>(K)V/A|S/G/C/L/D I/N S S V K(G)</td><td>C:Carbamidomethylation</td><td>114.8</td><td>675.3393</td><td>1349.673</td><td>-0.0016</td><td>-1.2</td><td>5.81</td><td>56837</td><td>7.19</td><td>HUMAN</td><td>E7ETT1</td><td>Propionyl-CoA carboxylase beta chain, mitochondrial</td></tr>
<tr><td>3798</td><td>dirMS_041712_NAR_30mM_plus2.14815.15006.2</td><td>2</td><td>14.12</td><td>0</td><td>2.30E+06</td><td></td><td>(K)DVNAAL</td><td>(K)D/V|N|A|A/I/A A I K(T)</td><td></td><td>130.12</td><td>493.2867</td><td>985.568</td><td>-0.0015</td><td>-1.6</td><td>5.84</td><td>49012.3</td><td>4.9</td><td>HUMAN</td><td>A8MUB1</td><td>Tubulin alpha-4A chain</td></tr>
<tr><td>3799</td><td>dirMS_041712_NAR_40mM_plus2.10721.10721.3</td><td>3</td><td>14.12</td><td>1</td><td>4.29E+05</td><td></td><td>(R)DGKLVS</td><td>(R)D G K\L/V S E|S/S/D V L/P K(-)</td><td></td><td>128.63</td><td>491.9315</td><td>1473.78</td><td>0.0004</td><td>0.3</td><td>4.56</td><td>56607.4</td><td>5.37</td><td>HUMAN</td><td>F8VXB4</td><td>Keratin, type II cytoskeletal 8</td></tr>
<tr><td>3800</td><td>dirMS_041712_NAR_40mM_plus2.15936.15936.3</td><td>3</td><td>14.12</td><td>0</td><td>2.88E+05</td><td></td><td>(K)VAIVKP</td><td>(K)V A|I\V\K P G V/P/M|E/I V L N K(E)</td><td></td><td>160.9</td><td>569.6778</td><td>1707.024</td><td>-0.0049</td><td>-2.9</td><td>8.56</td><td>73146.9</td><td>5.45</td><td>HUMAN</td><td>E9PJ21</td><td>Hypoxia up-regulated protein 1 (Fragment)</td></tr>
<tr><td>3801</td><td>dirMS_041712_NAR_40mM_plus2.8276.8276.2</td><td>2</td><td>14.12</td><td>1</td><td>8.43E+04</td><td></td><td>(R)KQNDV</td><td>(R)K Q N D/V/F/G E\A|E/Q(-)</td><td></td><td>109.62</td><td>632.7929</td><td>1264.58</td><td>-0.0019</td><td>-1.5</td><td>4.14</td><td>51209.4</td><td>5</td><td>HUMAN</td><td>P31150</td><td>Rab GDP dissociation inhibitor alpha</td></tr>
<tr><td>3802</td><td>dirMS_041712_NAR_50mM_plus2.17304.17304.3</td><td>3</td><td>14.12</td><td>1</td><td>4.93E+05</td><td></td><td>(K)LLVYEAL</td><td>(K)L/L|V/Y E A L/E Y A K/R(A)</td><td></td><td>159.23</td><td>489.9441</td><td>1467.821</td><td>-0.0028</td><td>-1.9</td><td>6.14</td><td>32535.6</td><td>9.19</td><td>HUMAN</td><td>B4DZW6</td><td>Regulator of microtubule dynamics protein 1</td></tr>
<tr><td>3803</td><td>dirMS_041712_NAR_50mM_plus2.7421.7421.3</td><td>3</td><td>14.12</td><td>2</td><td>2.21E+05</td><td></td><td>(K)KAVDED</td><td>(K)K/A V/D E D/P/E N/Q T/L R K(D)</td><td></td><td>105.35</td><td>548.2865</td><td>1642.84</td><td>0.0055</td><td>3.3</td><td>4.79</td><td>87525.2</td><td>4.91</td><td>HUMAN</td><td>B1AKU4</td><td>Kinesin-associated protein 3</td></tr>
<tr><td>3804</td><td>dirMS_041712_NAR_60mM_plus.5631.5631.3</td><td>3</td><td>14.12</td><td>0</td><td>1.11E+06</td><td></td><td>(K)TATPQQ</td><td>(K)T A|T/P Q Q A Q/E/V/H E K(L)</td><td></td><td>87.6</td><td>489.5727</td><td>1466.723</td><td>-0.0198</td><td>-13.5</td><td>5.37</td><td>31075.7</td><td>5.65</td><td>HUMAN</td><td>P60174</td><td>Triosephosphate isomerase</td></tr>
<tr><td>3805</td><td>dirMS_041712_NAR_30mM_minus2.12659.12988.2</td><td>2</td><td>14.11</td><td>0</td><td>1.09E+06</td><td></td><td>(K)FSVSPV</td><td>(K)F/S|V/S|P\V/V/R(V)</td><td></td><td>123.77</td><td>445.759</td><td>890.509</td><td>0.0013</td><td>1.5</td><td>9.75</td><td>96306.4</td><td>6.41</td><td>HUMAN</td><td>P13639</td><td>Elongation factor 2</td></tr>
<tr><td>3806</td><td>dirMS_041712_NAR_30mM_minus2.14003.14187.2</td><td>2</td><td>14.11</td><td>0</td><td>4.29E+05</td><td></td><td>(R)VVFQEF</td><td>(R)V/V|F/Q/E|F/R(Q)</td><td></td><td>130.25</td><td>462.7452</td><td>924.494</td><td>-0.0107</td><td>-11.5</td><td>5.97</td><td>227783.5</td><td>5.5</td><td>HUMAN</td><td>P35579</td><td>Myosin-9</td></tr>
<tr><td>3807</td><td>dirMS_041712_NAR_50mM_minus2.17773.17773.3</td><td>3</td><td>14.11</td><td>1</td><td>1.01E+05</td><td></td><td>(K)ITQDIFQ</td><td>(K)I/T/Q/D/I/F/Q Q L L K/R(G)</td><td></td><td>169.85</td><td>501.6277</td><td>1502.869</td><td>-0.0004</td><td>-0.3</td><td>8.75</td><td>72796.9</td><td>6.75</td><td>HUMAN</td><td>A6NC17</td><td>Methionine--tRNA ligase, cytoplasmic</td></tr>
<tr><td>3808</td><td>dirMS_041712_NAR_120mM_plus.8323.8324.3</td><td>3</td><td>14.11</td><td>1</td><td>4.48E+05</td><td></td><td>(K)SSGGRE</td><td>(K)S/S G/G R E D\L|E|S/S/G L Q/R(R)</td><td></td><td>97.07</td><td>526.5882</td><td>1577.751</td><td>-0.0014</td><td>-0.9</td><td>4.68</td><td>11625.5</td><td>8.01</td><td>HUMAN</td><td>B8ZZT7</td><td>Hematological and neurological-expressed 1 protein</td></tr>
<tr><td>3809</td><td>dirMS_041712_NAR_120mM_plus.8610.8631.3</td><td>3</td><td>14.11</td><td>0</td><td>2.11E+06</td><td></td><td>(K)HTGPNS</td><td>(K)H T\G P N\S\P D T|A/N/D/G F/V/R(L)</td><td></td><td>98.97</td><td>562.26</td><td>1684.767</td><td>-0.0019</td><td>-1.2</td><td>5.21</td><td>18511.8</td><td>5.62</td><td>HUMAN</td><td>D6R9T0</td><td>Heterogeneous nuclear ribonucleoprotein H (Fragment)</td></tr>
<tr><td>3810</td><td>dirMS_041712_NAR_30mM_plus2.12482.12482.2</td><td>2</td><td>14.11</td><td>0</td><td>4.58E+05</td><td></td><td>(R)QGVDA</td><td>(R)Q G/V|D|A/D I|N G\L R(Q)</td><td></td><td>118.12</td><td>579.2997</td><td>1157.591</td><td>0.0012</td><td>1</td><td>4.21</td><td>39641.8</td><td>4.93</td><td>HUMAN</td><td>K7EQQ3</td><td>Keratin, type I cytoskeletal 9</td></tr>
</table>

<table>
<tr><td>3811</td><td>dirMS_041712_NAR_30mM_plus2.6340.6695.2</td><td>2</td><td>14.11</td><td>0</td><td>1.66E+06</td><td></td><td>(K)LLEGEES</td><td>(K)L/L|E|G/E/E/S/R(I)</td><td></td><td>88.35</td><td>466.7379</td><td>932.468</td><td>0.0002</td><td>0.2</td><td>4.25</td><td>49709.6</td><td>5.19</td><td>HUMAN</td><td>B0YJC4</td><td>Vimentin</td></tr>
<tr><td>3812</td><td>dirMS_041712_NAR_30mM_plus2.7569.7578.2</td><td>2</td><td>14.11</td><td>0</td><td>5.36E+05</td><td></td><td>(K)IANPVE</td><td>(K)I/A|N|P/V/E/G S S G R(Q)</td><td></td><td>93.97</td><td>543.7824</td><td>1086.554</td><td>0.0037</td><td>3.4</td><td>6</td><td>38010.7</td><td>6.67</td><td>HUMAN</td><td>Q15365</td><td>Poly(rC)-binding protein 1</td></tr>
<tr><td>3813</td><td>dirMS_041712_NAR_40mM_plus2.19385.19480.3</td><td>3</td><td>14.11</td><td>1</td><td>4.58E+05</td><td></td><td>(K)ALFEEV</td><td>(K)A L|F|E/E V/P E L L T E A E K K(E)</td><td></td><td>181.77</td><td>615.9991</td><td>1845.984</td><td>-0.0017</td><td>-0.9</td><td>4.32</td><td>65036</td><td>6.27</td><td>HUMAN</td><td>E9PBU3</td><td>Phosphoribosylaminoimidazolecarboxamide formyltransferase</td></tr>
<tr><td>3814</td><td>dirMS_041712_NAR_50mM_plus2.17743.17784.3</td><td>3</td><td>14.11</td><td>0</td><td>6.69E+05</td><td></td><td>(K)ALVSHN</td><td>(K)A L V S H N G S\L\I|N|V|G S/L/L Q/R(A)</td><td></td><td>161.37</td><td>626.6872</td><td>1878.056</td><td>-0.0085</td><td>-4.5</td><td>9.8</td><td>67732.1</td><td>4.71</td><td>HUMAN</td><td>G3V119</td><td>DBIRD complex subunit KIAA1967</td></tr>
<tr><td>3815</td><td>dirMS_041712_NAR_120mM_minus.7833.7865.3</td><td>3</td><td>14.1</td><td>1</td><td>5.78E+05</td><td></td><td>(R)LQSIGTE</td><td>(R)L/Q|S|I/G/T E/N T/E E N R R(F)</td><td></td><td>97.25</td><td>549.607</td><td>1646.809</td><td>-0.0028</td><td>-1.7</td><td>4.79</td><td>15633.5</td><td>9.15</td><td>HUMAN</td><td>H3BMQ8</td><td>Fructose-bisphosphate aldolase A (Fragment)</td></tr>
<tr><td>3816</td><td>dirMS_041712_NAR_20mM_minus.12285.12285.2</td><td>2</td><td>14.1</td><td>0</td><td>4.02E+05</td><td>M73m</td><td>(R)AVLVDL</td><td>(R)A V L|V D\L/E|P G T\m D S V/R(S)</td><td>m:Oxidized methionine</td><td>172.08</td><td>809.4088</td><td>1601.82</td><td>15.99</td><td>-3</td><td>4.03</td><td>13112.4</td><td>5.23</td><td>HUMAN</td><td>F8W813</td><td>Tubulin beta-8 chain</td></tr>
<tr><td>3817</td><td>dirMS_041712_NAR_30mM_minus2.17506.17506.2</td><td>2</td><td>14.1</td><td>0</td><td>4.67E+05</td><td></td><td>(K)LLCGLLA</td><td>(K)L L|C/G|L L/A E/R(L)</td><td>C:Carbamidomethylation</td><td>149.98</td><td>522.7963</td><td>1044.587</td><td>-0.0017</td><td>-1.6</td><td>6</td><td>12647.2</td><td>8.07</td><td>HUMAN</td><td>P14174</td><td>Macrophage migration inhibitory factor</td></tr>
<tr><td>3818</td><td>dirMS_041712_NAR_50mM_minus2.6103.6117.3</td><td>3</td><td>14.1</td><td>0</td><td>4.44E+05</td><td>M437m</td><td>(R)LESGMC</td><td>(R)L/E|S\G/M Q/N m S I/H/T K(T)</td><td>m:Oxidized methionine</td><td>97.77</td><td>497.9018</td><td>1475.698</td><td>15.9927</td><td>-1.5</td><td>6.75</td><td>56607.4</td><td>5.37</td><td>HUMAN</td><td>F8VXB4</td><td>Keratin, type II cytoskeletal 8</td></tr>
<tr><td>3819</td><td>dirMS_041712_NAR_60mM_minus.11918.11918.3</td><td>3</td><td>14.1</td><td>0</td><td>1.45E+05</td><td></td><td>(R)DAYSGG</td><td>(R)D A/Y/S/G/G/A V/N/L Y/H V R(E)</td><td></td><td>126.78</td><td>507.9181</td><td>1521.745</td><td>-0.0047</td><td>-3.1</td><td>6.74</td><td>17952.8</td><td>6.73</td><td>HUMAN</td><td>E9PAV2</td><td>Proteasome subunit beta type-5</td></tr>
<tr><td>3820</td><td>dirMS_041712_NAR_60mM_minus.9652.9652.2</td><td>2</td><td>14.1</td><td>1</td><td>4.41E+05</td><td></td><td>(R)LQAEIE</td><td>(R)L Q|A E/I/E/G L/K/G Q R(A)</td><td></td><td>114.13</td><td>671.3768</td><td>1341.748</td><td>-0.0022</td><td>-1.6</td><td>6.14</td><td>56607.4</td><td>5.37</td><td>HUMAN</td><td>F8VXB4</td><td>Keratin, type II cytoskeletal 8</td></tr>
<tr><td>3821</td><td>dirMS_041712_NAR_120mM_plus.14891.15228.2</td><td>2</td><td>14.1</td><td>0</td><td>3.44E+06</td><td></td><td>(R)DWSHY</td><td>(R)D/W/S/H/Y/F|K(I)</td><td></td><td>132.03</td><td>491.725</td><td>982.442</td><td>0.001</td><td>1</td><td>6.74</td><td>43773.3</td><td>5.26</td><td>HUMAN</td><td>F8VZY9</td><td>Keratin, type I cytoskeletal 18</td></tr>
<tr><td>3822</td><td>dirMS_041712_NAR_20mM_plus.19198.19198.2</td><td>2</td><td>14.1</td><td>0</td><td>1.33E+04</td><td></td><td>(K)EIIDLVL</td><td>(K)E I|I/D/L/V/L D R(I)</td><td></td><td>203.7</td><td>543.313</td><td>1085.62</td><td>-0.0014</td><td>-1.3</td><td>4.03</td><td>58642</td><td>4.95</td><td>HUMAN</td><td>F5H5D3</td><td>Tubulin alpha-1C chain</td></tr>
<tr><td>3823</td><td>dirMS_041712_NAR_20mM_plus.7617.7617.2</td><td>2</td><td>14.1</td><td>0</td><td>1.57E+05</td><td></td><td>(K)IAVAAQ</td><td>(K)I A/V|A/A|Q N/C/Y K(V)</td><td>C:Carbamidomethylation</td><td>122.43</td><td>569.2902</td><td>1137.572</td><td>0.001</td><td>0.9</td><td>8.59</td><td>31075.7</td><td>5.65</td><td>HUMAN</td><td>P60174</td><td>Triosephosphate isomerase</td></tr>
<tr><td>3824</td><td>dirMS_041712_NAR_30mM_plus2.18718.18718.2</td><td>2</td><td>14.1</td><td>0</td><td>3.13E+05</td><td></td><td>(R)ESLEALL</td><td>(R)E S|L/E/A L/L/Q/R(A)</td><td></td><td>152</td><td>529.8026</td><td>1058.584</td><td>0.0139</td><td>13.1</td><td>4.53</td><td>107721.8</td><td>8.85</td><td>HUMAN</td><td>O94906</td><td>Pre-mRNA-processing factor 6</td></tr>
<tr><td>3825</td><td>dirMS_041712_NAR_30mM_plus2.19789.19789.2</td><td>2</td><td>14.1</td><td>0</td><td>1.30E+05</td><td></td><td>(R)NTWLY</td><td>(R)N T/W/L/Y Q A L/R(E)</td><td></td><td>157.12</td><td>582.8112</td><td>1164.616</td><td>-0.0009</td><td>-0.8</td><td>8.75</td><td>49457.5</td><td>6.47</td><td>HUMAN</td><td>P56545</td><td>C-terminal-binding protein 2</td></tr>
<tr><td>3826</td><td>dirMS_041712_NAR_50mM_plus2.4972.4980.3</td><td>3</td><td>14.1</td><td>1</td><td>1.04E+05</td><td></td><td>(K)VSSAEG</td><td>(K)V/S|S A E/G A/A K/E/E/P K(R)</td><td></td><td>83.1</td><td>434.8891</td><td>1302.654</td><td>-0.0008</td><td>-0.6</td><td>4.79</td><td>4801.6</td><td>11.17</td><td>HUMAN</td><td>F2Z2W6</td><td>Non-histone chromosomal protein HMG-14</td></tr>
<tr><td>3827</td><td>dirMS_041712_NAR_60mM_plus.20414.20414.3</td><td>3</td><td>14.1</td><td>1</td><td>4.21E+05</td><td></td><td>(K)NAAGAL</td><td>(K)N A|A\G A/L/D L L/K/E/L K(N)</td><td></td><td>166.93</td><td>452.6004</td><td>1355.789</td><td>-0.0026</td><td>-2</td><td>6.07</td><td>13786.8</td><td>9.33</td><td>HUMAN</td><td>B7Z4S1</td><td>Transcription elongation factor A protein 1</td></tr>
<tr><td>3828</td><td>dirMS_041712_NAR_120mM_minus.9814.9840.3</td><td>3</td><td>14.09</td><td>1</td><td>2.01E+05</td><td></td><td>(R)SRSGEG</td><td>(R)S R S G E\G E|V|S/G L/M/R(K)</td><td></td><td>108.07</td><td>455.559</td><td>1364.659</td><td>0.0038</td><td>2.8</td><td>5.87</td><td>90316.8</td><td>5.52</td><td>HUMAN</td><td>Q13263</td><td>Transcription intermediary factor 1-beta</td></tr>
<tr><td>3829</td><td>dirMS_041712_NAR_20mM_minus.14639.14639.2</td><td>2</td><td>14.09</td><td>0</td><td>2.03E+05</td><td></td><td>(K)TAVETA</td><td>(K)T/A\V E/T/A/V/L|L\L R(I)</td><td></td><td>201.18</td><td>593.3613</td><td>1185.72</td><td>-0.0048</td><td>-4.1</td><td>5.66</td><td>56186.7</td><td>5.47</td><td>HUMAN</td><td>B4DUR8</td><td>T-complex protein 1 subunit gamma</td></tr>
<tr><td>3830</td><td>dirMS_041712_NAR_30mM_minus2.10764.10764.2</td><td>2</td><td>14.09</td><td>0</td><td>6.30E+05</td><td></td><td>(K)GFSLESC</td><td>(K)G F/S/L/E/S C/R(S)</td><td>C:Carbamidomethylation</td><td>112.08</td><td>478.2237</td><td>955.43</td><td>0.0099</td><td>10.4</td><td>6</td><td>82516.1</td><td>5.49</td><td>HUMAN</td><td>P07384</td><td>Calpain-1 catalytic subunit</td></tr>
<tr><td>3831</td><td>dirMS_041712_NAR_30mM_minus2.18366.18366.2</td><td>2</td><td>14.09</td><td>0</td><td>2.28E+05</td><td></td><td>(K)YPQLLP</td><td>(K)Y/P/Q/L/L P/G I R(G)</td><td></td><td>154.92</td><td>528.816</td><td>1056.62</td><td>0.0047</td><td>4.4</td><td>8.75</td><td>45228.3</td><td>6.03</td><td>HUMAN</td><td>F5H737</td><td>Adenosylhomocysteinase</td></tr>
<tr><td>3832</td><td>dirMS_041712_NAR_120mM_plus.19375.19453.3</td><td>3</td><td>14.09</td><td>1</td><td>6.90E+05</td><td></td><td>(K)YNQLLR</td><td>(K)Y/N|Q/L/L/R I E E E/L G S/K(A)</td><td></td><td>155.23</td><td>564.6353</td><td>1691.896</td><td>-0.0049</td><td>-2.9</td><td>4.79</td><td>47510.4</td><td>7.04</td><td>HUMAN</td><td>P06733</td><td>Alpha-enolase</td></tr>
<tr><td>3833</td><td>dirMS_041712_NAR_120mM_plus.7391.7477.3</td><td>3</td><td>14.09</td><td>1</td><td>1.14E+06</td><td></td><td>(R)EKVPGG</td><td>(R)E K V|P G G\L|Q/G/S/Q D/R(G)</td><td></td><td>92.32</td><td>457.5737</td><td>1370.702</td><td>0.0043</td><td>3.1</td><td>6.17</td><td>180453.7</td><td>6.04</td><td>HUMAN</td><td>H0YIQ2</td><td>YLP motif-containing protein 1 (Fragment)</td></tr>
<tr><td>3834</td><td>dirMS_041712_NAR_20mM_plus.13727.13727.2</td><td>2</td><td>14.09</td><td>0</td><td>1.07E+05</td><td></td><td>(R)LVVPAT</td><td>(R)L V|V|P A T Q/C G S/L\I G K(G)</td><td>C:Carbamidomethylation</td><td>166.98</td><td>721.9033</td><td>1442.804</td><td>-0.0042</td><td>-2.9</td><td>8.75</td><td>38010.7</td><td>6.67</td><td>HUMAN</td><td>Q15365</td><td>Poly(rC)-binding protein 1</td></tr>
<tr><td>3835</td><td>dirMS_041712_NAR_20mM_plus.16243.16243.3</td><td>3</td><td>14.09</td><td>0</td><td>1.14E+06</td><td>M221m</td><td>(R)ALTVPEL</td><td>(R)A L\T\V|P E L/T/Q/Q m/F D/A K(N)</td><td>m:Oxidized methionine</td><td>184.33</td><td>569.9583</td><td>1691.867</td><td>15.9931</td><td>-1.1</td><td>4.37</td><td>42831.5</td><td>4.86</td><td>HUMAN</td><td>A8K854</td><td>HCG1983504, isoform CRA_f</td></tr>
<tr><td>3836</td><td>dirMS_041712_NAR_20mM_plus.16676.16676.3</td><td>3</td><td>14.09</td><td>0</td><td>2.39E+05</td><td></td><td>(K)VQIAVA</td><td>(K)V Q\I A V A N/A|Q/E/L/L/Q R(M)</td><td></td><td>186.48</td><td>518.2975</td><td>1552.881</td><td>-0.0026</td><td>-1.7</td><td>5.97</td><td>10728.1</td><td>9.15</td><td>HUMAN</td><td>Q9Y5L4</td><td>Mitochondrial import inner membrane translocase subunit Tim13</td></tr>
<tr><td>3837</td><td>dirMS_041712_NAR_20mM_plus.16679.16679.2</td><td>2</td><td>14.09</td><td>0</td><td>1.48E+05</td><td></td><td>(R)LLEAQIA</td><td>(R)L L/E\A/Q I\A/T/G\G I I D|P K(E)</td><td></td><td>185.75</td><td>769.9362</td><td>1538.879</td><td>-0.0137</td><td>-8.9</td><td>4.37</td><td>334221.3</td><td>6.44</td><td>HUMAN</td><td>P15924</td><td>Desmoplakin</td></tr>
<tr><td>3838</td><td>dirMS_041712_NAR_20mM_plus.17812.17812.2</td><td>2</td><td>14.09</td><td>0</td><td>1.67E+05</td><td></td><td>(K)GADFLV</td><td>(K)G A D|F\L|V/T/E V/E/N G G S L G S K(K)</td><td></td><td>195</td><td>890.4414</td><td>1779.876</td><td>-0.0004</td><td>-0.2</td><td>4.14</td><td>30947.9</td><td>6.47</td><td>HUMAN</td><td>H3BQ34</td><td>Pyruvate kinase</td></tr>
<tr><td>3839</td><td>dirMS_041712_NAR_30mM_plus2.16760.16813.2</td><td>2</td><td>14.09</td><td>0</td><td>3.06E+05</td><td></td><td>(R)SIQADG</td><td>(R)S I|Q|A|D|G L/V/W/G S S K(L)</td><td></td><td>140.72</td><td>674.3475</td><td>1347.69</td><td>-0.0026</td><td>-1.9</td><td>5.55</td><td>24934.4</td><td>4.5</td><td>HUMAN</td><td>P24534</td><td>Elongation factor 1-beta</td></tr>
<tr><td>3840</td><td>dirMS_041712_NAR_40mM_plus2.6980.7125.2</td><td>2</td><td>14.09</td><td>0</td><td>2.31E+05</td><td></td><td>(K)NMMAA</td><td>(K)N/M|M|A|A|C D|P/R(H)</td><td>C:Carbamidomethylation</td><td>101.42</td><td>533.2182</td><td>1065.427</td><td>0.0017</td><td>1.6</td><td>5.84</td><td>50199.4</td><td>4.75</td><td>HUMAN</td><td>A6NNZ2</td><td>Tubulin beta-8 chain-like protein LOC260334</td></tr>
<tr><td>3841</td><td>dirMS_041712_NAR_50mM_plus2.10892.10892.3</td><td>3</td><td>14.09</td><td>0</td><td>4.81E+05</td><td></td><td>(R)EALLSSA</td><td>(R)E A/L/L/S/S A|V D H G S D E V/K(F)</td><td></td><td>124.73</td><td>552.9403</td><td>1656.807</td><td>-0.0012</td><td>-0.7</td><td>4.31</td><td>57204.9</td><td>6.01</td><td>HUMAN</td><td>F5GWF6</td><td>T-complex protein 1 subunit beta</td></tr>
<tr><td>3842</td><td>dirMS_041712_NAR_50mM_plus2.15407.15407.3</td><td>3</td><td>14.09</td><td>1</td><td>5.45E+05</td><td></td><td>(R)GDVVPK</td><td>(R)G D V|V|P K D V\N\A A/I A T/I K(T)</td><td></td><td>148.45</td><td>537.6393</td><td>1610.911</td><td>-0.0079</td><td>-4.9</td><td>5.96</td><td>58642</td><td>4.95</td><td>HUMAN</td><td>F5H5D3</td><td>Tubulin alpha-1C chain</td></tr>
<tr><td>3843</td><td>dirMS_041712_NAR_50mM_plus2.17509.17510.3</td><td>3</td><td>14.09</td><td>1</td><td>5.25E+05</td><td></td><td>(K)VKLAAV</td><td>(K)V K L A\A\V D A T\V|N/Q|V|L/A/S R(Y)</td><td></td><td>160.33</td><td>585.6736</td><td>1755.012</td><td>-0.0061</td><td>-3.5</td><td>8.72</td><td>53659.5</td><td>5.35</td><td>HUMAN</td><td>B5MCQ5</td><td>Protein disulfide-isomerase A6</td></tr>
<tr><td>3844</td><td>dirMS_041712_NAR_120mM_minus.9684.9684.3</td><td>3</td><td>14.08</td><td>0</td><td>4.38E+05</td><td></td><td>(K)QEYDES</td><td>(K)Q E|Y/D E S/G/P S/I/V/H R(K)</td><td></td><td>107.47</td><td>506.2424</td><td>1516.703</td><td>0.01</td><td>6.6</td><td>4.65</td><td>123097.1</td><td>5.83</td><td>HUMAN</td><td>A5A3E0</td><td>POTE ankyrin domain family member F</td></tr>
<tr><td>3845</td><td>dirMS_041712_NAR_30mM_minus2.11630.11700.2</td><td>2</td><td>14.08</td><td>0</td><td>2.14E+05</td><td></td><td>(R)GYFSEE</td><td>(R)G Y|F|S/E E/M N/R(V)</td><td></td><td>117.05</td><td>566.7446</td><td>1132.473</td><td>0.0091</td><td>8.1</td><td>4.53</td><td>533778</td><td>5.74</td><td>HUMAN</td><td>Q15149</td><td>Plectin</td></tr>
<tr><td>3846</td><td>dirMS_041712_NAR_60mM_minus.9383.9402.3</td><td>3</td><td>14.08</td><td>1</td><td>2.76E+05</td><td>M198m</td><td>(R)VFDKEG</td><td>(R)V/F|D K E G N G/T|V/m/G A E/I R(H)</td><td>m:Oxidized methionine</td><td>112.15</td><td>580.2853</td><td>1722.848</td><td>15.9934</td><td>-0.9</td><td>4.68</td><td>26991.7</td><td>5.01</td><td>HUMAN</td><td>B7Z6Z4</td><td>Myosin light polypeptide 6</td></tr>
<tr><td>3847</td><td>dirMS_041712_NAR_20mM_plus.15487.15487.2</td><td>2</td><td>14.08</td><td>0</td><td>2.31E+05</td><td></td><td>(K)TITLEVE</td><td>(K)T I/T|L/E/V|E|P S D T I E N V K(A)</td><td></td><td>179.02</td><td>894.4658</td><td>1787.927</td><td>-0.003</td><td>-1.7</td><td>4</td><td>17271.6</td><td>6.75</td><td>HUMAN</td><td>B4DV12</td><td>Ubiquitin</td></tr>
<tr><td>3848</td><td>dirMS_041712_NAR_20mM_plus.18972.18972.2</td><td>2</td><td>14.08</td><td>0</td><td>1.91E+05</td><td></td><td>(R)STGEAF</td><td>(R)S T G E A F|V|Q/F/A S Q E/I/A E|K(A)</td><td></td><td>202.2</td><td>921.4477</td><td>1841.892</td><td>-0.0035</td><td>-1.9</td><td>4.25</td><td>24249.1</td><td>6.99</td><td>HUMAN</td><td>D6RBM0</td><td>Heterogeneous nuclear ribonucleoprotein H (Fragment)</td></tr>
<tr><td>3849</td><td>dirMS_041712_NAR_20mM_plus.19135.19135.3</td><td>3</td><td>14.08</td><td>0</td><td>1.98E+05</td><td></td><td>(R)LAGTQP</td><td>(R)L A G T Q/P L\E\V|L/E/A V/Q/R(S)</td><td></td><td>204</td><td>541.9731</td><td>1623.906</td><td>-0.0017</td><td>-1</td><td>4.53</td><td>118930.9</td><td>5.49</td><td>HUMAN</td><td>P22314</td><td>Ubiquitin-like modifier-activating enzyme 1</td></tr>
<tr><td>3850</td><td>dirMS_041712_NAR_40mM_plus2.20112.20112.3</td><td>3</td><td>14.08</td><td>1</td><td>5.30E+05</td><td></td><td>(K)EGGLGP</td><td>(K)E G G\L/G/P/L/N/I/P L\L A D/V T\R R(L)</td><td></td><td>189.92</td><td>631.0288</td><td>1891.076</td><td>-0.0041</td><td>-2.2</td><td>6.17</td><td>15252.2</td><td>5.82</td><td>HUMAN</td><td>A6NIW5</td><td>Peroxiredoxin 2, isoform CRA_a</td></tr>
<tr><td>3851</td><td>dirMS_041712_NAR_50mM_plus2.17114.17343.3</td><td>3</td><td>14.08</td><td>1</td><td>1.19E+06</td><td></td><td>(K)DNNGVI</td><td>(K)D N|N/G/V/I/G L/L E/P M K K(S)</td><td></td><td>158.77</td><td>509.9448</td><td>1527.82</td><td>-0.0001</td><td>-0.1</td><td>6.07</td><td>29854.8</td><td>9.49</td><td>HUMAN</td><td>C9J3R0</td><td>Regulator of chromosome condensation (Fragment)</td></tr>
<tr><td>3852</td><td>dirMS_041712_NAR_60mM_plus.17636.17681.3</td><td>3</td><td>14.08</td><td>1</td><td>2.35E+05</td><td></td><td>(R)KALDIAE</td><td>(R)K A L D\I A\E\N|E|M/P/G L M R(M)</td><td></td><td>149.22</td><td>563.2887</td><td>1687.851</td><td>0.001</td><td>0.6</td><td>4.68</td><td>48285.8</td><td>5.93</td><td>HUMAN</td><td>P23526</td><td>Adenosylhomocysteinase</td></tr>
<tr><td>3853</td><td>dirMS_041712_NAR_60mM_plus.9187.9187.3</td><td>3</td><td>14.08</td><td>1</td><td>2.08E+05</td><td></td><td>(K)YYDDTY</td><td>(K)Y/Y/D/D T/Y/P/S V K E/Q/K(A)</td><td></td><td>105.92</td><td>545.9214</td><td>1635.754</td><td>-0.004</td><td>-2.5</td><td>4.56</td><td>18430.9</td><td>4.79</td><td>HUMAN</td><td>F8VS17</td><td>Coatomer subunit zeta-1</td></tr>
<tr><td>3854</td><td>dirMS_041712_NAR_20mM_minus.10729.10729.2</td><td>2</td><td>14.07</td><td>0</td><td>1.89E+05</td><td></td><td>(R)TTPSYV</td><td>(R)T T/P S Y/V|A F/T/D/T E R(L)</td><td></td><td>154.68</td><td>744.3547</td><td>1487.701</td><td>0.0009</td><td>0.6</td><td>4.37</td><td>58502.6</td><td>6.09</td><td>HUMAN</td><td>B4DXY3</td><td>Heat shock 70 kDa protein 1-like</td></tr>
<tr><td>3855</td><td>dirMS_041712_NAR_60mM_minus.12033.12113.3</td><td>3</td><td>14.07</td><td>1</td><td>2.54E+03</td><td></td><td>(R)VIDRELY</td><td>(R)V/I/D/R E\L\Y|Q/Q/L Q/R(G)</td><td></td><td>128.12</td><td>520.9529</td><td>1560.849</td><td>-0.0051</td><td>-3.3</td><td>6.04</td><td>533778</td><td>5.74</td><td>HUMAN</td><td>Q15149</td><td>Plectin</td></tr>
<tr><td>3856</td><td>dirMS_041712_NAR_20mM_plus.13955.13956.2</td><td>2</td><td>14.07</td><td>0</td><td>1.34E+05</td><td></td><td>(R)GGIVDE</td><td>(R)G G/I|V|D/E\G A/L L/R(A)</td><td></td><td>167.83</td><td>550.3066</td><td>1099.611</td><td>-0.0047</td><td>-4.3</td><td>4.37</td><td>57391.2</td><td>6.29</td><td>HUMAN</td><td>O43175</td><td>D-3-phosphoglycerate dehydrogenase</td></tr>
<tr><td>3857</td><td>dirMS_041712_NAR_20mM_plus.15158.15158.2</td><td>2</td><td>14.07</td><td>0</td><td>5.88E+04</td><td></td><td>(R)EQLAIAE</td><td>(R)E Q|L/A/I/A/E/F A R(S)</td><td></td><td>176.1</td><td>574.312</td><td>1147.611</td><td>0.0061</td><td>5.3</td><td>4.53</td><td>44227.3</td><td>7.81</td><td>HUMAN</td><td>E7EQR6</td><td>T-complex protein 1 subunit alpha</td></tr>
<tr><td>3858</td><td>dirMS_041712_NAR_50mM_plus2.15927.15927.3</td><td>3</td><td>14.07</td><td>1</td><td>9.43E+04</td><td></td><td>(K)FAANINI</td><td>(K)F A|A N I/N K E S I\V/D/V/E/G V V R(K)</td><td></td><td>151.15</td><td>654.0215</td><td>1960.05</td><td>0.0001</td><td>0.1</td><td>4.68</td><td>20139.7</td><td>9.34</td><td>HUMAN</td><td>C9J7S3</td><td>Aspartate--tRNA ligase, cytoplasmic (Fragment)</td></tr>
<tr><td>3859</td><td>dirMS_041712_NAR_50mM_plus2.18207.18478.3</td><td>3</td><td>14.07</td><td>0</td><td>1.25E+06</td><td></td><td>(R)DFLLKPE</td><td>(R)D F|L/L/K/P/E L L R(A)</td><td></td><td>164.65</td><td>415.2519</td><td>1243.741</td><td>0.0003</td><td>0.2</td><td>6.07</td><td>30944.2</td><td>6.52</td><td>HUMAN</td><td>B1Q2N1</td><td>ATP-dependent RNA helicase DDX39A</td></tr>
<tr><td>3860</td><td>dirMS_041712_NAR_50mM_plus2.22256.22256.3</td><td>3</td><td>14.07</td><td>1</td><td>1.35E+06</td><td></td><td>(K)EGGLGP</td><td>(K)E G G L/G/P/L N/I/P L L A D V T R R(L)</td><td></td><td>191</td><td>631.029</td><td>1891.076</td><td>-0.0035</td><td>-1.9</td><td>6.17</td><td>15252.2</td><td>5.82</td><td>HUMAN</td><td>A6NIW5</td><td>Peroxiredoxin 2, isoform CRA_a</td></tr>
<tr><td>3861</td><td>dirMS_041712_NAR_50mM_plus2.5995.5995.3</td><td>3</td><td>14.07</td><td>1</td><td>2.40E+04</td><td></td><td>(R)GTDDSP</td><td>(R)G T/D/D/S/P K D S Q/E/D\L/Q E R(H)</td><td></td><td>95.62</td><td>607.2766</td><td>1819.794</td><td>0.0212</td><td>11.6</td><td>3.96</td><td>23420.8</td><td>4.74</td><td>HUMAN</td><td>Q5H9L2</td><td>Transcription elongation factor A protein-like 5</td></tr>
<tr><td>3862</td><td>dirMS_041712_NAR_120mM_minus.11994.11994.3</td><td>3</td><td>14.06</td><td>0</td><td>3.85E+05</td><td></td><td>(R)HSSLAG</td><td>(R)H S\S L A G\C|Q/I|I/N Y R(T)</td><td>C:Carbamidomethylation</td><td>119.48</td><td>506.9192</td><td>1518.748</td><td>-0.0051</td><td>-3.4</td><td>8.75</td><td>193379.8</td><td>5.48</td><td>HUMAN</td><td>Q00610</td><td>Clathrin heavy chain 1</td></tr>
<tr><td>3863</td><td>dirMS_041712_NAR_20mM_minus.11547.11547.2</td><td>2</td><td>14.06</td><td>0</td><td>1.40E+05</td><td>M98m</td><td>(R)TLTIVDT</td><td>(R)T L|T I/V/D T G I/G m T/K(A)</td><td>m:Oxidized methionine</td><td>163.17</td><td>683.3669</td><td>1349.735</td><td>15.992</td><td>-2.1</td><td>5.5</td><td>20151.2</td><td>4.58</td><td>HUMAN</td><td>G3V2J8</td><td>Heat shock protein HSP 90-alpha (Fragment)</td></tr>
<tr><td>3864</td><td>dirMS_041712_NAR_20mM_minus.12193.12193.2</td><td>2</td><td>14.06</td><td>0</td><td>1.08E+06</td><td>M313m</td><td>(R)TVQSLEI</td><td>(R)T V|Q|S L/E/I D L D S m R(N)</td><td>m:Oxidized methionine</td><td>170.78</td><td>761.8733</td><td>1506.747</td><td>15.9925</td><td>-1.6</td><td>4.03</td><td>43773.3</td><td>5.26</td><td>HUMAN</td><td>F8VZY9</td><td>Keratin, type I cytoskeletal 18</td></tr>
<tr><td>3865</td><td>dirMS_041712_NAR_50mM_minus2.13110.13110.3</td><td>3</td><td>14.06</td><td>0</td><td>3.56E+05</td><td></td><td>(R)ETNLDSI</td><td>(R)E T N L/D|S L/P/L V/D T/H S K(R)</td><td></td><td>142.45</td><td>556.9529</td><td>1668.844</td><td>0.0002</td><td>0.1</td><td>4.53</td><td>53707.8</td><td>5.06</td><td>HUMAN</td><td>P08670</td><td>Vimentin</td></tr>
<tr><td>3866</td><td>dirMS_041712_NAR_60mM_minus.4663.4663.3</td><td>3</td><td>14.06</td><td>1</td><td>6.72E+04</td><td></td><td>(R)AAEEED</td><td>(R)A/A/E/E E D/E/A D/P K R(Q)</td><td></td><td>77.55</td><td>453.872</td><td>1359.602</td><td>-0.0008</td><td>-0.6</td><td>4.08</td><td>11529.7</td><td>4.14</td><td>HUMAN</td><td>P20962</td><td>Parathymosin</td></tr>
<tr><td>3867</td><td>dirMS_041712_NAR_60mM_minus.9934.9934.3</td><td>3</td><td>14.06</td><td>1</td><td>5.63E+05</td><td>M230m M</td><td>(R)mSmKE</td><td>(R)m S\m K E V\D E/Q|M/L/N|V Q N K(N)</td><td>m:Oxidized methionine</td><td>114.97</td><td>652.6318</td><td>1923.897</td><td>31.9836</td><td>-3.2</td><td>4.68</td><td>40069.4</td><td>4.83</td><td>HUMAN</td><td>E7EWR1</td><td>Tubulin beta chain</td></tr>
<tr><td>3868</td><td>dirMS_041712_NAR_120mM_plus.10062.10245.3</td><td>3</td><td>14.06</td><td>1</td><td>1.30E+07</td><td></td><td>(R)VKYETEL</td><td>(R)V/K Y\E T/E|L|A/M/R(Q)</td><td></td><td>107.35</td><td>413.8849</td><td>1239.64</td><td>0</td><td>0</td><td>6.11</td><td>43773.3</td><td>5.26</td><td>HUMAN</td><td>F8VZY9</td><td>Keratin, type I cytoskeletal 18</td></tr>
<tr><td>3869</td><td>dirMS_041712_NAR_120mM_plus.11130.11274.3</td><td>3</td><td>14.06</td><td>0</td><td>1.25E+06</td><td></td><td>(R)TICSHV</td><td>(R)T/I|C/S/H V Q N M I/K(G)</td><td>C:Carbamidomethylation</td><td>112.02</td><td>444.2263</td><td>1330.661</td><td>0.0037</td><td>2.8</td><td>8.44</td><td>20931.1</td><td>10.28</td><td>HUMAN</td><td>D6RAN4</td><td>60S ribosomal protein L9 (Fragment)</td></tr>
<tr><td>3870</td><td>dirMS_041712_NAR_20mM_plus.12322.12547.2</td><td>2</td><td>14.06</td><td>0</td><td>1.78E+05</td><td></td><td>(K)TIIFVET</td><td>(K)T I|I/F V E/T/K(R)</td><td></td><td>158.87</td><td>475.7818</td><td>950.556</td><td>0.0006</td><td>0.6</td><td>5.66</td><td>73202</td><td>9.19</td><td>HUMAN</td><td>C9JMU5</td><td>Probable ATP-dependent RNA helicase DDX17</td></tr>
<tr><td>3871</td><td>dirMS_041712_NAR_20mM_plus.13686.13686.2</td><td>2</td><td>14.06</td><td>0</td><td>3.11E+05</td><td></td><td>(R)VLQALE</td><td>(R)V L|Q/A/L E/G L\K(M)</td><td></td><td>165.42</td><td>485.8004</td><td>970.593</td><td>0.0004</td><td>0.4</td><td>5.97</td><td>16060.2</td><td>10.31</td><td>HUMAN</td><td>P39019</td><td>40S ribosomal protein S19</td></tr>
<tr><td>3872</td><td>dirMS_041712_NAR_20mM_plus.18678.18678.2</td><td>2</td><td>14.06</td><td>0</td><td>1.61E+05</td><td></td><td>(R)IITLTGP</td><td>(R)I I|T/L/T/G/P T N A I F K(A)</td><td></td><td>200.33</td><td>694.9091</td><td>1388.815</td><td>-0.0039</td><td>-2.8</td><td>8.75</td><td>38010.7</td><td>6.67</td><td>HUMAN</td><td>Q15365</td><td>Poly(rC)-binding protein 1</td></tr>
<tr><td>3873</td><td>dirMS_041712_NAR_30mM_plus2.10525.10541.2</td><td>2</td><td>14.06</td><td>0</td><td>9.14E+05</td><td></td><td>(R)LTVEEA</td><td>(R)L/T|V/E|E/A/V R(H)</td><td></td><td>109.33</td><td>458.7641</td><td>916.51</td><td>0.0111</td><td>12.1</td><td>4.53</td><td>862169.3</td><td>5.27</td><td>HUMAN</td><td>H3BPE1</td><td>Microtubule-actin cross-linking factor 1, isoforms 1/2/3/5</td></tr>
<tr><td>3874</td><td>dirMS_041712_NAR_30mM_plus2.13759.14035.2</td><td>2</td><td>14.06</td><td>0</td><td>1.84E+06</td><td></td><td>(R)LILIESR(I</td><td>(R)L/I|L|I/E/S/R(I)</td><td></td><td>125.87</td><td>422.2682</td><td>843.53</td><td>-0.0007</td><td>-0.8</td><td>6</td><td>17222</td><td>10.53</td><td>HUMAN</td><td>P62277</td><td>40S ribosomal protein S13</td></tr>
<tr><td>3875</td><td>dirMS_041712_NAR_30mM_plus2.14586.14586.2</td><td>2</td><td>14.06</td><td>0</td><td>2.53E+05</td><td></td><td>(R)SIGEYD</td><td>(R)S I|G E/Y D|V L R(G)</td><td></td><td>129.85</td><td>526.2726</td><td>1051.542</td><td>-0.0039</td><td>-3.7</td><td>4.37</td><td>470342.8</td><td>6.83</td><td>HUMAN</td><td>E7EUY0</td><td>DNA-dependent protein kinase catalytic subunit</td></tr>
<tr><td>3876</td><td>dirMS_041712_NAR_30mM_plus2.19949.20074.2</td><td>2</td><td>14.06</td><td>0</td><td>2.47E+05</td><td></td><td>(K)LALFQAI</td><td>(K)L/A|L|F/Q A I/G/K(E)</td><td></td><td>158.53</td><td>480.795</td><td>960.588</td><td>-0.005</td><td>-5.2</td><td>8.75</td><td>266161</td><td>5.68</td><td>HUMAN</td><td>E9PPU0</td><td>Epiplakin</td></tr>
<tr><td>3877</td><td>dirMS_041712_NAR_30mM_plus2.21924.21962.2</td><td>2</td><td>14.06</td><td>0</td><td>1.55E+05</td><td></td><td>(K)ANFLQN</td><td>(K)A N|F|L|Q|N M/I E D/R(T)</td><td></td><td>170.25</td><td>675.8282</td><td>1350.647</td><td>0.0021</td><td>1.5</td><td>4.37</td><td>120546</td><td>7.82</td><td>HUMAN</td><td>O95486</td><td>Protein transport protein Sec24A</td></tr>
<tr><td>3878</td><td>dirMS_041712_NAR_30mM_plus2.8127.8127.2</td><td>2</td><td>14.06</td><td>0</td><td>1.36E+06</td><td></td><td>(K)QGGGG</td><td>(K)Q G\G/G G/G G G S/V|P G I E/R(M)</td><td></td><td>97.17</td><td>642.8178</td><td>1284.629</td><td>-0.0008</td><td>-0.6</td><td>6</td><td>77799.7</td><td>9.09</td><td>HUMAN</td><td>P52272</td><td>Heterogeneous nuclear ribonucleoprotein M</td></tr>
<tr><td>3879</td><td>dirMS_041712_NAR_50mM_plus2.17478.17478.3</td><td>3</td><td>14.06</td><td>0</td><td>6.20E+05</td><td></td><td>(R)IMEVID</td><td>(R)I/M|E|V/I/D A I/T T T A Q S H Q R(T)</td><td></td><td>160.32</td><td>638.6636</td><td>1913.975</td><td>0.0013</td><td>0.7</td><td>5.32</td><td>85815.7</td><td>7.35</td><td>HUMAN</td><td>P17858</td><td>6-phosphofructokinase, liver type</td></tr>
<tr><td>3880</td><td>dirMS_041712_NAR_50mM_plus2.19887.19887.3</td><td>3</td><td>14.06</td><td>0</td><td>2.50E+05</td><td></td><td>(K)LFLFHTS</td><td>(K)L F|L F H T S\L/P I/A/E/A/P/G K(L)</td><td></td><td>172.83</td><td>580.9943</td><td>1740.968</td><td>0</td><td>0</td><td>6.75</td><td>108105.4</td><td>6.57</td><td>HUMAN</td><td>E7EP00</td><td>Protein transport protein Sec24C</td></tr>
<tr><td>3881</td><td>dirMS_041712_NAR_30mM_minus2.11589.11589.2</td><td>2</td><td>14.05</td><td>0</td><td>5.18E+05</td><td></td><td>(R)DVCLYPI</td><td>(R)D V/C|L Y/P/R(F)</td><td>C:Carbamidomethylation</td><td>117.17</td><td>461.7315</td><td>922.445</td><td>0.0106</td><td>11.5</td><td>5.83</td><td>35548.6</td><td>4.91</td><td>HUMAN</td><td>B4DN60</td><td>Asparagine--tRNA ligase, cytoplasmic</td></tr>
<tr><td>3882</td><td>dirMS_041712_NAR_50mM_minus2.12506.12506.3</td><td>3</td><td>14.05</td><td>1</td><td>1.67E+05</td><td></td><td>(K)IINADSE</td><td>(K)I/I/N\A/D S E D/P K Y I I N V K(Q)</td><td></td><td>139.08</td><td>611.3243</td><td>1831.98</td><td>-0.0217</td><td>-11.8</td><td>4.56</td><td>14693.3</td><td>4.69</td><td>HUMAN</td><td>C9JLS9</td><td>26S protease regulatory subunit 7 (Fragment)</td></tr>
<tr><td>3883</td><td>dirMS_041712_NAR_20mM_plus.14714.14714.2</td><td>2</td><td>14.05</td><td>0</td><td>1.98E+05</td><td>M179m M</td><td>(K)mFVLDE</td><td>(K)m F|V|L/D E A/D/E m L|S R(G)</td><td>m:Oxidized methionine</td><td>173.6</td><td>794.3555</td><td>1555.713</td><td>31.9906</td><td>0.5</td><td>3.91</td><td>41517.9</td><td>5.45</td><td>HUMAN</td><td>E7EQG2</td><td>Eukaryotic initiation factor 4A-II</td></tr>
<tr><td>3884</td><td>dirMS_041712_NAR_20mM_plus.16566.16566.2</td><td>2</td><td>14.05</td><td>0</td><td>1.38E+05</td><td></td><td>(K)TVDNFV</td><td>(K)T V|D N F|V/A L/A/T G E K(G)</td><td></td><td>185.88</td><td>682.8565</td><td>1364.706</td><td>0.0001</td><td>0.1</td><td>4.37</td><td>23799.1</td><td>9.48</td><td>HUMAN</td><td>P23284</td><td>Peptidyl-prolyl cis-trans isomerase B</td></tr>
<tr><td>3885</td><td>dirMS_041712_NAR_30mM_plus2.13045.13105.2</td><td>2</td><td>14.05</td><td>0</td><td>2.47E+05</td><td></td><td>(K)IGGIGTV</td><td>(K)I/G|G\I|G/T/V|P V G R(V)</td><td></td><td>121.77</td><td>513.3084</td><td>1025.61</td><td>-0.0007</td><td>-0.7</td><td>9.75</td><td>50482.3</td><td>9.31</td><td>HUMAN</td><td>P68104</td><td>Elongation factor 1-alpha 1</td></tr>
<tr><td>3886</td><td>dirMS_041712_NAR_30mM_plus2.16064.16064.2</td><td>2</td><td>14.05</td><td>0</td><td>1.91E+06</td><td></td><td>(K)ICSWNV</td><td>(K)I/C|S/W/N V/D/G L\R(A)</td><td>C:Carbamidomethylation</td><td>137.87</td><td>610.3003</td><td>1219.589</td><td>0.0045</td><td>3.7</td><td>5.84</td><td>19395.5</td><td>5.38</td><td>HUMAN</td><td>G3V359</td><td>DNA-(apurinic or apyrimidinic site) lyase (Fragment)</td></tr>
<tr><td>3887</td><td>dirMS_041712_NAR_30mM_plus2.21835.22044.2</td><td>2</td><td>14.05</td><td>0</td><td>3.21E+05</td><td></td><td>(R)SFITWLF</td><td>(R)S F|I|T|W|L/R(D)</td><td></td><td>170.53</td><td>461.7608</td><td>922.515</td><td>-0.0002</td><td>-0.2</td><td>9.47</td><td>141616.6</td><td>6.1</td><td>HUMAN</td><td>B7ZKM8</td><td>Protein transport protein Sec24B</td></tr>
<tr><td>3888</td><td>dirMS_041712_NAR_50mM_plus2.18059.18059.3</td><td>3</td><td>14.05</td><td>1</td><td>9.62E+04</td><td></td><td>(K)VVLDDK</td><td>(K)V/V|L/D/D K D Y F L F/R(D)</td><td></td><td>162.73</td><td>510.6044</td><td>1529.8</td><td>-0.0012</td><td>-0.8</td><td>4.43</td><td>5189.9</td><td>4.56</td><td>HUMAN</td><td>B8ZZ54</td><td>10 kDa heat shock protein, mitochondrial</td></tr>
<tr><td>3889</td><td>dirMS_041712_NAR_60mM_plus.20661.20661.3</td><td>3</td><td>14.05</td><td>0</td><td>1.68E+04</td><td></td><td>(K)HNQLPL</td><td>(K)H N Q L P L/V|I/E/F T/E Q T/A/P/K(I)</td><td></td><td>168.58</td><td>655.6825</td><td>1965.044</td><td>-0.0111</td><td>-5.6</td><td>5.4</td><td>51524.1</td><td>4.82</td><td>HUMAN</td><td>F5H8J2</td><td>Protein disulfide-isomerase</td></tr>
<tr><td>3890</td><td>dirMS_041712_NAR_20mM_minus.12345.12345.2</td><td>2</td><td>14.04</td><td>0</td><td>7.22E+05</td><td>M185m</td><td>(K)LAVNm</td><td>(K)L/A|V N m/V/P F/P\R(L)</td><td>m:Oxidized methionine</td><td>171.98</td><td>580.3173</td><td>1143.634</td><td>15.993</td><td>-1.6</td><td>9.75</td><td>42230.8</td><td>4.77</td><td>HUMAN</td><td>A6NKZ8</td><td>Putative tubulin beta chain-like protein ENSP00000290377</td></tr>
<tr><td>3891</td><td>dirMS_041712_NAR_50mM_minus2.16041.16060.3</td><td>3</td><td>14.04</td><td>1</td><td>8.40E+04</td><td></td><td>(K)LQVDAL</td><td>(K)L/Q|V/D A L|I/D/T I/K K(S)</td><td></td><td>159.23</td><td>452.9385</td><td>1356.81</td><td>-0.0087</td><td>-6.4</td><td>5.96</td><td>234333.2</td><td>6.01</td><td>HUMAN</td><td>J3KNX9</td><td>Unconventional myosin-XVIIIa</td></tr>
<tr><td>3892</td><td>dirMS_041712_NAR_50mM_minus2.16637.16637.3</td><td>3</td><td>14.04</td><td>1</td><td>1.61E+05</td><td></td><td>(K)TVTAMD</td><td>(K)T V|T A/M D V/V/Y A L K R(Q)</td><td></td><td>162.78</td><td>489.6051</td><td>1466.804</td><td>-0.0028</td><td>-1.9</td><td>8.25</td><td>11367.1</td><td>11.36</td><td>HUMAN</td><td>P62805</td><td>Histone H4</td></tr>
<tr><td>3893</td><td>dirMS_041712_NAR_20mM_plus.8851.8851.2</td><td>2</td><td>14.04</td><td>0</td><td>1.30E+05</td><td></td><td>(K)VLEEAN</td><td>(K)V/L|E/E/A\N Q/A\I/N/P K(L)</td><td></td><td>131.6</td><td>663.353</td><td>1325.706</td><td>-0.0072</td><td>-5.5</td><td>4.53</td><td>73202</td><td>9.19</td><td>HUMAN</td><td>C9JMU5</td><td>Probable ATP-dependent RNA helicase DDX17</td></tr>
<tr><td>3894</td><td>dirMS_041712_NAR_50mM_plus2.11292.11292.3</td><td>3</td><td>14.04</td><td>1</td><td>2.98E+05</td><td></td><td>(R)ETGVDL</td><td>(R)E T/G V/D/L T K/D N M/A/L Q/R(V)</td><td></td><td>127.18</td><td>564.2856</td><td>1690.843</td><td>-0.0006</td><td>-0.4</td><td>4.56</td><td>73964.5</td><td>5.87</td><td>HUMAN</td><td>P38646</td><td>Stress-70 protein, mitochondrial</td></tr>
<tr><td>3895</td><td>dirMS_041712_NAR_50mM_plus2.15212.15212.3</td><td>3</td><td>14.04</td><td>1</td><td>5.13E+05</td><td></td><td>(K)TKDGQII</td><td>(K)T K D G Q I|L|P V/P N V V V/R(D)</td><td></td><td>148.45</td><td>545.6573</td><td>1634.959</td><td>-0.0015</td><td>-0.9</td><td>8.41</td><td>56825.2</td><td>9.49</td><td>HUMAN</td><td>Q9P258</td><td>Protein RCC2</td></tr>
<tr><td>3896</td><td>dirMS_041712_NAR_60mM_plus.11415.11415.3</td><td>3</td><td>14.04</td><td>1</td><td>4.52E+05</td><td></td><td>(K)TCVFEKE</td><td>(K)T C|V|F E/K E N D/P S V/M R(S)</td><td>C:Carbamidomethylation</td><td>117.33</td><td>571.2635</td><td>1711.778</td><td>-0.0019</td><td>-1.1</td><td>4.68</td><td>267260.7</td><td>5.95</td><td>HUMAN</td><td>Q13085</td><td>Acetyl-CoA carboxylase 1</td></tr>
<tr><td>3897</td><td>dirMS_041712_NAR_60mM_plus.18515.18515.3</td><td>3</td><td>14.04</td><td>1</td><td>7.19E+05</td><td></td><td>(R)IRIDSLS</td><td>(R)I R I D S L\S A|Q|L|S Q/L Q/K(Q)</td><td></td><td>154.13</td><td>567.3254</td><td>1699.97</td><td>-0.0085</td><td>-5</td><td>8.75</td><td>63136.9</td><td>5.83</td><td>HUMAN</td><td>D6RAQ3</td><td>Prelamin-A/C</td></tr>
<tr><td>3898</td><td>dirMS_041712_NAR_120mM_minus.12022.12022.2</td><td>2</td><td>14.03</td><td>1</td><td>3.39E+05</td><td></td><td>(K)MKEIAE</td><td>(K)M K|E|I/A/E A/Y/L G K(T)</td><td></td><td>119.63</td><td>626.8305</td><td>1252.661</td><td>-0.0069</td><td>-5.5</td><td>5.9</td><td>17973</td><td>5.16</td><td>HUMAN</td><td>E9PI65</td><td>Heat shock cognate 71 kDa protein (Fragment)</td></tr>
<tr><td>3899</td><td>dirMS_041712_NAR_30mM_minus2.14674.14916.2</td><td>2</td><td>14.03</td><td>0</td><td>4.17E+05</td><td></td><td>(R)LWVDE</td><td>(R)L/W|V|D/E A V/R(A)</td><td></td><td>134.55</td><td>494.2641</td><td>987.526</td><td>-0.0049</td><td>-4.9</td><td>4.37</td><td>266161</td><td>5.68</td><td>HUMAN</td><td>E9PPU0</td><td>Epiplakin</td></tr>
<tr><td>3900</td><td>dirMS_041712_NAR_40mM_minus2.9414.9414.3</td><td>3</td><td>14.03</td><td>0</td><td>9.99E+04</td><td></td><td>(K)AASDIAI</td><td>(K)A A|S/D I|A/M T E/L/P/P T H P I R(L)</td><td></td><td>138.25</td><td>607.3174</td><td>1819.937</td><td>0.0006</td><td>0.3</td><td>5.32</td><td>12926.1</td><td>4.4</td><td>HUMAN</td><td>K7EM20</td><td>14-3-3 protein epsilon (Fragment)</td></tr>
<tr><td>3901</td><td>dirMS_041712_NAR_50mM_minus2.18366.18366.3</td><td>3</td><td>14.03</td><td>0</td><td>4.62E+05</td><td></td><td>(K)ALIEVLQ</td><td>(K)A L|I/E V/L Q/P L/I/A E H Q A R(R)</td><td></td><td>175.42</td><td>601.0139</td><td>1801.033</td><td>-0.0059</td><td>-3.3</td><td>5.4</td><td>53506.8</td><td>5.83</td><td>HUMAN</td><td>P23381</td><td>Tryptophan--tRNA ligase, cytoplasmic</td></tr>
<tr><td>3902</td><td>dirMS_041712_NAR_50mM_minus2.8737.8755.3</td><td>3</td><td>14.03</td><td>1</td><td>1.53E+05</td><td></td><td>(K)SPFEVS</td><td>(K)S P|F/E|V S V D K/A Q G D/A S/K(V)</td><td></td><td>117.75</td><td>555.6099</td><td>1664.813</td><td>0.0025</td><td>1.5</td><td>4.56</td><td>258215.9</td><td>5.48</td><td>HUMAN</td><td>E7EN95</td><td>Filamin-B</td></tr>
<tr><td>3903</td><td>dirMS_041712_NAR_120mM_plus.13267.13424.4</td><td>4</td><td>14.03</td><td>1</td><td>1.43E+06</td><td></td><td>(K)SVHSSV</td><td>(K)S V H\S S\V|P|L/L N S K/D/P I D/R(I)</td><td></td><td>123.27</td><td>466.7533</td><td>1863.992</td><td>-0.0009</td><td>-0.5</td><td>6.47</td><td>54427.4</td><td>5.96</td><td>HUMAN</td><td>K4DID9</td><td>Acetyl-CoA carboxylase 1</td></tr>
<tr><td>3904</td><td>dirMS_041712_NAR_20mM_plus.15629.15629.2</td><td>2</td><td>14.03</td><td>0</td><td>3.47E+05</td><td></td><td>(K)VGINYQ</td><td>(K)V G I/N Y\Q|P P T V\V|P/G G D L A/K(V)</td><td></td><td>179.05</td><td>912.9952</td><td>1824.985</td><td>-0.0023</td><td>-1.3</td><td>5.8</td><td>49012.3</td><td>4.9</td><td>HUMAN</td><td>A8MUB1</td><td>Tubulin alpha-4A chain</td></tr>
<tr><td>3905</td><td>dirMS_041712_NAR_20mM_plus.18965.18965.2</td><td>2</td><td>14.03</td><td>0</td><td>4.27E+04</td><td></td><td>(R)SIQLDG</td><td>(R)S I/Q L/D/G L/V/W G\A/S K(L)</td><td></td><td>201.65</td><td>687.3716</td><td>1373.742</td><td>-0.0064</td><td>-4.7</td><td>5.55</td><td>29184.1</td><td>4.91</td><td>HUMAN</td><td>E9PBQ9</td><td>Elongation factor 1-delta</td></tr>
<tr><td>3906</td><td>dirMS_041712_NAR_20mM_plus.7948.7948.2</td><td>2</td><td>14.03</td><td>0</td><td>4.82E+04</td><td></td><td>(K)YIDQEEL</td><td>(K)Y/I|D|Q E E L N K(T)</td><td></td><td>124.37</td><td>576.2845</td><td>1151.558</td><td>0.0038</td><td>3.3</td><td>4.14</td><td>85057.6</td><td>4.94</td><td>HUMAN</td><td>P07900</td><td>Heat shock protein HSP 90-alpha</td></tr>
<tr><td>3907</td><td>dirMS_041712_NAR_30mM_plus2.10626.10779.2</td><td>2</td><td>14.03</td><td>0</td><td>7.22E+05</td><td></td><td>(K)FLDQNL</td><td>(K)F/L|D|Q N/L/Q/K(Y)</td><td></td><td>109.55</td><td>503.271</td><td>1005.536</td><td>-0.0016</td><td>-1.6</td><td>5.84</td><td>334221.3</td><td>6.44</td><td>HUMAN</td><td>P15924</td><td>Desmoplakin</td></tr>
<tr><td>3908</td><td>dirMS_041712_NAR_30mM_plus2.18598.18598.2</td><td>2</td><td>14.03</td><td>0</td><td>1.48E+05</td><td></td><td>(R)TLTIVDT</td><td>(R)T L/T\I/V D/T/G/I G M T K(A)</td><td></td><td>151.4</td><td>675.3687</td><td>1349.735</td><td>-0.0044</td><td>-3.2</td><td>5.5</td><td>20151.2</td><td>4.58</td><td>HUMAN</td><td>G3V2J8</td><td>Heat shock protein HSP 90-alpha (Fragment)</td></tr>
<tr><td>3909</td><td>dirMS_041712_NAR_30mM_plus2.20959.21193.2</td><td>2</td><td>14.03</td><td>0</td><td>3.92E+05</td><td></td><td>(K)TIFAYFT</td><td>(K)T/I|F|A/Y/F/T G S K(D)</td><td></td><td>165.37</td><td>567.7949</td><td>1134.583</td><td>-0.0005</td><td>-0.4</td><td>8.26</td><td>8599.5</td><td>5.51</td><td>HUMAN</td><td>I3L0K2</td><td>Thioredoxin domain-containing protein 17</td></tr>
<tr><td>3910</td><td>dirMS_041712_NAR_60mM_plus.12320.12380.3</td><td>3</td><td>14.03</td><td>1</td><td>3.98E+05</td><td></td><td>(R)TSDVGG</td><td>(R)T/S|D|V/G/G Y/Y/Y E K I|E R(T)</td><td></td><td>121.38</td><td>560.6017</td><td>1679.791</td><td>-0.0006</td><td>-0.4</td><td>4.68</td><td>271827.6</td><td>4.73</td><td>HUMAN</td><td>P46821</td><td>Microtubule-associated protein 1B</td></tr>
<tr><td>3911</td><td>dirMS_041712_NAR_60mM_plus.12479.12479.3</td><td>3</td><td>14.03</td><td>1</td><td>3.27E+06</td><td></td><td>(K)VKLEAEI</td><td>(K)V K L E A E|I/A/T/Y/R(R)</td><td></td><td>122.93</td><td>431.5782</td><td>1292.721</td><td>-0.0008</td><td>-0.6</td><td>6.11</td><td>43773.3</td><td>5.26</td><td>HUMAN</td><td>F8VZY9</td><td>Keratin, type I cytoskeletal 18</td></tr>
<tr><td>3912</td><td>dirMS_041712_NAR_50mM_minus2.12417.12417.3</td><td>3</td><td>14.02</td><td>1</td><td>8.29E+04</td><td></td><td>(K)QLQALS</td><td>(K)Q L/Q/A/L/S S E L/A/Q A R D E/T K(K)</td><td></td><td>138.55</td><td>629.9954</td><td>1887.977</td><td>-0.0054</td><td>-2.9</td><td>4.68</td><td>71218.8</td><td>6.3</td><td>HUMAN</td><td>A7YIJ8</td><td>Radixin</td></tr>
<tr><td>3913</td><td>dirMS_041712_NAR_50mM_minus2.7686.7686.3</td><td>3</td><td>14.02</td><td>0</td><td>2.46E+05</td><td></td><td>(R)AALAHS</td><td>(R)A A L A H S E E\V|T/A|S Q/V A/A\T/K(T)</td><td></td><td>110.93</td><td>595.3104</td><td>1783.919</td><td>-0.0018</td><td>-1</td><td>5.4</td><td>23903</td><td>4.94</td><td>HUMAN</td><td>H0YDN1</td><td>Plectin (Fragment)</td></tr>
<tr><td>3914</td><td>dirMS_041712_NAR_60mM_minus.17284.17284.3</td><td>3</td><td>14.02</td><td>0</td><td>3.14E+05</td><td></td><td>(K)GSDHSA</td><td>(K)G S D H\S A S\L/E|P/G E/L/A\E L\V R(S)</td><td></td><td>156.82</td><td>622.9764</td><td>1866.919</td><td>-0.0045</td><td>-2.4</td><td>4.4</td><td>12626.3</td><td>5.59</td><td>HUMAN</td><td>Q5TBR0</td><td>N-acetylneuraminic acid synthase (Fragment)</td></tr>
<tr><td>3915</td><td>dirMS_041712_NAR_120mM_plus.8150.8150.2</td><td>2</td><td>14.02</td><td>0</td><td>4.29E+05</td><td></td><td>(K)AGEVFI</td><td>(K)A/G\E|V/F I/H|K(D)</td><td></td><td>96.3</td><td>450.7531</td><td>900.494</td><td>0.0051</td><td>5.7</td><td>6.79</td><td>23729.5</td><td>9.89</td><td>HUMAN</td><td>C9IZL7</td><td>Non-POU domain-containing octamer-binding protein (Fragment)</td></tr>
<tr><td>3916</td><td>dirMS_041712_NAR_20mM_plus.18712.18712.2</td><td>2</td><td>14.02</td><td>0</td><td>2.34E+05</td><td></td><td>(K)IYVDDG</td><td>(K)I/Y|V|D D/G\L I/S L/Q V K(Q)</td><td></td><td>200.65</td><td>731.9068</td><td>1462.815</td><td>-0.0088</td><td>-6</td><td>4.21</td><td>30947.9</td><td>6.47</td><td>HUMAN</td><td>H3BQ34</td><td>Pyruvate kinase</td></tr>
<tr><td>3917</td><td>dirMS_041712_NAR_30mM_plus2.11688.11688.2</td><td>2</td><td>14.02</td><td>0</td><td>5.34E+05</td><td></td><td>(K)VASGCLI</td><td>(K)V/A|S/G/C L/D I/N S S V K(G)</td><td>C:Carbamidomethylation</td><td>114.8</td><td>675.3393</td><td>1349.673</td><td>-0.0016</td><td>-1.2</td><td>5.81</td><td>56837</td><td>7.19</td><td>HUMAN</td><td>E7ETT1</td><td>Propionyl-CoA carboxylase beta chain, mitochondrial</td></tr>
<tr><td>3918</td><td>dirMS_041712_NAR_60mM_plus.14788.14949.3</td><td>3</td><td>14.02</td><td>1</td><td>1.28E+06</td><td></td><td>(R)KFLDGIY</td><td>(R)K\F\L D\G\I|Y|V/S/E/K(G)</td><td></td><td>134.67</td><td>433.5671</td><td>1298.699</td><td>-0.0123</td><td>-9.5</td><td>6.07</td><td>20931.1</td><td>10.28</td><td>HUMAN</td><td>D6RAN4</td><td>60S ribosomal protein L9 (Fragment)</td></tr>
<tr><td>3919</td><td>dirMS_041712_NAR_120mM_minus.8180.8180.3</td><td>3</td><td>14.01</td><td>2</td><td>1.08E+06</td><td></td><td>(K)KGAVAE</td><td>(K)K|G/A\V/A/E D G D/E/L R T\E\P E\A|K(K)</td><td></td><td>99.27</td><td>638.9836</td><td>1914.94</td><td>-0.0041</td><td>-2.1</td><td>4.36</td><td>19284.3</td><td>5.23</td><td>HUMAN</td><td>G3V3C7</td><td>DNA-(apurinic or apyrimidinic site) lyase (Fragment)</td></tr>
</table>

3920	dirMS_041712_NAR_30mM_minus2.11130.11130.2	2	14.01	0	6.40E+05		(K)VAGQD	(K)V/A|G|Q/D/G S V V/Q/F K(I)		114.03	617.824	1234.643	-0.0019	-1.5	5.81	17127.4	10.05	HUMAN	A8MU27	Small ubiquitin-related modifier 3
3921	dirMS_041712_NAR_30mM_minus2.18285.18285.2	2	14.01	0	1.57E+05		(K)FGAYIVD	(K)F G/A|Y/I/V/D/G L R(Q)		153.6	555.7984	1110.594	-0.0047	-4.2	5.83	269989.9	5.92	HUMAN	F8W8T8	Acetyl-CoA carboxylase 2
3922	dirMS_041712_NAR_50mM_minus2.12487.12487.3	3	14.01	0	1.87E+05		(R)VLVVHD	(R)V/L/V/V/H D/G F E G L/A K(G)		139.63	461.9238	1383.763	-0.0062	-4.5	5.32	18719.9	6.91	HUMAN	F8VP00	6-phosphofructokinase, muscle type (Fragment)
3923	dirMS_041712_NAR_60mM_minus.10374.10374.3	3	14.01	0	5.97E+05		(K)AQVARP	(K)A/Q|V/A/R P G G D T I/F/G K(I)		117.88	472.9235	1416.759	-0.0034	-2.4	8.79	7323.5	9.4	HUMAN	D6RC06	Histidine triad nucleotide-binding protein 1
3924	dirMS_041712_NAR_60mM_minus.14868.14868.3	3	14.01	1	2.72E+05		(R)KLQEEEI	(R)K L Q E E E\L\L/A/T|Q/V/D|M R(A)		142.8	601.6438	1802.932	-0.0148	-8.2	4.41	35935.5	6.2	HUMAN	Q8IVM0	Coiled-coil domain-containing protein 50
3925	dirMS_041712_NAR_20mM_plus.12477.12477.2	2	14.01	0	1.88E+05		(R)GVEICIA	(R)G V\E/I|C I/A T/P G R(L)	C:Carbamidomethylation	158.73	586.8084	1172.609	0.0003	0.3	6	61018.6	9.44	HUMAN	B4DLW8	Probable ATP-dependent RNA helicase DDX5
3926	dirMS_041712_NAR_30mM_plus2.12059.12138.2	2	14.01	0	3.47E+05		(R)DVCLYPI	(R)D V|C/L/Y/P/R(F)	C:Carbamidomethylation	116.58	461.7327	922.445	0.013	14.1	5.83	35548.6	4.91	HUMAN	B4DN60	Asparagine--tRNA ligase, cytoplasmic
3927	dirMS_041712_NAR_30mM_plus2.19499.19499.2	2	14.01	0	2.88E+04		(R)FLLADNI	(R)F L|L|A/D/N\L Y C/K(A)	C:Carbamidomethylation	155.03	628.8226	1256.634	0.0036	2.8	5.83	22400.1	6.19	HUMAN	F5H2A7	Prefoldin subunit 3
3928	dirMS_041712_NAR_30mM_plus2.20437.20437.2	2	14.01	0	3.07E+05		(R)FEELNAI	(R)F/E/E|L/N A/D L/F R(G)		161.07	627.3106	1253.616	-0.0022	-1.7	4.14	45508.6	5.5	HUMAN	A8K7Q2	Heat shock cognate 71 kDa protein
3929	dirMS_041712_NAR_50mM_plus2.9135.9156.3	3	14.01	1	3.70E+05		(R)IITEGFE	(R)I|I|T|E/G F/E/A/A K E K(A)		115.27	445.9079	1335.716	-0.0063	-4.7	4.79	55323	6.02	HUMAN	B4DPJ8	T-complex protein 1 subunit zeta
3930	dirMS_041712_NAR_20mM_minus.7839.7872.2	2	14	0	1.69E+05		(K)SVEAAA	(K)S V|E|A/A/A/E/L S A/K(D)		128.45	538.2838	1075.563	-0.0027	-2.5	4.53	12131.5	11.14	HUMAN	F5GXR3	Parathymosin
3931	dirMS_041712_NAR_30mM_minus2.12205.12205.2	2	14	0	1.31E+05		(R)TLSGME	(R)T L|S G M/E/S/Y C V R(A)	C:Carbamidomethylation	119.47	651.795	1302.582	0.001	0.8	5.66	42755	7.81	HUMAN	B7Z2F4	T-complex protein 1 subunit delta
3932	dirMS_041712_NAR_30mM_minus2.19028.19114.2	2	14	0	2.61E+05		(K)SYELPD	(K)S Y E|L|P D G Q/V|I/T\I/G N E R(F)		158.88	895.9492	1790.892	-0.0008	-0.4	4.14	123097.1	5.83	HUMAN	A5A3E0	POTE ankyrin domain family member F
3933	dirMS_041712_NAR_60mM_minus.8097.8097.3	3	14	1	4.11E+05		(R)YLAEVA	(R)Y L/A E\V/A/T G E/K/R(A)		104.48	412.8902	1236.658	-0.0022	-1.8	6.14	28473.3	4.8	HUMAN	P61981	14-3-3 protein gamma
3934	dirMS_041712_NAR_120mM_plus.15261.15478.4	4	14	0	2.28E+06		(K)HLEINPD	(K)H L\E\I\N|P D H/P I V/E T L/R(Q)		133.9	446.4951	1782.95	0.0089	5	5.27	83605	4.97	HUMAN	P08238	Heat shock protein HSP 90-beta
3935	dirMS_041712_NAR_20mM_plus.11309.11309.3	3	14	0	1.13E+05		(R)GCITIIG	(R)G C\I T\I I/G G G D T/A/T/C C A K(W)	C:Carbamidomethylation	150.28	585.5999	1754.787	-0.0019	-1.1	5.84	41827.5	7	HUMAN	B7Z7A9	Phosphoglycerate kinase
3936	dirMS_041712_NAR_20mM_plus.17612.17612.2	2	14	0	1.07E+05	M90m	(R)LFVGNLI	(R)L F V G N/L|P/P D\I T/E E E m R(K)	m:Oxidized methionine	192.97	938.4602	1859.921	15.9923	-1.4	4	23729.5	9.89	HUMAN	C9IZL7	Non-POU domain-containing octamer-binding protein (Fragment)
3937	dirMS_041712_NAR_30mM_plus2.11588.11588.2	2	14	0	3.91E+06		(K)SCNCLLL	(K)S/C|N C L/L|L/K(V)	C:Carbamidomethylation	114.55	504.2556	1007.501	0.0027	2.7	8.47	47510.4	7.04	HUMAN	P06733	Alpha-enolase
3938	dirMS_041712_NAR_30mM_plus2.14019.14019.2	2	14	0	2.47E+05		(K)SSGGFV	(K)S S|G/G F|V/W/A/C/K(N)	C:Carbamidomethylation	126.2	549.7552	1098.504	-0.0006	-0.5	8.47	45578.2	7.83	HUMAN	B4DFL2	Isocitrate dehydrogenase [NADP]
3939	dirMS_041712_NAR_30mM_plus2.16688.16827.2	2	14	0	3.46E+05		(R)SIQADG	(R)S I/Q|A/D|G L/V|W G S S K(L)		140.6	674.3475	1347.69	-0.0026	-1.9	5.55	24934.4	4.5	HUMAN	P24534	Elongation factor 1-beta
3940	dirMS_041712_NAR_30mM_plus2.7618.7640.2	2	14	0	1.73E+06		(R)MGPVM	(R)M/G|P|V/M D/R(M)		94.18	403.1894	805.37	0.002	2.5	5.59	77799.7	9.09	HUMAN	P52272	Heterogeneous nuclear ribonucleoprotein M
3941	dirMS_041712_NAR_40mM_plus2.14581.14581.3	3	14	0	4.84E+05		(K)IAILTCPI	(K)I A|I L/T/C P F E/P/P K P K(T)	C:Carbamidomethylation	153.47	537.6381	1610.898	0.0023	1.4	8.59	49982	5.86	HUMAN	B4DYD8	T-complex protein 1 subunit epsilon
3942	dirMS_041712_NAR_60mM_plus.16901.16901.3	3	14	0	8.89E+05		(K)TTISVAH	(K)T/T|I/S V A H L\L/A A/R(Q)		144.63	418.2478	1252.737	-0.0083	-6.7	9.44	34143.2	10.07	HUMAN	B4DT23	Protein FAM98A
3943	dirMS_041712_NAR_30mM_minus2.12393.12807.2	2	13.99	0	5.64E+05		(K)GDYPLE	(K)G D/Y|P/L/E/A V/R(M)		122.22	510.2598	1019.516	-0.0033	-3.2	4.37	18466.1	5.78	HUMAN	H3BSU3	Pyruvate kinase (Fragment)
3944	dirMS_041712_NAR_30mM_minus2.15615.15615.2	2	13.99	0	1.51E+05		(K)AMTGVI	(K)A M|T|G V/E Q/W P Y/R(A)		138.37	669.3172	1337.631	-0.0036	-2.7	6.04	56186.7	5.47	HUMAN	B4DUR8	T-complex protein 1 subunit gamma
3945	dirMS_041712_NAR_50mM_minus2.11474.11474.3	3	13.99	1	2.41E+04		(R)ESDVPLI	(R)E/S D V/P/L K\T/E E F E V T K(T)		133.62	584.3049	1750.874	0.0256	14.6	4.25	18909	6.21	HUMAN	Q969H8	UPF0556 protein C19orf10
3946	dirMS_041712_NAR_50mM_minus2.13446.13446.3	3	13.99	1	5.31E+05		(K)LTEDKAI	(K)L/T/E D K A D V Q/S\I/I|G/L Q R(F)		144.8	595.9939	1785.97	-0.0034	-1.9	4.56	90316.8	5.52	HUMAN	Q13263	Transcription intermediary factor 1-beta
3947	dirMS_041712_NAR_60mM_minus.9631.9758.3	3	13.99	1	5.37E+05		(R)IITEGFE	(R)I/I|T E/G F/E A A/K E K(A)		113.88	445.91	1335.716	0	0	4.79	55323	6.02	HUMAN	B4DPJ8	T-complex protein 1 subunit zeta
3948	dirMS_041712_NAR_120mM_plus.8693.8738.2	2	13.99	0	8.36E+05		(K)HFVALST	(K)H/F|V/A/L|S T/N/T/T K(V)		99.1	609.8311	1218.648	0.0072	5.9	8.76	63374.2	8.64	HUMAN	P06744	Glucose-6-phosphate isomerase
3949	dirMS_041712_NAR_30mM_plus2.17274.17774.2	2	13.99	0	7.74E+05		(K)LMFEELI	(K)L/M|F/E/E/L/R(N)		145.37	469.2488	937.481	0.0092	9.8	4.53	19338.9	5.33	HUMAN	E7EQS9	tRNA-splicing ligase RtcB homolog
3950	dirMS_041712_NAR_40mM_plus2.15431.15431.3	3	13.99	0	2.03E+05		(K)GVTIIGP	(K)G V T/I/I/G/P A/T/V G G I K/P G C F K(I)	C:Carbamidomethylation	156.62	624.683	1872.041	-0.0067	-3.6	10	91839.6	9.22	HUMAN	B4E3P0	ATP-citrate synthase
3951	dirMS_041712_NAR_50mM_plus2.19032.19154.3	3	13.99	1	1.72E+06		(K)LIQLMEI	(K)L/I|Q|L/M E E I/M/A/E K E/N K(T)		169.7	606.9845	1818.934	0.005	2.7	4.49	73202	9.19	HUMAN	C9JMU5	Probable ATP-dependent RNA helicase DDX17
3952	dirMS_041712_NAR_60mM_plus.17883.17909.3	3	13.99	1	2.50E+05		(R)GQFSTD	(R)G Q|F/S/T D E L/V/A/E V/E/K R(N)		150.63	569.9588	1707.855	0.007	4.1	4.41	144109.9	6.01	HUMAN	J3KR87	Clathrin heavy chain 2
3953	dirMS_041712_NAR_120mM_minus.12144.12144.3	3	13.98	1	3.21E+05		(K)TLTTVQ	(K)T L|T/T V/Q/G I/A D D Y D K K/K(L)		120.02	599.3185	1795.944	-0.0027	-1.5	5.71	13778.5	7.96	HUMAN	K7EM18	Eukaryotic translation initiation factor 1
3954	dirMS_041712_NAR_30mM_minus2.13638.13673.2	2	13.98	0	6.95E+05		(R)LPLQDV	(R)L/P|L/Q/D V/Y/K(I)		128	488.273	975.551	-0.0122	-12.5	5.83	50482.3	9.31	HUMAN	P68104	Elongation factor 1-alpha 1
3955	dirMS_041712_NAR_20mM_plus.10185.10185.2	2	13.98	0	2.01E+05		(K)LSDGVA	(K)L/S/D/G V A V|L K(V)		143.02	451.2712	901.535	-0.0002	-0.2	5.84	61224.7	5.7	HUMAN	P10809	60 kDa heat shock protein, mitochondrial
3956	dirMS_041712_NAR_20mM_plus.18846.18846.2	2	13.98	0	1.81E+05		(K)TAVETA	(K)T A\V/E/T A/V L|L L/R(I)		200.4	593.3625	1185.72	-0.0024	-2	5.66	56186.7	5.47	HUMAN	B4DUR8	T-complex protein 1 subunit gamma
3957	dirMS_041712_NAR_30mM_plus2.6032.6032.2	2	13.98	0	1.69E+05		(R)SQESGY	(R)S Q|E|S/G/Y/Y D/R(M)		85.9	552.7331	1104.459	-0.0003	-0.3	4.37	100478.9	5.71	HUMAN	A8MXP9	Matrin-3
3958	dirMS_041712_NAR_30mM_plus2.6607.6888.2	2	13.98	0	1.10E+06		(K)SLDEME	(K)S/L|D|E M/E/K(Q)		90	426.1971	851.381	0.0054	6.4	4.14	26419.2	5.1	HUMAN	H0Y9K7	Splicing factor, proline- and glutamine-rich (Fragment)
3959	dirMS_041712_NAR_30mM_plus2.9392.9392.2	2	13.98	0	6.90E+05		(K)SVEAAA	(K)S V|E/A/A/A/E/L/S A K(D)		103.03	538.2855	1075.563	0.0007	0.7	4.53	12131.5	11.14	HUMAN	F5GXR3	Parathymosin
3960	dirMS_041712_NAR_50mM_plus2.21495.21495.3	3	13.98	0	8.95E+04		(K)GAEILEV	(K)G A|E/I/L/E/V L/H S L P A\V/R(Q)		183.4	535.3092	1603.917	-0.0036	-2.2	5.4	52378.2	6.32	HUMAN	C9IZE4	26S proteasome non-ATPase regulatory subunit 6
3961	dirMS_041712_NAR_60mM_plus.11782.11801.3	3	13.98	1	1.10E+06		(K)QSSSSRI	(K)Q/S/S S/S R D D N M/F/Q I/G/K(M)		119.07	567.2609	1699.77	-0.0023	-1.3	5.96	14395.1	9.6	HUMAN	P53999	Activated RNA polymerase II transcriptional coactivator p15
3962	dirMS_041712_NAR_60mM_plus.9382.9382.2	2	13.98	0	1.98E+06		(R)IGAEVYI	(R)I/G|A/E/V/Y/H N\L\K(N)		107.55	572.3143	1143.616	0.0056	4.9	6.75	21077	9.05	HUMAN	K7EM90	Alpha-enolase (Fragment)
3963	dirMS_041712_NAR_120mM_minus.11726.11726.3	3	13.97	2	3.38E+05		(R)SGKYDLI	(R)S G K Y/D L D/F K/S|P D D|P S R(Y)		117.87	609.6226	1826.855	-0.0023	-1.3	4.58	47510.4	7.04	HUMAN	P06733	Alpha-enolase
3964	dirMS_041712_NAR_30mM_minus2.14319.14319.2	2	13.97	0	7.55E+04		(R)TPYGASI	(R)T/P|Y/G/A|S I F\Q S K(E)		131.18	599.8093	1198.61	0.0011	0.9	8.26	44815.8	6.62	HUMAN	B3KQ29	Transducin-like enhancer protein 4
3965	dirMS_041712_NAR_30mM_minus2.14339.14339.2	2	13.97	0	1.70E+05		(K)IVVLLQR	(K)I/V|V/L L/Q/R(L)		131.83	420.7871	840.567	0.0004	0.4	9.75	28772.3	6.28	HUMAN	A6NJG9	Proteasome activator complex subunit 1
3966	dirMS_041712_NAR_30mM_minus2.16427.16427.2	2	13.97	0	5.06E+05	M185m	(K)LAVNm\	(K)L/A/V/N/m/V P F P R(L)	m:Oxidized methionine	142.53	580.3178	1143.634	15.994	-0.8	9.75	42230.8	4.77	HUMAN	A6NKZ8	Putative tubulin beta chain-like protein ENSP00000290377
3967	dirMS_041712_NAR_30mM_minus2.8899.8899.2	2	13.97	0	4.88E+05		(K)SDPVVS	(K)S D/P V/V/S Y/R(E)		102.7	461.7315	922.463	-0.0071	-7.7	5.55	96306.4	6.41	HUMAN	P13639	Elongation factor 2
3968	dirMS_041712_NAR_60mM_minus.15161.15206.3	3	13.97	1	3.39E+05		(K)AKLEQLI	(K)A K L E Q\L|F|Q/D E V/A/K(A)		144.37	506.9421	1518.816	-0.0045	-3	4.68	23903	4.94	HUMAN	H0YDN1	Plectin (Fragment)
3969	dirMS_041712_NAR_60mM_minus.15721.15721.3	3	13.97	1	1.58E+06		(R)KDDPVT	(R)K D D\P V\T\N L/N N/A|F E V/A E K(Y)		147.43	635.317	1903.94	-0.0032	-1.7	4.32	21601.4	6.27	HUMAN	K7EJH8	Alpha-actinin-4 (Fragment)
3970	dirMS_041712_NAR_60mM_minus.17275.17275.3	3	13.97	0	9.52E+04		(R)GFGHIG	(R)G F G H I G I\A\V|P D/V Y S A/C K(R)	C:Carbamidomethylation	156.02	597.6347	1790.889	0.0001	0.1	6.74	21005.6	5.12	HUMAN	Q04760	Lactoylglutathione lyase
3971	dirMS_041712_NAR_60mM_minus.18816.18816.3	3	13.97	0	2.56E+05		(K)LHDNLII	(K)L H\D/N L\I|I/S|D L E N T V K(K)		166.17	575.3117	1723.922	-0.0019	-1.1	4.54	88624.6	5.09	HUMAN	C9J8U1	Cytospin-A (Fragment)
3972	dirMS_041712_NAR_120mM_plus.9455.9455.3	3	13.97	0	3.32E+05		(K)QEYDES	(K)Q E Y|D E/S/G/P/S/I/V H/R(K)		102.88	506.2393	1516.703	0.0007	0.5	4.65	123097.1	5.83	HUMAN	A5A3E0	POTE ankyrin domain family member F
3973	dirMS_041712_NAR_120mM_plus.9745.9839.3	3	13.97	0	9.06E+05		(R)HGESAV	(R)H G\E S\A\W|N|L/E/N R(F)		104.47	438.2077	1312.603	0.0057	4.3	5.4	28917.5	6.68	HUMAN	P18669	Phosphoglycerate mutase 1
3974	dirMS_041712_NAR_20mM_plus.16208.16208.3	3	13.97	0	1.32E+05		(R)LLDAQL	(R)L/L D/A Q\L A/T/G G|I/V/D/P R(L)		183.53	513.6218	1538.854	-0.0028	-1.8	4.21	533778	5.74	HUMAN	Q15149	Plectin
3975	dirMS_041712_NAR_20mM_plus.18100.18115.2	2	13.97	0	1.72E+05		(K)DAGVIA	(K)D A\G|V|I/A/G L/N/V L/R(I)		196.72	599.3505	1197.695	-0.0013	-1.1	5.84	58502.6	6.09	HUMAN	B4DXY3	Heat shock 70 kDa protein 1-like
3976	dirMS_041712_NAR_30mM_plus2.17966.17966.2	2	13.97	0	2.91E+05		(R)VNPALA	(R)V N|P A|L|A E/L N L R(S)		147.78	605.3509	1209.695	-0.0005	-0.4	5.97	20026.6	4.67	HUMAN	E9PIK5	Ribonuclease inhibitor (Fragment)
3977	dirMS_041712_NAR_30mM_plus2.20672.20672.2	2	13.97	0	2.60E+05		(R)ASLSLW	(R)A S|L/S\L/W/Q/A M E K(G)		162.9	632.3231	1263.64	-0.0013	-1	6.05	266161	5.68	HUMAN	E9PPU0	Epiplakin
3978	dirMS_041712_NAR_30mM_plus2.22008.22008.2	2	13.97	0	1.96E+05		(R)LVFGFLI	(R)L V/F/G/F L N\G/R(A)		170.8	511.7906	1022.578	-0.0043	-4.2	9.75	10585.9	6.9	HUMAN	G3V2H3	Purine nucleoside phosphorylase (Fragment)
3979	dirMS_041712_NAR_40mM_plus2.12257.12257.3	3	13.97	0	2.19E+05		(K)AASDIAI	(K)A A/S/D\I/A M T/E L/P/P T H P I R(L)		139.18	607.3167	1819.937	-0.0015	-0.9	5.32	12926.1	4.4	HUMAN	K7EM20	14-3-3 protein epsilon (Fragment)
3980	dirMS_041712_NAR_50mM_plus2.14642.14642.3	3	13.97	1	5.92E+05		(R)ELVFKEI	(R)E L|V/F/K E D G Q E/Y/A Q V I/K(M)		144.98	632.6588	1895.975	-0.0131	-6.9	4.41	14568.9	5.17	HUMAN	A6NJH9	Eukaryotic translation initiation factor 1A, Y-chromosomal
3981	dirMS_041712_NAR_60mM_plus.13736.13904.3	3	13.97	1	1.40E+06		(R)EMDRET	(R)E|M/D R E T\L|I/D/V/A/R(T)		128.93	483.2457	1447.721	0.0016	1.1	4.32	55323	6.02	HUMAN	B4DPJ8	T-complex protein 1 subunit zeta
3982	dirMS_041712_NAR_60mM_plus.17942.17942.3	3	13.97	1	3.58E+05		(K)CEFQDA	(K)C E|F\Q/D A/Y/V L/L S E/K/K(I)	C:Carbamidomethylation	150.55	577.288	1729.847	0.0029	1.7	4.68	61224.7	5.7	HUMAN	P10809	60 kDa heat shock protein, mitochondrial
3983	dirMS_041712_NAR_40mM_minus2.8086.8086.2	2	13.96	0	7.00E+04		(R)IVLQIDN	(R)I V|L/Q/I/D N A R(L)		128.1	521.3059	1041.605	-0.0006	-0.6	5.84	20842.9	5.32	HUMAN	C9JM50	Keratin, type I cytoskeletal 19 (Fragment)
3984	dirMS_041712_NAR_50mM_minus2.7924.7924.3	3	13.96	1	1.81E+05		(R)IITEGFE	(R)I/I/T E/G F E A A/K E K(A)		112.27	445.912	1335.716	0.006	4.5	4.79	55323	6.02	HUMAN	B4DPJ8	T-complex protein 1 subunit zeta
3985	dirMS_041712_NAR_120mM_plus.4136.4136.3	3	13.96	1	1.59E+07	M310m	(R)TKTEISE	(R)T\K|T E\I|S/E/m N/R(N)	m:Oxidized methionine	69.77	408.8687	1208.594	15.9976	2.2	5.81	37296.3	5.3	HUMAN	F8VU64	Keratin, type II cytoskeletal 8
3986	dirMS_041712_NAR_120mM_plus.8674.8674.2	2	13.96	0	8.21E+05		(K)HFVALST	(K)H/F|V|A L/S/T N/T T K(V)		99.08	609.8311	1218.648	0.0072	5.9	8.76	63374.2	8.64	HUMAN	P06744	Glucose-6-phosphate isomerase
3987	dirMS_041712_NAR_20mM_plus.13705.13705.2	2	13.96	0	2.22E+05	M98m	(R)TLTIVDT	(R)T L\T\I/V|D/T G/I/G m T K(A)	m:Oxidized methionine	166.8	683.3671	1349.735	15.9924	-1.8	5.5	20151.2	4.58	HUMAN	G3V2J8	Heat shock protein HSP 90-alpha (Fragment)
3988	dirMS_041712_NAR_20mM_plus.18118.18266.2	2	13.96	0	1.13E+05		(K)LVLPSLIS	(K)L V|L|P/S/L I/S S R(I)		196.6	542.8388	1084.672	-0.0021	-2	9.75	39066	6.61	HUMAN	A6PVW9	Selenium binding protein 1 (Fragment)
3989	dirMS_041712_NAR_20mM_plus.18792.18792.2	2	13.96	0	2.87E+05		(K)LAVEALS	(K)L A V\E/A|L/S/S/L D/G D L A G R(Y)		200.48	793.9214	1586.838	-0.0029	-1.8	4.03	24286.7	4.89	HUMAN	G3V4N7	Creatine kinase B-type (Fragment)
3990	dirMS_041712_NAR_30mM_plus2.18403.18403.2	2	13.96	0	2.19E+05		(K)GVEGLID	(K)G V|E G L|I|D I/E/N/P\N R(V)		149.47	713.3669	1425.733	-0.0067	-4.7	4.14	20629.7	8.84	HUMAN	Q13442	28 kDa heat- and acid-stable phosphoprotein
3991	dirMS_041712_NAR_50mM_plus2.16910.16910.3	3	13.96	1	9.13E+05		(K)LFTAESL	(K)L/F|T/A E S L/I/G L K N P|E K(S)		157.53	553.9791	1659.932	-0.0089	-5.3	6.14	62195.8	5.63	HUMAN	B0YIW6	Archain 1, isoform CRA_a
3992	dirMS_041712_NAR_60mM_plus.11301.11301.3	3	13.96	0	1.93E+06		(R)EIVHIQA	(R)E/I V H I Q A G/Q/C/G N Q I|G A K(F)	C:Carbamidomethylation	116.42	608.311	1822.923	-0.0044	-2.4	6.85	40069.4	4.83	HUMAN	E7EWR1	Tubulin beta chain
3993	dirMS_041712_NAR_60mM_plus.11320.11320.3	3	13.96	0	1.93E+06		(R)EIVHIQA	(R)E/I V H I Q A/G/Q/C/G/N Q I G A K(F)	C:Carbamidomethylation	116.42	608.311	1822.923	-0.0044	-2.4	6.85	40069.4	4.83	HUMAN	E7EWR1	Tubulin beta chain
3994	dirMS_041712_NAR_60mM_plus.16411.16569.3	3	13.96	1	6.93E+05		(K)AAVEWI	(K)A/A/V|E/W/F/D/G K D F Q G S K(L)		142.92	562.2762	1684.797	0.0175	10.4	4.56	65213.5	9.57	HUMAN	B0QYK0	Ewing sarcoma breakpoint region 1
3995	dirMS_041712_NAR_60mM_plus.16699.16763.3	3	13.96	1	3.08E+05		(K)ARLEIEP	(K)A R L E\I\E|P|E/W A/Y/G K(K)		144.35	521.2734	1561.801	0.0047	3	4.79	25233.4	9.34	HUMAN	Q00688	Peptidyl-prolyl cis-trans isomerase FKBP3
3996	dirMS_041712_NAR_60mM_plus.17169.17169.3	3	13.96	1	1.45E+04		(R)LLDAQL	(R)L/L|D A/Q/L/A T G G L V C P A R R(L)	C:Carbamidomethylation	148.03	604.3409	1810.996	0.0125	6.9	9.6	266161	5.68	HUMAN	E9PPU0	Epiplakin
3997	dirMS_041712_NAR_60mM_plus.17680.17680.3	3	13.96	1	4.10E+05		(K)ISDSEGF	(K)I/S/D/S/E/G F/K/A N L S L L R(R)		149.75	550.632	1649.886	-0.0043	-2.6	6.07	76262.2	9.53	HUMAN	P23246	Splicing factor, proline- and glutamine-rich
3998	dirMS_041712_NAR_120mM_minus.5466.5466.2	2	13.95	0	1.59E+05	M161m	(R)mQHLIA	(R)m Q|H/L I|A R(E)	m:Oxidized methionine	83.22	442.7426	868.482	15.9958	1	9.52	18466.1	5.78	HUMAN	H3BSU3	Pyruvate kinase (Fragment)
3999	dirMS_041712_NAR_40mM_minus2.15537.15580.3	3	13.95	1	7.01E+05		(K)LLPLVSD	(K)L L|P/L/V/S D/E/V/F I R D/V A K(V)		180.3	605.3511	1814.042	-0.0035	-1.9	4.56	97378.8	6.4	HUMAN	P11216	Glycogen phosphorylase, brain form
4000	dirMS_041712_NAR_50mM_minus2.11386.11386.3	3	13.95	1	1.33E+05		(K)DVDEAY	(K)D V|D/E A/Y M/N K V/E L E S R(L)		133.2	599.9481	1797.832	-0.0026	-1.5	4.18	13588.1	4.66	HUMAN	F8VP67	Keratin, type II cytoskeletal 8 (Fragment)
4001	dirMS_041712_NAR_60mM_minus.14890.14890.3	3	13.95	0	3.37E+05		(R)AVAIDLI	(R)A V|A\I/D L/P G L G H S K(E)		142.65	426.578	1277.721	-0.0018	-1.4	6.79	19983.6	8.14	HUMAN	B4DQI4	Alpha/beta hydrolase domain-containing protein 14B
4002	dirMS_041712_NAR_60mM_minus.6264.6264.3	3	13.95	1	1.15E+05		(K)DSYVGD	(K)D S/Y/V|G D E A|Q S K R(G)		93.23	452.2129	1354.623	0.0008	0.6	4.56	32616.5	5.18	HUMAN	A6NL76	Actin, alpha skeletal muscle
4003	dirMS_041712_NAR_20mM_plus.13709.13709.3	3	13.95	0	1.00E+05		(K)IFVGNV	(K)I F V G\N V|S\A A/C/T/S/Q/E L R(S)	C:Carbamidomethylation	166.63	584.6279	1751.874	-0.0053	-3.1	6	37433.2	9.17	HUMAN	B0LM41	Protein RBM14-RBM4
4004	dirMS_041712_NAR_20mM_plus.17517.17517.2	2	13.95	0	1.61E+05	M74m	(R)mIAGQ	(R)m I|A|G Q\V|L/D I N L A A E\P K(V)	m:Oxidized methionine	192.85	849.9568	1682.915	15.9918	-1.9	4.37	15791.7	9.22	HUMAN	B4DSU6	Heterogeneous nuclear ribonucleoproteins C1/C2
4005	dirMS_041712_NAR_30mM_plus2.16200.16482.2	2	13.95	0	5.66E+05		(R)YIEIFR(S	(R)Y/I|E/I/F/R(S)		138.45	420.7344	840.461	0.0001	0.1	6	36982.9	6.37	HUMAN	P31942	Heterogeneous nuclear ribonucleoprotein H3
4006	dirMS_041712_NAR_30mM_plus2.8895.8895.2	2	13.95	0	3.86E+05		(R)CSEQVQ	(R)C S|E/Q\V/Q/D|F|T\K(C)	C:Carbamidomethylation	100.43	621.2771	1241.547	0.0002	0.2	4.37	13533.5	9.82	HUMAN	H7C3D8	COX assembly mitochondrial protein homolog (Fragment)
4007	dirMS_041712_NAR_120mM_minus.6940.6940.2	2	13.94	1	2.88E+06		(R)TKTEISEI	(R)T K|T/E/I/S E/M N R(N)		92.53	604.8012	1208.594	0.0012	1	5.81	37296.3	5.3	HUMAN	F8VU64	Keratin, type II cytoskeletal 8
4008	dirMS_041712_NAR_30mM_minus2.16227.16227.2	2	13.94	0	1.63E+05		(K)SSEELLS	(K)S S E/E|L|L|S A L Q/K(K)		141.32	602.816	1204.642	-0.0172	-14.3	4.53	150202.8	9.1	HUMAN	E7EW20	Unconventional myosin-VI
4009	dirMS_041712_NAR_30mM_minus2.8588.8588.2	2	13.94	0	9.67E+04		(R)VGQDP\	(R)V/G/Q/D|P V/L/R(Q)		101.38	442.2532	883.5	-0.0005	-0.5	5.81	14130.9	9.56	HUMAN	C9K0U8	Single-stranded DNA-binding protein (Fragment)
4010	dirMS_041712_NAR_50mM_minus2.9347.9389.3	3	13.94	0	2.64E+05	M167m	(R)ImLFTNI	(R)I/m|L|F/T N E D N/P H/G N D S A K(A)	m:Oxidized methionine	121.37	640.2914	1902.865	15.9946	-0.2	4.54	64302.5	6.4	HUMAN	B1AHC7	X-ray repair complementing defective repair in Chinese hamster cells 6 (Ku autoantigen, 70kDa)
4011	dirMS_041712_NAR_60mM_minus.15456.15579.3	3	13.94	1	4.69E+05		(K)YQLDKD	(K)Y/Q|L D/K/D/G V V/L/F/K(K)		146.33	475.5992	1424.778	0.0047	3.3	5.96	51524.1	4.82	HUMAN	F5H8J2	Protein disulfide-isomerase
4012	dirMS_041712_NAR_60mM_minus.18540.18540.3	3	13.94	1	1.69E+05		(K)VVLDDK	(K)V/V/L/D/D K D/Y F L F R(D)		165.1	510.6099	1529.8	0.0153	10	4.43	5189.9	4.56	HUMAN	B8ZZ54	10 kDa heat shock protein, mitochondrial
4013	dirMS_041712_NAR_20mM_plus.17706.17805.2	2	13.94	0	4.14E+05		(R)ILVATNL	(R)I/L|V|A/T N/L/F/G R(G)		194.48	552.3309	1103.657	-0.0026	-2.4	9.75	40918.8	9.33	HUMAN	B4DP52	HCG2005638, isoform CRA_c
4014	dirMS_041712_NAR_20mM_plus.8387.8387.2	2	13.94	0	8.56E+04		(R)LAPEYE	(R)L A|P/E/Y E/A/A A T R(L)		127.75	596.3035	1191.6	-0.0007	-0.6	4.53	55361.8	6.42	HUMAN	G5EA52	Protein disulfide isomerase family A, member 3, isoform CRA_b
4015	dirMS_041712_NAR_30mM_plus2.12095.12161.2	2	13.94	0	1.13E+05		(K)VWNLAI	(K)V/W|N|L/A/N C K(L)	C:Carbamidomethylation	116.4	502.7542	1004.498	0.0029	2.9	8.72	33029.4	9.62	HUMAN	D6R9L0	Guanine nucleotide-binding protein subunit beta-2-like 1 (Fragment)
4016	dirMS_041712_NAR_30mM_plus2.19888.19888.2	2	13.94	0	2.26E+05		(K)GFAFVQ	(K)G F|A|F/V|Q/Y V/N E R(N)		157.63	665.3326	1329.659	-0.0007	-0.5	6	15791.7	9.22	HUMAN	B4DSU6	Heterogeneous nuclear ribonucleoproteins C1/C2
4017	dirMS_041712_NAR_50mM_plus2.13442.13442.3	3	13.94	0	9.05E+05		(R)TVVTGIE	(R)T V|V/T/G I/E M F H K(S)		138.55	421.225	1261.661	-0.0005	-0.4	6.41	49883	7.3	HUMAN	P49411	Elongation factor Tu, mitochondrial
4018	dirMS_041712_NAR_60mM_plus.7339.7418.3	3	13.94	0	5.01E+05		(R)SSGHSS	(R)S S G H S S S E|L|S|P/D A V E K(A)		96.95	539.584	1616.74	-0.0024	-1.5	4.65	227779.5	12.08	HUMAN	I3L2L6	Serine/arginine repetitive matrix protein 2
4019	dirMS_041712_NAR_60mM_plus.8497.8497.3	3	13.94	0	5.94E+05		(K)GQVCV	(K)G Q|V/C V/M I/H|S G S R(G)	C:Carbamidomethylation	103.07	444.2219	1330.636	0.0157	11.8	9.75	55722.8	6.78	HUMAN	Q9Y3I0	tRNA-splicing ligase RtcB homolog
4020	dirMS_041712_NAR_60mM_plus.9332.9332.3	3	13.94	0	4.66E+05	M89m	(K)VYNYNH	(K)V/Y/N Y/N/H L m/P T R(Y)	m:Oxidized methionine	106.8	475.2309	1407.684	15.9944	-0.4	8.57	16471.9	10.59	HUMAN	K7ELC7	60S ribosomal protein L27 (Fragment)
4021	dirMS_041712_NAR_30mM_minus2.5793.6230.2	2	13.93	0	8.35E+05		(K)YAEAVTI	(K)Y/A|E|A|V/T/R(A)		86.87	405.2098	809.415	-0.0029	-3.5	6	41827.5	7	HUMAN	B7Z7A9	Phosphoglycerate kinase
4022	dirMS_041712_NAR_50mM_minus2.12792.12792.3	3	13.93	1	3.26E+06		(R)TEMENE	(R)T\E|M E N E F/V/L I/K K(D)		141.72	494.2621	1480.772	0.0002	0.1	4.78	13588.1	4.66	HUMAN	F8VP67	Keratin, type II cytoskeletal 8 (Fragment)
4023	dirMS_041712_NAR_60mM_minus.15558.15578.3	3	13.93	0	3.57E+05		(K)THLPGF	(K)T\H\L\P G\F\V|E|Q/A E A L/K(A)		146.42	513.947	1539.817	0.0099	6.4	5.37	22314.2	9.39	HUMAN	P30044	Peroxiredoxin-5, mitochondrial
4024	dirMS_041712_NAR_60mM_minus.17185.17680.3	3	13.93	1	1.28E+07	M148m	(R)LDLAGR	(R)L/D|L|A/G R D L/T/D/Y L/m K(I)	m:Oxidized methionine	157.95	547.2829	1623.841	15.9931	-1.1	4.43	37482.1	5.36	HUMAN	E9PG30	Actin, gamma-enteric smooth muscle
4025	dirMS_041712_NAR_40mM_plus2.10715.10715.3	3	13.93	0	1.85E+05		(K)VGSLDN	(K)V/G|S/L D/N V/G\H L/P A G G A V/K(I)		128.15	530.9567	1590.86	-0.0043	-2.7	6.71	26233.5	10.95	HUMAN	B4DSQ1	Microtubule-associated protein
4026	dirMS_041712_NAR_50mM_plus2.9602.9602.3	3	13.93	1	3.39E+05		(R)AGTGVD	(R)A/G/T/G/V/D/N V/D L E A/A T R\K(G)		117.4	539.6108	1616.824	-0.006	-3.7	4.56	57391.2	6.29	HUMAN	O43175	D-3-phosphoglycerate dehydrogenase
4027	dirMS_041712_NAR_60mM_plus.10817.10817.2	2	13.93	1	9.74E+05		(R)VKYETEI	(R)V/K/Y/E T E L/A/M R(Q)		113.65	620.326	1239.64	0.0045	3.7	6.11	43773.3	5.26	HUMAN	F8VZY9	Keratin, type I cytoskeletal 18
4028	dirMS_041712_NAR_60mM_plus.4780.4780.3	3	13.93	1	1.77E+06		(R)AAEEED	(R)A A\E/E/E/D E/A D/P K/R(Q)		80	453.872	1359.602	-0.0008	-0.6	4.08	11529.7	4.14	HUMAN	P20962	Parathymosin

4029	dirMS_041712_NAR_30mM_minus2.15470.15470.2	2	13.92	0	3.81E+05		(R)AAPFSLE	(R)A A/P/F/S/L E/Y/R(V)		138.3	527.2782	1053.536	0.0128	12.1	6.04	33115.9	5.54	HUMAN	Q15181	Inorganic pyrophosphatase
4030	dirMS_041712_NAR_30mM_minus2.18311.18534.2	2	13.92	0	2.28E+05		(K)YPQLLP(	(K)Y/P|Q/L|L/P G I R(G)		154.92	528.816	1056.62	0.0047	4.4	8.75	45228.3	6.03	HUMAN	F5H737	Adenosylhomocysteinase
4031	dirMS_041712_NAR_30mM_minus2.18572.18572.2	2	13.92	0	3.09E+05		(K)YPQLLP(	(K)Y/P/Q/L|L/P G I/R(G)		155.32	528.816	1056.62	0.0047	4.4	8.75	45228.3	6.03	HUMAN	F5H737	Adenosylhomocysteinase
4032	dirMS_041712_NAR_30mM_minus2.5205.5407.2	2	13.92	0	7.07E+05		(R)TDTGEP	(R)T/D|T|G|E P M/G R(G)		82.22	482.2145	963.42	0.0017	1.8	4.37	85057.6	4.94	HUMAN	P07900	Heat shock protein HSP 90-alpha
4033	dirMS_041712_NAR_40mM_minus2.14853.14853.3	3	13.92	1	1.13E+06	M116m	(R)ELAILLG	(R)E L A|I/L L/G m L D/P A E K D E K(G)	m:Oxidized methionine	171.42	634.338	1884.999	16.0008	3.1	4.18	25148.6	6	HUMAN	P30041	Peroxiredoxin-6
4034	dirMS_041712_NAR_60mM_minus.14281.14281.3	3	13.92	0	4.62E+05		(R)SDSFENI	(R)S D|S/F/E N/P V/L/Q Q H F/R(N)		139.27	568.6086	1703.814	-0.0024	-1.4	5.3	64302.5	6.4	HUMAN	B1AHC7	X-ray repair complementing defective repair in Chinese hamster cells 6 (Ku autoantigen, 70kDa)
4035	dirMS_041712_NAR_20mM_plus.7638.7837.2	2	13.92	0	6.94E+05		(K)VIDDTNI	(K)V/I|D|D/T N I/T/R(L)		123.18	523.7766	1046.548	-0.0017	-1.7	4.21	43773.3	5.26	HUMAN	F8VZY9	Keratin, type I cytoskeletal 18
4036	dirMS_041712_NAR_50mM_plus2.20866.20866.3	3	13.92	1	7.61E+04		(K)TSAQVV	(K)T S|A Q/V/V/L D G L V D K I G D/V/K(C)		178.37	619.6814	1857.033	-0.0031	-1.7	4.43	227202.2	8.26	HUMAN	Q14008	Cytoskeleton-associated protein 5
4037	dirMS_041712_NAR_60mM_plus.7240.7411.2	2	13.92	1	1.58E+06		(R)AAPGAE	(R)A/A/P/G A|E|F/A\P N K/R(R)		96.2	614.8248	1228.643	-0.001	-0.8	8.79	43979.5	8.92	HUMAN	F5GYZ3	Non-POU domain-containing octamer-binding protein
4038	dirMS_041712_NAR_60mM_plus.9429.9429.2	2	13.92	0	1.98E+06		(R)IGAEVYI	(R)I/G\A/E/V|Y/H/N L K(N)		107.55	572.3143	1143.616	0.0056	4.9	6.75	21077	9.05	HUMAN	K7EM90	Alpha-enolase (Fragment)
4039	dirMS_041712_NAR_30mM_minus2.11407.11407.2	2	13.91	0	1.21E+05	M39m	(K)mFLYAD	(K)m F|L/Y/A/D/N E D/R(A)	m:Oxidized methionine	115.58	645.2795	1273.552	16	3.9	4.03	24724.1	5.17	HUMAN	E7ETQ7	Vacuolar protein sorting-associated protein VTA1 homolog
4040	dirMS_041712_NAR_30mM_minus2.12151.12180.2	2	13.91	0	3.38E+05		(K)ILYSQCG	(K)I L|Y/S/Q/C G D V M/R(A)	C:Carbamidomethylation	119.22	671.3183	1341.629	0.0004	0.3	5.83	26991.7	5.01	HUMAN	B7Z6Z4	Myosin light polypeptide 6
4041	dirMS_041712_NAR_30mM_minus2.14104.14455.2	2	13.91	0	1.06E+06		(R)FLDPNTI	(R)F/L|D|P/N T L E/R(L)		131.2	552.7932	1104.568	0.0107	9.7	4.37	266161	5.68	HUMAN	E9PPU0	Epiplakin
4042	dirMS_041712_NAR_50mM_minus2.8660.8660.3	3	13.91	0	1.65E+05	M180m	(R)VVVLm(	(R)V V|V/L/m/G S T S D L G H/C E K(I)	C:Carbamidomethylation m:Oxid	117.55	583.2829	1731.84	15.9937	-0.7	5.32	37681.5	5.93	HUMAN	D6RF62	Phosphoribosylaminoimidazole carboxylase
4043	dirMS_041712_NAR_60mM_minus.11568.11568.3	3	13.91	1	2.94E+05		(R)VFDKEG	(R)V F/D K E G|N G T|V|M/G A/E I R(H)		124.35	574.9539	1722.848	-0.0008	-0.5	4.68	26991.7	5.01	HUMAN	B7Z6Z4	Myosin light polypeptide 6
4044	dirMS_041712_NAR_120mM_plus.10177.10435.3	3	13.91	1	1.33E+06		(R)YLAEFAT	(R)Y/L|A E/F/A/T/G N D R K(E)		107.65	462.2337	1384.686	0.001	0.7	6.07	12926.1	4.4	HUMAN	K7EM20	14-3-3 protein epsilon (Fragment)
4045	dirMS_041712_NAR_120mM_plus.10839.10881.3	3	13.91	1	4.58E+05		(R)RFDDAV	(R)R\F D D\A\V|V|Q/S D/M/K(H)		110.08	470.8945	1410.668	0.0008	0.5	4.43	20069.4	6.74	HUMAN	E9PK54	Heat shock cognate 71 kDa protein (Fragment)
4046	dirMS_041712_NAR_120mM_plus.8455.8476.4	4	13.91	0	3.85E+05		(K)LLETTDR	(K)L/L/E/T/T/D R P D G H Q/N N L/R(S)		97.58	470.4895	1878.942	-0.0055	-2.9	5.38	82375.7	4.6	HUMAN	B7ZAV6	Importin subunit beta-1
4047	dirMS_041712_NAR_120mM_plus.8614.8786.3	3	13.91	0	1.32E+06		(R)RPELEDS	(R)R P\E|L|E|D/S/T/L/R(Y)		98.35	405.8796	1215.633	-0.0085	-7	4.68	81007.1	6.31	HUMAN	E9PMV1	Plectin (Fragment)
4048	dirMS_041712_NAR_20mM_plus.16643.16643.3	3	13.91	0	2.14E+05		(K)VQIAVA	(K)V Q\I A V\A N\A|Q/E/L/L/Q R(M)		186.3	518.2975	1552.881	-0.0026	-1.7	5.97	10728.1	9.15	HUMAN	Q9Y5L4	Mitochondrial import inner membrane translocase subunit Tim13
4049	dirMS_041712_NAR_20mM_plus.18407.18407.2	2	13.91	0	1.95E+05		(R)AAVEEG	(R)A A V E\E/G/I|V|L\G|G G C A L/L R(C)	C:Carbamidomethylation	197.9	842.9549	1684.905	-0.0025	-1.5	4.53	61224.7	5.7	HUMAN	P10809	60 kDa heat shock protein, mitochondrial
4050	dirMS_041712_NAR_30mM_plus2.7258.7258.2	2	13.91	0	6.90E+04		(R)SQLQISN	(R)S Q|L|Q|I/S|N/N R(T)		92.6	530.2783	1059.554	-0.0048	-4.6	9.47	334221.3	6.44	HUMAN	P15924	Desmoplakin
4051	dirMS_041712_NAR_40mM_plus2.13445.13465.3	3	13.91	1	3.11E+05		(K)VIDPATA	(K)V/I|D|P A/T A/T\S V D L R D/I/K(I)		145.75	571.9836	1713.938	-0.0019	-1.1	4.43	42755	7.81	HUMAN	B7Z2F4	T-complex protein 1 subunit delta
4052	dirMS_041712_NAR_50mM_plus2.16374.16374.3	3	13.91	0	2.89E+05		(K)ACQSIYF	(K)A C|Q/S\I/Y/P L/H D/V F V R(K)	C:Carbamidomethylation	154.4	568.9562	1704.853	0.0014	0.8	6.79	16650	9.47	HUMAN	D6R9B6	40S ribosomal protein S3a
4053	dirMS_041712_NAR_20mM_minus.13276.13276.2	2	13.9	0	2.34E+05		(R)GVVDSE	(R)G V/V D S\E/D/I|P L N|L/S R(E)		183.02	757.3944	1513.786	-0.0041	-2.7	4.03	74494.3	7.79	HUMAN	F5H897	Heat shock protein 75 kDa, mitochondrial
4054	dirMS_041712_NAR_30mM_minus2.10239.10413.2	2	13.9	0	1.06E+06		(K)DVGSLA	(K)D V|G\S|L/A|S V Q|R(Y)		109.78	516.281	1031.548	0.0067	6.5	5.84	266161	5.68	HUMAN	E9PPU0	Epiplakin
4055	dirMS_041712_NAR_30mM_minus2.15664.15664.2	2	13.9	0	2.70E+04	M1342m	(R)ASLSLW	(R)A S/L|S|L\W Q A/m/E K(G)	m:Oxidized methionine	138.55	640.3185	1263.64	15.9895	-4.2	6.05	266161	5.68	HUMAN	E9PPU0	Epiplakin
4056	dirMS_041712_NAR_30mM_minus2.8013.8131.2	2	13.9	0	2.96E+05		(R)FLNAEN.	(R)F/L|N|A|E N/A|Q K(F)		98.62	517.7675	1034.526	0.0012	1.1	6	20592.3	9.75	HUMAN	B3KUP2	RAN binding protein 1, isoform CRA_f
4057	dirMS_041712_NAR_50mM_minus2.15448.15448.3	3	13.9	1	1.73E+05		(K)DVLFLKD	(K)D V/L|F/L K D C V/G P\E V/E K(A)	C:Carbamidomethylation	156.42	583.3014	1747.893	-0.0039	-2.2	4.32	41827.5	7	HUMAN	B7Z7A9	Phosphoglycerate kinase
4058	dirMS_041712_NAR_60mM_minus.9590.9671.3	3	13.9	1	2.76E+06	M239m	(R)MQKEIT	(R)M Q/K E I T A|L|A/P/S T m K(I)	m:Oxidized methionine	114.28	522.2733	1548.812	15.9929	-1.3	8.35	32616.5	5.18	HUMAN	A6NL76	Actin, alpha skeletal muscle
4059	dirMS_041712_NAR_120mM_plus.11648.11659.3	3	13.9	0	5.02E+05		(R)HSSLAG(	(R)H S\S L\A G\C|Q|I|I/N Y R(T)	C:Carbamidomethylation	114.17	506.9192	1518.748	-0.0051	-3.4	8.75	193379.8	5.48	HUMAN	Q00610	Clathrin heavy chain 1
4060	dirMS_041712_NAR_30mM_plus2.16699.16699.2	2	13.9	0	9.86E+05		(R)AALQELI	(R)A A/L/Q/E L/L/S K(G)		139.35	486.7894	972.572	-0.0009	-0.9	6.05	13798.9	10.17	HUMAN	P62851	40S ribosomal protein S25
4061	dirMS_041712_NAR_30mM_plus2.18556.18556.2	2	13.9	0	3.64E+05		(R)SSFYVN(	(R)S S/F\Y/V|N|G L T\L G/G Q/K(C)		150.97	735.8825	1470.759	-0.001	-0.7	8.31	17630.6	11.62	HUMAN	I3L3D5	Profilin-1 (Fragment)
4062	dirMS_041712_NAR_30mM_plus2.21112.21112.2	2	13.9	0	6.77E+05		(K)IPDWFLI	(K)I/P/D/W/F L/N R(Q)		165.77	530.785	1060.557	0.0053	5	5.84	9767.1	11.41	HUMAN	J3JS69	40S ribosomal protein S18
4063	dirMS_041712_NAR_30mM_plus2.6226.6226.2	2	13.9	0	5.49E+05		(K)IVEMSTS	(K)I/V|E/M/S T/S K(T)		87.1	447.7285	894.46	-0.0104	-11.6	6	13048.7	6.42	HUMAN	C9J4W5	Eukaryotic translation initiation factor 5A-2 (Fragment)
4064	dirMS_041712_NAR_30mM_plus2.9082.9082.2	2	13.9	0	2.75E+05	M406m	(R)EYQELm	(R)E Y/Q/E/L/m/N V/K(L)	m:Oxidized methionine	101.23	585.2792	1153.556	15.9953	0.4	4.53	56607.4	5.37	HUMAN	F8VXB4	Keratin, type II cytoskeletal 8
4065	dirMS_041712_NAR_40mM_plus2.7594.7594.2	2	13.9	0	6.02E+05		(K)ASLENSI	(K)A/S/L/E/N S L R(E)		105.33	445.2395	889.474	-0.002	-2.3	6.05	43773.3	5.26	HUMAN	F8VZY9	Keratin, type I cytoskeletal 18
4066	dirMS_041712_NAR_50mM_plus2.10708.10708.3	3	13.9	0	4.45E+05		(K)MIVDPV	(K)M I|V/D/P/V E/P H/G E M K(F)		123.4	494.5783	1481.713	0.0076	5.2	4.65	90316.8	5.52	HUMAN	Q13263	Transcription intermediary factor 1-beta
4067	dirMS_041712_NAR_50mM_plus2.11679.11679.3	3	13.9	0	3.41E+05		(K)SNMGH	(K)S N/M G H P E/P A/S/G/L A A/L A K(V)		128.55	550.9462	1650.827	-0.0028	-1.7	6.47	276046.1	6.01	HUMAN	P49327	Fatty acid synthase
4068	dirMS_041712_NAR_50mM_plus2.16381.16394.3	3	13.9	0	1.45E+06		(K)IEVIKPG	(K)I/E|V/I/K/P G D L/G V D L T S K(L)		154.62	561.9883	1683.953	-0.0024	-1.4	4.56	28071.3	8.6	HUMAN	P38117	Electron transfer flavoprotein subunit beta
4069	dirMS_041712_NAR_60mM_plus.12847.12910.3	3	13.9	0	3.95E+06		(R)ITLRPDP	(R)I/T|L R P D|P\L/P E/R(S)		123.53	436.2506	1306.748	-0.0105	-8	6.07	160853.1	6.11	HUMAN	F8VU51	YLP motif-containing protein 1
4070	dirMS_041712_NAR_60mM_plus.5129.5129.3	3	13.9	0	2.14E+04		(K)EILEGPS	(K)E I|L E G/P S P/E W/E T K(A)		85.37	505.5852	1514.737	0.0037	2.5	4.09	89503.2	9.38	HUMAN	Q9HBT7	Zinc finger protein 287
4071	dirMS_041712_NAR_60mM_minus.11151.11151.2	2	13.89	1	2.01E+05		(R)DGKLVS	(R)D G K/L|V|S E S S D/V L\P K(-)		122.23	737.3855	1473.78	-0.0158	-10.7	4.56	56607.4	5.37	HUMAN	F8VXB4	Keratin, type II cytoskeletal 8
4072	dirMS_041712_NAR_20mM_plus.15084.15126.2	2	13.89	0	2.17E+05		(K)GLAITFV	(K)G L A\I|T|F/V|S/D E N D A K(I)		175.3	740.3693	1479.733	-0.0012	-0.8	4.03	40918.8	9.33	HUMAN	B4DP52	HCG2005638, isoform CRA_c
4073	dirMS_041712_NAR_20mM_plus.8860.9097.2	2	13.89	0	1.23E+06	M239m	(K)EITALAP	(K)E I|T|A|L|A|P S T m K(I)	m:Oxidized methionine	132.6	589.309	1161.618	15.9923	-2.2	6.1	32616.5	5.18	HUMAN	A6NL76	Actin, alpha skeletal muscle
4074	dirMS_041712_NAR_40mM_plus2.19346.19346.3	3	13.89	0	1.61E+06		(R)AGKPVI(	(R)A G K P V\I\C A\T Q|M/L/E S M I K(K)	C:Carbamidomethylation	180.63	626.3273	1876.969	-0.002	-1.1	8.64	18466.1	5.78	HUMAN	H3BSU3	Pyruvate kinase (Fragment)
4075	dirMS_041712_NAR_60mM_plus.12728.12729.3	3	13.89	1	1.27E+06		(R)MLFKDE	(R)M/L|F K/D D Y|P/S S/P P K(C)		123.65	508.9175	1524.74	-0.0023	-1.5	5.71	20685.4	9.18	HUMAN	B0QYN7	SUMO-conjugating enzyme UBC9
4076	dirMS_041712_NAR_60mM_plus.7458.7458.3	3	13.89	1	9.72E+05		(K)GGETIK(	(K)G G|E|T/I/K Q L/Q/E R(A)		97.52	420.23	1258.675	0.0005	0.4	6.14	70034.8	6.89	HUMAN	B4DT31	Far upstream element-binding protein 1
4077	dirMS_041712_NAR_30mM_minus2.13393.13571.2	2	13.88	0	5.79E+05		(R)TIAQDY(	(R)T I|A|Q/D/Y/G\V/L K(A)		126.9	554.3051	1107.604	-0.0015	-1.4	5.5	22338.2	8.65	HUMAN	Q06830	Peroxiredoxin-1
4078	dirMS_041712_NAR_30mM_minus2.14353.14353.2	2	13.88	0	1.99E+05		(R)ELAFQIS	(R)E L/A/F/Q/I/S/K(E)		131.7	468.2614	935.52	-0.0041	-4.4	6.1	30944.2	6.52	HUMAN	B1Q2N1	ATP-dependent RNA helicase DDX39A
4079	dirMS_041712_NAR_30mM_minus2.6957.7297.2	2	13.88	0	9.72E+05		(K)IVEMSTS	(K)I/V|E/M/S/T/S/K(T)		93.58	447.7317	894.46	-0.004	-4.4	6	13048.7	6.42	HUMAN	C9J4W5	Eukaryotic translation initiation factor 5A-2 (Fragment)
4080	dirMS_041712_NAR_50mM_minus2.12368.12368.3	3	13.88	0	2.32E+05		(K)AASDIAI	(K)A A/S D\I|A/M/T/E L/P P T H P I R(L)		138.97	607.3158	1819.937	-0.0042	-2.3	5.32	12926.1	4.4	HUMAN	K7EM20	14-3-3 protein epsilon (Fragment)
4081	dirMS_041712_NAR_120mM_plus.7142.7206.3	3	13.88	1	1.11E+06		(R)SDYDGI(	(R)S/D|Y/D/G I/G S R/G D R(S)		91.03	433.1967	1297.577	-0.0012	-0.9	4.43	15031.5	6.72	HUMAN	B4DLA0	ATP-dependent RNA helicase DDX3X
4082	dirMS_041712_NAR_20mM_plus.12395.12395.2	2	13.88	0	1.87E+05		(R)GVEICIA	(R)G V|E/I/C/I/A\T/P G R(L)	C:Carbamidomethylation	158.58	586.8084	1172.609	0.0003	0.3	6	61018.6	9.44	HUMAN	B4DLW8	Probable ATP-dependent RNA helicase DDX5
4083	dirMS_041712_NAR_20mM_plus.13430.13683.2	2	13.88	0	2.13E+05	M98m	(R)TLTIVDT	(R)T L|T\I|V/D|T G I|G m T K(A)	m:Oxidized methionine	166.47	683.3671	1349.735	15.9924	-1.8	5.5	20151.2	4.58	HUMAN	G3V2J8	Heat shock protein HSP 90-alpha (Fragment)
4084	dirMS_041712_NAR_20mM_plus.17966.17966.2	2	13.88	0	1.95E+05		(K)GADFLV	(K)G A D/F L|V|T\E/V|E N G G S L/G S\K(K)		195.22	890.4414	1779.876	-0.0004	-0.2	4.14	30947.9	6.47	HUMAN	H3BQ34	Pyruvate kinase
4085	dirMS_041712_NAR_40mM_plus2.11210.11210.3	3	13.88	0	1.02E+05		(R)VLVASS\	(R)V/L|V/A/S/S V V A G E S Q K/P E L K(A)		132.05	614.3443	1841.038	-0.0195	-10.6	6.11	61441.6	5.93	HUMAN	G8JLE9	Fragile X mental retardation protein 1
4086	dirMS_041712_NAR_50mM_plus2.15329.15329.3	3	13.88	0	9.75E+05		(R)QVLIASH	(R)Q V\L/I/A/S H/L/P S Y E L/R(H)		149.48	542.6394	1625.901	0.0027	1.6	6.75	267260.7	5.95	HUMAN	Q13085	Acetyl-CoA carboxylase 1
4087	dirMS_041712_NAR_60mM_plus.7404.7404.3	3	13.88	0	4.28E+05		(K)ITPAHD(	(K)I/T/P A H D Q/N D/Y/E V/G Q R(H)		96.47	581.6074	1742.809	-0.0016	-0.9	4.54	141718.6	7.8	HUMAN	B0V043	Valine--tRNA ligase
4088	dirMS_041712_NAR_60mM_plus.8216.8216.3	3	13.88	1	8.11E+05		(R)FEPYANI	(R)F E|P/Y/A N/P T K R(Y)		101.27	408.2176	1222.622	0.0167	13.7	8.59	77799.7	9.09	HUMAN	P52272	Heterogeneous nuclear ribonucleoprotein M
4089	dirMS_041712_NAR_20mM_minus.10614.10680.2	2	13.87	0	2.00E+05		(R)TTPSYV/	(R)T/T|P S Y/V/A|F T D/T E R(L)		154.63	744.3547	1487.701	0.0009	0.6	4.37	58502.6	6.09	HUMAN	B4DXY3	Heat shock 70 kDa protein 1-like
4090	dirMS_041712_NAR_40mM_minus2.16109.16109.3	3	13.87	1	6.62E+05		(R)ELAILLG	(R)E L/A/I/L/L\G M/L D/P A E K D E K(G)		189.6	629.0034	1884.999	-0.003	-1.6	4.18	25148.6	6	HUMAN	P30041	Peroxiredoxin-6
4091	dirMS_041712_NAR_50mM_minus2.14733.14733.3	3	13.87	1	1.82E+05		(K)ELKIDIIP	(K)E L K I D I\I|P/N/P Q E R(T)		151.52	522.2945	1564.869	-0.0004	-0.2	4.68	83605	4.97	HUMAN	P08238	Heat shock protein HSP 90-beta
4092	dirMS_041712_NAR_60mM_minus.9322.9322.3	3	13.87	1	2.37E+05		(R)QDSAAV	(R)Q D S A/A/V/G/F D/Y K E K(L)		111.87	486.5639	1457.691	-0.0136	-9.3	4.56	61756.2	5.24	HUMAN	Q14247	Src substrate cortactin
4093	dirMS_041712_NAR_20mM_plus.16231.16231.3	3	13.87	0	1.32E+05		(R)LLDAQL/	(R)L L D/A\Q L A T G/G|I/V/D/P R(L)		183.53	513.6218	1538.854	-0.0028	-1.8	4.21	533778	5.74	HUMAN	Q15149	Plectin
4094	dirMS_041712_NAR_20mM_plus.17964.17964.2	2	13.87	0	1.96E+05		(K)VANVSL	(K)V A/N/V|S/L/L/A/L Y K(G)		195.33	595.8558	1190.714	-0.01	-8.4	8.56	15978.4	10.84	HUMAN	P62266	40S ribosomal protein S23
4095	dirMS_041712_NAR_30mM_plus2.8419.8419.2	2	13.87	0	1.01E+06		(R)ALANSL/	(R)A L|A/N/S/L A/C Q G/K(Y)	C:Carbamidomethylation	97.78	566.7922	1132.578	-0.0008	-0.7	8.8	13951.6	9.48	HUMAN	H3BR68	Fructose-bisphosphate aldolase A (Fragment)
4096	dirMS_041712_NAR_30mM_plus2.9055.9055.2	2	13.87	0	5.15E+05		(R)LATNTS/	(R)L/A|T N/T/S A P D L/K(N)		100.7	565.801	1130.605	-0.0105	-9.2	5.84	26233.5	10.95	HUMAN	B4DSQ1	Microtubule-associated protein
4097	dirMS_041712_NAR_40mM_plus2.14922.14922.3	3	13.87	0	3.03E+05		(R)IEGDMI\	(R)I/E|G D M I V C/A A/Y/A H E L/P K(Y)	C:Carbamidomethylation	153.93	639.6498	1916.925	0.0104	5.4	4.65	34590.2	9.84	HUMAN	P46777	60S ribosomal protein L5
4098	dirMS_041712_NAR_50mM_plus2.6147.6147.3	3	13.87	0	1.25E+05		(R)SGSMDF	(R)S/G|S M/D/P/S/G A H P S V R(Q)		96.5	462.214	1384.627	0.0001	0	6.46	48340.6	8.84	HUMAN	Q07666	KH domain-containing, RNA-binding, signal transduction-associated protein 1
4099	dirMS_041712_NAR_20mM_minus.10086.10086.2	2	13.86	0	1.24E+05		(R)YALYDA'	(R)Y A|L|Y/D A T Y E T K(E)		150.82	669.3156	1337.626	-0.0021	-1.5	4.37	22956	8.8	HUMAN	E9PK25	Cofilin-1
4100	dirMS_041712_NAR_50mM_minus2.14942.15170.3	3	13.86	0	4.69E+05		(K)IAILTCPF	(K)I/A|I|L/T/C/P F E/P P K/P K(T)	C:Carbamidomethylation	153.5	537.6375	1610.898	0.0005	0.3	8.59	49982	5.86	HUMAN	B4DYD8	T-complex protein 1 subunit epsilon
4101	dirMS_041712_NAR_120mM_plus.13623.13926.3	3	13.86	0	1.60E+06		(R)NLDIERF	(R)N/L|D|I/E R/P T Y/T/N L N R(L)		125.65	573.6317	1718.882	-0.0015	-0.9	6.07	49012.3	4.9	HUMAN	A8MUB1	Tubulin alpha-4A chain
4102	dirMS_041712_NAR_20mM_plus.12993.12993.2	2	13.86	0	2.33E+04		(K)AVDSLVI	(K)A V/D/S/L/V/P I G R(G)		162.43	513.8007	1026.594	-0.0001	-0.1	5.88	48851.3	9.2	HUMAN	K7EK63	ATP synthase subunit alpha, mitochondrial
4103	dirMS_041712_NAR_20mM_plus.13796.13796.2	2	13.86	0	4.19E+04		(K)IVLTNPV	(K)I V|L T|N/P V C T E V G E/K(I)	C:Carbamidomethylation	167.07	779.9082	1558.815	-0.0054	-3.5	4.53	51679.1	9.11	HUMAN	P41091	Eukaryotic translation initiation factor 2 subunit 3
4104	dirMS_041712_NAR_20mM_plus.15836.15836.2	2	13.86	0	7.36E+05	M73m	(R)AILVDLE	(R)A I L/V/D L|E|P G T m D\S V R(S)	m:Oxidized methionine	179.55	816.4181	1615.836	15.9929	-1.2	4.03	27549.9	4.39	HUMAN	E9PBJ4	Tubulin beta chain
4105	dirMS_041712_NAR_30mM_plus2.19241.19241.2	2	13.86	0	1.73E+05		(K)YPQLLP(	(K)Y/P/Q/L/L/P G I R(G)		153.33	528.814	1056.62	0.0007	0.6	8.75	45228.3	6.03	HUMAN	F5H737	Adenosylhomocysteinase
4106	dirMS_041712_NAR_30mM_plus2.20483.20483.2	2	13.86	0	1.76E+05		(K)GSFSEQ(	(K)G S/F/S|E/Q/G I/N E/F\L/R(E)		161.02	742.362	1483.718	-0.0009	-0.6	4.53	53659.5	5.35	HUMAN	B5MCQ5	Protein disulfide-isomerase A6
4107	dirMS_041712_NAR_40mM_plus2.17987.17987.3	3	13.86	0	2.83E+05		(K)LLYDLAD	(K)L L|Y/D/L A D Q/L/H A A V\G A S R(A)		171.38	604.9967	1812.96	0.0153	8.4	5.21	24273.5	8.98	HUMAN	H0YK49	Electron transfer flavoprotein subunit alpha, mitochondrial
4108	dirMS_041712_NAR_50mM_plus2.5148.5154.3	3	13.86	0	7.90E+04		(R)AEAEAG	(R)A E|A|E/A/G S P R/P D|P/R(E)		85.77	451.5555	1352.655	-0.0034	-2.5	4.68	558007.1	5.44	HUMAN	P58107	Epiplakin
4109	dirMS_041712_NAR_60mM_plus.10963.10963.3	3	13.86	1	2.14E+06		(R)KLDPGSI	(R)K\L D P G S|E E|T/Q|T/L/V R(E)		114.92	524.9456	1572.823	-0.0005	-0.3	4.68	56491.1	7.78	HUMAN	B4DTG2	Elongation factor 1-gamma
4110	dirMS_041712_NAR_20mM_minus.10994.11296.2	2	13.85	0	3.73E+06	M148m	(R)DLTDYLr	(R)D L|T|D|Y/L/m/K(I)	m:Oxidized methionine	161.35	507.7443	998.486	15.995	0.1	4.21	37482.1	5.36	HUMAN	E9PG30	Actin, gamma-enteric smooth muscle
4111	dirMS_041712_NAR_20mM_minus.14722.14890.2	2	13.85	0	3.01E+06		(K)EIIDLVLI	(K)E I|I/D/L/V/L/D/R(I)		203.67	543.3133	1085.62	-0.0008	-0.7	4.03	58642	4.95	HUMAN	F5H5D3	Tubulin alpha-1C chain
4112	dirMS_041712_NAR_40mM_minus2.8291.8291.3	3	13.85	0	2.03E+05	M35m	(K)AASDIAr	(K)A A|S D I/A m T E L/P P T/H P I R(L)	m:Oxidized methionine	128.85	612.6484	1819.937	15.9936	-0.7	5.32	12926.1	4.4	HUMAN	K7EM20	14-3-3 protein epsilon (Fragment)
4113	dirMS_041712_NAR_50mM_minus2.7412.7412.3	3	13.85	0	1.42E+04		(K)QEYDES(	(K)Q E/Y/D E/S G/P/S I/V H/R(K)		109.17	506.2404	1516.703	0.004	2.6	4.65	123097.1	5.83	HUMAN	A5A3E0	POTE ankyrin domain family member F
4114	dirMS_041712_NAR_60mM_minus.14870.14870.3	3	13.85	1	3.80E+05		(R)IKIGDPL	(R)I K/I G D/P\L/L/E/D/T R(M)		142.73	457.2595	1369.769	-0.0046	-3.4	4.56	47309.6	5.42	HUMAN	B4DXY7	4-trimethylaminobutyraldehyde dehydrogenase
4115	dirMS_041712_NAR_60mM_minus.6327.6327.3	3	13.85	1	3.26E+04		(K)LEEAEK/	(K)L E/E A E/K/A/A/D E/S E R(G)		93.93	492.8981	1476.681	-0.0015	-1	4.14	31923.6	4.85	HUMAN	B7Z596	Tropomyosin alpha-1 chain
4116	dirMS_041712_NAR_60mM_minus.9099.9193.3	3	13.85	1	1.02E+06		(R)ETAEAD'	(R)E/T/A/E/A D/V/A/S L N R R(I)		111.37	477.9107	1431.719	-0.0011	-0.8	4.68	31923.6	4.85	HUMAN	B7Z596	Tropomyosin alpha-1 chain
4117	dirMS_041712_NAR_120mM_plus.20351.20351.3	3	13.85	0	3.29E+05		(R)INLWHL	(R)I/N/L/W/H/L E I T D/R(S)		159.47	470.5889	1409.754	-0.0014	-1	5.32	52204.6	5.82	HUMAN	P63151	Serine/threonine-protein phosphatase 2A 55 kDa regulatory subunit B alpha isoform
4118	dirMS_041712_NAR_20mM_plus.14017.14017.2	2	13.85	0	9.86E+04	M313m	(R)TVQSLEI	(R)T V/Q/S/L/E|I D L D S m R(N)	m:Oxidized methionine	168.37	761.8738	1506.747	15.9935	-0.9	4.03	43773.3	5.26	HUMAN	F8VZY9	Keratin, type I cytoskeletal 18
4119	dirMS_041712_NAR_20mM_plus.17169.17190.2	2	13.85	0	9.08E+05		(R)VLITTDL	(R)V/L|I/T/T/D L L/A/R(G)		190.55	557.8442	1114.683	-0.0019	-1.7	5.81	41517.9	5.45	HUMAN	E7EQG2	Eukaryotic initiation factor 4A-II
4120	dirMS_041712_NAR_50mM_plus2.16977.16977.3	3	13.85	1	4.54E+05		(K)VLDSGA	(K)V/L|D/S G A/P I\K I/P V G P/E/T L G R(I)		156.95	640.3623	1919.096	-0.0237	-12.3	6.04	55307.5	5.25	HUMAN	F8VPV9	ATP synthase subunit beta
4121	dirMS_041712_NAR_50mM_plus2.21413.21413.3	3	13.85	0	4.74E+06		(R)LHFFMP	(R)L H F\F\M\P G\F|A|P\L T S R(G)		182.07	540.9498	1620.835	-0.0007	-0.4	9.75	42230.8	4.77	HUMAN	A6NKZ8	Putative tubulin beta chain-like protein ENSP00000290377
4122	dirMS_041712_NAR_60mM_plus.9175.9311.3	3	13.85	1	1.59E+06		(R)SRAEAE!	(R)S R A E\A\E|S|M/Y/Q/I/K(Y)		105.9	471.5691	1412.684	0.0089	6.3	5.86	37296.3	5.3	HUMAN	F8VU64	Keratin, type II cytoskeletal 8
4123	dirMS_041712_NAR_30mM_minus2.16116.16116.2	2	13.84	1	1.52E+05		(R)FVLSQAI	(R)F V|L|S/Q A K/D E\L(-)		141.4	575.3113	1149.615	0.0003	0.3	4.37	41053.3	5.58	HUMAN	Q86UY0	TXNDC5 protein
4124	dirMS_041712_NAR_40mM_minus2.14882.14911.3	3	13.84	1	5.46E+05		(R)GDLGIEI	(R)G D/L\G|I|E/I/P A E K V F L A Q K(M)		173.02	610.0109	1828.022	-0.0033	-1.8	4.68	18466.1	5.78	HUMAN	H3BSU3	Pyruvate kinase (Fragment)
4125	dirMS_041712_NAR_60mM_minus.8224.8224.3	3	13.84	0	1.91E+05		(R)QYDISDI	(R)Q Y/D/I/S D/P Q/R/P R(L)		105.63	458.8971	1374.676	0.0007	0.5	5.96	45924.5	5.29	HUMAN	B4E1F3	Selenium-binding protein 1
4126	dirMS_041712_NAR_30mM_plus2.13565.13688.2	2	13.84	0	2.41E+06		(K)LIGEYGL	(K)L/I|G/E/Y/G L/R(N)		124.88	460.7596	920.52	-0.0081	-8.8	6	9591.1	10.94	HUMAN	A8MXK4	40S ribosomal protein S9
4127	dirMS_041712_NAR_50mM_plus2.9827.9827.3	3	13.84	1	2.02E+05		(R)AESGPD	(R)A E|S G/P D L R\Y E V/T|S/G G G/G T S R(M)		119.13	665.9843	1995.937	0.0017	0.9	4.68	334221.3	6.44	HUMAN	P15924	Desmoplakin
4128	dirMS_041712_NAR_60mM_plus.6958.6958.3	3	13.84	1	1.77E+05		(K)VQVYQE	(K)V/Q|V/Y Q E/P N/R E/T/R(V)		94.82	506.9252	1518.766	-0.0049	-3.2	6.11	35946.5	9.41	HUMAN	B7ZKZ1	Phosphoribosyl pyrophosphate synthase-associated protein 2
4129	dirMS_041712_NAR_50mM_minus2.12544.12690.3	3	13.83	0	4.05E+05		(R)SLATTRF	(R)S L|A|T|T/R P T V/N A D D|L L/K(V)		140.2	572.3135	1714.933	-0.0074	-4.3	5.68	49182.3	7.8	HUMAN	Q9UN37	Vacuolar protein sorting-associated protein 4A
4130	dirMS_041712_NAR_50mM_minus2.15135.15135.3	3	13.83	0	2.95E+05		(R)DGLAFN	(R)D G L A/F/N/A/L/I H R(H)		155.02	409.5594	1226.664	-0.0004	-0.3	6.74	146974	5.55	HUMAN	C9JRP8	Spectrin beta chain, non-erythrocytic 4
4131	dirMS_041712_NAR_120mM_plus.6459.6621.2	2	13.83	0	4.57E+05		(K)HQPTAII	(K)H/Q|P T A I\I|A/K(T)		87.75	489.7897	978.573	-0.001	-1	8.76	63448.4	7.29	HUMAN	B4E022	Transketolase
4132	dirMS_041712_NAR_30mM_plus2.12982.13118.2	2	13.83	0	6.50E+05		(K)GDYPLE/	(K)G D|Y/P/L/E/A V/R(M)		121.92	510.2633	1019.516	0.0037	3.6	4.37	18466.1	5.78	HUMAN	H3BSU3	Pyruvate kinase (Fragment)
4133	dirMS_041712_NAR_50mM_plus2.10328.10328.3	3	13.83	1	2.19E+05		(R)TGLIKGS	(R)T|G\L|I K/G S G T A/E V/E/L K(K)		121.85	501.6143	1502.842	-0.0141	-9.4	5.81	30947.9	6.47	HUMAN	H3BQ34	Pyruvate kinase
4134	dirMS_041712_NAR_50mM_plus2.10640.10640.3	3	13.83	0	3.46E+05		(R)AIGSASE	(R)A I|G/S A/S E G A Q S S L Q E V Y/H K(S)		123.47	654.657	1961.956	0.0001	0.1	5.4	20148.5	4.65	HUMAN	B4E2V4	Proteasome (Prosome, macropain) subunit, alpha type, 5, isoform CRA_c
4135	dirMS_041712_NAR_50mM_plus2.7973.8205.3	3	13.83	0	2.70E+05		(K)STAGDT	(K)S T|A/G D/T/H/L/G G E D F D/N R(M)		109.7	564.5793	1691.726	-0.0022	-1.3	4.22	69032.8	5.37	HUMAN	E9PKE3	Heat shock cognate 71 kDa protein
4136	dirMS_041712_NAR_60mM_plus.14411.14447.3	3	13.83	1	1.86E+03		(R)TVENIKI	(R)T/V|E/N I/K/D/P\L F/R(F)		133.2	444.5813	1331.732	-0.0024	-1.8	5.74	535136.6	6.01	HUMAN	Q14204	Cytoplasmic dynein 1 heavy chain 1
4137	dirMS_041712_NAR_120mM_minus.13523.13544.3	3	13.82	0	5.03E+05		(K)YNPTWI	(K)Y N|P/T/W/H/C I V G R(N)	C:Carbamidomethylation	128.4	468.2346	1402.669	0.0208	14.8	8.75	10536.9	6.9	HUMAN	P63167	Dynein light chain 1, cytoplasmic

<table>
<tr><td>4138</td><td>dirMS_041712_NAR_20mM_minus.12055.12055.2</td><td>2</td><td>13.82</td><td>0</td><td>1.19E+06</td><td>M256m</td><td>(R)SLDmDS</td><td>(R)S L|D/m\D/S/I I|A E\V/K(A)</td><td>m:Oxidized methionine</td><td>170.22</td><td>668.8364</td><td>1320.671</td><td>15.994</td><td>-0.7</td><td>4.03</td><td>37296.3</td><td>5.3</td><td>HUMAN</td><td>F8VU64</td><td>Keratin, type II cytoskeletal 8</td></tr>
<tr><td>4139</td><td>dirMS_041712_NAR_30mM_minus2.19457.19457.2</td><td>2</td><td>13.82</td><td>0</td><td>1.03E+06</td><td></td><td>(R)LFDQAF</td><td>(R)L/F/D/Q/A/F G L|P R(L)</td><td></td><td>162.27</td><td>582.315</td><td>1163.621</td><td>0.002</td><td>1.7</td><td>5.84</td><td>20576.9</td><td>9.84</td><td>HUMAN</td><td>F8WE04</td><td>Heat shock protein beta-1</td></tr>
<tr><td>4140</td><td>dirMS_041712_NAR_30mM_minus2.19543.19543.2</td><td>2</td><td>13.82</td><td>0</td><td>2.75E+05</td><td></td><td>(R)ILVATNL</td><td>(R)I/L|V|A/T N L/F G R(G)</td><td></td><td>162.1</td><td>552.3309</td><td>1103.657</td><td>-0.0026</td><td>-2.4</td><td>9.75</td><td>40918.8</td><td>9.33</td><td>HUMAN</td><td>B4DP52</td><td>HCG2005638, isoform CRA_c</td></tr>
<tr><td>4141</td><td>dirMS_041712_NAR_50mM_minus2.10748.10748.3</td><td>3</td><td>13.82</td><td>0</td><td>1.78E+05</td><td></td><td>(R)LLELGPK</td><td>(R)L/L\E L/G P K/P E V|A Q Q T R(K)</td><td></td><td>129.08</td><td>560.3182</td><td>1678.949</td><td>-0.0086</td><td>-5.1</td><td>6.14</td><td>139883.7</td><td>8.06</td><td>HUMAN</td><td>P53621</td><td>Coatomer subunit alpha</td></tr>
<tr><td>4142</td><td>dirMS_041712_NAR_50mM_minus2.11342.11342.3</td><td>3</td><td>13.82</td><td>1</td><td>5.02E+05</td><td></td><td>(K)ILIANTG</td><td>(K)I/L|I/A N/T/G M D T D/K I/K(I)</td><td></td><td>133.4</td><td>511.6179</td><td>1532.835</td><td>0.0039</td><td>2.5</td><td>5.96</td><td>57204.9</td><td>6.01</td><td>HUMAN</td><td>F5GWF6</td><td>T-complex protein 1 subunit beta</td></tr>
<tr><td>4143</td><td>dirMS_041712_NAR_120mM_plus.14793.14954.3</td><td>3</td><td>13.82</td><td>0</td><td>1.55E+06</td><td></td><td>(-)MVNHFI</td><td>(-)M/V/N H/F/I/A E/F K(R)</td><td></td><td>131.67</td><td>412.5446</td><td>1235.624</td><td>-0.0049</td><td>-4</td><td>6.5</td><td>45508.6</td><td>5.5</td><td>HUMAN</td><td>A8K7Q2</td><td>Heat shock cognate 71 kDa protein</td></tr>
<tr><td>4144</td><td>dirMS_041712_NAR_20mM_plus.16787.16787.2</td><td>2</td><td>13.82</td><td>0</td><td>3.08E+05</td><td></td><td>(K)VQIAVA</td><td>(K)V Q|I/A|V|A/N A/Q E L/L Q R(M)</td><td></td><td>187.02</td><td>776.9419</td><td>1552.881</td><td>-0.004</td><td>-2.6</td><td>5.97</td><td>10728.1</td><td>9.15</td><td>HUMAN</td><td>Q9Y5L4</td><td>Mitochondrial import inner membrane translocase subunit Tim13</td></tr>
<tr><td>4145</td><td>dirMS_041712_NAR_30mM_plus2.12359.12861.2</td><td>2</td><td>13.82</td><td>0</td><td>7.56E+05</td><td></td><td>(R)LLVVTDI</td><td>(R)L/L|V|V/T D|P/R(A)</td><td></td><td>119.23</td><td>456.779</td><td>912.551</td><td>-0.0006</td><td>-0.6</td><td>5.84</td><td>33022.6</td><td>4.78</td><td>HUMAN</td><td>A6NE09</td><td>Protein RPSAP58</td></tr>
<tr><td>4146</td><td>dirMS_041712_NAR_30mM_plus2.18887.18887.2</td><td>2</td><td>13.82</td><td>0</td><td>1.87E+05</td><td></td><td>(K)LNLGTV</td><td>(K)L N|L/G/T V/G F/Y R(T)</td><td></td><td>152.8</td><td>570.3123</td><td>1139.621</td><td>-0.0034</td><td>-3</td><td>8.75</td><td>95141.7</td><td>5.22</td><td>HUMAN</td><td>B7Z463</td><td>Puromycin-sensitive aminopeptidase</td></tr>
<tr><td>4147</td><td>dirMS_041712_NAR_40mM_plus2.9052.9238.2</td><td>2</td><td>13.82</td><td>0</td><td>5.38E+05</td><td></td><td>(R)GGNFGF</td><td>(R)G G\N|F|G/F/G D/S/R(G)</td><td></td><td>116.43</td><td>507.2256</td><td>1013.444</td><td>0.0004</td><td>0.4</td><td>5.84</td><td>37486.1</td><td>9.03</td><td>HUMAN</td><td>P22626</td><td>Heterogeneous nuclear ribonucleoproteins A2/B1</td></tr>
<tr><td>4148</td><td>dirMS_041712_NAR_50mM_plus2.11496.11496.3</td><td>3</td><td>13.82</td><td>1</td><td>2.80E+06</td><td>M72m</td><td>(R)TEmENE</td><td>(R)T/E/m E N\E\F V/L I/K K(D)</td><td>m:Oxidized methionine</td><td>127.98</td><td>499.5943</td><td>1480.772</td><td>15.9968</td><td>1.2</td><td>4.78</td><td>13588.1</td><td>4.66</td><td>HUMAN</td><td>F8VP67</td><td>Keratin, type II cytoskeletal 8 (Fragment)</td></tr>
<tr><td>4149</td><td>dirMS_041712_NAR_50mM_plus2.12313.12451.3</td><td>3</td><td>13.82</td><td>1</td><td>7.86E+05</td><td></td><td>(K)IEVEKPF</td><td>(K)I/E|V E/K/P F A I A K E(-)</td><td></td><td>133.12</td><td>458.5944</td><td>1373.767</td><td>0.0012</td><td>0.8</td><td>5.01</td><td>23799.1</td><td>9.48</td><td>HUMAN</td><td>P23284</td><td>Peptidyl-prolyl cis-trans isomerase B</td></tr>
<tr><td>4150</td><td>dirMS_041712_NAR_50mM_plus2.5862.5862.3</td><td>3</td><td>13.82</td><td>1</td><td>1.71E+05</td><td></td><td>(R)ATEGM</td><td>(R)A T|E/G/M/V/V/A/D/K N C S K(I)</td><td>C:Carbamidomethylation</td><td>94.28</td><td>503.9061</td><td>1509.704</td><td>0.0002</td><td>0.1</td><td>6.11</td><td>15607.8</td><td>9.99</td><td>HUMAN</td><td>B4E0P1</td><td>Proteasome (Prosome, macropain) subunit, beta type, 7, isoform CRA_b</td></tr>
<tr><td>4151</td><td>dirMS_041712_NAR_60mM_plus.9245.9245.3</td><td>3</td><td>13.82</td><td>1</td><td>3.38E+05</td><td></td><td>(K)YIDENQI</td><td>(K)Y/I/D\E/N Q D R Y I/K(K)</td><td></td><td>106.53</td><td>486.2401</td><td>1456.707</td><td>-0.0009</td><td>-0.6</td><td>4.56</td><td>6412.4</td><td>9.52</td><td>HUMAN</td><td>J3KSS4</td><td>Cytosolic non-specific dipeptidase (Fragment)</td></tr>
<tr><td>4152</td><td>dirMS_041712_NAR_30mM_minus2.20190.20272.2</td><td>2</td><td>13.81</td><td>0</td><td>6.55E+05</td><td></td><td>(K)IGFPWSI</td><td>(K)I/G/F|P/W/S\E I R(N)</td><td></td><td>171.88</td><td>552.7932</td><td>1104.584</td><td>-0.0045</td><td>-4.1</td><td>6</td><td>71218.8</td><td>6.3</td><td>HUMAN</td><td>A7YIJ8</td><td>Radixin</td></tr>
<tr><td>4153</td><td>dirMS_041712_NAR_30mM_minus2.7344.7382.2</td><td>2</td><td>13.81</td><td>0</td><td>5.46E+05</td><td></td><td>(R)DATME</td><td>(R)D A|T|M/E/V/Q/R(G)</td><td></td><td>94.87</td><td>475.2269</td><td>949.441</td><td>0.0058</td><td>6.1</td><td>4.37</td><td>558007.1</td><td>5.44</td><td>HUMAN</td><td>P58107</td><td>Epiplakin</td></tr>
<tr><td>4154</td><td>dirMS_041712_NAR_40mM_minus2.9385.9445.3</td><td>3</td><td>13.81</td><td>0</td><td>2.76E+05</td><td></td><td>(K)AGVVGF</td><td>(K)A/G|V\V|G|P E L/H/E Q L L S A E/K(A)</td><td></td><td>138.13</td><td>592.9867</td><td>1776.949</td><td>-0.0035</td><td>-2</td><td>4.75</td><td>533778</td><td>5.74</td><td>HUMAN</td><td>Q15149</td><td>Plectin</td></tr>
<tr><td>4155</td><td>dirMS_041712_NAR_50mM_minus2.11264.11264.3</td><td>3</td><td>13.81</td><td>1</td><td>1.86E+05</td><td></td><td>(R)DNKQA</td><td>(R)D N K Q A\G\V/F/E/P T I V K(V)</td><td></td><td>132.05</td><td>515.9485</td><td>1545.827</td><td>0.0038</td><td>2.5</td><td>6.07</td><td>44227.3</td><td>7.81</td><td>HUMAN</td><td>E7EQR6</td><td>T-complex protein 1 subunit alpha</td></tr>
<tr><td>4156</td><td>dirMS_041712_NAR_50mM_minus2.14145.14145.3</td><td>3</td><td>13.81</td><td>1</td><td>1.56E+05</td><td></td><td>(K)KGVNLP</td><td>(K)K G V N L P G\A\A|V/D|L/P A V/S E K(D)</td><td></td><td>148.73</td><td>588.9987</td><td>1764.985</td><td>-0.0039</td><td>-2.2</td><td>6.07</td><td>30947.9</td><td>6.47</td><td>HUMAN</td><td>H3BQ34</td><td>Pyruvate kinase</td></tr>
<tr><td>4157</td><td>dirMS_041712_NAR_60mM_minus.12602.12764.3</td><td>3</td><td>13.81</td><td>1</td><td>1.12E+06</td><td></td><td>(K)AVLFCLS</td><td>(K)A V/L|F/C/L/S E D/K K(N)</td><td>C:Carbamidomethylation</td><td>131.03</td><td>437.2334</td><td>1309.682</td><td>0.0036</td><td>2.7</td><td>6.11</td><td>22956</td><td>8.8</td><td>HUMAN</td><td>E9PK25</td><td>Cofilin-1</td></tr>
<tr><td>4158</td><td>dirMS_041712_NAR_120mM_plus.9704.9704.2</td><td>2</td><td>13.81</td><td>0</td><td>8.09E+05</td><td></td><td>(R)FATHAA</td><td>(R)F A/T/H/A|A\A L S V|R(N)</td><td></td><td>104.23</td><td>572.3112</td><td>1143.627</td><td>-0.0118</td><td>-10.3</td><td>9.75</td><td>76262.2</td><td>9.53</td><td>HUMAN</td><td>P23246</td><td>Splicing factor, proline- and glutamine-rich</td></tr>
<tr><td>4159</td><td>dirMS_041712_NAR_20mM_plus.11926.11926.2</td><td>2</td><td>13.81</td><td>0</td><td>7.68E+04</td><td></td><td>(K)ILQDGG</td><td>(K)I/L|Q/D/G G L Q V/V E K(Q)</td><td></td><td>155.55</td><td>649.8662</td><td>1298.731</td><td>-0.0063</td><td>-4.9</td><td>4.37</td><td>57391.2</td><td>6.29</td><td>HUMAN</td><td>O43175</td><td>D-3-phosphoglycerate dehydrogenase</td></tr>
<tr><td>4160</td><td>dirMS_041712_NAR_30mM_plus2.16130.16130.2</td><td>2</td><td>13.81</td><td>0</td><td>2.79E+05</td><td></td><td>(K)MDQAII</td><td>(K)M/D Q|A|I/I/F/C/R(T)</td><td>C:Carbamidomethylation</td><td>137.37</td><td>577.2809</td><td>1153.549</td><td>0.0053</td><td>4.6</td><td>5.59</td><td>83400.6</td><td>6.82</td><td>HUMAN</td><td>Q92499</td><td>ATP-dependent RNA helicase DDX1</td></tr>
<tr><td>4161</td><td>dirMS_041712_NAR_40mM_plus2.13263.13421.3</td><td>3</td><td>13.81</td><td>0</td><td>6.01E+05</td><td></td><td>(R)QVSASE</td><td>(R)Q/V S A/S E L/H/T S G\I/L/G/P E T L R(D)</td><td></td><td>145.08</td><td>665.6807</td><td>1995.051</td><td>-0.023</td><td>-11.5</td><td>5.4</td><td>558007.1</td><td>5.44</td><td>HUMAN</td><td>P58107</td><td>Epiplakin</td></tr>
<tr><td>4162</td><td>dirMS_041712_NAR_50mM_plus2.9720.9720.3</td><td>3</td><td>13.81</td><td>1</td><td>3.83E+05</td><td></td><td>(K)LTSDDVI</td><td>(K)L/T/S D D/V K/E/Q I Y K(L)</td><td></td><td>118.77</td><td>480.2521</td><td>1438.742</td><td>-0.0007</td><td>-0.5</td><td>4.56</td><td>12573.5</td><td>10.39</td><td>HUMAN</td><td>E9PS50</td><td>40S ribosomal protein S13 (Fragment)</td></tr>
<tr><td>4163</td><td>dirMS_041712_NAR_60mM_plus.12448.12627.3</td><td>3</td><td>13.81</td><td>0</td><td>1.52E+06</td><td></td><td>(K)ADDGRF</td><td>(K)A/D|D/G R/P F/P Q V/I\K(S)</td><td></td><td>121.6</td><td>448.2414</td><td>1342.711</td><td>-0.0017</td><td>-1.3</td><td>6</td><td>15633.5</td><td>9.15</td><td>HUMAN</td><td>H3BMQ8</td><td>Fructose-bisphosphate aldolase A (Fragment)</td></tr>
<tr><td>4164</td><td>dirMS_041712_NAR_30mM_minus2.17126.17126.2</td><td>2</td><td>13.8</td><td>0</td><td>4.44E+04</td><td></td><td>(K)LGINSLC</td><td>(K)L G|I|N/S/L/Q E\L K(Q)</td><td></td><td>146.95</td><td>557.8242</td><td>1114.647</td><td>-0.0055</td><td>-5</td><td>6</td><td>25978.5</td><td>4.91</td><td>HUMAN</td><td>C9J502</td><td>Prostate leucine zipper variant 2</td></tr>
<tr><td>4165</td><td>dirMS_041712_NAR_30mM_minus2.9063.9084.2</td><td>2</td><td>13.8</td><td>0</td><td>8.36E+05</td><td></td><td>(R)ALANSL</td><td>(R)A L|A/N/S/L\A/C/Q/G K(Y)</td><td>C:Carbamidomethylation</td><td>103.73</td><td>566.7927</td><td>1132.578</td><td>0.0002</td><td>0.2</td><td>8.8</td><td>13951.6</td><td>9.48</td><td>HUMAN</td><td>H3BR68</td><td>Fructose-bisphosphate aldolase A (Fragment)</td></tr>
<tr><td>4166</td><td>dirMS_041712_NAR_50mM_minus2.13554.13554.3</td><td>3</td><td>13.8</td><td>0</td><td>3.38E+05</td><td></td><td>(K)MLDAED</td><td>(K)M/L/D/A E/D I/V N T A R\P D E K(A)</td><td></td><td>145.72</td><td>606.2965</td><td>1816.875</td><td>0.0004</td><td>0.2</td><td>4.1</td><td>105308.6</td><td>5.27</td><td>HUMAN</td><td>O43707</td><td>Alpha-actinin-4</td></tr>
<tr><td>4167</td><td>dirMS_041712_NAR_50mM_minus2.14706.14706.3</td><td>3</td><td>13.8</td><td>1</td><td>4.18E+05</td><td></td><td>(K)GCLELIK</td><td>(K)G C/L E L I/K E/T G/V/P I/A G/R(H)</td><td>C:Carbamidomethylation</td><td>151.82</td><td>571.6494</td><td>1712.936</td><td>-0.0027</td><td>-1.6</td><td>6.14</td><td>111409.8</td><td>9.02</td><td>HUMAN</td><td>F5H2F4</td><td>C-1-tetrahydrofolate synthase, cytoplasmic</td></tr>
<tr><td>4168</td><td>dirMS_041712_NAR_50mM_minus2.16456.16456.3</td><td>3</td><td>13.8</td><td>1</td><td>1.60E+05</td><td></td><td>(K)VVSSVL</td><td>(K)V V|S S/V L/Q/F/G N I S F K K(E)</td><td></td><td>162</td><td>551.6488</td><td>1652.937</td><td>-0.0052</td><td>-3.1</td><td>10</td><td>233493.8</td><td>5.45</td><td>HUMAN</td><td>F8VTL3</td><td>Myosin-10</td></tr>
<tr><td>4169</td><td>dirMS_041712_NAR_60mM_minus.15834.15834.3</td><td>3</td><td>13.8</td><td>1</td><td>3.14E+05</td><td></td><td>(R)IDTRNEL</td><td>(R)I D|T R N E L/E|S/Y/A Y S L/K(N)</td><td></td><td>148.13</td><td>601.3049</td><td>1801.897</td><td>0.0035</td><td>1.9</td><td>4.68</td><td>72445.7</td><td>5.07</td><td>HUMAN</td><td>P11021</td><td>78 kDa glucose-regulated protein</td></tr>
<tr><td>4170</td><td>dirMS_041712_NAR_20mM_plus.17996.17996.2</td><td>2</td><td>13.8</td><td>0</td><td>1.36E+05</td><td></td><td>(R)LFDQAF</td><td>(R)L F|D Q/A/F/G/L/P R(L)</td><td></td><td>195.52</td><td>582.3166</td><td>1163.621</td><td>0.0052</td><td>4.4</td><td>5.84</td><td>20576.9</td><td>9.84</td><td>HUMAN</td><td>F8WE04</td><td>Heat shock protein beta-1</td></tr>
<tr><td>4171</td><td>dirMS_041712_NAR_30mM_plus2.13844.13879.2</td><td>2</td><td>13.8</td><td>0</td><td>6.93E+04</td><td></td><td>(R)LNLNNT</td><td>(R)L/N|L|N|N T/V|L/S K(R)</td><td></td><td>127.2</td><td>558.323</td><td>1115.642</td><td>-0.0032</td><td>-2.8</td><td>8.75</td><td>172185.3</td><td>7.06</td><td>HUMAN</td><td>P07814</td><td>Bifunctional glutamate/proline--tRNA ligase</td></tr>
<tr><td>4172</td><td>dirMS_041712_NAR_30mM_plus2.16608.16608.2</td><td>2</td><td>13.8</td><td>0</td><td>3.86E+04</td><td></td><td>(K)LGEWV</td><td>(K)L/G/E/W/V/G L C K(I)</td><td>C:Carbamidomethylation</td><td>140.05</td><td>531.2766</td><td>1061.545</td><td>0.0011</td><td>1</td><td>6</td><td>14914.1</td><td>6.84</td><td>HUMAN</td><td>P25398</td><td>40S ribosomal protein S12</td></tr>
<tr><td>4173</td><td>dirMS_041712_NAR_30mM_plus2.16644.16644.2</td><td>2</td><td>13.8</td><td>0</td><td>3.08E+05</td><td></td><td>(R)SIQADG</td><td>(R)S I|Q/A|D/G L V/W G S/S K(L)</td><td></td><td>140.3</td><td>674.3475</td><td>1347.69</td><td>-0.0026</td><td>-1.9</td><td>5.55</td><td>24934.4</td><td>4.5</td><td>HUMAN</td><td>P24534</td><td>Elongation factor 1-beta</td></tr>
<tr><td>4174</td><td>dirMS_041712_NAR_40mM_plus2.13621.13621.3</td><td>3</td><td>13.8</td><td>1</td><td>1.31E+04</td><td></td><td>(K)DMELPT</td><td>(K)D M/E/L/P T E K E V A L/V K(D)</td><td></td><td>146.4</td><td>534.6192</td><td>1601.845</td><td>-0.0024</td><td>-1.5</td><td>4.41</td><td>85679.3</td><td>7.65</td><td>HUMAN</td><td>B9ZVR1</td><td>Microtubule-associated protein</td></tr>
<tr><td>4175</td><td>dirMS_041712_NAR_40mM_plus2.19985.19985.3</td><td>3</td><td>13.8</td><td>1</td><td>1.56E+06</td><td></td><td>(R)ELAILLG</td><td>(R)E L/A I/L/L G/M/L D/P A E\K D/E K(G)</td><td></td><td>189.1</td><td>629.003</td><td>1884.999</td><td>-0.0042</td><td>-2.3</td><td>4.18</td><td>25148.6</td><td>6</td><td>HUMAN</td><td>P30041</td><td>Peroxiredoxin-6</td></tr>
<tr><td>4176</td><td>dirMS_041712_NAR_50mM_plus2.10121.10121.3</td><td>3</td><td>13.8</td><td>0</td><td>4.13E+05</td><td></td><td>(R)MVQVH</td><td>(R)M V/Q|V/H/E/L|G C E G I/S K(S)</td><td>C:Carbamidomethylation</td><td>120.23</td><td>529.5984</td><td>1586.767</td><td>0.0141</td><td>8.9</td><td>5.38</td><td>83880.6</td><td>7.33</td><td>HUMAN</td><td>F5H365</td><td>Protein transport protein Sec23A</td></tr>
<tr><td>4177</td><td>dirMS_041712_NAR_60mM_plus.13794.13930.3</td><td>3</td><td>13.8</td><td>0</td><td>7.04E+05</td><td></td><td>(K)VLSLNFS</td><td>(K)V/L|S L/N/F/S\E/C H T K(I)</td><td>C:Carbamidomethylation</td><td>129.08</td><td>478.9108</td><td>1434.705</td><td>0.0133</td><td>9.3</td><td>6.72</td><td>16092.9</td><td>6.43</td><td>HUMAN</td><td>D6RAC7</td><td>Ras GTPase-activating protein-binding protein 2 (Fragment)</td></tr>
<tr><td>4178</td><td>dirMS_041712_NAR_60mM_plus.18330.18330.3</td><td>3</td><td>13.8</td><td>1</td><td>2.93E+05</td><td></td><td>(K)LLQDFF</td><td>(K)L L|Q/D/F/F N G R D L N K(S)</td><td></td><td>152.73</td><td>527.2793</td><td>1579.823</td><td>0.0006</td><td>0.4</td><td>5.96</td><td>60126</td><td>5.44</td><td>HUMAN</td><td>E7EP94</td><td>Heat shock 70 kDa protein 1A/1B</td></tr>
<tr><td>4179</td><td>dirMS_041712_NAR_120mM_minus.14882.14882.3</td><td>3</td><td>13.79</td><td>1</td><td>2.69E+05</td><td></td><td>(K)KKELEEI</td><td>(K)K K E L E E\I|V|Q/P I I/S K(L)</td><td></td><td>135.13</td><td>551.9968</td><td>1653.978</td><td>-0.0027</td><td>-1.6</td><td>6.23</td><td>72445.7</td><td>5.07</td><td>HUMAN</td><td>P11021</td><td>78 kDa glucose-regulated protein</td></tr>
<tr><td>4180</td><td>dirMS_041712_NAR_20mM_minus.9217.9217.2</td><td>2</td><td>13.79</td><td>0</td><td>1.90E+05</td><td>M239m</td><td>(K)EITALAP</td><td>(K)E I|T|A|L|A|P S T m K(I)</td><td>m:Oxidized methionine</td><td>141.92</td><td>589.3094</td><td>1161.618</td><td>15.9931</td><td>-1.5</td><td>6.1</td><td>32616.5</td><td>5.18</td><td>HUMAN</td><td>A6NL76</td><td>Actin, alpha skeletal muscle</td></tr>
<tr><td>4181</td><td>dirMS_041712_NAR_30mM_minus2.13925.14079.2</td><td>2</td><td>13.79</td><td>0</td><td>5.48E+05</td><td></td><td>(R)DVQEIFF</td><td>(R)D V|Q|E\I/F/R(L)</td><td></td><td>129.28</td><td>453.7335</td><td>906.468</td><td>-0.0082</td><td>-9.1</td><td>4.37</td><td>30944.2</td><td>6.52</td><td>HUMAN</td><td>B1Q2N1</td><td>ATP-dependent RNA helicase DDX39A</td></tr>
<tr><td>4182</td><td>dirMS_041712_NAR_20mM_plus.17622.17645.3</td><td>3</td><td>13.79</td><td>0</td><td>1.23E+05</td><td></td><td>(K)LATQSN</td><td>(K)L A T Q S N E\I/T\I/P/V/T/F/E S R(A)</td><td></td><td>193.2</td><td>636.0003</td><td>1905.992</td><td>-0.0053</td><td>-2.8</td><td>4.53</td><td>3888.3</td><td>5</td><td>HUMAN</td><td>C9J3N8</td><td>Heat shock protein beta-1</td></tr>
<tr><td>4183</td><td>dirMS_041712_NAR_20mM_plus.17655.17655.2</td><td>2</td><td>13.79</td><td>0</td><td>8.14E+04</td><td></td><td>(R)TTLLPGL</td><td>(R)T T/L|L|P G L L K(T)</td><td></td><td>193.85</td><td>478.3131</td><td>955.619</td><td>0.0003</td><td>0.3</td><td>8.41</td><td>55373.1</td><td>7.25</td><td>HUMAN</td><td>F5H6Y1</td><td>Phenylalanine--tRNA ligase beta subunit</td></tr>
<tr><td>4184</td><td>dirMS_041712_NAR_30mM_plus2.17876.18106.2</td><td>2</td><td>13.79</td><td>0</td><td>4.74E+05</td><td></td><td>(K)LLLWSQ</td><td>(K)L/L|L|W/S Q/R(M)</td><td></td><td>147.83</td><td>458.2693</td><td>915.541</td><td>-0.0097</td><td>-10.6</td><td>9.75</td><td>22576.6</td><td>9.45</td><td>HUMAN</td><td>E9PKG0</td><td>Plectin (Fragment)</td></tr>
<tr><td>4185</td><td>dirMS_041712_NAR_30mM_plus2.18587.18692.2</td><td>2</td><td>13.79</td><td>0</td><td>1.13E+06</td><td></td><td>(K)AAIDWF</td><td>(K)A A|I|D|W/F/D/G K(E)</td><td></td><td>151.52</td><td>511.7539</td><td>1022.494</td><td>0.0064</td><td>6.2</td><td>4.21</td><td>53724.2</td><td>9.54</td><td>HUMAN</td><td>H3BPE7</td><td>RNA-binding protein FUS</td></tr>
<tr><td>4186</td><td>dirMS_041712_NAR_30mM_plus2.19869.19917.2</td><td>2</td><td>13.79</td><td>0</td><td>2.00E+05</td><td></td><td>(R)IIPGFM</td><td>(R)I/I|P G/F M/C Q G G D/F T/R(H)</td><td>C:Carbamidomethylation</td><td>157.72</td><td>799.8749</td><td>1598.745</td><td>-0.0029</td><td>-1.8</td><td>5.84</td><td>13378.8</td><td>6.27</td><td>HUMAN</td><td>C9J5S7</td><td>Peptidyl-prolyl cis-trans isomerase</td></tr>
<tr><td>4187</td><td>dirMS_041712_NAR_40mM_plus2.10338.10338.2</td><td>2</td><td>13.79</td><td>0</td><td>9.40E+04</td><td></td><td>(K)IGGIGTV</td><td>(K)I/G/G\I/G/T/V|P V G R(V)</td><td></td><td>125.75</td><td>513.3081</td><td>1025.61</td><td>-0.0013</td><td>-1.2</td><td>9.75</td><td>50482.3</td><td>9.31</td><td>HUMAN</td><td>P68104</td><td>Elongation factor 1-alpha 1</td></tr>
<tr><td>4188</td><td>dirMS_041712_NAR_50mM_plus2.14696.14696.3</td><td>3</td><td>13.79</td><td>0</td><td>2.11E+06</td><td></td><td>(R)QVSASE</td><td>(R)Q V/S A/S E L H T S G I L/G P E T L/R(D)</td><td></td><td>144.9</td><td>665.6855</td><td>1995.051</td><td>-0.0086</td><td>-4.3</td><td>5.4</td><td>558007.1</td><td>5.44</td><td>HUMAN</td><td>P58107</td><td>Epiplakin</td></tr>
<tr><td>4189</td><td>dirMS_041712_NAR_50mM_plus2.15882.15882.3</td><td>3</td><td>13.79</td><td>1</td><td>3.70E+06</td><td></td><td>(K)ISDSEGF</td><td>(K)I/S|D/S/E/G F/K A N/L S/L L R(R)</td><td></td><td>151.2</td><td>550.6331</td><td>1649.886</td><td>-0.001</td><td>-0.6</td><td>6.07</td><td>76262.2</td><td>9.53</td><td>HUMAN</td><td>P23246</td><td>Splicing factor, proline- and glutamine-rich</td></tr>
<tr><td>4190</td><td>dirMS_041712_NAR_50mM_plus2.16705.16943.3</td><td>3</td><td>13.79</td><td>0</td><td>1.57E+06</td><td></td><td>(K)GSDHSA</td><td>(K)G S D H S A\S L\E|P/G E/L/A/E L V/R(S)</td><td></td><td>156.28</td><td>622.9846</td><td>1866.919</td><td>0.0201</td><td>10.7</td><td>4.4</td><td>12626.3</td><td>5.59</td><td>HUMAN</td><td>Q5TBR0</td><td>N-acetylneuraminic acid synthase (Fragment)</td></tr>
<tr><td>4191</td><td>dirMS_041712_NAR_120mM_minus.19660.19660.3</td><td>3</td><td>13.78</td><td>0</td><td>7.14E+05</td><td></td><td>(R)NSVSNF</td><td>(R)N|S|V/S N F L/H/S L E R(G)</td><td></td><td>162.22</td><td>468.2346</td><td>1402.707</td><td>-0.0181</td><td>-12.9</td><td>6.75</td><td>267260.7</td><td>5.95</td><td>HUMAN</td><td>Q13085</td><td>Acetyl-CoA carboxylase 1</td></tr>
<tr><td>4192</td><td>dirMS_041712_NAR_30mM_minus2.12998.12998.2</td><td>2</td><td>13.78</td><td>0</td><td>2.66E+05</td><td></td><td>(K)AFCGFEI</td><td>(K)A/F/C|G F/E D/P R(T)</td><td>C:Carbamidomethylation</td><td>124.05</td><td>549.7307</td><td>1098.467</td><td>-0.0132</td><td>-12</td><td>4.37</td><td>533778</td><td>5.74</td><td>HUMAN</td><td>Q15149</td><td>Plectin</td></tr>
<tr><td>4193</td><td>dirMS_041712_NAR_30mM_minus2.15571.15571.2</td><td>2</td><td>13.78</td><td>0</td><td>9.61E+04</td><td></td><td>(K)TLAAEM</td><td>(K)T\L|A|A/E/M/Q/E L R(V)</td><td></td><td>137.87</td><td>581.3003</td><td>1161.593</td><td>0.0001</td><td>0.1</td><td>4.53</td><td>67732.1</td><td>4.71</td><td>HUMAN</td><td>G3V119</td><td>DBIRD complex subunit KIAA1967</td></tr>
<tr><td>4194</td><td>dirMS_041712_NAR_30mM_minus2.5081.5081.2</td><td>2</td><td>13.78</td><td>0</td><td>4.08E+05</td><td></td><td>(R)TDTGEP</td><td>(R)T|D|T/G E P/M/G R(G)</td><td></td><td>81.3</td><td>482.2145</td><td>963.42</td><td>0.0017</td><td>1.8</td><td>4.37</td><td>85057.6</td><td>4.94</td><td>HUMAN</td><td>P07900</td><td>Heat shock protein HSP 90-alpha</td></tr>
<tr><td>4195</td><td>dirMS_041712_NAR_40mM_minus2.14086.14086.3</td><td>3</td><td>13.78</td><td>0</td><td>3.46E+05</td><td></td><td>(R)LLEVLSG</td><td>(R)L/L|E V/L S/G E I L/P K P/T K(G)</td><td></td><td>166.62</td><td>546.333</td><td>1636.988</td><td>-0.0039</td><td>-2.4</td><td>6.14</td><td>13611</td><td>6.13</td><td>HUMAN</td><td>E9PJZ2</td><td>Spectrin beta chain, non-erythrocytic 2 (Fragment)</td></tr>
<tr><td>4196</td><td>dirMS_041712_NAR_50mM_minus2.10415.10415.3</td><td>3</td><td>13.78</td><td>0</td><td>2.83E+05</td><td>M35m</td><td>(K)AASDIA</td><td>(K)A A/S D|I/A m/T E/L/P P T H P I R(L)</td><td>m:Oxidized methionine</td><td>127.63</td><td>612.6486</td><td>1819.937</td><td>15.9942</td><td>-0.4</td><td>5.32</td><td>12926.1</td><td>4.4</td><td>HUMAN</td><td>K7EM20</td><td>14-3-3 protein epsilon (Fragment)</td></tr>
<tr><td>4197</td><td>dirMS_041712_NAR_50mM_minus2.12503.12503.3</td><td>3</td><td>13.78</td><td>1</td><td>3.24E+05</td><td></td><td>(K)LYEQILA</td><td>(K)L/Y/E Q I\L|A|E/N E K L K(A)</td><td></td><td>139.15</td><td>530.9592</td><td>1590.874</td><td>-0.0107</td><td>-6.7</td><td>4.79</td><td>22046.7</td><td>5.8</td><td>HUMAN</td><td>H0YIM2</td><td>Protein phosphatase 1 regulatory subunit 12A (Fragment)</td></tr>
<tr><td>4198</td><td>dirMS_041712_NAR_50mM_minus2.12781.12781.3</td><td>3</td><td>13.78</td><td>1</td><td>3.77E+05</td><td></td><td>(K)NTVELL</td><td>(K)N/T|V/E/L L V/E D K G N Q V Y R(C)</td><td></td><td>140.5</td><td>626.3309</td><td>1876.976</td><td>0.0018</td><td>1</td><td>4.68</td><td>258215.9</td><td>5.48</td><td>HUMAN</td><td>E7EN95</td><td>Filamin-B</td></tr>
<tr><td>4199</td><td>dirMS_041712_NAR_60mM_minus.11025.11025.3</td><td>3</td><td>13.78</td><td>1</td><td>1.61E+05</td><td></td><td>(K)LLEDKN</td><td>(K)L/L|E D/K N|G E V Q N L A/V K(C)</td><td></td><td>121.37</td><td>557.3065</td><td>1669.912</td><td>-0.007</td><td>-4.2</td><td>4.68</td><td>6116.1</td><td>5.2</td><td>HUMAN</td><td>F5H6I6</td><td>Cullin-associated NEDD8-dissociated protein 1 (Fragment)</td></tr>
<tr><td>4200</td><td>dirMS_041712_NAR_120mM_plus.12204.12580.4</td><td>4</td><td>13.78</td><td>2</td><td>2.74E+06</td><td></td><td>(K)IGRIEDV</td><td>(K)I G R I E D\V|T|P I/P S/D/S T R/R(K)</td><td></td><td>118.42</td><td>478.7604</td><td>1912.025</td><td>-0.0049</td><td>-2.6</td><td>6.12</td><td>16443.6</td><td>10.45</td><td>HUMAN</td><td>P62263</td><td>40S ribosomal protein S14</td></tr>
<tr><td>4201</td><td>dirMS_041712_NAR_120mM_plus.4082.4293.3</td><td>3</td><td>13.78</td><td>1</td><td>1.73E+07</td><td>M310m</td><td>(R)TKTEISE</td><td>(R)T\K\T E\I|S/E/m/N/R(N)</td><td>m:Oxidized methionine</td><td>69.83</td><td>408.8687</td><td>1208.594</td><td>15.9976</td><td>2.2</td><td>5.81</td><td>37296.3</td><td>5.3</td><td>HUMAN</td><td>F8VU64</td><td>Keratin, type II cytoskeletal 8</td></tr>
<tr><td>4202</td><td>dirMS_041712_NAR_20mM_plus.10650.10650.3</td><td>3</td><td>13.78</td><td>0</td><td>7.11E+04</td><td>M175m</td><td>(R)LEQGQA</td><td>(R)L E Q G Q A\I/D/D|L/m|P A Q K(-)</td><td>m:Oxidized methionine</td><td>146.07</td><td>558.2795</td><td>1656.826</td><td>15.9978</td><td>1.7</td><td>4.03</td><td>20203.9</td><td>6.24</td><td>HUMAN</td><td>H0YJG0</td><td>Creatine kinase B-type (Fragment)</td></tr>
<tr><td>4203</td><td>dirMS_041712_NAR_20mM_plus.16981.16981.2</td><td>2</td><td>13.78</td><td>0</td><td>2.55E+05</td><td></td><td>(K)VQIAVA</td><td>(K)V Q|I/A|V/A N A/Q E L L/Q R(M)</td><td></td><td>188.03</td><td>776.9419</td><td>1552.881</td><td>-0.004</td><td>-2.6</td><td>5.97</td><td>10728.1</td><td>9.15</td><td>HUMAN</td><td>Q9Y5L4</td><td>Mitochondrial import inner membrane translocase subunit Tim13</td></tr>
<tr><td>4204</td><td>dirMS_041712_NAR_20mM_plus.8773.8773.2</td><td>2</td><td>13.78</td><td>0</td><td>6.19E+04</td><td></td><td>(K)EQVANS</td><td>(K)E Q/V/A/N/S/A F V E/R(C)</td><td></td><td>130.78</td><td>625.3102</td><td>1249.617</td><td>-0.004</td><td>-3.2</td><td>4.53</td><td>17664</td><td>8.77</td><td>HUMAN</td><td>H0Y6E4</td><td>Heat shock protein HSP 90-beta (Fragment)</td></tr>
<tr><td>4205</td><td>dirMS_041712_NAR_50mM_plus2.10618.10618.3</td><td>3</td><td>13.78</td><td>0</td><td>3.46E+05</td><td></td><td>(R)AIGSASE</td><td>(R)A I/G S/A/S|E G A Q S S L Q E V Y H K(S)</td><td></td><td>123.47</td><td>654.657</td><td>1961.956</td><td>0.0001</td><td>0.1</td><td>5.4</td><td>20148.5</td><td>4.65</td><td>HUMAN</td><td>B4E2V4</td><td>Proteasome (Prosome, macropain) subunit, alpha type, 5, isoform CRA_c</td></tr>
<tr><td>4206</td><td>dirMS_041712_NAR_50mM_plus2.9305.9305.3</td><td>3</td><td>13.78</td><td>1</td><td>1.01E+06</td><td></td><td>(K)TAVCDI</td><td>(K)T/A|V/C/D|I/P P/R G L K(M)</td><td>C:Carbamidomethylation</td><td>116.55</td><td>442.9121</td><td>1326.72</td><td>0.0019</td><td>1.4</td><td>8.41</td><td>50199.4</td><td>4.75</td><td>HUMAN</td><td>A6NNZ2</td><td>Tubulin beta-8 chain-like protein LOC260334</td></tr>
<tr><td>4207</td><td>dirMS_041712_NAR_20mM_minus.12182.12182.2</td><td>2</td><td>13.77</td><td>0</td><td>1.06E+06</td><td>M313m</td><td>(R)TVQSLEI</td><td>(R)T V|Q\S/L/E/I D L D/S m R(N)</td><td>m:Oxidized methionine</td><td>170.72</td><td>761.8733</td><td>1506.747</td><td>15.9925</td><td>-1.6</td><td>4.03</td><td>43773.3</td><td>5.26</td><td>HUMAN</td><td>F8VZY9</td><td>Keratin, type I cytoskeletal 18</td></tr>
<tr><td>4208</td><td>dirMS_041712_NAR_40mM_minus2.5905.5905.2</td><td>2</td><td>13.77</td><td>0</td><td>1.29E+06</td><td></td><td>(K)AGFAGD</td><td>(K)A G/F/A/G/D/D A P R(A)</td><td></td><td>98.13</td><td>488.7279</td><td>976.448</td><td>0.0002</td><td>0.2</td><td>4.21</td><td>123097.1</td><td>5.83</td><td>HUMAN</td><td>A5A3E0</td><td>POTE ankyrin domain family member F</td></tr>
<tr><td>4209</td><td>dirMS_041712_NAR_50mM_minus2.15255.15378.3</td><td>3</td><td>13.77</td><td>0</td><td>5.45E+05</td><td></td><td>(R)DGLAFN</td><td>(R)D G L|A/F/N A/L/I/H/R(H)</td><td></td><td>155.45</td><td>409.5594</td><td>1226.664</td><td>-0.0004</td><td>-0.3</td><td>6.74</td><td>146974</td><td>5.55</td><td>HUMAN</td><td>C9JRP8</td><td>Spectrin beta chain, non-erythrocytic 4</td></tr>
<tr><td>4210</td><td>dirMS_041712_NAR_120mM_plus.8436.8588.3</td><td>3</td><td>13.77</td><td>1</td><td>5.21E+06</td><td>M174m</td><td>(R)VKYETEL</td><td>(R)V/K Y E T\E|L/A/m/R(Q)</td><td>m:Oxidized methionine</td><td>97.8</td><td>419.2172</td><td>1239.64</td><td>15.9969</td><td>1.6</td><td>6.11</td><td>43773.3</td><td>5.26</td><td>HUMAN</td><td>F8VZY9</td><td>Keratin, type I cytoskeletal 18</td></tr>
<tr><td>4211</td><td>dirMS_041712_NAR_30mM_plus2.12841.12841.2</td><td>2</td><td>13.77</td><td>0</td><td>1.77E+05</td><td></td><td>(K)YFDSGD</td><td>(K)Y/F|D/S G/D/Y N M A\K(A)</td><td></td><td>120.37</td><td>655.7724</td><td>1310.536</td><td>0.0017</td><td>1.3</td><td>4.21</td><td>15633.5</td><td>7.82</td><td>HUMAN</td><td>A6NMQ3</td><td>Alpha-endosulfine</td></tr>
<tr><td>4212</td><td>dirMS_041712_NAR_30mM_plus2.15852.15979.2</td><td>2</td><td>13.77</td><td>0</td><td>5.70E+05</td><td></td><td>(K)LLYNNV</td><td>(K)L L|Y|N/N/V/S N F/G/R(L)</td><td></td><td>136.3</td><td>648.8354</td><td>1296.67</td><td>-0.006</td><td>-4.6</td><td>8.75</td><td>71165.3</td><td>5.57</td><td>HUMAN</td><td>J3KS13</td><td>Clathrin heavy chain 1</td></tr>
<tr><td>4213</td><td>dirMS_041712_NAR_30mM_plus2.8966.8966.2</td><td>2</td><td>13.77</td><td>0</td><td>2.25E+05</td><td></td><td>(R)TVIDYN</td><td>(R)T V|I|D/Y N G E R(T)</td><td></td><td>100.52</td><td>533.7622</td><td>1066.516</td><td>0.0008</td><td>0.7</td><td>4.37</td><td>51524.1</td><td>4.82</td><td>HUMAN</td><td>F5H8J2</td><td>Protein disulfide-isomerase</td></tr>
<tr><td>4214</td><td>dirMS_041712_NAR_40mM_plus2.15059.15059.3</td><td>3</td><td>13.77</td><td>0</td><td>2.29E+05</td><td></td><td>(R)EVIDLLK</td><td>(R)E V|I/D L/L/K P D Q V E G I Q K(S)</td><td></td><td>155.13</td><td>608.6749</td><td>1824.011</td><td>-0.0012</td><td>-0.6</td><td>4.32</td><td>156557.7</td><td>5.52</td><td>HUMAN</td><td>Q86UP2</td><td>Kinectin</td></tr>
<tr><td>4215</td><td>dirMS_041712_NAR_50mM_plus2.12385.12385.3</td><td>3</td><td>13.77</td><td>1</td><td>1.70E+06</td><td></td><td>(R)SGKAPIL</td><td>(R)S G K A P I\L\I|A|T/D/V A S R(G)</td><td></td><td>133.47</td><td>500.2911</td><td>1498.859</td><td>0</td><td>0</td><td>8.46</td><td>73202</td><td>9.19</td><td>HUMAN</td><td>C9JMU5</td><td>Probable ATP-dependent RNA helicase DDX17</td></tr>
<tr><td>4216</td><td>dirMS_041712_NAR_50mM_plus2.14213.14213.3</td><td>3</td><td>13.77</td><td>1</td><td>6.66E+05</td><td></td><td>(R)MSMKE</td><td>(R)M/S M K E V D\E\Q|M\L/N/V Q/N K(N)</td><td></td><td>142.87</td><td>641.9706</td><td>1923.897</td><td>0</td><td>0</td><td>4.68</td><td>40069.4</td><td>4.83</td><td>HUMAN</td><td>E7EWR1</td><td>Tubulin beta chain</td></tr>
<tr><td>4217</td><td>dirMS_041712_NAR_50mM_plus2.19524.19544.3</td><td>3</td><td>13.77</td><td>1</td><td>1.14E+06</td><td></td><td>(R)GDLGIEI</td><td>(R)G D|L/G|I\E/I/P A E K V F L A Q K(M)</td><td></td><td>171.68</td><td>610.0104</td><td>1828.022</td><td>-0.0048</td><td>-2.6</td><td>4.68</td><td>18466.1</td><td>5.78</td><td>HUMAN</td><td>H3BSU3</td><td>Pyruvate kinase (Fragment)</td></tr>
<tr><td>4218</td><td>dirMS_041712_NAR_60mM_plus.6727.6727.2</td><td>2</td><td>13.77</td><td>1</td><td>4.77E+05</td><td></td><td>(K)GPSSVEI</td><td>(K)G/P|S|S/V/E/D I K/A/K(M)</td><td></td><td>93.85</td><td>565.8054</td><td>1130.605</td><td>-0.0017</td><td>-1.5</td><td>6.07</td><td>32745.6</td><td>4.64</td><td>HUMAN</td><td>P06748</td><td>Nucleophosmin</td></tr>
<tr><td>4219</td><td>dirMS_041712_NAR_60mM_plus.9726.9826.3</td><td>3</td><td>13.77</td><td>1</td><td>1.31E+06</td><td></td><td>(K)LAELKQE</td><td>(K)L/A|E/L|K/Q/E/C L\A R(G)</td><td>C:Carbamidomethylation</td><td>109.57</td><td>444.2368</td><td>1330.715</td><td>-0.0189</td><td>-14.2</td><td>6.14</td><td>6106</td><td>9.22</td><td>HUMAN</td><td>F8VS12</td><td>SAP domain-containing ribonucleoprotein</td></tr>
<tr><td>4220</td><td>dirMS_041712_NAR_120mM_minus.12101.12101.4</td><td>4</td><td>13.76</td><td>2</td><td>2.92E+05</td><td></td><td>(R)ILELDQF</td><td>(R)I L/E/L/D Q/F/K/G Q/Q G Q K R(F)</td><td></td><td>120.05</td><td>447.7491</td><td>1787.976</td><td>-0.0017</td><td>-0.9</td><td>8.59</td><td>64302.5</td><td>6.4</td><td>HUMAN</td><td>B1AHC7</td><td>X-ray repair complementing defective repair in Chinese hamster cells 6 (Ku autoantigen, 70kDa)</td></tr>
<tr><td>4221</td><td>dirMS_041712_NAR_20mM_minus.13342.13477.2</td><td>2</td><td>13.76</td><td>0</td><td>6.91E+05</td><td></td><td>(K)DAGTIA</td><td>(K)D A\G\T|I|A/G/L/N V L/R(I)</td><td></td><td>184.93</td><td>600.3403</td><td>1199.674</td><td>-0.0009</td><td>-0.8</td><td>5.84</td><td>17973</td><td>5.16</td><td>HUMAN</td><td>E9PI65</td><td>Heat shock cognate 71 kDa protein (Fragment)</td></tr>
<tr><td>4222</td><td>dirMS_041712_NAR_30mM_minus2.20197.20235.2</td><td>2</td><td>13.76</td><td>0</td><td>6.55E+05</td><td></td><td>(K)IGFPWSI</td><td>(K)I/G/F|P/W/S E/I R(N)</td><td></td><td>171.88</td><td>552.7932</td><td>1104.584</td><td>-0.0045</td><td>-4.1</td><td>6</td><td>71218.8</td><td>6.3</td><td>HUMAN</td><td>A7YIJ8</td><td>Radixin</td></tr>
<tr><td>4223</td><td>dirMS_041712_NAR_50mM_minus2.11119.11259.3</td><td>3</td><td>13.76</td><td>1</td><td>4.95E+05</td><td>M327m</td><td>(R)VAGGPC</td><td>(R)V/A|G/G/P Q m/I/Q|L/S L D/G K R(L)</td><td>m:Oxidized methionine</td><td>131.88</td><td>562.6354</td><td>1669.905</td><td>15.9862</td><td>-5.1</td><td>8.72</td><td>45924.5</td><td>5.29</td><td>HUMAN</td><td>B4E1F3</td><td>Selenium-binding protein 1</td></tr>
<tr><td>4224</td><td>dirMS_041712_NAR_50mM_minus2.11372.11511.3</td><td>3</td><td>13.76</td><td>0</td><td>1.45E+05</td><td></td><td>(K)VSQPIE</td><td>(K)V/S/Q/P I E/G H/A/A S F A Q F K(M)</td><td></td><td>133.52</td><td>572.9609</td><td>1716.87</td><td>-0.0022</td><td>-1.3</td><td>6.72</td><td>193379.8</td><td>5.48</td><td>HUMAN</td><td>Q00610</td><td>Clathrin heavy chain 1</td></tr>
<tr><td>4225</td><td>dirMS_041712_NAR_50mM_minus2.13569.13723.3</td><td>3</td><td>13.76</td><td>1</td><td>5.31E+05</td><td></td><td>(K)LTEDKA</td><td>(K)L/T|E D/K A D V\Q|S/I/G L Q R(F)</td><td></td><td>144.8</td><td>595.9939</td><td>1785.97</td><td>-0.0034</td><td>-1.9</td><td>4.56</td><td>90316.8</td><td>5.52</td><td>HUMAN</td><td>Q13263</td><td>Transcription intermediary factor 1-beta</td></tr>
<tr><td>4226</td><td>dirMS_041712_NAR_50mM_minus2.15928.16015.3</td><td>3</td><td>13.76</td><td>0</td><td>2.60E+05</td><td></td><td>(R)GFGHIG</td><td>(R)G F G H I G I\A\V|P/D/V/Y/S A C K(R)</td><td>C:Carbamidomethylation</td><td>159</td><td>597.6337</td><td>1790.889</td><td>-0.0029</td><td>-1.6</td><td>6.74</td><td>21005.6</td><td>5.12</td><td>HUMAN</td><td>Q04760</td><td>Lactoylglutathione lyase</td></tr>
<tr><td>4227</td><td>dirMS_041712_NAR_60mM_minus.19279.19279.3</td><td>3</td><td>13.76</td><td>0</td><td>1.04E+06</td><td></td><td>(R)SLHDAL</td><td>(R)S L H/D A L\C|V|L/A Q T V K(D)</td><td>C:Carbamidomethylation</td><td>171.22</td><td>518.9484</td><td>1554.831</td><td>-0.0002</td><td>-0.1</td><td>6.46</td><td>57204.9</td><td>6.01</td><td>HUMAN</td><td>F5GWF6</td><td>T-complex protein 1 subunit beta</td></tr>
<tr><td>4228</td><td>dirMS_041712_NAR_20mM_plus.19051.19051.2</td><td>2</td><td>13.76</td><td>0</td><td>5.75E+04</td><td></td><td>(K)ILTFDQL</td><td>(K)I/L/T|F/D Q L A L D S\P K(G)</td><td></td><td>202.35</td><td>730.9063</td><td>1460.8</td><td>0.0058</td><td>4</td><td>4.21</td><td>14643</td><td>11.93</td><td>HUMAN</td><td>F8VUA6</td><td>60S ribosomal protein L18 (Fragment)</td></tr>
<tr><td>4229</td><td>dirMS_041712_NAR_30mM_plus2.19215.19215.2</td><td>2</td><td>13.76</td><td>0</td><td>9.99E+05</td><td></td><td>(R)SSIAGLL</td><td>(R)S/S|I|A/G L/L/L K(A)</td><td></td><td>154.92</td><td>451.2894</td><td>901.572</td><td>-0.0002</td><td>-0.2</td><td>8.47</td><td>533778</td><td>5.74</td><td>HUMAN</td><td>Q15149</td><td>Plectin</td></tr>
<tr><td>4230</td><td>dirMS_041712_NAR_30mM_plus2.19679.19679.2</td><td>2</td><td>13.76</td><td>0</td><td>2.35E+05</td><td></td><td>(K)APDFVF</td><td>(K)A/P/D/F|V/F/Y|A P R(L)</td><td></td><td>156.72</td><td>591.8008</td><td>1182.594</td><td>0.0001</td><td>0.1</td><td>5.88</td><td>71218.8</td><td>6.3</td><td>HUMAN</td><td>A7YIJ8</td><td>Radixin</td></tr>
<tr><td>4231</td><td>dirMS_041712_NAR_30mM_plus2.21854.21854.2</td><td>2</td><td>13.76</td><td>0</td><td>8.60E+04</td><td></td><td>(K)GLVQAL</td><td>(K)G L V\Q/A/L|G/L|S N/F\N S R(Q)</td><td></td><td>169.63</td><td>738.3989</td><td>1475.796</td><td>-0.006</td><td>-4.1</td><td>9.75</td><td>36914.7</td><td>6.33</td><td>HUMAN</td><td>P14550</td><td>Alcohol dehydrogenase [NADP(+)]</td></tr>
<tr><td>4232</td><td>dirMS_041712_NAR_50mM_plus2.14537.14537.3</td><td>3</td><td>13.76</td><td>1</td><td>5.76E+05</td><td></td><td>(R)SEGVVA</td><td>(R)S E/G V\V/A/V L L T K K(S)</td><td></td><td>144.37</td><td>415.2585</td><td>1243.762</td><td>-0.0011</td><td>-0.9</td><td>8.31</td><td>276046.1</td><td>6.01</td><td>HUMAN</td><td>P49327</td><td>Fatty acid synthase</td></tr>
<tr><td>4233</td><td>dirMS_041712_NAR_60mM_plus.15074.15137.3</td><td>3</td><td>13.76</td><td>0</td><td>7.31E+05</td><td></td><td>(K)IVLDNS</td><td>(K)I/V|L/D N S V/F/S E/H/R(N)</td><td></td><td>135.72</td><td>472.5788</td><td>1415.728</td><td>-0.0059</td><td>-4.2</td><td>5.32</td><td>144109.9</td><td>6.01</td><td>HUMAN</td><td>J3KR87</td><td>Clathrin heavy chain 2</td></tr>
<tr><td>4234</td><td>dirMS_041712_NAR_60mM_plus.17704.17764.3</td><td>3</td><td>13.76</td><td>1</td><td>2.85E+05</td><td></td><td>(K)IIVDELK</td><td>(K)I/I|V/D E L/K/Q E V/I/S T S S K(A)</td><td></td><td>149.28</td><td>597.0009</td><td>1788.995</td><td>-0.0072</td><td>-4</td><td>4.68</td><td>30149.5</td><td>5.96</td><td>HUMAN</td><td>D6RAC2</td><td>Guanine nucleotide-binding protein subunit beta-2-like 1</td></tr>
<tr><td>4235</td><td>dirMS_041712_NAR_30mM_minus2.16922.16922.2</td><td>2</td><td>13.75</td><td>0</td><td>1.24E+05</td><td></td><td>(R)ASLEAAI</td><td>(R)A S L|E/A A|I/A D A/E/Q R(G)</td><td></td><td>145.22</td><td>672.8404</td><td>1344.675</td><td>-0.0019</td><td>-1.4</td><td>4.14</td><td>56607.4</td><td>5.37</td><td>HUMAN</td><td>F8VXB4</td><td>Keratin, type II cytoskeletal 8</td></tr>
<tr><td>4236</td><td>dirMS_041712_NAR_40mM_minus2.11043.11128.3</td><td>3</td><td>13.75</td><td>0</td><td>4.70E+05</td><td></td><td>(R)DAGEGL</td><td>(R)D A|G|E/G/L/L A V Q I T/D/Q E G/K\P K(R)</td><td></td><td>148.08</td><td>657.0157</td><td>1969.024</td><td>0.0089</td><td>4.5</td><td>4.32</td><td>258215.9</td><td>5.48</td><td>HUMAN</td><td>E7EN95</td><td>Filamin-B</td></tr>
<tr><td>4237</td><td>dirMS_041712_NAR_50mM_minus2.14977.14977.3</td><td>3</td><td>13.75</td><td>0</td><td>4.42E+05</td><td></td><td>(R)LQQELD</td><td>(R)L Q|Q/E L D D L/T/V D L D/H Q\R(Q)</td><td></td><td>153.05</td><td>646.6559</td><td>1937.956</td><td>-0.0032</td><td>-1.6</td><td>4.03</td><td>233493.8</td><td>5.45</td><td>HUMAN</td><td>F8VTL3</td><td>Myosin-10</td></tr>
<tr><td>4238</td><td>dirMS_041712_NAR_60mM_minus.9414.9414.3</td><td>3</td><td>13.75</td><td>0</td><td>1.04E+05</td><td>M409m</td><td>(K)VLAmSG</td><td>(K)V/L|A\m S G D/P/N Y/L H/R(M)</td><td>m:Oxidized methionine</td><td>112.23</td><td>496.9147</td><td>1472.732</td><td>15.9981</td><td>2.1</td><td>6.71</td><td>57818.1</td><td>5.5</td><td>HUMAN</td><td>E9PH38</td><td>Serine/threonine-protein phosphatase 2A 65 kDa regulatory subunit A alpha isoform</td></tr>
<tr><td>4239</td><td>dirMS_041712_NAR_20mM_plus.12492.12492.3</td><td>3</td><td>13.75</td><td>0</td><td>3.48E+05</td><td></td><td>(K)IISNASC</td><td>(K)I I\S N\A\S/C T T N/C L/A/P L A K(V)</td><td>C:Carbamidomethylation</td><td>159.53</td><td>611.9768</td><td>1833.92</td><td>-0.0039</td><td>-2.1</td><td>8.75</td><td>31718.5</td><td>7.17</td><td>HUMAN</td><td>E7EUT4</td><td>Glyceraldehyde-3-phosphate dehydrogenase</td></tr>
<tr><td>4240</td><td>dirMS_041712_NAR_30mM_plus2.17209.17209.2</td><td>2</td><td>13.75</td><td>0</td><td>4.62E+05</td><td>M256m</td><td>(R)SLDmDS</td><td>(R)S L/D m D\S I|I A E\V K(A)</td><td>m:Oxidized methionine</td><td>142.6</td><td>668.8384</td><td>1320.671</td><td>15.998</td><td>2.3</td><td>4.03</td><td>37296.3</td><td>5.3</td><td>HUMAN</td><td>F8VU64</td><td>Keratin, type II cytoskeletal 8</td></tr>
<tr><td>4241</td><td>dirMS_041712_NAR_30mM_plus2.7359.7359.2</td><td>2</td><td>13.75</td><td>1</td><td>2.13E+05</td><td></td><td>(R)YSGGNY</td><td>(R)Y/S/G G N Y R/D N Y|D|N(-)</td><td></td><td>93.03</td><td>719.2864</td><td>1437.567</td><td>-0.001</td><td>-0.7</td><td>4.21</td><td>17170.1</td><td>8.86</td><td>HUMAN</td><td>P98179</td><td>Putative RNA-binding protein 3</td></tr>
<tr><td>4242</td><td>dirMS_041712_NAR_30mM_plus2.7523.7542.2</td><td>2</td><td>13.75</td><td>0</td><td>6.70E+05</td><td></td><td>(K)LLADQA</td><td>(K)L/L|A|D/Q|A E/A/R(R)</td><td></td><td>93.78</td><td>493.7641</td><td>986.527</td><td>-0.0056</td><td>-5.7</td><td>4.37</td><td>23361.4</td><td>11.6</td><td>HUMAN</td><td>J3KTE4</td><td>Ribosomal protein L19</td></tr>
<tr><td>4243</td><td>dirMS_041712_NAR_40mM_plus2.13228.13228.3</td><td>3</td><td>13.75</td><td>1</td><td>1.17E+05</td><td></td><td>(R)ETFEKTF</td><td>(R)E T|F/E\K T P\V/E V/P/V/G G F K(G)</td><td></td><td>144.53</td><td>588.6444</td><td>1763.921</td><td>-0.0028</td><td>-1.6</td><td>4.79</td><td>533778</td><td>5.74</td><td>HUMAN</td><td>Q15149</td><td>Plectin</td></tr>
<tr><td>4244</td><td>dirMS_041712_NAR_50mM_plus2.18401.18401.3</td><td>3</td><td>13.75</td><td>1</td><td>3.90E+05</td><td></td><td>(R)IAKEEIF</td><td>(R)I A K E E I/F/G/P/V/Q P L F/K(F)</td><td></td><td>164.9</td><td>572.6586</td><td>1715.973</td><td>-0.0118</td><td>-6.9</td><td>6.14</td><td>57661.7</td><td>6.36</td><td>HUMAN</td><td>P30837</td><td>Aldehyde dehydrogenase X, mitochondrial</td></tr>
<tr><td>4245</td><td>dirMS_041712_NAR_50mM_plus2.22162.22162.3</td><td>3</td><td>13.75</td><td>1</td><td>5.40E+05</td><td></td><td>(R)ELAILLG</td><td>(R)E L|A/I/L L/G/M L/D/P A E K D E K(G)</td><td></td><td>190.32</td><td>629.004</td><td>1884.999</td><td>-0.0012</td><td>-0.7</td><td>4.18</td><td>25148.6</td><td>6</td><td>HUMAN</td><td>P30041</td><td>Peroxiredoxin-6</td></tr>
<tr><td>4246</td><td>dirMS_041712_NAR_120mM_minus.10477.10477.3</td><td>3</td><td>13.74</td><td>1</td><td>1.88E+05</td><td></td><td>(K)VEYSEEE</td><td>(K)V/E/Y/S E/E E L K T H I/S K(G)</td><td></td><td>111.32</td><td>564.6199</td><td>1691.849</td><td>-0.0035</td><td>-2.1</td><td>4.91</td><td>65375.7</td><td>6.12</td><td>HUMAN</td><td>B1AHC8</td><td>X-ray repair complementing defective repair in Chinese hamster cells 6 (Ku autoantigen, 70kDa)</td></tr>
</table>

4247	dirMS_041712_NAR_20mM_minus.13391.13391.2	2	13.74	0	6.80E+05		(K)DAGTIA(	(K)D A G\T|I|A/G L N/V L R(I)		184.9	600.3403	1199.674	-0.0009	-0.8	5.84	17973	5.16	HUMAN	E9PI65	Heat shock cognate 71 kDa protein (Fragment)
4248	dirMS_041712_NAR_20mM_minus.14375.14375.2	2	13.74	0	2.36E+05		(K)DAGVIA(	(K)D A G V|I|A/G L N V L/R(I)		198.13	599.3503	1197.695	-0.0017	-1.4	5.84	58502.6	6.09	HUMAN	B4DXY3	Heat shock 70 kDa protein 1-like
4249	dirMS_041712_NAR_20mM_minus.9666.9747.2	2	13.74	0	2.44E+05		(K)LVIITAG	(K)L/V|I|I/T|A/G A R(Q)		147.62	457.2947	913.583	-0.0008	-0.9	9.75	12539.4	5.78	HUMAN	F5GXH2	L-lactate dehydrogenase A chain (Fragment)
4250	dirMS_041712_NAR_30mM_minus2.12724.12724.2	2	13.74	0	2.31E+05		(R)DTYVSSI	(R)D T/Y|V|S/S F P/R(A)		122.43	536.2608	1071.511	0.0038	3.5	5.83	34425.5	9.08	HUMAN	P23193	Transcription elongation factor A protein 1
4251	dirMS_041712_NAR_30mM_minus2.16153.16153.2	2	13.74	0	3.19E+05	M185m	(K)LAVNm\	(K)L A|V|N|m/V/P F P R(L)	m:Oxidized methionine	142.33	580.3178	1143.634	15.994	-0.8	9.75	42230.8	4.77	HUMAN	A6NKZ8	Putative tubulin beta chain-like protein ENSP00000290377
4252	dirMS_041712_NAR_50mM_minus2.16931.16931.3	3	13.74	0	2.37E+05		(R)LGIEGLS	(R)L/G/I/E/G L S L/H N V/L K(L)		164.22	464.9452	1392.821	0.0001	0.1	6.75	177928	5.42	HUMAN	Q9NYU2	UDP-glucose:glycoprotein glucosyltransferase 1
4253	dirMS_041712_NAR_60mM_minus.8283.8283.3	3	13.74	1	1.04E+04		(R)SSPVPP\	(R)S S|P V/P/P V P E/R\R(G)		106.18	407.5617	1220.675	-0.004	-3.3	9.32	96735.1	9.2	HUMAN	J3KPS2	Protein FAM83H (Fragment)
4254	dirMS_041712_NAR_20mM_plus.14316.14316.3	3	13.74	0	8.76E+05	M73m	(R)AVLVDLI	(R)A V\L|V D L E/P G T m/D S V R(S)	m:Oxidized methionine	171.03	539.9421	1601.82	15.9914	-2.2	4.03	13112.4	5.23	HUMAN	F8W813	Tubulin beta-8 chain
4255	dirMS_041712_NAR_30mM_plus2.16977.17513.2	2	13.74	0	1.53E+06		(K)NLYIISVI	(K)N/L|Y|I/I/S V/K(G)		143.67	475.2893	949.572	-0.0004	-0.4	8.59	12270.4	11.49	HUMAN	B9ZVP7	60S ribosomal protein L23
4256	dirMS_041712_NAR_30mM_plus2.6259.6405.2	2	13.74	0	3.31E+05	M367m	(R)FGSGmI	(R)F/G|S|G/m N/M/G R(I)	m:Oxidized methionine	87.53	486.7048	956.408	15.9946	-0.3	9.75	77799.7	9.09	HUMAN	P52272	Heterogeneous nuclear ribonucleoprotein M
4257	dirMS_041712_NAR_50mM_plus2.11576.11844.3	3	13.74	1	3.79E+05		(K)IGRIEDV	(K)I G R I E D V|T|P\I|P S/D/S T R(R)		130.28	585.9784	1755.924	-0.0029	-1.7	4.56	16443.6	10.45	HUMAN	P62263	40S ribosomal protein S14
4258	dirMS_041712_NAR_50mM_plus2.16727.16727.3	3	13.74	0	4.75E+05		(K)SPVTTLL	(K)S/P/V T T L/L E C M H/K(L)	C:Carbamidomethylation	155.32	472.5735	1415.702	0.0038	2.7	6.47	142137.5	9.17	HUMAN	E7ENU4	Double-stranded RNA-specific adenosine deaminase
4259	dirMS_041712_NAR_60mM_plus.17916.17916.3	3	13.74	1	1.75E+05		(K)FNADEF	(K)F/N/A D E F/E D/M/V/A E K R(L)		150.68	567.5906	1700.758	-0.0012	-0.7	4.18	18819.6	10.31	HUMAN	F8W7C6	60S ribosomal protein L10
4260	dirMS_041712_NAR_60mM_plus.8342.8342.3	3	13.74	1	6.44E+05		(K)EPLYEKD	(K)E P L/Y|E K D/S|S/V/A A R(F)		102.25	488.9148	1464.733	-0.0031	-2.1	4.68	26283.8	8.96	HUMAN	O43809	Cleavage and polyadenylation specificity factor subunit 5
4261	dirMS_041712_NAR_30mM_minus2.19101.19101.2	2	13.73	0	2.61E+05		(K)SYELPD(	(K)S Y E|L|P D G Q/V I T I\G/N E R(F)		158.88	895.9492	1790.892	-0.0008	-0.4	4.14	123097.1	5.83	HUMAN	A5A3E0	POTE ankyrin domain family member F
4262	dirMS_041712_NAR_40mM_minus2.12008.12008.3	3	13.73	0	2.78E+05		(K)DAGEGL	(K)D A|G E|G/L/L A V/Q I T D/P E G K/P K(K)		154.02	646.6785	1938.018	0.0031	1.6	4.32	283472.4	5.71	HUMAN	P21333	Filamin-A
4263	dirMS_041712_NAR_50mM_minus2.12227.12350.3	3	13.73	1	2.81E+06		(R)GYSFTT1	(R)G Y|S|F/T T/T A E/R E I V/R(D)		138.1	543.9458	1629.823	-0.0003	-0.2	6.14	23901.9	5.28	HUMAN	I3L1U9	Actin, cytoplasmic 2, N-terminally processed (Fragment)
4264	dirMS_041712_NAR_50mM_minus2.8739.8739.3	3	13.73	1	9.59E+04		(K)ALDLDS	(K)A L|D|L/D/S S C K E/A A D G Y Q R(C)	C:Carbamidomethylation	117.47	633.6221	1898.855	-0.0031	-1.7	4.23	60404.6	6.4	HUMAN	F5H0T1	Stress-induced-phosphoprotein 1
4265	dirMS_041712_NAR_60mM_minus.6746.6746.3	3	13.73	0	4.43E+04	M437m	(R)LESGMC	(R)L E|S G/M Q N m/S I H/T/K(T)	m:Oxidized methionine	96.18	497.9023	1475.698	15.9942	-0.5	6.75	56607.4	5.37	HUMAN	F8VXB4	Keratin, type II cytoskeletal 8
4266	dirMS_041712_NAR_60mM_minus.9416.9416.3	3	13.73	0	1.04E+05	M409m	(K)VLAmSG	(K)V L|A/m/S/G D|P\N Y/L|H R(M)	m:Oxidized methionine	112.23	496.9147	1472.732	15.9981	2.1	6.71	57818.1	5.5	HUMAN	E9PH38	Serine/threonine-protein phosphatase 2A 65 kDa regulatory subunit A alpha isoform
4267	dirMS_041712_NAR_20mM_plus.16519.16519.2	2	13.73	0	1.71E+05		(R)LLEAQIA	(R)L L E A/Q I/A/T|G G\I I D/P K(E)		185.37	769.9362	1538.879	-0.0137	-8.9	4.37	334221.3	6.44	HUMAN	P15924	Desmoplakin
4268	dirMS_041712_NAR_30mM_plus2.19576.19804.2	2	13.73	0	4.94E+05		(K)NLIDAG\	(K)N L|I|D|A|G V|D/A L R(V)		156.75	578.8183	1156.632	-0.0027	-2.4	4.21	51583.4	8.8	HUMAN	H0Y4R1	Inosine-5'-monophosphate dehydrogenase 2 (Fragment)
4269	dirMS_041712_NAR_50mM_plus2.10118.10118.3	3	13.73	0	7.46E+05		(R)GISEETT	(R)G I|S E/E/T T/T G V H N L Y/K(M)		120.05	550.2759	1648.818	-0.0045	-2.8	5.4	45228.3	6.03	HUMAN	F5H737	Adenosylhomocysteinase
4270	dirMS_041712_NAR_50mM_plus2.6458.6479.3	3	13.73	1	1.97E+05		(K)IVSGKDY	(K)I/V|S G K/D Y/N/V/T/A N S K(L)		98.92	499.2611	1495.775	-0.0064	-4.2	8.5	12539.4	5.78	HUMAN	F5GXH2	L-lactate dehydrogenase A chain (Fragment)
4271	dirMS_041712_NAR_120mM_minus.10510.10912.2	2	13.72	1	3.38E+06		(R)VKYETEL	(R)V/K|Y|E/T E/L/A/M/R(Q)		113.43	620.3232	1239.64	-0.0011	-0.9	6.11	43773.3	5.26	HUMAN	F8VZY9	Keratin, type I cytoskeletal 18
4272	dirMS_041712_NAR_120mM_minus.17504.17521.4	4	13.72	1	5.43E+05		(R)GFGHIG	(R)G F G H I G I A\V/P D V/Y/S A C K R(F)	C:Carbamidomethylation	150.4	487.502	1946.991	-0.0044	-2.2	8.6	21005.6	5.12	HUMAN	Q04760	Lactoylglutathione lyase
4273	dirMS_041712_NAR_20mM_minus.10395.10395.2	2	13.72	0	4.57E+04	M145m	(R)VVLFEm	(R)V V|L/F/E m E/A R(K)	m:Oxidized methionine	152.52	555.2868	1093.571	15.9953	0.3	4.53	27142.4	5.34	HUMAN	E5RG68	Copine-3 (Fragment)
4274	dirMS_041712_NAR_50mM_minus2.12905.12905.3	3	13.72	1	1.79E+05		(K)GGELVY	(K)G G\E/L|V|Y/T D S E A R D V F E K(A)		141.53	638.9739	1914.908	-0.0008	-0.4	4.18	533778	5.74	HUMAN	Q15149	Plectin
4275	dirMS_041712_NAR_50mM_minus2.13402.13548.3	3	13.72	1	5.31E+05		(K)LTEDKAI	(K)L/T|E D K A D\V\Q S|I|I/G L Q/R(F)		144.8	595.9939	1785.97	-0.0034	-1.9	4.56	90316.8	5.52	HUMAN	Q13263	Transcription intermediary factor 1-beta
4276	dirMS_041712_NAR_50mM_minus2.7891.7891.3	3	13.72	1	1.10E+05		(K)VLLQSKI	(K)V L|L/Q/S K D Q|I/T A/G N A A R(K)		111.77	562.3158	1684.934	-0.0012	-0.7	8.72	46335.4	6.29	HUMAN	E9PBS1	Phosphoribosylaminoimidazole carboxylase (Fragment)
4277	dirMS_041712_NAR_60mM_minus.6480.6480.3	3	13.72	1	8.87E+04		(K)SESPKEP	(K)S E/S/P/K E/P E/Q/L/R(K)		94.9	433.8887	1299.654	-0.0024	-1.8	4.78	33268.7	9.31	HUMAN	F8VRQ1	Heterogeneous nuclear ribonucleoprotein A1
4278	dirMS_041712_NAR_60mM_minus.9411.9424.3	3	13.72	0	2.36E+05		(K)GISVHIS	(K)G I|S/V/H I/S N A E/P K(H)		112.42	417.9007	1251.669	0.0184	14.7	6.75	33803.8	6.12	HUMAN	B1AKP7	TAR DNA binding protein
4279	dirMS_041712_NAR_120mM_plus.20222.20433.3	3	13.72	1	3.71E+06		(K)AAIDWF	(K)A A|I/D|W/F/D/G K E/F H G N I\I K(V)		158.78	654.3334	1960.992	-0.0059	-3	5.38	49181	4.93	HUMAN	K7EPT6	TATA-binding protein-associated factor 2N
4280	dirMS_041712_NAR_30mM_plus2.18778.18778.2	2	13.72	0	3.32E+05		(R)ESLEALL	(R)E S/L/E/A L/L/Q/R(A)		152.07	529.8026	1058.584	0.0139	13.1	4.53	107721.8	8.85	HUMAN	O94906	Pre-mRNA-processing factor 6
4281	dirMS_041712_NAR_40mM_plus2.11333.11333.3	3	13.72	0	3.11E+04		(K)LQALAN	(K)L Q|A|L/A N E Q/A\A A A/H E L E/K(M)		132.83	602.981	1806.935	-0.006	-3.3	4.75	156557.7	5.52	HUMAN	Q86UP2	Kinectin
4282	dirMS_041712_NAR_50mM_plus2.17780.17780.3	3	13.72	1	6.96E+05		(K)SLLGKD\	(K)S L|L G K/D V L F/L/K(D)		161.7	411.5915	1232.761	-0.0013	-1.1	8.31	41827.5	7	HUMAN	B7Z7A9	Phosphoglycerate kinase
4283	dirMS_041712_NAR_120mM_minus.6624.6687.2	2	13.71	1	4.24E+06		(K)IREEYPD	(K)I/R|E E|Y|P D|R(I)		90.72	539.2683	1077.532	-0.003	-2.8	4.68	42230.8	4.77	HUMAN	A6NKZ8	Putative tubulin beta chain-like protein ENSP00000290377
4284	dirMS_041712_NAR_30mM_minus2.14041.14328.2	2	13.71	0	6.95E+05		(K)ALEIIPR(	(K)A/L|E|I\I/P/R(Q)		130.62	406.2554	811.504	-0.0001	-0.1	6.05	46550.7	7.81	HUMAN	B7Z1C9	Chaperonin containing TCP1, subunit 7 (Eta), isoform CRA_a
4285	dirMS_041712_NAR_50mM_minus2.16381.16381.3	3	13.71	0	1.72E+05		(R)GGYVLH	(R)G G Y/V/L/H I G T I/Y/G D/L K(V)		161.75	535.9583	1605.863	-0.0032	-2	6.74	30427.2	7.21	HUMAN	H3BPK7	Alanine--tRNA ligase, cytoplasmic (Fragment)
4286	dirMS_041712_NAR_120mM_plus.10184.10354.3	3	13.71	0	1.24E+06		(R)ILRPWQ	(R)I/L/R P W|Q|S S E|T/R(S)		106.65	458.2486	1372.733	-0.0019	-1.4	9.6	46648.4	9.67	HUMAN	B4DX42	Splicing factor 1
4287	dirMS_041712_NAR_120mM_plus.10464.10591.3	3	13.71	1	3.83E+05		(K)KGENYV	(K)K\G E/N Y V|Y|I/N/E/A T R(H)		108.4	519.5943	1556.77	-0.002	-1.3	6.14	266161	5.68	HUMAN	E9PPU0	Epiplakin
4288	dirMS_041712_NAR_120mM_plus.18636.18636.4	4	13.71	1	4.62E+05		(R)SKDVVE	(R)S K D V V\E|P/L L R P Q W Y V/R(C)		150.48	497.0291	1985.097	-0.0021	-1.1	8.31	141718.6	7.8	HUMAN	B0V043	Valine--tRNA ligase
4289	dirMS_041712_NAR_20mM_plus.12254.12363.2	2	13.71	0	2.36E+05		(K)DLAGSII	(K)D L|A|G S I/I/G/K(G)		158.22	437.2558	873.504	0.0003	0.4	5.84	51260.6	5.39	HUMAN	P61978	Heterogeneous nuclear ribonucleoprotein K
4290	dirMS_041712_NAR_20mM_plus.16771.16771.2	2	13.71	0	3.05E+05		(K)VQIAVA	(K)V Q|I|A\V/A N A Q/E/L L/Q R(M)		186.97	776.9419	1552.881	-0.004	-2.6	5.97	10728.1	9.15	HUMAN	Q9Y5L4	Mitochondrial import inner membrane translocase subunit Tim13
4291	dirMS_041712_NAR_30mM_plus2.21851.21851.2	2	13.71	0	3.21E+05		(R)SFITWLF	(R)S F/I|T/W L R(D)		170.53	461.7608	922.515	-0.0002	-0.2	9.47	141616.6	6.1	HUMAN	B7ZKM8	Protein transport protein Sec24B
4292	dirMS_041712_NAR_40mM_plus2.13861.13861.3	3	13.71	0	2.23E+05		(K)VALLLLD	(K)V A\L\L|L/L/D Q G/A/S/P H A A A K(N)		148.13	558.9886	1674.954	-0.0025	-1.5	6.71	482683.6	6.07	HUMAN	Q12955	Ankyrin-3
4293	dirMS_041712_NAR_50mM_plus2.15354.15354.3	3	13.71	1	7.54E+05		(K)EAFSLFD	(K)E A/F|S/L/F/D K D G D/G T/I T T K(E)		149.33	615.6348	1844.891	-0.0014	-0.8	4.23	21860.1	4.45	HUMAN	E7EMB3	Calmodulin
4294	dirMS_041712_NAR_50mM_plus2.7352.7352.3	3	13.71	0	8.75E+04		(R)EGATVY.	(R)E G A/T V/Y A/T G T H/A Q V\E D/G R(L)		104.62	621.2948	1861.868	0.0024	1.3	4.65	36208.1	7.21	HUMAN	B4DMU0	Pyrroline-5-carboxylate reductase
4295	dirMS_041712_NAR_60mM_plus.8473.8473.2	2	13.71	0	4.27E+05		(R)FACHSA	(R)F/A|C/H/S A/S/L T\V R(N)	C:Carbamidomethylation	102.73	624.8118	1248.615	0.001	0.8	9.75	23729.5	9.89	HUMAN	C9IZL7	Non-POU domain-containing octamer-binding protein (Fragment)
4296	dirMS_041712_NAR_40mM_minus2.14606.14606.3	3	13.7	1	1.43E+05		(K)GTVPDD	(K)G T|V/P/D D A V E A L A D S L G K K(E)		170.32	595.9791	1785.923	-0.0001	-0.1	4.23	81397.3	4.99	HUMAN	B7Z468	Calpastatin
4297	dirMS_041712_NAR_40mM_minus2.14958.14958.3	3	13.7	0	1.07E+05		(R)EAVELPI	(R)E A V/E/L/P L/T/H F E L Y K(Q)		173.08	563.6324	1688.889	-0.0068	-4	4.75	47479.5	5.09	HUMAN	P43686	26S protease regulatory subunit 6B
4298	dirMS_041712_NAR_60mM_minus.15724.15724.3	3	13.7	1	4.02E+05		(R)AEGAIA(	(R)A E|G A/I/A G F R D/P F S G K(Q)		147.97	508.2601	1522.765	0.0009	0.6	6.12	266161	5.68	HUMAN	E9PPU0	Epiplakin
4299	dirMS_041712_NAR_60mM_minus.5040.5050.3	3	13.7	1	2.11E+05		(R)MQNDT	(R)M/Q|N/D/T A E/N E/T T E/K/E E K(S)		82.83	632.9407	1896.813	-0.0052	-2.7	4.14	54214.9	5.66	HUMAN	C9J813	Caldesmon (Fragment)
4300	dirMS_041712_NAR_30mM_plus2.21242.21242.2	2	13.7	0	1.88E+05		(R)QTLMW	(R)Q T/L/M/W/S/A T/W/P K(E)		165.95	674.8389	1348.672	-0.0013	-1	8.75	61018.6	9.44	HUMAN	B4DLW8	Probable ATP-dependent RNA helicase DDX5
4301	dirMS_041712_NAR_50mM_plus2.18799.18799.3	3	13.7	0	4.20E+05		(R)AGLGSG	(R)A G L/G|S/G L/S L/S G L/V/H P E\L S R(S)		167.15	617.3412	1850.013	-0.004	-2.2	6.79	73325.2	9.19	HUMAN	F5H2A9	Very long-chain-specific acyl-CoA dehydrogenase, mitochondrial
4302	dirMS_041712_NAR_120mM_minus.19451.19486.3	3	13.69	1	1.83E+04		(K)RSIQFVI	(K)R S I Q\F\V/D\W|C/P T G F K(V)	C:Carbamidomethylation	161.3	580.9561	1740.853	0.0011	0.6	8.75	49012.3	4.9	HUMAN	A8MUB1	Tubulin alpha-4A chain
4303	dirMS_041712_NAR_20mM_minus.14643.14643.2	2	13.69	0	2.10E+05		(K)LAVEALS	(K)L A V E/A\L/S S/L/D\G D L A|G R(Y)		201.28	793.922	1586.838	-0.0017	-1.1	4.03	24286.7	4.89	HUMAN	G3V4N7	Creatine kinase B-type (Fragment)
4304	dirMS_041712_NAR_30mM_minus2.14874.14874.2	2	13.69	0	4.25E+05		(R)LCFEGLF	(R)L/C|F|E G L/R(S)	C:Carbamidomethylation	134.25	447.7317	894.45	0.0059	6.6	6	533778	5.74	HUMAN	Q15149	Plectin
4305	dirMS_041712_NAR_50mM_minus2.13670.13822.3	3	13.69	0	1.70E+06		(R)VAPDEH	(R)V A|P D E H P I L\L/T/E A/P L N/P K(I)		146.33	652.0252	1954.064	-0.0034	-1.7	4.65	42344.8	5.39	HUMAN	Q562R1	Beta-actin-like protein 2
4306	dirMS_041712_NAR_50mM_minus2.14171.14171.3	3	13.69	0	1.50E+05		(K)SIEIPRP\	(K)S I|E\I/P R\P V D G V/E V|P/G C G K(I)	C:Carbamidomethylation	148.28	636.9917	1908.985	-0.0242	-12.7	4.68	34243.5	4.88	HUMAN	K7ENG2	Splicing factor U2AF 65 kDa subunit
4307	dirMS_041712_NAR_30mM_plus2.6385.6779.2	2	13.69	0	1.71E+06		(R)MGANN	(R)M/G|A|N/N/L/E/R(M)		89.4	452.7228	904.431	0.0078	8.6	5.75	77799.7	9.09	HUMAN	P52272	Heterogeneous nuclear ribonucleoprotein M
4308	dirMS_041712_NAR_40mM_plus2.8043.8168.2	2	13.69	0	2.13E+05		(R)AVVIVDI	(R)A V|V|I/V/D/D/R(G)		108.22	443.7528	886.499	-0.0009	-1.1	4.21	23729.5	9.89	HUMAN	C9IZL7	Non-POU domain-containing octamer-binding protein (Fragment)
4309	dirMS_041712_NAR_50mM_plus2.20346.20346.3	3	13.69	1	3.35E+06		(R)TLAEIAK	(R)T L/A/E I/A K V E L/D N M/P L R(G)		174.87	604.9965	1812.989	-0.0138	-7.6	4.68	23729.5	9.89	HUMAN	C9IZL7	Non-POU domain-containing octamer-binding protein (Fragment)
4310	dirMS_041712_NAR_50mM_plus2.9821.9867.3	3	13.69	1	1.86E+05	M244m	(K)ILIANTG	(K)I/L|I/A/N T G m D T D/K I/K(I)	m:Oxidized methionine	118.97	516.9418	1532.835	15.9756	-12.5	5.96	57204.9	6.01	HUMAN	F5GWF6	T-complex protein 1 subunit beta
4311	dirMS_041712_NAR_30mM_minus2.13899.13899.2	2	13.68	0	1.66E+05		(K)NVIGLQI	(K)N V|I|G/L Q/M|G T N\R(G)		129.38	601.8183	1202.631	-0.0017	-1.4	9.75	22562.2	8.8	HUMAN	P37802	Transgelin-2
4312	dirMS_041712_NAR_30mM_minus2.15417.15458.2	2	13.68	0	5.21E+05		(R)YPVNSV	(R)Y/P|V|N/S V N I/L/K(A)		137.12	573.8282	1146.652	-0.0026	-2.3	8.59	44227.3	7.81	HUMAN	E7EQR6	T-complex protein 1 subunit alpha
4313	dirMS_041712_NAR_120mM_plus.20387.20387.3	3	13.68	0	3.09E+05		(R)INLWHL	(R)I/N\L/W|H L E I/T D R(S)		159.53	470.5889	1409.754	-0.0014	-1	5.32	52204.6	5.82	HUMAN	P63151	Serine/threonine-protein phosphatase 2A 55 kDa regulatory subunit B alpha isoform
4314	dirMS_041712_NAR_20mM_plus.17672.17872.2	2	13.68	0	2.00E+05		(R)YISPDQI	(R)Y I|S|P/D Q L/A\D L Y\K(S)		194.23	713.3667	1425.726	0.0001	0.1	4.21	47510.4	7.04	HUMAN	P06733	Alpha-enolase
4315	dirMS_041712_NAR_30mM_plus2.10624.10839.2	2	13.68	0	1.47E+06		(K)FYEQFSI	(K)F/Y|E/Q/F/S/K(N)		109.57	474.7276	948.446	0.0018	1.9	6	85057.6	4.94	HUMAN	P07900	Heat shock protein HSP 90-alpha
4316	dirMS_041712_NAR_30mM_plus2.16841.16991.2	2	13.68	0	4.42E+05		(R)DFFLAN.	(R)D F|F|L A/N/A/S/R(A)		141.87	520.7632	1040.516	0.0032	3	5.84	82516.1	5.49	HUMAN	P07384	Calpain-1 catalytic subunit
4317	dirMS_041712_NAR_40mM_plus2.7960.7960.2	2	13.68	0	3.26E+05		(R)MGYMD	(R)M/G\Y|M/D|P/R(E)		107.5	435.1865	869.364	0.0013	1.5	5.59	26419.2	5.1	HUMAN	H0Y9K7	Splicing factor, proline- and glutamine-rich (Fragment)
4318	dirMS_041712_NAR_50mM_plus2.18016.18016.3	3	13.68	1	1.54E+06		(R)ELIKVLEI	(R)E L\I K V\L E E A/N/Q/A/I/N|P K(L)		163.3	603.6741	1809.012	-0.0039	-2.2	4.79	73202	9.19	HUMAN	C9JMU5	Probable ATP-dependent RNA helicase DDX17
4319	dirMS_041712_NAR_50mM_plus2.18300.18300.3	3	13.68	0	2.22E+05		(R)IDILDSA	(R)I/D/I|L/D/S A L L R P G/R(I)		164.5	480.2828	1438.838	-0.0038	-2.6	5.96	44954.5	8.44	HUMAN	A8K3Z3	26S protease regulatory subunit 8
4320	dirMS_041712_NAR_50mM_plus2.19030.19030.3	3	13.68	0	5.50E+05		(R)GFGFITF	(R)G F/G/F/I/T/F T/N/P E H A S V A M R(A)		168.75	661.3258	1981.959	0.004	2	6.75	17170.1	8.86	HUMAN	P98179	Putative RNA-binding protein 3
4321	dirMS_041712_NAR_120mM_minus.11345.11480.3	3	13.67	0	2.31E+05		(R)GFCLPPI	(R)G F/C L/P/P/H C S/R(G)	C:Carbamidomethylation	116.42	410.858	1230.551	0.0088	7.1	9.75	14391.8	4.99	HUMAN	G3V461	Creatine kinase B-type (Fragment)
4322	dirMS_041712_NAR_120mM_minus.12569.12569.3	3	13.67	1	2.56E+05		(K)ARLENEI	(K)A R L E N E/I/A/T Y R(N)		122.33	445.9006	1335.701	-0.0143	-10.7	6.19	51115.6	4.9	HUMAN	O76014	Keratin, type I cuticular Ha7
4323	dirMS_041712_NAR_120mM_minus.12598.13043.3	3	13.67	1	3.62E+06		(K)VKLEAEI	(K)V K\L E\A\E|I|A/T/Y/R(R)		123.37	431.5783	1292.721	-0.0005	-0.4	6.11	43773.3	5.26	HUMAN	F8VZY9	Keratin, type I cytoskeletal 18
4324	dirMS_041712_NAR_40mM_minus2.15041.15042.3	3	13.67	0	2.40E+06		(K)ALESPER	(K)A/L|E/S/P E R P F\L/A I L/G G A K(V)		174.68	590.3353	1768.996	-0.0042	-2.4	6.19	41827.5	7	HUMAN	B7Z7A9	Phosphoglycerate kinase
4325	dirMS_041712_NAR_50mM_minus2.13195.13195.3	3	13.67	0	2.72E+04		(R)IYDAQN	(R)I/Y/D A Q N/E L/S A A T H/L/T S/K(L)		141.85	621.3116	1861.929	-0.0088	-4.7	5.32	15355.1	5.12	HUMAN	C9JAB0	DCC-interacting protein 13-alpha (Fragment)
4326	dirMS_041712_NAR_50mM_minus2.14483.14580.3	3	13.67	0	4.40E+05		(R)QVLIASI	(R)Q V|L/I/A/S/H L/P S Y E/L R(H)		150.95	542.6354	1625.901	-0.0093	-5.7	6.75	267260.7	5.95	HUMAN	Q13085	Acetyl-CoA carboxylase 1
4327	dirMS_041712_NAR_50mM_minus2.15500.15500.3	3	13.67	0	7.78E+04		(R)DLTPEHI	(R)D L/T/P E/H L/P/L L/R(N)		155.98	435.2497	1303.737	-0.0023	-1.8	5.32	38779.2	5.93	HUMAN	Q96C86	m7GpppX diphosphatase
4328	dirMS_041712_NAR_50mM_minus2.17037.17037.3	3	13.67	1	5.94E+04		(R)EFTRLEI(	(R)E F T R L E I/C/N|L/T/P D A L K(S)	C:Carbamidomethylation	164.93	640.6675	1919.989	-0.0016	-0.8	4.68	82516.1	5.49	HUMAN	P07384	Calpain-1 catalytic subunit
4329	dirMS_041712_NAR_60mM_minus.17095.17118.3	3	13.67	1	1.90E+05		(K)LLQDFFI	(K)L/L|Q/D/F F N G R D L/N/K(S)		155.28	527.277	1579.823	-0.0063	-4	5.96	60126	5.44	HUMAN	E7EP94	Heat shock 70 kDa protein 1A/1B
4330	dirMS_041712_NAR_60mM_minus.7085.7293.3	3	13.67	0	2.51E+06	M434m	(R)LESGmC	(R)L/E|S G m Q/N/M/S I/H/T/K(T)	m:Oxidized methionine	98.97	497.9023	1475.698	15.9942	-0.5	6.75	56607.4	5.37	HUMAN	F8VXB4	Keratin, type II cytoskeletal 8
4331	dirMS_041712_NAR_30mM_plus2.12778.12988.2	2	13.67	0	5.63E+05		(R)CNNVLY	(R)C/N|N|V|L/Y/I/R(G)	C:Carbamidomethylation	120.8	526.2726	1051.535	0.0026	2.5	8.75	9782.1	4.7	HUMAN	P62306	Small nuclear ribonucleoprotein F
4332	dirMS_041712_NAR_30mM_plus2.13832.13865.2	2	13.67	0	4.18E+04		(R)LNLNNT	(R)L/N|L|N|N T/V/L\S K(R)		127.13	558.323	1115.642	-0.0032	-2.8	8.75	172185.3	7.06	HUMAN	P07814	Bifunctional glutamate/proline--tRNA ligase
4333	dirMS_041712_NAR_30mM_plus2.19806.19806.2	2	13.67	0	2.35E+05		(K)GFAFVQ	(K)G F|A|F|V/Q/Y\V N E R(N)		157.57	665.3326	1329.659	-0.0007	-0.5	6	15791.7	9.22	HUMAN	B4DSU6	Heterogeneous nuclear ribonucleoproteins C1/C2
4334	dirMS_041712_NAR_30mM_plus2.20439.20439.2	2	13.67	0	6.53E+04		(K)SYELPD(	(K)S Y\E|L|P D G Q V I/T/I G N E\R(F)		160.92	895.9487	1790.892	-0.0018	-1	4.14	123097.1	5.83	HUMAN	A5A3E0	POTE ankyrin domain family member F
4335	dirMS_041712_NAR_40mM_plus2.11297.11297.3	3	13.67	0	2.83E+04		(K)AGVSIS\	(K)A G V|S/I/S V V/H G N L S E E A A K(Q)		132.63	589.9796	1767.924	0.0007	0.4	5.4	50949.8	9.28	HUMAN	P36551	Coproporphyrinogen-III oxidase, mitochondrial
4336	dirMS_041712_NAR_40mM_plus2.12885.12885.3	3	13.67	0	4.88E+03		(R)EQTLSP1	(R)E Q\T/L/S/P T I T/S G L H N\I A R(S)		142.85	613.3236	1837.977	-0.0204	-11.1	6.85	128523.4	7.06	HUMAN	B7ZL00	Protein transport protein Sec31A
4337	dirMS_041712_NAR_60mM_plus.11651.11671.3	3	13.67	1	2.93E+06		(K)KAEAGA	(K)K\A E A G\A G\S\A|T|E/F/Q/F/R(G)		118.23	523.9277	1569.766	0.003	1.9	6.14	18897.4	10.15	HUMAN	P46783	40S ribosomal protein S10
4338	dirMS_041712_NAR_30mM_minus2.13569.13569.2	2	13.66	0	5.78E+05		(K)TCTFILR(	(K)T/C|T/F/I/L R(G)	C:Carbamidomethylation	127.4	455.7458	910.481	0.0028	3.1	9.41	46550.7	7.81	HUMAN	B7Z1C9	Chaperonin containing TCP1, subunit 7 (Eta), isoform CRA_a
4339	dirMS_041712_NAR_30mM_minus2.7774.7774.2	2	13.66	0	1.29E+05		(K)VTQVDG	(K)V T/Q/V/D/G N/S P V/R(F)		96.45	586.3085	1171.607	0.0031	2.7	5.81	62195.8	5.63	HUMAN	B0YIW6	Archain 1, isoform CRA_a
4340	dirMS_041712_NAR_120mM_plus.20727.20727.4	4	13.66	2	3.87E+05		(R)TLAEIAK	(R)T\L|A E/I/A K V/E L D N M/P L R G K(Q)		161.73	500.2814	1998.105	-0.0015	-0.7	5.85	23729.5	9.89	HUMAN	C9IZL7	Non-POU domain-containing octamer-binding protein (Fragment)
4341	dirMS_041712_NAR_20mM_plus.10468.10468.2	2	13.66	0	1.10E+05	M267m	(R)LITLEEEr	(R)L/I|T\L/E/E E m/T K(Y)	m:Oxidized methionine	145.17	611.8175	1206.629	15.9991	3.4	4.25	51524.1	4.82	HUMAN	F5H8J2	Protein disulfide-isomerase
4342	dirMS_041712_NAR_20mM_plus.11344.11344.2	2	13.66	0	1.74E+05		(K)VGLQVV	(K)V/G/L Q V/V/A V K(A)		150.73	456.7977	912.588	0.0004	0.5	8.72	61224.7	5.7	HUMAN	P10809	60 kDa heat shock protein, mitochondrial
4343	dirMS_041712_NAR_20mM_plus.13960.13960.2	2	13.66	0	6.90E+04		(K)IEVLEEEI	(K)I E|V|L/E E/E L/R(L)		168.03	565.3132	1129.61	0.0092	8.1	4.09	334221.3	6.44	HUMAN	P15924	Desmoplakin
4344	dirMS_041712_NAR_20mM_plus.8296.8320.2	2	13.66	0	9.05E+04		(K)GGEIQP'	(K)G G\E|I|Q/P V\S/V|K(V)		127.42	507.2841	1013.563	-0.0017	-1.6	6	10803.5	9.82	HUMAN	B8ZZL8	10 kDa heat shock protein, mitochondrial
4345	dirMS_041712_NAR_30mM_plus2.16703.16703.2	2	13.66	0	4.62E+05		(K)GCSFLPI	(K)G C\S\F/L|P D/P Y Q K(Q)	C:Carbamidomethylation	140.67	656.306	1311.604	0.0009	0.7	5.83	63688.6	5.11	HUMAN	B1AVU8	Prosaposin
4346	dirMS_041712_NAR_30mM_plus2.17856.17895.2	2	13.66	0	1.40E+05		(R)NELSGA	(R)N E|L|S|G/A/L/T/G L T R(V)		146.73	616.3341	1231.664	-0.0032	-2.6	6	71231.7	9.22	HUMAN	E7ERI3	Threonine--tRNA ligase, cytoplasmic
4347	dirMS_041712_NAR_30mM_plus2.19323.19323.2	2	13.66	0	2.98E+05		(K)TVDNFV	(K)T V|D|N F/V/A/L A T G E K(G)		155	682.8539	1364.706	-0.0051	-3.7	4.37	23799.1	9.48	HUMAN	P23284	Peptidyl-prolyl cis-trans isomerase B
4348	dirMS_041712_NAR_30mM_plus2.22077.22077.2	2	13.66	0	1.51E+05		(K)GLSSLLC	(K)G L|S/S/L/L C N F/T/K(S)	C:Carbamidomethylation	171.68	620.3199	1239.64	-0.0077	-6.2	8.75	470342.8	6.83	HUMAN	E7EUY0	DNA-dependent protein kinase catalytic subunit
4349	dirMS_041712_NAR_30mM_plus2.8912.8912.2	2	13.66	0	1.86E+05		(R)QPLGAT	(R)Q P/L|G/A/T S G A/S L/K(T)		100.28	565.3227	1129.621	0.017	15	8.75	65763.5	9.81	HUMAN	Q9HCD5	Nuclear receptor coactivator 5
4350	dirMS_041712_NAR_40mM_plus2.14071.14071.3	3	13.66	0	1.11E+05		(R)LVLEVA(	(R)L V|L/E/V A/Q/H L G E S T V R(T)		149.15	550.9762	1650.917	-0.0033	-2	5.4	55307.5	5.25	HUMAN	F8VPV9	ATP synthase subunit beta
4351	dirMS_041712_NAR_40mM_plus2.17731.17731.3	3	13.66	1	1.96E+05		(R)LKLPSIPI	(R)L K L P S I\P L/V|P V/S A Q K(R)		170.73	530.671	1589.999	-0.0004	-0.3	10	34958.1	9.42	HUMAN	Q15785	Mitochondrial import receptor subunit TOM34
4352	dirMS_041712_NAR_50mM_plus2.16556.16557.3	3	13.66	1	3.33E+05		(K)LLTGELL	(K)L L|T/G E L/L/P T D/G M I R/K(H)		155.22	552.9815	1656.935	-0.0054	-3.2	6.07	73070	7.16	HUMAN	Q75MJ1	ATP-binding cassette sub-family F member 2
4353	dirMS_041712_NAR_60mM_plus.10544.10650.3	3	13.66	1	1.05E+06		(K)MADEAI	(K)M/A|D/E/A I\L Q E R E/R(G)		113	487.578	1460.716	0.0032	2.2	4.41	65763.5	9.81	HUMAN	Q9HCD5	Nuclear receptor coactivator 5
4354	dirMS_041712_NAR_30mM_minus2.12445.12445.2	2	13.65	0	4.97E+05		(K)GDYPLE.	(K)G D/Y|P/L/E/A V R(M)		122.13	510.2598	1019.516	-0.0033	-3.2	4.37	18466.1	5.78	HUMAN	H3BSU3	Pyruvate kinase (Fragment)
4355	dirMS_041712_NAR_30mM_minus2.15557.15557.2	2	13.65	0	1.82E+05		(K)EILFYDR	(K)E I|L/F/Y D/R(I)		137.7	478.2479	955.488	0.0002	0.2	4.37	59953.2	6.14	HUMAN	G3V1R4	NOL1/NOP2/Sun domain family, member 2, isoform CRA_b

4356	dirMS_041712_NAR_40mM_minus2.12653.12727.3	3	13.65	1	2.01E+05		(K)DVLFLKD	(K)D V|L|F/L/K/D C V/G P E V E K(A)	C:Carbamidomethylation	158.43	583.3021	1747.893	-0.0018	-1	4.32	41827.5	7	HUMAN	B7Z7A9	Phosphoglycerate kinase
4357	dirMS_041712_NAR_50mM_minus2.6779.6779.3	3	13.65	1	2.52E+05		(R)ASVDTK	(R)A S V/D T K E A E/G A|P Q/V E A/G K(R)		104.8	596.2904	1786.882	-0.0251	-14	4.41	54214.9	5.66	HUMAN	C9J813	Caldesmon (Fragment)
4358	dirMS_041712_NAR_60mM_minus.17199.17199.3	3	13.65	1	3.11E+05		(K)DANAKL	(K)D A|N A K L\S E\L/E|A/A/L/Q R(A)		156.03	543.6239	1628.86	-0.0031	-1.9	4.68	56607.4	5.37	HUMAN	F8VXB4	Keratin, type II cytoskeletal 8
4359	dirMS_041712_NAR_120mM_plus.13958.13958.3	3	13.65	2	7.22E+04		(K)AFAAQE	(K)A/F|A|A/Q E D L E K T K E E/L K(T)		126.38	617.3224	1849.954	-0.0015	-0.8	4.58	71218.8	6.3	HUMAN	A7YIJ8	Radixin
4360	dirMS_041712_NAR_20mM_plus.17838.17871.2	2	13.65	0	1.98E+05		(R)SLVPAAI	(R)S L|V|P/A/A E L/L E/S R(V)		194.77	642.8613	1284.716	-0.0005	-0.4	4.53	533778	5.74	HUMAN	Q15149	Plectin
4361	dirMS_041712_NAR_20mM_plus.7325.7325.2	2	13.65	0	2.82E+05		(K)TGQAPG	(K)T G Q|A|P G Y|S Y/T A\A N K(N)		119.5	714.8394	1428.675	-0.0039	-2.7	8.16	11447.2	9.81	HUMAN	C9JFR7	Cytochrome c (Fragment)
4362	dirMS_041712_NAR_50mM_plus2.14065.14065.3	3	13.65	0	1.72E+06	M457m	(R)mGPLGL	(R)m|G/P/L/G L D H/M/A\S S I E/R(M)	m:Oxidized methionine	142.67	543.9304	1613.777	15.9992	2.6	5.3	77799.7	9.09	HUMAN	P52272	Heterogeneous nuclear ribonucleoprotein M
4363	dirMS_041712_NAR_60mM_plus.11432.11626.3	3	13.65	1	1.47E+06		(R)GASKEIL	(R)G A/S K E I\L|S/E/V/E/R(N)		117.95	439.9069	1317.701	0.0053	4	4.79	56186.7	5.47	HUMAN	B4DUR8	T-complex protein 1 subunit gamma
4364	dirMS_041712_NAR_120mM_minus.8247.8247.3	3	13.64	1	5.91E+05		(K)SSGGREI	(K)S/S/G G R E D L E|S|S/G/L Q\R(R)		99.45	526.5882	1577.751	-0.0014	-0.9	4.68	11625.5	8.01	HUMAN	B8ZZT7	Hematological and neurological-expressed 1 protein
4365	dirMS_041712_NAR_30mM_minus2.7129.7129.2	2	13.64	0	3.86E+05		(K)QQTLEA	(K)Q\Q/T|L/E/A/E E/A K(R)		93.4	573.7846	1146.564	-0.0018	-1.6	4.25	76925.9	4.46	HUMAN	A8MV58	Drebrin
4366	dirMS_041712_NAR_50mM_minus2.17232.17445.3	3	13.64	0	1.05E+06	M195m	(R)LHFFmP	(R)L H F\F m|P G|F|A/P L T S R(G)	m:Oxidized methionine	167.33	546.2872	1620.835	16.0115	10.1	9.75	42230.8	4.77	HUMAN	A6NKZ8	Putative tubulin beta chain-like protein ENSP00000290377
4367	dirMS_041712_NAR_60mM_minus.13930.13930.3	3	13.64	1	2.82E+05		(R)SLIANLA	(R)S L/I/A/N L|A A/A/N C Y K K(E)	C:Carbamidomethylation	137.85	512.9419	1536.82	-0.0091	-5.9	9.7	36978.6	6.33	HUMAN	B7Z800	Adenosine kinase
4368	dirMS_041712_NAR_120mM_plus.20207.20432.3	3	13.64	1	3.71E+06		(K)AAIDWF	(K)A/A|I/D|W/F D/G K E F H\G N I I/K(V)		158.78	654.3334	1960.992	-0.0059	-3	5.38	49181	4.93	HUMAN	K7EPT6	TATA-binding protein-associated factor 2N
4369	dirMS_041712_NAR_30mM_plus2.12962.13402.2	2	13.64	1	5.17E+06		(K)KITIADC	(K)K\I T\I|A|D\C\G\Q|L|E(-)	C:Carbamidomethylation	122.8	624.3183	1247.63	-0.0007	-0.6	4.75	18240.4	7.96	HUMAN	P62937	Peptidyl-prolyl cis-trans isomerase A
4370	dirMS_041712_NAR_30mM_plus2.14483.14723.2	2	13.64	0	6.60E+05		(R)DLAPIGI	(R)D/L|A|P/I/G I/R(V)		129.75	427.7575	854.509	-0.0017	-2	5.84	27150.8	7.95	HUMAN	Q99714	3-hydroxyacyl-CoA dehydrogenase type-2
4371	dirMS_041712_NAR_30mM_plus2.18933.18933.2	2	13.64	0	5.13E+04		(R)MGPGIG	(R)M/G/P G\I/G/A I L E R(S)		152.52	557.3091	1113.609	0.0024	2.2	5.75	14175.1	9.89	HUMAN	H0YKS1	Myelin expression factor 2 (Fragment)
4372	dirMS_041712_NAR_30mM_plus2.22414.22414.2	2	13.64	0	2.83E+05		(K)CMPTFC	(K)C M/P/T F Q F/F K(K)	C:Carbamidomethylation	172.85	603.2777	1205.548	-0.0001	-0.1	8.75	9680	5.77	HUMAN	B1ALW1	Thioredoxin
4373	dirMS_041712_NAR_30mM_plus2.6031.6047.2	2	13.64	0	1.59E+05		(R)LQDTSS	(R)L/Q|D|T/S S/Y/A K(I)		85.92	506.7507	1012.495	-0.0004	-0.4	5.83	334221.3	6.44	HUMAN	P15924	Desmoplakin
4374	dirMS_041712_NAR_60mM_plus.6752.6752.3	3	13.64	1	3.47E+05		(K)TQMTSA	(K)T Q|M/T/S A V R/E/P K(L)		93.58	416.5533	1247.641	0.0041	3.3	8.41	470342.8	6.83	HUMAN	E7EUY0	DNA-dependent protein kinase catalytic subunit
4375	dirMS_041712_NAR_60mM_plus.8930.9162.3	3	13.64	0	1.59E+06		(K)EFHLNES	(K)E F H L N\E/S/G/D/P/S/S K(S)		105.52	482.8882	1446.65	0.0005	0.3	4.65	30992.3	4.14	HUMAN	B2REB8	Protein SET
4376	dirMS_041712_NAR_60mM_plus.9422.9422.3	3	13.64	1	4.42E+05		(K)CLDENLI	(K)C L|D/E/N L/E D A/S Q/C K K(L)	C:Carbamidomethylation	107.22	570.5876	1709.747	0.0013	0.8	4.32	14458.2	9.22	HUMAN	Q5JTJ3	Cytochrome c oxidase assembly factor 6 homolog
4377	dirMS_041712_NAR_120mM_minus.14124.14350.3	3	13.63	2	1.13E+06		(K)VLAGDK	(K)V/L|A|G/D/K/N F I T A E E/L R/R(E)		131.63	611.338	1832.003	-0.003	-1.7	6.15	80254.2	5.05	HUMAN	D6PXK4	Alpha actinin 4 short isoform
4378	dirMS_041712_NAR_30mM_minus2.5060.5060.2	2	13.63	0	3.65E+05		(R)TDTGEP	(R)T D|T/G/E P M G/R(G)		81.17	482.2145	963.42	0.0017	1.8	4.37	85057.6	4.94	HUMAN	P07900	Heat shock protein HSP 90-alpha
4379	dirMS_041712_NAR_30mM_minus2.8427.8427.2	2	13.63	0	1.09E+05		(K)CAMTAL	(K)C/A|M/T A/L/S S K(L)	C:Carbamidomethylation	100.45	484.7299	968.454	-0.0014	-1.5	8.75	46550.7	7.81	HUMAN	B7Z1C9	Chaperonin containing TCP1, subunit 7 (Eta), isoform CRA_a
4380	dirMS_041712_NAR_40mM_minus2.11267.11627.3	3	13.63	0	8.05E+05		(R)TIQVENS	(R)T I|Q|V/E/N\S H L\I/L/T/G A G A L N K(V)		151	660.3663	1979.092	-0.0077	-3.9	6.41	267260.7	5.95	HUMAN	Q13085	Acetyl-CoA carboxylase 1
4381	dirMS_041712_NAR_60mM_minus.13658.13658.3	3	13.63	0	2.50E+05		(K)VSHVST	(K)V S H V\S T G G G\A|S|L/E/L L/E/G K(V)		136.38	580.9746	1740.913	-0.0034	-2	5.4	41827.5	7	HUMAN	B7Z7A9	Phosphoglycerate kinase
4382	dirMS_041712_NAR_60mM_minus.7841.8056.3	3	13.63	1	5.85E+05		(K)IQDKEGI	(K)I Q\D K E G|I|P|P D/Q Q R(L)		103.83	508.5978	1523.781	-0.0024	-1.6	4.56	17271.6	6.75	HUMAN	B4DV12	Ubiquitin
4383	dirMS_041712_NAR_120mM_plus.8432.8432.3	3	13.63	1	1.83E+05		(K)ALSRQEI	(K)A L/S R Q E|M/Q|E/V/Q S S R(S)		97.45	550.2746	1648.807	0.0021	1.3	6.19	37486.1	9.03	HUMAN	P22626	Heterogeneous nuclear ribonucleoproteins A2/B1
4384	dirMS_041712_NAR_30mM_plus2.14360.14360.2	2	13.63	0	5.73E+05		(K)CCSGAII	(K)C/C|S/G A/I I/V/L T/K(S)	C:Carbamidomethylation	128.2	611.3189	1221.633	-0.0025	-2	8.75	58506.4	8.38	HUMAN	P14618	Pyruvate kinase isozymes M1/M2
4385	dirMS_041712_NAR_40mM_plus2.6280.6387.2	2	13.63	0	2.04E+05		(K)TEISEMI	(K)T/E|I|S/E M/N/R(N)		96.33	490.2292	979.451	-0.0002	-0.2	4.53	37296.3	5.3	HUMAN	F8VU64	Keratin, type II cytoskeletal 8
4386	dirMS_041712_NAR_50mM_plus2.12325.12325.3	3	13.63	1	4.91E+05		(K)ILDSVGII	(K)I/L|D/S V/G I E/A D D D R\L N K(V)		132.85	591.6385	1772.902	-0.0015	-0.9	4.04	11664.7	4.42	HUMAN	P05387	60S acidic ribosomal protein P2
4387	dirMS_041712_NAR_50mM_plus2.7568.7773.3	3	13.63	0	1.97E+06		(K)EAPPME	(K)E A\P/P/M/E/K/P E V V/K(T)		106.32	451.9073	1353.708	-0.0009	-0.7	4.79	14798.1	10.35	HUMAN	K7EJ78	40S ribosomal protein S15 (Fragment)
4388	dirMS_041712_NAR_60mM_plus.10343.10901.3	3	13.63	1	2.26E+06		(K)TAVCDIF	(K)T/A|V/C D/I/P/P R G L K(M)	C:Carbamidomethylation	115.63	442.9125	1326.72	0.0031	2.3	8.41	50199.4	4.75	HUMAN	A6NNZ2	Tubulin beta-8 chain-like protein LOC260334
4389	dirMS_041712_NAR_120mM_minus.13512.13512.3	3	13.62	2	9.83E+05		(R)AQKDEE	(R)A Q|K/D E/E/K\M|E I/Q\E/I Q L K(E)		128.13	654.0058	1960.006	-0.0027	-1.4	4.58	31923.6	4.85	HUMAN	B7Z596	Tropomyosin alpha-1 chain
4390	dirMS_041712_NAR_30mM_minus2.6822.6822.2	2	13.62	0	1.51E+04		(K)AMEAVA	(K)A M|E A|V/A|A Q G/K(A)		91.35	488.2495	975.493	-0.0011	-1.1	6.05	28936.8	9.22	HUMAN	P15259	Phosphoglycerate mutase 2
4391	dirMS_041712_NAR_40mM_minus2.12130.12130.3	3	13.62	0	3.21E+05		(K)IAILTCPF	(K)I A/I L/T/C|P F E/P P K/P K(T)	C:Carbamidomethylation	155.3	537.6382	1610.898	0.0026	1.6	8.59	49982	5.86	HUMAN	B4DYD8	T-complex protein 1 subunit epsilon
4392	dirMS_041712_NAR_50mM_minus2.10228.10228.3	3	13.62	0	1.39E+05		(K)VGSLDN	(K)V/G|S/L/D N V/G H L/P A G G A V K(I)		126.85	530.9592	1590.86	0.0032	2	6.71	26233.5	10.95	HUMAN	B4DSQ1	Microtubule-associated protein
4393	dirMS_041712_NAR_60mM_minus.15466.15466.3	3	13.62	1	2.23E+05		(R)GPIKFN	(R)G P/I/K F N V|W/D/T A/G Q E\K(F)		146.48	563.9575	1689.86	-0.0015	-0.9	6.07	26394.9	6.5	HUMAN	B5MDF5	GTP-binding nuclear protein Ran
4394	dirMS_041712_NAR_20mM_plus.10938.11020.2	2	13.62	0	1.17E+05		(K)EITALAP	(K)E I|T|A|L|A P S/T M K(I)		148.15	581.3129	1161.618	0.0001	0.1	6.1	32616.5	5.18	HUMAN	A6NL76	Actin, alpha skeletal muscle
4395	dirMS_041712_NAR_20mM_plus.13288.13408.2	2	13.62	0	4.79E+05	M148m	(R)DLTDYLr	(R)D L|T/D/Y/L/m/K(I)	m:Oxidized methionine	164.72	507.7439	998.486	15.9942	-0.7	4.21	37482.1	5.36	HUMAN	E9PG30	Actin, gamma-enteric smooth muscle
4396	dirMS_041712_NAR_20mM_plus.7792.7792.3	3	13.62	0	1.06E+05		(K)AQAAAF	(K)A Q A\A A/P\A S V|P A/Q A/P K(R)		123.6	459.9204	1377.748	-0.0018	-1.3	8.8	17808.8	11.77	HUMAN	P47914	60S ribosomal protein L29
4397	dirMS_041712_NAR_30mM_plus2.6333.6333.2	2	13.62	0	4.73E+04	M924m	(K)FGDSNT	(K)F/G\D/S/N T/V/m R(F)	m:Oxidized methionine	87.4	521.7347	1026.467	15.9948	-0.1	5.84	334221.3	6.44	HUMAN	P15924	Desmoplakin
4398	dirMS_041712_NAR_40mM_plus2.14863.14863.3	3	13.62	1	2.18E+04		(K)VTADVII	(K)V/T|A/D|V/I/N A/A E/K L Q V V G R(A)		154.18	595.0062	1783.007	-0.0032	-1.8	6.04	57391.2	6.29	HUMAN	O43175	D-3-phosphoglycerate dehydrogenase
4399	dirMS_041712_NAR_40mM_plus2.17403.17403.3	3	13.62	1	2.00E+05		(R)GDSVIV	(R)G D/S V/I/V V/L R N P L/I A G K(-)		168.75	551.0007	1650.99	-0.0026	-1.6	8.75	6144.3	10.23	HUMAN	K7EJB5	Small nuclear ribonucleoprotein Sm D2
4400	dirMS_041712_NAR_50mM_plus2.20514.20514.3	3	13.62	1	1.87E+04		(K)SSVLENF	(K)S S|V|L/E N/F\V/G R D/F L P\R(G)		176.38	579.3128	1735.913	0.0113	6.5	5.79	73288.1	8.96	HUMAN	E5RHK8	Dynamin-3
4401	dirMS_041712_NAR_50mM_plus2.21533.21533.3	3	13.62	0	8.95E+04		(K)GAEILEV	(K)G A|E|I/L E V/L H/S L/P A V R(Q)		183.4	535.3092	1603.917	-0.0036	-2.2	5.4	52378.2	6.32	HUMAN	C9IZE4	26S proteasome non-ATPase regulatory subunit 6
4402	dirMS_041712_NAR_60mM_plus.12081.12218.3	3	13.62	1	1.99E+06		(R)MQKEIT	(R)M Q\K E I T/A|L|A/P/S T M K(I)		120.7	516.9402	1548.812	-0.0064	-4.1	8.35	32616.5	5.18	HUMAN	A6NL76	Actin, alpha skeletal muscle
4403	dirMS_041712_NAR_20mM_minus.11276.11276.2	2	13.61	0	7.93E+04	M148m	(R)DLTDYLr	(R)D L|T/D Y L/m K(I)	m:Oxidized methionine	160.1	507.7443	998.486	15.995	0.1	4.21	37482.1	5.36	HUMAN	E9PG30	Actin, gamma-enteric smooth muscle
4404	dirMS_041712_NAR_20mM_minus.13773.13847.2	2	13.61	0	2.58E+05		(R)IVLLDSS	(R)I/V|L|L/D S S/L\E/Y/K(K)		189.27	640.3594	1279.714	-0.0029	-2.2	4.37	56186.7	5.47	HUMAN	B4DUR8	T-complex protein 1 subunit gamma
4405	dirMS_041712_NAR_30mM_minus2.18093.18114.2	2	13.61	0	2.92E+05		(K)LLCGLLA	(K)L L|C/G L/L/A E/R(L)	C:Carbamidomethylation	152.05	522.7963	1044.587	-0.0017	-1.6	6	12647.2	8.07	HUMAN	P14174	Macrophage migration inhibitory factor
4406	dirMS_041712_NAR_40mM_minus2.16016.16016.3	3	13.61	0	1.60E+06	M166m	(K)VLDFEHI	(K)V/L/D/F E H F L/P m L/Q/T V/A K(N)	m:Oxidized methionine	187.72	635.3376	1888.004	15.9945	-0.2	5.32	26991.7	5.01	HUMAN	B7Z6Z4	Myosin light polypeptide 6
4407	dirMS_041712_NAR_50mM_minus2.12480.12504.3	3	13.61	1	3.32E+05		(K)LYEQILA	(K)L/Y|E/Q I/L|A|E N E K L K(A)		139.12	530.9592	1590.874	-0.0107	-6.7	4.79	22046.7	5.8	HUMAN	H0YIM2	Protein phosphatase 1 regulatory subunit 12A (Fragment)
4408	dirMS_041712_NAR_50mM_minus2.16668.16668.3	3	13.61	1	2.61E+05		(R)SELELTL	(R)S E|L E/L T/L G K/L E Q V R(S)		163.6	538.9725	1614.906	-0.0032	-2	4.78	533778	5.74	HUMAN	Q15149	Plectin
4409	dirMS_041712_NAR_50mM_minus2.8826.8826.3	3	13.61	1	2.18E+05		(K)VCGDSD	(K)V/C|G D S D K G F V/V/I/N Q/K(G)	C:Carbamidomethylation	118.03	555.9474	1665.826	0.0012	0.7	5.93	55323	6.02	HUMAN	B4DPJ8	T-complex protein 1 subunit zeta
4410	dirMS_041712_NAR_60mM_minus.7822.7822.3	3	13.61	0	1.10E+05		(K)LFQECCI	(K)L/F/Q/E C C/P/H S T D/R(V)	C:Carbamidomethylation	103.25	517.2229	1549.652	0.0019	1.2	5.32	51260.6	5.39	HUMAN	P61978	Heterogeneous nuclear ribonucleoprotein K
4411	dirMS_041712_NAR_120mM_plus.13271.13335.4	4	13.61	2	1.88E+06		(R)ELNITAA	(R)E L/N I/T/A/A K E I/E V/G G G R K(A)		123.02	447.0018	1784.987	-0.0011	-0.6	6.33	21311.5	10.28	HUMAN	B5MCP9	40S ribosomal protein S7
4412	dirMS_041712_NAR_20mM_plus.14461.14461.2	2	13.61	0	1.33E+05		(R)FIDTTLA	(R)F I|D T T/L/A/I T S R(F)		171.52	619.3413	1237.679	-0.0034	-2.7	5.84	11439.8	9.3	HUMAN	G3XAN8	Mitochondrial import inner membrane translocase subunit Tim8 B
4413	dirMS_041712_NAR_20mM_plus.18644.18644.3	3	13.61	0	5.11E+05		(K)LAVEALS	(K)L A V E\A\L S/S L/D/G D/L/A G R(Y)		200.45	529.6164	1586.838	-0.0038	-2.4	4.03	24286.7	4.89	HUMAN	G3V4N7	Creatine kinase B-type (Fragment)
4414	dirMS_041712_NAR_40mM_plus2.12300.12300.2	2	13.61	0	3.90E+04		(R)YIELLTR(	(R)Y I/E/L/L/T R(S)		138.3	454.2649	907.525	-0.0022	-2.4	6	334221.3	6.44	HUMAN	P15924	Desmoplakin
4415	dirMS_041712_NAR_40mM_plus2.5408.5408.2	2	13.61	0	4.44E+04		(R)SQESGY	(R)S Q|E|S|G|Y/Y D R(M)		88.63	552.733	1104.459	-0.0005	-0.5	4.37	100478.9	5.71	HUMAN	A8MXP9	Matrin-3
4416	dirMS_041712_NAR_50mM_plus2.13703.13703.3	3	13.61	0	3.05E+05		(K)ASLASLP	(K)A S L A/S L/P/P L/H V A K(L)		140.47	435.2622	1303.773	-0.0012	-0.9	8.8	141626	5.5	HUMAN	A8MY36	Dynactin subunit 1
4417	dirMS_041712_NAR_60mM_plus.12103.12146.3	3	13.61	1	3.93E+05		(K)FADLSEA	(K)F/A|D|L|S/E/A A N R N N D/A L R(Q)		120.47	592.9581	1776.862	-0.0026	-1.5	4.56	49709.6	5.19	HUMAN	B0YJC4	Vimentin
4418	dirMS_041712_NAR_60mM_minus.11524.11524.3	3	13.6	1	2.94E+05		(R)VFDKEG	(R)V/F/D K E G\N G T|V/M/G/A E I R(H)		124.35	574.9539	1722.848	-0.0008	-0.5	4.68	26991.7	5.01	HUMAN	B7Z6Z4	Myosin light polypeptide 6
4419	dirMS_041712_NAR_60mM_minus.12121.12641.3	3	13.6	0	2.01E+07		(R)NLDIERF	(R)N/L|D|I/E R/P T Y/T/N L N R(L)		129.65	573.6304	1718.882	-0.0054	-3.1	6.07	49012.3	4.9	HUMAN	A8MUB1	Tubulin alpha-4A chain
4420	dirMS_041712_NAR_120mM_plus.8766.8766.3	3	13.6	0	3.36E+05		(R)FACHSA	(R)F/A/C/H S|A/S/L/T\V/R(N)	C:Carbamidomethylation	99.38	416.8772	1248.615	0.0017	1.3	9.75	23729.5	9.89	HUMAN	C9IZL7	Non-POU domain-containing octamer-binding protein (Fragment)
4421	dirMS_041712_NAR_20mM_plus.7459.7482.2	2	13.6	0	2.44E+05		(R)TVGIVGI	(R)T/V|G|I|V/G N Q P/K(V)		119.85	506.7922	1012.579	-0.0014	-1.4	8.41	61363	8.73	HUMAN	C9JQS9	Propionyl-CoA carboxylase beta chain, mitochondrial
4422	dirMS_041712_NAR_30mM_plus2.16002.16002.2	2	13.6	0	4.81E+05		(R)MGPNIY	(R)M G/P/N|I/Y E L/R(T)		136.78	546.7844	1092.551	0.0109	10	5.75	8641.9	9.77	HUMAN	C9JDV8	Protein NipSnap homolog 1 (Fragment)
4423	dirMS_041712_NAR_30mM_plus2.9769.9769.2	2	13.6	0	1.36E+06		(R)QLSSGV	(R)Q/L|S/S/G/V/S E/I R(H)		104.9	538.2855	1075.574	-0.0105	-9.8	6	20576.9	9.84	HUMAN	F8WE04	Heat shock protein beta-1
4424	dirMS_041712_NAR_40mM_plus2.12876.12899.3	3	13.6	0	3.13E+05		(K)VVEIAPA	(K)V V|E|I/A/P A A/H L/D/P Q L R(T)		142.63	543.6417	1628.912	-0.0013	-0.8	5.32	58266.4	9.46	HUMAN	B4DN00	Pyruvate carboxylase, mitochondrial
4425	dirMS_041712_NAR_50mM_plus2.20893.20893.3	3	13.6	1	7.61E+04		(K)TSAQVV	(K)T S/A/Q|V/V/L D G\L V D K I G D V K(C)		178.37	619.6814	1857.033	-0.0031	-1.7	4.43	227202.2	8.26	HUMAN	Q14008	Cytoskeleton-associated protein 5
4426	dirMS_041712_NAR_50mM_plus2.7792.7792.3	3	13.6	0	2.10E+05		(R)DGQVIN	(R)D G Q|V/I/N E T S Q/H/H D D L|E(-)		106.82	612.9457	1836.8	0.0231	12.6	4.28	53707.8	5.06	HUMAN	P08670	Vimentin
4427	dirMS_041712_NAR_120mM_minus.7359.7509.2	2	13.59	1	1.94E+06		(R)LSVDYGI	(R)L/S|V/D|Y/G K K(S)		94.83	455.2555	909.504	-0.0003	-0.3	8.5	49012.3	4.9	HUMAN	A8MUB1	Tubulin alpha-4A chain
4428	dirMS_041712_NAR_20mM_minus.14719.14719.2	2	13.59	0	9.82E+05		(K)EIIDLVLI	(K)E I/I/D/L/V/L D/R(I)		202.93	543.3133	1085.62	-0.0008	-0.7	4.03	58642	4.95	HUMAN	F5H5D3	Tubulin alpha-1C chain
4429	dirMS_041712_NAR_30mM_minus2.10723.10791.2	2	13.59	0	6.30E+05		(K)GFSLESC	(K)G F|S|L/E/S C/R(S)	C:Carbamidomethylation	112.08	478.2237	955.43	0.0099	10.4	6	82516.1	5.49	HUMAN	P07384	Calpain-1 catalytic subunit
4430	dirMS_041712_NAR_30mM_minus2.12009.12075.2	2	13.59	0	2.55E+05		(K)YFDSGD	(K)Y/F|D|S/G D/Y N/M A K(A)		118.87	655.7712	1310.536	-0.0007	-0.5	4.21	15633.5	7.82	HUMAN	A6NMQ3	Alpha-endosulfine
4431	dirMS_041712_NAR_30mM_minus2.16654.16806.3	3	13.59	0	7.83E+05		(R)ASLEAAI	(R)A S\L E A\A|I/A/D/A/E/Q R(G)		144.25	448.8965	1344.675	-0.0004	-0.3	4.14	56607.4	5.37	HUMAN	F8VXB4	Keratin, type II cytoskeletal 8
4432	dirMS_041712_NAR_30mM_minus2.17182.17182.2	2	13.59	0	2.69E+05		(R)LLNTFLE	(R)L/L|N|T/F L E R(L)		146.93	503.2871	1005.573	-0.0058	-5.8	6	535136.6	6.01	HUMAN	Q14204	Cytoplasmic dynein 1 heavy chain 1
4433	dirMS_041712_NAR_30mM_minus2.19559.19616.2	2	13.59	0	4.72E+05		(R)ASLSLW	(R)A S/L/S/L/W/Q/A/M E/K(G)		162.75	632.3234	1263.64	-0.0007	-0.5	6.05	266161	5.68	HUMAN	E9PPU0	Epiplakin
4434	dirMS_041712_NAR_20mM_plus.15338.15338.2	2	13.59	0	1.68E+04	M73m	(R)AILVDLE	(R)A I/L\V|D\L/E/P G T m D S V R(S)	m:Oxidized methionine	177.98	816.4181	1615.836	15.9929	-1.2	4.03	27549.9	4.39	HUMAN	E9PBJ4	Tubulin beta chain
4435	dirMS_041712_NAR_20mM_minus.10747.10747.2	2	13.58	0	4.12E+05		(K)DVNAAL	(K)D V|N|A/A I/A A\I K(T)		155.97	493.287	985.568	-0.0009	-1	5.84	49012.3	4.9	HUMAN	A8MUB1	Tubulin alpha-4A chain
4436	dirMS_041712_NAR_20mM_minus.11554.11554.2	2	13.58	0	1.40E+05	M98m	(R)TLTIVDT	(R)T L/T|I/V/D/T/G I G m\T K(A)	m:Oxidized methionine	163.17	683.3669	1349.735	15.992	-2.1	5.5	20151.2	4.58	HUMAN	G3V2J8	Heat shock protein HSP 90-alpha (Fragment)
4437	dirMS_041712_NAR_60mM_minus.11505.11507.3	3	13.58	1	1.31E+05		(R)LSFDKDA	(R)L/S|F D K/D/A|M/V A R(A)		124.22	418.2174	1252.635	0.0022	1.8	5.96	35556.4	9.3	HUMAN	F2Z393	Transaldolase
4438	dirMS_041712_NAR_20mM_plus.8107.8107.2	2	13.58	0	2.09E+05		(R)FGQGGA	(R)F G Q/G/G/A|G/P V G G Q G P R(G)		125.22	671.3361	1341.666	-0.0009	-0.7	9.75	76262.2	9.53	HUMAN	P23246	Splicing factor, proline- and glutamine-rich
4439	dirMS_041712_NAR_20mM_plus.8872.9144.2	2	13.58	0	1.23E+06	M239m	(K)EITALAP	(K)E I|T|A|L|A|P/S T m K(I)	m:Oxidized methionine	132.6	589.309	1161.618	15.9923	-2.2	6.1	32616.5	5.18	HUMAN	A6NL76	Actin, alpha skeletal muscle
4440	dirMS_041712_NAR_30mM_plus2.11961.11961.2	2	13.58	0	1.36E+05		(R)YITQSGD	(R)Y I|T/Q/S\G D Y Q L R(T)		115.88	672.3314	1343.659	-0.0035	-2.6	5.83	5236	9.87	HUMAN	K7EK69	AP-1 complex subunit mu-2 (Fragment)
4441	dirMS_041712_NAR_30mM_plus2.13993.14309.2	2	13.58	0	1.22E+06		(R)SVPTWL	(R)S V|P/T|W/L/K(L)		126.78	415.7394	830.477	-0.0055	-6.7	8.47	12573.5	10.39	HUMAN	E9PS50	40S ribosomal protein S13 (Fragment)
4442	dirMS_041712_NAR_30mM_plus2.14416.14573.2	2	13.58	0	5.64E+05		(R)IILLAEGI	(R)I/I|L|L/A E/G R(L)		129.25	442.7821	884.556	0.0005	0.6	6	45228.3	6.03	HUMAN	F5H737	Adenosylhomocysteinase
4443	dirMS_041712_NAR_30mM_plus2.19971.19992.2	2	13.58	0	8.31E+04		(R)FEELTNL	(R)F E|E|L|T|N/L I/R(T)		158.62	567.8152	1134.615	0.0078	6.9	4.53	106155.7	5.45	HUMAN	B5ME19	Eukaryotic translation initiation factor 3 subunit C
4444	dirMS_041712_NAR_40mM_plus2.16185.16185.3	3	13.58	1	1.95E+05		(K)LGVQVV	(K)L G V/Q/V/V/I/T D/P E K L D Q I R(Q)		161.77	641.701	1923.091	-0.0025	-1.3	4.56	44227.3	7.81	HUMAN	E7EQR6	T-complex protein 1 subunit alpha
4445	dirMS_041712_NAR_40mM_plus2.18673.18673.3	3	13.58	0	4.72E+04		(R)NEVSFVI	(R)N/E/V/S F V/I/H N L P V L A K(M)		175.93	560.6533	1679.948	-0.0026	-1.5	6.75	35663.7	5.91	HUMAN	B4DWK0	Selenide, water dikinase 1
4446	dirMS_041712_NAR_50mM_plus2.11459.11459.3	3	13.58	0	3.42E+05		(K)YTVQDE	(K)Y/T/V/Q/D E S H S E\W/V S C V R(F)	C:Carbamidomethylation	127.98	661.2946	1981.871	-0.0016	-0.8	4.65	33029.4	9.62	HUMAN	D6R9L0	Guanine nucleotide-binding protein subunit beta-2-like 1 (Fragment)
4447	dirMS_041712_NAR_50mM_plus2.12384.12426.3	3	13.58	1	1.70E+06		(R)SGKAPIL	(R)S G K A\P I\L|I|A/T/D/V/A/S R(G)		133.47	500.2911	1498.859	0	0	8.46	73202	9.19	HUMAN	C9JMU5	Probable ATP-dependent RNA helicase DDX17
4448	dirMS_041712_NAR_50mM_plus2.6484.6484.3	3	13.58	1	5.14E+04		(R)AGGEEL	(R)A/G/G E/E/L D/E/G V A K D N/A K(I)		99	534.9257	1602.761	0.002	1.2	4.18	89879.1	4.58	HUMAN	Q32MZ4	Leucine-rich repeat flightless-interacting protein 1
4449	dirMS_041712_NAR_40mM_minus2.12584.12584.3	3	13.57	1	5.98E+05		(R)VCENIPI	(R)V/C|E N I/P I\V L/C G N K V D\I/K(D)	C:Carbamidomethylation	157.7	657.6842	1971.04	-0.0021	-1.1	6.04	14844.8	5.89	HUMAN	B4DV51	GTP-binding nuclear protein Ran
4450	dirMS_041712_NAR_40mM_minus2.14258.14258.3	3	13.57	1	2.95E+05		(K)VIAATNI	(K)V I|A A/T N R V D|I L D/P A/L L R(S)		167.82	617.3653	1850.086	-0.0045	-2.4	5.93	32827.5	5.92	HUMAN	E9PKD5	26S protease regulatory subunit 6A (Fragment)
4451	dirMS_041712_NAR_120mM_plus.23917.23917.3	3	13.57	1	3.42E+05	M145m	(R)GVmLA\	(R)G V/m/L/A V/D A V|I A E L K K(Q)	m:Oxidized methionine	179.77	524.9713	1556.908	15.9913	-2.3	6.07	25225.7	9.52	HUMAN	E7ESH4	60 kDa heat shock protein, mitochondrial (Fragment)
4452	dirMS_041712_NAR_120mM_plus.8479.8479.3	3	13.57	1	2.94E+05		(R)ASGNYA	(R)A/S|G/N/Y/A T/V I S H N P E T K K(T)		97.62	606.3102	1816.919	-0.0028	-1.5	8.55	24115.6	11.59	HUMAN	E9PIZ3	60S ribosomal protein L8
4453	dirMS_041712_NAR_20mM_plus.7693.7693.2	2	13.57	0	1.57E+05		(K)IAVAAQ	(K)I/A|V/A/A/Q N C Y K(V)	C:Carbamidomethylation	122.43	569.2902	1137.572	0.001	0.9	8.59	31075.7	5.65	HUMAN	P60174	Triosephosphate isomerase
4454	dirMS_041712_NAR_30mM_plus2.19403.19403.2	2	13.57	0	2.93E+05		(K)TVDNFV	(K)T V|D/N F/V/A/L A T G E/K(G)		155.2	682.8539	1364.706	-0.0051	-3.7	4.37	23799.1	9.48	HUMAN	P23284	Peptidyl-prolyl cis-trans isomerase B
4455	dirMS_041712_NAR_40mM_plus2.18254.18254.3	3	13.57	1	2.86E+05		(R)VAAKLE	(R)V/A A K L E V\A|P\I/S D I I A I K(S)		174.02	584.3591	1751.068	-0.005	-2.8	6.04	24273.5	8.98	HUMAN	H0YK49	Electron transfer flavoprotein subunit alpha, mitochondrial
4456	dirMS_041712_NAR_60mM_plus.15393.15393.3	3	13.57	1	5.97E+05		(R)FVNVVP	(R)F/V|N\V|V/P T F G K K(K)		137.58	412.5761	1235.715	-0.0009	-0.7	10	10904.6	11.56	HUMAN	E9PR30	40S ribosomal protein S30
4457	dirMS_041712_NAR_40mM_minus2.14622.14622.3	3	13.56	1	1.39E+05		(K)GTVPDD	(K)G T/V/P/D D A V/E A L/A D/S L G K K(E)		170.35	595.9791	1785.923	-0.0001	-0.1	4.23	81397.3	4.99	HUMAN	B7Z468	Calpastatin
4458	dirMS_041712_NAR_50mM_minus2.12756.12756.3	3	13.56	1	3.86E+05		(K)NTVELL\	(K)N T|V/E/L/L/V E D K G N/Q V Y/R(C)		140.47	626.3309	1876.976	0.0018	1	4.68	258215.9	5.48	HUMAN	E7EN95	Filamin-B
4459	dirMS_041712_NAR_50mM_minus2.15946.16012.3	3	13.56	1	3.45E+05		(K)QLDDLK	(K)Q L/D D/L K/V/E/L/S Q L R(V)		159.03	519.6261	1556.864	-0.0005	-0.3	4.56	10815.7	10.72	HUMAN	F2Z388	60S ribosomal protein L35
4460	dirMS_041712_NAR_60mM_minus.14159.14159.3	3	13.56	0	3.03E+05		(K)YGYTHLS	(K)Y/G\Y/T/H/L/S/T G/D/L L/R(S)		138.93	499.2614	1495.754	0.0157	10.5	6.74	21748.6	8.92	HUMAN	P00568	Adenylate kinase isoenzyme 1
4461	dirMS_041712_NAR_120mM_plus.15269.15269.4	4	13.56	0	2.28E+06		(K)HLEINPD	(K)H L\E\I N/P D H/P/I/V/E T L R(Q)		133.9	446.4951	1782.95	0.0089	5	5.27	83605	4.97	HUMAN	P08238	Heat shock protein HSP 90-beta
4462	dirMS_041712_NAR_20mM_plus.8086.8086.2	2	13.56	0	2.12E+05		(R)FGQGGA	(R)F/G|Q|G|G A\G/P V|G G/Q G P R(G)		125.2	671.3361	1341.666	-0.0009	-0.7	9.75	76262.2	9.53	HUMAN	P23246	Splicing factor, proline- and glutamine-rich
4463	dirMS_041712_NAR_30mM_plus2.10324.10324.2	2	13.56	0	2.86E+05		(R)LNQDQL	(R)L/N|Q|D Q L/D/A/V/S K(Y)		107.42	615.819	1230.632	-0.0017	-1.4	4.21	20235.4	6.77	HUMAN	E9PLA9	Caprin-1 (Fragment)
4464	dirMS_041712_NAR_30mM_plus2.6388.6654.2	2	13.56	0	3.84E+05		(R)SPDNPM	(R)S/P|D|N|P/M/N Q R(A)		88.78	529.7357	1058.468	-0.0042	-4	5.55	25834.8	9.54	HUMAN	Q96CT7	Coiled-coil domain-containing protein 124

4465	dirMS_041712_NAR_40mM_plus2.10694.10714.3	3	13.56	0	1.85E+05		(K)VGSLDN	(K)V G|S/L/D/N V G H/L/P A G G A V K(I)		128.15	530.9567	1590.86	-0.0043	-2.7	6.71	26233.5	10.95	HUMAN	B4DSQ1	Microtubule-associated protein
4466	dirMS_041712_NAR_40mM_plus2.12538.12538.3	3	13.56	0	3.87E+05		(K)AASDIAI	(K)A A/S D I A/M/T E/L/P P/T H P I R(L)		139.97	607.3167	1819.937	-0.0015	-0.9	5.32	12926.1	4.4	HUMAN	K7EM20	14-3-3 protein epsilon (Fragment)
4467	dirMS_041712_NAR_50mM_plus2.12511.12511.3	3	13.56	1	3.06E+05		(R)SGVGLS	(R)S G V/G/L/S/S/Y L/S T E K D A S\P K(T)		133.63	609.3102	1825.918	-0.0018	-1	5.79	64248.4	9.08	HUMAN	Q9BW04	Specifically androgen-regulated gene protein
4468	dirMS_041712_NAR_50mM_plus2.13321.13322.3	3	13.56	1	4.37E+05		(K)QLQALS	(K)Q L/Q/A/L/S\S E L A/Q A R D E/T/K(K)		138.2	629.9955	1887.977	-0.0051	-2.7	4.68	71218.8	6.3	HUMAN	A7YIJ8	Radixin
4469	dirMS_041712_NAR_50mM_plus2.21762.21762.3	3	13.56	0	3.03E+05		(K)AYNMVI	(K)A Y/N/M/V D I I/H/S V V D/E/R(E)		185.65	587.6346	1760.864	0.0257	14.6	4.54	61363	8.73	HUMAN	C9JQS9	Propionyl-CoA carboxylase beta chain, mitochondrial
4470	dirMS_041712_NAR_50mM_plus2.6093.6429.3	3	13.56	0	5.83E+05	M70m	(K)EAPPmE	(K)E A|P/P/m E K/P E V/V/K(T)	m:Oxidized methionine	96.68	457.2387	1353.708	15.9933	-1.2	4.79	14798.1	10.35	HUMAN	K7EJ78	40S ribosomal protein S15 (Fragment)
4471	dirMS_041712_NAR_60mM_plus.14386.14386.3	3	13.56	1	8.33E+05		(R)TIRYPDP	(R)T/I/R Y P D/P/L/I K(V)		131.87	405.9078	1215.71	-0.0007	-0.6	8.25	29825.4	10.37	HUMAN	P62701	40S ribosomal protein S4, X isoform
4472	dirMS_041712_NAR_60mM_plus.7688.7786.3	3	13.56	1	1.31E+06		(K)KGTVEG	(K)K G T V\E\G\F|E|P/A/D/N K(C)		98.42	464.5649	1391.68	0	0	4.68	13286.2	9.99	HUMAN	H0YLA2	Signal recognition particle 14 kDa protein
4473	dirMS_041712_NAR_60mM_plus.8416.8437.3	3	13.56	1	2.08E+06		(K)EAIEGTY	(K)E A|I/E/G T Y/I/D/K K(C)		102.38	422.8929	1266.658	0.0065	5.2	4.68	18601.6	10.52	HUMAN	P62280	40S ribosomal protein S11
4474	dirMS_041712_NAR_30mM_minus2.8415.8668.2	2	13.55	0	2.91E+06		(R)IMDQTL	(R)I/M|D/Q/T/L/K(A)		101.18	424.7339	848.455	0.0059	7	5.84	31923.6	4.85	HUMAN	B7Z596	Tropomyosin alpha-1 chain
4475	dirMS_041712_NAR_120mM_plus.9659.9722.3	3	13.55	1	1.50E+06		(R)SRNTDE	(R)S R N T D|E|M|V|E/L/R(I)		104.13	450.556	1349.648	0.0057	4.2	4.68	51260.6	5.39	HUMAN	P61978	Heterogeneous nuclear ribonucleoprotein K
4476	dirMS_041712_NAR_60mM_plus.13865.13865.3	3	13.55	1	4.89E+05		(K)VKTDTV	(K)V K T D\T V|L|I/L/C R(K)	C:Carbamidomethylation	129.2	439.9237	1317.756	0.0007	0.5	8.72	21341.9	7.77	HUMAN	Q5R370	Calcyclin binding protein
4477	dirMS_041712_NAR_30mM_minus2.10189.10225.2	2	13.54	0	3.91E+05		(K)ATAVVD	(K)A/T|A|V|V/D G A\F K(E)		109.9	489.7645	978.525	-0.0037	-3.8	5.88	15252.2	5.82	HUMAN	A6NIW5	Peroxiredoxin 2, isoform CRA_a
4478	dirMS_041712_NAR_30mM_minus2.5796.5796.2	2	13.54	0	6.55E+04		(K)TSETLSC	(K)T S|E|T/L/S Q A G Q/K(A)		85.58	575.2877	1149.575	-0.0065	-5.6	5.66	25978.5	4.91	HUMAN	C9J502	Prostate leucine zipper variant 2
4479	dirMS_041712_NAR_50mM_minus2.16579.16579.3	3	13.54	1	1.60E+05		(K)VVSSVL(	(K)V/V/S S V L Q/F/G N I/S/F K K(E)		162	551.6488	1652.937	-0.0052	-3.1	10	233493.8	5.45	HUMAN	F8VTL3	Myosin-10
4480	dirMS_041712_NAR_120mM_plus.15972.15972.3	3	13.54	1	8.08E+04		(K)THLPGF	(K)T\H|L|P G F\V/E\Q/A|E A L K A K(G)		137.02	580.3208	1738.949	-0.0008	-0.5	6.42	22314.2	9.39	HUMAN	P30044	Peroxiredoxin-5, mitochondrial
4481	dirMS_041712_NAR_20mM_plus.12638.12638.2	2	13.54	0	1.19E+05		(K)IISNASC	(K)I I|S N|A|S C T T N C L|A|P L A/K(V)	C:Carbamidomethylation	159.55	917.4626	1833.92	-0.0018	-1	8.75	31718.5	7.17	HUMAN	E7EUT4	Glyceraldehyde-3-phosphate dehydrogenase
4482	dirMS_041712_NAR_20mM_plus.16523.16523.3	3	13.54	0	2.04E+05		(K)TVTNAV	(K)T V T N A V V T V/P/A/Y/F/N D S Q R(Q)		184.62	661.3368	1981.998	-0.0019	-1	5.5	17973	5.16	HUMAN	E9PI65	Heat shock cognate 71 kDa protein (Fragment)
4483	dirMS_041712_NAR_30mM_plus2.14391.14857.2	2	13.54	0	7.11E+06		(R)NLLSVA	(R)N/L|L|S/V/A/Y|K(N)		129.83	454.2621	907.525	-0.0078	-8.6	8.59	18789.1	6.77	HUMAN	A2IDB2	14-3-3 protein eta (Fragment)
4484	dirMS_041712_NAR_30mM_plus2.19426.19426.2	2	13.54	0	2.87E+05		(K)TVDNFV	(K)T V|D N/F/V/A L/A T/G E K(G)		155.25	682.8539	1364.706	-0.0051	-3.7	4.37	23799.1	9.48	HUMAN	P23284	Peptidyl-prolyl cis-trans isomerase B
4485	dirMS_041712_NAR_30mM_plus2.21982.21982.2	2	13.54	0	1.87E+05		(R)EGDVLT	(R)E G D|V/L|T/L|L/E/S E\R(E)		170.55	680.8538	1360.696	0.0049	3.6	4	7898	11.1	HUMAN	P62857	40S ribosomal protein S28
4486	dirMS_041712_NAR_30mM_plus2.22093.22093.2	2	13.54	0	5.37E+03		(R)DLLFQAI	(R)D L|L/F|Q/A/L/G R(T)		171.48	516.7959	1032.584	0.0009	0.8	5.84	66742	6.4	HUMAN	Q9NSD9	Phenylalanine--tRNA ligase beta subunit
4487	dirMS_041712_NAR_30mM_plus2.7106.7106.2	2	13.54	0	1.37E+05		(R)TTTGSYI	(R)T T|T/G/S/Y I A N/R(V)		91.82	542.274	1083.543	-0.0022	-2	8.41	10799.8	5.07	HUMAN	I3L3X7	Proteasome subunit beta type-6 (Fragment)
4488	dirMS_041712_NAR_50mM_plus2.12866.12866.3	3	13.54	0	5.97E+05		(R)FDAERP	(R)F/D|A/E R P V\D C S V/I V/V N K(Q)	C:Carbamidomethylation	135.82	616.6476	1847.932	-0.0038	-2	4.56	13246.4	9.21	HUMAN	Q5JY17	Nuclear receptor coactivator 5 (Fragment)
4489	dirMS_041712_NAR_50mM_plus2.18202.18202.3	3	13.54	1	1.60E+06		(R)ELIKVLEI	(R)E L|I K V L\E E A\N/Q/A|I/N/P K(L)		163.67	603.6741	1809.012	-0.0039	-2.2	4.79	73202	9.19	HUMAN	C9JMU5	Probable ATP-dependent RNA helicase DDX17
4490	dirMS_041712_NAR_60mM_plus.13043.13043.3	3	13.54	1	3.99E+05		(K)LAEQAE	(K)L/A|E/Q/A E/R Y D D M A A A M K(A)		124.77	604.9452	1812.825	-0.0044	-2.5	4.32	28196	4.76	HUMAN	P31946	14-3-3 protein beta/alpha
4491	dirMS_041712_NAR_60mM_plus.19380.19404.3	3	13.54	1	6.47E+05		(R)TRIEGLL	(R)T R I E G L|L|A/A/F/P/K(L)		159.78	439.262	1315.773	-0.0018	-1.4	8.41	62195.8	5.63	HUMAN	B0YIW6	Archain 1, isoform CRA_a
4492	dirMS_041712_NAR_60mM_plus.20627.20627.3	3	13.54	1	2.87E+05		(K)ITQDIFQ	(K)I/T/Q/D/I F/Q Q L L K/R(G)		168.08	501.626	1502.869	-0.0055	-3.7	8.75	72796.9	6.75	HUMAN	A6NC17	Methionine--tRNA ligase, cytoplasmic
4493	dirMS_041712_NAR_60mM_plus.9141.9141.3	3	13.54	1	2.99E+05		(R)FVKQDC	(R)F/V/K Q D|Q|V/C/I A R(L)	C:Carbamidomethylation	106	455.2428	1363.715	-0.0012	-0.9	8.75	53458.1	5.47	HUMAN	H3BR35	Eukaryotic peptide chain release factor GTP-binding subunit ERF3A (Fragment)
4494	dirMS_041712_NAR_60mM_plus.9963.9998.3	3	13.54	1	7.09E+05		(K)ATFIKVP	(K)A T|F/I/K/V/P Q/N Q N G K(S)		110.03	482.2682	1444.791	-0.0006	-0.4	10	32503.1	5.29	HUMAN	H7BY16	Nucleolin (Fragment)
4495	dirMS_041712_NAR_30mM_minus2.17108.17122.2	2	13.53	0	8.20E+04		(K)FAEAFE	(K)F A|E|A/F/E|A/I/P R(A)		146.93	575.7971	1150.589	-0.0022	-1.9	4.53	58214.7	5.25	HUMAN	B4DEM7	T-complex protein 1 subunit theta
4496	dirMS_041712_NAR_40mM_minus2.9912.9912.3	3	13.53	0	6.21E+04		(R)VVIIGAG	(R)V V\I/I/G A G K/P A A\V V/L/Q T K(G)		139.62	555.3529	1664.047	-0.0028	-1.7	10	96827.3	5.58	HUMAN	E9PKU7	Neutral alpha-glucosidase AB
4497	dirMS_041712_NAR_50mM_minus2.12250.12250.3	3	13.53	1	1.37E+05		(R)VFDKDG	(R)V F/D K D G N G\Y|I|S/A/A/E L R(H)		137.82	585.6286	1754.871	0.0005	0.3	4.56	21860.1	4.45	HUMAN	E7EMB3	Calmodulin
4498	dirMS_041712_NAR_120mM_plus.9120.9120.3	3	13.53	1	3.33E+05		(R)SSGGSYI	(R)S S|G G/S/Y R D S Y D S/Y/A/T H N E(-)		101.37	665.9373	1995.795	0.0022	1.1	4.81	18647.7	9.51	HUMAN	Q14011	Cold-inducible RNA-binding protein
4499	dirMS_041712_NAR_20mM_plus.13659.13659.2	2	13.53	0	1.80E+05	M98m	(R)TLTIVDT	(R)T L|T|I/V|D T G I G m T/K(A)	m:Oxidized methionine	166.4	683.3671	1349.735	15.9924	-1.8	5.5	20151.2	4.58	HUMAN	G3V2J8	Heat shock protein HSP 90-alpha (Fragment)
4500	dirMS_041712_NAR_30mM_plus2.14061.14336.2	2	13.53	0	7.39E+05		(K)ALEIIPR(	(K)A L|E|I\I/P/R(Q)		127.17	406.255	811.504	-0.0009	-1.1	6.05	46550.7	7.81	HUMAN	B7Z1C9	Chaperonin containing TCP1, subunit 7 (Eta), isoform CRA_a
4501	dirMS_041712_NAR_30mM_plus2.16177.16177.2	2	13.53	0	2.79E+05		(K)MDQAII	(K)M/D/Q|A/I/I/F C R(T)	C:Carbamidomethylation	137.37	577.2809	1153.549	0.0053	4.6	5.59	83400.6	6.82	HUMAN	Q92499	ATP-dependent RNA helicase DDX1
4502	dirMS_041712_NAR_30mM_plus2.17376.17376.2	2	13.53	0	7.45E+05		(R)IMNTFS	(R)I M|N/T/F/S/V V P S/P K(V)		144.9	660.3537	1319.703	-0.0027	-2	8.75	42831.5	4.86	HUMAN	A8K854	HCG1983504, isoform CRA_f
4503	dirMS_041712_NAR_40mM_plus2.15040.15040.3	3	13.53	1	1.82E+05		(R)EGIVTAT	(R)E G I/V/T/A T E Q E V K E D I A K(L)		155.1	620.6583	1859.96	0.0007	0.4	4.25	74494.3	7.79	HUMAN	F5H897	Heat shock protein 75 kDa, mitochondrial
4504	dirMS_041712_NAR_50mM_plus2.10751.10971.3	3	13.53	1	2.59E+06		(R)VCEEIAII	(R)V/C|E E\I\A\I|I P S K K(L)	C:Carbamidomethylation	125.03	462.9247	1386.766	-0.0066	-4.7	6.11	21927.8	9.55	HUMAN	H0YK46	40S ribosomal protein S17 (Fragment)
4505	dirMS_041712_NAR_50mM_plus2.12021.12021.3	3	13.53	0	4.17E+05		(K)QADLYIS	(K)Q/A/D/L/Y/I/S E G/L H P/R(I)		131.4	500.2606	1498.765	0.0024	1.6	5.32	55323	6.02	HUMAN	B4DPJ8	T-complex protein 1 subunit zeta
4506	dirMS_041712_NAR_50mM_plus2.14902.14902.3	3	13.53	1	1.13E+06		(R)VEKIPGC	(R)V/E K I|P G G\I|I/E/D/S C/V\L R(G)	C:Carbamidomethylation	146.32	595.6548	1784.958	-0.0076	-4.3	4.68	56186.7	5.47	HUMAN	B4DUR8	T-complex protein 1 subunit gamma
4507	dirMS_041712_NAR_50mM_plus2.6731.6731.3	3	13.53	0	1.60E+05		(K)IGEEEIQ	(K)I/G/E/E E/I/Q K P/E E K(V)		100.35	476.9126	1428.722	0.0016	1.1	4.32	81733.5	5.24	HUMAN	E9PAW1	Splicing factor 3A subunit 1
4508	dirMS_041712_NAR_60mM_plus.11291.11291.2	2	13.53	0	6.36E+05		(K)NFYVEH	(K)N F|Y|V/E/H/P E V/A R(L)		116.55	680.8363	1360.664	0.0009	0.7	5.4	73202	9.19	HUMAN	C9JMU5	Probable ATP-dependent RNA helicase DDX17
4509	dirMS_041712_NAR_60mM_plus.12009.12154.3	3	13.53	1	7.31E+06		(R)DGKLVS	(R)D G K L\V\S\E|S/S/D/V L/P/K(-)		120.27	491.928	1473.78	-0.0101	-6.8	4.56	56607.4	5.37	HUMAN	F8VXB4	Keratin, type II cytoskeletal 8
4510	dirMS_041712_NAR_60mM_plus.8553.8553.3	3	13.53	1	4.16E+05		(K)QGIETPE	(K)Q G I/E/T/P E/D Q/N D L R K(M)		102.63	548.2719	1642.803	-0.002	-1.2	4.32	46648.4	9.67	HUMAN	B4DX42	Splicing factor 1
4511	dirMS_041712_NAR_120mM_minus.11453.11487.3	3	13.52	1	1.32E+06	M406m	(R)QLREYQ	(R)Q L R E Y\Q|E|L m/N V/K(L)	m:Oxidized methionine	116.77	522.9361	1550.8	15.9942	-0.5	6.14	56607.4	5.37	HUMAN	F8VXB4	Keratin, type II cytoskeletal 8
4512	dirMS_041712_NAR_30mM_minus2.11887.12011.2	2	13.52	0	3.33E+05		(R)FGISSVP	(R)F/G|I/S/S V/P T/K(G)		118.22	468.2614	935.52	-0.0041	-4.4	8.75	24161.9	5.27	HUMAN	F8VZQ9	SAP domain-containing ribonucleoprotein
4513	dirMS_041712_NAR_40mM_minus2.5919.5919.2	2	13.52	0	1.33E+06		(K)AGFAGD	(K)A G/F|A/G/D/D A\P R(A)		98.17	488.7279	976.448	0.0002	0.2	4.21	123097.1	5.83	HUMAN	A5A3E0	POTE ankyrin domain family member F
4514	dirMS_041712_NAR_40mM_minus2.9405.9405.3	3	13.52	0	1.42E+05		(K)QLTEED(	(K)Q L/T/E/E/D G V/H S V I E E N I K(C)		137.88	647.3235	1939.961	-0.0048	-2.5	4.25	54427.4	5.96	HUMAN	K4DID9	Acetyl-CoA carboxylase 1
4515	dirMS_041712_NAR_50mM_minus2.10348.10348.3	3	13.52	0	1.46E+05		(K)VGSLDN	(K)V/G|S L/D N V/G H L/P A G G A V K(I)		126.9	530.9592	1590.86	0.0032	2	6.71	26233.5	10.95	HUMAN	B4DSQ1	Microtubule-associated protein
4516	dirMS_041712_NAR_60mM_minus.12140.12140.3	3	13.52	1	3.77E+05		(K)AAAGKA	(K)A/A|A G K/A\E L/E|L E/L/G R(I)		127.53	476.5974	1427.785	-0.0076	-5.3	4.79	533778	5.74	HUMAN	Q15149	Plectin
4517	dirMS_041712_NAR_120mM_plus.15209.15225.4	4	13.52	1	1.03E+06		(K)DHASIQI	(K)D H A S I\Q/M/N/V/A E/V/D K/V T G R(F)		133.17	493.25	1969.976	0.0022	1.1	5.38	9225.3	9.16	HUMAN	P63220	40S ribosomal protein S21
4518	dirMS_041712_NAR_120mM_plus.22512.22512.4	4	13.52	1	5.59E+05		(K)SKGLAPI	(K)S K G\L A\P D\L|P E D/L Y H L I K(K)		171.52	478.0137	1909.043	-0.01	-5.2	5.36	16789.5	10.63	HUMAN	J3KMX5	40S ribosomal protein S13
4519	dirMS_041712_NAR_20mM_plus.17053.17053.2	2	13.52	0	1.36E+05		(K)FATEAAI	(K)F A/T/E/A|A/I/T I L R(I)		188.82	603.3442	1205.689	-0.0077	-6.4	6	44227.3	7.81	HUMAN	E7EQR6	T-complex protein 1 subunit alpha
4520	dirMS_041712_NAR_20mM_plus.17670.17670.2	2	13.52	0	8.56E+04		(R)GDGPICI	(R)G/D/G P I|C\L/V/L/A|P T R(E)	C:Carbamidomethylation	193.13	684.8681	1368.73	-0.0015	-1.1	5.84	61018.6	9.44	HUMAN	B4DLW8	Probable ATP-dependent RNA helicase DDX5
4521	dirMS_041712_NAR_20mM_plus.9587.9587.2	2	13.52	0	8.24E+04	M2819m	(K)GLPSPYI	(K)G L/P/S/P/Y N/m S S A P G S R(S)	m:Oxidized methionine	137.4	768.8596	1520.716	15.9957	0.5	8.75	334221.3	6.44	HUMAN	P15924	Desmoplakin
4522	dirMS_041712_NAR_30mM_plus2.13221.13221.2	2	13.52	0	2.78E+05		(R)GGAEQF	(R)G G A\E|Q|F/M/E\E/T E R(S)		122.33	692.2951	1383.584	-0.0016	-1.2	4.09	46550.7	7.81	HUMAN	B7Z1C9	Chaperonin containing TCP1, subunit 7 (Eta), isoform CRA_a
4523	dirMS_041712_NAR_40mM_plus2.19951.19951.3	3	13.52	0	4.60E+05	M166m	(K)VLDFEHI	(K)V/L/D F E H F/L/P m/L Q T\V A K(N)	m:Oxidized methionine	187.63	635.3379	1888.004	15.9954	0.3	5.32	26991.7	5.01	HUMAN	B7Z6Z4	Myosin light polypeptide 6
4524	dirMS_041712_NAR_50mM_plus2.10106.10106.3	3	13.52	1	5.94E+04		(K)ITIKNDP	(K)I T/I/K/N D/P S L/P E/P K(C)		120.53	484.6079	1451.81	-0.0013	-0.9	6.07	20203.8	9.79	HUMAN	B4DG16	Asparagine--tRNA ligase, cytoplasmic
4525	dirMS_041712_NAR_50mM_plus2.19261.19261.3	3	13.52	0	4.30E+05		(R)GFGFITF	(R)G F|G F/I|T/F/T N P E H A S V A M R(A)		169.17	661.3258	1981.959	0.004	2	6.75	17170.1	8.86	HUMAN	P98179	Putative RNA-binding protein 3
4526	dirMS_041712_NAR_50mM_plus2.9470.9470.3	3	13.52	0	7.71E+05		(R)DNIQGI	(R)D N|I\Q G I|T/K/P A I R(R)		117.22	442.5902	1325.754	0.0025	1.9	8.75	11367.1	11.36	HUMAN	P62805	Histone H4
4527	dirMS_041712_NAR_60mM_plus.13701.13742.3	3	13.52	1	4.08E+05		(K)SGYLAGI	(K)S/G|Y|L|A G D K/L|L/P Q R(V)		128.58	473.2654	1417.78	0.0019	1.3	8.31	67933	6.08	HUMAN	P26038	Moesin
4528	dirMS_041712_NAR_120mM_minus.12085.12085.3	3	13.51	0	2.17E+05		(R)QADFEA	(R)Q/A D F E/A/H N I/L/R(E)		119.82	438.5572	1313.66	-0.0026	-2	5.32	66612.6	7.28	HUMAN	F8W720	GMP synthase [glutamine-hydrolyzing]
4529	dirMS_041712_NAR_30mM_minus2.18951.19015.2	2	13.51	0	6.16E+05		(K)LALFQAI	(K)L/A|L/F/Q A/I/G/K(E)		158.7	480.7964	960.588	-0.0022	-2.2	8.75	266161	5.68	HUMAN	E9PPU0	Epiplakin
4530	dirMS_041712_NAR_30mM_minus2.9028.9045.2	2	13.51	0	4.00E+05		(K)DVDEAY	(K)D V|D|E|A|Y M/N K(V)		103.58	542.736	1084.462	0.0032	2.9	4.03	13588.1	4.66	HUMAN	F8VP67	Keratin, type II cytoskeletal 8 (Fragment)
4531	dirMS_041712_NAR_40mM_minus2.10799.10799.3	3	13.51	0	1.24E+05	M56m	(K)VAIVKP(	(K)V/A|I|V|K/P G\V/P m E I V L N K(E)	m:Oxidized methionine	146.9	575.0112	1707.024	15.9953	0.2	8.56	73146.9	5.45	HUMAN	E9PJ21	Hypoxia up-regulated protein 1 (Fragment)
4532	dirMS_041712_NAR_40mM_minus2.14153.14153.3	3	13.51	0	1.71E+05		(K)LQAVEV	(K)L Q|A V/E V/V/I/T H L A P G/T K(H)		167.22	559.3273	1675.974	-0.0068	-4	6.75	16120	9.05	HUMAN	Q9Y2V2	Calcium-regulated heat stable protein 1
4533	dirMS_041712_NAR_40mM_minus2.7678.7678.3	3	13.51	0	6.54E+04		(K)EVVKPV	(K)E V V K\P V/P|I/T S/P A V/S K(V)		123.68	517.6416	1550.915	-0.005	-3.2	8.69	65142.1	9.24	HUMAN	Q96HC4	PDZ and LIM domain protein 5
4534	dirMS_041712_NAR_50mM_minus2.14675.14675.3	3	13.51	0	1.16E+04		(K)GILADEI	(K)G I/L/A/D/E D S S R P V W L K(A)		150.35	562.6354	1685.886	0.0059	3.5	4.56	276046.1	6.01	HUMAN	P49327	Fatty acid synthase
4535	dirMS_041712_NAR_50mM_minus2.18500.18500.3	3	13.51	1	1.47E+06		(R)VISGVLC	(R)V I|S/G\V/L/Q L G N I/V F K K(E)		177	539.0018	1614.994	-0.0033	-2	10	227783.5	5.5	HUMAN	P35579	Myosin-9
4536	dirMS_041712_NAR_50mM_minus2.9346.9346.3	3	13.51	1	1.36E+05		(R)QEEELQ	(R)Q/E/E/E/L/Q A/K/D E E L L/K(V)		121.4	567.9554	1701.854	-0.0025	-1.5	4.14	233493.8	5.45	HUMAN	F8VTL3	Myosin-10
4537	dirMS_041712_NAR_20mM_plus.14684.14684.2	2	13.51	0	1.99E+05		(K)VSTLPAI	(K)V/S|T L|P A I/T/L K(L)		172.6	521.8283	1042.651	-0.0013	-1.3	8.72	20599.3	9.19	HUMAN	H7C1V0	Cathepsin D light chain (Fragment)
4538	dirMS_041712_NAR_20mM_plus.15772.15772.2	2	13.51	0	4.76E+05		(R)GVVDSE	(R)G V V/D S E/D|I/P/L N L S\R(E)		179.47	757.3956	1513.786	-0.0017	-1.1	4.03	74494.3	7.79	HUMAN	F5H897	Heat shock protein 75 kDa, mitochondrial
4539	dirMS_041712_NAR_30mM_plus2.19520.19520.2	2	13.51	0	9.95E+03		(R)FLLADNI	(R)F L|L/A/D N/L/Y/C/K(A)	C:Carbamidomethylation	155.87	628.8226	1256.634	0.0036	2.8	5.83	22400.1	6.19	HUMAN	F5H2A7	Prefoldin subunit 3
4540	dirMS_041712_NAR_30mM_plus2.5731.5814.2	2	13.51	0	4.13E+05		(K)GSSNSY	(K)G/S|S/N/S/Y/A|I/K(K)		84.45	463.7311	926.458	-0.0029	-3.1	8.59	23047.2	9.95	HUMAN	P46782	40S ribosomal protein S5
4541	dirMS_041712_NAR_40mM_plus2.17059.17059.3	3	13.51	0	6.64E+04		(R)IHFPLAT	(R)I H F\P L A\T/Y|A/P V/I S A E K(A)		166.77	586.3246	1756.963	-0.004	-2.3	6.75	49012.3	4.9	HUMAN	A8MUB1	Tubulin alpha-4A chain
4542	dirMS_041712_NAR_40mM_plus2.17958.17958.3	3	13.51	1	7.73E+04	M116m	(R)ELAILLG	(R)E L\A/I/L/L/G/m L D/P A E K\D/E K(G)	m:Oxidized methionine	171.67	634.3333	1884.999	15.9867	-4.3	4.18	25148.6	6	HUMAN	P30041	Peroxiredoxin-6
4543	dirMS_041712_NAR_50mM_plus2.10082.10125.3	3	13.51	0	2.23E+05		(R)TITVALA	(R)T I|T|V/A/L A D/G/G R P D/N T G R(G)		120.23	571.9661	1713.888	-0.0041	-2.4	5.63	107550.2	5.34	HUMAN	P49588	Alanine--tRNA ligase, cytoplasmic
4544	dirMS_041712_NAR_50mM_plus2.13259.13259.3	3	13.51	0	3.85E+05		(K)VQASLA	(K)V Q|A/S/L A/A N T F T I/T G H A/E T K(Q)		137.68	654.0087	1960.013	-0.0019	-1	6.72	11802.2	5.6	HUMAN	D6RDG3	Transcription factor BTF3 (Fragment)
4545	dirMS_041712_NAR_60mM_plus.12427.12474.3	3	13.51	0	1.52E+06		(K)ADDGRF	(K)A/D|D/G/R P F/P Q\V/I/K(S)		121.6	448.2414	1342.711	-0.0017	-1.3	6	15633.5	9.15	HUMAN	H3BMQ8	Fructose-bisphosphate aldolase A (Fragment)
4546	dirMS_041712_NAR_20mM_minus.13287.13287.3	3	13.5	0	2.65E+05		(K)VGINYQ	(K)V G I N Y Q P P T V/V/P G G D L A K(V)		183.15	608.9971	1824.985	-0.0087	-4.8	5.8	49012.3	4.9	HUMAN	A8MUB1	Tubulin alpha-4A chain
4547	dirMS_041712_NAR_40mM_minus2.14425.14425.3	3	13.5	1	1.33E+05		(R)LKLPSIPI	(R)L K L P S I/P L/V|P V S A Q K(R)		169.38	530.6703	1589.999	-0.0025	-1.6	10	34958.1	9.42	HUMAN	Q15785	Mitochondrial import receptor subunit TOM34
4548	dirMS_041712_NAR_50mM_minus2.10264.10264.3	3	13.5	0	2.72E+05		(R)TFIAIKPI	(R)T/F|I/A/I K/P D/G V Q R(G)		126.83	448.923	1344.763	-0.009	-6.7	8.41	6640.6	8.25	HUMAN	C9K028	Nucleoside diphosphate kinase A (Fragment)
4549	dirMS_041712_NAR_50mM_minus2.8023.8023.3	3	13.5	1	1.15E+05		(K)TAVCDIF	(K)T/A/V/C D/I/P P R/G L K(M)	C:Carbamidomethylation	112.25	442.9126	1326.72	0.0034	2.6	8.41	50199.4	4.75	HUMAN	A6NNZ2	Tubulin beta-8 chain-like protein LOC260334
4550	dirMS_041712_NAR_30mM_plus2.6658.6658.2	2	13.5	0	2.92E+05		(R)EAGEQG	(R)E A/G E/Q/G D/I E/P R(R)		89.77	600.7772	1200.549	-0.002	-1.7	4	31450.7	5.73	HUMAN	F5H4D6	Ras GTPase-activating protein-binding protein 1
4551	dirMS_041712_NAR_30mM_plus2.9686.9686.2	2	13.5	0	3.55E+05		(R)TSLGPN(	(R)T S/L/G/P N G L D/K(M)		104.43	501.2717	1001.526	0.0099	9.9	5.5	55804.4	5.33	HUMAN	B7ZAR1	T-complex protein 1 subunit epsilon
4552	dirMS_041712_NAR_40mM_plus2.10066.10066.2	2	13.5	0	1.20E+05		(R)YSGSYNI	(R)Y/S/G S|Y/N\D|Y L R(A)		123.83	619.2788	1237.548	0.0019	1.6	5.83	69661.6	9.75	HUMAN	Q96PK6	RNA-binding protein 14
4553	dirMS_041712_NAR_50mM_plus2.15039.15080.3	3	13.5	1	4.04E+05		(K)LIVAYVD	(K)L/I|V/A/Y V D D L D R/R(G)		147.27	483.2728	1447.79	0.0135	9.3	4.43	124127.1	8.81	HUMAN	O14776	Transcription elongation regulator 1
4554	dirMS_041712_NAR_60mM_plus.14989.15165.3	3	13.5	0	7.90E+05		(R)SCTVNIN	(R)S C/T/V/N/I/M|F/G T P Q E/H R(N)	C:Carbamidomethylation	135.17	592.9433	1776.816	-0.0003	-0.1	6.47	65763.5	9.81	HUMAN	Q9HCD5	Nuclear receptor coactivator 5
4555	dirMS_041712_NAR_120mM_minus.13806.14169.3	3	13.49	1	4.47E+06		(K)ASLENSI	(K)A/S|L/E/N S L/R E V/E/A R(Y)		130.5	491.9266	1473.766	-0.0003	-0.2	4.79	43773.3	5.26	HUMAN	F8VZY9	Keratin, type I cytoskeletal 18
4556	dirMS_041712_NAR_30mM_minus2.11646.11899.3	3	13.49	0	3.96E+06		(R)AQIFAN	(R)A Q\I\F A N/T|V/D/N A/R(I)		117.57	440.5617	1319.67	0.0003	0.2	5.88	43773.3	5.26	HUMAN	F8VZY9	Keratin, type I cytoskeletal 18
4557	dirMS_041712_NAR_30mM_minus2.18397.18742.2	2	13.49	0	2.30E+05		(K)AVVMDI	(K)A V|V|M|D|L L/R(Q)		155.32	458.7685	916.528	0.0013	1.4	5.88	267260.7	5.95	HUMAN	Q13085	Acetyl-CoA carboxylase 1
4558	dirMS_041712_NAR_60mM_minus.16960.16960.3	3	13.49	1	2.51E+05		(K)MFGIDR	(K)M/F/G/I D R D A I A Q/A V R(G)		154.6	521.6047	1562.811	-0.0112	-7.2	5.71	63448.4	7.29	HUMAN	B4E022	Transketolase
4559	dirMS_041712_NAR_120mM_plus.10588.10588.3	3	13.49	1	7.58E+05		(K)IKGEHP(	(K)I/K/G E H/P/G L/S I|G\D/V A K(K)		108.45	507.6167	1520.843	-0.0076	-5	6.75	24408.7	5.92	HUMAN	B2RPK0	Putative high mobility group protein B1-like 1
4560	dirMS_041712_NAR_120mM_plus.7396.7396.3	3	13.49	0	1.08E+05		(K)CIGKPG(	(K)C I/G K/P G G/S L/D N/S E/Q K(C)	C:Carbamidomethylation	92.13	530.5901	1589.759	-0.003	-1.9	6.07	9122.2	9.22	HUMAN	K7EIT2	Mitochondrial import inner membrane translocase subunit Tim13
4561	dirMS_041712_NAR_20mM_plus.14746.14766.2	2	13.49	0	1.98E+05	M179m M:	(K)mFVLDE	(K)m F|V|L/D|E A/D/E/m\L S R(G)	m:Oxidized methionine	173.6	794.3555	1555.713	31.9906	0.5	3.91	41517.9	5.45	HUMAN	E7EQG2	Eukaryotic initiation factor 4A-II
4562	dirMS_041712_NAR_40mM_plus2.5424.5441.2	2	13.49	0	2.45E+05	M300m	(K)NMmAA	(K)N/M|m|A|A/C/D|P R(H)	C:Carbamidomethylation m:Oxid	89.2	541.2145	1065.427	15.9943	-0.6	5.84	50199.4	4.75	HUMAN	A6NNZ2	Tubulin beta-8 chain-like protein LOC260334
4563	dirMS_041712_NAR_40mM_plus2.7573.7573.2	2	13.49	0	5.87E+05		(K)ASLENSI	(K)A/S|L/E/N/S L/R(E)		105.32	445.2395	889.474	-0.002	-2.3	6.05	43773.3	5.26	HUMAN	F8VZY9	Keratin, type I cytoskeletal 18
4564	dirMS_041712_NAR_50mM_plus2.10954.10954.3	3	13.49	0	2.31E+05		(K)TKPYIQV	(K)T/K/P Y\I Q/V D|I/G/G G Q T K(T)		125.32	535.6231	1604.864	-0.0095	-5.9	8.17	72445.7	5.07	HUMAN	P11021	78 kDa glucose-regulated protein
4565	dirMS_041712_NAR_50mM_plus2.15969.15969.3	3	13.49	1	7.39E+04		(K)LGINSLC	(K)L/G/I/N/S/L Q E L K Q N I A K(G)		151.78	556.9915	1668.964	-0.0043	-2.6	8.59	25978.5	4.91	HUMAN	C9J502	Prostate leucine zipper variant 2
4566	dirMS_041712_NAR_50mM_plus2.16304.16445.3	3	13.49	1	4.23E+05		(K)DVLFLKI	(K)D V|L\F|L K/D C V/G P/E V E/K(A)	C:Carbamidomethylation	154.42	583.3027	1747.893	0	0	4.32	41827.5	7	HUMAN	B7Z7A9	Phosphoglycerate kinase
4567	dirMS_041712_NAR_30mM_minus2.13180.13180.2	2	13.48	0	2.99E+04		(K)IALLEEA	(K)I/A|L|L/E E/A K(K)		125.27	443.7658	886.524	-0.0001	-0.1	4.53	71218.8	6.3	HUMAN	A7YIJ8	Radixin
4568	dirMS_041712_NAR_30mM_minus2.19132.19132.2	2	13.48	0	4.66E+04		(R)YLTVAA	(R)Y/L|T/V/A A\V F R(G)		159.13	520.2998	1039.593	-0.0012	-1.1	8.75	40069.4	4.83	HUMAN	E7EWR1	Tubulin beta chain
4569	dirMS_041712_NAR_30mM_minus2.6881.6924.2	2	13.48	0	1.89E+05		(K)DYNVTA	(K)D Y|N|V/T/A/N S K(L)		92	506.2404	1011.474	-0.0006	-0.6	5.83	12539.4	5.78	HUMAN	F5GXH2	L-lactate dehydrogenase A chain (Fragment)
4570	dirMS_041712_NAR_50mM_minus2.16447.16447.3	3	13.48	1	1.04E+06		(R)VTFSGLF	(R)V/T|F|S/G L R D T V T/P G E L L K(A)		162.47	611.672	1833.012	-0.0102	-5.6	6.04	266161	5.68	HUMAN	E9PPU0	Epiplakin
4571	dirMS_041712_NAR_60mM_minus.10169.10169.3	3	13.48	1	1.75E+05	M88m	(K)DVDEAY	(K)D V|D|E/A Y m N K V E L/E/S/R(L)	m:Oxidized methionine	117.05	605.2791	1797.832	15.9904	-2.5	4.18	13588.1	4.66	HUMAN	F8VP67	Keratin, type II cytoskeletal 8 (Fragment)
4572	dirMS_041712_NAR_60mM_minus.18659.18659.3	3	13.48	0	2.93E+05		(K)TFLVWV	(K)T F/L/V/W V/N E/E D H L R(V)		165.78	553.2818	1657.833	-0.0024	-1.5	4.65	20203.9	6.24	HUMAN	H0YJG0	Creatine kinase B-type (Fragment)
4573	dirMS_041712_NAR_60mM_minus.6251.6251.3	3	13.48	1	1.15E+05		(K)DSYVGD	(K)D S\Y|V/G D/E/A/Q S K/R(G)		93.23	452.2129	1354.623	0.0008	0.6	4.56	32616.5	5.18	HUMAN	A6NL76	Actin, alpha skeletal muscle

4574	dirMS_041712_NAR_120mM_plus.8175.8218.3	3	13.48	1	7.73E+05		(R)SQYEQL	(R)S Q|Y/E Q L/A/E/Q N R\K(D)		96.67	498.5821	1493.734	-0.0025	-1.7	5.86	59054.3	5.14	HUMAN	P13645	Keratin, type I cytoskeletal 10
4575	dirMS_041712_NAR_40mM_plus2.18131.18131.3	3	13.48	0	9.77E+04	M159m	(R)SVAAIHF	(R)S V|A/A I H P S L E\I/P m/L I/P K(E)	m:Oxidized methionine	172.8	611.3472	1816.04	15.9869	-4.4	6.47	31606.3	6.72	HUMAN	B4E329	Phosphoribosyl pyrophosphate synthase-associated protein 2
4576	dirMS_041712_NAR_50mM_plus2.19263.19263.3	3	13.48	0	3.59E+05		(-)MKPLVV	(-)M K P L V V/F V|L/G/G/P G A G K(G)		169.73	523.9715	1569.919	-0.0186	-11.8	10	22450.2	5.44	HUMAN	P30085	UMP-CMP kinase
4577	dirMS_041712_NAR_50mM_plus2.9093.9093.3	3	13.48	1	2.45E+05		(K)LLDGPST	(K)L L/D/G P S T E K/D/L D E K(K)		115.15	520.5958	1559.78	-0.0071	-4.5	4.11	12611.4	8.8	HUMAN	E5RIS7	Transcription elongation factor A protein 1
4578	dirMS_041712_NAR_30mM_minus2.12056.12584.2	2	13.47	0	1.76E+06		(R)LQAEIEG	(R)L/Q|A\E/I/E/G L/K(G)		121.93	500.7842	1000.567	-0.0062	-6.2	4.53	37296.3	5.3	HUMAN	F8VU64	Keratin, type II cytoskeletal 8
4579	dirMS_041712_NAR_50mM_minus2.12866.12867.3	3	13.47	0	1.53E+05		(K)ASLASLP	(K)A S|L|A/S/L/P/P\L H V A K(L)		141.37	435.2622	1303.773	-0.0012	-0.9	8.8	141626	5.5	HUMAN	A8MY36	Dynactin subunit 1
4580	dirMS_041712_NAR_20mM_plus.15582.15652.2	2	13.47	0	3.47E+05		(K)VGINYQ	(K)V G I N/Y|Q|P P T V|V|P G G D L/A K(V)		179.05	912.9952	1824.985	-0.0023	-1.3	5.8	49012.3	4.9	HUMAN	A8MUB1	Tubulin alpha-4A chain
4581	dirMS_041712_NAR_30mM_plus2.10660.10807.2	2	13.47	0	4.56E+05		(K)CIAMCM	(K)C|I|A|M/C/M D/R(Y)	C:Carbamidomethylation	109.65	528.7093	1056.409	0.002	1.9	5.84	9122.2	9.22	HUMAN	K7EIT2	Mitochondrial import inner membrane translocase subunit Tim13
4582	dirMS_041712_NAR_30mM_plus2.12672.12672.2	2	13.47	0	1.61E+05		(R)ELAEDG	(R)E L|A/E D/G Y/S/G V/E V R(V)		118.68	712.3382	1423.67	-0.0008	-0.6	4	13054.7	9.59	HUMAN	E9PJH4	40S ribosomal protein S3
4583	dirMS_041712_NAR_30mM_plus2.13736.13736.2	2	13.47	0	1.10E+06		(R)ANTFVA	(R)A N|T/F/V/A E L K(G)		124.65	496.774	992.541	-0.0004	-0.4	6.05	24936.4	8.67	HUMAN	G3XAL0	Malate dehydrogenase
4584	dirMS_041712_NAR_30mM_plus2.16181.16181.2	2	13.47	0	6.33E+05		(R)GLCAIAC	(R)G L/C\A/I|A Q A/E/S L R(Y)	C:Carbamidomethylation	138.37	644.8372	1288.668	-0.0007	-0.5	6	18529.2	9.98	HUMAN	E9PL45	40S ribosomal protein S3
4585	dirMS_041712_NAR_40mM_plus2.13942.13943.3	3	13.47	0	2.92E+05		(R)LASTLVH	(R)L/A\S/T/L V H L G E Y/Q A|A|V D/G A R(K)		148.43	657.6841	1971.029	0.0083	4.2	5.32	71165.3	5.57	HUMAN	J3KS13	Clathrin heavy chain 1
4586	dirMS_041712_NAR_40mM_plus2.19575.19642.3	3	13.47	0	1.06E+06		(R)GLAPVQ	(R)G L|A|P/V Q A Y L/H/I P D/I I/K(V)		183.65	583.3378	1748.011	-0.0117	-6.7	6.74	58266.4	9.46	HUMAN	B4DN00	Pyruvate carboxylase, mitochondrial
4587	dirMS_041712_NAR_50mM_plus2.16149.16149.3	3	13.47	1	1.29E+06		(K)GADFLV	(K)G A/D\F L|V|T E/V E N G G S L G S K K(G)		152.28	636.6618	1907.971	-0.0001	0	4.68	30947.9	6.47	HUMAN	H3BQ34	Pyruvate kinase
4588	dirMS_041712_NAR_60mM_plus.12753.12753.3	3	13.47	1	1.03E+06		(R)DLDRIAC	(R)D L/D R I A|G Q|V/A A A N K(K)		123.8	481.263	1441.776	-0.0013	-0.9	5.96	16060.2	10.31	HUMAN	P39019	40S ribosomal protein S19
4589	dirMS_041712_NAR_60mM_plus.5732.5732.3	3	13.47	0	1.87E+05		(R)STFETEH	(R)S T/F/E/T E/H/A/G Q R(D)		88.4	421.5294	1262.576	-0.0024	-1.9	5.38	160853.1	6.11	HUMAN	F8VU51	YLP motif-containing protein 1
4590	dirMS_041712_NAR_60mM_plus.6917.7075.3	3	13.47	0	1.22E+06	M437m	(R)LESGMC	(R)L/E|S G M Q N m/S I/H/T/K(T)	m:Oxidized methionine	95.27	497.903	1475.698	15.9963	1	6.75	56607.4	5.37	HUMAN	F8VXB4	Keratin, type II cytoskeletal 8
4591	dirMS_041712_NAR_120mM_minus.12146.12274.4	4	13.46	2	4.00E+05		(K)KGTVEG	(K)K\G T V\E\G\F\E|P/A D N K/C L L/R(A)	C:Carbamidomethylation	120.57	484.251	1933.98	0.0021	1.1	6.18	13286.2	9.99	HUMAN	H0YLA2	Signal recognition particle 14 kDa protein
4592	dirMS_041712_NAR_30mM_minus2.10329.10367.2	2	13.46	0	6.38E+05		(K)AVGQDT	(K)A/V|G/Q/D/T L/L/R(S)		110.68	486.7751	972.547	-0.0043	-4.5	5.88	72873.4	5.73	HUMAN	Q96S55	ATPase WRNIP1
4593	dirMS_041712_NAR_50mM_minus2.18212.18285.3	3	13.46	1	1.04E+06		(R)KAIIIFVP	(R)K A I I\I\F|V|P V P Q L K(S)		173.77	489.3206	1465.95	-0.0032	-2.2	10	21311.5	10.28	HUMAN	B5MCP9	40S ribosomal protein S7
4594	dirMS_041712_NAR_20mM_plus.18710.18768.3	3	13.46	0	5.22E+05		(K)LAVEALS	(K)L A\V E A\L\S\S L/D/G D/L/A/G R(Y)		200.47	529.6164	1586.838	-0.0038	-2.4	4.03	24286.7	4.89	HUMAN	G3V4N7	Creatine kinase B-type (Fragment)
4595	dirMS_041712_NAR_20mM_plus.6950.6950.2	2	13.46	0	9.56E+04		(K)AAAAAA	(K)A A|A|A|A|A/A\L Q A K(S)		114.28	478.7792	956.552	-0.0012	-1.3	8.8	37884.5	11.27	HUMAN	H3BM89	60S ribosomal protein L4
4596	dirMS_041712_NAR_30mM_plus2.10913.10913.2	2	13.46	0	5.70E+05		(K)EAAENS	(K)E A|A E/N/S L/V/A/Y|K(A)		110.22	597.8036	1194.6	-0.0002	-0.1	4.53	12926.1	4.4	HUMAN	K7EM20	14-3-3 protein epsilon (Fragment)
4597	dirMS_041712_NAR_30mM_plus2.11318.11318.2	2	13.46	0	9.73E+04		(R)VEFMDD	(R)V/E|F/M\D/D/T S/R(S)		112.67	550.2401	1099.473	0.0005	0.4	4.03	7898	11.1	HUMAN	P62857	40S ribosomal protein S28
4598	dirMS_041712_NAR_30mM_plus2.17759.17759.2	2	13.46	0	1.40E+05		(R)NELSGA	(R)N E|L|S G/A|L/T/G L T/R(V)		146.73	616.3341	1231.664	-0.0032	-2.6	6	71231.7	9.22	HUMAN	E7ERI3	Threonine--tRNA ligase, cytoplasmic
4599	dirMS_041712_NAR_30mM_plus2.18552.18552.2	2	13.46	0	1.54E+05		(R)AMLDQI	(R)A/M|L/D/Q L/M/G/T S/R(D)		150.35	611.8	1222.592	0.0009	0.7	5.88	5509.4	10.84	HUMAN	F8WB41	Putative RNA-binding protein Luc7-like 2
4600	dirMS_041712_NAR_30mM_plus2.9861.9941.2	2	13.46	0	3.98E+05		(R)VPLSAYE	(R)V/P|L|S/A Y E/R(V)		105.42	467.7537	934.499	0.0009	0.9	5.97	300351.7	12.1	HUMAN	Q9UQ35	Serine/arginine repetitive matrix protein 2
4601	dirMS_041712_NAR_40mM_plus2.18491.18503.3	3	13.46	0	5.33E+05		(R)DYLHLPF	(R)D Y L H\L\P P\E\I|V|P/A T L/R(R)		175.73	578.6565	1733.959	-0.0035	-2	5.32	19924.8	9.99	HUMAN	F6U211	40S ribosomal protein S10
4602	dirMS_041712_NAR_40mM_plus2.18796.18796.3	3	13.46	0	7.21E+05		(R)DYLHLPF	(R)D Y L H L\P P E\I\V/P/A/T L R(R)		176.53	578.6565	1733.959	-0.0035	-2	5.32	19924.8	9.99	HUMAN	F6U211	40S ribosomal protein S10
4603	dirMS_041712_NAR_50mM_plus2.10247.10247.3	3	13.46	1	1.76E+05		(R)QELILSN	(R)Q E/L\I/L/S N S E/D K/S/I R(V)		121.43	544.624	1631.86	-0.0024	-1.5	4.68	139883.7	8.06	HUMAN	P53621	Coatomer subunit alpha
4604	dirMS_041712_NAR_50mM_plus2.19031.19031.3	3	13.46	0	5.50E+05		(R)GFGFITF	(R)G F\G|F|I/T/F T N P E/H/A S V A M R(A)		168.75	661.3258	1981.959	0.004	2	6.75	17170.1	8.86	HUMAN	P98179	Putative RNA-binding protein 3
4605	dirMS_041712_NAR_50mM_plus2.21788.21788.3	3	13.46	0	3.29E+05		(K)AYNMVI	(K)A/Y/N|M\V/D I/I H S V V D/E R(E)		185.73	587.6346	1760.864	0.0257	14.6	4.54	61363	8.73	HUMAN	C9JQS9	Propionyl-CoA carboxylase beta chain, mitochondrial
4606	dirMS_041712_NAR_60mM_plus.12391.12391.3	3	13.46	0	1.77E+06		(K)RPGAAL	(K)R P G\A A\L D|P/G C V L\A K(M)	C:Carbamidomethylation	121.05	475.5976	1424.768	0.0104	7.3	8.75	267260.7	5.95	HUMAN	Q13085	Acetyl-CoA carboxylase 1
4607	dirMS_041712_NAR_60mM_plus.20481.20481.3	3	13.46	1	4.21E+05		(K)NAAGAL	(K)N A|A/G A L D/L/L K E/L K(N)		166.93	452.6004	1355.789	-0.0026	-2	6.07	13786.8	9.33	HUMAN	B7Z4S1	Transcription elongation factor A protein 1
4608	dirMS_041712_NAR_50mM_minus2.17264.17276.3	3	13.45	1	2.93E+04		(K)IILDLISE	(K)I/I|L/D/L I/S/E S/P I K G R(A)		166.75	518.6444	1553.926	-0.0075	-4.8	6.07	51260.6	5.39	HUMAN	P61978	Heterogeneous nuclear ribonucleoprotein K
4609	dirMS_041712_NAR_50mM_minus2.17497.17497.3	3	13.45	1	2.94E+05		(K)GYPPED	(K)G Y/P/P E D I F/N L V G K/K(V)		167.38	526.2832	1576.837	-0.0019	-1.2	6.07	40763.2	6.29	HUMAN	Q9NR45	Sialic acid synthase
4610	dirMS_041712_NAR_60mM_minus.12136.12153.3	3	13.45	0	7.93E+05	M1m	(-)mVNHFI	(-)m V/N H/F/I/A E/F/K(R)	m:Oxidized methionine	127.92	417.8768	1235.624	15.9917	-2.6	6.5	45508.6	5.5	HUMAN	A8K7Q2	Heat shock cognate 71 kDa protein
4611	dirMS_041712_NAR_60mM_minus.18638.18638.3	3	13.45	0	2.91E+05		(K)TFLVWV	(K)T F/L/V/W/V/N E E D H L R(V)		165.78	553.2818	1657.833	-0.0024	-1.5	4.65	20203.9	6.24	HUMAN	H0YJG0	Creatine kinase B-type (Fragment)
4612	dirMS_041712_NAR_60mM_minus.8456.8457.3	3	13.45	0	1.53E+05		(K)ISSDLDG	(K)I/S/S/D/L/D G H/P V/P K(Q)		107.3	422.2222	1264.653	-0.0011	-0.9	5.21	63448.4	7.29	HUMAN	B4E022	Transketolase
4613	dirMS_041712_NAR_60mM_minus.9399.9544.3	3	13.45	1	8.61E+05	M142m	(K)VKEGMI	(K)V K E G M N\I|V/E/A/m E R(F)	m:Oxidized methionine	112.42	507.9181	1505.745	15.9947	-0.1	4.79	18240.4	7.96	HUMAN	P62937	Peptidyl-prolyl cis-trans isomerase A
4614	dirMS_041712_NAR_30mM_plus2.13364.13364.2	2	13.45	0	2.13E+05		(R)QQGLAS	(R)Q Q/G L/A/S/Y D/Y V R(R)		122.92	650.3201	1299.633	0.0001	0.1	5.83	533778	5.74	HUMAN	Q15149	Plectin
4615	dirMS_041712_NAR_40mM_plus2.17173.17173.3	3	13.45	0	7.36E+05		(R)IHFPLAT	(R)I H F P L A T Y|A/P V I S/A E K(A)		168.3	586.3246	1756.963	-0.004	-2.3	6.75	49012.3	4.9	HUMAN	A8MUB1	Tubulin alpha-4A chain
4616	dirMS_041712_NAR_50mM_plus2.13556.13556.3	3	13.45	1	3.68E+05		(R)SLIANLA	(R)S L|I/A/N L|A A/A/N C Y K K(E)	C:Carbamidomethylation	139	512.9444	1536.82	-0.0016	-1.1	9.7	36978.6	6.33	HUMAN	B7Z800	Adenosine kinase
4617	dirMS_041712_NAR_50mM_plus2.22117.22117.3	3	13.45	1	2.51E+05		(R)KEGGLG	(R)K E G G L G P L N\I|P L/L A/D V T R(R)		189.68	621.6925	1863.07	-0.0069	-3.7	6.07	15252.2	5.82	HUMAN	A6NIW5	Peroxiredoxin 2, isoform CRA_a
4618	dirMS_041712_NAR_50mM_plus2.8186.8186.3	3	13.45	1	3.71E+05		(R)VLVNDA	(R)V/L|V/N D/A Q K V/T\E G Q Q E R(L)		109.88	605.3151	1813.94	-0.0095	-5.2	4.68	90316.8	5.52	HUMAN	Q13263	Transcription intermediary factor 1-beta
4619	dirMS_041712_NAR_30mM_minus2.7661.7661.2	2	13.44	0	2.31E+05		(R)QDVDNA	(R)Q D/V/D/N A/S L/A/R(L)		96.17	544.7694	1088.533	-0.0016	-1.4	4.21	49709.6	5.19	HUMAN	B0YJC4	Vimentin
4620	dirMS_041712_NAR_40mM_minus2.9930.9930.3	3	13.44	0	1.70E+04		(R)VVIIGAG	(R)V V|I|I/G A G K/P A A V V L Q T K(G)		139.7	555.3529	1664.047	-0.0028	-1.7	10	96827.3	5.58	HUMAN	E9PKU7	Neutral alpha-glucosidase AB
4621	dirMS_041712_NAR_50mM_minus2.17180.17514.3	3	13.44	1	4.37E+05		(K)GYPPED	(K)G Y|P/P E D I F/N/L/V/G K K(V)		166.97	526.2832	1576.837	-0.0019	-1.2	6.07	40763.2	6.29	HUMAN	Q9NR45	Sialic acid synthase
4622	dirMS_041712_NAR_50mM_minus2.7868.7890.3	3	13.44	1	1.26E+05		(K)LLDGPST	(K)L/L|D|G/P S T E/K D L D/E/K(K)		112	520.5968	1559.78	-0.0041	-2.6	4.11	12611.4	8.8	HUMAN	E5RIS7	Transcription elongation factor A protein 1
4623	dirMS_041712_NAR_20mM_plus.14673.14673.3	3	13.44	0	1.12E+05		(R)LQEAGIL	(R)L Q E A G I|L|S/A/E E L Q/R(L)		172.33	519.6131	1556.828	-0.0031	-2	4.25	533778	5.74	HUMAN	Q15149	Plectin
4624	dirMS_041712_NAR_20mM_plus.16758.16758.3	3	13.44	0	2.96E+05		(K)VQIAVA	(K)V Q\I A V\A\N A/Q/E|L/L/Q R(M)		186.87	518.2975	1552.881	-0.0026	-1.7	5.97	10728.1	9.15	HUMAN	Q9Y5L4	Mitochondrial import inner membrane translocase subunit Tim13
4625	dirMS_041712_NAR_20mM_plus.17596.17596.2	2	13.44	0	1.61E+05	M74m	(R)mIAGQV	(R)m I|A\G Q/V|L/D I\N L/A\A E/P K(V)	m:Oxidized methionine	192.85	849.9568	1682.915	15.9918	-1.9	4.37	15791.7	9.22	HUMAN	B4DSU6	Heterogeneous nuclear ribonucleoproteins C1/C2
4626	dirMS_041712_NAR_30mM_plus2.10090.10490.2	2	13.44	0	7.47E+05		(R)LGAPAL	(R)L/G|A|P/A/L/T S R(Q)		107.25	443.2607	885.515	-0.0011	-1.3	9.75	45186.1	9.89	HUMAN	B4DLV4	Serine hydroxymethyltransferase
4627	dirMS_041712_NAR_30mM_plus2.12403.12403.2	2	13.44	0	5.80E+05		(R)YSGSYNI	(R)Y/S|G S Y/N D/Y/L/R(A)		118.28	619.2776	1237.548	-0.0005	-0.4	5.83	69661.6	9.75	HUMAN	Q96PK6	RNA-binding protein 14
4628	dirMS_041712_NAR_30mM_plus2.12719.12823.2	2	13.44	0	2.74E+05		(K)YFDSGD	(K)Y/F|D|S/G/D/Y/N M A K(A)		119.78	655.7724	1310.536	0.0017	1.3	4.21	15633.5	7.82	HUMAN	A6NMQ3	Alpha-endosulfine
4629	dirMS_041712_NAR_30mM_plus2.13013.13013.2	2	13.44	0	5.45E+05		(R)CNNVLY	(R)C N|N/V/L/Y I/R(G)	C:Carbamidomethylation	120.83	526.2726	1051.535	0.0026	2.5	8.75	9782.1	4.7	HUMAN	P62306	Small nuclear ribonucleoprotein F
4630	dirMS_041712_NAR_30mM_plus2.7147.7284.2	2	13.44	0	1.70E+06		(K)TEISEMI	(K)T\E|I|S/E/M/N R(N)		92.85	490.2294	979.451	0.0002	0.2	4.53	37296.3	5.3	HUMAN	F8VU64	Keratin, type II cytoskeletal 8
4631	dirMS_041712_NAR_50mM_plus2.16976.16976.3	3	13.44	1	4.54E+05		(K)VLDSGA	(K)V L/D|S/G A/P I/K/I/P V/G P E T\L G R(I)		156.95	640.3623	1919.096	-0.0237	-12.3	6.04	55307.5	5.25	HUMAN	F8VPV9	ATP synthase subunit beta
4632	dirMS_041712_NAR_50mM_plus2.19137.19137.3	3	13.44	0	4.79E+05		(R)CRPDQL	(R)C R P D Q L T/G|L/S/L/L|P\L S E K(A)	C:Carbamidomethylation	168.18	643.0148	1927.032	-0.0019	-1	6.07	533778	5.74	HUMAN	Q15149	Plectin
4633	dirMS_041712_NAR_50mM_plus2.22198.22198.3	3	13.44	1	5.40E+05		(R)ELAILLG	(R)E L/A I/L/L G M/L/D/P A E K D E K(G)		190.32	629.004	1884.999	-0.0012	-0.7	4.18	25148.6	6	HUMAN	P30041	Peroxiredoxin-6
4634	dirMS_041712_NAR_50mM_plus2.6150.6150.3	3	13.44	1	1.12E+05		(R)VVSSIEC	(R)V/V/S\S/I E Q K T E G A E K(K)		96.75	502.2651	1504.785	-0.0046	-3	4.79	8491.4	6.28	HUMAN	E5RIR4	14-3-3 protein zeta/delta (Fragment)
4635	dirMS_041712_NAR_60mM_plus.12071.12071.2	2	13.44	1	1.52E+06		(R)DGKLVS	(R)D/G K L|V|S E S S D/V/L|P K(-)		120.25	737.3869	1473.78	-0.013	-8.8	4.56	56607.4	5.37	HUMAN	F8VXB4	Keratin, type II cytoskeletal 8
4636	dirMS_041712_NAR_60mM_plus.6306.6306.3	3	13.44	1	2.17E+05		(K)QMNDE	(K)Q M N D/E/K/T A/A/D/Y/K(I)		91.65	471.8823	1413.632	0.0009	0.6	4.56	9786.1	5.52	HUMAN	E9PS38	Protein NEDD8-MDP1
4637	dirMS_041712_NAR_120mM_minus.17543.17543.3	3	13.43	1	5.17E+05		(R)KVNNAD	(R)K V N\N A D D\F|P/N L F R(Q)		150.13	517.2634	1549.776	-0.0001	-0.1	5.96	267260.7	5.95	HUMAN	Q13085	Acetyl-CoA carboxylase 1
4638	dirMS_041712_NAR_20mM_minus.13153.13153.2	2	13.43	0	4.63E+05		(K)TITLEVEI	(K)T I T L/E/V/E|P S/D T I/E/N V K(A)		183.05	894.4654	1787.927	-0.0038	-2.1	4	17271.6	6.75	HUMAN	B4DV12	Ubiquitin
4639	dirMS_041712_NAR_40mM_minus2.11214.11214.3	3	13.43	1	1.21E+05		(K)VIDPATA	(K)V I|D/P/A/T A T S V/D L R/D/I K(I)		149.38	571.9828	1713.938	-0.0043	-2.5	4.43	42755	7.81	HUMAN	B7Z2F4	T-complex protein 1 subunit delta
4640	dirMS_041712_NAR_40mM_minus2.12193.12193.3	3	13.43	0	4.30E+05		(K)IAILTCPF	(K)I A/I/L/T C/P F E/P P K P K(T)	C:Carbamidomethylation	155.62	537.6382	1610.898	0.0026	1.6	8.59	49982	5.86	HUMAN	B4DYD8	T-complex protein 1 subunit epsilon
4641	dirMS_041712_NAR_50mM_minus2.7167.7167.3	3	13.43	1	2.39E+04		(K)GAVAED	(K)G A V|A/E D G D E L R T E P E/A K(K)		107.67	596.2904	1786.845	0.0113	6.3	4.08	19284.3	5.23	HUMAN	G3V3C7	DNA-(apurinic or apyrimidinic site) lyase (Fragment)
4642	dirMS_041712_NAR_50mM_minus2.9552.9552.3	3	13.43	1	2.33E+06		(R)MQKEIT	(R)M Q K E I T/A|L/A|P S T M K(I)		123.35	516.9426	1548.812	0.0008	0.5	8.35	32616.5	5.18	HUMAN	A6NL76	Actin, alpha skeletal muscle
4643	dirMS_041712_NAR_120mM_plus.8110.8151.2	2	13.43	0	6.21E+05		(R)YAHVVL	(R)Y/A|H|V/V L/R(K)		96.72	429.2529	857.499	-0.0007	-0.8	8.75	17718.4	10.99	HUMAN	P62269	40S ribosomal protein S18
4644	dirMS_041712_NAR_20mM_plus.14277.14277.2	2	13.43	0	3.41E+04		(R)IALTDNA	(R)I/A|L/T/D N\A\L I A R(S)		170.65	585.8432	1170.684	-0.005	-4.2	5.84	24489.6	10.74	HUMAN	A8MUD9	60S ribosomal protein L7
4645	dirMS_041712_NAR_20mM_plus.18839.18839.2	2	13.43	0	2.79E+05		(K)LAVEALS	(K)L A/V E/A/L|S/S/L D|G\D L/A/G R(Y)		200.52	793.9214	1586.838	-0.0029	-1.8	4.03	24286.7	4.89	HUMAN	G3V4N7	Creatine kinase B-type (Fragment)
4646	dirMS_041712_NAR_30mM_plus2.15227.15691.2	2	13.43	0	1.64E+06		(R)YIELLTR(	(R)Y/I|E|L/L|T/R(S)		132.8	454.2621	907.525	-0.0078	-8.6	6	334221.3	6.44	HUMAN	P15924	Desmoplakin
4647	dirMS_041712_NAR_30mM_plus2.19088.19153.2	2	13.43	0	2.78E+05		(R)LTAEDLF	(R)L/T|A/E/D/L|F E/A R(I)		153.22	582.796	1164.59	-0.0048	-4.1	4.14	533778	5.74	HUMAN	Q15149	Plectin
4648	dirMS_041712_NAR_40mM_plus2.17443.17443.3	3	13.43	0	4.17E+05		(R)DVCTELI	(R)D V/C|T E L/L/P L/I/K/P Q G R(V)	C:Carbamidomethylation	169.57	580.3213	1738.952	-0.0027	-1.5	6.07	30659.7	9.02	HUMAN	P16152	Carbonyl reductase [NADPH] 1
4649	dirMS_041712_NAR_60mM_plus.11043.11043.3	3	13.43	1	1.97E+06		(R)KLDPGSI	(R)K L D|P G S/E|E/T/Q/T/L V R(E)		115.02	524.9456	1572.823	-0.0005	-0.3	4.68	56491.1	7.78	HUMAN	B4DTG2	Elongation factor 1-gamma
4650	dirMS_041712_NAR_60mM_plus.13947.14370.3	3	13.43	0	1.99E+06		(K)GIHPTIIS	(K)G I H\P T\I|I|S/E/S F Q K(A)		130.72	486.2646	1456.78	-0.0002	-0.1	6.75	52843.5	6.17	HUMAN	B7Z9L0	T-complex protein 1 subunit delta
4651	dirMS_041712_NAR_20mM_minus.9779.9779.2	2	13.42	0	3.36E+05		(K)IGGIGTV	(K)I/G/G\I/G\T/V/P V G R(V)		147.47	513.3075	1025.61	-0.0025	-2.4	9.75	50482.3	9.31	HUMAN	P68104	Elongation factor 1-alpha 1
4652	dirMS_041712_NAR_30mM_minus2.14988.15196.2	2	13.42	0	5.95E+05		(K)LYANMF	(K)L/Y|A|N/M/F E/R(L)		135.42	522.2529	1043.498	0.0006	0.6	6	9740.1	10.08	HUMAN	H0YFG2	Peptidyl-prolyl cis-trans isomerase FKBP4, N-terminally processed (Fragment)
4653	dirMS_041712_NAR_60mM_minus.11061.11061.3	3	13.42	1	3.17E+06		(R)DGKLVS	(R)D G K L V S E/S/S/D/V/L/P K(-)		122.2	491.927	1473.78	-0.0131	-8.9	4.56	56607.4	5.37	HUMAN	F8VXB4	Keratin, type II cytoskeletal 8
4654	dirMS_041712_NAR_120mM_plus.17880.17880.4	4	13.42	0	3.77E+05		(R)HLLQAP	(R)H L L Q\A|P V/D/D\A Q/E/I L/H/S R(F)		146.78	486.2589	1942.014	-0.0003	-0.2	5.21	63613.4	7.9	HUMAN	B3KXI2	Protein transport protein Sec23A
4655	dirMS_041712_NAR_30mM_plus2.13914.14015.2	2	13.42	0	4.51E+05		(K)LVPVGY	(K)L V|P|V/G/Y G I/R(K)		126.23	487.2951	973.583	0	0	8.75	29184.1	4.91	HUMAN	E9PBQ9	Elongation factor 1-delta
4656	dirMS_041712_NAR_30mM_plus2.18449.18669.2	2	13.42	0	8.06E+05		(K)QLLLQLI	(K)Q L|L|L|Q/L|N Q/Q R(T)		150.85	627.3688	1253.732	-0.0021	-1.7	9.75	67181.8	5.96	HUMAN	Q96JB3	Hypermethylated in cancer 2 protein
4657	dirMS_041712_NAR_30mM_plus2.18501.18501.2	2	13.42	0	6.99E+05		(K)EGMNIV	(K)E G M/N/I|V E/A M E R(F)		150.78	639.7958	1278.582	0.0026	2.1	4.25	18240.4	7.96	HUMAN	P62937	Peptidyl-prolyl cis-trans isomerase A
4658	dirMS_041712_NAR_30mM_plus2.20957.20957.2	2	13.42	0	2.33E+05		(K)VFSIGPV	(K)V/F|S\I/G/P V F/R(A)		164.52	511.2941	1021.583	-0.002	-2	9.72	57534.6	6.11	HUMAN	P14868	Aspartate--tRNA ligase, cytoplasmic
4659	dirMS_041712_NAR_30mM_plus2.7005.7166.2	2	13.42	0	2.52E+05		(R)FVDPNT	(R)F V|D|P N/T Q E K(V)		91.67	539.2643	1077.521	0.0002	0.2	4.37	266161	5.68	HUMAN	E9PPU0	Epiplakin
4660	dirMS_041712_NAR_40mM_plus2.13508.13569.3	3	13.42	0	2.43E+05		(K)SIEIPRP\	(K)S I|E|I/P R P V D G V/E|V/P G C G K(I)	C:Carbamidomethylation	145.88	637.0002	1908.985	0.0013	0.7	4.68	34243.5	4.88	HUMAN	K7ENG2	Splicing factor U2AF 65 kDa subunit
4661	dirMS_041712_NAR_50mM_plus2.10848.10893.3	3	13.42	0	4.89E+05		(R)EALLSSA	(R)E A|L/L/S/S A\V|D|H G S D E/V/K(F)		124.7	552.9403	1656.807	-0.0012	-0.7	4.31	57204.9	6.01	HUMAN	F5GWF6	T-complex protein 1 subunit beta
4662	dirMS_041712_NAR_50mM_plus2.12198.12218.3	3	13.42	0	3.98E+05		(K)IVLEDGT	(K)I/V|L/E/D G T L/H V/T/E G S G R(Y)		132.23	561.6272	1682.871	-0.0037	-2.2	4.65	62748.9	5.95	HUMAN	Q16555	Dihydropyrimidinase-related protein 2
4663	dirMS_041712_NAR_60mM_plus.6875.6905.3	3	13.42	1	2.99E+06		(R)LQSIGTE	(R)L/Q|S|I/G/T E/N T/E E N R/R(F)		94.57	549.6075	1646.809	-0.0013	-0.8	4.79	15633.5	9.15	HUMAN	H3BMQ8	Fructose-bisphosphate aldolase A (Fragment)
4664	dirMS_041712_NAR_120mM_minus.6881.7172.3	3	13.41	1	2.14E+07		(R)TKTEISEI	(R)T K\T E\I|S|E/M/N/R(N)		92.57	403.5363	1208.594	0.0004	0.3	5.81	37296.3	5.3	HUMAN	F8VU64	Keratin, type II cytoskeletal 8
4665	dirMS_041712_NAR_30mM_minus2.7452.7513.2	2	13.41	0	5.81E+05		(K)AVELAAI	(K)A V|E|L/A|A/N T/K(G)		95.82	458.753	916.51	-0.0111	-12.1	6.05	63448.4	7.29	HUMAN	B4E022	Transketolase
4666	dirMS_041712_NAR_50mM_minus2.15971.15971.3	3	13.41	0	2.36E+05		(K)AQDGSH	(K)A Q|D/G S H/P L S L/Q D L I E K(V)		159.08	584.3049	1750.897	0.0031	1.8	4.54	128523.4	7.06	HUMAN	B7ZL00	Protein transport protein Sec31A
4667	dirMS_041712_NAR_120mM_plus.9734.9736.4	4	13.41	0	2.37E+05	M275m	(K)SGVYQH	(K)S/G|V/Y Q/H V/T G E M m/G G H/A I R(I)	m:Oxidized methionine	104.28	487.2313	1929.906	15.9976	1.4	6.66	38790.8	5.88	HUMAN	P07858	Cathepsin B
4668	dirMS_041712_NAR_30mM_plus2.13915.14152.2	2	13.41	0	1.45E+06		(R)DVQEIFF	(R)D V|Q|E I/F/R(L)		126.7	453.7343	906.468	-0.0066	-7.3	4.37	30944.2	6.52	HUMAN	B1Q2N1	ATP-dependent RNA helicase DDX39A
4669	dirMS_041712_NAR_50mM_plus2.16472.16472.3	3	13.41	1	4.23E+05		(K)DVLFLKD	(K)D V|L/F/L K D/C V/G P E V E K(A)	C:Carbamidomethylation	154.42	583.3027	1747.893	0	0	4.32	41827.5	7	HUMAN	B7Z7A9	Phosphoglycerate kinase
4670	dirMS_041712_NAR_120mM_minus.8622.8808.3	3	13.4	1	7.08E+06	M174m	(R)VKYETEL	(R)V/K\Y E T E|L/A/m/R(Q)	m:Oxidized methionine	101.98	419.2167	1239.64	15.9954	0.4	6.11	43773.3	5.26	HUMAN	F8VZY9	Keratin, type I cytoskeletal 18
4671	dirMS_041712_NAR_30mM_minus2.17485.17485.2	2	13.4	0	2.29E+05		(R)NGGQIL	(R)N G|G|Q/I|L I/A/D/L R(G)		149.22	585.3352	1169.664	-0.0006	-0.5	5.84	34524.7	5.4	HUMAN	A8MV37	Protein SEC13 homolog
4672	dirMS_041712_NAR_50mM_minus2.14076.14098.3	3	13.4	1	3.57E+05		(K)VIDPATA	(K)V/I|D|P A/T A T|S V D L R D I K(I)		148.1	571.9822	1713.938	-0.0061	-3.6	4.43	42755	7.81	HUMAN	B7Z2F4	T-complex protein 1 subunit delta
4673	dirMS_041712_NAR_50mM_minus2.14312.14312.3	3	13.4	0	4.31E+05		(R)QIFHPEC	(R)Q I/F H P/E Q/L I/T/G/K(E)		149.72	470.9286	1410.774	-0.0027	-1.9	6.75	27836	8	HUMAN	Q9H853	Putative tubulin-like protein alpha-4B
4674	dirMS_041712_NAR_120mM_plus.24456.24636.4	4	13.4	1	1.18E+06		(R)GHYTIGI	(R)G H\Y\T I\G\K E\I\I\D\L|V/L/D/R(I)		184.3	461.2589	1842.012	0.0018	1	5.38	58642	4.95	HUMAN	F5H5D3	Tubulin alpha-1C chain
4675	dirMS_041712_NAR_30mM_plus2.10528.10528.2	2	13.4	0	3.54E+05		(R)DPYGFG	(R)D/P|Y|G|F\G\D/S/R(D)		108.9	507.2274	1013.432	0.0152	15	4.21	65763.5	9.81	HUMAN	Q9HCD5	Nuclear receptor coactivator 5
4676	dirMS_041712_NAR_30mM_plus2.4879.4879.2	2	13.4	0	5.87E+05	M571m	(R)mGANSI	(R)m/G/A|N/S L/E R(M)	m:Oxidized methionine	76.8	447.2119	877.42	15.9969	2.2	5.75	77799.7	9.09	HUMAN	P52272	Heterogeneous nuclear ribonucleoprotein M
4677	dirMS_041712_NAR_40mM_plus2.19959.19959.3	3	13.4	0	4.60E+05	M166m	(K)VLDFEHI	(K)V L/D F E H F L|P/m/L Q T V A K(N)	m:Oxidized methionine	187.63	635.3379	1888.004	15.9954	0.3	5.32	26991.7	5.01	HUMAN	B7Z6Z4	Myosin light polypeptide 6
4678	dirMS_041712_NAR_50mM_plus2.18756.18968.3	3	13.4	1	4.36E+05		(K)AAIDWF	(K)A/A|I/D/W|F D G K E F S G N/P I K(V)		167.18	632.3186	1894.933	0.0079	4.1	4.56	53724.2	9.54	HUMAN	H3BPE7	RNA-binding protein FUS
4679	dirMS_041712_NAR_50mM_plus2.6133.6133.3	3	13.4	1	6.55E+04		(R)NTDVAC	(R)N T|D|V|A/Q S P E A/P K/Q E A P A K(K)		96.33	627.6463	1880.935	-0.0105	-5.6	4.68	152868.9	8.79	HUMAN	Q9P2E9	Ribosome-binding protein 1
4680	dirMS_041712_NAR_50mM_plus2.7141.7141.3	3	13.4	0	9.72E+04		(K)VLATVTI	(K)V L|A/T V T K/P V G/G D/K(N)		103.27	428.9213	1284.752	-0.0028	-2.2	8.56	12738	10.72	HUMAN	F8VWR1	60S ribosomal protein L6 (Fragment)
4681	dirMS_041712_NAR_20mM_minus.9576.9580.2	2	13.39	0	3.70E+05		(R)LQAEIEG	(R)L/Q|A|E|I E/G L/K(G)		145.97	500.7862	1000.567	-0.0022	-2.2	4.53	37296.3	5.3	HUMAN	F8VU64	Keratin, type II cytoskeletal 8
4682	dirMS_041712_NAR_30mM_minus2.10458.10458.2	2	13.39	0	8.52E+05		(K)YETELAN	(K)Y/E|T/E L A M/R(Q)		111.27	506.7468	1012.477	0.0095	9.4	4.53	43773.3	5.26	HUMAN	F8VZY9	Keratin, type I cytoskeletal 18

<table>
<tr><td>4683</td><td>dirMS_041712_NAR_30mM_minus2.19751.19812.2</td><td>2</td><td>13.39</td><td>0</td><td>1.11E+06</td><td></td><td>(K)FLPLFDR</td><td>(K)F/L|P/L F D/R(V)</td><td></td><td>164.83</td><td>454.2604</td><td>907.504</td><td>0.0099</td><td>10.9</td><td>5.84</td><td>10803.5</td><td>9.82</td><td>HUMAN</td><td>B8ZZL8</td><td>10 kDa heat shock protein, mitochondrial</td></tr>
<tr><td>4684</td><td>dirMS_041712_NAR_50mM_minus2.10059.10059.3</td><td>3</td><td>13.39</td><td>0</td><td>5.64E+05</td><td>M147m</td><td>(K)LQmEAP</td><td>(K)L\Q|m|E\A P/H\I|I|V G/T P G R(V)</td><td>m:Oxidized methionine</td><td>125.58</td><td>545.6267</td><td>1618.873</td><td>15.9922</td><td>-1.7</td><td>6.75</td><td>20859.5</td><td>4.85</td><td>HUMAN</td><td>J3KS25</td><td>Eukaryotic initiation factor 4A-I (Fragment)</td></tr>
<tr><td>4685</td><td>dirMS_041712_NAR_120mM_plus.8401.8401.3</td><td>3</td><td>13.39</td><td>0</td><td>3.40E+05</td><td></td><td>(K)YKPESEE</td><td>(K)Y K P E S E E|L/T/A/E/R(I)</td><td></td><td>97.4</td><td>484.5712</td><td>1451.701</td><td>-0.0022</td><td>-1.5</td><td>4.5</td><td>51524.1</td><td>4.82</td><td>HUMAN</td><td>F5H8J2</td><td>Protein disulfide-isomerase</td></tr>
<tr><td>4686</td><td>dirMS_041712_NAR_20mM_plus.11556.11556.2</td><td>2</td><td>13.39</td><td>0</td><td>1.72E+05</td><td></td><td>(K)VGLQVV</td><td>(K)V/G/L\Q\V\V A V\K(A)</td><td></td><td>151.75</td><td>456.7977</td><td>912.588</td><td>0.0004</td><td>0.5</td><td>8.72</td><td>61224.7</td><td>5.7</td><td>HUMAN</td><td>P10809</td><td>60 kDa heat shock protein, mitochondrial</td></tr>
<tr><td>4687</td><td>dirMS_041712_NAR_20mM_plus.12394.12394.2</td><td>2</td><td>13.39</td><td>0</td><td>1.87E+05</td><td></td><td>(R)GVEICIA</td><td>(R)G V E|I/C/I A/T/P G R(L)</td><td>C:Carbamidomethylation</td><td>158.58</td><td>586.8084</td><td>1172.609</td><td>0.0003</td><td>0.3</td><td>6</td><td>61018.6</td><td>9.44</td><td>HUMAN</td><td>B4DLW8</td><td>Probable ATP-dependent RNA helicase DDX5</td></tr>
<tr><td>4688</td><td>dirMS_041712_NAR_20mM_plus.15936.15936.2</td><td>2</td><td>13.39</td><td>0</td><td>3.24E+05</td><td>M81m</td><td>(K)SFYPEEV</td><td>(K)S F/Y/P\E E V/S S m V L T K(M)</td><td>m:Oxidized methionine</td><td>180.88</td><td>816.8988</td><td>1616.788</td><td>16.0027</td><td>4.8</td><td>4.53</td><td>17973</td><td>5.16</td><td>HUMAN</td><td>E9PI65</td><td>Heat shock cognate 71 kDa protein (Fragment)</td></tr>
<tr><td>4689</td><td>dirMS_041712_NAR_20mM_plus.19049.19049.3</td><td>3</td><td>13.39</td><td>0</td><td>2.07E+05</td><td></td><td>(K)TLETQS/</td><td>(K)T L\E T Q S A L|G/P/A L Q|A/A F K(L)</td><td></td><td>202.57</td><td>582.6516</td><td>1745.943</td><td>-0.003</td><td>-1.7</td><td>5.66</td><td>120546</td><td>7.82</td><td>HUMAN</td><td>O95486</td><td>Protein transport protein Sec24A</td></tr>
<tr><td>4690</td><td>dirMS_041712_NAR_20mM_plus.8616.8803.2</td><td>2</td><td>13.39</td><td>0</td><td>2.49E+05</td><td></td><td>(R)VPPPPPI</td><td>(R)V/P|P|P/P P I A R(A)</td><td></td><td>130.63</td><td>472.2851</td><td>943.572</td><td>-0.0094</td><td>-10</td><td>9.72</td><td>32085.2</td><td>8.93</td><td>HUMAN</td><td>B4DY08</td><td>Heterogeneous nuclear ribonucleoproteins C1/C2</td></tr>
<tr><td>4691</td><td>dirMS_041712_NAR_30mM_plus2.7310.7353.2</td><td>2</td><td>13.39</td><td>0</td><td>3.37E+05</td><td></td><td>(K)DYAESV</td><td>(K)D Y|A|E/S/V/G R(K)</td><td></td><td>93.07</td><td>448.71</td><td>896.411</td><td>0.0019</td><td>2.1</td><td>4.37</td><td>65763.5</td><td>9.81</td><td>HUMAN</td><td>Q9HCD5</td><td>Nuclear receptor coactivator 5</td></tr>
<tr><td>4692</td><td>dirMS_041712_NAR_40mM_plus2.17470.17470.3</td><td>3</td><td>13.39</td><td>0</td><td>1.58E+05</td><td></td><td>(R)VYAILTH</td><td>(R)V Y/A I/L T/H G I|F S G P\A I S R(I)</td><td></td><td>169.33</td><td>601.3376</td><td>1801.996</td><td>0.0023</td><td>1.3</td><td>8.73</td><td>24482.9</td><td>7.96</td><td>HUMAN</td><td>B1ALA9</td><td>Phosphoribosyl pyrophosphate synthetase 1</td></tr>
<tr><td>4693</td><td>dirMS_041712_NAR_50mM_plus2.9936.9956.3</td><td>3</td><td>13.39</td><td>1</td><td>3.03E+05</td><td></td><td>(K)VCGDSD</td><td>(K)V/C|G D/S D K G/F|V/V I N Q K(G)</td><td>C:Carbamidomethylation</td><td>119.55</td><td>555.947</td><td>1665.826</td><td>0</td><td>0</td><td>5.93</td><td>55323</td><td>6.02</td><td>HUMAN</td><td>B4DPJ8</td><td>T-complex protein 1 subunit zeta</td></tr>
<tr><td>4694</td><td>dirMS_041712_NAR_60mM_plus.15252.15309.3</td><td>3</td><td>13.39</td><td>1</td><td>8.29E+04</td><td></td><td>(R)KEFSPFG</td><td>(R)K\E F/S\P\F|G T/I/T S/A/K(V)</td><td></td><td>137.1</td><td>471.585</td><td>1412.742</td><td>-0.0016</td><td>-1.1</td><td>8.59</td><td>65918.2</td><td>9.74</td><td>HUMAN</td><td>E7EQV3</td><td>Polyadenylate-binding protein 1</td></tr>
<tr><td>4695</td><td>dirMS_041712_NAR_50mM_minus2.11845.11845.3</td><td>3</td><td>13.38</td><td>1</td><td>6.27E+06</td><td></td><td>(K)KVPQVS</td><td>(K)K\V P Q\V\S T/P T|L/V|E V/S R(N)</td><td></td><td>136.32</td><td>547.3172</td><td>1639.938</td><td>-0.0007</td><td>-0.4</td><td>8.75</td><td>57751.3</td><td>6.9</td><td>HUMAN</td><td>B7WNR0</td><td>Serum albumin</td></tr>
<tr><td>4696</td><td>dirMS_041712_NAR_50mM_minus2.15910.15910.3</td><td>3</td><td>13.38</td><td>1</td><td>8.21E+04</td><td></td><td>(K)DANAKL</td><td>(K)D A N\A K L S E\L|E/A/A L/Q R(A)</td><td></td><td>158.97</td><td>543.6248</td><td>1628.86</td><td>-0.0004</td><td>-0.2</td><td>4.68</td><td>56607.4</td><td>5.37</td><td>HUMAN</td><td>F8VXB4</td><td>Keratin, type II cytoskeletal 8</td></tr>
<tr><td>4697</td><td>dirMS_041712_NAR_120mM_plus.21328.21447.3</td><td>3</td><td>13.38</td><td>1</td><td>3.11E+05</td><td></td><td>(R)KVESLW</td><td>(R)K V E S L\W|P/I/F/R(I)</td><td></td><td>165.75</td><td>425.5799</td><td>1274.726</td><td>-0.0004</td><td>-0.3</td><td>8.75</td><td>14138.1</td><td>9.89</td><td>HUMAN</td><td>C9JNV2</td><td>Protein BUD31 homolog</td></tr>
<tr><td>4698</td><td>dirMS_041712_NAR_120mM_plus.9076.9365.2</td><td>2</td><td>13.38</td><td>0</td><td>4.16E+06</td><td></td><td>(K)HVVFIA(</td><td>(K)H|V|V|F I|A Q/R(R)</td><td></td><td>101.58</td><td>485.2849</td><td>969.563</td><td>-0.0003</td><td>-0.3</td><td>9.75</td><td>21311.5</td><td>10.28</td><td>HUMAN</td><td>B5MCP9</td><td>40S ribosomal protein S7</td></tr>
<tr><td>4699</td><td>dirMS_041712_NAR_30mM_plus2.16261.16261.2</td><td>2</td><td>13.38</td><td>0</td><td>1.38E+06</td><td></td><td>(R)GGIVDE(</td><td>(R)G G I|V/D/E G A/L L R(A)</td><td></td><td>138.9</td><td>550.3082</td><td>1099.611</td><td>-0.0015</td><td>-1.3</td><td>4.37</td><td>57391.2</td><td>6.29</td><td>HUMAN</td><td>O43175</td><td>D-3-phosphoglycerate dehydrogenase</td></tr>
<tr><td>4700</td><td>dirMS_041712_NAR_40mM_plus2.17466.17466.3</td><td>3</td><td>13.38</td><td>1</td><td>3.17E+05</td><td></td><td>(K)TGISDVF</td><td>(K)T G\I|S/D/V\F A K N D\L/A/V V D V/R(I)</td><td></td><td>169.33</td><td>640.3451</td><td>1919.023</td><td>-0.0025</td><td>-1.3</td><td>4.43</td><td>76670.1</td><td>4.6</td><td>HUMAN</td><td>P19338</td><td>Nucleolin</td></tr>
<tr><td>4701</td><td>dirMS_041712_NAR_60mM_plus.16770.16770.3</td><td>3</td><td>13.38</td><td>0</td><td>2.63E+05</td><td></td><td>(R)YQEALH</td><td>(R)Y Q|E/A L/H|L/G S Q/L L R(E)</td><td></td><td>144.15</td><td>509.946</td><td>1527.828</td><td>-0.0044</td><td>-2.9</td><td>6.75</td><td>47748.2</td><td>6.08</td><td>HUMAN</td><td>O00231</td><td>26S proteasome non-ATPase regulatory subunit 11</td></tr>
<tr><td>4702</td><td>dirMS_041712_NAR_120mM_minus.11368.11481.3</td><td>3</td><td>13.37</td><td>0</td><td>2.31E+05</td><td></td><td>(R)GFCLPPI</td><td>(R)G F|C L/P/P/H/C S/R(G)</td><td>C:Carbamidomethylation</td><td>116.42</td><td>410.858</td><td>1230.551</td><td>0.0088</td><td>7.1</td><td>9.75</td><td>14391.8</td><td>4.99</td><td>HUMAN</td><td>G3V461</td><td>Creatine kinase B-type (Fragment)</td></tr>
<tr><td>4703</td><td>dirMS_041712_NAR_20mM_minus.14886.14888.2</td><td>2</td><td>13.37</td><td>0</td><td>2.08E+06</td><td></td><td>(K)EIIDLVLI</td><td>(K)E I|I/D/L|V/L D/R(I)</td><td></td><td>204</td><td>543.3133</td><td>1085.62</td><td>-0.0008</td><td>-0.7</td><td>4.03</td><td>58642</td><td>4.95</td><td>HUMAN</td><td>F5H5D3</td><td>Tubulin alpha-1C chain</td></tr>
<tr><td>4704</td><td>dirMS_041712_NAR_30mM_minus2.16345.16345.2</td><td>2</td><td>13.37</td><td>0</td><td>1.94E+05</td><td></td><td>(K)ACGNFG</td><td>(K)A C/G/N/F|G I P C E L R(V)</td><td>C:Carbamidomethylation</td><td>142.53</td><td>697.3207</td><td>1393.635</td><td>-0.001</td><td>-0.7</td><td>6.05</td><td>37681.5</td><td>5.93</td><td>HUMAN</td><td>D6RF62</td><td>Phosphoribosylaminoimidazole carboxylase</td></tr>
<tr><td>4705</td><td>dirMS_041712_NAR_30mM_minus2.19443.19443.2</td><td>2</td><td>13.37</td><td>0</td><td>2.19E+05</td><td></td><td>(R)ILVATNL</td><td>(R)I L|V/A|T/N L/F G R(G)</td><td></td><td>161.95</td><td>552.3309</td><td>1103.657</td><td>-0.0026</td><td>-2.4</td><td>9.75</td><td>40918.8</td><td>9.33</td><td>HUMAN</td><td>B4DP52</td><td>HCG2005638, isoform CRA_c</td></tr>
<tr><td>4706</td><td>dirMS_041712_NAR_40mM_minus2.5909.5909.2</td><td>2</td><td>13.37</td><td>0</td><td>1.30E+06</td><td></td><td>(K)AGFAGD</td><td>(K)A G/F|A/G D D/A/P R(A)</td><td></td><td>98.15</td><td>488.7279</td><td>976.448</td><td>0.0002</td><td>0.2</td><td>4.21</td><td>123097.1</td><td>5.83</td><td>HUMAN</td><td>A5A3E0</td><td>POTE ankyrin domain family member F</td></tr>
<tr><td>4707</td><td>dirMS_041712_NAR_50mM_minus2.12648.12673.3</td><td>3</td><td>13.37</td><td>1</td><td>3.98E+05</td><td></td><td>(K)NTVELL\</td><td>(K)N T|V|E/L/L V E/D K/G N Q V Y R(C)</td><td></td><td>140.43</td><td>626.3309</td><td>1876.976</td><td>0.0018</td><td>1</td><td>4.68</td><td>258215.9</td><td>5.48</td><td>HUMAN</td><td>E7EN95</td><td>Filamin-B</td></tr>
<tr><td>4708</td><td>dirMS_041712_NAR_50mM_minus2.13092.13092.3</td><td>3</td><td>13.37</td><td>1</td><td>2.73E+04</td><td></td><td>(K)VVSSSIV</td><td>(K)V V|S\S S I V D K/Y I G E/S A R(L)</td><td></td><td>142.6</td><td>570.6413</td><td>1709.907</td><td>0.0025</td><td>1.5</td><td>6.04</td><td>29938.9</td><td>6.07</td><td>HUMAN</td><td>H0YJC0</td><td>26S protease regulatory subunit 10B (Fragment)</td></tr>
<tr><td>4709</td><td>dirMS_041712_NAR_50mM_minus2.13319.13319.3</td><td>3</td><td>13.37</td><td>0</td><td>3.99E+05</td><td></td><td>(R)EQTLSP1</td><td>(R)E Q T/L/S/P T/I/T/S G L H N\I A R(S)</td><td></td><td>143.55</td><td>613.3274</td><td>1837.977</td><td>-0.009</td><td>-4.9</td><td>6.85</td><td>128523.4</td><td>7.06</td><td>HUMAN</td><td>B7ZL00</td><td>Protein transport protein Sec31A</td></tr>
<tr><td>4710</td><td>dirMS_041712_NAR_50mM_minus2.9744.9744.3</td><td>3</td><td>13.37</td><td>0</td><td>2.51E+05</td><td></td><td>(K)TSEVDL/</td><td>(K)T/S/E V D/L A/K/P L/V/K(F)</td><td></td><td>123.18</td><td>433.9205</td><td>1299.752</td><td>-0.0049</td><td>-3.8</td><td>5.73</td><td>17178.3</td><td>5.63</td><td>HUMAN</td><td>C9IZF9</td><td>Programmed cell death 6-interacting protein (Fragment)</td></tr>
<tr><td>4711</td><td>dirMS_041712_NAR_60mM_minus.12120.12120.3</td><td>3</td><td>13.37</td><td>1</td><td>2.15E+05</td><td></td><td>(K)YADEEIP</td><td>(K)Y/A|D/E E I/P R S P F K(V)</td><td></td><td>127.3</td><td>484.5732</td><td>1451.717</td><td>-0.0115</td><td>-7.9</td><td>4.68</td><td>258215.9</td><td>5.48</td><td>HUMAN</td><td>E7EN95</td><td>Filamin-B</td></tr>
<tr><td>4712</td><td>dirMS_041712_NAR_60mM_minus.6159.6166.3</td><td>3</td><td>13.37</td><td>1</td><td>2.64E+05</td><td></td><td>(K)LEEAEK/</td><td>(K)L/E|E A E/K/A A/D/E S E/R(G)</td><td></td><td>92.57</td><td>492.8981</td><td>1476.681</td><td>-0.0015</td><td>-1</td><td>4.14</td><td>31923.6</td><td>4.85</td><td>HUMAN</td><td>B7Z596</td><td>Tropomyosin alpha-1 chain</td></tr>
<tr><td>4713</td><td>dirMS_041712_NAR_20mM_plus.11000.11000.2</td><td>2</td><td>13.37</td><td>0</td><td>1.17E+05</td><td></td><td>(K)EITALAP</td><td>(K)E I/T|A/L|A P S T M K(I)</td><td></td><td>148.15</td><td>581.3129</td><td>1161.618</td><td>0.0001</td><td>0.1</td><td>6.1</td><td>32616.5</td><td>5.18</td><td>HUMAN</td><td>A6NL76</td><td>Actin, alpha skeletal muscle</td></tr>
<tr><td>4714</td><td>dirMS_041712_NAR_50mM_plus2.11427.11427.3</td><td>3</td><td>13.37</td><td>1</td><td>3.29E+05</td><td></td><td>(K)VAVVAG</td><td>(K)V/A|V|V/A/G Y G D V G/K G C A Q A L R(G)</td><td>C:Carbamidomethylation</td><td>127.85</td><td>630.9984</td><td>1890.985</td><td>-0.0048</td><td>-2.5</td><td>8.56</td><td>45228.3</td><td>6.03</td><td>HUMAN</td><td>F5H737</td><td>Adenosylhomocysteinase</td></tr>
<tr><td>4715</td><td>dirMS_041712_NAR_50mM_plus2.15125.15125.3</td><td>3</td><td>13.37</td><td>1</td><td>4.23E+05</td><td></td><td>(R)LSQELEY</td><td>(R)L/S|Q/E/L E/Y L T E D V K R(L)</td><td></td><td>147.3</td><td>574.9669</td><td>1722.891</td><td>-0.0047</td><td>-2.7</td><td>4.41</td><td>267687.4</td><td>4.97</td><td>HUMAN</td><td>P12270</td><td>Nucleoprotein TPR</td></tr>
<tr><td>4716</td><td>dirMS_041712_NAR_60mM_plus.11861.12249.3</td><td>3</td><td>13.37</td><td>1</td><td>7.49E+06</td><td></td><td>(R)FPGQLN</td><td>(R)F/P/G Q|L|N/A/D/L R K(L)</td><td></td><td>119.62</td><td>420.23</td><td>1258.69</td><td>-0.0148</td><td>-11.8</td><td>8.75</td><td>42230.8</td><td>4.77</td><td>HUMAN</td><td>A6NKZ8</td><td>Putative tubulin beta chain-like protein ENSP00000290377</td></tr>
<tr><td>4717</td><td>dirMS_041712_NAR_60mM_plus.11870.11870.3</td><td>3</td><td>13.37</td><td>1</td><td>7.13E+05</td><td>M239m</td><td>(R)MSMKE'</td><td>(R)M/S M K E V D E Q/m|L/N/V\Q/N\K(N)</td><td>m:Oxidized methionine</td><td>119.38</td><td>647.3021</td><td>1923.897</td><td>15.9945</td><td>-0.2</td><td>4.68</td><td>40069.4</td><td>4.83</td><td>HUMAN</td><td>E7EWR1</td><td>Tubulin beta chain</td></tr>
<tr><td>4718</td><td>dirMS_041712_NAR_120mM_minus.10924.11026.3</td><td>3</td><td>13.36</td><td>1</td><td>6.07E+05</td><td></td><td>(R)FYKNEG</td><td>(R)F Y/K N E G/G/T/W/S/V/E\K(V)</td><td></td><td>113.78</td><td>515.5834</td><td>1544.738</td><td>-0.0023</td><td>-1.5</td><td>6.14</td><td>39066</td><td>6.61</td><td>HUMAN</td><td>A6PVW9</td><td>Selenium binding protein 1 (Fragment)</td></tr>
<tr><td>4719</td><td>dirMS_041712_NAR_20mM_minus.13741.13741.2</td><td>2</td><td>13.36</td><td>0</td><td>3.90E+05</td><td></td><td>(K)LGGSAV</td><td>(K)L G G S\A V\I|S L E/G K|P L(-)</td><td></td><td>188.65</td><td>670.8912</td><td>1340.778</td><td>-0.0033</td><td>-2.4</td><td>6</td><td>22956</td><td>8.8</td><td>HUMAN</td><td>E9PK25</td><td>Cofilin-1</td></tr>
<tr><td>4720</td><td>dirMS_041712_NAR_30mM_minus2.14083.14329.2</td><td>2</td><td>13.36</td><td>0</td><td>5.90E+05</td><td></td><td>(R)SEQFINL</td><td>(R)S E|Q|F|I/N/L/R(E)</td><td></td><td>130.73</td><td>503.7677</td><td>1006.532</td><td>-0.0035</td><td>-3.5</td><td>5.72</td><td>82516.1</td><td>5.49</td><td>HUMAN</td><td>P07384</td><td>Calpain-1 catalytic subunit</td></tr>
<tr><td>4721</td><td>dirMS_041712_NAR_30mM_minus2.15799.15799.2</td><td>2</td><td>13.36</td><td>0</td><td>2.84E+05</td><td></td><td>(K)EGALDL</td><td>(K)E G\A|L/D/L/S G L R(A)</td><td></td><td>139.08</td><td>515.7847</td><td>1030.553</td><td>0.0094</td><td>9.1</td><td>4.37</td><td>56522.6</td><td>6.66</td><td>HUMAN</td><td>B4DW73</td><td>Phosphoenolpyruvate carboxykinase [GTP], mitochondrial</td></tr>
<tr><td>4722</td><td>dirMS_041712_NAR_30mM_minus2.16076.16076.2</td><td>2</td><td>13.36</td><td>0</td><td>1.21E+05</td><td></td><td>(K)SSEELLS</td><td>(K)S S|E E|L|L|S A L\Q/K(K)</td><td></td><td>141.47</td><td>602.816</td><td>1204.642</td><td>-0.0172</td><td>-14.3</td><td>4.53</td><td>150202.8</td><td>9.1</td><td>HUMAN</td><td>E7EW20</td><td>Unconventional myosin-VI</td></tr>
<tr><td>4723</td><td>dirMS_041712_NAR_40mM_minus2.8165.8165.3</td><td>3</td><td>13.36</td><td>0</td><td>2.24E+05</td><td>M35m</td><td>(K)AASDIAr</td><td>(K)A A|S D I A/m T E L/P/P T H P/I R(L)</td><td>m:Oxidized methionine</td><td>128.48</td><td>612.6484</td><td>1819.937</td><td>15.9936</td><td>-0.7</td><td>5.32</td><td>12926.1</td><td>4.4</td><td>HUMAN</td><td>K7EM20</td><td>14-3-3 protein epsilon (Fragment)</td></tr>
<tr><td>4724</td><td>dirMS_041712_NAR_60mM_minus.13418.13418.3</td><td>3</td><td>13.36</td><td>1</td><td>4.64E+05</td><td></td><td>(K)TKEVYEL</td><td>(K)T K/E V\Y E|L|L D/S/P G K(V)</td><td></td><td>134.5</td><td>493.5966</td><td>1478.774</td><td>0.0015</td><td>1</td><td>4.68</td><td>46335.4</td><td>6.29</td><td>HUMAN</td><td>E9PBS1</td><td>Phosphoribosylaminoimidazole carboxylase (Fragment)</td></tr>
<tr><td>4725</td><td>dirMS_041712_NAR_60mM_minus.9769.9865.3</td><td>3</td><td>13.36</td><td>1</td><td>8.70E+05</td><td></td><td>(R)VKYETEL</td><td>(R)V/K Y E\T\E|L/A/M/R(Q)</td><td></td><td>114.7</td><td>413.8839</td><td>1239.64</td><td>-0.003</td><td>-2.5</td><td>6.11</td><td>43773.3</td><td>5.26</td><td>HUMAN</td><td>F8VZY9</td><td>Keratin, type I cytoskeletal 18</td></tr>
<tr><td>4726</td><td>dirMS_041712_NAR_120mM_plus.8149.8149.2</td><td>2</td><td>13.36</td><td>0</td><td>4.29E+05</td><td></td><td>(K)AGEVFIH</td><td>(K)A/G E|V/F/I/H/K(D)</td><td></td><td>96.3</td><td>450.7531</td><td>900.494</td><td>0.0051</td><td>5.7</td><td>6.79</td><td>23729.5</td><td>9.89</td><td>HUMAN</td><td>C9IZL7</td><td>Non-POU domain-containing octamer-binding protein (Fragment)</td></tr>
<tr><td>4727</td><td>dirMS_041712_NAR_40mM_plus2.17766.17767.3</td><td>3</td><td>13.36</td><td>1</td><td>1.96E+05</td><td></td><td>(R)LKLPSIPI</td><td>(R)L K\L|P S I|P\L|V|P V S A Q K(R)</td><td></td><td>170.73</td><td>530.671</td><td>1589.999</td><td>-0.0004</td><td>-0.3</td><td>10</td><td>34958.1</td><td>9.42</td><td>HUMAN</td><td>Q15785</td><td>Mitochondrial import receptor subunit TOM34</td></tr>
<tr><td>4728</td><td>dirMS_041712_NAR_50mM_plus2.10069.10069.3</td><td>3</td><td>13.36</td><td>1</td><td>2.46E+06</td><td></td><td>(K)ATAVVD</td><td>(K)A T/A/V/V/D G A F/K E V K(L)</td><td></td><td>120.07</td><td>445.5822</td><td>1334.731</td><td>0.0006</td><td>0.5</td><td>6.11</td><td>15252.2</td><td>5.82</td><td>HUMAN</td><td>A6NIW5</td><td>Peroxiredoxin 2, isoform CRA_a</td></tr>
<tr><td>4729</td><td>dirMS_041712_NAR_50mM_plus2.14758.14967.3</td><td>3</td><td>13.36</td><td>1</td><td>1.17E+04</td><td></td><td>(K)YMACCL</td><td>(K)Y/M|A/C/C L L Y R G D\V V/P/K(E)</td><td>C:Carbamidomethylation</td><td>144.47</td><td>615.6348</td><td>1844.886</td><td>0.0043</td><td>2.3</td><td>8.49</td><td>50706.6</td><td>5.68</td><td>HUMAN</td><td>A6NHL2</td><td>Tubulin alpha chain-like 3</td></tr>
<tr><td>4730</td><td>dirMS_041712_NAR_50mM_plus2.5685.5685.3</td><td>3</td><td>13.36</td><td>1</td><td>1.06E+06</td><td></td><td>(K)TAGPIAS</td><td>(K)T A/G\P I/A/S A Q\K Q P A G K(V)</td><td></td><td>91.9</td><td>475.5987</td><td>1424.786</td><td>-0.0041</td><td>-2.8</td><td>10</td><td>75259.2</td><td>9.59</td><td>HUMAN</td><td>B5MEG9</td><td>Microtubule-associated protein 4 (Fragment)</td></tr>
<tr><td>4731</td><td>dirMS_041712_NAR_60mM_plus.12928.13025.3</td><td>3</td><td>13.36</td><td>0</td><td>6.26E+05</td><td></td><td>(K)VFEGNR</td><td>(K)V/F|E/G N R P T N S I V/F/T/K(L)</td><td></td><td>124.57</td><td>570.3039</td><td>1708.902</td><td>-0.0045</td><td>-2.7</td><td>8.72</td><td>63374.2</td><td>8.64</td><td>HUMAN</td><td>P06744</td><td>Glucose-6-phosphate isomerase</td></tr>
<tr><td>4732</td><td>dirMS_041712_NAR_60mM_plus.17830.17888.3</td><td>3</td><td>13.36</td><td>1</td><td>8.12E+05</td><td></td><td>(R)LKLPAV\</td><td>(R)L K L P A\V|V|T/A/D/L/R(L)</td><td></td><td>150.67</td><td>432.6056</td><td>1295.805</td><td>-0.0023</td><td>-1.8</td><td>8.75</td><td>28071.3</td><td>8.6</td><td>HUMAN</td><td>P38117</td><td>Electron transfer flavoprotein subunit beta</td></tr>
<tr><td>4733</td><td>dirMS_041712_NAR_20mM_minus.13070.13070.2</td><td>2</td><td>13.35</td><td>0</td><td>1.79E+05</td><td></td><td>(K)LLCGLLA</td><td>(K)L L|C/G/L/L A E/R(L)</td><td>C:Carbamidomethylation</td><td>181.28</td><td>522.7982</td><td>1044.587</td><td>0.0021</td><td>2</td><td>6</td><td>12647.2</td><td>8.07</td><td>HUMAN</td><td>P14174</td><td>Macrophage migration inhibitory factor</td></tr>
<tr><td>4734</td><td>dirMS_041712_NAR_20mM_minus.8618.8711.2</td><td>2</td><td>13.35</td><td>0</td><td>1.86E+05</td><td></td><td>(K)LVLVGD(</td><td>(K)L V|L|V/G D/G G/T G K(T)</td><td></td><td>136.13</td><td>508.2919</td><td>1015.578</td><td>-0.0017</td><td>-1.7</td><td>5.84</td><td>26394.9</td><td>6.5</td><td>HUMAN</td><td>B5MDF5</td><td>GTP-binding nuclear protein Ran</td></tr>
<tr><td>4735</td><td>dirMS_041712_NAR_30mM_minus2.10129.10205.2</td><td>2</td><td>13.35</td><td>0</td><td>7.62E+05</td><td>M161m</td><td>(K)LLIEmEC</td><td>(K)L/L|I/E/m E/Q/R(L)</td><td>m:Oxidized methionine</td><td>109.23</td><td>524.2789</td><td>1031.555</td><td>15.9951</td><td>0.2</td><td>4.53</td><td>20203.9</td><td>6.24</td><td>HUMAN</td><td>H0YJG0</td><td>Creatine kinase B-type (Fragment)</td></tr>
<tr><td>4736</td><td>dirMS_041712_NAR_30mM_minus2.13432.13667.2</td><td>2</td><td>13.35</td><td>0</td><td>4.74E+05</td><td></td><td>(K)NLVTED'</td><td>(K)N L|V|T|E D/V M/R(M)</td><td></td><td>126.98</td><td>538.7747</td><td>1076.541</td><td>0.0017</td><td>1.5</td><td>4.37</td><td>12797.5</td><td>10.58</td><td>HUMAN</td><td>K7EIL4</td><td>Glucose-6-phosphate isomerase (Fragment)</td></tr>
<tr><td>4737</td><td>dirMS_041712_NAR_30mM_minus2.17903.18390.2</td><td>2</td><td>13.35</td><td>0</td><td>1.62E+06</td><td></td><td>(R)GLFIIDG</td><td>(R)G L|F|I I D/G K(G)</td><td></td><td>153.33</td><td>431.7497</td><td>862.503</td><td>-0.0112</td><td>-12.9</td><td>5.84</td><td>22062.7</td><td>5.66</td><td>HUMAN</td><td>P32119</td><td>Peroxiredoxin-2</td></tr>
<tr><td>4738</td><td>dirMS_041712_NAR_40mM_minus2.14721.14721.3</td><td>3</td><td>13.35</td><td>1</td><td>1.38E+05</td><td></td><td>(R)EQIPAG'</td><td>(R)E/Q\I/P A G T L L/L A V E D P E K R(V)</td><td></td><td>170.83</td><td>660.3663</td><td>1979.081</td><td>0.0036</td><td>1.8</td><td>4.41</td><td>19773.1</td><td>4.48</td><td>HUMAN</td><td>B4DEU5</td><td>L-fucose kinase</td></tr>
<tr><td>4739</td><td>dirMS_041712_NAR_50mM_minus2.9237.9239.3</td><td>3</td><td>13.35</td><td>1</td><td>2.01E+05</td><td></td><td>(K)GITEQQI</td><td>(K)G I|T E Q/Q/K/E G L E I/V/K(M)</td><td></td><td>120.58</td><td>524.6237</td><td>1571.864</td><td>-0.0074</td><td>-4.7</td><td>4.79</td><td>83273.6</td><td>5.55</td><td>HUMAN</td><td>P13010</td><td>X-ray repair cross-complementing protein 5</td></tr>
<tr><td>4740</td><td>dirMS_041712_NAR_50mM_minus2.9460.9460.3</td><td>3</td><td>13.35</td><td>0</td><td>1.88E+04</td><td></td><td>(K)ADHTLS</td><td>(K)A D H T L S S L/E|P/A/Y S A K(W)</td><td></td><td>122.27</td><td>530.5989</td><td>1589.781</td><td>0.0016</td><td>1</td><td>5.32</td><td>43384.7</td><td>7.78</td><td>HUMAN</td><td>B7Z9G5</td><td>BRO1 domain-containing protein BROX</td></tr>
<tr><td>4741</td><td>dirMS_041712_NAR_60mM_minus.13131.13151.3</td><td>3</td><td>13.35</td><td>1</td><td>1.52E+05</td><td></td><td>(R)VLITEND</td><td>(R)V/L|I|T E N D/V/K/E G L Q R(S)</td><td></td><td>132.97</td><td>538.6313</td><td>1613.886</td><td>-0.0064</td><td>-3.9</td><td>4.68</td><td>72873.4</td><td>5.73</td><td>HUMAN</td><td>Q96S55</td><td>ATPase WRNIP1</td></tr>
<tr><td>4742</td><td>dirMS_041712_NAR_60mM_minus.9469.9469.3</td><td>3</td><td>13.35</td><td>1</td><td>6.72E+05</td><td></td><td>(K)TFDQLTI</td><td>(K)T F|D Q L/T/P D/E S K E R(L)</td><td></td><td>113.37</td><td>522.5846</td><td>1565.744</td><td>-0.0049</td><td>-3.2</td><td>4.32</td><td>17272.1</td><td>6.63</td><td>HUMAN</td><td>E9PJD5</td><td>Reticulocalbin-1</td></tr>
<tr><td>4743</td><td>dirMS_041712_NAR_120mM_plus.11218.11218.3</td><td>3</td><td>13.35</td><td>0</td><td>5.10E+05</td><td></td><td>(R)SIFQHIQ</td><td>(R)S I|F Q/H/I Q S A Q S Q R(S)</td><td></td><td>112.47</td><td>510.5986</td><td>1529.782</td><td>-0.0007</td><td>-0.4</td><td>9.49</td><td>108720.9</td><td>10.17</td><td>HUMAN</td><td>Q9Y2W1</td><td>Thyroid hormone receptor-associated protein 3</td></tr>
<tr><td>4744</td><td>dirMS_041712_NAR_20mM_plus.16397.16415.3</td><td>3</td><td>13.35</td><td>0</td><td>1.35E+05</td><td></td><td>(R)QAQEYE</td><td>(R)Q A\Q E\Y\E/A|L/L N I K(V)</td><td></td><td>184.45</td><td>473.9191</td><td>1419.748</td><td>-0.0051</td><td>-3.6</td><td>4.53</td><td>43773.3</td><td>5.26</td><td>HUMAN</td><td>F8VZY9</td><td>Keratin, type I cytoskeletal 18</td></tr>
<tr><td>4745</td><td>dirMS_041712_NAR_20mM_plus.7308.7308.2</td><td>2</td><td>13.35</td><td>0</td><td>2.85E+04</td><td></td><td>(R)VLEEGS\</td><td>(R)V/L|E\E G S V E A/R(T)</td><td></td><td>118.03</td><td>544.7829</td><td>1088.558</td><td>0.0003</td><td>0.3</td><td>4.25</td><td>25834.8</td><td>9.54</td><td>HUMAN</td><td>Q96CT7</td><td>Coiled-coil domain-containing protein 124</td></tr>
<tr><td>4746</td><td>dirMS_041712_NAR_30mM_plus2.18151.18151.2</td><td>2</td><td>13.35</td><td>0</td><td>2.14E+05</td><td></td><td>(R)QITLNDI</td><td>(R)Q I/T\L|N/D\L/P V G R(S)</td><td></td><td>147.93</td><td>613.3465</td><td>1225.69</td><td>-0.0042</td><td>-3.4</td><td>5.84</td><td>18498.6</td><td>5.82</td><td>HUMAN</td><td>H7C3T4</td><td>Peroxiredoxin-4 (Fragment)</td></tr>
<tr><td>4747</td><td>dirMS_041712_NAR_30mM_plus2.20688.20688.2</td><td>2</td><td>13.35</td><td>0</td><td>8.74E+05</td><td></td><td>(R)TVQSLEI</td><td>(R)T V/Q/S L/E|I D L/D S M R(N)</td><td></td><td>162.18</td><td>753.8768</td><td>1506.747</td><td>-0.0005</td><td>-0.3</td><td>4.03</td><td>43773.3</td><td>5.26</td><td>HUMAN</td><td>F8VZY9</td><td>Keratin, type I cytoskeletal 18</td></tr>
<tr><td>4748</td><td>dirMS_041712_NAR_30mM_plus2.21395.21395.2</td><td>2</td><td>13.35</td><td>0</td><td>3.37E+04</td><td></td><td>(R)GAFQNL</td><td>(R)G A/F/Q|N/L/F Q S/V R(E)</td><td></td><td>168.02</td><td>633.8315</td><td>1266.659</td><td>-0.0032</td><td>-2.5</td><td>9.75</td><td>68629.9</td><td>5.59</td><td>HUMAN</td><td>E7EVX6</td><td>Androgen receptor</td></tr>
<tr><td>4749</td><td>dirMS_041712_NAR_30mM_plus2.22014.22014.2</td><td>2</td><td>13.35</td><td>0</td><td>1.55E+05</td><td></td><td>(K)ANFLQN</td><td>(K)A N/F/L/Q/N/M I E/D/R(T)</td><td></td><td>170.25</td><td>675.8282</td><td>1350.647</td><td>0.0021</td><td>1.5</td><td>4.37</td><td>120546</td><td>7.82</td><td>HUMAN</td><td>O95486</td><td>Protein transport protein Sec24A</td></tr>
<tr><td>4750</td><td>dirMS_041712_NAR_30mM_plus2.23516.23516.2</td><td>2</td><td>13.35</td><td>0</td><td>1.80E+05</td><td></td><td>(R)IAIYELLF</td><td>(R)I/A|I/Y/E L/L F/K(E)</td><td></td><td>190.25</td><td>555.3331</td><td>1109.661</td><td>-0.0016</td><td>-1.4</td><td>6</td><td>19924.8</td><td>9.99</td><td>HUMAN</td><td>F6U211</td><td>40S ribosomal protein S10</td></tr>
<tr><td>4751</td><td>dirMS_041712_NAR_40mM_plus2.9068.9068.2</td><td>2</td><td>13.35</td><td>0</td><td>4.81E+05</td><td></td><td>(R)GGNFGI</td><td>(R)G G/N F/G F|G D/S/R(G)</td><td></td><td>116.33</td><td>507.2256</td><td>1013.444</td><td>0.0004</td><td>0.4</td><td>5.84</td><td>37486.1</td><td>9.03</td><td>HUMAN</td><td>P22626</td><td>Heterogeneous nuclear ribonucleoproteins A2/B1</td></tr>
<tr><td>4752</td><td>dirMS_041712_NAR_50mM_plus2.14800.14800.3</td><td>3</td><td>13.35</td><td>0</td><td>6.51E+05</td><td></td><td>(K)SPAPPLL</td><td>(K)S/P|A|P/P L L H V/A A L G Q K(Q)</td><td></td><td>145.78</td><td>500.2911</td><td>1498.874</td><td>-0.0153</td><td>-10.2</td><td>8.49</td><td>17549.1</td><td>10.39</td><td>HUMAN</td><td>E5RFJ3</td><td>DBIRD complex subunit KIAA1967 (Fragment)</td></tr>
<tr><td>4753</td><td>dirMS_041712_NAR_60mM_plus.10377.10458.3</td><td>3</td><td>13.35</td><td>1</td><td>1.61E+06</td><td></td><td>(R)TNAENE</td><td>(R)T/N|A/E N E F V/T I/K\K(D)</td><td></td><td>112.27</td><td>465.2512</td><td>1393.732</td><td>0.0069</td><td>4.9</td><td>5.81</td><td>66208.7</td><td>8.32</td><td>HUMAN</td><td>P04264</td><td>Keratin, type II cytoskeletal 1</td></tr>
<tr><td>4754</td><td>dirMS_041712_NAR_60mM_plus.16336.16487.3</td><td>3</td><td>13.35</td><td>0</td><td>6.18E+05</td><td></td><td>(K)MTHLPE</td><td>(K)M T H\L P E\L/D|T/L/S/S E/R(A)</td><td></td><td>142.28</td><td>543.6023</td><td>1628.795</td><td>-0.0025</td><td>-1.5</td><td>4.65</td><td>141616.6</td><td>6.1</td><td>HUMAN</td><td>B7ZKM8</td><td>Protein transport protein Sec24B</td></tr>
<tr><td>4755</td><td>dirMS_041712_NAR_60mM_plus.20601.20726.3</td><td>3</td><td>13.35</td><td>0</td><td>7.27E+05</td><td></td><td>(R)LGFHLPI</td><td>(R)L G F H L\P L/E|V/A/Y/Q/R(G)</td><td></td><td>168.62</td><td>514.9568</td><td>1542.843</td><td>0.0131</td><td>8.5</td><td>6.75</td><td>533778</td><td>5.74</td><td>HUMAN</td><td>Q15149</td><td>Plectin</td></tr>
<tr><td>4756</td><td>dirMS_041712_NAR_120mM_minus.13976.13976.4</td><td>4</td><td>13.34</td><td>1</td><td>1.60E+06</td><td></td><td>(K)ASIHEA\</td><td>(K)A/S|I/H/E A W/T D/G K/E A M L K(H)</td><td></td><td>129.98</td><td>447.476</td><td>1786.879</td><td>0.0029</td><td>1.6</td><td>5.45</td><td>80254.2</td><td>5.05</td><td>HUMAN</td><td>D6PXK4</td><td>Alpha actinin 4 short isoform</td></tr>
<tr><td>4757</td><td>dirMS_041712_NAR_30mM_minus2.10243.10642.2</td><td>2</td><td>13.34</td><td>0</td><td>2.07E+06</td><td></td><td>(R)QTVAVG</td><td>(R)Q T|V/A/V/G V/I/K(A)</td><td></td><td>110.7</td><td>457.7858</td><td>914.567</td><td>-0.0026</td><td>-2.9</td><td>8.75</td><td>50482.3</td><td>9.31</td><td>HUMAN</td><td>P68104</td><td>Elongation factor 1-alpha 1</td></tr>
<tr><td>4758</td><td>dirMS_041712_NAR_50mM_minus2.10400.10400.3</td><td>3</td><td>13.34</td><td>0</td><td>2.78E+05</td><td>M35m</td><td>(K)AASDIAr</td><td>(K)A A/S D\I/A/m T E L/P/P T H/P I R(L)</td><td>m:Oxidized methionine</td><td>127.6</td><td>612.6486</td><td>1819.937</td><td>15.9942</td><td>-0.4</td><td>5.32</td><td>12926.1</td><td>4.4</td><td>HUMAN</td><td>K7EM20</td><td>14-3-3 protein epsilon (Fragment)</td></tr>
<tr><td>4759</td><td>dirMS_041712_NAR_50mM_minus2.11268.11269.3</td><td>3</td><td>13.34</td><td>0</td><td>1.66E+04</td><td></td><td>(R)VVVLMC</td><td>(R)V/V|V|L/M/G S T/S D L G H C E K(I)</td><td>C:Carbamidomethylation</td><td>132.28</td><td>577.9535</td><td>1731.84</td><td>0.0055</td><td>3.2</td><td>5.32</td><td>37681.5</td><td>5.93</td><td>HUMAN</td><td>D6RF62</td><td>Phosphoribosylaminoimidazole carboxylase</td></tr>
<tr><td>4760</td><td>dirMS_041712_NAR_50mM_minus2.11764.11764.3</td><td>3</td><td>13.34</td><td>1</td><td>4.83E+05</td><td></td><td>(K)KVPQVS</td><td>(K)K\V P Q V S/T/P T|L/V/E/V S R(N)</td><td></td><td>135.22</td><td>547.3172</td><td>1639.938</td><td>-0.0007</td><td>-0.4</td><td>8.75</td><td>57751.3</td><td>6.9</td><td>HUMAN</td><td>B7WNR0</td><td>Serum albumin</td></tr>
<tr><td>4761</td><td>dirMS_041712_NAR_50mM_minus2.7935.7935.3</td><td>3</td><td>13.34</td><td>0</td><td>8.88E+04</td><td></td><td>(K)TAVEHA</td><td>(K)T/A/V E/H A/T/D E D I/L A K(G)</td><td></td><td>112.23</td><td>504.9229</td><td>1512.754</td><td>0.0001</td><td>0.1</td><td>4.31</td><td>288852.1</td><td>5.33</td><td>HUMAN</td><td>E9PEM5</td><td>Lipopolysaccharide-responsive and beige-like anchor protein</td></tr>
<tr><td>4762</td><td>dirMS_041712_NAR_60mM_minus.15391.15519.3</td><td>3</td><td>13.34</td><td>0</td><td>4.11E+05</td><td></td><td>(K)EGHVEV</td><td>(K)E/G H V E\V\V|S/E/L/L Q/R(E)</td><td></td><td>146.25</td><td>498.9349</td><td>1494.791</td><td>-0.0009</td><td>-0.6</td><td>4.75</td><td>48586.6</td><td>9.32</td><td>HUMAN</td><td>D6RCB2</td><td>Ankyrin-3</td></tr>
<tr><td>4763</td><td>dirMS_041712_NAR_120mM_plus.6241.6385.3</td><td>3</td><td>13.34</td><td>1</td><td>5.04E+05</td><td></td><td>(R)ARSDEG</td><td>(R)A R S D E\G\Q|L|S|P/A T R(G)</td><td></td><td>86.7</td><td>463.2395</td><td>1387.692</td><td>0.0115</td><td>8.3</td><td>6.12</td><td>81007.1</td><td>6.31</td><td>HUMAN</td><td>E9PMV1</td><td>Plectin (Fragment)</td></tr>
<tr><td>4764</td><td>dirMS_041712_NAR_30mM_plus2.6362.6363.2</td><td>2</td><td>13.34</td><td>0</td><td>2.46E+05</td><td></td><td>(K)SGYLSSE</td><td>(K)S/G|Y|L|S/S E/R(L)</td><td></td><td>87.22</td><td>449.7152</td><td>898.427</td><td>-0.0034</td><td>-3.7</td><td>5.72</td><td>65692.2</td><td>5.7</td><td>HUMAN</td><td>E7EQR4</td><td>Ezrin</td></tr>
<tr><td>4765</td><td>dirMS_041712_NAR_40mM_plus2.19762.19762.3</td><td>3</td><td>13.34</td><td>1</td><td>4.44E+05</td><td></td><td>(R)ILDDDTI</td><td>(R)I L|D/D D T I/I/T T L E N L K R(E)</td><td></td><td>185.12</td><td>625.0111</td><td>1873.028</td><td>-0.0089</td><td>-4.8</td><td>4.23</td><td>535136.6</td><td>6.01</td><td>HUMAN</td><td>Q14204</td><td>Cytoplasmic dynein 1 heavy chain 1</td></tr>
<tr><td>4766</td><td>dirMS_041712_NAR_50mM_plus2.10974.11014.3</td><td>3</td><td>13.34</td><td>1</td><td>4.55E+05</td><td></td><td>(R)AIPNNQ</td><td>(R)A I|P/N N Q/V/L/G\K I/E R(A)</td><td></td><td>125.48</td><td>484.6151</td><td>1451.833</td><td>-0.0021</td><td>-1.5</td><td>8.79</td><td>16368.4</td><td>9.95</td><td>HUMAN</td><td>O60869</td><td>Endothelial differentiation-related factor 1</td></tr>
<tr><td>4767</td><td>dirMS_041712_NAR_50mM_plus2.11818.11818.3</td><td>3</td><td>13.34</td><td>1</td><td>1.75E+06</td><td></td><td>(K)TGQPMI</td><td>(K)T G\Q|P/M/I N L Y T D R E T G K(L)</td><td></td><td>130.72</td><td>608.6361</td><td>1823.896</td><td>-0.0019</td><td>-1</td><td>5.73</td><td>53724.2</td><td>9.54</td><td>HUMAN</td><td>H3BPE7</td><td>RNA-binding protein FUS</td></tr>
<tr><td>4768</td><td>dirMS_041712_NAR_120mM_minus.7431.7431.3</td><td>3</td><td>13.33</td><td>0</td><td>1.20E+05</td><td></td><td>(R)NIDEHA</td><td>(R)N I/D E/H/A/N/E D V E/R(M)</td><td></td><td>94.67</td><td>480.884</td><td>1440.635</td><td>0.0025</td><td>1.7</td><td>4.17</td><td>22768.7</td><td>8.94</td><td>HUMAN</td><td>P61026</td><td>Ras-related protein Rab-10</td></tr>
<tr><td>4769</td><td>dirMS_041712_NAR_50mM_minus2.12147.12147.3</td><td>3</td><td>13.33</td><td>0</td><td>9.09E+03</td><td></td><td>(K)AASAGV</td><td>(K)A A|S\A/G\V/P Y H G E/V/P V S L A R(A)</td><td></td><td>135.98</td><td>594.315</td><td>1780.934</td><td>-0.0036</td><td>-2</td><td>6.79</td><td>272689.3</td><td>5.79</td><td>HUMAN</td><td>O15020</td><td>Spectrin beta chain, non-erythrocytic 2</td></tr>
<tr><td>4770</td><td>dirMS_041712_NAR_50mM_minus2.14372.14372.3</td><td>3</td><td>13.33</td><td>1</td><td>1.09E+06</td><td></td><td>(K)EAFSLFD</td><td>(K)E A/F/S L/F/D\K D G D G T I T T/K(E)</td><td></td><td>149.93</td><td>615.6336</td><td>1844.891</td><td>-0.005</td><td>-2.7</td><td>4.23</td><td>21860.1</td><td>4.45</td><td>HUMAN</td><td>E7EMB3</td><td>Calmodulin</td></tr>
<tr><td>4771</td><td>dirMS_041712_NAR_50mM_minus2.15740.15740.3</td><td>3</td><td>13.33</td><td>0</td><td>1.29E+05</td><td></td><td>(K)FTISDHP</td><td>(K)F/T|I/S/D H|P Q\P I D/P L L K(N)</td><td></td><td>157.77</td><td>574.3122</td><td>1720.927</td><td>-0.0048</td><td>-2.8</td><td>5.21</td><td>138084.9</td><td>5.52</td><td>HUMAN</td><td>Q86VP6</td><td>Cullin-associated NEDD8-dissociated protein 1</td></tr>
<tr><td>4772</td><td>dirMS_041712_NAR_60mM_minus.14207.14207.3</td><td>3</td><td>13.33</td><td>1</td><td>2.27E+05</td><td></td><td>(R)TFNTST(</td><td>(R)T F|N/T S\T G G L/L/L|P S D T K R(S)</td><td></td><td>138.82</td><td>603.3262</td><td>1807.955</td><td>0.0092</td><td>5.1</td><td>8.41</td><td>64302.5</td><td>6.4</td><td>HUMAN</td><td>B1AHC7</td><td>X-ray repair complementing defective repair in Chinese hamster cells 6 (Ku autoantigen, 70kDa)</td></tr>
<tr><td>4773</td><td>dirMS_041712_NAR_120mM_plus.15951.15951.3</td><td>3</td><td>13.33</td><td>1</td><td>8.08E+04</td><td></td><td>(K)THLPGF'</td><td>(K)T\H/L|P G F\V|E\Q/A/E A/L K/A K(G)</td><td></td><td>137.02</td><td>580.3208</td><td>1738.949</td><td>-0.0008</td><td>-0.5</td><td>6.42</td><td>22314.2</td><td>9.39</td><td>HUMAN</td><td>P30044</td><td>Peroxiredoxin-5, mitochondrial</td></tr>
<tr><td>4774</td><td>dirMS_041712_NAR_120mM_plus.18256.18316.4</td><td>4</td><td>13.33</td><td>1</td><td>1.06E+06</td><td></td><td>(K)EMVELP</td><td>(K)E M V|E/L/P L R H/P A L/F/K(A)</td><td></td><td>149.07</td><td>420.7376</td><td>1679.93</td><td>-0.0016</td><td>-0.9</td><td>6.86</td><td>90004.9</td><td>5.14</td><td>HUMAN</td><td>P55072</td><td>Transitional endoplasmic reticulum ATPase</td></tr>
<tr><td>4775</td><td>dirMS_041712_NAR_120mM_plus.21172.21172.4</td><td>4</td><td>13.33</td><td>1</td><td>1.17E+06</td><td></td><td>(R)DYLHLPI</td><td>(R)D Y L H L\P/P/E I/V/P A T L R R(S)</td><td></td><td>164.15</td><td>473.2689</td><td>1890.06</td><td>-0.0058</td><td>-3.1</td><td>6.75</td><td>19924.8</td><td>9.99</td><td>HUMAN</td><td>F6U211</td><td>40S ribosomal protein S10</td></tr>
<tr><td>4776</td><td>dirMS_041712_NAR_30mM_plus2.10174.10174.2</td><td>2</td><td>13.33</td><td>0</td><td>2.33E+05</td><td></td><td>(K)TAVCDIF</td><td>(K)T/A|V|C D/I/P|P R(G)</td><td>C:Carbamidomethylation</td><td>105.85</td><td>514.7643</td><td>1028.519</td><td>0.002</td><td>1.9</td><td>5.5</td><td>50199.4</td><td>4.75</td><td>HUMAN</td><td>A6NNZ2</td><td>Tubulin beta-8 chain-like protein LOC260334</td></tr>
<tr><td>4777</td><td>dirMS_041712_NAR_30mM_plus2.18561.18561.2</td><td>2</td><td>13.33</td><td>0</td><td>6.99E+05</td><td></td><td>(K)EGMNIV</td><td>(K)E G M/N/I|V E/A/M E/R(F)</td><td></td><td>150.78</td><td>639.7958</td><td>1278.582</td><td>0.0026</td><td>2.1</td><td>4.25</td><td>18240.4</td><td>7.96</td><td>HUMAN</td><td>P62937</td><td>Peptidyl-prolyl cis-trans isomerase A</td></tr>
<tr><td>4778</td><td>dirMS_041712_NAR_30mM_plus2.8955.9005.2</td><td>2</td><td>13.33</td><td>0</td><td>3.45E+05</td><td></td><td>(K)GGEIQP'</td><td>(K)G G\E|I|Q|P V/S V/K(V)</td><td></td><td>100.77</td><td>507.2843</td><td>1013.563</td><td>-0.0013</td><td>-1.2</td><td>6</td><td>10803.5</td><td>9.82</td><td>HUMAN</td><td>B8ZZL8</td><td>10 kDa heat shock protein, mitochondrial</td></tr>
<tr><td>4779</td><td>dirMS_041712_NAR_40mM_plus2.13883.13883.3</td><td>3</td><td>13.33</td><td>0</td><td>2.23E+05</td><td></td><td>(K)VALLLLD</td><td>(K)V A L|L/L/L D Q/G A S P H A A A K(N)</td><td></td><td>148.13</td><td>558.9886</td><td>1674.954</td><td>-0.0025</td><td>-1.5</td><td>6.71</td><td>482683.6</td><td>6.07</td><td>HUMAN</td><td>Q12955</td><td>Ankyrin-3</td></tr>
<tr><td>4780</td><td>dirMS_041712_NAR_40mM_plus2.17566.17587.3</td><td>3</td><td>13.33</td><td>1</td><td>5.62E+05</td><td></td><td>(R)GLLPGLI</td><td>(R)G L|L\P\G L/L/P\A/P A D K L T/P K(G)</td><td></td><td>170.58</td><td>567.6811</td><td>1701.031</td><td>-0.0022</td><td>-1.3</td><td>8.59</td><td>153084.9</td><td>5.55</td><td>HUMAN</td><td>Q6P2E9</td><td>Enhancer of mRNA-decapping protein 4</td></tr>
<tr><td>4781</td><td>dirMS_041712_NAR_120mM_minus.15258.15258.3</td><td>3</td><td>13.32</td><td>2</td><td>9.57E+05</td><td></td><td>(R)ALKDEEI</td><td>(R)A L/K D E E/K/M/E|L/Q|E I\Q/L/K(E)</td><td></td><td>137.72</td><td>649.0139</td><td>1945.031</td><td>-0.0039</td><td>-2</td><td>4.58</td><td>33450.2</td><td>4.73</td><td>HUMAN</td><td>J3KN67</td><td>Tropomyosin alpha-3 chain</td></tr>
<tr><td>4782</td><td>dirMS_041712_NAR_30mM_minus2.14024.14024.2</td><td>2</td><td>13.32</td><td>0</td><td>2.71E+05</td><td></td><td>(R)IYVVDV(</td><td>(R)I Y|V|V|D V/G S E/P R(A)</td><td></td><td>129.55</td><td>617.3267</td><td>1233.647</td><td>-0.0013</td><td>-1</td><td>4.37</td><td>39066</td><td>6.61</td><td>HUMAN</td><td>A6PVW9</td><td>Selenium binding protein 1 (Fragment)</td></tr>
<tr><td>4783</td><td>dirMS_041712_NAR_30mM_minus2.6776.6776.2</td><td>2</td><td>13.32</td><td>0</td><td>2.56E+05</td><td></td><td>(R)IGDEDV(</td><td>(R)I/G|D E D/V/G/R(V)</td><td></td><td>91.67</td><td>430.7116</td><td>860.411</td><td>0.0051</td><td>5.9</td><td>4.03</td><td>23799.1</td><td>9.48</td><td>HUMAN</td><td>P23284</td><td>Peptidyl-prolyl cis-trans isomerase B</td></tr>
<tr><td>4784</td><td>dirMS_041712_NAR_60mM_minus.11395.11395.3</td><td>3</td><td>13.32</td><td>1</td><td>8.92E+05</td><td>M232m</td><td>(R)MSmKE\</td><td>(R)M/S/m K E V D E/Q|M/L/N\V/Q N K(N)</td><td>m:Oxidized methionine</td><td>122.82</td><td>647.3011</td><td>1923.897</td><td>15.9915</td><td>-1.8</td><td>4.68</td><td>40069.4</td><td>4.83</td><td>HUMAN</td><td>E7EWR1</td><td>Tubulin beta chain</td></tr>
<tr><td>4785</td><td>dirMS_041712_NAR_60mM_minus.4992.4992.3</td><td>3</td><td>13.32</td><td>1</td><td>1.11E+05</td><td></td><td>(K)DSYVGD</td><td>(K)D S/Y/V/G/D E/A Q S K R(G)</td><td></td><td>82.27</td><td>452.2129</td><td>1354.623</td><td>0.0008</td><td>0.6</td><td>4.56</td><td>32616.5</td><td>5.18</td><td>HUMAN</td><td>A6NL76</td><td>Actin, alpha skeletal muscle</td></tr>
<tr><td>4786</td><td>dirMS_041712_NAR_120mM_plus.9666.9720.4</td><td>4</td><td>13.32</td><td>2</td><td>3.92E+06</td><td></td><td>(R)VLTVINC</td><td>(R)V/L T/V/I/N Q T Q K/E/N L R K(F)</td><td></td><td>104.23</td><td>446.7653</td><td>1784.039</td><td>0.0005</td><td>0.3</td><td>9.99</td><td>10815.7</td><td>10.72</td><td>HUMAN</td><td>F2Z388</td><td>60S ribosomal protein L35</td></tr>
<tr><td>4787</td><td>dirMS_041712_NAR_20mM_plus.10922.10922.2</td><td>2</td><td>13.32</td><td>0</td><td>2.62E+04</td><td></td><td>(K)ALIAAQ\</td><td>(K)A L I/A/A/Q|Y S|G A Q/V R(V)</td><td></td><td>147.72</td><td>674.3695</td><td>1347.738</td><td>-0.0062</td><td>-4.6</td><td>8.79</td><td>56491.1</td><td>7.78</td><td>HUMAN</td><td>B4DTG2</td><td>Elongation factor 1-gamma</td></tr>
<tr><td>4788</td><td>dirMS_041712_NAR_20mM_plus.13278.13278.2</td><td>2</td><td>13.32</td><td>0</td><td>9.69E+04</td><td></td><td>(R)AELIVQF</td><td>(R)A E|L\I/V|Q/P/E L K(Y)</td><td></td><td>164.3</td><td>570.3361</td><td>1139.667</td><td>-0.0021</td><td>-1.9</td><td>4.53</td><td>334221.3</td><td>6.44</td><td>HUMAN</td><td>P15924</td><td>Desmoplakin</td></tr>
<tr><td>4789</td><td>dirMS_041712_NAR_30mM_plus2.22642.22642.3</td><td>3</td><td>13.32</td><td>0</td><td>5.00E+05</td><td></td><td>(R)ALTVPEI</td><td>(R)A L T\V/P E L/T Q Q/V/F D A K(N)</td><td></td><td>175.77</td><td>553.9697</td><td>1659.895</td><td>-0.0007</td><td>-0.4</td><td>4.37</td><td>40069.4</td><td>4.83</td><td>HUMAN</td><td>E7EWR1</td><td>Tubulin beta chain</td></tr>
<tr><td>4790</td><td>dirMS_041712_NAR_40mM_plus2.8979.8979.2</td><td>2</td><td>13.32</td><td>0</td><td>1.19E+05</td><td></td><td>(R)GYSFTT1</td><td>(R)G Y|S\F/T T/T A E/R(E)</td><td></td><td>114.9</td><td>566.7672</td><td>1132.527</td><td>0.0002</td><td>0.2</td><td>6</td><td>23901.9</td><td>5.28</td><td>HUMAN</td><td>I3L1U9</td><td>Actin, cytoplasmic 2, N-terminally processed (Fragment)</td></tr>
<tr><td>4791</td><td>dirMS_041712_NAR_50mM_plus2.9965.9965.3</td><td>3</td><td>13.32</td><td>0</td><td>1.16E+06</td><td></td><td>(K)INEKPQ\</td><td>(K)I/N/E/K/P Q V|I/A/D/Y E S G R(A)</td><td></td><td>119.77</td><td>573.6314</td><td>1718.871</td><td>0.0089</td><td>5.2</td><td>4.68</td><td>16368.4</td><td>9.95</td><td>HUMAN</td><td>O60869</td><td>Endothelial differentiation-related factor 1</td></tr>
</table>

<table>
<tr><th></th><th></th><th></th><th></th><th></th><th></th><th></th><th></th><th></th><th></th><th></th><th></th><th></th><th></th><th></th><th></th><th></th><th></th><th></th><th></th><th></th></tr>
<tr><td>4792</td><td>dirMS_041712_NAR_60mM_plus.19303.19303.3</td><td>3</td><td>13.32</td><td>1</td><td>1.96E+05</td><td></td><td>(R)AGEAGK</td><td>(R)A G/E\A|G K L E E\V\M/Q/E/L/R(A)</td><td></td><td>159.22</td><td>553.9525</td><td>1659.837</td><td>0.0059</td><td>3.6</td><td>4.49</td><td>54918.3</td><td>5.61</td><td>HUMAN</td><td>Q9BR76</td><td>Coronin-1B</td></tr>
<tr><td>4793</td><td>dirMS_041712_NAR_60mM_plus.7536.7672.3</td><td>3</td><td>13.32</td><td>0</td><td>1.38E+06</td><td>M80m</td><td>(R)TICSHVC</td><td>(R)T/I|C/S/H/V/Q N m I K(G)</td><td>C:Carbamidomethylation m:Oxid</td><td>97.92</td><td>449.5594</td><td>1330.661</td><td>16.003</td><td>6</td><td>8.44</td><td>20931.1</td><td>10.28</td><td>HUMAN</td><td>D6RAN4</td><td>60S ribosomal protein L9 (Fragment)</td></tr>
<tr><td>4794</td><td>dirMS_041712_NAR_20mM_minus.12556.12556.3</td><td>3</td><td>13.31</td><td>0</td><td>5.42E+05</td><td>M73m</td><td>(R)AVLVDLI</td><td>(R)A V\L/V D\L/E|P G T\m D S/V R(S)</td><td>m:Oxidized methionine</td><td>173.05</td><td>539.9423</td><td>1601.82</td><td>15.992</td><td>-1.8</td><td>4.03</td><td>13112.4</td><td>5.23</td><td>HUMAN</td><td>F8W813</td><td>Tubulin beta-8 chain</td></tr>
<tr><td>4795</td><td>dirMS_041712_NAR_20mM_minus.14710.14710.2</td><td>2</td><td>13.31</td><td>0</td><td>1.74E+05</td><td></td><td>(K)TAVETA</td><td>(K)T A|V/E/T/A V L|L L R(I)</td><td></td><td>201.37</td><td>593.3613</td><td>1185.72</td><td>-0.0048</td><td>-4.1</td><td>5.66</td><td>56186.7</td><td>5.47</td><td>HUMAN</td><td>B4DUR8</td><td>T-complex protein 1 subunit gamma</td></tr>
<tr><td>4796</td><td>dirMS_041712_NAR_30mM_minus2.10714.11210.2</td><td>2</td><td>13.31</td><td>0</td><td>9.63E+05</td><td></td><td>(K)SSFLVDC</td><td>(K)S S|F|L|V/D/C/S K(A)</td><td>C:Carbamidomethylation</td><td>113.15</td><td>521.7502</td><td>1042.487</td><td>0.0058</td><td>5.5</td><td>5.55</td><td>258215.9</td><td>5.48</td><td>HUMAN</td><td>E7EN95</td><td>Filamin-B</td></tr>
<tr><td>4797</td><td>dirMS_041712_NAR_30mM_minus2.16078.16078.2</td><td>2</td><td>13.31</td><td>0</td><td>2.17E+04</td><td></td><td>(K)VNYILES</td><td>(K)V N/Y I/L E/S/R(A)</td><td></td><td>140.85</td><td>497.265</td><td>993.536</td><td>-0.0136</td><td>-13.7</td><td>5.97</td><td>26855.2</td><td>9.54</td><td>HUMAN</td><td>B7Z5E5</td><td>Target of rapamycin complex 2 subunit MAPKAP1</td></tr>
<tr><td>4798</td><td>dirMS_041712_NAR_20mM_plus.18879.18879.2</td><td>2</td><td>13.31</td><td>0</td><td>9.89E+04</td><td></td><td>(K)LTVYTTL</td><td>(K)L T\V|Y/T T L/I D V T/K(G)</td><td></td><td>200.9</td><td>683.8948</td><td>1366.783</td><td>-0.0005</td><td>-0.4</td><td>5.83</td><td>42194.9</td><td>8.75</td><td>HUMAN</td><td>E7EMM4</td><td>Acid ceramidase</td></tr>
<tr><td>4799</td><td>dirMS_041712_NAR_50mM_plus2.15410.15410.3</td><td>3</td><td>13.31</td><td>0</td><td>1.43E+06</td><td></td><td>(R)LASTLVH</td><td>(R)L A|S T L V|H L G E Y Q A/A/V D G A R(K)</td><td></td><td>149.62</td><td>657.6802</td><td>1971.029</td><td>-0.0034</td><td>-1.7</td><td>5.32</td><td>71165.3</td><td>5.57</td><td>HUMAN</td><td>J3KS13</td><td>Clathrin heavy chain 1</td></tr>
<tr><td>4800</td><td>dirMS_041712_NAR_50mM_plus2.5316.5316.3</td><td>3</td><td>13.31</td><td>0</td><td>3.81E+05</td><td>M116m</td><td>(R)ATAPQT</td><td>(R)A/T/A/P Q/T Q/H V S/P m R(Q)</td><td>m:Oxidized methionine</td><td>87.6</td><td>480.5727</td><td>1423.711</td><td>15.9925</td><td>-1.7</td><td>9.8</td><td>29184.1</td><td>4.91</td><td>HUMAN</td><td>E9PBQ9</td><td>Elongation factor 1-delta</td></tr>
<tr><td>4801</td><td>dirMS_041712_NAR_50mM_plus2.7488.7488.3</td><td>3</td><td>13.31</td><td>1</td><td>3.52E+05</td><td></td><td>(R)VVASDK</td><td>(R)V/V/A/S D K E/T Y E L/R(Y)</td><td></td><td>105.87</td><td>470.5836</td><td>1409.727</td><td>0.0092</td><td>6.5</td><td>4.68</td><td>14478.1</td><td>9.37</td><td>HUMAN</td><td>C9JYQ9</td><td>60S ribosomal protein L22-like 1</td></tr>
<tr><td>4802</td><td>dirMS_041712_NAR_60mM_plus.18287.18287.3</td><td>3</td><td>13.31</td><td>1</td><td>9.14E+05</td><td></td><td>(K)EVNKFQ</td><td>(K)E V/N K F Q M A/Y/S N/L L R(A)</td><td></td><td>152.85</td><td>571.6293</td><td>1712.879</td><td>-0.0055</td><td>-3.2</td><td>8.69</td><td>14683.8</td><td>10.19</td><td>HUMAN</td><td>P37108</td><td>Signal recognition particle 14 kDa protein</td></tr>
<tr><td>4803</td><td>dirMS_041712_NAR_30mM_minus2.19195.19195.2</td><td>2</td><td>13.3</td><td>0</td><td>1.03E+05</td><td></td><td>(K)TVALWD</td><td>(K)T/V/A\L|W/D/L R(N)</td><td></td><td>159.23</td><td>487.2765</td><td>973.547</td><td>-0.0008</td><td>-0.8</td><td>5.5</td><td>19598</td><td>4.45</td><td>HUMAN</td><td>B4DRT0</td><td>Histone-binding protein RBBP4</td></tr>
<tr><td>4804</td><td>dirMS_041712_NAR_50mM_minus2.12500.13012.3</td><td>3</td><td>13.3</td><td>1</td><td>7.56E+06</td><td></td><td>(R)IQLVEEE</td><td>(R)I/Q|L|V/E E E L D R A Q E/R(L)</td><td></td><td>140.42</td><td>576.6348</td><td>1727.892</td><td>-0.0024</td><td>-1.4</td><td>4.25</td><td>31923.6</td><td>4.85</td><td>HUMAN</td><td>B7Z596</td><td>Tropomyosin alpha-1 chain</td></tr>
<tr><td>4805</td><td>dirMS_041712_NAR_60mM_minus.14322.14460.3</td><td>3</td><td>13.3</td><td>1</td><td>3.35E+05</td><td></td><td>(K)KSDIDEI</td><td>(K)K S D I\D E\I|V\L|V/G/G S T R(I)</td><td></td><td>139.68</td><td>530.2846</td><td>1588.854</td><td>-0.0148</td><td>-9.3</td><td>4.56</td><td>72445.7</td><td>5.07</td><td>HUMAN</td><td>P11021</td><td>78 kDa glucose-regulated protein</td></tr>
<tr><td>4806</td><td>dirMS_041712_NAR_60mM_minus.16973.16993.3</td><td>3</td><td>13.3</td><td>1</td><td>2.04E+06</td><td></td><td>(K)LLQDFFN</td><td>(K)L/L|Q/D/F/F/N/G K E L N K(S)</td><td></td><td>155.22</td><td>522.6138</td><td>1565.832</td><td>-0.0054</td><td>-3.4</td><td>6.07</td><td>45508.6</td><td>5.5</td><td>HUMAN</td><td>A8K7Q2</td><td>Heat shock cognate 71 kDa protein</td></tr>
<tr><td>4807</td><td>dirMS_041712_NAR_60mM_minus.5365.5365.3</td><td>3</td><td>13.3</td><td>0</td><td>2.19E+04</td><td></td><td>(R)ESPGAA</td><td>(R)E S/P G A/A A T/S/S S G P Q A Q Q H/R(G)</td><td></td><td>85.48</td><td>622.9613</td><td>1866.869</td><td>0.0005</td><td>0.2</td><td>6.85</td><td>74981.5</td><td>9.96</td><td>HUMAN</td><td>Q7L2J0</td><td>7SK snRNA methylphosphate capping enzyme</td></tr>
<tr><td>4808</td><td>dirMS_041712_NAR_120mM_plus.23757.23757.4</td><td>4</td><td>13.3</td><td>2</td><td>2.20E+05</td><td></td><td>(R)RGVML</td><td>(R)R G V\M|L|A V/D A V/I A E L K K(Q)</td><td></td><td>179.67</td><td>429.0075</td><td>1713.009</td><td>-0.001</td><td>-0.6</td><td>8.59</td><td>25225.7</td><td>9.52</td><td>HUMAN</td><td>E7ESH4</td><td>60 kDa heat shock protein, mitochondrial (Fragment)</td></tr>
<tr><td>4809</td><td>dirMS_041712_NAR_30mM_plus2.11785.11785.2</td><td>2</td><td>13.3</td><td>0</td><td>5.40E+05</td><td></td><td>(K)VASGCLI</td><td>(K)V/A|S/G/C/L|D I/N S S V K(G)</td><td>C:Carbamidomethylation</td><td>114.82</td><td>675.3393</td><td>1349.673</td><td>-0.0016</td><td>-1.2</td><td>5.81</td><td>56837</td><td>7.19</td><td>HUMAN</td><td>E7ETT1</td><td>Propionyl-CoA carboxylase beta chain, mitochondrial</td></tr>
<tr><td>4810</td><td>dirMS_041712_NAR_30mM_plus2.20771.20771.2</td><td>2</td><td>13.3</td><td>0</td><td>3.91E+05</td><td></td><td>(R)SLVPAAI</td><td>(R)S L\V|P A/A/E\L/L E S R(V)</td><td></td><td>162.33</td><td>642.8602</td><td>1284.716</td><td>-0.0027</td><td>-2.1</td><td>4.53</td><td>533778</td><td>5.74</td><td>HUMAN</td><td>Q15149</td><td>Plectin</td></tr>
<tr><td>4811</td><td>dirMS_041712_NAR_30mM_plus2.7560.7577.2</td><td>2</td><td>13.3</td><td>0</td><td>7.76E+05</td><td></td><td>(K)CCTESLV</td><td>(K)C|C|T|E/S/L/V/N R(R)</td><td>C:Carbamidomethylation</td><td>93.95</td><td>569.7533</td><td>1138.498</td><td>0.0014</td><td>1.2</td><td>6</td><td>57751.3</td><td>6.9</td><td>HUMAN</td><td>B7WNR0</td><td>Serum albumin</td></tr>
<tr><td>4812</td><td>dirMS_041712_NAR_40mM_plus2.10416.10416.2</td><td>2</td><td>13.3</td><td>0</td><td>9.40E+04</td><td></td><td>(K)IGGIGTV</td><td>(K)I/G|G|I/G T V/P V/G R(V)</td><td></td><td>125.75</td><td>513.3081</td><td>1025.61</td><td>-0.0013</td><td>-1.2</td><td>9.75</td><td>50482.3</td><td>9.31</td><td>HUMAN</td><td>P68104</td><td>Elongation factor 1-alpha 1</td></tr>
<tr><td>4813</td><td>dirMS_041712_NAR_40mM_plus2.9428.9428.2</td><td>2</td><td>13.3</td><td>0</td><td>9.68E+04</td><td></td><td>(R)ISVYYNE</td><td>(R)I/S/V/Y/Y N\E A T G G K(Y)</td><td></td><td>118.55</td><td>651.3206</td><td>1301.637</td><td>-0.0033</td><td>-2.5</td><td>6</td><td>27549.9</td><td>4.39</td><td>HUMAN</td><td>E9PBJ4</td><td>Tubulin beta chain</td></tr>
<tr><td>4814</td><td>dirMS_041712_NAR_60mM_plus.5300.5310.3</td><td>3</td><td>13.3</td><td>1</td><td>1.42E+05</td><td>M117m</td><td>(K)EmQLRC</td><td>(K)E m/Q/L/R/Q/E/E/E/R(R)</td><td>m:Oxidized methionine</td><td>85.4</td><td>455.2157</td><td>1347.632</td><td>16.0004</td><td>4</td><td>4.49</td><td>26419.2</td><td>5.1</td><td>HUMAN</td><td>H0Y9K7</td><td>Splicing factor, proline- and glutamine-rich (Fragment)</td></tr>
<tr><td>4815</td><td>dirMS_041712_NAR_30mM_minus2.16330.16330.2</td><td>2</td><td>13.29</td><td>0</td><td>1.45E+05</td><td></td><td>(R)SLVSGLI</td><td>(R)S L|V/S\G/L I/R(L)</td><td></td><td>142.08</td><td>422.7649</td><td>844.525</td><td>-0.0026</td><td>-3</td><td>9.47</td><td>295149.3</td><td>7.4</td><td>HUMAN</td><td>Q92616</td><td>Translational activator GCN1</td></tr>
<tr><td>4816</td><td>dirMS_041712_NAR_40mM_minus2.9624.9624.2</td><td>2</td><td>13.29</td><td>0</td><td>1.24E+06</td><td></td><td>(R)VAPEEH</td><td>(R)V A/P E E/H|P V L/L T E A P L/N/P K(A)</td><td></td><td>139.8</td><td>977.5343</td><td>1954.064</td><td>-0.0031</td><td>-1.6</td><td>4.75</td><td>18032.2</td><td>5.25</td><td>HUMAN</td><td>E7EVS6</td><td>Actin, cytoplasmic 1 (Fragment)</td></tr>
<tr><td>4817</td><td>dirMS_041712_NAR_50mM_minus2.14940.14940.3</td><td>3</td><td>13.29</td><td>0</td><td>4.19E+05</td><td></td><td>(K)IAILTCPF</td><td>(K)I/A|I/L T/C/P F E P P K P K(T)</td><td>C:Carbamidomethylation</td><td>153.32</td><td>537.6375</td><td>1610.898</td><td>0.0005</td><td>0.3</td><td>8.59</td><td>49982</td><td>5.86</td><td>HUMAN</td><td>B4DYD8</td><td>T-complex protein 1 subunit epsilon</td></tr>
<tr><td>4818</td><td>dirMS_041712_NAR_60mM_minus.14259.14259.3</td><td>3</td><td>13.29</td><td>1</td><td>8.82E+05</td><td></td><td>(R)MSMKE</td><td>(R)M/S M K E V D E Q|M|L/N V Q/N K(N)</td><td></td><td>140.17</td><td>641.9701</td><td>1923.897</td><td>-0.0015</td><td>-0.8</td><td>4.68</td><td>40069.4</td><td>4.83</td><td>HUMAN</td><td>E7EWR1</td><td>Tubulin beta chain</td></tr>
<tr><td>4819</td><td>dirMS_041712_NAR_120mM_plus.11424.11506.4</td><td>4</td><td>13.29</td><td>2</td><td>2.22E+06</td><td></td><td>(K)QAGPAS</td><td>(K)Q A/G P A/S\V/P/L R T E E E F K/K(F)</td><td></td><td>113.4</td><td>472.5046</td><td>1886.997</td><td>-0.0005</td><td>-0.3</td><td>6.23</td><td>55361.8</td><td>6.42</td><td>HUMAN</td><td>G5EA52</td><td>Protein disulfide isomerase family A, member 3, isoform CRA_b</td></tr>
<tr><td>4820</td><td>dirMS_041712_NAR_20mM_plus.15480.15480.2</td><td>2</td><td>13.29</td><td>0</td><td>4.07E+05</td><td></td><td>(R)GVVDSE</td><td>(R)G V V|D/S\E|D\I|P L\N/L/S R(E)</td><td></td><td>179.15</td><td>757.3956</td><td>1513.786</td><td>-0.0017</td><td>-1.1</td><td>4.03</td><td>74494.3</td><td>7.79</td><td>HUMAN</td><td>F5H897</td><td>Heat shock protein 75 kDa, mitochondrial</td></tr>
<tr><td>4821</td><td>dirMS_041712_NAR_30mM_plus2.13529.13529.2</td><td>2</td><td>13.29</td><td>0</td><td>1.10E+06</td><td></td><td>(R)ANTFVA</td><td>(R)A N/T|F/V/A E/L/K(G)</td><td></td><td>124.65</td><td>496.774</td><td>992.541</td><td>-0.0004</td><td>-0.4</td><td>6.05</td><td>24936.4</td><td>8.67</td><td>HUMAN</td><td>G3XAL0</td><td>Malate dehydrogenase</td></tr>
<tr><td>4822</td><td>dirMS_041712_NAR_30mM_plus2.18971.19221.2</td><td>2</td><td>13.29</td><td>0</td><td>2.07E+05</td><td></td><td>(K)YPQLLP(</td><td>(K)Y/P|Q/L|L/P G I/R(G)</td><td></td><td>153.25</td><td>528.814</td><td>1056.62</td><td>0.0007</td><td>0.6</td><td>8.75</td><td>45228.3</td><td>6.03</td><td>HUMAN</td><td>F5H737</td><td>Adenosylhomocysteinase</td></tr>
<tr><td>4823</td><td>dirMS_041712_NAR_30mM_plus2.22113.22113.2</td><td>2</td><td>13.29</td><td>0</td><td>2.01E+05</td><td></td><td>(K)NSSYFVE</td><td>(K)N S S/Y F\V/E/W/I/P N N V K(V)</td><td></td><td>171.82</td><td>848.9189</td><td>1696.833</td><td>-0.0024</td><td>-1.4</td><td>6</td><td>42831.5</td><td>4.86</td><td>HUMAN</td><td>A8K854</td><td>HCG1983504, isoform CRA_f</td></tr>
<tr><td>4824</td><td>dirMS_041712_NAR_50mM_plus2.11426.11426.3</td><td>3</td><td>13.29</td><td>1</td><td>3.29E+05</td><td></td><td>(K)VAVVAG</td><td>(K)V A|V|V|A|G/Y G D V G K G C A Q A L R(G)</td><td>C:Carbamidomethylation</td><td>127.85</td><td>630.9984</td><td>1890.985</td><td>-0.0048</td><td>-2.5</td><td>8.56</td><td>45228.3</td><td>6.03</td><td>HUMAN</td><td>F5H737</td><td>Adenosylhomocysteinase</td></tr>
<tr><td>4825</td><td>dirMS_041712_NAR_50mM_plus2.5256.5256.3</td><td>3</td><td>13.29</td><td>0</td><td>1.37E+06</td><td></td><td>(K)TATPQQ</td><td>(K)T A|T/P Q Q/A Q E V/H E K(L)</td><td></td><td>86.38</td><td>489.5774</td><td>1466.723</td><td>-0.0057</td><td>-3.9</td><td>5.37</td><td>31075.7</td><td>5.65</td><td>HUMAN</td><td>P60174</td><td>Triosephosphate isomerase</td></tr>
<tr><td>4826</td><td>dirMS_041712_NAR_60mM_plus.20624.20647.3</td><td>3</td><td>13.29</td><td>0</td><td>7.27E+05</td><td></td><td>(R)LGFHLPI</td><td>(R)L G F H L\P L|E|V/A/Y/Q/R(G)</td><td></td><td>168.62</td><td>514.9568</td><td>1542.843</td><td>0.0131</td><td>8.5</td><td>6.75</td><td>533778</td><td>5.74</td><td>HUMAN</td><td>Q15149</td><td>Plectin</td></tr>
<tr><td>4827</td><td>dirMS_041712_NAR_40mM_minus2.6164.6234.2</td><td>2</td><td>13.28</td><td>0</td><td>9.42E+05</td><td></td><td>(R)STFSTNY</td><td>(R)S T|F|S/T/N/Y R(S)</td><td></td><td>101.98</td><td>488.2302</td><td>975.453</td><td>0.0001</td><td>0.1</td><td>8.46</td><td>43773.3</td><td>5.26</td><td>HUMAN</td><td>F8VZY9</td><td>Keratin, type I cytoskeletal 18</td></tr>
<tr><td>4828</td><td>dirMS_041712_NAR_60mM_minus.10872.11021.3</td><td>3</td><td>13.28</td><td>1</td><td>4.59E+05</td><td></td><td>(K)GSITISAI</td><td>(K)G S|I|T/I/S A E E/I K D/N R(V)</td><td></td><td>121.13</td><td>511.6045</td><td>1532.792</td><td>0.0075</td><td>4.9</td><td>4.68</td><td>27142.4</td><td>5.34</td><td>HUMAN</td><td>E5RG68</td><td>Copine-3 (Fragment)</td></tr>
<tr><td>4829</td><td>dirMS_041712_NAR_120mM_plus.18392.18392.4</td><td>4</td><td>13.28</td><td>0</td><td>4.29E+05</td><td></td><td>(R)QALHSLI</td><td>(R)Q A L H S L/E L H Y Q/A/F/L/R(D)</td><td></td><td>149.4</td><td>457.2493</td><td>1825.971</td><td>0.0046</td><td>2.5</td><td>6.92</td><td>533778</td><td>5.74</td><td>HUMAN</td><td>Q15149</td><td>Plectin</td></tr>
<tr><td>4830</td><td>dirMS_041712_NAR_20mM_plus.16939.16939.3</td><td>3</td><td>13.28</td><td>0</td><td>3.07E+05</td><td></td><td>(K)VQIAVA</td><td>(K)V Q I A V/A\N A|Q/E|L|L Q R(M)</td><td></td><td>187.85</td><td>518.2975</td><td>1552.881</td><td>-0.0026</td><td>-1.7</td><td>5.97</td><td>10728.1</td><td>9.15</td><td>HUMAN</td><td>Q9Y5L4</td><td>Mitochondrial import inner membrane translocase subunit Tim13</td></tr>
<tr><td>4831</td><td>dirMS_041712_NAR_30mM_plus2.18927.18927.2</td><td>2</td><td>13.28</td><td>0</td><td>1.87E+05</td><td></td><td>(K)LNLGTV(</td><td>(K)L/N|L|G T/V/G F Y R(T)</td><td></td><td>152.8</td><td>570.3123</td><td>1139.621</td><td>-0.0034</td><td>-3</td><td>8.75</td><td>95141.7</td><td>5.22</td><td>HUMAN</td><td>B7Z463</td><td>Puromycin-sensitive aminopeptidase</td></tr>
<tr><td>4832</td><td>dirMS_041712_NAR_30mM_plus2.20780.20780.3</td><td>3</td><td>13.28</td><td>0</td><td>1.06E+06</td><td></td><td>(R)TVQSLEI</td><td>(R)T V Q\S L E|I/D|L/D S M R(N)</td><td></td><td>162.32</td><td>502.9201</td><td>1506.747</td><td>-0.0011</td><td>-0.7</td><td>4.03</td><td>43773.3</td><td>5.26</td><td>HUMAN</td><td>F8VZY9</td><td>Keratin, type I cytoskeletal 18</td></tr>
<tr><td>4833</td><td>dirMS_041712_NAR_30mM_plus2.6709.6709.2</td><td>2</td><td>13.28</td><td>0</td><td>5.75E+04</td><td></td><td>(K)TVGVEP.</td><td>(K)T/V|G|V\E/P A/A D G K(G)</td><td></td><td>89.45</td><td>522.2782</td><td>1043.537</td><td>0.0124</td><td>11.8</td><td>4.37</td><td>7945.1</td><td>11.03</td><td>HUMAN</td><td>C9JB50</td><td>60S ribosomal protein L28</td></tr>
<tr><td>4834</td><td>dirMS_041712_NAR_40mM_plus2.6857.6857.2</td><td>2</td><td>13.28</td><td>0</td><td>5.56E+03</td><td></td><td>(R)LSVAAQ</td><td>(R)L/S|V/A|A/Q/E A A R(L)</td><td></td><td>100.35</td><td>508.2775</td><td>1015.553</td><td>-0.0054</td><td>-5.3</td><td>6</td><td>533778</td><td>5.74</td><td>HUMAN</td><td>Q15149</td><td>Plectin</td></tr>
<tr><td>4835</td><td>dirMS_041712_NAR_50mM_plus2.10871.10871.3</td><td>3</td><td>13.28</td><td>0</td><td>4.89E+05</td><td></td><td>(R)EALLSSA</td><td>(R)E A/L/L/S/S A|V D H G S D E V K(F)</td><td></td><td>124.7</td><td>552.9403</td><td>1656.807</td><td>-0.0012</td><td>-0.7</td><td>4.31</td><td>57204.9</td><td>6.01</td><td>HUMAN</td><td>F5GWF6</td><td>T-complex protein 1 subunit beta</td></tr>
<tr><td>4836</td><td>dirMS_041712_NAR_60mM_plus.14426.14426.2</td><td>2</td><td>13.28</td><td>1</td><td>8.40E+05</td><td></td><td>(K)KVPQVS</td><td>(K)K|V/P Q V/S T/P T\L V E V S R(N)</td><td></td><td>132.12</td><td>820.4708</td><td>1639.938</td><td>-0.0034</td><td>-2.1</td><td>8.75</td><td>57751.3</td><td>6.9</td><td>HUMAN</td><td>B7WNR0</td><td>Serum albumin</td></tr>
<tr><td>4837</td><td>dirMS_041712_NAR_60mM_plus.15564.15589.3</td><td>3</td><td>13.28</td><td>1</td><td>2.59E+05</td><td></td><td>(K)ALELDSN</td><td>(K)A L|E|L/D/S/N N E/K G L/F R(R)</td><td></td><td>138.02</td><td>535.9438</td><td>1605.823</td><td>-0.0063</td><td>-3.9</td><td>4.68</td><td>52088.9</td><td>5.35</td><td>HUMAN</td><td>Q02790</td><td>Peptidyl-prolyl cis-trans isomerase FKBP4</td></tr>
<tr><td>4838</td><td>dirMS_041712_NAR_60mM_plus.7690.7704.3</td><td>3</td><td>13.28</td><td>0</td><td>9.11E+05</td><td></td><td>(K)LFQECCI</td><td>(K)L/F|Q/E C/C/P H S T D R(V)</td><td>C:Carbamidomethylation</td><td>98.37</td><td>517.2234</td><td>1549.652</td><td>0.0034</td><td>2.2</td><td>5.32</td><td>51260.6</td><td>5.39</td><td>HUMAN</td><td>P61978</td><td>Heterogeneous nuclear ribonucleoprotein K</td></tr>
<tr><td>4839</td><td>dirMS_041712_NAR_20mM_minus.6903.6903.2</td><td>2</td><td>13.27</td><td>0</td><td>1.34E+05</td><td>M453m</td><td>(K)ImATPE(</td><td>(K)I m|A|T/P E Q V|G K(M)</td><td>m:Oxidized methionine</td><td>115.42</td><td>545.2807</td><td>1073.566</td><td>15.9882</td><td>-6.2</td><td>6</td><td>64302.5</td><td>6.4</td><td>HUMAN</td><td>B1AHC7</td><td>X-ray repair complementing defective repair in Chinese hamster cells 6 (Ku autoantigen, 70kDa)</td></tr>
<tr><td>4840</td><td>dirMS_041712_NAR_30mM_minus2.9963.10045.2</td><td>2</td><td>13.27</td><td>0</td><td>8.46E+05</td><td></td><td>(K)IAGPGL(</td><td>(K)I/A|G|P/G L G S G V/R(A)</td><td></td><td>108.65</td><td>492.2788</td><td>983.563</td><td>-0.0129</td><td>-13.1</td><td>9.75</td><td>258215.9</td><td>5.48</td><td>HUMAN</td><td>E7EN95</td><td>Filamin-B</td></tr>
<tr><td>4841</td><td>dirMS_041712_NAR_20mM_plus.18759.18759.2</td><td>2</td><td>13.27</td><td>0</td><td>2.00E+05</td><td></td><td>(R)VFIGNLN</td><td>(R)V/F|I|G N/L N T L V/V/K(K)</td><td></td><td>200.13</td><td>658.8999</td><td>1316.794</td><td>-0.0011</td><td>-0.9</td><td>8.72</td><td>15791.7</td><td>9.22</td><td>HUMAN</td><td>B4DSU6</td><td>Heterogeneous nuclear ribonucleoproteins C1/C2</td></tr>
<tr><td>4842</td><td>dirMS_041712_NAR_20mM_plus.6401.6405.2</td><td>2</td><td>13.27</td><td>0</td><td>1.60E+05</td><td></td><td>(K)DSYVGD</td><td>(K)D S/Y|V|G D/E/A Q/S/K(R)</td><td></td><td>107.17</td><td>599.7645</td><td>1198.522</td><td>-0.0005</td><td>-0.4</td><td>4.03</td><td>32616.5</td><td>5.18</td><td>HUMAN</td><td>A6NL76</td><td>Actin, alpha skeletal muscle</td></tr>
<tr><td>4843</td><td>dirMS_041712_NAR_30mM_plus2.11426.11426.2</td><td>2</td><td>13.27</td><td>0</td><td>4.41E+05</td><td></td><td>(R)FASGGC</td><td>(R)F A|S/G/G C D N L/I/K(L)</td><td>C:Carbamidomethylation</td><td>113.55</td><td>591.286</td><td>1181.562</td><td>0.0028</td><td>2.4</td><td>5.84</td><td>34524.7</td><td>5.4</td><td>HUMAN</td><td>A8MV37</td><td>Protein SEC13 homolog</td></tr>
<tr><td>4844</td><td>dirMS_041712_NAR_30mM_plus2.22327.22327.2</td><td>2</td><td>13.27</td><td>0</td><td>3.79E+05</td><td></td><td>(R)VGNLGL</td><td>(R)V G N|L/G\L|A\T/S F F\N/E R(N)</td><td></td><td>172.8</td><td>762.8926</td><td>1524.781</td><td>-0.0026</td><td>-1.7</td><td>5.97</td><td>73641.5</td><td>6.74</td><td>HUMAN</td><td>O00571</td><td>ATP-dependent RNA helicase DDX3X</td></tr>
<tr><td>4845</td><td>dirMS_041712_NAR_40mM_plus2.19242.19279.3</td><td>3</td><td>13.27</td><td>1</td><td>4.28E+05</td><td></td><td>(K)LLPLVSD</td><td>(K)L L\P/L/V/S D E/V/F I/R D/V/A K(V)</td><td></td><td>180.72</td><td>605.3503</td><td>1814.042</td><td>-0.0059</td><td>-3.2</td><td>4.56</td><td>97378.8</td><td>6.4</td><td>HUMAN</td><td>P11216</td><td>Glycogen phosphorylase, brain form</td></tr>
<tr><td>4846</td><td>dirMS_041712_NAR_50mM_plus2.12640.12640.3</td><td>3</td><td>13.27</td><td>0</td><td>5.15E+05</td><td></td><td>(R)IMDPNI</td><td>(R)I/M/D/P N/I V/G S E H/Y D/V A R(G)</td><td></td><td>134.6</td><td>605.9694</td><td>1815.869</td><td>0.0242</td><td>13.3</td><td>4.54</td><td>55307.5</td><td>5.25</td><td>HUMAN</td><td>F8VPV9</td><td>ATP synthase subunit beta</td></tr>
<tr><td>4847</td><td>dirMS_041712_NAR_50mM_plus2.16580.16580.3</td><td>3</td><td>13.27</td><td>1</td><td>2.04E+05</td><td></td><td>(R)LDDFFK</td><td>(R)L/D/D/F F K/V T G S L S S A K(R)</td><td></td><td>155.03</td><td>538.95</td><td>1614.837</td><td>-0.0019</td><td>-1.2</td><td>5.96</td><td>42934.5</td><td>9.09</td><td>HUMAN</td><td>P39748</td><td>Flap endonuclease 1</td></tr>
<tr><td>4848</td><td>dirMS_041712_NAR_50mM_plus2.18216.18216.2</td><td>2</td><td>13.27</td><td>0</td><td>5.20E+05</td><td></td><td>(R)IHFPLAT</td><td>(R)I/H|F|P L/A/T\Y A P V I/S A/E K(A)</td><td></td><td>164.6</td><td>878.9842</td><td>1756.963</td><td>-0.0021</td><td>-1.2</td><td>6.75</td><td>49012.3</td><td>4.9</td><td>HUMAN</td><td>A8MUB1</td><td>Tubulin alpha-4A chain</td></tr>
<tr><td>4849</td><td>dirMS_041712_NAR_60mM_plus.5211.5211.3</td><td>3</td><td>13.27</td><td>1</td><td>4.08E+05</td><td></td><td>(K)VSSAEG</td><td>(K)V/S/S/A/E G A\A K E E P K(R)</td><td></td><td>85.05</td><td>434.8867</td><td>1302.654</td><td>-0.008</td><td>-6.2</td><td>4.79</td><td>4801.6</td><td>11.17</td><td>HUMAN</td><td>F2Z2W6</td><td>Non-histone chromosomal protein HMG-14</td></tr>
<tr><td>4850</td><td>dirMS_041712_NAR_30mM_minus2.9347.9357.2</td><td>2</td><td>13.26</td><td>0</td><td>1.01E+06</td><td></td><td>(K)QADVNI</td><td>(K)Q/A|D\V/N/L|V/N/A K(L)</td><td></td><td>105.43</td><td>536.2917</td><td>1071.579</td><td>-0.0032</td><td>-3</td><td>5.84</td><td>57391.2</td><td>6.29</td><td>HUMAN</td><td>O43175</td><td>D-3-phosphoglycerate dehydrogenase</td></tr>
<tr><td>4851</td><td>dirMS_041712_NAR_40mM_minus2.11805.11887.3</td><td>3</td><td>13.26</td><td>0</td><td>1.02E+04</td><td></td><td>(K)LVEALC</td><td>(K)L/V|E/A/L|C A/E H Q I N L I K(V)</td><td>C:Carbamidomethylation</td><td>152.93</td><td>584.3199</td><td>1750.952</td><td>-0.0069</td><td>-3.9</td><td>5.4</td><td>14914.1</td><td>6.84</td><td>HUMAN</td><td>P25398</td><td>40S ribosomal protein S12</td></tr>
<tr><td>4852</td><td>dirMS_041712_NAR_50mM_minus2.13286.13286.3</td><td>3</td><td>13.26</td><td>1</td><td>2.02E+05</td><td>M17m</td><td>(K)GAEEmE</td><td>(K)G A|E/E/m/E T\V I P V D V M R R(A)</td><td>m:Oxidized methionine</td><td>143.75</td><td>616.6435</td><td>1831.904</td><td>16.0119</td><td>9.2</td><td>4.41</td><td>18080.7</td><td>7.92</td><td>HUMAN</td><td>K7ELW0</td><td>Protein DJ-1</td></tr>
<tr><td>4853</td><td>dirMS_041712_NAR_20mM_plus.15773.15773.2</td><td>2</td><td>13.26</td><td>0</td><td>4.76E+05</td><td></td><td>(R)GVVDSE</td><td>(R)G V V\D|S E/D/I|P L/N L S R(E)</td><td></td><td>179.47</td><td>757.3956</td><td>1513.786</td><td>-0.0017</td><td>-1.1</td><td>4.03</td><td>74494.3</td><td>7.79</td><td>HUMAN</td><td>F5H897</td><td>Heat shock protein 75 kDa, mitochondrial</td></tr>
<tr><td>4854</td><td>dirMS_041712_NAR_30mM_plus2.12569.12569.2</td><td>2</td><td>13.26</td><td>0</td><td>1.85E+05</td><td></td><td>(R)ELAEDG</td><td>(R)E L/A/E/D/G Y/S G V E/V R(V)</td><td></td><td>118.53</td><td>712.3382</td><td>1423.67</td><td>-0.0008</td><td>-0.6</td><td>4</td><td>13054.7</td><td>9.59</td><td>HUMAN</td><td>E9PJH4</td><td>40S ribosomal protein S3</td></tr>
<tr><td>4855</td><td>dirMS_041712_NAR_30mM_plus2.20691.20691.2</td><td>2</td><td>13.26</td><td>0</td><td>2.60E+05</td><td></td><td>(R)ASLSLW</td><td>(R)A S/L/S/L/W/Q\A\M/E K(G)</td><td></td><td>162.9</td><td>632.3231</td><td>1263.64</td><td>-0.0013</td><td>-1</td><td>6.05</td><td>266161</td><td>5.68</td><td>HUMAN</td><td>E9PPU0</td><td>Epiplakin</td></tr>
<tr><td>4856</td><td>dirMS_041712_NAR_50mM_plus2.20018.20018.3</td><td>3</td><td>13.26</td><td>1</td><td>1.05E+04</td><td></td><td>(K)QNDQV</td><td>(K)Q N D\Q/V/S F/A/S L V|E E L K/K(V)</td><td></td><td>172.43</td><td>612.3205</td><td>1834.955</td><td>-0.0076</td><td>-4.1</td><td>4.68</td><td>69285</td><td>5.2</td><td>HUMAN</td><td>G3V4Y7</td><td>Kinectin</td></tr>
<tr><td>4857</td><td>dirMS_041712_NAR_50mM_plus2.8931.8931.3</td><td>3</td><td>13.26</td><td>1</td><td>2.37E+04</td><td></td><td>(R)GGSDDS</td><td>(R)G G S/D/D|S/S K/D/P I D\V N\Y E K(L)</td><td></td><td>114.08</td><td>609.2765</td><td>1825.809</td><td>0.0063</td><td>3.5</td><td>4.03</td><td>113880.5</td><td>9.18</td><td>HUMAN</td><td>P09874</td><td>Poly [ADP-ribose] polymerase 1</td></tr>
<tr><td>4858</td><td>dirMS_041712_NAR_120mM_minus.14167.14167.3</td><td>3</td><td>13.25</td><td>1</td><td>6.63E+04</td><td></td><td>(K)ASLENSL</td><td>(K)A/S|L/E/N S L R E V E A\R(Y)</td><td></td><td>131.78</td><td>491.9266</td><td>1473.766</td><td>-0.0003</td><td>-0.2</td><td>4.79</td><td>43773.3</td><td>5.26</td><td>HUMAN</td><td>F8VZY9</td><td>Keratin, type I cytoskeletal 18</td></tr>
<tr><td>4859</td><td>dirMS_041712_NAR_20mM_minus.14111.14111.2</td><td>2</td><td>13.25</td><td>0</td><td>1.31E+06</td><td></td><td>(K)SYELPD(</td><td>(K)S Y E|L/P D G Q V I|T I/G N E R(F)</td><td></td><td>194.08</td><td>895.9481</td><td>1790.892</td><td>-0.003</td><td>-1.7</td><td>4.14</td><td>123097.1</td><td>5.83</td><td>HUMAN</td><td>A5A3E0</td><td>POTE ankyrin domain family member F</td></tr>
<tr><td>4860</td><td>dirMS_041712_NAR_60mM_minus.10301.10542.3</td><td>3</td><td>13.25</td><td>1</td><td>6.05E+05</td><td>M88m</td><td>(K)DVDEAY</td><td>(K)D V|D/E/A/Y m N K V E/L E/S R(L)</td><td>m:Oxidized methionine</td><td>118.37</td><td>605.2791</td><td>1797.832</td><td>15.9904</td><td>-2.5</td><td>4.18</td><td>13588.1</td><td>4.66</td><td>HUMAN</td><td>F8VP67</td><td>Keratin, type II cytoskeletal 8 (Fragment)</td></tr>
<tr><td>4861</td><td>dirMS_041712_NAR_120mM_plus.13399.13399.4</td><td>4</td><td>13.25</td><td>1</td><td>1.43E+06</td><td></td><td>(K)SVHSSVI</td><td>(K)S V H S S V|P/L/L N S K D/P I D R(I)</td><td></td><td>123.27</td><td>466.7533</td><td>1863.992</td><td>-0.0009</td><td>-0.5</td><td>6.47</td><td>54427.4</td><td>5.96</td><td>HUMAN</td><td>K4DID9</td><td>Acetyl-CoA carboxylase 1</td></tr>
<tr><td>4862</td><td>dirMS_041712_NAR_20mM_plus.10909.10909.3</td><td>3</td><td>13.25</td><td>0</td><td>1.01E+05</td><td></td><td>(K)ALIAAQ</td><td>(K)A L\I A A Q|Y/S/G/A/Q V R(V)</td><td></td><td>147.88</td><td>449.9174</td><td>1347.738</td><td>-0.0003</td><td>-0.2</td><td>8.79</td><td>56491.1</td><td>7.78</td><td>HUMAN</td><td>B4DTG2</td><td>Elongation factor 1-gamma</td></tr>
<tr><td>4863</td><td>dirMS_041712_NAR_20mM_plus.18264.18264.2</td><td>2</td><td>13.25</td><td>0</td><td>1.68E+05</td><td></td><td>(K)DAGVIA</td><td>(K)D A G\V|I/A/G L\N/V L R(I)</td><td></td><td>196.73</td><td>599.3505</td><td>1197.695</td><td>-0.0013</td><td>-1.1</td><td>5.84</td><td>58502.6</td><td>6.09</td><td>HUMAN</td><td>B4DXY3</td><td>Heat shock 70 kDa protein 1-like</td></tr>
<tr><td>4864</td><td>dirMS_041712_NAR_20mM_plus.19226.19226.2</td><td>2</td><td>13.25</td><td>0</td><td>1.30E+05</td><td></td><td>(R)EGDVLT</td><td>(R)E G D\V/L|T/L/L E S E R(E)</td><td></td><td>203.6</td><td>680.8536</td><td>1360.696</td><td>0.0045</td><td>3.3</td><td>4</td><td>7898</td><td>11.1</td><td>HUMAN</td><td>P62857</td><td>40S ribosomal protein S28</td></tr>
<tr><td>4865</td><td>dirMS_041712_NAR_30mM_plus2.12816.12816.2</td><td>2</td><td>13.25</td><td>0</td><td>3.70E+05</td><td></td><td>(R)ELAEDG</td><td>(R)E L|A\E|D G\Y S|G V/E/V R(V)</td><td></td><td>120.6</td><td>712.3382</td><td>1423.67</td><td>-0.0008</td><td>-0.6</td><td>4</td><td>13054.7</td><td>9.59</td><td>HUMAN</td><td>E9PJH4</td><td>40S ribosomal protein S3</td></tr>
<tr><td>4866</td><td>dirMS_041712_NAR_30mM_plus2.20994.21014.2</td><td>2</td><td>13.25</td><td>0</td><td>2.61E+05</td><td></td><td>(K)TIFAYFT</td><td>(K)T I|F|A/Y F T G/S K(D)</td><td></td><td>164.7</td><td>567.7949</td><td>1134.583</td><td>-0.0005</td><td>-0.4</td><td>8.26</td><td>8599.5</td><td>5.51</td><td>HUMAN</td><td>I3L0K2</td><td>Thioredoxin domain-containing protein 17</td></tr>
<tr><td>4867</td><td>dirMS_041712_NAR_30mM_plus2.7004.7004.2</td><td>2</td><td>13.25</td><td>0</td><td>1.34E+05</td><td></td><td>(K)YTQSNS</td><td>(K)Y T/Q/S/N S/V/C Y/A/K(N)</td><td>C:Carbamidomethylation</td><td>91.15</td><td>660.7963</td><td>1320.589</td><td>-0.0036</td><td>-2.7</td><td>8.5</td><td>65036</td><td>6.27</td><td>HUMAN</td><td>E9PBU3</td><td>Phosphoribosylaminoimidazolecarboxamide formyltransferase</td></tr>
<tr><td>4868</td><td>dirMS_041712_NAR_30mM_plus2.9306.9306.2</td><td>2</td><td>13.25</td><td>0</td><td>2.98E+05</td><td></td><td>(K)SVEAAA</td><td>(K)S V|E/A/A A E L S/A/K(D)</td><td></td><td>103.27</td><td>538.2855</td><td>1075.563</td><td>0.0007</td><td>0.7</td><td>4.53</td><td>12131.5</td><td>11.14</td><td>HUMAN</td><td>F5GXR3</td><td>Parathymosin</td></tr>
<tr><td>4869</td><td>dirMS_041712_NAR_40mM_plus2.14030.14030.3</td><td>3</td><td>13.25</td><td>1</td><td>8.78E+04</td><td></td><td>(R)STGLELE</td><td>(R)S T|G L/E L E T/P S L V/P V K\K(N)</td><td></td><td>148.73</td><td>566.6552</td><td>1697.968</td><td>-0.0173</td><td>-10.2</td><td>5.86</td><td>34828</td><td>9.96</td><td>HUMAN</td><td>F5H5K4</td><td>Serine/threonine-protein phosphatase 1 regulatory subunit 10</td></tr>
<tr><td>4870</td><td>dirMS_041712_NAR_50mM_plus2.18645.18645.3</td><td>3</td><td>13.25</td><td>1</td><td>5.52E+05</td><td></td><td>(K)NAAGAL</td><td>(K)N A|A|G A/L/D L/L K E L K(N)</td><td></td><td>165.92</td><td>452.601</td><td>1355.789</td><td>-0.0008</td><td>-0.6</td><td>6.07</td><td>13786.8</td><td>9.33</td><td>HUMAN</td><td>B7Z4S1</td><td>Transcription elongation factor A protein 1</td></tr>
<tr><td>4871</td><td>dirMS_041712_NAR_60mM_plus.15661.15682.3</td><td>3</td><td>13.25</td><td>1</td><td>2.49E+05</td><td></td><td>(R)TEMENE</td><td>(R)T E|M E N E/F/V/L I/K/K(D)</td><td></td><td>138.75</td><td>494.2622</td><td>1480.772</td><td>0.0005</td><td>0.3</td><td>4.78</td><td>13588.1</td><td>4.66</td><td>HUMAN</td><td>F8VP67</td><td>Keratin, type II cytoskeletal 8 (Fragment)</td></tr>
<tr><td>4872</td><td>dirMS_041712_NAR_60mM_plus.8014.8246.3</td><td>3</td><td>13.25</td><td>1</td><td>6.20E+05</td><td></td><td>(R)LSQIGVE</td><td>(R)L/S|Q|I/G V E/N T E E N R R(L)</td><td></td><td>101.08</td><td>548.9468</td><td>1644.83</td><td>-0.0041</td><td>-2.5</td><td>4.79</td><td>36636.9</td><td>7.84</td><td>HUMAN</td><td>A8MVZ9</td><td>Fructose-bisphosphate aldolase</td></tr>
<tr><td>4873</td><td>dirMS_041712_NAR_60mM_plus.8380.8383.3</td><td>3</td><td>13.25</td><td>1</td><td>2.51E+05</td><td></td><td>(R)TERFGQ</td><td>(R)T E R F G Q\G G\A|G|P/V\G/G Q G P R(G)</td><td></td><td>101.95</td><td>576.6232</td><td>1727.857</td><td>-0.0022</td><td>-1.2</td><td>9.26</td><td>76262.2</td><td>9.53</td><td>HUMAN</td><td>P23246</td><td>Splicing factor, proline- and glutamine-rich</td></tr>
<tr><td>4874</td><td>dirMS_041712_NAR_120mM_minus.14880.14880.3</td><td>3</td><td>13.24</td><td>0</td><td>3.67E+05</td><td></td><td>(K)HLEINPD</td><td>(K)H L|E I N/P D\H|P/I V E T\L R(Q)</td><td></td><td>135.25</td><td>594.9872</td><td>1782.95</td><td>-0.0027</td><td>-1.5</td><td>5.27</td><td>83605</td><td>4.97</td><td>HUMAN</td><td>P08238</td><td>Heat shock protein HSP 90-beta</td></tr>
<tr><td>4875</td><td>dirMS_041712_NAR_120mM_minus.15278.15427.3</td><td>3</td><td>13.24</td><td>2</td><td>9.57E+05</td><td></td><td>(R)ALKDEEI</td><td>(R)A L|K|D E/E/K M E/L/Q|E|I Q L/K(E)</td><td></td><td>137.72</td><td>649.0139</td><td>1945.031</td><td>-0.0039</td><td>-2</td><td>4.58</td><td>33450.2</td><td>4.73</td><td>HUMAN</td><td>J3KN67</td><td>Tropomyosin alpha-3 chain</td></tr>
<tr><td>4876</td><td>dirMS_041712_NAR_120mM_minus.8498.8498.3</td><td>3</td><td>13.24</td><td>1</td><td>4.31E+05</td><td></td><td>(R)YLAEVA</td><td>(R)Y/L/A E V A/T G E/K R(A)</td><td></td><td>100.85</td><td>412.8849</td><td>1236.658</td><td>-0.0181</td><td>-14.7</td><td>6.14</td><td>28473.3</td><td>4.8</td><td>HUMAN</td><td>P61981</td><td>14-3-3 protein gamma</td></tr>
<tr><td>4877</td><td>dirMS_041712_NAR_50mM_minus2.11680.11680.3</td><td>3</td><td>13.24</td><td>0</td><td>3.27E+04</td><td>M425m M</td><td>(R)VFITDDF</td><td>(R)V/F/I/T/D D/F H D m m/P K(Y)</td><td>m:Oxidized methionine</td><td>134.58</td><td>543.2416</td><td>1595.723</td><td>31.987</td><td>-1.7</td><td>4.41</td><td>92752.5</td><td>4.76</td><td>HUMAN</td><td>P14625</td><td>Endoplasmin</td></tr>
<tr><td>4878</td><td>dirMS_041712_NAR_50mM_minus2.15082.15082.3</td><td>3</td><td>13.24</td><td>1</td><td>1.11E+06</td><td></td><td>(R)DVDFELI</td><td>(R)D V|D F/E/L/I K V E G K(V)</td><td></td><td>154</td><td>464.5852</td><td>1391.742</td><td>-0.0006</td><td>-0.4</td><td>4.32</td><td>49982</td><td>5.86</td><td>HUMAN</td><td>B4DYD8</td><td>T-complex protein 1 subunit epsilon</td></tr>
<tr><td>4879</td><td>dirMS_041712_NAR_50mM_minus2.19058.19066.3</td><td>3</td><td>13.24</td><td>1</td><td>1.34E+06</td><td></td><td>(R)KEGGLG</td><td>(R)K E G G L G P L N\I|P/L|L/A D V T R(R)</td><td></td><td>187.5</td><td>621.6941</td><td>1863.07</td><td>-0.0021</td><td>-1.1</td><td>6.07</td><td>15252.2</td><td>5.82</td><td>HUMAN</td><td>A6NIW5</td><td>Peroxiredoxin 2, isoform CRA_a</td></tr>
<tr><td>4880</td><td>dirMS_041712_NAR_60mM_minus.18486.18486.3</td><td>3</td><td>13.24</td><td>1</td><td>1.29E+05</td><td></td><td>(R)RFDEILE</td><td>(R)R F D E\I\L\E|A/S D/G\I/M V A R(G)</td><td></td><td>164.42</td><td>607.9739</td><td>1821.916</td><td>-0.0092</td><td>-5.1</td><td>4.32</td><td>18466.1</td><td>5.78</td><td>HUMAN</td><td>H3BSU3</td><td>Pyruvate kinase (Fragment)</td></tr>
<tr><td>4881</td><td>dirMS_041712_NAR_60mM_minus.6653.6653.3</td><td>3</td><td>13.24</td><td>0</td><td>4.07E+04</td><td></td><td>(K)VDCTAH</td><td>(K)V D/C T A/H S D V/C/S/A/Q G V R(G)</td><td>C:Carbamidomethylation</td><td>95.73</td><td>587.9248</td><td>1761.764</td><td>-0.0044</td><td>-2.5</td><td>5.21</td><td>41053.3</td><td>5.58</td><td>HUMAN</td><td>Q86UY0</td><td>TXNDC5 protein</td></tr>
<tr><td>4882</td><td>dirMS_041712_NAR_120mM_plus.8301.8319.2</td><td>2</td><td>13.24</td><td>1</td><td>1.05E+06</td><td></td><td>(R)KVIDDTI</td><td>(R)K|V|I|D|D/T N\I/T/R(L)</td><td></td><td>97.03</td><td>587.8203</td><td>1174.643</td><td>-0.0093</td><td>-7.9</td><td>5.96</td><td>43773.3</td><td>5.26</td><td>HUMAN</td><td>F8VZY9</td><td>Keratin, type I cytoskeletal 18</td></tr>
<tr><td>4883</td><td>dirMS_041712_NAR_20mM_plus.12304.12304.2</td><td>2</td><td>13.24</td><td>0</td><td>1.37E+05</td><td></td><td>(R)GVEICIA</td><td>(R)G V/E\I/C/I/A/T|P G R(L)</td><td>C:Carbamidomethylation</td><td>158.27</td><td>586.8084</td><td>1172.609</td><td>0.0003</td><td>0.3</td><td>6</td><td>61018.6</td><td>9.44</td><td>HUMAN</td><td>B4DLW8</td><td>Probable ATP-dependent RNA helicase DDX5</td></tr>
<tr><td>4884</td><td>dirMS_041712_NAR_20mM_plus.17702.17702.2</td><td>2</td><td>13.24</td><td>0</td><td>1.91E+05</td><td></td><td>(R)YISPDQL</td><td>(R)Y I|S/P D Q/L A D/L Y/K(S)</td><td></td><td>194.18</td><td>713.3667</td><td>1425.726</td><td>0.0001</td><td>0.1</td><td>4.21</td><td>47510.4</td><td>7.04</td><td>HUMAN</td><td>P06733</td><td>Alpha-enolase</td></tr>
<tr><td>4885</td><td>dirMS_041712_NAR_20mM_plus.18101.18101.2</td><td>2</td><td>13.24</td><td>0</td><td>1.13E+05</td><td></td><td>(K)LVLPSLIS</td><td>(K)L/V|L/P S L I|S S/R(I)</td><td></td><td>196.6</td><td>542.8388</td><td>1084.672</td><td>-0.0021</td><td>-2</td><td>9.75</td><td>39066</td><td>6.61</td><td>HUMAN</td><td>A6PVW9</td><td>Selenium binding protein 1 (Fragment)</td></tr>
<tr><td>4886</td><td>dirMS_041712_NAR_30mM_plus2.15236.15236.2</td><td>2</td><td>13.24</td><td>0</td><td>1.49E+05</td><td></td><td>(K)AFQNVF</td><td>(K)A F|Q|N|V|F A/P/R(N)</td><td></td><td>132.4</td><td>525.2819</td><td>1049.553</td><td>0.0038</td><td>3.7</td><td>9.79</td><td>60786.2</td><td>8.94</td><td>HUMAN</td><td>B4DYH8</td><td>N-acetylglucosamine-6-sulfatase</td></tr>
<tr><td>4887</td><td>dirMS_041712_NAR_30mM_plus2.15486.15486.2</td><td>2</td><td>13.24</td><td>0</td><td>4.18E+05</td><td></td><td>(R)MAGAIA</td><td>(R)M/A/G\A/I/A/Q/G L I R(A)</td><td></td><td>133.57</td><td>550.816</td><td>1100.624</td><td>0.0002</td><td>0.2</td><td>9.5</td><td>4449.1</td><td>11.42</td><td>HUMAN</td><td>F8WEI0</td><td>Pyrroline-5-carboxylate reductase 3</td></tr>
<tr><td>4888</td><td>dirMS_041712_NAR_30mM_plus2.20136.20136.2</td><td>2</td><td>13.24</td><td>0</td><td>1.49E+05</td><td></td><td>(K)TVALWD</td><td>(K)T V/A|L/W/D L/R(N)</td><td></td><td>159.03</td><td>487.2769</td><td>973.547</td><td>0</td><td>0</td><td>5.5</td><td>19598</td><td>4.45</td><td>HUMAN</td><td>B4DRT0</td><td>Histone-binding protein RBBP4</td></tr>
<tr><td>4889</td><td>dirMS_041712_NAR_50mM_plus2.18201.18201.3</td><td>3</td><td>13.24</td><td>1</td><td>1.60E+06</td><td></td><td>(R)ELIKVLEI</td><td>(R)E L I K V L\E E A/N/Q/A\I/N/P K(L)</td><td></td><td>163.67</td><td>603.6741</td><td>1809.012</td><td>-0.0039</td><td>-2.2</td><td>4.79</td><td>73202</td><td>9.19</td><td>HUMAN</td><td>C9JMU5</td><td>Probable ATP-dependent RNA helicase DDX17</td></tr>
<tr><td>4890</td><td>dirMS_041712_NAR_60mM_plus.9413.9434.3</td><td>3</td><td>13.24</td><td>1</td><td>6.18E+05</td><td></td><td>(R)LTYEIED</td><td>(R)L/T|Y/E/I/E D E K R(R)</td><td></td><td>107.38</td><td>432.5543</td><td>1295.648</td><td>0.0006</td><td>0.4</td><td>4.41</td><td>334221.3</td><td>6.44</td><td>HUMAN</td><td>P15924</td><td>Desmoplakin</td></tr>
<tr><td>4891</td><td>dirMS_041712_NAR_30mM_minus2.17041.17205.2</td><td>2</td><td>13.23</td><td>0</td><td>3.12E+05</td><td></td><td>(R)LLNTFLE</td><td>(R)L/L|N/T/F\L/E|R(L)</td><td></td><td>146.72</td><td>503.2871</td><td>1005.573</td><td>-0.0058</td><td>-5.8</td><td>6</td><td>535136.6</td><td>6.01</td><td>HUMAN</td><td>Q14204</td><td>Cytoplasmic dynein 1 heavy chain 1</td></tr>
<tr><td>4892</td><td>dirMS_041712_NAR_30mM_minus2.7280.7280.2</td><td>2</td><td>13.23</td><td>0</td><td>2.62E+05</td><td></td><td>(K)DAQGQI</td><td>(K)D A|Q/G Q|P\G L E R(A)</td><td></td><td>93.63</td><td>535.762</td><td>1070.523</td><td>-0.0058</td><td>-5.4</td><td>4.37</td><td>253486.5</td><td>5.51</td><td>HUMAN</td><td>J3KNL6</td><td>Protein transport protein Sec16A</td></tr>
<tr><td>4893</td><td>dirMS_041712_NAR_50mM_minus2.12623.12623.3</td><td>3</td><td>13.23</td><td>0</td><td>1.09E+06</td><td>M114m M</td><td>(R)AGKPVI(</td><td>(R)A G K|P V\I/C\A\T/Q/m|L E/S m I K(K)</td><td>C:Carbamidomethylation m:Oxid</td><td>139.87</td><td>636.9917</td><td>1876.969</td><td>31.9912</td><td>0.7</td><td>8.64</td><td>18466.1</td><td>5.78</td><td>HUMAN</td><td>H3BSU3</td><td>Pyruvate kinase (Fragment)</td></tr>
<tr><td>4894</td><td>dirMS_041712_NAR_50mM_minus2.14137.14137.3</td><td>3</td><td>13.23</td><td>1</td><td>1.67E+06</td><td></td><td>(R)ELISNAS</td><td>(R)E L|I/S N/A S/D\A L D K I R(L)</td><td></td><td>148.03</td><td>515.6179</td><td>1544.828</td><td>0.0113</td><td>7.3</td><td>4.56</td><td>5582.4</td><td>4.81</td><td>HUMAN</td><td>F8W026</td><td>Endoplasmin (Fragment)</td></tr>
<tr><td>4895</td><td>dirMS_041712_NAR_50mM_minus2.8677.8677.3</td><td>3</td><td>13.23</td><td>0</td><td>1.65E+05</td><td>M180m</td><td>(R)VVVLmC</td><td>(R)V V/V/L/m/G S/T S D L G H C E K(I)</td><td>C:Carbamidomethylation m:Oxid</td><td>117.55</td><td>583.2829</td><td>1731.84</td><td>15.9937</td><td>-0.7</td><td>5.32</td><td>37681.5</td><td>5.93</td><td>HUMAN</td><td>D6RF62</td><td>Phosphoribosylaminoimidazole carboxylase</td></tr>
<tr><td>4896</td><td>dirMS_041712_NAR_60mM_minus.10600.10600.3</td><td>3</td><td>13.23</td><td>0</td><td>2.57E+05</td><td></td><td>(R)SVIHSLA</td><td>(R)S V/I H/S L/A|S V/E A Q K(S)</td><td></td><td>119.02</td><td>456.9195</td><td>1368.748</td><td>-0.0042</td><td>-3.1</td><td>6.47</td><td>183049.2</td><td>5.32</td><td>HUMAN</td><td>Q9GZU2</td><td>Paternally-expressed gene 3 protein</td></tr>
<tr><td>4897</td><td>dirMS_041712_NAR_60mM_minus.15359.15359.2</td><td>2</td><td>13.23</td><td>0</td><td>3.82E+05</td><td></td><td>(R)EHALLA</td><td>(R)E H/A|L|L/A/Y/T L G\V K(Q)</td><td></td><td>144.73</td><td>657.873</td><td>1314.742</td><td>-0.0029</td><td>-2.2</td><td>6.85</td><td>50482.3</td><td>9.31</td><td>HUMAN</td><td>P68104</td><td>Elongation factor 1-alpha 1</td></tr>
<tr><td>4898</td><td>dirMS_041712_NAR_60mM_minus.16755.16755.3</td><td>3</td><td>13.23</td><td>1</td><td>4.77E+04</td><td></td><td>(K)ALQFLEE</td><td>(K)A L|Q/F/L E E\V/K/V S/R(E)</td><td></td><td>152.85</td><td>473.6009</td><td>1418.8</td><td>-0.012</td><td>-8.5</td><td>6.19</td><td>55323</td><td>6.02</td><td>HUMAN</td><td>B4DPJ8</td><td>T-complex protein 1 subunit zeta</td></tr>
<tr><td>4899</td><td>dirMS_041712_NAR_20mM_plus.13821.13821.2</td><td>2</td><td>13.23</td><td>0</td><td>2.90E+04</td><td></td><td>(K)TGAAPII</td><td>(K)T G|A|A/P I I/D V V R(S)</td><td></td><td>167.35</td><td>556.3256</td><td>1111.647</td><td>-0.0031</td><td>-2.8</td><td>5.5</td><td>10240.9</td><td>10.46</td><td>HUMAN</td><td>E9PJD9</td><td>60S ribosomal protein L27a</td></tr>
<tr><td>4900</td><td>dirMS_041712_NAR_20mM_plus.19063.19063.2</td><td>2</td><td>13.23</td><td>0</td><td>5.75E+04</td><td></td><td>(K)ILTFDQL</td><td>(K)I L/T\F/D Q\L|A\L D S\P K(G)</td><td></td><td>202.35</td><td>730.9063</td><td>1460.8</td><td>0.0058</td><td>4</td><td>4.21</td><td>14643</td><td>11.93</td><td>HUMAN</td><td>F8VUA6</td><td>60S ribosomal protein L18 (Fragment)</td></tr>
</table>

<table>
<tr><th></th><th></th><th></th><th></th><th></th><th></th><th></th><th></th><th></th><th></th><th></th><th></th><th></th><th></th><th></th><th></th><th></th><th></th><th></th><th></th><th></th></tr>
<tr><td>4901</td><td>dirMS_041712_NAR_30mM_plus2.13660.13682.2</td><td>2</td><td>13.23</td><td>0</td><td>5.27E+05</td><td></td><td>(K)VSIYEAM</td><td>(K)V/S|I/Y/E/A M/R(R)</td><td></td><td>124.8</td><td>484.7469</td><td>968.487</td><td>-0.0005</td><td>-0.5</td><td>5.97</td><td>533778</td><td>5.74</td><td>HUMAN</td><td>Q15149</td><td>Plectin</td></tr>
<tr><td>4902</td><td>dirMS_041712_NAR_30mM_plus2.21891.21891.2</td><td>2</td><td>13.23</td><td>0</td><td>8.17E+04</td><td></td><td>(K)GLVQAL</td><td>(K)G L V/Q/A|L/G/L/S N F/N S R(Q)</td><td></td><td>169.73</td><td>738.3989</td><td>1475.796</td><td>-0.006</td><td>-4.1</td><td>9.75</td><td>36914.7</td><td>6.33</td><td>HUMAN</td><td>P14550</td><td>Alcohol dehydrogenase [NADP(+)]</td></tr>
<tr><td>4903</td><td>dirMS_041712_NAR_30mM_plus2.7180.7267.2</td><td>2</td><td>13.23</td><td>0</td><td>1.70E+06</td><td></td><td>(K)TEISEMN</td><td>(K)T|E|I|S/E/M N R(N)</td><td></td><td>92.85</td><td>490.2294</td><td>979.451</td><td>0.0002</td><td>0.2</td><td>4.53</td><td>37296.3</td><td>5.3</td><td>HUMAN</td><td>F8VU64</td><td>Keratin, type II cytoskeletal 8</td></tr>
<tr><td>4904</td><td>dirMS_041712_NAR_50mM_plus2.15777.15792.3</td><td>3</td><td>13.23</td><td>0</td><td>1.18E+05</td><td></td><td>(K)GQYFGE</td><td>(K)G Q|Y/F/G E L/A/L/V/T N K P R(A)</td><td></td><td>151.15</td><td>564.9788</td><td>1692.907</td><td>0.0151</td><td>8.9</td><td>8.59</td><td>10305.6</td><td>6.94</td><td>HUMAN</td><td>H7C330</td><td>cAMP-dependent protein kinase type II-alpha regulatory subunit (Fragment)</td></tr>
<tr><td>4905</td><td>dirMS_041712_NAR_60mM_plus.6353.6353.3</td><td>3</td><td>13.23</td><td>0</td><td>9.24E+04</td><td></td><td>(R)HNGAAE</td><td>(R)H N\G A\A\E|P/E|P E A/E\T A R(G)</td><td></td><td>91.8</td><td>526.9106</td><td>1578.714</td><td>0.003</td><td>1.9</td><td>4.48</td><td>87537</td><td>9.67</td><td>HUMAN</td><td>Q9Y446</td><td>Plakophilin-3</td></tr>
<tr><td>4906</td><td>dirMS_041712_NAR_60mM_plus.9945.9981.3</td><td>3</td><td>13.23</td><td>1</td><td>4.99E+05</td><td></td><td>(R)GSNMD</td><td>(R)G S/N|M/D/F/R E/P T E E E/R(A)</td><td></td><td>109.93</td><td>566.2455</td><td>1696.723</td><td>-0.0012</td><td>-0.7</td><td>4.25</td><td>27441.6</td><td>6.68</td><td>HUMAN</td><td>Q15056</td><td>Eukaryotic translation initiation factor 4H</td></tr>
<tr><td>4907</td><td>dirMS_041712_NAR_20mM_minus.10770.10770.2</td><td>2</td><td>13.22</td><td>0</td><td>4.20E+05</td><td></td><td>(K)DVNAAI</td><td>(K)D V|N/A/A I/A/A I/K(T)</td><td></td><td>155.98</td><td>493.287</td><td>985.568</td><td>-0.0009</td><td>-1</td><td>5.84</td><td>49012.3</td><td>4.9</td><td>HUMAN</td><td>A8MUB1</td><td>Tubulin alpha-4A chain</td></tr>
<tr><td>4908</td><td>dirMS_041712_NAR_20mM_minus.9753.9753.2</td><td>2</td><td>13.22</td><td>0</td><td>1.57E+05</td><td></td><td>(K)LSELEPG</td><td>(K)L S/E|L|E|P G T G\D L R(F)</td><td></td><td>147.78</td><td>643.8318</td><td>1286.659</td><td>-0.0023</td><td>-1.8</td><td>4.14</td><td>266161</td><td>5.68</td><td>HUMAN</td><td>E9PPU0</td><td>Epiplakin</td></tr>
<tr><td>4909</td><td>dirMS_041712_NAR_40mM_minus2.10982.10982.3</td><td>3</td><td>13.22</td><td>0</td><td>4.88E+05</td><td></td><td>(R)DAGEGL</td><td>(R)D A|G\E/G L/L A V Q I T D Q E/G K/P K(R)</td><td></td><td>147.97</td><td>657.0157</td><td>1969.024</td><td>0.0089</td><td>4.5</td><td>4.32</td><td>258215.9</td><td>5.48</td><td>HUMAN</td><td>E7EN95</td><td>Filamin-B</td></tr>
[truncated: 6,632,665 more chars]
